# Melatonin Inhibits hIAPP Oligomerization by Preventing β-Sheet and Hydrogen Bond Formation of the Amyloidogenic Region Revealed by Replica-Exchange Molecular Dynamics Simulation

**DOI:** 10.3390/ijms231810264

**Published:** 2022-09-06

**Authors:** Gang Wang, Xinyi Zhu, Xiaona Song, Qingwen Zhang, Zhenyu Qian

**Affiliations:** 1 Key Laboratory of Exercise and Health Sciences (Ministry of Education), School of Kinesiology, Shanghai University of Sport, 399 Changhai Road, Shanghai 200438, China; 2 College of Physical Education and Training, Shanghai University of Sport, 399 Changhai Road, Shanghai 200438, China

**Keywords:** hIAPP, molecular dynamics simulation, oligomerization, melatonin, inhibitory mechanism

## Abstract

The pathogenesis of type 2 diabetes (T2D) is highly related to the abnormal self-assembly of the human islet amyloid polypeptide (hIAPP) into amyloid aggregates. To inhibit hIAPP aggregation is considered a promising therapeutic strategy for T2D treatment. Melatonin (Mel) was reported to effectively impede the accumulation of hIAPP aggregates and dissolve preformed fibrils. However, the underlying mechanism at the atomic level remains elusive. Here, we performed replica-exchange molecular dynamics (REMD) simulations to investigate the inhibitory effect of Mel on hIAPP oligomerization by using hIAPP_20–29_ octamer as templates. The conformational ensemble shows that Mel molecules can significantly prevent the β-sheet and backbone hydrogen bond formation of hIAPP_20–29_ octamer and remodel hIAPP oligomers and transform them into less compact conformations with more disordered contents. The interaction analysis shows that the binding behavior of Mel is dominated by hydrogen bonding with a peptide backbone and strengthened by aromatic stacking and CH–π interactions with peptide sidechains. The strong hIAPP–Mel interaction disrupts the hIAPP_20–29_ association, which is supposed to inhibit amyloid aggregation and cytotoxicity. We also performed conventional MD simulations to investigate the influence and binding affinity of Mel on the preformed hIAPP_1–37_ fibrillar octamer. Mel was found to preferentially bind to the amyloidogenic region hIAPP_20–29_, whereas it has a slight influence on the structural stability of the preformed fibrils. Our findings illustrate a possible pathway by which Mel alleviates diabetes symptoms from the perspective of Mel inhibiting amyloid deposits. This work reveals the inhibitory mechanism of Mel against hIAPP_20–29_ oligomerization, which provides useful clues for the development of efficient anti-amyloid agents.

## 1. Introduction

Diabetes is globally a growing burden on public health. There were 463 million adults with diabetes in 2019 [1], of which 90% had type 2 diabetes (T2D) mellitus [2]. Most of T2D patients show amyloid deposits in pancreatic tissues whose main component is human islet amyloid polypeptide (hIAPP) or amylin [3,4]. Islet amyloid polypeptide is also detected in pancreas biopsies from patients with recent onset T1D mellitus [5]. hIAPP is co-secreted with insulin by pancreatic β cells [6], and its aggregation and consequent aggregates are closely related to β-cell failure and insulin deficiency [7,8,9].

Similar to most intrinsically disordered proteins (IDPs), hIAPP lacks a stable, well-defined conformation under physiological conditions [10,11], and monomeric hIAPP mainly adopts a soluble random coil conformation in aqueous solution [12]. hIAPP displays a high propensity to aggregate in a nucleation-dependent manner, undergoing a conformational transition from misfolding into in-pathway oligomers to mature β-sheet-rich fibrils [13,14,15,16]. Mounting evidence indicates that the intermediate oligomers are the most cytotoxic agents [17,18]. In particular, residues 20−29 are essential for the hIAPP amyloid, identified as the amyloidogenic region [16,19,20,21]. However, it is still challenging to characterize the structural properties of oligomers due to their heterogeneity and transience.

To inhibit hIAPP oligomerization is a promising strategy to prevent the pathological process of hIAPP. Inhibitors such as small molecules [22,23,24,25,26,27,28,29,30], nanoparticles [31,32,33], short peptides [34,35,36], etc. have been developed to act on hIAPP misfolding and oligomerization. Scherzer-Attali et al. found that the quinone–tryptophan hybrids NQTrp and Cl-NQTrp showed a strong inhibition of hIAPP fibril formation (respectively, 85% and 75% inhibition in fibrils) at a low concentration (2:1 peptide molar excess) [23]. Tang et al. showed that cloridarol can inhibit hIAPP aggregation from its monomeric and oligomeric states and lead to the fibril reduction and cell viability increment. Further MD simulations revealed the binding of cloridarol results from a combination of hydrophobic interactions, aromatic stacking, and hydrogen bonding [30]. Wang et al. demonstrated that graphene quantum dots (GQDs) inhibit hIAPP fibrillization and eliminate the toxic intermediates in vitro. GQDs can also mitigate the aggregation and the damage elicited by IAPP in vivo; the strong binding of amphiphilic GQDs to IAPP converts coexisting helix and β-hairpin conformations into random coils in silicon [31]. These experimental and computational studies expand our understanding of hIAPP aggregation inhibition.

Melatonin (Mel), a human endogenous small molecule, is mainly synthesized by the pineal gland. It is essential to maintaining normal biological functions, such as antidiabetic, antioxidant, and anti-obesity activities [37], and its levels can be increased by physical exercise [38,39]. Yang et al. showed that Mel helps restore intestinal permeability by suppressing ERK/MLCK- and ROCK/MCLP-dependent MLC phosphorylation in diabetic rats [40]. Ergenc et al. found that melatonin administration may reverse depressive and anxiety-like behaviors in diabetic rats, which is mediated by the attenuation of oxidative stress, age, rage, and S100B levels in the hippocampus and prefrontal cortex [41]. Costes et al. reported that the activation of Mel signaling can alleviate β-cell loss and the dysfunction associated with molecular stress present in T2D [42]. Jung et al. showed that Mel can regulate the expression and oligomerization of amylin in rat INS-1E cells, which improves the proliferation and cellular functions of pancreatic β cells [43]. These previous studies prove that Mel is widely involved in the pathogenesis of T2D.

Interestingly, Aarabi et al. found that Mel can significantly inhibit amyloid formation and destabilize the preformed fibrils of amylin [44]. However, the inhibitory mechanism at the atomic level remains elusive. Here, we performed a replica-exchange molecular dynamics (REMD) simulation to investigate the inhibitory effect of Mel on hIAPP oligomerization by using the peptides of amyloidogenic region hIAPP_20–29_ as templates. The conformational ensembles and the involved key interactions of the hIAPP_20–29_ octamer in the absence and presence of Mel molecules were studied. Then, the influence and binding affinity of Mel on the preformed hIAPP_1–37_ fibrillar octamer were examined by conventional MD simulation. Our results show that Mel inhibits the β-sheet and backbone hydrogen bond formation of hIAPP_20–29_, and as a result, the oligomeric conformations are remodeled and less compacted. A detailed peptide–Mel interaction analysis revealed the important roles of hydrogen bonding and π–π and CH–π interactions during amyloid inhibition.

## 2. Results and Discussion

**Figure 1:**
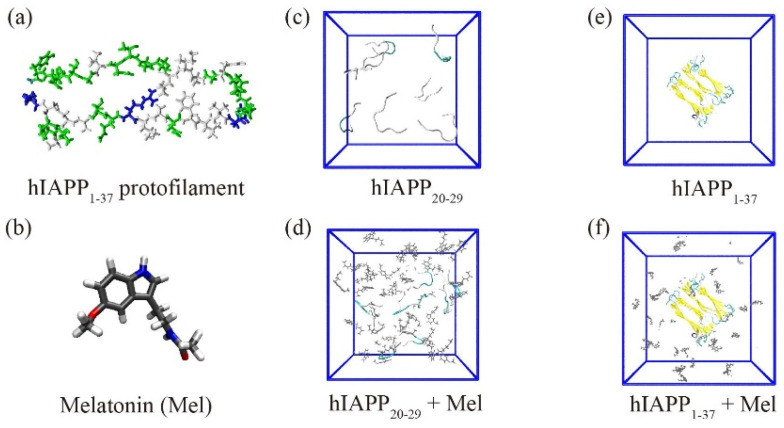
System setup. (**a**,**b**) The chemical structures of the hIAPP_1–37_ protofilament and Mel. (**c**–**f**) The initial structures of the hIAPP_20–29_ octamer and hIAPP_1–37_ fibrillar octamer in the absence and presence of Mel.

Two systems were studied using REMD: the isolated hIAPP_20–29_ octamer and hIAPP_20–29_ octamer with Mel, respectively, labeled as the hIAPP_20–29_ and hIAPP_20–29_ + Mel systems. The other two systems were studied using conventional MD: the isolated hIAPP_1–37_ fibrillar octamer and hIAPP_1–37_ fibrillar octamer with Mel, respectively, labeled as the hIAPP_1–37_ and hIAPP_1–37_ + Mel systems. The molar ratio of hIAPP peptides to Mel molecules was 1:4, consistent with the previous experimental study [44]. The system setup is shown in Figure 1. More details are given in the Supplementary Materials.

REMD simulations were performed on a total of 48 replicas whose temperatures ranged from 305 to 425 K, and the analysis used the data at 310 K. The convergence of the REMD simulation data was examined at two different time intervals (150–200 and 150–200 ns) by comparing three parameters as follows: the probability of different secondary structures, PDF of the peptide end-to-end distance, and PDF of the H-bond number for the hIAPP_20–29_ octamer. As shown in Figures S1 and S2, these parameters are well-converged, and the analysis in the main text is based on the converged data.

### 2.1. Mel Significantly Reduces β-Sheet Formation of hIAPP_20–29_

**Figure 2:**
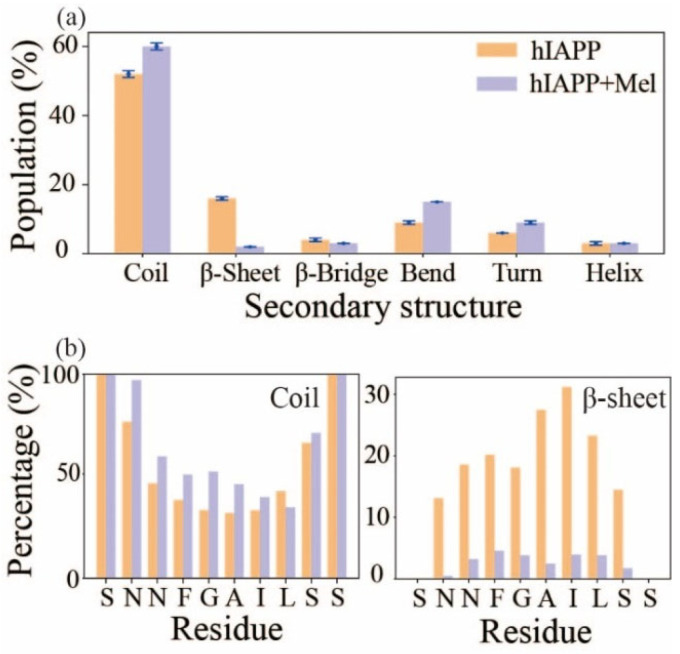
The secondary structure of the hIAPP_20–29_ octamer in the absence and presence of Mel. (**a**) The populations of different secondary structures with error bars. (**b**) The percentages of coil and β-sheet of each amino acid residue.

To examine the influence of Mel on the secondary structure of the hIAPP_20–29_ octamer, Figure 2a presents the populations of different secondary structures. With the addition of Mel, the coil population increases from 52% to 60% and β-sheet drops significantly from 16% to 2%; the bend and turn contents are also increased, and β-bridge and helix are almost unchanged. It indicates that Mel can significantly reduce β-sheet formation in hIAPP_20–29_. This observation is consistent with the previous Thioflavin T fluorescence study in which the fluorescence signal was significantly decreased when hIAPP aggregation was treated with Mel [44]. To further identify which amino acid is most affected, we calculated the dominant secondary structure (coil and β-sheet) probability of individual residues in Figure 2b. The coil probability of all the residues increases with the addition of Mel except for L27 (the coil probability of terminals S20 and S29 is 100%). All the residues in the absence of Mel have a relatively high probability to form β-sheet, among which the hydrophobic residues I26, A25, and L27 have the highest probabilities of 31%, 28%, and 23%, respectively. The β-sheet probabilities dramatically decrease for all the residues accompanied by Mel, and the hydrophobic residues I26, A25, and L27 have the most β-sheet reduction.

### 2.2. Mel Reduces Hydrogen Bond Formation of hIAPP_20–29_ and Transforms Peptide Conformations into Less Compacted Oligomers

**Figure 3:**
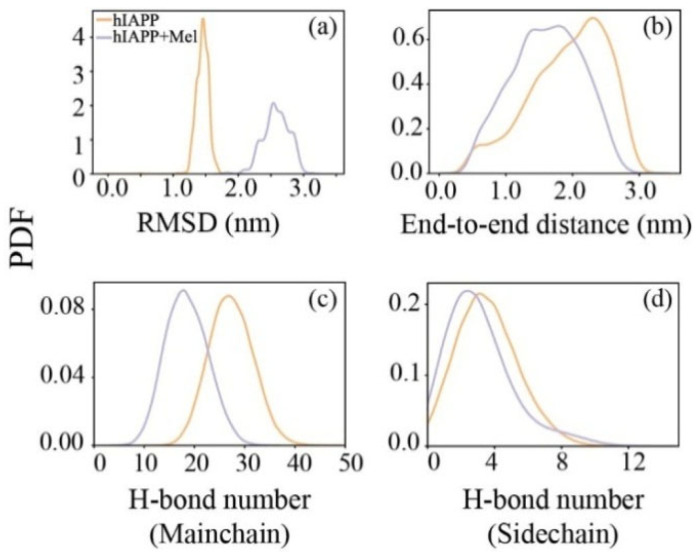
PDF of Cα-atom RMSD (**a**), end-to-end distance (**b**), and H-bond number of the peptide main chain (**c**) and side chain (**d**) for the hIAPP_20–29_ octamer in the absence and presence of Mel.

The conformational changes under the influence of Mel were explored by calculating the probability density function (PDF) of the Cα-atom root mean square deviation (RMSD) of hIAPP_20–29_. As shown in Figure 3a, the RMSD of isolated hIAPP mainly covers the range of 1.2–1.7 nm, and with the addition of Mel, the range expands to 2.0–3.1 nm. The obvious increment of the RMSD range and deviation value relative to the initial structure suggests a substantial enrichment of the structural diversity for the hIAPP_20–29_ octamer induced by Mel. The PDF of the peptide end-to-end distance in Figure 3b shows that the peptide chains become less extended in the company of Mel. The hydrogen bond (H-bond) number of peptides (Figure 3c,d) shows that, with the addition of Mel, the number of H-bonds between the main chains is greatly reduced and that of the side chains also has a small reduction. This indicates that Mel effectively blocks the formation of H-bonds between peptide backbones, unconducive to the formation of an on-pathway hIAPP oligomer. It also provides an explanation for the hIAPP_20–29_ β-sheet inhibition of Mel, as it is able to alter the backbone dihedral angle and simultaneously hinder the formation of backbone H-bonds.

**Figure 4:**
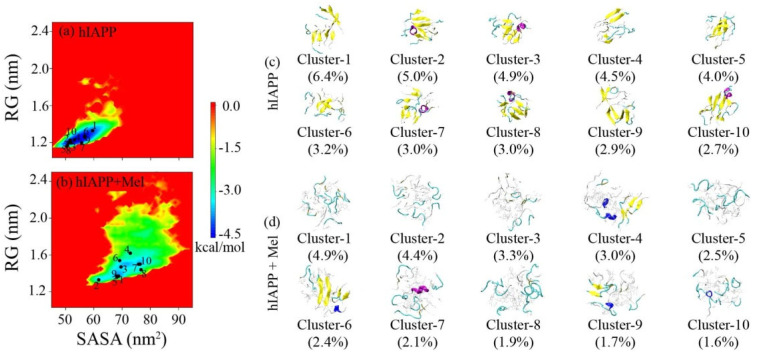
(**a**,**b**) Potential mean force (PMF) (in kcal/mol) as a function of SASA and RG of the hIAPP_20–29_ octamer and hIAPP_20–29_ + Mel systems in the absence and presence of Mel. (**c**,**d**) Representative conformations of the first ten most-populated clusters in the hIAPP_20–29_ and hIAPP_20–29_ + Mel systems. The corresponding percentage is given in parentheses.

The two-dimensional free energy landscape as a function of the solvent-accessible surface area (SASA) and the radius of gyration (RG) of hIAPP_20–29_ in Figure 4a,b display an overall view of the influence of Mel molecules on the whole conformational space of the hIAPP_20–29_ octamer. In the hIAPP_20–29_ system, the vast majority of conformations are in the range of SASA = 45–70 nm^2^ and RG = 1.1–1.6 nm, and there is only one major free energy basin. In the hIAPP_20–29_ + Mel system, the free energy surface becomes much broader, with the range of SASA expanded to 58–95 nm^2^ and that of RG to 1.2–2.2 nm. There are also several local minimum energy basins within the free energy landscape. It reveals that Mel exposes more residues to the aqueous environment and makes the hIAPP_20–29_ aggregates less compact.

The sampled conformations were further clustered, ranked, and projected onto the free energy landscape. Using a Cα-RMSD cutoff of 0.45 nm, the hIAPP_20–29_ octamers in the hIAPP_20–29_ and hIAPP_20–29_ + Mel systems were, respectively, separated into 295 and 584 clusters. The top ten most-populated clusters and corresponding proportions are presented in Figure 4c,d, and they constitute 40% (hIAPP_20–29_) and 28% (hIAPP_20–29_ + Mel) of the total conformations in their respective systems. In the absence of Mel, the hIAPP_20–29_ octamers primarily adopt β-sheet-rich structures, and β-sheets consisting of three or four β-strands in rows were observed in most clusters. The top ten clusters are relatively concentrated within the minimum energy basin. In the presence of Mel, hIAPP_20–29_ exhibits more components of a random coil, and very few β-sheet structures are detected. The distribution of the top ten clusters on the free energy landscape also becomes more discrete. Previous studies have proved the essential role of β-sheet propensity in the hIAPP_20–29_ region for hIAPP aggregation and cytotoxic oligomer formation [16,20,21]. Our results indicate that Mel can greatly increase the structural diversity of hIAPP_20–29_ oligomers and significantly reduce the β-sheet content to disordered conformations, which are supposed to go against amyloid aggregation and cytotoxicity.

### 2.3. Peptide-Mel Interaction Analysis Indicates That Hydrogen Bonding, π–π, and CH–π Interactions Play Important Roles in Amyloid Inhibition

**Figure 5:**
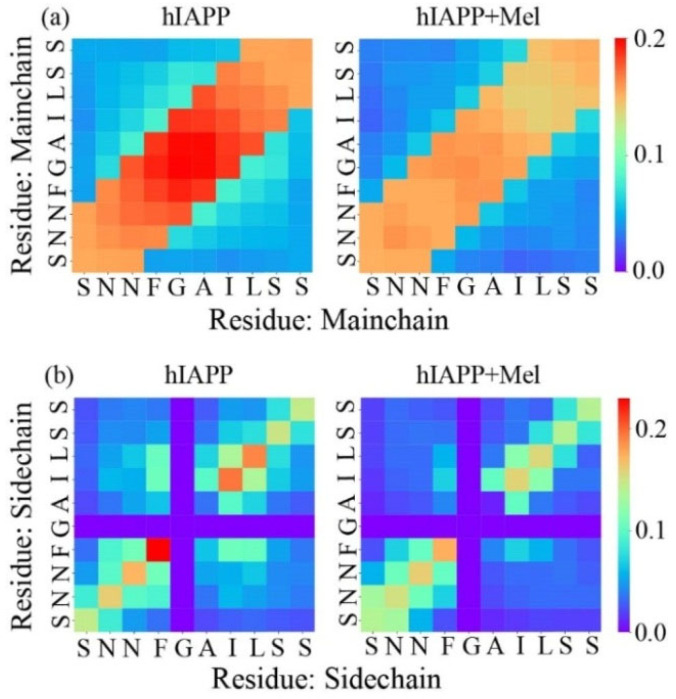
The interpeptide MC–MC (**a**) and SC–SC (**b**) contact probability maps for the hIAPP_20–29_ octamer in the absence and presence of Mel.

The effects of Mel on the hIAPP_20–29_ interactions are identified in Figure 5 by calculating the main chain–main chain (MC–MC) and side chain–side chain (SC–SC) contact probability between interpeptide pairwise residues. In the hIAPP_20–29_ system, the main chains are predominantly arranged in parallel. The side chain interaction pattern indicates that there is a very strong aromatic stacking between the F23–F23 pair and a strong hydrophobic interaction between I26–I26 and L27–L27. Thus, these paired anchors determine the parallel arrangement of the peptide chains. In addition, the terminal hydrophilic residues Ser and Asn form hydrogen bonds with each other, and F23 forms a CH–π interaction with I26 and L27, which helps to further stabilize the oligomer structure. In the hIAPP_20–29_ + Mel system, the main chains keep a parallel alignment, but the contact probability has a remarkable reduction globally due to the backbone H-bonds reduction by Mel. The side chain interaction pattern shows that the F23–F23 aromatic stacking and hydrophobic interactions between I26–I26 and L27–L27 are significantly weakened. The side chain interactions between hydrophilic residues are also slightly reduced. The aromatic stacking involving F23 and hydrophobic interactions involving L27 were reported to be critical in hIAPP aggregation and the maintenance of structural stability for hIAPP fibrils [30,45,46,47]. Our results further support these points and show that Mel can greatly interfere with the aromatic stacking and hydrophobic interactions in hIAPP_20–29_ association, which destabilizes and remodels the oligomers to facilitate amyloid inhibition.

**Figure 6:**
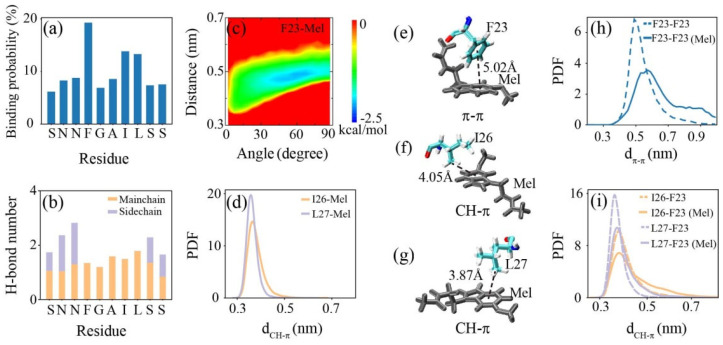
Analysis of the interactions between the hIAPP_20–29_ octamer and Mel. (**a**) The binding probability of Mel with each residue of the hIAPP_20–29_ octamer. (**b**) The number of H-bonds formed by Mel with the main chain and side chain of individual amino acid residues. (**c**) PMF (in kcal/mol) of aromatic stacking between the closest benzene rings of Phe and Mel, projected in the two-dimensional plane of the angle and centroid distance of the ring pairs. (**d**) PDF of the minimum distance between the methyl group of I26 or L27 and the benzene ring of Mel. (**e**–**g**) Snapshots of the π–π and CH–π interactions formed in a trajectory. (**h**) PDF of the centroid distance between the closest pairwise rings of F23 residues in the absence and presence of Mel. (**i**) PDF of the minimum distance between the methyl group of I26 or L27 and the benzene ring of F23 in the absence and presence of Mel.

The interactions between the hIAPP_20–29_ octamer and Mel are analyzed in Figure 6. Mel preferentially binds to aromatic F23, followed by hydrophobic I26 and L27, while it has a similar binding affinity to the rest of the residues. Our previous study showed that the binding of Mel to a tau protein is dominated by H-bonding between Mel and the peptide backbone and is synergistically aided by other interactions [48]. Here, the binding of Mel to the hIAPP_20–29_ octamer exhibits a similar behavior. Mel forms H-bonds with each residue backbone almost uniformly, and the binding to the F23, I26, and L27 residues is further strengthened by aromatic stacking and CH–π interactions, respectively. Mel forms hydrogen bonds with side chains of the terminal hydrophilic residues Ser and Asn, while the binding probability of Mel to these residues does not increase, and the side chain interplay between these residues is hardly affected (see Figure 3d and Figure 5b). Hence, we conclude that the binding of Mel to the hIAPP_20–29_ octamer is dominated by H-bonding between Mel and the peptide backbone, which is strengthened by aromatic stacking and CH–π interactions and is less affected by side chain H-bonding reinforcement.

Note that, for small molecules, there is no necessary connection between the ability to form H-bonds and prevent fibril formation. Small molecules that form abundant H-bonds may have no or negligible inhibitory effects on fibril formation. Kamihira-Ishijima et al. studied the influence of tea catechins on the amyloid fibril formation of hIAPP_22–27_ [47]. Although both EC and ECg formed abundant H-bonds with hIAPP_22–27_, only the ECg was able to inhibit fibrillization. Levy et al. found that not phenolphthalein but phenolsulfonphthalein was effective in inhibiting the fibril formation of hIAPP_20–29_, although the two compounds have similar structures and can both act as a donor or an acceptor of H-bond formation [49]. These reveal that, even for the same intrinsically disordered protein, small molecules with similar structures may have distinct inhibitory effects, which highly rely on the outcome of the specific binding of small molecules to a protein sequence.

The aromatic stacking in the hIAPP–Mel interaction was further examined in the PMF (in kcal/mol) as a function of the angle and centroid distance between the closest benzene rings of F23 and Mel. The basin center is located at about (60, 0.48), indicating the stacking pairwise rings of F23 and Mel mainly adopt a herringbone alignment. A representative snapshot displays a typical π–π stacking with a herringbone alignment formed between F23 and Mel when the centroid distance of the pairwise rings is about 5.02 Å in parallel. This aromatic stacking between the benzene rings of F23 and Mel competes and disturbs the aromatic stacking between F23 residues. The PDF of the centroid distance (*d*_π–π_) between the pairwise rings of the F23 residues without/with Mel shows that the peak position increases from 0.48 to 0.58 nm in the presence of Mel rather than isolated in the hIAPP_20–29_ system, and the peak value drops by almost half. A previous nuclear magnetic resonance (NMR) study on tea catechins and hIAPP_22−27_ and MD study on flavonoids and hIAPP_20–29_ proved that aromatic stacking is vital in binding and inhibition [45,47]. Considering the critical role of F23 in the fibril formation and amyloid inhibition of hIAPP [22,30,45], the disruption of the F23–F23 aromatic stacking interaction induced by Mel is supposed to greatly prevent hIAPP aggregation.

The CH–π interaction was estimated by calculating the PDF of the minimum distance (*d*_CH–π_) between the methyl group of I26/L27 and the benzene ring of Mel. The peaks of the PDF curves are, respectively, located at 0.36 and 0.35 nm for I26–Mel and L27–Mel, indicating an intense CH–π interaction of Mel with the hydrophobic I26 and L27. The snapshots display that an intense CH–π stacking is formed when the centroid distance between the methyl group of I26 (L27) and the benzene ring of Mel is about 4.05 Å (3.87 Å). The CH–π interaction between Ile/Leu and the aromatic residues was reported to be important for protein structural stability [50,51]. The PDF of *d*_π–π_ between the methyl group of I26/L27 and the benzene ring of F23 without/with Mel shows that the peak value becomes lower, and the distribution shifts to the right with the addition of Mel. The increased *d*_π–π_ indicates that Mel greatly weakens the intensity of the CH–π interaction between peptides. It may be attributed to the smaller steric occupation of Mel compared with F23, which makes its benzene ring interplay with the methyl groups of I26/L27 more flexibly and efficiently. A previous NMR spectrum study showed that phenolsulfonphthalein exerts a potent inhibitory effect on fibril formation by hIAPP_20–29_ and strongly binds to I26, while phenolphthalein sharing the same chemical shift deviation resulting from the binding to F23 displays low anti-amyloidogenic activity [49]. Here, the competition of Mel with the CH–π interaction between I26/L27 and F23 further destroys the peptide associations in hIAPP_20–29_ oligomerization.

### 2.4. Mel Has a Slight Influence on the Structural Stability of Preformed hIAPP_1–37_ Fibrillar Octamer and Has a High Binding Affinity to the Amyloidogenic Region

**Figure 7:**
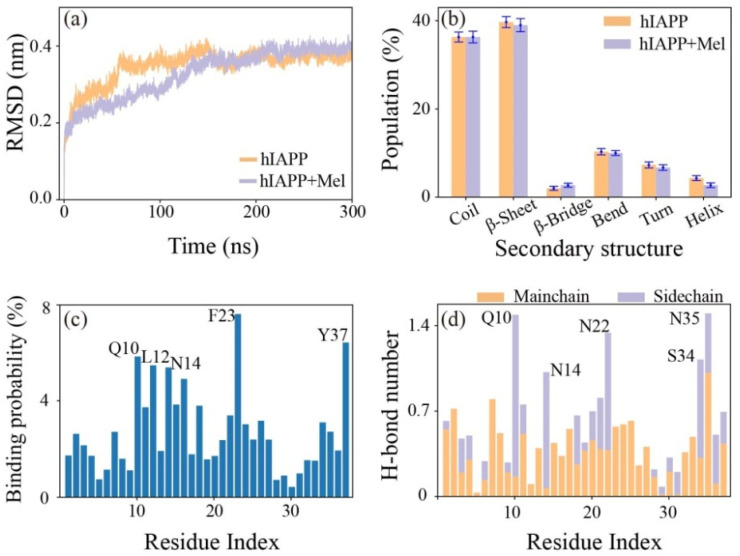
Analysis of the interactions between the hIAPP_1–37_ fibrillar octamer and Mel. (**a**) The average time evolution of Cα-atom RMSD without/with Mel. (**b**) The population of different secondary structures with error bars without/with Mel. (**c**) The binding probability of Mel to individual residues. (**d**) The number of H-bonds formed by Mel with the main chain and side chain of each residue.

In order to investigate the effect of Mel on the preformed hIAPP_1–37_ fibril and to identify the binding affinity of Mel to the full-length hIAPP_1–37_ fibril, we performed conventional MD simulations on the hIAPP_1–37_ and hIAPP_1–37_ + Mel systems. As shown in Figure 7, the average time evolution of Cα-atom RMSD and the populations of different secondary structures in the absence and presence of Mel indicate that Mel displays a slight influence on the tertiary and secondary structures of preformed hIAPP_1–37_ fibrillar octamers. The binding probability of Mel to individual residues shows that Mel prefers to bind to three sites: residues 10QRLANFL16 in the N-terminal region, residues 22NFGAIL27 in the amyloidogenic region, and residues 34SNTY37 in the C-terminal region. The residues F23, Y37, Q10, L12, and N14 have the highest binding probabilities of 7.6%, 6.4%, 5.8%, 5.5%, and 5.4%, respectively, reflecting the important roles of aromatic stacking and H-bonding interactions in Mel binding. Interesting, F23 is consistently identified as the specific binding site in the studies of chloride, C_60_(OH)_24_, and dopamine interacting with full-length hIAPP protofibrils, and all the three inhibitors preferentially bind to the amyloidogenic region of hIAPP [30,46,52]. Note that the binding behavior of Mel to the amyloidogenic region 20–29 of preformed full-length fibrils is different from that interacting with the hIAPP_20–29_ octamer, because the hydrophobicity and curvature of specific regions on the fibril surface can greatly affect the binding affinity. The number of H-bonds between individual residues and Mel shows that Mel mainly forms H-bonds with the residues N35, Q10, N22, S34, and N14. Our results indicate that Mel has a high binding affinity to the amyloidogenic region of the hIAPP_1–37_ fibrillar octamer.

We believe that our computational findings of the influence of Mel on the hIAPP secondary structure and oligomeric morphology, as well as the binding sites of Mel to IAPP will further inspire the experimental exploration of Mel inhibiting hIAPP in vitro. Additional experimental studies using the circular dichroism spectrum, NMR, cryo-electron microscopy, gel electrophoresis, etc. are expected to further confirm our simulation results. Still, there is a huge gap in the experimental observations in vitro or in animal models to clinical trials. The failure of the anti-AD drug Bapineuzumab in two phase III clinical trials has fully demonstrated the complexity of the amyloid inhibition mechanism.

## 3. Materials and Methods

### 3.1. Modeling hIAPP and Melatonin

The amino acid sequence of hIAPP is KCNTATCATQ^10^RLANFLVHSS^20^NNFGAILSST^30^NVGSNTY. The hIAPP_1–37_ fibrillar octamer was modeled based on a previous solid-state NMR study [53]. One single hIAPP_1–37_ peptide consists of the N-terminal region (residues 1−19), the amyloidogenic region (residues 20−29), and the C-terminal region (residues 30−37). The N-terminus was capped by NH3+, and the C-terminus was amidated in accordance with the experiment [53]. The hIAPP_20–29_ peptide was capped by CH_3_CO at the N-terminus and by NH_2_ at the C-terminus. The initial structure of the Mel molecule was taken from the ChemSpider database (ID = 872), shown in Figure 1b. The topology of Mel was generated by the GlycoBioChem PRODRG2 Server [54]. The structure of Mel was optimized with Spartan’10 first [55] and was energy-minimized by GAMESS [56]. The hIAPP_20–29_ + Mel system consists of eight hIAPP_20–29_ peptides randomly placed in the simulation box and Mel molecules placed 2.0 nm (minimum distance) away from hIAPP (see Figure 1). The simulation box is filled with TIP3P water [57], and NaCl was added to neutralize the system and to provide a salt concentration of 0.1 M. The hIAPP_1–37_ + Mel system consists of the hIAPP_1–37_ fibrillar octamer and 32 Mel molecules. The systems of the isolated hIAPP_20–29_ octamer and hIAPP_1–37_ fibrillar octamer in water were run as control groups. The details of the simulated systems are listed in Table S1.

### 3.2. Simulation Details

All-atom MD simulations were performed in an isothermal−isobaric (NPT) ensemble using Gromacs-2018.4 software [58]. Given that characterizing the structure and the oligomerization of hIAPP is very complex, the simulation results are highly dependent on the initial structure, the force field, and the method employed [59]. Widely used and recommended in previous studies [60,61,62], we employed the AMBER99SB-ILDN force field [63] to simulate our systems. Periodic boundary conditions were applied in all three directions. The pressures and temperatures of the systems were coupled using the Parinello–Rahman algorithm [64,65] (1 bar, τ_P_ = 1.0 ps) and velocity rescaling [66] (τ_T_ = 0.2 ps), respectively. The time step of the simulation was 2 fs, and all bonds were constrained by the LINCS algorithm [67]. The van der Waals interaction was calculated using a cutoff of 1.0 nm, and the electrostatic interaction was treated by means of the PME method [68], with a real space cutoff of 1.0 nm. The simulation parameter settings applied in our work are consistent with previous studies [46,52].

### 3.3. Analysis Methods

A trajectory analysis was performed using in-house developed codes and GROMACS toolkits. The DSSP program [69] was be used to calculate the secondary structure. The Daura cluster analysis method [70] was used to cluster the conformations sampled in the REMD simulations with a Cα-RMSD cutoff of 0.45 nm. PMF was calculated using the relation −*RTlnH*(*x*, *y*), where *H*(*x*, *y*) is the histogram of two selected reaction coordinates SASA and RG of the hIAPP_20–29_ peptides. An atomic contact is defined when two nonhydrogen atoms come within 0.54 nm. An H-bond is defined as formed when the distance between donor D and acceptor A is less than 0.35 nm and the D–H–A angle is larger than 150°. A water probe radius of 0.14 nm was used to calculate the SASA.

## 4. Conclusions

In this work, REMD simulations were performed to investigate the inhibitory effect of Mel on hIAPP oligomerization by using the hIAPP_20–29_ octamer as the templates. We found that Mel can significantly reduce β-sheet and backbone H-bond formation, and the hIAPP_20–29_ octamer is transformed into less compact conformations with the disordered components increased. A detailed interaction analysis indicated that the binding behavior of Mel is dominated by H-bonding between Mel and the hIAPP_20–29_ backbone and strengthened by aromatic stacking and CH–π interactions between Mel and the peptide side chains. The hIAPP–Mel interaction greatly interferes with the hIAPP_20–29_ association and destabilizes and remodels the oligomers, which is supposed to prevent amyloid aggregation and cytotoxicity. The influence and binding affinity of Mel on the preformed hIAPP_1–37_ fibrillar octamer were further examined by performing conventional MD simulations. The results show that Mel prefers to interact with the amyloidogenic region of preformed the hIAPP_1–37_ fibrillar octamer, whereas it has a slight influence on the structural stability. Our findings illustrate a possible pathway by which Mel alleviates diabetes symptoms from the perspective of Mel inhibiting amyloid deposits. This work reveals the inhibitory mechanism of Mel against hIAPP_20–29_ oligomerization, which facilitates the development of efficient anti-amyloid agents and provides inspiration for a novel cellular approach in amyloid detection and inhibition.

## Data Availability

The datasets generated during and/or analyzed during the current study are available from the corresponding author upon reasonable request.

## Supplementary Materials

The following supporting information can be downloaded at: https://www.mdpi.com/article/10.3390/ijms231810264/s1.
