# Supplementary material for: Melatonin Inhibits hIAPP Oligomerization by Preventing β-Sheet and Hydrogen Bond Formation of the Amyloidogenic Region Revealed by Replica-Exchange Molecular Dynamics Simulation
Source: Int J Mol Sci. 2022 Sep 6;23(18):10264. doi: 10.3390/ijms231810264 (PMC9499688; doi:10.3390/ijms231810264)
Supplement: Supplementary file 1 [file ijms-23-10264-s001.zip › ijms-1892476-supplementary.pdf]

Supplementary Materials

Melatonin inhibits hIAPP oligomerization by preventing β-sheet and hydrogen bond formation of the amyloidogenic region revealed by replica-exchange molecular dynamics simulation

Gang Wang [1], Xinyi Zhu [1], Xiaona Song [1], Qingwen Zhang [2] and Zhenyu Qian [1,*]

[1] Key Laboratory of Exercise and Health Sciences (Ministry of Education), School of Kinesiology, Shanghai University of Sport, 399 Changhai Road, Shanghai 200438, China;

[2] College of Physical Education and Training, Shanghai University of Sport, 399 Changhai Road,Shanghai 200438, China

[*] Correspondence: qianzhenyu@sus.edu.cn

The simulation details of all the systems are listed in Table S1.

Table S1. Simulation details of different systems. The $hIAPP_{20-29}$ system and $hIAPP_{20-29}$ + Mel system are performed by REMD simulations, and the other systems by means of conventional MD simulations

System	Number of atoms	Number of replicas	Time (ns)	Temperature (K)	Acceptation ratio
**$hIAPP_{20-29}$**	17121	48	200	305-425	18.04%
**$hIAPP_{20-29}$ + Mel**	16921	48	200	305-425	18.22%
**$hIAPP_{1-37}$**	23541	3	300	310	None
**$hIAPP_{1-37}$ + Mel**	23424	3	300	310	None

Setup of $hIAPP_{20-29}$ and $hIAPP_{20-29}$ + Mel systems. The REMD simulations of $hIAPP_{20-29}$ octamer in the absence/presence of melatonin include 48 replicas, and the temperatures are varied from 305 K to 425 K (temperature list: 305.00, 309.34, 311.53, 313.73, 315.96, 318.20, 320.45, 322.72, 325.01, 327.31, 329.63, 331.96, 334.31, 336.68, 339.07, 341.47, 343.89, 346.32, 348.78, 351.25, 353.74, 356.24, 358.77, 361.31, 363.87, 366.44, 369.04, 371.66, 374.29, 376.94, 379.61, 382.30, 385.01, 387.73, 390.48, 393.25, 396.03, 398.84, 401.66, 404.51, 407.38, 410.26, 413.17, 416.09, 419.04, 422.01, 425.00 K).

Convergence check of the REMD simulations. The propensity of secondary structures in the time intervals of 100-150 and 150-200 ns is shown in Figure S1. The probability density function (PDF) of end-to-end distance and H-bond number is shown in Figure S2. The propensity and PDF of the REMD runs in two independent time interval are almost the same, which suggests the REMD simulations are reasonably converged. Thus, our analyses are based on the last 100 ns data.

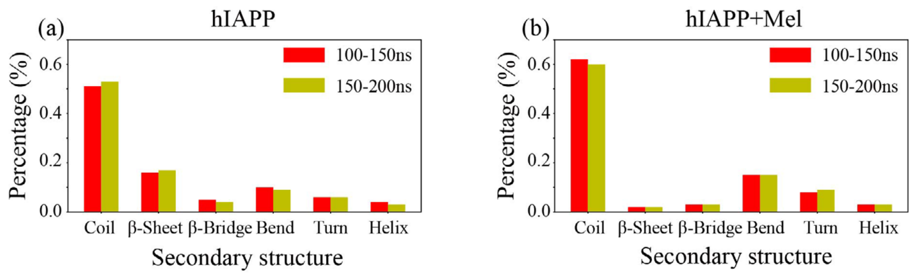

Figure S1. The secondary structure of $hIAPP_{20-29}$ octamer in the absence and presence of Mel in the time intervals of 100-150 and 150-200 ns.

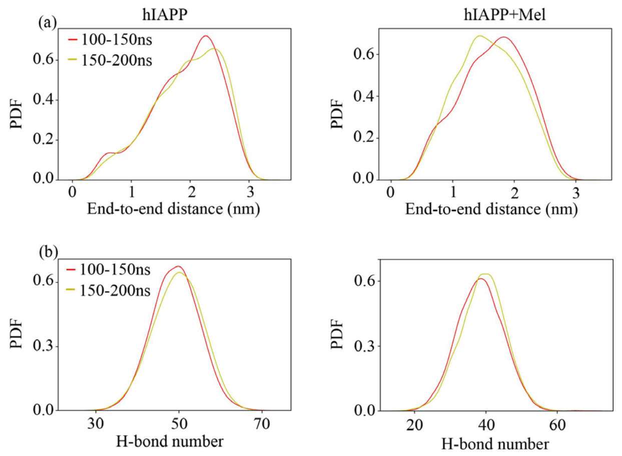

Figure S2. PDF of end-to-end distance (a) and H-bond number (b) for $hIAPP_{20-29}$ octamer in the absence and presence of Mel in the time intervals of 100-150 and 150-200 ns.

PDB file of hIAPP$_{20\text{-}29}$ system：

ATOM	1	CH3	ACE	1	42.170	32.840	27.950	1.00	0.00
ATOM	2	1HH3	ACE	1	42.740	33.490	27.270	1.00	0.00
ATOM	3	2HH3	ACE	1	41.890	31.880	27.530	1.00	0.00
ATOM	4	3HH3	ACE	1	41.230	33.250	28.300	1.00	0.00
ATOM	5	C	ACE	1	43.040	32.490	29.150	1.00	0.00
ATOM	6	O	ACE	1	43.800	33.360	29.560	1.00	0.00
ATOM	7	N	SER	2	42.880	31.310	29.750	1.00	0.00
ATOM	8	H	SER	2	42.100	30.750	29.430	1.00	0.00
ATOM	9	CA	SER	2	43.650	30.790	30.870	1.00	0.00
ATOM	10	HA	SER	2	44.650	31.210	30.840	1.00	0.00
ATOM	11	CB	SER	2	43.030	31.300	32.170	1.00	0.00
ATOM	12	HB1	SER	2	43.120	32.350	32.410	1.00	0.00
ATOM	13	HB2	SER	2	43.360	30.600	32.940	1.00	0.00
ATOM	14	OG	SER	2	41.640	31.040	32.220	1.00	0.00
ATOM	15	HG	SER	2	41.480	31.180	33.160	1.00	0.00
ATOM	16	C	SER	2	43.900	29.290	30.750	1.00	0.00
ATOM	17	O	SER	2	43.200	28.580	30.020	1.00	0.00
ATOM	18	N	ASN	3	44.860	28.830	31.550	1.00	0.00
ATOM	19	H	ASN	3	45.260	29.440	32.250	1.00	0.00
ATOM	20	CA	ASN	3	45.130	27.410	31.640	1.00	0.00
ATOM	21	HA	ASN	3	44.240	26.840	31.380	1.00	0.00
ATOM	22	CB	ASN	3	46.290	26.970	30.750	1.00	0.00
ATOM	23	HB1	ASN	3	46.380	25.880	30.780	1.00	0.00
ATOM	24	HB2	ASN	3	47.260	27.370	31.070	1.00	0.00
ATOM	25	CG	ASN	3	46.110	27.160	29.250	1.00	0.00
ATOM	26	OD1	ASN	3	46.880	27.800	28.540	1.00	0.00
ATOM	27	ND2	ASN	3	45.070	26.610	28.620	1.00	0.00
ATOM	28	1HD2	ASN	3	44.410	25.970	29.040	1.00	0.00
ATOM	29	2HD2	ASN	3	44.930	26.700	27.620	1.00	0.00
ATOM	30	C	ASN	3	45.460	27.000	33.070	1.00	0.00
ATOM	31	O	ASN	3	45.860	27.810	33.910	1.00	0.00
ATOM	32	N	ASN	4	45.160	25.750	33.410	1.00	0.00
ATOM	33	H	ASN	4	44.660	25.200	32.730	1.00	0.00
ATOM	34	CA	ASN	4	45.580	25.030	34.600	1.00	0.00
ATOM	35	HA	ASN	4	45.630	25.670	35.480	1.00	0.00
ATOM	36	CB	ASN	4	44.690	23.810	34.840	1.00	0.00
ATOM	37	HB1	ASN	4	45.060	23.210	35.670	1.00	0.00
ATOM	38	HB2	ASN	4	44.750	23.170	33.960	1.00	0.00
ATOM	39	CG	ASN	4	43.260	24.170	35.240	1.00	0.00
ATOM	40	OD1	ASN	4	42.840	25.320	35.330	1.00	0.00
ATOM	41	ND2	ASN	4	42.490	23.140	35.600	1.00	0.00
ATOM	42	1HD2	ASN	4	42.890	22.210	35.570	1.00	0.00
ATOM	43	2HD2	ASN	4	41.500	23.270	35.710	1.00	0.00

ATOM	44	C	ASN	4	46.960	24.460	34.330	1.00	0.00
ATOM	45	O	ASN	4	47.430	24.410	33.190	1.00	0.00
ATOM	46	N	PHE	5	47.590	23.860	35.340	1.00	0.00
ATOM	47	H	PHE	5	47.110	23.770	36.220	1.00	0.00
ATOM	48	CA	PHE	5	48.880	23.200	35.260	1.00	0.00
ATOM	49	HA	PHE	5	49.500	23.670	34.500	1.00	0.00
ATOM	50	CB	PHE	5	49.550	23.220	36.630	1.00	0.00
ATOM	51	HB1	PHE	5	50.440	22.600	36.580	1.00	0.00
ATOM	52	HB2	PHE	5	48.920	22.660	37.320	1.00	0.00
ATOM	53	CG	PHE	5	50.090	24.530	37.150	1.00	0.00
ATOM	54	CD1	PHE	5	50.970	25.350	36.430	1.00	0.00
ATOM	55	HD1	PHE	5	51.370	24.900	35.540	1.00	0.00
ATOM	56	CE1	PHE	5	51.410	26.580	36.940	1.00	0.00
ATOM	57	HE1	PHE	5	52.220	27.170	36.530	1.00	0.00
ATOM	58	CZ	PHE	5	50.780	27.070	38.090	1.00	0.00
ATOM	59	HZ	PHE	5	51.070	27.970	38.610	1.00	0.00
ATOM	60	CE2	PHE	5	49.790	26.330	38.760	1.00	0.00
ATOM	61	HE2	PHE	5	49.240	26.810	39.550	1.00	0.00
ATOM	62	CD2	PHE	5	49.450	25.060	38.270	1.00	0.00
ATOM	63	HD2	PHE	5	48.620	24.500	38.680	1.00	0.00
ATOM	64	C	PHE	5	48.710	21.790	34.720	1.00	0.00
ATOM	65	O	PHE	5	48.530	20.890	35.530	1.00	0.00
ATOM	66	N	GLY	6	48.840	21.590	33.400	1.00	0.00
ATOM	67	H	GLY	6	49.090	22.360	32.810	1.00	0.00
ATOM	68	CA	GLY	6	48.770	20.250	32.840	1.00	0.00
ATOM	69	HA1	GLY	6	49.550	19.780	33.440	1.00	0.00
ATOM	70	HA2	GLY	6	48.960	20.260	31.770	1.00	0.00
ATOM	71	C	GLY	6	47.410	19.590	33.000	1.00	0.00
ATOM	72	O	GLY	6	46.410	20.300	32.960	1.00	0.00
ATOM	73	N	ALA	7	47.350	18.270	33.230	1.00	0.00
ATOM	74	H	ALA	7	48.210	17.730	33.250	1.00	0.00
ATOM	75	CA	ALA	7	46.140	17.520	33.480	1.00	0.00
ATOM	76	HA	ALA	7	45.430	17.730	32.670	1.00	0.00
ATOM	77	CB	ALA	7	46.520	16.050	33.390	1.00	0.00
ATOM	78	HB1	ALA	7	47.430	15.840	33.950	1.00	0.00
ATOM	79	HB2	ALA	7	45.710	15.470	33.840	1.00	0.00
ATOM	80	HB3	ALA	7	46.600	15.880	32.320	1.00	0.00
ATOM	81	C	ALA	7	45.280	17.880	34.690	1.00	0.00
ATOM	82	O	ALA	7	44.220	17.260	34.790	1.00	0.00
ATOM	83	N	ILE	8	45.710	18.750	35.600	1.00	0.00
ATOM	84	H	ILE	8	46.520	19.280	35.320	1.00	0.00
ATOM	85	CA	ILE	8	45.200	18.950	36.940	1.00	0.00
ATOM	86	HA	ILE	8	44.970	17.960	37.340	1.00	0.00
ATOM	87	CB	ILE	8	46.290	19.650	37.750	1.00	0.00

ATOM	88	HB	ILE	8	46.520	20.600	37.270	1.00	0.00
ATOM	89	CG2	ILE	8	45.900	19.910	39.200	1.00	0.00
ATOM	90	1HG2	ILE	8	45.320	20.830	39.300	1.00	0.00
ATOM	91	2HG2	ILE	8	45.360	19.030	39.520	1.00	0.00
ATOM	92	3HG2	ILE	8	46.680	20.060	39.950	1.00	0.00
ATOM	93	CG1	ILE	8	47.580	18.840	37.780	1.00	0.00
ATOM	94	1HG1	ILE	8	48.300	19.470	38.290	1.00	0.00
ATOM	95	2HG1	ILE	8	47.950	18.600	36.780	1.00	0.00
ATOM	96	CD	ILE	8	47.420	17.550	38.590	1.00	0.00
ATOM	97	HD1	ILE	8	47.340	17.790	39.650	1.00	0.00
ATOM	98	HD2	ILE	8	46.520	17.030	38.270	1.00	0.00
ATOM	99	HD3	ILE	8	48.340	16.970	38.550	1.00	0.00
ATOM	100	C	ILE	8	43.970	19.840	36.880	1.00	0.00
ATOM	101	O	ILE	8	43.890	20.750	36.060	1.00	0.00
ATOM	102	N	LEU	9	43.030	19.700	37.830	1.00	0.00
ATOM	103	H	LEU	9	43.010	18.840	38.360	1.00	0.00
ATOM	104	CA	LEU	9	41.850	20.520	37.960	1.00	0.00
ATOM	105	HA	LEU	9	41.660	21.000	37.000	1.00	0.00
ATOM	106	CB	LEU	9	40.600	19.680	38.210	1.00	0.00
ATOM	107	HB1	LEU	9	39.800	20.410	38.260	1.00	0.00
ATOM	108	HB2	LEU	9	40.740	19.250	39.200	1.00	0.00
ATOM	109	CG	LEU	9	40.210	18.550	37.270	1.00	0.00
ATOM	110	HG	LEU	9	41.130	18.020	37.020	1.00	0.00
ATOM	111	CD1	LEU	9	39.260	17.500	37.840	1.00	0.00
ATOM	112	1HD1	LEU	9	38.340	18.050	37.980	1.00	0.00
ATOM	113	2HD1	LEU	9	39.240	16.660	37.140	1.00	0.00
ATOM	114	3HD1	LEU	9	39.670	17.080	38.760	1.00	0.00
ATOM	115	CD2	LEU	9	39.690	19.100	35.940	1.00	0.00
ATOM	116	1HD2	LEU	9	39.480	18.160	35.420	1.00	0.00
ATOM	117	2HD2	LEU	9	38.860	19.790	35.950	1.00	0.00
ATOM	118	3HD2	LEU	9	40.510	19.600	35.430	1.00	0.00
ATOM	119	C	LEU	9	42.000	21.570	39.060	1.00	0.00
ATOM	120	O	LEU	9	42.710	21.290	40.030	1.00	0.00
ATOM	121	N	SER	10	41.370	22.740	38.940	1.00	0.00
ATOM	122	H	SER	10	40.810	22.830	38.100	1.00	0.00
ATOM	123	CA	SER	10	41.340	23.760	39.970	1.00	0.00
ATOM	124	HA	SER	10	40.860	24.650	39.550	1.00	0.00
ATOM	125	CB	SER	10	40.530	23.370	41.200	1.00	0.00
ATOM	126	HB1	SER	10	40.380	24.280	41.780	1.00	0.00
ATOM	127	HB2	SER	10	40.980	22.550	41.750	1.00	0.00
ATOM	128	OG	SER	10	39.200	23.020	40.910	1.00	0.00
ATOM	129	HG	SER	10	38.810	22.820	41.770	1.00	0.00
ATOM	130	C	SER	10	42.680	24.370	40.350	1.00	0.00
ATOM	131	O	SER	10	42.960	24.840	41.450	1.00	0.00

ATOM	132	N	SER	11	43.640	24.440	39.420	1.00	0.00
ATOM	133	H	SER	11	43.480	23.950	38.550	1.00	0.00
ATOM	134	CA	SER	11	44.840	25.210	39.650	1.00	0.00
ATOM	135	HA	SER	11	44.490	26.220	39.880	1.00	0.00
ATOM	136	CB	SER	11	45.740	24.720	40.780	1.00	0.00
ATOM	137	HB1	SER	11	45.290	25.070	41.720	1.00	0.00
ATOM	138	HB2	SER	11	46.760	25.110	40.760	1.00	0.00
ATOM	139	OG	SER	11	45.800	23.310	40.770	1.00	0.00
ATOM	140	HG	SER	11	44.880	23.080	40.840	1.00	0.00
ATOM	141	C	SER	11	45.750	25.280	38.430	1.00	0.00
ATOM	142	O	SER	11	46.090	24.230	37.880	1.00	0.00
ATOM	143	N	NH2	12	46.350	26.430	38.140	1.00	0.00
ATOM	144	H1	NH2	12	45.930	27.280	38.500	1.00	0.00
ATOM	145	H2	NH2	12	47.000	26.480	37.370	1.00	0.00
ATOM	146	CH3	ACE	1	35.490	29.140	30.390	1.00	0.00
ATOM	147	1HH3	ACE	1	35.920	28.150	30.590	1.00	0.00
ATOM	148	2HH3	ACE	1	35.760	29.690	31.290	1.00	0.00
ATOM	149	3HH3	ACE	1	34.420	29.080	30.200	1.00	0.00
ATOM	150	C	ACE	1	36.080	29.780	29.150	1.00	0.00
ATOM	151	O	ACE	1	35.360	30.030	28.190	1.00	0.00
ATOM	152	N	SER	2	37.410	29.880	29.110	1.00	0.00
ATOM	153	H	SER	2	37.890	29.630	29.970	1.00	0.00
ATOM	154	CA	SER	2	38.220	30.360	28.010	1.00	0.00
ATOM	155	HA	SER	2	37.680	30.390	27.060	1.00	0.00
ATOM	156	CB	SER	2	38.600	31.800	28.340	1.00	0.00
ATOM	157	HB1	SER	2	39.270	32.090	27.530	1.00	0.00
ATOM	158	HB2	SER	2	38.960	31.870	29.370	1.00	0.00
ATOM	159	OG	SER	2	37.420	32.560	28.240	1.00	0.00
ATOM	160	HG	SER	2	36.950	32.740	29.050	1.00	0.00
ATOM	161	C	SER	2	39.510	29.570	27.830	1.00	0.00
ATOM	162	O	SER	2	40.580	30.010	28.230	1.00	0.00
ATOM	163	N	ASN	3	39.370	28.430	27.160	1.00	0.00
ATOM	164	H	ASN	3	38.430	28.060	27.110	1.00	0.00
ATOM	165	CA	ASN	3	40.520	27.570	26.930	1.00	0.00
ATOM	166	HA	ASN	3	40.930	27.290	27.900	1.00	0.00
ATOM	167	CB	ASN	3	40.070	26.320	26.170	1.00	0.00
ATOM	168	HB1	ASN	3	39.560	26.540	25.230	1.00	0.00
ATOM	169	HB2	ASN	3	39.390	25.750	26.800	1.00	0.00
ATOM	170	CG	ASN	3	41.240	25.380	25.910	1.00	0.00
ATOM	171	OD1	ASN	3	41.690	25.120	24.800	1.00	0.00
ATOM	172	ND2	ASN	3	41.900	24.820	26.930	1.00	0.00
ATOM	173	1HD2	ASN	3	41.700	25.030	27.890	1.00	0.00
ATOM	174	2HD2	ASN	3	42.710	24.270	26.690	1.00	0.00
ATOM	175	C	ASN	3	41.590	28.250	26.100	1.00	0.00

ATOM	176	O	ASN	3	41.370	28.240	24.890	1.00	0.00
ATOM	177	N	ASN	4	42.730	28.730	26.600	1.00	0.00
ATOM	178	H	ASN	4	42.810	28.780	27.600	1.00	0.00
ATOM	179	CA	ASN	4	43.860	29.160	25.790	1.00	0.00
ATOM	180	HA	ASN	4	43.410	29.810	25.040	1.00	0.00
ATOM	181	CB	ASN	4	44.710	29.990	26.750	1.00	0.00
ATOM	182	HB1	ASN	4	45.070	29.340	27.550	1.00	0.00
ATOM	183	HB2	ASN	4	44.250	30.910	27.110	1.00	0.00
ATOM	184	CG	ASN	4	45.910	30.510	25.980	1.00	0.00
ATOM	185	OD1	ASN	4	45.760	31.250	25.010	1.00	0.00
ATOM	186	ND2	ASN	4	47.160	30.100	26.250	1.00	0.00
ATOM	187	1HD2	ASN	4	47.310	29.420	26.970	1.00	0.00
ATOM	188	2HD2	ASN	4	47.850	30.560	25.680	1.00	0.00
ATOM	189	C	ASN	4	44.570	27.980	25.160	1.00	0.00
ATOM	190	O	ASN	4	44.920	26.960	25.750	1.00	0.00
ATOM	191	N	PHE	5	44.820	28.130	23.860	1.00	0.00
ATOM	192	H	PHE	5	44.390	28.940	23.440	1.00	0.00
ATOM	193	CA	PHE	5	45.910	27.480	23.150	1.00	0.00
ATOM	194	HA	PHE	5	46.690	27.160	23.840	1.00	0.00
ATOM	195	CB	PHE	5	45.500	26.120	22.600	1.00	0.00
ATOM	196	HB1	PHE	5	45.130	26.230	21.580	1.00	0.00
ATOM	197	HB2	PHE	5	44.690	25.740	23.220	1.00	0.00
ATOM	198	CG	PHE	5	46.560	25.050	22.400	1.00	0.00
ATOM	199	CD1	PHE	5	46.460	23.870	23.150	1.00	0.00
ATOM	200	HD1	PHE	5	45.720	23.840	23.940	1.00	0.00
ATOM	201	CE1	PHE	5	47.430	22.870	23.020	1.00	0.00
ATOM	202	HE1	PHE	5	47.190	22.040	23.670	1.00	0.00
ATOM	203	CZ	PHE	5	48.520	23.060	22.170	1.00	0.00
ATOM	204	HZ	PHE	5	49.280	22.290	22.160	1.00	0.00
ATOM	205	CE2	PHE	5	48.640	24.230	21.410	1.00	0.00
ATOM	206	HE2	PHE	5	49.530	24.450	20.840	1.00	0.00
ATOM	207	CD2	PHE	5	47.680	25.240	21.580	1.00	0.00
ATOM	208	HD2	PHE	5	47.690	26.120	20.960	1.00	0.00
ATOM	209	C	PHE	5	46.510	28.410	22.110	1.00	0.00
ATOM	210	O	PHE	5	47.710	28.390	21.820	1.00	0.00
ATOM	211	N	GLY	6	45.730	29.370	21.610	1.00	0.00
ATOM	212	H	GLY	6	44.830	29.510	22.030	1.00	0.00
ATOM	213	CA	GLY	6	46.140	30.310	20.580	1.00	0.00
ATOM	214	HA1	GLY	6	45.350	30.980	20.260	1.00	0.00
ATOM	215	HA2	GLY	6	46.430	29.750	19.690	1.00	0.00
ATOM	216	C	GLY	6	47.220	31.320	20.940	1.00	0.00
ATOM	217	O	GLY	6	47.840	31.880	20.030	1.00	0.00
ATOM	218	N	ALA	7	47.490	31.610	22.210	1.00	0.00
ATOM	219	H	ALA	7	47.130	31.020	22.960	1.00	0.00

ATOM	220	CA	ALA	7	48.440	32.610	22.650	1.00	0.00
ATOM	221	HA	ALA	7	49.070	32.870	21.800	1.00	0.00
ATOM	222	CB	ALA	7	47.540	33.780	23.060	1.00	0.00
ATOM	223	HB1	ALA	7	48.150	34.680	23.140	1.00	0.00
ATOM	224	HB2	ALA	7	46.850	33.980	22.240	1.00	0.00
ATOM	225	HB3	ALA	7	47.000	33.410	23.930	1.00	0.00
ATOM	226	C	ALA	7	49.330	31.980	23.710	1.00	0.00
ATOM	227	O	ALA	7	48.990	30.910	24.210	1.00	0.00
ATOM	228	N	ILE	8	50.380	32.690	24.140	1.00	0.00
ATOM	229	H	ILE	8	50.490	33.600	23.690	1.00	0.00
ATOM	230	CA	ILE	8	51.320	32.370	25.190	1.00	0.00
ATOM	231	HA	ILE	8	51.300	31.290	25.360	1.00	0.00
ATOM	232	CB	ILE	8	52.760	32.740	24.870	1.00	0.00
ATOM	233	HB	ILE	8	52.760	33.780	24.520	1.00	0.00
ATOM	234	CG2	ILE	8	53.590	32.690	26.150	1.00	0.00
ATOM	235	1HG2	ILE	8	53.220	33.410	26.880	1.00	0.00
ATOM	236	2HG2	ILE	8	53.610	31.680	26.540	1.00	0.00
ATOM	237	3HG2	ILE	8	54.600	33.080	25.970	1.00	0.00
ATOM	238	CG1	ILE	8	53.520	31.870	23.880	1.00	0.00
ATOM	239	1HG1	ILE	8	53.610	30.850	24.240	1.00	0.00
ATOM	240	2HG1	ILE	8	54.570	32.140	23.740	1.00	0.00
ATOM	241	CD	ILE	8	52.910	32.010	22.490	1.00	0.00
ATOM	242	HD1	ILE	8	51.940	31.520	22.420	1.00	0.00
ATOM	243	HD2	ILE	8	52.750	33.040	22.160	1.00	0.00
ATOM	244	HD3	ILE	8	53.550	31.510	21.750	1.00	0.00
ATOM	245	C	ILE	8	50.780	33.060	26.430	1.00	0.00
ATOM	246	O	ILE	8	50.580	34.270	26.380	1.00	0.00
ATOM	247	N	LEU	9	50.580	32.290	27.510	1.00	0.00
ATOM	248	H	LEU	9	50.790	31.300	27.490	1.00	0.00
ATOM	249	CA	LEU	9	50.240	32.830	28.810	1.00	0.00
ATOM	250	HA	LEU	9	49.300	33.360	28.700	1.00	0.00
ATOM	251	CB	LEU	9	50.030	31.660	29.760	1.00	0.00
ATOM	252	HB1	LEU	9	49.770	32.040	30.750	1.00	0.00
ATOM	253	HB2	LEU	9	50.990	31.180	29.950	1.00	0.00
ATOM	254	CG	LEU	9	48.970	30.620	29.400	1.00	0.00
ATOM	255	HG	LEU	9	49.130	30.210	28.410	1.00	0.00
ATOM	256	CD1	LEU	9	49.220	29.570	30.480	1.00	0.00
ATOM	257	1HD1	LEU	9	50.260	29.440	30.770	1.00	0.00
ATOM	258	2HD1	LEU	9	48.610	29.720	31.380	1.00	0.00
ATOM	259	3HD1	LEU	9	48.900	28.640	30.020	1.00	0.00
ATOM	260	CD2	LEU	9	47.560	31.170	29.620	1.00	0.00
ATOM	261	1HD2	LEU	9	46.850	30.380	29.370	1.00	0.00
ATOM	262	2HD2	LEU	9	47.460	31.480	30.660	1.00	0.00
ATOM	263	3HD2	LEU	9	47.450	32.050	28.980	1.00	0.00

ATOM	264	C	LEU	9	51.320	33.770	29.330	1.00	0.00
ATOM	265	O	LEU	9	52.420	33.300	29.630	1.00	0.00
ATOM	266	N	SER	10	51.080	35.080	29.370	1.00	0.00
ATOM	267	H	SER	10	50.130	35.390	29.190	1.00	0.00
ATOM	268	CA	SER	10	52.070	36.110	29.610	1.00	0.00
ATOM	269	HA	SER	10	53.020	35.720	29.250	1.00	0.00
ATOM	270	CB	SER	10	51.730	37.300	28.720	1.00	0.00
ATOM	271	HB1	SER	10	51.660	36.940	27.690	1.00	0.00
ATOM	272	HB2	SER	10	52.620	37.910	28.830	1.00	0.00
ATOM	273	OG	SER	10	50.590	38.060	29.080	1.00	0.00
ATOM	274	HG	SER	10	50.430	38.710	28.390	1.00	0.00
ATOM	275	C	SER	10	52.090	36.510	31.080	1.00	0.00
ATOM	276	O	SER	10	52.740	37.460	31.510	1.00	0.00
ATOM	277	N	SER	11	51.550	35.650	31.950	1.00	0.00
ATOM	278	H	SER	11	50.900	34.970	31.570	1.00	0.00
ATOM	279	CA	SER	11	51.590	35.840	33.390	1.00	0.00
ATOM	280	HA	SER	11	51.380	36.880	33.660	1.00	0.00
ATOM	281	CB	SER	11	50.480	35.020	34.030	1.00	0.00
ATOM	282	HB1	SER	11	50.370	35.430	35.040	1.00	0.00
ATOM	283	HB2	SER	11	50.800	33.980	34.060	1.00	0.00
ATOM	284	OG	SER	11	49.280	35.260	33.320	1.00	0.00
ATOM	285	HG	SER	11	49.030	36.190	33.310	1.00	0.00
ATOM	286	C	SER	11	52.970	35.510	33.940	1.00	0.00
ATOM	287	O	SER	11	53.680	34.610	33.510	1.00	0.00
ATOM	288	N	NH2	12	53.360	36.170	35.040	1.00	0.00
ATOM	289	H1	NH2	12	52.790	36.980	35.230	1.00	0.00
ATOM	290	H2	NH2	12	54.170	35.950	35.600	1.00	0.00
ATOM	291	CH3	ACE	1	38.920	26.880	42.810	1.00	0.00
ATOM	292	1HH3	ACE	1	39.650	27.620	42.500	1.00	0.00
ATOM	293	2HH3	ACE	1	39.030	26.630	43.860	1.00	0.00
ATOM	294	3HH3	ACE	1	39.010	25.940	42.270	1.00	0.00
ATOM	295	C	ACE	1	37.500	27.430	42.690	1.00	0.00
ATOM	296	O	ACE	1	36.540	26.730	43.000	1.00	0.00
ATOM	297	N	SER	2	37.400	28.690	42.270	1.00	0.00
ATOM	298	H	SER	2	38.240	29.190	42.010	1.00	0.00
ATOM	299	CA	SER	2	36.190	29.480	42.380	1.00	0.00
ATOM	300	HA	SER	2	35.390	28.790	42.650	1.00	0.00
ATOM	301	CB	SER	2	36.300	30.510	43.500	1.00	0.00
ATOM	302	HB1	SER	2	35.590	31.340	43.470	1.00	0.00
ATOM	303	HB2	SER	2	37.290	30.940	43.650	1.00	0.00
ATOM	304	OG	SER	2	35.890	29.900	44.700	1.00	0.00
ATOM	305	HG	SER	2	36.680	29.470	45.030	1.00	0.00
ATOM	306	C	SER	2	35.930	30.230	41.080	1.00	0.00
ATOM	307	O	SER	2	36.800	30.500	40.250	1.00	0.00

ATOM	308	N	ASN	3	34.640	30.510	40.880	1.00	0.00
ATOM	309	H	ASN	3	33.890	30.160	41.460	1.00	0.00
ATOM	310	CA	ASN	3	34.080	31.260	39.780	1.00	0.00
ATOM	311	HA	ASN	3	34.550	31.010	38.830	1.00	0.00
ATOM	312	CB	ASN	3	32.610	30.890	39.620	1.00	0.00
ATOM	313	HB1	ASN	3	32.120	31.460	38.830	1.00	0.00
ATOM	314	HB2	ASN	3	32.030	31.180	40.500	1.00	0.00
ATOM	315	CG	ASN	3	32.300	29.420	39.380	1.00	0.00
ATOM	316	OD1	ASN	3	32.470	28.820	38.330	1.00	0.00
ATOM	317	ND2	ASN	3	31.960	28.670	40.440	1.00	0.00
ATOM	318	1HD2	ASN	3	31.910	29.110	41.340	1.00	0.00
ATOM	319	2HD2	ASN	3	31.850	27.670	40.380	1.00	0.00
ATOM	320	C	ASN	3	34.220	32.750	40.080	1.00	0.00
ATOM	321	O	ASN	3	34.340	33.110	41.250	1.00	0.00
ATOM	322	N	ASN	4	34.010	33.640	39.110	1.00	0.00
ATOM	323	H	ASN	4	33.830	33.280	38.180	1.00	0.00
ATOM	324	CA	ASN	4	34.410	35.030	39.180	1.00	0.00
ATOM	325	HA	ASN	4	34.280	35.360	40.210	1.00	0.00
ATOM	326	CB	ASN	4	35.790	35.310	38.590	1.00	0.00
ATOM	327	HB1	ASN	4	35.970	36.370	38.430	1.00	0.00
ATOM	328	HB2	ASN	4	35.920	34.940	37.570	1.00	0.00
ATOM	329	CG	ASN	4	37.030	34.950	39.390	1.00	0.00
ATOM	330	OD1	ASN	4	37.150	33.990	40.140	1.00	0.00
ATOM	331	ND2	ASN	4	38.020	35.850	39.350	1.00	0.00
ATOM	332	1HD2	ASN	4	37.920	36.750	38.920	1.00	0.00
ATOM	333	2HD2	ASN	4	38.860	35.550	39.830	1.00	0.00
ATOM	334	C	ASN	4	33.350	35.840	38.450	1.00	0.00
ATOM	335	O	ASN	4	32.670	35.440	37.510	1.00	0.00
ATOM	336	N	PHE	5	33.150	37.050	38.980	1.00	0.00
ATOM	337	H	PHE	5	33.730	37.370	39.740	1.00	0.00
ATOM	338	CA	PHE	5	32.020	37.880	38.620	1.00	0.00
ATOM	339	HA	PHE	5	31.030	37.470	38.820	1.00	0.00
ATOM	340	CB	PHE	5	32.110	39.190	39.410	1.00	0.00
ATOM	341	HB1	PHE	5	32.820	39.850	38.910	1.00	0.00
ATOM	342	HB2	PHE	5	32.740	39.120	40.290	1.00	0.00
ATOM	343	CG	PHE	5	30.820	39.910	39.730	1.00	0.00
ATOM	344	CD1	PHE	5	30.080	39.540	40.850	1.00	0.00
ATOM	345	HD1	PHE	5	30.380	38.780	41.560	1.00	0.00
ATOM	346	CE1	PHE	5	28.860	40.210	40.990	1.00	0.00
ATOM	347	HE1	PHE	5	28.220	39.990	41.840	1.00	0.00
ATOM	348	CZ	PHE	5	28.310	41.130	40.080	1.00	0.00
ATOM	349	HZ	PHE	5	27.390	41.650	40.270	1.00	0.00
ATOM	350	CE2	PHE	5	29.080	41.440	38.950	1.00	0.00
ATOM	351	HE2	PHE	5	28.780	42.100	38.150	1.00	0.00

ATOM	352	CD2	PHE	5	30.360	40.880	38.830	1.00	0.00
ATOM	353	HD2	PHE	5	31.050	41.270	38.100	1.00	0.00
ATOM	354	C	PHE	5	32.010	38.090	37.110	1.00	0.00
ATOM	355	O	PHE	5	33.040	38.530	36.600	1.00	0.00
ATOM	356	N	GLY	6	30.870	38.010	36.420	1.00	0.00
ATOM	357	H	GLY	6	30.070	37.890	37.030	1.00	0.00
ATOM	358	CA	GLY	6	30.610	38.510	35.080	1.00	0.00
ATOM	359	HA1	GLY	6	29.830	37.890	34.630	1.00	0.00
ATOM	360	HA2	GLY	6	31.520	38.530	34.470	1.00	0.00
ATOM	361	C	GLY	6	30.070	39.930	35.050	1.00	0.00
ATOM	362	O	GLY	6	30.830	40.880	34.970	1.00	0.00
ATOM	363	N	ALA	7	28.740	40.080	35.030	1.00	0.00
ATOM	364	H	ALA	7	28.160	39.260	34.930	1.00	0.00
ATOM	365	CA	ALA	7	27.970	41.310	34.890	1.00	0.00
ATOM	366	HA	ALA	7	28.620	42.120	35.210	1.00	0.00
ATOM	367	CB	ALA	7	27.610	41.440	33.410	1.00	0.00
ATOM	368	HB1	ALA	7	28.470	41.790	32.840	1.00	0.00
ATOM	369	HB2	ALA	7	27.190	40.540	32.960	1.00	0.00
ATOM	370	HB3	ALA	7	27.010	42.310	33.160	1.00	0.00
ATOM	371	C	ALA	7	26.670	41.370	35.670	1.00	0.00
ATOM	372	O	ALA	7	26.100	40.320	35.980	1.00	0.00
ATOM	373	N	ILE	8	26.160	42.560	35.990	1.00	0.00
ATOM	374	H	ILE	8	26.700	43.360	35.710	1.00	0.00
ATOM	375	CA	ILE	8	24.940	42.850	36.720	1.00	0.00
ATOM	376	HA	ILE	8	24.920	42.210	37.600	1.00	0.00
ATOM	377	CB	ILE	8	24.910	44.340	37.090	1.00	0.00
ATOM	378	HB	ILE	8	24.750	44.940	36.200	1.00	0.00
ATOM	379	CG2	ILE	8	23.670	44.760	37.860	1.00	0.00
ATOM	380	1HG2	ILE	8	23.720	44.430	38.900	1.00	0.00
ATOM	381	2HG2	ILE	8	23.540	45.840	37.940	1.00	0.00
ATOM	382	3HG2	ILE	8	22.720	44.350	37.520	1.00	0.00
ATOM	383	CG1	ILE	8	26.200	44.940	37.630	1.00	0.00
ATOM	384	1HG1	ILE	8	26.030	45.970	37.940	1.00	0.00
ATOM	385	2HG1	ILE	8	26.970	44.930	36.850	1.00	0.00
ATOM	386	CD	ILE	8	26.710	44.130	38.820	1.00	0.00
ATOM	387	HD1	ILE	8	26.840	43.080	38.550	1.00	0.00
ATOM	388	HD2	ILE	8	27.600	44.600	39.240	1.00	0.00
ATOM	389	HD3	ILE	8	26.040	44.250	39.670	1.00	0.00
ATOM	390	C	ILE	8	23.730	42.580	35.850	1.00	0.00
ATOM	391	O	ILE	8	23.660	43.020	34.700	1.00	0.00
ATOM	392	N	LEU	9	22.820	41.760	36.360	1.00	0.00
ATOM	393	H	LEU	9	22.930	41.450	37.320	1.00	0.00
ATOM	394	CA	LEU	9	21.540	41.380	35.790	1.00	0.00
ATOM	395	HA	LEU	9	21.090	40.570	36.370	1.00	0.00

ATOM	396	CB	LEU	9	20.530	42.520	35.850	1.00	0.00
ATOM	397	HB1	LEU	9	19.700	42.310	35.170	1.00	0.00
ATOM	398	HB2	LEU	9	21.030	43.440	35.540	1.00	0.00
ATOM	399	CG	LEU	9	19.970	42.780	37.240	1.00	0.00
ATOM	400	HG	LEU	9	20.800	43.140	37.840	1.00	0.00
ATOM	401	CD1	LEU	9	18.980	43.940	37.290	1.00	0.00
ATOM	402	1HD1	LEU	9	18.840	44.210	38.340	1.00	0.00
ATOM	403	2HD1	LEU	9	19.370	44.890	36.910	1.00	0.00
ATOM	404	3HD1	LEU	9	18.000	43.620	36.940	1.00	0.00
ATOM	405	CD2	LEU	9	19.380	41.570	37.960	1.00	0.00
ATOM	406	1HD2	LEU	9	18.850	41.940	38.840	1.00	0.00
ATOM	407	2HD2	LEU	9	18.700	41.030	37.310	1.00	0.00
ATOM	408	3HD2	LEU	9	20.180	40.920	38.320	1.00	0.00
ATOM	409	C	LEU	9	21.690	40.840	34.370	1.00	0.00
ATOM	410	O	LEU	9	20.870	41.160	33.510	1.00	0.00
ATOM	411	N	SER	10	22.790	40.130	34.100	1.00	0.00
ATOM	412	H	SER	10	23.200	39.810	34.960	1.00	0.00
ATOM	413	CA	SER	10	23.210	39.570	32.830	1.00	0.00
ATOM	414	HA	SER	10	24.230	39.230	33.020	1.00	0.00
ATOM	415	CB	SER	10	22.290	38.410	32.500	1.00	0.00
ATOM	416	HB1	SER	10	22.580	38.010	31.530	1.00	0.00
ATOM	417	HB2	SER	10	21.250	38.730	32.490	1.00	0.00
ATOM	418	OG	SER	10	22.620	37.400	33.420	1.00	0.00
ATOM	419	HG	SER	10	22.030	36.680	33.180	1.00	0.00
ATOM	420	C	SER	10	23.370	40.540	31.660	1.00	0.00
ATOM	421	O	SER	10	23.390	40.140	30.500	1.00	0.00
ATOM	422	N	SER	11	23.510	41.850	31.890	1.00	0.00
ATOM	423	H	SER	11	23.680	42.170	32.830	1.00	0.00
ATOM	424	CA	SER	11	23.200	42.840	30.880	1.00	0.00
ATOM	425	HA	SER	11	22.330	42.390	30.410	1.00	0.00
ATOM	426	CB	SER	11	22.580	44.040	31.610	1.00	0.00
ATOM	427	HB1	SER	11	23.430	44.450	32.150	1.00	0.00
ATOM	428	HB2	SER	11	21.870	43.650	32.340	1.00	0.00
ATOM	429	OG	SER	11	22.010	44.960	30.710	1.00	0.00
ATOM	430	HG	SER	11	21.160	44.550	30.570	1.00	0.00
ATOM	431	C	SER	11	24.340	43.240	29.950	1.00	0.00
ATOM	432	O	SER	11	25.420	43.540	30.450	1.00	0.00
ATOM	433	N	NH2	12	24.030	43.230	28.650	1.00	0.00
ATOM	434	H1	NH2	12	23.090	43.020	28.360	1.00	0.00
ATOM	435	H2	NH2	12	24.660	43.720	28.030	1.00	0.00
ATOM	436	CH3	ACE	1	36.150	31.870	47.790	1.00	0.00
ATOM	437	1HH3	ACE	1	36.050	30.840	47.450	1.00	0.00
ATOM	438	2HH3	ACE	1	36.810	31.900	48.650	1.00	0.00
ATOM	439	3HH3	ACE	1	35.180	32.280	48.040	1.00	0.00

ATOM	440	C	ACE	1	36.730	32.640	46.610	1.00	0.00
ATOM	441	O	ACE	1	36.000	33.250	45.820	1.00	0.00
ATOM	442	N	SER	2	38.060	32.690	46.470	1.00	0.00
ATOM	443	H	SER	2	38.640	32.170	47.110	1.00	0.00
ATOM	444	CA	SER	2	38.770	33.470	45.480	1.00	0.00
ATOM	445	HA	SER	2	38.150	33.780	44.640	1.00	0.00
ATOM	446	CB	SER	2	39.290	34.810	46.010	1.00	0.00
ATOM	447	HB1	SER	2	39.940	35.370	45.350	1.00	0.00
ATOM	448	HB2	SER	2	39.880	34.690	46.920	1.00	0.00
ATOM	449	OG	SER	2	38.210	35.680	46.230	1.00	0.00
ATOM	450	HG	SER	2	38.170	35.920	47.160	1.00	0.00
ATOM	451	C	SER	2	39.970	32.740	44.920	1.00	0.00
ATOM	452	O	SER	2	40.280	31.580	45.200	1.00	0.00
ATOM	453	N	ASN	3	40.470	33.350	43.840	1.00	0.00
ATOM	454	H	ASN	3	40.130	34.290	43.690	1.00	0.00
ATOM	455	CA	ASN	3	41.540	32.870	43.000	1.00	0.00
ATOM	456	HA	ASN	3	42.040	31.990	43.420	1.00	0.00
ATOM	457	CB	ASN	3	40.980	32.450	41.650	1.00	0.00
ATOM	458	HB1	ASN	3	41.770	32.030	41.040	1.00	0.00
ATOM	459	HB2	ASN	3	40.680	33.350	41.090	1.00	0.00
ATOM	460	CG	ASN	3	39.770	31.540	41.750	1.00	0.00
ATOM	461	OD1	ASN	3	39.900	30.320	41.610	1.00	0.00
ATOM	462	ND2	ASN	3	38.550	32.080	41.740	1.00	0.00
ATOM	463	1HD2	ASN	3	38.450	33.090	41.730	1.00	0.00
ATOM	464	2HD2	ASN	3	37.780	31.560	41.350	1.00	0.00
ATOM	465	C	ASN	3	42.570	33.970	42.800	1.00	0.00
ATOM	466	O	ASN	3	42.190	35.130	42.980	1.00	0.00
ATOM	467	N	ASN	4	43.780	33.660	42.320	1.00	0.00
ATOM	468	H	ASN	4	43.870	32.670	42.160	1.00	0.00
ATOM	469	CA	ASN	4	44.840	34.560	41.900	1.00	0.00
ATOM	470	HA	ASN	4	44.340	35.520	41.800	1.00	0.00
ATOM	471	CB	ASN	4	45.910	34.670	42.990	1.00	0.00
ATOM	472	HB1	ASN	4	46.690	35.390	42.740	1.00	0.00
ATOM	473	HB2	ASN	4	46.400	33.700	43.040	1.00	0.00
ATOM	474	CG	ASN	4	45.300	34.990	44.340	1.00	0.00
ATOM	475	OD1	ASN	4	44.600	35.990	44.500	1.00	0.00
ATOM	476	ND2	ASN	4	45.630	34.160	45.340	1.00	0.00
ATOM	477	1HD2	ASN	4	46.320	33.470	45.090	1.00	0.00
ATOM	478	2HD2	ASN	4	45.510	34.530	46.270	1.00	0.00
ATOM	479	C	ASN	4	45.320	34.280	40.480	1.00	0.00
ATOM	480	O	ASN	4	45.260	33.180	39.950	1.00	0.00
ATOM	481	N	PHE	5	45.970	35.270	39.870	1.00	0.00
ATOM	482	H	PHE	5	45.840	36.200	40.250	1.00	0.00
ATOM	483	CA	PHE	5	46.810	35.170	38.700	1.00	0.00

ATOM	484	HA	PHE	5	46.740	34.180	38.230	1.00	0.00
ATOM	485	CB	PHE	5	46.400	36.200	37.650	1.00	0.00
ATOM	486	HB1	PHE	5	47.100	36.270	36.810	1.00	0.00
ATOM	487	HB2	PHE	5	46.400	37.150	38.180	1.00	0.00
ATOM	488	CG	PHE	5	45.010	36.010	37.070	1.00	0.00
ATOM	489	CD1	PHE	5	43.950	36.770	37.580	1.00	0.00
ATOM	490	HD1	PHE	5	44.120	37.510	38.350	1.00	0.00
ATOM	491	CE1	PHE	5	42.730	36.720	36.890	1.00	0.00
ATOM	492	HE1	PHE	5	41.940	37.310	37.320	1.00	0.00
ATOM	493	CZ	PHE	5	42.520	35.790	35.860	1.00	0.00
ATOM	494	HZ	PHE	5	41.530	35.710	35.430	1.00	0.00
ATOM	495	CE2	PHE	5	43.550	34.930	35.490	1.00	0.00
ATOM	496	HE2	PHE	5	43.350	34.170	34.740	1.00	0.00
ATOM	497	CD2	PHE	5	44.790	34.990	36.140	1.00	0.00
ATOM	498	HD2	PHE	5	45.550	34.340	35.750	1.00	0.00
ATOM	499	C	PHE	5	48.280	35.350	39.030	1.00	0.00
ATOM	500	O	PHE	5	48.630	36.000	40.020	1.00	0.00
ATOM	501	N	GLY	6	49.100	34.830	38.120	1.00	0.00
ATOM	502	H	GLY	6	48.620	34.300	37.400	1.00	0.00
ATOM	503	CA	GLY	6	50.540	34.990	38.080	1.00	0.00
ATOM	504	HA1	GLY	6	50.950	35.520	38.940	1.00	0.00
ATOM	505	HA2	GLY	6	50.800	35.730	37.310	1.00	0.00
ATOM	506	C	GLY	6	51.410	33.760	37.860	1.00	0.00
ATOM	507	O	GLY	6	52.610	33.920	38.050	1.00	0.00
ATOM	508	N	ALA	7	50.830	32.600	37.530	1.00	0.00
ATOM	509	H	ALA	7	49.820	32.610	37.520	1.00	0.00
ATOM	510	CA	ALA	7	51.550	31.360	37.350	1.00	0.00
ATOM	511	HA	ALA	7	50.870	30.510	37.230	1.00	0.00
ATOM	512	CB	ALA	7	52.240	31.460	35.990	1.00	0.00
ATOM	513	HB1	ALA	7	52.920	32.320	36.000	1.00	0.00
ATOM	514	HB2	ALA	7	52.750	30.550	35.680	1.00	0.00
ATOM	515	HB3	ALA	7	51.420	31.600	35.290	1.00	0.00
ATOM	516	C	ALA	7	52.530	31.010	38.460	1.00	0.00
ATOM	517	O	ALA	7	53.660	30.580	38.240	1.00	0.00
ATOM	518	N	ILE	8	52.160	31.240	39.720	1.00	0.00
ATOM	519	H	ILE	8	51.200	31.540	39.750	1.00	0.00
ATOM	520	CA	ILE	8	52.810	30.820	40.940	1.00	0.00
ATOM	521	HA	ILE	8	53.880	31.030	40.940	1.00	0.00
ATOM	522	CB	ILE	8	52.370	31.670	42.130	1.00	0.00
ATOM	523	HB	ILE	8	51.300	31.530	42.270	1.00	0.00
ATOM	524	CG2	ILE	8	52.970	31.140	43.430	1.00	0.00
ATOM	525	1HG2	ILE	8	52.950	30.060	43.570	1.00	0.00
ATOM	526	2HG2	ILE	8	54.050	31.230	43.340	1.00	0.00
ATOM	527	3HG2	ILE	8	52.600	31.670	44.310	1.00	0.00

ATOM	528	CG1	ILE	8	52.510	33.180	42.020	1.00	0.00
ATOM	529	1HG1	ILE	8	52.110	33.550	42.960	1.00	0.00
ATOM	530	2HG1	ILE	8	51.810	33.550	41.270	1.00	0.00
ATOM	531	CD	ILE	8	53.900	33.830	41.990	1.00	0.00
ATOM	532	HD1	ILE	8	54.450	33.430	41.140	1.00	0.00
ATOM	533	HD2	ILE	8	53.780	34.900	41.860	1.00	0.00
ATOM	534	HD3	ILE	8	54.360	33.550	42.940	1.00	0.00
ATOM	535	C	ILE	8	52.630	29.320	41.140	1.00	0.00
ATOM	536	O	ILE	8	51.510	28.810	41.020	1.00	0.00
ATOM	537	N	LEU	9	53.690	28.550	41.400	1.00	0.00
ATOM	538	H	LEU	9	54.590	29.000	41.530	1.00	0.00
ATOM	539	CA	LEU	9	53.700	27.100	41.330	1.00	0.00
ATOM	540	HA	LEU	9	52.830	26.690	40.820	1.00	0.00
ATOM	541	CB	LEU	9	54.900	26.530	40.580	1.00	0.00
ATOM	542	HB1	LEU	9	54.720	25.530	40.190	1.00	0.00
ATOM	543	HB2	LEU	9	55.690	26.510	41.330	1.00	0.00
ATOM	544	CG	LEU	9	55.500	27.400	39.480	1.00	0.00
ATOM	545	HG	LEU	9	55.690	28.420	39.790	1.00	0.00
ATOM	546	CD1	LEU	9	56.850	26.850	39.020	1.00	0.00
ATOM	547	1HD1	LEU	9	56.690	25.860	38.590	1.00	0.00
ATOM	548	2HD1	LEU	9	57.330	27.490	38.270	1.00	0.00
ATOM	549	3HD1	LEU	9	57.470	26.720	39.900	1.00	0.00
ATOM	550	CD2	LEU	9	54.550	27.490	38.290	1.00	0.00
ATOM	551	1HD2	LEU	9	53.630	27.970	38.620	1.00	0.00
ATOM	552	2HD2	LEU	9	54.820	28.170	37.490	1.00	0.00
ATOM	553	3HD2	LEU	9	54.340	26.500	37.880	1.00	0.00
ATOM	554	C	LEU	9	53.540	26.450	42.700	1.00	0.00
ATOM	555	O	LEU	9	52.860	25.430	42.730	1.00	0.00
ATOM	556	N	SER	10	53.930	27.120	43.780	1.00	0.00
ATOM	557	H	SER	10	54.460	27.940	43.530	1.00	0.00
ATOM	558	CA	SER	10	53.600	26.760	45.140	1.00	0.00
ATOM	559	HA	SER	10	53.750	25.690	45.310	1.00	0.00
ATOM	560	CB	SER	10	54.460	27.600	46.090	1.00	0.00
ATOM	561	HB1	SER	10	54.250	27.440	47.140	1.00	0.00
ATOM	562	HB2	SER	10	54.270	28.600	45.710	1.00	0.00
ATOM	563	OG	SER	10	55.780	27.170	45.840	1.00	0.00
ATOM	564	HG	SER	10	56.000	26.450	46.430	1.00	0.00
ATOM	565	C	SER	10	52.130	26.950	45.490	1.00	0.00
ATOM	566	O	SER	10	51.780	26.820	46.660	1.00	0.00
ATOM	567	N	SER	11	51.310	27.410	44.550	1.00	0.00
ATOM	568	H	SER	11	51.730	27.480	43.630	1.00	0.00
ATOM	569	CA	SER	11	49.890	27.640	44.750	1.00	0.00
ATOM	570	HA	SER	11	49.530	27.000	45.550	1.00	0.00
ATOM	571	CB	SER	11	49.410	29.060	45.030	1.00	0.00

ATOM	572	HB1	SER	11	48.320	29.130	44.940	1.00	0.00
ATOM	573	HB2	SER	11	49.670	29.810	44.290	1.00	0.00
ATOM	574	OG	SER	11	49.840	29.450	46.320	1.00	0.00
ATOM	575	HG	SER	11	49.580	30.370	46.390	1.00	0.00
ATOM	576	C	SER	11	49.020	27.090	43.630	1.00	0.00
ATOM	577	O	SER	11	47.930	27.580	43.330	1.00	0.00
ATOM	578	N	NH2	12	49.460	26.020	42.950	1.00	0.00
ATOM	579	H1	NH2	12	50.280	25.600	43.350	1.00	0.00
ATOM	580	H2	NH2	12	48.960	25.660	42.150	1.00	0.00
ATOM	581	CH3	ACE	1	28.050	44.690	28.050	1.00	0.00
ATOM	582	1HH3	ACE	1	28.780	44.860	27.260	1.00	0.00
ATOM	583	2HH3	ACE	1	27.000	44.480	27.820	1.00	0.00
ATOM	584	3HH3	ACE	1	28.010	45.670	28.530	1.00	0.00
ATOM	585	C	ACE	1	28.440	43.640	29.070	1.00	0.00
ATOM	586	O	ACE	1	29.310	43.820	29.920	1.00	0.00
ATOM	587	N	SER	2	27.880	42.430	28.940	1.00	0.00
ATOM	588	H	SER	2	27.070	42.310	28.340	1.00	0.00
ATOM	589	CA	SER	2	28.390	41.210	29.510	1.00	0.00
ATOM	590	HA	SER	2	29.110	41.500	30.270	1.00	0.00
ATOM	591	CB	SER	2	27.180	40.540	30.170	1.00	0.00
ATOM	592	HB1	SER	2	26.320	40.530	29.500	1.00	0.00
ATOM	593	HB2	SER	2	26.830	41.110	31.020	1.00	0.00
ATOM	594	OG	SER	2	27.640	39.230	30.460	1.00	0.00
ATOM	595	HG	SER	2	28.310	39.230	31.130	1.00	0.00
ATOM	596	C	SER	2	29.090	40.360	28.450	1.00	0.00
ATOM	597	O	SER	2	28.540	40.130	27.380	1.00	0.00
ATOM	598	N	ASN	3	30.300	39.920	28.760	1.00	0.00
ATOM	599	H	ASN	3	30.660	40.200	29.660	1.00	0.00
ATOM	600	CA	ASN	3	31.180	39.140	27.910	1.00	0.00
ATOM	601	HA	ASN	3	30.450	38.610	27.290	1.00	0.00
ATOM	602	CB	ASN	3	31.940	40.140	27.040	1.00	0.00
ATOM	603	HB1	ASN	3	32.800	39.690	26.550	1.00	0.00
ATOM	604	HB2	ASN	3	32.330	41.000	27.590	1.00	0.00
ATOM	605	CG	ASN	3	31.080	40.690	25.910	1.00	0.00
ATOM	606	OD1	ASN	3	30.600	41.810	26.090	1.00	0.00
ATOM	607	ND2	ASN	3	30.900	39.970	24.790	1.00	0.00
ATOM	608	1HD2	ASN	3	31.160	39.000	24.700	1.00	0.00
ATOM	609	2HD2	ASN	3	30.280	40.280	24.060	1.00	0.00
ATOM	610	C	ASN	3	32.140	38.210	28.630	1.00	0.00
ATOM	611	O	ASN	3	32.720	38.630	29.630	1.00	0.00
ATOM	612	N	ASN	4	32.260	36.940	28.220	1.00	0.00
ATOM	613	H	ASN	4	31.820	36.790	27.320	1.00	0.00
ATOM	614	CA	ASN	4	32.930	35.860	28.910	1.00	0.00
ATOM	615	HA	ASN	4	32.270	35.440	29.670	1.00	0.00

ATOM	616	CB	ASN	4	33.110	34.680	27.970	1.00	0.00
ATOM	617	HB1	ASN	4	33.700	34.850	27.070	1.00	0.00
ATOM	618	HB2	ASN	4	32.180	34.480	27.430	1.00	0.00
ATOM	619	CG	ASN	4	33.600	33.380	28.600	1.00	0.00
ATOM	620	OD1	ASN	4	33.870	33.180	29.790	1.00	0.00
ATOM	621	ND2	ASN	4	33.900	32.380	27.780	1.00	0.00
ATOM	622	1HD2	ASN	4	33.770	32.530	26.790	1.00	0.00
ATOM	623	2HD2	ASN	4	34.390	31.540	28.070	1.00	0.00
ATOM	624	C	ASN	4	34.210	36.280	29.620	1.00	0.00
ATOM	625	O	ASN	4	34.280	36.180	30.840	1.00	0.00
ATOM	626	N	PHE	5	35.100	36.860	28.810	1.00	0.00
ATOM	627	H	PHE	5	34.750	37.060	27.890	1.00	0.00
ATOM	628	CA	PHE	5	36.490	37.160	29.110	1.00	0.00
ATOM	629	HA	PHE	5	36.930	36.250	29.510	1.00	0.00
ATOM	630	CB	PHE	5	37.250	37.560	27.850	1.00	0.00
ATOM	631	HB1	PHE	5	36.920	38.510	27.420	1.00	0.00
ATOM	632	HB2	PHE	5	37.000	36.820	27.090	1.00	0.00
ATOM	633	CG	PHE	5	38.750	37.650	28.020	1.00	0.00
ATOM	634	CD1	PHE	5	39.540	36.500	28.150	1.00	0.00
ATOM	635	HD1	PHE	5	39.220	35.470	28.120	1.00	0.00
ATOM	636	CE1	PHE	5	40.890	36.710	28.470	1.00	0.00
ATOM	637	HE1	PHE	5	41.550	35.870	28.620	1.00	0.00
ATOM	638	CZ	PHE	5	41.490	37.970	28.540	1.00	0.00
ATOM	639	HZ	PHE	5	42.560	38.020	28.680	1.00	0.00
ATOM	640	CE2	PHE	5	40.690	39.100	28.360	1.00	0.00
ATOM	641	HE2	PHE	5	41.160	40.070	28.430	1.00	0.00
ATOM	642	CD2	PHE	5	39.320	38.920	28.120	1.00	0.00
ATOM	643	HD2	PHE	5	38.720	39.800	27.950	1.00	0.00
ATOM	644	C	PHE	5	36.590	38.180	30.230	1.00	0.00
ATOM	645	O	PHE	5	37.680	38.270	30.800	1.00	0.00
ATOM	646	N	GLY	6	35.500	38.750	30.750	1.00	0.00
ATOM	647	H	GLY	6	34.650	38.620	30.230	1.00	0.00
ATOM	648	CA	GLY	6	35.370	39.660	31.870	1.00	0.00
ATOM	649	HA1	GLY	6	34.310	39.890	31.980	1.00	0.00
ATOM	650	HA2	GLY	6	35.940	40.580	31.690	1.00	0.00
ATOM	651	C	GLY	6	35.960	39.120	33.160	1.00	0.00
ATOM	652	O	GLY	6	36.570	39.860	33.930	1.00	0.00
ATOM	653	N	ALA	7	35.830	37.820	33.430	1.00	0.00
ATOM	654	H	ALA	7	35.340	37.280	32.740	1.00	0.00
ATOM	655	CA	ALA	7	36.440	37.020	34.480	1.00	0.00
ATOM	656	HA	ALA	7	36.130	37.450	35.430	1.00	0.00
ATOM	657	CB	ALA	7	35.880	35.600	34.390	1.00	0.00
ATOM	658	HB1	ALA	7	34.800	35.560	34.240	1.00	0.00
ATOM	659	HB2	ALA	7	36.360	35.080	33.560	1.00	0.00

ATOM	660	HB3	ALA	7	36.030	35.110	35.360	1.00	0.00
ATOM	661	C	ALA	7	37.950	37.130	34.390	1.00	0.00
ATOM	662	O	ALA	7	38.650	37.150	35.400	1.00	0.00
ATOM	663	N	ILE	8	38.590	37.260	33.220	1.00	0.00
ATOM	664	H	ILE	8	38.020	37.410	32.400	1.00	0.00
ATOM	665	CA	ILE	8	40.020	37.450	33.160	1.00	0.00
ATOM	666	HA	ILE	8	40.550	37.280	34.100	1.00	0.00
ATOM	667	CB	ILE	8	40.650	36.470	32.170	1.00	0.00
ATOM	668	HB	ILE	8	40.290	36.800	31.190	1.00	0.00
ATOM	669	CG2	ILE	8	42.170	36.630	32.080	1.00	0.00
ATOM	670	1HG2	ILE	8	42.670	36.550	33.050	1.00	0.00
ATOM	671	2HG2	ILE	8	42.600	35.930	31.380	1.00	0.00
ATOM	672	3HG2	ILE	8	42.450	37.650	31.800	1.00	0.00
ATOM	673	CG1	ILE	8	40.260	35.030	32.470	1.00	0.00
ATOM	674	1HG1	ILE	8	39.170	35.020	32.530	1.00	0.00
ATOM	675	2HG1	ILE	8	40.590	34.750	33.460	1.00	0.00
ATOM	676	CD	ILE	8	40.700	33.980	31.450	1.00	0.00
ATOM	677	HD1	ILE	8	41.780	33.910	31.370	1.00	0.00
ATOM	678	HD2	ILE	8	40.430	32.950	31.690	1.00	0.00
ATOM	679	HD3	ILE	8	40.440	34.270	30.430	1.00	0.00
ATOM	680	C	ILE	8	40.460	38.880	32.900	1.00	0.00
ATOM	681	O	ILE	8	41.370	39.320	33.620	1.00	0.00
ATOM	682	N	LEU	9	39.760	39.660	32.070	1.00	0.00
ATOM	683	H	LEU	9	39.150	39.190	31.410	1.00	0.00
ATOM	684	CA	LEU	9	39.970	41.070	31.840	1.00	0.00
ATOM	685	HA	LEU	9	40.840	41.240	31.210	1.00	0.00
ATOM	686	CB	LEU	9	38.750	41.580	31.080	1.00	0.00
ATOM	687	HB1	LEU	9	37.850	41.520	31.690	1.00	0.00
ATOM	688	HB2	LEU	9	38.480	40.880	30.290	1.00	0.00
ATOM	689	CG	LEU	9	38.760	43.010	30.550	1.00	0.00
ATOM	690	HG	LEU	9	39.020	43.670	31.380	1.00	0.00
ATOM	691	CD1	LEU	9	39.710	43.150	29.370	1.00	0.00
ATOM	692	1HD1	LEU	9	40.720	43.130	29.780	1.00	0.00
ATOM	693	2HD1	LEU	9	39.640	42.280	28.730	1.00	0.00
ATOM	694	3HD1	LEU	9	39.450	44.060	28.840	1.00	0.00
ATOM	695	CD2	LEU	9	37.350	43.250	30.020	1.00	0.00
ATOM	696	1HD2	LEU	9	36.550	43.160	30.760	1.00	0.00
ATOM	697	2HD2	LEU	9	37.220	44.140	29.400	1.00	0.00
ATOM	698	3HD2	LEU	9	37.190	42.580	29.170	1.00	0.00
ATOM	699	C	LEU	9	40.120	41.940	33.080	1.00	0.00
ATOM	700	O	LEU	9	41.090	42.680	33.250	1.00	0.00
ATOM	701	N	SER	10	39.310	41.740	34.130	1.00	0.00
ATOM	702	H	SER	10	38.720	40.960	33.890	1.00	0.00
ATOM	703	CA	SER	10	39.160	42.390	35.420	1.00	0.00

ATOM	704	HA	SER	10	39.030	43.420	35.120	1.00	0.00
ATOM	705	CB	SER	10	37.860	41.900	36.050	1.00	0.00
ATOM	706	HB1	SER	10	37.730	42.380	37.020	1.00	0.00
ATOM	707	HB2	SER	10	37.900	40.820	36.120	1.00	0.00
ATOM	708	OG	SER	10	36.780	42.230	35.200	1.00	0.00
ATOM	709	HG	SER	10	36.790	41.630	34.450	1.00	0.00
ATOM	710	C	SER	10	40.340	42.330	36.380	1.00	0.00
ATOM	711	O	SER	10	40.310	42.750	37.540	1.00	0.00
ATOM	712	N	SER	11	41.420	41.650	35.980	1.00	0.00
ATOM	713	H	SER	11	41.350	41.110	35.130	1.00	0.00
ATOM	714	CA	SER	11	42.660	41.520	36.720	1.00	0.00
ATOM	715	HA	SER	11	42.420	41.470	37.790	1.00	0.00
ATOM	716	CB	SER	11	43.290	40.160	36.420	1.00	0.00
ATOM	717	HB1	SER	11	42.580	39.380	36.660	1.00	0.00
ATOM	718	HB2	SER	11	44.110	40.010	37.120	1.00	0.00
ATOM	719	OG	SER	11	43.660	40.190	35.060	1.00	0.00
ATOM	720	HG	SER	11	43.020	39.630	34.610	1.00	0.00
ATOM	721	C	SER	11	43.580	42.690	36.420	1.00	0.00
ATOM	722	O	SER	11	44.630	42.840	37.040	1.00	0.00
ATOM	723	N	NH2	12	43.210	43.650	35.580	1.00	0.00
ATOM	724	H1	NH2	12	42.370	43.610	35.010	1.00	0.00
ATOM	725	H2	NH2	12	43.840	44.430	35.440	1.00	0.00
ATOM	726	CH3	ACE	1	20.440	36.010	40.740	1.00	0.00
ATOM	727	1HH3	ACE	1	19.500	35.500	40.930	1.00	0.00
ATOM	728	2HH3	ACE	1	20.850	35.700	39.780	1.00	0.00
ATOM	729	3HH3	ACE	1	21.100	35.720	41.560	1.00	0.00
ATOM	730	C	ACE	1	20.280	37.520	40.750	1.00	0.00
ATOM	731	O	ACE	1	19.230	38.010	40.330	1.00	0.00
ATOM	732	N	SER	2	21.330	38.210	41.190	1.00	0.00
ATOM	733	H	SER	2	22.120	37.680	41.530	1.00	0.00
ATOM	734	CA	SER	2	21.580	39.590	40.810	1.00	0.00
ATOM	735	HA	SER	2	20.650	40.110	40.560	1.00	0.00
ATOM	736	CB	SER	2	22.100	40.330	42.030	1.00	0.00
ATOM	737	HB1	SER	2	22.410	41.350	41.780	1.00	0.00
ATOM	738	HB2	SER	2	23.060	39.900	42.320	1.00	0.00
ATOM	739	OG	SER	2	21.060	40.380	42.980	1.00	0.00
ATOM	740	HG	SER	2	20.890	39.490	43.300	1.00	0.00
ATOM	741	C	SER	2	22.590	39.980	39.740	1.00	0.00
ATOM	742	O	SER	2	22.610	41.070	39.170	1.00	0.00
ATOM	743	N	ASN	3	23.280	38.950	39.250	1.00	0.00
ATOM	744	H	ASN	3	23.000	38.020	39.540	1.00	0.00
ATOM	745	CA	ASN	3	24.370	39.020	38.300	1.00	0.00
ATOM	746	HA	ASN	3	24.170	39.620	37.410	1.00	0.00
ATOM	747	CB	ASN	3	25.610	39.550	39.020	1.00	0.00

ATOM	748	HB1	ASN	3	25.330	40.430	39.610	1.00	0.00
ATOM	749	HB2	ASN	3	26.350	39.910	38.310	1.00	0.00
ATOM	750	CG	ASN	3	26.110	38.530	40.020	1.00	0.00
ATOM	751	OD1	ASN	3	25.520	38.340	41.090	1.00	0.00
ATOM	752	ND2	ASN	3	27.190	37.830	39.670	1.00	0.00
ATOM	753	1HD2	ASN	3	27.680	37.940	38.790	1.00	0.00
ATOM	754	2HD2	ASN	3	27.400	37.030	40.250	1.00	0.00
ATOM	755	C	ASN	3	24.640	37.620	37.780	1.00	0.00
ATOM	756	O	ASN	3	24.150	36.620	38.310	1.00	0.00
ATOM	757	N	ASN	4	25.380	37.590	36.670	1.00	0.00
ATOM	758	H	ASN	4	25.700	38.480	36.300	1.00	0.00
ATOM	759	CA	ASN	4	25.990	36.370	36.180	1.00	0.00
ATOM	760	HA	ASN	4	25.500	35.560	36.720	1.00	0.00
ATOM	761	CB	ASN	4	25.680	36.160	34.700	1.00	0.00
ATOM	762	HB1	ASN	4	24.590	36.100	34.670	1.00	0.00
ATOM	763	HB2	ASN	4	26.010	35.170	34.360	1.00	0.00
ATOM	764	CG	ASN	4	26.280	37.140	33.710	1.00	0.00
ATOM	765	OD1	ASN	4	27.180	37.910	34.030	1.00	0.00
ATOM	766	ND2	ASN	4	25.730	37.130	32.490	1.00	0.00
ATOM	767	1HD2	ASN	4	25.010	36.460	32.250	1.00	0.00
ATOM	768	2HD2	ASN	4	26.250	37.600	31.770	1.00	0.00
ATOM	769	C	ASN	4	27.430	36.130	36.590	1.00	0.00
ATOM	770	O	ASN	4	28.120	37.000	37.120	1.00	0.00
ATOM	771	N	PHE	5	28.020	34.960	36.320	1.00	0.00
ATOM	772	H	PHE	5	27.370	34.260	36.000	1.00	0.00
ATOM	773	CA	PHE	5	29.400	34.550	36.520	1.00	0.00
ATOM	774	HA	PHE	5	29.920	35.490	36.670	1.00	0.00
ATOM	775	CB	PHE	5	29.390	33.670	37.770	1.00	0.00
ATOM	776	HB1	PHE	5	30.430	33.390	37.950	1.00	0.00
ATOM	777	HB2	PHE	5	28.890	32.730	37.540	1.00	0.00
ATOM	778	CG	PHE	5	28.880	34.280	39.050	1.00	0.00
ATOM	779	CD1	PHE	5	27.570	34.020	39.480	1.00	0.00
ATOM	780	HD1	PHE	5	26.930	33.320	38.960	1.00	0.00
ATOM	781	CE1	PHE	5	27.130	34.610	40.670	1.00	0.00
ATOM	782	HE1	PHE	5	26.130	34.400	41.000	1.00	0.00
ATOM	783	CZ	PHE	5	28.010	35.410	41.410	1.00	0.00
ATOM	784	HZ	PHE	5	27.840	35.800	42.400	1.00	0.00
ATOM	785	CE2	PHE	5	29.310	35.690	40.960	1.00	0.00
ATOM	786	HE2	PHE	5	30.010	36.260	41.540	1.00	0.00
ATOM	787	CD2	PHE	5	29.730	35.150	39.740	1.00	0.00
ATOM	788	HD2	PHE	5	30.780	35.300	39.510	1.00	0.00
ATOM	789	C	PHE	5	30.060	34.030	35.260	1.00	0.00
ATOM	790	O	PHE	5	29.500	34.040	34.160	1.00	0.00
ATOM	791	N	GLY	6	31.330	33.660	35.400	1.00	0.00

ATOM	792	H	GLY	6	31.770	33.590	36.310	1.00	0.00
ATOM	793	CA	GLY	6	32.300	33.240	34.410	1.00	0.00
ATOM	794	HA1	GLY	6	32.760	34.050	33.830	1.00	0.00
ATOM	795	HA2	GLY	6	31.730	32.520	33.820	1.00	0.00
ATOM	796	C	GLY	6	33.420	32.560	35.170	1.00	0.00
ATOM	797	O	GLY	6	33.400	32.610	36.400	1.00	0.00
ATOM	798	N	ALA	7	34.290	31.810	34.490	1.00	0.00
ATOM	799	H	ALA	7	34.300	31.740	33.480	1.00	0.00
ATOM	800	CA	ALA	7	35.450	31.190	35.120	1.00	0.00
ATOM	801	HA	ALA	7	35.370	31.240	36.210	1.00	0.00
ATOM	802	CB	ALA	7	35.350	29.700	34.840	1.00	0.00
ATOM	803	HB1	ALA	7	35.520	29.410	33.810	1.00	0.00
ATOM	804	HB2	ALA	7	36.190	29.240	35.370	1.00	0.00
ATOM	805	HB3	ALA	7	34.350	29.380	35.150	1.00	0.00
ATOM	806	C	ALA	7	36.740	31.880	34.700	1.00	0.00
ATOM	807	O	ALA	7	36.790	32.570	33.680	1.00	0.00
ATOM	808	N	ILE	8	37.800	31.650	35.480	1.00	0.00
ATOM	809	H	ILE	8	37.620	31.140	36.340	1.00	0.00
ATOM	810	CA	ILE	8	39.180	32.050	35.340	1.00	0.00
ATOM	811	HA	ILE	8	39.250	32.560	34.370	1.00	0.00
ATOM	812	CB	ILE	8	39.730	32.980	36.420	1.00	0.00
ATOM	813	HB	ILE	8	40.740	33.250	36.110	1.00	0.00
ATOM	814	CG2	ILE	8	38.890	34.250	36.260	1.00	0.00
ATOM	815	1HG2	ILE	8	37.890	34.110	36.670	1.00	0.00
ATOM	816	2HG2	ILE	8	39.420	34.950	36.910	1.00	0.00
ATOM	817	3HG2	ILE	8	38.880	34.600	35.230	1.00	0.00
ATOM	818	CG1	ILE	8	39.800	32.470	37.860	1.00	0.00
ATOM	819	1HG1	ILE	8	38.870	32.500	38.420	1.00	0.00
ATOM	820	2HG1	ILE	8	40.460	33.100	38.450	1.00	0.00
ATOM	821	CD	ILE	8	40.600	31.200	38.120	1.00	0.00
ATOM	822	HD1	ILE	8	39.950	30.370	37.850	1.00	0.00
ATOM	823	HD2	ILE	8	41.590	31.120	37.680	1.00	0.00
ATOM	824	HD3	ILE	8	40.810	31.140	39.190	1.00	0.00
ATOM	825	C	ILE	8	40.080	30.850	35.120	1.00	0.00
ATOM	826	O	ILE	8	41.230	31.000	34.720	1.00	0.00
ATOM	827	N	LEU	9	39.670	29.610	35.440	1.00	0.00
ATOM	828	H	LEU	9	38.730	29.480	35.780	1.00	0.00
ATOM	829	CA	LEU	9	40.410	28.380	35.250	1.00	0.00
ATOM	830	HA	LEU	9	41.460	28.670	35.270	1.00	0.00
ATOM	831	CB	LEU	9	40.090	27.330	36.310	1.00	0.00
ATOM	832	HB1	LEU	9	40.440	27.750	37.260	1.00	0.00
ATOM	833	HB2	LEU	9	40.620	26.410	36.080	1.00	0.00
ATOM	834	CG	LEU	9	38.620	26.930	36.400	1.00	0.00
ATOM	835	HG	LEU	9	37.940	27.790	36.430	1.00	0.00

ATOM	836	CD1	LEU	9	38.160	25.930	35.350	1.00	0.00
ATOM	837	1HD1	LEU	9	37.220	25.440	35.630	1.00	0.00
ATOM	838	2HD1	LEU	9	37.950	26.350	34.370	1.00	0.00
ATOM	839	3HD1	LEU	9	38.820	25.060	35.310	1.00	0.00
ATOM	840	CD2	LEU	9	38.530	26.370	37.820	1.00	0.00
ATOM	841	1HD2	LEU	9	37.450	26.300	37.970	1.00	0.00
ATOM	842	2HD2	LEU	9	38.860	25.340	37.870	1.00	0.00
ATOM	843	3HD2	LEU	9	39.040	26.940	38.590	1.00	0.00
ATOM	844	C	LEU	9	40.220	27.810	33.850	1.00	0.00
ATOM	845	O	LEU	9	39.270	28.230	33.210	1.00	0.00
ATOM	846	N	SER	10	41.020	26.830	33.420	1.00	0.00
ATOM	847	H	SER	10	41.690	26.390	34.040	1.00	0.00
ATOM	848	CA	SER	10	40.600	25.930	32.360	1.00	0.00
ATOM	849	HA	SER	10	39.560	25.690	32.590	1.00	0.00
ATOM	850	CB	SER	10	40.670	26.620	31.000	1.00	0.00
ATOM	851	HB1	SER	10	41.690	27.000	30.940	1.00	0.00
ATOM	852	HB2	SER	10	39.940	27.420	30.910	1.00	0.00
ATOM	853	OG	SER	10	40.570	25.690	29.940	1.00	0.00
ATOM	854	HG	SER	10	39.640	25.610	29.730	1.00	0.00
ATOM	855	C	SER	10	41.390	24.640	32.410	1.00	0.00
ATOM	856	O	SER	10	42.620	24.680	32.450	1.00	0.00
ATOM	857	N	SER	11	40.670	23.540	32.210	1.00	0.00
ATOM	858	H	SER	11	39.680	23.730	32.140	1.00	0.00
ATOM	859	CA	SER	11	41.260	22.220	32.330	1.00	0.00
ATOM	860	HA	SER	11	42.140	22.300	32.960	1.00	0.00
ATOM	861	CB	SER	11	40.240	21.270	32.960	1.00	0.00
ATOM	862	HB1	SER	11	40.640	20.320	33.310	1.00	0.00
ATOM	863	HB2	SER	11	39.490	20.930	32.250	1.00	0.00
ATOM	864	OG	SER	11	39.480	21.860	33.980	1.00	0.00
ATOM	865	HG	SER	11	38.770	22.310	33.520	1.00	0.00
ATOM	866	C	SER	11	41.780	21.680	31.010	1.00	0.00
ATOM	867	O	SER	11	42.540	20.720	30.880	1.00	0.00
ATOM	868	N	NH2	12	41.490	22.410	29.930	1.00	0.00
ATOM	869	H1	NH2	12	41.000	23.280	30.060	1.00	0.00
ATOM	870	H2	NH2	12	41.970	22.200	29.060	1.00	0.00
ATOM	871	CH3	ACE	1	43.580	29.620	50.210	1.00	0.00
ATOM	872	1HH3	ACE	1	43.960	28.600	50.180	1.00	0.00
ATOM	873	2HH3	ACE	1	44.440	30.280	50.280	1.00	0.00
ATOM	874	3HH3	ACE	1	42.830	29.900	50.950	1.00	0.00
ATOM	875	C	ACE	1	42.970	29.890	48.840	1.00	0.00
ATOM	876	O	ACE	1	41.930	29.310	48.540	1.00	0.00
ATOM	877	N	SER	2	43.630	30.770	48.080	1.00	0.00
ATOM	878	H	SER	2	44.410	31.280	48.450	1.00	0.00
ATOM	879	CA	SER	2	43.310	30.960	46.680	1.00	0.00

ATOM	880	HA	SER	2	42.370	30.450	46.470	1.00	0.00
ATOM	881	CB	SER	2	43.210	32.470	46.430	1.00	0.00
ATOM	882	HB1	SER	2	42.900	32.660	45.410	1.00	0.00
ATOM	883	HB2	SER	2	44.170	32.940	46.630	1.00	0.00
ATOM	884	OG	SER	2	42.300	33.120	47.280	1.00	0.00
ATOM	885	HG	SER	2	42.340	34.060	47.080	1.00	0.00
ATOM	886	C	SER	2	44.340	30.290	45.790	1.00	0.00
ATOM	887	O	SER	2	45.520	30.410	46.110	1.00	0.00
ATOM	888	N	ASN	3	43.940	29.640	44.690	1.00	0.00
ATOM	889	H	ASN	3	42.980	29.770	44.400	1.00	0.00
ATOM	890	CA	ASN	3	44.770	28.990	43.700	1.00	0.00
ATOM	891	HA	ASN	3	45.690	28.570	44.080	1.00	0.00
ATOM	892	CB	ASN	3	43.950	27.860	43.090	1.00	0.00
ATOM	893	HB1	ASN	3	44.490	27.430	42.240	1.00	0.00
ATOM	894	HB2	ASN	3	43.030	28.250	42.660	1.00	0.00
ATOM	895	CG	ASN	3	43.690	26.800	44.150	1.00	0.00
ATOM	896	OD1	ASN	3	44.510	25.960	44.510	1.00	0.00
ATOM	897	ND2	ASN	3	42.490	26.800	44.740	1.00	0.00
ATOM	898	1HD2	ASN	3	41.840	27.510	44.450	1.00	0.00
ATOM	899	2HD2	ASN	3	42.240	26.130	45.460	1.00	0.00
ATOM	900	C	ASN	3	45.270	29.990	42.660	1.00	0.00
ATOM	901	O	ASN	3	44.590	30.990	42.420	1.00	0.00
ATOM	902	N	ASN	4	46.480	29.780	42.150	1.00	0.00
ATOM	903	H	ASN	4	46.940	29.000	42.590	1.00	0.00
ATOM	904	CA	ASN	4	47.150	30.700	41.250	1.00	0.00
ATOM	905	HA	ASN	4	46.660	31.670	41.310	1.00	0.00
ATOM	906	CB	ASN	4	48.580	30.830	41.750	1.00	0.00
ATOM	907	HB1	ASN	4	49.140	29.950	41.420	1.00	0.00
ATOM	908	HB2	ASN	4	48.540	30.800	42.840	1.00	0.00
ATOM	909	CG	ASN	4	49.120	32.160	41.230	1.00	0.00
ATOM	910	OD1	ASN	4	49.510	32.310	40.080	1.00	0.00
ATOM	911	ND2	ASN	4	49.130	33.220	42.040	1.00	0.00
ATOM	912	1HD2	ASN	4	48.630	33.250	42.920	1.00	0.00
ATOM	913	2HD2	ASN	4	49.450	34.080	41.620	1.00	0.00
ATOM	914	C	ASN	4	47.060	30.120	39.840	1.00	0.00
ATOM	915	O	ASN	4	47.330	28.930	39.690	1.00	0.00
ATOM	916	N	PHE	5	46.910	30.960	38.820	1.00	0.00
ATOM	917	H	PHE	5	46.720	31.950	38.970	1.00	0.00
ATOM	918	CA	PHE	5	46.800	30.580	37.430	1.00	0.00
ATOM	919	HA	PHE	5	47.130	29.550	37.300	1.00	0.00
ATOM	920	CB	PHE	5	45.330	30.550	37.010	1.00	0.00
ATOM	921	HB1	PHE	5	45.270	30.380	35.930	1.00	0.00
ATOM	922	HB2	PHE	5	44.880	31.540	37.040	1.00	0.00
ATOM	923	CG	PHE	5	44.480	29.580	37.790	1.00	0.00

ATOM	924	CD1	PHE	5	43.880	30.060	38.960	1.00	0.00
ATOM	925	HD1	PHE	5	44.100	31.050	39.310	1.00	0.00
ATOM	926	CE1	PHE	5	43.060	29.180	39.690	1.00	0.00
ATOM	927	HE1	PHE	5	42.740	29.550	40.650	1.00	0.00
ATOM	928	CZ	PHE	5	42.640	27.980	39.100	1.00	0.00
ATOM	929	HZ	PHE	5	41.940	27.310	39.570	1.00	0.00
ATOM	930	CE2	PHE	5	43.130	27.610	37.840	1.00	0.00
ATOM	931	HE2	PHE	5	42.800	26.670	37.420	1.00	0.00
ATOM	932	CD2	PHE	5	44.100	28.380	37.180	1.00	0.00
ATOM	933	HD2	PHE	5	44.430	28.130	36.180	1.00	0.00
ATOM	934	C	PHE	5	47.530	31.440	36.410	1.00	0.00
ATOM	935	O	PHE	5	47.860	32.580	36.720	1.00	0.00
ATOM	936	N	GLY	6	47.850	30.910	35.230	1.00	0.00
ATOM	937	H	GLY	6	47.580	29.950	35.080	1.00	0.00
ATOM	938	CA	GLY	6	48.280	31.700	34.090	1.00	0.00
ATOM	939	HA1	GLY	6	48.850	31.100	33.370	1.00	0.00
ATOM	940	HA2	GLY	6	48.790	32.620	34.360	1.00	0.00
ATOM	941	C	GLY	6	47.090	32.140	33.240	1.00	0.00
ATOM	942	O	GLY	6	46.110	31.430	33.030	1.00	0.00
ATOM	943	N	ALA	7	47.270	33.260	32.520	1.00	0.00
ATOM	944	H	ALA	7	48.110	33.810	32.680	1.00	0.00
ATOM	945	CA	ALA	7	46.300	33.890	31.650	1.00	0.00
ATOM	946	HA	ALA	7	45.600	33.170	31.230	1.00	0.00
ATOM	947	CB	ALA	7	45.420	34.850	32.440	1.00	0.00
ATOM	948	HB1	ALA	7	45.970	35.730	32.760	1.00	0.00
ATOM	949	HB2	ALA	7	44.700	35.270	31.740	1.00	0.00
ATOM	950	HB3	ALA	7	44.990	34.430	33.350	1.00	0.00
ATOM	951	C	ALA	7	46.990	34.520	30.450	1.00	0.00
ATOM	952	O	ALA	7	48.190	34.780	30.440	1.00	0.00
ATOM	953	N	ILE	8	46.320	34.930	29.370	1.00	0.00
ATOM	954	H	ILE	8	45.330	34.710	29.400	1.00	0.00
ATOM	955	CA	ILE	8	46.740	35.780	28.280	1.00	0.00
ATOM	956	HA	ILE	8	47.740	35.390	28.090	1.00	0.00
ATOM	957	CB	ILE	8	45.910	35.650	27.010	1.00	0.00
ATOM	958	HB	ILE	8	46.330	36.180	26.150	1.00	0.00
ATOM	959	CG2	ILE	8	46.010	34.210	26.500	1.00	0.00
ATOM	960	1HG2	ILE	8	45.550	34.130	25.520	1.00	0.00
ATOM	961	2HG2	ILE	8	47.020	33.810	26.460	1.00	0.00
ATOM	962	3HG2	ILE	8	45.420	33.630	27.210	1.00	0.00
ATOM	963	CG1	ILE	8	44.440	36.060	27.130	1.00	0.00
ATOM	964	1HG1	ILE	8	44.470	37.100	27.450	1.00	0.00
ATOM	965	2HG1	ILE	8	43.910	35.420	27.840	1.00	0.00
ATOM	966	CD	ILE	8	43.480	36.030	25.950	1.00	0.00
ATOM	967	HD1	ILE	8	43.620	36.910	25.320	1.00	0.00

ATOM	968	HD2	ILE	8	43.650	35.190	25.290	1.00	0.00
ATOM	969	HD3	ILE	8	42.470	36.000	26.350	1.00	0.00
ATOM	970	C	ILE	8	46.990	37.220	28.720	1.00	0.00
ATOM	971	O	ILE	8	46.990	38.120	27.880	1.00	0.00
ATOM	972	N	LEU	9	47.110	37.530	30.010	1.00	0.00
ATOM	973	H	LEU	9	47.390	36.740	30.570	1.00	0.00
ATOM	974	CA	LEU	9	47.240	38.860	30.560	1.00	0.00
ATOM	975	HA	LEU	9	47.470	39.560	29.750	1.00	0.00
ATOM	976	CB	LEU	9	46.060	39.350	31.390	1.00	0.00
ATOM	977	HB1	LEU	9	46.380	40.260	31.890	1.00	0.00
ATOM	978	HB2	LEU	9	45.830	38.630	32.170	1.00	0.00
ATOM	979	CG	LEU	9	44.810	39.550	30.540	1.00	0.00
ATOM	980	HG	LEU	9	44.320	38.680	30.100	1.00	0.00
ATOM	981	CD1	LEU	9	43.830	40.190	31.530	1.00	0.00
ATOM	982	1HD1	LEU	9	44.190	41.190	31.790	1.00	0.00
ATOM	983	2HD1	LEU	9	42.890	40.330	31.000	1.00	0.00
ATOM	984	3HD1	LEU	9	43.750	39.460	32.330	1.00	0.00
ATOM	985	CD2	LEU	9	45.070	40.540	29.410	1.00	0.00
ATOM	986	1HD2	LEU	9	45.490	39.920	28.620	1.00	0.00
ATOM	987	2HD2	LEU	9	44.260	41.180	29.070	1.00	0.00
ATOM	988	3HD2	LEU	9	45.730	41.300	29.820	1.00	0.00
ATOM	989	C	LEU	9	48.430	38.800	31.500	1.00	0.00
ATOM	990	O	LEU	9	48.570	37.770	32.160	1.00	0.00
ATOM	991	N	SER	10	49.220	39.870	31.670	1.00	0.00
ATOM	992	H	SER	10	49.090	40.700	31.110	1.00	0.00
ATOM	993	CA	SER	10	50.410	39.710	32.480	1.00	0.00
ATOM	994	HA	SER	10	50.740	38.680	32.400	1.00	0.00
ATOM	995	CB	SER	10	51.490	40.570	31.830	1.00	0.00
ATOM	996	HB1	SER	10	51.570	40.310	30.780	1.00	0.00
ATOM	997	HB2	SER	10	52.480	40.570	32.280	1.00	0.00
ATOM	998	OG	SER	10	51.100	41.920	31.690	1.00	0.00
ATOM	999	HG	SER	10	51.860	42.440	31.410	1.00	0.00
ATOM	1000	C	SER	10	50.200	39.930	33.970	1.00	0.00
ATOM	1001	O	SER	10	51.080	40.320	34.730	1.00	0.00
ATOM	1002	N	SER	11	48.950	39.840	34.430	1.00	0.00
ATOM	1003	H	SER	11	48.250	39.620	33.730	1.00	0.00
ATOM	1004	CA	SER	11	48.380	39.660	35.750	1.00	0.00
ATOM	1005	HA	SER	11	48.350	40.630	36.240	1.00	0.00
ATOM	1006	CB	SER	11	46.910	39.290	35.560	1.00	0.00
ATOM	1007	HB1	SER	11	46.300	39.510	36.440	1.00	0.00
ATOM	1008	HB2	SER	11	46.860	38.280	35.170	1.00	0.00
ATOM	1009	OG	SER	11	46.360	40.130	34.570	1.00	0.00
ATOM	1010	HG	SER	11	45.420	39.940	34.540	1.00	0.00
ATOM	1011	C	SER	11	49.080	38.620	36.610	1.00	0.00

ATOM	1012	O	SER	11	49.400	37.500	36.230	1.00	0.00
ATOM	1013	N	NH2	12	49.350	38.930	37.890	1.00	0.00
ATOM	1014	H1	NH2	12	49.000	39.820	38.240	1.00	0.00
ATOM	1015	H2	NH2	12	49.850	38.230	38.410	1.00	0.00
ATOM	1016	CH3	ACE	1	17.660	26.420	42.210	1.00	0.00
ATOM	1017	1HH3	ACE	1	17.410	27.020	41.330	1.00	0.00
ATOM	1018	2HH3	ACE	1	18.540	25.790	42.050	1.00	0.00
ATOM	1019	3HH3	ACE	1	16.790	25.790	42.380	1.00	0.00
ATOM	1020	C	ACE	1	17.900	27.230	43.470	1.00	0.00
ATOM	1021	O	ACE	1	18.890	27.950	43.580	1.00	0.00
ATOM	1022	N	SER	2	16.960	27.180	44.410	1.00	0.00
ATOM	1023	H	SER	2	16.100	26.670	44.230	1.00	0.00
ATOM	1024	CA	SER	2	17.260	27.690	45.730	1.00	0.00
ATOM	1025	HA	SER	2	17.450	28.760	45.670	1.00	0.00
ATOM	1026	CB	SER	2	16.190	27.440	46.790	1.00	0.00
ATOM	1027	HB1	SER	2	16.680	27.260	47.750	1.00	0.00
ATOM	1028	HB2	SER	2	15.620	26.560	46.510	1.00	0.00
ATOM	1029	OG	SER	2	15.460	28.630	46.970	1.00	0.00
ATOM	1030	HG	SER	2	14.680	28.290	47.410	1.00	0.00
ATOM	1031	C	SER	2	18.490	26.980	46.250	1.00	0.00
ATOM	1032	O	SER	2	18.630	25.750	46.210	1.00	0.00
ATOM	1033	N	ASN	3	19.350	27.810	46.860	1.00	0.00
ATOM	1034	H	ASN	3	19.190	28.800	46.940	1.00	0.00
ATOM	1035	CA	ASN	3	20.660	27.410	47.310	1.00	0.00
ATOM	1036	HA	ASN	3	21.190	28.330	47.540	1.00	0.00
ATOM	1037	CB	ASN	3	20.510	26.620	48.610	1.00	0.00
ATOM	1038	HB1	ASN	3	19.980	25.670	48.480	1.00	0.00
ATOM	1039	HB2	ASN	3	19.970	27.180	49.370	1.00	0.00
ATOM	1040	CG	ASN	3	21.810	26.300	49.340	1.00	0.00
ATOM	1041	OD1	ASN	3	22.170	25.160	49.620	1.00	0.00
ATOM	1042	ND2	ASN	3	22.560	27.310	49.780	1.00	0.00
ATOM	1043	1HD2	ASN	3	22.120	28.210	49.960	1.00	0.00
ATOM	1044	2HD2	ASN	3	23.290	26.990	50.390	1.00	0.00
ATOM	1045	C	ASN	3	21.580	26.770	46.270	1.00	0.00
ATOM	1046	O	ASN	3	22.430	25.960	46.610	1.00	0.00
ATOM	1047	N	ASN	4	21.450	27.040	44.970	1.00	0.00
ATOM	1048	H	ASN	4	20.650	27.590	44.720	1.00	0.00
ATOM	1049	CA	ASN	4	22.430	26.700	43.950	1.00	0.00
ATOM	1050	HA	ASN	4	23.180	26.070	44.410	1.00	0.00
ATOM	1051	CB	ASN	4	21.660	26.010	42.830	1.00	0.00
ATOM	1052	HB1	ASN	4	20.790	26.600	42.520	1.00	0.00
ATOM	1053	HB2	ASN	4	21.220	25.050	43.110	1.00	0.00
ATOM	1054	CG	ASN	4	22.510	25.730	41.610	1.00	0.00
ATOM	1055	OD1	ASN	4	23.540	25.050	41.650	1.00	0.00

ATOM	1056	ND2	ASN	4	22.100	26.260	40.460	1.00	0.00
ATOM	1057	1HD2	ASN	4	21.360	26.950	40.410	1.00	0.00
ATOM	1058	2HD2	ASN	4	22.770	26.200	39.700	1.00	0.00
ATOM	1059	C	ASN	4	23.040	28.020	43.500	1.00	0.00
ATOM	1060	O	ASN	4	22.520	29.110	43.730	1.00	0.00
ATOM	1061	N	PHE	5	24.290	27.990	43.040	1.00	0.00
ATOM	1062	H	PHE	5	24.620	27.050	42.890	1.00	0.00
ATOM	1063	CA	PHE	5	25.020	29.060	42.390	1.00	0.00
ATOM	1064	HA	PHE	5	25.020	29.850	43.150	1.00	0.00
ATOM	1065	CB	PHE	5	26.480	28.620	42.360	1.00	0.00
ATOM	1066	HB1	PHE	5	26.630	28.000	41.480	1.00	0.00
ATOM	1067	HB2	PHE	5	26.660	28.030	43.260	1.00	0.00
ATOM	1068	CG	PHE	5	27.460	29.760	42.280	1.00	0.00
ATOM	1069	CD1	PHE	5	28.020	30.390	43.400	1.00	0.00
ATOM	1070	HD1	PHE	5	27.890	29.940	44.370	1.00	0.00
ATOM	1071	CE1	PHE	5	28.840	31.500	43.180	1.00	0.00
ATOM	1072	HE1	PHE	5	29.330	31.940	44.030	1.00	0.00
ATOM	1073	CZ	PHE	5	28.980	32.080	41.910	1.00	0.00
ATOM	1074	HZ	PHE	5	29.500	33.010	41.740	1.00	0.00
ATOM	1075	CE2	PHE	5	28.580	31.310	40.810	1.00	0.00
ATOM	1076	HE2	PHE	5	28.850	31.650	39.820	1.00	0.00
ATOM	1077	CD2	PHE	5	27.770	30.180	40.980	1.00	0.00
ATOM	1078	HD2	PHE	5	27.500	29.490	40.190	1.00	0.00
ATOM	1079	C	PHE	5	24.500	29.540	41.040	1.00	0.00
ATOM	1080	O	PHE	5	24.000	28.780	40.220	1.00	0.00
ATOM	1081	N	GLY	6	24.670	30.850	40.890	1.00	0.00
ATOM	1082	H	GLY	6	25.010	31.480	41.610	1.00	0.00
ATOM	1083	CA	GLY	6	24.170	31.510	39.700	1.00	0.00
ATOM	1084	HA1	GLY	6	24.580	32.520	39.610	1.00	0.00
ATOM	1085	HA2	GLY	6	23.090	31.470	39.750	1.00	0.00
ATOM	1086	C	GLY	6	24.770	31.030	38.380	1.00	0.00
ATOM	1087	O	GLY	6	25.540	30.070	38.390	1.00	0.00
ATOM	1088	N	ALA	7	24.330	31.620	37.270	1.00	0.00
ATOM	1089	H	ALA	7	23.710	32.410	37.330	1.00	0.00
ATOM	1090	CA	ALA	7	24.650	31.160	35.940	1.00	0.00
ATOM	1091	HA	ALA	7	24.570	30.080	35.810	1.00	0.00
ATOM	1092	CB	ALA	7	23.630	31.830	35.020	1.00	0.00
ATOM	1093	HB1	ALA	7	23.610	32.900	35.220	1.00	0.00
ATOM	1094	HB2	ALA	7	23.920	31.640	33.990	1.00	0.00
ATOM	1095	HB3	ALA	7	22.640	31.410	35.220	1.00	0.00
ATOM	1096	C	ALA	7	26.050	31.540	35.470	1.00	0.00
ATOM	1097	O	ALA	7	26.610	32.530	35.940	1.00	0.00
ATOM	1098	N	ILE	8	26.680	30.630	34.730	1.00	0.00
ATOM	1099	H	ILE	8	26.140	29.830	34.470	1.00	0.00

ATOM	1100	CA	ILE	8	28.070	30.780	34.330	1.00	0.00
ATOM	1101	HA	ILE	8	28.520	31.670	34.770	1.00	0.00
ATOM	1102	CB	ILE	8	28.900	29.590	34.800	1.00	0.00
ATOM	1103	HB	ILE	8	28.710	28.740	34.140	1.00	0.00
ATOM	1104	CG2	ILE	8	30.410	29.740	34.610	1.00	0.00
ATOM	1105	1HG2	ILE	8	30.750	30.540	35.270	1.00	0.00
ATOM	1106	2HG2	ILE	8	30.980	28.820	34.760	1.00	0.00
ATOM	1107	3HG2	ILE	8	30.630	30.060	33.590	1.00	0.00
ATOM	1108	CG1	ILE	8	28.710	29.030	36.210	1.00	0.00
ATOM	1109	1HG1	ILE	8	29.450	28.270	36.440	1.00	0.00
ATOM	1110	2HG1	ILE	8	27.690	28.630	36.200	1.00	0.00
ATOM	1111	CD	ILE	8	28.930	30.070	37.290	1.00	0.00
ATOM	1112	HD1	ILE	8	28.950	29.690	38.310	1.00	0.00
ATOM	1113	HD2	ILE	8	29.880	30.590	37.170	1.00	0.00
ATOM	1114	HD3	ILE	8	28.100	30.790	37.300	1.00	0.00
ATOM	1115	C	ILE	8	28.150	30.800	32.810	1.00	0.00
ATOM	1116	O	ILE	8	27.670	29.980	32.040	1.00	0.00
ATOM	1117	N	LEU	9	28.870	31.820	32.310	1.00	0.00
ATOM	1118	H	LEU	9	29.100	32.600	32.910	1.00	0.00
ATOM	1119	CA	LEU	9	29.140	32.030	30.910	1.00	0.00
ATOM	1120	HA	LEU	9	28.270	32.040	30.250	1.00	0.00
ATOM	1121	CB	LEU	9	29.740	33.420	30.690	1.00	0.00
ATOM	1122	HB1	LEU	9	30.040	33.510	29.640	1.00	0.00
ATOM	1123	HB2	LEU	9	30.640	33.590	31.260	1.00	0.00
ATOM	1124	CG	LEU	9	28.760	34.510	31.100	1.00	0.00
ATOM	1125	HG	LEU	9	28.120	34.390	31.970	1.00	0.00
ATOM	1126	CD1	LEU	9	29.590	35.730	31.510	1.00	0.00
ATOM	1127	1HD1	LEU	9	30.290	35.400	32.280	1.00	0.00
ATOM	1128	2HD1	LEU	9	30.040	35.980	30.550	1.00	0.00
ATOM	1129	3HD1	LEU	9	29.040	36.540	31.990	1.00	0.00
ATOM	1130	CD2	LEU	9	27.840	34.870	29.930	1.00	0.00
ATOM	1131	1HD2	LEU	9	28.410	34.970	29.010	1.00	0.00
ATOM	1132	2HD2	LEU	9	27.120	34.050	29.820	1.00	0.00
ATOM	1133	3HD2	LEU	9	27.270	35.790	30.040	1.00	0.00
ATOM	1134	C	LEU	9	30.070	30.950	30.360	1.00	0.00
ATOM	1135	O	LEU	9	31.070	30.630	30.990	1.00	0.00
ATOM	1136	N	SER	10	29.810	30.430	29.160	1.00	0.00
ATOM	1137	H	SER	10	29.150	30.800	28.480	1.00	0.00
ATOM	1138	CA	SER	10	30.510	29.320	28.550	1.00	0.00
ATOM	1139	HA	SER	10	29.960	29.040	27.650	1.00	0.00
ATOM	1140	CB	SER	10	31.850	29.710	27.950	1.00	0.00
ATOM	1141	HB1	SER	10	32.240	28.830	27.450	1.00	0.00
ATOM	1142	HB2	SER	10	32.610	30.030	28.680	1.00	0.00
ATOM	1143	OG	SER	10	31.590	30.790	27.080	1.00	0.00

ATOM	1144	HG	SER	10	32.270	30.970	26.430	1.00	0.00
ATOM	1145	C	SER	10	30.480	28.120	29.480	1.00	0.00
ATOM	1146	O	SER	10	31.560	27.580	29.690	1.00	0.00
ATOM	1147	N	SER	11	29.310	27.850	30.080	1.00	0.00
ATOM	1148	H	SER	11	28.580	28.530	30.000	1.00	0.00
ATOM	1149	CA	SER	11	28.950	26.530	30.570	1.00	0.00
ATOM	1150	HA	SER	11	29.380	25.770	29.930	1.00	0.00
ATOM	1151	CB	SER	11	29.280	26.430	32.060	1.00	0.00
ATOM	1152	HB1	SER	11	28.680	27.080	32.690	1.00	0.00
ATOM	1153	HB2	SER	11	30.320	26.690	32.270	1.00	0.00
ATOM	1154	OG	SER	11	29.140	25.100	32.490	1.00	0.00
ATOM	1155	HG	SER	11	29.630	24.530	31.890	1.00	0.00
ATOM	1156	C	SER	11	27.440	26.340	30.440	1.00	0.00
ATOM	1157	O	SER	11	26.650	27.260	30.630	1.00	0.00
ATOM	1158	N	NH2	12	27.030	25.190	29.910	1.00	0.00
ATOM	1159	H1	NH2	12	27.810	24.550	29.880	1.00	0.00
ATOM	1160	H2	NH2	12	26.040	25.030	30.070	1.00	0.00
ATOM	1161	OW	SOL	13	47.990	42.650	22.020	1.00	0.00
ATOM	1162	HW1	SOL	13	48.800	42.130	21.970	1.00	0.00
ATOM	1163	HW2	SOL	13	48.290	43.520	22.290	1.00	0.00
ATOM	1164	OW	SOL	14	18.210	24.490	14.970	1.00	0.00
ATOM	1165	HW1	SOL	14	18.540	23.860	15.620	1.00	0.00
ATOM	1166	HW2	SOL	14	17.810	23.950	14.290	1.00	0.00
ATOM	1167	OW	SOL	15	24.860	16.490	41.570	1.00	0.00
ATOM	1168	HW1	SOL	15	24.760	16.780	42.470	1.00	0.00
ATOM	1169	HW2	SOL	15	24.570	15.570	41.570	1.00	0.00
ATOM	1170	OW	SOL	16	28.170	39.790	4.750	1.00	0.00
ATOM	1171	HW1	SOL	16	28.730	40.510	4.460	1.00	0.00
ATOM	1172	HW2	SOL	16	27.280	40.150	4.700	1.00	0.00
ATOM	1173	OW	SOL	17	16.260	40.290	50.440	1.00	0.00
ATOM	1174	HW1	SOL	17	15.480	40.170	49.910	1.00	0.00
ATOM	1175	HW2	SOL	17	15.980	40.110	51.330	1.00	0.00
ATOM	1176	OW	SOL	18	51.090	46.460	2.140	1.00	0.00
ATOM	1177	HW1	SOL	18	50.650	47.310	2.140	1.00	0.00
ATOM	1178	HW2	SOL	18	51.500	46.410	3.000	1.00	0.00
ATOM	1179	OW	SOL	19	10.050	45.160	33.060	1.00	0.00
ATOM	1180	HW1	SOL	19	9.680	46.000	33.360	1.00	0.00
ATOM	1181	HW2	SOL	19	10.620	44.890	33.780	1.00	0.00
ATOM	1182	OW	SOL	20	6.490	36.010	26.170	1.00	0.00
ATOM	1183	HW1	SOL	20	5.730	35.770	25.640	1.00	0.00
ATOM	1184	HW2	SOL	20	6.120	36.510	26.900	1.00	0.00
ATOM	1185	OW	SOL	21	31.200	6.990	43.860	1.00	0.00
ATOM	1186	HW1	SOL	21	31.570	7.620	44.480	1.00	0.00
ATOM	1187	HW2	SOL	21	31.010	7.510	43.080	1.00	0.00

ATOM	1188	OW	SOL	22	21.470	38.920	17.390	1.00	0.00
ATOM	1189	HW1	SOL	22	22.170	39.080	16.750	1.00	0.00
ATOM	1190	HW2	SOL	22	21.780	39.350	18.180	1.00	0.00
ATOM	1191	OW	SOL	23	30.290	29.880	0.900	1.00	0.00
ATOM	1192	HW1	SOL	23	29.920	30.550	0.320	1.00	0.00
ATOM	1193	HW2	SOL	23	31.220	29.840	0.670	1.00	0.00
ATOM	1194	OW	SOL	24	18.480	20.370	15.050	1.00	0.00
ATOM	1195	HW1	SOL	24	18.220	20.430	14.130	1.00	0.00
ATOM	1196	HW2	SOL	24	18.600	21.280	15.330	1.00	0.00
ATOM	1197	OW	SOL	25	17.770	13.670	15.880	1.00	0.00
ATOM	1198	HW1	SOL	25	18.370	14.380	15.650	1.00	0.00
ATOM	1199	HW2	SOL	25	17.080	14.100	16.390	1.00	0.00
ATOM	1200	OW	SOL	26	8.560	25.990	13.970	1.00	0.00
ATOM	1201	HW1	SOL	26	8.570	26.520	13.170	1.00	0.00
ATOM	1202	HW2	SOL	26	7.650	26.030	14.260	1.00	0.00
ATOM	1203	OW	SOL	27	32.640	35.110	23.980	1.00	0.00
ATOM	1204	HW1	SOL	27	32.270	35.250	23.110	1.00	0.00
ATOM	1205	HW2	SOL	27	33.590	35.220	23.860	1.00	0.00
ATOM	1206	OW	SOL	28	44.110	26.840	52.700	1.00	0.00
ATOM	1207	HW1	SOL	28	44.860	26.990	53.280	1.00	0.00
ATOM	1208	HW2	SOL	28	43.530	27.590	52.860	1.00	0.00
ATOM	1209	OW	SOL	29	34.300	9.110	30.220	1.00	0.00
ATOM	1210	HW1	SOL	29	35.260	9.070	30.280	1.00	0.00
ATOM	1211	HW2	SOL	29	34.090	8.590	29.450	1.00	0.00
ATOM	1212	OW	SOL	30	2.290	32.070	22.050	1.00	0.00
ATOM	1213	HW1	SOL	30	2.500	31.750	21.170	1.00	0.00
ATOM	1214	HW2	SOL	30	2.100	31.270	22.550	1.00	0.00
ATOM	1215	OW	SOL	31	23.790	37.330	47.620	1.00	0.00
ATOM	1216	HW1	SOL	31	23.170	38.050	47.460	1.00	0.00
ATOM	1217	HW2	SOL	31	24.430	37.410	46.910	1.00	0.00
ATOM	1218	OW	SOL	32	10.010	7.060	32.890	1.00	0.00
ATOM	1219	HW1	SOL	32	10.790	6.520	33.030	1.00	0.00
ATOM	1220	HW2	SOL	32	10.330	7.830	32.420	1.00	0.00
ATOM	1221	OW	SOL	33	21.260	47.950	16.030	1.00	0.00
ATOM	1222	HW1	SOL	33	20.540	47.840	15.410	1.00	0.00
ATOM	1223	HW2	SOL	33	20.860	47.830	16.890	1.00	0.00
ATOM	1224	OW	SOL	34	44.440	55.780	39.560	1.00	0.00
ATOM	1225	HW1	SOL	34	44.730	55.830	38.650	1.00	0.00
ATOM	1226	HW2	SOL	34	45.260	55.800	40.070	1.00	0.00
ATOM	1227	OW	SOL	35	6.990	40.930	52.950	1.00	0.00
ATOM	1228	HW1	SOL	35	7.480	41.610	52.490	1.00	0.00
ATOM	1229	HW2	SOL	35	7.410	40.870	53.810	1.00	0.00
ATOM	1230	OW	SOL	36	55.250	42.800	9.800	1.00	0.00
ATOM	1231	HW1	SOL	36	56.070	43.230	10.040	1.00	0.00

ATOM	1232	HW2	SOL	36	55.120	43.030	8.880	1.00	0.00
ATOM	1233	OW	SOL	37	31.020	7.160	40.860	1.00	0.00
ATOM	1234	HW1	SOL	37	30.370	6.470	40.780	1.00	0.00
ATOM	1235	HW2	SOL	37	30.840	7.750	40.130	1.00	0.00
ATOM	1236	OW	SOL	38	44.060	50.300	30.770	1.00	0.00
ATOM	1237	HW1	SOL	38	44.420	51.120	31.090	1.00	0.00
ATOM	1238	HW2	SOL	38	44.370	50.240	29.860	1.00	0.00
ATOM	1239	OW	SOL	39	26.650	43.010	44.350	1.00	0.00
ATOM	1240	HW1	SOL	39	27.580	43.240	44.420	1.00	0.00
ATOM	1241	HW2	SOL	39	26.290	43.210	45.210	1.00	0.00
ATOM	1242	OW	SOL	40	55.650	41.500	22.090	1.00	0.00
ATOM	1243	HW1	SOL	40	55.540	40.580	22.330	1.00	0.00
ATOM	1244	HW2	SOL	40	55.210	41.580	21.240	1.00	0.00
ATOM	1245	OW	SOL	41	36.310	49.510	46.580	1.00	0.00
ATOM	1246	HW1	SOL	41	36.180	50.330	47.050	1.00	0.00
ATOM	1247	HW2	SOL	41	37.030	49.080	47.040	1.00	0.00
ATOM	1248	OW	SOL	42	25.300	0.430	40.110	1.00	0.00
ATOM	1249	HW1	SOL	42	25.270	1.020	40.870	1.00	0.00
ATOM	1250	HW2	SOL	42	26.200	0.510	39.790	1.00	0.00
ATOM	1251	OW	SOL	43	46.690	4.030	6.150	1.00	0.00
ATOM	1252	HW1	SOL	43	47.160	4.780	5.760	1.00	0.00
ATOM	1253	HW2	SOL	43	45.790	4.140	5.840	1.00	0.00
ATOM	1254	OW	SOL	44	48.830	13.630	20.170	1.00	0.00
ATOM	1255	HW1	SOL	44	49.700	13.950	20.420	1.00	0.00
ATOM	1256	HW2	SOL	44	48.840	13.640	19.210	1.00	0.00
ATOM	1257	OW	SOL	45	43.030	6.390	9.550	1.00	0.00
ATOM	1258	HW1	SOL	45	42.850	5.450	9.640	1.00	0.00
ATOM	1259	HW2	SOL	45	43.920	6.430	9.210	1.00	0.00
ATOM	1260	OW	SOL	46	8.150	55.600	54.360	1.00	0.00
ATOM	1261	HW1	SOL	46	7.730	56.410	54.630	1.00	0.00
ATOM	1262	HW2	SOL	46	8.840	55.450	55.010	1.00	0.00
ATOM	1263	OW	SOL	47	51.090	7.950	22.480	1.00	0.00
ATOM	1264	HW1	SOL	47	50.460	7.830	23.190	1.00	0.00
ATOM	1265	HW2	SOL	47	51.290	8.890	22.490	1.00	0.00
ATOM	1266	OW	SOL	48	33.810	40.460	54.750	1.00	0.00
ATOM	1267	HW1	SOL	48	34.080	41.000	55.490	1.00	0.00
ATOM	1268	HW2	SOL	48	33.340	39.730	55.150	1.00	0.00
ATOM	1269	OW	SOL	49	5.120	27.480	17.250	1.00	0.00
ATOM	1270	HW1	SOL	49	4.990	27.170	18.150	1.00	0.00
ATOM	1271	HW2	SOL	49	4.240	27.650	16.920	1.00	0.00
ATOM	1272	OW	SOL	50	36.560	31.220	55.540	1.00	0.00
ATOM	1273	HW1	SOL	50	37.320	30.660	55.740	1.00	0.00
ATOM	1274	HW2	SOL	50	36.750	31.580	54.680	1.00	0.00
ATOM	1275	OW	SOL	51	12.130	51.140	1.840	1.00	0.00

ATOM	1276	HW1	SOL	51	12.270	50.690	1.000	1.00	0.00
ATOM	1277	HW2	SOL	51	12.990	51.510	2.050	1.00	0.00
ATOM	1278	OW	SOL	52	5.060	8.020	50.440	1.00	0.00
ATOM	1279	HW1	SOL	52	5.980	7.840	50.650	1.00	0.00
ATOM	1280	HW2	SOL	52	4.690	7.160	50.220	1.00	0.00
ATOM	1281	OW	SOL	53	14.130	20.770	34.320	1.00	0.00
ATOM	1282	HW1	SOL	53	14.930	21.240	34.550	1.00	0.00
ATOM	1283	HW2	SOL	53	14.320	19.850	34.500	1.00	0.00
ATOM	1284	OW	SOL	54	51.630	13.910	21.130	1.00	0.00
ATOM	1285	HW1	SOL	54	51.170	14.090	21.950	1.00	0.00
ATOM	1286	HW2	SOL	54	51.910	13.000	21.210	1.00	0.00
ATOM	1287	OW	SOL	55	3.640	7.400	28.370	1.00	0.00
ATOM	1288	HW1	SOL	55	3.200	8.210	28.620	1.00	0.00
ATOM	1289	HW2	SOL	55	4.570	7.580	28.500	1.00	0.00
ATOM	1290	OW	SOL	56	19.610	40.490	25.350	1.00	0.00
ATOM	1291	HW1	SOL	56	20.480	40.740	25.020	1.00	0.00
ATOM	1292	HW2	SOL	56	19.010	40.810	24.680	1.00	0.00
ATOM	1293	OW	SOL	57	5.490	4.760	21.300	1.00	0.00
ATOM	1294	HW1	SOL	57	5.680	4.530	22.210	1.00	0.00
ATOM	1295	HW2	SOL	57	6.170	5.390	21.060	1.00	0.00
ATOM	1296	OW	SOL	58	6.280	30.070	34.630	1.00	0.00
ATOM	1297	HW1	SOL	58	7.180	30.250	34.370	1.00	0.00
ATOM	1298	HW2	SOL	58	5.750	30.700	34.130	1.00	0.00
ATOM	1299	OW	SOL	59	30.800	30.190	5.550	1.00	0.00
ATOM	1300	HW1	SOL	59	30.660	29.710	4.740	1.00	0.00
ATOM	1301	HW2	SOL	59	30.870	29.520	6.220	1.00	0.00
ATOM	1302	OW	SOL	60	24.030	0.390	47.960	1.00	0.00
ATOM	1303	HW1	SOL	60	23.110	0.590	48.090	1.00	0.00
ATOM	1304	HW2	SOL	60	24.100	-0.550	48.130	1.00	0.00
ATOM	1305	OW	SOL	61	25.260	44.710	17.900	1.00	0.00
ATOM	1306	HW1	SOL	61	25.300	43.990	17.270	1.00	0.00
ATOM	1307	HW2	SOL	61	25.000	45.470	17.380	1.00	0.00
ATOM	1308	OW	SOL	62	8.390	13.250	32.200	1.00	0.00
ATOM	1309	HW1	SOL	62	7.440	13.130	32.180	1.00	0.00
ATOM	1310	HW2	SOL	62	8.720	12.480	32.660	1.00	0.00
ATOM	1311	OW	SOL	63	4.130	34.990	11.410	1.00	0.00
ATOM	1312	HW1	SOL	63	4.420	35.660	12.030	1.00	0.00
ATOM	1313	HW2	SOL	63	4.730	35.080	10.670	1.00	0.00
ATOM	1314	OW	SOL	64	17.510	39.700	0.910	1.00	0.00
ATOM	1315	HW1	SOL	64	17.910	40.570	0.870	1.00	0.00
ATOM	1316	HW2	SOL	64	17.230	39.520	0.010	1.00	0.00
ATOM	1317	OW	SOL	65	49.370	52.360	31.960	1.00	0.00
ATOM	1318	HW1	SOL	65	49.770	52.040	31.150	1.00	0.00
ATOM	1319	HW2	SOL	65	49.440	53.310	31.900	1.00	0.00

ATOM	1320	OW	SOL	66	14.430	49.630	20.990	1.00	0.00
ATOM	1321	HW1	SOL	66	14.230	48.700	21.150	1.00	0.00
ATOM	1322	HW2	SOL	66	13.910	49.850	20.220	1.00	0.00
ATOM	1323	OW	SOL	67	51.440	7.850	54.730	1.00	0.00
ATOM	1324	HW1	SOL	67	51.450	7.610	55.650	1.00	0.00
ATOM	1325	HW2	SOL	67	50.760	8.520	54.670	1.00	0.00
ATOM	1326	OW	SOL	68	5.960	54.450	49.290	1.00	0.00
ATOM	1327	HW1	SOL	68	6.400	54.370	50.130	1.00	0.00
ATOM	1328	HW2	SOL	68	5.510	55.300	49.330	1.00	0.00
ATOM	1329	OW	SOL	69	48.570	2.570	54.320	1.00	0.00
ATOM	1330	HW1	SOL	69	49.040	2.630	53.500	1.00	0.00
ATOM	1331	HW2	SOL	69	48.150	3.430	54.420	1.00	0.00
ATOM	1332	OW	SOL	70	19.470	22.050	50.290	1.00	0.00
ATOM	1333	HW1	SOL	70	19.800	21.250	50.710	1.00	0.00
ATOM	1334	HW2	SOL	70	18.840	22.410	50.910	1.00	0.00
ATOM	1335	OW	SOL	71	0.280	40.400	39.000	1.00	0.00
ATOM	1336	HW1	SOL	71	0.620	39.510	39.150	1.00	0.00
ATOM	1337	HW2	SOL	71	0.940	40.970	39.410	1.00	0.00
ATOM	1338	OW	SOL	72	20.030	9.010	30.240	1.00	0.00
ATOM	1339	HW1	SOL	72	20.880	9.420	30.380	1.00	0.00
ATOM	1340	HW2	SOL	72	19.430	9.490	30.800	1.00	0.00
ATOM	1341	OW	SOL	73	19.760	5.470	50.710	1.00	0.00
ATOM	1342	HW1	SOL	73	20.140	4.600	50.610	1.00	0.00
ATOM	1343	HW2	SOL	73	18.860	5.310	50.990	1.00	0.00
ATOM	1344	OW	SOL	74	36.610	16.690	36.140	1.00	0.00
ATOM	1345	HW1	SOL	74	36.770	16.500	35.210	1.00	0.00
ATOM	1346	HW2	SOL	74	35.790	17.190	36.150	1.00	0.00
ATOM	1347	OW	SOL	75	36.950	42.180	55.470	1.00	0.00
ATOM	1348	HW1	SOL	75	37.870	41.940	55.340	1.00	0.00
ATOM	1349	HW2	SOL	75	36.510	41.930	54.660	1.00	0.00
ATOM	1350	OW	SOL	76	28.140	5.450	13.770	1.00	0.00
ATOM	1351	HW1	SOL	76	29.070	5.660	13.910	1.00	0.00
ATOM	1352	HW2	SOL	76	28.150	4.800	13.070	1.00	0.00
ATOM	1353	OW	SOL	77	7.940	52.560	30.910	1.00	0.00
ATOM	1354	HW1	SOL	77	8.460	52.300	31.670	1.00	0.00
ATOM	1355	HW2	SOL	77	7.860	51.760	30.390	1.00	0.00
ATOM	1356	OW	SOL	78	47.800	45.990	49.950	1.00	0.00
ATOM	1357	HW1	SOL	78	47.300	45.720	49.180	1.00	0.00
ATOM	1358	HW2	SOL	78	47.550	45.370	50.630	1.00	0.00
ATOM	1359	OW	SOL	79	40.620	55.460	38.630	1.00	0.00
ATOM	1360	HW1	SOL	79	41.340	55.130	38.100	1.00	0.00
ATOM	1361	HW2	SOL	79	39.840	55.100	38.230	1.00	0.00
ATOM	1362	OW	SOL	80	47.100	54.200	28.700	1.00	0.00
ATOM	1363	HW1	SOL	80	46.380	54.460	29.270	1.00	0.00

ATOM	1364	HW2	SOL	80	47.480	55.030	28.410	1.00	0.00
ATOM	1365	OW	SOL	81	26.480	27.540	54.670	1.00	0.00
ATOM	1366	HW1	SOL	81	26.480	26.950	53.920	1.00	0.00
ATOM	1367	HW2	SOL	81	27.170	27.210	55.240	1.00	0.00
ATOM	1368	OW	SOL	82	30.610	42.350	49.850	1.00	0.00
ATOM	1369	HW1	SOL	82	29.780	42.640	49.480	1.00	0.00
ATOM	1370	HW2	SOL	82	30.450	42.320	50.790	1.00	0.00
ATOM	1371	OW	SOL	83	53.980	26.460	25.740	1.00	0.00
ATOM	1372	HW1	SOL	83	53.080	26.290	25.450	1.00	0.00
ATOM	1373	HW2	SOL	83	53.920	27.270	26.240	1.00	0.00
ATOM	1374	OW	SOL	84	9.180	36.670	51.310	1.00	0.00
ATOM	1375	HW1	SOL	84	8.330	36.950	50.990	1.00	0.00
ATOM	1376	HW2	SOL	84	9.810	37.000	50.660	1.00	0.00
ATOM	1377	OW	SOL	85	17.910	38.150	49.950	1.00	0.00
ATOM	1378	HW1	SOL	85	17.500	38.970	50.240	1.00	0.00
ATOM	1379	HW2	SOL	85	17.640	37.500	50.600	1.00	0.00
ATOM	1380	OW	SOL	86	4.740	27.060	19.980	1.00	0.00
ATOM	1381	HW1	SOL	86	5.320	26.300	19.980	1.00	0.00
ATOM	1382	HW2	SOL	86	4.050	26.830	20.610	1.00	0.00
ATOM	1383	OW	SOL	87	33.590	18.500	51.820	1.00	0.00
ATOM	1384	HW1	SOL	87	32.700	18.160	51.720	1.00	0.00
ATOM	1385	HW2	SOL	87	33.470	19.450	51.930	1.00	0.00
ATOM	1386	OW	SOL	88	14.830	20.770	18.000	1.00	0.00
ATOM	1387	HW1	SOL	88	13.970	20.410	17.780	1.00	0.00
ATOM	1388	HW2	SOL	88	15.360	20.010	18.240	1.00	0.00
ATOM	1389	OW	SOL	89	1.600	24.230	30.760	1.00	0.00
ATOM	1390	HW1	SOL	89	1.390	24.650	31.600	1.00	0.00
ATOM	1391	HW2	SOL	89	0.780	23.840	30.470	1.00	0.00
ATOM	1392	OW	SOL	90	52.070	17.770	25.390	1.00	0.00
ATOM	1393	HW1	SOL	90	51.430	18.480	25.410	1.00	0.00
ATOM	1394	HW2	SOL	90	52.750	18.040	26.000	1.00	0.00
ATOM	1395	OW	SOL	91	32.050	43.010	2.690	1.00	0.00
ATOM	1396	HW1	SOL	91	32.540	43.510	2.040	1.00	0.00
ATOM	1397	HW2	SOL	91	32.720	42.500	3.150	1.00	0.00
ATOM	1398	OW	SOL	92	11.630	45.400	42.070	1.00	0.00
ATOM	1399	HW1	SOL	92	11.390	45.960	42.810	1.00	0.00
ATOM	1400	HW2	SOL	92	12.570	45.540	41.970	1.00	0.00
ATOM	1401	OW	SOL	93	41.850	42.620	23.530	1.00	0.00
ATOM	1402	HW1	SOL	93	41.680	42.230	24.390	1.00	0.00
ATOM	1403	HW2	SOL	93	42.600	42.140	23.190	1.00	0.00
ATOM	1404	OW	SOL	94	33.920	7.110	6.560	1.00	0.00
ATOM	1405	HW1	SOL	94	33.140	6.930	7.090	1.00	0.00
ATOM	1406	HW2	SOL	94	34.510	7.580	7.160	1.00	0.00
ATOM	1407	OW	SOL	95	28.190	27.200	49.350	1.00	0.00

ATOM	1408	HW1	SOL	95	28.750	26.890	48.630	1.00	0.00
ATOM	1409	HW2	SOL	95	28.590	28.020	49.630	1.00	0.00
ATOM	1410	OW	SOL	96	7.350	26.330	38.000	1.00	0.00
ATOM	1411	HW1	SOL	96	8.180	26.350	37.530	1.00	0.00
ATOM	1412	HW2	SOL	96	6.790	26.970	37.550	1.00	0.00
ATOM	1413	OW	SOL	97	10.390	55.150	0.050	1.00	0.00
ATOM	1414	HW1	SOL	97	10.760	55.780	0.670	1.00	0.00
ATOM	1415	HW2	SOL	97	10.900	54.350	0.190	1.00	0.00
ATOM	1416	OW	SOL	98	6.100	26.250	14.830	1.00	0.00
ATOM	1417	HW1	SOL	98	5.330	26.150	14.260	1.00	0.00
ATOM	1418	HW2	SOL	98	5.750	26.590	15.650	1.00	0.00
ATOM	1419	OW	SOL	99	42.330	34.280	39.170	1.00	0.00
ATOM	1420	HW1	SOL	99	42.490	34.700	38.330	1.00	0.00
ATOM	1421	HW2	SOL	99	42.890	33.510	39.160	1.00	0.00
ATOM	1422	OW	SOL	100	32.530	15.210	30.790	1.00	0.00
ATOM	1423	HW1	SOL	100	33.400	15.510	30.530	1.00	0.00
ATOM	1424	HW2	SOL	100	32.420	15.510	31.690	1.00	0.00
ATOM	1425	OW	SOL	101	32.710	5.340	38.180	1.00	0.00
ATOM	1426	HW1	SOL	101	33.130	6.170	37.940	1.00	0.00
ATOM	1427	HW2	SOL	101	33.010	4.720	37.520	1.00	0.00
ATOM	1428	OW	SOL	102	39.690	40.890	42.780	1.00	0.00
ATOM	1429	HW1	SOL	102	40.570	40.960	43.140	1.00	0.00
ATOM	1430	HW2	SOL	102	39.780	40.320	42.010	1.00	0.00
ATOM	1431	OW	SOL	103	34.610	45.040	21.560	1.00	0.00
ATOM	1432	HW1	SOL	103	35.480	45.420	21.670	1.00	0.00
ATOM	1433	HW2	SOL	103	34.650	44.580	20.720	1.00	0.00
ATOM	1434	OW	SOL	104	39.610	17.480	12.780	1.00	0.00
ATOM	1435	HW1	SOL	104	40.450	17.080	12.530	1.00	0.00
ATOM	1436	HW2	SOL	104	39.170	17.660	11.950	1.00	0.00
ATOM	1437	OW	SOL	105	25.840	46.070	53.000	1.00	0.00
ATOM	1438	HW1	SOL	105	25.740	45.730	53.890	1.00	0.00
ATOM	1439	HW2	SOL	105	25.170	45.610	52.490	1.00	0.00
ATOM	1440	OW	SOL	106	48.190	44.120	39.090	1.00	0.00
ATOM	1441	HW1	SOL	106	48.820	44.060	38.380	1.00	0.00
ATOM	1442	HW2	SOL	106	48.720	44.110	39.890	1.00	0.00
ATOM	1443	OW	SOL	107	1.510	36.970	12.340	1.00	0.00
ATOM	1444	HW1	SOL	107	1.230	36.100	12.600	1.00	0.00
ATOM	1445	HW2	SOL	107	2.340	37.100	12.800	1.00	0.00
ATOM	1446	OW	SOL	108	28.930	33.910	24.980	1.00	0.00
ATOM	1447	HW1	SOL	108	27.990	33.920	24.830	1.00	0.00
ATOM	1448	HW2	SOL	108	29.300	34.410	24.260	1.00	0.00
ATOM	1449	OW	SOL	109	23.240	22.010	7.150	1.00	0.00
ATOM	1450	HW1	SOL	109	23.040	22.940	7.050	1.00	0.00
ATOM	1451	HW2	SOL	109	23.870	21.830	6.450	1.00	0.00

ATOM	1452	OW	SOL	110	46.100	39.310	51.090	1.00	0.00
ATOM	1453	HW1	SOL	110	46.820	39.680	50.580	1.00	0.00
ATOM	1454	HW2	SOL	110	46.200	39.690	51.960	1.00	0.00
ATOM	1455	OW	SOL	111	17.270	6.590	49.370	1.00	0.00
ATOM	1456	HW1	SOL	111	18.200	6.500	49.170	1.00	0.00
ATOM	1457	HW2	SOL	111	17.180	6.210	50.250	1.00	0.00
ATOM	1458	OW	SOL	112	5.350	10.730	42.180	1.00	0.00
ATOM	1459	HW1	SOL	112	4.910	10.110	42.760	1.00	0.00
ATOM	1460	HW2	SOL	112	5.830	10.180	41.560	1.00	0.00
ATOM	1461	OW	SOL	113	14.660	55.030	7.830	1.00	0.00
ATOM	1462	HW1	SOL	113	15.580	54.790	7.890	1.00	0.00
ATOM	1463	HW2	SOL	113	14.660	55.930	7.510	1.00	0.00
ATOM	1464	OW	SOL	114	20.430	46.960	18.370	1.00	0.00
ATOM	1465	HW1	SOL	114	19.810	46.240	18.240	1.00	0.00
ATOM	1466	HW2	SOL	114	20.600	46.960	19.310	1.00	0.00
ATOM	1467	OW	SOL	115	52.540	46.930	19.910	1.00	0.00
ATOM	1468	HW1	SOL	115	52.610	47.880	19.860	1.00	0.00
ATOM	1469	HW2	SOL	115	51.770	46.710	19.380	1.00	0.00
ATOM	1470	OW	SOL	116	34.470	15.940	47.910	1.00	0.00
ATOM	1471	HW1	SOL	116	34.220	16.740	47.460	1.00	0.00
ATOM	1472	HW2	SOL	116	33.950	15.260	47.480	1.00	0.00
ATOM	1473	OW	SOL	117	39.840	29.560	19.080	1.00	0.00
ATOM	1474	HW1	SOL	117	40.370	28.920	19.570	1.00	0.00
ATOM	1475	HW2	SOL	117	40.010	30.390	19.520	1.00	0.00
ATOM	1476	OW	SOL	118	23.770	25.410	54.790	1.00	0.00
ATOM	1477	HW1	SOL	118	23.390	25.640	55.640	1.00	0.00
ATOM	1478	HW2	SOL	118	23.110	24.860	54.370	1.00	0.00
ATOM	1479	OW	SOL	119	5.780	39.720	0.090	1.00	0.00
ATOM	1480	HW1	SOL	119	5.480	40.630	0.020	1.00	0.00
ATOM	1481	HW2	SOL	119	6.710	39.760	-0.140	1.00	0.00
ATOM	1482	OW	SOL	120	32.890	10.050	49.910	1.00	0.00
ATOM	1483	HW1	SOL	120	33.090	10.590	50.670	1.00	0.00
ATOM	1484	HW2	SOL	120	33.540	9.350	49.940	1.00	0.00
ATOM	1485	OW	SOL	121	49.940	38.890	26.600	1.00	0.00
ATOM	1486	HW1	SOL	121	49.090	39.140	26.230	1.00	0.00
ATOM	1487	HW2	SOL	121	50.010	37.950	26.420	1.00	0.00
ATOM	1488	OW	SOL	122	9.720	10.500	17.730	1.00	0.00
ATOM	1489	HW1	SOL	122	9.710	9.900	16.980	1.00	0.00
ATOM	1490	HW2	SOL	122	10.560	10.320	18.170	1.00	0.00
ATOM	1491	OW	SOL	123	35.720	50.830	3.960	1.00	0.00
ATOM	1492	HW1	SOL	123	35.420	51.570	4.480	1.00	0.00
ATOM	1493	HW2	SOL	123	36.670	50.830	4.060	1.00	0.00
ATOM	1494	OW	SOL	124	27.880	21.020	46.380	1.00	0.00
ATOM	1495	HW1	SOL	124	28.430	20.500	45.790	1.00	0.00

ATOM	1496	HW2	SOL	124	28.230	21.910	46.310	1.00	0.00
ATOM	1497	OW	SOL	125	0.860	45.000	39.470	1.00	0.00
ATOM	1498	HW1	SOL	125	0.850	44.780	38.540	1.00	0.00
ATOM	1499	HW2	SOL	125	0.940	44.150	39.910	1.00	0.00
ATOM	1500	OW	SOL	126	42.920	0.410	14.250	1.00	0.00
ATOM	1501	HW1	SOL	126	42.220	0.830	13.760	1.00	0.00
ATOM	1502	HW2	SOL	126	43.490	0.030	13.580	1.00	0.00
ATOM	1503	OW	SOL	127	53.610	6.040	41.890	1.00	0.00
ATOM	1504	HW1	SOL	127	54.140	6.710	41.440	1.00	0.00
ATOM	1505	HW2	SOL	127	54.000	5.210	41.620	1.00	0.00
ATOM	1506	OW	SOL	128	46.880	16.990	19.030	1.00	0.00
ATOM	1507	HW1	SOL	128	47.590	17.600	19.230	1.00	0.00
ATOM	1508	HW2	SOL	128	46.800	16.450	19.820	1.00	0.00
ATOM	1509	OW	SOL	129	14.410	35.770	20.280	1.00	0.00
ATOM	1510	HW1	SOL	129	14.150	36.470	20.880	1.00	0.00
ATOM	1511	HW2	SOL	129	15.340	35.920	20.120	1.00	0.00
ATOM	1512	OW	SOL	130	46.480	37.540	7.660	1.00	0.00
ATOM	1513	HW1	SOL	130	45.820	37.690	6.980	1.00	0.00
ATOM	1514	HW2	SOL	130	46.470	36.600	7.800	1.00	0.00
ATOM	1515	OW	SOL	131	32.850	54.340	34.530	1.00	0.00
ATOM	1516	HW1	SOL	131	33.010	53.490	34.960	1.00	0.00
ATOM	1517	HW2	SOL	131	33.080	54.990	35.200	1.00	0.00
ATOM	1518	OW	SOL	132	7.120	25.010	53.940	1.00	0.00
ATOM	1519	HW1	SOL	132	7.900	25.350	53.490	1.00	0.00
ATOM	1520	HW2	SOL	132	6.790	24.330	53.360	1.00	0.00
ATOM	1521	OW	SOL	133	36.130	38.750	14.430	1.00	0.00
ATOM	1522	HW1	SOL	133	36.330	39.640	14.160	1.00	0.00
ATOM	1523	HW2	SOL	133	35.490	38.440	13.790	1.00	0.00
ATOM	1524	OW	SOL	134	55.130	52.150	37.510	1.00	0.00
ATOM	1525	HW1	SOL	134	54.390	52.190	36.910	1.00	0.00
ATOM	1526	HW2	SOL	134	54.790	51.700	38.290	1.00	0.00
ATOM	1527	OW	SOL	135	52.660	19.540	34.310	1.00	0.00
ATOM	1528	HW1	SOL	135	53.380	19.060	34.730	1.00	0.00
ATOM	1529	HW2	SOL	135	51.870	19.230	34.740	1.00	0.00
ATOM	1530	OW	SOL	136	6.810	13.110	1.830	1.00	0.00
ATOM	1531	HW1	SOL	136	7.170	13.780	2.410	1.00	0.00
ATOM	1532	HW2	SOL	136	6.080	12.740	2.320	1.00	0.00
ATOM	1533	OW	SOL	137	42.040	13.980	48.500	1.00	0.00
ATOM	1534	HW1	SOL	137	42.330	14.590	47.810	1.00	0.00
ATOM	1535	HW2	SOL	137	42.180	13.110	48.130	1.00	0.00
ATOM	1536	OW	SOL	138	50.910	1.010	45.480	1.00	0.00
ATOM	1537	HW1	SOL	138	51.300	0.140	45.560	1.00	0.00
ATOM	1538	HW2	SOL	138	51.600	1.610	45.740	1.00	0.00
ATOM	1539	OW	SOL	139	28.900	27.630	23.000	1.00	0.00

ATOM	1540	HW1	SOL	139	28.650	27.470	22.090	1.00	0.00
ATOM	1541	HW2	SOL	139	28.980	28.580	23.070	1.00	0.00
ATOM	1542	OW	SOL	140	39.760	41.780	16.980	1.00	0.00
ATOM	1543	HW1	SOL	140	39.420	41.010	17.440	1.00	0.00
ATOM	1544	HW2	SOL	140	40.060	42.360	17.680	1.00	0.00
ATOM	1545	OW	SOL	141	52.710	19.700	10.780	1.00	0.00
ATOM	1546	HW1	SOL	141	52.550	20.640	10.890	1.00	0.00
ATOM	1547	HW2	SOL	141	52.890	19.590	9.850	1.00	0.00
ATOM	1548	OW	SOL	142	3.440	47.530	17.190	1.00	0.00
ATOM	1549	HW1	SOL	142	3.940	47.530	18.010	1.00	0.00
ATOM	1550	HW2	SOL	142	2.570	47.210	17.450	1.00	0.00
ATOM	1551	OW	SOL	143	52.320	12.010	47.650	1.00	0.00
ATOM	1552	HW1	SOL	143	52.280	11.290	47.020	1.00	0.00
ATOM	1553	HW2	SOL	143	51.830	12.720	47.240	1.00	0.00
ATOM	1554	OW	SOL	144	2.230	10.460	37.550	1.00	0.00
ATOM	1555	HW1	SOL	144	2.130	11.390	37.740	1.00	0.00
ATOM	1556	HW2	SOL	144	2.700	10.430	36.710	1.00	0.00
ATOM	1557	OW	SOL	145	48.220	37.330	53.980	1.00	0.00
ATOM	1558	HW1	SOL	145	48.140	36.510	54.470	1.00	0.00
ATOM	1559	HW2	SOL	145	48.430	37.060	53.090	1.00	0.00
ATOM	1560	OW	SOL	146	14.450	42.680	5.730	1.00	0.00
ATOM	1561	HW1	SOL	146	14.340	41.750	5.900	1.00	0.00
ATOM	1562	HW2	SOL	146	15.380	42.780	5.530	1.00	0.00
ATOM	1563	OW	SOL	147	10.210	53.200	20.210	1.00	0.00
ATOM	1564	HW1	SOL	147	10.750	53.790	20.750	1.00	0.00
ATOM	1565	HW2	SOL	147	10.140	53.660	19.370	1.00	0.00
ATOM	1566	OW	SOL	148	34.220	26.240	55.150	1.00	0.00
ATOM	1567	HW1	SOL	148	33.670	26.660	55.810	1.00	0.00
ATOM	1568	HW2	SOL	148	35.110	26.540	55.360	1.00	0.00
ATOM	1569	OW	SOL	149	9.940	7.270	23.500	1.00	0.00
ATOM	1570	HW1	SOL	149	10.450	7.220	22.690	1.00	0.00
ATOM	1571	HW2	SOL	149	9.680	8.180	23.560	1.00	0.00
ATOM	1572	OW	SOL	150	31.900	6.860	21.790	1.00	0.00
ATOM	1573	HW1	SOL	150	30.960	7.040	21.800	1.00	0.00
ATOM	1574	HW2	SOL	150	31.980	6.060	21.270	1.00	0.00
ATOM	1575	OW	SOL	151	50.010	45.780	32.670	1.00	0.00
ATOM	1576	HW1	SOL	151	50.580	46.530	32.490	1.00	0.00
ATOM	1577	HW2	SOL	151	49.180	46.000	32.250	1.00	0.00
ATOM	1578	OW	SOL	152	3.440	6.350	35.430	1.00	0.00
ATOM	1579	HW1	SOL	152	2.640	6.840	35.240	1.00	0.00
ATOM	1580	HW2	SOL	152	3.530	5.740	34.690	1.00	0.00
ATOM	1581	OW	SOL	153	13.090	28.720	2.430	1.00	0.00
ATOM	1582	HW1	SOL	153	12.380	28.380	2.980	1.00	0.00
ATOM	1583	HW2	SOL	153	13.570	27.940	2.150	1.00	0.00

ATOM	1584	OW	SOL	154	39.530	43.190	1.450	1.00	0.00
ATOM	1585	HW1	SOL	154	39.430	42.240	1.380	1.00	0.00
ATOM	1586	HW2	SOL	154	38.670	43.500	1.730	1.00	0.00
ATOM	1587	OW	SOL	155	2.120	7.520	7.270	1.00	0.00
ATOM	1588	HW1	SOL	155	1.180	7.690	7.180	1.00	0.00
ATOM	1589	HW2	SOL	155	2.180	6.960	8.050	1.00	0.00
ATOM	1590	OW	SOL	156	31.480	33.760	14.220	1.00	0.00
ATOM	1591	HW1	SOL	156	30.750	33.180	14.000	1.00	0.00
ATOM	1592	HW2	SOL	156	31.180	34.630	13.920	1.00	0.00
ATOM	1593	OW	SOL	157	49.220	44.720	9.300	1.00	0.00
ATOM	1594	HW1	SOL	157	49.760	45.320	8.790	1.00	0.00
ATOM	1595	HW2	SOL	157	49.830	44.340	9.940	1.00	0.00
ATOM	1596	OW	SOL	158	7.270	9.290	23.970	1.00	0.00
ATOM	1597	HW1	SOL	158	8.040	9.770	23.680	1.00	0.00
ATOM	1598	HW2	SOL	158	6.870	8.970	23.160	1.00	0.00
ATOM	1599	OW	SOL	159	36.920	2.990	13.160	1.00	0.00
ATOM	1600	HW1	SOL	159	37.740	3.100	12.670	1.00	0.00
ATOM	1601	HW2	SOL	159	36.720	2.050	13.090	1.00	0.00
ATOM	1602	OW	SOL	160	11.070	7.040	20.520	1.00	0.00
ATOM	1603	HW1	SOL	160	11.790	6.410	20.470	1.00	0.00
ATOM	1604	HW2	SOL	160	10.280	6.510	20.430	1.00	0.00
ATOM	1605	OW	SOL	161	52.020	23.070	41.940	1.00	0.00
ATOM	1606	HW1	SOL	161	51.400	23.040	42.670	1.00	0.00
ATOM	1607	HW2	SOL	161	52.430	23.930	42.010	1.00	0.00
ATOM	1608	OW	SOL	162	20.150	16.040	6.750	1.00	0.00
ATOM	1609	HW1	SOL	162	19.550	15.490	7.250	1.00	0.00
ATOM	1610	HW2	SOL	162	20.580	16.590	7.400	1.00	0.00
ATOM	1611	OW	SOL	163	18.300	48.230	1.350	1.00	0.00
ATOM	1612	HW1	SOL	163	18.930	47.970	2.030	1.00	0.00
ATOM	1613	HW2	SOL	163	18.840	48.400	0.580	1.00	0.00
ATOM	1614	OW	SOL	164	15.380	51.180	30.640	1.00	0.00
ATOM	1615	HW1	SOL	164	16.150	50.630	30.470	1.00	0.00
ATOM	1616	HW2	SOL	164	15.500	51.510	31.520	1.00	0.00
ATOM	1617	OW	SOL	165	16.680	47.750	47.300	1.00	0.00
ATOM	1618	HW1	SOL	165	16.930	48.380	46.620	1.00	0.00
ATOM	1619	HW2	SOL	165	17.480	47.240	47.460	1.00	0.00
ATOM	1620	OW	SOL	166	29.270	5.810	50.510	1.00	0.00
ATOM	1621	HW1	SOL	166	29.080	6.160	49.630	1.00	0.00
ATOM	1622	HW2	SOL	166	28.960	4.910	50.490	1.00	0.00
ATOM	1623	OW	SOL	167	16.590	35.480	48.370	1.00	0.00
ATOM	1624	HW1	SOL	167	16.300	36.120	49.020	1.00	0.00
ATOM	1625	HW2	SOL	167	17.410	35.830	48.030	1.00	0.00
ATOM	1626	OW	SOL	168	14.390	8.790	46.000	1.00	0.00
ATOM	1627	HW1	SOL	168	15.290	9.110	46.050	1.00	0.00

ATOM	1628	HW2	SOL	168	13.860	9.500	46.360	1.00	0.00
ATOM	1629	OW	SOL	169	6.030	54.180	27.970	1.00	0.00
ATOM	1630	HW1	SOL	169	5.540	53.630	28.590	1.00	0.00
ATOM	1631	HW2	SOL	169	5.390	54.810	27.650	1.00	0.00
ATOM	1632	OW	SOL	170	1.740	27.320	9.690	1.00	0.00
ATOM	1633	HW1	SOL	170	2.500	27.860	9.890	1.00	0.00
ATOM	1634	HW2	SOL	170	1.610	27.430	8.750	1.00	0.00
ATOM	1635	OW	SOL	171	23.520	47.370	43.800	1.00	0.00
ATOM	1636	HW1	SOL	171	23.960	48.220	43.910	1.00	0.00
ATOM	1637	HW2	SOL	171	24.190	46.730	44.040	1.00	0.00
ATOM	1638	OW	SOL	172	42.680	51.010	33.800	1.00	0.00
ATOM	1639	HW1	SOL	172	42.310	51.880	33.660	1.00	0.00
ATOM	1640	HW2	SOL	172	42.390	50.510	33.040	1.00	0.00
ATOM	1641	OW	SOL	173	49.590	54.160	42.390	1.00	0.00
ATOM	1642	HW1	SOL	173	49.110	54.080	43.210	1.00	0.00
ATOM	1643	HW2	SOL	173	48.990	53.830	41.720	1.00	0.00
ATOM	1644	OW	SOL	174	41.650	18.600	45.050	1.00	0.00
ATOM	1645	HW1	SOL	174	42.070	19.270	45.590	1.00	0.00
ATOM	1646	HW2	SOL	174	40.890	18.320	45.560	1.00	0.00
ATOM	1647	OW	SOL	175	4.620	28.860	13.020	1.00	0.00
ATOM	1648	HW1	SOL	175	3.670	28.860	12.900	1.00	0.00
ATOM	1649	HW2	SOL	175	4.860	27.930	12.980	1.00	0.00
ATOM	1650	OW	SOL	176	17.220	39.830	31.420	1.00	0.00
ATOM	1651	HW1	SOL	176	17.760	39.070	31.600	1.00	0.00
ATOM	1652	HW2	SOL	176	17.700	40.570	31.780	1.00	0.00
ATOM	1653	OW	SOL	177	17.620	48.360	15.900	1.00	0.00
ATOM	1654	HW1	SOL	177	17.880	47.610	15.370	1.00	0.00
ATOM	1655	HW2	SOL	177	17.950	49.120	15.420	1.00	0.00
ATOM	1656	OW	SOL	178	14.440	26.890	28.970	1.00	0.00
ATOM	1657	HW1	SOL	178	14.750	26.160	29.510	1.00	0.00
ATOM	1658	HW2	SOL	178	14.950	26.810	28.160	1.00	0.00
ATOM	1659	OW	SOL	179	3.920	29.090	30.850	1.00	0.00
ATOM	1660	HW1	SOL	179	3.810	28.950	31.790	1.00	0.00
ATOM	1661	HW2	SOL	179	3.920	30.050	30.750	1.00	0.00
ATOM	1662	OW	SOL	180	4.700	38.480	42.500	1.00	0.00
ATOM	1663	HW1	SOL	180	4.310	39.330	42.660	1.00	0.00
ATOM	1664	HW2	SOL	180	4.380	37.930	43.210	1.00	0.00
ATOM	1665	OW	SOL	181	42.270	9.400	10.310	1.00	0.00
ATOM	1666	HW1	SOL	181	42.030	8.480	10.240	1.00	0.00
ATOM	1667	HW2	SOL	181	43.220	9.400	10.410	1.00	0.00
ATOM	1668	OW	SOL	182	1.860	29.550	35.120	1.00	0.00
ATOM	1669	HW1	SOL	182	2.410	29.220	35.830	1.00	0.00
ATOM	1670	HW2	SOL	182	1.130	29.990	35.570	1.00	0.00
ATOM	1671	OW	SOL	183	35.520	55.470	10.960	1.00	0.00

ATOM	1672	HW1	SOL	183	34.660	55.200	10.640	1.00	0.00
ATOM	1673	HW2	SOL	183	35.400	55.610	11.900	1.00	0.00
ATOM	1674	OW	SOL	184	13.980	15.810	38.920	1.00	0.00
ATOM	1675	HW1	SOL	184	14.390	15.560	39.740	1.00	0.00
ATOM	1676	HW2	SOL	184	14.470	15.330	38.250	1.00	0.00
ATOM	1677	OW	SOL	185	55.870	29.190	7.840	1.00	0.00
ATOM	1678	HW1	SOL	185	56.830	29.250	7.860	1.00	0.00
ATOM	1679	HW2	SOL	185	55.630	29.570	6.990	1.00	0.00
ATOM	1680	OW	SOL	186	14.440	9.070	50.660	1.00	0.00
ATOM	1681	HW1	SOL	186	13.750	9.400	51.230	1.00	0.00
ATOM	1682	HW2	SOL	186	15.010	8.570	51.240	1.00	0.00
ATOM	1683	OW	SOL	187	22.730	2.990	55.020	1.00	0.00
ATOM	1684	HW1	SOL	187	22.780	2.670	55.920	1.00	0.00
ATOM	1685	HW2	SOL	187	21.970	2.530	54.650	1.00	0.00
ATOM	1686	OW	SOL	188	46.180	55.760	17.140	1.00	0.00
ATOM	1687	HW1	SOL	188	46.380	56.180	17.980	1.00	0.00
ATOM	1688	HW2	SOL	188	46.030	56.490	16.540	1.00	0.00
ATOM	1689	OW	SOL	189	15.330	49.890	35.110	1.00	0.00
ATOM	1690	HW1	SOL	189	15.660	50.240	35.940	1.00	0.00
ATOM	1691	HW2	SOL	189	14.580	49.350	35.350	1.00	0.00
ATOM	1692	OW	SOL	190	47.110	47.690	3.600	1.00	0.00
ATOM	1693	HW1	SOL	190	47.010	47.480	2.670	1.00	0.00
ATOM	1694	HW2	SOL	190	46.730	46.940	4.060	1.00	0.00
ATOM	1695	OW	SOL	191	33.470	38.600	22.540	1.00	0.00
ATOM	1696	HW1	SOL	191	33.570	39.540	22.620	1.00	0.00
ATOM	1697	HW2	SOL	191	33.480	38.430	21.590	1.00	0.00
ATOM	1698	OW	SOL	192	42.750	39.440	24.420	1.00	0.00
ATOM	1699	HW1	SOL	192	42.190	38.730	24.720	1.00	0.00
ATOM	1700	HW2	SOL	192	42.990	39.190	23.530	1.00	0.00
ATOM	1701	OW	SOL	193	7.130	42.830	36.400	1.00	0.00
ATOM	1702	HW1	SOL	193	7.420	43.040	35.510	1.00	0.00
ATOM	1703	HW2	SOL	193	7.020	41.880	36.400	1.00	0.00
ATOM	1704	OW	SOL	194	31.180	14.120	5.370	1.00	0.00
ATOM	1705	HW1	SOL	194	31.620	14.970	5.400	1.00	0.00
ATOM	1706	HW2	SOL	194	31.530	13.650	6.130	1.00	0.00
ATOM	1707	OW	SOL	195	17.760	23.800	21.560	1.00	0.00
ATOM	1708	HW1	SOL	195	17.110	23.660	20.870	1.00	0.00
ATOM	1709	HW2	SOL	195	18.580	23.480	21.180	1.00	0.00
ATOM	1710	OW	SOL	196	29.220	43.540	45.130	1.00	0.00
ATOM	1711	HW1	SOL	196	29.630	43.170	44.350	1.00	0.00
ATOM	1712	HW2	SOL	196	29.950	43.950	45.610	1.00	0.00
ATOM	1713	OW	SOL	197	16.430	22.970	28.930	1.00	0.00
ATOM	1714	HW1	SOL	197	17.030	22.560	29.560	1.00	0.00
ATOM	1715	HW2	SOL	197	15.820	23.460	29.480	1.00	0.00

ATOM	1716	OW	SOL	198	13.060	47.520	28.240	1.00	0.00
ATOM	1717	HW1	SOL	198	13.290	46.630	28.520	1.00	0.00
ATOM	1718	HW2	SOL	198	13.020	48.030	29.050	1.00	0.00
ATOM	1719	OW	SOL	199	48.780	11.720	4.430	1.00	0.00
ATOM	1720	HW1	SOL	199	48.500	12.320	5.120	1.00	0.00
ATOM	1721	HW2	SOL	199	47.990	11.230	4.200	1.00	0.00
ATOM	1722	OW	SOL	200	3.940	31.810	24.480	1.00	0.00
ATOM	1723	HW1	SOL	200	4.700	31.240	24.530	1.00	0.00
ATOM	1724	HW2	SOL	200	4.140	32.420	23.760	1.00	0.00
ATOM	1725	OW	SOL	201	5.930	45.160	3.170	1.00	0.00
ATOM	1726	HW1	SOL	201	5.690	44.240	3.260	1.00	0.00
ATOM	1727	HW2	SOL	201	5.320	45.510	2.520	1.00	0.00
ATOM	1728	OW	SOL	202	12.640	21.370	11.580	1.00	0.00
ATOM	1729	HW1	SOL	202	11.720	21.200	11.390	1.00	0.00
ATOM	1730	HW2	SOL	202	12.630	21.970	12.330	1.00	0.00
ATOM	1731	OW	SOL	203	50.430	41.350	22.370	1.00	0.00
ATOM	1732	HW1	SOL	203	50.670	41.030	23.240	1.00	0.00
ATOM	1733	HW2	SOL	203	51.250	41.690	22.010	1.00	0.00
ATOM	1734	OW	SOL	204	36.390	37.020	52.330	1.00	0.00
ATOM	1735	HW1	SOL	204	36.530	37.790	51.780	1.00	0.00
ATOM	1736	HW2	SOL	204	35.570	37.200	52.780	1.00	0.00
ATOM	1737	OW	SOL	205	23.990	17.530	49.290	1.00	0.00
ATOM	1738	HW1	SOL	205	24.050	18.120	48.540	1.00	0.00
ATOM	1739	HW2	SOL	205	23.820	16.670	48.910	1.00	0.00
ATOM	1740	OW	SOL	206	34.320	22.860	40.480	1.00	0.00
ATOM	1741	HW1	SOL	206	33.760	22.910	39.710	1.00	0.00
ATOM	1742	HW2	SOL	206	35.040	23.470	40.300	1.00	0.00
ATOM	1743	OW	SOL	207	3.350	54.530	32.380	1.00	0.00
ATOM	1744	HW1	SOL	207	3.830	54.870	31.620	1.00	0.00
ATOM	1745	HW2	SOL	207	3.910	53.850	32.740	1.00	0.00
ATOM	1746	OW	SOL	208	13.830	26.160	54.890	1.00	0.00
ATOM	1747	HW1	SOL	208	14.000	26.260	55.830	1.00	0.00
ATOM	1748	HW2	SOL	208	13.090	25.560	54.850	1.00	0.00
ATOM	1749	OW	SOL	209	49.370	25.490	29.000	1.00	0.00
ATOM	1750	HW1	SOL	209	48.470	25.150	28.970	1.00	0.00
ATOM	1751	HW2	SOL	209	49.810	25.060	28.270	1.00	0.00
ATOM	1752	OW	SOL	210	32.030	43.680	29.830	1.00	0.00
ATOM	1753	HW1	SOL	210	32.220	42.790	30.120	1.00	0.00
ATOM	1754	HW2	SOL	210	31.130	43.830	30.100	1.00	0.00
ATOM	1755	OW	SOL	211	0.860	43.840	45.180	1.00	0.00
ATOM	1756	HW1	SOL	211	1.670	44.000	45.670	1.00	0.00
ATOM	1757	HW2	SOL	211	0.370	44.650	45.270	1.00	0.00
ATOM	1758	OW	SOL	212	47.900	45.640	52.870	1.00	0.00
ATOM	1759	HW1	SOL	212	47.310	46.380	52.740	1.00	0.00

ATOM	1760	HW2	SOL	212	47.370	44.990	53.330	1.00	0.00
ATOM	1761	OW	SOL	213	21.280	23.540	14.860	1.00	0.00
ATOM	1762	HW1	SOL	213	20.680	23.450	15.610	1.00	0.00
ATOM	1763	HW2	SOL	213	21.610	22.650	14.720	1.00	0.00
ATOM	1764	OW	SOL	214	32.390	53.450	52.380	1.00	0.00
ATOM	1765	HW1	SOL	214	31.850	52.720	52.070	1.00	0.00
ATOM	1766	HW2	SOL	214	33.280	53.190	52.170	1.00	0.00
ATOM	1767	OW	SOL	215	23.680	24.230	35.860	1.00	0.00
ATOM	1768	HW1	SOL	215	23.300	23.410	36.190	1.00	0.00
ATOM	1769	HW2	SOL	215	24.600	24.020	35.720	1.00	0.00
ATOM	1770	OW	SOL	216	11.630	17.580	29.550	1.00	0.00
ATOM	1771	HW1	SOL	216	12.200	17.480	30.320	1.00	0.00
ATOM	1772	HW2	SOL	216	10.940	18.170	29.830	1.00	0.00
ATOM	1773	OW	SOL	217	14.620	45.210	26.490	1.00	0.00
ATOM	1774	HW1	SOL	217	14.000	44.570	26.840	1.00	0.00
ATOM	1775	HW2	SOL	217	14.970	44.800	25.700	1.00	0.00
ATOM	1776	OW	SOL	218	11.830	2.040	55.510	1.00	0.00
ATOM	1777	HW1	SOL	218	11.560	2.430	54.680	1.00	0.00
ATOM	1778	HW2	SOL	218	11.130	2.260	56.120	1.00	0.00
ATOM	1779	OW	SOL	219	27.440	29.180	6.940	1.00	0.00
ATOM	1780	HW1	SOL	219	28.330	29.120	7.290	1.00	0.00
ATOM	1781	HW2	SOL	219	26.880	28.990	7.690	1.00	0.00
ATOM	1782	OW	SOL	220	54.960	23.730	38.270	1.00	0.00
ATOM	1783	HW1	SOL	220	54.220	24.240	38.610	1.00	0.00
ATOM	1784	HW2	SOL	220	54.670	22.820	38.300	1.00	0.00
ATOM	1785	OW	SOL	221	40.550	32.130	19.880	1.00	0.00
ATOM	1786	HW1	SOL	221	40.490	32.300	18.940	1.00	0.00
ATOM	1787	HW2	SOL	221	40.080	32.860	20.290	1.00	0.00
ATOM	1788	OW	SOL	222	32.880	20.970	27.080	1.00	0.00
ATOM	1789	HW1	SOL	222	32.810	21.910	26.890	1.00	0.00
ATOM	1790	HW2	SOL	222	33.300	20.930	27.940	1.00	0.00
ATOM	1791	OW	SOL	223	14.240	41.460	47.370	1.00	0.00
ATOM	1792	HW1	SOL	223	15.040	41.440	46.850	1.00	0.00
ATOM	1793	HW2	SOL	223	13.960	42.370	47.340	1.00	0.00
ATOM	1794	OW	SOL	224	44.700	8.570	7.130	1.00	0.00
ATOM	1795	HW1	SOL	224	44.970	9.270	7.740	1.00	0.00
ATOM	1796	HW2	SOL	224	44.280	7.920	7.700	1.00	0.00
ATOM	1797	OW	SOL	225	19.260	50.690	48.310	1.00	0.00
ATOM	1798	HW1	SOL	225	18.370	50.370	48.450	1.00	0.00
ATOM	1799	HW2	SOL	225	19.150	51.600	48.020	1.00	0.00
ATOM	1800	OW	SOL	226	41.980	14.260	51.500	1.00	0.00
ATOM	1801	HW1	SOL	226	41.210	14.680	51.890	1.00	0.00
ATOM	1802	HW2	SOL	226	41.790	14.220	50.570	1.00	0.00
ATOM	1803	OW	SOL	227	24.220	17.810	12.060	1.00	0.00

ATOM	1804	HW1	SOL	227	24.940	17.560	11.480	1.00	0.00
ATOM	1805	HW2	SOL	227	23.680	17.020	12.120	1.00	0.00
ATOM	1806	OW	SOL	228	10.130	21.910	17.180	1.00	0.00
ATOM	1807	HW1	SOL	228	9.200	22.020	16.990	1.00	0.00
ATOM	1808	HW2	SOL	228	10.350	21.060	16.790	1.00	0.00
ATOM	1809	OW	SOL	229	21.070	17.960	33.280	1.00	0.00
ATOM	1810	HW1	SOL	229	20.930	17.220	33.870	1.00	0.00
ATOM	1811	HW2	SOL	229	21.540	17.580	32.530	1.00	0.00
ATOM	1812	OW	SOL	230	24.120	29.060	0.030	1.00	0.00
ATOM	1813	HW1	SOL	230	24.690	29.210	0.790	1.00	0.00
ATOM	1814	HW2	SOL	230	24.580	28.400	-0.490	1.00	0.00
ATOM	1815	OW	SOL	231	39.030	4.460	14.920	1.00	0.00
ATOM	1816	HW1	SOL	231	38.680	3.570	14.910	1.00	0.00
ATOM	1817	HW2	SOL	231	38.480	4.940	14.300	1.00	0.00
ATOM	1818	OW	SOL	232	19.850	53.700	48.040	1.00	0.00
ATOM	1819	HW1	SOL	232	20.470	53.790	47.320	1.00	0.00
ATOM	1820	HW2	SOL	232	20.300	54.100	48.790	1.00	0.00
ATOM	1821	OW	SOL	233	34.220	34.550	49.900	1.00	0.00
ATOM	1822	HW1	SOL	233	34.690	34.830	49.110	1.00	0.00
ATOM	1823	HW2	SOL	233	33.730	35.320	50.180	1.00	0.00
ATOM	1824	OW	SOL	234	9.710	1.020	25.270	1.00	0.00
ATOM	1825	HW1	SOL	234	9.430	1.900	25.540	1.00	0.00
ATOM	1826	HW2	SOL	234	8.930	0.630	24.880	1.00	0.00
ATOM	1827	OW	SOL	235	1.060	6.480	18.730	1.00	0.00
ATOM	1828	HW1	SOL	235	1.020	6.040	19.570	1.00	0.00
ATOM	1829	HW2	SOL	235	1.950	6.810	18.660	1.00	0.00
ATOM	1830	OW	SOL	236	24.810	54.050	29.780	1.00	0.00
ATOM	1831	HW1	SOL	236	24.390	53.190	29.800	1.00	0.00
ATOM	1832	HW2	SOL	236	25.570	53.940	29.210	1.00	0.00
ATOM	1833	OW	SOL	237	44.200	23.040	43.220	1.00	0.00
ATOM	1834	HW1	SOL	237	43.650	23.790	43.000	1.00	0.00
ATOM	1835	HW2	SOL	237	45.030	23.410	43.500	1.00	0.00
ATOM	1836	OW	SOL	238	53.190	49.120	5.590	1.00	0.00
ATOM	1837	HW1	SOL	238	52.620	48.860	4.870	1.00	0.00
ATOM	1838	HW2	SOL	238	53.700	49.860	5.250	1.00	0.00
ATOM	1839	OW	SOL	239	12.800	2.220	2.710	1.00	0.00
ATOM	1840	HW1	SOL	239	13.620	2.710	2.700	1.00	0.00
ATOM	1841	HW2	SOL	239	12.130	2.860	2.450	1.00	0.00
ATOM	1842	OW	SOL	240	9.230	35.820	53.840	1.00	0.00
ATOM	1843	HW1	SOL	240	9.180	36.080	52.920	1.00	0.00
ATOM	1844	HW2	SOL	240	8.330	35.900	54.160	1.00	0.00
ATOM	1845	OW	SOL	241	35.020	2.360	44.540	1.00	0.00
ATOM	1846	HW1	SOL	241	35.750	2.610	45.100	1.00	0.00
ATOM	1847	HW2	SOL	241	34.680	3.190	44.210	1.00	0.00

ATOM	1848	OW	SOL	242	26.940	22.900	49.860	1.00	0.00
ATOM	1849	HW1	SOL	242	26.390	23.200	49.140	1.00	0.00
ATOM	1850	HW2	SOL	242	27.530	23.640	50.030	1.00	0.00
ATOM	1851	OW	SOL	243	22.540	28.220	10.360	1.00	0.00
ATOM	1852	HW1	SOL	243	21.700	28.690	10.350	1.00	0.00
ATOM	1853	HW2	SOL	243	23.040	28.650	11.050	1.00	0.00
ATOM	1854	OW	SOL	244	52.090	55.650	38.250	1.00	0.00
ATOM	1855	HW1	SOL	244	53.040	55.680	38.200	1.00	0.00
ATOM	1856	HW2	SOL	244	51.850	54.820	37.850	1.00	0.00
ATOM	1857	OW	SOL	245	12.920	14.920	5.480	1.00	0.00
ATOM	1858	HW1	SOL	245	12.910	15.860	5.280	1.00	0.00
ATOM	1859	HW2	SOL	245	13.830	14.650	5.330	1.00	0.00
ATOM	1860	OW	SOL	246	16.680	17.060	7.560	1.00	0.00
ATOM	1861	HW1	SOL	246	16.710	17.230	6.610	1.00	0.00
ATOM	1862	HW2	SOL	246	17.070	17.850	7.950	1.00	0.00
ATOM	1863	OW	SOL	247	31.190	6.800	7.530	1.00	0.00
ATOM	1864	HW1	SOL	247	30.670	7.260	8.190	1.00	0.00
ATOM	1865	HW2	SOL	247	30.790	7.050	6.690	1.00	0.00
ATOM	1866	OW	SOL	248	32.320	1.390	44.430	1.00	0.00
ATOM	1867	HW1	SOL	248	33.270	1.520	44.460	1.00	0.00
ATOM	1868	HW2	SOL	248	32.130	0.800	45.160	1.00	0.00
ATOM	1869	OW	SOL	249	13.520	15.020	49.400	1.00	0.00
ATOM	1870	HW1	SOL	249	13.160	15.460	48.630	1.00	0.00
ATOM	1871	HW2	SOL	249	12.830	15.070	50.060	1.00	0.00
ATOM	1872	OW	SOL	250	32.460	41.290	30.880	1.00	0.00
ATOM	1873	HW1	SOL	250	32.810	41.760	31.650	1.00	0.00
ATOM	1874	HW2	SOL	250	33.240	40.910	30.470	1.00	0.00
ATOM	1875	OW	SOL	251	20.150	7.890	12.380	1.00	0.00
ATOM	1876	HW1	SOL	251	21.040	7.530	12.310	1.00	0.00
ATOM	1877	HW2	SOL	251	20.030	8.030	13.320	1.00	0.00
ATOM	1878	OW	SOL	252	26.840	0.600	3.960	1.00	0.00
ATOM	1879	HW1	SOL	252	27.530	1.240	4.100	1.00	0.00
ATOM	1880	HW2	SOL	252	26.120	1.090	3.570	1.00	0.00
ATOM	1881	OW	SOL	253	17.390	0.500	47.590	1.00	0.00
ATOM	1882	HW1	SOL	253	18.200	0.800	47.180	1.00	0.00
ATOM	1883	HW2	SOL	253	17.120	-0.250	47.060	1.00	0.00
ATOM	1884	OW	SOL	254	22.470	44.680	54.840	1.00	0.00
ATOM	1885	HW1	SOL	254	22.410	44.020	54.140	1.00	0.00
ATOM	1886	HW2	SOL	254	23.260	44.450	55.310	1.00	0.00
ATOM	1887	OW	SOL	255	49.510	1.020	41.260	1.00	0.00
ATOM	1888	HW1	SOL	255	49.720	1.780	41.810	1.00	0.00
ATOM	1889	HW2	SOL	255	49.730	0.270	41.810	1.00	0.00
ATOM	1890	OW	SOL	256	30.100	12.730	18.280	1.00	0.00
ATOM	1891	HW1	SOL	256	29.460	13.410	18.500	1.00	0.00

ATOM	1892	HW2	SOL	256	29.790	11.950	18.730	1.00	0.00
ATOM	1893	OW	SOL	257	1.930	23.010	2.780	1.00	0.00
ATOM	1894	HW1	SOL	257	2.490	23.060	3.560	1.00	0.00
ATOM	1895	HW2	SOL	257	1.620	23.900	2.640	1.00	0.00
ATOM	1896	OW	SOL	258	8.860	37.220	14.600	1.00	0.00
ATOM	1897	HW1	SOL	258	8.960	36.310	14.850	1.00	0.00
ATOM	1898	HW2	SOL	258	8.590	37.670	15.400	1.00	0.00
ATOM	1899	OW	SOL	259	9.470	1.490	7.980	1.00	0.00
ATOM	1900	HW1	SOL	259	10.370	1.220	8.200	1.00	0.00
ATOM	1901	HW2	SOL	259	9.130	1.860	8.800	1.00	0.00
ATOM	1902	OW	SOL	260	55.210	9.430	22.240	1.00	0.00
ATOM	1903	HW1	SOL	260	55.390	8.560	22.580	1.00	0.00
ATOM	1904	HW2	SOL	260	55.970	9.650	21.710	1.00	0.00
ATOM	1905	OW	SOL	261	5.830	42.560	3.280	1.00	0.00
ATOM	1906	HW1	SOL	261	5.100	42.040	3.600	1.00	0.00
ATOM	1907	HW2	SOL	261	6.610	42.090	3.590	1.00	0.00
ATOM	1908	OW	SOL	262	20.990	41.710	45.390	1.00	0.00
ATOM	1909	HW1	SOL	262	20.080	41.610	45.680	1.00	0.00
ATOM	1910	HW2	SOL	262	21.060	41.140	44.620	1.00	0.00
ATOM	1911	OW	SOL	263	49.070	13.760	25.930	1.00	0.00
ATOM	1912	HW1	SOL	263	48.390	13.700	25.270	1.00	0.00
ATOM	1913	HW2	SOL	263	48.660	13.460	26.740	1.00	0.00
ATOM	1914	OW	SOL	264	50.950	51.270	34.990	1.00	0.00
ATOM	1915	HW1	SOL	264	50.870	50.600	34.310	1.00	0.00
ATOM	1916	HW2	SOL	264	51.760	51.740	34.770	1.00	0.00
ATOM	1917	OW	SOL	265	37.340	0.280	39.540	1.00	0.00
ATOM	1918	HW1	SOL	265	37.730	-0.050	38.730	1.00	0.00
ATOM	1919	HW2	SOL	265	37.540	-0.390	40.190	1.00	0.00
ATOM	1920	OW	SOL	266	39.510	41.460	54.840	1.00	0.00
ATOM	1921	HW1	SOL	266	39.490	40.510	54.710	1.00	0.00
ATOM	1922	HW2	SOL	266	40.270	41.760	54.340	1.00	0.00
ATOM	1923	OW	SOL	267	42.280	17.890	32.920	1.00	0.00
ATOM	1924	HW1	SOL	267	42.650	18.650	32.480	1.00	0.00
ATOM	1925	HW2	SOL	267	42.840	17.740	33.680	1.00	0.00
ATOM	1926	OW	SOL	268	22.610	50.960	6.360	1.00	0.00
ATOM	1927	HW1	SOL	268	23.310	50.980	5.710	1.00	0.00
ATOM	1928	HW2	SOL	268	22.770	50.170	6.870	1.00	0.00
ATOM	1929	OW	SOL	269	55.340	34.280	20.800	1.00	0.00
ATOM	1930	HW1	SOL	269	55.750	33.980	21.610	1.00	0.00
ATOM	1931	HW2	SOL	269	56.060	34.300	20.170	1.00	0.00
ATOM	1932	OW	SOL	270	54.630	42.140	19.580	1.00	0.00
ATOM	1933	HW1	SOL	270	53.820	41.690	19.360	1.00	0.00
ATOM	1934	HW2	SOL	270	54.850	42.650	18.800	1.00	0.00
ATOM	1935	OW	SOL	271	11.230	12.360	52.670	1.00	0.00

ATOM	1936	HW1	SOL	271	11.310	12.270	53.620	1.00	0.00
ATOM	1937	HW2	SOL	271	10.290	12.360	52.510	1.00	0.00
ATOM	1938	OW	SOL	272	8.620	9.660	3.800	1.00	0.00
ATOM	1939	HW1	SOL	272	7.860	9.640	4.390	1.00	0.00
ATOM	1940	HW2	SOL	272	8.600	8.820	3.350	1.00	0.00
ATOM	1941	OW	SOL	273	26.990	53.360	22.380	1.00	0.00
ATOM	1942	HW1	SOL	273	27.040	53.770	21.520	1.00	0.00
ATOM	1943	HW2	SOL	273	27.670	53.800	22.900	1.00	0.00
ATOM	1944	OW	SOL	274	14.420	1.410	6.870	1.00	0.00
ATOM	1945	HW1	SOL	274	14.310	2.360	6.860	1.00	0.00
ATOM	1946	HW2	SOL	274	14.770	1.200	6.010	1.00	0.00
ATOM	1947	OW	SOL	275	9.940	33.220	50.160	1.00	0.00
ATOM	1948	HW1	SOL	275	9.260	33.850	50.430	1.00	0.00
ATOM	1949	HW2	SOL	275	9.570	32.370	50.380	1.00	0.00
ATOM	1950	OW	SOL	276	19.150	15.360	44.700	1.00	0.00
ATOM	1951	HW1	SOL	276	20.020	15.410	44.280	1.00	0.00
ATOM	1952	HW2	SOL	276	18.860	16.270	44.740	1.00	0.00
ATOM	1953	OW	SOL	277	16.600	2.500	3.720	1.00	0.00
ATOM	1954	HW1	SOL	277	15.830	2.080	3.350	1.00	0.00
ATOM	1955	HW2	SOL	277	17.230	2.530	3.010	1.00	0.00
ATOM	1956	OW	SOL	278	55.810	11.010	9.140	1.00	0.00
ATOM	1957	HW1	SOL	278	55.930	11.340	8.250	1.00	0.00
ATOM	1958	HW2	SOL	278	55.590	11.790	9.650	1.00	0.00
ATOM	1959	OW	SOL	279	24.780	18.780	32.660	1.00	0.00
ATOM	1960	HW1	SOL	279	24.070	18.370	32.180	1.00	0.00
ATOM	1961	HW2	SOL	279	24.380	19.560	33.060	1.00	0.00
ATOM	1962	OW	SOL	280	44.120	40.990	6.900	1.00	0.00
ATOM	1963	HW1	SOL	280	43.540	41.160	7.640	1.00	0.00
ATOM	1964	HW2	SOL	280	44.910	41.500	7.090	1.00	0.00
ATOM	1965	OW	SOL	281	10.580	12.490	37.830	1.00	0.00
ATOM	1966	HW1	SOL	281	10.800	13.390	37.570	1.00	0.00
ATOM	1967	HW2	SOL	281	11.360	12.180	38.300	1.00	0.00
ATOM	1968	OW	SOL	282	8.170	29.120	12.300	1.00	0.00
ATOM	1969	HW1	SOL	282	8.450	29.930	11.900	1.00	0.00
ATOM	1970	HW2	SOL	282	8.200	29.290	13.240	1.00	0.00
ATOM	1971	OW	SOL	283	22.450	17.440	18.490	1.00	0.00
ATOM	1972	HW1	SOL	283	22.730	16.890	17.760	1.00	0.00
ATOM	1973	HW2	SOL	283	22.790	18.310	18.270	1.00	0.00
ATOM	1974	OW	SOL	284	35.050	38.110	40.880	1.00	0.00
ATOM	1975	HW1	SOL	284	35.140	39.030	41.130	1.00	0.00
ATOM	1976	HW2	SOL	284	35.530	37.640	41.550	1.00	0.00
ATOM	1977	OW	SOL	285	8.920	53.260	47.420	1.00	0.00
ATOM	1978	HW1	SOL	285	9.320	52.400	47.290	1.00	0.00
ATOM	1979	HW2	SOL	285	8.670	53.540	46.540	1.00	0.00

ATOM	1980	OW	SOL	286	27.670	24.960	36.920	1.00	0.00
ATOM	1981	HW1	SOL	286	27.320	25.060	37.800	1.00	0.00
ATOM	1982	HW2	SOL	286	28.620	25.100	37.030	1.00	0.00
ATOM	1983	OW	SOL	287	48.670	9.160	29.390	1.00	0.00
ATOM	1984	HW1	SOL	287	49.000	9.580	28.600	1.00	0.00
ATOM	1985	HW2	SOL	287	48.450	9.890	29.980	1.00	0.00
ATOM	1986	OW	SOL	288	46.160	54.360	1.560	1.00	0.00
ATOM	1987	HW1	SOL	288	46.260	55.190	2.010	1.00	0.00
ATOM	1988	HW2	SOL	288	46.980	53.890	1.740	1.00	0.00
ATOM	1989	OW	SOL	289	13.440	28.000	16.930	1.00	0.00
ATOM	1990	HW1	SOL	289	14.070	27.310	16.740	1.00	0.00
ATOM	1991	HW2	SOL	289	12.650	27.530	17.210	1.00	0.00
ATOM	1992	OW	SOL	290	30.960	54.630	12.340	1.00	0.00
ATOM	1993	HW1	SOL	290	30.070	54.500	12.660	1.00	0.00
ATOM	1994	HW2	SOL	290	31.470	53.940	12.760	1.00	0.00
ATOM	1995	OW	SOL	291	43.940	50.690	21.950	1.00	0.00
ATOM	1996	HW1	SOL	291	44.730	50.520	21.440	1.00	0.00
ATOM	1997	HW2	SOL	291	43.250	50.180	21.510	1.00	0.00
ATOM	1998	OW	SOL	292	40.070	48.690	52.660	1.00	0.00
ATOM	1999	HW1	SOL	292	40.870	48.360	53.060	1.00	0.00
ATOM	2000	HW2	SOL	292	40.230	49.630	52.550	1.00	0.00
ATOM	2001	OW	SOL	293	33.080	19.310	19.080	1.00	0.00
ATOM	2002	HW1	SOL	293	32.980	18.560	19.660	1.00	0.00
ATOM	2003	HW2	SOL	293	34.030	19.480	19.070	1.00	0.00
ATOM	2004	OW	SOL	294	0.500	48.160	11.190	1.00	0.00
ATOM	2005	HW1	SOL	294	0.740	48.000	12.100	1.00	0.00
ATOM	2006	HW2	SOL	294	1.110	47.620	10.690	1.00	0.00
ATOM	2007	OW	SOL	295	30.980	55.460	49.680	1.00	0.00
ATOM	2008	HW1	SOL	295	31.460	55.660	50.480	1.00	0.00
ATOM	2009	HW2	SOL	295	31.190	56.180	49.090	1.00	0.00
ATOM	2010	OW	SOL	296	12.140	0.700	14.950	1.00	0.00
ATOM	2011	HW1	SOL	296	12.580	1.500	15.240	1.00	0.00
ATOM	2012	HW2	SOL	296	12.390	0.610	14.030	1.00	0.00
ATOM	2013	OW	SOL	297	47.100	1.290	38.020	1.00	0.00
ATOM	2014	HW1	SOL	297	47.300	0.470	38.470	1.00	0.00
ATOM	2015	HW2	SOL	297	47.090	1.950	38.730	1.00	0.00
ATOM	2016	OW	SOL	298	6.430	30.200	27.990	1.00	0.00
ATOM	2017	HW1	SOL	298	5.670	29.630	27.980	1.00	0.00
ATOM	2018	HW2	SOL	298	6.070	31.090	27.910	1.00	0.00
ATOM	2019	OW	SOL	299	16.760	16.050	37.870	1.00	0.00
ATOM	2020	HW1	SOL	299	16.860	17.000	37.880	1.00	0.00
ATOM	2021	HW2	SOL	299	16.460	15.850	36.980	1.00	0.00
ATOM	2022	OW	SOL	300	35.090	16.050	12.850	1.00	0.00
ATOM	2023	HW1	SOL	300	34.590	15.450	12.290	1.00	0.00

ATOM	2024	HW2	SOL	300	35.380	15.510	13.580	1.00	0.00
ATOM	2025	OW	SOL	301	3.820	45.990	38.900	1.00	0.00
ATOM	2026	HW1	SOL	301	4.220	45.700	38.080	1.00	0.00
ATOM	2027	HW2	SOL	301	3.250	46.720	38.640	1.00	0.00
ATOM	2028	OW	SOL	302	32.140	46.660	47.240	1.00	0.00
ATOM	2029	HW1	SOL	302	31.680	46.590	48.080	1.00	0.00
ATOM	2030	HW2	SOL	302	32.750	47.390	47.360	1.00	0.00
ATOM	2031	OW	SOL	303	2.820	24.690	10.680	1.00	0.00
ATOM	2032	HW1	SOL	303	2.510	23.910	11.150	1.00	0.00
ATOM	2033	HW2	SOL	303	2.030	25.120	10.370	1.00	0.00
ATOM	2034	OW	SOL	304	32.220	18.900	43.290	1.00	0.00
ATOM	2035	HW1	SOL	304	31.560	18.390	42.820	1.00	0.00
ATOM	2036	HW2	SOL	304	32.830	18.240	43.630	1.00	0.00
ATOM	2037	OW	SOL	305	48.120	15.590	52.660	1.00	0.00
ATOM	2038	HW1	SOL	305	48.240	15.390	51.730	1.00	0.00
ATOM	2039	HW2	SOL	305	47.700	14.820	53.020	1.00	0.00
ATOM	2040	OW	SOL	306	32.020	36.370	16.380	1.00	0.00
ATOM	2041	HW1	SOL	306	31.160	36.020	16.620	1.00	0.00
ATOM	2042	HW2	SOL	306	32.370	35.740	15.760	1.00	0.00
ATOM	2043	OW	SOL	307	49.440	32.120	46.910	1.00	0.00
ATOM	2044	HW1	SOL	307	48.720	32.320	47.520	1.00	0.00
ATOM	2045	HW2	SOL	307	50.160	32.680	47.190	1.00	0.00
ATOM	2046	OW	SOL	308	33.850	23.880	25.730	1.00	0.00
ATOM	2047	HW1	SOL	308	33.180	23.610	25.100	1.00	0.00
ATOM	2048	HW2	SOL	308	34.680	23.780	25.260	1.00	0.00
ATOM	2049	OW	SOL	309	22.030	51.970	10.320	1.00	0.00
ATOM	2050	HW1	SOL	309	22.710	52.090	9.660	1.00	0.00
ATOM	2051	HW2	SOL	309	21.230	52.300	9.910	1.00	0.00
ATOM	2052	OW	SOL	310	23.300	20.710	55.260	1.00	0.00
ATOM	2053	HW1	SOL	310	23.140	20.920	56.180	1.00	0.00
ATOM	2054	HW2	SOL	310	23.900	21.390	54.960	1.00	0.00
ATOM	2055	OW	SOL	311	46.140	2.620	42.540	1.00	0.00
ATOM	2056	HW1	SOL	311	45.600	3.010	43.230	1.00	0.00
ATOM	2057	HW2	SOL	311	46.700	1.990	42.990	1.00	0.00
ATOM	2058	OW	SOL	312	20.870	10.710	38.080	1.00	0.00
ATOM	2059	HW1	SOL	312	20.420	11.450	38.490	1.00	0.00
ATOM	2060	HW2	SOL	312	20.630	10.770	37.160	1.00	0.00
ATOM	2061	OW	SOL	313	1.720	50.500	37.310	1.00	0.00
ATOM	2062	HW1	SOL	313	2.040	50.680	36.430	1.00	0.00
ATOM	2063	HW2	SOL	313	1.040	51.160	37.460	1.00	0.00
ATOM	2064	OW	SOL	314	29.060	33.170	8.950	1.00	0.00
ATOM	2065	HW1	SOL	314	28.380	33.620	8.440	1.00	0.00
ATOM	2066	HW2	SOL	314	29.850	33.690	8.800	1.00	0.00
ATOM	2067	OW	SOL	315	41.550	23.300	16.700	1.00	0.00

ATOM	2068	HW1	SOL	315	41.110	23.780	17.400	1.00	0.00
ATOM	2069	HW2	SOL	315	41.490	22.380	16.960	1.00	0.00
ATOM	2070	OW	SOL	316	18.290	44.590	7.350	1.00	0.00
ATOM	2071	HW1	SOL	316	18.100	44.340	6.440	1.00	0.00
ATOM	2072	HW2	SOL	316	18.610	43.780	7.750	1.00	0.00
ATOM	2073	OW	SOL	317	54.700	1.900	7.910	1.00	0.00
ATOM	2074	HW1	SOL	317	54.760	1.540	7.030	1.00	0.00
ATOM	2075	HW2	SOL	317	54.310	1.200	8.430	1.00	0.00
ATOM	2076	OW	SOL	318	27.840	7.370	52.190	1.00	0.00
ATOM	2077	HW1	SOL	318	27.570	8.130	51.680	1.00	0.00
ATOM	2078	HW2	SOL	318	28.550	6.980	51.680	1.00	0.00
ATOM	2079	OW	SOL	319	25.200	51.650	1.740	1.00	0.00
ATOM	2080	HW1	SOL	319	25.700	51.060	2.300	1.00	0.00
ATOM	2081	HW2	SOL	319	25.650	51.610	0.890	1.00	0.00
ATOM	2082	OW	SOL	320	16.430	1.350	15.490	1.00	0.00
ATOM	2083	HW1	SOL	320	17.070	0.640	15.430	1.00	0.00
ATOM	2084	HW2	SOL	320	16.720	1.870	16.240	1.00	0.00
ATOM	2085	OW	SOL	321	52.330	21.980	16.710	1.00	0.00
ATOM	2086	HW1	SOL	321	51.410	21.760	16.830	1.00	0.00
ATOM	2087	HW2	SOL	321	52.490	22.700	17.320	1.00	0.00
ATOM	2088	OW	SOL	322	30.360	1.010	24.390	1.00	0.00
ATOM	2089	HW1	SOL	322	31.160	0.490	24.340	1.00	0.00
ATOM	2090	HW2	SOL	322	30.660	1.920	24.380	1.00	0.00
ATOM	2091	OW	SOL	323	15.940	26.860	53.130	1.00	0.00
ATOM	2092	HW1	SOL	323	16.140	27.800	53.060	1.00	0.00
ATOM	2093	HW2	SOL	323	15.080	26.830	53.550	1.00	0.00
ATOM	2094	OW	SOL	324	33.540	20.050	41.320	1.00	0.00
ATOM	2095	HW1	SOL	324	33.550	20.930	41.680	1.00	0.00
ATOM	2096	HW2	SOL	324	32.950	19.560	41.890	1.00	0.00
ATOM	2097	OW	SOL	325	34.520	49.110	1.940	1.00	0.00
ATOM	2098	HW1	SOL	325	35.060	49.750	2.410	1.00	0.00
ATOM	2099	HW2	SOL	325	33.620	49.430	2.050	1.00	0.00
ATOM	2100	OW	SOL	326	31.530	45.600	21.400	1.00	0.00
ATOM	2101	HW1	SOL	326	32.450	45.580	21.690	1.00	0.00
ATOM	2102	HW2	SOL	326	31.040	45.820	22.180	1.00	0.00
ATOM	2103	OW	SOL	327	30.770	16.300	20.240	1.00	0.00
ATOM	2104	HW1	SOL	327	30.870	16.290	21.190	1.00	0.00
ATOM	2105	HW2	SOL	327	30.030	15.720	20.080	1.00	0.00
ATOM	2106	OW	SOL	328	37.850	15.530	50.510	1.00	0.00
ATOM	2107	HW1	SOL	328	36.990	15.770	50.850	1.00	0.00
ATOM	2108	HW2	SOL	328	37.830	14.580	50.440	1.00	0.00
ATOM	2109	OW	SOL	329	44.730	45.070	39.320	1.00	0.00
ATOM	2110	HW1	SOL	329	44.810	44.440	38.600	1.00	0.00
ATOM	2111	HW2	SOL	329	45.520	45.610	39.250	1.00	0.00

ATOM	2112	OW	SOL	330	12.690	16.850	32.380	1.00	0.00
ATOM	2113	HW1	SOL	330	13.520	16.450	32.120	1.00	0.00
ATOM	2114	HW2	SOL	330	12.090	16.110	32.490	1.00	0.00
ATOM	2115	OW	SOL	331	26.890	29.090	25.340	1.00	0.00
ATOM	2116	HW1	SOL	331	27.240	29.760	25.920	1.00	0.00
ATOM	2117	HW2	SOL	331	27.590	28.460	25.240	1.00	0.00
ATOM	2118	OW	SOL	332	12.080	29.050	13.830	1.00	0.00
ATOM	2119	HW1	SOL	332	11.380	28.410	13.710	1.00	0.00
ATOM	2120	HW2	SOL	332	12.830	28.520	14.120	1.00	0.00
ATOM	2121	OW	SOL	333	11.900	46.880	15.810	1.00	0.00
ATOM	2122	HW1	SOL	333	11.870	46.070	16.320	1.00	0.00
ATOM	2123	HW2	SOL	333	12.660	47.350	16.160	1.00	0.00
ATOM	2124	OW	SOL	334	29.270	24.750	4.990	1.00	0.00
ATOM	2125	HW1	SOL	334	28.620	24.060	5.140	1.00	0.00
ATOM	2126	HW2	SOL	334	28.740	25.540	4.860	1.00	0.00
ATOM	2127	OW	SOL	335	53.110	28.430	31.470	1.00	0.00
ATOM	2128	HW1	SOL	335	52.970	28.240	32.400	1.00	0.00
ATOM	2129	HW2	SOL	335	52.400	27.970	31.020	1.00	0.00
ATOM	2130	OW	SOL	336	51.800	1.480	17.530	1.00	0.00
ATOM	2131	HW1	SOL	336	51.570	1.110	16.670	1.00	0.00
ATOM	2132	HW2	SOL	336	51.740	2.420	17.410	1.00	0.00
ATOM	2133	OW	SOL	337	7.470	33.390	35.680	1.00	0.00
ATOM	2134	HW1	SOL	337	6.840	33.780	35.080	1.00	0.00
ATOM	2135	HW2	SOL	337	7.880	32.680	35.180	1.00	0.00
ATOM	2136	OW	SOL	338	46.390	15.980	6.160	1.00	0.00
ATOM	2137	HW1	SOL	338	46.890	16.740	5.870	1.00	0.00
ATOM	2138	HW2	SOL	338	46.650	15.850	7.070	1.00	0.00
ATOM	2139	OW	SOL	339	46.270	44.870	8.970	1.00	0.00
ATOM	2140	HW1	SOL	339	46.530	44.870	8.050	1.00	0.00
ATOM	2141	HW2	SOL	339	47.070	44.640	9.440	1.00	0.00
ATOM	2142	OW	SOL	340	16.920	12.320	39.740	1.00	0.00
ATOM	2143	HW1	SOL	340	16.000	12.570	39.670	1.00	0.00
ATOM	2144	HW2	SOL	340	16.970	11.810	40.550	1.00	0.00
ATOM	2145	OW	SOL	341	12.030	50.800	35.620	1.00	0.00
ATOM	2146	HW1	SOL	341	11.920	51.150	36.500	1.00	0.00
ATOM	2147	HW2	SOL	341	12.460	49.950	35.760	1.00	0.00
ATOM	2148	OW	SOL	342	7.770	54.420	21.450	1.00	0.00
ATOM	2149	HW1	SOL	342	7.190	53.780	21.870	1.00	0.00
ATOM	2150	HW2	SOL	342	8.340	53.890	20.900	1.00	0.00
ATOM	2151	OW	SOL	343	26.150	1.790	47.250	1.00	0.00
ATOM	2152	HW1	SOL	343	26.210	1.480	46.340	1.00	0.00
ATOM	2153	HW2	SOL	343	25.250	1.570	47.510	1.00	0.00
ATOM	2154	OW	SOL	344	13.970	6.680	44.200	1.00	0.00
ATOM	2155	HW1	SOL	344	13.830	7.410	44.800	1.00	0.00

ATOM	2156	HW2	SOL	344	13.910	7.070	43.330	1.00	0.00
ATOM	2157	OW	SOL	345	33.730	51.710	39.410	1.00	0.00
ATOM	2158	HW1	SOL	345	34.200	52.220	38.750	1.00	0.00
ATOM	2159	HW2	SOL	345	34.010	50.800	39.250	1.00	0.00
ATOM	2160	OW	SOL	346	45.930	44.010	51.400	1.00	0.00
ATOM	2161	HW1	SOL	346	45.340	43.800	50.670	1.00	0.00
ATOM	2162	HW2	SOL	346	45.850	43.270	51.990	1.00	0.00
ATOM	2163	OW	SOL	347	38.690	4.440	37.370	1.00	0.00
ATOM	2164	HW1	SOL	347	38.400	3.860	36.670	1.00	0.00
ATOM	2165	HW2	SOL	347	38.580	3.930	38.170	1.00	0.00
ATOM	2166	OW	SOL	348	49.050	1.270	31.580	1.00	0.00
ATOM	2167	HW1	SOL	348	48.820	1.300	30.650	1.00	0.00
ATOM	2168	HW2	SOL	348	48.240	1.030	32.030	1.00	0.00
ATOM	2169	OW	SOL	349	3.460	24.680	24.570	1.00	0.00
ATOM	2170	HW1	SOL	349	2.590	24.430	24.870	1.00	0.00
ATOM	2171	HW2	SOL	349	3.870	25.100	25.330	1.00	0.00
ATOM	2172	OW	SOL	350	36.460	1.620	3.690	1.00	0.00
ATOM	2173	HW1	SOL	350	36.990	1.350	4.430	1.00	0.00
ATOM	2174	HW2	SOL	350	37.080	2.040	3.090	1.00	0.00
ATOM	2175	OW	SOL	351	46.550	24.140	44.370	1.00	0.00
ATOM	2176	HW1	SOL	351	46.890	23.910	45.240	1.00	0.00
ATOM	2177	HW2	SOL	351	46.280	25.050	44.450	1.00	0.00
ATOM	2178	OW	SOL	352	1.680	14.400	34.800	1.00	0.00
ATOM	2179	HW1	SOL	352	2.390	14.700	35.370	1.00	0.00
ATOM	2180	HW2	SOL	352	2.130	13.920	34.100	1.00	0.00
ATOM	2181	OW	SOL	353	26.570	51.520	17.120	1.00	0.00
ATOM	2182	HW1	SOL	353	26.130	51.770	17.920	1.00	0.00
ATOM	2183	HW2	SOL	353	27.230	50.880	17.400	1.00	0.00
ATOM	2184	OW	SOL	354	48.910	54.330	17.090	1.00	0.00
ATOM	2185	HW1	SOL	354	49.190	54.550	17.980	1.00	0.00
ATOM	2186	HW2	SOL	354	48.010	54.630	17.040	1.00	0.00
ATOM	2187	OW	SOL	355	34.110	9.710	0.080	1.00	0.00
ATOM	2188	HW1	SOL	355	34.840	10.040	-0.450	1.00	0.00
ATOM	2189	HW2	SOL	355	33.820	8.930	-0.380	1.00	0.00
ATOM	2190	OW	SOL	356	22.220	34.970	47.440	1.00	0.00
ATOM	2191	HW1	SOL	356	22.740	34.670	46.690	1.00	0.00
ATOM	2192	HW2	SOL	356	22.790	35.600	47.880	1.00	0.00
ATOM	2193	OW	SOL	357	45.890	37.230	16.110	1.00	0.00
ATOM	2194	HW1	SOL	357	45.170	37.580	16.640	1.00	0.00
ATOM	2195	HW2	SOL	357	45.580	36.370	15.820	1.00	0.00
ATOM	2196	OW	SOL	358	38.500	30.660	16.390	1.00	0.00
ATOM	2197	HW1	SOL	358	39.260	30.090	16.280	1.00	0.00
ATOM	2198	HW2	SOL	358	37.880	30.130	16.900	1.00	0.00
ATOM	2199	OW	SOL	359	9.150	48.320	49.790	1.00	0.00

ATOM	2200	HW1	SOL	359	9.410	48.940	49.120	1.00	0.00
ATOM	2201	HW2	SOL	359	9.120	48.840	50.600	1.00	0.00
ATOM	2202	OW	SOL	360	35.270	40.100	26.460	1.00	0.00
ATOM	2203	HW1	SOL	360	34.600	39.440	26.280	1.00	0.00
ATOM	2204	HW2	SOL	360	35.070	40.820	25.860	1.00	0.00
ATOM	2205	OW	SOL	361	6.800	8.230	13.070	1.00	0.00
ATOM	2206	HW1	SOL	361	6.170	7.600	13.420	1.00	0.00
ATOM	2207	HW2	SOL	361	6.420	9.080	13.270	1.00	0.00
ATOM	2208	OW	SOL	362	10.070	54.020	51.620	1.00	0.00
ATOM	2209	HW1	SOL	362	9.680	54.700	51.080	1.00	0.00
ATOM	2210	HW2	SOL	362	10.810	54.460	52.060	1.00	0.00
ATOM	2211	OW	SOL	363	12.640	24.130	23.270	1.00	0.00
ATOM	2212	HW1	SOL	363	12.860	23.590	24.030	1.00	0.00
ATOM	2213	HW2	SOL	363	12.330	23.510	22.620	1.00	0.00
ATOM	2214	OW	SOL	364	18.890	47.030	5.590	1.00	0.00
ATOM	2215	HW1	SOL	364	18.550	46.330	6.150	1.00	0.00
ATOM	2216	HW2	SOL	364	18.190	47.190	4.950	1.00	0.00
ATOM	2217	OW	SOL	365	39.800	22.710	36.540	1.00	0.00
ATOM	2218	HW1	SOL	365	38.970	22.820	37.000	1.00	0.00
ATOM	2219	HW2	SOL	365	39.560	22.320	35.700	1.00	0.00
ATOM	2220	OW	SOL	366	54.390	23.840	25.370	1.00	0.00
ATOM	2221	HW1	SOL	366	53.460	23.740	25.160	1.00	0.00
ATOM	2222	HW2	SOL	366	54.490	24.780	25.540	1.00	0.00
ATOM	2223	OW	SOL	367	13.190	22.580	54.730	1.00	0.00
ATOM	2224	HW1	SOL	367	13.490	22.820	55.610	1.00	0.00
ATOM	2225	HW2	SOL	367	12.990	21.640	54.790	1.00	0.00
ATOM	2226	OW	SOL	368	19.830	1.830	46.670	1.00	0.00
ATOM	2227	HW1	SOL	368	19.250	2.460	46.260	1.00	0.00
ATOM	2228	HW2	SOL	368	19.940	1.140	46.010	1.00	0.00
ATOM	2229	OW	SOL	369	11.370	42.800	14.220	1.00	0.00
ATOM	2230	HW1	SOL	369	11.330	43.470	13.540	1.00	0.00
ATOM	2231	HW2	SOL	369	11.380	41.970	13.740	1.00	0.00
ATOM	2232	OW	SOL	370	48.950	14.850	49.680	1.00	0.00
ATOM	2233	HW1	SOL	370	48.210	15.020	49.100	1.00	0.00
ATOM	2234	HW2	SOL	370	49.430	15.690	49.700	1.00	0.00
ATOM	2235	OW	SOL	371	18.190	11.870	1.520	1.00	0.00
ATOM	2236	HW1	SOL	371	18.820	11.850	0.790	1.00	0.00
ATOM	2237	HW2	SOL	371	18.710	12.110	2.280	1.00	0.00
ATOM	2238	OW	SOL	372	35.800	12.260	55.060	1.00	0.00
ATOM	2239	HW1	SOL	372	36.570	12.750	55.360	1.00	0.00
ATOM	2240	HW2	SOL	372	36.030	11.980	54.170	1.00	0.00
ATOM	2241	OW	SOL	373	36.940	13.030	12.260	1.00	0.00
ATOM	2242	HW1	SOL	373	36.210	12.480	11.980	1.00	0.00
ATOM	2243	HW2	SOL	373	36.720	13.270	13.160	1.00	0.00

ATOM	2244	OW	SOL	374	17.570	15.320	51.780	1.00	0.00
ATOM	2245	HW1	SOL	374	17.700	15.130	52.710	1.00	0.00
ATOM	2246	HW2	SOL	374	16.750	14.860	51.550	1.00	0.00
ATOM	2247	OW	SOL	375	33.570	21.410	53.090	1.00	0.00
ATOM	2248	HW1	SOL	375	32.810	20.840	53.260	1.00	0.00
ATOM	2249	HW2	SOL	375	33.500	21.630	52.170	1.00	0.00
ATOM	2250	OW	SOL	376	4.110	52.390	42.420	1.00	0.00
ATOM	2251	HW1	SOL	376	4.550	52.740	41.640	1.00	0.00
ATOM	2252	HW2	SOL	376	3.970	51.460	42.220	1.00	0.00
ATOM	2253	OW	SOL	377	8.990	43.140	23.480	1.00	0.00
ATOM	2254	HW1	SOL	377	8.870	44.090	23.430	1.00	0.00
ATOM	2255	HW2	SOL	377	9.470	42.910	22.690	1.00	0.00
ATOM	2256	OW	SOL	378	41.660	44.080	3.180	1.00	0.00
ATOM	2257	HW1	SOL	378	41.530	45.020	3.310	1.00	0.00
ATOM	2258	HW2	SOL	378	41.080	43.860	2.450	1.00	0.00
ATOM	2259	OW	SOL	379	19.180	7.810	21.880	1.00	0.00
ATOM	2260	HW1	SOL	379	19.430	7.360	22.680	1.00	0.00
ATOM	2261	HW2	SOL	379	18.400	8.310	22.120	1.00	0.00
ATOM	2262	OW	SOL	380	16.350	27.770	4.480	1.00	0.00
ATOM	2263	HW1	SOL	380	16.850	27.120	3.980	1.00	0.00
ATOM	2264	HW2	SOL	380	15.910	27.260	5.160	1.00	0.00
ATOM	2265	OW	SOL	381	31.070	10.390	35.530	1.00	0.00
ATOM	2266	HW1	SOL	381	31.070	10.710	34.630	1.00	0.00
ATOM	2267	HW2	SOL	381	30.650	11.100	36.030	1.00	0.00
ATOM	2268	OW	SOL	382	31.970	31.290	8.160	1.00	0.00
ATOM	2269	HW1	SOL	382	32.800	30.990	8.530	1.00	0.00
ATOM	2270	HW2	SOL	382	31.330	30.640	8.430	1.00	0.00
ATOM	2271	OW	SOL	383	52.970	12.820	17.380	1.00	0.00
ATOM	2272	HW1	SOL	383	52.870	12.080	16.780	1.00	0.00
ATOM	2273	HW2	SOL	383	53.660	12.540	17.990	1.00	0.00
ATOM	2274	OW	SOL	384	37.820	33.260	1.110	1.00	0.00
ATOM	2275	HW1	SOL	384	37.080	33.870	1.040	1.00	0.00
ATOM	2276	HW2	SOL	384	37.720	32.670	0.360	1.00	0.00
ATOM	2277	OW	SOL	385	17.140	15.650	22.970	1.00	0.00
ATOM	2278	HW1	SOL	385	17.160	16.530	23.360	1.00	0.00
ATOM	2279	HW2	SOL	385	16.390	15.230	23.370	1.00	0.00
ATOM	2280	OW	SOL	386	10.300	42.950	20.960	1.00	0.00
ATOM	2281	HW1	SOL	386	10.610	43.850	20.950	1.00	0.00
ATOM	2282	HW2	SOL	386	9.670	42.900	20.240	1.00	0.00
ATOM	2283	OW	SOL	387	30.700	1.490	11.930	1.00	0.00
ATOM	2284	HW1	SOL	387	31.190	1.610	11.110	1.00	0.00
ATOM	2285	HW2	SOL	387	30.620	0.540	12.010	1.00	0.00
ATOM	2286	OW	SOL	388	13.720	20.550	14.610	1.00	0.00
ATOM	2287	HW1	SOL	388	14.240	21.200	14.150	1.00	0.00

ATOM	2288	HW2	SOL	388	12.820	20.730	14.340	1.00	0.00
ATOM	2289	OW	SOL	389	20.020	32.220	53.180	1.00	0.00
ATOM	2290	HW1	SOL	389	20.320	33.060	53.530	1.00	0.00
ATOM	2291	HW2	SOL	389	20.630	31.570	53.530	1.00	0.00
ATOM	2292	OW	SOL	390	17.390	46.830	9.240	1.00	0.00
ATOM	2293	HW1	SOL	390	17.420	46.130	8.590	1.00	0.00
ATOM	2294	HW2	SOL	390	16.450	46.950	9.410	1.00	0.00
ATOM	2295	OW	SOL	391	20.180	26.580	4.280	1.00	0.00
ATOM	2296	HW1	SOL	391	20.450	27.450	3.980	1.00	0.00
ATOM	2297	HW2	SOL	391	20.300	26.610	5.230	1.00	0.00
ATOM	2298	OW	SOL	392	27.130	18.060	30.890	1.00	0.00
ATOM	2299	HW1	SOL	392	28.030	18.280	30.660	1.00	0.00
ATOM	2300	HW2	SOL	392	26.820	18.800	31.400	1.00	0.00
ATOM	2301	OW	SOL	393	48.420	37.790	41.930	1.00	0.00
ATOM	2302	HW1	SOL	393	47.880	38.420	41.460	1.00	0.00
ATOM	2303	HW2	SOL	393	48.420	37.010	41.380	1.00	0.00
ATOM	2304	OW	SOL	394	36.750	47.130	10.490	1.00	0.00
ATOM	2305	HW1	SOL	394	36.560	47.780	9.820	1.00	0.00
ATOM	2306	HW2	SOL	394	36.140	46.420	10.310	1.00	0.00
ATOM	2307	OW	SOL	395	18.980	49.880	18.530	1.00	0.00
ATOM	2308	HW1	SOL	395	18.130	49.920	18.960	1.00	0.00
ATOM	2309	HW2	SOL	395	18.980	49.040	18.070	1.00	0.00
ATOM	2310	OW	SOL	396	29.670	49.140	44.490	1.00	0.00
ATOM	2311	HW1	SOL	396	29.200	48.380	44.840	1.00	0.00
ATOM	2312	HW2	SOL	396	29.520	49.830	45.130	1.00	0.00
ATOM	2313	OW	SOL	397	36.040	26.320	5.830	1.00	0.00
ATOM	2314	HW1	SOL	397	36.980	26.170	5.830	1.00	0.00
ATOM	2315	HW2	SOL	397	35.690	25.720	6.490	1.00	0.00
ATOM	2316	OW	SOL	398	9.750	35.700	26.370	1.00	0.00
ATOM	2317	HW1	SOL	398	8.810	35.560	26.360	1.00	0.00
ATOM	2318	HW2	SOL	398	10.100	35.140	25.680	1.00	0.00
ATOM	2319	OW	SOL	399	22.470	10.530	31.130	1.00	0.00
ATOM	2320	HW1	SOL	399	23.030	10.840	30.420	1.00	0.00
ATOM	2321	HW2	SOL	399	22.990	9.860	31.560	1.00	0.00
ATOM	2322	OW	SOL	400	2.950	44.850	46.550	1.00	0.00
ATOM	2323	HW1	SOL	400	3.780	45.310	46.530	1.00	0.00
ATOM	2324	HW2	SOL	400	2.300	45.490	46.250	1.00	0.00
ATOM	2325	OW	SOL	401	23.970	18.820	21.930	1.00	0.00
ATOM	2326	HW1	SOL	401	24.570	19.560	22.050	1.00	0.00
ATOM	2327	HW2	SOL	401	24.530	18.130	21.560	1.00	0.00
ATOM	2328	OW	SOL	402	7.730	5.010	34.620	1.00	0.00
ATOM	2329	HW1	SOL	402	7.200	4.390	34.120	1.00	0.00
ATOM	2330	HW2	SOL	402	8.600	4.600	34.650	1.00	0.00
ATOM	2331	OW	SOL	403	30.240	9.790	2.220	1.00	0.00

ATOM	2332	HW1	SOL	403	30.690	9.390	1.480	1.00	0.00
ATOM	2333	HW2	SOL	403	30.630	10.660	2.310	1.00	0.00
ATOM	2334	OW	SOL	404	40.700	24.400	50.010	1.00	0.00
ATOM	2335	HW1	SOL	404	40.890	25.340	50.030	1.00	0.00
ATOM	2336	HW2	SOL	404	40.990	24.080	50.870	1.00	0.00
ATOM	2337	OW	SOL	405	35.010	12.270	26.680	1.00	0.00
ATOM	2338	HW1	SOL	405	35.230	12.340	25.750	1.00	0.00
ATOM	2339	HW2	SOL	405	35.190	11.360	26.900	1.00	0.00
ATOM	2340	OW	SOL	406	7.560	3.330	18.720	1.00	0.00
ATOM	2341	HW1	SOL	406	7.860	4.030	19.300	1.00	0.00
ATOM	2342	HW2	SOL	406	7.400	2.580	19.300	1.00	0.00
ATOM	2343	OW	SOL	407	51.450	0.390	6.810	1.00	0.00
ATOM	2344	HW1	SOL	407	51.760	1.210	7.210	1.00	0.00
ATOM	2345	HW2	SOL	407	51.140	-0.130	7.540	1.00	0.00
ATOM	2346	OW	SOL	408	8.320	30.450	55.550	1.00	0.00
ATOM	2347	HW1	SOL	408	8.300	29.870	54.780	1.00	0.00
ATOM	2348	HW2	SOL	408	7.700	30.050	56.160	1.00	0.00
ATOM	2349	OW	SOL	409	7.300	6.760	42.790	1.00	0.00
ATOM	2350	HW1	SOL	409	6.810	7.400	42.280	1.00	0.00
ATOM	2351	HW2	SOL	409	8.200	7.080	42.780	1.00	0.00
ATOM	2352	OW	SOL	410	1.540	52.120	41.330	1.00	0.00
ATOM	2353	HW1	SOL	410	1.110	52.300	42.170	1.00	0.00
ATOM	2354	HW2	SOL	410	2.350	52.630	41.350	1.00	0.00
ATOM	2355	OW	SOL	411	29.780	10.110	5.950	1.00	0.00
ATOM	2356	HW1	SOL	411	29.650	10.770	5.270	1.00	0.00
ATOM	2357	HW2	SOL	411	30.250	10.580	6.640	1.00	0.00
ATOM	2358	OW	SOL	412	24.970	37.740	29.120	1.00	0.00
ATOM	2359	HW1	SOL	412	25.720	37.270	28.760	1.00	0.00
ATOM	2360	HW2	SOL	412	25.260	38.650	29.190	1.00	0.00
ATOM	2361	OW	SOL	413	40.590	1.410	41.740	1.00	0.00
ATOM	2362	HW1	SOL	413	40.380	2.340	41.670	1.00	0.00
ATOM	2363	HW2	SOL	413	39.970	1.070	42.380	1.00	0.00
ATOM	2364	OW	SOL	414	11.280	40.290	1.010	1.00	0.00
ATOM	2365	HW1	SOL	414	11.850	39.670	0.550	1.00	0.00
ATOM	2366	HW2	SOL	414	10.950	39.800	1.760	1.00	0.00
ATOM	2367	OW	SOL	415	45.950	45.030	4.100	1.00	0.00
ATOM	2368	HW1	SOL	415	45.120	45.490	3.960	1.00	0.00
ATOM	2369	HW2	SOL	415	45.970	44.360	3.420	1.00	0.00
ATOM	2370	OW	SOL	416	3.560	28.910	38.960	1.00	0.00
ATOM	2371	HW1	SOL	416	3.330	28.040	38.640	1.00	0.00
ATOM	2372	HW2	SOL	416	4.040	28.740	39.770	1.00	0.00
ATOM	2373	OW	SOL	417	53.380	44.930	37.350	1.00	0.00
ATOM	2374	HW1	SOL	417	53.380	44.320	36.610	1.00	0.00
ATOM	2375	HW2	SOL	417	52.750	44.560	37.960	1.00	0.00

ATOM	2376	OW	SOL	418	13.900	5.310	10.410	1.00	0.00
ATOM	2377	HW1	SOL	418	13.180	5.810	10.800	1.00	0.00
ATOM	2378	HW2	SOL	418	14.430	5.960	9.950	1.00	0.00
ATOM	2379	OW	SOL	419	14.280	22.920	1.060	1.00	0.00
ATOM	2380	HW1	SOL	419	14.310	22.040	1.420	1.00	0.00
ATOM	2381	HW2	SOL	419	15.140	23.050	0.660	1.00	0.00
ATOM	2382	OW	SOL	420	6.330	44.960	19.240	1.00	0.00
ATOM	2383	HW1	SOL	420	6.620	45.760	19.680	1.00	0.00
ATOM	2384	HW2	SOL	420	6.560	44.260	19.840	1.00	0.00
ATOM	2385	OW	SOL	421	48.090	25.020	7.220	1.00	0.00
ATOM	2386	HW1	SOL	421	48.050	24.070	7.380	1.00	0.00
ATOM	2387	HW2	SOL	421	48.660	25.110	6.460	1.00	0.00
ATOM	2388	OW	SOL	422	0.710	47.680	4.670	1.00	0.00
ATOM	2389	HW1	SOL	422	0.630	46.750	4.900	1.00	0.00
ATOM	2390	HW2	SOL	422	1.650	47.830	4.610	1.00	0.00
ATOM	2391	OW	SOL	423	22.980	14.200	15.440	1.00	0.00
ATOM	2392	HW1	SOL	423	23.290	13.840	16.270	1.00	0.00
ATOM	2393	HW2	SOL	423	22.270	13.610	15.170	1.00	0.00
ATOM	2394	OW	SOL	424	25.520	19.460	36.760	1.00	0.00
ATOM	2395	HW1	SOL	424	25.650	20.390	36.540	1.00	0.00
ATOM	2396	HW2	SOL	424	25.950	18.990	36.050	1.00	0.00
ATOM	2397	OW	SOL	425	5.480	12.640	7.520	1.00	0.00
ATOM	2398	HW1	SOL	425	4.590	12.400	7.780	1.00	0.00
ATOM	2399	HW2	SOL	425	6.020	12.380	8.260	1.00	0.00
ATOM	2400	OW	SOL	426	15.590	28.370	35.250	1.00	0.00
ATOM	2401	HW1	SOL	426	16.340	28.430	34.650	1.00	0.00
ATOM	2402	HW2	SOL	426	15.660	27.490	35.610	1.00	0.00
ATOM	2403	OW	SOL	427	29.170	24.930	22.150	1.00	0.00
ATOM	2404	HW1	SOL	427	29.690	24.820	21.350	1.00	0.00
ATOM	2405	HW2	SOL	427	28.510	25.590	21.910	1.00	0.00
ATOM	2406	OW	SOL	428	10.800	44.500	12.140	1.00	0.00
ATOM	2407	HW1	SOL	428	11.140	45.380	12.280	1.00	0.00
ATOM	2408	HW2	SOL	428	9.840	44.610	12.160	1.00	0.00
ATOM	2409	OW	SOL	429	6.280	23.310	32.440	1.00	0.00
ATOM	2410	HW1	SOL	429	6.340	23.740	31.590	1.00	0.00
ATOM	2411	HW2	SOL	429	5.680	23.850	32.950	1.00	0.00
ATOM	2412	OW	SOL	430	42.450	11.260	6.430	1.00	0.00
ATOM	2413	HW1	SOL	430	42.390	11.190	5.480	1.00	0.00
ATOM	2414	HW2	SOL	430	42.820	10.420	6.710	1.00	0.00
ATOM	2415	OW	SOL	431	26.530	45.490	31.910	1.00	0.00
ATOM	2416	HW1	SOL	431	27.450	45.370	32.160	1.00	0.00
ATOM	2417	HW2	SOL	431	26.370	44.800	31.260	1.00	0.00
ATOM	2418	OW	SOL	432	42.620	51.460	9.130	1.00	0.00
ATOM	2419	HW1	SOL	432	42.950	51.750	9.980	1.00	0.00

ATOM	2420	HW2	SOL	432	42.780	50.520	9.110	1.00	0.00
ATOM	2421	OW	SOL	433	0.820	38.540	16.790	1.00	0.00
ATOM	2422	HW1	SOL	433	0.610	38.180	17.650	1.00	0.00
ATOM	2423	HW2	SOL	433	0.150	39.220	16.640	1.00	0.00
ATOM	2424	OW	SOL	434	9.940	16.200	36.630	1.00	0.00
ATOM	2425	HW1	SOL	434	10.660	16.640	36.180	1.00	0.00
ATOM	2426	HW2	SOL	434	9.270	16.090	35.950	1.00	0.00
ATOM	2427	OW	SOL	435	35.980	15.090	44.100	1.00	0.00
ATOM	2428	HW1	SOL	435	36.520	15.020	43.300	1.00	0.00
ATOM	2429	HW2	SOL	435	36.620	15.190	44.800	1.00	0.00
ATOM	2430	OW	SOL	436	3.360	12.600	23.570	1.00	0.00
ATOM	2431	HW1	SOL	436	3.910	12.740	24.340	1.00	0.00
ATOM	2432	HW2	SOL	436	3.810	13.070	22.860	1.00	0.00
ATOM	2433	OW	SOL	437	46.970	39.610	25.580	1.00	0.00
ATOM	2434	HW1	SOL	437	46.830	39.020	26.320	1.00	0.00
ATOM	2435	HW2	SOL	437	46.930	39.050	24.810	1.00	0.00
ATOM	2436	OW	SOL	438	15.620	11.750	27.080	1.00	0.00
ATOM	2437	HW1	SOL	438	15.800	11.570	26.150	1.00	0.00
ATOM	2438	HW2	SOL	438	16.140	11.110	27.560	1.00	0.00
ATOM	2439	OW	SOL	439	2.720	7.730	25.460	1.00	0.00
ATOM	2440	HW1	SOL	439	2.090	7.620	26.170	1.00	0.00
ATOM	2441	HW2	SOL	439	3.110	8.580	25.610	1.00	0.00
ATOM	2442	OW	SOL	440	35.330	23.830	43.710	1.00	0.00
ATOM	2443	HW1	SOL	440	35.180	24.300	44.530	1.00	0.00
ATOM	2444	HW2	SOL	440	35.700	24.490	43.120	1.00	0.00
ATOM	2445	OW	SOL	441	0.310	52.960	17.300	1.00	0.00
ATOM	2446	HW1	SOL	441	0.920	52.430	16.790	1.00	0.00
ATOM	2447	HW2	SOL	441	0.330	52.570	18.180	1.00	0.00
ATOM	2448	OW	SOL	442	28.400	26.660	0.740	1.00	0.00
ATOM	2449	HW1	SOL	442	27.850	26.100	1.290	1.00	0.00
ATOM	2450	HW2	SOL	442	29.180	26.130	0.570	1.00	0.00
ATOM	2451	OW	SOL	443	29.200	27.120	40.500	1.00	0.00
ATOM	2452	HW1	SOL	443	29.180	26.570	41.280	1.00	0.00
ATOM	2453	HW2	SOL	443	29.850	27.790	40.700	1.00	0.00
ATOM	2454	OW	SOL	444	9.910	3.600	45.240	1.00	0.00
ATOM	2455	HW1	SOL	444	10.800	3.840	44.980	1.00	0.00
ATOM	2456	HW2	SOL	444	9.360	3.920	44.530	1.00	0.00
ATOM	2457	OW	SOL	445	52.960	8.230	37.910	1.00	0.00
ATOM	2458	HW1	SOL	445	53.250	9.120	37.700	1.00	0.00
ATOM	2459	HW2	SOL	445	52.070	8.180	37.560	1.00	0.00
ATOM	2460	OW	SOL	446	29.370	23.410	45.840	1.00	0.00
ATOM	2461	HW1	SOL	446	30.090	23.360	46.470	1.00	0.00
ATOM	2462	HW2	SOL	446	29.750	23.110	45.020	1.00	0.00
ATOM	2463	OW	SOL	447	29.670	47.940	39.160	1.00	0.00

ATOM	2464	HW1	SOL	447	30.070	47.070	39.080	1.00	0.00
ATOM	2465	HW2	SOL	447	29.660	48.280	38.270	1.00	0.00
ATOM	2466	OW	SOL	448	21.130	47.090	36.490	1.00	0.00
ATOM	2467	HW1	SOL	448	20.200	47.270	36.320	1.00	0.00
ATOM	2468	HW2	SOL	448	21.210	47.150	37.450	1.00	0.00
ATOM	2469	OW	SOL	449	48.530	1.660	6.060	1.00	0.00
ATOM	2470	HW1	SOL	449	49.260	2.180	6.390	1.00	0.00
ATOM	2471	HW2	SOL	449	47.880	2.300	5.780	1.00	0.00
ATOM	2472	OW	SOL	450	53.510	45.800	53.720	1.00	0.00
ATOM	2473	HW1	SOL	450	53.610	45.810	54.670	1.00	0.00
ATOM	2474	HW2	SOL	450	53.750	46.680	53.440	1.00	0.00
ATOM	2475	OW	SOL	451	50.270	28.480	8.760	1.00	0.00
ATOM	2476	HW1	SOL	451	50.860	28.780	9.440	1.00	0.00
ATOM	2477	HW2	SOL	451	50.630	27.630	8.480	1.00	0.00
ATOM	2478	OW	SOL	452	3.320	54.200	19.910	1.00	0.00
ATOM	2479	HW1	SOL	452	2.960	54.880	20.470	1.00	0.00
ATOM	2480	HW2	SOL	452	2.850	54.300	19.080	1.00	0.00
ATOM	2481	OW	SOL	453	47.200	47.530	13.020	1.00	0.00
ATOM	2482	HW1	SOL	453	46.790	48.170	13.600	1.00	0.00
ATOM	2483	HW2	SOL	453	47.750	48.050	12.430	1.00	0.00
ATOM	2484	OW	SOL	454	20.220	12.170	55.620	1.00	0.00
ATOM	2485	HW1	SOL	454	21.050	11.700	55.720	1.00	0.00
ATOM	2486	HW2	SOL	454	19.660	11.570	55.130	1.00	0.00
ATOM	2487	OW	SOL	455	9.520	30.800	38.920	1.00	0.00
ATOM	2488	HW1	SOL	455	10.280	30.770	39.510	1.00	0.00
ATOM	2489	HW2	SOL	455	9.900	30.780	38.040	1.00	0.00
ATOM	2490	OW	SOL	456	20.430	54.570	9.920	1.00	0.00
ATOM	2491	HW1	SOL	456	19.940	55.380	10.010	1.00	0.00
ATOM	2492	HW2	SOL	456	21.130	54.780	9.310	1.00	0.00
ATOM	2493	OW	SOL	457	46.920	29.300	50.990	1.00	0.00
ATOM	2494	HW1	SOL	457	46.970	29.640	50.100	1.00	0.00
ATOM	2495	HW2	SOL	457	47.120	28.370	50.910	1.00	0.00
ATOM	2496	OW	SOL	458	4.440	13.150	46.930	1.00	0.00
ATOM	2497	HW1	SOL	458	4.560	12.210	46.790	1.00	0.00
ATOM	2498	HW2	SOL	458	3.660	13.220	47.480	1.00	0.00
ATOM	2499	OW	SOL	459	4.410	15.780	55.490	1.00	0.00
ATOM	2500	HW1	SOL	459	4.410	15.230	54.710	1.00	0.00
ATOM	2501	HW2	SOL	459	4.380	15.150	56.220	1.00	0.00
ATOM	2502	OW	SOL	460	11.320	46.590	44.590	1.00	0.00
ATOM	2503	HW1	SOL	460	10.450	46.840	44.270	1.00	0.00
ATOM	2504	HW2	SOL	460	11.200	46.470	45.530	1.00	0.00
ATOM	2505	OW	SOL	461	12.340	5.530	32.680	1.00	0.00
ATOM	2506	HW1	SOL	461	13.300	5.560	32.710	1.00	0.00
ATOM	2507	HW2	SOL	461	12.100	4.990	33.430	1.00	0.00

ATOM	2508	OW	SOL	462	47.530	21.920	6.450	1.00	0.00
ATOM	2509	HW1	SOL	462	48.410	21.730	6.780	1.00	0.00
ATOM	2510	HW2	SOL	462	46.980	21.940	7.230	1.00	0.00
ATOM	2511	OW	SOL	463	46.320	49.520	9.790	1.00	0.00
ATOM	2512	HW1	SOL	463	46.370	49.770	8.870	1.00	0.00
ATOM	2513	HW2	SOL	463	45.650	48.830	9.820	1.00	0.00
ATOM	2514	OW	SOL	464	48.240	22.290	3.790	1.00	0.00
ATOM	2515	HW1	SOL	464	48.030	21.990	4.670	1.00	0.00
ATOM	2516	HW2	SOL	464	47.470	22.070	3.270	1.00	0.00
ATOM	2517	OW	SOL	465	14.830	31.160	46.170	1.00	0.00
ATOM	2518	HW1	SOL	465	14.980	30.470	46.810	1.00	0.00
ATOM	2519	HW2	SOL	465	14.950	30.740	45.320	1.00	0.00
ATOM	2520	OW	SOL	466	16.970	14.250	45.890	1.00	0.00
ATOM	2521	HW1	SOL	466	17.710	14.730	45.500	1.00	0.00
ATOM	2522	HW2	SOL	466	17.130	14.280	46.830	1.00	0.00
ATOM	2523	OW	SOL	467	19.760	35.980	37.260	1.00	0.00
ATOM	2524	HW1	SOL	467	19.130	35.300	37.000	1.00	0.00
ATOM	2525	HW2	SOL	467	19.220	36.680	37.630	1.00	0.00
ATOM	2526	OW	SOL	468	43.940	44.010	44.940	1.00	0.00
ATOM	2527	HW1	SOL	468	44.470	43.220	45.070	1.00	0.00
ATOM	2528	HW2	SOL	468	43.040	43.680	44.930	1.00	0.00
ATOM	2529	OW	SOL	469	9.060	29.090	8.060	1.00	0.00
ATOM	2530	HW1	SOL	469	8.320	28.530	8.290	1.00	0.00
ATOM	2531	HW2	SOL	469	9.700	28.500	7.670	1.00	0.00
ATOM	2532	OW	SOL	470	8.300	7.550	10.090	1.00	0.00
ATOM	2533	HW1	SOL	470	8.980	6.910	10.310	1.00	0.00
ATOM	2534	HW2	SOL	470	7.610	7.380	10.730	1.00	0.00
ATOM	2535	OW	SOL	471	14.440	14.390	54.010	1.00	0.00
ATOM	2536	HW1	SOL	471	14.040	14.070	54.820	1.00	0.00
ATOM	2537	HW2	SOL	471	14.500	13.620	53.450	1.00	0.00
ATOM	2538	OW	SOL	472	38.340	35.760	54.130	1.00	0.00
ATOM	2539	HW1	SOL	472	38.160	36.340	53.390	1.00	0.00
ATOM	2540	HW2	SOL	472	37.490	35.640	54.560	1.00	0.00
ATOM	2541	OW	SOL	473	19.030	17.790	29.520	1.00	0.00
ATOM	2542	HW1	SOL	473	19.710	17.810	28.840	1.00	0.00
ATOM	2543	HW2	SOL	473	18.760	16.870	29.560	1.00	0.00
ATOM	2544	OW	SOL	474	17.270	52.180	27.070	1.00	0.00
ATOM	2545	HW1	SOL	474	17.820	52.610	26.420	1.00	0.00
ATOM	2546	HW2	SOL	474	16.810	51.500	26.580	1.00	0.00
ATOM	2547	OW	SOL	475	11.730	21.800	19.880	1.00	0.00
ATOM	2548	HW1	SOL	475	11.640	21.660	18.930	1.00	0.00
ATOM	2549	HW2	SOL	475	10.840	21.950	20.180	1.00	0.00
ATOM	2550	OW	SOL	476	23.130	18.310	35.540	1.00	0.00
ATOM	2551	HW1	SOL	476	23.870	18.900	35.630	1.00	0.00

ATOM	2552	HW2	SOL	476	22.510	18.780	34.980	1.00	0.00
ATOM	2553	OW	SOL	477	2.110	42.090	7.960	1.00	0.00
ATOM	2554	HW1	SOL	477	2.560	42.820	8.380	1.00	0.00
ATOM	2555	HW2	SOL	477	2.000	41.450	8.660	1.00	0.00
ATOM	2556	OW	SOL	478	4.700	6.090	45.440	1.00	0.00
ATOM	2557	HW1	SOL	478	4.700	5.300	45.980	1.00	0.00
ATOM	2558	HW2	SOL	478	5.390	6.630	45.800	1.00	0.00
ATOM	2559	OW	SOL	479	15.880	47.330	0.300	1.00	0.00
ATOM	2560	HW1	SOL	479	16.630	47.770	0.720	1.00	0.00
ATOM	2561	HW2	SOL	479	16.070	47.400	-0.640	1.00	0.00
ATOM	2562	OW	SOL	480	22.570	2.550	14.520	1.00	0.00
ATOM	2563	HW1	SOL	480	23.520	2.480	14.500	1.00	0.00
ATOM	2564	HW2	SOL	480	22.390	3.420	14.140	1.00	0.00
ATOM	2565	OW	SOL	481	36.010	44.490	16.460	1.00	0.00
ATOM	2566	HW1	SOL	481	36.120	44.810	15.560	1.00	0.00
ATOM	2567	HW2	SOL	481	36.480	45.120	17.000	1.00	0.00
ATOM	2568	OW	SOL	482	29.360	30.170	8.630	1.00	0.00
ATOM	2569	HW1	SOL	482	29.040	31.070	8.530	1.00	0.00
ATOM	2570	HW2	SOL	482	28.910	29.850	9.410	1.00	0.00
ATOM	2571	OW	SOL	483	24.000	49.200	54.210	1.00	0.00
ATOM	2572	HW1	SOL	483	23.790	49.830	53.520	1.00	0.00
ATOM	2573	HW2	SOL	483	24.930	49.350	54.390	1.00	0.00
ATOM	2574	OW	SOL	484	34.900	25.410	10.840	1.00	0.00
ATOM	2575	HW1	SOL	484	35.470	24.720	10.510	1.00	0.00
ATOM	2576	HW2	SOL	484	35.440	25.860	11.500	1.00	0.00
ATOM	2577	OW	SOL	485	35.500	39.780	37.480	1.00	0.00
ATOM	2578	HW1	SOL	485	34.710	39.290	37.250	1.00	0.00
ATOM	2579	HW2	SOL	485	35.580	40.450	36.790	1.00	0.00
ATOM	2580	OW	SOL	486	16.040	21.310	51.080	1.00	0.00
ATOM	2581	HW1	SOL	486	16.330	22.200	51.270	1.00	0.00
ATOM	2582	HW2	SOL	486	16.740	20.760	51.430	1.00	0.00
ATOM	2583	OW	SOL	487	1.310	30.760	32.800	1.00	0.00
ATOM	2584	HW1	SOL	487	0.480	31.160	33.060	1.00	0.00
ATOM	2585	HW2	SOL	487	1.590	30.260	33.560	1.00	0.00
ATOM	2586	OW	SOL	488	25.050	29.790	50.210	1.00	0.00
ATOM	2587	HW1	SOL	488	25.250	28.860	50.160	1.00	0.00
ATOM	2588	HW2	SOL	488	24.110	29.850	50.060	1.00	0.00
ATOM	2589	OW	SOL	489	46.220	48.400	34.920	1.00	0.00
ATOM	2590	HW1	SOL	489	46.870	47.720	35.090	1.00	0.00
ATOM	2591	HW2	SOL	489	46.590	49.180	35.340	1.00	0.00
ATOM	2592	OW	SOL	490	36.370	9.570	41.160	1.00	0.00
ATOM	2593	HW1	SOL	490	35.580	9.190	40.780	1.00	0.00
ATOM	2594	HW2	SOL	490	36.600	10.290	40.580	1.00	0.00
ATOM	2595	OW	SOL	491	55.560	49.590	16.120	1.00	0.00

ATOM	2596	HW1	SOL	491	55.380	50.520	15.940	1.00	0.00
ATOM	2597	HW2	SOL	491	55.330	49.480	17.040	1.00	0.00
ATOM	2598	OW	SOL	492	47.250	10.810	31.070	1.00	0.00
ATOM	2599	HW1	SOL	492	47.610	10.210	31.720	1.00	0.00
ATOM	2600	HW2	SOL	492	46.650	11.370	31.560	1.00	0.00
ATOM	2601	OW	SOL	493	11.490	45.830	26.690	1.00	0.00
ATOM	2602	HW1	SOL	493	12.270	45.950	27.250	1.00	0.00
ATOM	2603	HW2	SOL	493	11.010	46.650	26.790	1.00	0.00
ATOM	2604	OW	SOL	494	2.380	5.910	9.320	1.00	0.00
ATOM	2605	HW1	SOL	494	2.200	5.030	8.990	1.00	0.00
ATOM	2606	HW2	SOL	494	2.100	5.890	10.230	1.00	0.00
ATOM	2607	OW	SOL	495	53.540	43.210	31.880	1.00	0.00
ATOM	2608	HW1	SOL	495	53.610	42.740	32.710	1.00	0.00
ATOM	2609	HW2	SOL	495	54.320	42.950	31.390	1.00	0.00
ATOM	2610	OW	SOL	496	26.620	22.140	2.710	1.00	0.00
ATOM	2611	HW1	SOL	496	27.570	22.000	2.640	1.00	0.00
ATOM	2612	HW2	SOL	496	26.240	21.340	2.350	1.00	0.00
ATOM	2613	OW	SOL	497	14.710	13.200	34.190	1.00	0.00
ATOM	2614	HW1	SOL	497	13.820	12.910	34.410	1.00	0.00
ATOM	2615	HW2	SOL	497	14.690	13.310	33.230	1.00	0.00
ATOM	2616	OW	SOL	498	4.120	7.920	21.550	1.00	0.00
ATOM	2617	HW1	SOL	498	4.040	7.250	22.230	1.00	0.00
ATOM	2618	HW2	SOL	498	3.830	8.730	21.970	1.00	0.00
ATOM	2619	OW	SOL	499	38.850	25.620	5.210	1.00	0.00
ATOM	2620	HW1	SOL	499	39.650	25.580	4.690	1.00	0.00
ATOM	2621	HW2	SOL	499	38.370	24.820	4.990	1.00	0.00
ATOM	2622	OW	SOL	500	41.110	19.180	55.030	1.00	0.00
ATOM	2623	HW1	SOL	500	41.010	18.410	54.460	1.00	0.00
ATOM	2624	HW2	SOL	500	41.620	18.860	55.780	1.00	0.00
ATOM	2625	OW	SOL	501	1.720	5.510	3.900	1.00	0.00
ATOM	2626	HW1	SOL	501	2.410	5.140	4.460	1.00	0.00
ATOM	2627	HW2	SOL	501	2.080	6.350	3.620	1.00	0.00
ATOM	2628	OW	SOL	502	52.080	39.240	51.690	1.00	0.00
ATOM	2629	HW1	SOL	502	52.620	39.920	51.280	1.00	0.00
ATOM	2630	HW2	SOL	502	52.360	38.430	51.270	1.00	0.00
ATOM	2631	OW	SOL	503	17.120	51.190	12.140	1.00	0.00
ATOM	2632	HW1	SOL	503	17.670	51.920	11.850	1.00	0.00
ATOM	2633	HW2	SOL	503	17.360	50.460	11.570	1.00	0.00
ATOM	2634	OW	SOL	504	18.510	2.130	17.260	1.00	0.00
ATOM	2635	HW1	SOL	504	18.590	1.790	18.150	1.00	0.00
ATOM	2636	HW2	SOL	504	19.410	2.100	16.910	1.00	0.00
ATOM	2637	OW	SOL	505	32.130	30.420	42.930	1.00	0.00
ATOM	2638	HW1	SOL	505	31.540	31.180	42.940	1.00	0.00
ATOM	2639	HW2	SOL	505	32.720	30.560	43.660	1.00	0.00

ATOM	2640	OW	SOL	506	23.990	14.690	53.770	1.00	0.00
ATOM	2641	HW1	SOL	506	24.560	15.430	54.010	1.00	0.00
ATOM	2642	HW2	SOL	506	23.330	14.670	54.460	1.00	0.00
ATOM	2643	OW	SOL	507	1.920	9.960	10.830	1.00	0.00
ATOM	2644	HW1	SOL	507	1.120	9.980	10.320	1.00	0.00
ATOM	2645	HW2	SOL	507	2.190	10.880	10.890	1.00	0.00
ATOM	2646	OW	SOL	508	22.360	22.570	49.900	1.00	0.00
ATOM	2647	HW1	SOL	508	21.630	22.430	50.500	1.00	0.00
ATOM	2648	HW2	SOL	508	22.390	23.520	49.770	1.00	0.00
ATOM	2649	OW	SOL	509	9.950	8.000	47.650	1.00	0.00
ATOM	2650	HW1	SOL	509	9.990	7.690	48.550	1.00	0.00
ATOM	2651	HW2	SOL	509	9.640	7.240	47.150	1.00	0.00
ATOM	2652	OW	SOL	510	24.470	6.850	41.670	1.00	0.00
ATOM	2653	HW1	SOL	510	25.190	7.450	41.870	1.00	0.00
ATOM	2654	HW2	SOL	510	23.860	6.970	42.390	1.00	0.00
ATOM	2655	OW	SOL	511	48.230	21.780	15.590	1.00	0.00
ATOM	2656	HW1	SOL	511	48.750	22.580	15.640	1.00	0.00
ATOM	2657	HW2	SOL	511	47.380	22.070	15.240	1.00	0.00
ATOM	2658	OW	SOL	512	31.900	18.780	22.700	1.00	0.00
ATOM	2659	HW1	SOL	512	32.480	19.060	23.400	1.00	0.00
ATOM	2660	HW2	SOL	512	31.020	18.930	23.040	1.00	0.00
ATOM	2661	OW	SOL	513	17.170	19.690	8.870	1.00	0.00
ATOM	2662	HW1	SOL	513	16.240	19.860	9.040	1.00	0.00
ATOM	2663	HW2	SOL	513	17.600	20.530	9.020	1.00	0.00
ATOM	2664	OW	SOL	514	17.910	55.170	27.550	1.00	0.00
ATOM	2665	HW1	SOL	514	17.770	55.060	28.490	1.00	0.00
ATOM	2666	HW2	SOL	514	17.380	55.940	27.320	1.00	0.00
ATOM	2667	OW	SOL	515	21.410	5.230	40.000	1.00	0.00
ATOM	2668	HW1	SOL	515	22.230	5.100	39.520	1.00	0.00
ATOM	2669	HW2	SOL	515	21.650	5.090	40.920	1.00	0.00
ATOM	2670	OW	SOL	516	54.380	47.040	47.900	1.00	0.00
ATOM	2671	HW1	SOL	516	54.450	47.720	48.570	1.00	0.00
ATOM	2672	HW2	SOL	516	55.220	47.090	47.440	1.00	0.00
ATOM	2673	OW	SOL	517	50.830	43.080	17.010	1.00	0.00
ATOM	2674	HW1	SOL	517	50.330	42.270	16.980	1.00	0.00
ATOM	2675	HW2	SOL	517	51.040	43.270	16.100	1.00	0.00
ATOM	2676	OW	SOL	518	20.520	20.000	17.100	1.00	0.00
ATOM	2677	HW1	SOL	518	20.250	20.030	16.180	1.00	0.00
ATOM	2678	HW2	SOL	518	21.460	19.790	17.070	1.00	0.00
ATOM	2679	OW	SOL	519	38.850	53.300	34.850	1.00	0.00
ATOM	2680	HW1	SOL	519	39.410	53.910	34.360	1.00	0.00
ATOM	2681	HW2	SOL	519	38.080	53.170	34.280	1.00	0.00
ATOM	2682	OW	SOL	520	26.860	10.400	13.210	1.00	0.00
ATOM	2683	HW1	SOL	520	26.970	11.250	13.630	1.00	0.00

ATOM	2684	HW2	SOL	520	27.410	9.810	13.710	1.00	0.00
ATOM	2685	OW	SOL	521	46.100	36.550	48.780	1.00	0.00
ATOM	2686	HW1	SOL	521	45.900	36.600	49.710	1.00	0.00
ATOM	2687	HW2	SOL	521	45.410	37.060	48.360	1.00	0.00
ATOM	2688	OW	SOL	522	40.640	9.290	12.940	1.00	0.00
ATOM	2689	HW1	SOL	522	40.560	10.220	13.140	1.00	0.00
ATOM	2690	HW2	SOL	522	41.430	9.230	12.400	1.00	0.00
ATOM	2691	OW	SOL	523	13.760	20.200	1.770	1.00	0.00
ATOM	2692	HW1	SOL	523	12.920	19.890	1.420	1.00	0.00
ATOM	2693	HW2	SOL	523	14.410	19.640	1.350	1.00	0.00
ATOM	2694	OW	SOL	524	13.700	32.850	41.110	1.00	0.00
ATOM	2695	HW1	SOL	524	13.600	33.780	40.890	1.00	0.00
ATOM	2696	HW2	SOL	524	13.660	32.400	40.260	1.00	0.00
ATOM	2697	OW	SOL	525	7.620	8.730	50.270	1.00	0.00
ATOM	2698	HW1	SOL	525	8.500	8.350	50.260	1.00	0.00
ATOM	2699	HW2	SOL	525	7.710	9.570	49.820	1.00	0.00
ATOM	2700	OW	SOL	526	45.470	4.850	14.030	1.00	0.00
ATOM	2701	HW1	SOL	526	46.110	4.870	13.320	1.00	0.00
ATOM	2702	HW2	SOL	526	45.980	5.010	14.820	1.00	0.00
ATOM	2703	OW	SOL	527	3.150	21.400	20.840	1.00	0.00
ATOM	2704	HW1	SOL	527	2.750	21.540	19.980	1.00	0.00
ATOM	2705	HW2	SOL	527	2.450	21.040	21.380	1.00	0.00
ATOM	2706	OW	SOL	528	23.460	29.580	7.750	1.00	0.00
ATOM	2707	HW1	SOL	528	22.810	29.540	8.450	1.00	0.00
ATOM	2708	HW2	SOL	528	24.280	29.310	8.160	1.00	0.00
ATOM	2709	OW	SOL	529	38.260	48.720	22.770	1.00	0.00
ATOM	2710	HW1	SOL	529	37.620	49.270	23.220	1.00	0.00
ATOM	2711	HW2	SOL	529	38.590	48.130	23.450	1.00	0.00
ATOM	2712	OW	SOL	530	54.160	46.300	6.040	1.00	0.00
ATOM	2713	HW1	SOL	530	54.790	45.580	6.020	1.00	0.00
ATOM	2714	HW2	SOL	530	54.640	47.040	5.640	1.00	0.00
ATOM	2715	OW	SOL	531	44.280	20.800	18.450	1.00	0.00
ATOM	2716	HW1	SOL	531	43.380	20.980	18.170	1.00	0.00
ATOM	2717	HW2	SOL	531	44.210	20.650	19.390	1.00	0.00
ATOM	2718	OW	SOL	532	12.900	53.770	34.920	1.00	0.00
ATOM	2719	HW1	SOL	532	13.400	52.980	35.150	1.00	0.00
ATOM	2720	HW2	SOL	532	13.200	53.990	34.040	1.00	0.00
ATOM	2721	OW	SOL	533	39.430	10.280	54.430	1.00	0.00
ATOM	2722	HW1	SOL	533	39.340	11.160	54.060	1.00	0.00
ATOM	2723	HW2	SOL	533	38.790	9.750	53.950	1.00	0.00
ATOM	2724	OW	SOL	534	22.790	53.670	7.160	1.00	0.00
ATOM	2725	HW1	SOL	534	23.720	53.750	6.930	1.00	0.00
ATOM	2726	HW2	SOL	534	22.570	52.760	6.950	1.00	0.00
ATOM	2727	OW	SOL	535	51.230	21.420	47.810	1.00	0.00

ATOM	2728	HW1	SOL	535	51.230	21.040	48.690	1.00	0.00
ATOM	2729	HW2	SOL	535	50.920	22.320	47.930	1.00	0.00
ATOM	2730	OW	SOL	536	3.690	3.820	34.110	1.00	0.00
ATOM	2731	HW1	SOL	536	4.470	3.310	34.360	1.00	0.00
ATOM	2732	HW2	SOL	536	3.080	3.170	33.770	1.00	0.00
ATOM	2733	OW	SOL	537	22.480	13.380	37.950	1.00	0.00
ATOM	2734	HW1	SOL	537	21.940	12.870	37.350	1.00	0.00
ATOM	2735	HW2	SOL	537	22.720	14.160	37.460	1.00	0.00
ATOM	2736	OW	SOL	538	53.010	18.780	16.610	1.00	0.00
ATOM	2737	HW1	SOL	538	52.600	19.470	17.130	1.00	0.00
ATOM	2738	HW2	SOL	538	53.120	18.050	17.230	1.00	0.00
ATOM	2739	OW	SOL	539	29.030	54.320	24.360	1.00	0.00
ATOM	2740	HW1	SOL	539	29.570	53.620	23.980	1.00	0.00
ATOM	2741	HW2	SOL	539	29.640	55.030	24.530	1.00	0.00
ATOM	2742	OW	SOL	540	34.770	48.690	29.820	1.00	0.00
ATOM	2743	HW1	SOL	540	34.110	48.010	29.960	1.00	0.00
ATOM	2744	HW2	SOL	540	34.280	49.440	29.470	1.00	0.00
ATOM	2745	OW	SOL	541	26.820	47.910	11.580	1.00	0.00
ATOM	2746	HW1	SOL	541	26.170	47.670	10.930	1.00	0.00
ATOM	2747	HW2	SOL	541	27.650	47.570	11.240	1.00	0.00
ATOM	2748	OW	SOL	542	26.630	25.660	52.380	1.00	0.00
ATOM	2749	HW1	SOL	542	27.210	25.270	51.730	1.00	0.00
ATOM	2750	HW2	SOL	542	26.770	25.150	53.170	1.00	0.00
ATOM	2751	OW	SOL	543	55.350	40.330	43.830	1.00	0.00
ATOM	2752	HW1	SOL	543	54.760	39.720	43.390	1.00	0.00
ATOM	2753	HW2	SOL	543	54.820	40.710	44.530	1.00	0.00
ATOM	2754	OW	SOL	544	52.510	50.380	20.710	1.00	0.00
ATOM	2755	HW1	SOL	544	51.590	50.140	20.630	1.00	0.00
ATOM	2756	HW2	SOL	544	52.490	51.320	20.900	1.00	0.00
ATOM	2757	OW	SOL	545	40.510	49.490	11.030	1.00	0.00
ATOM	2758	HW1	SOL	545	41.020	48.710	10.840	1.00	0.00
ATOM	2759	HW2	SOL	545	40.810	49.780	11.890	1.00	0.00
ATOM	2760	OW	SOL	546	51.880	30.080	17.800	1.00	0.00
ATOM	2761	HW1	SOL	546	51.500	30.710	18.420	1.00	0.00
ATOM	2762	HW2	SOL	546	51.120	29.630	17.430	1.00	0.00
ATOM	2763	OW	SOL	547	40.780	0.940	35.320	1.00	0.00
ATOM	2764	HW1	SOL	547	40.490	0.340	34.620	1.00	0.00
ATOM	2765	HW2	SOL	547	41.460	0.460	35.780	1.00	0.00
ATOM	2766	OW	SOL	548	54.820	17.160	14.130	1.00	0.00
ATOM	2767	HW1	SOL	548	55.730	16.990	14.390	1.00	0.00
ATOM	2768	HW2	SOL	548	54.890	17.470	13.220	1.00	0.00
ATOM	2769	OW	SOL	549	52.560	46.910	45.720	1.00	0.00
ATOM	2770	HW1	SOL	549	53.180	46.510	45.100	1.00	0.00
ATOM	2771	HW2	SOL	549	53.020	46.880	46.560	1.00	0.00

ATOM	2772	OW	SOL	550	4.220	19.740	17.350	1.00	0.00
ATOM	2773	HW1	SOL	550	3.680	19.620	18.130	1.00	0.00
ATOM	2774	HW2	SOL	550	3.590	19.930	16.660	1.00	0.00
ATOM	2775	OW	SOL	551	45.520	41.260	21.960	1.00	0.00
ATOM	2776	HW1	SOL	551	46.270	41.850	22.060	1.00	0.00
ATOM	2777	HW2	SOL	551	45.110	41.260	22.830	1.00	0.00
ATOM	2778	OW	SOL	552	39.390	0.520	46.400	1.00	0.00
ATOM	2779	HW1	SOL	552	39.250	0.140	47.270	1.00	0.00
ATOM	2780	HW2	SOL	552	39.270	-0.210	45.800	1.00	0.00
ATOM	2781	OW	SOL	553	45.570	50.870	0.330	1.00	0.00
ATOM	2782	HW1	SOL	553	45.060	50.460	1.020	1.00	0.00
ATOM	2783	HW2	SOL	553	45.760	50.160	-0.280	1.00	0.00
ATOM	2784	OW	SOL	554	3.670	38.510	24.600	1.00	0.00
ATOM	2785	HW1	SOL	554	4.620	38.350	24.580	1.00	0.00
ATOM	2786	HW2	SOL	554	3.590	39.450	24.500	1.00	0.00
ATOM	2787	OW	SOL	555	32.530	10.390	8.490	1.00	0.00
ATOM	2788	HW1	SOL	555	33.160	10.010	9.100	1.00	0.00
ATOM	2789	HW2	SOL	555	31.770	9.810	8.530	1.00	0.00
ATOM	2790	OW	SOL	556	0.020	18.000	19.560	1.00	0.00
ATOM	2791	HW1	SOL	556	-0.610	17.810	20.250	1.00	0.00
ATOM	2792	HW2	SOL	556	-0.200	18.880	19.260	1.00	0.00
ATOM	2793	OW	SOL	557	27.930	27.360	27.240	1.00	0.00
ATOM	2794	HW1	SOL	557	27.490	26.890	26.540	1.00	0.00
ATOM	2795	HW2	SOL	557	27.210	27.780	27.730	1.00	0.00
ATOM	2796	OW	SOL	558	33.140	31.460	22.200	1.00	0.00
ATOM	2797	HW1	SOL	558	32.980	30.540	22.420	1.00	0.00
ATOM	2798	HW2	SOL	558	33.610	31.430	21.370	1.00	0.00
ATOM	2799	OW	SOL	559	3.410	18.210	4.970	1.00	0.00
ATOM	2800	HW1	SOL	559	3.170	19.130	5.020	1.00	0.00
ATOM	2801	HW2	SOL	559	2.590	17.740	4.840	1.00	0.00
ATOM	2802	OW	SOL	560	44.570	18.850	30.670	1.00	0.00
ATOM	2803	HW1	SOL	560	43.990	19.590	30.520	1.00	0.00
ATOM	2804	HW2	SOL	560	45.440	19.190	30.450	1.00	0.00
ATOM	2805	OW	SOL	561	36.510	33.640	25.560	1.00	0.00
ATOM	2806	HW1	SOL	561	36.790	33.070	26.270	1.00	0.00
ATOM	2807	HW2	SOL	561	36.450	33.060	24.800	1.00	0.00
ATOM	2808	OW	SOL	562	44.260	34.700	0.620	1.00	0.00
ATOM	2809	HW1	SOL	562	43.590	34.440	1.250	1.00	0.00
ATOM	2810	HW2	SOL	562	44.830	35.300	1.100	1.00	0.00
ATOM	2811	OW	SOL	563	53.140	41.440	49.660	1.00	0.00
ATOM	2812	HW1	SOL	563	53.510	41.930	50.400	1.00	0.00
ATOM	2813	HW2	SOL	563	52.190	41.570	49.740	1.00	0.00
ATOM	2814	OW	SOL	564	50.440	9.390	25.250	1.00	0.00
ATOM	2815	HW1	SOL	564	50.880	10.220	25.460	1.00	0.00

ATOM	2816	HW2	SOL	564	49.660	9.650	24.760	1.00	0.00
ATOM	2817	OW	SOL	565	36.720	51.560	27.070	1.00	0.00
ATOM	2818	HW1	SOL	565	36.080	50.920	26.770	1.00	0.00
ATOM	2819	HW2	SOL	565	36.380	51.870	27.910	1.00	0.00
ATOM	2820	OW	SOL	566	27.290	20.750	31.700	1.00	0.00
ATOM	2821	HW1	SOL	566	28.200	20.990	31.520	1.00	0.00
ATOM	2822	HW2	SOL	566	26.930	21.510	32.160	1.00	0.00
ATOM	2823	OW	SOL	567	49.040	19.390	42.060	1.00	0.00
ATOM	2824	HW1	SOL	567	48.600	20.190	41.780	1.00	0.00
ATOM	2825	HW2	SOL	567	48.600	19.150	42.880	1.00	0.00
ATOM	2826	OW	SOL	568	6.290	31.630	9.480	1.00	0.00
ATOM	2827	HW1	SOL	568	6.170	32.580	9.490	1.00	0.00
ATOM	2828	HW2	SOL	568	7.220	31.500	9.630	1.00	0.00
ATOM	2829	OW	SOL	569	28.340	17.340	12.290	1.00	0.00
ATOM	2830	HW1	SOL	569	28.190	17.540	13.210	1.00	0.00
ATOM	2831	HW2	SOL	569	27.770	16.590	12.110	1.00	0.00
ATOM	2832	OW	SOL	570	49.400	11.980	8.400	1.00	0.00
ATOM	2833	HW1	SOL	570	49.700	12.720	7.860	1.00	0.00
ATOM	2834	HW2	SOL	570	49.130	11.320	7.760	1.00	0.00
ATOM	2835	OW	SOL	571	33.500	40.660	35.260	1.00	0.00
ATOM	2836	HW1	SOL	571	32.580	40.730	35.010	1.00	0.00
ATOM	2837	HW2	SOL	571	33.510	39.970	35.930	1.00	0.00
ATOM	2838	OW	SOL	572	51.990	0.340	35.270	1.00	0.00
ATOM	2839	HW1	SOL	572	51.420	1.100	35.390	1.00	0.00
ATOM	2840	HW2	SOL	572	51.630	-0.320	35.870	1.00	0.00
ATOM	2841	OW	SOL	573	46.930	1.160	33.790	1.00	0.00
ATOM	2842	HW1	SOL	573	46.020	1.290	34.040	1.00	0.00
ATOM	2843	HW2	SOL	573	47.090	0.230	33.950	1.00	0.00
ATOM	2844	OW	SOL	574	13.240	24.050	9.400	1.00	0.00
ATOM	2845	HW1	SOL	574	13.100	23.630	8.550	1.00	0.00
ATOM	2846	HW2	SOL	574	14.190	23.970	9.550	1.00	0.00
ATOM	2847	OW	SOL	575	43.340	16.440	17.250	1.00	0.00
ATOM	2848	HW1	SOL	575	42.910	16.740	18.050	1.00	0.00
ATOM	2849	HW2	SOL	575	42.630	16.330	16.610	1.00	0.00
ATOM	2850	OW	SOL	576	19.090	44.450	18.250	1.00	0.00
ATOM	2851	HW1	SOL	576	18.490	44.810	18.910	1.00	0.00
ATOM	2852	HW2	SOL	576	18.610	44.520	17.430	1.00	0.00
ATOM	2853	OW	SOL	577	35.410	25.720	27.380	1.00	0.00
ATOM	2854	HW1	SOL	577	34.870	25.060	26.960	1.00	0.00
ATOM	2855	HW2	SOL	577	34.800	26.270	27.850	1.00	0.00
ATOM	2856	OW	SOL	578	35.560	29.360	22.980	1.00	0.00
ATOM	2857	HW1	SOL	578	36.380	28.860	22.970	1.00	0.00
ATOM	2858	HW2	SOL	578	35.300	29.380	23.900	1.00	0.00
ATOM	2859	OW	SOL	579	21.460	38.030	36.080	1.00	0.00

ATOM	2860	HW1	SOL	579	21.730	37.530	35.310	1.00	0.00
ATOM	2861	HW2	SOL	579	21.020	37.390	36.640	1.00	0.00
ATOM	2862	OW	SOL	580	17.950	53.280	51.370	1.00	0.00
ATOM	2863	HW1	SOL	580	17.170	53.220	50.820	1.00	0.00
ATOM	2864	HW2	SOL	580	18.110	54.220	51.450	1.00	0.00
ATOM	2865	OW	SOL	581	43.530	40.220	51.680	1.00	0.00
ATOM	2866	HW1	SOL	581	43.380	40.710	50.880	1.00	0.00
ATOM	2867	HW2	SOL	581	44.310	39.690	51.490	1.00	0.00
ATOM	2868	OW	SOL	582	1.050	52.250	5.040	1.00	0.00
ATOM	2869	HW1	SOL	582	0.450	52.920	5.360	1.00	0.00
ATOM	2870	HW2	SOL	582	1.880	52.720	4.900	1.00	0.00
ATOM	2871	OW	SOL	583	38.240	18.030	9.980	1.00	0.00
ATOM	2872	HW1	SOL	583	38.370	18.730	9.340	1.00	0.00
ATOM	2873	HW2	SOL	583	37.340	18.170	10.290	1.00	0.00
ATOM	2874	OW	SOL	584	39.550	3.640	47.750	1.00	0.00
ATOM	2875	HW1	SOL	584	39.540	3.610	48.710	1.00	0.00
ATOM	2876	HW2	SOL	584	39.540	2.720	47.490	1.00	0.00
ATOM	2877	OW	SOL	585	6.320	51.170	37.800	1.00	0.00
ATOM	2878	HW1	SOL	585	6.960	50.640	38.280	1.00	0.00
ATOM	2879	HW2	SOL	585	5.530	51.150	38.340	1.00	0.00
ATOM	2880	OW	SOL	586	29.000	16.070	8.400	1.00	0.00
ATOM	2881	HW1	SOL	586	29.320	16.170	7.500	1.00	0.00
ATOM	2882	HW2	SOL	586	29.590	16.610	8.920	1.00	0.00
ATOM	2883	OW	SOL	587	22.430	51.740	31.220	1.00	0.00
ATOM	2884	HW1	SOL	587	21.570	51.320	31.190	1.00	0.00
ATOM	2885	HW2	SOL	587	22.730	51.620	32.120	1.00	0.00
ATOM	2886	OW	SOL	588	0.820	36.660	18.980	1.00	0.00
ATOM	2887	HW1	SOL	588	0.830	35.750	18.690	1.00	0.00
ATOM	2888	HW2	SOL	588	1.500	36.710	19.650	1.00	0.00
ATOM	2889	OW	SOL	589	20.730	53.720	55.430	1.00	0.00
ATOM	2890	HW1	SOL	589	21.200	53.920	56.240	1.00	0.00
ATOM	2891	HW2	SOL	589	20.200	52.960	55.650	1.00	0.00
ATOM	2892	OW	SOL	590	44.330	46.400	19.240	1.00	0.00
ATOM	2893	HW1	SOL	590	43.930	45.560	19.490	1.00	0.00
ATOM	2894	HW2	SOL	590	44.860	46.640	20.000	1.00	0.00
ATOM	2895	OW	SOL	591	4.340	8.530	43.130	1.00	0.00
ATOM	2896	HW1	SOL	591	3.930	7.860	42.590	1.00	0.00
ATOM	2897	HW2	SOL	591	4.670	8.060	43.890	1.00	0.00
ATOM	2898	OW	SOL	592	55.610	40.840	6.740	1.00	0.00
ATOM	2899	HW1	SOL	592	55.050	41.120	6.020	1.00	0.00
ATOM	2900	HW2	SOL	592	56.080	41.630	7.000	1.00	0.00
ATOM	2901	OW	SOL	593	3.350	31.820	31.470	1.00	0.00
ATOM	2902	HW1	SOL	593	2.910	32.670	31.440	1.00	0.00
ATOM	2903	HW2	SOL	593	2.690	31.220	31.830	1.00	0.00

ATOM	2904	OW	SOL	594	36.800	17.240	5.870	1.00	0.00
ATOM	2905	HW1	SOL	594	37.510	17.140	6.500	1.00	0.00
ATOM	2906	HW2	SOL	594	36.570	18.170	5.910	1.00	0.00
ATOM	2907	OW	SOL	595	24.560	55.350	16.360	1.00	0.00
ATOM	2908	HW1	SOL	595	25.120	54.580	16.260	1.00	0.00
ATOM	2909	HW2	SOL	595	24.780	55.910	15.610	1.00	0.00
ATOM	2910	OW	SOL	596	41.240	42.090	8.270	1.00	0.00
ATOM	2911	HW1	SOL	596	41.250	42.440	7.380	1.00	0.00
ATOM	2912	HW2	SOL	596	41.230	41.140	8.160	1.00	0.00
ATOM	2913	OW	SOL	597	37.700	23.140	32.030	1.00	0.00
ATOM	2914	HW1	SOL	597	37.330	23.990	31.780	1.00	0.00
ATOM	2915	HW2	SOL	597	37.160	22.500	31.560	1.00	0.00
ATOM	2916	OW	SOL	598	35.140	48.900	12.680	1.00	0.00
ATOM	2917	HW1	SOL	598	34.350	49.230	12.250	1.00	0.00
ATOM	2918	HW2	SOL	598	35.280	48.030	12.320	1.00	0.00
ATOM	2919	OW	SOL	599	43.370	32.710	24.170	1.00	0.00
ATOM	2920	HW1	SOL	599	43.550	33.330	23.460	1.00	0.00
ATOM	2921	HW2	SOL	599	44.230	32.330	24.370	1.00	0.00
ATOM	2922	OW	SOL	600	47.010	6.430	11.750	1.00	0.00
ATOM	2923	HW1	SOL	600	47.340	7.100	12.360	1.00	0.00
ATOM	2924	HW2	SOL	600	47.730	5.800	11.670	1.00	0.00
ATOM	2925	OW	SOL	601	24.050	51.080	33.310	1.00	0.00
ATOM	2926	HW1	SOL	601	24.970	50.850	33.200	1.00	0.00
ATOM	2927	HW2	SOL	601	23.680	50.390	33.850	1.00	0.00
ATOM	2928	OW	SOL	602	22.300	10.680	25.890	1.00	0.00
ATOM	2929	HW1	SOL	602	22.810	11.480	25.740	1.00	0.00
ATOM	2930	HW2	SOL	602	21.460	10.990	26.220	1.00	0.00
ATOM	2931	OW	SOL	603	3.400	41.010	26.300	1.00	0.00
ATOM	2932	HW1	SOL	603	3.360	41.920	26.000	1.00	0.00
ATOM	2933	HW2	SOL	603	2.550	40.640	26.050	1.00	0.00
ATOM	2934	OW	SOL	604	5.910	28.200	36.820	1.00	0.00
ATOM	2935	HW1	SOL	604	5.840	28.880	37.480	1.00	0.00
ATOM	2936	HW2	SOL	604	6.260	28.650	36.050	1.00	0.00
ATOM	2937	OW	SOL	605	2.070	32.920	42.900	1.00	0.00
ATOM	2938	HW1	SOL	605	2.910	32.840	43.350	1.00	0.00
ATOM	2939	HW2	SOL	605	2.000	32.110	42.380	1.00	0.00
ATOM	2940	OW	SOL	606	45.900	12.610	32.740	1.00	0.00
ATOM	2941	HW1	SOL	606	45.210	12.150	33.210	1.00	0.00
ATOM	2942	HW2	SOL	606	45.650	13.530	32.780	1.00	0.00
ATOM	2943	OW	SOL	607	10.470	26.340	41.470	1.00	0.00
ATOM	2944	HW1	SOL	607	10.940	26.770	40.760	1.00	0.00
ATOM	2945	HW2	SOL	607	11.160	25.890	41.970	1.00	0.00
ATOM	2946	OW	SOL	608	36.780	4.880	55.850	1.00	0.00
ATOM	2947	HW1	SOL	608	35.960	5.370	55.890	1.00	0.00

ATOM	2948	HW2	SOL	608	37.360	5.420	55.320	1.00	0.00
ATOM	2949	OW	SOL	609	3.610	11.270	53.890	1.00	0.00
ATOM	2950	HW1	SOL	609	2.850	11.280	54.460	1.00	0.00
ATOM	2951	HW2	SOL	609	3.340	10.760	53.130	1.00	0.00
ATOM	2952	OW	SOL	610	10.740	35.990	4.200	1.00	0.00
ATOM	2953	HW1	SOL	610	10.550	36.560	4.940	1.00	0.00
ATOM	2954	HW2	SOL	610	11.170	35.220	4.580	1.00	0.00
ATOM	2955	OW	SOL	611	16.600	5.520	55.850	1.00	0.00
ATOM	2956	HW1	SOL	611	16.710	5.430	54.910	1.00	0.00
ATOM	2957	HW2	SOL	611	17.460	5.330	56.220	1.00	0.00
ATOM	2958	OW	SOL	612	54.880	20.590	19.130	1.00	0.00
ATOM	2959	HW1	SOL	612	53.990	20.800	18.860	1.00	0.00
ATOM	2960	HW2	SOL	612	55.150	21.320	19.680	1.00	0.00
ATOM	2961	OW	SOL	613	34.600	23.340	21.110	1.00	0.00
ATOM	2962	HW1	SOL	613	33.910	22.730	20.860	1.00	0.00
ATOM	2963	HW2	SOL	613	34.970	22.970	21.920	1.00	0.00
ATOM	2964	OW	SOL	614	54.740	18.460	48.190	1.00	0.00
ATOM	2965	HW1	SOL	614	55.610	18.670	48.530	1.00	0.00
ATOM	2966	HW2	SOL	614	54.890	18.290	47.250	1.00	0.00
ATOM	2967	OW	SOL	615	13.580	48.310	25.690	1.00	0.00
ATOM	2968	HW1	SOL	615	13.930	47.530	25.260	1.00	0.00
ATOM	2969	HW2	SOL	615	13.340	48.010	26.570	1.00	0.00
ATOM	2970	OW	SOL	616	8.470	10.760	33.310	1.00	0.00
ATOM	2971	HW1	SOL	616	9.330	10.450	33.580	1.00	0.00
ATOM	2972	HW2	SOL	616	7.930	10.720	34.100	1.00	0.00
ATOM	2973	OW	SOL	617	46.570	15.980	48.780	1.00	0.00
ATOM	2974	HW1	SOL	617	46.450	15.950	49.730	1.00	0.00
ATOM	2975	HW2	SOL	617	45.810	16.460	48.460	1.00	0.00
ATOM	2976	OW	SOL	618	37.260	38.740	39.320	1.00	0.00
ATOM	2977	HW1	SOL	618	36.630	38.800	40.040	1.00	0.00
ATOM	2978	HW2	SOL	618	36.780	39.050	38.550	1.00	0.00
ATOM	2979	OW	SOL	619	8.630	9.260	20.000	1.00	0.00
ATOM	2980	HW1	SOL	619	8.680	9.660	19.140	1.00	0.00
ATOM	2981	HW2	SOL	619	9.380	9.630	20.480	1.00	0.00
ATOM	2982	OW	SOL	620	7.340	13.400	40.730	1.00	0.00
ATOM	2983	HW1	SOL	620	8.270	13.590	40.890	1.00	0.00
ATOM	2984	HW2	SOL	620	7.350	12.680	40.100	1.00	0.00
ATOM	2985	OW	SOL	621	54.990	48.000	8.440	1.00	0.00
ATOM	2986	HW1	SOL	621	54.770	48.610	7.730	1.00	0.00
ATOM	2987	HW2	SOL	621	54.330	48.180	9.110	1.00	0.00
ATOM	2988	OW	SOL	622	0.270	12.180	29.570	1.00	0.00
ATOM	2989	HW1	SOL	622	0.960	12.370	28.930	1.00	0.00
ATOM	2990	HW2	SOL	622	-0.480	11.900	29.040	1.00	0.00
ATOM	2991	OW	SOL	623	2.220	20.570	37.080	1.00	0.00

ATOM	2992	HW1	SOL	623	2.640	19.710	36.950	1.00	0.00
ATOM	2993	HW2	SOL	623	2.770	21.180	36.590	1.00	0.00
ATOM	2994	OW	SOL	624	15.150	47.080	31.060	1.00	0.00
ATOM	2995	HW1	SOL	624	15.900	47.650	30.880	1.00	0.00
ATOM	2996	HW2	SOL	624	14.390	47.620	30.850	1.00	0.00
ATOM	2997	OW	SOL	625	0.790	35.660	54.060	1.00	0.00
ATOM	2998	HW1	SOL	625	1.490	36.220	53.720	1.00	0.00
ATOM	2999	HW2	SOL	625	0.090	35.730	53.410	1.00	0.00
ATOM	3000	OW	SOL	626	50.240	21.410	24.540	1.00	0.00
ATOM	3001	HW1	SOL	626	50.550	21.430	23.630	1.00	0.00
ATOM	3002	HW2	SOL	626	49.820	20.560	24.630	1.00	0.00
ATOM	3003	OW	SOL	627	31.830	43.970	27.150	1.00	0.00
ATOM	3004	HW1	SOL	627	31.100	43.370	27.030	1.00	0.00
ATOM	3005	HW2	SOL	627	32.060	43.890	28.070	1.00	0.00
ATOM	3006	OW	SOL	628	51.500	45.170	13.130	1.00	0.00
ATOM	3007	HW1	SOL	628	51.700	45.870	12.500	1.00	0.00
ATOM	3008	HW2	SOL	628	50.580	44.970	12.970	1.00	0.00
ATOM	3009	OW	SOL	629	29.110	31.580	26.410	1.00	0.00
ATOM	3010	HW1	SOL	629	30.060	31.450	26.450	1.00	0.00
ATOM	3011	HW2	SOL	629	29.000	32.470	26.090	1.00	0.00
ATOM	3012	OW	SOL	630	42.100	24.510	52.700	1.00	0.00
ATOM	3013	HW1	SOL	630	41.450	24.250	53.360	1.00	0.00
ATOM	3014	HW2	SOL	630	42.470	25.330	53.050	1.00	0.00
ATOM	3015	OW	SOL	631	8.520	5.060	28.960	1.00	0.00
ATOM	3016	HW1	SOL	631	8.870	4.430	29.600	1.00	0.00
ATOM	3017	HW2	SOL	631	7.710	5.380	29.360	1.00	0.00
ATOM	3018	OW	SOL	632	53.870	38.910	41.970	1.00	0.00
ATOM	3019	HW1	SOL	632	53.020	38.810	41.540	1.00	0.00
ATOM	3020	HW2	SOL	632	54.440	38.290	41.500	1.00	0.00
ATOM	3021	OW	SOL	633	28.810	45.270	47.740	1.00	0.00
ATOM	3022	HW1	SOL	633	28.220	44.570	48.010	1.00	0.00
ATOM	3023	HW2	SOL	633	29.690	44.880	47.820	1.00	0.00
ATOM	3024	OW	SOL	634	54.160	8.930	25.230	1.00	0.00
ATOM	3025	HW1	SOL	634	54.330	8.360	24.480	1.00	0.00
ATOM	3026	HW2	SOL	634	54.880	8.760	25.840	1.00	0.00
ATOM	3027	OW	SOL	635	24.020	3.750	22.890	1.00	0.00
ATOM	3028	HW1	SOL	635	23.900	3.500	21.980	1.00	0.00
ATOM	3029	HW2	SOL	635	23.160	4.070	23.170	1.00	0.00
ATOM	3030	OW	SOL	636	47.890	30.810	16.690	1.00	0.00
ATOM	3031	HW1	SOL	636	47.390	31.330	17.310	1.00	0.00
ATOM	3032	HW2	SOL	636	48.370	31.460	16.170	1.00	0.00
ATOM	3033	OW	SOL	637	49.020	24.730	46.060	1.00	0.00
ATOM	3034	HW1	SOL	637	49.710	24.750	46.730	1.00	0.00
ATOM	3035	HW2	SOL	637	48.300	24.240	46.470	1.00	0.00

ATOM	3036	OW	SOL	638	20.080	28.610	21.540	1.00	0.00
ATOM	3037	HW1	SOL	638	20.440	29.400	21.950	1.00	0.00
ATOM	3038	HW2	SOL	638	20.840	28.180	21.130	1.00	0.00
ATOM	3039	OW	SOL	639	50.130	25.560	11.840	1.00	0.00
ATOM	3040	HW1	SOL	639	49.450	26.130	11.490	1.00	0.00
ATOM	3041	HW2	SOL	639	50.160	25.760	12.770	1.00	0.00
ATOM	3042	OW	SOL	640	17.280	11.380	48.810	1.00	0.00
ATOM	3043	HW1	SOL	640	16.470	11.140	49.260	1.00	0.00
ATOM	3044	HW2	SOL	640	17.070	12.200	48.360	1.00	0.00
ATOM	3045	OW	SOL	641	51.400	22.120	39.330	1.00	0.00
ATOM	3046	HW1	SOL	641	51.520	21.700	40.180	1.00	0.00
ATOM	3047	HW2	SOL	641	50.500	22.460	39.370	1.00	0.00
ATOM	3048	OW	SOL	642	24.480	27.220	3.330	1.00	0.00
ATOM	3049	HW1	SOL	642	23.710	27.690	3.630	1.00	0.00
ATOM	3050	HW2	SOL	642	24.150	26.360	3.080	1.00	0.00
ATOM	3051	OW	SOL	643	10.720	38.300	9.030	1.00	0.00
ATOM	3052	HW1	SOL	643	11.630	38.060	8.890	1.00	0.00
ATOM	3053	HW2	SOL	643	10.750	39.150	9.470	1.00	0.00
ATOM	3054	OW	SOL	644	30.430	27.150	25.750	1.00	0.00
ATOM	3055	HW1	SOL	644	29.540	27.290	26.070	1.00	0.00
ATOM	3056	HW2	SOL	644	30.530	26.190	25.740	1.00	0.00
ATOM	3057	OW	SOL	645	50.710	24.620	48.250	1.00	0.00
ATOM	3058	HW1	SOL	645	50.320	24.820	49.100	1.00	0.00
ATOM	3059	HW2	SOL	645	51.120	25.440	47.970	1.00	0.00
ATOM	3060	OW	SOL	646	0.580	16.580	36.830	1.00	0.00
ATOM	3061	HW1	SOL	646	1.080	16.480	36.020	1.00	0.00
ATOM	3062	HW2	SOL	646	1.070	16.050	37.470	1.00	0.00
ATOM	3063	OW	SOL	647	34.930	13.010	24.060	1.00	0.00
ATOM	3064	HW1	SOL	647	34.050	12.650	24.160	1.00	0.00
ATOM	3065	HW2	SOL	647	35.100	12.980	23.120	1.00	0.00
ATOM	3066	OW	SOL	648	38.630	7.730	15.420	1.00	0.00
ATOM	3067	HW1	SOL	648	38.740	7.600	14.480	1.00	0.00
ATOM	3068	HW2	SOL	648	39.470	8.070	15.730	1.00	0.00
ATOM	3069	OW	SOL	649	9.980	49.810	38.110	1.00	0.00
ATOM	3070	HW1	SOL	649	9.960	50.450	38.820	1.00	0.00
ATOM	3071	HW2	SOL	649	10.480	49.080	38.460	1.00	0.00
ATOM	3072	OW	SOL	650	10.040	0.560	21.830	1.00	0.00
ATOM	3073	HW1	SOL	650	9.140	0.330	22.070	1.00	0.00
ATOM	3074	HW2	SOL	650	10.550	-0.220	22.030	1.00	0.00
ATOM	3075	OW	SOL	651	36.250	17.690	2.040	1.00	0.00
ATOM	3076	HW1	SOL	651	35.550	17.120	1.720	1.00	0.00
ATOM	3077	HW2	SOL	651	36.740	17.940	1.250	1.00	0.00
ATOM	3078	OW	SOL	652	55.030	29.280	27.560	1.00	0.00
ATOM	3079	HW1	SOL	652	55.790	29.860	27.640	1.00	0.00

ATOM	3080	HW2	SOL	652	54.500	29.480	28.320	1.00	0.00
ATOM	3081	OW	SOL	653	35.380	12.240	48.000	1.00	0.00
ATOM	3082	HW1	SOL	653	34.470	12.080	47.740	1.00	0.00
ATOM	3083	HW2	SOL	653	35.840	12.400	47.170	1.00	0.00
ATOM	3084	OW	SOL	654	27.660	43.140	49.240	1.00	0.00
ATOM	3085	HW1	SOL	654	27.170	42.590	48.630	1.00	0.00
ATOM	3086	HW2	SOL	654	26.980	43.620	49.730	1.00	0.00
ATOM	3087	OW	SOL	655	14.840	18.450	12.550	1.00	0.00
ATOM	3088	HW1	SOL	655	14.570	17.950	11.780	1.00	0.00
ATOM	3089	HW2	SOL	655	14.070	18.950	12.800	1.00	0.00
ATOM	3090	OW	SOL	656	27.340	8.880	47.570	1.00	0.00
ATOM	3091	HW1	SOL	656	27.310	9.380	48.390	1.00	0.00
ATOM	3092	HW2	SOL	656	27.790	9.450	46.950	1.00	0.00
ATOM	3093	OW	SOL	657	39.420	3.920	31.900	1.00	0.00
ATOM	3094	HW1	SOL	657	39.910	3.760	32.700	1.00	0.00
ATOM	3095	HW2	SOL	657	40.080	3.850	31.200	1.00	0.00
ATOM	3096	OW	SOL	658	48.460	7.810	41.140	1.00	0.00
ATOM	3097	HW1	SOL	658	49.410	7.640	41.170	1.00	0.00
ATOM	3098	HW2	SOL	658	48.070	6.970	41.380	1.00	0.00
ATOM	3099	OW	SOL	659	51.330	34.040	50.500	1.00	0.00
ATOM	3100	HW1	SOL	659	50.420	34.170	50.750	1.00	0.00
ATOM	3101	HW2	SOL	659	51.300	33.890	49.550	1.00	0.00
ATOM	3102	OW	SOL	660	28.350	42.470	0.910	1.00	0.00
ATOM	3103	HW1	SOL	660	27.410	42.450	1.100	1.00	0.00
ATOM	3104	HW2	SOL	660	28.490	43.330	0.510	1.00	0.00
ATOM	3105	OW	SOL	661	28.690	4.510	37.880	1.00	0.00
ATOM	3106	HW1	SOL	661	28.330	5.290	37.450	1.00	0.00
ATOM	3107	HW2	SOL	661	29.460	4.280	37.360	1.00	0.00
ATOM	3108	OW	SOL	662	47.610	42.690	14.560	1.00	0.00
ATOM	3109	HW1	SOL	662	47.030	43.050	15.240	1.00	0.00
ATOM	3110	HW2	SOL	662	48.440	42.540	15.020	1.00	0.00
ATOM	3111	OW	SOL	663	25.400	0.280	55.730	1.00	0.00
ATOM	3112	HW1	SOL	663	24.600	0.800	55.800	1.00	0.00
ATOM	3113	HW2	SOL	663	25.230	-0.500	56.260	1.00	0.00
ATOM	3114	OW	SOL	664	53.770	49.290	1.340	1.00	0.00
ATOM	3115	HW1	SOL	664	52.850	49.460	1.540	1.00	0.00
ATOM	3116	HW2	SOL	664	53.740	48.620	0.650	1.00	0.00
ATOM	3117	OW	SOL	665	23.420	2.000	1.590	1.00	0.00
ATOM	3118	HW1	SOL	665	24.210	2.540	1.540	1.00	0.00
ATOM	3119	HW2	SOL	665	23.470	1.580	2.440	1.00	0.00
ATOM	3120	OW	SOL	666	55.120	2.000	26.570	1.00	0.00
ATOM	3121	HW1	SOL	666	55.160	2.290	27.490	1.00	0.00
ATOM	3122	HW2	SOL	666	55.670	2.630	26.100	1.00	0.00
ATOM	3123	OW	SOL	667	17.560	5.110	29.080	1.00	0.00

ATOM	3124	HW1	SOL	667	16.650	4.810	29.110	1.00	0.00
ATOM	3125	HW2	SOL	667	17.970	4.530	28.430	1.00	0.00
ATOM	3126	OW	SOL	668	54.020	18.770	55.830	1.00	0.00
ATOM	3127	HW1	SOL	668	53.250	18.210	55.740	1.00	0.00
ATOM	3128	HW2	SOL	668	53.820	19.330	56.580	1.00	0.00
ATOM	3129	OW	SOL	669	13.670	36.320	23.960	1.00	0.00
ATOM	3130	HW1	SOL	669	13.940	35.650	23.340	1.00	0.00
ATOM	3131	HW2	SOL	669	14.480	36.740	24.240	1.00	0.00
ATOM	3132	OW	SOL	670	11.430	43.950	50.380	1.00	0.00
ATOM	3133	HW1	SOL	670	11.850	43.890	49.520	1.00	0.00
ATOM	3134	HW2	SOL	670	11.620	43.100	50.790	1.00	0.00
ATOM	3135	OW	SOL	671	35.670	20.600	37.880	1.00	0.00
ATOM	3136	HW1	SOL	671	35.910	19.800	38.340	1.00	0.00
ATOM	3137	HW2	SOL	671	34.720	20.660	37.980	1.00	0.00
ATOM	3138	OW	SOL	672	21.920	30.340	18.140	1.00	0.00
ATOM	3139	HW1	SOL	672	21.950	31.300	18.150	1.00	0.00
ATOM	3140	HW2	SOL	672	21.060	30.130	17.780	1.00	0.00
ATOM	3141	OW	SOL	673	7.090	23.710	48.810	1.00	0.00
ATOM	3142	HW1	SOL	673	7.250	24.590	49.160	1.00	0.00
ATOM	3143	HW2	SOL	673	6.260	23.450	49.200	1.00	0.00
ATOM	3144	OW	SOL	674	32.430	27.340	1.590	1.00	0.00
ATOM	3145	HW1	SOL	674	32.820	27.480	2.450	1.00	0.00
ATOM	3146	HW2	SOL	674	32.000	28.170	1.380	1.00	0.00
ATOM	3147	OW	SOL	675	54.050	50.650	39.290	1.00	0.00
ATOM	3148	HW1	SOL	675	53.650	50.830	40.140	1.00	0.00
ATOM	3149	HW2	SOL	675	53.450	50.020	38.870	1.00	0.00
ATOM	3150	OW	SOL	676	9.930	6.930	27.720	1.00	0.00
ATOM	3151	HW1	SOL	676	9.570	6.130	28.110	1.00	0.00
ATOM	3152	HW2	SOL	676	9.640	7.630	28.310	1.00	0.00
ATOM	3153	OW	SOL	677	35.730	47.390	45.150	1.00	0.00
ATOM	3154	HW1	SOL	677	35.580	46.680	45.770	1.00	0.00
ATOM	3155	HW2	SOL	677	35.840	48.170	45.700	1.00	0.00
ATOM	3156	OW	SOL	678	1.390	37.770	3.550	1.00	0.00
ATOM	3157	HW1	SOL	678	1.210	38.030	2.650	1.00	0.00
ATOM	3158	HW2	SOL	678	1.580	36.830	3.500	1.00	0.00
ATOM	3159	OW	SOL	679	0.400	47.600	50.400	1.00	0.00
ATOM	3160	HW1	SOL	679	0.300	48.540	50.280	1.00	0.00
ATOM	3161	HW2	SOL	679	1.210	47.510	50.900	1.00	0.00
ATOM	3162	OW	SOL	680	36.210	35.240	0.160	1.00	0.00
ATOM	3163	HW1	SOL	680	35.830	35.520	0.990	1.00	0.00
ATOM	3164	HW2	SOL	680	35.460	34.910	-0.350	1.00	0.00
ATOM	3165	OW	SOL	681	22.350	12.850	40.580	1.00	0.00
ATOM	3166	HW1	SOL	681	22.420	13.120	39.660	1.00	0.00
ATOM	3167	HW2	SOL	681	21.450	12.520	40.670	1.00	0.00

ATOM	3168	OW	SOL	682	6.170	38.700	16.740	1.00	0.00
ATOM	3169	HW1	SOL	682	5.500	38.320	17.300	1.00	0.00
ATOM	3170	HW2	SOL	682	6.990	38.330	17.050	1.00	0.00
ATOM	3171	OW	SOL	683	34.830	38.880	10.470	1.00	0.00
ATOM	3172	HW1	SOL	683	35.300	38.050	10.410	1.00	0.00
ATOM	3173	HW2	SOL	683	34.230	38.750	11.210	1.00	0.00
ATOM	3174	OW	SOL	684	27.310	34.940	15.760	1.00	0.00
ATOM	3175	HW1	SOL	684	26.730	35.560	16.200	1.00	0.00
ATOM	3176	HW2	SOL	684	26.730	34.220	15.510	1.00	0.00
ATOM	3177	OW	SOL	685	29.600	47.270	27.680	1.00	0.00
ATOM	3178	HW1	SOL	685	30.270	46.940	27.070	1.00	0.00
ATOM	3179	HW2	SOL	685	28.840	47.420	27.130	1.00	0.00
ATOM	3180	OW	SOL	686	22.460	4.620	43.040	1.00	0.00
ATOM	3181	HW1	SOL	686	22.920	5.150	43.700	1.00	0.00
ATOM	3182	HW2	SOL	686	23.070	3.920	42.820	1.00	0.00
ATOM	3183	OW	SOL	687	11.450	37.040	13.230	1.00	0.00
ATOM	3184	HW1	SOL	687	10.640	37.480	13.450	1.00	0.00
ATOM	3185	HW2	SOL	687	11.190	36.300	12.680	1.00	0.00
ATOM	3186	OW	SOL	688	35.950	11.980	19.270	1.00	0.00
ATOM	3187	HW1	SOL	688	36.220	11.630	20.120	1.00	0.00
ATOM	3188	HW2	SOL	688	36.770	12.130	18.800	1.00	0.00
ATOM	3189	OW	SOL	689	55.030	23.290	31.560	1.00	0.00
ATOM	3190	HW1	SOL	689	55.360	22.430	31.820	1.00	0.00
ATOM	3191	HW2	SOL	689	54.240	23.420	32.080	1.00	0.00
ATOM	3192	OW	SOL	690	4.480	13.340	26.430	1.00	0.00
ATOM	3193	HW1	SOL	690	4.270	14.110	26.950	1.00	0.00
ATOM	3194	HW2	SOL	690	5.060	12.820	27.000	1.00	0.00
ATOM	3195	OW	SOL	691	50.560	31.610	8.500	1.00	0.00
ATOM	3196	HW1	SOL	691	50.340	30.810	8.970	1.00	0.00
ATOM	3197	HW2	SOL	691	51.490	31.750	8.670	1.00	0.00
ATOM	3198	OW	SOL	692	17.990	46.930	21.890	1.00	0.00
ATOM	3199	HW1	SOL	692	17.460	47.090	22.670	1.00	0.00
ATOM	3200	HW2	SOL	692	18.700	47.570	21.950	1.00	0.00
ATOM	3201	OW	SOL	693	29.560	41.760	3.280	1.00	0.00
ATOM	3202	HW1	SOL	693	30.420	42.160	3.110	1.00	0.00
ATOM	3203	HW2	SOL	693	29.090	41.860	2.460	1.00	0.00
ATOM	3204	OW	SOL	694	32.570	1.360	17.910	1.00	0.00
ATOM	3205	HW1	SOL	694	33.240	1.360	17.220	1.00	0.00
ATOM	3206	HW2	SOL	694	33.070	1.300	18.720	1.00	0.00
ATOM	3207	OW	SOL	695	25.960	34.600	1.600	1.00	0.00
ATOM	3208	HW1	SOL	695	26.270	33.730	1.360	1.00	0.00
ATOM	3209	HW2	SOL	695	26.620	35.200	1.240	1.00	0.00
ATOM	3210	OW	SOL	696	25.250	50.930	22.870	1.00	0.00
ATOM	3211	HW1	SOL	696	25.990	51.370	22.450	1.00	0.00

ATOM	3212	HW2	SOL	696	25.430	51.010	23.800	1.00	0.00
ATOM	3213	OW	SOL	697	19.710	4.280	6.480	1.00	0.00
ATOM	3214	HW1	SOL	697	19.370	3.480	6.080	1.00	0.00
ATOM	3215	HW2	SOL	697	20.470	3.990	6.990	1.00	0.00
ATOM	3216	OW	SOL	698	32.770	51.300	16.930	1.00	0.00
ATOM	3217	HW1	SOL	698	33.290	50.880	16.250	1.00	0.00
ATOM	3218	HW2	SOL	698	32.500	50.580	17.500	1.00	0.00
ATOM	3219	OW	SOL	699	1.230	28.100	31.040	1.00	0.00
ATOM	3220	HW1	SOL	699	1.090	28.090	31.990	1.00	0.00
ATOM	3221	HW2	SOL	699	2.180	28.120	30.940	1.00	0.00
ATOM	3222	OW	SOL	700	18.540	9.180	10.420	1.00	0.00
ATOM	3223	HW1	SOL	700	18.000	9.390	11.180	1.00	0.00
ATOM	3224	HW2	SOL	700	19.440	9.230	10.750	1.00	0.00
ATOM	3225	OW	SOL	701	1.970	14.230	5.820	1.00	0.00
ATOM	3226	HW1	SOL	701	1.710	15.140	5.680	1.00	0.00
ATOM	3227	HW2	SOL	701	2.790	14.140	5.330	1.00	0.00
ATOM	3228	OW	SOL	702	7.310	0.830	19.700	1.00	0.00
ATOM	3229	HW1	SOL	702	6.580	0.610	19.130	1.00	0.00
ATOM	3230	HW2	SOL	702	7.360	0.110	20.330	1.00	0.00
ATOM	3231	OW	SOL	703	51.540	43.830	25.900	1.00	0.00
ATOM	3232	HW1	SOL	703	50.860	43.510	26.500	1.00	0.00
ATOM	3233	HW2	SOL	703	52.030	43.050	25.660	1.00	0.00
ATOM	3234	OW	SOL	704	17.640	25.410	28.510	1.00	0.00
ATOM	3235	HW1	SOL	704	17.790	24.520	28.190	1.00	0.00
ATOM	3236	HW2	SOL	704	16.830	25.680	28.080	1.00	0.00
ATOM	3237	OW	SOL	705	19.370	37.850	31.320	1.00	0.00
ATOM	3238	HW1	SOL	705	19.220	37.820	30.380	1.00	0.00
ATOM	3239	HW2	SOL	705	19.880	37.060	31.510	1.00	0.00
ATOM	3240	OW	SOL	706	27.520	9.030	10.690	1.00	0.00
ATOM	3241	HW1	SOL	706	27.480	9.800	10.110	1.00	0.00
ATOM	3242	HW2	SOL	706	27.630	9.400	11.570	1.00	0.00
ATOM	3243	OW	SOL	707	21.890	32.980	21.250	1.00	0.00
ATOM	3244	HW1	SOL	707	22.650	33.490	20.980	1.00	0.00
ATOM	3245	HW2	SOL	707	21.160	33.320	20.720	1.00	0.00
ATOM	3246	OW	SOL	708	26.700	15.310	6.920	1.00	0.00
ATOM	3247	HW1	SOL	708	27.340	14.620	7.100	1.00	0.00
ATOM	3248	HW2	SOL	708	26.810	15.930	7.640	1.00	0.00
ATOM	3249	OW	SOL	709	23.410	37.360	24.190	1.00	0.00
ATOM	3250	HW1	SOL	709	23.820	37.710	24.980	1.00	0.00
ATOM	3251	HW2	SOL	709	22.970	38.110	23.800	1.00	0.00
ATOM	3252	OW	SOL	710	1.660	23.460	41.110	1.00	0.00
ATOM	3253	HW1	SOL	710	1.170	24.210	40.780	1.00	0.00
ATOM	3254	HW2	SOL	710	1.090	22.710	40.940	1.00	0.00
ATOM	3255	OW	SOL	711	30.600	11.050	38.280	1.00	0.00

ATOM	3256	HW1	SOL	711	31.530	11.190	38.450	1.00	0.00
ATOM	3257	HW2	SOL	711	30.150	11.730	38.790	1.00	0.00
ATOM	3258	OW	SOL	712	0.400	32.380	54.260	1.00	0.00
ATOM	3259	HW1	SOL	712	-0.080	33.150	54.560	1.00	0.00
ATOM	3260	HW2	SOL	712	1.150	32.330	54.860	1.00	0.00
ATOM	3261	OW	SOL	713	33.460	25.260	19.100	1.00	0.00
ATOM	3262	HW1	SOL	713	33.420	26.190	19.350	1.00	0.00
ATOM	3263	HW2	SOL	713	33.920	24.840	19.820	1.00	0.00
ATOM	3264	OW	SOL	714	53.840	35.940	5.440	1.00	0.00
ATOM	3265	HW1	SOL	714	53.290	35.660	6.180	1.00	0.00
ATOM	3266	HW2	SOL	714	54.740	35.810	5.750	1.00	0.00
ATOM	3267	OW	SOL	715	23.490	53.400	49.140	1.00	0.00
ATOM	3268	HW1	SOL	715	22.680	52.900	49.170	1.00	0.00
ATOM	3269	HW2	SOL	715	23.900	53.250	49.990	1.00	0.00
ATOM	3270	OW	SOL	716	18.230	3.540	45.270	1.00	0.00
ATOM	3271	HW1	SOL	716	18.660	3.920	44.510	1.00	0.00
ATOM	3272	HW2	SOL	716	17.530	4.170	45.480	1.00	0.00
ATOM	3273	OW	SOL	717	19.660	48.000	31.390	1.00	0.00
ATOM	3274	HW1	SOL	717	19.400	47.120	31.100	1.00	0.00
ATOM	3275	HW2	SOL	717	20.470	47.860	31.880	1.00	0.00
ATOM	3276	OW	SOL	718	34.570	11.870	12.140	1.00	0.00
ATOM	3277	HW1	SOL	718	34.360	11.480	12.990	1.00	0.00
ATOM	3278	HW2	SOL	718	33.970	11.450	11.530	1.00	0.00
ATOM	3279	OW	SOL	719	43.530	55.780	52.300	1.00	0.00
ATOM	3280	HW1	SOL	719	43.310	55.810	53.230	1.00	0.00
ATOM	3281	HW2	SOL	719	44.120	56.520	52.170	1.00	0.00
ATOM	3282	OW	SOL	720	4.320	13.760	15.910	1.00	0.00
ATOM	3283	HW1	SOL	720	5.250	13.680	16.130	1.00	0.00
ATOM	3284	HW2	SOL	720	3.900	13.000	16.310	1.00	0.00
ATOM	3285	OW	SOL	721	9.170	11.220	10.220	1.00	0.00
ATOM	3286	HW1	SOL	721	9.320	10.510	9.590	1.00	0.00
ATOM	3287	HW2	SOL	721	10.040	11.600	10.370	1.00	0.00
ATOM	3288	OW	SOL	722	5.250	12.980	37.310	1.00	0.00
ATOM	3289	HW1	SOL	722	5.730	13.690	37.720	1.00	0.00
ATOM	3290	HW2	SOL	722	4.930	12.450	38.040	1.00	0.00
ATOM	3291	OW	SOL	723	8.450	49.630	16.470	1.00	0.00
ATOM	3292	HW1	SOL	723	7.630	50.100	16.580	1.00	0.00
ATOM	3293	HW2	SOL	723	9.080	50.110	17.020	1.00	0.00
ATOM	3294	OW	SOL	724	49.260	53.120	10.750	1.00	0.00
ATOM	3295	HW1	SOL	724	49.150	53.580	9.920	1.00	0.00
ATOM	3296	HW2	SOL	724	48.580	52.460	10.750	1.00	0.00
ATOM	3297	OW	SOL	725	12.240	10.160	18.520	1.00	0.00
ATOM	3298	HW1	SOL	725	12.140	9.240	18.280	1.00	0.00
ATOM	3299	HW2	SOL	725	13.190	10.290	18.570	1.00	0.00

ATOM	3300	OW	SOL	726	12.690	24.020	41.390	1.00	0.00
ATOM	3301	HW1	SOL	726	13.440	23.420	41.440	1.00	0.00
ATOM	3302	HW2	SOL	726	13.060	24.820	41.020	1.00	0.00
ATOM	3303	OW	SOL	727	34.090	14.700	37.270	1.00	0.00
ATOM	3304	HW1	SOL	727	33.840	15.510	36.830	1.00	0.00
ATOM	3305	HW2	SOL	727	33.270	14.380	37.660	1.00	0.00
ATOM	3306	OW	SOL	728	13.780	42.330	54.860	1.00	0.00
ATOM	3307	HW1	SOL	728	13.810	42.490	53.920	1.00	0.00
ATOM	3308	HW2	SOL	728	13.690	43.200	55.250	1.00	0.00
ATOM	3309	OW	SOL	729	27.480	54.370	19.770	1.00	0.00
ATOM	3310	HW1	SOL	729	27.290	55.190	19.320	1.00	0.00
ATOM	3311	HW2	SOL	729	28.430	54.350	19.840	1.00	0.00
ATOM	3312	OW	SOL	730	33.400	54.740	43.610	1.00	0.00
ATOM	3313	HW1	SOL	730	33.080	55.640	43.530	1.00	0.00
ATOM	3314	HW2	SOL	730	33.570	54.470	42.710	1.00	0.00
ATOM	3315	OW	SOL	731	15.070	17.840	48.530	1.00	0.00
ATOM	3316	HW1	SOL	731	15.540	17.160	49.000	1.00	0.00
ATOM	3317	HW2	SOL	731	14.940	18.540	49.180	1.00	0.00
ATOM	3318	OW	SOL	732	0.960	19.910	6.490	1.00	0.00
ATOM	3319	HW1	SOL	732	1.340	20.160	5.640	1.00	0.00
ATOM	3320	HW2	SOL	732	0.950	20.720	6.990	1.00	0.00
ATOM	3321	OW	SOL	733	32.720	45.680	50.520	1.00	0.00
ATOM	3322	HW1	SOL	733	33.530	46.180	50.590	1.00	0.00
ATOM	3323	HW2	SOL	733	32.040	46.340	50.380	1.00	0.00
ATOM	3324	OW	SOL	734	42.980	7.610	30.060	1.00	0.00
ATOM	3325	HW1	SOL	734	43.740	8.090	29.730	1.00	0.00
ATOM	3326	HW2	SOL	734	42.440	7.460	29.290	1.00	0.00
ATOM	3327	OW	SOL	735	13.330	48.090	5.580	1.00	0.00
ATOM	3328	HW1	SOL	735	13.030	47.770	4.730	1.00	0.00
ATOM	3329	HW2	SOL	735	12.530	48.390	6.020	1.00	0.00
ATOM	3330	OW	SOL	736	31.290	39.650	12.080	1.00	0.00
ATOM	3331	HW1	SOL	736	31.440	40.270	12.790	1.00	0.00
ATOM	3332	HW2	SOL	736	30.780	40.140	11.440	1.00	0.00
ATOM	3333	OW	SOL	737	48.620	8.220	50.780	1.00	0.00
ATOM	3334	HW1	SOL	737	49.220	8.920	50.510	1.00	0.00
ATOM	3335	HW2	SOL	737	49.200	7.510	51.060	1.00	0.00
ATOM	3336	OW	SOL	738	5.930	44.460	31.150	1.00	0.00
ATOM	3337	HW1	SOL	738	5.600	44.870	30.350	1.00	0.00
ATOM	3338	HW2	SOL	738	5.290	44.680	31.820	1.00	0.00
ATOM	3339	OW	SOL	739	2.550	0.930	14.410	1.00	0.00
ATOM	3340	HW1	SOL	739	2.990	0.120	14.160	1.00	0.00
ATOM	3341	HW2	SOL	739	3.200	1.610	14.260	1.00	0.00
ATOM	3342	OW	SOL	740	4.760	8.780	38.220	1.00	0.00
ATOM	3343	HW1	SOL	740	4.480	9.690	38.170	1.00	0.00

ATOM	3344	HW2	SOL	740	3.950	8.280	38.270	1.00	0.00
ATOM	3345	OW	SOL	741	28.640	24.760	50.420	1.00	0.00
ATOM	3346	HW1	SOL	741	28.460	25.640	50.100	1.00	0.00
ATOM	3347	HW2	SOL	741	29.510	24.820	50.820	1.00	0.00
ATOM	3348	OW	SOL	742	55.480	26.790	13.980	1.00	0.00
ATOM	3349	HW1	SOL	742	55.710	27.360	14.720	1.00	0.00
ATOM	3350	HW2	SOL	742	55.050	26.040	14.380	1.00	0.00
ATOM	3351	OW	SOL	743	42.580	14.560	12.510	1.00	0.00
ATOM	3352	HW1	SOL	743	42.690	15.510	12.460	1.00	0.00
ATOM	3353	HW2	SOL	743	43.330	14.260	13.020	1.00	0.00
ATOM	3354	OW	SOL	744	13.400	42.850	27.900	1.00	0.00
ATOM	3355	HW1	SOL	744	13.600	43.110	28.800	1.00	0.00
ATOM	3356	HW2	SOL	744	13.250	41.910	27.950	1.00	0.00
ATOM	3357	OW	SOL	745	42.290	49.830	19.730	1.00	0.00
ATOM	3358	HW1	SOL	745	41.610	49.910	19.060	1.00	0.00
ATOM	3359	HW2	SOL	745	41.900	49.250	20.390	1.00	0.00
ATOM	3360	OW	SOL	746	40.810	54.600	25.260	1.00	0.00
ATOM	3361	HW1	SOL	746	39.930	54.470	25.610	1.00	0.00
ATOM	3362	HW2	SOL	746	40.920	55.550	25.230	1.00	0.00
ATOM	3363	OW	SOL	747	46.260	54.330	25.700	1.00	0.00
ATOM	3364	HW1	SOL	747	46.400	53.680	26.390	1.00	0.00
ATOM	3365	HW2	SOL	747	45.330	54.270	25.500	1.00	0.00
ATOM	3366	OW	SOL	748	27.650	26.800	11.280	1.00	0.00
ATOM	3367	HW1	SOL	748	27.660	27.730	11.510	1.00	0.00
ATOM	3368	HW2	SOL	748	26.730	26.540	11.340	1.00	0.00
ATOM	3369	OW	SOL	749	9.260	55.820	48.420	1.00	0.00
ATOM	3370	HW1	SOL	749	8.960	56.370	47.700	1.00	0.00
ATOM	3371	HW2	SOL	749	9.230	54.930	48.080	1.00	0.00
ATOM	3372	OW	SOL	750	31.810	0.100	52.260	1.00	0.00
ATOM	3373	HW1	SOL	750	32.160	-0.790	52.350	1.00	0.00
ATOM	3374	HW2	SOL	750	31.010	0.100	52.790	1.00	0.00
ATOM	3375	OW	SOL	751	23.140	25.980	25.450	1.00	0.00
ATOM	3376	HW1	SOL	751	22.970	26.210	24.530	1.00	0.00
ATOM	3377	HW2	SOL	751	23.670	25.190	25.400	1.00	0.00
ATOM	3378	OW	SOL	752	29.970	55.190	20.430	1.00	0.00
ATOM	3379	HW1	SOL	752	30.740	55.460	20.920	1.00	0.00
ATOM	3380	HW2	SOL	752	30.230	54.380	19.980	1.00	0.00
ATOM	3381	OW	SOL	753	14.500	51.620	11.870	1.00	0.00
ATOM	3382	HW1	SOL	753	13.910	50.980	11.470	1.00	0.00
ATOM	3383	HW2	SOL	753	15.370	51.240	11.750	1.00	0.00
ATOM	3384	OW	SOL	754	11.040	13.720	23.150	1.00	0.00
ATOM	3385	HW1	SOL	754	10.550	14.250	23.790	1.00	0.00
ATOM	3386	HW2	SOL	754	11.180	14.310	22.410	1.00	0.00
ATOM	3387	OW	SOL	755	33.090	1.210	13.890	1.00	0.00

ATOM	3388	HW1	SOL	755	32.720	0.370	14.130	1.00	0.00
ATOM	3389	HW2	SOL	755	32.430	1.610	13.320	1.00	0.00
ATOM	3390	OW	SOL	756	51.590	54.800	52.090	1.00	0.00
ATOM	3391	HW1	SOL	756	52.250	55.490	52.130	1.00	0.00
ATOM	3392	HW2	SOL	756	51.520	54.490	53.000	1.00	0.00
ATOM	3393	OW	SOL	757	10.870	48.960	4.010	1.00	0.00
ATOM	3394	HW1	SOL	757	11.670	49.430	3.760	1.00	0.00
ATOM	3395	HW2	SOL	757	11.090	48.040	3.860	1.00	0.00
ATOM	3396	OW	SOL	758	27.090	44.430	7.910	1.00	0.00
ATOM	3397	HW1	SOL	758	27.050	43.990	7.060	1.00	0.00
ATOM	3398	HW2	SOL	758	27.800	45.070	7.820	1.00	0.00
ATOM	3399	OW	SOL	759	7.290	48.480	38.130	1.00	0.00
ATOM	3400	HW1	SOL	759	8.210	48.260	38.070	1.00	0.00
ATOM	3401	HW2	SOL	759	6.840	47.800	37.630	1.00	0.00
ATOM	3402	OW	SOL	760	20.450	41.310	15.530	1.00	0.00
ATOM	3403	HW1	SOL	760	19.990	40.510	15.760	1.00	0.00
ATOM	3404	HW2	SOL	760	19.750	41.950	15.350	1.00	0.00
ATOM	3405	OW	SOL	761	48.540	12.460	11.930	1.00	0.00
ATOM	3406	HW1	SOL	761	48.520	13.350	11.560	1.00	0.00
ATOM	3407	HW2	SOL	761	48.540	11.880	11.170	1.00	0.00
ATOM	3408	OW	SOL	762	21.790	39.070	47.410	1.00	0.00
ATOM	3409	HW1	SOL	762	21.700	39.620	48.200	1.00	0.00
ATOM	3410	HW2	SOL	762	20.900	39.010	47.070	1.00	0.00
ATOM	3411	OW	SOL	763	4.910	49.110	51.440	1.00	0.00
ATOM	3412	HW1	SOL	763	5.050	49.910	51.940	1.00	0.00
ATOM	3413	HW2	SOL	763	5.230	49.330	50.560	1.00	0.00
ATOM	3414	OW	SOL	764	15.080	41.660	32.390	1.00	0.00
ATOM	3415	HW1	SOL	764	15.760	41.030	32.120	1.00	0.00
ATOM	3416	HW2	SOL	764	15.490	42.190	33.070	1.00	0.00
ATOM	3417	OW	SOL	765	14.940	30.150	3.770	1.00	0.00
ATOM	3418	HW1	SOL	765	14.170	29.740	3.370	1.00	0.00
ATOM	3419	HW2	SOL	765	15.540	29.430	3.940	1.00	0.00
ATOM	3420	OW	SOL	766	15.180	45.560	37.730	1.00	0.00
ATOM	3421	HW1	SOL	766	14.260	45.730	37.540	1.00	0.00
ATOM	3422	HW2	SOL	766	15.510	46.380	38.090	1.00	0.00
ATOM	3423	OW	SOL	767	20.590	40.020	55.610	1.00	0.00
ATOM	3424	HW1	SOL	767	20.440	39.080	55.690	1.00	0.00
ATOM	3425	HW2	SOL	767	21.300	40.200	56.230	1.00	0.00
ATOM	3426	OW	SOL	768	19.400	38.110	9.540	1.00	0.00
ATOM	3427	HW1	SOL	768	19.930	38.620	10.160	1.00	0.00
ATOM	3428	HW2	SOL	768	19.090	38.750	8.900	1.00	0.00
ATOM	3429	OW	SOL	769	53.980	5.780	38.490	1.00	0.00
ATOM	3430	HW1	SOL	769	53.600	6.650	38.390	1.00	0.00
ATOM	3431	HW2	SOL	769	53.280	5.270	38.910	1.00	0.00

ATOM	3432	OW	SOL	770	18.790	23.380	41.310	1.00	0.00
ATOM	3433	HW1	SOL	770	18.150	22.790	41.710	1.00	0.00
ATOM	3434	HW2	SOL	770	18.290	23.870	40.660	1.00	0.00
ATOM	3435	OW	SOL	771	3.470	46.620	29.870	1.00	0.00
ATOM	3436	HW1	SOL	771	3.420	47.350	30.490	1.00	0.00
ATOM	3437	HW2	SOL	771	2.880	45.960	30.220	1.00	0.00
ATOM	3438	OW	SOL	772	53.540	11.080	44.950	1.00	0.00
ATOM	3439	HW1	SOL	772	54.210	11.060	44.260	1.00	0.00
ATOM	3440	HW2	SOL	772	53.320	12.000	45.040	1.00	0.00
ATOM	3441	OW	SOL	773	54.090	34.430	16.640	1.00	0.00
ATOM	3442	HW1	SOL	773	53.570	33.640	16.790	1.00	0.00
ATOM	3443	HW2	SOL	773	54.050	34.900	17.470	1.00	0.00
ATOM	3444	OW	SOL	774	34.680	54.730	40.970	1.00	0.00
ATOM	3445	HW1	SOL	774	34.510	55.330	40.250	1.00	0.00
ATOM	3446	HW2	SOL	774	35.260	54.070	40.610	1.00	0.00
ATOM	3447	OW	SOL	775	43.580	25.790	49.070	1.00	0.00
ATOM	3448	HW1	SOL	775	44.160	25.140	49.470	1.00	0.00
ATOM	3449	HW2	SOL	775	43.570	25.550	48.140	1.00	0.00
ATOM	3450	OW	SOL	776	38.220	46.970	36.810	1.00	0.00
ATOM	3451	HW1	SOL	776	37.520	46.320	36.800	1.00	0.00
ATOM	3452	HW2	SOL	776	38.790	46.720	36.080	1.00	0.00
ATOM	3453	OW	SOL	777	5.450	3.400	9.320	1.00	0.00
ATOM	3454	HW1	SOL	777	5.120	3.670	8.470	1.00	0.00
ATOM	3455	HW2	SOL	777	5.360	2.440	9.320	1.00	0.00
ATOM	3456	OW	SOL	778	11.530	4.890	2.200	1.00	0.00
ATOM	3457	HW1	SOL	778	11.620	5.680	1.660	1.00	0.00
ATOM	3458	HW2	SOL	778	10.870	4.360	1.750	1.00	0.00
ATOM	3459	OW	SOL	779	28.390	43.670	10.430	1.00	0.00
ATOM	3460	HW1	SOL	779	28.160	44.080	9.600	1.00	0.00
ATOM	3461	HW2	SOL	779	28.280	44.360	11.080	1.00	0.00
ATOM	3462	OW	SOL	780	1.640	41.020	1.670	1.00	0.00
ATOM	3463	HW1	SOL	780	2.070	41.470	2.400	1.00	0.00
ATOM	3464	HW2	SOL	780	1.440	41.720	1.040	1.00	0.00
ATOM	3465	OW	SOL	781	19.760	24.690	2.170	1.00	0.00
ATOM	3466	HW1	SOL	781	20.010	25.290	2.870	1.00	0.00
ATOM	3467	HW2	SOL	781	19.370	23.940	2.630	1.00	0.00
ATOM	3468	OW	SOL	782	10.810	17.450	17.610	1.00	0.00
ATOM	3469	HW1	SOL	782	11.040	17.160	16.730	1.00	0.00
ATOM	3470	HW2	SOL	782	11.390	16.940	18.180	1.00	0.00
ATOM	3471	OW	SOL	783	23.460	28.340	19.100	1.00	0.00
ATOM	3472	HW1	SOL	783	22.850	29.020	18.840	1.00	0.00
ATOM	3473	HW2	SOL	783	23.840	28.030	18.280	1.00	0.00
ATOM	3474	OW	SOL	784	8.560	42.260	48.570	1.00	0.00
ATOM	3475	HW1	SOL	784	9.410	41.880	48.810	1.00	0.00

ATOM	3476	HW2	SOL	784	8.090	41.540	48.160	1.00	0.00
ATOM	3477	OW	SOL	785	51.750	54.210	45.490	1.00	0.00
ATOM	3478	HW1	SOL	785	52.450	53.760	45.010	1.00	0.00
ATOM	3479	HW2	SOL	785	51.640	53.690	46.280	1.00	0.00
ATOM	3480	OW	SOL	786	2.300	12.820	19.610	1.00	0.00
ATOM	3481	HW1	SOL	786	2.030	13.420	18.930	1.00	0.00
ATOM	3482	HW2	SOL	786	3.160	13.140	19.890	1.00	0.00
ATOM	3483	OW	SOL	787	46.850	0.990	19.890	1.00	0.00
ATOM	3484	HW1	SOL	787	47.350	1.770	19.630	1.00	0.00
ATOM	3485	HW2	SOL	787	46.910	0.990	20.850	1.00	0.00
ATOM	3486	OW	SOL	788	37.590	14.600	21.950	1.00	0.00
ATOM	3487	HW1	SOL	788	36.760	14.670	21.480	1.00	0.00
ATOM	3488	HW2	SOL	788	37.510	15.220	22.680	1.00	0.00
ATOM	3489	OW	SOL	789	28.280	5.310	1.700	1.00	0.00
ATOM	3490	HW1	SOL	789	29.190	5.410	1.430	1.00	0.00
ATOM	3491	HW2	SOL	789	28.310	5.320	2.660	1.00	0.00
ATOM	3492	OW	SOL	790	19.560	39.780	5.110	1.00	0.00
ATOM	3493	HW1	SOL	790	19.540	39.300	4.270	1.00	0.00
ATOM	3494	HW2	SOL	790	19.070	39.210	5.710	1.00	0.00
ATOM	3495	OW	SOL	791	54.390	44.030	27.150	1.00	0.00
ATOM	3496	HW1	SOL	791	53.690	43.810	27.760	1.00	0.00
ATOM	3497	HW2	SOL	791	53.950	44.530	26.460	1.00	0.00
ATOM	3498	OW	SOL	792	24.180	10.420	50.980	1.00	0.00
ATOM	3499	HW1	SOL	792	25.060	10.070	50.820	1.00	0.00
ATOM	3500	HW2	SOL	792	24.330	11.250	51.440	1.00	0.00
ATOM	3501	OW	SOL	793	10.660	6.080	11.220	1.00	0.00
ATOM	3502	HW1	SOL	793	10.550	6.930	11.630	1.00	0.00
ATOM	3503	HW2	SOL	793	10.890	5.490	11.940	1.00	0.00
ATOM	3504	OW	SOL	794	7.920	55.320	5.020	1.00	0.00
ATOM	3505	HW1	SOL	794	8.710	55.810	4.820	1.00	0.00
ATOM	3506	HW2	SOL	794	7.650	54.950	4.180	1.00	0.00
ATOM	3507	OW	SOL	795	22.780	49.070	0.730	1.00	0.00
ATOM	3508	HW1	SOL	795	23.430	48.600	1.240	1.00	0.00
ATOM	3509	HW2	SOL	795	23.130	49.090	-0.160	1.00	0.00
ATOM	3510	OW	SOL	796	39.730	12.980	11.460	1.00	0.00
ATOM	3511	HW1	SOL	796	39.980	13.830	11.090	1.00	0.00
ATOM	3512	HW2	SOL	796	38.780	12.980	11.440	1.00	0.00
ATOM	3513	OW	SOL	797	29.660	42.470	6.390	1.00	0.00
ATOM	3514	HW1	SOL	797	30.090	42.920	5.660	1.00	0.00
ATOM	3515	HW2	SOL	797	28.730	42.460	6.150	1.00	0.00
ATOM	3516	OW	SOL	798	47.480	6.680	38.510	1.00	0.00
ATOM	3517	HW1	SOL	798	47.760	6.050	37.840	1.00	0.00
ATOM	3518	HW2	SOL	798	48.250	6.780	39.080	1.00	0.00
ATOM	3519	OW	SOL	799	23.800	47.930	19.550	1.00	0.00

ATOM	3520	HW1	SOL	799	23.470	47.880	18.650	1.00	0.00
ATOM	3521	HW2	SOL	799	24.750	47.900	19.450	1.00	0.00
ATOM	3522	OW	SOL	800	36.900	13.890	14.870	1.00	0.00
ATOM	3523	HW1	SOL	800	37.620	14.360	15.300	1.00	0.00
ATOM	3524	HW2	SOL	800	36.250	13.780	15.560	1.00	0.00
ATOM	3525	OW	SOL	801	14.540	23.770	30.590	1.00	0.00
ATOM	3526	HW1	SOL	801	14.510	24.300	31.390	1.00	0.00
ATOM	3527	HW2	SOL	801	14.230	22.910	30.860	1.00	0.00
ATOM	3528	OW	SOL	802	47.170	36.350	12.570	1.00	0.00
ATOM	3529	HW1	SOL	802	47.330	35.980	11.700	1.00	0.00
ATOM	3530	HW2	SOL	802	47.670	35.790	13.170	1.00	0.00
ATOM	3531	OW	SOL	803	3.770	54.900	40.720	1.00	0.00
ATOM	3532	HW1	SOL	803	3.880	55.450	41.500	1.00	0.00
ATOM	3533	HW2	SOL	803	3.520	55.520	40.030	1.00	0.00
ATOM	3534	OW	SOL	804	12.450	22.840	38.380	1.00	0.00
ATOM	3535	HW1	SOL	804	12.220	23.760	38.360	1.00	0.00
ATOM	3536	HW2	SOL	804	12.080	22.510	39.200	1.00	0.00
ATOM	3537	OW	SOL	805	17.500	28.290	15.970	1.00	0.00
ATOM	3538	HW1	SOL	805	16.960	28.180	15.190	1.00	0.00
ATOM	3539	HW2	SOL	805	16.870	28.430	16.680	1.00	0.00
ATOM	3540	OW	SOL	806	5.850	16.720	17.610	1.00	0.00
ATOM	3541	HW1	SOL	806	6.040	16.430	16.720	1.00	0.00
ATOM	3542	HW2	SOL	806	5.650	17.650	17.520	1.00	0.00
ATOM	3543	OW	SOL	807	7.350	55.860	24.170	1.00	0.00
ATOM	3544	HW1	SOL	807	7.250	55.250	23.440	1.00	0.00
ATOM	3545	HW2	SOL	807	6.510	56.300	24.240	1.00	0.00
ATOM	3546	OW	SOL	808	23.010	14.710	49.030	1.00	0.00
ATOM	3547	HW1	SOL	808	22.260	15.040	48.530	1.00	0.00
ATOM	3548	HW2	SOL	808	22.610	14.280	49.790	1.00	0.00
ATOM	3549	OW	SOL	809	36.670	44.700	50.940	1.00	0.00
ATOM	3550	HW1	SOL	809	37.520	44.850	50.540	1.00	0.00
ATOM	3551	HW2	SOL	809	36.190	45.520	50.810	1.00	0.00
ATOM	3552	OW	SOL	810	18.140	16.520	2.160	1.00	0.00
ATOM	3553	HW1	SOL	810	18.420	16.730	1.270	1.00	0.00
ATOM	3554	HW2	SOL	810	17.230	16.230	2.060	1.00	0.00
ATOM	3555	OW	SOL	811	21.940	13.510	2.430	1.00	0.00
ATOM	3556	HW1	SOL	811	22.450	13.700	3.220	1.00	0.00
ATOM	3557	HW2	SOL	811	21.030	13.500	2.730	1.00	0.00
ATOM	3558	OW	SOL	812	6.030	42.520	21.390	1.00	0.00
ATOM	3559	HW1	SOL	812	6.740	41.900	21.220	1.00	0.00
ATOM	3560	HW2	SOL	812	6.070	42.660	22.340	1.00	0.00
ATOM	3561	OW	SOL	813	51.010	39.300	46.860	1.00	0.00
ATOM	3562	HW1	SOL	813	50.610	39.780	46.140	1.00	0.00
ATOM	3563	HW2	SOL	813	51.530	39.950	47.330	1.00	0.00

ATOM	3564	OW	SOL	814	36.290	45.030	36.800	1.00	0.00
ATOM	3565	HW1	SOL	814	36.010	44.310	37.370	1.00	0.00
ATOM	3566	HW2	SOL	814	35.480	45.340	36.390	1.00	0.00
ATOM	3567	OW	SOL	815	20.710	16.060	36.640	1.00	0.00
ATOM	3568	HW1	SOL	815	21.590	15.900	36.280	1.00	0.00
ATOM	3569	HW2	SOL	815	20.820	16.850	37.170	1.00	0.00
ATOM	3570	OW	SOL	816	34.690	19.810	48.430	1.00	0.00
ATOM	3571	HW1	SOL	816	35.420	19.340	48.840	1.00	0.00
ATOM	3572	HW2	SOL	816	34.250	19.150	47.900	1.00	0.00
ATOM	3573	OW	SOL	817	0.020	41.890	3.900	1.00	0.00
ATOM	3574	HW1	SOL	817	-0.060	41.320	3.140	1.00	0.00
ATOM	3575	HW2	SOL	817	0.800	41.570	4.360	1.00	0.00
ATOM	3576	OW	SOL	818	22.050	41.430	1.460	1.00	0.00
ATOM	3577	HW1	SOL	818	23.000	41.550	1.540	1.00	0.00
ATOM	3578	HW2	SOL	818	21.720	41.520	2.350	1.00	0.00
ATOM	3579	OW	SOL	819	35.550	21.390	17.860	1.00	0.00
ATOM	3580	HW1	SOL	819	36.110	20.860	17.290	1.00	0.00
ATOM	3581	HW2	SOL	819	35.710	21.060	18.740	1.00	0.00
ATOM	3582	OW	SOL	820	50.710	49.710	47.480	1.00	0.00
ATOM	3583	HW1	SOL	820	50.400	48.850	47.780	1.00	0.00
ATOM	3584	HW2	SOL	820	50.960	49.570	46.570	1.00	0.00
ATOM	3585	OW	SOL	821	19.340	34.960	4.490	1.00	0.00
ATOM	3586	HW1	SOL	821	19.560	35.120	5.410	1.00	0.00
ATOM	3587	HW2	SOL	821	19.350	34.000	4.400	1.00	0.00
ATOM	3588	OW	SOL	822	50.620	48.980	26.400	1.00	0.00
ATOM	3589	HW1	SOL	822	51.440	48.900	25.910	1.00	0.00
ATOM	3590	HW2	SOL	822	50.650	48.250	27.030	1.00	0.00
ATOM	3591	OW	SOL	823	43.720	54.070	25.040	1.00	0.00
ATOM	3592	HW1	SOL	823	42.780	53.870	25.080	1.00	0.00
ATOM	3593	HW2	SOL	823	43.970	53.850	24.140	1.00	0.00
ATOM	3594	OW	SOL	824	16.400	47.110	24.060	1.00	0.00
ATOM	3595	HW1	SOL	824	16.710	46.460	24.700	1.00	0.00
ATOM	3596	HW2	SOL	824	16.440	47.940	24.530	1.00	0.00
ATOM	3597	OW	SOL	825	7.430	47.390	40.790	1.00	0.00
ATOM	3598	HW1	SOL	825	7.740	48.060	40.170	1.00	0.00
ATOM	3599	HW2	SOL	825	6.490	47.520	40.840	1.00	0.00
ATOM	3600	OW	SOL	826	46.760	10.300	0.590	1.00	0.00
ATOM	3601	HW1	SOL	826	46.890	10.530	-0.330	1.00	0.00
ATOM	3602	HW2	SOL	826	47.560	9.840	0.840	1.00	0.00
ATOM	3603	OW	SOL	827	10.850	11.700	55.590	1.00	0.00
ATOM	3604	HW1	SOL	827	10.580	12.610	55.540	1.00	0.00
ATOM	3605	HW2	SOL	827	10.380	11.360	56.350	1.00	0.00
ATOM	3606	OW	SOL	828	13.760	54.700	37.260	1.00	0.00
ATOM	3607	HW1	SOL	828	13.580	54.510	36.340	1.00	0.00

ATOM	3608	HW2	SOL	828	14.080	55.600	37.270	1.00	0.00
ATOM	3609	OW	SOL	829	53.840	27.870	17.190	1.00	0.00
ATOM	3610	HW1	SOL	829	53.580	27.170	16.580	1.00	0.00
ATOM	3611	HW2	SOL	829	53.010	28.240	17.490	1.00	0.00
ATOM	3612	OW	SOL	830	8.370	23.680	0.150	1.00	0.00
ATOM	3613	HW1	SOL	830	7.910	24.310	-0.410	1.00	0.00
ATOM	3614	HW2	SOL	830	8.610	22.970	-0.450	1.00	0.00
ATOM	3615	OW	SOL	831	28.620	51.180	31.760	1.00	0.00
ATOM	3616	HW1	SOL	831	27.980	51.840	31.520	1.00	0.00
ATOM	3617	HW2	SOL	831	29.460	51.640	31.730	1.00	0.00
ATOM	3618	OW	SOL	832	32.390	3.810	23.370	1.00	0.00
ATOM	3619	HW1	SOL	832	33.140	3.770	22.770	1.00	0.00
ATOM	3620	HW2	SOL	832	31.620	3.810	22.800	1.00	0.00
ATOM	3621	OW	SOL	833	33.700	41.270	22.110	1.00	0.00
ATOM	3622	HW1	SOL	833	33.050	41.960	22.030	1.00	0.00
ATOM	3623	HW2	SOL	833	34.260	41.380	21.340	1.00	0.00
ATOM	3624	OW	SOL	834	27.900	47.650	25.530	1.00	0.00
ATOM	3625	HW1	SOL	834	27.220	48.200	25.140	1.00	0.00
ATOM	3626	HW2	SOL	834	28.490	47.440	24.810	1.00	0.00
ATOM	3627	OW	SOL	835	34.970	30.440	20.240	1.00	0.00
ATOM	3628	HW1	SOL	835	34.940	29.670	19.680	1.00	0.00
ATOM	3629	HW2	SOL	835	35.190	30.100	21.110	1.00	0.00
ATOM	3630	OW	SOL	836	42.160	49.830	0.320	1.00	0.00
ATOM	3631	HW1	SOL	836	42.300	50.210	-0.540	1.00	0.00
ATOM	3632	HW2	SOL	836	41.550	50.430	0.760	1.00	0.00
ATOM	3633	OW	SOL	837	26.020	38.490	11.430	1.00	0.00
ATOM	3634	HW1	SOL	837	26.040	39.320	10.960	1.00	0.00
ATOM	3635	HW2	SOL	837	25.150	38.130	11.250	1.00	0.00
ATOM	3636	OW	SOL	838	48.080	5.990	48.690	1.00	0.00
ATOM	3637	HW1	SOL	838	48.110	6.810	49.180	1.00	0.00
ATOM	3638	HW2	SOL	838	48.990	5.700	48.650	1.00	0.00
ATOM	3639	OW	SOL	839	52.460	39.990	18.260	1.00	0.00
ATOM	3640	HW1	SOL	839	52.900	39.420	18.890	1.00	0.00
ATOM	3641	HW2	SOL	839	52.940	39.880	17.440	1.00	0.00
ATOM	3642	OW	SOL	840	44.100	28.070	12.080	1.00	0.00
ATOM	3643	HW1	SOL	840	43.260	27.620	12.200	1.00	0.00
ATOM	3644	HW2	SOL	840	44.270	28.000	11.150	1.00	0.00
ATOM	3645	OW	SOL	841	44.820	39.660	42.740	1.00	0.00
ATOM	3646	HW1	SOL	841	45.500	40.290	43.000	1.00	0.00
ATOM	3647	HW2	SOL	841	44.090	40.200	42.470	1.00	0.00
ATOM	3648	OW	SOL	842	11.420	51.580	24.700	1.00	0.00
ATOM	3649	HW1	SOL	842	10.890	50.840	24.380	1.00	0.00
ATOM	3650	HW2	SOL	842	10.870	52.350	24.540	1.00	0.00
ATOM	3651	OW	SOL	843	35.470	53.040	0.710	1.00	0.00

ATOM	3652	HW1	SOL	843	35.410	53.990	0.630	1.00	0.00
ATOM	3653	HW2	SOL	843	34.580	52.750	0.880	1.00	0.00
ATOM	3654	OW	SOL	844	30.960	52.030	38.880	1.00	0.00
ATOM	3655	HW1	SOL	844	31.900	51.880	39.010	1.00	0.00
ATOM	3656	HW2	SOL	844	30.580	51.860	39.740	1.00	0.00
ATOM	3657	OW	SOL	845	50.660	46.860	28.170	1.00	0.00
ATOM	3658	HW1	SOL	845	51.540	46.600	28.450	1.00	0.00
ATOM	3659	HW2	SOL	845	50.090	46.190	28.540	1.00	0.00
ATOM	3660	OW	SOL	846	28.760	37.230	14.860	1.00	0.00
ATOM	3661	HW1	SOL	846	28.310	36.390	14.920	1.00	0.00
ATOM	3662	HW2	SOL	846	28.290	37.800	15.470	1.00	0.00
ATOM	3663	OW	SOL	847	2.120	48.310	34.920	1.00	0.00
ATOM	3664	HW1	SOL	847	2.600	48.770	35.600	1.00	0.00
ATOM	3665	HW2	SOL	847	1.580	48.980	34.500	1.00	0.00
ATOM	3666	OW	SOL	848	45.940	19.920	11.540	1.00	0.00
ATOM	3667	HW1	SOL	848	46.050	20.800	11.190	1.00	0.00
ATOM	3668	HW2	SOL	848	45.130	19.600	11.130	1.00	0.00
ATOM	3669	OW	SOL	849	3.460	27.190	45.430	1.00	0.00
ATOM	3670	HW1	SOL	849	3.670	27.940	44.880	1.00	0.00
ATOM	3671	HW2	SOL	849	2.560	26.970	45.190	1.00	0.00
ATOM	3672	OW	SOL	850	52.640	22.500	7.680	1.00	0.00
ATOM	3673	HW1	SOL	850	52.460	21.760	7.100	1.00	0.00
ATOM	3674	HW2	SOL	850	53.360	22.970	7.260	1.00	0.00
ATOM	3675	OW	SOL	851	41.740	4.240	44.310	1.00	0.00
ATOM	3676	HW1	SOL	851	41.460	5.150	44.330	1.00	0.00
ATOM	3677	HW2	SOL	851	41.880	4.050	43.380	1.00	0.00
ATOM	3678	OW	SOL	852	48.490	49.340	24.140	1.00	0.00
ATOM	3679	HW1	SOL	852	48.880	49.130	24.990	1.00	0.00
ATOM	3680	HW2	SOL	852	48.990	48.830	23.510	1.00	0.00
ATOM	3681	OW	SOL	853	21.170	23.180	32.730	1.00	0.00
ATOM	3682	HW1	SOL	853	20.620	23.880	33.090	1.00	0.00
ATOM	3683	HW2	SOL	853	20.860	22.380	33.160	1.00	0.00
ATOM	3684	OW	SOL	854	26.650	48.050	14.780	1.00	0.00
ATOM	3685	HW1	SOL	854	26.660	47.760	13.860	1.00	0.00
ATOM	3686	HW2	SOL	854	27.440	48.560	14.880	1.00	0.00
ATOM	3687	OW	SOL	855	13.840	55.640	29.890	1.00	0.00
ATOM	3688	HW1	SOL	855	14.220	55.420	30.740	1.00	0.00
ATOM	3689	HW2	SOL	855	12.940	55.310	29.940	1.00	0.00
ATOM	3690	OW	SOL	856	16.980	53.700	20.150	1.00	0.00
ATOM	3691	HW1	SOL	856	17.590	53.890	20.860	1.00	0.00
ATOM	3692	HW2	SOL	856	17.510	53.790	19.350	1.00	0.00
ATOM	3693	OW	SOL	857	7.890	55.150	30.170	1.00	0.00
ATOM	3694	HW1	SOL	857	7.810	54.220	30.400	1.00	0.00
ATOM	3695	HW2	SOL	857	7.690	55.180	29.240	1.00	0.00

ATOM	3696	OW	SOL	858	15.600	2.180	45.410	1.00	0.00
ATOM	3697	HW1	SOL	858	16.400	2.510	45.810	1.00	0.00
ATOM	3698	HW2	SOL	858	15.230	2.940	44.960	1.00	0.00
ATOM	3699	OW	SOL	859	12.170	22.900	35.030	1.00	0.00
ATOM	3700	HW1	SOL	859	12.900	22.280	34.930	1.00	0.00
ATOM	3701	HW2	SOL	859	12.210	23.160	35.950	1.00	0.00
ATOM	3702	OW	SOL	860	25.260	43.140	15.540	1.00	0.00
ATOM	3703	HW1	SOL	860	24.790	42.380	15.190	1.00	0.00
ATOM	3704	HW2	SOL	860	25.410	43.700	14.780	1.00	0.00
ATOM	3705	OW	SOL	861	4.640	32.960	43.560	1.00	0.00
ATOM	3706	HW1	SOL	861	4.800	33.760	43.050	1.00	0.00
ATOM	3707	HW2	SOL	861	5.010	33.150	44.420	1.00	0.00
ATOM	3708	OW	SOL	862	55.860	25.450	48.080	1.00	0.00
ATOM	3709	HW1	SOL	862	56.330	25.720	48.870	1.00	0.00
ATOM	3710	HW2	SOL	862	54.970	25.300	48.370	1.00	0.00
ATOM	3711	OW	SOL	863	23.740	31.160	31.430	1.00	0.00
ATOM	3712	HW1	SOL	863	24.160	30.330	31.620	1.00	0.00
ATOM	3713	HW2	SOL	863	22.800	30.960	31.430	1.00	0.00
ATOM	3714	OW	SOL	864	29.910	44.910	12.940	1.00	0.00
ATOM	3715	HW1	SOL	864	29.840	44.300	13.670	1.00	0.00
ATOM	3716	HW2	SOL	864	30.460	45.620	13.270	1.00	0.00
ATOM	3717	OW	SOL	865	36.470	41.540	3.600	1.00	0.00
ATOM	3718	HW1	SOL	865	36.860	41.360	4.450	1.00	0.00
ATOM	3719	HW2	SOL	865	37.210	41.560	2.990	1.00	0.00
ATOM	3720	OW	SOL	866	28.290	3.550	55.390	1.00	0.00
ATOM	3721	HW1	SOL	866	28.280	2.680	55.780	1.00	0.00
ATOM	3722	HW2	SOL	866	28.290	4.150	56.140	1.00	0.00
ATOM	3723	OW	SOL	867	2.990	38.220	15.180	1.00	0.00
ATOM	3724	HW1	SOL	867	2.850	39.040	14.710	1.00	0.00
ATOM	3725	HW2	SOL	867	2.170	38.070	15.650	1.00	0.00
ATOM	3726	OW	SOL	868	39.360	34.380	25.850	1.00	0.00
ATOM	3727	HW1	SOL	868	39.320	35.210	25.370	1.00	0.00
ATOM	3728	HW2	SOL	868	38.460	34.050	25.840	1.00	0.00
ATOM	3729	OW	SOL	869	0.030	8.340	0.940	1.00	0.00
ATOM	3730	HW1	SOL	869	0.860	8.280	1.420	1.00	0.00
ATOM	3731	HW2	SOL	869	0.290	8.470	0.030	1.00	0.00
ATOM	3732	OW	SOL	870	38.770	7.950	32.530	1.00	0.00
ATOM	3733	HW1	SOL	870	39.710	7.780	32.530	1.00	0.00
ATOM	3734	HW2	SOL	870	38.370	7.090	32.650	1.00	0.00
ATOM	3735	OW	SOL	871	52.490	30.760	55.610	1.00	0.00
ATOM	3736	HW1	SOL	871	52.950	30.720	54.770	1.00	0.00
ATOM	3737	HW2	SOL	871	53.180	30.680	56.270	1.00	0.00
ATOM	3738	OW	SOL	872	4.160	5.450	49.560	1.00	0.00
ATOM	3739	HW1	SOL	872	4.500	4.590	49.310	1.00	0.00

ATOM	3740	HW2	SOL	872	3.220	5.310	49.690	1.00	0.00
ATOM	3741	OW	SOL	873	19.390	32.840	43.180	1.00	0.00
ATOM	3742	HW1	SOL	873	19.310	31.940	43.510	1.00	0.00
ATOM	3743	HW2	SOL	873	18.510	33.050	42.850	1.00	0.00
ATOM	3744	OW	SOL	874	43.930	45.340	28.820	1.00	0.00
ATOM	3745	HW1	SOL	874	43.330	45.190	28.080	1.00	0.00
ATOM	3746	HW2	SOL	874	44.650	44.730	28.660	1.00	0.00
ATOM	3747	OW	SOL	875	29.070	42.830	18.820	1.00	0.00
ATOM	3748	HW1	SOL	875	28.470	43.250	18.200	1.00	0.00
ATOM	3749	HW2	SOL	875	28.880	43.240	19.660	1.00	0.00
ATOM	3750	OW	SOL	876	50.510	55.820	19.320	1.00	0.00
ATOM	3751	HW1	SOL	876	51.050	55.030	19.370	1.00	0.00
ATOM	3752	HW2	SOL	876	51.060	56.460	18.870	1.00	0.00
ATOM	3753	OW	SOL	877	38.840	28.350	12.420	1.00	0.00
ATOM	3754	HW1	SOL	877	39.040	27.910	13.240	1.00	0.00
ATOM	3755	HW2	SOL	877	37.940	28.100	12.230	1.00	0.00
ATOM	3756	OW	SOL	878	6.210	19.740	29.800	1.00	0.00
ATOM	3757	HW1	SOL	878	5.290	19.470	29.830	1.00	0.00
ATOM	3758	HW2	SOL	878	6.260	20.480	30.410	1.00	0.00
ATOM	3759	OW	SOL	879	42.000	10.800	37.630	1.00	0.00
ATOM	3760	HW1	SOL	879	42.790	10.260	37.700	1.00	0.00
ATOM	3761	HW2	SOL	879	42.250	11.640	38.010	1.00	0.00
ATOM	3762	OW	SOL	880	53.480	18.240	5.030	1.00	0.00
ATOM	3763	HW1	SOL	880	54.370	17.980	5.270	1.00	0.00
ATOM	3764	HW2	SOL	880	53.600	18.830	4.290	1.00	0.00
ATOM	3765	OW	SOL	881	21.910	40.740	19.400	1.00	0.00
ATOM	3766	HW1	SOL	881	22.550	41.430	19.570	1.00	0.00
ATOM	3767	HW2	SOL	881	21.070	41.190	19.330	1.00	0.00
ATOM	3768	OW	SOL	882	16.220	22.570	34.290	1.00	0.00
ATOM	3769	HW1	SOL	882	15.900	22.870	35.140	1.00	0.00
ATOM	3770	HW2	SOL	882	17.080	22.980	34.200	1.00	0.00
ATOM	3771	OW	SOL	883	11.730	40.350	4.160	1.00	0.00
ATOM	3772	HW1	SOL	883	11.790	40.330	5.110	1.00	0.00
ATOM	3773	HW2	SOL	883	11.460	41.240	3.950	1.00	0.00
ATOM	3774	OW	SOL	884	54.810	9.930	6.550	1.00	0.00
ATOM	3775	HW1	SOL	884	54.140	10.270	7.130	1.00	0.00
ATOM	3776	HW2	SOL	884	54.700	8.980	6.590	1.00	0.00
ATOM	3777	OW	SOL	885	35.650	5.390	30.480	1.00	0.00
ATOM	3778	HW1	SOL	885	34.770	5.740	30.380	1.00	0.00
ATOM	3779	HW2	SOL	885	35.550	4.440	30.430	1.00	0.00
ATOM	3780	OW	SOL	886	44.810	52.120	14.140	1.00	0.00
ATOM	3781	HW1	SOL	886	45.430	52.380	14.830	1.00	0.00
ATOM	3782	HW2	SOL	886	44.930	51.170	14.070	1.00	0.00
ATOM	3783	OW	SOL	887	3.850	34.070	52.800	1.00	0.00

ATOM	3784	HW1	SOL	887	3.580	34.910	52.420	1.00	0.00
ATOM	3785	HW2	SOL	887	3.590	33.420	52.150	1.00	0.00
ATOM	3786	OW	SOL	888	24.160	24.400	28.300	1.00	0.00
ATOM	3787	HW1	SOL	888	23.510	24.580	28.980	1.00	0.00
ATOM	3788	HW2	SOL	888	24.450	25.270	28.010	1.00	0.00
ATOM	3789	OW	SOL	889	21.260	44.010	40.510	1.00	0.00
ATOM	3790	HW1	SOL	889	22.190	43.830	40.610	1.00	0.00
ATOM	3791	HW2	SOL	889	20.850	43.140	40.560	1.00	0.00
ATOM	3792	OW	SOL	890	49.210	6.870	24.300	1.00	0.00
ATOM	3793	HW1	SOL	890	48.450	7.450	24.270	1.00	0.00
ATOM	3794	HW2	SOL	890	49.030	6.280	25.040	1.00	0.00
ATOM	3795	OW	SOL	891	51.710	0.800	24.390	1.00	0.00
ATOM	3796	HW1	SOL	891	52.320	0.980	23.680	1.00	0.00
ATOM	3797	HW2	SOL	891	51.800	-0.130	24.570	1.00	0.00
ATOM	3798	OW	SOL	892	12.300	31.120	30.310	1.00	0.00
ATOM	3799	HW1	SOL	892	12.450	31.510	29.450	1.00	0.00
ATOM	3800	HW2	SOL	892	11.350	31.030	30.360	1.00	0.00
ATOM	3801	OW	SOL	893	21.600	9.620	49.930	1.00	0.00
ATOM	3802	HW1	SOL	893	22.380	9.920	50.390	1.00	0.00
ATOM	3803	HW2	SOL	893	21.630	8.670	49.980	1.00	0.00
ATOM	3804	OW	SOL	894	1.980	19.700	15.750	1.00	0.00
ATOM	3805	HW1	SOL	894	1.030	19.840	15.730	1.00	0.00
ATOM	3806	HW2	SOL	894	2.300	20.100	14.940	1.00	0.00
ATOM	3807	OW	SOL	895	4.470	45.900	13.190	1.00	0.00
ATOM	3808	HW1	SOL	895	5.350	45.740	13.530	1.00	0.00
ATOM	3809	HW2	SOL	895	3.890	45.640	13.910	1.00	0.00
ATOM	3810	OW	SOL	896	38.390	7.680	50.320	1.00	0.00
ATOM	3811	HW1	SOL	896	39.340	7.570	50.310	1.00	0.00
ATOM	3812	HW2	SOL	896	38.100	7.270	49.510	1.00	0.00
ATOM	3813	OW	SOL	897	50.870	48.250	4.470	1.00	0.00
ATOM	3814	HW1	SOL	897	50.150	48.880	4.550	1.00	0.00
ATOM	3815	HW2	SOL	897	50.590	47.490	4.980	1.00	0.00
ATOM	3816	OW	SOL	898	39.800	26.170	7.890	1.00	0.00
ATOM	3817	HW1	SOL	898	39.680	26.120	6.940	1.00	0.00
ATOM	3818	HW2	SOL	898	38.960	26.490	8.220	1.00	0.00
ATOM	3819	OW	SOL	899	14.700	29.470	29.860	1.00	0.00
ATOM	3820	HW1	SOL	899	13.800	29.780	29.940	1.00	0.00
ATOM	3821	HW2	SOL	899	14.620	28.580	29.520	1.00	0.00
ATOM	3822	OW	SOL	900	34.290	8.720	10.860	1.00	0.00
ATOM	3823	HW1	SOL	900	35.180	8.820	11.200	1.00	0.00
ATOM	3824	HW2	SOL	900	34.410	8.500	9.930	1.00	0.00
ATOM	3825	OW	SOL	901	9.600	41.080	33.900	1.00	0.00
ATOM	3826	HW1	SOL	901	9.700	41.090	34.850	1.00	0.00
ATOM	3827	HW2	SOL	901	10.490	40.960	33.560	1.00	0.00

ATOM	3828	OW	SOL	902	29.720	28.640	3.650	1.00	0.00
ATOM	3829	HW1	SOL	902	28.840	28.310	3.850	1.00	0.00
ATOM	3830	HW2	SOL	902	29.730	28.770	2.710	1.00	0.00
ATOM	3831	OW	SOL	903	43.900	50.630	50.190	1.00	0.00
ATOM	3832	HW1	SOL	903	43.810	51.310	50.860	1.00	0.00
ATOM	3833	HW2	SOL	903	43.080	50.670	49.700	1.00	0.00
ATOM	3834	OW	SOL	904	34.930	9.950	35.750	1.00	0.00
ATOM	3835	HW1	SOL	904	35.770	9.520	35.600	1.00	0.00
ATOM	3836	HW2	SOL	904	34.580	9.530	36.530	1.00	0.00
ATOM	3837	OW	SOL	905	19.570	31.370	39.060	1.00	0.00
ATOM	3838	HW1	SOL	905	20.050	31.890	39.710	1.00	0.00
ATOM	3839	HW2	SOL	905	20.150	30.620	38.880	1.00	0.00
ATOM	3840	OW	SOL	906	20.160	51.790	20.160	1.00	0.00
ATOM	3841	HW1	SOL	906	20.950	51.300	20.390	1.00	0.00
ATOM	3842	HW2	SOL	906	19.660	51.200	19.590	1.00	0.00
ATOM	3843	OW	SOL	907	28.030	0.970	0.370	1.00	0.00
ATOM	3844	HW1	SOL	907	27.080	1.070	0.380	1.00	0.00
ATOM	3845	HW2	SOL	907	28.220	0.390	1.110	1.00	0.00
ATOM	3846	OW	SOL	908	45.170	3.610	19.390	1.00	0.00
ATOM	3847	HW1	SOL	908	46.120	3.510	19.350	1.00	0.00
ATOM	3848	HW2	SOL	908	44.830	2.720	19.250	1.00	0.00
ATOM	3849	OW	SOL	909	41.440	13.110	21.900	1.00	0.00
ATOM	3850	HW1	SOL	909	41.460	13.300	20.960	1.00	0.00
ATOM	3851	HW2	SOL	909	40.540	12.830	22.070	1.00	0.00
ATOM	3852	OW	SOL	910	12.940	49.700	10.730	1.00	0.00
ATOM	3853	HW1	SOL	910	12.670	48.850	10.380	1.00	0.00
ATOM	3854	HW2	SOL	910	12.150	50.230	10.710	1.00	0.00
ATOM	3855	OW	SOL	911	32.010	46.840	13.760	1.00	0.00
ATOM	3856	HW1	SOL	911	31.670	47.200	14.580	1.00	0.00
ATOM	3857	HW2	SOL	911	32.430	46.020	14.010	1.00	0.00
ATOM	3858	OW	SOL	912	25.520	23.360	10.020	1.00	0.00
ATOM	3859	HW1	SOL	912	26.200	23.750	9.470	1.00	0.00
ATOM	3860	HW2	SOL	912	26.000	22.810	10.640	1.00	0.00
ATOM	3861	OW	SOL	913	3.540	44.830	15.600	1.00	0.00
ATOM	3862	HW1	SOL	913	3.080	44.740	16.440	1.00	0.00
ATOM	3863	HW2	SOL	913	4.370	45.230	15.830	1.00	0.00
ATOM	3864	OW	SOL	914	13.190	20.030	6.170	1.00	0.00
ATOM	3865	HW1	SOL	914	12.960	20.520	6.950	1.00	0.00
ATOM	3866	HW2	SOL	914	12.450	19.450	6.020	1.00	0.00
ATOM	3867	OW	SOL	915	42.680	3.780	26.850	1.00	0.00
ATOM	3868	HW1	SOL	915	43.490	4.280	26.840	1.00	0.00
ATOM	3869	HW2	SOL	915	42.080	4.280	26.290	1.00	0.00
ATOM	3870	OW	SOL	916	55.120	7.440	11.870	1.00	0.00
ATOM	3871	HW1	SOL	916	54.310	7.020	12.130	1.00	0.00

ATOM	3872	HW2	SOL	916	55.260	8.120	12.520	1.00	0.00
ATOM	3873	OW	SOL	917	19.540	37.840	19.540	1.00	0.00
ATOM	3874	HW1	SOL	917	19.390	38.770	19.360	1.00	0.00
ATOM	3875	HW2	SOL	917	19.320	37.400	18.720	1.00	0.00
ATOM	3876	OW	SOL	918	28.110	44.820	24.120	1.00	0.00
ATOM	3877	HW1	SOL	918	28.640	45.540	23.780	1.00	0.00
ATOM	3878	HW2	SOL	918	28.740	44.120	24.270	1.00	0.00
ATOM	3879	OW	SOL	919	4.260	50.910	9.020	1.00	0.00
ATOM	3880	HW1	SOL	919	3.360	50.900	8.720	1.00	0.00
ATOM	3881	HW2	SOL	919	4.730	50.350	8.400	1.00	0.00
ATOM	3882	OW	SOL	920	54.120	41.780	34.260	1.00	0.00
ATOM	3883	HW1	SOL	920	54.280	40.840	34.270	1.00	0.00
ATOM	3884	HW2	SOL	920	54.680	42.130	34.940	1.00	0.00
ATOM	3885	OW	SOL	921	32.280	44.220	16.950	1.00	0.00
ATOM	3886	HW1	SOL	921	32.840	44.540	16.240	1.00	0.00
ATOM	3887	HW2	SOL	921	31.640	43.660	16.500	1.00	0.00
ATOM	3888	OW	SOL	922	15.370	42.580	14.290	1.00	0.00
ATOM	3889	HW1	SOL	922	14.750	42.400	15.000	1.00	0.00
ATOM	3890	HW2	SOL	922	15.000	43.340	13.840	1.00	0.00
ATOM	3891	OW	SOL	923	42.760	7.170	12.370	1.00	0.00
ATOM	3892	HW1	SOL	923	43.470	7.700	12.020	1.00	0.00
ATOM	3893	HW2	SOL	923	43.100	6.270	12.340	1.00	0.00
ATOM	3894	OW	SOL	924	23.140	54.420	11.780	1.00	0.00
ATOM	3895	HW1	SOL	924	22.510	55.070	12.110	1.00	0.00
ATOM	3896	HW2	SOL	924	22.590	53.760	11.360	1.00	0.00
ATOM	3897	OW	SOL	925	50.830	5.490	48.430	1.00	0.00
ATOM	3898	HW1	SOL	925	51.740	5.570	48.720	1.00	0.00
ATOM	3899	HW2	SOL	925	50.840	4.730	47.850	1.00	0.00
ATOM	3900	OW	SOL	926	18.990	51.280	2.130	1.00	0.00
ATOM	3901	HW1	SOL	926	19.870	50.950	2.290	1.00	0.00
ATOM	3902	HW2	SOL	926	18.830	51.090	1.200	1.00	0.00
ATOM	3903	OW	SOL	927	35.040	41.340	13.180	1.00	0.00
ATOM	3904	HW1	SOL	927	34.840	41.520	14.100	1.00	0.00
ATOM	3905	HW2	SOL	927	34.190	41.390	12.740	1.00	0.00
ATOM	3906	OW	SOL	928	6.230	51.180	4.270	1.00	0.00
ATOM	3907	HW1	SOL	928	6.890	50.570	3.940	1.00	0.00
ATOM	3908	HW2	SOL	928	6.730	51.870	4.710	1.00	0.00
ATOM	3909	OW	SOL	929	15.860	10.140	24.420	1.00	0.00
ATOM	3910	HW1	SOL	929	16.080	9.330	23.950	1.00	0.00
ATOM	3911	HW2	SOL	929	15.070	10.450	23.990	1.00	0.00
ATOM	3912	OW	SOL	930	33.620	16.330	44.110	1.00	0.00
ATOM	3913	HW1	SOL	930	34.350	15.720	44.130	1.00	0.00
ATOM	3914	HW2	SOL	930	32.860	15.790	44.340	1.00	0.00
ATOM	3915	OW	SOL	931	3.980	2.980	2.130	1.00	0.00

ATOM	3916	HW1	SOL	931	3.590	3.640	1.560	1.00	0.00
ATOM	3917	HW2	SOL	931	4.700	3.440	2.570	1.00	0.00
ATOM	3918	OW	SOL	932	28.220	11.240	21.590	1.00	0.00
ATOM	3919	HW1	SOL	932	27.860	12.070	21.290	1.00	0.00
ATOM	3920	HW2	SOL	932	28.570	11.430	22.470	1.00	0.00
ATOM	3921	OW	SOL	933	4.750	3.260	14.450	1.00	0.00
ATOM	3922	HW1	SOL	933	4.800	4.220	14.450	1.00	0.00
ATOM	3923	HW2	SOL	933	5.390	2.990	13.800	1.00	0.00
ATOM	3924	OW	SOL	934	52.330	28.130	10.820	1.00	0.00
ATOM	3925	HW1	SOL	934	51.980	27.850	11.670	1.00	0.00
ATOM	3926	HW2	SOL	934	53.230	27.820	10.820	1.00	0.00
ATOM	3927	OW	SOL	935	53.100	47.370	31.470	1.00	0.00
ATOM	3928	HW1	SOL	935	52.430	47.900	31.040	1.00	0.00
ATOM	3929	HW2	SOL	935	53.160	46.580	30.940	1.00	0.00
ATOM	3930	OW	SOL	936	22.410	1.830	21.660	1.00	0.00
ATOM	3931	HW1	SOL	936	22.930	1.190	21.180	1.00	0.00
ATOM	3932	HW2	SOL	936	22.060	1.350	22.410	1.00	0.00
ATOM	3933	OW	SOL	937	11.540	25.070	33.040	1.00	0.00
ATOM	3934	HW1	SOL	937	11.650	24.220	33.480	1.00	0.00
ATOM	3935	HW2	SOL	937	12.330	25.170	32.520	1.00	0.00
ATOM	3936	OW	SOL	938	1.690	46.670	27.330	1.00	0.00
ATOM	3937	HW1	SOL	938	2.630	46.630	27.470	1.00	0.00
ATOM	3938	HW2	SOL	938	1.370	45.790	27.520	1.00	0.00
ATOM	3939	OW	SOL	939	36.690	24.090	39.570	1.00	0.00
ATOM	3940	HW1	SOL	939	37.140	23.700	38.820	1.00	0.00
ATOM	3941	HW2	SOL	939	37.310	24.000	40.300	1.00	0.00
ATOM	3942	OW	SOL	940	6.260	52.610	6.640	1.00	0.00
ATOM	3943	HW1	SOL	940	6.500	53.510	6.850	1.00	0.00
ATOM	3944	HW2	SOL	940	5.360	52.670	6.330	1.00	0.00
ATOM	3945	OW	SOL	941	24.570	6.000	6.770	1.00	0.00
ATOM	3946	HW1	SOL	941	24.230	6.870	6.550	1.00	0.00
ATOM	3947	HW2	SOL	941	25.520	6.090	6.700	1.00	0.00
ATOM	3948	OW	SOL	942	28.270	54.110	52.400	1.00	0.00
ATOM	3949	HW1	SOL	942	29.070	54.620	52.460	1.00	0.00
ATOM	3950	HW2	SOL	942	27.890	54.330	51.550	1.00	0.00
ATOM	3951	OW	SOL	943	34.880	50.070	25.650	1.00	0.00
ATOM	3952	HW1	SOL	943	34.980	49.260	25.150	1.00	0.00
ATOM	3953	HW2	SOL	943	34.210	49.870	26.300	1.00	0.00
ATOM	3954	OW	SOL	944	4.650	22.130	54.260	1.00	0.00
ATOM	3955	HW1	SOL	944	5.080	22.780	53.710	1.00	0.00
ATOM	3956	HW2	SOL	944	4.420	22.600	55.060	1.00	0.00
ATOM	3957	OW	SOL	945	10.290	20.000	52.870	1.00	0.00
ATOM	3958	HW1	SOL	945	11.110	19.690	53.260	1.00	0.00
ATOM	3959	HW2	SOL	945	10.010	19.280	52.310	1.00	0.00

ATOM	3960	OW	SOL	946	9.020	12.390	44.340	1.00	0.00
ATOM	3961	HW1	SOL	946	8.860	11.450	44.400	1.00	0.00
ATOM	3962	HW2	SOL	946	8.180	12.750	44.050	1.00	0.00
ATOM	3963	OW	SOL	947	23.150	17.000	25.940	1.00	0.00
ATOM	3964	HW1	SOL	947	23.210	17.900	26.250	1.00	0.00
ATOM	3965	HW2	SOL	947	22.290	16.700	26.230	1.00	0.00
ATOM	3966	OW	SOL	948	30.040	33.660	47.660	1.00	0.00
ATOM	3967	HW1	SOL	948	29.870	33.290	48.520	1.00	0.00
ATOM	3968	HW2	SOL	948	30.120	32.890	47.080	1.00	0.00
ATOM	3969	OW	SOL	949	46.610	8.410	24.970	1.00	0.00
ATOM	3970	HW1	SOL	949	46.340	8.500	25.890	1.00	0.00
ATOM	3971	HW2	SOL	949	45.960	8.910	24.480	1.00	0.00
ATOM	3972	OW	SOL	950	27.370	33.810	6.810	1.00	0.00
ATOM	3973	HW1	SOL	950	26.560	33.810	6.300	1.00	0.00
ATOM	3974	HW2	SOL	950	27.860	33.060	6.460	1.00	0.00
ATOM	3975	OW	SOL	951	26.160	18.140	18.220	1.00	0.00
ATOM	3976	HW1	SOL	951	26.880	17.610	17.890	1.00	0.00
ATOM	3977	HW2	SOL	951	25.600	18.290	17.450	1.00	0.00
ATOM	3978	OW	SOL	952	52.830	36.950	50.560	1.00	0.00
ATOM	3979	HW1	SOL	952	53.610	36.570	50.150	1.00	0.00
ATOM	3980	HW2	SOL	952	52.150	36.300	50.430	1.00	0.00
ATOM	3981	OW	SOL	953	38.800	26.930	47.480	1.00	0.00
ATOM	3982	HW1	SOL	953	39.600	26.930	48.000	1.00	0.00
ATOM	3983	HW2	SOL	953	38.810	26.080	47.020	1.00	0.00
ATOM	3984	OW	SOL	954	1.650	4.250	49.560	1.00	0.00
ATOM	3985	HW1	SOL	954	1.380	3.350	49.710	1.00	0.00
ATOM	3986	HW2	SOL	954	1.410	4.430	48.650	1.00	0.00
ATOM	3987	OW	SOL	955	9.090	22.750	12.320	1.00	0.00
ATOM	3988	HW1	SOL	955	8.180	22.920	12.110	1.00	0.00
ATOM	3989	HW2	SOL	955	9.390	23.550	12.770	1.00	0.00
ATOM	3990	OW	SOL	956	50.030	9.770	16.190	1.00	0.00
ATOM	3991	HW1	SOL	956	50.840	10.030	16.640	1.00	0.00
ATOM	3992	HW2	SOL	956	49.460	9.470	16.900	1.00	0.00
ATOM	3993	OW	SOL	957	0.220	26.260	7.610	1.00	0.00
ATOM	3994	HW1	SOL	957	-0.540	26.840	7.620	1.00	0.00
ATOM	3995	HW2	SOL	957	-0.040	25.520	8.160	1.00	0.00
ATOM	3996	OW	SOL	958	55.460	54.670	35.180	1.00	0.00
ATOM	3997	HW1	SOL	958	55.360	55.350	34.520	1.00	0.00
ATOM	3998	HW2	SOL	958	55.740	53.900	34.680	1.00	0.00
ATOM	3999	OW	SOL	959	1.030	40.640	49.960	1.00	0.00
ATOM	4000	HW1	SOL	959	0.690	40.320	49.130	1.00	0.00
ATOM	4001	HW2	SOL	959	1.600	41.370	49.720	1.00	0.00
ATOM	4002	OW	SOL	960	25.360	7.890	21.040	1.00	0.00
ATOM	4003	HW1	SOL	960	26.250	7.910	21.380	1.00	0.00

ATOM	4004	HW2	SOL	960	25.000	8.760	21.240	1.00	0.00
ATOM	4005	OW	SOL	961	36.730	33.720	14.460	1.00	0.00
ATOM	4006	HW1	SOL	961	37.570	33.800	14.910	1.00	0.00
ATOM	4007	HW2	SOL	961	36.750	34.410	13.800	1.00	0.00
ATOM	4008	OW	SOL	962	13.270	37.640	7.750	1.00	0.00
ATOM	4009	HW1	SOL	962	13.070	36.750	7.470	1.00	0.00
ATOM	4010	HW2	SOL	962	14.200	37.620	7.950	1.00	0.00
ATOM	4011	OW	SOL	963	40.070	39.940	5.850	1.00	0.00
ATOM	4012	HW1	SOL	963	40.790	39.820	6.460	1.00	0.00
ATOM	4013	HW2	SOL	963	40.360	39.500	5.050	1.00	0.00
ATOM	4014	OW	SOL	964	11.080	31.210	35.900	1.00	0.00
ATOM	4015	HW1	SOL	964	11.860	31.340	35.360	1.00	0.00
ATOM	4016	HW2	SOL	964	10.460	31.880	35.590	1.00	0.00
ATOM	4017	OW	SOL	965	15.110	5.840	25.610	1.00	0.00
ATOM	4018	HW1	SOL	965	14.720	5.320	26.320	1.00	0.00
ATOM	4019	HW2	SOL	965	15.580	6.550	26.050	1.00	0.00
ATOM	4020	OW	SOL	966	7.890	52.620	50.590	1.00	0.00
ATOM	4021	HW1	SOL	966	8.560	53.030	51.130	1.00	0.00
ATOM	4022	HW2	SOL	966	7.860	53.160	49.800	1.00	0.00
ATOM	4023	OW	SOL	967	0.050	53.070	43.700	1.00	0.00
ATOM	4024	HW1	SOL	967	0.330	52.990	44.610	1.00	0.00
ATOM	4025	HW2	SOL	967	-0.730	52.530	43.630	1.00	0.00
ATOM	4026	OW	SOL	968	1.970	4.700	1.190	1.00	0.00
ATOM	4027	HW1	SOL	968	2.110	5.540	0.740	1.00	0.00
ATOM	4028	HW2	SOL	968	1.710	4.950	2.080	1.00	0.00
ATOM	4029	OW	SOL	969	5.740	5.410	36.610	1.00	0.00
ATOM	4030	HW1	SOL	969	4.960	5.440	36.060	1.00	0.00
ATOM	4031	HW2	SOL	969	6.460	5.240	36.010	1.00	0.00
ATOM	4032	OW	SOL	970	14.530	0.100	46.550	1.00	0.00
ATOM	4033	HW1	SOL	970	15.200	0.590	46.070	1.00	0.00
ATOM	4034	HW2	SOL	970	13.970	0.770	46.940	1.00	0.00
ATOM	4035	OW	SOL	971	28.910	54.580	14.240	1.00	0.00
ATOM	4036	HW1	SOL	971	28.390	55.280	13.840	1.00	0.00
ATOM	4037	HW2	SOL	971	28.300	53.850	14.320	1.00	0.00
ATOM	4038	OW	SOL	972	40.600	31.740	14.310	1.00	0.00
ATOM	4039	HW1	SOL	972	40.210	31.170	13.660	1.00	0.00
ATOM	4040	HW2	SOL	972	39.900	31.890	14.950	1.00	0.00
ATOM	4041	OW	SOL	973	28.020	22.650	54.310	1.00	0.00
ATOM	4042	HW1	SOL	973	27.820	22.190	53.500	1.00	0.00
ATOM	4043	HW2	SOL	973	27.200	23.090	54.550	1.00	0.00
ATOM	4044	OW	SOL	974	47.700	48.250	6.890	1.00	0.00
ATOM	4045	HW1	SOL	974	48.390	48.100	7.530	1.00	0.00
ATOM	4046	HW2	SOL	974	47.540	47.390	6.510	1.00	0.00
ATOM	4047	OW	SOL	975	0.410	32.060	38.520	1.00	0.00

ATOM	4048	HW1	SOL	975	1.010	31.520	38.000	1.00	0.00
ATOM	4049	HW2	SOL	975	-0.460	31.820	38.210	1.00	0.00
ATOM	4050	OW	SOL	976	30.210	8.400	38.410	1.00	0.00
ATOM	4051	HW1	SOL	976	30.770	7.870	37.840	1.00	0.00
ATOM	4052	HW2	SOL	976	30.410	9.300	38.180	1.00	0.00
ATOM	4053	OW	SOL	977	20.370	23.940	53.100	1.00	0.00
ATOM	4054	HW1	SOL	977	19.430	23.770	52.980	1.00	0.00
ATOM	4055	HW2	SOL	977	20.770	23.070	53.090	1.00	0.00
ATOM	4056	OW	SOL	978	26.380	24.420	18.570	1.00	0.00
ATOM	4057	HW1	SOL	978	26.090	24.760	19.410	1.00	0.00
ATOM	4058	HW2	SOL	978	27.250	24.820	18.440	1.00	0.00
ATOM	4059	OW	SOL	979	48.430	35.360	16.290	1.00	0.00
ATOM	4060	HW1	SOL	979	47.630	34.960	16.640	1.00	0.00
ATOM	4061	HW2	SOL	979	48.540	34.960	15.430	1.00	0.00
ATOM	4062	OW	SOL	980	44.920	49.050	18.750	1.00	0.00
ATOM	4063	HW1	SOL	980	44.600	48.270	18.310	1.00	0.00
ATOM	4064	HW2	SOL	980	44.150	49.420	19.170	1.00	0.00
ATOM	4065	OW	SOL	981	18.800	15.250	28.590	1.00	0.00
ATOM	4066	HW1	SOL	981	17.870	15.430	28.420	1.00	0.00
ATOM	4067	HW2	SOL	981	18.830	14.330	28.840	1.00	0.00
ATOM	4068	OW	SOL	982	31.330	12.200	44.660	1.00	0.00
ATOM	4069	HW1	SOL	982	31.010	11.760	43.870	1.00	0.00
ATOM	4070	HW2	SOL	982	31.800	11.520	45.140	1.00	0.00
ATOM	4071	OW	SOL	983	46.890	36.730	1.520	1.00	0.00
ATOM	4072	HW1	SOL	983	47.350	37.280	2.160	1.00	0.00
ATOM	4073	HW2	SOL	983	47.370	35.900	1.530	1.00	0.00
ATOM	4074	OW	SOL	984	12.640	19.620	54.400	1.00	0.00
ATOM	4075	HW1	SOL	984	12.420	19.310	55.280	1.00	0.00
ATOM	4076	HW2	SOL	984	13.110	18.890	53.990	1.00	0.00
ATOM	4077	OW	SOL	985	43.640	22.930	2.110	1.00	0.00
ATOM	4078	HW1	SOL	985	43.260	22.800	1.240	1.00	0.00
ATOM	4079	HW2	SOL	985	43.870	23.860	2.130	1.00	0.00
ATOM	4080	OW	SOL	986	9.320	37.260	40.510	1.00	0.00
ATOM	4081	HW1	SOL	986	9.430	36.950	41.410	1.00	0.00
ATOM	4082	HW2	SOL	986	9.370	36.470	39.980	1.00	0.00
ATOM	4083	OW	SOL	987	52.460	38.120	38.380	1.00	0.00
ATOM	4084	HW1	SOL	987	52.010	37.960	39.210	1.00	0.00
ATOM	4085	HW2	SOL	987	53.170	37.470	38.360	1.00	0.00
ATOM	4086	OW	SOL	988	2.600	10.000	4.330	1.00	0.00
ATOM	4087	HW1	SOL	988	2.860	9.900	5.240	1.00	0.00
ATOM	4088	HW2	SOL	988	1.810	10.530	4.360	1.00	0.00
ATOM	4089	OW	SOL	989	45.570	40.210	46.070	1.00	0.00
ATOM	4090	HW1	SOL	989	46.170	40.700	46.630	1.00	0.00
ATOM	4091	HW2	SOL	989	46.070	39.430	45.820	1.00	0.00

ATOM	4092	OW	SOL	990	30.610	24.840	0.830	1.00	0.00
ATOM	4093	HW1	SOL	990	31.180	25.300	1.440	1.00	0.00
ATOM	4094	HW2	SOL	990	31.080	24.860	-0.010	1.00	0.00
ATOM	4095	OW	SOL	991	4.830	53.070	30.230	1.00	0.00
ATOM	4096	HW1	SOL	991	3.970	52.650	30.240	1.00	0.00
ATOM	4097	HW2	SOL	991	5.420	52.430	30.610	1.00	0.00
ATOM	4098	OW	SOL	992	44.290	5.560	33.890	1.00	0.00
ATOM	4099	HW1	SOL	992	45.060	6.100	34.090	1.00	0.00
ATOM	4100	HW2	SOL	992	43.540	6.090	34.150	1.00	0.00
ATOM	4101	OW	SOL	993	17.730	32.050	4.210	1.00	0.00
ATOM	4102	HW1	SOL	993	17.160	32.580	4.760	1.00	0.00
ATOM	4103	HW2	SOL	993	17.740	31.190	4.640	1.00	0.00
ATOM	4104	OW	SOL	994	9.660	24.450	19.350	1.00	0.00
ATOM	4105	HW1	SOL	994	9.330	23.580	19.610	1.00	0.00
ATOM	4106	HW2	SOL	994	9.420	24.530	18.430	1.00	0.00
ATOM	4107	OW	SOL	995	22.690	8.760	53.070	1.00	0.00
ATOM	4108	HW1	SOL	995	23.170	9.120	52.320	1.00	0.00
ATOM	4109	HW2	SOL	995	22.900	7.830	53.060	1.00	0.00
ATOM	4110	OW	SOL	996	39.790	49.030	14.460	1.00	0.00
ATOM	4111	HW1	SOL	996	39.020	48.590	14.090	1.00	0.00
ATOM	4112	HW2	SOL	996	39.590	49.960	14.390	1.00	0.00
ATOM	4113	OW	SOL	997	27.650	7.610	29.080	1.00	0.00
ATOM	4114	HW1	SOL	997	26.950	7.160	28.610	1.00	0.00
ATOM	4115	HW2	SOL	997	28.460	7.220	28.770	1.00	0.00
ATOM	4116	OW	SOL	998	54.690	52.130	14.910	1.00	0.00
ATOM	4117	HW1	SOL	998	55.330	52.580	14.360	1.00	0.00
ATOM	4118	HW2	SOL	998	55.000	52.280	15.800	1.00	0.00
ATOM	4119	OW	SOL	999	35.450	52.270	8.680	1.00	0.00
ATOM	4120	HW1	SOL	999	35.310	52.920	9.370	1.00	0.00
ATOM	4121	HW2	SOL	999	34.570	52.090	8.340	1.00	0.00
ATOM	4122	OW	SOL	1000	54.420	46.030	2.750	1.00	0.00
ATOM	4123	HW1	SOL	1000	53.630	46.020	3.290	1.00	0.00
ATOM	4124	HW2	SOL	1000	55.100	45.670	3.310	1.00	0.00
ATOM	4125	OW	SOL	1001	7.550	19.300	17.050	1.00	0.00
ATOM	4126	HW1	SOL	1001	7.670	20.110	16.550	1.00	0.00
ATOM	4127	HW2	SOL	1001	7.720	18.610	16.410	1.00	0.00
ATOM	4128	OW	SOL	1002	28.760	39.860	8.420	1.00	0.00
ATOM	4129	HW1	SOL	1002	28.950	40.410	7.650	1.00	0.00
ATOM	4130	HW2	SOL	1002	28.630	38.980	8.060	1.00	0.00
ATOM	4131	OW	SOL	1003	18.570	21.920	10.090	1.00	0.00
ATOM	4132	HW1	SOL	1003	19.070	22.470	10.690	1.00	0.00
ATOM	4133	HW2	SOL	1003	19.230	21.340	9.700	1.00	0.00
ATOM	4134	OW	SOL	1004	24.250	47.950	3.480	1.00	0.00
ATOM	4135	HW1	SOL	1004	24.550	47.810	2.580	1.00	0.00

ATOM	4136	HW2	SOL	1004	25.050	47.940	4.010	1.00	0.00
ATOM	4137	OW	SOL	1005	26.350	12.560	29.360	1.00	0.00
ATOM	4138	HW1	SOL	1005	26.880	11.850	29.010	1.00	0.00
ATOM	4139	HW2	SOL	1005	26.980	13.180	29.730	1.00	0.00
ATOM	4140	OW	SOL	1006	41.290	16.960	41.050	1.00	0.00
ATOM	4141	HW1	SOL	1006	40.470	17.360	41.330	1.00	0.00
ATOM	4142	HW2	SOL	1006	41.970	17.500	41.460	1.00	0.00
ATOM	4143	OW	SOL	1007	25.960	22.450	36.090	1.00	0.00
ATOM	4144	HW1	SOL	1007	26.300	23.200	36.580	1.00	0.00
ATOM	4145	HW2	SOL	1007	26.740	22.060	35.680	1.00	0.00
ATOM	4146	OW	SOL	1008	38.370	21.800	52.570	1.00	0.00
ATOM	4147	HW1	SOL	1008	38.610	21.030	52.060	1.00	0.00
ATOM	4148	HW2	SOL	1008	37.420	21.750	52.660	1.00	0.00
ATOM	4149	OW	SOL	1009	34.830	33.870	5.150	1.00	0.00
ATOM	4150	HW1	SOL	1009	34.100	33.600	4.600	1.00	0.00
ATOM	4151	HW2	SOL	1009	35.360	34.440	4.590	1.00	0.00
ATOM	4152	OW	SOL	1010	5.900	18.500	51.600	1.00	0.00
ATOM	4153	HW1	SOL	1010	5.730	18.890	52.460	1.00	0.00
ATOM	4154	HW2	SOL	1010	6.080	17.580	51.780	1.00	0.00
ATOM	4155	OW	SOL	1011	52.520	8.010	30.350	1.00	0.00
ATOM	4156	HW1	SOL	1011	52.570	8.440	29.490	1.00	0.00
ATOM	4157	HW2	SOL	1011	52.960	8.630	30.940	1.00	0.00
ATOM	4158	OW	SOL	1012	51.770	29.910	3.070	1.00	0.00
ATOM	4159	HW1	SOL	1012	51.110	29.880	3.760	1.00	0.00
ATOM	4160	HW2	SOL	1012	52.410	30.550	3.390	1.00	0.00
ATOM	4161	OW	SOL	1013	42.270	38.820	17.100	1.00	0.00
ATOM	4162	HW1	SOL	1013	41.670	38.150	16.780	1.00	0.00
ATOM	4163	HW2	SOL	1013	42.700	39.160	16.320	1.00	0.00
ATOM	4164	OW	SOL	1014	43.990	43.890	10.280	1.00	0.00
ATOM	4165	HW1	SOL	1014	43.990	43.010	10.640	1.00	0.00
ATOM	4166	HW2	SOL	1014	44.880	44.010	9.950	1.00	0.00
ATOM	4167	OW	SOL	1015	2.320	38.530	0.710	1.00	0.00
ATOM	4168	HW1	SOL	1015	1.480	38.140	0.470	1.00	0.00
ATOM	4169	HW2	SOL	1015	2.170	39.470	0.690	1.00	0.00
ATOM	4170	OW	SOL	1016	3.930	3.600	51.880	1.00	0.00
ATOM	4171	HW1	SOL	1016	4.060	4.210	51.160	1.00	0.00
ATOM	4172	HW2	SOL	1016	3.000	3.380	51.850	1.00	0.00
ATOM	4173	OW	SOL	1017	18.370	29.500	5.840	1.00	0.00
ATOM	4174	HW1	SOL	1017	17.730	28.990	5.340	1.00	0.00
ATOM	4175	HW2	SOL	1017	17.970	29.590	6.700	1.00	0.00
ATOM	4176	OW	SOL	1018	11.310	11.240	25.110	1.00	0.00
ATOM	4177	HW1	SOL	1018	11.770	10.750	25.790	1.00	0.00
ATOM	4178	HW2	SOL	1018	10.840	11.920	25.590	1.00	0.00
ATOM	4179	OW	SOL	1019	30.350	3.940	35.550	1.00	0.00

ATOM	4180	HW1	SOL	1019	29.920	4.670	35.100	1.00	0.00
ATOM	4181	HW2	SOL	1019	31.230	3.900	35.160	1.00	0.00
ATOM	4182	OW	SOL	1020	3.080	15.860	38.080	1.00	0.00
ATOM	4183	HW1	SOL	1020	3.950	15.490	38.160	1.00	0.00
ATOM	4184	HW2	SOL	1020	3.220	16.810	38.030	1.00	0.00
ATOM	4185	OW	SOL	1021	52.340	3.400	30.050	1.00	0.00
ATOM	4186	HW1	SOL	1021	52.470	2.980	29.200	1.00	0.00
ATOM	4187	HW2	SOL	1021	53.130	3.920	30.180	1.00	0.00
ATOM	4188	OW	SOL	1022	55.530	7.880	47.400	1.00	0.00
ATOM	4189	HW1	SOL	1022	56.280	8.100	46.850	1.00	0.00
ATOM	4190	HW2	SOL	1022	54.770	8.000	46.830	1.00	0.00
ATOM	4191	OW	SOL	1023	23.960	53.880	21.470	1.00	0.00
ATOM	4192	HW1	SOL	1023	24.610	53.640	22.130	1.00	0.00
ATOM	4193	HW2	SOL	1023	23.120	53.600	21.840	1.00	0.00
ATOM	4194	OW	SOL	1024	17.030	1.900	32.020	1.00	0.00
ATOM	4195	HW1	SOL	1024	16.830	2.780	31.700	1.00	0.00
ATOM	4196	HW2	SOL	1024	17.960	1.790	31.840	1.00	0.00
ATOM	4197	OW	SOL	1025	37.450	20.760	11.720	1.00	0.00
ATOM	4198	HW1	SOL	1025	37.920	20.740	10.880	1.00	0.00
ATOM	4199	HW2	SOL	1025	36.590	21.100	11.510	1.00	0.00
ATOM	4200	OW	SOL	1026	45.510	18.300	13.940	1.00	0.00
ATOM	4201	HW1	SOL	1026	45.750	18.390	14.860	1.00	0.00
ATOM	4202	HW2	SOL	1026	46.210	18.740	13.460	1.00	0.00
ATOM	4203	OW	SOL	1027	29.020	49.160	48.610	1.00	0.00
ATOM	4204	HW1	SOL	1027	28.310	48.520	48.620	1.00	0.00
ATOM	4205	HW2	SOL	1027	29.010	49.520	47.720	1.00	0.00
ATOM	4206	OW	SOL	1028	47.780	52.990	50.630	1.00	0.00
ATOM	4207	HW1	SOL	1028	48.090	52.490	49.880	1.00	0.00
ATOM	4208	HW2	SOL	1028	47.910	52.410	51.380	1.00	0.00
ATOM	4209	OW	SOL	1029	11.730	22.260	43.650	1.00	0.00
ATOM	4210	HW1	SOL	1029	11.750	22.550	42.740	1.00	0.00
ATOM	4211	HW2	SOL	1029	11.970	23.040	44.150	1.00	0.00
ATOM	4212	OW	SOL	1030	14.980	23.030	36.710	1.00	0.00
ATOM	4213	HW1	SOL	1030	15.620	22.580	37.260	1.00	0.00
ATOM	4214	HW2	SOL	1030	14.230	23.170	37.280	1.00	0.00
ATOM	4215	OW	SOL	1031	42.140	26.660	22.470	1.00	0.00
ATOM	4216	HW1	SOL	1031	42.480	27.390	22.990	1.00	0.00
ATOM	4217	HW2	SOL	1031	41.960	25.980	23.120	1.00	0.00
ATOM	4218	OW	SOL	1032	22.900	32.500	14.730	1.00	0.00
ATOM	4219	HW1	SOL	1032	22.500	32.910	15.490	1.00	0.00
ATOM	4220	HW2	SOL	1032	22.210	32.520	14.060	1.00	0.00
ATOM	4221	OW	SOL	1033	3.600	40.980	42.920	1.00	0.00
ATOM	4222	HW1	SOL	1033	4.090	41.400	42.210	1.00	0.00
ATOM	4223	HW2	SOL	1033	2.850	41.560	43.070	1.00	0.00

ATOM	4224	OW	SOL	1034	22.780	16.940	30.990	1.00	0.00
ATOM	4225	HW1	SOL	1034	23.360	16.220	30.720	1.00	0.00
ATOM	4226	HW2	SOL	1034	22.510	17.340	30.170	1.00	0.00
ATOM	4227	OW	SOL	1035	41.660	20.400	17.910	1.00	0.00
ATOM	4228	HW1	SOL	1035	41.460	20.610	18.830	1.00	0.00
ATOM	4229	HW2	SOL	1035	41.110	19.650	17.720	1.00	0.00
ATOM	4230	OW	SOL	1036	16.780	36.140	34.180	1.00	0.00
ATOM	4231	HW1	SOL	1036	16.640	35.800	33.300	1.00	0.00
ATOM	4232	HW2	SOL	1036	16.680	37.090	34.090	1.00	0.00
ATOM	4233	OW	SOL	1037	16.410	46.130	28.320	1.00	0.00
ATOM	4234	HW1	SOL	1037	15.800	45.690	27.730	1.00	0.00
ATOM	4235	HW2	SOL	1037	16.120	45.860	29.200	1.00	0.00
ATOM	4236	OW	SOL	1038	10.810	2.790	30.230	1.00	0.00
ATOM	4237	HW1	SOL	1038	11.510	3.420	30.060	1.00	0.00
ATOM	4238	HW2	SOL	1038	10.820	2.210	29.470	1.00	0.00
ATOM	4239	OW	SOL	1039	49.220	8.490	18.790	1.00	0.00
ATOM	4240	HW1	SOL	1039	49.700	7.700	19.040	1.00	0.00
ATOM	4241	HW2	SOL	1039	49.800	9.210	19.040	1.00	0.00
ATOM	4242	OW	SOL	1040	12.580	44.780	17.310	1.00	0.00
ATOM	4243	HW1	SOL	1040	13.380	44.640	17.810	1.00	0.00
ATOM	4244	HW2	SOL	1040	12.400	43.940	16.890	1.00	0.00
ATOM	4245	OW	SOL	1041	1.360	54.480	23.280	1.00	0.00
ATOM	4246	HW1	SOL	1041	0.950	54.830	24.070	1.00	0.00
ATOM	4247	HW2	SOL	1041	0.750	53.820	22.960	1.00	0.00
ATOM	4248	OW	SOL	1042	15.060	49.330	42.600	1.00	0.00
ATOM	4249	HW1	SOL	1042	14.500	49.610	43.320	1.00	0.00
ATOM	4250	HW2	SOL	1042	15.110	50.090	42.030	1.00	0.00
ATOM	4251	OW	SOL	1043	39.270	11.890	18.650	1.00	0.00
ATOM	4252	HW1	SOL	1043	39.290	12.510	19.380	1.00	0.00
ATOM	4253	HW2	SOL	1043	38.820	11.120	18.990	1.00	0.00
ATOM	4254	OW	SOL	1044	0.930	14.950	55.090	1.00	0.00
ATOM	4255	HW1	SOL	1044	0.500	14.460	54.390	1.00	0.00
ATOM	4256	HW2	SOL	1044	1.660	14.390	55.350	1.00	0.00
ATOM	4257	OW	SOL	1045	38.330	10.670	31.840	1.00	0.00
ATOM	4258	HW1	SOL	1045	38.690	9.810	32.050	1.00	0.00
ATOM	4259	HW2	SOL	1045	38.720	11.260	32.490	1.00	0.00
ATOM	4260	OW	SOL	1046	4.720	13.770	5.170	1.00	0.00
ATOM	4261	HW1	SOL	1046	4.900	13.480	6.060	1.00	0.00
ATOM	4262	HW2	SOL	1046	4.940	13.020	4.620	1.00	0.00
ATOM	4263	OW	SOL	1047	37.800	49.500	37.440	1.00	0.00
ATOM	4264	HW1	SOL	1047	37.850	48.600	37.130	1.00	0.00
ATOM	4265	HW2	SOL	1047	37.410	49.440	38.310	1.00	0.00
ATOM	4266	OW	SOL	1048	50.690	43.720	2.000	1.00	0.00
ATOM	4267	HW1	SOL	1048	51.590	43.440	2.160	1.00	0.00

ATOM	4268	HW2	SOL	1048	50.720	44.680	2.070	1.00	0.00
ATOM	4269	OW	SOL	1049	21.310	16.410	43.840	1.00	0.00
ATOM	4270	HW1	SOL	1049	20.750	17.110	43.530	1.00	0.00
ATOM	4271	HW2	SOL	1049	21.610	15.970	43.040	1.00	0.00
ATOM	4272	OW	SOL	1050	5.700	50.130	40.910	1.00	0.00
ATOM	4273	HW1	SOL	1050	5.030	50.370	40.270	1.00	0.00
ATOM	4274	HW2	SOL	1050	5.480	49.230	41.160	1.00	0.00
ATOM	4275	OW	SOL	1051	51.260	7.510	41.150	1.00	0.00
ATOM	4276	HW1	SOL	1051	51.710	6.700	40.920	1.00	0.00
ATOM	4277	HW2	SOL	1051	51.960	8.170	41.160	1.00	0.00
ATOM	4278	OW	SOL	1052	53.210	16.880	9.230	1.00	0.00
ATOM	4279	HW1	SOL	1052	53.140	17.710	8.760	1.00	0.00
ATOM	4280	HW2	SOL	1052	53.870	16.380	8.740	1.00	0.00
ATOM	4281	OW	SOL	1053	3.970	6.620	5.300	1.00	0.00
ATOM	4282	HW1	SOL	1053	3.240	7.020	5.760	1.00	0.00
ATOM	4283	HW2	SOL	1053	4.050	5.750	5.690	1.00	0.00
ATOM	4284	OW	SOL	1054	24.680	22.100	18.070	1.00	0.00
ATOM	4285	HW1	SOL	1054	25.360	22.590	18.530	1.00	0.00
ATOM	4286	HW2	SOL	1054	24.580	22.570	17.230	1.00	0.00
ATOM	4287	OW	SOL	1055	4.780	50.020	31.620	1.00	0.00
ATOM	4288	HW1	SOL	1055	4.160	49.430	32.050	1.00	0.00
ATOM	4289	HW2	SOL	1055	4.710	49.810	30.690	1.00	0.00
ATOM	4290	OW	SOL	1056	21.690	49.660	24.870	1.00	0.00
ATOM	4291	HW1	SOL	1056	21.980	50.300	24.220	1.00	0.00
ATOM	4292	HW2	SOL	1056	22.490	49.420	25.340	1.00	0.00
ATOM	4293	OW	SOL	1057	9.910	48.700	21.630	1.00	0.00
ATOM	4294	HW1	SOL	1057	9.710	49.620	21.790	1.00	0.00
ATOM	4295	HW2	SOL	1057	10.540	48.460	22.310	1.00	0.00
ATOM	4296	OW	SOL	1058	3.590	20.110	50.170	1.00	0.00
ATOM	4297	HW1	SOL	1058	3.640	20.680	49.400	1.00	0.00
ATOM	4298	HW2	SOL	1058	4.260	19.450	50.020	1.00	0.00
ATOM	4299	OW	SOL	1059	39.320	52.890	5.010	1.00	0.00
ATOM	4300	HW1	SOL	1059	38.810	52.940	5.810	1.00	0.00
ATOM	4301	HW2	SOL	1059	38.670	52.970	4.310	1.00	0.00
ATOM	4302	OW	SOL	1060	30.070	22.820	30.600	1.00	0.00
ATOM	4303	HW1	SOL	1060	29.720	22.810	29.710	1.00	0.00
ATOM	4304	HW2	SOL	1060	31.020	22.840	30.500	1.00	0.00
ATOM	4305	OW	SOL	1061	33.750	14.410	10.910	1.00	0.00
ATOM	4306	HW1	SOL	1061	33.830	13.610	11.430	1.00	0.00
ATOM	4307	HW2	SOL	1061	34.320	14.250	10.150	1.00	0.00
ATOM	4308	OW	SOL	1062	13.980	2.130	28.300	1.00	0.00
ATOM	4309	HW1	SOL	1062	14.340	1.910	27.440	1.00	0.00
ATOM	4310	HW2	SOL	1062	13.680	1.290	28.650	1.00	0.00
ATOM	4311	OW	SOL	1063	27.450	51.520	9.650	1.00	0.00

ATOM	4312	HW1	SOL	1063	28.180	51.070	10.060	1.00	0.00
ATOM	4313	HW2	SOL	1063	27.200	50.970	8.910	1.00	0.00
ATOM	4314	OW	SOL	1064	50.620	36.600	25.310	1.00	0.00
ATOM	4315	HW1	SOL	1064	50.750	36.380	24.390	1.00	0.00
ATOM	4316	HW2	SOL	1064	50.570	35.750	25.750	1.00	0.00
ATOM	4317	OW	SOL	1065	54.120	8.550	16.870	1.00	0.00
ATOM	4318	HW1	SOL	1065	54.020	7.940	16.130	1.00	0.00
ATOM	4319	HW2	SOL	1065	54.350	9.380	16.460	1.00	0.00
ATOM	4320	OW	SOL	1066	24.840	7.830	10.300	1.00	0.00
ATOM	4321	HW1	SOL	1066	25.210	7.040	10.680	1.00	0.00
ATOM	4322	HW2	SOL	1066	25.490	8.510	10.480	1.00	0.00
ATOM	4323	OW	SOL	1067	51.960	45.830	7.770	1.00	0.00
ATOM	4324	HW1	SOL	1067	52.780	45.890	7.280	1.00	0.00
ATOM	4325	HW2	SOL	1067	51.920	44.930	8.090	1.00	0.00
ATOM	4326	OW	SOL	1068	36.980	11.900	39.160	1.00	0.00
ATOM	4327	HW1	SOL	1068	37.530	11.470	38.510	1.00	0.00
ATOM	4328	HW2	SOL	1068	37.130	12.830	39.020	1.00	0.00
ATOM	4329	OW	SOL	1069	6.240	1.920	26.910	1.00	0.00
ATOM	4330	HW1	SOL	1069	7.090	2.340	26.790	1.00	0.00
ATOM	4331	HW2	SOL	1069	6.100	1.950	27.860	1.00	0.00
ATOM	4332	OW	SOL	1070	50.450	0.730	3.660	1.00	0.00
ATOM	4333	HW1	SOL	1070	50.970	1.530	3.730	1.00	0.00
ATOM	4334	HW2	SOL	1070	50.680	0.380	2.790	1.00	0.00
ATOM	4335	OW	SOL	1071	0.070	54.580	48.740	1.00	0.00
ATOM	4336	HW1	SOL	1071	-0.120	54.790	49.650	1.00	0.00
ATOM	4337	HW2	SOL	1071	-0.410	55.240	48.230	1.00	0.00
ATOM	4338	OW	SOL	1072	49.840	49.890	20.100	1.00	0.00
ATOM	4339	HW1	SOL	1072	49.620	50.680	19.610	1.00	0.00
ATOM	4340	HW2	SOL	1072	49.020	49.390	20.130	1.00	0.00
ATOM	4341	OW	SOL	1073	5.350	45.540	27.970	1.00	0.00
ATOM	4342	HW1	SOL	1073	4.970	46.180	28.570	1.00	0.00
ATOM	4343	HW2	SOL	1073	6.000	46.030	27.460	1.00	0.00
ATOM	4344	OW	SOL	1074	26.560	3.020	42.840	1.00	0.00
ATOM	4345	HW1	SOL	1074	26.160	3.420	42.070	1.00	0.00
ATOM	4346	HW2	SOL	1074	26.490	3.690	43.520	1.00	0.00
ATOM	4347	OW	SOL	1075	10.230	14.720	41.700	1.00	0.00
ATOM	4348	HW1	SOL	1075	10.790	13.980	41.950	1.00	0.00
ATOM	4349	HW2	SOL	1075	10.500	14.920	40.800	1.00	0.00
ATOM	4350	OW	SOL	1076	13.720	12.750	11.070	1.00	0.00
ATOM	4351	HW1	SOL	1076	13.950	12.740	10.140	1.00	0.00
ATOM	4352	HW2	SOL	1076	14.480	12.380	11.510	1.00	0.00
ATOM	4353	OW	SOL	1077	27.140	37.680	2.530	1.00	0.00
ATOM	4354	HW1	SOL	1077	27.350	37.270	1.690	1.00	0.00
ATOM	4355	HW2	SOL	1077	27.820	38.340	2.660	1.00	0.00

ATOM	4356	OW	SOL	1078	55.240	11.780	1.100	1.00	0.00
ATOM	4357	HW1	SOL	1078	54.670	11.060	0.830	1.00	0.00
ATOM	4358	HW2	SOL	1078	54.640	12.480	1.360	1.00	0.00
ATOM	4359	OW	SOL	1079	31.730	8.000	14.980	1.00	0.00
ATOM	4360	HW1	SOL	1079	31.360	8.700	15.510	1.00	0.00
ATOM	4361	HW2	SOL	1079	32.450	7.650	15.510	1.00	0.00
ATOM	4362	OW	SOL	1080	21.280	44.160	21.350	1.00	0.00
ATOM	4363	HW1	SOL	1080	21.620	43.670	20.600	1.00	0.00
ATOM	4364	HW2	SOL	1080	21.450	45.080	21.130	1.00	0.00
ATOM	4365	OW	SOL	1081	7.830	31.790	17.320	1.00	0.00
ATOM	4366	HW1	SOL	1081	7.820	30.860	17.130	1.00	0.00
ATOM	4367	HW2	SOL	1081	8.760	32.030	17.340	1.00	0.00
ATOM	4368	OW	SOL	1082	15.460	38.190	33.010	1.00	0.00
ATOM	4369	HW1	SOL	1082	15.370	38.680	33.830	1.00	0.00
ATOM	4370	HW2	SOL	1082	16.020	38.730	32.460	1.00	0.00
ATOM	4371	OW	SOL	1083	13.870	12.940	40.170	1.00	0.00
ATOM	4372	HW1	SOL	1083	13.810	12.410	40.970	1.00	0.00
ATOM	4373	HW2	SOL	1083	14.400	13.690	40.420	1.00	0.00
ATOM	4374	OW	SOL	1084	43.110	35.710	46.750	1.00	0.00
ATOM	4375	HW1	SOL	1084	43.750	36.040	46.120	1.00	0.00
ATOM	4376	HW2	SOL	1084	42.830	36.480	47.240	1.00	0.00
ATOM	4377	OW	SOL	1085	33.150	42.910	40.400	1.00	0.00
ATOM	4378	HW1	SOL	1085	32.710	42.140	40.750	1.00	0.00
ATOM	4379	HW2	SOL	1085	32.600	43.650	40.670	1.00	0.00
ATOM	4380	OW	SOL	1086	40.530	3.920	12.290	1.00	0.00
ATOM	4381	HW1	SOL	1086	40.360	4.800	11.960	1.00	0.00
ATOM	4382	HW2	SOL	1086	41.300	4.020	12.850	1.00	0.00
ATOM	4383	OW	SOL	1087	1.940	34.140	31.670	1.00	0.00
ATOM	4384	HW1	SOL	1087	1.090	34.360	31.300	1.00	0.00
ATOM	4385	HW2	SOL	1087	2.510	34.880	31.460	1.00	0.00
ATOM	4386	OW	SOL	1088	8.930	41.300	8.680	1.00	0.00
ATOM	4387	HW1	SOL	1088	9.840	41.270	8.940	1.00	0.00
ATOM	4388	HW2	SOL	1088	8.560	42.030	9.160	1.00	0.00
ATOM	4389	OW	SOL	1089	48.760	55.110	51.880	1.00	0.00
ATOM	4390	HW1	SOL	1089	48.420	54.460	51.280	1.00	0.00
ATOM	4391	HW2	SOL	1089	49.620	54.770	52.140	1.00	0.00
ATOM	4392	OW	SOL	1090	44.900	6.790	39.420	1.00	0.00
ATOM	4393	HW1	SOL	1090	44.990	7.540	40.010	1.00	0.00
ATOM	4394	HW2	SOL	1090	45.770	6.690	39.030	1.00	0.00
ATOM	4395	OW	SOL	1091	44.170	41.330	11.520	1.00	0.00
ATOM	4396	HW1	SOL	1091	44.990	41.220	11.040	1.00	0.00
ATOM	4397	HW2	SOL	1091	43.830	40.430	11.620	1.00	0.00
ATOM	4398	OW	SOL	1092	49.210	51.040	40.060	1.00	0.00
ATOM	4399	HW1	SOL	1092	49.120	50.110	40.240	1.00	0.00

ATOM	4400	HW2	SOL	1092	49.520	51.080	39.160	1.00	0.00
ATOM	4401	OW	SOL	1093	44.400	25.780	1.650	1.00	0.00
ATOM	4402	HW1	SOL	1093	45.170	26.030	2.160	1.00	0.00
ATOM	4403	HW2	SOL	1093	43.660	26.130	2.150	1.00	0.00
ATOM	4404	OW	SOL	1094	3.040	11.640	8.340	1.00	0.00
ATOM	4405	HW1	SOL	1094	2.240	11.760	7.830	1.00	0.00
ATOM	4406	HW2	SOL	1094	3.520	10.940	7.890	1.00	0.00
ATOM	4407	OW	SOL	1095	11.940	33.100	46.600	1.00	0.00
ATOM	4408	HW1	SOL	1095	11.140	32.590	46.690	1.00	0.00
ATOM	4409	HW2	SOL	1095	12.620	32.440	46.400	1.00	0.00
ATOM	4410	OW	SOL	1096	38.560	15.930	15.440	1.00	0.00
ATOM	4411	HW1	SOL	1096	37.870	16.580	15.590	1.00	0.00
ATOM	4412	HW2	SOL	1096	38.530	15.760	14.500	1.00	0.00
ATOM	4413	OW	SOL	1097	41.410	45.190	51.240	1.00	0.00
ATOM	4414	HW1	SOL	1097	40.840	44.800	51.900	1.00	0.00
ATOM	4415	HW2	SOL	1097	41.970	45.790	51.740	1.00	0.00
ATOM	4416	OW	SOL	1098	29.730	10.220	43.010	1.00	0.00
ATOM	4417	HW1	SOL	1098	29.800	9.590	42.300	1.00	0.00
ATOM	4418	HW2	SOL	1098	29.820	11.070	42.590	1.00	0.00
ATOM	4419	OW	SOL	1099	24.200	54.210	1.030	1.00	0.00
ATOM	4420	HW1	SOL	1099	23.290	54.240	1.320	1.00	0.00
ATOM	4421	HW2	SOL	1099	24.620	53.610	1.650	1.00	0.00
ATOM	4422	OW	SOL	1100	9.290	46.610	38.240	1.00	0.00
ATOM	4423	HW1	SOL	1100	9.980	46.840	38.860	1.00	0.00
ATOM	4424	HW2	SOL	1100	9.020	45.730	38.500	1.00	0.00
ATOM	4425	OW	SOL	1101	12.230	30.740	43.950	1.00	0.00
ATOM	4426	HW1	SOL	1101	11.640	31.420	43.640	1.00	0.00
ATOM	4427	HW2	SOL	1101	13.100	31.020	43.680	1.00	0.00
ATOM	4428	OW	SOL	1102	50.640	11.210	22.050	1.00	0.00
ATOM	4429	HW1	SOL	1102	51.370	11.030	22.650	1.00	0.00
ATOM	4430	HW2	SOL	1102	50.120	11.870	22.510	1.00	0.00
ATOM	4431	OW	SOL	1103	35.690	43.010	53.100	1.00	0.00
ATOM	4432	HW1	SOL	1103	34.870	43.500	53.110	1.00	0.00
ATOM	4433	HW2	SOL	1103	36.250	43.500	52.500	1.00	0.00
ATOM	4434	OW	SOL	1104	52.340	52.710	50.150	1.00	0.00
ATOM	4435	HW1	SOL	1104	51.990	52.850	49.270	1.00	0.00
ATOM	4436	HW2	SOL	1104	52.030	53.470	50.650	1.00	0.00
ATOM	4437	OW	SOL	1105	29.280	5.190	31.600	1.00	0.00
ATOM	4438	HW1	SOL	1105	28.350	5.260	31.800	1.00	0.00
ATOM	4439	HW2	SOL	1105	29.550	6.080	31.380	1.00	0.00
ATOM	4440	OW	SOL	1106	44.390	36.680	13.030	1.00	0.00
ATOM	4441	HW1	SOL	1106	45.270	36.400	12.760	1.00	0.00
ATOM	4442	HW2	SOL	1106	44.230	36.220	13.850	1.00	0.00
ATOM	4443	OW	SOL	1107	49.450	29.040	13.550	1.00	0.00

ATOM	4444	HW1	SOL	1107	49.500	28.140	13.880	1.00	0.00
ATOM	4445	HW2	SOL	1107	50.240	29.460	13.900	1.00	0.00
ATOM	4446	OW	SOL	1108	13.400	5.100	20.560	1.00	0.00
ATOM	4447	HW1	SOL	1108	13.190	4.290	20.100	1.00	0.00
ATOM	4448	HW2	SOL	1108	13.030	4.980	21.440	1.00	0.00
ATOM	4449	OW	SOL	1109	50.710	8.510	35.760	1.00	0.00
ATOM	4450	HW1	SOL	1109	50.170	9.070	36.310	1.00	0.00
ATOM	4451	HW2	SOL	1109	50.760	7.680	36.240	1.00	0.00
ATOM	4452	OW	SOL	1110	17.680	13.100	37.320	1.00	0.00
ATOM	4453	HW1	SOL	1110	16.920	13.580	36.990	1.00	0.00
ATOM	4454	HW2	SOL	1110	17.490	12.950	38.250	1.00	0.00
ATOM	4455	OW	SOL	1111	22.320	23.250	47.100	1.00	0.00
ATOM	4456	HW1	SOL	1111	21.640	23.150	47.770	1.00	0.00
ATOM	4457	HW2	SOL	1111	22.330	24.190	46.920	1.00	0.00
ATOM	4458	OW	SOL	1112	14.710	18.650	30.680	1.00	0.00
ATOM	4459	HW1	SOL	1112	14.950	17.770	30.970	1.00	0.00
ATOM	4460	HW2	SOL	1112	15.470	18.960	30.190	1.00	0.00
ATOM	4461	OW	SOL	1113	45.180	13.930	14.120	1.00	0.00
ATOM	4462	HW1	SOL	1113	44.400	13.510	14.470	1.00	0.00
ATOM	4463	HW2	SOL	1113	45.870	13.730	14.750	1.00	0.00
ATOM	4464	OW	SOL	1114	31.460	20.170	47.710	1.00	0.00
ATOM	4465	HW1	SOL	1114	31.170	20.550	48.540	1.00	0.00
ATOM	4466	HW2	SOL	1114	31.690	20.910	47.160	1.00	0.00
ATOM	4467	OW	SOL	1115	44.370	34.900	22.240	1.00	0.00
ATOM	4468	HW1	SOL	1115	43.560	35.080	21.760	1.00	0.00
ATOM	4469	HW2	SOL	1115	45.060	35.260	21.680	1.00	0.00
ATOM	4470	OW	SOL	1116	3.710	38.030	9.910	1.00	0.00
ATOM	4471	HW1	SOL	1116	4.110	37.650	10.700	1.00	0.00
ATOM	4472	HW2	SOL	1116	4.450	38.240	9.350	1.00	0.00
ATOM	4473	OW	SOL	1117	14.480	0.250	26.140	1.00	0.00
ATOM	4474	HW1	SOL	1117	15.190	0.010	26.740	1.00	0.00
ATOM	4475	HW2	SOL	1117	13.860	-0.470	26.200	1.00	0.00
ATOM	4476	OW	SOL	1118	32.570	32.160	47.610	1.00	0.00
ATOM	4477	HW1	SOL	1118	32.320	31.490	46.980	1.00	0.00
ATOM	4478	HW2	SOL	1118	32.650	32.960	47.090	1.00	0.00
ATOM	4479	OW	SOL	1119	20.840	30.350	31.550	1.00	0.00
ATOM	4480	HW1	SOL	1119	20.260	30.570	30.820	1.00	0.00
ATOM	4481	HW2	SOL	1119	21.070	29.430	31.400	1.00	0.00
ATOM	4482	OW	SOL	1120	52.190	53.600	18.960	1.00	0.00
ATOM	4483	HW1	SOL	1120	52.140	53.090	18.150	1.00	0.00
ATOM	4484	HW2	SOL	1120	53.080	53.960	18.950	1.00	0.00
ATOM	4485	OW	SOL	1121	15.330	34.530	1.930	1.00	0.00
ATOM	4486	HW1	SOL	1121	15.490	35.470	2.020	1.00	0.00
ATOM	4487	HW2	SOL	1121	16.170	34.170	1.630	1.00	0.00

ATOM	4488	OW	SOL	1122	4.740	9.740	25.550	1.00	0.00
ATOM	4489	HW1	SOL	1122	5.590	9.410	25.290	1.00	0.00
ATOM	4490	HW2	SOL	1122	4.870	10.100	26.430	1.00	0.00
ATOM	4491	OW	SOL	1123	17.730	33.620	1.830	1.00	0.00
ATOM	4492	HW1	SOL	1123	17.530	33.050	2.570	1.00	0.00
ATOM	4493	HW2	SOL	1123	18.260	33.080	1.250	1.00	0.00
ATOM	4494	OW	SOL	1124	53.730	24.670	49.980	1.00	0.00
ATOM	4495	HW1	SOL	1124	52.780	24.540	50.060	1.00	0.00
ATOM	4496	HW2	SOL	1124	54.030	24.780	50.880	1.00	0.00
ATOM	4497	OW	SOL	1125	55.240	11.130	48.540	1.00	0.00
ATOM	4498	HW1	SOL	1125	55.580	10.910	47.670	1.00	0.00
ATOM	4499	HW2	SOL	1125	54.390	10.700	48.580	1.00	0.00
ATOM	4500	OW	SOL	1126	47.830	41.060	5.100	1.00	0.00
ATOM	4501	HW1	SOL	1126	47.200	41.260	4.410	1.00	0.00
ATOM	4502	HW2	SOL	1126	48.620	40.780	4.640	1.00	0.00
ATOM	4503	OW	SOL	1127	25.210	15.580	32.670	1.00	0.00
ATOM	4504	HW1	SOL	1127	25.430	15.510	31.740	1.00	0.00
ATOM	4505	HW2	SOL	1127	25.140	16.520	32.830	1.00	0.00
ATOM	4506	OW	SOL	1128	41.870	2.500	1.530	1.00	0.00
ATOM	4507	HW1	SOL	1128	42.140	3.140	2.190	1.00	0.00
ATOM	4508	HW2	SOL	1128	42.160	1.660	1.870	1.00	0.00
ATOM	4509	OW	SOL	1129	12.910	3.720	38.460	1.00	0.00
ATOM	4510	HW1	SOL	1129	12.450	4.280	37.840	1.00	0.00
ATOM	4511	HW2	SOL	1129	12.220	3.260	38.930	1.00	0.00
ATOM	4512	OW	SOL	1130	33.020	17.060	10.380	1.00	0.00
ATOM	4513	HW1	SOL	1130	33.410	16.260	10.740	1.00	0.00
ATOM	4514	HW2	SOL	1130	32.490	16.750	9.640	1.00	0.00
ATOM	4515	OW	SOL	1131	42.310	35.390	20.530	1.00	0.00
ATOM	4516	HW1	SOL	1131	42.710	36.020	19.930	1.00	0.00
ATOM	4517	HW2	SOL	1131	41.360	35.490	20.390	1.00	0.00
ATOM	4518	OW	SOL	1132	41.240	4.960	36.400	1.00	0.00
ATOM	4519	HW1	SOL	1132	41.500	5.780	35.980	1.00	0.00
ATOM	4520	HW2	SOL	1132	40.600	5.210	37.060	1.00	0.00
ATOM	4521	OW	SOL	1133	13.300	42.230	16.130	1.00	0.00
ATOM	4522	HW1	SOL	1133	13.040	41.510	16.700	1.00	0.00
ATOM	4523	HW2	SOL	1133	12.640	42.250	15.440	1.00	0.00
ATOM	4524	OW	SOL	1134	10.510	35.400	36.480	1.00	0.00
ATOM	4525	HW1	SOL	1134	9.750	35.960	36.320	1.00	0.00
ATOM	4526	HW2	SOL	1134	10.470	34.740	35.780	1.00	0.00
ATOM	4527	OW	SOL	1135	27.730	7.270	19.350	1.00	0.00
ATOM	4528	HW1	SOL	1135	26.990	7.610	18.850	1.00	0.00
ATOM	4529	HW2	SOL	1135	28.300	6.870	18.690	1.00	0.00
ATOM	4530	OW	SOL	1136	14.230	6.700	15.670	1.00	0.00
ATOM	4531	HW1	SOL	1136	14.360	6.330	14.790	1.00	0.00

ATOM	4532	HW2	SOL	1136	13.460	6.240	16.010	1.00	0.00
ATOM	4533	OW	SOL	1137	48.140	13.880	5.900	1.00	0.00
ATOM	4534	HW1	SOL	1137	48.650	14.230	6.620	1.00	0.00
ATOM	4535	HW2	SOL	1137	47.330	14.390	5.910	1.00	0.00
ATOM	4536	OW	SOL	1138	46.900	55.170	5.980	1.00	0.00
ATOM	4537	HW1	SOL	1138	45.950	55.090	5.940	1.00	0.00
ATOM	4538	HW2	SOL	1138	47.180	54.470	6.570	1.00	0.00
ATOM	4539	OW	SOL	1139	6.230	27.380	46.960	1.00	0.00
ATOM	4540	HW1	SOL	1139	6.200	28.200	46.470	1.00	0.00
ATOM	4541	HW2	SOL	1139	5.310	27.180	47.140	1.00	0.00
ATOM	4542	OW	SOL	1140	53.380	48.570	42.490	1.00	0.00
ATOM	4543	HW1	SOL	1140	53.280	49.310	41.890	1.00	0.00
ATOM	4544	HW2	SOL	1140	52.610	48.030	42.320	1.00	0.00
ATOM	4545	OW	SOL	1141	21.240	15.600	41.240	1.00	0.00
ATOM	4546	HW1	SOL	1141	20.590	15.760	40.550	1.00	0.00
ATOM	4547	HW2	SOL	1141	21.390	14.660	41.210	1.00	0.00
ATOM	4548	OW	SOL	1142	32.380	47.130	9.200	1.00	0.00
ATOM	4549	HW1	SOL	1142	32.300	46.560	9.960	1.00	0.00
ATOM	4550	HW2	SOL	1142	32.620	46.540	8.480	1.00	0.00
ATOM	4551	OW	SOL	1143	10.240	46.170	47.850	1.00	0.00
ATOM	4552	HW1	SOL	1143	9.770	46.670	48.520	1.00	0.00
ATOM	4553	HW2	SOL	1143	10.590	45.410	48.310	1.00	0.00
ATOM	4554	OW	SOL	1144	33.580	42.280	33.130	1.00	0.00
ATOM	4555	HW1	SOL	1144	33.910	43.100	33.490	1.00	0.00
ATOM	4556	HW2	SOL	1144	33.730	41.630	33.820	1.00	0.00
ATOM	4557	OW	SOL	1145	6.910	34.240	49.740	1.00	0.00
ATOM	4558	HW1	SOL	1145	6.610	35.080	50.070	1.00	0.00
ATOM	4559	HW2	SOL	1145	7.010	33.700	50.530	1.00	0.00
ATOM	4560	OW	SOL	1146	19.520	27.340	28.730	1.00	0.00
ATOM	4561	HW1	SOL	1146	18.890	26.620	28.710	1.00	0.00
ATOM	4562	HW2	SOL	1146	20.350	26.940	28.480	1.00	0.00
ATOM	4563	OW	SOL	1147	8.060	39.680	31.790	1.00	0.00
ATOM	4564	HW1	SOL	1147	7.740	40.540	31.490	1.00	0.00
ATOM	4565	HW2	SOL	1147	9.010	39.730	31.690	1.00	0.00
ATOM	4566	OW	SOL	1148	43.940	33.270	18.670	1.00	0.00
ATOM	4567	HW1	SOL	1148	43.430	32.730	18.070	1.00	0.00
ATOM	4568	HW2	SOL	1148	43.290	33.620	19.280	1.00	0.00
ATOM	4569	OW	SOL	1149	11.040	18.110	12.730	1.00	0.00
ATOM	4570	HW1	SOL	1149	11.720	18.210	12.060	1.00	0.00
ATOM	4571	HW2	SOL	1149	10.220	18.050	12.240	1.00	0.00
ATOM	4572	OW	SOL	1150	49.830	11.460	44.660	1.00	0.00
ATOM	4573	HW1	SOL	1150	49.750	10.510	44.530	1.00	0.00
ATOM	4574	HW2	SOL	1150	49.510	11.840	43.840	1.00	0.00
ATOM	4575	OW	SOL	1151	47.590	30.490	55.830	1.00	0.00

ATOM	4576	HW1	SOL	1151	46.740	30.740	56.210	1.00	0.00
ATOM	4577	HW2	SOL	1151	48.190	30.520	56.570	1.00	0.00
ATOM	4578	OW	SOL	1152	44.000	44.000	7.090	1.00	0.00
ATOM	4579	HW1	SOL	1152	44.810	44.230	6.650	1.00	0.00
ATOM	4580	HW2	SOL	1152	44.210	44.040	8.030	1.00	0.00
ATOM	4581	OW	SOL	1153	51.100	24.580	51.190	1.00	0.00
ATOM	4582	HW1	SOL	1153	50.740	23.700	51.340	1.00	0.00
ATOM	4583	HW2	SOL	1153	50.840	25.090	51.950	1.00	0.00
ATOM	4584	OW	SOL	1154	26.470	31.080	13.010	1.00	0.00
ATOM	4585	HW1	SOL	1154	26.030	31.600	13.680	1.00	0.00
ATOM	4586	HW2	SOL	1154	26.840	31.730	12.400	1.00	0.00
ATOM	4587	OW	SOL	1155	21.330	53.760	21.910	1.00	0.00
ATOM	4588	HW1	SOL	1155	20.510	54.220	22.090	1.00	0.00
ATOM	4589	HW2	SOL	1155	21.080	53.060	21.300	1.00	0.00
ATOM	4590	OW	SOL	1156	25.540	22.880	33.010	1.00	0.00
ATOM	4591	HW1	SOL	1156	26.090	23.210	33.720	1.00	0.00
ATOM	4592	HW2	SOL	1156	25.110	23.660	32.650	1.00	0.00
ATOM	4593	OW	SOL	1157	35.150	47.180	25.350	1.00	0.00
ATOM	4594	HW1	SOL	1157	34.410	46.610	25.560	1.00	0.00
ATOM	4595	HW2	SOL	1157	35.870	46.840	25.890	1.00	0.00
ATOM	4596	OW	SOL	1158	0.650	34.040	45.220	1.00	0.00
ATOM	4597	HW1	SOL	1158	0.330	33.300	45.740	1.00	0.00
ATOM	4598	HW2	SOL	1158	1.200	33.630	44.550	1.00	0.00
ATOM	4599	OW	SOL	1159	45.840	13.440	49.810	1.00	0.00
ATOM	4600	HW1	SOL	1159	45.180	14.060	50.140	1.00	0.00
ATOM	4601	HW2	SOL	1159	46.260	13.910	49.090	1.00	0.00
ATOM	4602	OW	SOL	1160	41.280	53.480	16.140	1.00	0.00
ATOM	4603	HW1	SOL	1160	41.780	52.670	16.240	1.00	0.00
ATOM	4604	HW2	SOL	1160	41.520	54.010	16.900	1.00	0.00
ATOM	4605	OW	SOL	1161	16.610	9.300	6.540	1.00	0.00
ATOM	4606	HW1	SOL	1161	16.110	9.350	7.360	1.00	0.00
ATOM	4607	HW2	SOL	1161	15.990	8.960	5.900	1.00	0.00
ATOM	4608	OW	SOL	1162	2.150	47.810	38.060	1.00	0.00
ATOM	4609	HW1	SOL	1162	1.500	47.480	38.680	1.00	0.00
ATOM	4610	HW2	SOL	1162	1.820	48.670	37.820	1.00	0.00
ATOM	4611	OW	SOL	1163	44.280	54.920	6.310	1.00	0.00
ATOM	4612	HW1	SOL	1163	43.900	55.370	7.060	1.00	0.00
ATOM	4613	HW2	SOL	1163	44.040	55.460	5.560	1.00	0.00
ATOM	4614	OW	SOL	1164	8.780	2.950	37.650	1.00	0.00
ATOM	4615	HW1	SOL	1164	9.660	2.780	37.330	1.00	0.00
ATOM	4616	HW2	SOL	1164	8.840	2.810	38.600	1.00	0.00
ATOM	4617	OW	SOL	1165	38.410	44.150	39.800	1.00	0.00
ATOM	4618	HW1	SOL	1165	39.070	43.800	39.200	1.00	0.00
ATOM	4619	HW2	SOL	1165	37.890	43.390	40.060	1.00	0.00

ATOM	4620	OW	SOL	1166	25.410	11.330	32.010	1.00	0.00
ATOM	4621	HW1	SOL	1166	24.800	11.990	32.340	1.00	0.00
ATOM	4622	HW2	SOL	1166	25.430	11.480	31.060	1.00	0.00
ATOM	4623	OW	SOL	1167	22.320	25.180	20.890	1.00	0.00
ATOM	4624	HW1	SOL	1167	21.610	24.550	21.040	1.00	0.00
ATOM	4625	HW2	SOL	1167	22.230	25.430	19.970	1.00	0.00
ATOM	4626	OW	SOL	1168	23.660	8.860	1.770	1.00	0.00
ATOM	4627	HW1	SOL	1168	23.520	8.670	0.840	1.00	0.00
ATOM	4628	HW2	SOL	1168	24.580	9.140	1.820	1.00	0.00
ATOM	4629	OW	SOL	1169	16.510	49.610	3.490	1.00	0.00
ATOM	4630	HW1	SOL	1169	16.170	50.410	3.890	1.00	0.00
ATOM	4631	HW2	SOL	1169	17.380	49.840	3.180	1.00	0.00
ATOM	4632	OW	SOL	1170	35.120	4.850	13.260	1.00	0.00
ATOM	4633	HW1	SOL	1170	35.830	5.420	13.550	1.00	0.00
ATOM	4634	HW2	SOL	1170	35.530	3.980	13.210	1.00	0.00
ATOM	4635	OW	SOL	1171	52.190	15.220	11.010	1.00	0.00
ATOM	4636	HW1	SOL	1171	51.940	14.430	10.520	1.00	0.00
ATOM	4637	HW2	SOL	1171	52.260	15.900	10.340	1.00	0.00
ATOM	4638	OW	SOL	1172	7.910	43.360	33.310	1.00	0.00
ATOM	4639	HW1	SOL	1172	7.480	42.950	32.560	1.00	0.00
ATOM	4640	HW2	SOL	1172	8.570	42.730	33.590	1.00	0.00
ATOM	4641	OW	SOL	1173	11.500	2.500	12.670	1.00	0.00
ATOM	4642	HW1	SOL	1173	10.810	2.300	12.030	1.00	0.00
ATOM	4643	HW2	SOL	1173	12.270	2.020	12.350	1.00	0.00
ATOM	4644	OW	SOL	1174	39.380	24.640	45.110	1.00	0.00
ATOM	4645	HW1	SOL	1174	38.690	24.040	44.810	1.00	0.00
ATOM	4646	HW2	SOL	1174	40.190	24.260	44.780	1.00	0.00
ATOM	4647	OW	SOL	1175	14.350	51.650	8.190	1.00	0.00
ATOM	4648	HW1	SOL	1175	14.440	51.080	7.420	1.00	0.00
ATOM	4649	HW2	SOL	1175	14.840	51.210	8.880	1.00	0.00
ATOM	4650	OW	SOL	1176	52.640	35.160	53.810	1.00	0.00
ATOM	4651	HW1	SOL	1176	52.510	36.100	53.760	1.00	0.00
ATOM	4652	HW2	SOL	1176	51.760	34.790	53.930	1.00	0.00
ATOM	4653	OW	SOL	1177	8.550	10.350	42.120	1.00	0.00
ATOM	4654	HW1	SOL	1177	7.690	10.450	41.710	1.00	0.00
ATOM	4655	HW2	SOL	1177	8.990	9.680	41.600	1.00	0.00
ATOM	4656	OW	SOL	1178	11.730	3.900	27.520	1.00	0.00
ATOM	4657	HW1	SOL	1178	11.940	3.490	26.680	1.00	0.00
ATOM	4658	HW2	SOL	1178	12.520	3.770	28.050	1.00	0.00
ATOM	4659	OW	SOL	1179	30.690	1.950	48.610	1.00	0.00
ATOM	4660	HW1	SOL	1179	30.490	2.140	47.700	1.00	0.00
ATOM	4661	HW2	SOL	1179	29.870	2.080	49.070	1.00	0.00
ATOM	4662	OW	SOL	1180	44.260	43.960	20.720	1.00	0.00
ATOM	4663	HW1	SOL	1180	44.380	44.360	21.580	1.00	0.00

ATOM	4664	HW2	SOL	1180	43.430	43.490	20.790	1.00	0.00
ATOM	4665	OW	SOL	1181	2.370	45.930	54.970	1.00	0.00
ATOM	4666	HW1	SOL	1181	1.890	45.700	54.170	1.00	0.00
ATOM	4667	HW2	SOL	1181	1.690	46.100	55.620	1.00	0.00
ATOM	4668	OW	SOL	1182	40.920	51.000	37.880	1.00	0.00
ATOM	4669	HW1	SOL	1182	40.320	50.700	37.190	1.00	0.00
ATOM	4670	HW2	SOL	1182	41.710	51.270	37.400	1.00	0.00
ATOM	4671	OW	SOL	1183	18.560	52.170	22.770	1.00	0.00
ATOM	4672	HW1	SOL	1183	19.030	51.680	22.100	1.00	0.00
ATOM	4673	HW2	SOL	1183	17.630	52.030	22.570	1.00	0.00
ATOM	4674	OW	SOL	1184	7.210	7.530	26.290	1.00	0.00
ATOM	4675	HW1	SOL	1184	7.310	7.900	25.410	1.00	0.00
ATOM	4676	HW2	SOL	1184	8.080	7.220	26.520	1.00	0.00
ATOM	4677	OW	SOL	1185	0.130	38.030	46.650	1.00	0.00
ATOM	4678	HW1	SOL	1185	0.860	38.230	46.070	1.00	0.00
ATOM	4679	HW2	SOL	1185	-0.130	38.880	47.010	1.00	0.00
ATOM	4680	OW	SOL	1186	32.010	49.560	42.970	1.00	0.00
ATOM	4681	HW1	SOL	1186	31.370	49.480	43.690	1.00	0.00
ATOM	4682	HW2	SOL	1186	31.470	49.750	42.200	1.00	0.00
ATOM	4683	OW	SOL	1187	18.730	0.870	10.830	1.00	0.00
ATOM	4684	HW1	SOL	1187	18.060	0.490	11.400	1.00	0.00
ATOM	4685	HW2	SOL	1187	18.830	1.770	11.140	1.00	0.00
ATOM	4686	OW	SOL	1188	7.240	46.360	49.240	1.00	0.00
ATOM	4687	HW1	SOL	1188	6.710	46.720	48.530	1.00	0.00
ATOM	4688	HW2	SOL	1188	7.870	47.050	49.440	1.00	0.00
ATOM	4689	OW	SOL	1189	2.690	40.550	13.770	1.00	0.00
ATOM	4690	HW1	SOL	1189	2.890	41.260	14.380	1.00	0.00
ATOM	4691	HW2	SOL	1189	3.280	40.690	13.030	1.00	0.00
ATOM	4692	OW	SOL	1190	13.020	26.450	13.950	1.00	0.00
ATOM	4693	HW1	SOL	1190	12.810	26.370	13.010	1.00	0.00
ATOM	4694	HW2	SOL	1190	13.910	26.090	14.020	1.00	0.00
ATOM	4695	OW	SOL	1191	18.210	41.570	48.740	1.00	0.00
ATOM	4696	HW1	SOL	1191	19.050	41.380	49.160	1.00	0.00
ATOM	4697	HW2	SOL	1191	17.560	41.140	49.290	1.00	0.00
ATOM	4698	OW	SOL	1192	17.630	0.660	19.550	1.00	0.00
ATOM	4699	HW1	SOL	1192	16.920	0.030	19.470	1.00	0.00
ATOM	4700	HW2	SOL	1192	17.250	1.390	20.040	1.00	0.00
ATOM	4701	OW	SOL	1193	15.810	1.250	53.470	1.00	0.00
ATOM	4702	HW1	SOL	1193	15.150	0.770	52.970	1.00	0.00
ATOM	4703	HW2	SOL	1193	15.360	1.540	54.260	1.00	0.00
ATOM	4704	OW	SOL	1194	45.060	53.090	54.270	1.00	0.00
ATOM	4705	HW1	SOL	1194	45.350	54.010	54.320	1.00	0.00
ATOM	4706	HW2	SOL	1194	45.330	52.720	55.110	1.00	0.00
ATOM	4707	OW	SOL	1195	47.610	14.210	41.170	1.00	0.00

ATOM	4708	HW1	SOL	1195	48.210	14.290	40.420	1.00	0.00
ATOM	4709	HW2	SOL	1195	48.150	13.820	41.860	1.00	0.00
ATOM	4710	OW	SOL	1196	20.390	8.190	24.900	1.00	0.00
ATOM	4711	HW1	SOL	1196	20.240	7.730	25.730	1.00	0.00
ATOM	4712	HW2	SOL	1196	19.960	9.040	25.010	1.00	0.00
ATOM	4713	OW	SOL	1197	32.720	23.030	30.990	1.00	0.00
ATOM	4714	HW1	SOL	1197	33.360	23.120	31.700	1.00	0.00
ATOM	4715	HW2	SOL	1197	32.990	22.250	30.520	1.00	0.00
ATOM	4716	OW	SOL	1198	34.180	41.840	24.790	1.00	0.00
ATOM	4717	HW1	SOL	1198	33.760	42.690	24.920	1.00	0.00
ATOM	4718	HW2	SOL	1198	34.020	41.630	23.870	1.00	0.00
ATOM	4719	OW	SOL	1199	6.230	14.690	13.580	1.00	0.00
ATOM	4720	HW1	SOL	1199	6.120	15.070	12.710	1.00	0.00
ATOM	4721	HW2	SOL	1199	6.180	15.440	14.180	1.00	0.00
ATOM	4722	OW	SOL	1200	9.550	13.930	54.710	1.00	0.00
ATOM	4723	HW1	SOL	1200	8.880	14.610	54.820	1.00	0.00
ATOM	4724	HW2	SOL	1200	9.850	14.030	53.810	1.00	0.00
ATOM	4725	OW	SOL	1201	38.580	39.270	18.360	1.00	0.00
ATOM	4726	HW1	SOL	1201	38.540	38.890	17.480	1.00	0.00
ATOM	4727	HW2	SOL	1201	38.370	38.550	18.950	1.00	0.00
ATOM	4728	OW	SOL	1202	52.510	45.590	24.190	1.00	0.00
ATOM	4729	HW1	SOL	1202	53.180	45.010	23.830	1.00	0.00
ATOM	4730	HW2	SOL	1202	52.040	45.060	24.840	1.00	0.00
ATOM	4731	OW	SOL	1203	43.320	6.460	49.320	1.00	0.00
ATOM	4732	HW1	SOL	1203	44.140	6.660	49.770	1.00	0.00
ATOM	4733	HW2	SOL	1203	43.260	5.510	49.360	1.00	0.00
ATOM	4734	OW	SOL	1204	30.620	40.940	19.770	1.00	0.00
ATOM	4735	HW1	SOL	1204	31.340	40.620	19.220	1.00	0.00
ATOM	4736	HW2	SOL	1204	30.190	41.600	19.240	1.00	0.00
ATOM	4737	OW	SOL	1205	49.050	53.440	34.740	1.00	0.00
ATOM	4738	HW1	SOL	1205	49.360	52.540	34.580	1.00	0.00
ATOM	4739	HW2	SOL	1205	49.480	53.960	34.060	1.00	0.00
ATOM	4740	OW	SOL	1206	12.400	2.300	25.600	1.00	0.00
ATOM	4741	HW1	SOL	1206	11.810	1.920	24.950	1.00	0.00
ATOM	4742	HW2	SOL	1206	13.050	1.620	25.760	1.00	0.00
ATOM	4743	OW	SOL	1207	39.150	16.950	7.320	1.00	0.00
ATOM	4744	HW1	SOL	1207	38.590	17.050	8.090	1.00	0.00
ATOM	4745	HW2	SOL	1207	39.960	16.590	7.660	1.00	0.00
ATOM	4746	OW	SOL	1208	23.630	20.230	9.340	1.00	0.00
ATOM	4747	HW1	SOL	1208	23.680	21.010	8.790	1.00	0.00
ATOM	4748	HW2	SOL	1208	24.430	19.750	9.150	1.00	0.00
ATOM	4749	OW	SOL	1209	8.210	43.920	12.520	1.00	0.00
ATOM	4750	HW1	SOL	1209	7.620	44.330	13.160	1.00	0.00
ATOM	4751	HW2	SOL	1209	8.100	42.980	12.670	1.00	0.00

ATOM	4752	OW	SOL	1210	34.920	24.730	52.990	1.00	0.00
ATOM	4753	HW1	SOL	1210	34.950	25.450	52.370	1.00	0.00
ATOM	4754	HW2	SOL	1210	34.540	25.110	53.780	1.00	0.00
ATOM	4755	OW	SOL	1211	38.920	17.140	29.470	1.00	0.00
ATOM	4756	HW1	SOL	1211	39.580	16.980	28.790	1.00	0.00
ATOM	4757	HW2	SOL	1211	38.520	17.980	29.210	1.00	0.00
ATOM	4758	OW	SOL	1212	17.120	37.620	11.540	1.00	0.00
ATOM	4759	HW1	SOL	1212	16.490	38.170	11.080	1.00	0.00
ATOM	4760	HW2	SOL	1212	17.980	37.950	11.260	1.00	0.00
ATOM	4761	OW	SOL	1213	14.580	15.610	9.100	1.00	0.00
ATOM	4762	HW1	SOL	1213	14.300	14.970	8.450	1.00	0.00
ATOM	4763	HW2	SOL	1213	15.230	16.140	8.660	1.00	0.00
ATOM	4764	OW	SOL	1214	4.810	19.110	42.990	1.00	0.00
ATOM	4765	HW1	SOL	1214	4.420	19.920	42.660	1.00	0.00
ATOM	4766	HW2	SOL	1214	5.510	18.920	42.370	1.00	0.00
ATOM	4767	OW	SOL	1215	28.100	40.290	16.730	1.00	0.00
ATOM	4768	HW1	SOL	1215	28.230	39.880	17.590	1.00	0.00
ATOM	4769	HW2	SOL	1215	27.220	40.000	16.470	1.00	0.00
ATOM	4770	OW	SOL	1216	37.980	9.610	19.060	1.00	0.00
ATOM	4771	HW1	SOL	1216	38.600	9.020	18.630	1.00	0.00
ATOM	4772	HW2	SOL	1216	37.140	9.160	19.010	1.00	0.00
ATOM	4773	OW	SOL	1217	11.120	27.960	4.270	1.00	0.00
ATOM	4774	HW1	SOL	1217	10.280	28.420	4.180	1.00	0.00
ATOM	4775	HW2	SOL	1217	10.930	27.250	4.880	1.00	0.00
ATOM	4776	OW	SOL	1218	43.030	11.470	52.520	1.00	0.00
ATOM	4777	HW1	SOL	1218	42.900	12.270	52.020	1.00	0.00
ATOM	4778	HW2	SOL	1218	42.630	10.780	51.980	1.00	0.00
ATOM	4779	OW	SOL	1219	1.600	50.400	24.480	1.00	0.00
ATOM	4780	HW1	SOL	1219	0.900	50.960	24.140	1.00	0.00
ATOM	4781	HW2	SOL	1219	1.160	49.860	25.150	1.00	0.00
ATOM	4782	OW	SOL	1220	23.360	34.460	50.990	1.00	0.00
ATOM	4783	HW1	SOL	1220	22.810	35.250	50.960	1.00	0.00
ATOM	4784	HW2	SOL	1220	24.200	34.740	50.640	1.00	0.00
ATOM	4785	OW	SOL	1221	45.720	5.770	8.350	1.00	0.00
ATOM	4786	HW1	SOL	1221	45.920	4.880	8.620	1.00	0.00
ATOM	4787	HW2	SOL	1221	45.610	5.720	7.400	1.00	0.00
ATOM	4788	OW	SOL	1222	53.690	22.290	29.170	1.00	0.00
ATOM	4789	HW1	SOL	1222	53.190	23.080	28.950	1.00	0.00
ATOM	4790	HW2	SOL	1222	53.590	22.210	30.120	1.00	0.00
ATOM	4791	OW	SOL	1223	13.000	36.990	49.000	1.00	0.00
ATOM	4792	HW1	SOL	1223	13.800	36.790	49.490	1.00	0.00
ATOM	4793	HW2	SOL	1223	12.630	36.130	48.790	1.00	0.00
ATOM	4794	OW	SOL	1224	30.860	43.490	35.740	1.00	0.00
ATOM	4795	HW1	SOL	1224	31.750	43.700	36.040	1.00	0.00

ATOM	4796	HW2	SOL	1224	30.950	42.670	35.260	1.00	0.00
ATOM	4797	OW	SOL	1225	8.500	7.100	16.940	1.00	0.00
ATOM	4798	HW1	SOL	1225	7.780	7.380	17.500	1.00	0.00
ATOM	4799	HW2	SOL	1225	8.660	6.190	17.190	1.00	0.00
ATOM	4800	OW	SOL	1226	31.150	23.790	54.250	1.00	0.00
ATOM	4801	HW1	SOL	1226	30.220	23.560	54.280	1.00	0.00
ATOM	4802	HW2	SOL	1226	31.610	22.960	54.430	1.00	0.00
ATOM	4803	OW	SOL	1227	0.190	22.020	8.040	1.00	0.00
ATOM	4804	HW1	SOL	1227	-0.230	22.650	7.460	1.00	0.00
ATOM	4805	HW2	SOL	1227	-0.160	22.230	8.910	1.00	0.00
ATOM	4806	OW	SOL	1228	26.500	22.380	42.440	1.00	0.00
ATOM	4807	HW1	SOL	1228	26.580	22.960	43.210	1.00	0.00
ATOM	4808	HW2	SOL	1228	25.610	22.520	42.130	1.00	0.00
ATOM	4809	OW	SOL	1229	7.460	41.150	26.840	1.00	0.00
ATOM	4810	HW1	SOL	1229	6.980	41.910	27.150	1.00	0.00
ATOM	4811	HW2	SOL	1229	8.320	41.230	27.240	1.00	0.00
ATOM	4812	OW	SOL	1230	34.090	37.900	53.290	1.00	0.00
ATOM	4813	HW1	SOL	1230	33.980	38.840	53.370	1.00	0.00
ATOM	4814	HW2	SOL	1230	33.200	37.550	53.250	1.00	0.00
ATOM	4815	OW	SOL	1231	13.260	20.140	22.840	1.00	0.00
ATOM	4816	HW1	SOL	1231	12.970	20.390	23.710	1.00	0.00
ATOM	4817	HW2	SOL	1231	13.840	19.390	22.980	1.00	0.00
ATOM	4818	OW	SOL	1232	0.140	5.140	47.390	1.00	0.00
ATOM	4819	HW1	SOL	1232	-0.230	5.000	46.510	1.00	0.00
ATOM	4820	HW2	SOL	1232	0.150	6.090	47.490	1.00	0.00
ATOM	4821	OW	SOL	1233	4.350	41.090	31.420	1.00	0.00
ATOM	4822	HW1	SOL	1233	3.920	41.710	30.830	1.00	0.00
ATOM	4823	HW2	SOL	1233	5.270	41.360	31.430	1.00	0.00
ATOM	4824	OW	SOL	1234	14.250	39.990	6.510	1.00	0.00
ATOM	4825	HW1	SOL	1234	13.670	39.290	6.810	1.00	0.00
ATOM	4826	HW2	SOL	1234	14.780	39.600	5.820	1.00	0.00
ATOM	4827	OW	SOL	1235	8.370	30.950	50.060	1.00	0.00
ATOM	4828	HW1	SOL	1235	8.470	30.010	50.210	1.00	0.00
ATOM	4829	HW2	SOL	1235	7.660	31.020	49.420	1.00	0.00
ATOM	4830	OW	SOL	1236	22.030	52.500	42.710	1.00	0.00
ATOM	4831	HW1	SOL	1236	22.350	53.400	42.750	1.00	0.00
ATOM	4832	HW2	SOL	1236	21.450	52.480	41.950	1.00	0.00
ATOM	4833	OW	SOL	1237	50.340	47.850	8.120	1.00	0.00
ATOM	4834	HW1	SOL	1237	50.970	48.580	8.120	1.00	0.00
ATOM	4835	HW2	SOL	1237	50.880	47.080	7.940	1.00	0.00
ATOM	4836	OW	SOL	1238	4.330	14.240	44.280	1.00	0.00
ATOM	4837	HW1	SOL	1238	5.280	14.120	44.370	1.00	0.00
ATOM	4838	HW2	SOL	1238	3.970	13.880	45.090	1.00	0.00
ATOM	4839	OW	SOL	1239	10.390	40.510	13.050	1.00	0.00

ATOM	4840	HW1	SOL	1239	10.130	40.800	12.180	1.00	0.00
ATOM	4841	HW2	SOL	1239	9.590	40.560	13.570	1.00	0.00
ATOM	4842	OW	SOL	1240	44.750	6.000	43.230	1.00	0.00
ATOM	4843	HW1	SOL	1240	44.620	5.060	43.160	1.00	0.00
ATOM	4844	HW2	SOL	1240	44.720	6.180	44.170	1.00	0.00
ATOM	4845	OW	SOL	1241	45.990	45.180	43.190	1.00	0.00
ATOM	4846	HW1	SOL	1241	45.180	44.690	43.260	1.00	0.00
ATOM	4847	HW2	SOL	1241	45.760	45.970	42.690	1.00	0.00
ATOM	4848	OW	SOL	1242	7.490	53.120	45.140	1.00	0.00
ATOM	4849	HW1	SOL	1242	6.930	53.620	45.730	1.00	0.00
ATOM	4850	HW2	SOL	1242	7.210	53.380	44.260	1.00	0.00
ATOM	4851	OW	SOL	1243	43.680	4.390	39.540	1.00	0.00
ATOM	4852	HW1	SOL	1243	43.160	4.350	40.340	1.00	0.00
ATOM	4853	HW2	SOL	1243	43.910	5.320	39.450	1.00	0.00
ATOM	4854	OW	SOL	1244	50.430	37.120	14.890	1.00	0.00
ATOM	4855	HW1	SOL	1244	49.750	37.590	15.390	1.00	0.00
ATOM	4856	HW2	SOL	1244	49.970	36.380	14.500	1.00	0.00
ATOM	4857	OW	SOL	1245	40.470	37.570	43.900	1.00	0.00
ATOM	4858	HW1	SOL	1245	41.380	37.410	44.120	1.00	0.00
ATOM	4859	HW2	SOL	1245	40.470	38.460	43.530	1.00	0.00
ATOM	4860	OW	SOL	1246	50.330	1.860	38.860	1.00	0.00
ATOM	4861	HW1	SOL	1246	50.900	1.090	38.850	1.00	0.00
ATOM	4862	HW2	SOL	1246	49.630	1.630	39.460	1.00	0.00
ATOM	4863	OW	SOL	1247	27.860	12.170	9.270	1.00	0.00
ATOM	4864	HW1	SOL	1247	28.010	12.760	10.010	1.00	0.00
ATOM	4865	HW2	SOL	1247	28.470	12.480	8.600	1.00	0.00
ATOM	4866	OW	SOL	1248	20.810	28.670	35.780	1.00	0.00
ATOM	4867	HW1	SOL	1248	20.860	29.100	36.640	1.00	0.00
ATOM	4868	HW2	SOL	1248	20.410	29.320	35.210	1.00	0.00
ATOM	4869	OW	SOL	1249	14.190	12.170	52.460	1.00	0.00
ATOM	4870	HW1	SOL	1249	13.240	12.110	52.460	1.00	0.00
ATOM	4871	HW2	SOL	1249	14.480	11.390	52.930	1.00	0.00
ATOM	4872	OW	SOL	1250	51.210	12.610	49.910	1.00	0.00
ATOM	4873	HW1	SOL	1250	50.830	13.100	49.180	1.00	0.00
ATOM	4874	HW2	SOL	1250	52.070	12.350	49.590	1.00	0.00
ATOM	4875	OW	SOL	1251	24.620	15.580	22.730	1.00	0.00
ATOM	4876	HW1	SOL	1251	24.920	16.060	21.960	1.00	0.00
ATOM	4877	HW2	SOL	1251	25.000	16.050	23.470	1.00	0.00
ATOM	4878	OW	SOL	1252	8.540	12.190	52.020	1.00	0.00
ATOM	4879	HW1	SOL	1252	7.770	11.980	52.550	1.00	0.00
ATOM	4880	HW2	SOL	1252	8.220	12.770	51.330	1.00	0.00
ATOM	4881	OW	SOL	1253	45.450	30.040	13.060	1.00	0.00
ATOM	4882	HW1	SOL	1253	44.900	29.280	12.890	1.00	0.00
ATOM	4883	HW2	SOL	1253	44.860	30.790	12.990	1.00	0.00

ATOM	4884	OW	SOL	1254	53.110	33.940	45.440	1.00	0.00
ATOM	4885	HW1	SOL	1254	53.650	34.730	45.510	1.00	0.00
ATOM	4886	HW2	SOL	1254	53.580	33.280	45.940	1.00	0.00
ATOM	4887	OW	SOL	1255	12.050	34.390	30.390	1.00	0.00
ATOM	4888	HW1	SOL	1255	11.870	35.240	30.790	1.00	0.00
ATOM	4889	HW2	SOL	1255	12.910	34.490	29.980	1.00	0.00
ATOM	4890	OW	SOL	1256	49.820	40.910	9.140	1.00	0.00
ATOM	4891	HW1	SOL	1256	49.540	40.100	9.560	1.00	0.00
ATOM	4892	HW2	SOL	1256	49.570	41.600	9.770	1.00	0.00
ATOM	4893	OW	SOL	1257	5.910	35.080	42.130	1.00	0.00
ATOM	4894	HW1	SOL	1257	5.530	34.460	41.500	1.00	0.00
ATOM	4895	HW2	SOL	1257	6.100	35.860	41.620	1.00	0.00
ATOM	4896	OW	SOL	1258	1.120	6.680	15.980	1.00	0.00
ATOM	4897	HW1	SOL	1258	0.460	6.940	15.350	1.00	0.00
ATOM	4898	HW2	SOL	1258	0.640	6.550	16.800	1.00	0.00
ATOM	4899	OW	SOL	1259	52.080	53.200	21.700	1.00	0.00
ATOM	4900	HW1	SOL	1259	52.180	53.130	20.750	1.00	0.00
ATOM	4901	HW2	SOL	1259	51.150	53.090	21.850	1.00	0.00
ATOM	4902	OW	SOL	1260	20.910	33.460	12.960	1.00	0.00
ATOM	4903	HW1	SOL	1260	20.900	34.280	13.440	1.00	0.00
ATOM	4904	HW2	SOL	1260	20.000	33.180	12.920	1.00	0.00
ATOM	4905	OW	SOL	1261	45.790	19.090	7.020	1.00	0.00
ATOM	4906	HW1	SOL	1261	45.670	19.790	7.660	1.00	0.00
ATOM	4907	HW2	SOL	1261	46.710	19.130	6.790	1.00	0.00
ATOM	4908	OW	SOL	1262	53.290	8.750	45.990	1.00	0.00
ATOM	4909	HW1	SOL	1262	53.450	9.560	45.500	1.00	0.00
ATOM	4910	HW2	SOL	1262	52.800	8.200	45.370	1.00	0.00
ATOM	4911	OW	SOL	1263	37.080	29.610	37.890	1.00	0.00
ATOM	4912	HW1	SOL	1263	36.370	28.970	37.770	1.00	0.00
ATOM	4913	HW2	SOL	1263	36.840	30.090	38.680	1.00	0.00
ATOM	4914	OW	SOL	1264	2.010	39.560	52.040	1.00	0.00
ATOM	4915	HW1	SOL	1264	2.540	40.270	52.420	1.00	0.00
ATOM	4916	HW2	SOL	1264	1.630	39.940	51.260	1.00	0.00
ATOM	4917	OW	SOL	1265	14.750	23.070	20.110	1.00	0.00
ATOM	4918	HW1	SOL	1265	14.290	23.900	20.140	1.00	0.00
ATOM	4919	HW2	SOL	1265	15.170	23.060	19.250	1.00	0.00
ATOM	4920	OW	SOL	1266	14.120	36.070	55.570	1.00	0.00
ATOM	4921	HW1	SOL	1266	14.450	35.720	56.400	1.00	0.00
ATOM	4922	HW2	SOL	1266	13.790	35.310	55.100	1.00	0.00
ATOM	4923	OW	SOL	1267	30.590	31.920	21.110	1.00	0.00
ATOM	4924	HW1	SOL	1267	31.200	32.410	21.660	1.00	0.00
ATOM	4925	HW2	SOL	1267	30.130	31.340	21.710	1.00	0.00
ATOM	4926	OW	SOL	1268	25.340	52.060	12.650	1.00	0.00
ATOM	4927	HW1	SOL	1268	25.860	51.300	12.380	1.00	0.00

ATOM	4928	HW2	SOL	1268	25.930	52.570	13.200	1.00	0.00
ATOM	4929	OW	SOL	1269	9.190	48.750	7.340	1.00	0.00
ATOM	4930	HW1	SOL	1269	9.270	48.100	6.640	1.00	0.00
ATOM	4931	HW2	SOL	1269	9.980	49.290	7.260	1.00	0.00
ATOM	4932	OW	SOL	1270	50.670	35.450	22.950	1.00	0.00
ATOM	4933	HW1	SOL	1270	50.080	35.300	22.220	1.00	0.00
ATOM	4934	HW2	SOL	1270	51.540	35.250	22.600	1.00	0.00
ATOM	4935	OW	SOL	1271	14.900	46.800	44.020	1.00	0.00
ATOM	4936	HW1	SOL	1271	15.230	47.680	43.840	1.00	0.00
ATOM	4937	HW2	SOL	1271	14.300	46.920	44.760	1.00	0.00
ATOM	4938	OW	SOL	1272	14.740	5.650	13.370	1.00	0.00
ATOM	4939	HW1	SOL	1272	14.860	4.700	13.290	1.00	0.00
ATOM	4940	HW2	SOL	1272	14.250	5.890	12.580	1.00	0.00
ATOM	4941	OW	SOL	1273	50.230	7.370	14.900	1.00	0.00
ATOM	4942	HW1	SOL	1273	49.790	8.050	15.400	1.00	0.00
ATOM	4943	HW2	SOL	1273	51.010	7.790	14.530	1.00	0.00
ATOM	4944	OW	SOL	1274	3.190	19.370	29.760	1.00	0.00
ATOM	4945	HW1	SOL	1274	3.170	19.510	28.810	1.00	0.00
ATOM	4946	HW2	SOL	1274	2.330	19.670	30.060	1.00	0.00
ATOM	4947	OW	SOL	1275	37.610	38.220	4.350	1.00	0.00
ATOM	4948	HW1	SOL	1275	38.110	38.560	5.100	1.00	0.00
ATOM	4949	HW2	SOL	1275	37.200	38.990	3.970	1.00	0.00
ATOM	4950	OW	SOL	1276	0.840	46.620	18.680	1.00	0.00
ATOM	4951	HW1	SOL	1276	0.540	46.390	17.800	1.00	0.00
ATOM	4952	HW2	SOL	1276	0.080	46.450	19.240	1.00	0.00
ATOM	4953	OW	SOL	1277	45.600	17.800	52.010	1.00	0.00
ATOM	4954	HW1	SOL	1277	45.970	17.540	52.850	1.00	0.00
ATOM	4955	HW2	SOL	1277	45.980	18.660	51.830	1.00	0.00
ATOM	4956	OW	SOL	1278	7.490	32.450	42.580	1.00	0.00
ATOM	4957	HW1	SOL	1278	6.940	33.000	43.140	1.00	0.00
ATOM	4958	HW2	SOL	1278	7.510	32.910	41.740	1.00	0.00
ATOM	4959	OW	SOL	1279	21.430	1.880	26.180	1.00	0.00
ATOM	4960	HW1	SOL	1279	21.640	1.110	26.710	1.00	0.00
ATOM	4961	HW2	SOL	1279	22.280	2.230	25.920	1.00	0.00
ATOM	4962	OW	SOL	1280	13.640	11.370	36.870	1.00	0.00
ATOM	4963	HW1	SOL	1280	13.300	10.750	37.510	1.00	0.00
ATOM	4964	HW2	SOL	1280	13.510	10.950	36.030	1.00	0.00
ATOM	4965	OW	SOL	1281	50.540	9.990	49.720	1.00	0.00
ATOM	4966	HW1	SOL	1281	50.650	10.940	49.650	1.00	0.00
ATOM	4967	HW2	SOL	1281	50.210	9.720	48.860	1.00	0.00
ATOM	4968	OW	SOL	1282	18.120	1.100	34.830	1.00	0.00
ATOM	4969	HW1	SOL	1282	17.880	1.290	33.920	1.00	0.00
ATOM	4970	HW2	SOL	1282	18.250	1.960	35.230	1.00	0.00
ATOM	4971	OW	SOL	1283	1.940	40.150	9.840	1.00	0.00

ATOM	4972	HW1	SOL	1283	1.310	40.010	10.550	1.00	0.00
ATOM	4973	HW2	SOL	1283	2.490	39.370	9.850	1.00	0.00
ATOM	4974	OW	SOL	1284	27.050	17.700	53.420	1.00	0.00
ATOM	4975	HW1	SOL	1284	27.100	17.400	52.510	1.00	0.00
ATOM	4976	HW2	SOL	1284	26.110	17.840	53.570	1.00	0.00
ATOM	4977	OW	SOL	1285	27.170	19.670	40.610	1.00	0.00
ATOM	4978	HW1	SOL	1285	26.730	18.850	40.840	1.00	0.00
ATOM	4979	HW2	SOL	1285	26.460	20.310	40.530	1.00	0.00
ATOM	4980	OW	SOL	1286	20.510	39.420	11.750	1.00	0.00
ATOM	4981	HW1	SOL	1286	20.910	38.860	12.420	1.00	0.00
ATOM	4982	HW2	SOL	1286	19.730	39.780	12.180	1.00	0.00
ATOM	4983	OW	SOL	1287	20.310	49.200	45.950	1.00	0.00
ATOM	4984	HW1	SOL	1287	20.740	49.870	45.420	1.00	0.00
ATOM	4985	HW2	SOL	1287	20.100	49.630	46.770	1.00	0.00
ATOM	4986	OW	SOL	1288	30.810	39.180	22.190	1.00	0.00
ATOM	4987	HW1	SOL	1288	30.770	39.640	21.350	1.00	0.00
ATOM	4988	HW2	SOL	1288	31.730	38.940	22.290	1.00	0.00
ATOM	4989	OW	SOL	1289	12.320	23.810	28.950	1.00	0.00
ATOM	4990	HW1	SOL	1289	13.030	23.810	29.590	1.00	0.00
ATOM	4991	HW2	SOL	1289	12.640	24.360	28.240	1.00	0.00
ATOM	4992	OW	SOL	1290	15.880	37.460	24.830	1.00	0.00
ATOM	4993	HW1	SOL	1290	16.590	36.820	24.880	1.00	0.00
ATOM	4994	HW2	SOL	1290	16.180	38.190	25.370	1.00	0.00
ATOM	4995	OW	SOL	1291	37.150	8.560	30.270	1.00	0.00
ATOM	4996	HW1	SOL	1291	37.250	8.280	31.180	1.00	0.00
ATOM	4997	HW2	SOL	1291	37.850	9.210	30.140	1.00	0.00
ATOM	4998	OW	SOL	1292	6.930	2.870	12.560	1.00	0.00
ATOM	4999	HW1	SOL	1292	6.870	3.490	11.830	1.00	0.00
ATOM	5000	HW2	SOL	1292	6.360	2.150	12.300	1.00	0.00
ATOM	5001	OW	SOL	1293	9.690	31.670	46.490	1.00	0.00
ATOM	5002	HW1	SOL	1293	9.250	31.280	45.740	1.00	0.00
ATOM	5003	HW2	SOL	1293	9.880	30.920	47.070	1.00	0.00
ATOM	5004	OW	SOL	1294	17.920	7.460	2.940	1.00	0.00
ATOM	5005	HW1	SOL	1294	18.530	7.910	3.530	1.00	0.00
ATOM	5006	HW2	SOL	1294	18.090	6.530	3.090	1.00	0.00
ATOM	5007	OW	SOL	1295	5.940	47.320	24.570	1.00	0.00
ATOM	5008	HW1	SOL	1295	6.630	47.280	25.240	1.00	0.00
ATOM	5009	HW2	SOL	1295	6.380	47.110	23.750	1.00	0.00
ATOM	5010	OW	SOL	1296	23.150	36.390	5.090	1.00	0.00
ATOM	5011	HW1	SOL	1296	23.840	35.730	4.980	1.00	0.00
ATOM	5012	HW2	SOL	1296	22.860	36.280	6.000	1.00	0.00
ATOM	5013	OW	SOL	1297	0.420	8.200	27.490	1.00	0.00
ATOM	5014	HW1	SOL	1297	1.130	8.840	27.520	1.00	0.00
ATOM	5015	HW2	SOL	1297	0.460	7.760	28.340	1.00	0.00

ATOM	5016	OW	SOL	1298	11.980	2.000	40.390	1.00	0.00
ATOM	5017	HW1	SOL	1298	11.800	1.100	40.660	1.00	0.00
ATOM	5018	HW2	SOL	1298	12.820	2.210	40.790	1.00	0.00
ATOM	5019	OW	SOL	1299	1.840	3.450	8.480	1.00	0.00
ATOM	5020	HW1	SOL	1299	2.190	2.940	9.200	1.00	0.00
ATOM	5021	HW2	SOL	1299	0.900	3.230	8.460	1.00	0.00
ATOM	5022	OW	SOL	1300	38.680	19.580	0.410	1.00	0.00
ATOM	5023	HW1	SOL	1300	38.260	18.770	0.120	1.00	0.00
ATOM	5024	HW2	SOL	1300	39.590	19.500	0.110	1.00	0.00
ATOM	5025	OW	SOL	1301	44.180	49.420	6.040	1.00	0.00
ATOM	5026	HW1	SOL	1301	43.900	48.940	5.270	1.00	0.00
ATOM	5027	HW2	SOL	1301	43.650	50.220	6.030	1.00	0.00
ATOM	5028	OW	SOL	1302	37.230	28.740	5.750	1.00	0.00
ATOM	5029	HW1	SOL	1302	37.020	29.080	6.620	1.00	0.00
ATOM	5030	HW2	SOL	1302	36.720	27.940	5.680	1.00	0.00
ATOM	5031	OW	SOL	1303	12.000	46.830	12.980	1.00	0.00
ATOM	5032	HW1	SOL	1303	11.290	47.430	13.180	1.00	0.00
ATOM	5033	HW2	SOL	1303	12.260	46.480	13.840	1.00	0.00
ATOM	5034	OW	SOL	1304	11.390	53.620	27.740	1.00	0.00
ATOM	5035	HW1	SOL	1304	10.660	53.050	27.960	1.00	0.00
ATOM	5036	HW2	SOL	1304	11.550	54.130	28.530	1.00	0.00
ATOM	5037	OW	SOL	1305	22.390	51.130	28.330	1.00	0.00
ATOM	5038	HW1	SOL	1305	22.160	51.270	29.250	1.00	0.00
ATOM	5039	HW2	SOL	1305	21.540	51.080	27.880	1.00	0.00
ATOM	5040	OW	SOL	1306	39.410	45.040	5.570	1.00	0.00
ATOM	5041	HW1	SOL	1306	40.020	44.410	5.970	1.00	0.00
ATOM	5042	HW2	SOL	1306	38.880	44.510	4.980	1.00	0.00
ATOM	5043	OW	SOL	1307	18.710	7.900	52.740	1.00	0.00
ATOM	5044	HW1	SOL	1307	18.880	8.580	52.090	1.00	0.00
ATOM	5045	HW2	SOL	1307	19.560	7.500	52.900	1.00	0.00
ATOM	5046	OW	SOL	1308	0.670	31.490	14.980	1.00	0.00
ATOM	5047	HW1	SOL	1308	1.400	31.490	15.600	1.00	0.00
ATOM	5048	HW2	SOL	1308	0.640	32.390	14.650	1.00	0.00
ATOM	5049	OW	SOL	1309	51.870	20.630	54.500	1.00	0.00
ATOM	5050	HW1	SOL	1309	52.300	20.090	53.840	1.00	0.00
ATOM	5051	HW2	SOL	1309	51.180	20.070	54.860	1.00	0.00
ATOM	5052	OW	SOL	1310	5.300	25.610	1.980	1.00	0.00
ATOM	5053	HW1	SOL	1310	5.800	26.420	1.890	1.00	0.00
ATOM	5054	HW2	SOL	1310	4.920	25.670	2.850	1.00	0.00
ATOM	5055	OW	SOL	1311	33.870	3.810	20.950	1.00	0.00
ATOM	5056	HW1	SOL	1311	34.620	4.320	20.670	1.00	0.00
ATOM	5057	HW2	SOL	1311	33.120	4.210	20.490	1.00	0.00
ATOM	5058	OW	SOL	1312	22.500	42.460	25.580	1.00	0.00
ATOM	5059	HW1	SOL	1312	23.350	42.890	25.440	1.00	0.00

ATOM	5060	HW2	SOL	1312	21.880	43.000	25.090	1.00	0.00
ATOM	5061	OW	SOL	1313	15.570	23.460	25.560	1.00	0.00
ATOM	5062	HW1	SOL	1313	15.860	24.340	25.310	1.00	0.00
ATOM	5063	HW2	SOL	1313	15.940	23.330	26.430	1.00	0.00
ATOM	5064	OW	SOL	1314	52.990	49.760	45.550	1.00	0.00
ATOM	5065	HW1	SOL	1314	52.600	49.170	44.910	1.00	0.00
ATOM	5066	HW2	SOL	1314	53.840	49.370	45.750	1.00	0.00
ATOM	5067	OW	SOL	1315	25.420	0.830	13.890	1.00	0.00
ATOM	5068	HW1	SOL	1315	25.380	1.580	13.290	1.00	0.00
ATOM	5069	HW2	SOL	1315	25.350	0.060	13.320	1.00	0.00
ATOM	5070	OW	SOL	1316	44.530	2.330	11.100	1.00	0.00
ATOM	5071	HW1	SOL	1316	45.300	2.490	10.550	1.00	0.00
ATOM	5072	HW2	SOL	1316	44.890	2.020	11.930	1.00	0.00
ATOM	5073	OW	SOL	1317	25.600	32.790	43.190	1.00	0.00
ATOM	5074	HW1	SOL	1317	26.510	33.040	43.300	1.00	0.00
ATOM	5075	HW2	SOL	1317	25.430	32.180	43.910	1.00	0.00
ATOM	5076	OW	SOL	1318	3.660	51.060	39.280	1.00	0.00
ATOM	5077	HW1	SOL	1318	3.730	51.860	38.770	1.00	0.00
ATOM	5078	HW2	SOL	1318	3.090	50.490	38.770	1.00	0.00
ATOM	5079	OW	SOL	1319	43.850	19.780	4.830	1.00	0.00
ATOM	5080	HW1	SOL	1319	43.920	19.000	5.380	1.00	0.00
ATOM	5081	HW2	SOL	1319	43.730	20.500	5.460	1.00	0.00
ATOM	5082	OW	SOL	1320	17.780	40.460	28.870	1.00	0.00
ATOM	5083	HW1	SOL	1320	17.650	39.990	29.690	1.00	0.00
ATOM	5084	HW2	SOL	1320	18.400	39.920	28.380	1.00	0.00
ATOM	5085	OW	SOL	1321	41.730	54.720	41.220	1.00	0.00
ATOM	5086	HW1	SOL	1321	41.800	54.960	42.140	1.00	0.00
ATOM	5087	HW2	SOL	1321	40.830	54.400	41.120	1.00	0.00
ATOM	5088	OW	SOL	1322	52.650	52.890	7.490	1.00	0.00
ATOM	5089	HW1	SOL	1322	51.950	52.270	7.290	1.00	0.00
ATOM	5090	HW2	SOL	1322	52.810	53.350	6.670	1.00	0.00
ATOM	5091	OW	SOL	1323	47.480	23.550	11.430	1.00	0.00
ATOM	5092	HW1	SOL	1323	47.420	24.480	11.640	1.00	0.00
ATOM	5093	HW2	SOL	1323	46.640	23.340	11.000	1.00	0.00
ATOM	5094	OW	SOL	1324	41.890	13.650	38.880	1.00	0.00
ATOM	5095	HW1	SOL	1324	42.620	14.060	39.340	1.00	0.00
ATOM	5096	HW2	SOL	1324	41.360	13.250	39.570	1.00	0.00
ATOM	5097	OW	SOL	1325	7.400	29.910	25.090	1.00	0.00
ATOM	5098	HW1	SOL	1325	6.910	29.510	25.820	1.00	0.00
ATOM	5099	HW2	SOL	1325	8.220	30.200	25.490	1.00	0.00
ATOM	5100	OW	SOL	1326	32.000	17.290	4.000	1.00	0.00
ATOM	5101	HW1	SOL	1326	31.080	17.400	3.740	1.00	0.00
ATOM	5102	HW2	SOL	1326	32.500	17.650	3.260	1.00	0.00
ATOM	5103	OW	SOL	1327	3.580	43.970	9.610	1.00	0.00

ATOM	5104	HW1	SOL	1327	3.090	44.030	10.430	1.00	0.00
ATOM	5105	HW2	SOL	1327	4.390	44.450	9.770	1.00	0.00
ATOM	5106	OW	SOL	1328	25.630	19.040	46.810	1.00	0.00
ATOM	5107	HW1	SOL	1328	25.940	19.490	46.030	1.00	0.00
ATOM	5108	HW2	SOL	1328	25.970	19.560	47.540	1.00	0.00
ATOM	5109	OW	SOL	1329	27.080	25.850	39.420	1.00	0.00
ATOM	5110	HW1	SOL	1329	26.940	25.030	39.900	1.00	0.00
ATOM	5111	HW2	SOL	1329	27.700	26.340	39.970	1.00	0.00
ATOM	5112	OW	SOL	1330	9.320	19.590	26.690	1.00	0.00
ATOM	5113	HW1	SOL	1330	8.650	18.900	26.690	1.00	0.00
ATOM	5114	HW2	SOL	1330	10.140	19.110	26.830	1.00	0.00
ATOM	5115	OW	SOL	1331	36.610	49.140	8.700	1.00	0.00
ATOM	5116	HW1	SOL	1331	36.010	49.870	8.880	1.00	0.00
ATOM	5117	HW2	SOL	1331	37.410	49.350	9.180	1.00	0.00
ATOM	5118	OW	SOL	1332	45.830	9.780	15.380	1.00	0.00
ATOM	5119	HW1	SOL	1332	46.160	9.400	16.200	1.00	0.00
ATOM	5120	HW2	SOL	1332	46.520	10.380	15.110	1.00	0.00
ATOM	5121	OW	SOL	1333	2.630	54.810	52.740	1.00	0.00
ATOM	5122	HW1	SOL	1333	2.650	53.850	52.680	1.00	0.00
ATOM	5123	HW2	SOL	1333	2.800	55.110	51.850	1.00	0.00
ATOM	5124	OW	SOL	1334	14.840	20.950	4.190	1.00	0.00
ATOM	5125	HW1	SOL	1334	14.300	20.510	4.850	1.00	0.00
ATOM	5126	HW2	SOL	1334	14.430	20.720	3.350	1.00	0.00
ATOM	5127	OW	SOL	1335	9.730	25.730	49.730	1.00	0.00
ATOM	5128	HW1	SOL	1335	9.340	26.270	49.050	1.00	0.00
ATOM	5129	HW2	SOL	1335	10.080	24.970	49.270	1.00	0.00
ATOM	5130	OW	SOL	1336	40.720	43.900	18.610	1.00	0.00
ATOM	5131	HW1	SOL	1336	41.260	44.020	19.390	1.00	0.00
ATOM	5132	HW2	SOL	1336	41.110	44.490	17.960	1.00	0.00
ATOM	5133	OW	SOL	1337	44.190	6.430	3.440	1.00	0.00
ATOM	5134	HW1	SOL	1337	43.540	6.740	2.810	1.00	0.00
ATOM	5135	HW2	SOL	1337	44.550	5.640	3.050	1.00	0.00
ATOM	5136	OW	SOL	1338	14.960	52.180	41.340	1.00	0.00
ATOM	5137	HW1	SOL	1338	15.270	52.960	40.880	1.00	0.00
ATOM	5138	HW2	SOL	1338	15.740	51.810	41.750	1.00	0.00
ATOM	5139	OW	SOL	1339	2.920	32.940	12.780	1.00	0.00
ATOM	5140	HW1	SOL	1339	3.450	33.640	12.410	1.00	0.00
ATOM	5141	HW2	SOL	1339	2.160	33.380	13.160	1.00	0.00
ATOM	5142	OW	SOL	1340	27.910	39.710	19.290	1.00	0.00
ATOM	5143	HW1	SOL	1340	28.680	40.250	19.440	1.00	0.00
ATOM	5144	HW2	SOL	1340	27.320	39.940	20.010	1.00	0.00
ATOM	5145	OW	SOL	1341	37.250	6.440	9.400	1.00	0.00
ATOM	5146	HW1	SOL	1341	37.340	7.320	9.770	1.00	0.00
ATOM	5147	HW2	SOL	1341	37.250	6.580	8.450	1.00	0.00

ATOM	5148	OW	SOL	1342	25.530	39.860	0.140	1.00	0.00
ATOM	5149	HW1	SOL	1342	25.230	40.610	0.670	1.00	0.00
ATOM	5150	HW2	SOL	1342	25.020	39.120	0.470	1.00	0.00
ATOM	5151	OW	SOL	1343	52.550	14.040	39.330	1.00	0.00
ATOM	5152	HW1	SOL	1343	52.780	13.750	40.210	1.00	0.00
ATOM	5153	HW2	SOL	1343	53.190	14.730	39.140	1.00	0.00
ATOM	5154	OW	SOL	1344	29.040	13.210	45.990	1.00	0.00
ATOM	5155	HW1	SOL	1344	29.880	12.850	46.280	1.00	0.00
ATOM	5156	HW2	SOL	1344	29.210	14.140	45.850	1.00	0.00
ATOM	5157	OW	SOL	1345	13.780	32.950	20.930	1.00	0.00
ATOM	5158	HW1	SOL	1345	13.970	33.790	20.520	1.00	0.00
ATOM	5159	HW2	SOL	1345	12.820	32.940	21.010	1.00	0.00
ATOM	5160	OW	SOL	1346	2.540	35.170	3.780	1.00	0.00
ATOM	5161	HW1	SOL	1346	2.650	35.270	2.830	1.00	0.00
ATOM	5162	HW2	SOL	1346	3.430	35.200	4.130	1.00	0.00
ATOM	5163	OW	SOL	1347	32.880	18.960	2.220	1.00	0.00
ATOM	5164	HW1	SOL	1347	32.750	19.910	2.270	1.00	0.00
ATOM	5165	HW2	SOL	1347	33.810	18.830	2.400	1.00	0.00
ATOM	5166	OW	SOL	1348	3.100	5.930	23.350	1.00	0.00
ATOM	5167	HW1	SOL	1348	2.890	5.110	23.790	1.00	0.00
ATOM	5168	HW2	SOL	1348	2.810	6.610	23.960	1.00	0.00
ATOM	5169	OW	SOL	1349	43.460	12.280	25.480	1.00	0.00
ATOM	5170	HW1	SOL	1349	43.970	12.410	24.680	1.00	0.00
ATOM	5171	HW2	SOL	1349	43.190	11.370	25.450	1.00	0.00
ATOM	5172	OW	SOL	1350	15.970	35.380	53.420	1.00	0.00
ATOM	5173	HW1	SOL	1350	16.110	35.910	54.210	1.00	0.00
ATOM	5174	HW2	SOL	1350	15.030	35.410	53.270	1.00	0.00
ATOM	5175	OW	SOL	1351	37.960	20.590	40.670	1.00	0.00
ATOM	5176	HW1	SOL	1351	37.120	20.330	40.290	1.00	0.00
ATOM	5177	HW2	SOL	1351	38.090	21.490	40.380	1.00	0.00
ATOM	5178	OW	SOL	1352	55.130	40.550	48.050	1.00	0.00
ATOM	5179	HW1	SOL	1352	54.450	40.760	48.690	1.00	0.00
ATOM	5180	HW2	SOL	1352	55.190	41.320	47.500	1.00	0.00
ATOM	5181	OW	SOL	1353	25.350	36.100	7.630	1.00	0.00
ATOM	5182	HW1	SOL	1353	25.170	35.740	6.760	1.00	0.00
ATOM	5183	HW2	SOL	1353	26.200	36.540	7.530	1.00	0.00
ATOM	5184	OW	SOL	1354	47.520	41.850	49.870	1.00	0.00
ATOM	5185	HW1	SOL	1354	48.030	42.390	50.470	1.00	0.00
ATOM	5186	HW2	SOL	1354	47.930	41.990	49.010	1.00	0.00
ATOM	5187	OW	SOL	1355	5.560	35.780	15.380	1.00	0.00
ATOM	5188	HW1	SOL	1355	5.490	36.540	15.960	1.00	0.00
ATOM	5189	HW2	SOL	1355	6.490	35.540	15.410	1.00	0.00
ATOM	5190	OW	SOL	1356	24.100	43.020	7.770	1.00	0.00
ATOM	5191	HW1	SOL	1356	24.040	42.220	8.300	1.00	0.00

ATOM	5192	HW2	SOL	1356	24.830	43.510	8.160	1.00	0.00
ATOM	5193	OW	SOL	1357	8.140	19.990	45.670	1.00	0.00
ATOM	5194	HW1	SOL	1357	8.350	20.730	45.100	1.00	0.00
ATOM	5195	HW2	SOL	1357	8.930	19.880	46.220	1.00	0.00
ATOM	5196	OW	SOL	1358	41.660	39.210	3.060	1.00	0.00
ATOM	5197	HW1	SOL	1358	42.370	38.720	2.650	1.00	0.00
ATOM	5198	HW2	SOL	1358	42.100	39.930	3.510	1.00	0.00
ATOM	5199	OW	SOL	1359	16.520	5.050	4.610	1.00	0.00
ATOM	5200	HW1	SOL	1359	16.790	4.810	5.500	1.00	0.00
ATOM	5201	HW2	SOL	1359	16.370	4.210	4.170	1.00	0.00
ATOM	5202	OW	SOL	1360	24.130	5.660	2.010	1.00	0.00
ATOM	5203	HW1	SOL	1360	24.200	4.710	2.000	1.00	0.00
ATOM	5204	HW2	SOL	1360	23.600	5.870	1.230	1.00	0.00
ATOM	5205	OW	SOL	1361	44.500	51.730	27.000	1.00	0.00
ATOM	5206	HW1	SOL	1361	43.880	51.110	27.370	1.00	0.00
ATOM	5207	HW2	SOL	1361	44.370	51.660	26.050	1.00	0.00
ATOM	5208	OW	SOL	1362	6.410	2.660	42.850	1.00	0.00
ATOM	5209	HW1	SOL	1362	5.740	2.580	43.530	1.00	0.00
ATOM	5210	HW2	SOL	1362	5.960	2.470	42.040	1.00	0.00
ATOM	5211	OW	SOL	1363	34.940	2.710	36.400	1.00	0.00
ATOM	5212	HW1	SOL	1363	34.850	2.810	37.350	1.00	0.00
ATOM	5213	HW2	SOL	1363	34.270	3.300	36.040	1.00	0.00
ATOM	5214	OW	SOL	1364	1.860	30.850	40.960	1.00	0.00
ATOM	5215	HW1	SOL	1364	1.450	31.490	40.390	1.00	0.00
ATOM	5216	HW2	SOL	1364	2.340	30.270	40.370	1.00	0.00
ATOM	5217	OW	SOL	1365	10.440	52.610	32.930	1.00	0.00
ATOM	5218	HW1	SOL	1365	11.090	53.290	33.090	1.00	0.00
ATOM	5219	HW2	SOL	1365	10.080	52.410	33.790	1.00	0.00
ATOM	5220	OW	SOL	1366	19.350	29.740	17.730	1.00	0.00
ATOM	5221	HW1	SOL	1366	18.710	29.490	17.070	1.00	0.00
ATOM	5222	HW2	SOL	1366	19.370	29.010	18.340	1.00	0.00
ATOM	5223	OW	SOL	1367	37.920	28.040	17.970	1.00	0.00
ATOM	5224	HW1	SOL	1367	38.560	28.660	18.310	1.00	0.00
ATOM	5225	HW2	SOL	1367	38.210	27.180	18.280	1.00	0.00
ATOM	5226	OW	SOL	1368	25.470	27.240	49.140	1.00	0.00
ATOM	5227	HW1	SOL	1368	25.280	27.590	48.270	1.00	0.00
ATOM	5228	HW2	SOL	1368	26.430	27.230	49.200	1.00	0.00
ATOM	5229	OW	SOL	1369	43.540	39.670	14.940	1.00	0.00
ATOM	5230	HW1	SOL	1369	43.670	40.580	14.690	1.00	0.00
ATOM	5231	HW2	SOL	1369	43.610	39.180	14.110	1.00	0.00
ATOM	5232	OW	SOL	1370	34.640	54.400	25.090	1.00	0.00
ATOM	5233	HW1	SOL	1370	35.090	54.880	25.780	1.00	0.00
ATOM	5234	HW2	SOL	1370	35.300	54.300	24.400	1.00	0.00
ATOM	5235	OW	SOL	1371	53.670	55.740	18.520	1.00	0.00

ATOM	5236	HW1	SOL	1371	54.450	56.250	18.750	1.00	0.00
ATOM	5237	HW2	SOL	1371	53.100	56.350	18.050	1.00	0.00
ATOM	5238	OW	SOL	1372	39.130	55.470	48.970	1.00	0.00
ATOM	5239	HW1	SOL	1372	38.360	56.000	49.170	1.00	0.00
ATOM	5240	HW2	SOL	1372	39.750	55.660	49.680	1.00	0.00
ATOM	5241	OW	SOL	1373	17.650	26.030	51.340	1.00	0.00
ATOM	5242	HW1	SOL	1373	17.000	26.180	52.030	1.00	0.00
ATOM	5243	HW2	SOL	1373	17.140	26.070	50.520	1.00	0.00
ATOM	5244	OW	SOL	1374	7.030	46.030	33.810	1.00	0.00
ATOM	5245	HW1	SOL	1374	7.030	46.320	32.900	1.00	0.00
ATOM	5246	HW2	SOL	1374	7.390	45.140	33.790	1.00	0.00
ATOM	5247	OW	SOL	1375	53.750	23.630	35.170	1.00	0.00
ATOM	5248	HW1	SOL	1375	54.590	24.020	35.400	1.00	0.00
ATOM	5249	HW2	SOL	1375	53.750	22.780	35.610	1.00	0.00
ATOM	5250	OW	SOL	1376	26.840	24.980	34.530	1.00	0.00
ATOM	5251	HW1	SOL	1376	27.130	24.980	35.440	1.00	0.00
ATOM	5252	HW2	SOL	1376	27.560	24.580	34.050	1.00	0.00
ATOM	5253	OW	SOL	1377	23.740	36.990	42.520	1.00	0.00
ATOM	5254	HW1	SOL	1377	24.450	37.220	41.910	1.00	0.00
ATOM	5255	HW2	SOL	1377	24.190	36.740	43.330	1.00	0.00
ATOM	5256	OW	SOL	1378	1.590	34.190	10.320	1.00	0.00
ATOM	5257	HW1	SOL	1378	2.540	34.260	10.340	1.00	0.00
ATOM	5258	HW2	SOL	1378	1.290	35.010	9.930	1.00	0.00
ATOM	5259	OW	SOL	1379	29.920	17.710	47.250	1.00	0.00
ATOM	5260	HW1	SOL	1379	30.290	18.500	47.650	1.00	0.00
ATOM	5261	HW2	SOL	1379	29.600	17.200	47.990	1.00	0.00
ATOM	5262	OW	SOL	1380	0.370	32.920	7.980	1.00	0.00
ATOM	5263	HW1	SOL	1380	0.820	33.190	8.780	1.00	0.00
ATOM	5264	HW2	SOL	1380	0.450	33.670	7.390	1.00	0.00
ATOM	5265	OW	SOL	1381	12.480	53.050	54.840	1.00	0.00
ATOM	5266	HW1	SOL	1381	13.170	52.720	55.420	1.00	0.00
ATOM	5267	HW2	SOL	1381	12.480	53.990	54.980	1.00	0.00
ATOM	5268	OW	SOL	1382	40.560	32.580	24.400	1.00	0.00
ATOM	5269	HW1	SOL	1382	41.470	32.620	24.120	1.00	0.00
ATOM	5270	HW2	SOL	1382	40.480	33.240	25.090	1.00	0.00
ATOM	5271	OW	SOL	1383	53.320	0.970	21.590	1.00	0.00
ATOM	5272	HW1	SOL	1383	53.500	0.440	20.810	1.00	0.00
ATOM	5273	HW2	SOL	1383	52.870	1.740	21.250	1.00	0.00
ATOM	5274	OW	SOL	1384	5.570	54.990	3.080	1.00	0.00
ATOM	5275	HW1	SOL	1384	6.330	54.720	2.560	1.00	0.00
ATOM	5276	HW2	SOL	1384	4.820	54.830	2.510	1.00	0.00
ATOM	5277	OW	SOL	1385	14.030	2.620	9.850	1.00	0.00
ATOM	5278	HW1	SOL	1385	13.910	3.550	10.000	1.00	0.00
ATOM	5279	HW2	SOL	1385	14.430	2.560	8.990	1.00	0.00

ATOM	5280	OW	SOL	1386	39.550	3.850	9.570	1.00	0.00
ATOM	5281	HW1	SOL	1386	40.020	4.670	9.440	1.00	0.00
ATOM	5282	HW2	SOL	1386	39.940	3.470	10.360	1.00	0.00
ATOM	5283	OW	SOL	1387	48.390	22.370	40.170	1.00	0.00
ATOM	5284	HW1	SOL	1387	48.570	22.470	41.110	1.00	0.00
ATOM	5285	HW2	SOL	1387	47.590	22.880	40.030	1.00	0.00
ATOM	5286	OW	SOL	1388	41.180	3.370	34.130	1.00	0.00
ATOM	5287	HW1	SOL	1388	41.180	3.820	34.970	1.00	0.00
ATOM	5288	HW2	SOL	1388	40.870	2.490	34.320	1.00	0.00
ATOM	5289	OW	SOL	1389	15.300	23.610	46.970	1.00	0.00
ATOM	5290	HW1	SOL	1389	14.590	23.710	46.340	1.00	0.00
ATOM	5291	HW2	SOL	1389	15.110	22.780	47.410	1.00	0.00
ATOM	5292	OW	SOL	1390	15.740	26.850	26.750	1.00	0.00
ATOM	5293	HW1	SOL	1390	16.300	26.720	25.990	1.00	0.00
ATOM	5294	HW2	SOL	1390	15.280	27.670	26.590	1.00	0.00
ATOM	5295	OW	SOL	1391	7.010	16.790	41.590	1.00	0.00
ATOM	5296	HW1	SOL	1391	7.220	17.680	41.310	1.00	0.00
ATOM	5297	HW2	SOL	1391	7.850	16.440	41.890	1.00	0.00
ATOM	5298	OW	SOL	1392	8.100	37.640	47.960	1.00	0.00
ATOM	5299	HW1	SOL	1392	7.840	38.390	47.430	1.00	0.00
ATOM	5300	HW2	SOL	1392	8.940	37.900	48.340	1.00	0.00
ATOM	5301	OW	SOL	1393	28.610	33.590	3.550	1.00	0.00
ATOM	5302	HW1	SOL	1393	28.550	32.910	4.220	1.00	0.00
ATOM	5303	HW2	SOL	1393	27.900	34.200	3.760	1.00	0.00
ATOM	5304	OW	SOL	1394	55.430	24.520	9.890	1.00	0.00
ATOM	5305	HW1	SOL	1394	55.340	25.340	10.380	1.00	0.00
ATOM	5306	HW2	SOL	1394	54.590	24.080	10.000	1.00	0.00
ATOM	5307	OW	SOL	1395	11.210	51.650	52.740	1.00	0.00
ATOM	5308	HW1	SOL	1395	11.900	52.190	53.120	1.00	0.00
ATOM	5309	HW2	SOL	1395	10.610	52.280	52.340	1.00	0.00
ATOM	5310	OW	SOL	1396	19.370	46.530	46.510	1.00	0.00
ATOM	5311	HW1	SOL	1396	20.200	46.180	46.170	1.00	0.00
ATOM	5312	HW2	SOL	1396	19.490	47.480	46.520	1.00	0.00
ATOM	5313	OW	SOL	1397	6.680	19.180	49.100	1.00	0.00
ATOM	5314	HW1	SOL	1397	6.240	19.210	49.950	1.00	0.00
ATOM	5315	HW2	SOL	1397	5.970	18.980	48.480	1.00	0.00
ATOM	5316	OW	SOL	1398	31.510	3.160	52.190	1.00	0.00
ATOM	5317	HW1	SOL	1398	31.620	2.230	52.000	1.00	0.00
ATOM	5318	HW2	SOL	1398	30.570	3.320	52.080	1.00	0.00
ATOM	5319	OW	SOL	1399	7.210	6.900	32.670	1.00	0.00
ATOM	5320	HW1	SOL	1399	7.210	6.390	33.470	1.00	0.00
ATOM	5321	HW2	SOL	1399	8.140	6.990	32.440	1.00	0.00
ATOM	5322	OW	SOL	1400	50.010	17.320	50.130	1.00	0.00
ATOM	5323	HW1	SOL	1400	50.610	18.050	49.970	1.00	0.00

ATOM	5324	HW2	SOL	1400	50.380	16.880	50.890	1.00	0.00
ATOM	5325	OW	SOL	1401	48.900	17.930	29.660	1.00	0.00
ATOM	5326	HW1	SOL	1401	49.180	17.410	28.910	1.00	0.00
ATOM	5327	HW2	SOL	1401	49.720	18.210	30.070	1.00	0.00
ATOM	5328	OW	SOL	1402	47.230	33.690	48.270	1.00	0.00
ATOM	5329	HW1	SOL	1402	47.320	34.640	48.180	1.00	0.00
ATOM	5330	HW2	SOL	1402	46.410	33.580	48.760	1.00	0.00
ATOM	5331	OW	SOL	1403	17.790	23.380	52.150	1.00	0.00
ATOM	5332	HW1	SOL	1403	17.900	24.240	51.760	1.00	0.00
ATOM	5333	HW2	SOL	1403	16.920	23.390	52.540	1.00	0.00
ATOM	5334	OW	SOL	1404	33.890	33.220	0.380	1.00	0.00
ATOM	5335	HW1	SOL	1404	34.010	32.610	1.110	1.00	0.00
ATOM	5336	HW2	SOL	1404	33.800	32.650	-0.380	1.00	0.00
ATOM	5337	OW	SOL	1405	7.280	39.840	35.190	1.00	0.00
ATOM	5338	HW1	SOL	1405	8.040	39.840	34.610	1.00	0.00
ATOM	5339	HW2	SOL	1405	6.520	39.870	34.610	1.00	0.00
ATOM	5340	OW	SOL	1406	19.390	52.550	44.270	1.00	0.00
ATOM	5341	HW1	SOL	1406	20.310	52.350	44.100	1.00	0.00
ATOM	5342	HW2	SOL	1406	18.920	52.170	43.530	1.00	0.00
ATOM	5343	OW	SOL	1407	4.700	13.410	20.740	1.00	0.00
ATOM	5344	HW1	SOL	1407	5.630	13.350	20.940	1.00	0.00
ATOM	5345	HW2	SOL	1407	4.500	14.340	20.840	1.00	0.00
ATOM	5346	OW	SOL	1408	27.900	38.820	54.870	1.00	0.00
ATOM	5347	HW1	SOL	1408	27.280	39.250	55.450	1.00	0.00
ATOM	5348	HW2	SOL	1408	28.590	39.470	54.720	1.00	0.00
ATOM	5349	OW	SOL	1409	15.800	3.010	19.920	1.00	0.00
ATOM	5350	HW1	SOL	1409	15.840	3.300	19.010	1.00	0.00
ATOM	5351	HW2	SOL	1409	15.150	2.290	19.920	1.00	0.00
ATOM	5352	OW	SOL	1410	14.640	29.400	26.230	1.00	0.00
ATOM	5353	HW1	SOL	1410	13.970	29.360	25.550	1.00	0.00
ATOM	5354	HW2	SOL	1410	15.450	29.610	25.760	1.00	0.00
ATOM	5355	OW	SOL	1411	34.850	42.130	1.250	1.00	0.00
ATOM	5356	HW1	SOL	1411	35.720	42.430	1.000	1.00	0.00
ATOM	5357	HW2	SOL	1411	34.920	41.950	2.190	1.00	0.00
ATOM	5358	OW	SOL	1412	1.210	33.770	25.900	1.00	0.00
ATOM	5359	HW1	SOL	1412	1.420	33.350	26.730	1.00	0.00
ATOM	5360	HW2	SOL	1412	1.040	34.690	26.130	1.00	0.00
ATOM	5361	OW	SOL	1413	32.160	8.150	4.020	1.00	0.00
ATOM	5362	HW1	SOL	1413	32.400	9.070	3.960	1.00	0.00
ATOM	5363	HW2	SOL	1413	31.280	8.160	4.400	1.00	0.00
ATOM	5364	OW	SOL	1414	19.510	33.330	31.810	1.00	0.00
ATOM	5365	HW1	SOL	1414	18.680	33.210	32.270	1.00	0.00
ATOM	5366	HW2	SOL	1414	19.600	32.560	31.260	1.00	0.00
ATOM	5367	OW	SOL	1415	37.150	32.220	53.050	1.00	0.00

ATOM	5368	HW1	SOL	1415	37.820	31.570	52.840	1.00	0.00
ATOM	5369	HW2	SOL	1415	37.050	32.740	52.250	1.00	0.00
ATOM	5370	OW	SOL	1416	48.430	15.570	46.470	1.00	0.00
ATOM	5371	HW1	SOL	1416	47.700	15.050	46.140	1.00	0.00
ATOM	5372	HW2	SOL	1416	48.560	16.250	45.810	1.00	0.00
ATOM	5373	OW	SOL	1417	55.160	13.300	37.170	1.00	0.00
ATOM	5374	HW1	SOL	1417	54.760	13.960	37.740	1.00	0.00
ATOM	5375	HW2	SOL	1417	56.010	13.120	37.570	1.00	0.00
ATOM	5376	OW	SOL	1418	40.190	50.870	32.600	1.00	0.00
ATOM	5377	HW1	SOL	1418	39.460	50.550	32.080	1.00	0.00
ATOM	5378	HW2	SOL	1418	39.920	50.770	33.510	1.00	0.00
ATOM	5379	OW	SOL	1419	4.340	15.180	35.600	1.00	0.00
ATOM	5380	HW1	SOL	1419	4.650	14.500	36.190	1.00	0.00
ATOM	5381	HW2	SOL	1419	4.380	15.980	36.120	1.00	0.00
ATOM	5382	OW	SOL	1420	34.050	10.580	6.190	1.00	0.00
ATOM	5383	HW1	SOL	1420	33.390	10.600	6.880	1.00	0.00
ATOM	5384	HW2	SOL	1420	33.580	10.830	5.400	1.00	0.00
ATOM	5385	OW	SOL	1421	52.420	21.020	31.790	1.00	0.00
ATOM	5386	HW1	SOL	1421	52.140	20.460	31.060	1.00	0.00
ATOM	5387	HW2	SOL	1421	52.550	20.410	32.520	1.00	0.00
ATOM	5388	OW	SOL	1422	38.710	11.980	21.990	1.00	0.00
ATOM	5389	HW1	SOL	1422	38.000	12.580	22.230	1.00	0.00
ATOM	5390	HW2	SOL	1422	38.270	11.140	21.850	1.00	0.00
ATOM	5391	OW	SOL	1423	9.580	40.070	39.920	1.00	0.00
ATOM	5392	HW1	SOL	1423	9.990	40.230	40.770	1.00	0.00
ATOM	5393	HW2	SOL	1423	9.360	39.140	39.930	1.00	0.00
ATOM	5394	OW	SOL	1424	0.270	40.650	28.600	1.00	0.00
ATOM	5395	HW1	SOL	1424	-0.130	39.970	29.140	1.00	0.00
ATOM	5396	HW2	SOL	1424	-0.470	41.090	28.180	1.00	0.00
ATOM	5397	OW	SOL	1425	7.770	46.920	20.540	1.00	0.00
ATOM	5398	HW1	SOL	1425	8.370	47.260	21.200	1.00	0.00
ATOM	5399	HW2	SOL	1425	7.960	47.430	19.750	1.00	0.00
ATOM	5400	OW	SOL	1426	10.920	55.520	37.600	1.00	0.00
ATOM	5401	HW1	SOL	1426	10.300	54.810	37.760	1.00	0.00
ATOM	5402	HW2	SOL	1426	11.780	55.120	37.730	1.00	0.00
ATOM	5403	OW	SOL	1427	13.870	50.610	14.510	1.00	0.00
ATOM	5404	HW1	SOL	1427	13.500	51.220	15.140	1.00	0.00
ATOM	5405	HW2	SOL	1427	14.290	51.170	13.850	1.00	0.00
ATOM	5406	OW	SOL	1428	37.570	53.810	51.760	1.00	0.00
ATOM	5407	HW1	SOL	1428	37.190	53.520	50.940	1.00	0.00
ATOM	5408	HW2	SOL	1428	37.610	53.020	52.310	1.00	0.00
ATOM	5409	OW	SOL	1429	50.260	10.560	53.570	1.00	0.00
ATOM	5410	HW1	SOL	1429	49.800	10.330	52.770	1.00	0.00
ATOM	5411	HW2	SOL	1429	50.150	11.510	53.650	1.00	0.00

ATOM	5412	OW	SOL	1430	14.800	14.010	21.100	1.00	0.00
ATOM	5413	HW1	SOL	1430	14.450	14.760	21.580	1.00	0.00
ATOM	5414	HW2	SOL	1430	15.750	14.120	21.140	1.00	0.00
ATOM	5415	OW	SOL	1431	15.100	39.490	10.350	1.00	0.00
ATOM	5416	HW1	SOL	1431	14.350	39.360	10.930	1.00	0.00
ATOM	5417	HW2	SOL	1431	15.060	40.420	10.120	1.00	0.00
ATOM	5418	OW	SOL	1432	1.730	42.620	40.560	1.00	0.00
ATOM	5419	HW1	SOL	1432	1.760	42.500	41.510	1.00	0.00
ATOM	5420	HW2	SOL	1432	2.610	42.930	40.330	1.00	0.00
ATOM	5421	OW	SOL	1433	8.980	46.470	5.750	1.00	0.00
ATOM	5422	HW1	SOL	1433	9.590	45.780	5.990	1.00	0.00
ATOM	5423	HW2	SOL	1433	8.840	46.360	4.810	1.00	0.00
ATOM	5424	OW	SOL	1434	30.960	36.610	12.880	1.00	0.00
ATOM	5425	HW1	SOL	1434	30.120	36.640	13.330	1.00	0.00
ATOM	5426	HW2	SOL	1434	30.850	37.220	12.140	1.00	0.00
ATOM	5427	OW	SOL	1435	41.580	43.120	40.250	1.00	0.00
ATOM	5428	HW1	SOL	1435	40.930	43.640	40.720	1.00	0.00
ATOM	5429	HW2	SOL	1435	41.300	43.150	39.330	1.00	0.00
ATOM	5430	OW	SOL	1436	45.360	2.800	53.220	1.00	0.00
ATOM	5431	HW1	SOL	1436	44.830	2.400	53.910	1.00	0.00
ATOM	5432	HW2	SOL	1436	46.130	2.230	53.140	1.00	0.00
ATOM	5433	OW	SOL	1437	24.140	22.280	52.380	1.00	0.00
ATOM	5434	HW1	SOL	1437	24.660	21.820	51.720	1.00	0.00
ATOM	5435	HW2	SOL	1437	24.250	23.210	52.160	1.00	0.00
ATOM	5436	OW	SOL	1438	2.480	27.940	16.480	1.00	0.00
ATOM	5437	HW1	SOL	1438	2.000	28.270	17.230	1.00	0.00
ATOM	5438	HW2	SOL	1438	2.040	28.330	15.720	1.00	0.00
ATOM	5439	OW	SOL	1439	33.000	14.010	46.910	1.00	0.00
ATOM	5440	HW1	SOL	1439	32.650	13.870	46.040	1.00	0.00
ATOM	5441	HW2	SOL	1439	33.090	13.130	47.280	1.00	0.00
ATOM	5442	OW	SOL	1440	25.500	32.960	15.070	1.00	0.00
ATOM	5443	HW1	SOL	1440	25.720	32.360	15.790	1.00	0.00
ATOM	5444	HW2	SOL	1440	24.550	32.910	15.010	1.00	0.00
ATOM	5445	OW	SOL	1441	37.070	8.410	23.650	1.00	0.00
ATOM	5446	HW1	SOL	1441	37.520	8.420	24.500	1.00	0.00
ATOM	5447	HW2	SOL	1441	36.310	7.840	23.790	1.00	0.00
ATOM	5448	OW	SOL	1442	48.940	4.230	12.190	1.00	0.00
ATOM	5449	HW1	SOL	1442	49.050	3.410	11.710	1.00	0.00
ATOM	5450	HW2	SOL	1442	49.210	4.030	13.080	1.00	0.00
ATOM	5451	OW	SOL	1443	44.860	47.540	41.880	1.00	0.00
ATOM	5452	HW1	SOL	1443	43.930	47.330	41.770	1.00	0.00
ATOM	5453	HW2	SOL	1443	45.020	48.250	41.260	1.00	0.00
ATOM	5454	OW	SOL	1444	16.620	52.380	33.340	1.00	0.00
ATOM	5455	HW1	SOL	1444	17.390	51.840	33.160	1.00	0.00

ATOM	5456	HW2	SOL	1444	16.180	51.940	34.070	1.00	0.00
ATOM	5457	OW	SOL	1445	27.390	36.800	52.850	1.00	0.00
ATOM	5458	HW1	SOL	1445	27.490	37.560	53.410	1.00	0.00
ATOM	5459	HW2	SOL	1445	27.390	36.050	53.450	1.00	0.00
ATOM	5460	OW	SOL	1446	25.800	3.360	2.130	1.00	0.00
ATOM	5461	HW1	SOL	1446	26.060	3.510	3.040	1.00	0.00
ATOM	5462	HW2	SOL	1446	26.570	3.590	1.610	1.00	0.00
ATOM	5463	OW	SOL	1447	18.490	25.680	7.440	1.00	0.00
ATOM	5464	HW1	SOL	1447	18.020	25.870	8.260	1.00	0.00
ATOM	5465	HW2	SOL	1447	19.340	26.110	7.560	1.00	0.00
ATOM	5466	OW	SOL	1448	43.300	40.790	4.240	1.00	0.00
ATOM	5467	HW1	SOL	1448	43.680	40.880	5.120	1.00	0.00
ATOM	5468	HW2	SOL	1448	43.560	41.590	3.790	1.00	0.00
ATOM	5469	OW	SOL	1449	29.440	19.290	23.710	1.00	0.00
ATOM	5470	HW1	SOL	1449	29.200	19.990	24.310	1.00	0.00
ATOM	5471	HW2	SOL	1449	28.790	19.330	23.010	1.00	0.00
ATOM	5472	OW	SOL	1450	10.270	8.430	12.610	1.00	0.00
ATOM	5473	HW1	SOL	1450	9.530	8.840	13.050	1.00	0.00
ATOM	5474	HW2	SOL	1450	10.860	9.160	12.400	1.00	0.00
ATOM	5475	OW	SOL	1451	39.340	20.440	3.370	1.00	0.00
ATOM	5476	HW1	SOL	1451	40.100	21.030	3.310	1.00	0.00
ATOM	5477	HW2	SOL	1451	39.260	20.060	2.490	1.00	0.00
ATOM	5478	OW	SOL	1452	37.590	33.250	7.260	1.00	0.00
ATOM	5479	HW1	SOL	1452	37.480	32.810	8.110	1.00	0.00
ATOM	5480	HW2	SOL	1452	36.700	33.310	6.920	1.00	0.00
ATOM	5481	OW	SOL	1453	7.370	9.420	1.140	1.00	0.00
ATOM	5482	HW1	SOL	1453	7.960	10.170	1.170	1.00	0.00
ATOM	5483	HW2	SOL	1453	7.900	8.720	0.750	1.00	0.00
ATOM	5484	OW	SOL	1454	42.570	39.050	21.330	1.00	0.00
ATOM	5485	HW1	SOL	1454	43.220	38.690	20.720	1.00	0.00
ATOM	5486	HW2	SOL	1454	42.080	39.690	20.800	1.00	0.00
ATOM	5487	OW	SOL	1455	23.530	50.640	43.870	1.00	0.00
ATOM	5488	HW1	SOL	1455	22.780	51.170	43.600	1.00	0.00
ATOM	5489	HW2	SOL	1455	23.800	51.020	44.710	1.00	0.00
ATOM	5490	OW	SOL	1456	7.270	50.670	47.170	1.00	0.00
ATOM	5491	HW1	SOL	1456	8.230	50.690	47.190	1.00	0.00
ATOM	5492	HW2	SOL	1456	7.030	51.410	46.610	1.00	0.00
ATOM	5493	OW	SOL	1457	2.850	37.990	20.760	1.00	0.00
ATOM	5494	HW1	SOL	1457	2.820	37.620	21.650	1.00	0.00
ATOM	5495	HW2	SOL	1457	2.560	38.900	20.870	1.00	0.00
ATOM	5496	OW	SOL	1458	17.120	4.920	52.060	1.00	0.00
ATOM	5497	HW1	SOL	1458	16.280	4.480	51.960	1.00	0.00
ATOM	5498	HW2	SOL	1458	17.610	4.370	52.670	1.00	0.00
ATOM	5499	OW	SOL	1459	25.880	4.250	4.720	1.00	0.00

ATOM	5500	HW1	SOL	1459	25.860	5.140	4.370	1.00	0.00
ATOM	5501	HW2	SOL	1459	25.580	4.340	5.630	1.00	0.00
ATOM	5502	OW	SOL	1460	31.680	25.690	48.410	1.00	0.00
ATOM	5503	HW1	SOL	1460	32.370	25.080	48.660	1.00	0.00
ATOM	5504	HW2	SOL	1460	30.930	25.450	48.940	1.00	0.00
ATOM	5505	OW	SOL	1461	46.050	28.860	1.950	1.00	0.00
ATOM	5506	HW1	SOL	1461	45.700	29.300	2.720	1.00	0.00
ATOM	5507	HW2	SOL	1461	46.310	27.990	2.260	1.00	0.00
ATOM	5508	OW	SOL	1462	44.420	21.300	21.250	1.00	0.00
ATOM	5509	HW1	SOL	1462	44.630	20.790	22.030	1.00	0.00
ATOM	5510	HW2	SOL	1462	43.670	21.830	21.490	1.00	0.00
ATOM	5511	OW	SOL	1463	25.230	4.220	40.980	1.00	0.00
ATOM	5512	HW1	SOL	1463	25.170	4.210	40.020	1.00	0.00
ATOM	5513	HW2	SOL	1463	24.730	4.990	41.240	1.00	0.00
ATOM	5514	OW	SOL	1464	40.430	36.600	16.910	1.00	0.00
ATOM	5515	HW1	SOL	1464	40.500	35.950	16.210	1.00	0.00
ATOM	5516	HW2	SOL	1464	39.610	36.390	17.350	1.00	0.00
ATOM	5517	OW	SOL	1465	38.870	20.570	29.760	1.00	0.00
ATOM	5518	HW1	SOL	1465	38.080	20.440	30.290	1.00	0.00
ATOM	5519	HW2	SOL	1465	38.610	20.330	28.870	1.00	0.00
ATOM	5520	OW	SOL	1466	9.170	22.810	6.400	1.00	0.00
ATOM	5521	HW1	SOL	1466	9.070	22.490	7.290	1.00	0.00
ATOM	5522	HW2	SOL	1466	10.000	23.300	6.420	1.00	0.00
ATOM	5523	OW	SOL	1467	47.730	52.860	8.290	1.00	0.00
ATOM	5524	HW1	SOL	1467	46.970	53.150	8.810	1.00	0.00
ATOM	5525	HW2	SOL	1467	47.440	52.030	7.900	1.00	0.00
ATOM	5526	OW	SOL	1468	51.980	45.620	4.550	1.00	0.00
ATOM	5527	HW1	SOL	1468	51.370	45.100	5.070	1.00	0.00
ATOM	5528	HW2	SOL	1468	52.410	46.200	5.180	1.00	0.00
ATOM	5529	OW	SOL	1469	26.070	31.360	2.010	1.00	0.00
ATOM	5530	HW1	SOL	1469	26.710	31.850	1.500	1.00	0.00
ATOM	5531	HW2	SOL	1469	26.420	30.470	2.030	1.00	0.00
ATOM	5532	OW	SOL	1470	8.930	5.760	46.810	1.00	0.00
ATOM	5533	HW1	SOL	1470	8.000	5.660	46.630	1.00	0.00
ATOM	5534	HW2	SOL	1470	9.350	5.010	46.370	1.00	0.00
ATOM	5535	OW	SOL	1471	39.460	18.440	18.530	1.00	0.00
ATOM	5536	HW1	SOL	1471	39.960	18.040	19.240	1.00	0.00
ATOM	5537	HW2	SOL	1471	39.870	18.100	17.730	1.00	0.00
ATOM	5538	OW	SOL	1472	36.580	33.340	30.540	1.00	0.00
ATOM	5539	HW1	SOL	1472	35.640	33.520	30.490	1.00	0.00
ATOM	5540	HW2	SOL	1472	36.760	33.290	31.480	1.00	0.00
ATOM	5541	OW	SOL	1473	14.770	25.550	32.530	1.00	0.00
ATOM	5542	HW1	SOL	1473	14.580	26.490	32.430	1.00	0.00
ATOM	5543	HW2	SOL	1473	15.340	25.500	33.290	1.00	0.00

ATOM	5544	OW	SOL	1474	26.840	4.010	52.940	1.00	0.00
ATOM	5545	HW1	SOL	1474	26.890	4.950	53.100	1.00	0.00
ATOM	5546	HW2	SOL	1474	27.080	3.610	53.770	1.00	0.00
ATOM	5547	OW	SOL	1475	26.850	10.880	52.880	1.00	0.00
ATOM	5548	HW1	SOL	1475	26.490	11.760	52.800	1.00	0.00
ATOM	5549	HW2	SOL	1475	27.270	10.860	53.740	1.00	0.00
ATOM	5550	OW	SOL	1476	9.020	36.090	45.740	1.00	0.00
ATOM	5551	HW1	SOL	1476	8.750	36.790	46.340	1.00	0.00
ATOM	5552	HW2	SOL	1476	8.660	35.300	46.130	1.00	0.00
ATOM	5553	OW	SOL	1477	21.940	27.700	6.410	1.00	0.00
ATOM	5554	HW1	SOL	1477	22.640	28.220	6.810	1.00	0.00
ATOM	5555	HW2	SOL	1477	21.500	28.300	5.810	1.00	0.00
ATOM	5556	OW	SOL	1478	13.750	26.610	41.070	1.00	0.00
ATOM	5557	HW1	SOL	1478	14.000	27.460	41.430	1.00	0.00
ATOM	5558	HW2	SOL	1478	13.950	26.670	40.140	1.00	0.00
ATOM	5559	OW	SOL	1479	52.990	8.330	1.270	1.00	0.00
ATOM	5560	HW1	SOL	1479	53.250	8.700	2.120	1.00	0.00
ATOM	5561	HW2	SOL	1479	53.810	7.980	0.910	1.00	0.00
ATOM	5562	OW	SOL	1480	26.880	18.710	34.400	1.00	0.00
ATOM	5563	HW1	SOL	1480	26.500	18.790	33.530	1.00	0.00
ATOM	5564	HW2	SOL	1480	27.690	18.220	34.270	1.00	0.00
ATOM	5565	OW	SOL	1481	43.130	38.680	54.740	1.00	0.00
ATOM	5566	HW1	SOL	1481	44.060	38.900	54.830	1.00	0.00
ATOM	5567	HW2	SOL	1481	43.000	37.970	55.360	1.00	0.00
ATOM	5568	OW	SOL	1482	35.420	22.580	54.530	1.00	0.00
ATOM	5569	HW1	SOL	1482	35.850	23.280	54.030	1.00	0.00
ATOM	5570	HW2	SOL	1482	34.620	22.390	54.040	1.00	0.00
ATOM	5571	OW	SOL	1483	2.970	47.650	2.800	1.00	0.00
ATOM	5572	HW1	SOL	1483	3.660	47.150	2.350	1.00	0.00
ATOM	5573	HW2	SOL	1483	2.210	47.570	2.230	1.00	0.00
ATOM	5574	OW	SOL	1484	14.940	11.200	2.020	1.00	0.00
ATOM	5575	HW1	SOL	1484	14.990	10.250	2.010	1.00	0.00
ATOM	5576	HW2	SOL	1484	15.800	11.490	2.330	1.00	0.00
ATOM	5577	OW	SOL	1485	43.740	48.400	8.410	1.00	0.00
ATOM	5578	HW1	SOL	1485	42.900	47.930	8.350	1.00	0.00
ATOM	5579	HW2	SOL	1485	43.950	48.620	7.500	1.00	0.00
ATOM	5580	OW	SOL	1486	9.440	7.550	53.040	1.00	0.00
ATOM	5581	HW1	SOL	1486	9.890	8.160	53.630	1.00	0.00
ATOM	5582	HW2	SOL	1486	9.930	6.730	53.120	1.00	0.00
ATOM	5583	OW	SOL	1487	29.250	20.860	3.040	1.00	0.00
ATOM	5584	HW1	SOL	1487	29.230	20.800	3.990	1.00	0.00
ATOM	5585	HW2	SOL	1487	30.170	20.760	2.810	1.00	0.00
ATOM	5586	OW	SOL	1488	37.680	25.180	29.370	1.00	0.00
ATOM	5587	HW1	SOL	1488	37.250	25.110	28.520	1.00	0.00

ATOM	5588	HW2	SOL	1488	36.960	25.280	30.000	1.00	0.00
ATOM	5589	OW	SOL	1489	46.310	48.780	39.410	1.00	0.00
ATOM	5590	HW1	SOL	1489	45.470	49.130	39.100	1.00	0.00
ATOM	5591	HW2	SOL	1489	46.390	47.930	38.990	1.00	0.00
ATOM	5592	OW	SOL	1490	32.870	13.170	29.080	1.00	0.00
ATOM	5593	HW1	SOL	1490	32.810	13.830	29.770	1.00	0.00
ATOM	5594	HW2	SOL	1490	32.920	13.680	28.260	1.00	0.00
ATOM	5595	OW	SOL	1491	16.770	50.160	19.940	1.00	0.00
ATOM	5596	HW1	SOL	1491	16.510	51.070	19.780	1.00	0.00
ATOM	5597	HW2	SOL	1491	16.100	49.820	20.530	1.00	0.00
ATOM	5598	OW	SOL	1492	45.830	1.770	13.710	1.00	0.00
ATOM	5599	HW1	SOL	1492	45.100	2.110	14.230	1.00	0.00
ATOM	5600	HW2	SOL	1492	46.600	2.160	14.100	1.00	0.00
ATOM	5601	OW	SOL	1493	4.260	26.010	39.310	1.00	0.00
ATOM	5602	HW1	SOL	1493	5.210	25.890	39.360	1.00	0.00
ATOM	5603	HW2	SOL	1493	4.040	26.530	40.080	1.00	0.00
ATOM	5604	OW	SOL	1494	5.810	0.050	34.720	1.00	0.00
ATOM	5605	HW1	SOL	1494	5.200	0.640	34.280	1.00	0.00
ATOM	5606	HW2	SOL	1494	5.990	0.480	35.560	1.00	0.00
ATOM	5607	OW	SOL	1495	44.430	2.720	34.330	1.00	0.00
ATOM	5608	HW1	SOL	1495	45.180	3.160	33.940	1.00	0.00
ATOM	5609	HW2	SOL	1495	43.770	3.410	34.400	1.00	0.00
ATOM	5610	OW	SOL	1496	49.170	48.290	0.140	1.00	0.00
ATOM	5611	HW1	SOL	1496	49.660	49.010	0.530	1.00	0.00
ATOM	5612	HW2	SOL	1496	49.760	47.920	-0.510	1.00	0.00
ATOM	5613	OW	SOL	1497	52.850	10.790	23.320	1.00	0.00
ATOM	5614	HW1	SOL	1497	53.540	10.910	22.660	1.00	0.00
ATOM	5615	HW2	SOL	1497	53.260	10.260	24.000	1.00	0.00
ATOM	5616	OW	SOL	1498	10.220	23.590	31.120	1.00	0.00
ATOM	5617	HW1	SOL	1498	9.880	23.810	30.260	1.00	0.00
ATOM	5618	HW2	SOL	1498	10.280	24.430	31.580	1.00	0.00
ATOM	5619	OW	SOL	1499	3.110	38.570	30.800	1.00	0.00
ATOM	5620	HW1	SOL	1499	3.890	39.090	30.620	1.00	0.00
ATOM	5621	HW2	SOL	1499	2.460	39.200	31.090	1.00	0.00
ATOM	5622	OW	SOL	1500	19.290	52.780	15.790	1.00	0.00
ATOM	5623	HW1	SOL	1500	18.890	52.090	15.250	1.00	0.00
ATOM	5624	HW2	SOL	1500	20.010	52.340	16.240	1.00	0.00
ATOM	5625	OW	SOL	1501	55.080	32.360	33.500	1.00	0.00
ATOM	5626	HW1	SOL	1501	54.760	33.240	33.670	1.00	0.00
ATOM	5627	HW2	SOL	1501	54.470	32.000	32.850	1.00	0.00
ATOM	5628	OW	SOL	1502	55.820	50.830	1.420	1.00	0.00
ATOM	5629	HW1	SOL	1502	56.080	50.460	2.270	1.00	0.00
ATOM	5630	HW2	SOL	1502	54.930	50.520	1.290	1.00	0.00
ATOM	5631	OW	SOL	1503	50.650	16.450	2.310	1.00	0.00

ATOM	5632	HW1	SOL	1503	49.810	16.340	1.870	1.00	0.00
ATOM	5633	HW2	SOL	1503	50.430	16.550	3.230	1.00	0.00
ATOM	5634	OW	SOL	1504	51.720	39.420	24.390	1.00	0.00
ATOM	5635	HW1	SOL	1504	51.480	38.950	23.590	1.00	0.00
ATOM	5636	HW2	SOL	1504	51.000	39.230	25.000	1.00	0.00
ATOM	5637	OW	SOL	1505	7.100	54.310	42.690	1.00	0.00
ATOM	5638	HW1	SOL	1505	7.010	54.300	41.730	1.00	0.00
ATOM	5639	HW2	SOL	1505	7.750	54.990	42.860	1.00	0.00
ATOM	5640	OW	SOL	1506	51.420	3.830	24.240	1.00	0.00
ATOM	5641	HW1	SOL	1506	52.000	3.080	24.350	1.00	0.00
ATOM	5642	HW2	SOL	1506	52.000	4.590	24.230	1.00	0.00
ATOM	5643	OW	SOL	1507	50.520	13.100	29.220	1.00	0.00
ATOM	5644	HW1	SOL	1507	50.060	13.930	29.290	1.00	0.00
ATOM	5645	HW2	SOL	1507	49.860	12.490	28.900	1.00	0.00
ATOM	5646	OW	SOL	1508	26.210	7.000	3.810	1.00	0.00
ATOM	5647	HW1	SOL	1508	25.760	6.840	2.980	1.00	0.00
ATOM	5648	HW2	SOL	1508	25.630	7.600	4.280	1.00	0.00
ATOM	5649	OW	SOL	1509	45.410	4.470	1.890	1.00	0.00
ATOM	5650	HW1	SOL	1509	45.910	3.680	2.110	1.00	0.00
ATOM	5651	HW2	SOL	1509	45.070	4.300	1.020	1.00	0.00
ATOM	5652	OW	SOL	1510	54.610	9.390	33.610	1.00	0.00
ATOM	5653	HW1	SOL	1510	53.970	8.730	33.880	1.00	0.00
ATOM	5654	HW2	SOL	1510	54.190	9.840	32.870	1.00	0.00
ATOM	5655	OW	SOL	1511	49.880	10.790	27.670	1.00	0.00
ATOM	5656	HW1	SOL	1511	49.670	11.140	26.800	1.00	0.00
ATOM	5657	HW2	SOL	1511	50.710	10.330	27.540	1.00	0.00
ATOM	5658	OW	SOL	1512	37.570	2.980	53.690	1.00	0.00
ATOM	5659	HW1	SOL	1512	38.020	3.790	53.460	1.00	0.00
ATOM	5660	HW2	SOL	1512	36.680	3.090	53.350	1.00	0.00
ATOM	5661	OW	SOL	1513	4.590	30.090	54.450	1.00	0.00
ATOM	5662	HW1	SOL	1513	5.360	29.750	53.990	1.00	0.00
ATOM	5663	HW2	SOL	1513	4.210	29.330	54.890	1.00	0.00
ATOM	5664	OW	SOL	1514	3.160	43.600	25.250	1.00	0.00
ATOM	5665	HW1	SOL	1514	3.310	43.960	24.380	1.00	0.00
ATOM	5666	HW2	SOL	1514	3.190	44.350	25.840	1.00	0.00
ATOM	5667	OW	SOL	1515	14.950	19.870	46.040	1.00	0.00
ATOM	5668	HW1	SOL	1515	15.490	19.080	45.950	1.00	0.00
ATOM	5669	HW2	SOL	1515	15.210	20.250	46.880	1.00	0.00
ATOM	5670	OW	SOL	1516	49.900	16.810	27.450	1.00	0.00
ATOM	5671	HW1	SOL	1516	50.700	16.850	27.980	1.00	0.00
ATOM	5672	HW2	SOL	1516	50.130	16.240	26.720	1.00	0.00
ATOM	5673	OW	SOL	1517	54.160	1.680	54.810	1.00	0.00
ATOM	5674	HW1	SOL	1517	54.830	1.080	54.490	1.00	0.00
ATOM	5675	HW2	SOL	1517	53.820	2.110	54.030	1.00	0.00

ATOM	5676	OW	SOL	1518	53.030	21.310	45.580	1.00	0.00
ATOM	5677	HW1	SOL	1518	52.590	21.320	46.430	1.00	0.00
ATOM	5678	HW2	SOL	1518	53.840	21.800	45.730	1.00	0.00
ATOM	5679	OW	SOL	1519	48.710	36.490	51.230	1.00	0.00
ATOM	5680	HW1	SOL	1519	48.390	35.670	50.850	1.00	0.00
ATOM	5681	HW2	SOL	1519	48.680	37.110	50.500	1.00	0.00
ATOM	5682	OW	SOL	1520	47.160	6.530	2.330	1.00	0.00
ATOM	5683	HW1	SOL	1520	46.210	6.520	2.410	1.00	0.00
ATOM	5684	HW2	SOL	1520	47.390	5.610	2.180	1.00	0.00
ATOM	5685	OW	SOL	1521	21.410	37.020	2.280	1.00	0.00
ATOM	5686	HW1	SOL	1521	21.760	36.960	3.170	1.00	0.00
ATOM	5687	HW2	SOL	1521	20.550	37.430	2.380	1.00	0.00
ATOM	5688	OW	SOL	1522	55.260	42.080	36.970	1.00	0.00
ATOM	5689	HW1	SOL	1522	55.580	41.430	37.600	1.00	0.00
ATOM	5690	HW2	SOL	1522	55.980	42.700	36.880	1.00	0.00
ATOM	5691	OW	SOL	1523	30.910	24.670	20.170	1.00	0.00
ATOM	5692	HW1	SOL	1523	30.790	24.020	19.480	1.00	0.00
ATOM	5693	HW2	SOL	1523	31.660	25.190	19.880	1.00	0.00
ATOM	5694	OW	SOL	1524	28.610	43.690	42.070	1.00	0.00
ATOM	5695	HW1	SOL	1524	28.030	43.150	42.610	1.00	0.00
ATOM	5696	HW2	SOL	1524	29.300	43.090	41.790	1.00	0.00
ATOM	5697	OW	SOL	1525	5.680	19.700	54.080	1.00	0.00
ATOM	5698	HW1	SOL	1525	5.500	20.610	54.330	1.00	0.00
ATOM	5699	HW2	SOL	1525	4.970	19.200	54.470	1.00	0.00
ATOM	5700	OW	SOL	1526	52.240	36.900	17.570	1.00	0.00
ATOM	5701	HW1	SOL	1526	53.080	37.350	17.670	1.00	0.00
ATOM	5702	HW2	SOL	1526	51.800	37.370	16.860	1.00	0.00
ATOM	5703	OW	SOL	1527	6.390	18.350	27.100	1.00	0.00
ATOM	5704	HW1	SOL	1527	5.470	18.460	26.840	1.00	0.00
ATOM	5705	HW2	SOL	1527	6.410	18.600	28.020	1.00	0.00
ATOM	5706	OW	SOL	1528	42.850	4.350	14.030	1.00	0.00
ATOM	5707	HW1	SOL	1528	43.790	4.470	14.100	1.00	0.00
ATOM	5708	HW2	SOL	1528	42.480	5.030	14.600	1.00	0.00
ATOM	5709	OW	SOL	1529	44.880	19.940	23.690	1.00	0.00
ATOM	5710	HW1	SOL	1529	45.050	20.290	24.560	1.00	0.00
ATOM	5711	HW2	SOL	1529	45.260	19.060	23.690	1.00	0.00
ATOM	5712	OW	SOL	1530	39.080	39.930	23.480	1.00	0.00
ATOM	5713	HW1	SOL	1530	39.320	39.040	23.730	1.00	0.00
ATOM	5714	HW2	SOL	1530	38.160	39.870	23.220	1.00	0.00
ATOM	5715	OW	SOL	1531	34.920	26.250	48.260	1.00	0.00
ATOM	5716	HW1	SOL	1531	34.220	26.400	48.880	1.00	0.00
ATOM	5717	HW2	SOL	1531	35.650	26.780	48.570	1.00	0.00
ATOM	5718	OW	SOL	1532	22.370	7.400	4.430	1.00	0.00
ATOM	5719	HW1	SOL	1532	23.220	7.020	4.240	1.00	0.00

ATOM	5720	HW2	SOL	1532	22.560	8.300	4.700	1.00	0.00
ATOM	5721	OW	SOL	1533	39.360	29.800	23.760	1.00	0.00
ATOM	5722	HW1	SOL	1533	39.550	30.670	24.100	1.00	0.00
ATOM	5723	HW2	SOL	1533	39.970	29.220	24.210	1.00	0.00
ATOM	5724	OW	SOL	1534	15.060	3.410	51.640	1.00	0.00
ATOM	5725	HW1	SOL	1534	15.140	2.930	50.810	1.00	0.00
ATOM	5726	HW2	SOL	1534	15.190	2.750	52.320	1.00	0.00
ATOM	5727	OW	SOL	1535	25.560	8.080	45.020	1.00	0.00
ATOM	5728	HW1	SOL	1535	26.160	7.860	45.740	1.00	0.00
ATOM	5729	HW2	SOL	1535	25.230	8.950	45.230	1.00	0.00
ATOM	5730	OW	SOL	1536	11.510	13.270	9.640	1.00	0.00
ATOM	5731	HW1	SOL	1536	11.670	12.770	8.840	1.00	0.00
ATOM	5732	HW2	SOL	1536	12.240	13.050	10.220	1.00	0.00
ATOM	5733	OW	SOL	1537	33.740	24.410	33.400	1.00	0.00
ATOM	5734	HW1	SOL	1537	34.070	25.070	32.790	1.00	0.00
ATOM	5735	HW2	SOL	1537	33.550	24.890	34.200	1.00	0.00
ATOM	5736	OW	SOL	1538	26.320	16.680	9.300	1.00	0.00
ATOM	5737	HW1	SOL	1538	26.390	17.630	9.360	1.00	0.00
ATOM	5738	HW2	SOL	1538	27.060	16.350	9.800	1.00	0.00
ATOM	5739	OW	SOL	1539	24.260	40.370	45.990	1.00	0.00
ATOM	5740	HW1	SOL	1539	24.600	40.010	45.170	1.00	0.00
ATOM	5741	HW2	SOL	1539	23.330	40.130	45.990	1.00	0.00
ATOM	5742	OW	SOL	1540	6.590	17.410	35.870	1.00	0.00
ATOM	5743	HW1	SOL	1540	7.010	16.660	35.450	1.00	0.00
ATOM	5744	HW2	SOL	1540	7.180	18.140	35.710	1.00	0.00
ATOM	5745	OW	SOL	1541	19.720	5.490	43.620	1.00	0.00
ATOM	5746	HW1	SOL	1541	19.870	6.190	44.250	1.00	0.00
ATOM	5747	HW2	SOL	1541	20.560	5.030	43.570	1.00	0.00
ATOM	5748	OW	SOL	1542	20.970	42.130	4.680	1.00	0.00
ATOM	5749	HW1	SOL	1542	20.440	41.340	4.710	1.00	0.00
ATOM	5750	HW2	SOL	1542	20.390	42.820	5.010	1.00	0.00
ATOM	5751	OW	SOL	1543	45.400	54.140	49.750	1.00	0.00
ATOM	5752	HW1	SOL	1543	44.840	53.370	49.670	1.00	0.00
ATOM	5753	HW2	SOL	1543	46.130	53.840	50.300	1.00	0.00
ATOM	5754	OW	SOL	1544	33.060	10.530	52.800	1.00	0.00
ATOM	5755	HW1	SOL	1544	32.290	10.980	53.150	1.00	0.00
ATOM	5756	HW2	SOL	1544	33.740	11.210	52.770	1.00	0.00
ATOM	5757	OW	SOL	1545	50.460	50.240	37.750	1.00	0.00
ATOM	5758	HW1	SOL	1545	50.400	50.420	36.810	1.00	0.00
ATOM	5759	HW2	SOL	1545	51.010	49.460	37.810	1.00	0.00
ATOM	5760	OW	SOL	1546	29.190	7.120	54.600	1.00	0.00
ATOM	5761	HW1	SOL	1546	29.050	7.200	53.650	1.00	0.00
ATOM	5762	HW2	SOL	1546	29.610	6.270	54.710	1.00	0.00
ATOM	5763	OW	SOL	1547	8.540	52.970	27.440	1.00	0.00

ATOM	5764	HW1	SOL	1547	7.740	53.490	27.480	1.00	0.00
ATOM	5765	HW2	SOL	1547	8.270	52.090	27.690	1.00	0.00
ATOM	5766	OW	SOL	1548	25.660	14.190	49.900	1.00	0.00
ATOM	5767	HW1	SOL	1548	24.810	14.230	49.470	1.00	0.00
ATOM	5768	HW2	SOL	1548	26.280	14.100	49.170	1.00	0.00
ATOM	5769	OW	SOL	1549	37.490	20.060	6.470	1.00	0.00
ATOM	5770	HW1	SOL	1549	36.990	20.110	5.650	1.00	0.00
ATOM	5771	HW2	SOL	1549	37.960	20.880	6.510	1.00	0.00
ATOM	5772	OW	SOL	1550	37.440	6.470	6.330	1.00	0.00
ATOM	5773	HW1	SOL	1550	37.240	7.220	5.770	1.00	0.00
ATOM	5774	HW2	SOL	1550	37.340	5.710	5.760	1.00	0.00
ATOM	5775	OW	SOL	1551	40.040	34.810	0.060	1.00	0.00
ATOM	5776	HW1	SOL	1551	39.320	35.070	-0.510	1.00	0.00
ATOM	5777	HW2	SOL	1551	40.390	34.010	-0.340	1.00	0.00
ATOM	5778	OW	SOL	1552	34.820	12.770	40.890	1.00	0.00
ATOM	5779	HW1	SOL	1552	35.410	12.600	40.160	1.00	0.00
ATOM	5780	HW2	SOL	1552	34.550	13.680	40.780	1.00	0.00
ATOM	5781	OW	SOL	1553	13.620	32.400	1.750	1.00	0.00
ATOM	5782	HW1	SOL	1553	13.770	32.020	2.620	1.00	0.00
ATOM	5783	HW2	SOL	1553	14.120	33.210	1.760	1.00	0.00
ATOM	5784	OW	SOL	1554	26.170	49.680	32.770	1.00	0.00
ATOM	5785	HW1	SOL	1554	25.980	48.780	32.500	1.00	0.00
ATOM	5786	HW2	SOL	1554	27.110	49.780	32.620	1.00	0.00
ATOM	5787	OW	SOL	1555	18.930	35.520	44.440	1.00	0.00
ATOM	5788	HW1	SOL	1555	18.900	34.610	44.140	1.00	0.00
ATOM	5789	HW2	SOL	1555	18.580	36.020	43.700	1.00	0.00
ATOM	5790	OW	SOL	1556	32.620	13.520	19.380	1.00	0.00
ATOM	5791	HW1	SOL	1556	32.670	14.410	19.020	1.00	0.00
ATOM	5792	HW2	SOL	1556	31.870	13.130	18.930	1.00	0.00
ATOM	5793	OW	SOL	1557	11.680	1.070	43.790	1.00	0.00
ATOM	5794	HW1	SOL	1557	11.920	0.750	44.650	1.00	0.00
ATOM	5795	HW2	SOL	1557	11.730	2.030	43.860	1.00	0.00
ATOM	5796	OW	SOL	1558	27.910	30.790	47.060	1.00	0.00
ATOM	5797	HW1	SOL	1558	28.280	30.550	47.910	1.00	0.00
ATOM	5798	HW2	SOL	1558	28.650	30.750	46.460	1.00	0.00
ATOM	5799	OW	SOL	1559	6.290	37.170	40.720	1.00	0.00
ATOM	5800	HW1	SOL	1559	5.830	37.750	41.320	1.00	0.00
ATOM	5801	HW2	SOL	1559	6.830	37.760	40.180	1.00	0.00
ATOM	5802	OW	SOL	1560	28.980	14.020	11.170	1.00	0.00
ATOM	5803	HW1	SOL	1560	29.770	14.480	11.450	1.00	0.00
ATOM	5804	HW2	SOL	1560	28.260	14.480	11.610	1.00	0.00
ATOM	5805	OW	SOL	1561	55.800	20.660	35.940	1.00	0.00
ATOM	5806	HW1	SOL	1561	56.620	20.840	36.400	1.00	0.00
ATOM	5807	HW2	SOL	1561	55.900	21.080	35.090	1.00	0.00

ATOM	5808	OW	SOL	1562	47.040	44.600	6.520	1.00	0.00
ATOM	5809	HW1	SOL	1562	46.610	44.770	5.680	1.00	0.00
ATOM	5810	HW2	SOL	1562	47.970	44.570	6.310	1.00	0.00
ATOM	5811	OW	SOL	1563	8.960	15.480	20.000	1.00	0.00
ATOM	5812	HW1	SOL	1563	8.950	14.650	19.520	1.00	0.00
ATOM	5813	HW2	SOL	1563	8.500	16.090	19.430	1.00	0.00
ATOM	5814	OW	SOL	1564	8.150	34.290	11.370	1.00	0.00
ATOM	5815	HW1	SOL	1564	8.990	34.740	11.430	1.00	0.00
ATOM	5816	HW2	SOL	1564	8.150	33.690	12.120	1.00	0.00
ATOM	5817	OW	SOL	1565	13.400	17.000	27.360	1.00	0.00
ATOM	5818	HW1	SOL	1565	12.690	17.030	28.010	1.00	0.00
ATOM	5819	HW2	SOL	1565	13.830	17.840	27.450	1.00	0.00
ATOM	5820	OW	SOL	1566	29.600	7.510	5.390	1.00	0.00
ATOM	5821	HW1	SOL	1566	28.840	7.090	5.790	1.00	0.00
ATOM	5822	HW2	SOL	1566	29.480	8.440	5.570	1.00	0.00
ATOM	5823	OW	SOL	1567	18.890	42.000	32.100	1.00	0.00
ATOM	5824	HW1	SOL	1567	19.710	41.720	32.490	1.00	0.00
ATOM	5825	HW2	SOL	1567	18.360	42.300	32.840	1.00	0.00
ATOM	5826	OW	SOL	1568	7.970	36.110	35.730	1.00	0.00
ATOM	5827	HW1	SOL	1568	7.040	36.280	35.590	1.00	0.00
ATOM	5828	HW2	SOL	1568	8.010	35.170	35.910	1.00	0.00
ATOM	5829	OW	SOL	1569	45.600	4.550	50.760	1.00	0.00
ATOM	5830	HW1	SOL	1569	44.690	4.300	50.650	1.00	0.00
ATOM	5831	HW2	SOL	1569	45.960	3.890	51.360	1.00	0.00
ATOM	5832	OW	SOL	1570	49.740	43.240	51.180	1.00	0.00
ATOM	5833	HW1	SOL	1570	49.430	43.840	51.850	1.00	0.00
ATOM	5834	HW2	SOL	1570	50.660	43.480	51.040	1.00	0.00
ATOM	5835	OW	SOL	1571	48.430	1.640	50.840	1.00	0.00
ATOM	5836	HW1	SOL	1571	48.630	0.840	51.310	1.00	0.00
ATOM	5837	HW2	SOL	1571	47.510	1.560	50.600	1.00	0.00
ATOM	5838	OW	SOL	1572	44.990	16.630	43.860	1.00	0.00
ATOM	5839	HW1	SOL	1572	44.180	17.060	43.600	1.00	0.00
ATOM	5840	HW2	SOL	1572	44.840	16.370	44.770	1.00	0.00
ATOM	5841	OW	SOL	1573	25.940	11.690	38.160	1.00	0.00
ATOM	5842	HW1	SOL	1573	25.240	11.270	37.660	1.00	0.00
ATOM	5843	HW2	SOL	1573	26.710	11.620	37.590	1.00	0.00
ATOM	5844	OW	SOL	1574	22.230	9.960	6.970	1.00	0.00
ATOM	5845	HW1	SOL	1574	22.150	9.290	7.650	1.00	0.00
ATOM	5846	HW2	SOL	1574	21.420	10.470	7.040	1.00	0.00
ATOM	5847	OW	SOL	1575	47.670	13.520	54.600	1.00	0.00
ATOM	5848	HW1	SOL	1575	46.830	13.960	54.640	1.00	0.00
ATOM	5849	HW2	SOL	1575	47.470	12.630	54.310	1.00	0.00
ATOM	5850	OW	SOL	1576	28.790	23.190	28.130	1.00	0.00
ATOM	5851	HW1	SOL	1576	29.260	23.400	27.330	1.00	0.00

ATOM	5852	HW2	SOL	1576	27.880	23.070	27.850	1.00	0.00
ATOM	5853	OW	SOL	1577	20.910	35.870	55.520	1.00	0.00
ATOM	5854	HW1	SOL	1577	21.680	35.380	55.240	1.00	0.00
ATOM	5855	HW2	SOL	1577	21.150	36.250	56.370	1.00	0.00
ATOM	5856	OW	SOL	1578	42.740	53.620	33.010	1.00	0.00
ATOM	5857	HW1	SOL	1578	43.210	54.380	33.350	1.00	0.00
ATOM	5858	HW2	SOL	1578	43.010	53.560	32.090	1.00	0.00
ATOM	5859	OW	SOL	1579	33.270	42.960	50.490	1.00	0.00
ATOM	5860	HW1	SOL	1579	33.170	43.920	50.520	1.00	0.00
ATOM	5861	HW2	SOL	1579	32.690	42.680	49.790	1.00	0.00
ATOM	5862	OW	SOL	1580	42.930	42.980	16.730	1.00	0.00
ATOM	5863	HW1	SOL	1580	43.860	42.820	16.560	1.00	0.00
ATOM	5864	HW2	SOL	1580	42.480	42.570	15.990	1.00	0.00
ATOM	5865	OW	SOL	1581	20.110	38.580	44.770	1.00	0.00
ATOM	5866	HW1	SOL	1581	20.750	37.870	44.820	1.00	0.00
ATOM	5867	HW2	SOL	1581	19.640	38.550	45.600	1.00	0.00
ATOM	5868	OW	SOL	1582	25.630	6.600	53.930	1.00	0.00
ATOM	5869	HW1	SOL	1582	26.330	7.210	53.680	1.00	0.00
ATOM	5870	HW2	SOL	1582	25.020	6.620	53.190	1.00	0.00
ATOM	5871	OW	SOL	1583	8.280	33.060	40.030	1.00	0.00
ATOM	5872	HW1	SOL	1583	8.790	33.820	39.750	1.00	0.00
ATOM	5873	HW2	SOL	1583	8.830	32.300	39.800	1.00	0.00
ATOM	5874	OW	SOL	1584	54.970	31.140	50.000	1.00	0.00
ATOM	5875	HW1	SOL	1584	55.800	30.960	50.440	1.00	0.00
ATOM	5876	HW2	SOL	1584	54.470	31.670	50.620	1.00	0.00
ATOM	5877	OW	SOL	1585	44.950	22.770	10.870	1.00	0.00
ATOM	5878	HW1	SOL	1585	44.600	22.480	11.720	1.00	0.00
ATOM	5879	HW2	SOL	1585	44.250	23.310	10.500	1.00	0.00
ATOM	5880	OW	SOL	1586	41.000	5.620	25.620	1.00	0.00
ATOM	5881	HW1	SOL	1586	40.590	5.800	26.460	1.00	0.00
ATOM	5882	HW2	SOL	1586	40.630	4.780	25.350	1.00	0.00
ATOM	5883	OW	SOL	1587	23.090	7.660	39.020	1.00	0.00
ATOM	5884	HW1	SOL	1587	23.730	7.220	39.580	1.00	0.00
ATOM	5885	HW2	SOL	1587	23.620	8.010	38.300	1.00	0.00
ATOM	5886	OW	SOL	1588	44.410	15.560	50.990	1.00	0.00
ATOM	5887	HW1	SOL	1588	44.560	16.410	51.400	1.00	0.00
ATOM	5888	HW2	SOL	1588	43.560	15.270	51.320	1.00	0.00
ATOM	5889	OW	SOL	1589	30.060	17.830	54.500	1.00	0.00
ATOM	5890	HW1	SOL	1589	29.350	17.700	53.880	1.00	0.00
ATOM	5891	HW2	SOL	1589	29.620	18.060	55.320	1.00	0.00
ATOM	5892	OW	SOL	1590	3.930	39.160	4.080	1.00	0.00
ATOM	5893	HW1	SOL	1590	3.110	38.940	3.640	1.00	0.00
ATOM	5894	HW2	SOL	1590	4.610	38.730	3.560	1.00	0.00
ATOM	5895	OW	SOL	1591	50.450	52.200	29.460	1.00	0.00

ATOM	5896	HW1	SOL	1591	49.720	52.140	28.840	1.00	0.00
ATOM	5897	HW2	SOL	1591	50.850	53.050	29.280	1.00	0.00
ATOM	5898	OW	SOL	1592	35.050	53.310	37.560	1.00	0.00
ATOM	5899	HW1	SOL	1592	35.390	52.900	36.770	1.00	0.00
ATOM	5900	HW2	SOL	1592	34.660	54.130	37.260	1.00	0.00
ATOM	5901	OW	SOL	1593	47.410	11.210	53.260	1.00	0.00
ATOM	5902	HW1	SOL	1593	47.940	11.870	52.800	1.00	0.00
ATOM	5903	HW2	SOL	1593	46.620	11.120	52.730	1.00	0.00
ATOM	5904	OW	SOL	1594	0.810	35.150	6.430	1.00	0.00
ATOM	5905	HW1	SOL	1594	1.430	35.630	6.980	1.00	0.00
ATOM	5906	HW2	SOL	1594	1.240	35.080	5.580	1.00	0.00
ATOM	5907	OW	SOL	1595	48.070	5.140	54.730	1.00	0.00
ATOM	5908	HW1	SOL	1595	48.050	5.100	55.680	1.00	0.00
ATOM	5909	HW2	SOL	1595	47.950	6.060	54.520	1.00	0.00
ATOM	5910	OW	SOL	1596	26.980	31.090	53.690	1.00	0.00
ATOM	5911	HW1	SOL	1596	26.940	30.140	53.840	1.00	0.00
ATOM	5912	HW2	SOL	1596	26.240	31.440	54.200	1.00	0.00
ATOM	5913	OW	SOL	1597	25.530	42.910	24.820	1.00	0.00
ATOM	5914	HW1	SOL	1597	26.230	43.510	24.560	1.00	0.00
ATOM	5915	HW2	SOL	1597	25.010	42.770	24.030	1.00	0.00
ATOM	5916	OW	SOL	1598	51.960	38.700	5.230	1.00	0.00
ATOM	5917	HW1	SOL	1598	52.700	38.610	5.830	1.00	0.00
ATOM	5918	HW2	SOL	1598	52.190	39.450	4.680	1.00	0.00
ATOM	5919	OW	SOL	1599	0.260	11.390	34.730	1.00	0.00
ATOM	5920	HW1	SOL	1599	1.180	11.170	34.900	1.00	0.00
ATOM	5921	HW2	SOL	1599	-0.130	10.570	34.440	1.00	0.00
ATOM	5922	OW	SOL	1600	13.170	10.370	34.230	1.00	0.00
ATOM	5923	HW1	SOL	1600	13.740	10.770	33.570	1.00	0.00
ATOM	5924	HW2	SOL	1600	12.330	10.830	34.130	1.00	0.00
ATOM	5925	OW	SOL	1601	46.480	49.010	32.200	1.00	0.00
ATOM	5926	HW1	SOL	1601	46.650	49.930	31.970	1.00	0.00
ATOM	5927	HW2	SOL	1601	45.990	49.060	33.030	1.00	0.00
ATOM	5928	OW	SOL	1602	14.410	52.640	18.430	1.00	0.00
ATOM	5929	HW1	SOL	1602	14.710	53.280	19.070	1.00	0.00
ATOM	5930	HW2	SOL	1602	14.940	52.820	17.650	1.00	0.00
ATOM	5931	OW	SOL	1603	26.950	15.710	15.180	1.00	0.00
ATOM	5932	HW1	SOL	1603	27.450	16.330	15.710	1.00	0.00
ATOM	5933	HW2	SOL	1603	26.610	15.080	15.820	1.00	0.00
ATOM	5934	OW	SOL	1604	6.690	49.420	33.950	1.00	0.00
ATOM	5935	HW1	SOL	1604	7.390	50.080	34.020	1.00	0.00
ATOM	5936	HW2	SOL	1604	6.420	49.470	33.030	1.00	0.00
ATOM	5937	OW	SOL	1605	27.770	1.370	49.540	1.00	0.00
ATOM	5938	HW1	SOL	1605	27.500	0.460	49.630	1.00	0.00
ATOM	5939	HW2	SOL	1605	27.410	1.640	48.700	1.00	0.00

ATOM	5940	OW	SOL	1606	17.440	39.530	7.460	1.00	0.00
ATOM	5941	HW1	SOL	1606	16.900	39.040	8.080	1.00	0.00
ATOM	5942	HW2	SOL	1606	17.030	39.360	6.610	1.00	0.00
ATOM	5943	OW	SOL	1607	32.160	42.280	13.020	1.00	0.00
ATOM	5944	HW1	SOL	1607	32.400	42.990	13.620	1.00	0.00
ATOM	5945	HW2	SOL	1607	32.250	42.650	12.150	1.00	0.00
ATOM	5946	OW	SOL	1608	20.680	40.950	49.660	1.00	0.00
ATOM	5947	HW1	SOL	1608	21.370	41.580	49.440	1.00	0.00
ATOM	5948	HW2	SOL	1608	20.860	40.710	50.570	1.00	0.00
ATOM	5949	OW	SOL	1609	21.740	37.750	13.810	1.00	0.00
ATOM	5950	HW1	SOL	1609	22.070	36.870	14.040	1.00	0.00
ATOM	5951	HW2	SOL	1609	22.520	38.310	13.850	1.00	0.00
ATOM	5952	OW	SOL	1610	11.180	5.490	52.910	1.00	0.00
ATOM	5953	HW1	SOL	1610	10.950	4.650	53.290	1.00	0.00
ATOM	5954	HW2	SOL	1610	12.130	5.470	52.840	1.00	0.00
ATOM	5955	OW	SOL	1611	40.150	23.040	0.780	1.00	0.00
ATOM	5956	HW1	SOL	1611	41.020	22.720	0.530	1.00	0.00
ATOM	5957	HW2	SOL	1611	39.540	22.400	0.410	1.00	0.00
ATOM	5958	OW	SOL	1612	45.070	8.580	52.880	1.00	0.00
ATOM	5959	HW1	SOL	1612	44.830	7.980	52.180	1.00	0.00
ATOM	5960	HW2	SOL	1612	45.270	9.410	52.450	1.00	0.00
ATOM	5961	OW	SOL	1613	30.680	54.690	28.000	1.00	0.00
ATOM	5962	HW1	SOL	1613	30.430	54.700	28.920	1.00	0.00
ATOM	5963	HW2	SOL	1613	30.790	53.770	27.790	1.00	0.00
ATOM	5964	OW	SOL	1614	38.910	40.200	1.760	1.00	0.00
ATOM	5965	HW1	SOL	1614	39.760	39.760	1.760	1.00	0.00
ATOM	5966	HW2	SOL	1614	38.290	39.530	1.460	1.00	0.00
ATOM	5967	OW	SOL	1615	5.990	11.760	13.600	1.00	0.00
ATOM	5968	HW1	SOL	1615	5.950	12.720	13.630	1.00	0.00
ATOM	5969	HW2	SOL	1615	5.140	11.500	13.250	1.00	0.00
ATOM	5970	OW	SOL	1616	42.480	50.550	53.660	1.00	0.00
ATOM	5971	HW1	SOL	1616	42.560	49.890	52.970	1.00	0.00
ATOM	5972	HW2	SOL	1616	42.680	51.380	53.210	1.00	0.00
ATOM	5973	OW	SOL	1617	7.180	55.320	7.960	1.00	0.00
ATOM	5974	HW1	SOL	1617	7.700	55.650	7.220	1.00	0.00
ATOM	5975	HW2	SOL	1617	7.640	54.540	8.250	1.00	0.00
ATOM	5976	OW	SOL	1618	2.600	21.230	52.430	1.00	0.00
ATOM	5977	HW1	SOL	1618	2.950	20.750	51.680	1.00	0.00
ATOM	5978	HW2	SOL	1618	3.380	21.510	52.920	1.00	0.00
ATOM	5979	OW	SOL	1619	49.390	52.570	21.900	1.00	0.00
ATOM	5980	HW1	SOL	1619	48.870	53.190	21.390	1.00	0.00
ATOM	5981	HW2	SOL	1619	48.940	51.730	21.780	1.00	0.00
ATOM	5982	OW	SOL	1620	2.140	10.050	28.530	1.00	0.00
ATOM	5983	HW1	SOL	1620	1.960	10.170	29.470	1.00	0.00

ATOM	5984	HW2	SOL	1620	1.950	10.900	28.140	1.00	0.00
ATOM	5985	OW	SOL	1621	2.910	38.050	27.080	1.00	0.00
ATOM	5986	HW1	SOL	1621	3.130	38.210	26.160	1.00	0.00
ATOM	5987	HW2	SOL	1621	3.250	38.810	27.550	1.00	0.00
ATOM	5988	OW	SOL	1622	45.150	9.750	29.610	1.00	0.00
ATOM	5989	HW1	SOL	1622	45.890	9.980	30.170	1.00	0.00
ATOM	5990	HW2	SOL	1622	44.740	10.590	29.400	1.00	0.00
ATOM	5991	OW	SOL	1623	9.750	36.230	32.170	1.00	0.00
ATOM	5992	HW1	SOL	1623	10.390	36.910	31.990	1.00	0.00
ATOM	5993	HW2	SOL	1623	8.900	36.660	32.080	1.00	0.00
ATOM	5994	OW	SOL	1624	15.700	14.790	5.370	1.00	0.00
ATOM	5995	HW1	SOL	1624	16.480	14.250	5.500	1.00	0.00
ATOM	5996	HW2	SOL	1624	16.020	15.690	5.400	1.00	0.00
ATOM	5997	OW	SOL	1625	26.640	48.270	28.300	1.00	0.00
ATOM	5998	HW1	SOL	1625	26.070	47.590	28.670	1.00	0.00
ATOM	5999	HW2	SOL	1625	27.000	47.860	27.510	1.00	0.00
ATOM	6000	OW	SOL	1626	52.950	9.540	40.610	1.00	0.00
ATOM	6001	HW1	SOL	1626	53.860	9.370	40.840	1.00	0.00
ATOM	6002	HW2	SOL	1626	52.920	9.450	39.650	1.00	0.00
ATOM	6003	OW	SOL	1627	11.220	54.840	30.200	1.00	0.00
ATOM	6004	HW1	SOL	1627	11.370	55.190	31.080	1.00	0.00
ATOM	6005	HW2	SOL	1627	10.510	55.380	29.850	1.00	0.00
ATOM	6006	OW	SOL	1628	11.430	43.890	45.240	1.00	0.00
ATOM	6007	HW1	SOL	1628	10.970	43.500	44.500	1.00	0.00
ATOM	6008	HW2	SOL	1628	11.680	44.770	44.940	1.00	0.00
ATOM	6009	OW	SOL	1629	52.660	42.580	21.860	1.00	0.00
ATOM	6010	HW1	SOL	1629	52.080	43.080	21.280	1.00	0.00
ATOM	6011	HW2	SOL	1629	53.440	43.130	21.950	1.00	0.00
ATOM	6012	OW	SOL	1630	4.980	51.200	53.370	1.00	0.00
ATOM	6013	HW1	SOL	1630	4.830	50.570	54.080	1.00	0.00
ATOM	6014	HW2	SOL	1630	5.940	51.240	53.290	1.00	0.00
ATOM	6015	OW	SOL	1631	36.880	22.260	22.720	1.00	0.00
ATOM	6016	HW1	SOL	1631	37.600	22.450	22.130	1.00	0.00
ATOM	6017	HW2	SOL	1631	36.760	23.060	23.230	1.00	0.00
ATOM	6018	OW	SOL	1632	24.650	28.460	32.100	1.00	0.00
ATOM	6019	HW1	SOL	1632	25.370	28.260	31.490	1.00	0.00
ATOM	6020	HW2	SOL	1632	24.990	28.200	32.960	1.00	0.00
ATOM	6021	OW	SOL	1633	13.860	49.290	45.060	1.00	0.00
ATOM	6022	HW1	SOL	1633	12.910	49.420	45.010	1.00	0.00
ATOM	6023	HW2	SOL	1633	14.100	49.610	45.930	1.00	0.00
ATOM	6024	OW	SOL	1634	47.850	51.450	45.610	1.00	0.00
ATOM	6025	HW1	SOL	1634	48.160	52.360	45.640	1.00	0.00
ATOM	6026	HW2	SOL	1634	47.050	51.490	45.090	1.00	0.00
ATOM	6027	OW	SOL	1635	0.380	19.410	52.540	1.00	0.00

ATOM	6028	HW1	SOL	1635	1.030	20.080	52.720	1.00	0.00
ATOM	6029	HW2	SOL	1635	0.370	18.860	53.320	1.00	0.00
ATOM	6030	OW	SOL	1636	9.320	33.730	28.460	1.00	0.00
ATOM	6031	HW1	SOL	1636	8.400	33.940	28.580	1.00	0.00
ATOM	6032	HW2	SOL	1636	9.780	34.550	28.610	1.00	0.00
ATOM	6033	OW	SOL	1637	52.540	32.970	2.420	1.00	0.00
ATOM	6034	HW1	SOL	1637	52.880	32.950	3.310	1.00	0.00
ATOM	6035	HW2	SOL	1637	51.670	32.560	2.480	1.00	0.00
ATOM	6036	OW	SOL	1638	26.690	9.900	33.790	1.00	0.00
ATOM	6037	HW1	SOL	1638	26.210	10.550	33.280	1.00	0.00
ATOM	6038	HW2	SOL	1638	27.280	9.490	33.160	1.00	0.00
ATOM	6039	OW	SOL	1639	3.180	23.550	5.200	1.00	0.00
ATOM	6040	HW1	SOL	1639	3.650	24.370	5.060	1.00	0.00
ATOM	6041	HW2	SOL	1639	3.730	23.050	5.800	1.00	0.00
ATOM	6042	OW	SOL	1640	51.500	6.110	7.930	1.00	0.00
ATOM	6043	HW1	SOL	1640	51.680	6.850	8.510	1.00	0.00
ATOM	6044	HW2	SOL	1640	52.360	5.850	7.610	1.00	0.00
ATOM	6045	OW	SOL	1641	22.560	17.890	40.710	1.00	0.00
ATOM	6046	HW1	SOL	1641	22.190	17.040	40.960	1.00	0.00
ATOM	6047	HW2	SOL	1641	23.510	17.760	40.780	1.00	0.00
ATOM	6048	OW	SOL	1642	27.980	8.400	31.800	1.00	0.00
ATOM	6049	HW1	SOL	1642	28.920	8.350	31.940	1.00	0.00
ATOM	6050	HW2	SOL	1642	27.870	8.170	30.870	1.00	0.00
ATOM	6051	OW	SOL	1643	35.100	1.910	50.050	1.00	0.00
ATOM	6052	HW1	SOL	1643	34.430	2.450	49.650	1.00	0.00
ATOM	6053	HW2	SOL	1643	34.770	1.720	50.930	1.00	0.00
ATOM	6054	OW	SOL	1644	29.900	19.370	39.870	1.00	0.00
ATOM	6055	HW1	SOL	1644	28.990	19.550	40.140	1.00	0.00
ATOM	6056	HW2	SOL	1644	29.840	19.210	38.930	1.00	0.00
ATOM	6057	OW	SOL	1645	30.880	18.710	17.500	1.00	0.00
ATOM	6058	HW1	SOL	1645	31.550	18.650	18.180	1.00	0.00
ATOM	6059	HW2	SOL	1645	30.850	19.630	17.260	1.00	0.00
ATOM	6060	OW	SOL	1646	40.980	51.170	24.410	1.00	0.00
ATOM	6061	HW1	SOL	1646	40.560	50.990	25.250	1.00	0.00
ATOM	6062	HW2	SOL	1646	40.610	52.010	24.130	1.00	0.00
ATOM	6063	OW	SOL	1647	15.190	16.120	2.660	1.00	0.00
ATOM	6064	HW1	SOL	1647	14.670	15.940	3.450	1.00	0.00
ATOM	6065	HW2	SOL	1647	15.170	15.300	2.170	1.00	0.00
ATOM	6066	OW	SOL	1648	23.860	4.080	38.600	1.00	0.00
ATOM	6067	HW1	SOL	1648	24.590	3.630	38.170	1.00	0.00
ATOM	6068	HW2	SOL	1648	23.130	3.460	38.550	1.00	0.00
ATOM	6069	OW	SOL	1649	12.620	30.740	24.400	1.00	0.00
ATOM	6070	HW1	SOL	1649	11.820	30.890	23.890	1.00	0.00
ATOM	6071	HW2	SOL	1649	13.050	31.590	24.410	1.00	0.00

ATOM	6072	OW	SOL	1650	9.980	7.610	42.330	1.00	0.00
ATOM	6073	HW1	SOL	1650	10.340	7.870	43.180	1.00	0.00
ATOM	6074	HW2	SOL	1650	10.630	7.000	41.980	1.00	0.00
ATOM	6075	OW	SOL	1651	44.870	41.410	24.590	1.00	0.00
ATOM	6076	HW1	SOL	1651	44.140	40.980	25.030	1.00	0.00
ATOM	6077	HW2	SOL	1651	45.620	40.840	24.740	1.00	0.00
ATOM	6078	OW	SOL	1652	16.930	55.720	2.150	1.00	0.00
ATOM	6079	HW1	SOL	1652	17.330	56.490	1.750	1.00	0.00
ATOM	6080	HW2	SOL	1652	16.910	55.070	1.450	1.00	0.00
ATOM	6081	OW	SOL	1653	17.990	44.900	15.840	1.00	0.00
ATOM	6082	HW1	SOL	1653	18.310	44.050	15.530	1.00	0.00
ATOM	6083	HW2	SOL	1653	17.590	45.300	15.070	1.00	0.00
ATOM	6084	OW	SOL	1654	22.150	15.400	4.810	1.00	0.00
ATOM	6085	HW1	SOL	1654	22.700	15.250	5.580	1.00	0.00
ATOM	6086	HW2	SOL	1654	21.300	15.630	5.160	1.00	0.00
ATOM	6087	OW	SOL	1655	26.370	3.730	34.570	1.00	0.00
ATOM	6088	HW1	SOL	1655	26.680	4.150	33.770	1.00	0.00
ATOM	6089	HW2	SOL	1655	26.480	4.390	35.250	1.00	0.00
ATOM	6090	OW	SOL	1656	2.080	1.560	29.710	1.00	0.00
ATOM	6091	HW1	SOL	1656	1.610	0.740	29.850	1.00	0.00
ATOM	6092	HW2	SOL	1656	3.000	1.310	29.680	1.00	0.00
ATOM	6093	OW	SOL	1657	15.130	16.960	18.240	1.00	0.00
ATOM	6094	HW1	SOL	1657	15.710	17.600	17.820	1.00	0.00
ATOM	6095	HW2	SOL	1657	15.610	16.130	18.200	1.00	0.00
ATOM	6096	OW	SOL	1658	26.040	49.010	43.870	1.00	0.00
ATOM	6097	HW1	SOL	1658	25.390	49.640	43.570	1.00	0.00
ATOM	6098	HW2	SOL	1658	26.840	49.230	43.390	1.00	0.00
ATOM	6099	OW	SOL	1659	54.620	55.300	27.960	1.00	0.00
ATOM	6100	HW1	SOL	1659	55.210	55.170	28.710	1.00	0.00
ATOM	6101	HW2	SOL	1659	55.190	55.210	27.200	1.00	0.00
ATOM	6102	OW	SOL	1660	39.150	8.790	22.010	1.00	0.00
ATOM	6103	HW1	SOL	1660	38.560	9.040	21.290	1.00	0.00
ATOM	6104	HW2	SOL	1660	38.610	8.820	22.790	1.00	0.00
ATOM	6105	OW	SOL	1661	6.930	36.980	12.010	1.00	0.00
ATOM	6106	HW1	SOL	1661	7.150	36.170	11.540	1.00	0.00
ATOM	6107	HW2	SOL	1661	7.510	36.980	12.770	1.00	0.00
ATOM	6108	OW	SOL	1662	55.030	22.030	23.270	1.00	0.00
ATOM	6109	HW1	SOL	1662	54.860	22.870	23.700	1.00	0.00
ATOM	6110	HW2	SOL	1662	55.560	21.540	23.900	1.00	0.00
ATOM	6111	OW	SOL	1663	39.120	20.430	20.490	1.00	0.00
ATOM	6112	HW1	SOL	1663	39.790	20.030	21.040	1.00	0.00
ATOM	6113	HW2	SOL	1663	38.960	19.790	19.800	1.00	0.00
ATOM	6114	OW	SOL	1664	53.580	8.850	53.690	1.00	0.00
ATOM	6115	HW1	SOL	1664	52.890	8.380	54.160	1.00	0.00

ATOM	6116	HW2	SOL	1664	53.430	8.650	52.770	1.00	0.00
ATOM	6117	OW	SOL	1665	27.380	22.290	14.320	1.00	0.00
ATOM	6118	HW1	SOL	1665	27.670	23.200	14.350	1.00	0.00
ATOM	6119	HW2	SOL	1665	28.010	21.850	13.750	1.00	0.00
ATOM	6120	OW	SOL	1666	50.420	22.460	12.820	1.00	0.00
ATOM	6121	HW1	SOL	1666	49.550	22.200	13.110	1.00	0.00
ATOM	6122	HW2	SOL	1666	50.260	23.060	12.090	1.00	0.00
ATOM	6123	OW	SOL	1667	8.130	46.390	9.420	1.00	0.00
ATOM	6124	HW1	SOL	1667	8.610	46.800	10.140	1.00	0.00
ATOM	6125	HW2	SOL	1667	8.460	46.830	8.630	1.00	0.00
ATOM	6126	OW	SOL	1668	6.570	29.910	1.960	1.00	0.00
ATOM	6127	HW1	SOL	1668	6.100	30.700	1.670	1.00	0.00
ATOM	6128	HW2	SOL	1668	5.950	29.470	2.550	1.00	0.00
ATOM	6129	OW	SOL	1669	10.130	10.700	48.070	1.00	0.00
ATOM	6130	HW1	SOL	1669	10.050	9.780	47.820	1.00	0.00
ATOM	6131	HW2	SOL	1669	10.990	10.970	47.740	1.00	0.00
ATOM	6132	OW	SOL	1670	28.350	10.510	28.550	1.00	0.00
ATOM	6133	HW1	SOL	1670	29.210	10.410	28.960	1.00	0.00
ATOM	6134	HW2	SOL	1670	28.010	9.610	28.490	1.00	0.00
ATOM	6135	OW	SOL	1671	2.030	37.900	38.170	1.00	0.00
ATOM	6136	HW1	SOL	1671	1.950	36.990	37.890	1.00	0.00
ATOM	6137	HW2	SOL	1671	2.960	38.100	38.060	1.00	0.00
ATOM	6138	OW	SOL	1672	39.670	43.680	53.050	1.00	0.00
ATOM	6139	HW1	SOL	1672	39.370	44.590	53.160	1.00	0.00
ATOM	6140	HW2	SOL	1672	39.810	43.370	53.940	1.00	0.00
ATOM	6141	OW	SOL	1673	47.440	51.410	25.590	1.00	0.00
ATOM	6142	HW1	SOL	1673	47.910	50.860	24.960	1.00	0.00
ATOM	6143	HW2	SOL	1673	46.540	51.410	25.280	1.00	0.00
ATOM	6144	OW	SOL	1674	42.100	45.800	39.250	1.00	0.00
ATOM	6145	HW1	SOL	1674	42.620	46.440	38.770	1.00	0.00
ATOM	6146	HW2	SOL	1674	42.740	45.180	39.600	1.00	0.00
ATOM	6147	OW	SOL	1675	15.620	54.560	31.810	1.00	0.00
ATOM	6148	HW1	SOL	1675	15.900	53.920	32.470	1.00	0.00
ATOM	6149	HW2	SOL	1675	16.360	54.620	31.210	1.00	0.00
ATOM	6150	OW	SOL	1676	2.900	31.060	37.830	1.00	0.00
ATOM	6151	HW1	SOL	1676	3.600	31.540	37.390	1.00	0.00
ATOM	6152	HW2	SOL	1676	3.340	30.330	38.270	1.00	0.00
ATOM	6153	OW	SOL	1677	6.420	41.880	24.120	1.00	0.00
ATOM	6154	HW1	SOL	1677	7.250	42.360	24.010	1.00	0.00
ATOM	6155	HW2	SOL	1677	6.530	41.410	24.950	1.00	0.00
ATOM	6156	OW	SOL	1678	0.990	11.700	6.640	1.00	0.00
ATOM	6157	HW1	SOL	1678	0.770	12.450	6.090	1.00	0.00
ATOM	6158	HW2	SOL	1678	0.260	11.090	6.520	1.00	0.00
ATOM	6159	OW	SOL	1679	31.510	40.270	5.100	1.00	0.00

ATOM	6160	HW1	SOL	1679	31.140	39.400	5.020	1.00	0.00
ATOM	6161	HW2	SOL	1679	30.810	40.800	5.490	1.00	0.00
ATOM	6162	OW	SOL	1680	1.550	34.000	19.080	1.00	0.00
ATOM	6163	HW1	SOL	1680	2.320	34.060	19.650	1.00	0.00
ATOM	6164	HW2	SOL	1680	1.910	33.870	18.200	1.00	0.00
ATOM	6165	OW	SOL	1681	51.330	52.570	47.460	1.00	0.00
ATOM	6166	HW1	SOL	1681	51.680	51.680	47.560	1.00	0.00
ATOM	6167	HW2	SOL	1681	50.440	52.520	47.800	1.00	0.00
ATOM	6168	OW	SOL	1682	21.700	21.530	52.960	1.00	0.00
ATOM	6169	HW1	SOL	1682	22.570	21.650	52.600	1.00	0.00
ATOM	6170	HW2	SOL	1682	21.810	20.900	53.670	1.00	0.00
ATOM	6171	OW	SOL	1683	19.510	21.450	30.420	1.00	0.00
ATOM	6172	HW1	SOL	1683	19.130	20.870	31.080	1.00	0.00
ATOM	6173	HW2	SOL	1683	19.210	22.320	30.670	1.00	0.00
ATOM	6174	OW	SOL	1684	18.320	38.810	35.770	1.00	0.00
ATOM	6175	HW1	SOL	1684	18.140	38.680	36.700	1.00	0.00
ATOM	6176	HW2	SOL	1684	19.250	38.600	35.670	1.00	0.00
ATOM	6177	OW	SOL	1685	45.520	0.830	55.790	1.00	0.00
ATOM	6178	HW1	SOL	1685	46.140	0.110	55.870	1.00	0.00
ATOM	6179	HW2	SOL	1685	45.810	1.470	56.430	1.00	0.00
ATOM	6180	OW	SOL	1686	48.730	45.160	13.000	1.00	0.00
ATOM	6181	HW1	SOL	1686	48.390	44.620	13.710	1.00	0.00
ATOM	6182	HW2	SOL	1686	48.190	45.940	13.010	1.00	0.00
ATOM	6183	OW	SOL	1687	36.040	13.940	28.540	1.00	0.00
ATOM	6184	HW1	SOL	1687	35.800	13.550	27.700	1.00	0.00
ATOM	6185	HW2	SOL	1687	36.680	13.340	28.910	1.00	0.00
ATOM	6186	OW	SOL	1688	15.710	42.020	45.090	1.00	0.00
ATOM	6187	HW1	SOL	1688	16.200	41.200	45.150	1.00	0.00
ATOM	6188	HW2	SOL	1688	16.380	42.700	44.980	1.00	0.00
ATOM	6189	OW	SOL	1689	13.050	28.780	52.960	1.00	0.00
ATOM	6190	HW1	SOL	1689	12.560	29.590	53.110	1.00	0.00
ATOM	6191	HW2	SOL	1689	13.230	28.440	53.840	1.00	0.00
ATOM	6192	OW	SOL	1690	5.620	0.980	5.270	1.00	0.00
ATOM	6193	HW1	SOL	1690	6.460	0.520	5.360	1.00	0.00
ATOM	6194	HW2	SOL	1690	5.020	0.310	4.940	1.00	0.00
ATOM	6195	OW	SOL	1691	34.200	16.780	0.600	1.00	0.00
ATOM	6196	HW1	SOL	1691	33.540	17.400	0.270	1.00	0.00
ATOM	6197	HW2	SOL	1691	34.750	16.580	-0.160	1.00	0.00
ATOM	6198	OW	SOL	1692	23.720	12.460	5.200	1.00	0.00
ATOM	6199	HW1	SOL	1692	23.680	13.230	5.760	1.00	0.00
ATOM	6200	HW2	SOL	1692	22.900	12.000	5.360	1.00	0.00
ATOM	6201	OW	SOL	1693	41.930	10.340	28.940	1.00	0.00
ATOM	6202	HW1	SOL	1693	42.280	9.950	28.130	1.00	0.00
ATOM	6203	HW2	SOL	1693	42.080	11.280	28.840	1.00	0.00

ATOM	6204	OW	SOL	1694	18.100	33.220	12.670	1.00	0.00
ATOM	6205	HW1	SOL	1694	17.300	33.500	13.100	1.00	0.00
ATOM	6206	HW2	SOL	1694	17.890	33.250	11.730	1.00	0.00
ATOM	6207	OW	SOL	1695	8.790	32.560	54.090	1.00	0.00
ATOM	6208	HW1	SOL	1695	8.710	31.700	54.510	1.00	0.00
ATOM	6209	HW2	SOL	1695	9.010	33.150	54.810	1.00	0.00
ATOM	6210	OW	SOL	1696	51.890	4.590	27.210	1.00	0.00
ATOM	6211	HW1	SOL	1696	52.740	5.020	27.350	1.00	0.00
ATOM	6212	HW2	SOL	1696	51.370	5.230	26.730	1.00	0.00
ATOM	6213	OW	SOL	1697	13.420	39.090	12.570	1.00	0.00
ATOM	6214	HW1	SOL	1697	12.940	39.920	12.500	1.00	0.00
ATOM	6215	HW2	SOL	1697	12.760	38.460	12.860	1.00	0.00
ATOM	6216	OW	SOL	1698	8.130	21.030	54.690	1.00	0.00
ATOM	6217	HW1	SOL	1698	7.420	20.390	54.690	1.00	0.00
ATOM	6218	HW2	SOL	1698	8.740	20.710	54.030	1.00	0.00
ATOM	6219	OW	SOL	1699	26.390	8.920	36.240	1.00	0.00
ATOM	6220	HW1	SOL	1699	26.530	9.550	35.520	1.00	0.00
ATOM	6221	HW2	SOL	1699	25.580	9.210	36.650	1.00	0.00
ATOM	6222	OW	SOL	1700	46.610	53.480	15.630	1.00	0.00
ATOM	6223	HW1	SOL	1700	46.330	54.310	16.010	1.00	0.00
ATOM	6224	HW2	SOL	1700	47.200	53.730	14.920	1.00	0.00
ATOM	6225	OW	SOL	1701	27.330	51.900	5.610	1.00	0.00
ATOM	6226	HW1	SOL	1701	26.510	51.770	6.080	1.00	0.00
ATOM	6227	HW2	SOL	1701	27.820	52.530	6.140	1.00	0.00
ATOM	6228	OW	SOL	1702	44.440	19.670	54.360	1.00	0.00
ATOM	6229	HW1	SOL	1702	44.490	19.590	53.410	1.00	0.00
ATOM	6230	HW2	SOL	1702	44.570	18.770	54.680	1.00	0.00
ATOM	6231	OW	SOL	1703	30.890	14.650	51.890	1.00	0.00
ATOM	6232	HW1	SOL	1703	31.210	14.100	51.180	1.00	0.00
ATOM	6233	HW2	SOL	1703	29.940	14.690	51.760	1.00	0.00
ATOM	6234	OW	SOL	1704	5.200	15.980	33.060	1.00	0.00
ATOM	6235	HW1	SOL	1704	5.570	16.860	33.160	1.00	0.00
ATOM	6236	HW2	SOL	1704	4.510	15.930	33.730	1.00	0.00
ATOM	6237	OW	SOL	1705	19.080	10.110	45.170	1.00	0.00
ATOM	6238	HW1	SOL	1705	19.840	10.290	45.730	1.00	0.00
ATOM	6239	HW2	SOL	1705	19.120	10.780	44.490	1.00	0.00
ATOM	6240	OW	SOL	1706	14.830	29.030	41.470	1.00	0.00
ATOM	6241	HW1	SOL	1706	14.680	29.760	42.060	1.00	0.00
ATOM	6242	HW2	SOL	1706	15.720	29.140	41.150	1.00	0.00
ATOM	6243	OW	SOL	1707	9.510	47.460	34.950	1.00	0.00
ATOM	6244	HW1	SOL	1707	8.550	47.550	35.010	1.00	0.00
ATOM	6245	HW2	SOL	1707	9.750	46.950	35.720	1.00	0.00
ATOM	6246	OW	SOL	1708	19.370	13.690	3.390	1.00	0.00
ATOM	6247	HW1	SOL	1708	18.690	13.370	3.980	1.00	0.00

ATOM	6248	HW2	SOL	1708	19.140	14.610	3.250	1.00	0.00
ATOM	6249	OW	SOL	1709	4.460	29.880	51.280	1.00	0.00
ATOM	6250	HW1	SOL	1709	4.650	30.750	50.940	1.00	0.00
ATOM	6251	HW2	SOL	1709	5.130	29.730	51.950	1.00	0.00
ATOM	6252	OW	SOL	1710	17.000	29.840	12.390	1.00	0.00
ATOM	6253	HW1	SOL	1710	16.460	29.290	11.830	1.00	0.00
ATOM	6254	HW2	SOL	1710	16.430	30.070	13.120	1.00	0.00
ATOM	6255	OW	SOL	1711	2.560	0.590	6.740	1.00	0.00
ATOM	6256	HW1	SOL	1711	3.070	1.010	7.430	1.00	0.00
ATOM	6257	HW2	SOL	1711	2.870	0.980	5.930	1.00	0.00
ATOM	6258	OW	SOL	1712	48.090	20.890	49.300	1.00	0.00
ATOM	6259	HW1	SOL	1712	48.110	20.140	48.710	1.00	0.00
ATOM	6260	HW2	SOL	1712	49.010	21.050	49.520	1.00	0.00
ATOM	6261	OW	SOL	1713	5.800	3.660	46.360	1.00	0.00
ATOM	6262	HW1	SOL	1713	5.650	3.590	47.300	1.00	0.00
ATOM	6263	HW2	SOL	1713	5.220	3.010	45.960	1.00	0.00
ATOM	6264	OW	SOL	1714	0.040	48.030	42.980	1.00	0.00
ATOM	6265	HW1	SOL	1714	0.670	48.320	42.320	1.00	0.00
ATOM	6266	HW2	SOL	1714	-0.790	48.410	42.700	1.00	0.00
ATOM	6267	OW	SOL	1715	54.180	49.470	22.600	1.00	0.00
ATOM	6268	HW1	SOL	1715	54.350	48.550	22.370	1.00	0.00
ATOM	6269	HW2	SOL	1715	53.490	49.740	22.000	1.00	0.00
ATOM	6270	OW	SOL	1716	28.230	0.590	33.510	1.00	0.00
ATOM	6271	HW1	SOL	1716	28.310	1.180	32.760	1.00	0.00
ATOM	6272	HW2	SOL	1716	27.670	-0.120	33.190	1.00	0.00
ATOM	6273	OW	SOL	1717	19.690	54.810	31.010	1.00	0.00
ATOM	6274	HW1	SOL	1717	19.240	54.090	30.570	1.00	0.00
ATOM	6275	HW2	SOL	1717	20.610	54.720	30.730	1.00	0.00
ATOM	6276	OW	SOL	1718	3.080	3.380	41.320	1.00	0.00
ATOM	6277	HW1	SOL	1718	2.440	2.790	40.920	1.00	0.00
ATOM	6278	HW2	SOL	1718	3.910	3.140	40.910	1.00	0.00
ATOM	6279	OW	SOL	1719	30.890	8.240	29.400	1.00	0.00
ATOM	6280	HW1	SOL	1719	31.190	8.170	28.490	1.00	0.00
ATOM	6281	HW2	SOL	1719	30.800	9.180	29.540	1.00	0.00
ATOM	6282	OW	SOL	1720	45.000	8.760	32.330	1.00	0.00
ATOM	6283	HW1	SOL	1720	45.900	9.090	32.340	1.00	0.00
ATOM	6284	HW2	SOL	1720	44.520	9.360	32.900	1.00	0.00
ATOM	6285	OW	SOL	1721	29.710	53.530	36.610	1.00	0.00
ATOM	6286	HW1	SOL	1721	30.020	52.850	37.210	1.00	0.00
ATOM	6287	HW2	SOL	1721	29.800	53.140	35.740	1.00	0.00
ATOM	6288	OW	SOL	1722	35.640	54.990	17.070	1.00	0.00
ATOM	6289	HW1	SOL	1722	35.080	54.450	17.640	1.00	0.00
ATOM	6290	HW2	SOL	1722	35.220	55.850	17.080	1.00	0.00
ATOM	6291	OW	SOL	1723	39.730	16.800	49.110	1.00	0.00

ATOM	6292	HW1	SOL	1723	40.440	16.190	49.310	1.00	0.00
ATOM	6293	HW2	SOL	1723	38.970	16.430	49.550	1.00	0.00
ATOM	6294	OW	SOL	1724	35.300	35.690	47.150	1.00	0.00
ATOM	6295	HW1	SOL	1724	35.990	36.350	47.210	1.00	0.00
ATOM	6296	HW2	SOL	1724	35.680	34.990	46.630	1.00	0.00
ATOM	6297	OW	SOL	1725	24.760	42.210	42.130	1.00	0.00
ATOM	6298	HW1	SOL	1725	24.610	43.000	41.620	1.00	0.00
ATOM	6299	HW2	SOL	1725	25.410	42.460	42.790	1.00	0.00
ATOM	6300	OW	SOL	1726	23.550	33.940	41.710	1.00	0.00
ATOM	6301	HW1	SOL	1726	24.260	33.740	42.320	1.00	0.00
ATOM	6302	HW2	SOL	1726	23.240	34.810	41.970	1.00	0.00
ATOM	6303	OW	SOL	1727	39.770	46.090	27.510	1.00	0.00
ATOM	6304	HW1	SOL	1727	38.870	46.030	27.200	1.00	0.00
ATOM	6305	HW2	SOL	1727	39.700	46.450	28.390	1.00	0.00
ATOM	6306	OW	SOL	1728	16.020	50.980	22.920	1.00	0.00
ATOM	6307	HW1	SOL	1728	15.560	51.580	23.510	1.00	0.00
ATOM	6308	HW2	SOL	1728	15.340	50.630	22.350	1.00	0.00
ATOM	6309	OW	SOL	1729	3.680	27.140	35.910	1.00	0.00
ATOM	6310	HW1	SOL	1729	3.400	26.260	36.160	1.00	0.00
ATOM	6311	HW2	SOL	1729	4.300	27.400	36.590	1.00	0.00
ATOM	6312	OW	SOL	1730	17.480	25.110	5.080	1.00	0.00
ATOM	6313	HW1	SOL	1730	18.020	24.400	4.740	1.00	0.00
ATOM	6314	HW2	SOL	1730	17.850	25.310	5.940	1.00	0.00
ATOM	6315	OW	SOL	1731	40.290	4.740	3.280	1.00	0.00
ATOM	6316	HW1	SOL	1731	39.570	4.300	2.830	1.00	0.00
ATOM	6317	HW2	SOL	1731	39.930	5.590	3.530	1.00	0.00
ATOM	6318	OW	SOL	1732	43.110	1.810	47.850	1.00	0.00
ATOM	6319	HW1	SOL	1732	42.680	2.130	47.060	1.00	0.00
ATOM	6320	HW2	SOL	1732	43.110	0.850	47.750	1.00	0.00
ATOM	6321	OW	SOL	1733	52.300	34.730	11.280	1.00	0.00
ATOM	6322	HW1	SOL	1733	53.000	34.270	10.810	1.00	0.00
ATOM	6323	HW2	SOL	1733	52.750	35.190	11.990	1.00	0.00
ATOM	6324	OW	SOL	1734	51.650	29.200	27.630	1.00	0.00
ATOM	6325	HW1	SOL	1734	51.960	28.670	28.360	1.00	0.00
ATOM	6326	HW2	SOL	1734	50.800	28.820	27.400	1.00	0.00
ATOM	6327	OW	SOL	1735	4.710	0.330	23.100	1.00	0.00
ATOM	6328	HW1	SOL	1735	4.060	-0.200	22.640	1.00	0.00
ATOM	6329	HW2	SOL	1735	4.580	1.220	22.750	1.00	0.00
ATOM	6330	OW	SOL	1736	30.270	20.230	27.190	1.00	0.00
ATOM	6331	HW1	SOL	1736	30.100	19.920	26.300	1.00	0.00
ATOM	6332	HW2	SOL	1736	31.200	20.450	27.190	1.00	0.00
ATOM	6333	OW	SOL	1737	6.170	31.750	48.450	1.00	0.00
ATOM	6334	HW1	SOL	1737	5.250	31.460	48.450	1.00	0.00
ATOM	6335	HW2	SOL	1737	6.130	32.660	48.730	1.00	0.00

ATOM	6336	OW	SOL	1738	39.640	10.580	9.870	1.00	0.00
ATOM	6337	HW1	SOL	1738	39.790	11.330	10.450	1.00	0.00
ATOM	6338	HW2	SOL	1738	40.520	10.250	9.680	1.00	0.00
ATOM	6339	OW	SOL	1739	23.170	42.110	11.990	1.00	0.00
ATOM	6340	HW1	SOL	1739	23.470	41.550	11.280	1.00	0.00
ATOM	6341	HW2	SOL	1739	22.380	42.530	11.650	1.00	0.00
ATOM	6342	OW	SOL	1740	25.010	12.750	51.890	1.00	0.00
ATOM	6343	HW1	SOL	1740	24.870	13.300	52.660	1.00	0.00
ATOM	6344	HW2	SOL	1740	25.320	13.360	51.220	1.00	0.00
ATOM	6345	OW	SOL	1741	45.940	20.940	2.650	1.00	0.00
ATOM	6346	HW1	SOL	1741	45.350	20.470	3.240	1.00	0.00
ATOM	6347	HW2	SOL	1741	45.350	21.420	2.070	1.00	0.00
ATOM	6348	OW	SOL	1742	18.150	31.520	22.020	1.00	0.00
ATOM	6349	HW1	SOL	1742	18.250	32.380	21.620	1.00	0.00
ATOM	6350	HW2	SOL	1742	17.560	31.050	21.430	1.00	0.00
ATOM	6351	OW	SOL	1743	55.160	18.680	30.610	1.00	0.00
ATOM	6352	HW1	SOL	1743	55.370	17.760	30.430	1.00	0.00
ATOM	6353	HW2	SOL	1743	54.200	18.720	30.590	1.00	0.00
ATOM	6354	OW	SOL	1744	1.070	10.940	46.100	1.00	0.00
ATOM	6355	HW1	SOL	1744	1.830	10.780	46.650	1.00	0.00
ATOM	6356	HW2	SOL	1744	1.420	11.030	45.210	1.00	0.00
ATOM	6357	OW	SOL	1745	33.220	39.270	15.820	1.00	0.00
ATOM	6358	HW1	SOL	1745	33.500	38.750	15.070	1.00	0.00
ATOM	6359	HW2	SOL	1745	32.290	39.060	15.930	1.00	0.00
ATOM	6360	OW	SOL	1746	33.000	3.160	48.790	1.00	0.00
ATOM	6361	HW1	SOL	1746	32.190	2.650	48.830	1.00	0.00
ATOM	6362	HW2	SOL	1746	32.710	4.070	48.890	1.00	0.00
ATOM	6363	OW	SOL	1747	23.540	47.970	46.770	1.00	0.00
ATOM	6364	HW1	SOL	1747	24.110	47.350	47.230	1.00	0.00
ATOM	6365	HW2	SOL	1747	22.760	47.460	46.560	1.00	0.00
ATOM	6366	OW	SOL	1748	7.730	46.970	26.770	1.00	0.00
ATOM	6367	HW1	SOL	1748	8.530	47.240	27.210	1.00	0.00
ATOM	6368	HW2	SOL	1748	8.030	46.440	26.030	1.00	0.00
ATOM	6369	OW	SOL	1749	7.400	32.680	51.820	1.00	0.00
ATOM	6370	HW1	SOL	1749	7.780	32.660	52.700	1.00	0.00
ATOM	6371	HW2	SOL	1749	7.820	31.970	51.350	1.00	0.00
ATOM	6372	OW	SOL	1750	37.730	2.870	39.740	1.00	0.00
ATOM	6373	HW1	SOL	1750	36.900	3.330	39.630	1.00	0.00
ATOM	6374	HW2	SOL	1750	37.500	1.940	39.740	1.00	0.00
ATOM	6375	OW	SOL	1751	47.970	19.010	17.030	1.00	0.00
ATOM	6376	HW1	SOL	1751	47.080	18.660	17.070	1.00	0.00
ATOM	6377	HW2	SOL	1751	47.870	19.850	16.580	1.00	0.00
ATOM	6378	OW	SOL	1752	52.640	30.110	46.990	1.00	0.00
ATOM	6379	HW1	SOL	1752	51.770	29.920	46.650	1.00	0.00

ATOM	6380	HW2	SOL	1752	52.510	30.190	47.940	1.00	0.00
ATOM	6381	OW	SOL	1753	30.900	8.160	10.040	1.00	0.00
ATOM	6382	HW1	SOL	1753	30.900	8.500	10.930	1.00	0.00
ATOM	6383	HW2	SOL	1753	30.700	7.220	10.150	1.00	0.00
ATOM	6384	OW	SOL	1754	3.430	41.560	18.550	1.00	0.00
ATOM	6385	HW1	SOL	1754	3.610	40.660	18.310	1.00	0.00
ATOM	6386	HW2	SOL	1754	2.850	41.500	19.300	1.00	0.00
ATOM	6387	OW	SOL	1755	8.840	55.470	11.670	1.00	0.00
ATOM	6388	HW1	SOL	1755	8.700	54.760	11.050	1.00	0.00
ATOM	6389	HW2	SOL	1755	8.060	55.460	12.220	1.00	0.00
ATOM	6390	OW	SOL	1756	41.950	2.600	38.390	1.00	0.00
ATOM	6391	HW1	SOL	1756	42.420	3.290	38.860	1.00	0.00
ATOM	6392	HW2	SOL	1756	41.570	3.050	37.630	1.00	0.00
ATOM	6393	OW	SOL	1757	8.050	4.540	3.790	1.00	0.00
ATOM	6394	HW1	SOL	1757	8.480	4.310	4.610	1.00	0.00
ATOM	6395	HW2	SOL	1757	8.280	3.830	3.190	1.00	0.00
ATOM	6396	OW	SOL	1758	49.230	39.650	51.590	1.00	0.00
ATOM	6397	HW1	SOL	1758	50.180	39.720	51.680	1.00	0.00
ATOM	6398	HW2	SOL	1758	49.100	39.130	50.810	1.00	0.00
ATOM	6399	OW	SOL	1759	15.300	53.770	15.920	1.00	0.00
ATOM	6400	HW1	SOL	1759	14.770	53.730	15.130	1.00	0.00
ATOM	6401	HW2	SOL	1759	16.080	54.260	15.660	1.00	0.00
ATOM	6402	OW	SOL	1760	27.640	9.790	50.560	1.00	0.00
ATOM	6403	HW1	SOL	1760	28.500	10.120	50.270	1.00	0.00
ATOM	6404	HW2	SOL	1760	27.550	10.130	51.450	1.00	0.00
ATOM	6405	OW	SOL	1761	5.710	45.580	53.270	1.00	0.00
ATOM	6406	HW1	SOL	1761	5.460	46.240	52.640	1.00	0.00
ATOM	6407	HW2	SOL	1761	5.130	44.840	53.090	1.00	0.00
ATOM	6408	OW	SOL	1762	29.330	4.470	18.030	1.00	0.00
ATOM	6409	HW1	SOL	1762	29.910	4.180	17.330	1.00	0.00
ATOM	6410	HW2	SOL	1762	28.480	4.590	17.600	1.00	0.00
ATOM	6411	OW	SOL	1763	32.580	32.320	50.240	1.00	0.00
ATOM	6412	HW1	SOL	1763	32.890	33.220	50.310	1.00	0.00
ATOM	6413	HW2	SOL	1763	32.640	32.120	49.310	1.00	0.00
ATOM	6414	OW	SOL	1764	20.580	19.440	50.240	1.00	0.00
ATOM	6415	HW1	SOL	1764	21.060	18.750	50.710	1.00	0.00
ATOM	6416	HW2	SOL	1764	21.190	19.730	49.550	1.00	0.00
ATOM	6417	OW	SOL	1765	25.550	16.990	20.620	1.00	0.00
ATOM	6418	HW1	SOL	1765	26.210	16.290	20.600	1.00	0.00
ATOM	6419	HW2	SOL	1765	25.600	17.390	19.750	1.00	0.00
ATOM	6420	OW	SOL	1766	48.430	54.470	38.070	1.00	0.00
ATOM	6421	HW1	SOL	1766	47.850	54.000	37.470	1.00	0.00
ATOM	6422	HW2	SOL	1766	49.300	54.120	37.900	1.00	0.00
ATOM	6423	OW	SOL	1767	29.170	49.720	15.290	1.00	0.00

ATOM	6424	HW1	SOL	1767	29.850	50.400	15.320	1.00	0.00
ATOM	6425	HW2	SOL	1767	29.560	48.980	15.760	1.00	0.00
ATOM	6426	OW	SOL	1768	28.070	14.850	51.910	1.00	0.00
ATOM	6427	HW1	SOL	1768	27.790	15.010	52.820	1.00	0.00
ATOM	6428	HW2	SOL	1768	27.250	14.660	51.450	1.00	0.00
ATOM	6429	OW	SOL	1769	54.220	50.380	18.600	1.00	0.00
ATOM	6430	HW1	SOL	1769	53.660	50.280	19.370	1.00	0.00
ATOM	6431	HW2	SOL	1769	55.050	50.710	18.940	1.00	0.00
ATOM	6432	OW	SOL	1770	21.890	11.380	22.170	1.00	0.00
ATOM	6433	HW1	SOL	1770	21.190	11.030	22.720	1.00	0.00
ATOM	6434	HW2	SOL	1770	21.610	12.270	21.960	1.00	0.00
ATOM	6435	OW	SOL	1771	31.600	33.360	45.130	1.00	0.00
ATOM	6436	HW1	SOL	1771	32.020	33.940	44.500	1.00	0.00
ATOM	6437	HW2	SOL	1771	31.250	33.950	45.800	1.00	0.00
ATOM	6438	OW	SOL	1772	0.840	17.770	54.850	1.00	0.00
ATOM	6439	HW1	SOL	1772	0.090	17.840	55.450	1.00	0.00
ATOM	6440	HW2	SOL	1772	0.980	16.820	54.770	1.00	0.00
ATOM	6441	OW	SOL	1773	40.980	46.820	3.470	1.00	0.00
ATOM	6442	HW1	SOL	1773	40.190	46.790	4.020	1.00	0.00
ATOM	6443	HW2	SOL	1773	40.690	47.230	2.660	1.00	0.00
ATOM	6444	OW	SOL	1774	55.180	43.360	16.990	1.00	0.00
ATOM	6445	HW1	SOL	1774	54.510	43.780	16.440	1.00	0.00
ATOM	6446	HW2	SOL	1774	56.000	43.790	16.730	1.00	0.00
ATOM	6447	OW	SOL	1775	23.660	29.940	12.200	1.00	0.00
ATOM	6448	HW1	SOL	1775	24.490	30.140	12.630	1.00	0.00
ATOM	6449	HW2	SOL	1775	23.370	30.770	11.830	1.00	0.00
ATOM	6450	OW	SOL	1776	6.500	34.640	1.690	1.00	0.00
ATOM	6451	HW1	SOL	1776	6.100	35.260	2.300	1.00	0.00
ATOM	6452	HW2	SOL	1776	7.310	34.370	2.130	1.00	0.00
ATOM	6453	OW	SOL	1777	50.530	20.030	17.620	1.00	0.00
ATOM	6454	HW1	SOL	1777	50.130	19.660	16.830	1.00	0.00
ATOM	6455	HW2	SOL	1777	50.430	19.340	18.280	1.00	0.00
ATOM	6456	OW	SOL	1778	32.600	0.510	5.960	1.00	0.00
ATOM	6457	HW1	SOL	1778	31.850	0.490	5.370	1.00	0.00
ATOM	6458	HW2	SOL	1778	32.800	1.440	6.080	1.00	0.00
ATOM	6459	OW	SOL	1779	30.870	47.460	54.160	1.00	0.00
ATOM	6460	HW1	SOL	1779	30.790	46.530	53.950	1.00	0.00
ATOM	6461	HW2	SOL	1779	31.700	47.540	54.620	1.00	0.00
ATOM	6462	OW	SOL	1780	12.900	45.370	29.930	1.00	0.00
ATOM	6463	HW1	SOL	1780	13.680	45.530	30.460	1.00	0.00
ATOM	6464	HW2	SOL	1780	12.550	44.550	30.260	1.00	0.00
ATOM	6465	OW	SOL	1781	46.720	13.190	16.610	1.00	0.00
ATOM	6466	HW1	SOL	1781	46.460	12.710	17.400	1.00	0.00
ATOM	6467	HW2	SOL	1781	46.670	14.110	16.870	1.00	0.00

ATOM	6468	OW	SOL	1782	14.170	26.980	38.020	1.00	0.00
ATOM	6469	HW1	SOL	1782	14.680	26.640	37.290	1.00	0.00
ATOM	6470	HW2	SOL	1782	13.850	27.830	37.720	1.00	0.00
ATOM	6471	OW	SOL	1783	20.690	54.550	27.770	1.00	0.00
ATOM	6472	HW1	SOL	1783	20.160	54.840	27.020	1.00	0.00
ATOM	6473	HW2	SOL	1783	20.130	53.910	28.220	1.00	0.00
ATOM	6474	OW	SOL	1784	15.890	21.300	48.240	1.00	0.00
ATOM	6475	HW1	SOL	1784	16.840	21.260	48.310	1.00	0.00
ATOM	6476	HW2	SOL	1784	15.580	21.250	49.140	1.00	0.00
ATOM	6477	OW	SOL	1785	32.270	4.110	32.510	1.00	0.00
ATOM	6478	HW1	SOL	1785	31.330	4.250	32.360	1.00	0.00
ATOM	6479	HW2	SOL	1785	32.700	4.670	31.880	1.00	0.00
ATOM	6480	OW	SOL	1786	36.000	46.820	34.510	1.00	0.00
ATOM	6481	HW1	SOL	1786	36.950	46.700	34.460	1.00	0.00
ATOM	6482	HW2	SOL	1786	35.640	45.940	34.440	1.00	0.00
ATOM	6483	OW	SOL	1787	2.130	16.590	32.360	1.00	0.00
ATOM	6484	HW1	SOL	1787	2.540	15.780	32.660	1.00	0.00
ATOM	6485	HW2	SOL	1787	1.480	16.310	31.710	1.00	0.00
ATOM	6486	OW	SOL	1788	12.990	52.060	37.960	1.00	0.00
ATOM	6487	HW1	SOL	1788	12.450	52.030	38.750	1.00	0.00
ATOM	6488	HW2	SOL	1788	13.050	52.990	37.750	1.00	0.00
ATOM	6489	OW	SOL	1789	53.980	14.470	51.160	1.00	0.00
ATOM	6490	HW1	SOL	1789	54.400	14.280	50.320	1.00	0.00
ATOM	6491	HW2	SOL	1789	54.670	14.300	51.810	1.00	0.00
ATOM	6492	OW	SOL	1790	33.530	48.550	5.040	1.00	0.00
ATOM	6493	HW1	SOL	1790	34.410	48.190	4.910	1.00	0.00
ATOM	6494	HW2	SOL	1790	33.440	49.200	4.340	1.00	0.00
ATOM	6495	OW	SOL	1791	11.800	9.480	51.630	1.00	0.00
ATOM	6496	HW1	SOL	1791	11.060	9.040	51.210	1.00	0.00
ATOM	6497	HW2	SOL	1791	11.500	10.390	51.740	1.00	0.00
ATOM	6498	OW	SOL	1792	33.710	23.800	48.470	1.00	0.00
ATOM	6499	HW1	SOL	1792	34.320	24.530	48.490	1.00	0.00
ATOM	6500	HW2	SOL	1792	34.170	23.110	47.990	1.00	0.00
ATOM	6501	OW	SOL	1793	0.520	37.310	34.160	1.00	0.00
ATOM	6502	HW1	SOL	1793	0.660	36.450	34.550	1.00	0.00
ATOM	6503	HW2	SOL	1793	0.340	37.880	34.910	1.00	0.00
ATOM	6504	OW	SOL	1794	4.060	26.140	12.970	1.00	0.00
ATOM	6505	HW1	SOL	1794	3.960	25.750	12.100	1.00	0.00
ATOM	6506	HW2	SOL	1794	3.350	25.750	13.480	1.00	0.00
ATOM	6507	OW	SOL	1795	47.770	10.600	19.990	1.00	0.00
ATOM	6508	HW1	SOL	1795	48.380	9.920	19.700	1.00	0.00
ATOM	6509	HW2	SOL	1795	48.290	11.400	20.030	1.00	0.00
ATOM	6510	OW	SOL	1796	29.910	21.410	32.770	1.00	0.00
ATOM	6511	HW1	SOL	1796	30.000	22.060	32.080	1.00	0.00

ATOM	6512	HW2	SOL	1796	30.480	20.690	32.490	1.00	0.00
ATOM	6513	OW	SOL	1797	10.090	35.070	39.220	1.00	0.00
ATOM	6514	HW1	SOL	1797	10.960	34.890	39.580	1.00	0.00
ATOM	6515	HW2	SOL	1797	10.240	35.250	38.300	1.00	0.00
ATOM	6516	OW	SOL	1798	20.420	35.020	25.670	1.00	0.00
ATOM	6517	HW1	SOL	1798	20.020	34.420	26.310	1.00	0.00
ATOM	6518	HW2	SOL	1798	20.720	35.760	26.190	1.00	0.00
ATOM	6519	OW	SOL	1799	9.160	18.390	51.060	1.00	0.00
ATOM	6520	HW1	SOL	1799	9.850	18.090	50.470	1.00	0.00
ATOM	6521	HW2	SOL	1799	8.390	18.510	50.500	1.00	0.00
ATOM	6522	OW	SOL	1800	34.890	6.260	16.880	1.00	0.00
ATOM	6523	HW1	SOL	1800	35.750	5.830	16.900	1.00	0.00
ATOM	6524	HW2	SOL	1800	34.990	7.010	17.470	1.00	0.00
ATOM	6525	OW	SOL	1801	55.670	39.020	55.380	1.00	0.00
ATOM	6526	HW1	SOL	1801	56.100	39.770	54.970	1.00	0.00
ATOM	6527	HW2	SOL	1801	55.080	38.690	54.710	1.00	0.00
ATOM	6528	OW	SOL	1802	39.960	35.820	12.660	1.00	0.00
ATOM	6529	HW1	SOL	1802	40.330	35.560	11.820	1.00	0.00
ATOM	6530	HW2	SOL	1802	40.190	36.750	12.740	1.00	0.00
ATOM	6531	OW	SOL	1803	35.810	34.460	21.240	1.00	0.00
ATOM	6532	HW1	SOL	1803	35.780	34.920	22.080	1.00	0.00
ATOM	6533	HW2	SOL	1803	35.430	35.080	20.610	1.00	0.00
ATOM	6534	OW	SOL	1804	32.100	28.550	22.260	1.00	0.00
ATOM	6535	HW1	SOL	1804	31.640	27.740	22.480	1.00	0.00
ATOM	6536	HW2	SOL	1804	32.470	28.390	21.390	1.00	0.00
ATOM	6537	OW	SOL	1805	49.130	14.420	17.750	1.00	0.00
ATOM	6538	HW1	SOL	1805	48.590	14.110	17.030	1.00	0.00
ATOM	6539	HW2	SOL	1805	49.860	14.870	17.330	1.00	0.00
ATOM	6540	OW	SOL	1806	18.920	23.050	4.060	1.00	0.00
ATOM	6541	HW1	SOL	1806	18.420	22.270	3.820	1.00	0.00
ATOM	6542	HW2	SOL	1806	19.460	22.770	4.800	1.00	0.00
ATOM	6543	OW	SOL	1807	20.540	7.720	38.100	1.00	0.00
ATOM	6544	HW1	SOL	1807	20.440	8.500	38.640	1.00	0.00
ATOM	6545	HW2	SOL	1807	21.450	7.460	38.220	1.00	0.00
ATOM	6546	OW	SOL	1808	33.180	5.760	41.460	1.00	0.00
ATOM	6547	HW1	SOL	1808	33.110	5.590	42.400	1.00	0.00
ATOM	6548	HW2	SOL	1808	32.320	6.100	41.210	1.00	0.00
ATOM	6549	OW	SOL	1809	31.030	12.680	25.320	1.00	0.00
ATOM	6550	HW1	SOL	1809	31.430	11.820	25.440	1.00	0.00
ATOM	6551	HW2	SOL	1809	31.730	13.300	25.470	1.00	0.00
ATOM	6552	OW	SOL	1810	34.340	48.170	37.120	1.00	0.00
ATOM	6553	HW1	SOL	1810	33.690	48.130	36.420	1.00	0.00
ATOM	6554	HW2	SOL	1810	35.160	48.370	36.680	1.00	0.00
ATOM	6555	OW	SOL	1811	19.710	16.560	17.000	1.00	0.00

ATOM	6556	HW1	SOL	1811	19.480	16.360	17.910	1.00	0.00
ATOM	6557	HW2	SOL	1811	20.400	17.210	17.070	1.00	0.00
ATOM	6558	OW	SOL	1812	52.640	36.850	43.830	1.00	0.00
ATOM	6559	HW1	SOL	1812	53.430	36.440	43.470	1.00	0.00
ATOM	6560	HW2	SOL	1812	52.420	37.530	43.200	1.00	0.00
ATOM	6561	OW	SOL	1813	51.940	8.900	33.510	1.00	0.00
ATOM	6562	HW1	SOL	1813	51.590	9.750	33.240	1.00	0.00
ATOM	6563	HW2	SOL	1813	51.460	8.680	34.310	1.00	0.00
ATOM	6564	OW	SOL	1814	3.650	37.290	48.160	1.00	0.00
ATOM	6565	HW1	SOL	1814	3.720	36.440	47.730	1.00	0.00
ATOM	6566	HW2	SOL	1814	2.990	37.150	48.850	1.00	0.00
ATOM	6567	OW	SOL	1815	37.200	48.260	28.740	1.00	0.00
ATOM	6568	HW1	SOL	1815	37.320	47.340	28.990	1.00	0.00
ATOM	6569	HW2	SOL	1815	36.270	48.440	28.880	1.00	0.00
ATOM	6570	OW	SOL	1816	53.200	32.690	9.620	1.00	0.00
ATOM	6571	HW1	SOL	1816	52.560	32.240	10.170	1.00	0.00
ATOM	6572	HW2	SOL	1816	54.020	32.210	9.770	1.00	0.00
ATOM	6573	OW	SOL	1817	16.630	33.640	42.880	1.00	0.00
ATOM	6574	HW1	SOL	1817	16.320	33.860	43.760	1.00	0.00
ATOM	6575	HW2	SOL	1817	16.350	34.380	42.340	1.00	0.00
ATOM	6576	OW	SOL	1818	9.360	46.030	23.920	1.00	0.00
ATOM	6577	HW1	SOL	1818	9.730	46.840	24.290	1.00	0.00
ATOM	6578	HW2	SOL	1818	10.000	45.760	23.260	1.00	0.00
ATOM	6579	OW	SOL	1819	9.900	51.520	40.120	1.00	0.00
ATOM	6580	HW1	SOL	1819	9.350	51.500	40.910	1.00	0.00
ATOM	6581	HW2	SOL	1819	10.790	51.590	40.460	1.00	0.00
ATOM	6582	OW	SOL	1820	5.320	37.110	28.410	1.00	0.00
ATOM	6583	HW1	SOL	1820	4.980	38.000	28.480	1.00	0.00
ATOM	6584	HW2	SOL	1820	4.570	36.550	28.630	1.00	0.00
ATOM	6585	OW	SOL	1821	20.200	0.510	0.920	1.00	0.00
ATOM	6586	HW1	SOL	1821	20.930	0.310	1.510	1.00	0.00
ATOM	6587	HW2	SOL	1821	20.390	0.010	0.130	1.00	0.00
ATOM	6588	OW	SOL	1822	18.860	45.870	25.320	1.00	0.00
ATOM	6589	HW1	SOL	1822	19.660	45.590	24.880	1.00	0.00
ATOM	6590	HW2	SOL	1822	18.920	46.830	25.330	1.00	0.00
ATOM	6591	OW	SOL	1823	1.720	29.510	47.060	1.00	0.00
ATOM	6592	HW1	SOL	1823	1.010	28.950	46.750	1.00	0.00
ATOM	6593	HW2	SOL	1823	2.480	28.930	47.090	1.00	0.00
ATOM	6594	OW	SOL	1824	54.930	5.910	9.600	1.00	0.00
ATOM	6595	HW1	SOL	1824	55.340	6.510	10.220	1.00	0.00
ATOM	6596	HW2	SOL	1824	53.990	5.980	9.780	1.00	0.00
ATOM	6597	OW	SOL	1825	54.810	46.240	35.040	1.00	0.00
ATOM	6598	HW1	SOL	1825	54.560	46.460	35.940	1.00	0.00
ATOM	6599	HW2	SOL	1825	54.020	45.870	34.650	1.00	0.00

ATOM	6600	OW	SOL	1826	27.400	45.440	2.350	1.00	0.00
ATOM	6601	HW1	SOL	1826	28.330	45.240	2.210	1.00	0.00
ATOM	6602	HW2	SOL	1826	26.990	45.250	1.500	1.00	0.00
ATOM	6603	OW	SOL	1827	50.770	52.250	53.830	1.00	0.00
ATOM	6604	HW1	SOL	1827	50.350	52.890	54.410	1.00	0.00
ATOM	6605	HW2	SOL	1827	50.070	51.650	53.590	1.00	0.00
ATOM	6606	OW	SOL	1828	27.120	5.170	21.770	1.00	0.00
ATOM	6607	HW1	SOL	1828	26.270	5.380	21.380	1.00	0.00
ATOM	6608	HW2	SOL	1828	27.730	5.770	21.340	1.00	0.00
ATOM	6609	OW	SOL	1829	30.620	47.360	49.800	1.00	0.00
ATOM	6610	HW1	SOL	1829	30.060	47.970	49.310	1.00	0.00
ATOM	6611	HW2	SOL	1829	31.000	47.900	50.500	1.00	0.00
ATOM	6612	OW	SOL	1830	34.120	44.820	11.840	1.00	0.00
ATOM	6613	HW1	SOL	1830	33.290	44.550	11.450	1.00	0.00
ATOM	6614	HW2	SOL	1830	34.780	44.620	11.170	1.00	0.00
ATOM	6615	OW	SOL	1831	25.040	34.240	9.820	1.00	0.00
ATOM	6616	HW1	SOL	1831	25.810	34.610	10.250	1.00	0.00
ATOM	6617	HW2	SOL	1831	24.920	34.790	9.040	1.00	0.00
ATOM	6618	OW	SOL	1832	32.950	28.050	11.270	1.00	0.00
ATOM	6619	HW1	SOL	1832	33.900	27.970	11.350	1.00	0.00
ATOM	6620	HW2	SOL	1832	32.750	27.700	10.400	1.00	0.00
ATOM	6621	OW	SOL	1833	16.170	21.690	39.080	1.00	0.00
ATOM	6622	HW1	SOL	1833	15.910	20.780	38.940	1.00	0.00
ATOM	6623	HW2	SOL	1833	15.740	21.950	39.890	1.00	0.00
ATOM	6624	OW	SOL	1834	13.130	31.210	33.880	1.00	0.00
ATOM	6625	HW1	SOL	1834	14.050	31.200	33.650	1.00	0.00
ATOM	6626	HW2	SOL	1834	12.740	30.510	33.360	1.00	0.00
ATOM	6627	OW	SOL	1835	19.920	37.910	22.600	1.00	0.00
ATOM	6628	HW1	SOL	1835	19.270	38.600	22.500	1.00	0.00
ATOM	6629	HW2	SOL	1835	20.600	38.130	21.970	1.00	0.00
ATOM	6630	OW	SOL	1836	21.070	35.530	31.810	1.00	0.00
ATOM	6631	HW1	SOL	1836	21.990	35.260	31.770	1.00	0.00
ATOM	6632	HW2	SOL	1836	20.580	34.750	31.550	1.00	0.00
ATOM	6633	OW	SOL	1837	33.680	6.590	46.650	1.00	0.00
ATOM	6634	HW1	SOL	1837	32.810	6.430	47.020	1.00	0.00
ATOM	6635	HW2	SOL	1837	33.630	7.480	46.310	1.00	0.00
ATOM	6636	OW	SOL	1838	5.390	5.130	3.100	1.00	0.00
ATOM	6637	HW1	SOL	1838	6.180	5.290	3.620	1.00	0.00
ATOM	6638	HW2	SOL	1838	4.700	5.620	3.550	1.00	0.00
ATOM	6639	OW	SOL	1839	47.110	0.020	12.190	1.00	0.00
ATOM	6640	HW1	SOL	1839	46.290	-0.170	11.730	1.00	0.00
ATOM	6641	HW2	SOL	1839	46.850	0.620	12.890	1.00	0.00
ATOM	6642	OW	SOL	1840	28.240	21.100	25.400	1.00	0.00
ATOM	6643	HW1	SOL	1840	27.830	20.730	26.180	1.00	0.00

ATOM	6644	HW2	SOL	1840	28.730	21.860	25.730	1.00	0.00
ATOM	6645	OW	SOL	1841	1.920	44.920	52.510	1.00	0.00
ATOM	6646	HW1	SOL	1841	1.200	44.320	52.690	1.00	0.00
ATOM	6647	HW2	SOL	1841	2.230	44.670	51.640	1.00	0.00
ATOM	6648	OW	SOL	1842	12.660	17.340	3.740	1.00	0.00
ATOM	6649	HW1	SOL	1842	12.190	17.490	2.920	1.00	0.00
ATOM	6650	HW2	SOL	1842	12.380	18.050	4.310	1.00	0.00
ATOM	6651	OW	SOL	1843	52.440	18.840	49.730	1.00	0.00
ATOM	6652	HW1	SOL	1843	53.130	18.620	49.110	1.00	0.00
ATOM	6653	HW2	SOL	1843	52.770	18.540	50.580	1.00	0.00
ATOM	6654	OW	SOL	1844	23.150	33.900	44.680	1.00	0.00
ATOM	6655	HW1	SOL	1844	22.850	33.080	45.060	1.00	0.00
ATOM	6656	HW2	SOL	1844	23.970	33.680	44.240	1.00	0.00
ATOM	6657	OW	SOL	1845	24.150	16.350	16.530	1.00	0.00
ATOM	6658	HW1	SOL	1845	24.680	16.780	15.860	1.00	0.00
ATOM	6659	HW2	SOL	1845	23.760	15.600	16.080	1.00	0.00
ATOM	6660	OW	SOL	1846	44.210	14.470	31.180	1.00	0.00
ATOM	6661	HW1	SOL	1846	43.560	14.900	31.740	1.00	0.00
ATOM	6662	HW2	SOL	1846	43.870	13.580	31.060	1.00	0.00
ATOM	6663	OW	SOL	1847	2.000	28.740	51.680	1.00	0.00
ATOM	6664	HW1	SOL	1847	1.550	29.580	51.690	1.00	0.00
ATOM	6665	HW2	SOL	1847	2.920	28.960	51.510	1.00	0.00
ATOM	6666	OW	SOL	1848	27.350	39.810	48.110	1.00	0.00
ATOM	6667	HW1	SOL	1848	28.010	39.210	48.450	1.00	0.00
ATOM	6668	HW2	SOL	1848	27.370	40.550	48.720	1.00	0.00
ATOM	6669	OW	SOL	1849	30.060	17.590	25.710	1.00	0.00
ATOM	6670	HW1	SOL	1849	29.670	18.050	24.970	1.00	0.00
ATOM	6671	HW2	SOL	1849	29.680	16.710	25.680	1.00	0.00
ATOM	6672	OW	SOL	1850	46.690	5.910	45.610	1.00	0.00
ATOM	6673	HW1	SOL	1850	47.300	6.630	45.740	1.00	0.00
ATOM	6674	HW2	SOL	1850	45.830	6.270	45.820	1.00	0.00
ATOM	6675	OW	SOL	1851	41.440	20.280	27.850	1.00	0.00
ATOM	6676	HW1	SOL	1851	41.350	19.330	27.890	1.00	0.00
ATOM	6677	HW2	SOL	1851	40.670	20.610	28.310	1.00	0.00
ATOM	6678	OW	SOL	1852	49.330	30.310	4.170	1.00	0.00
ATOM	6679	HW1	SOL	1852	49.030	29.620	4.770	1.00	0.00
ATOM	6680	HW2	SOL	1852	49.310	31.110	4.690	1.00	0.00
ATOM	6681	OW	SOL	1853	53.990	38.480	19.990	1.00	0.00
ATOM	6682	HW1	SOL	1853	54.260	38.700	20.880	1.00	0.00
ATOM	6683	HW2	SOL	1853	54.750	38.690	19.450	1.00	0.00
ATOM	6684	OW	SOL	1854	0.170	1.310	18.140	1.00	0.00
ATOM	6685	HW1	SOL	1854	0.880	1.410	18.770	1.00	0.00
ATOM	6686	HW2	SOL	1854	-0.000	2.200	17.820	1.00	0.00
ATOM	6687	OW	SOL	1855	47.660	16.940	0.470	1.00	0.00

ATOM	6688	HW1	SOL	1855	48.280	16.810	-0.250	1.00	0.00
ATOM	6689	HW2	SOL	1855	47.960	17.740	0.910	1.00	0.00
ATOM	6690	OW	SOL	1856	19.300	54.530	3.400	1.00	0.00
ATOM	6691	HW1	SOL	1856	18.530	55.080	3.240	1.00	0.00
ATOM	6692	HW2	SOL	1856	19.130	54.120	4.240	1.00	0.00
ATOM	6693	OW	SOL	1857	15.740	28.120	20.950	1.00	0.00
ATOM	6694	HW1	SOL	1857	16.400	27.770	21.540	1.00	0.00
ATOM	6695	HW2	SOL	1857	15.830	29.070	21.020	1.00	0.00
ATOM	6696	OW	SOL	1858	11.850	27.720	44.430	1.00	0.00
ATOM	6697	HW1	SOL	1858	11.970	28.660	44.390	1.00	0.00
ATOM	6698	HW2	SOL	1858	10.900	27.590	44.320	1.00	0.00
ATOM	6699	OW	SOL	1859	36.800	52.240	53.760	1.00	0.00
ATOM	6700	HW1	SOL	1859	36.230	52.610	54.430	1.00	0.00
ATOM	6701	HW2	SOL	1859	37.440	51.720	54.250	1.00	0.00
ATOM	6702	OW	SOL	1860	9.580	51.900	2.500	1.00	0.00
ATOM	6703	HW1	SOL	1860	9.070	51.140	2.790	1.00	0.00
ATOM	6704	HW2	SOL	1860	10.140	51.550	1.800	1.00	0.00
ATOM	6705	OW	SOL	1861	39.010	49.720	30.230	1.00	0.00
ATOM	6706	HW1	SOL	1861	38.170	49.280	30.100	1.00	0.00
ATOM	6707	HW2	SOL	1861	38.800	50.660	30.190	1.00	0.00
ATOM	6708	OW	SOL	1862	55.870	10.790	15.990	1.00	0.00
ATOM	6709	HW1	SOL	1862	56.200	11.640	16.270	1.00	0.00
ATOM	6710	HW2	SOL	1862	56.150	10.180	16.680	1.00	0.00
ATOM	6711	OW	SOL	1863	16.910	10.400	29.690	1.00	0.00
ATOM	6712	HW1	SOL	1863	16.530	9.540	29.500	1.00	0.00
ATOM	6713	HW2	SOL	1863	16.580	10.610	30.570	1.00	0.00
ATOM	6714	OW	SOL	1864	46.090	52.510	4.670	1.00	0.00
ATOM	6715	HW1	SOL	1864	45.580	51.930	4.100	1.00	0.00
ATOM	6716	HW2	SOL	1864	46.240	51.990	5.470	1.00	0.00
ATOM	6717	OW	SOL	1865	34.700	50.460	44.570	1.00	0.00
ATOM	6718	HW1	SOL	1865	35.270	50.030	45.210	1.00	0.00
ATOM	6719	HW2	SOL	1865	35.240	50.580	43.800	1.00	0.00
ATOM	6720	OW	SOL	1866	11.060	43.660	31.110	1.00	0.00
ATOM	6721	HW1	SOL	1866	10.460	44.020	31.770	1.00	0.00
ATOM	6722	HW2	SOL	1866	11.230	42.770	31.410	1.00	0.00
ATOM	6723	OW	SOL	1867	30.900	19.470	7.890	1.00	0.00
ATOM	6724	HW1	SOL	1867	30.700	19.110	8.750	1.00	0.00
ATOM	6725	HW2	SOL	1867	31.810	19.740	7.950	1.00	0.00
ATOM	6726	OW	SOL	1868	32.220	49.850	45.810	1.00	0.00
ATOM	6727	HW1	SOL	1868	32.720	49.370	46.470	1.00	0.00
ATOM	6728	HW2	SOL	1868	32.760	49.830	45.020	1.00	0.00
ATOM	6729	OW	SOL	1869	15.410	38.710	17.930	1.00	0.00
ATOM	6730	HW1	SOL	1869	15.740	37.930	17.480	1.00	0.00
ATOM	6731	HW2	SOL	1869	15.850	39.440	17.500	1.00	0.00

ATOM	6732	OW	SOL	1870	55.340	34.950	30.960	1.00	0.00
ATOM	6733	HW1	SOL	1870	55.160	34.050	30.670	1.00	0.00
ATOM	6734	HW2	SOL	1870	54.920	35.010	31.820	1.00	0.00
ATOM	6735	OW	SOL	1871	48.790	50.000	5.120	1.00	0.00
ATOM	6736	HW1	SOL	1871	48.370	50.440	4.390	1.00	0.00
ATOM	6737	HW2	SOL	1871	48.250	49.230	5.280	1.00	0.00
ATOM	6738	OW	SOL	1872	14.260	48.540	16.250	1.00	0.00
ATOM	6739	HW1	SOL	1872	14.200	49.250	15.610	1.00	0.00
ATOM	6740	HW2	SOL	1872	15.200	48.370	16.340	1.00	0.00
ATOM	6741	OW	SOL	1873	23.630	20.980	33.650	1.00	0.00
ATOM	6742	HW1	SOL	1873	24.150	21.630	33.170	1.00	0.00
ATOM	6743	HW2	SOL	1873	23.380	21.420	34.460	1.00	0.00
ATOM	6744	OW	SOL	1874	31.110	50.100	53.330	1.00	0.00
ATOM	6745	HW1	SOL	1874	30.300	50.610	53.260	1.00	0.00
ATOM	6746	HW2	SOL	1874	30.830	49.230	53.610	1.00	0.00
ATOM	6747	OW	SOL	1875	21.250	2.620	17.860	1.00	0.00
ATOM	6748	HW1	SOL	1875	22.080	3.070	17.710	1.00	0.00
ATOM	6749	HW2	SOL	1875	21.500	1.730	18.130	1.00	0.00
ATOM	6750	OW	SOL	1876	41.370	21.800	9.690	1.00	0.00
ATOM	6751	HW1	SOL	1876	40.490	21.760	10.050	1.00	0.00
ATOM	6752	HW2	SOL	1876	41.510	22.720	9.480	1.00	0.00
ATOM	6753	OW	SOL	1877	35.010	17.160	17.400	1.00	0.00
ATOM	6754	HW1	SOL	1877	35.370	17.240	16.510	1.00	0.00
ATOM	6755	HW2	SOL	1877	35.570	16.520	17.830	1.00	0.00
ATOM	6756	OW	SOL	1878	30.630	54.990	16.100	1.00	0.00
ATOM	6757	HW1	SOL	1878	30.290	55.610	16.740	1.00	0.00
ATOM	6758	HW2	SOL	1878	29.970	54.960	15.410	1.00	0.00
ATOM	6759	OW	SOL	1879	33.410	44.480	14.420	1.00	0.00
ATOM	6760	HW1	SOL	1879	33.880	44.720	13.610	1.00	0.00
ATOM	6761	HW2	SOL	1879	33.860	43.690	14.730	1.00	0.00
ATOM	6762	OW	SOL	1880	4.000	9.370	6.860	1.00	0.00
ATOM	6763	HW1	SOL	1880	3.330	8.690	6.970	1.00	0.00
ATOM	6764	HW2	SOL	1880	4.670	9.160	7.510	1.00	0.00
ATOM	6765	OW	SOL	1881	33.030	27.090	8.490	1.00	0.00
ATOM	6766	HW1	SOL	1881	32.700	27.330	7.620	1.00	0.00
ATOM	6767	HW2	SOL	1881	32.760	26.180	8.610	1.00	0.00
ATOM	6768	OW	SOL	1882	9.810	14.800	49.210	1.00	0.00
ATOM	6769	HW1	SOL	1882	10.050	14.380	48.380	1.00	0.00
ATOM	6770	HW2	SOL	1882	9.130	15.430	48.970	1.00	0.00
ATOM	6771	OW	SOL	1883	17.560	6.580	19.050	1.00	0.00
ATOM	6772	HW1	SOL	1883	18.510	6.510	18.920	1.00	0.00
ATOM	6773	HW2	SOL	1883	17.360	5.920	19.710	1.00	0.00
ATOM	6774	OW	SOL	1884	41.030	1.480	25.180	1.00	0.00
ATOM	6775	HW1	SOL	1884	41.820	1.710	24.680	1.00	0.00

ATOM	6776	HW2	SOL	1884	41.250	1.700	26.090	1.00	0.00
ATOM	6777	OW	SOL	1885	54.140	31.130	52.760	1.00	0.00
ATOM	6778	HW1	SOL	1885	54.990	31.410	53.080	1.00	0.00
ATOM	6779	HW2	SOL	1885	53.750	31.920	52.380	1.00	0.00
ATOM	6780	OW	SOL	1886	20.210	6.150	19.900	1.00	0.00
ATOM	6781	HW1	SOL	1886	19.810	6.800	20.480	1.00	0.00
ATOM	6782	HW2	SOL	1886	21.100	6.470	19.760	1.00	0.00
ATOM	6783	OW	SOL	1887	29.710	15.740	44.990	1.00	0.00
ATOM	6784	HW1	SOL	1887	29.990	16.360	45.660	1.00	0.00
ATOM	6785	HW2	SOL	1887	29.190	16.260	44.380	1.00	0.00
ATOM	6786	OW	SOL	1888	19.330	44.120	10.800	1.00	0.00
ATOM	6787	HW1	SOL	1888	18.680	43.460	10.560	1.00	0.00
ATOM	6788	HW2	SOL	1888	19.430	44.660	10.020	1.00	0.00
ATOM	6789	OW	SOL	1889	41.940	4.780	41.530	1.00	0.00
ATOM	6790	HW1	SOL	1889	41.110	4.630	41.070	1.00	0.00
ATOM	6791	HW2	SOL	1889	41.860	5.680	41.850	1.00	0.00
ATOM	6792	OW	SOL	1890	7.190	41.570	39.860	1.00	0.00
ATOM	6793	HW1	SOL	1890	6.570	40.850	39.700	1.00	0.00
ATOM	6794	HW2	SOL	1890	8.050	41.160	39.760	1.00	0.00
ATOM	6795	OW	SOL	1891	3.120	11.490	16.670	1.00	0.00
ATOM	6796	HW1	SOL	1891	2.570	11.780	17.400	1.00	0.00
ATOM	6797	HW2	SOL	1891	2.760	10.640	16.420	1.00	0.00
ATOM	6798	OW	SOL	1892	36.660	17.770	31.840	1.00	0.00
ATOM	6799	HW1	SOL	1892	37.500	17.350	32.020	1.00	0.00
ATOM	6800	HW2	SOL	1892	36.110	17.070	31.490	1.00	0.00
ATOM	6801	OW	SOL	1893	39.920	5.690	28.010	1.00	0.00
ATOM	6802	HW1	SOL	1893	39.550	4.810	27.950	1.00	0.00
ATOM	6803	HW2	SOL	1893	39.170	6.270	28.090	1.00	0.00
ATOM	6804	OW	SOL	1894	33.650	19.170	15.340	1.00	0.00
ATOM	6805	HW1	SOL	1894	33.640	18.830	16.240	1.00	0.00
ATOM	6806	HW2	SOL	1894	33.030	18.620	14.870	1.00	0.00
ATOM	6807	OW	SOL	1895	11.440	9.350	54.420	1.00	0.00
ATOM	6808	HW1	SOL	1895	11.820	9.480	53.550	1.00	0.00
ATOM	6809	HW2	SOL	1895	11.300	10.240	54.760	1.00	0.00
ATOM	6810	OW	SOL	1896	26.440	51.140	50.710	1.00	0.00
ATOM	6811	HW1	SOL	1896	25.620	51.270	51.190	1.00	0.00
ATOM	6812	HW2	SOL	1896	27.120	51.160	51.390	1.00	0.00
ATOM	6813	OW	SOL	1897	37.100	20.230	33.480	1.00	0.00
ATOM	6814	HW1	SOL	1897	37.690	19.570	33.120	1.00	0.00
ATOM	6815	HW2	SOL	1897	37.390	20.360	34.380	1.00	0.00
ATOM	6816	OW	SOL	1898	35.760	3.000	24.870	1.00	0.00
ATOM	6817	HW1	SOL	1898	36.490	2.460	24.570	1.00	0.00
ATOM	6818	HW2	SOL	1898	35.890	3.840	24.430	1.00	0.00
ATOM	6819	OW	SOL	1899	53.360	5.890	33.540	1.00	0.00

ATOM	6820	HW1	SOL	1899	53.400	4.940	33.510	1.00	0.00
ATOM	6821	HW2	SOL	1899	52.420	6.090	33.560	1.00	0.00
ATOM	6822	OW	SOL	1900	50.310	19.580	21.600	1.00	0.00
ATOM	6823	HW1	SOL	1900	49.410	19.500	21.280	1.00	0.00
ATOM	6824	HW2	SOL	1900	50.370	18.940	22.310	1.00	0.00
ATOM	6825	OW	SOL	1901	19.570	8.590	35.610	1.00	0.00
ATOM	6826	HW1	SOL	1901	18.940	9.260	35.860	1.00	0.00
ATOM	6827	HW2	SOL	1901	19.870	8.220	36.440	1.00	0.00
ATOM	6828	OW	SOL	1902	9.560	7.790	1.450	1.00	0.00
ATOM	6829	HW1	SOL	1902	10.410	7.640	1.030	1.00	0.00
ATOM	6830	HW2	SOL	1902	9.220	6.920	1.620	1.00	0.00
ATOM	6831	OW	SOL	1903	49.860	25.780	5.380	1.00	0.00
ATOM	6832	HW1	SOL	1903	49.900	24.980	4.850	1.00	0.00
ATOM	6833	HW2	SOL	1903	50.290	26.450	4.830	1.00	0.00
ATOM	6834	OW	SOL	1904	49.530	22.330	55.730	1.00	0.00
ATOM	6835	HW1	SOL	1904	49.300	23.250	55.860	1.00	0.00
ATOM	6836	HW2	SOL	1904	48.730	21.930	55.370	1.00	0.00
ATOM	6837	OW	SOL	1905	22.160	8.540	34.960	1.00	0.00
ATOM	6838	HW1	SOL	1905	22.530	7.870	35.540	1.00	0.00
ATOM	6839	HW2	SOL	1905	21.210	8.430	35.060	1.00	0.00
ATOM	6840	OW	SOL	1906	6.100	15.970	11.150	1.00	0.00
ATOM	6841	HW1	SOL	1906	6.750	15.470	10.650	1.00	0.00
ATOM	6842	HW2	SOL	1906	5.530	16.350	10.480	1.00	0.00
ATOM	6843	OW	SOL	1907	35.310	55.490	7.170	1.00	0.00
ATOM	6844	HW1	SOL	1907	36.180	55.770	6.880	1.00	0.00
ATOM	6845	HW2	SOL	1907	34.720	55.850	6.510	1.00	0.00
ATOM	6846	OW	SOL	1908	3.110	45.200	5.120	1.00	0.00
ATOM	6847	HW1	SOL	1908	3.490	45.600	4.330	1.00	0.00
ATOM	6848	HW2	SOL	1908	3.780	44.600	5.430	1.00	0.00
ATOM	6849	OW	SOL	1909	3.040	52.300	55.790	1.00	0.00
ATOM	6850	HW1	SOL	1909	3.980	52.240	55.970	1.00	0.00
ATOM	6851	HW2	SOL	1909	2.650	51.620	56.340	1.00	0.00
ATOM	6852	OW	SOL	1910	41.350	5.800	53.050	1.00	0.00
ATOM	6853	HW1	SOL	1910	41.120	5.810	53.980	1.00	0.00
ATOM	6854	HW2	SOL	1910	42.290	5.650	53.040	1.00	0.00
ATOM	6855	OW	SOL	1911	39.690	39.160	37.210	1.00	0.00
ATOM	6856	HW1	SOL	1911	39.030	38.830	37.820	1.00	0.00
ATOM	6857	HW2	SOL	1911	39.580	38.620	36.430	1.00	0.00
ATOM	6858	OW	SOL	1912	0.220	45.100	49.300	1.00	0.00
ATOM	6859	HW1	SOL	1912	1.140	44.850	49.370	1.00	0.00
ATOM	6860	HW2	SOL	1912	0.160	45.930	49.760	1.00	0.00
ATOM	6861	OW	SOL	1913	12.970	8.220	7.940	1.00	0.00
ATOM	6862	HW1	SOL	1913	12.340	8.040	7.240	1.00	0.00
ATOM	6863	HW2	SOL	1913	13.570	7.480	7.910	1.00	0.00

ATOM	6864	OW	SOL	1914	18.110	43.150	5.090	1.00	0.00
ATOM	6865	HW1	SOL	1914	18.120	43.390	4.170	1.00	0.00
ATOM	6866	HW2	SOL	1914	17.630	42.320	5.110	1.00	0.00
ATOM	6867	OW	SOL	1915	49.170	27.700	27.490	1.00	0.00
ATOM	6868	HW1	SOL	1915	49.530	27.000	28.040	1.00	0.00
ATOM	6869	HW2	SOL	1915	48.260	27.800	27.780	1.00	0.00
ATOM	6870	OW	SOL	1916	41.190	15.290	45.190	1.00	0.00
ATOM	6871	HW1	SOL	1916	41.910	15.630	45.710	1.00	0.00
ATOM	6872	HW2	SOL	1916	41.510	15.320	44.290	1.00	0.00
ATOM	6873	OW	SOL	1917	14.100	26.150	43.870	1.00	0.00
ATOM	6874	HW1	SOL	1917	13.390	26.790	43.910	1.00	0.00
ATOM	6875	HW2	SOL	1917	14.160	25.900	42.950	1.00	0.00
ATOM	6876	OW	SOL	1918	35.160	47.120	50.490	1.00	0.00
ATOM	6877	HW1	SOL	1918	36.040	47.480	50.540	1.00	0.00
ATOM	6878	HW2	SOL	1918	34.580	47.880	50.570	1.00	0.00
ATOM	6879	OW	SOL	1919	0.900	3.060	14.220	1.00	0.00
ATOM	6880	HW1	SOL	1919	1.490	2.310	14.150	1.00	0.00
ATOM	6881	HW2	SOL	1919	1.480	3.820	14.160	1.00	0.00
ATOM	6882	OW	SOL	1920	52.060	20.730	5.770	1.00	0.00
ATOM	6883	HW1	SOL	1920	52.460	19.920	5.440	1.00	0.00
ATOM	6884	HW2	SOL	1920	51.550	21.060	5.030	1.00	0.00
ATOM	6885	OW	SOL	1921	12.270	22.990	13.790	1.00	0.00
ATOM	6886	HW1	SOL	1921	12.050	22.980	14.720	1.00	0.00
ATOM	6887	HW2	SOL	1921	12.440	23.910	13.600	1.00	0.00
ATOM	6888	OW	SOL	1922	0.660	55.240	29.720	1.00	0.00
ATOM	6889	HW1	SOL	1922	1.350	54.920	29.130	1.00	0.00
ATOM	6890	HW2	SOL	1922	0.710	54.660	30.470	1.00	0.00
ATOM	6891	OW	SOL	1923	41.870	13.520	18.170	1.00	0.00
ATOM	6892	HW1	SOL	1923	40.970	13.190	18.230	1.00	0.00
ATOM	6893	HW2	SOL	1923	41.850	14.360	18.630	1.00	0.00
ATOM	6894	OW	SOL	1924	48.080	25.390	52.640	1.00	0.00
ATOM	6895	HW1	SOL	1924	48.210	25.740	51.760	1.00	0.00
ATOM	6896	HW2	SOL	1924	48.960	25.230	52.980	1.00	0.00
ATOM	6897	OW	SOL	1925	17.710	16.520	41.790	1.00	0.00
ATOM	6898	HW1	SOL	1925	18.240	17.050	42.390	1.00	0.00
ATOM	6899	HW2	SOL	1925	17.600	15.690	42.250	1.00	0.00
ATOM	6900	OW	SOL	1926	7.150	37.230	31.600	1.00	0.00
ATOM	6901	HW1	SOL	1926	7.340	38.160	31.720	1.00	0.00
ATOM	6902	HW2	SOL	1926	6.220	37.130	31.800	1.00	0.00
ATOM	6903	OW	SOL	1927	10.020	5.950	39.320	1.00	0.00
ATOM	6904	HW1	SOL	1927	9.140	5.980	38.930	1.00	0.00
ATOM	6905	HW2	SOL	1927	10.180	5.020	39.470	1.00	0.00
ATOM	6906	OW	SOL	1928	43.370	22.080	27.280	1.00	0.00
ATOM	6907	HW1	SOL	1928	43.860	21.830	26.490	1.00	0.00

ATOM	6908	HW2	SOL	1928	42.770	21.350	27.430	1.00	0.00
ATOM	6909	OW	SOL	1929	21.510	43.330	51.660	1.00	0.00
ATOM	6910	HW1	SOL	1929	21.610	42.830	52.480	1.00	0.00
ATOM	6911	HW2	SOL	1929	20.690	43.810	51.770	1.00	0.00
ATOM	6912	OW	SOL	1930	18.540	50.120	39.870	1.00	0.00
ATOM	6913	HW1	SOL	1930	18.400	49.170	39.860	1.00	0.00
ATOM	6914	HW2	SOL	1930	18.550	50.360	38.940	1.00	0.00
ATOM	6915	OW	SOL	1931	35.490	28.740	0.100	1.00	0.00
ATOM	6916	HW1	SOL	1931	35.870	28.600	0.970	1.00	0.00
ATOM	6917	HW2	SOL	1931	35.920	29.540	-0.220	1.00	0.00
ATOM	6918	OW	SOL	1932	10.260	19.600	37.390	1.00	0.00
ATOM	6919	HW1	SOL	1932	9.650	19.800	38.100	1.00	0.00
ATOM	6920	HW2	SOL	1932	10.260	20.390	36.850	1.00	0.00
ATOM	6921	OW	SOL	1933	32.000	10.570	27.060	1.00	0.00
ATOM	6922	HW1	SOL	1933	32.930	10.430	27.220	1.00	0.00
ATOM	6923	HW2	SOL	1933	31.740	11.220	27.720	1.00	0.00
ATOM	6924	OW	SOL	1934	0.300	51.620	54.300	1.00	0.00
ATOM	6925	HW1	SOL	1934	0.460	50.680	54.250	1.00	0.00
ATOM	6926	HW2	SOL	1934	0.270	51.810	55.240	1.00	0.00
ATOM	6927	OW	SOL	1935	20.350	3.570	21.150	1.00	0.00
ATOM	6928	HW1	SOL	1935	20.450	4.330	20.580	1.00	0.00
ATOM	6929	HW2	SOL	1935	21.210	3.150	21.140	1.00	0.00
ATOM	6930	OW	SOL	1936	11.250	4.420	48.730	1.00	0.00
ATOM	6931	HW1	SOL	1936	11.250	4.720	47.830	1.00	0.00
ATOM	6932	HW2	SOL	1936	11.710	5.110	49.220	1.00	0.00
ATOM	6933	OW	SOL	1937	45.530	25.400	5.350	1.00	0.00
ATOM	6934	HW1	SOL	1937	44.900	24.740	5.650	1.00	0.00
ATOM	6935	HW2	SOL	1937	46.250	25.350	5.980	1.00	0.00
ATOM	6936	OW	SOL	1938	16.580	17.600	45.700	1.00	0.00
ATOM	6937	HW1	SOL	1938	16.430	17.490	46.640	1.00	0.00
ATOM	6938	HW2	SOL	1938	15.940	17.010	45.290	1.00	0.00
ATOM	6939	OW	SOL	1939	36.440	32.830	19.180	1.00	0.00
ATOM	6940	HW1	SOL	1939	36.020	32.040	19.530	1.00	0.00
ATOM	6941	HW2	SOL	1939	36.280	33.500	19.840	1.00	0.00
ATOM	6942	OW	SOL	1940	3.730	12.720	50.520	1.00	0.00
ATOM	6943	HW1	SOL	1940	3.230	12.320	51.240	1.00	0.00
ATOM	6944	HW2	SOL	1940	4.360	12.050	50.260	1.00	0.00
ATOM	6945	OW	SOL	1941	8.380	43.380	43.490	1.00	0.00
ATOM	6946	HW1	SOL	1941	8.400	43.570	44.430	1.00	0.00
ATOM	6947	HW2	SOL	1941	9.260	43.040	43.300	1.00	0.00
ATOM	6948	OW	SOL	1942	6.030	42.190	17.950	1.00	0.00
ATOM	6949	HW1	SOL	1942	6.200	43.090	18.230	1.00	0.00
ATOM	6950	HW2	SOL	1942	5.150	42.000	18.290	1.00	0.00
ATOM	6951	OW	SOL	1943	3.820	7.790	18.930	1.00	0.00

ATOM	6952	HW1	SOL	1943	4.140	6.940	18.650	1.00	0.00
ATOM	6953	HW2	SOL	1943	3.780	7.740	19.890	1.00	0.00
ATOM	6954	OW	SOL	1944	22.740	5.480	15.160	1.00	0.00
ATOM	6955	HW1	SOL	1944	23.500	5.370	15.730	1.00	0.00
ATOM	6956	HW2	SOL	1944	22.050	5.780	15.760	1.00	0.00
ATOM	6957	OW	SOL	1945	36.340	55.780	53.370	1.00	0.00
ATOM	6958	HW1	SOL	1945	35.670	56.330	52.950	1.00	0.00
ATOM	6959	HW2	SOL	1945	36.720	55.270	52.650	1.00	0.00
ATOM	6960	OW	SOL	1946	6.710	36.060	55.150	1.00	0.00
ATOM	6961	HW1	SOL	1946	5.830	36.410	54.990	1.00	0.00
ATOM	6962	HW2	SOL	1946	6.630	35.580	55.970	1.00	0.00
ATOM	6963	OW	SOL	1947	39.490	50.500	35.510	1.00	0.00
ATOM	6964	HW1	SOL	1947	38.700	50.250	35.990	1.00	0.00
ATOM	6965	HW2	SOL	1947	39.340	51.400	35.240	1.00	0.00
ATOM	6966	OW	SOL	1948	8.040	16.080	45.360	1.00	0.00
ATOM	6967	HW1	SOL	1948	7.440	16.820	45.290	1.00	0.00
ATOM	6968	HW2	SOL	1948	8.670	16.220	44.660	1.00	0.00
ATOM	6969	OW	SOL	1949	7.280	1.590	49.670	1.00	0.00
ATOM	6970	HW1	SOL	1949	7.850	1.080	49.090	1.00	0.00
ATOM	6971	HW2	SOL	1949	7.500	1.290	50.550	1.00	0.00
ATOM	6972	OW	SOL	1950	17.270	20.050	12.560	1.00	0.00
ATOM	6973	HW1	SOL	1950	16.420	19.610	12.520	1.00	0.00
ATOM	6974	HW2	SOL	1950	17.660	19.870	11.700	1.00	0.00
ATOM	6975	OW	SOL	1951	0.640	54.160	39.320	1.00	0.00
ATOM	6976	HW1	SOL	1951	1.590	54.150	39.420	1.00	0.00
ATOM	6977	HW2	SOL	1951	0.420	53.260	39.070	1.00	0.00
ATOM	6978	OW	SOL	1952	15.500	36.560	50.580	1.00	0.00
ATOM	6979	HW1	SOL	1952	15.150	37.220	51.180	1.00	0.00
ATOM	6980	HW2	SOL	1952	15.600	35.770	51.110	1.00	0.00
ATOM	6981	OW	SOL	1953	19.460	35.840	47.120	1.00	0.00
ATOM	6982	HW1	SOL	1953	20.280	35.390	47.280	1.00	0.00
ATOM	6983	HW2	SOL	1953	19.290	35.710	46.180	1.00	0.00
ATOM	6984	OW	SOL	1954	24.080	5.100	17.340	1.00	0.00
ATOM	6985	HW1	SOL	1954	24.170	5.390	18.250	1.00	0.00
ATOM	6986	HW2	SOL	1954	24.450	4.220	17.320	1.00	0.00
ATOM	6987	OW	SOL	1955	55.290	8.340	50.360	1.00	0.00
ATOM	6988	HW1	SOL	1955	55.120	8.200	49.430	1.00	0.00
ATOM	6989	HW2	SOL	1955	54.430	8.320	50.780	1.00	0.00
ATOM	6990	OW	SOL	1956	7.950	27.000	30.290	1.00	0.00
ATOM	6991	HW1	SOL	1956	8.600	27.320	29.670	1.00	0.00
ATOM	6992	HW2	SOL	1956	7.940	26.050	30.150	1.00	0.00
ATOM	6993	OW	SOL	1957	10.600	54.460	7.710	1.00	0.00
ATOM	6994	HW1	SOL	1957	11.300	53.950	8.110	1.00	0.00
ATOM	6995	HW2	SOL	1957	10.760	55.360	8.020	1.00	0.00

ATOM	6996	OW	SOL	1958	12.470	40.410	27.790	1.00	0.00
ATOM	6997	HW1	SOL	1958	13.160	39.820	28.090	1.00	0.00
ATOM	6998	HW2	SOL	1958	11.710	39.840	27.670	1.00	0.00
ATOM	6999	OW	SOL	1959	31.770	34.180	8.080	1.00	0.00
ATOM	7000	HW1	SOL	1959	31.440	34.580	7.280	1.00	0.00
ATOM	7001	HW2	SOL	1959	32.070	33.310	7.810	1.00	0.00
ATOM	7002	OW	SOL	1960	3.140	44.290	18.280	1.00	0.00
ATOM	7003	HW1	SOL	1960	3.000	43.340	18.230	1.00	0.00
ATOM	7004	HW2	SOL	1960	3.190	44.470	19.210	1.00	0.00
ATOM	7005	OW	SOL	1961	1.940	29.900	12.330	1.00	0.00
ATOM	7006	HW1	SOL	1961	2.210	30.780	12.070	1.00	0.00
ATOM	7007	HW2	SOL	1961	1.070	30.010	12.710	1.00	0.00
ATOM	7008	OW	SOL	1962	32.670	5.120	11.920	1.00	0.00
ATOM	7009	HW1	SOL	1962	33.120	5.240	11.080	1.00	0.00
ATOM	7010	HW2	SOL	1962	33.360	5.260	12.580	1.00	0.00
ATOM	7011	OW	SOL	1963	44.980	32.920	49.680	1.00	0.00
ATOM	7012	HW1	SOL	1963	44.520	33.750	49.560	1.00	0.00
ATOM	7013	HW2	SOL	1963	44.950	32.750	50.620	1.00	0.00
ATOM	7014	OW	SOL	1964	46.110	11.180	41.070	1.00	0.00
ATOM	7015	HW1	SOL	1964	45.550	10.540	41.520	1.00	0.00
ATOM	7016	HW2	SOL	1964	46.610	10.650	40.440	1.00	0.00
ATOM	7017	OW	SOL	1965	22.950	50.730	40.020	1.00	0.00
ATOM	7018	HW1	SOL	1965	22.530	51.550	39.740	1.00	0.00
ATOM	7019	HW2	SOL	1965	23.580	50.540	39.330	1.00	0.00
ATOM	7020	OW	SOL	1966	7.000	9.900	31.100	1.00	0.00
ATOM	7021	HW1	SOL	1966	7.530	10.320	31.780	1.00	0.00
ATOM	7022	HW2	SOL	1966	6.130	9.820	31.500	1.00	0.00
ATOM	7023	OW	SOL	1967	41.270	55.360	50.910	1.00	0.00
ATOM	7024	HW1	SOL	1967	42.130	55.570	51.280	1.00	0.00
ATOM	7025	HW2	SOL	1967	40.680	55.320	51.670	1.00	0.00
ATOM	7026	OW	SOL	1968	47.470	9.640	47.810	1.00	0.00
ATOM	7027	HW1	SOL	1968	48.280	9.140	47.720	1.00	0.00
ATOM	7028	HW2	SOL	1968	47.670	10.500	47.430	1.00	0.00
ATOM	7029	OW	SOL	1969	4.330	29.640	43.440	1.00	0.00
ATOM	7030	HW1	SOL	1969	4.750	29.770	44.290	1.00	0.00
ATOM	7031	HW2	SOL	1969	4.670	30.350	42.890	1.00	0.00
ATOM	7032	OW	SOL	1970	13.930	40.060	2.530	1.00	0.00
ATOM	7033	HW1	SOL	1970	13.050	40.090	2.910	1.00	0.00
ATOM	7034	HW2	SOL	1970	13.790	39.710	1.650	1.00	0.00
ATOM	7035	OW	SOL	1971	41.480	43.950	47.430	1.00	0.00
ATOM	7036	HW1	SOL	1971	40.740	44.400	47.840	1.00	0.00
ATOM	7037	HW2	SOL	1971	41.410	44.160	46.500	1.00	0.00
ATOM	7038	OW	SOL	1972	41.740	1.400	27.950	1.00	0.00
ATOM	7039	HW1	SOL	1972	42.290	2.160	27.750	1.00	0.00

ATOM	7040	HW2	SOL	1972	40.980	1.760	28.410	1.00	0.00
ATOM	7041	OW	SOL	1973	0.950	46.790	45.290	1.00	0.00
ATOM	7042	HW1	SOL	1973	0.640	47.120	44.440	1.00	0.00
ATOM	7043	HW2	SOL	1973	0.920	47.540	45.870	1.00	0.00
ATOM	7044	OW	SOL	1974	5.020	39.940	40.040	1.00	0.00
ATOM	7045	HW1	SOL	1974	4.710	39.620	39.190	1.00	0.00
ATOM	7046	HW2	SOL	1974	4.690	39.310	40.670	1.00	0.00
ATOM	7047	OW	SOL	1975	48.110	42.280	26.250	1.00	0.00
ATOM	7048	HW1	SOL	1975	47.530	42.880	25.780	1.00	0.00
ATOM	7049	HW2	SOL	1975	47.910	41.420	25.900	1.00	0.00
ATOM	7050	OW	SOL	1976	24.800	55.350	6.070	1.00	0.00
ATOM	7051	HW1	SOL	1976	25.520	55.600	5.480	1.00	0.00
ATOM	7052	HW2	SOL	1976	24.010	55.470	5.540	1.00	0.00
ATOM	7053	OW	SOL	1977	48.440	27.830	11.230	1.00	0.00
ATOM	7054	HW1	SOL	1977	48.730	28.390	11.960	1.00	0.00
ATOM	7055	HW2	SOL	1977	48.950	28.150	10.480	1.00	0.00
ATOM	7056	OW	SOL	1978	22.290	3.330	32.710	1.00	0.00
ATOM	7057	HW1	SOL	1978	22.930	3.420	32.010	1.00	0.00
ATOM	7058	HW2	SOL	1978	22.790	3.490	33.510	1.00	0.00
ATOM	7059	OW	SOL	1979	23.690	39.970	54.100	1.00	0.00
ATOM	7060	HW1	SOL	1979	24.060	39.540	53.330	1.00	0.00
ATOM	7061	HW2	SOL	1979	24.300	39.760	54.810	1.00	0.00
ATOM	7062	OW	SOL	1980	50.720	42.790	10.910	1.00	0.00
ATOM	7063	HW1	SOL	1980	50.230	42.290	11.560	1.00	0.00
ATOM	7064	HW2	SOL	1980	51.590	42.910	11.290	1.00	0.00
ATOM	7065	OW	SOL	1981	41.750	53.310	13.110	1.00	0.00
ATOM	7066	HW1	SOL	1981	40.840	53.280	12.800	1.00	0.00
ATOM	7067	HW2	SOL	1981	41.690	53.710	13.980	1.00	0.00
ATOM	7068	OW	SOL	1982	23.440	49.070	12.840	1.00	0.00
ATOM	7069	HW1	SOL	1982	23.210	48.530	13.590	1.00	0.00
ATOM	7070	HW2	SOL	1982	24.290	49.460	13.080	1.00	0.00
ATOM	7071	OW	SOL	1983	37.860	24.090	10.290	1.00	0.00
ATOM	7072	HW1	SOL	1983	38.030	23.730	11.160	1.00	0.00
ATOM	7073	HW2	SOL	1983	38.230	24.970	10.320	1.00	0.00
ATOM	7074	OW	SOL	1984	38.240	33.320	17.370	1.00	0.00
ATOM	7075	HW1	SOL	1984	38.370	32.460	16.980	1.00	0.00
ATOM	7076	HW2	SOL	1984	37.430	33.230	17.880	1.00	0.00
ATOM	7077	OW	SOL	1985	44.710	15.120	40.120	1.00	0.00
ATOM	7078	HW1	SOL	1985	45.530	15.140	40.600	1.00	0.00
ATOM	7079	HW2	SOL	1985	44.630	14.220	39.810	1.00	0.00
ATOM	7080	OW	SOL	1986	44.120	0.840	19.020	1.00	0.00
ATOM	7081	HW1	SOL	1986	44.740	0.350	19.570	1.00	0.00
ATOM	7082	HW2	SOL	1986	43.370	0.990	19.600	1.00	0.00
ATOM	7083	OW	SOL	1987	41.130	32.520	50.180	1.00	0.00

ATOM	7084	HW1	SOL	1987	41.630	32.290	50.970	1.00	0.00
ATOM	7085	HW2	SOL	1987	41.720	33.090	49.680	1.00	0.00
ATOM	7086	OW	SOL	1988	13.490	41.490	51.670	1.00	0.00
ATOM	7087	HW1	SOL	1988	13.620	40.670	51.200	1.00	0.00
ATOM	7088	HW2	SOL	1988	12.600	41.420	52.040	1.00	0.00
ATOM	7089	OW	SOL	1989	51.410	12.020	25.560	1.00	0.00
ATOM	7090	HW1	SOL	1989	52.300	12.320	25.370	1.00	0.00
ATOM	7091	HW2	SOL	1989	50.920	12.830	25.710	1.00	0.00
ATOM	7092	OW	SOL	1990	11.880	16.780	15.140	1.00	0.00
ATOM	7093	HW1	SOL	1990	12.480	16.040	15.000	1.00	0.00
ATOM	7094	HW2	SOL	1990	11.740	17.140	14.260	1.00	0.00
ATOM	7095	OW	SOL	1991	50.900	4.390	18.840	1.00	0.00
ATOM	7096	HW1	SOL	1991	51.110	4.490	17.910	1.00	0.00
ATOM	7097	HW2	SOL	1991	50.920	5.280	19.190	1.00	0.00
ATOM	7098	OW	SOL	1992	39.500	51.940	42.930	1.00	0.00
ATOM	7099	HW1	SOL	1992	39.580	51.700	42.000	1.00	0.00
ATOM	7100	HW2	SOL	1992	40.400	52.060	43.230	1.00	0.00
ATOM	7101	OW	SOL	1993	55.250	38.820	22.210	1.00	0.00
ATOM	7102	HW1	SOL	1993	56.010	38.250	22.100	1.00	0.00
ATOM	7103	HW2	SOL	1993	54.850	38.540	23.040	1.00	0.00
ATOM	7104	OW	SOL	1994	53.850	40.870	27.210	1.00	0.00
ATOM	7105	HW1	SOL	1994	54.130	39.990	26.970	1.00	0.00
ATOM	7106	HW2	SOL	1994	53.720	41.320	26.380	1.00	0.00
ATOM	7107	OW	SOL	1995	41.170	17.640	4.060	1.00	0.00
ATOM	7108	HW1	SOL	1995	40.250	17.390	3.990	1.00	0.00
ATOM	7109	HW2	SOL	1995	41.200	18.250	4.800	1.00	0.00
ATOM	7110	OW	SOL	1996	54.010	40.450	13.520	1.00	0.00
ATOM	7111	HW1	SOL	1996	53.520	41.040	12.950	1.00	0.00
ATOM	7112	HW2	SOL	1996	54.850	40.340	13.080	1.00	0.00
ATOM	7113	OW	SOL	1997	3.860	1.920	44.670	1.00	0.00
ATOM	7114	HW1	SOL	1997	3.810	1.360	45.440	1.00	0.00
ATOM	7115	HW2	SOL	1997	3.040	1.760	44.200	1.00	0.00
ATOM	7116	OW	SOL	1998	36.740	0.800	29.370	1.00	0.00
ATOM	7117	HW1	SOL	1998	35.970	1.380	29.340	1.00	0.00
ATOM	7118	HW2	SOL	1998	37.120	0.950	30.240	1.00	0.00
ATOM	7119	OW	SOL	1999	26.580	4.320	8.560	1.00	0.00
ATOM	7120	HW1	SOL	1999	26.750	5.260	8.630	1.00	0.00
ATOM	7121	HW2	SOL	1999	25.770	4.190	9.070	1.00	0.00
ATOM	7122	OW	SOL	2000	9.560	19.570	29.650	1.00	0.00
ATOM	7123	HW1	SOL	2000	10.190	20.090	30.160	1.00	0.00
ATOM	7124	HW2	SOL	2000	8.720	20.010	29.800	1.00	0.00
ATOM	7125	OW	SOL	2001	3.440	54.830	55.300	1.00	0.00
ATOM	7126	HW1	SOL	2001	3.170	54.880	54.380	1.00	0.00
ATOM	7127	HW2	SOL	2001	3.310	53.920	55.550	1.00	0.00

ATOM	7128	OW	SOL	2002	17.250	32.550	26.250	1.00	0.00
ATOM	7129	HW1	SOL	2002	18.060	33.050	26.330	1.00	0.00
ATOM	7130	HW2	SOL	2002	16.650	32.990	26.860	1.00	0.00
ATOM	7131	OW	SOL	2003	37.300	31.840	10.180	1.00	0.00
ATOM	7132	HW1	SOL	2003	37.910	31.570	10.870	1.00	0.00
ATOM	7133	HW2	SOL	2003	36.770	32.530	10.580	1.00	0.00
ATOM	7134	OW	SOL	2004	10.410	22.660	3.170	1.00	0.00
ATOM	7135	HW1	SOL	2004	9.540	22.260	3.090	1.00	0.00
ATOM	7136	HW2	SOL	2004	10.290	23.560	2.870	1.00	0.00
ATOM	7137	OW	SOL	2005	17.540	9.460	13.720	1.00	0.00
ATOM	7138	HW1	SOL	2005	18.210	9.280	14.380	1.00	0.00
ATOM	7139	HW2	SOL	2005	17.520	10.410	13.640	1.00	0.00
ATOM	7140	OW	SOL	2006	39.750	12.410	6.380	1.00	0.00
ATOM	7141	HW1	SOL	2006	39.990	13.070	5.730	1.00	0.00
ATOM	7142	HW2	SOL	2006	40.490	11.800	6.390	1.00	0.00
ATOM	7143	OW	SOL	2007	23.410	37.350	11.590	1.00	0.00
ATOM	7144	HW1	SOL	2007	22.860	36.690	11.150	1.00	0.00
ATOM	7145	HW2	SOL	2007	23.210	38.170	11.140	1.00	0.00
ATOM	7146	OW	SOL	2008	53.500	12.260	34.820	1.00	0.00
ATOM	7147	HW1	SOL	2008	54.180	11.610	34.960	1.00	0.00
ATOM	7148	HW2	SOL	2008	52.910	11.860	34.180	1.00	0.00
ATOM	7149	OW	SOL	2009	2.680	54.690	49.970	1.00	0.00
ATOM	7150	HW1	SOL	2009	1.910	54.520	49.440	1.00	0.00
ATOM	7151	HW2	SOL	2009	3.040	53.830	50.180	1.00	0.00
ATOM	7152	OW	SOL	2010	20.220	48.860	55.260	1.00	0.00
ATOM	7153	HW1	SOL	2010	21.030	49.020	55.740	1.00	0.00
ATOM	7154	HW2	SOL	2010	20.500	48.650	54.370	1.00	0.00
ATOM	7155	OW	SOL	2011	27.290	43.380	5.460	1.00	0.00
ATOM	7156	HW1	SOL	2011	27.250	43.930	4.680	1.00	0.00
ATOM	7157	HW2	SOL	2011	26.770	42.610	5.240	1.00	0.00
ATOM	7158	OW	SOL	2012	50.940	5.360	53.820	1.00	0.00
ATOM	7159	HW1	SOL	2012	51.360	6.170	54.140	1.00	0.00
ATOM	7160	HW2	SOL	2012	50.070	5.640	53.530	1.00	0.00
ATOM	7161	OW	SOL	2013	11.290	11.920	14.150	1.00	0.00
ATOM	7162	HW1	SOL	2013	10.990	12.380	14.940	1.00	0.00
ATOM	7163	HW2	SOL	2013	10.510	11.480	13.810	1.00	0.00
ATOM	7164	OW	SOL	2014	42.720	6.980	19.760	1.00	0.00
ATOM	7165	HW1	SOL	2014	43.070	7.030	18.870	1.00	0.00
ATOM	7166	HW2	SOL	2014	41.890	6.500	19.660	1.00	0.00
ATOM	7167	OW	SOL	2015	21.570	42.310	9.510	1.00	0.00
ATOM	7168	HW1	SOL	2015	20.800	41.860	9.180	1.00	0.00
ATOM	7169	HW2	SOL	2015	21.460	43.220	9.230	1.00	0.00
ATOM	7170	OW	SOL	2016	10.620	32.660	17.620	1.00	0.00
ATOM	7171	HW1	SOL	2016	11.050	31.920	18.040	1.00	0.00

ATOM	7172	HW2	SOL	2016	11.110	33.430	17.910	1.00	0.00
ATOM	7173	OW	SOL	2017	24.420	25.380	31.950	1.00	0.00
ATOM	7174	HW1	SOL	2017	23.470	25.440	32.050	1.00	0.00
ATOM	7175	HW2	SOL	2017	24.730	26.270	32.110	1.00	0.00
ATOM	7176	OW	SOL	2018	35.380	43.110	6.190	1.00	0.00
ATOM	7177	HW1	SOL	2018	35.080	42.210	6.030	1.00	0.00
ATOM	7178	HW2	SOL	2018	35.910	43.320	5.420	1.00	0.00
ATOM	7179	OW	SOL	2019	15.670	38.940	42.780	1.00	0.00
ATOM	7180	HW1	SOL	2019	16.240	38.890	43.550	1.00	0.00
ATOM	7181	HW2	SOL	2019	16.230	39.320	42.100	1.00	0.00
ATOM	7182	OW	SOL	2020	28.760	11.530	24.270	1.00	0.00
ATOM	7183	HW1	SOL	2020	28.000	11.520	24.850	1.00	0.00
ATOM	7184	HW2	SOL	2020	29.420	12.040	24.760	1.00	0.00
ATOM	7185	OW	SOL	2021	13.640	12.970	18.000	1.00	0.00
ATOM	7186	HW1	SOL	2021	13.230	12.430	18.670	1.00	0.00
ATOM	7187	HW2	SOL	2021	13.950	12.340	17.350	1.00	0.00
ATOM	7188	OW	SOL	2022	46.540	11.120	50.490	1.00	0.00
ATOM	7189	HW1	SOL	2022	47.140	10.980	49.760	1.00	0.00
ATOM	7190	HW2	SOL	2022	46.110	11.950	50.290	1.00	0.00
ATOM	7191	OW	SOL	2023	26.540	31.990	51.140	1.00	0.00
ATOM	7192	HW1	SOL	2023	26.040	31.220	50.860	1.00	0.00
ATOM	7193	HW2	SOL	2023	26.790	31.800	52.040	1.00	0.00
ATOM	7194	OW	SOL	2024	19.380	29.330	8.790	1.00	0.00
ATOM	7195	HW1	SOL	2024	19.770	29.680	7.990	1.00	0.00
ATOM	7196	HW2	SOL	2024	20.020	29.530	9.470	1.00	0.00
ATOM	7197	OW	SOL	2025	41.940	15.370	26.250	1.00	0.00
ATOM	7198	HW1	SOL	2025	42.810	15.730	26.070	1.00	0.00
ATOM	7199	HW2	SOL	2025	41.630	15.050	25.410	1.00	0.00
ATOM	7200	OW	SOL	2026	15.530	30.310	37.340	1.00	0.00
ATOM	7201	HW1	SOL	2026	15.670	29.750	36.570	1.00	0.00
ATOM	7202	HW2	SOL	2026	14.920	30.990	37.030	1.00	0.00
ATOM	7203	OW	SOL	2027	55.320	25.170	52.480	1.00	0.00
ATOM	7204	HW1	SOL	2027	56.050	24.590	52.710	1.00	0.00
ATOM	7205	HW2	SOL	2027	54.590	24.850	53.010	1.00	0.00
ATOM	7206	OW	SOL	2028	22.310	26.140	8.490	1.00	0.00
ATOM	7207	HW1	SOL	2028	22.580	26.690	9.230	1.00	0.00
ATOM	7208	HW2	SOL	2028	22.040	26.770	7.810	1.00	0.00
ATOM	7209	OW	SOL	2029	38.170	29.190	46.060	1.00	0.00
ATOM	7210	HW1	SOL	2029	38.810	29.820	46.400	1.00	0.00
ATOM	7211	HW2	SOL	2029	38.310	28.410	46.580	1.00	0.00
ATOM	7212	OW	SOL	2030	45.550	21.600	8.270	1.00	0.00
ATOM	7213	HW1	SOL	2030	45.490	22.060	9.100	1.00	0.00
ATOM	7214	HW2	SOL	2030	44.700	21.740	7.850	1.00	0.00
ATOM	7215	OW	SOL	2031	9.570	53.670	24.850	1.00	0.00

ATOM	7216	HW1	SOL	2031	8.910	54.370	24.760	1.00	0.00
ATOM	7217	HW2	SOL	2031	9.440	53.340	25.740	1.00	0.00
ATOM	7218	OW	SOL	2032	10.070	2.580	53.240	1.00	0.00
ATOM	7219	HW1	SOL	2032	9.280	2.790	53.730	1.00	0.00
ATOM	7220	HW2	SOL	2032	9.910	2.920	52.360	1.00	0.00
ATOM	7221	OW	SOL	2033	51.910	18.990	41.460	1.00	0.00
ATOM	7222	HW1	SOL	2033	52.170	18.750	42.350	1.00	0.00
ATOM	7223	HW2	SOL	2033	50.970	18.840	41.430	1.00	0.00
ATOM	7224	OW	SOL	2034	37.830	54.000	25.610	1.00	0.00
ATOM	7225	HW1	SOL	2034	37.270	53.520	26.220	1.00	0.00
ATOM	7226	HW2	SOL	2034	37.380	53.920	24.760	1.00	0.00
ATOM	7227	OW	SOL	2035	8.420	36.140	19.410	1.00	0.00
ATOM	7228	HW1	SOL	2035	9.210	36.200	19.960	1.00	0.00
ATOM	7229	HW2	SOL	2035	8.630	35.460	18.770	1.00	0.00
ATOM	7230	OW	SOL	2036	7.350	22.250	16.960	1.00	0.00
ATOM	7231	HW1	SOL	2036	6.660	22.150	16.300	1.00	0.00
ATOM	7232	HW2	SOL	2036	7.190	23.110	17.350	1.00	0.00
ATOM	7233	OW	SOL	2037	14.600	35.800	5.450	1.00	0.00
ATOM	7234	HW1	SOL	2037	15.070	35.390	6.180	1.00	0.00
ATOM	7235	HW2	SOL	2037	14.000	35.110	5.140	1.00	0.00
ATOM	7236	OW	SOL	2038	21.080	21.880	5.680	1.00	0.00
ATOM	7237	HW1	SOL	2038	21.590	21.880	6.480	1.00	0.00
ATOM	7238	HW2	SOL	2038	20.860	20.960	5.530	1.00	0.00
ATOM	7239	OW	SOL	2039	43.040	17.230	12.710	1.00	0.00
ATOM	7240	HW1	SOL	2039	43.720	17.690	13.190	1.00	0.00
ATOM	7241	HW2	SOL	2039	42.840	17.800	11.970	1.00	0.00
ATOM	7242	OW	SOL	2040	31.230	42.930	42.910	1.00	0.00
ATOM	7243	HW1	SOL	2040	31.980	42.360	43.070	1.00	0.00
ATOM	7244	HW2	SOL	2040	31.410	43.720	43.410	1.00	0.00
ATOM	7245	OW	SOL	2041	8.480	38.820	7.770	1.00	0.00
ATOM	7246	HW1	SOL	2041	9.150	38.300	8.230	1.00	0.00
ATOM	7247	HW2	SOL	2041	8.690	39.730	8.010	1.00	0.00
ATOM	7248	OW	SOL	2042	34.070	51.950	31.400	1.00	0.00
ATOM	7249	HW1	SOL	2042	34.030	52.880	31.600	1.00	0.00
ATOM	7250	HW2	SOL	2042	34.760	51.610	31.980	1.00	0.00
ATOM	7251	OW	SOL	2043	54.740	38.360	26.500	1.00	0.00
ATOM	7252	HW1	SOL	2043	54.660	37.760	25.760	1.00	0.00
ATOM	7253	HW2	SOL	2043	55.210	37.860	27.160	1.00	0.00
ATOM	7254	OW	SOL	2044	20.940	26.270	54.190	1.00	0.00
ATOM	7255	HW1	SOL	2044	20.760	26.930	53.520	1.00	0.00
ATOM	7256	HW2	SOL	2044	20.800	25.430	53.740	1.00	0.00
ATOM	7257	OW	SOL	2045	45.880	32.290	1.100	1.00	0.00
ATOM	7258	HW1	SOL	2045	45.210	32.910	0.820	1.00	0.00
ATOM	7259	HW2	SOL	2045	46.690	32.800	1.090	1.00	0.00

ATOM	7260	OW	SOL	2046	1.540	5.250	20.930	1.00	0.00
ATOM	7261	HW1	SOL	2046	1.190	4.360	20.970	1.00	0.00
ATOM	7262	HW2	SOL	2046	2.340	5.210	21.450	1.00	0.00
ATOM	7263	OW	SOL	2047	46.290	49.580	48.860	1.00	0.00
ATOM	7264	HW1	SOL	2047	45.820	50.350	49.180	1.00	0.00
ATOM	7265	HW2	SOL	2047	45.610	48.970	48.590	1.00	0.00
ATOM	7266	OW	SOL	2048	45.960	48.770	54.450	1.00	0.00
ATOM	7267	HW1	SOL	2048	45.940	47.980	54.990	1.00	0.00
ATOM	7268	HW2	SOL	2048	46.050	48.440	53.550	1.00	0.00
ATOM	7269	OW	SOL	2049	21.810	20.810	23.230	1.00	0.00
ATOM	7270	HW1	SOL	2049	21.860	19.910	22.910	1.00	0.00
ATOM	7271	HW2	SOL	2049	22.590	20.910	23.780	1.00	0.00
ATOM	7272	OW	SOL	2050	34.580	27.680	15.850	1.00	0.00
ATOM	7273	HW1	SOL	2050	35.350	27.110	15.840	1.00	0.00
ATOM	7274	HW2	SOL	2050	34.600	28.110	14.990	1.00	0.00
ATOM	7275	OW	SOL	2051	37.690	47.490	3.410	1.00	0.00
ATOM	7276	HW1	SOL	2051	37.110	47.130	4.080	1.00	0.00
ATOM	7277	HW2	SOL	2051	37.930	48.350	3.730	1.00	0.00
ATOM	7278	OW	SOL	2052	48.530	28.040	34.470	1.00	0.00
ATOM	7279	HW1	SOL	2052	47.600	28.020	34.690	1.00	0.00
ATOM	7280	HW2	SOL	2052	48.580	27.590	33.620	1.00	0.00
ATOM	7281	OW	SOL	2053	21.900	21.730	19.410	1.00	0.00
ATOM	7282	HW1	SOL	2053	22.840	21.560	19.360	1.00	0.00
ATOM	7283	HW2	SOL	2053	21.510	20.860	19.550	1.00	0.00
ATOM	7284	OW	SOL	2054	11.620	18.420	0.700	1.00	0.00
ATOM	7285	HW1	SOL	2054	10.790	18.730	1.060	1.00	0.00
ATOM	7286	HW2	SOL	2054	11.390	17.600	0.250	1.00	0.00
ATOM	7287	OW	SOL	2055	50.530	38.080	22.030	1.00	0.00
ATOM	7288	HW1	SOL	2055	49.680	38.110	21.570	1.00	0.00
ATOM	7289	HW2	SOL	2055	50.700	37.150	22.140	1.00	0.00
ATOM	7290	OW	SOL	2056	0.180	30.820	45.340	1.00	0.00
ATOM	7291	HW1	SOL	2056	0.950	31.070	45.850	1.00	0.00
ATOM	7292	HW2	SOL	2056	-0.510	30.690	45.990	1.00	0.00
ATOM	7293	OW	SOL	2057	29.160	25.050	16.330	1.00	0.00
ATOM	7294	HW1	SOL	2057	28.670	25.850	16.510	1.00	0.00
ATOM	7295	HW2	SOL	2057	29.070	24.930	15.380	1.00	0.00
ATOM	7296	OW	SOL	2058	33.450	51.070	49.760	1.00	0.00
ATOM	7297	HW1	SOL	2058	32.710	51.600	50.060	1.00	0.00
ATOM	7298	HW2	SOL	2058	34.180	51.320	50.320	1.00	0.00
ATOM	7299	OW	SOL	2059	10.820	30.370	19.720	1.00	0.00
ATOM	7300	HW1	SOL	2059	10.740	29.580	20.260	1.00	0.00
ATOM	7301	HW2	SOL	2059	9.960	30.790	19.770	1.00	0.00
ATOM	7302	OW	SOL	2060	10.250	50.700	47.680	1.00	0.00
ATOM	7303	HW1	SOL	2060	11.060	50.700	48.190	1.00	0.00

ATOM	7304	HW2	SOL	2060	10.530	50.540	46.780	1.00	0.00
ATOM	7305	OW	SOL	2061	50.240	6.700	1.010	1.00	0.00
ATOM	7306	HW1	SOL	2061	49.500	6.160	1.280	1.00	0.00
ATOM	7307	HW2	SOL	2061	50.710	6.880	1.820	1.00	0.00
ATOM	7308	OW	SOL	2062	16.720	28.770	9.460	1.00	0.00
ATOM	7309	HW1	SOL	2062	17.510	29.130	9.060	1.00	0.00
ATOM	7310	HW2	SOL	2062	16.920	27.840	9.580	1.00	0.00
ATOM	7311	OW	SOL	2063	17.500	4.770	31.670	1.00	0.00
ATOM	7312	HW1	SOL	2063	17.500	4.890	30.720	1.00	0.00
ATOM	7313	HW2	SOL	2063	18.190	5.350	31.980	1.00	0.00
ATOM	7314	OW	SOL	2064	12.660	25.730	51.140	1.00	0.00
ATOM	7315	HW1	SOL	2064	12.960	25.950	52.020	1.00	0.00
ATOM	7316	HW2	SOL	2064	12.360	24.820	51.210	1.00	0.00
ATOM	7317	OW	SOL	2065	25.850	20.610	22.100	1.00	0.00
ATOM	7318	HW1	SOL	2065	25.980	21.550	21.970	1.00	0.00
ATOM	7319	HW2	SOL	2065	26.690	20.220	21.870	1.00	0.00
ATOM	7320	OW	SOL	2066	49.470	10.500	37.710	1.00	0.00
ATOM	7321	HW1	SOL	2066	49.880	10.760	38.540	1.00	0.00
ATOM	7322	HW2	SOL	2066	48.550	10.400	37.930	1.00	0.00
ATOM	7323	OW	SOL	2067	23.400	47.070	24.550	1.00	0.00
ATOM	7324	HW1	SOL	2067	24.080	47.470	24.020	1.00	0.00
ATOM	7325	HW2	SOL	2067	23.750	47.100	25.440	1.00	0.00
ATOM	7326	OW	SOL	2068	12.140	52.660	40.620	1.00	0.00
ATOM	7327	HW1	SOL	2068	11.910	53.500	41.000	1.00	0.00
ATOM	7328	HW2	SOL	2068	13.060	52.530	40.860	1.00	0.00
ATOM	7329	OW	SOL	2069	43.710	51.560	3.480	1.00	0.00
ATOM	7330	HW1	SOL	2069	43.680	52.250	2.810	1.00	0.00
ATOM	7331	HW2	SOL	2069	43.240	51.940	4.220	1.00	0.00
ATOM	7332	OW	SOL	2070	45.760	22.600	14.560	1.00	0.00
ATOM	7333	HW1	SOL	2070	45.400	23.270	13.970	1.00	0.00
ATOM	7334	HW2	SOL	2070	45.090	21.920	14.590	1.00	0.00
ATOM	7335	OW	SOL	2071	54.510	4.970	15.690	1.00	0.00
ATOM	7336	HW1	SOL	2071	53.740	4.470	15.390	1.00	0.00
ATOM	7337	HW2	SOL	2071	55.240	4.600	15.190	1.00	0.00
ATOM	7338	OW	SOL	2072	16.590	27.860	0.940	1.00	0.00
ATOM	7339	HW1	SOL	2072	16.780	27.040	1.400	1.00	0.00
ATOM	7340	HW2	SOL	2072	17.430	28.120	0.560	1.00	0.00
ATOM	7341	OW	SOL	2073	14.750	1.270	23.140	1.00	0.00
ATOM	7342	HW1	SOL	2073	14.510	1.050	22.250	1.00	0.00
ATOM	7343	HW2	SOL	2073	14.190	0.730	23.690	1.00	0.00
ATOM	7344	OW	SOL	2074	27.730	36.470	0.190	1.00	0.00
ATOM	7345	HW1	SOL	2074	28.540	35.970	0.100	1.00	0.00
ATOM	7346	HW2	SOL	2074	27.930	37.320	-0.220	1.00	0.00
ATOM	7347	OW	SOL	2075	39.790	43.600	42.590	1.00	0.00

ATOM	7348	HW1	SOL	2075	39.120	43.970	42.020	1.00	0.00
ATOM	7349	HW2	SOL	2075	39.560	42.680	42.660	1.00	0.00
ATOM	7350	OW	SOL	2076	41.570	48.600	35.380	1.00	0.00
ATOM	7351	HW1	SOL	2076	41.990	49.350	34.970	1.00	0.00
ATOM	7352	HW2	SOL	2076	40.740	48.940	35.710	1.00	0.00
ATOM	7353	OW	SOL	2077	3.270	7.270	47.460	1.00	0.00
ATOM	7354	HW1	SOL	2077	3.630	6.770	46.740	1.00	0.00
ATOM	7355	HW2	SOL	2077	3.360	6.700	48.220	1.00	0.00
ATOM	7356	OW	SOL	2078	27.260	32.520	19.280	1.00	0.00
ATOM	7357	HW1	SOL	2078	26.380	32.720	18.950	1.00	0.00
ATOM	7358	HW2	SOL	2078	27.230	32.790	20.200	1.00	0.00
ATOM	7359	OW	SOL	2079	41.250	4.350	21.350	1.00	0.00
ATOM	7360	HW1	SOL	2079	42.070	3.870	21.520	1.00	0.00
ATOM	7361	HW2	SOL	2079	41.310	5.120	21.910	1.00	0.00
ATOM	7362	OW	SOL	2080	3.420	51.190	19.570	1.00	0.00
ATOM	7363	HW1	SOL	2080	3.190	50.850	20.430	1.00	0.00
ATOM	7364	HW2	SOL	2080	3.670	52.100	19.730	1.00	0.00
ATOM	7365	OW	SOL	2081	34.040	54.990	32.090	1.00	0.00
ATOM	7366	HW1	SOL	2081	34.880	55.010	32.550	1.00	0.00
ATOM	7367	HW2	SOL	2081	33.400	54.770	32.760	1.00	0.00
ATOM	7368	OW	SOL	2082	35.690	16.130	26.360	1.00	0.00
ATOM	7369	HW1	SOL	2082	35.400	16.990	26.670	1.00	0.00
ATOM	7370	HW2	SOL	2082	35.870	15.640	27.160	1.00	0.00
ATOM	7371	OW	SOL	2083	54.000	11.890	27.900	1.00	0.00
ATOM	7372	HW1	SOL	2083	53.830	12.830	28.000	1.00	0.00
ATOM	7373	HW2	SOL	2083	53.450	11.620	27.170	1.00	0.00
ATOM	7374	OW	SOL	2084	43.800	21.400	13.000	1.00	0.00
ATOM	7375	HW1	SOL	2084	43.070	21.460	13.600	1.00	0.00
ATOM	7376	HW2	SOL	2084	43.760	20.500	12.650	1.00	0.00
ATOM	7377	OW	SOL	2085	22.000	1.980	38.700	1.00	0.00
ATOM	7378	HW1	SOL	2085	21.960	1.210	39.270	1.00	0.00
ATOM	7379	HW2	SOL	2085	22.360	1.650	37.880	1.00	0.00
ATOM	7380	OW	SOL	2086	20.360	12.420	26.960	1.00	0.00
ATOM	7381	HW1	SOL	2086	19.680	12.580	27.620	1.00	0.00
ATOM	7382	HW2	SOL	2086	19.880	12.290	26.140	1.00	0.00
ATOM	7383	OW	SOL	2087	23.730	31.220	47.470	1.00	0.00
ATOM	7384	HW1	SOL	2087	23.620	31.620	48.340	1.00	0.00
ATOM	7385	HW2	SOL	2087	22.940	31.460	47.000	1.00	0.00
ATOM	7386	OW	SOL	2088	48.400	55.640	24.930	1.00	0.00
ATOM	7387	HW1	SOL	2088	48.020	55.770	24.060	1.00	0.00
ATOM	7388	HW2	SOL	2088	47.810	55.010	25.350	1.00	0.00
ATOM	7389	OW	SOL	2089	32.660	11.860	21.700	1.00	0.00
ATOM	7390	HW1	SOL	2089	32.400	12.420	20.960	1.00	0.00
ATOM	7391	HW2	SOL	2089	31.960	11.970	22.340	1.00	0.00

ATOM	7392	OW	SOL	2090	23.800	1.090	4.260	1.00	0.00
ATOM	7393	HW1	SOL	2090	23.640	1.580	5.070	1.00	0.00
ATOM	7394	HW2	SOL	2090	23.110	0.430	4.240	1.00	0.00
ATOM	7395	OW	SOL	2091	48.050	40.320	53.820	1.00	0.00
ATOM	7396	HW1	SOL	2091	48.530	40.410	52.990	1.00	0.00
ATOM	7397	HW2	SOL	2091	48.020	39.380	53.980	1.00	0.00
ATOM	7398	OW	SOL	2092	33.210	1.450	30.830	1.00	0.00
ATOM	7399	HW1	SOL	2092	33.240	2.030	31.600	1.00	0.00
ATOM	7400	HW2	SOL	2092	33.610	0.630	31.130	1.00	0.00
ATOM	7401	OW	SOL	2093	3.490	32.510	6.180	1.00	0.00
ATOM	7402	HW1	SOL	2093	2.550	32.390	6.270	1.00	0.00
ATOM	7403	HW2	SOL	2093	3.770	31.790	5.610	1.00	0.00
ATOM	7404	OW	SOL	2094	52.460	21.190	13.930	1.00	0.00
ATOM	7405	HW1	SOL	2094	51.740	21.630	13.470	1.00	0.00
ATOM	7406	HW2	SOL	2094	52.390	21.520	14.830	1.00	0.00
ATOM	7407	OW	SOL	2095	50.610	6.320	50.830	1.00	0.00
ATOM	7408	HW1	SOL	2095	50.680	6.050	49.920	1.00	0.00
ATOM	7409	HW2	SOL	2095	50.910	5.570	51.340	1.00	0.00
ATOM	7410	OW	SOL	2096	48.880	50.530	27.770	1.00	0.00
ATOM	7411	HW1	SOL	2096	49.580	50.210	27.200	1.00	0.00
ATOM	7412	HW2	SOL	2096	48.220	50.880	27.160	1.00	0.00
ATOM	7413	OW	SOL	2097	15.690	25.640	36.140	1.00	0.00
ATOM	7414	HW1	SOL	2097	16.620	25.670	35.910	1.00	0.00
ATOM	7415	HW2	SOL	2097	15.560	24.790	36.550	1.00	0.00
ATOM	7416	OW	SOL	2098	2.090	25.640	19.380	1.00	0.00
ATOM	7417	HW1	SOL	2098	2.120	25.750	20.330	1.00	0.00
ATOM	7418	HW2	SOL	2098	1.330	26.150	19.100	1.00	0.00
ATOM	7419	OW	SOL	2099	25.230	52.900	42.620	1.00	0.00
ATOM	7420	HW1	SOL	2099	25.590	53.140	41.770	1.00	0.00
ATOM	7421	HW2	SOL	2099	24.830	52.040	42.480	1.00	0.00
ATOM	7422	OW	SOL	2100	18.830	18.980	26.380	1.00	0.00
ATOM	7423	HW1	SOL	2100	19.510	19.450	26.860	1.00	0.00
ATOM	7424	HW2	SOL	2100	18.250	19.670	26.060	1.00	0.00
ATOM	7425	OW	SOL	2101	54.510	41.750	40.600	1.00	0.00
ATOM	7426	HW1	SOL	2101	54.900	41.350	39.830	1.00	0.00
ATOM	7427	HW2	SOL	2101	54.710	41.160	41.320	1.00	0.00
ATOM	7428	OW	SOL	2102	26.560	0.750	44.770	1.00	0.00
ATOM	7429	HW1	SOL	2102	27.190	0.050	44.610	1.00	0.00
ATOM	7430	HW2	SOL	2102	26.710	1.370	44.050	1.00	0.00
ATOM	7431	OW	SOL	2103	9.610	32.930	31.480	1.00	0.00
ATOM	7432	HW1	SOL	2103	9.070	32.870	30.690	1.00	0.00
ATOM	7433	HW2	SOL	2103	10.400	33.390	31.200	1.00	0.00
ATOM	7434	OW	SOL	2104	32.990	14.570	15.620	1.00	0.00
ATOM	7435	HW1	SOL	2104	32.870	15.320	16.200	1.00	0.00

ATOM	7436	HW2	SOL	2104	33.780	14.140	15.930	1.00	0.00
ATOM	7437	OW	SOL	2105	3.260	20.820	9.220	1.00	0.00
ATOM	7438	HW1	SOL	2105	2.680	20.060	9.310	1.00	0.00
ATOM	7439	HW2	SOL	2105	2.900	21.470	9.820	1.00	0.00
ATOM	7440	OW	SOL	2106	34.540	47.950	22.670	1.00	0.00
ATOM	7441	HW1	SOL	2106	35.200	47.610	22.060	1.00	0.00
ATOM	7442	HW2	SOL	2106	34.920	47.810	23.530	1.00	0.00
ATOM	7443	OW	SOL	2107	46.660	45.300	35.760	1.00	0.00
ATOM	7444	HW1	SOL	2107	46.690	44.420	35.390	1.00	0.00
ATOM	7445	HW2	SOL	2107	45.870	45.690	35.400	1.00	0.00
ATOM	7446	OW	SOL	2108	52.810	3.630	10.790	1.00	0.00
ATOM	7447	HW1	SOL	2108	53.580	3.090	10.600	1.00	0.00
ATOM	7448	HW2	SOL	2108	52.190	3.400	10.110	1.00	0.00
ATOM	7449	OW	SOL	2109	20.920	19.040	5.190	1.00	0.00
ATOM	7450	HW1	SOL	2109	21.230	18.390	5.830	1.00	0.00
ATOM	7451	HW2	SOL	2109	20.140	18.640	4.800	1.00	0.00
ATOM	7452	OW	SOL	2110	6.310	10.610	46.240	1.00	0.00
ATOM	7453	HW1	SOL	2110	6.800	11.220	46.790	1.00	0.00
ATOM	7454	HW2	SOL	2110	6.930	10.350	45.560	1.00	0.00
ATOM	7455	OW	SOL	2111	46.100	44.570	16.070	1.00	0.00
ATOM	7456	HW1	SOL	2111	46.900	45.100	16.020	1.00	0.00
ATOM	7457	HW2	SOL	2111	45.980	44.410	17.000	1.00	0.00
ATOM	7458	OW	SOL	2112	50.580	18.040	19.320	1.00	0.00
ATOM	7459	HW1	SOL	2112	51.130	17.260	19.430	1.00	0.00
ATOM	7460	HW2	SOL	2112	50.630	18.500	20.160	1.00	0.00
ATOM	7461	OW	SOL	2113	12.410	52.050	16.190	1.00	0.00
ATOM	7462	HW1	SOL	2113	12.560	52.640	16.930	1.00	0.00
ATOM	7463	HW2	SOL	2113	11.500	52.220	15.930	1.00	0.00
ATOM	7464	OW	SOL	2114	36.320	26.980	12.810	1.00	0.00
ATOM	7465	HW1	SOL	2114	35.650	27.660	12.920	1.00	0.00
ATOM	7466	HW2	SOL	2114	36.530	26.710	13.710	1.00	0.00
ATOM	7467	OW	SOL	2115	22.540	48.610	34.690	1.00	0.00
ATOM	7468	HW1	SOL	2115	22.190	48.060	35.390	1.00	0.00
ATOM	7469	HW2	SOL	2115	22.540	48.040	33.920	1.00	0.00
ATOM	7470	OW	SOL	2116	27.370	26.730	20.520	1.00	0.00
ATOM	7471	HW1	SOL	2116	27.380	27.420	19.850	1.00	0.00
ATOM	7472	HW2	SOL	2116	26.450	26.490	20.590	1.00	0.00
ATOM	7473	OW	SOL	2117	15.070	53.700	27.740	1.00	0.00
ATOM	7474	HW1	SOL	2117	14.850	54.200	28.530	1.00	0.00
ATOM	7475	HW2	SOL	2117	15.930	53.330	27.930	1.00	0.00
ATOM	7476	OW	SOL	2118	26.670	8.530	42.320	1.00	0.00
ATOM	7477	HW1	SOL	2118	26.820	9.460	42.140	1.00	0.00
ATOM	7478	HW2	SOL	2118	26.630	8.470	43.270	1.00	0.00
ATOM	7479	OW	SOL	2119	0.120	16.850	5.700	1.00	0.00

ATOM	7480	HW1	SOL	2119	0.620	17.040	6.490	1.00	0.00
ATOM	7481	HW2	SOL	2119	0.550	17.390	5.020	1.00	0.00
ATOM	7482	OW	SOL	2120	27.430	53.990	31.350	1.00	0.00
ATOM	7483	HW1	SOL	2120	27.480	54.310	30.440	1.00	0.00
ATOM	7484	HW2	SOL	2120	26.500	53.890	31.520	1.00	0.00
ATOM	7485	OW	SOL	2121	41.530	10.070	15.700	1.00	0.00
ATOM	7486	HW1	SOL	2121	40.880	10.700	15.380	1.00	0.00
ATOM	7487	HW2	SOL	2121	42.310	10.250	15.180	1.00	0.00
ATOM	7488	OW	SOL	2122	10.300	11.160	6.360	1.00	0.00
ATOM	7489	HW1	SOL	2122	9.650	11.280	5.670	1.00	0.00
ATOM	7490	HW2	SOL	2122	9.860	10.600	7.000	1.00	0.00
ATOM	7491	OW	SOL	2123	47.850	15.570	14.800	1.00	0.00
ATOM	7492	HW1	SOL	2123	47.130	15.740	15.400	1.00	0.00
ATOM	7493	HW2	SOL	2123	47.490	15.720	13.930	1.00	0.00
ATOM	7494	OW	SOL	2124	32.320	25.410	23.050	1.00	0.00
ATOM	7495	HW1	SOL	2124	32.280	24.660	22.450	1.00	0.00
ATOM	7496	HW2	SOL	2124	31.450	25.440	23.440	1.00	0.00
ATOM	7497	OW	SOL	2125	51.970	18.590	13.280	1.00	0.00
ATOM	7498	HW1	SOL	2125	52.080	19.450	13.680	1.00	0.00
ATOM	7499	HW2	SOL	2125	52.150	18.740	12.350	1.00	0.00
ATOM	7500	OW	SOL	2126	55.600	0.300	14.320	1.00	0.00
ATOM	7501	HW1	SOL	2126	55.260	-0.070	15.140	1.00	0.00
ATOM	7502	HW2	SOL	2126	56.560	0.280	14.430	1.00	0.00
ATOM	7503	OW	SOL	2127	50.260	46.760	37.950	1.00	0.00
ATOM	7504	HW1	SOL	2127	49.980	46.800	37.040	1.00	0.00
ATOM	7505	HW2	SOL	2127	50.770	45.950	38.010	1.00	0.00
ATOM	7506	OW	SOL	2128	3.020	8.690	16.080	1.00	0.00
ATOM	7507	HW1	SOL	2128	3.670	8.520	16.760	1.00	0.00
ATOM	7508	HW2	SOL	2128	2.380	7.990	16.170	1.00	0.00
ATOM	7509	OW	SOL	2129	19.710	18.460	11.340	1.00	0.00
ATOM	7510	HW1	SOL	2129	19.660	18.240	12.270	1.00	0.00
ATOM	7511	HW2	SOL	2129	19.660	17.610	10.890	1.00	0.00
ATOM	7512	OW	SOL	2130	29.910	55.050	41.280	1.00	0.00
ATOM	7513	HW1	SOL	2130	29.510	55.070	42.140	1.00	0.00
ATOM	7514	HW2	SOL	2130	30.620	55.690	41.330	1.00	0.00
ATOM	7515	OW	SOL	2131	27.550	50.270	39.380	1.00	0.00
ATOM	7516	HW1	SOL	2131	27.350	49.610	40.050	1.00	0.00
ATOM	7517	HW2	SOL	2131	27.920	50.990	39.870	1.00	0.00
ATOM	7518	OW	SOL	2132	32.270	34.480	42.630	1.00	0.00
ATOM	7519	HW1	SOL	2132	33.080	34.060	42.320	1.00	0.00
ATOM	7520	HW2	SOL	2132	32.470	35.420	42.610	1.00	0.00
ATOM	7521	OW	SOL	2133	7.760	54.240	1.850	1.00	0.00
ATOM	7522	HW1	SOL	2133	8.490	53.680	2.120	1.00	0.00
ATOM	7523	HW2	SOL	2133	7.330	53.750	1.150	1.00	0.00

ATOM	7524	OW	SOL	2134	23.640	47.450	40.450	1.00	0.00
ATOM	7525	HW1	SOL	2134	24.580	47.550	40.560	1.00	0.00
ATOM	7526	HW2	SOL	2134	23.330	48.310	40.170	1.00	0.00
ATOM	7527	OW	SOL	2135	44.680	47.790	28.050	1.00	0.00
ATOM	7528	HW1	SOL	2135	45.530	47.980	28.440	1.00	0.00
ATOM	7529	HW2	SOL	2135	44.380	46.990	28.490	1.00	0.00
ATOM	7530	OW	SOL	2136	13.200	34.670	52.810	1.00	0.00
ATOM	7531	HW1	SOL	2136	12.550	34.070	52.430	1.00	0.00
ATOM	7532	HW2	SOL	2136	13.620	34.150	53.500	1.00	0.00
ATOM	7533	OW	SOL	2137	15.260	30.520	14.630	1.00	0.00
ATOM	7534	HW1	SOL	2137	15.990	30.950	15.090	1.00	0.00
ATOM	7535	HW2	SOL	2137	14.480	30.970	14.940	1.00	0.00
ATOM	7536	OW	SOL	2138	40.530	6.300	9.270	1.00	0.00
ATOM	7537	HW1	SOL	2138	41.460	6.480	9.400	1.00	0.00
ATOM	7538	HW2	SOL	2138	40.480	6.010	8.360	1.00	0.00
ATOM	7539	OW	SOL	2139	14.730	22.000	41.280	1.00	0.00
ATOM	7540	HW1	SOL	2139	15.040	21.840	42.170	1.00	0.00
ATOM	7541	HW2	SOL	2139	13.890	21.540	41.230	1.00	0.00
ATOM	7542	OW	SOL	2140	39.610	3.860	50.530	1.00	0.00
ATOM	7543	HW1	SOL	2140	40.150	3.560	51.260	1.00	0.00
ATOM	7544	HW2	SOL	2140	39.180	4.660	50.850	1.00	0.00
ATOM	7545	OW	SOL	2141	4.300	32.450	28.590	1.00	0.00
ATOM	7546	HW1	SOL	2141	4.280	33.160	29.230	1.00	0.00
ATOM	7547	HW2	SOL	2141	4.870	32.770	27.890	1.00	0.00
ATOM	7548	OW	SOL	2142	40.020	7.870	3.280	1.00	0.00
ATOM	7549	HW1	SOL	2142	40.470	8.070	2.450	1.00	0.00
ATOM	7550	HW2	SOL	2142	40.290	8.580	3.870	1.00	0.00
ATOM	7551	OW	SOL	2143	29.760	31.930	18.360	1.00	0.00
ATOM	7552	HW1	SOL	2143	30.280	31.730	19.140	1.00	0.00
ATOM	7553	HW2	SOL	2143	28.900	32.150	18.690	1.00	0.00
ATOM	7554	OW	SOL	2144	39.240	10.160	28.420	1.00	0.00
ATOM	7555	HW1	SOL	2144	39.560	10.260	27.520	1.00	0.00
ATOM	7556	HW2	SOL	2144	40.030	10.290	28.960	1.00	0.00
ATOM	7557	OW	SOL	2145	10.970	31.110	5.490	1.00	0.00
ATOM	7558	HW1	SOL	2145	10.240	31.600	5.890	1.00	0.00
ATOM	7559	HW2	SOL	2145	10.580	30.280	5.220	1.00	0.00
ATOM	7560	OW	SOL	2146	23.390	6.140	20.120	1.00	0.00
ATOM	7561	HW1	SOL	2146	23.040	6.190	21.010	1.00	0.00
ATOM	7562	HW2	SOL	2146	24.020	6.860	20.070	1.00	0.00
ATOM	7563	OW	SOL	2147	27.910	32.820	11.320	1.00	0.00
ATOM	7564	HW1	SOL	2147	28.060	33.750	11.450	1.00	0.00
ATOM	7565	HW2	SOL	2147	27.960	32.700	10.370	1.00	0.00
ATOM	7566	OW	SOL	2148	49.100	53.350	14.290	1.00	0.00
ATOM	7567	HW1	SOL	2148	49.470	54.060	13.780	1.00	0.00

ATOM	7568	HW2	SOL	2148	49.090	53.670	15.190	1.00	0.00
ATOM	7569	OW	SOL	2149	25.180	46.590	29.600	1.00	0.00
ATOM	7570	HW1	SOL	2149	25.580	46.170	30.370	1.00	0.00
ATOM	7571	HW2	SOL	2149	24.680	47.320	29.950	1.00	0.00
ATOM	7572	OW	SOL	2150	33.840	37.330	13.210	1.00	0.00
ATOM	7573	HW1	SOL	2150	33.770	36.380	13.100	1.00	0.00
ATOM	7574	HW2	SOL	2150	32.950	37.650	13.060	1.00	0.00
ATOM	7575	OW	SOL	2151	10.370	31.560	22.890	1.00	0.00
ATOM	7576	HW1	SOL	2151	10.460	32.440	22.530	1.00	0.00
ATOM	7577	HW2	SOL	2151	10.100	31.030	22.140	1.00	0.00
ATOM	7578	OW	SOL	2152	47.210	40.010	40.610	1.00	0.00
ATOM	7579	HW1	SOL	2152	47.190	40.640	39.890	1.00	0.00
ATOM	7580	HW2	SOL	2152	47.360	40.540	41.390	1.00	0.00
ATOM	7581	OW	SOL	2153	12.010	37.080	16.030	1.00	0.00
ATOM	7582	HW1	SOL	2153	11.570	37.070	15.180	1.00	0.00
ATOM	7583	HW2	SOL	2153	11.500	37.710	16.550	1.00	0.00
ATOM	7584	OW	SOL	2154	32.400	28.860	24.870	1.00	0.00
ATOM	7585	HW1	SOL	2154	32.120	28.930	23.960	1.00	0.00
ATOM	7586	HW2	SOL	2154	31.790	28.230	25.260	1.00	0.00
ATOM	7587	OW	SOL	2155	11.250	47.260	40.050	1.00	0.00
ATOM	7588	HW1	SOL	2155	12.150	47.580	40.100	1.00	0.00
ATOM	7589	HW2	SOL	2155	11.250	46.480	40.610	1.00	0.00
ATOM	7590	OW	SOL	2156	54.810	46.860	21.670	1.00	0.00
ATOM	7591	HW1	SOL	2156	54.120	46.590	21.060	1.00	0.00
ATOM	7592	HW2	SOL	2156	55.170	46.030	21.990	1.00	0.00
ATOM	7593	OW	SOL	2157	24.260	55.260	45.200	1.00	0.00
ATOM	7594	HW1	SOL	2157	25.100	55.540	44.840	1.00	0.00
ATOM	7595	HW2	SOL	2157	24.220	55.670	46.060	1.00	0.00
ATOM	7596	OW	SOL	2158	46.210	51.940	19.640	1.00	0.00
ATOM	7597	HW1	SOL	2158	46.500	51.240	20.210	1.00	0.00
ATOM	7598	HW2	SOL	2158	46.980	52.490	19.520	1.00	0.00
ATOM	7599	OW	SOL	2159	3.950	47.520	8.800	1.00	0.00
ATOM	7600	HW1	SOL	2159	3.090	47.100	8.760	1.00	0.00
ATOM	7601	HW2	SOL	2159	4.400	47.080	9.520	1.00	0.00
ATOM	7602	OW	SOL	2160	18.920	53.670	18.300	1.00	0.00
ATOM	7603	HW1	SOL	2160	19.070	53.270	17.440	1.00	0.00
ATOM	7604	HW2	SOL	2160	19.310	53.040	18.920	1.00	0.00
ATOM	7605	OW	SOL	2161	30.750	10.840	30.260	1.00	0.00
ATOM	7606	HW1	SOL	2161	30.280	11.430	30.850	1.00	0.00
ATOM	7607	HW2	SOL	2161	31.660	11.090	30.350	1.00	0.00
ATOM	7608	OW	SOL	2162	19.020	29.200	55.470	1.00	0.00
ATOM	7609	HW1	SOL	2162	19.540	28.490	55.830	1.00	0.00
ATOM	7610	HW2	SOL	2162	19.020	29.050	54.520	1.00	0.00
ATOM	7611	OW	SOL	2163	43.520	4.540	17.110	1.00	0.00

ATOM	7612	HW1	SOL	2163	43.950	4.080	17.820	1.00	0.00
ATOM	7613	HW2	SOL	2163	43.720	4.030	16.330	1.00	0.00
ATOM	7614	OW	SOL	2164	3.980	26.400	48.550	1.00	0.00
ATOM	7615	HW1	SOL	2164	3.940	25.680	47.920	1.00	0.00
ATOM	7616	HW2	SOL	2164	3.070	26.650	48.670	1.00	0.00
ATOM	7617	OW	SOL	2165	7.000	17.090	0.390	1.00	0.00
ATOM	7618	HW1	SOL	2165	7.440	16.970	-0.450	1.00	0.00
ATOM	7619	HW2	SOL	2165	6.140	16.680	0.260	1.00	0.00
ATOM	7620	OW	SOL	2166	18.490	18.740	53.610	1.00	0.00
ATOM	7621	HW1	SOL	2166	19.230	18.330	53.160	1.00	0.00
ATOM	7622	HW2	SOL	2166	17.790	18.090	53.570	1.00	0.00
ATOM	7623	OW	SOL	2167	27.510	14.320	26.790	1.00	0.00
ATOM	7624	HW1	SOL	2167	27.520	13.610	26.150	1.00	0.00
ATOM	7625	HW2	SOL	2167	26.600	14.610	26.810	1.00	0.00
ATOM	7626	OW	SOL	2168	13.560	55.660	55.780	1.00	0.00
ATOM	7627	HW1	SOL	2168	14.360	56.170	55.890	1.00	0.00
ATOM	7628	HW2	SOL	2168	12.850	56.300	55.830	1.00	0.00
ATOM	7629	OW	SOL	2169	3.120	30.870	19.830	1.00	0.00
ATOM	7630	HW1	SOL	2169	2.420	30.540	19.270	1.00	0.00
ATOM	7631	HW2	SOL	2169	3.820	31.120	19.220	1.00	0.00
ATOM	7632	OW	SOL	2170	34.280	3.590	1.790	1.00	0.00
ATOM	7633	HW1	SOL	2170	34.190	3.980	2.660	1.00	0.00
ATOM	7634	HW2	SOL	2170	34.500	4.320	1.210	1.00	0.00
ATOM	7635	OW	SOL	2171	24.050	20.760	24.580	1.00	0.00
ATOM	7636	HW1	SOL	2171	24.870	20.440	24.210	1.00	0.00
ATOM	7637	HW2	SOL	2171	23.830	20.110	25.260	1.00	0.00
ATOM	7638	OW	SOL	2172	32.550	19.940	38.930	1.00	0.00
ATOM	7639	HW1	SOL	2172	31.650	19.680	39.120	1.00	0.00
ATOM	7640	HW2	SOL	2172	33.040	19.710	39.720	1.00	0.00
ATOM	7641	OW	SOL	2173	40.100	55.090	33.310	1.00	0.00
ATOM	7642	HW1	SOL	2173	40.020	55.290	32.380	1.00	0.00
ATOM	7643	HW2	SOL	2173	40.920	54.610	33.380	1.00	0.00
ATOM	7644	OW	SOL	2174	19.530	13.190	40.830	1.00	0.00
ATOM	7645	HW1	SOL	2174	19.020	13.710	40.220	1.00	0.00
ATOM	7646	HW2	SOL	2174	18.880	12.660	41.300	1.00	0.00
ATOM	7647	OW	SOL	2175	42.480	16.000	54.240	1.00	0.00
ATOM	7648	HW1	SOL	2175	41.890	15.830	53.500	1.00	0.00
ATOM	7649	HW2	SOL	2175	41.940	15.840	55.010	1.00	0.00
ATOM	7650	OW	SOL	2176	26.740	33.250	21.870	1.00	0.00
ATOM	7651	HW1	SOL	2176	26.200	34.010	22.090	1.00	0.00
ATOM	7652	HW2	SOL	2176	26.490	32.590	22.510	1.00	0.00
ATOM	7653	OW	SOL	2177	8.100	24.290	26.510	1.00	0.00
ATOM	7654	HW1	SOL	2177	8.610	24.280	27.320	1.00	0.00
ATOM	7655	HW2	SOL	2177	7.700	23.420	26.470	1.00	0.00

ATOM	7656	OW	SOL	2178	41.270	45.150	24.350	1.00	0.00
ATOM	7657	HW1	SOL	2178	41.880	45.390	25.040	1.00	0.00
ATOM	7658	HW2	SOL	2178	41.590	44.310	24.020	1.00	0.00
ATOM	7659	OW	SOL	2179	50.980	34.020	47.760	1.00	0.00
ATOM	7660	HW1	SOL	2179	50.410	34.700	47.410	1.00	0.00
ATOM	7661	HW2	SOL	2179	51.790	34.100	47.270	1.00	0.00
ATOM	7662	OW	SOL	2180	38.540	23.310	48.750	1.00	0.00
ATOM	7663	HW1	SOL	2180	37.750	23.690	49.140	1.00	0.00
ATOM	7664	HW2	SOL	2180	39.260	23.700	49.230	1.00	0.00
ATOM	7665	OW	SOL	2181	42.630	3.870	4.150	1.00	0.00
ATOM	7666	HW1	SOL	2181	43.010	4.610	4.630	1.00	0.00
ATOM	7667	HW2	SOL	2181	41.710	4.110	4.040	1.00	0.00
ATOM	7668	OW	SOL	2182	32.170	12.110	33.760	1.00	0.00
ATOM	7669	HW1	SOL	2182	32.860	12.120	34.420	1.00	0.00
ATOM	7670	HW2	SOL	2182	32.630	11.990	32.930	1.00	0.00
ATOM	7671	OW	SOL	2183	30.750	2.030	29.490	1.00	0.00
ATOM	7672	HW1	SOL	2183	31.220	2.040	30.320	1.00	0.00
ATOM	7673	HW2	SOL	2183	31.340	1.580	28.880	1.00	0.00
ATOM	7674	OW	SOL	2184	17.890	45.580	33.510	1.00	0.00
ATOM	7675	HW1	SOL	2184	17.300	46.330	33.530	1.00	0.00
ATOM	7676	HW2	SOL	2184	17.420	44.900	34.000	1.00	0.00
ATOM	7677	OW	SOL	2185	17.150	40.630	41.060	1.00	0.00
ATOM	7678	HW1	SOL	2185	17.460	41.480	41.380	1.00	0.00
ATOM	7679	HW2	SOL	2185	17.380	40.630	40.130	1.00	0.00
ATOM	7680	OW	SOL	2186	8.710	21.270	39.130	1.00	0.00
ATOM	7681	HW1	SOL	2186	9.230	21.680	39.820	1.00	0.00
ATOM	7682	HW2	SOL	2186	8.110	21.960	38.840	1.00	0.00
ATOM	7683	OW	SOL	2187	10.230	43.550	26.850	1.00	0.00
ATOM	7684	HW1	SOL	2187	11.000	42.990	26.960	1.00	0.00
ATOM	7685	HW2	SOL	2187	10.580	44.380	26.520	1.00	0.00
ATOM	7686	OW	SOL	2188	5.260	2.750	17.230	1.00	0.00
ATOM	7687	HW1	SOL	2188	4.940	2.980	16.360	1.00	0.00
ATOM	7688	HW2	SOL	2188	6.190	2.950	17.220	1.00	0.00
ATOM	7689	OW	SOL	2189	11.440	30.480	54.430	1.00	0.00
ATOM	7690	HW1	SOL	2189	11.520	31.150	53.750	1.00	0.00
ATOM	7691	HW2	SOL	2189	10.720	29.930	54.140	1.00	0.00
ATOM	7692	OW	SOL	2190	37.230	51.160	0.630	1.00	0.00
ATOM	7693	HW1	SOL	2190	37.310	50.970	1.570	1.00	0.00
ATOM	7694	HW2	SOL	2190	36.650	51.920	0.590	1.00	0.00
ATOM	7695	OW	SOL	2191	26.170	30.750	16.800	1.00	0.00
ATOM	7696	HW1	SOL	2191	26.640	30.060	17.270	1.00	0.00
ATOM	7697	HW2	SOL	2191	25.600	30.270	16.200	1.00	0.00
ATOM	7698	OW	SOL	2192	23.210	7.400	13.440	1.00	0.00
ATOM	7699	HW1	SOL	2192	22.940	6.870	14.190	1.00	0.00

ATOM	7700	HW2	SOL	2192	23.890	6.890	13.000	1.00	0.00
ATOM	7701	OW	SOL	2193	41.930	47.320	15.260	1.00	0.00
ATOM	7702	HW1	SOL	2193	42.240	46.980	14.430	1.00	0.00
ATOM	7703	HW2	SOL	2193	41.350	48.050	15.020	1.00	0.00
ATOM	7704	OW	SOL	2194	35.930	14.190	1.800	1.00	0.00
ATOM	7705	HW1	SOL	2194	35.180	13.600	1.820	1.00	0.00
ATOM	7706	HW2	SOL	2194	35.740	14.850	2.460	1.00	0.00
ATOM	7707	OW	SOL	2195	30.670	52.200	50.680	1.00	0.00
ATOM	7708	HW1	SOL	2195	30.310	51.390	51.020	1.00	0.00
ATOM	7709	HW2	SOL	2195	30.610	52.110	49.730	1.00	0.00
ATOM	7710	OW	SOL	2196	29.060	10.140	18.870	1.00	0.00
ATOM	7711	HW1	SOL	2196	28.520	10.230	19.650	1.00	0.00
ATOM	7712	HW2	SOL	2196	29.530	9.320	19.010	1.00	0.00
ATOM	7713	OW	SOL	2197	15.940	7.700	29.770	1.00	0.00
ATOM	7714	HW1	SOL	2197	16.150	6.910	30.270	1.00	0.00
ATOM	7715	HW2	SOL	2197	16.320	7.540	28.910	1.00	0.00
ATOM	7716	OW	SOL	2198	47.500	38.720	13.520	1.00	0.00
ATOM	7717	HW1	SOL	2198	47.650	38.400	14.410	1.00	0.00
ATOM	7718	HW2	SOL	2198	47.230	37.950	13.020	1.00	0.00
ATOM	7719	OW	SOL	2199	29.750	1.650	17.300	1.00	0.00
ATOM	7720	HW1	SOL	2199	30.510	1.420	17.820	1.00	0.00
ATOM	7721	HW2	SOL	2199	30.080	2.300	16.670	1.00	0.00
ATOM	7722	OW	SOL	2200	49.420	5.650	6.010	1.00	0.00
ATOM	7723	HW1	SOL	2200	49.950	6.030	6.710	1.00	0.00
ATOM	7724	HW2	SOL	2200	49.550	6.240	5.270	1.00	0.00
ATOM	7725	OW	SOL	2201	31.110	54.000	4.290	1.00	0.00
ATOM	7726	HW1	SOL	2201	30.710	54.360	3.500	1.00	0.00
ATOM	7727	HW2	SOL	2201	30.500	54.240	5.000	1.00	0.00
ATOM	7728	OW	SOL	2202	12.710	41.650	21.920	1.00	0.00
ATOM	7729	HW1	SOL	2202	11.780	41.550	21.740	1.00	0.00
ATOM	7730	HW2	SOL	2202	12.830	41.330	22.810	1.00	0.00
ATOM	7731	OW	SOL	2203	34.270	44.230	18.840	1.00	0.00
ATOM	7732	HW1	SOL	2203	33.550	44.260	18.200	1.00	0.00
ATOM	7733	HW2	SOL	2203	35.050	44.080	18.310	1.00	0.00
ATOM	7734	OW	SOL	2204	30.110	30.070	52.270	1.00	0.00
ATOM	7735	HW1	SOL	2204	31.030	30.260	52.480	1.00	0.00
ATOM	7736	HW2	SOL	2204	29.660	30.140	53.110	1.00	0.00
ATOM	7737	OW	SOL	2205	17.860	31.660	10.280	1.00	0.00
ATOM	7738	HW1	SOL	2205	17.640	31.280	11.130	1.00	0.00
ATOM	7739	HW2	SOL	2205	18.720	31.300	10.070	1.00	0.00
ATOM	7740	OW	SOL	2206	47.770	53.140	40.720	1.00	0.00
ATOM	7741	HW1	SOL	2206	48.320	52.400	40.460	1.00	0.00
ATOM	7742	HW2	SOL	2206	47.830	53.750	39.980	1.00	0.00
ATOM	7743	OW	SOL	2207	19.550	29.190	13.620	1.00	0.00

ATOM	7744	HW1	SOL	2207	19.230	28.990	14.500	1.00	0.00
ATOM	7745	HW2	SOL	2207	18.760	29.400	13.120	1.00	0.00
ATOM	7746	OW	SOL	2208	42.770	18.530	49.380	1.00	0.00
ATOM	7747	HW1	SOL	2208	41.990	19.050	49.570	1.00	0.00
ATOM	7748	HW2	SOL	2208	43.390	18.750	50.070	1.00	0.00
ATOM	7749	OW	SOL	2209	25.780	27.500	34.590	1.00	0.00
ATOM	7750	HW1	SOL	2209	25.610	27.420	35.530	1.00	0.00
ATOM	7751	HW2	SOL	2209	26.250	26.700	34.360	1.00	0.00
ATOM	7752	OW	SOL	2210	5.300	19.460	10.770	1.00	0.00
ATOM	7753	HW1	SOL	2210	4.720	19.860	10.130	1.00	0.00
ATOM	7754	HW2	SOL	2210	5.530	18.610	10.400	1.00	0.00
ATOM	7755	OW	SOL	2211	8.410	21.520	48.120	1.00	0.00
ATOM	7756	HW1	SOL	2211	7.810	22.270	48.150	1.00	0.00
ATOM	7757	HW2	SOL	2211	8.100	20.940	48.820	1.00	0.00
ATOM	7758	OW	SOL	2212	39.630	27.130	20.790	1.00	0.00
ATOM	7759	HW1	SOL	2212	38.940	27.200	21.450	1.00	0.00
ATOM	7760	HW2	SOL	2212	40.420	26.940	21.290	1.00	0.00
ATOM	7761	OW	SOL	2213	23.720	26.520	22.830	1.00	0.00
ATOM	7762	HW1	SOL	2213	24.510	25.990	22.860	1.00	0.00
ATOM	7763	HW2	SOL	2213	23.130	26.040	22.250	1.00	0.00
ATOM	7764	OW	SOL	2214	31.000	2.790	0.890	1.00	0.00
ATOM	7765	HW1	SOL	2214	30.730	3.360	1.610	1.00	0.00
ATOM	7766	HW2	SOL	2214	31.010	1.910	1.260	1.00	0.00
ATOM	7767	OW	SOL	2215	20.080	13.600	49.940	1.00	0.00
ATOM	7768	HW1	SOL	2215	20.660	14.240	50.340	1.00	0.00
ATOM	7769	HW2	SOL	2215	19.970	12.920	50.610	1.00	0.00
ATOM	7770	OW	SOL	2216	37.300	19.150	49.390	1.00	0.00
ATOM	7771	HW1	SOL	2216	37.990	19.510	49.960	1.00	0.00
ATOM	7772	HW2	SOL	2216	37.560	19.400	48.510	1.00	0.00
ATOM	7773	OW	SOL	2217	47.360	2.300	46.220	1.00	0.00
ATOM	7774	HW1	SOL	2217	48.050	2.950	46.060	1.00	0.00
ATOM	7775	HW2	SOL	2217	46.750	2.750	46.800	1.00	0.00
ATOM	7776	OW	SOL	2218	54.800	6.580	35.800	1.00	0.00
ATOM	7777	HW1	SOL	2218	54.310	6.670	34.980	1.00	0.00
ATOM	7778	HW2	SOL	2218	54.120	6.470	36.470	1.00	0.00
ATOM	7779	OW	SOL	2219	30.410	7.740	18.190	1.00	0.00
ATOM	7780	HW1	SOL	2219	30.570	8.450	17.570	1.00	0.00
ATOM	7781	HW2	SOL	2219	31.100	7.110	18.010	1.00	0.00
ATOM	7782	OW	SOL	2220	43.890	49.020	2.170	1.00	0.00
ATOM	7783	HW1	SOL	2220	43.830	49.820	2.690	1.00	0.00
ATOM	7784	HW2	SOL	2220	43.230	49.140	1.480	1.00	0.00
ATOM	7785	OW	SOL	2221	4.420	14.980	41.530	1.00	0.00
ATOM	7786	HW1	SOL	2221	5.190	15.510	41.320	1.00	0.00
ATOM	7787	HW2	SOL	2221	4.460	14.850	42.480	1.00	0.00

ATOM	7788	OW	SOL	2222	9.940	25.320	2.370	1.00	0.00
ATOM	7789	HW1	SOL	2222	10.020	26.220	2.710	1.00	0.00
ATOM	7790	HW2	SOL	2222	9.090	25.300	1.940	1.00	0.00
ATOM	7791	OW	SOL	2223	46.210	15.200	20.920	1.00	0.00
ATOM	7792	HW1	SOL	2223	45.540	14.620	20.560	1.00	0.00
ATOM	7793	HW2	SOL	2223	46.990	14.640	20.980	1.00	0.00
ATOM	7794	OW	SOL	2224	4.690	39.070	37.390	1.00	0.00
ATOM	7795	HW1	SOL	2224	5.630	39.190	37.340	1.00	0.00
ATOM	7796	HW2	SOL	2224	4.330	39.710	36.760	1.00	0.00
ATOM	7797	OW	SOL	2225	51.940	10.210	7.010	1.00	0.00
ATOM	7798	HW1	SOL	2225	51.840	11.160	7.010	1.00	0.00
ATOM	7799	HW2	SOL	2225	51.180	9.880	6.540	1.00	0.00
ATOM	7800	OW	SOL	2226	31.340	37.160	25.420	1.00	0.00
ATOM	7801	HW1	SOL	2226	30.500	36.780	25.680	1.00	0.00
ATOM	7802	HW2	SOL	2226	31.640	36.620	24.690	1.00	0.00
ATOM	7803	OW	SOL	2227	0.610	50.620	28.470	1.00	0.00
ATOM	7804	HW1	SOL	2227	0.550	49.880	29.070	1.00	0.00
ATOM	7805	HW2	SOL	2227	-0.220	51.090	28.580	1.00	0.00
ATOM	7806	OW	SOL	2228	12.940	8.700	25.690	1.00	0.00
ATOM	7807	HW1	SOL	2228	13.880	8.870	25.570	1.00	0.00
ATOM	7808	HW2	SOL	2228	12.750	7.970	25.100	1.00	0.00
ATOM	7809	OW	SOL	2229	50.840	53.000	37.380	1.00	0.00
ATOM	7810	HW1	SOL	2229	51.120	52.170	37.770	1.00	0.00
ATOM	7811	HW2	SOL	2229	50.590	52.760	36.480	1.00	0.00
ATOM	7812	OW	SOL	2230	21.770	53.260	38.420	1.00	0.00
ATOM	7813	HW1	SOL	2230	22.180	53.270	37.550	1.00	0.00
ATOM	7814	HW2	SOL	2230	21.910	54.140	38.760	1.00	0.00
ATOM	7815	OW	SOL	2231	47.370	42.240	0.440	1.00	0.00
ATOM	7816	HW1	SOL	2231	47.560	41.910	-0.440	1.00	0.00
ATOM	7817	HW2	SOL	2231	47.740	43.130	0.440	1.00	0.00
ATOM	7818	OW	SOL	2232	19.280	38.650	27.310	1.00	0.00
ATOM	7819	HW1	SOL	2232	19.380	39.010	26.430	1.00	0.00
ATOM	7820	HW2	SOL	2232	20.160	38.680	27.680	1.00	0.00
ATOM	7821	OW	SOL	2233	20.630	30.120	11.310	1.00	0.00
ATOM	7822	HW1	SOL	2233	21.030	30.980	11.410	1.00	0.00
ATOM	7823	HW2	SOL	2233	20.460	29.820	12.200	1.00	0.00
ATOM	7824	OW	SOL	2234	6.450	24.320	29.830	1.00	0.00
ATOM	7825	HW1	SOL	2234	6.790	24.470	28.950	1.00	0.00
ATOM	7826	HW2	SOL	2234	5.510	24.180	29.700	1.00	0.00
ATOM	7827	OW	SOL	2235	48.180	15.060	11.230	1.00	0.00
ATOM	7828	HW1	SOL	2235	47.330	15.370	11.530	1.00	0.00
ATOM	7829	HW2	SOL	2235	48.810	15.680	11.610	1.00	0.00
ATOM	7830	OW	SOL	2236	22.330	14.040	23.730	1.00	0.00
ATOM	7831	HW1	SOL	2236	21.510	14.410	23.430	1.00	0.00

ATOM	7832	HW2	SOL	2236	23.000	14.510	23.230	1.00	0.00
ATOM	7833	OW	SOL	2237	50.330	23.980	3.300	1.00	0.00
ATOM	7834	HW1	SOL	2237	50.130	24.350	2.440	1.00	0.00
ATOM	7835	HW2	SOL	2237	49.720	23.240	3.390	1.00	0.00
ATOM	7836	OW	SOL	2238	35.470	38.830	20.110	1.00	0.00
ATOM	7837	HW1	SOL	2238	35.740	39.730	19.920	1.00	0.00
ATOM	7838	HW2	SOL	2238	35.210	38.480	19.260	1.00	0.00
ATOM	7839	OW	SOL	2239	28.420	20.930	19.030	1.00	0.00
ATOM	7840	HW1	SOL	2239	28.010	20.510	18.270	1.00	0.00
ATOM	7841	HW2	SOL	2239	29.050	21.540	18.660	1.00	0.00
ATOM	7842	OW	SOL	2240	18.080	55.170	15.610	1.00	0.00
ATOM	7843	HW1	SOL	2240	18.420	54.320	15.880	1.00	0.00
ATOM	7844	HW2	SOL	2240	18.840	55.740	15.580	1.00	0.00
ATOM	7845	OW	SOL	2241	6.160	7.570	47.040	1.00	0.00
ATOM	7846	HW1	SOL	2241	6.210	8.520	46.910	1.00	0.00
ATOM	7847	HW2	SOL	2241	6.460	7.440	47.940	1.00	0.00
ATOM	7848	OW	SOL	2242	33.380	40.780	52.000	1.00	0.00
ATOM	7849	HW1	SOL	2242	33.540	41.570	51.480	1.00	0.00
ATOM	7850	HW2	SOL	2242	33.650	41.010	52.890	1.00	0.00
ATOM	7851	OW	SOL	2243	52.220	2.470	49.430	1.00	0.00
ATOM	7852	HW1	SOL	2243	51.480	2.300	48.840	1.00	0.00
ATOM	7853	HW2	SOL	2243	52.730	3.140	48.990	1.00	0.00
ATOM	7854	OW	SOL	2244	1.020	1.630	50.180	1.00	0.00
ATOM	7855	HW1	SOL	2244	1.050	0.680	50.300	1.00	0.00
ATOM	7856	HW2	SOL	2244	0.460	1.760	49.410	1.00	0.00
ATOM	7857	OW	SOL	2245	42.280	41.770	26.370	1.00	0.00
ATOM	7858	HW1	SOL	2245	42.390	40.950	26.840	1.00	0.00
ATOM	7859	HW2	SOL	2245	41.380	41.750	26.050	1.00	0.00
ATOM	7860	OW	SOL	2246	44.040	53.900	30.390	1.00	0.00
ATOM	7861	HW1	SOL	2246	43.960	54.850	30.430	1.00	0.00
ATOM	7862	HW2	SOL	2246	43.580	53.650	29.600	1.00	0.00
ATOM	7863	OW	SOL	2247	32.230	27.680	52.690	1.00	0.00
ATOM	7864	HW1	SOL	2247	32.120	28.550	52.320	1.00	0.00
ATOM	7865	HW2	SOL	2247	32.700	27.820	53.510	1.00	0.00
ATOM	7866	OW	SOL	2248	22.500	51.360	2.010	1.00	0.00
ATOM	7867	HW1	SOL	2248	22.320	50.490	1.640	1.00	0.00
ATOM	7868	HW2	SOL	2248	23.400	51.540	1.760	1.00	0.00
ATOM	7869	OW	SOL	2249	41.740	10.780	44.610	1.00	0.00
ATOM	7870	HW1	SOL	2249	42.490	11.060	45.130	1.00	0.00
ATOM	7871	HW2	SOL	2249	41.200	10.280	45.220	1.00	0.00
ATOM	7872	OW	SOL	2250	15.130	10.750	31.910	1.00	0.00
ATOM	7873	HW1	SOL	2250	15.640	10.150	32.460	1.00	0.00
ATOM	7874	HW2	SOL	2250	14.740	10.180	31.240	1.00	0.00
ATOM	7875	OW	SOL	2251	19.840	52.590	36.350	1.00	0.00

ATOM	7876	HW1	SOL	2251	20.310	52.800	37.160	1.00	0.00
ATOM	7877	HW2	SOL	2251	19.940	53.370	35.810	1.00	0.00
ATOM	7878	OW	SOL	2252	4.700	0.270	30.430	1.00	0.00
ATOM	7879	HW1	SOL	2252	5.060	-0.620	30.400	1.00	0.00
ATOM	7880	HW2	SOL	2252	5.380	0.820	30.050	1.00	0.00
ATOM	7881	OW	SOL	2253	14.110	54.140	13.400	1.00	0.00
ATOM	7882	HW1	SOL	2253	13.600	54.830	12.980	1.00	0.00
ATOM	7883	HW2	SOL	2253	14.220	53.470	12.730	1.00	0.00
ATOM	7884	OW	SOL	2254	2.740	18.300	9.920	1.00	0.00
ATOM	7885	HW1	SOL	2254	3.540	17.950	9.540	1.00	0.00
ATOM	7886	HW2	SOL	2254	2.770	18.040	10.840	1.00	0.00
ATOM	7887	OW	SOL	2255	5.340	27.770	29.010	1.00	0.00
ATOM	7888	HW1	SOL	2255	6.070	27.590	29.610	1.00	0.00
ATOM	7889	HW2	SOL	2255	4.720	28.280	29.530	1.00	0.00
ATOM	7890	OW	SOL	2256	32.300	48.830	19.780	1.00	0.00
ATOM	7891	HW1	SOL	2256	32.320	48.910	20.730	1.00	0.00
ATOM	7892	HW2	SOL	2256	32.120	47.910	19.620	1.00	0.00
ATOM	7893	OW	SOL	2257	23.160	19.600	17.170	1.00	0.00
ATOM	7894	HW1	SOL	2257	23.770	20.110	17.710	1.00	0.00
ATOM	7895	HW2	SOL	2257	23.580	19.570	16.310	1.00	0.00
ATOM	7896	OW	SOL	2258	11.340	18.060	6.360	1.00	0.00
ATOM	7897	HW1	SOL	2258	11.050	17.210	6.710	1.00	0.00
ATOM	7898	HW2	SOL	2258	10.540	18.590	6.340	1.00	0.00
ATOM	7899	OW	SOL	2259	40.390	47.530	44.230	1.00	0.00
ATOM	7900	HW1	SOL	2259	39.590	47.630	43.720	1.00	0.00
ATOM	7901	HW2	SOL	2259	40.510	48.380	44.650	1.00	0.00
ATOM	7902	OW	SOL	2260	17.350	23.000	12.680	1.00	0.00
ATOM	7903	HW1	SOL	2260	17.200	22.060	12.620	1.00	0.00
ATOM	7904	HW2	SOL	2260	16.890	23.370	11.930	1.00	0.00
ATOM	7905	OW	SOL	2261	24.220	12.410	0.540	1.00	0.00
ATOM	7906	HW1	SOL	2261	23.990	13.320	0.720	1.00	0.00
ATOM	7907	HW2	SOL	2261	24.560	12.080	1.370	1.00	0.00
ATOM	7908	OW	SOL	2262	19.010	19.020	31.990	1.00	0.00
ATOM	7909	HW1	SOL	2262	19.680	18.750	32.610	1.00	0.00
ATOM	7910	HW2	SOL	2262	19.290	18.660	31.150	1.00	0.00
ATOM	7911	OW	SOL	2263	41.730	23.440	44.480	1.00	0.00
ATOM	7912	HW1	SOL	2263	42.520	23.070	44.090	1.00	0.00
ATOM	7913	HW2	SOL	2263	41.630	22.970	45.300	1.00	0.00
ATOM	7914	OW	SOL	2264	2.610	18.230	44.080	1.00	0.00
ATOM	7915	HW1	SOL	2264	2.150	19.040	43.850	1.00	0.00
ATOM	7916	HW2	SOL	2264	3.540	18.470	44.050	1.00	0.00
ATOM	7917	OW	SOL	2265	15.710	29.960	53.020	1.00	0.00
ATOM	7918	HW1	SOL	2265	15.990	30.810	52.670	1.00	0.00
ATOM	7919	HW2	SOL	2265	15.050	29.660	52.400	1.00	0.00

ATOM	7920	OW	SOL	2266	20.440	34.570	7.350	1.00	0.00
ATOM	7921	HW1	SOL	2266	20.950	33.820	7.670	1.00	0.00
ATOM	7922	HW2	SOL	2266	21.080	35.280	7.290	1.00	0.00
ATOM	7923	OW	SOL	2267	23.630	44.940	51.500	1.00	0.00
ATOM	7924	HW1	SOL	2267	22.770	44.510	51.520	1.00	0.00
ATOM	7925	HW2	SOL	2267	23.420	45.860	51.320	1.00	0.00
ATOM	7926	OW	SOL	2268	28.980	17.490	33.300	1.00	0.00
ATOM	7927	HW1	SOL	2268	29.870	17.220	33.510	1.00	0.00
ATOM	7928	HW2	SOL	2268	28.870	17.270	32.370	1.00	0.00
ATOM	7929	OW	SOL	2269	22.300	0.400	29.010	1.00	0.00
ATOM	7930	HW1	SOL	2269	21.790	-0.140	28.410	1.00	0.00
ATOM	7931	HW2	SOL	2269	22.540	-0.190	29.730	1.00	0.00
ATOM	7932	OW	SOL	2270	11.140	9.610	31.730	1.00	0.00
ATOM	7933	HW1	SOL	2270	11.010	10.380	31.180	1.00	0.00
ATOM	7934	HW2	SOL	2270	11.980	9.250	31.440	1.00	0.00
ATOM	7935	OW	SOL	2271	5.970	15.130	38.570	1.00	0.00
ATOM	7936	HW1	SOL	2271	6.740	15.700	38.640	1.00	0.00
ATOM	7937	HW2	SOL	2271	5.810	14.840	39.470	1.00	0.00
ATOM	7938	OW	SOL	2272	53.160	54.290	31.710	1.00	0.00
ATOM	7939	HW1	SOL	2272	52.670	54.220	30.890	1.00	0.00
ATOM	7940	HW2	SOL	2272	53.730	53.530	31.710	1.00	0.00
ATOM	7941	OW	SOL	2273	1.500	22.130	18.680	1.00	0.00
ATOM	7942	HW1	SOL	2273	0.930	22.450	19.370	1.00	0.00
ATOM	7943	HW2	SOL	2273	0.950	21.570	18.140	1.00	0.00
ATOM	7944	OW	SOL	2274	4.120	43.450	39.950	1.00	0.00
ATOM	7945	HW1	SOL	2274	4.250	44.330	39.610	1.00	0.00
ATOM	7946	HW2	SOL	2274	4.730	43.380	40.680	1.00	0.00
ATOM	7947	OW	SOL	2275	36.350	22.850	34.720	1.00	0.00
ATOM	7948	HW1	SOL	2275	36.400	22.000	34.270	1.00	0.00
ATOM	7949	HW2	SOL	2275	36.100	23.470	34.040	1.00	0.00
ATOM	7950	OW	SOL	2276	6.310	15.910	52.480	1.00	0.00
ATOM	7951	HW1	SOL	2276	6.480	15.400	51.700	1.00	0.00
ATOM	7952	HW2	SOL	2276	5.720	15.370	53.000	1.00	0.00
ATOM	7953	OW	SOL	2277	49.240	37.970	7.570	1.00	0.00
ATOM	7954	HW1	SOL	2277	48.320	38.190	7.730	1.00	0.00
ATOM	7955	HW2	SOL	2277	49.270	37.680	6.660	1.00	0.00
ATOM	7956	OW	SOL	2278	33.970	11.520	31.410	1.00	0.00
ATOM	7957	HW1	SOL	2278	33.620	12.190	30.820	1.00	0.00
ATOM	7958	HW2	SOL	2278	34.330	10.850	30.810	1.00	0.00
ATOM	7959	OW	SOL	2279	36.020	45.380	4.710	1.00	0.00
ATOM	7960	HW1	SOL	2279	35.400	45.890	4.190	1.00	0.00
ATOM	7961	HW2	SOL	2279	35.810	45.590	5.620	1.00	0.00
ATOM	7962	OW	SOL	2280	12.680	24.990	47.430	1.00	0.00
ATOM	7963	HW1	SOL	2280	12.500	24.440	46.660	1.00	0.00

ATOM	7964	HW2	SOL	2280	12.130	24.630	48.120	1.00	0.00
ATOM	7965	OW	SOL	2281	32.730	48.640	51.250	1.00	0.00
ATOM	7966	HW1	SOL	2281	33.020	49.540	51.070	1.00	0.00
ATOM	7967	HW2	SOL	2281	32.080	48.740	51.950	1.00	0.00
ATOM	7968	OW	SOL	2282	37.690	53.660	40.410	1.00	0.00
ATOM	7969	HW1	SOL	2282	37.440	53.400	41.290	1.00	0.00
ATOM	7970	HW2	SOL	2282	38.500	53.180	40.230	1.00	0.00
ATOM	7971	OW	SOL	2283	19.650	9.570	4.590	1.00	0.00
ATOM	7972	HW1	SOL	2283	20.150	10.220	4.100	1.00	0.00
ATOM	7973	HW2	SOL	2283	20.170	9.410	5.380	1.00	0.00
ATOM	7974	OW	SOL	2284	0.050	21.100	29.430	1.00	0.00
ATOM	7975	HW1	SOL	2284	-0.320	20.280	29.770	1.00	0.00
ATOM	7976	HW2	SOL	2284	-0.710	21.640	29.220	1.00	0.00
ATOM	7977	OW	SOL	2285	38.700	49.760	4.250	1.00	0.00
ATOM	7978	HW1	SOL	2285	39.120	50.230	4.970	1.00	0.00
ATOM	7979	HW2	SOL	2285	39.430	49.350	3.790	1.00	0.00
ATOM	7980	OW	SOL	2286	36.710	44.280	43.720	1.00	0.00
ATOM	7981	HW1	SOL	2286	36.070	44.970	43.920	1.00	0.00
ATOM	7982	HW2	SOL	2286	36.890	43.870	44.560	1.00	0.00
ATOM	7983	OW	SOL	2287	33.560	10.090	42.360	1.00	0.00
ATOM	7984	HW1	SOL	2287	33.350	9.770	43.240	1.00	0.00
ATOM	7985	HW2	SOL	2287	34.080	10.880	42.510	1.00	0.00
ATOM	7986	OW	SOL	2288	52.480	54.810	0.230	1.00	0.00
ATOM	7987	HW1	SOL	2288	52.730	55.570	0.750	1.00	0.00
ATOM	7988	HW2	SOL	2288	53.320	54.410	-0.030	1.00	0.00
ATOM	7989	OW	SOL	2289	52.030	39.040	0.800	1.00	0.00
ATOM	7990	HW1	SOL	2289	51.920	38.710	-0.100	1.00	0.00
ATOM	7991	HW2	SOL	2289	51.970	38.260	1.350	1.00	0.00
ATOM	7992	OW	SOL	2290	33.250	17.230	20.830	1.00	0.00
ATOM	7993	HW1	SOL	2290	32.920	17.630	21.630	1.00	0.00
ATOM	7994	HW2	SOL	2290	32.490	16.770	20.460	1.00	0.00
ATOM	7995	OW	SOL	2291	46.850	7.910	17.620	1.00	0.00
ATOM	7996	HW1	SOL	2291	47.560	8.430	17.990	1.00	0.00
ATOM	7997	HW2	SOL	2291	46.140	7.990	18.250	1.00	0.00
ATOM	7998	OW	SOL	2292	2.950	28.840	7.670	1.00	0.00
ATOM	7999	HW1	SOL	2292	3.110	29.660	8.130	1.00	0.00
ATOM	8000	HW2	SOL	2292	3.650	28.790	7.020	1.00	0.00
ATOM	8001	OW	SOL	2293	8.790	8.230	30.210	1.00	0.00
ATOM	8002	HW1	SOL	2293	8.140	8.910	30.420	1.00	0.00
ATOM	8003	HW2	SOL	2293	9.530	8.420	30.780	1.00	0.00
ATOM	8004	OW	SOL	2294	3.790	42.130	37.170	1.00	0.00
ATOM	8005	HW1	SOL	2294	3.860	42.460	38.070	1.00	0.00
ATOM	8006	HW2	SOL	2294	4.010	42.880	36.620	1.00	0.00
ATOM	8007	OW	SOL	2295	51.070	38.290	40.970	1.00	0.00

ATOM	8008	HW1	SOL	2295	51.190	39.240	40.900	1.00	0.00
ATOM	8009	HW2	SOL	2295	50.290	38.190	41.520	1.00	0.00
ATOM	8010	OW	SOL	2296	31.560	42.580	8.450	1.00	0.00
ATOM	8011	HW1	SOL	2296	30.840	42.590	7.830	1.00	0.00
ATOM	8012	HW2	SOL	2296	32.040	41.780	8.250	1.00	0.00
ATOM	8013	OW	SOL	2297	31.810	48.820	22.470	1.00	0.00
ATOM	8014	HW1	SOL	2297	32.730	48.570	22.530	1.00	0.00
ATOM	8015	HW2	SOL	2297	31.650	49.360	23.250	1.00	0.00
ATOM	8016	OW	SOL	2298	45.820	38.420	55.270	1.00	0.00
ATOM	8017	HW1	SOL	2298	46.290	37.940	54.590	1.00	0.00
ATOM	8018	HW2	SOL	2298	46.160	38.060	56.090	1.00	0.00
ATOM	8019	OW	SOL	2299	10.330	3.450	21.920	1.00	0.00
ATOM	8020	HW1	SOL	2299	11.070	3.540	22.510	1.00	0.00
ATOM	8021	HW2	SOL	2299	10.120	2.520	21.930	1.00	0.00
ATOM	8022	OW	SOL	2300	40.250	12.760	40.740	1.00	0.00
ATOM	8023	HW1	SOL	2300	39.960	12.200	41.470	1.00	0.00
ATOM	8024	HW2	SOL	2300	39.660	13.510	40.770	1.00	0.00
ATOM	8025	OW	SOL	2301	26.900	14.620	12.770	1.00	0.00
ATOM	8026	HW1	SOL	2301	26.890	15.150	13.570	1.00	0.00
ATOM	8027	HW2	SOL	2301	26.200	13.980	12.900	1.00	0.00
ATOM	8028	OW	SOL	2302	29.930	45.610	2.070	1.00	0.00
ATOM	8029	HW1	SOL	2302	29.730	45.800	1.150	1.00	0.00
ATOM	8030	HW2	SOL	2302	30.880	45.570	2.110	1.00	0.00
ATOM	8031	OW	SOL	2303	26.490	4.370	29.490	1.00	0.00
ATOM	8032	HW1	SOL	2303	27.180	3.840	29.100	1.00	0.00
ATOM	8033	HW2	SOL	2303	26.260	5.000	28.800	1.00	0.00
ATOM	8034	OW	SOL	2304	14.300	37.610	39.850	1.00	0.00
ATOM	8035	HW1	SOL	2304	13.950	38.220	40.500	1.00	0.00
ATOM	8036	HW2	SOL	2304	14.140	38.030	39.010	1.00	0.00
ATOM	8037	OW	SOL	2305	11.170	12.250	42.650	1.00	0.00
ATOM	8038	HW1	SOL	2305	10.810	11.460	42.250	1.00	0.00
ATOM	8039	HW2	SOL	2305	10.680	12.350	43.460	1.00	0.00
ATOM	8040	OW	SOL	2306	30.040	38.620	1.510	1.00	0.00
ATOM	8041	HW1	SOL	2306	29.510	39.300	1.100	1.00	0.00
ATOM	8042	HW2	SOL	2306	30.910	38.730	1.130	1.00	0.00
ATOM	8043	OW	SOL	2307	13.340	5.070	4.090	1.00	0.00
ATOM	8044	HW1	SOL	2307	12.660	5.100	4.760	1.00	0.00
ATOM	8045	HW2	SOL	2307	12.880	4.870	3.280	1.00	0.00
ATOM	8046	OW	SOL	2308	20.770	12.360	11.680	1.00	0.00
ATOM	8047	HW1	SOL	2308	20.500	12.900	10.930	1.00	0.00
ATOM	8048	HW2	SOL	2308	21.310	11.670	11.300	1.00	0.00
ATOM	8049	OW	SOL	2309	43.860	20.610	42.120	1.00	0.00
ATOM	8050	HW1	SOL	2309	43.560	21.050	41.320	1.00	0.00
ATOM	8051	HW2	SOL	2309	43.910	21.310	42.770	1.00	0.00

ATOM	8052	OW	SOL	2310	28.750	44.950	55.480	1.00	0.00
ATOM	8053	HW1	SOL	2310	27.790	45.040	55.540	1.00	0.00
ATOM	8054	HW2	SOL	2310	29.000	45.490	54.740	1.00	0.00
ATOM	8055	OW	SOL	2311	37.510	26.920	8.810	1.00	0.00
ATOM	8056	HW1	SOL	2311	37.710	27.800	9.130	1.00	0.00
ATOM	8057	HW2	SOL	2311	36.580	26.950	8.600	1.00	0.00
ATOM	8058	OW	SOL	2312	49.180	14.740	36.710	1.00	0.00
ATOM	8059	HW1	SOL	2312	49.520	14.990	37.580	1.00	0.00
ATOM	8060	HW2	SOL	2312	48.360	14.290	36.900	1.00	0.00
ATOM	8061	OW	SOL	2313	55.640	55.770	25.080	1.00	0.00
ATOM	8062	HW1	SOL	2313	54.870	55.410	24.630	1.00	0.00
ATOM	8063	HW2	SOL	2313	55.300	56.510	25.580	1.00	0.00
ATOM	8064	OW	SOL	2314	12.920	16.320	11.030	1.00	0.00
ATOM	8065	HW1	SOL	2314	13.410	15.750	10.430	1.00	0.00
ATOM	8066	HW2	SOL	2314	12.380	15.720	11.540	1.00	0.00
ATOM	8067	OW	SOL	2315	50.880	2.550	43.060	1.00	0.00
ATOM	8068	HW1	SOL	2315	50.480	2.180	43.840	1.00	0.00
ATOM	8069	HW2	SOL	2315	51.820	2.430	43.180	1.00	0.00
ATOM	8070	OW	SOL	2316	35.570	19.220	54.250	1.00	0.00
ATOM	8071	HW1	SOL	2316	34.860	18.610	54.080	1.00	0.00
ATOM	8072	HW2	SOL	2316	35.300	20.030	53.820	1.00	0.00
ATOM	8073	OW	SOL	2317	34.910	17.980	34.160	1.00	0.00
ATOM	8074	HW1	SOL	2317	34.870	18.930	34.050	1.00	0.00
ATOM	8075	HW2	SOL	2317	35.430	17.680	33.410	1.00	0.00
ATOM	8076	OW	SOL	2318	35.430	43.270	38.710	1.00	0.00
ATOM	8077	HW1	SOL	2318	34.660	42.980	39.200	1.00	0.00
ATOM	8078	HW2	SOL	2318	36.160	43.110	39.310	1.00	0.00
ATOM	8079	OW	SOL	2319	37.040	7.320	35.170	1.00	0.00
ATOM	8080	HW1	SOL	2319	37.450	7.330	36.030	1.00	0.00
ATOM	8081	HW2	SOL	2319	37.490	6.640	34.690	1.00	0.00
ATOM	8082	OW	SOL	2320	30.340	54.520	33.470	1.00	0.00
ATOM	8083	HW1	SOL	2320	31.190	54.660	33.870	1.00	0.00
ATOM	8084	HW2	SOL	2320	29.840	55.310	33.670	1.00	0.00
ATOM	8085	OW	SOL	2321	21.190	42.270	28.030	1.00	0.00
ATOM	8086	HW1	SOL	2321	21.620	42.400	27.190	1.00	0.00
ATOM	8087	HW2	SOL	2321	20.260	42.200	27.820	1.00	0.00
ATOM	8088	OW	SOL	2322	2.610	42.210	48.180	1.00	0.00
ATOM	8089	HW1	SOL	2322	2.430	43.100	47.870	1.00	0.00
ATOM	8090	HW2	SOL	2322	2.660	41.690	47.380	1.00	0.00
ATOM	8091	OW	SOL	2323	12.460	13.920	46.290	1.00	0.00
ATOM	8092	HW1	SOL	2323	11.570	14.260	46.400	1.00	0.00
ATOM	8093	HW2	SOL	2323	13.020	14.630	46.610	1.00	0.00
ATOM	8094	OW	SOL	2324	4.870	7.860	1.420	1.00	0.00
ATOM	8095	HW1	SOL	2324	5.690	8.350	1.510	1.00	0.00

ATOM	8096	HW2	SOL	2324	4.860	7.580	0.500	1.00	0.00
ATOM	8097	OW	SOL	2325	6.540	34.690	33.120	1.00	0.00
ATOM	8098	HW1	SOL	2325	5.860	35.310	33.390	1.00	0.00
ATOM	8099	HW2	SOL	2325	7.330	35.220	33.010	1.00	0.00
ATOM	8100	OW	SOL	2326	32.310	5.840	17.070	1.00	0.00
ATOM	8101	HW1	SOL	2326	33.250	6.020	17.100	1.00	0.00
ATOM	8102	HW2	SOL	2326	32.220	5.130	16.440	1.00	0.00
ATOM	8103	OW	SOL	2327	8.360	4.380	43.210	1.00	0.00
ATOM	8104	HW1	SOL	2327	7.670	3.780	42.930	1.00	0.00
ATOM	8105	HW2	SOL	2327	7.940	5.250	43.210	1.00	0.00
ATOM	8106	OW	SOL	2328	54.350	47.970	14.420	1.00	0.00
ATOM	8107	HW1	SOL	2328	54.940	48.460	14.990	1.00	0.00
ATOM	8108	HW2	SOL	2328	53.510	47.980	14.880	1.00	0.00
ATOM	8109	OW	SOL	2329	43.530	13.970	7.040	1.00	0.00
ATOM	8110	HW1	SOL	2329	43.310	13.050	7.180	1.00	0.00
ATOM	8111	HW2	SOL	2329	44.070	14.210	7.790	1.00	0.00
ATOM	8112	OW	SOL	2330	8.450	47.250	17.760	1.00	0.00
ATOM	8113	HW1	SOL	2330	7.710	46.720	17.450	1.00	0.00
ATOM	8114	HW2	SOL	2330	8.360	48.080	17.290	1.00	0.00
ATOM	8115	OW	SOL	2331	53.460	52.600	35.160	1.00	0.00
ATOM	8116	HW1	SOL	2331	53.560	53.430	34.690	1.00	0.00
ATOM	8117	HW2	SOL	2331	54.080	52.010	34.750	1.00	0.00
ATOM	8118	OW	SOL	2332	26.140	34.380	53.810	1.00	0.00
ATOM	8119	HW1	SOL	2332	25.590	34.950	54.340	1.00	0.00
ATOM	8120	HW2	SOL	2332	25.770	33.510	53.940	1.00	0.00
ATOM	8121	OW	SOL	2333	54.770	51.570	8.680	1.00	0.00
ATOM	8122	HW1	SOL	2333	54.050	52.030	8.240	1.00	0.00
ATOM	8123	HW2	SOL	2333	54.390	50.720	8.910	1.00	0.00
ATOM	8124	OW	SOL	2334	47.860	34.960	10.260	1.00	0.00
ATOM	8125	HW1	SOL	2334	47.720	35.080	9.320	1.00	0.00
ATOM	8126	HW2	SOL	2334	47.430	34.130	10.460	1.00	0.00
ATOM	8127	OW	SOL	2335	39.830	15.660	10.150	1.00	0.00
ATOM	8128	HW1	SOL	2335	40.290	15.600	9.310	1.00	0.00
ATOM	8129	HW2	SOL	2335	39.450	16.540	10.150	1.00	0.00
ATOM	8130	OW	SOL	2336	21.090	0.380	12.370	1.00	0.00
ATOM	8131	HW1	SOL	2336	20.930	0.450	13.310	1.00	0.00
ATOM	8132	HW2	SOL	2336	20.240	0.140	12.000	1.00	0.00
ATOM	8133	OW	SOL	2337	12.130	55.730	32.590	1.00	0.00
ATOM	8134	HW1	SOL	2337	11.570	55.860	33.350	1.00	0.00
ATOM	8135	HW2	SOL	2337	12.580	56.560	32.480	1.00	0.00
ATOM	8136	OW	SOL	2338	7.850	17.050	48.320	1.00	0.00
ATOM	8137	HW1	SOL	2338	7.710	17.960	48.570	1.00	0.00
ATOM	8138	HW2	SOL	2338	7.050	16.600	48.590	1.00	0.00
ATOM	8139	OW	SOL	2339	47.480	34.570	7.510	1.00	0.00

ATOM	8140	HW1	SOL	2339	47.400	33.650	7.770	1.00	0.00
ATOM	8141	HW2	SOL	2339	47.410	34.560	6.560	1.00	0.00
ATOM	8142	OW	SOL	2340	27.710	38.370	43.740	1.00	0.00
ATOM	8143	HW1	SOL	2340	27.010	37.870	43.330	1.00	0.00
ATOM	8144	HW2	SOL	2340	27.280	39.160	44.070	1.00	0.00
ATOM	8145	OW	SOL	2341	15.020	32.710	4.220	1.00	0.00
ATOM	8146	HW1	SOL	2341	15.620	33.150	3.620	1.00	0.00
ATOM	8147	HW2	SOL	2341	15.130	31.780	4.030	1.00	0.00
ATOM	8148	OW	SOL	2342	40.290	55.530	30.740	1.00	0.00
ATOM	8149	HW1	SOL	2342	40.120	54.970	29.990	1.00	0.00
ATOM	8150	HW2	SOL	2342	41.230	55.660	30.740	1.00	0.00
ATOM	8151	OW	SOL	2343	35.060	16.110	3.830	1.00	0.00
ATOM	8152	HW1	SOL	2343	35.480	16.560	4.570	1.00	0.00
ATOM	8153	HW2	SOL	2343	34.370	16.710	3.540	1.00	0.00
ATOM	8154	OW	SOL	2344	17.960	50.810	37.300	1.00	0.00
ATOM	8155	HW1	SOL	2344	17.250	51.400	37.530	1.00	0.00
ATOM	8156	HW2	SOL	2344	18.670	51.390	37.020	1.00	0.00
ATOM	8157	OW	SOL	2345	17.650	48.780	7.510	1.00	0.00
ATOM	8158	HW1	SOL	2345	17.610	48.170	8.250	1.00	0.00
ATOM	8159	HW2	SOL	2345	18.590	48.980	7.410	1.00	0.00
ATOM	8160	OW	SOL	2346	0.200	44.860	4.280	1.00	0.00
ATOM	8161	HW1	SOL	2346	1.100	44.720	4.600	1.00	0.00
ATOM	8162	HW2	SOL	2346	-0.180	43.980	4.270	1.00	0.00
ATOM	8163	OW	SOL	2347	35.250	48.280	17.200	1.00	0.00
ATOM	8164	HW1	SOL	2347	35.490	48.800	17.980	1.00	0.00
ATOM	8165	HW2	SOL	2347	35.870	47.550	17.210	1.00	0.00
ATOM	8166	OW	SOL	2348	2.690	20.810	4.460	1.00	0.00
ATOM	8167	HW1	SOL	2348	2.900	20.970	3.540	1.00	0.00
ATOM	8168	HW2	SOL	2348	2.780	21.670	4.880	1.00	0.00
ATOM	8169	OW	SOL	2349	45.350	11.080	8.500	1.00	0.00
ATOM	8170	HW1	SOL	2349	45.650	11.340	7.630	1.00	0.00
ATOM	8171	HW2	SOL	2349	44.890	11.850	8.830	1.00	0.00
ATOM	8172	OW	SOL	2350	27.660	54.150	28.310	1.00	0.00
ATOM	8173	HW1	SOL	2350	27.100	54.100	27.540	1.00	0.00
ATOM	8174	HW2	SOL	2350	28.490	54.510	27.990	1.00	0.00
ATOM	8175	OW	SOL	2351	14.340	28.250	32.710	1.00	0.00
ATOM	8176	HW1	SOL	2351	14.650	29.080	32.360	1.00	0.00
ATOM	8177	HW2	SOL	2351	14.260	28.400	33.650	1.00	0.00
ATOM	8178	OW	SOL	2352	47.190	42.950	34.880	1.00	0.00
ATOM	8179	HW1	SOL	2352	46.830	42.140	35.250	1.00	0.00
ATOM	8180	HW2	SOL	2352	48.140	42.860	34.990	1.00	0.00
ATOM	8181	OW	SOL	2353	50.210	43.740	37.070	1.00	0.00
ATOM	8182	HW1	SOL	2353	49.800	44.060	36.260	1.00	0.00
ATOM	8183	HW2	SOL	2353	50.640	42.920	36.810	1.00	0.00

ATOM	8184	OW	SOL	2354	2.700	34.710	0.890	1.00	0.00
ATOM	8185	HW1	SOL	2354	3.160	34.260	0.190	1.00	0.00
ATOM	8186	HW2	SOL	2354	2.120	35.330	0.440	1.00	0.00
ATOM	8187	OW	SOL	2355	40.210	39.030	12.790	1.00	0.00
ATOM	8188	HW1	SOL	2355	40.160	39.420	11.920	1.00	0.00
ATOM	8189	HW2	SOL	2355	40.220	39.780	13.390	1.00	0.00
ATOM	8190	OW	SOL	2356	37.150	46.410	21.690	1.00	0.00
ATOM	8191	HW1	SOL	2356	37.660	47.160	21.980	1.00	0.00
ATOM	8192	HW2	SOL	2356	37.790	45.700	21.610	1.00	0.00
ATOM	8193	OW	SOL	2357	44.400	12.660	39.110	1.00	0.00
ATOM	8194	HW1	SOL	2357	44.560	12.330	38.230	1.00	0.00
ATOM	8195	HW2	SOL	2357	44.880	12.070	39.680	1.00	0.00
ATOM	8196	OW	SOL	2358	50.970	47.100	53.940	1.00	0.00
ATOM	8197	HW1	SOL	2358	50.330	46.850	53.280	1.00	0.00
ATOM	8198	HW2	SOL	2358	51.770	46.630	53.700	1.00	0.00
ATOM	8199	OW	SOL	2359	18.570	43.230	27.140	1.00	0.00
ATOM	8200	HW1	SOL	2359	17.870	43.050	26.510	1.00	0.00
ATOM	8201	HW2	SOL	2359	18.840	44.130	26.960	1.00	0.00
ATOM	8202	OW	SOL	2360	45.080	18.880	16.740	1.00	0.00
ATOM	8203	HW1	SOL	2360	44.550	18.120	16.950	1.00	0.00
ATOM	8204	HW2	SOL	2360	44.700	19.600	17.250	1.00	0.00
ATOM	8205	OW	SOL	2361	47.210	5.590	15.950	1.00	0.00
ATOM	8206	HW1	SOL	2361	47.910	5.730	15.320	1.00	0.00
ATOM	8207	HW2	SOL	2361	47.120	6.420	16.410	1.00	0.00
ATOM	8208	OW	SOL	2362	44.330	38.890	9.520	1.00	0.00
ATOM	8209	HW1	SOL	2362	45.040	38.400	9.110	1.00	0.00
ATOM	8210	HW2	SOL	2362	44.760	39.600	9.980	1.00	0.00
ATOM	8211	OW	SOL	2363	0.770	53.250	13.520	1.00	0.00
ATOM	8212	HW1	SOL	2363	0.910	53.980	12.920	1.00	0.00
ATOM	8213	HW2	SOL	2363	0.860	52.470	12.980	1.00	0.00
ATOM	8214	OW	SOL	2364	18.890	16.660	49.710	1.00	0.00
ATOM	8215	HW1	SOL	2364	19.430	16.890	50.470	1.00	0.00
ATOM	8216	HW2	SOL	2364	18.150	16.180	50.090	1.00	0.00
ATOM	8217	OW	SOL	2365	52.030	21.440	1.230	1.00	0.00
ATOM	8218	HW1	SOL	2365	51.140	21.760	1.090	1.00	0.00
ATOM	8219	HW2	SOL	2365	52.440	21.480	0.370	1.00	0.00
ATOM	8220	OW	SOL	2366	10.900	14.550	3.110	1.00	0.00
ATOM	8221	HW1	SOL	2366	11.330	13.810	2.670	1.00	0.00
ATOM	8222	HW2	SOL	2366	11.410	14.690	3.900	1.00	0.00
ATOM	8223	OW	SOL	2367	3.360	10.110	46.910	1.00	0.00
ATOM	8224	HW1	SOL	2367	3.340	9.280	47.390	1.00	0.00
ATOM	8225	HW2	SOL	2367	4.240	10.140	46.520	1.00	0.00
ATOM	8226	OW	SOL	2368	14.530	13.320	31.180	1.00	0.00
ATOM	8227	HW1	SOL	2368	14.630	12.390	31.380	1.00	0.00

ATOM	8228	HW2	SOL	2368	13.960	13.350	30.410	1.00	0.00
ATOM	8229	OW	SOL	2369	24.620	9.210	5.710	1.00	0.00
ATOM	8230	HW1	SOL	2369	25.250	9.930	5.710	1.00	0.00
ATOM	8231	HW2	SOL	2369	23.850	9.560	6.150	1.00	0.00
ATOM	8232	OW	SOL	2370	41.870	0.550	20.370	1.00	0.00
ATOM	8233	HW1	SOL	2370	41.910	0.870	21.270	1.00	0.00
ATOM	8234	HW2	SOL	2370	40.930	0.450	20.190	1.00	0.00
ATOM	8235	OW	SOL	2371	6.290	9.270	28.160	1.00	0.00
ATOM	8236	HW1	SOL	2371	6.450	8.780	28.960	1.00	0.00
ATOM	8237	HW2	SOL	2371	6.660	8.720	27.460	1.00	0.00
ATOM	8238	OW	SOL	2372	35.640	5.230	39.700	1.00	0.00
ATOM	8239	HW1	SOL	2372	34.850	5.540	40.140	1.00	0.00
ATOM	8240	HW2	SOL	2372	35.340	4.480	39.180	1.00	0.00
ATOM	8241	OW	SOL	2373	32.630	16.020	18.080	1.00	0.00
ATOM	8242	HW1	SOL	2373	31.970	16.670	18.340	1.00	0.00
ATOM	8243	HW2	SOL	2373	33.460	16.470	18.180	1.00	0.00
ATOM	8244	OW	SOL	2374	55.800	13.430	45.840	1.00	0.00
ATOM	8245	HW1	SOL	2374	56.430	12.720	45.910	1.00	0.00
ATOM	8246	HW2	SOL	2374	56.330	14.230	45.920	1.00	0.00
ATOM	8247	OW	SOL	2375	3.470	31.070	48.740	1.00	0.00
ATOM	8248	HW1	SOL	2375	2.860	30.730	48.080	1.00	0.00
ATOM	8249	HW2	SOL	2375	3.430	30.440	49.460	1.00	0.00
ATOM	8250	OW	SOL	2376	55.880	48.870	26.030	1.00	0.00
ATOM	8251	HW1	SOL	2376	56.050	48.020	26.440	1.00	0.00
ATOM	8252	HW2	SOL	2376	55.870	49.490	26.760	1.00	0.00
ATOM	8253	OW	SOL	2377	6.230	20.400	13.130	1.00	0.00
ATOM	8254	HW1	SOL	2377	6.640	21.260	13.220	1.00	0.00
ATOM	8255	HW2	SOL	2377	6.080	20.310	12.190	1.00	0.00
ATOM	8256	OW	SOL	2378	2.690	51.610	16.710	1.00	0.00
ATOM	8257	HW1	SOL	2378	3.090	51.320	17.530	1.00	0.00
ATOM	8258	HW2	SOL	2378	2.730	50.840	16.140	1.00	0.00
ATOM	8259	OW	SOL	2379	40.530	8.850	46.370	1.00	0.00
ATOM	8260	HW1	SOL	2379	40.130	9.250	47.140	1.00	0.00
ATOM	8261	HW2	SOL	2379	40.400	7.910	46.490	1.00	0.00
ATOM	8262	OW	SOL	2380	24.650	42.060	1.730	1.00	0.00
ATOM	8263	HW1	SOL	2380	24.720	42.130	2.680	1.00	0.00
ATOM	8264	HW2	SOL	2380	25.070	42.860	1.400	1.00	0.00
ATOM	8265	OW	SOL	2381	12.070	54.740	25.300	1.00	0.00
ATOM	8266	HW1	SOL	2381	12.150	54.300	26.140	1.00	0.00
ATOM	8267	HW2	SOL	2381	11.210	54.470	24.970	1.00	0.00
ATOM	8268	OW	SOL	2382	14.100	19.450	50.640	1.00	0.00
ATOM	8269	HW1	SOL	2382	13.680	19.020	51.380	1.00	0.00
ATOM	8270	HW2	SOL	2382	14.730	20.050	51.040	1.00	0.00
ATOM	8271	OW	SOL	2383	42.800	30.010	19.510	1.00	0.00

ATOM	8272	HW1	SOL	2383	42.300	30.810	19.320	1.00	0.00
ATOM	8273	HW2	SOL	2383	43.210	29.780	18.670	1.00	0.00
ATOM	8274	OW	SOL	2384	7.620	37.900	22.800	1.00	0.00
ATOM	8275	HW1	SOL	2384	6.810	38.330	22.540	1.00	0.00
ATOM	8276	HW2	SOL	2384	7.440	36.970	22.680	1.00	0.00
ATOM	8277	OW	SOL	2385	21.800	27.650	32.020	1.00	0.00
ATOM	8278	HW1	SOL	2385	22.640	28.070	32.200	1.00	0.00
ATOM	8279	HW2	SOL	2385	21.510	27.310	32.870	1.00	0.00
ATOM	8280	OW	SOL	2386	33.350	48.760	39.550	1.00	0.00
ATOM	8281	HW1	SOL	2386	34.080	48.310	39.980	1.00	0.00
ATOM	8282	HW2	SOL	2386	33.360	48.430	38.650	1.00	0.00
ATOM	8283	OW	SOL	2387	5.550	39.480	22.010	1.00	0.00
ATOM	8284	HW1	SOL	2387	6.350	40.000	22.010	1.00	0.00
ATOM	8285	HW2	SOL	2387	4.960	39.940	21.410	1.00	0.00
ATOM	8286	OW	SOL	2388	2.910	14.560	3.010	1.00	0.00
ATOM	8287	HW1	SOL	2388	2.790	14.150	2.160	1.00	0.00
ATOM	8288	HW2	SOL	2388	3.700	14.160	3.370	1.00	0.00
ATOM	8289	OW	SOL	2389	37.910	47.600	43.180	1.00	0.00
ATOM	8290	HW1	SOL	2389	37.260	47.410	43.850	1.00	0.00
ATOM	8291	HW2	SOL	2389	37.620	47.100	42.410	1.00	0.00
ATOM	8292	OW	SOL	2390	54.650	14.240	12.680	1.00	0.00
ATOM	8293	HW1	SOL	2390	54.000	14.870	12.980	1.00	0.00
ATOM	8294	HW2	SOL	2390	55.320	14.250	13.370	1.00	0.00
ATOM	8295	OW	SOL	2391	42.540	46.990	22.740	1.00	0.00
ATOM	8296	HW1	SOL	2391	41.960	46.510	23.330	1.00	0.00
ATOM	8297	HW2	SOL	2391	41.950	47.590	22.270	1.00	0.00
ATOM	8298	OW	SOL	2392	30.120	48.740	3.820	1.00	0.00
ATOM	8299	HW1	SOL	2392	30.950	48.950	3.380	1.00	0.00
ATOM	8300	HW2	SOL	2392	30.360	48.120	4.510	1.00	0.00
ATOM	8301	OW	SOL	2393	13.060	38.630	42.300	1.00	0.00
ATOM	8302	HW1	SOL	2393	13.940	38.680	42.670	1.00	0.00
ATOM	8303	HW2	SOL	2393	12.810	37.710	42.400	1.00	0.00
ATOM	8304	OW	SOL	2394	15.890	45.260	42.090	1.00	0.00
ATOM	8305	HW1	SOL	2394	15.470	45.840	42.730	1.00	0.00
ATOM	8306	HW2	SOL	2394	16.820	45.500	42.120	1.00	0.00
ATOM	8307	OW	SOL	2395	49.690	16.830	32.350	1.00	0.00
ATOM	8308	HW1	SOL	2395	49.880	16.480	33.220	1.00	0.00
ATOM	8309	HW2	SOL	2395	50.330	17.530	32.230	1.00	0.00
ATOM	8310	OW	SOL	2396	30.050	36.700	51.940	1.00	0.00
ATOM	8311	HW1	SOL	2396	30.410	37.560	52.140	1.00	0.00
ATOM	8312	HW2	SOL	2396	29.140	36.740	52.250	1.00	0.00
ATOM	8313	OW	SOL	2397	17.260	55.340	12.720	1.00	0.00
ATOM	8314	HW1	SOL	2397	17.300	55.530	13.660	1.00	0.00
ATOM	8315	HW2	SOL	2397	16.420	55.710	12.440	1.00	0.00

ATOM	8316	OW	SOL	2398	38.280	34.330	4.220	1.00	0.00
ATOM	8317	HW1	SOL	2398	38.590	33.530	3.790	1.00	0.00
ATOM	8318	HW2	SOL	2398	39.070	34.840	4.380	1.00	0.00
ATOM	8319	OW	SOL	2399	51.120	3.220	0.680	1.00	0.00
ATOM	8320	HW1	SOL	2399	50.800	3.550	-0.160	1.00	0.00
ATOM	8321	HW2	SOL	2399	52.030	3.510	0.730	1.00	0.00
ATOM	8322	OW	SOL	2400	54.700	40.160	10.310	1.00	0.00
ATOM	8323	HW1	SOL	2400	55.380	39.800	10.880	1.00	0.00
ATOM	8324	HW2	SOL	2400	54.990	41.050	10.120	1.00	0.00
ATOM	8325	OW	SOL	2401	17.020	14.030	42.910	1.00	0.00
ATOM	8326	HW1	SOL	2401	17.710	13.380	43.080	1.00	0.00
ATOM	8327	HW2	SOL	2401	16.450	13.970	43.680	1.00	0.00
ATOM	8328	OW	SOL	2402	9.640	49.360	52.430	1.00	0.00
ATOM	8329	HW1	SOL	2402	9.650	48.620	53.030	1.00	0.00
ATOM	8330	HW2	SOL	2402	10.290	49.960	52.780	1.00	0.00
ATOM	8331	OW	SOL	2403	35.430	52.470	21.390	1.00	0.00
ATOM	8332	HW1	SOL	2403	34.850	52.460	20.620	1.00	0.00
ATOM	8333	HW2	SOL	2403	36.300	52.300	21.040	1.00	0.00
ATOM	8334	OW	SOL	2404	52.860	15.620	17.230	1.00	0.00
ATOM	8335	HW1	SOL	2404	52.730	14.670	17.210	1.00	0.00
ATOM	8336	HW2	SOL	2404	52.750	15.860	18.150	1.00	0.00
ATOM	8337	OW	SOL	2405	41.240	45.360	32.910	1.00	0.00
ATOM	8338	HW1	SOL	2405	41.170	44.420	33.100	1.00	0.00
ATOM	8339	HW2	SOL	2405	41.760	45.390	32.100	1.00	0.00
ATOM	8340	OW	SOL	2406	52.160	12.620	6.100	1.00	0.00
ATOM	8341	HW1	SOL	2406	52.940	12.950	5.650	1.00	0.00
ATOM	8342	HW2	SOL	2406	51.430	12.870	5.530	1.00	0.00
ATOM	8343	OW	SOL	2407	46.430	41.330	2.830	1.00	0.00
ATOM	8344	HW1	SOL	2407	46.730	41.780	2.040	1.00	0.00
ATOM	8345	HW2	SOL	2407	45.480	41.420	2.810	1.00	0.00
ATOM	8346	OW	SOL	2408	9.780	9.300	8.400	1.00	0.00
ATOM	8347	HW1	SOL	2408	9.320	8.540	8.760	1.00	0.00
ATOM	8348	HW2	SOL	2408	10.710	9.070	8.460	1.00	0.00
ATOM	8349	OW	SOL	2409	16.410	22.070	23.190	1.00	0.00
ATOM	8350	HW1	SOL	2409	15.820	22.500	23.810	1.00	0.00
ATOM	8351	HW2	SOL	2409	16.740	22.790	22.640	1.00	0.00
ATOM	8352	OW	SOL	2410	9.050	27.280	47.580	1.00	0.00
ATOM	8353	HW1	SOL	2410	8.150	27.190	47.280	1.00	0.00
ATOM	8354	HW2	SOL	2410	9.570	26.840	46.900	1.00	0.00
ATOM	8355	OW	SOL	2411	9.660	40.090	25.520	1.00	0.00
ATOM	8356	HW1	SOL	2411	9.710	41.040	25.450	1.00	0.00
ATOM	8357	HW2	SOL	2411	8.730	39.900	25.640	1.00	0.00
ATOM	8358	OW	SOL	2412	9.320	38.410	3.330	1.00	0.00
ATOM	8359	HW1	SOL	2412	10.080	37.920	3.640	1.00	0.00

ATOM	8360	HW2	SOL	2412	8.670	37.740	3.090	1.00	0.00
ATOM	8361	OW	SOL	2413	22.010	53.530	46.370	1.00	0.00
ATOM	8362	HW1	SOL	2413	22.800	54.060	46.260	1.00	0.00
ATOM	8363	HW2	SOL	2413	22.340	52.690	46.680	1.00	0.00
ATOM	8364	OW	SOL	2414	25.150	34.520	4.960	1.00	0.00
ATOM	8365	HW1	SOL	2414	25.050	34.650	4.010	1.00	0.00
ATOM	8366	HW2	SOL	2414	24.920	33.600	5.090	1.00	0.00
ATOM	8367	OW	SOL	2415	17.950	43.180	29.860	1.00	0.00
ATOM	8368	HW1	SOL	2415	18.270	42.920	29.000	1.00	0.00
ATOM	8369	HW2	SOL	2415	18.310	42.520	30.460	1.00	0.00
ATOM	8370	OW	SOL	2416	47.090	41.530	43.170	1.00	0.00
ATOM	8371	HW1	SOL	2416	46.420	41.760	43.810	1.00	0.00
ATOM	8372	HW2	SOL	2416	47.680	42.290	43.140	1.00	0.00
ATOM	8373	OW	SOL	2417	5.080	36.210	35.520	1.00	0.00
ATOM	8374	HW1	SOL	2417	4.580	36.920	35.120	1.00	0.00
ATOM	8375	HW2	SOL	2417	4.810	36.230	36.440	1.00	0.00
ATOM	8376	OW	SOL	2418	35.320	13.600	17.030	1.00	0.00
ATOM	8377	HW1	SOL	2418	35.390	12.880	17.660	1.00	0.00
ATOM	8378	HW2	SOL	2418	35.760	14.340	17.460	1.00	0.00
ATOM	8379	OW	SOL	2419	34.310	31.040	2.550	1.00	0.00
ATOM	8380	HW1	SOL	2419	35.130	31.190	3.040	1.00	0.00
ATOM	8381	HW2	SOL	2419	33.920	30.280	2.980	1.00	0.00
ATOM	8382	OW	SOL	2420	40.830	47.650	0.620	1.00	0.00
ATOM	8383	HW1	SOL	2420	39.990	48.000	0.310	1.00	0.00
ATOM	8384	HW2	SOL	2420	41.470	48.320	0.390	1.00	0.00
ATOM	8385	OW	SOL	2421	1.530	1.430	40.100	1.00	0.00
ATOM	8386	HW1	SOL	2421	2.130	1.120	39.430	1.00	0.00
ATOM	8387	HW2	SOL	2421	0.940	2.040	39.650	1.00	0.00
ATOM	8388	OW	SOL	2422	19.480	3.520	11.150	1.00	0.00
ATOM	8389	HW1	SOL	2422	19.470	4.310	10.610	1.00	0.00
ATOM	8390	HW2	SOL	2422	19.650	3.830	12.030	1.00	0.00
ATOM	8391	OW	SOL	2423	26.070	31.280	23.530	1.00	0.00
ATOM	8392	HW1	SOL	2423	25.160	31.380	23.790	1.00	0.00
ATOM	8393	HW2	SOL	2423	26.400	30.560	24.070	1.00	0.00
ATOM	8394	OW	SOL	2424	14.840	51.350	47.080	1.00	0.00
ATOM	8395	HW1	SOL	2424	14.510	52.110	47.550	1.00	0.00
ATOM	8396	HW2	SOL	2424	15.700	51.180	47.460	1.00	0.00
ATOM	8397	OW	SOL	2425	29.570	23.460	25.480	1.00	0.00
ATOM	8398	HW1	SOL	2425	29.110	24.170	25.020	1.00	0.00
ATOM	8399	HW2	SOL	2425	30.180	23.110	24.830	1.00	0.00
ATOM	8400	OW	SOL	2426	48.720	34.500	13.750	1.00	0.00
ATOM	8401	HW1	SOL	2426	48.490	33.720	13.250	1.00	0.00
ATOM	8402	HW2	SOL	2426	49.590	34.310	14.100	1.00	0.00
ATOM	8403	OW	SOL	2427	0.670	1.120	10.240	1.00	0.00

ATOM	8404	HW1	SOL	2427	0.430	1.140	9.310	1.00	0.00
ATOM	8405	HW2	SOL	2427	0.840	0.190	10.410	1.00	0.00
ATOM	8406	OW	SOL	2428	39.510	47.020	39.170	1.00	0.00
ATOM	8407	HW1	SOL	2428	40.300	46.480	39.080	1.00	0.00
ATOM	8408	HW2	SOL	2428	39.110	47.010	38.300	1.00	0.00
ATOM	8409	OW	SOL	2429	9.360	10.570	28.120	1.00	0.00
ATOM	8410	HW1	SOL	2429	8.850	11.370	27.990	1.00	0.00
ATOM	8411	HW2	SOL	2429	9.020	9.960	27.460	1.00	0.00
ATOM	8412	OW	SOL	2430	45.900	53.930	10.050	1.00	0.00
ATOM	8413	HW1	SOL	2430	45.050	54.250	10.350	1.00	0.00
ATOM	8414	HW2	SOL	2430	46.190	53.330	10.730	1.00	0.00
ATOM	8415	OW	SOL	2431	12.410	17.660	45.000	1.00	0.00
ATOM	8416	HW1	SOL	2431	13.210	17.790	44.500	1.00	0.00
ATOM	8417	HW2	SOL	2431	11.740	18.130	44.510	1.00	0.00
ATOM	8418	OW	SOL	2432	23.560	0.020	31.750	1.00	0.00
ATOM	8419	HW1	SOL	2432	23.960	-0.580	31.110	1.00	0.00
ATOM	8420	HW2	SOL	2432	24.150	0.770	31.770	1.00	0.00
ATOM	8421	OW	SOL	2433	20.800	12.610	14.440	1.00	0.00
ATOM	8422	HW1	SOL	2433	20.870	12.530	13.490	1.00	0.00
ATOM	8423	HW2	SOL	2433	19.980	12.160	14.650	1.00	0.00
ATOM	8424	OW	SOL	2434	36.230	11.070	52.720	1.00	0.00
ATOM	8425	HW1	SOL	2434	35.670	11.370	52.010	1.00	0.00
ATOM	8426	HW2	SOL	2434	36.310	10.130	52.590	1.00	0.00
ATOM	8427	OW	SOL	2435	6.210	2.330	29.460	1.00	0.00
ATOM	8428	HW1	SOL	2435	5.810	2.900	30.120	1.00	0.00
ATOM	8429	HW2	SOL	2435	7.150	2.360	29.650	1.00	0.00
ATOM	8430	OW	SOL	2436	5.020	13.730	53.620	1.00	0.00
ATOM	8431	HW1	SOL	2436	4.420	13.000	53.680	1.00	0.00
ATOM	8432	HW2	SOL	2436	5.860	13.380	53.930	1.00	0.00
ATOM	8433	OW	SOL	2437	37.090	46.230	18.350	1.00	0.00
ATOM	8434	HW1	SOL	2437	37.470	45.440	18.740	1.00	0.00
ATOM	8435	HW2	SOL	2437	37.530	46.950	18.790	1.00	0.00
ATOM	8436	OW	SOL	2438	28.250	8.350	14.580	1.00	0.00
ATOM	8437	HW1	SOL	2438	29.030	8.580	15.080	1.00	0.00
ATOM	8438	HW2	SOL	2438	28.480	7.550	14.110	1.00	0.00
ATOM	8439	OW	SOL	2439	4.050	41.250	52.760	1.00	0.00
ATOM	8440	HW1	SOL	2439	3.720	41.730	53.520	1.00	0.00
ATOM	8441	HW2	SOL	2439	4.550	41.890	52.260	1.00	0.00
ATOM	8442	OW	SOL	2440	3.840	28.170	0.270	1.00	0.00
ATOM	8443	HW1	SOL	2440	2.910	28.190	0.020	1.00	0.00
ATOM	8444	HW2	SOL	2440	3.850	28.550	1.150	1.00	0.00
ATOM	8445	OW	SOL	2441	39.230	55.590	52.940	1.00	0.00
ATOM	8446	HW1	SOL	2441	38.840	56.460	52.920	1.00	0.00
ATOM	8447	HW2	SOL	2441	38.700	55.080	52.330	1.00	0.00

ATOM	8448	OW	SOL	2442	27.030	10.310	3.970	1.00	0.00
ATOM	8449	HW1	SOL	2442	27.150	9.380	3.760	1.00	0.00
ATOM	8450	HW2	SOL	2442	27.830	10.730	3.640	1.00	0.00
ATOM	8451	OW	SOL	2443	2.710	35.260	46.710	1.00	0.00
ATOM	8452	HW1	SOL	2443	2.720	36.070	46.200	1.00	0.00
ATOM	8453	HW2	SOL	2443	2.000	34.740	46.330	1.00	0.00
ATOM	8454	OW	SOL	2444	40.820	44.540	44.910	1.00	0.00
ATOM	8455	HW1	SOL	2444	41.230	45.350	44.590	1.00	0.00
ATOM	8456	HW2	SOL	2444	40.440	44.150	44.120	1.00	0.00
ATOM	8457	OW	SOL	2445	53.870	14.120	1.390	1.00	0.00
ATOM	8458	HW1	SOL	2445	53.520	13.960	0.520	1.00	0.00
ATOM	8459	HW2	SOL	2445	53.690	15.050	1.550	1.00	0.00
ATOM	8460	OW	SOL	2446	46.760	21.880	19.440	1.00	0.00
ATOM	8461	HW1	SOL	2446	46.210	21.950	18.650	1.00	0.00
ATOM	8462	HW2	SOL	2446	46.220	22.250	20.130	1.00	0.00
ATOM	8463	OW	SOL	2447	24.050	5.370	47.320	1.00	0.00
ATOM	8464	HW1	SOL	2447	24.900	5.080	47.630	1.00	0.00
ATOM	8465	HW2	SOL	2447	23.730	5.960	48.010	1.00	0.00
ATOM	8466	OW	SOL	2448	36.050	52.000	12.030	1.00	0.00
ATOM	8467	HW1	SOL	2448	35.230	51.720	11.620	1.00	0.00
ATOM	8468	HW2	SOL	2448	35.770	52.480	12.810	1.00	0.00
ATOM	8469	OW	SOL	2449	11.920	7.400	0.230	1.00	0.00
ATOM	8470	HW1	SOL	2449	11.640	8.200	-0.210	1.00	0.00
ATOM	8471	HW2	SOL	2449	12.680	7.100	-0.270	1.00	0.00
ATOM	8472	OW	SOL	2450	53.510	15.260	34.360	1.00	0.00
ATOM	8473	HW1	SOL	2450	52.690	14.870	34.670	1.00	0.00
ATOM	8474	HW2	SOL	2450	53.580	16.080	34.850	1.00	0.00
ATOM	8475	OW	SOL	2451	21.580	54.030	2.060	1.00	0.00
ATOM	8476	HW1	SOL	2451	21.850	53.130	2.220	1.00	0.00
ATOM	8477	HW2	SOL	2451	20.810	54.150	2.620	1.00	0.00
ATOM	8478	OW	SOL	2452	12.020	12.520	32.990	1.00	0.00
ATOM	8479	HW1	SOL	2452	11.740	13.430	32.850	1.00	0.00
ATOM	8480	HW2	SOL	2452	11.840	12.080	32.160	1.00	0.00
ATOM	8481	OW	SOL	2453	10.010	44.700	18.370	1.00	0.00
ATOM	8482	HW1	SOL	2453	9.810	45.460	18.910	1.00	0.00
ATOM	8483	HW2	SOL	2453	10.920	44.840	18.080	1.00	0.00
ATOM	8484	OW	SOL	2454	19.390	20.670	34.790	1.00	0.00
ATOM	8485	HW1	SOL	2454	19.570	19.850	34.330	1.00	0.00
ATOM	8486	HW2	SOL	2454	19.180	20.400	35.690	1.00	0.00
ATOM	8487	OW	SOL	2455	39.750	15.470	52.780	1.00	0.00
ATOM	8488	HW1	SOL	2455	39.060	15.490	52.120	1.00	0.00
ATOM	8489	HW2	SOL	2455	39.340	15.070	53.550	1.00	0.00
ATOM	8490	OW	SOL	2456	6.270	28.440	5.390	1.00	0.00
ATOM	8491	HW1	SOL	2456	6.200	28.870	6.240	1.00	0.00

ATOM	8492	HW2	SOL	2456	7.150	28.060	5.370	1.00	0.00
ATOM	8493	OW	SOL	2457	3.870	48.610	25.770	1.00	0.00
ATOM	8494	HW1	SOL	2457	4.490	48.250	25.140	1.00	0.00
ATOM	8495	HW2	SOL	2457	3.260	49.120	25.240	1.00	0.00
ATOM	8496	OW	SOL	2458	8.510	8.010	35.350	1.00	0.00
ATOM	8497	HW1	SOL	2458	8.940	7.920	34.500	1.00	0.00
ATOM	8498	HW2	SOL	2458	8.680	7.180	35.790	1.00	0.00
ATOM	8499	OW	SOL	2459	9.450	51.720	35.380	1.00	0.00
ATOM	8500	HW1	SOL	2459	10.320	51.330	35.420	1.00	0.00
ATOM	8501	HW2	SOL	2459	9.020	51.440	36.190	1.00	0.00
ATOM	8502	OW	SOL	2460	3.680	12.890	10.600	1.00	0.00
ATOM	8503	HW1	SOL	2460	4.590	12.590	10.640	1.00	0.00
ATOM	8504	HW2	SOL	2460	3.390	12.670	9.710	1.00	0.00
ATOM	8505	OW	SOL	2461	28.440	3.430	12.230	1.00	0.00
ATOM	8506	HW1	SOL	2461	27.660	2.960	11.940	1.00	0.00
ATOM	8507	HW2	SOL	2461	29.100	2.750	12.360	1.00	0.00
ATOM	8508	OW	SOL	2462	4.820	39.800	28.530	1.00	0.00
ATOM	8509	HW1	SOL	2462	5.640	40.260	28.720	1.00	0.00
ATOM	8510	HW2	SOL	2462	4.420	40.310	27.830	1.00	0.00
ATOM	8511	OW	SOL	2463	19.630	22.640	16.840	1.00	0.00
ATOM	8512	HW1	SOL	2463	20.280	22.050	17.230	1.00	0.00
ATOM	8513	HW2	SOL	2463	18.880	22.590	17.430	1.00	0.00
ATOM	8514	OW	SOL	2464	26.470	5.840	27.190	1.00	0.00
ATOM	8515	HW1	SOL	2464	25.950	5.680	26.410	1.00	0.00
ATOM	8516	HW2	SOL	2464	27.350	5.530	26.960	1.00	0.00
ATOM	8517	OW	SOL	2465	13.900	37.120	2.770	1.00	0.00
ATOM	8518	HW1	SOL	2465	13.780	36.870	3.690	1.00	0.00
ATOM	8519	HW2	SOL	2465	14.070	38.060	2.790	1.00	0.00
ATOM	8520	OW	SOL	2466	35.840	7.680	44.030	1.00	0.00
ATOM	8521	HW1	SOL	2466	35.160	7.790	44.700	1.00	0.00
ATOM	8522	HW2	SOL	2466	36.260	8.540	43.980	1.00	0.00
ATOM	8523	OW	SOL	2467	31.820	7.800	36.000	1.00	0.00
ATOM	8524	HW1	SOL	2467	32.730	7.730	35.700	1.00	0.00
ATOM	8525	HW2	SOL	2467	31.640	8.740	35.970	1.00	0.00
ATOM	8526	OW	SOL	2468	45.120	31.110	9.080	1.00	0.00
ATOM	8527	HW1	SOL	2468	46.010	30.930	8.780	1.00	0.00
ATOM	8528	HW2	SOL	2468	44.560	30.800	8.370	1.00	0.00
ATOM	8529	OW	SOL	2469	2.670	24.000	51.390	1.00	0.00
ATOM	8530	HW1	SOL	2469	2.290	24.860	51.180	1.00	0.00
ATOM	8531	HW2	SOL	2469	2.340	23.800	52.260	1.00	0.00
ATOM	8532	OW	SOL	2470	50.790	21.030	50.400	1.00	0.00
ATOM	8533	HW1	SOL	2470	50.710	21.280	51.320	1.00	0.00
ATOM	8534	HW2	SOL	2470	51.550	20.440	50.370	1.00	0.00
ATOM	8535	OW	SOL	2471	5.580	2.070	40.260	1.00	0.00

ATOM	8536	HW1	SOL	2471	6.080	1.340	39.910	1.00	0.00
ATOM	8537	HW2	SOL	2471	5.280	2.550	39.490	1.00	0.00
ATOM	8538	OW	SOL	2472	41.770	22.870	19.920	1.00	0.00
ATOM	8539	HW1	SOL	2472	40.840	23.110	19.950	1.00	0.00
ATOM	8540	HW2	SOL	2472	42.010	22.760	20.840	1.00	0.00
ATOM	8541	OW	SOL	2473	18.490	50.660	14.350	1.00	0.00
ATOM	8542	HW1	SOL	2473	17.900	50.910	13.650	1.00	0.00
ATOM	8543	HW2	SOL	2473	19.210	50.210	13.910	1.00	0.00
ATOM	8544	OW	SOL	2474	22.020	9.930	47.090	1.00	0.00
ATOM	8545	HW1	SOL	2474	22.490	10.560	46.530	1.00	0.00
ATOM	8546	HW2	SOL	2474	22.350	10.100	47.970	1.00	0.00
ATOM	8547	OW	SOL	2475	32.460	37.840	32.310	1.00	0.00
ATOM	8548	HW1	SOL	2475	32.490	37.990	31.360	1.00	0.00
ATOM	8549	HW2	SOL	2475	32.950	37.020	32.430	1.00	0.00
ATOM	8550	OW	SOL	2476	17.220	6.730	16.080	1.00	0.00
ATOM	8551	HW1	SOL	2476	17.370	6.280	16.910	1.00	0.00
ATOM	8552	HW2	SOL	2476	16.270	6.750	15.980	1.00	0.00
ATOM	8553	OW	SOL	2477	34.770	29.250	25.770	1.00	0.00
ATOM	8554	HW1	SOL	2477	34.830	29.620	26.650	1.00	0.00
ATOM	8555	HW2	SOL	2477	33.830	29.140	25.620	1.00	0.00
ATOM	8556	OW	SOL	2478	30.780	53.360	47.630	1.00	0.00
ATOM	8557	HW1	SOL	2478	31.120	53.690	46.800	1.00	0.00
ATOM	8558	HW2	SOL	2478	31.110	53.970	48.290	1.00	0.00
ATOM	8559	OW	SOL	2479	51.580	21.560	26.900	1.00	0.00
ATOM	8560	HW1	SOL	2479	50.750	21.400	26.460	1.00	0.00
ATOM	8561	HW2	SOL	2479	52.240	21.460	26.210	1.00	0.00
ATOM	8562	OW	SOL	2480	7.710	54.860	33.260	1.00	0.00
ATOM	8563	HW1	SOL	2480	7.860	55.260	32.400	1.00	0.00
ATOM	8564	HW2	SOL	2480	7.030	55.400	33.650	1.00	0.00
ATOM	8565	OW	SOL	2481	34.930	42.260	15.720	1.00	0.00
ATOM	8566	HW1	SOL	2481	34.590	41.800	16.480	1.00	0.00
ATOM	8567	HW2	SOL	2481	35.370	43.030	16.070	1.00	0.00
ATOM	8568	OW	SOL	2482	3.130	9.800	35.090	1.00	0.00
ATOM	8569	HW1	SOL	2482	2.700	8.990	34.800	1.00	0.00
ATOM	8570	HW2	SOL	2482	4.060	9.660	34.880	1.00	0.00
ATOM	8571	OW	SOL	2483	43.490	15.380	46.770	1.00	0.00
ATOM	8572	HW1	SOL	2483	44.220	14.780	46.620	1.00	0.00
ATOM	8573	HW2	SOL	2483	43.900	16.160	47.150	1.00	0.00
ATOM	8574	OW	SOL	2484	46.370	50.690	7.080	1.00	0.00
ATOM	8575	HW1	SOL	2484	45.580	50.650	6.550	1.00	0.00
ATOM	8576	HW2	SOL	2484	46.800	49.840	6.950	1.00	0.00
ATOM	8577	OW	SOL	2485	2.200	36.140	50.070	1.00	0.00
ATOM	8578	HW1	SOL	2485	1.260	35.930	50.130	1.00	0.00
ATOM	8579	HW2	SOL	2485	2.420	36.500	50.930	1.00	0.00

ATOM	8580	OW	SOL	2486	5.770	52.670	19.720	1.00	0.00
ATOM	8581	HW1	SOL	2486	5.910	53.120	18.890	1.00	0.00
ATOM	8582	HW2	SOL	2486	5.060	53.150	20.150	1.00	0.00
ATOM	8583	OW	SOL	2487	33.840	21.800	15.520	1.00	0.00
ATOM	8584	HW1	SOL	2487	33.820	20.870	15.280	1.00	0.00
ATOM	8585	HW2	SOL	2487	34.290	21.820	16.360	1.00	0.00
ATOM	8586	OW	SOL	2488	44.330	54.550	12.570	1.00	0.00
ATOM	8587	HW1	SOL	2488	44.780	53.770	12.920	1.00	0.00
ATOM	8588	HW2	SOL	2488	43.440	54.250	12.410	1.00	0.00
ATOM	8589	OW	SOL	2489	36.880	52.090	16.220	1.00	0.00
ATOM	8590	HW1	SOL	2489	37.110	52.520	17.050	1.00	0.00
ATOM	8591	HW2	SOL	2489	37.720	51.850	15.830	1.00	0.00
ATOM	8592	OW	SOL	2490	17.370	18.910	39.960	1.00	0.00
ATOM	8593	HW1	SOL	2490	16.450	18.670	39.900	1.00	0.00
ATOM	8594	HW2	SOL	2490	17.760	18.210	40.490	1.00	0.00
ATOM	8595	OW	SOL	2491	1.840	13.600	39.190	1.00	0.00
ATOM	8596	HW1	SOL	2491	1.590	13.830	40.090	1.00	0.00
ATOM	8597	HW2	SOL	2491	2.280	14.380	38.860	1.00	0.00
ATOM	8598	OW	SOL	2492	15.070	4.360	29.300	1.00	0.00
ATOM	8599	HW1	SOL	2492	14.270	4.840	29.490	1.00	0.00
ATOM	8600	HW2	SOL	2492	14.780	3.460	29.110	1.00	0.00
ATOM	8601	OW	SOL	2493	15.080	43.200	30.060	1.00	0.00
ATOM	8602	HW1	SOL	2493	16.030	43.240	29.950	1.00	0.00
ATOM	8603	HW2	SOL	2493	14.940	42.690	30.860	1.00	0.00
ATOM	8604	OW	SOL	2494	18.050	46.670	44.100	1.00	0.00
ATOM	8605	HW1	SOL	2494	17.660	47.530	44.240	1.00	0.00
ATOM	8606	HW2	SOL	2494	18.280	46.370	44.980	1.00	0.00
ATOM	8607	OW	SOL	2495	42.640	32.830	10.030	1.00	0.00
ATOM	8608	HW1	SOL	2495	42.740	32.470	9.150	1.00	0.00
ATOM	8609	HW2	SOL	2495	41.900	32.350	10.400	1.00	0.00
ATOM	8610	OW	SOL	2496	32.080	50.980	24.550	1.00	0.00
ATOM	8611	HW1	SOL	2496	33.010	51.150	24.420	1.00	0.00
ATOM	8612	HW2	SOL	2496	31.900	51.290	25.440	1.00	0.00
ATOM	8613	OW	SOL	2497	31.890	16.670	7.870	1.00	0.00
ATOM	8614	HW1	SOL	2497	31.860	17.500	7.410	1.00	0.00
ATOM	8615	HW2	SOL	2497	32.260	16.050	7.240	1.00	0.00
ATOM	8616	OW	SOL	2498	10.270	34.210	0.050	1.00	0.00
ATOM	8617	HW1	SOL	2498	10.830	34.810	0.540	1.00	0.00
ATOM	8618	HW2	SOL	2498	9.970	34.730	-0.700	1.00	0.00
ATOM	8619	OW	SOL	2499	50.800	12.390	13.860	1.00	0.00
ATOM	8620	HW1	SOL	2499	50.120	12.240	13.200	1.00	0.00
ATOM	8621	HW2	SOL	2499	50.570	11.810	14.580	1.00	0.00
ATOM	8622	OW	SOL	2500	24.310	12.970	46.980	1.00	0.00
ATOM	8623	HW1	SOL	2500	23.930	13.620	46.380	1.00	0.00

ATOM	8624	HW2	SOL	2500	25.110	13.390	47.310	1.00	0.00
ATOM	8625	OW	SOL	2501	43.990	49.820	38.690	1.00	0.00
ATOM	8626	HW1	SOL	2501	43.660	49.090	38.170	1.00	0.00
ATOM	8627	HW2	SOL	2501	43.800	50.590	38.170	1.00	0.00
ATOM	8628	OW	SOL	2502	26.420	46.570	34.370	1.00	0.00
ATOM	8629	HW1	SOL	2502	26.060	46.420	33.490	1.00	0.00
ATOM	8630	HW2	SOL	2502	27.190	46.010	34.410	1.00	0.00
ATOM	8631	OW	SOL	2503	14.320	53.330	4.150	1.00	0.00
ATOM	8632	HW1	SOL	2503	14.590	53.950	4.820	1.00	0.00
ATOM	8633	HW2	SOL	2503	15.120	52.840	3.940	1.00	0.00
ATOM	8634	OW	SOL	2504	50.030	52.890	25.730	1.00	0.00
ATOM	8635	HW1	SOL	2504	49.730	53.690	25.310	1.00	0.00
ATOM	8636	HW2	SOL	2504	49.230	52.390	25.900	1.00	0.00
ATOM	8637	OW	SOL	2505	54.070	55.470	40.890	1.00	0.00
ATOM	8638	HW1	SOL	2505	53.480	55.720	40.180	1.00	0.00
ATOM	8639	HW2	SOL	2505	54.850	55.150	40.450	1.00	0.00
ATOM	8640	OW	SOL	2506	55.290	1.780	48.070	1.00	0.00
ATOM	8641	HW1	SOL	2506	54.670	1.140	47.720	1.00	0.00
ATOM	8642	HW2	SOL	2506	55.450	2.380	47.340	1.00	0.00
ATOM	8643	OW	SOL	2507	29.520	21.220	50.250	1.00	0.00
ATOM	8644	HW1	SOL	2507	30.080	20.890	50.950	1.00	0.00
ATOM	8645	HW2	SOL	2507	28.990	21.900	50.670	1.00	0.00
ATOM	8646	OW	SOL	2508	9.830	39.280	22.460	1.00	0.00
ATOM	8647	HW1	SOL	2508	9.910	39.720	23.310	1.00	0.00
ATOM	8648	HW2	SOL	2508	9.170	38.610	22.600	1.00	0.00
ATOM	8649	OW	SOL	2509	13.020	35.880	42.660	1.00	0.00
ATOM	8650	HW1	SOL	2509	12.250	35.560	43.140	1.00	0.00
ATOM	8651	HW2	SOL	2509	13.480	35.080	42.390	1.00	0.00
ATOM	8652	OW	SOL	2510	42.080	13.040	28.940	1.00	0.00
ATOM	8653	HW1	SOL	2510	41.230	13.150	29.360	1.00	0.00
ATOM	8654	HW2	SOL	2510	42.180	13.840	28.420	1.00	0.00
ATOM	8655	OW	SOL	2511	8.870	19.060	10.180	1.00	0.00
ATOM	8656	HW1	SOL	2511	9.550	18.500	9.800	1.00	0.00
ATOM	8657	HW2	SOL	2511	8.500	18.540	10.890	1.00	0.00
ATOM	8658	OW	SOL	2512	30.970	35.600	5.750	1.00	0.00
ATOM	8659	HW1	SOL	2512	30.880	36.470	5.380	1.00	0.00
ATOM	8660	HW2	SOL	2512	31.120	35.020	5.000	1.00	0.00
ATOM	8661	OW	SOL	2513	54.480	48.280	52.370	1.00	0.00
ATOM	8662	HW1	SOL	2513	53.800	48.860	52.050	1.00	0.00
ATOM	8663	HW2	SOL	2513	54.980	48.050	51.580	1.00	0.00
ATOM	8664	OW	SOL	2514	22.680	10.880	10.760	1.00	0.00
ATOM	8665	HW1	SOL	2514	22.990	10.960	11.670	1.00	0.00
ATOM	8666	HW2	SOL	2514	23.430	11.140	10.230	1.00	0.00
ATOM	8667	OW	SOL	2515	2.280	27.440	42.820	1.00	0.00

ATOM	8668	HW1	SOL	2515	3.030	27.750	42.310	1.00	0.00
ATOM	8669	HW2	SOL	2515	1.560	28.030	42.570	1.00	0.00
ATOM	8670	OW	SOL	2516	0.420	45.910	15.970	1.00	0.00
ATOM	8671	HW1	SOL	2516	1.230	45.580	15.590	1.00	0.00
ATOM	8672	HW2	SOL	2516	-0.050	46.310	15.230	1.00	0.00
ATOM	8673	OW	SOL	2517	24.910	45.230	3.990	1.00	0.00
ATOM	8674	HW1	SOL	2517	24.800	46.170	4.160	1.00	0.00
ATOM	8675	HW2	SOL	2517	25.570	45.190	3.300	1.00	0.00
ATOM	8676	OW	SOL	2518	8.270	2.030	31.550	1.00	0.00
ATOM	8677	HW1	SOL	2518	9.180	2.340	31.530	1.00	0.00
ATOM	8678	HW2	SOL	2518	8.240	1.320	30.920	1.00	0.00
ATOM	8679	OW	SOL	2519	39.550	38.760	54.060	1.00	0.00
ATOM	8680	HW1	SOL	2519	38.760	38.780	53.530	1.00	0.00
ATOM	8681	HW2	SOL	2519	40.210	38.370	53.490	1.00	0.00
ATOM	8682	OW	SOL	2520	32.990	44.820	7.300	1.00	0.00
ATOM	8683	HW1	SOL	2520	32.760	44.060	7.840	1.00	0.00
ATOM	8684	HW2	SOL	2520	33.790	44.550	6.830	1.00	0.00
ATOM	8685	OW	SOL	2521	21.880	6.760	22.630	1.00	0.00
ATOM	8686	HW1	SOL	2521	21.530	7.330	23.310	1.00	0.00
ATOM	8687	HW2	SOL	2521	21.890	5.890	23.030	1.00	0.00
ATOM	8688	OW	SOL	2522	40.950	29.200	16.400	1.00	0.00
ATOM	8689	HW1	SOL	2522	40.740	28.810	17.250	1.00	0.00
ATOM	8690	HW2	SOL	2522	41.680	28.680	16.070	1.00	0.00
ATOM	8691	OW	SOL	2523	43.430	7.490	1.050	1.00	0.00
ATOM	8692	HW1	SOL	2523	43.760	8.370	1.230	1.00	0.00
ATOM	8693	HW2	SOL	2523	44.130	7.060	0.560	1.00	0.00
ATOM	8694	OW	SOL	2524	39.390	47.590	7.070	1.00	0.00
ATOM	8695	HW1	SOL	2524	38.520	47.830	7.360	1.00	0.00
ATOM	8696	HW2	SOL	2524	39.300	46.690	6.760	1.00	0.00
ATOM	8697	OW	SOL	2525	17.500	51.180	42.090	1.00	0.00
ATOM	8698	HW1	SOL	2525	17.880	50.420	42.520	1.00	0.00
ATOM	8699	HW2	SOL	2525	17.590	51.000	41.150	1.00	0.00
ATOM	8700	OW	SOL	2526	0.290	43.860	13.900	1.00	0.00
ATOM	8701	HW1	SOL	2526	-0.250	44.290	13.240	1.00	0.00
ATOM	8702	HW2	SOL	2526	-0.330	43.380	14.440	1.00	0.00
ATOM	8703	OW	SOL	2527	29.470	36.370	47.740	1.00	0.00
ATOM	8704	HW1	SOL	2527	29.740	35.450	47.810	1.00	0.00
ATOM	8705	HW2	SOL	2527	28.820	36.480	48.430	1.00	0.00
ATOM	8706	OW	SOL	2528	44.890	8.310	41.800	1.00	0.00
ATOM	8707	HW1	SOL	2528	44.080	8.650	42.180	1.00	0.00
ATOM	8708	HW2	SOL	2528	45.000	7.450	42.190	1.00	0.00
ATOM	8709	OW	SOL	2529	26.670	13.920	17.190	1.00	0.00
ATOM	8710	HW1	SOL	2529	27.010	14.300	18.000	1.00	0.00
ATOM	8711	HW2	SOL	2529	25.730	14.130	17.200	1.00	0.00

ATOM	8712	OW	SOL	2530	10.610	33.140	42.990	1.00	0.00
ATOM	8713	HW1	SOL	2530	10.900	33.990	43.320	1.00	0.00
ATOM	8714	HW2	SOL	2530	10.070	33.350	42.230	1.00	0.00
ATOM	8715	OW	SOL	2531	22.580	28.860	52.330	1.00	0.00
ATOM	8716	HW1	SOL	2531	21.690	28.520	52.250	1.00	0.00
ATOM	8717	HW2	SOL	2531	23.140	28.110	52.110	1.00	0.00
ATOM	8718	OW	SOL	2532	45.490	51.770	34.430	1.00	0.00
ATOM	8719	HW1	SOL	2532	44.600	51.640	34.110	1.00	0.00
ATOM	8720	HW2	SOL	2532	46.000	51.970	33.650	1.00	0.00
ATOM	8721	OW	SOL	2533	7.740	0.570	38.970	1.00	0.00
ATOM	8722	HW1	SOL	2533	7.770	1.140	38.200	1.00	0.00
ATOM	8723	HW2	SOL	2533	8.090	-0.270	38.650	1.00	0.00
ATOM	8724	OW	SOL	2534	17.600	55.480	8.230	1.00	0.00
ATOM	8725	HW1	SOL	2534	17.740	56.350	8.590	1.00	0.00
ATOM	8726	HW2	SOL	2534	17.130	55.000	8.910	1.00	0.00
ATOM	8727	OW	SOL	2535	21.230	29.390	4.450	1.00	0.00
ATOM	8728	HW1	SOL	2535	21.380	29.080	3.550	1.00	0.00
ATOM	8729	HW2	SOL	2535	20.440	29.920	4.390	1.00	0.00
ATOM	8730	OW	SOL	2536	10.730	46.670	1.200	1.00	0.00
ATOM	8731	HW1	SOL	2536	11.220	46.350	0.440	1.00	0.00
ATOM	8732	HW2	SOL	2536	11.360	46.630	1.930	1.00	0.00
ATOM	8733	OW	SOL	2537	53.030	18.050	38.910	1.00	0.00
ATOM	8734	HW1	SOL	2537	53.070	18.750	38.250	1.00	0.00
ATOM	8735	HW2	SOL	2537	52.390	18.370	39.550	1.00	0.00
ATOM	8736	OW	SOL	2538	29.460	32.220	54.510	1.00	0.00
ATOM	8737	HW1	SOL	2538	28.580	31.860	54.410	1.00	0.00
ATOM	8738	HW2	SOL	2538	29.650	32.610	53.660	1.00	0.00
ATOM	8739	OW	SOL	2539	55.410	52.010	23.090	1.00	0.00
ATOM	8740	HW1	SOL	2539	55.070	52.260	23.950	1.00	0.00
ATOM	8741	HW2	SOL	2539	55.190	51.090	23.000	1.00	0.00
ATOM	8742	OW	SOL	2540	17.340	28.890	33.380	1.00	0.00
ATOM	8743	HW1	SOL	2540	17.560	28.480	32.550	1.00	0.00
ATOM	8744	HW2	SOL	2540	18.000	29.570	33.500	1.00	0.00
ATOM	8745	OW	SOL	2541	11.070	5.130	13.800	1.00	0.00
ATOM	8746	HW1	SOL	2541	11.140	4.200	13.560	1.00	0.00
ATOM	8747	HW2	SOL	2541	11.360	5.160	14.720	1.00	0.00
ATOM	8748	OW	SOL	2542	12.000	9.290	37.740	1.00	0.00
ATOM	8749	HW1	SOL	2542	12.400	8.850	38.490	1.00	0.00
ATOM	8750	HW2	SOL	2542	11.060	9.180	37.860	1.00	0.00
ATOM	8751	OW	SOL	2543	2.170	28.130	23.870	1.00	0.00
ATOM	8752	HW1	SOL	2543	2.060	29.070	23.950	1.00	0.00
ATOM	8753	HW2	SOL	2543	2.080	27.800	24.770	1.00	0.00
ATOM	8754	OW	SOL	2544	35.360	13.940	32.620	1.00	0.00
ATOM	8755	HW1	SOL	2544	35.130	13.610	33.490	1.00	0.00

ATOM	8756	HW2	SOL	2544	35.330	13.170	32.060	1.00	0.00
ATOM	8757	OW	SOL	2545	3.740	46.110	22.420	1.00	0.00
ATOM	8758	HW1	SOL	2545	3.140	46.760	22.070	1.00	0.00
ATOM	8759	HW2	SOL	2545	4.590	46.540	22.410	1.00	0.00
ATOM	8760	OW	SOL	2546	54.020	50.280	12.950	1.00	0.00
ATOM	8761	HW1	SOL	2546	54.140	49.540	13.540	1.00	0.00
ATOM	8762	HW2	SOL	2546	53.790	51.010	13.520	1.00	0.00
ATOM	8763	OW	SOL	2547	17.370	14.910	12.860	1.00	0.00
ATOM	8764	HW1	SOL	2547	18.200	15.260	13.200	1.00	0.00
ATOM	8765	HW2	SOL	2547	16.700	15.270	13.440	1.00	0.00
ATOM	8766	OW	SOL	2548	32.160	50.430	2.660	1.00	0.00
ATOM	8767	HW1	SOL	2548	32.280	51.110	3.320	1.00	0.00
ATOM	8768	HW2	SOL	2548	32.090	50.920	1.830	1.00	0.00
ATOM	8769	OW	SOL	2549	8.880	34.620	15.520	1.00	0.00
ATOM	8770	HW1	SOL	2549	9.660	34.070	15.390	1.00	0.00
ATOM	8771	HW2	SOL	2549	8.210	34.200	14.990	1.00	0.00
ATOM	8772	OW	SOL	2550	6.300	38.510	2.810	1.00	0.00
ATOM	8773	HW1	SOL	2550	6.100	38.440	1.880	1.00	0.00
ATOM	8774	HW2	SOL	2550	6.940	39.210	2.860	1.00	0.00
ATOM	8775	OW	SOL	2551	21.860	6.810	49.920	1.00	0.00
ATOM	8776	HW1	SOL	2551	21.640	7.200	49.070	1.00	0.00
ATOM	8777	HW2	SOL	2551	21.140	6.210	50.100	1.00	0.00
ATOM	8778	OW	SOL	2552	55.260	53.740	55.880	1.00	0.00
ATOM	8779	HW1	SOL	2552	55.890	54.290	55.400	1.00	0.00
ATOM	8780	HW2	SOL	2552	55.770	53.370	56.600	1.00	0.00
ATOM	8781	OW	SOL	2553	49.930	14.400	23.360	1.00	0.00
ATOM	8782	HW1	SOL	2553	49.140	13.870	23.500	1.00	0.00
ATOM	8783	HW2	SOL	2553	50.330	14.460	24.230	1.00	0.00
ATOM	8784	OW	SOL	2554	17.150	48.760	30.500	1.00	0.00
ATOM	8785	HW1	SOL	2554	17.940	48.380	30.880	1.00	0.00
ATOM	8786	HW2	SOL	2554	17.390	48.970	29.600	1.00	0.00
ATOM	8787	OW	SOL	2555	49.920	17.280	15.900	1.00	0.00
ATOM	8788	HW1	SOL	2555	49.320	17.860	16.390	1.00	0.00
ATOM	8789	HW2	SOL	2555	49.350	16.620	15.520	1.00	0.00
ATOM	8790	OW	SOL	2556	54.750	4.800	45.020	1.00	0.00
ATOM	8791	HW1	SOL	2556	55.300	4.840	44.240	1.00	0.00
ATOM	8792	HW2	SOL	2556	53.950	5.260	44.770	1.00	0.00
ATOM	8793	OW	SOL	2557	7.830	21.660	22.720	1.00	0.00
ATOM	8794	HW1	SOL	2557	7.850	20.700	22.720	1.00	0.00
ATOM	8795	HW2	SOL	2557	8.500	21.910	23.360	1.00	0.00
ATOM	8796	OW	SOL	2558	27.890	22.560	5.990	1.00	0.00
ATOM	8797	HW1	SOL	2558	26.990	22.630	5.670	1.00	0.00
ATOM	8798	HW2	SOL	2558	28.100	21.630	5.880	1.00	0.00
ATOM	8799	OW	SOL	2559	46.330	14.440	37.120	1.00	0.00

ATOM	8800	HW1	SOL	2559	45.740	13.890	36.600	1.00	0.00
ATOM	8801	HW2	SOL	2559	45.850	14.640	37.920	1.00	0.00
ATOM	8802	OW	SOL	2560	28.530	24.740	13.290	1.00	0.00
ATOM	8803	HW1	SOL	2560	29.110	24.680	12.530	1.00	0.00
ATOM	8804	HW2	SOL	2560	28.130	25.600	13.220	1.00	0.00
ATOM	8805	OW	SOL	2561	12.990	40.040	17.940	1.00	0.00
ATOM	8806	HW1	SOL	2561	12.910	40.360	18.830	1.00	0.00
ATOM	8807	HW2	SOL	2561	13.750	39.460	17.960	1.00	0.00
ATOM	8808	OW	SOL	2562	34.750	12.750	35.040	1.00	0.00
ATOM	8809	HW1	SOL	2562	34.920	11.860	35.360	1.00	0.00
ATOM	8810	HW2	SOL	2562	34.680	13.280	35.840	1.00	0.00
ATOM	8811	OW	SOL	2563	38.250	24.820	52.820	1.00	0.00
ATOM	8812	HW1	SOL	2563	38.900	24.120	52.840	1.00	0.00
ATOM	8813	HW2	SOL	2563	37.790	24.750	53.660	1.00	0.00
ATOM	8814	OW	SOL	2564	48.910	51.680	17.910	1.00	0.00
ATOM	8815	HW1	SOL	2564	48.470	51.240	17.180	1.00	0.00
ATOM	8816	HW2	SOL	2564	49.020	52.580	17.600	1.00	0.00
ATOM	8817	OW	SOL	2565	41.090	6.540	14.820	1.00	0.00
ATOM	8818	HW1	SOL	2565	41.400	6.930	14.000	1.00	0.00
ATOM	8819	HW2	SOL	2565	40.290	6.080	14.580	1.00	0.00
ATOM	8820	OW	SOL	2566	47.010	20.170	51.670	1.00	0.00
ATOM	8821	HW1	SOL	2566	47.350	20.450	50.820	1.00	0.00
ATOM	8822	HW2	SOL	2566	46.880	20.990	52.150	1.00	0.00
ATOM	8823	OW	SOL	2567	35.020	25.980	45.580	1.00	0.00
ATOM	8824	HW1	SOL	2567	35.110	25.620	46.460	1.00	0.00
ATOM	8825	HW2	SOL	2567	35.300	26.890	45.660	1.00	0.00
ATOM	8826	OW	SOL	2568	42.050	21.950	48.750	1.00	0.00
ATOM	8827	HW1	SOL	2568	42.830	21.900	49.300	1.00	0.00
ATOM	8828	HW2	SOL	2568	41.710	22.830	48.880	1.00	0.00
ATOM	8829	OW	SOL	2569	28.890	26.210	46.400	1.00	0.00
ATOM	8830	HW1	SOL	2569	28.080	26.160	45.900	1.00	0.00
ATOM	8831	HW2	SOL	2569	29.190	25.300	46.460	1.00	0.00
ATOM	8832	OW	SOL	2570	45.220	38.030	40.480	1.00	0.00
ATOM	8833	HW1	SOL	2570	44.800	38.360	41.270	1.00	0.00
ATOM	8834	HW2	SOL	2570	46.020	38.540	40.400	1.00	0.00
ATOM	8835	OW	SOL	2571	27.580	36.010	11.030	1.00	0.00
ATOM	8836	HW1	SOL	2571	28.400	36.480	10.880	1.00	0.00
ATOM	8837	HW2	SOL	2571	26.980	36.680	11.360	1.00	0.00
ATOM	8838	OW	SOL	2572	3.180	12.960	0.690	1.00	0.00
ATOM	8839	HW1	SOL	2572	3.920	12.360	0.780	1.00	0.00
ATOM	8840	HW2	SOL	2572	2.530	12.480	0.180	1.00	0.00
ATOM	8841	OW	SOL	2573	29.230	36.210	26.810	1.00	0.00
ATOM	8842	HW1	SOL	2573	28.470	36.360	27.370	1.00	0.00
ATOM	8843	HW2	SOL	2573	29.000	35.440	26.300	1.00	0.00

ATOM	8844	OW	SOL	2574	51.810	15.150	13.560	1.00	0.00
ATOM	8845	HW1	SOL	2574	51.780	15.340	12.620	1.00	0.00
ATOM	8846	HW2	SOL	2574	51.570	14.230	13.630	1.00	0.00
ATOM	8847	OW	SOL	2575	1.100	17.650	7.920	1.00	0.00
ATOM	8848	HW1	SOL	2575	1.630	17.790	8.700	1.00	0.00
ATOM	8849	HW2	SOL	2575	1.220	18.450	7.400	1.00	0.00
ATOM	8850	OW	SOL	2576	11.420	23.720	52.550	1.00	0.00
ATOM	8851	HW1	SOL	2576	11.200	22.920	52.080	1.00	0.00
ATOM	8852	HW2	SOL	2576	11.910	23.420	53.310	1.00	0.00
ATOM	8853	OW	SOL	2577	49.480	46.190	35.350	1.00	0.00
ATOM	8854	HW1	SOL	2577	49.740	46.110	34.430	1.00	0.00
ATOM	8855	HW2	SOL	2577	48.580	45.870	35.370	1.00	0.00
ATOM	8856	OW	SOL	2578	32.340	2.190	9.650	1.00	0.00
ATOM	8857	HW1	SOL	2578	31.820	2.680	9.000	1.00	0.00
ATOM	8858	HW2	SOL	2578	33.190	2.070	9.230	1.00	0.00
ATOM	8859	OW	SOL	2579	47.570	31.820	7.810	1.00	0.00
ATOM	8860	HW1	SOL	2579	47.360	31.510	6.930	1.00	0.00
ATOM	8861	HW2	SOL	2579	48.450	31.490	7.970	1.00	0.00
ATOM	8862	OW	SOL	2580	35.770	24.960	31.220	1.00	0.00
ATOM	8863	HW1	SOL	2580	35.650	25.710	31.810	1.00	0.00
ATOM	8864	HW2	SOL	2580	35.150	25.120	30.500	1.00	0.00
ATOM	8865	OW	SOL	2581	34.200	5.540	55.820	1.00	0.00
ATOM	8866	HW1	SOL	2581	33.780	6.340	56.130	1.00	0.00
ATOM	8867	HW2	SOL	2581	33.780	5.360	54.980	1.00	0.00
ATOM	8868	OW	SOL	2582	41.280	39.890	45.640	1.00	0.00
ATOM	8869	HW1	SOL	2582	41.150	39.300	46.380	1.00	0.00
ATOM	8870	HW2	SOL	2582	41.860	39.400	45.050	1.00	0.00
ATOM	8871	OW	SOL	2583	54.730	38.850	30.470	1.00	0.00
ATOM	8872	HW1	SOL	2583	55.190	39.220	31.220	1.00	0.00
ATOM	8873	HW2	SOL	2583	54.140	38.200	30.840	1.00	0.00
ATOM	8874	OW	SOL	2584	45.470	51.660	44.080	1.00	0.00
ATOM	8875	HW1	SOL	2584	44.820	51.430	44.750	1.00	0.00
ATOM	8876	HW2	SOL	2584	45.280	51.070	43.350	1.00	0.00
ATOM	8877	OW	SOL	2585	23.570	37.340	50.500	1.00	0.00
ATOM	8878	HW1	SOL	2585	22.680	37.440	50.840	1.00	0.00
ATOM	8879	HW2	SOL	2585	23.510	37.620	49.580	1.00	0.00
ATOM	8880	OW	SOL	2586	8.310	17.340	38.700	1.00	0.00
ATOM	8881	HW1	SOL	2586	8.960	16.880	38.170	1.00	0.00
ATOM	8882	HW2	SOL	2586	7.700	17.720	38.070	1.00	0.00
ATOM	8883	OW	SOL	2587	25.210	43.960	11.570	1.00	0.00
ATOM	8884	HW1	SOL	2587	24.800	44.510	10.900	1.00	0.00
ATOM	8885	HW2	SOL	2587	24.500	43.390	11.870	1.00	0.00
ATOM	8886	OW	SOL	2588	16.170	33.370	14.990	1.00	0.00
ATOM	8887	HW1	SOL	2588	15.350	33.490	15.480	1.00	0.00

ATOM	8888	HW2	SOL	2588	16.770	34.020	15.370	1.00	0.00
ATOM	8889	OW	SOL	2589	52.820	38.340	10.750	1.00	0.00
ATOM	8890	HW1	SOL	2589	53.200	39.170	10.450	1.00	0.00
ATOM	8891	HW2	SOL	2589	53.230	38.180	11.600	1.00	0.00
ATOM	8892	OW	SOL	2590	26.280	37.330	17.710	1.00	0.00
ATOM	8893	HW1	SOL	2590	25.710	38.080	17.870	1.00	0.00
ATOM	8894	HW2	SOL	2590	26.720	37.180	18.550	1.00	0.00
ATOM	8895	OW	SOL	2591	4.580	46.060	49.700	1.00	0.00
ATOM	8896	HW1	SOL	2591	4.970	45.210	49.490	1.00	0.00
ATOM	8897	HW2	SOL	2591	5.340	46.650	49.790	1.00	0.00
ATOM	8898	OW	SOL	2592	51.380	26.610	18.960	1.00	0.00
ATOM	8899	HW1	SOL	2592	51.320	26.630	19.920	1.00	0.00
ATOM	8900	HW2	SOL	2592	50.710	27.230	18.670	1.00	0.00
ATOM	8901	OW	SOL	2593	40.890	10.570	4.040	1.00	0.00
ATOM	8902	HW1	SOL	2593	40.930	11.510	3.860	1.00	0.00
ATOM	8903	HW2	SOL	2593	41.650	10.200	3.600	1.00	0.00
ATOM	8904	OW	SOL	2594	23.480	51.290	52.330	1.00	0.00
ATOM	8905	HW1	SOL	2594	22.650	51.270	51.840	1.00	0.00
ATOM	8906	HW2	SOL	2594	23.340	51.950	53.000	1.00	0.00
ATOM	8907	OW	SOL	2595	29.950	34.970	55.390	1.00	0.00
ATOM	8908	HW1	SOL	2595	30.420	34.410	56.010	1.00	0.00
ATOM	8909	HW2	SOL	2595	30.030	34.520	54.550	1.00	0.00
ATOM	8910	OW	SOL	2596	14.170	47.150	53.640	1.00	0.00
ATOM	8911	HW1	SOL	2596	13.440	46.770	53.150	1.00	0.00
ATOM	8912	HW2	SOL	2596	14.050	48.100	53.540	1.00	0.00
ATOM	8913	OW	SOL	2597	8.560	40.210	55.500	1.00	0.00
ATOM	8914	HW1	SOL	2597	9.130	40.680	56.110	1.00	0.00
ATOM	8915	HW2	SOL	2597	9.050	39.420	55.270	1.00	0.00
ATOM	8916	OW	SOL	2598	50.720	34.960	44.700	1.00	0.00
ATOM	8917	HW1	SOL	2598	51.630	34.750	44.930	1.00	0.00
ATOM	8918	HW2	SOL	2598	50.770	35.850	44.350	1.00	0.00
ATOM	8919	OW	SOL	2599	10.910	40.530	52.340	1.00	0.00
ATOM	8920	HW1	SOL	2599	10.020	40.870	52.280	1.00	0.00
ATOM	8921	HW2	SOL	2599	10.920	40.000	53.130	1.00	0.00
ATOM	8922	OW	SOL	2600	47.110	0.850	22.570	1.00	0.00
ATOM	8923	HW1	SOL	2600	48.050	0.810	22.370	1.00	0.00
ATOM	8924	HW2	SOL	2600	47.050	1.450	23.320	1.00	0.00
ATOM	8925	OW	SOL	2601	3.000	26.010	22.190	1.00	0.00
ATOM	8926	HW1	SOL	2601	2.440	26.750	22.420	1.00	0.00
ATOM	8927	HW2	SOL	2601	3.210	25.590	23.020	1.00	0.00
ATOM	8928	OW	SOL	2602	49.510	32.730	5.980	1.00	0.00
ATOM	8929	HW1	SOL	2602	49.760	33.630	5.730	1.00	0.00
ATOM	8930	HW2	SOL	2602	49.980	32.570	6.800	1.00	0.00
ATOM	8931	OW	SOL	2603	6.180	2.800	33.510	1.00	0.00

ATOM	8932	HW1	SOL	2603	6.500	2.220	34.200	1.00	0.00
ATOM	8933	HW2	SOL	2603	6.790	2.660	32.780	1.00	0.00
ATOM	8934	OW	SOL	2604	54.690	17.780	45.430	1.00	0.00
ATOM	8935	HW1	SOL	2604	54.100	18.230	44.820	1.00	0.00
ATOM	8936	HW2	SOL	2604	55.050	17.060	44.920	1.00	0.00
ATOM	8937	OW	SOL	2605	5.590	30.940	41.320	1.00	0.00
ATOM	8938	HW1	SOL	2605	6.080	31.650	41.730	1.00	0.00
ATOM	8939	HW2	SOL	2605	6.130	30.660	40.580	1.00	0.00
ATOM	8940	OW	SOL	2606	0.910	44.430	36.160	1.00	0.00
ATOM	8941	HW1	SOL	2606	1.720	44.820	35.830	1.00	0.00
ATOM	8942	HW2	SOL	2606	0.260	45.120	36.060	1.00	0.00
ATOM	8943	OW	SOL	2607	39.540	47.280	50.430	1.00	0.00
ATOM	8944	HW1	SOL	2607	39.840	47.750	51.210	1.00	0.00
ATOM	8945	HW2	SOL	2607	40.270	46.700	50.210	1.00	0.00
ATOM	8946	OW	SOL	2608	9.530	3.050	40.300	1.00	0.00
ATOM	8947	HW1	SOL	2608	10.360	2.810	40.700	1.00	0.00
ATOM	8948	HW2	SOL	2608	9.180	3.740	40.870	1.00	0.00
ATOM	8949	OW	SOL	2609	3.660	5.740	42.580	1.00	0.00
ATOM	8950	HW1	SOL	2609	4.030	5.530	43.430	1.00	0.00
ATOM	8951	HW2	SOL	2609	3.510	4.890	42.160	1.00	0.00
ATOM	8952	OW	SOL	2610	45.540	12.750	1.710	1.00	0.00
ATOM	8953	HW1	SOL	2610	46.150	12.050	1.920	1.00	0.00
ATOM	8954	HW2	SOL	2610	46.080	13.540	1.670	1.00	0.00
ATOM	8955	OW	SOL	2611	4.460	28.140	33.520	1.00	0.00
ATOM	8956	HW1	SOL	2611	5.120	28.830	33.650	1.00	0.00
ATOM	8957	HW2	SOL	2611	4.220	27.890	34.410	1.00	0.00
ATOM	8958	OW	SOL	2612	32.750	30.370	52.140	1.00	0.00
ATOM	8959	HW1	SOL	2612	33.660	30.070	52.050	1.00	0.00
ATOM	8960	HW2	SOL	2612	32.630	30.980	51.410	1.00	0.00
ATOM	8961	OW	SOL	2613	43.170	38.210	44.320	1.00	0.00
ATOM	8962	HW1	SOL	2613	43.790	38.820	43.930	1.00	0.00
ATOM	8963	HW2	SOL	2613	43.530	37.340	44.140	1.00	0.00
ATOM	8964	OW	SOL	2614	8.940	0.490	41.560	1.00	0.00
ATOM	8965	HW1	SOL	2614	8.640	1.330	41.900	1.00	0.00
ATOM	8966	HW2	SOL	2614	8.520	0.410	40.710	1.00	0.00
ATOM	8967	OW	SOL	2615	10.680	46.860	53.370	1.00	0.00
ATOM	8968	HW1	SOL	2615	11.190	46.640	52.590	1.00	0.00
ATOM	8969	HW2	SOL	2615	10.690	46.050	53.890	1.00	0.00
ATOM	8970	OW	SOL	2616	44.450	32.980	52.440	1.00	0.00
ATOM	8971	HW1	SOL	2616	44.330	33.860	52.810	1.00	0.00
ATOM	8972	HW2	SOL	2616	43.950	32.400	53.030	1.00	0.00
ATOM	8973	OW	SOL	2617	54.940	46.870	38.570	1.00	0.00
ATOM	8974	HW1	SOL	2617	54.370	46.190	38.190	1.00	0.00
ATOM	8975	HW2	SOL	2617	55.600	46.370	39.050	1.00	0.00

ATOM	8976	OW	SOL	2618	2.180	22.330	27.860	1.00	0.00
ATOM	8977	HW1	SOL	2618	1.790	21.920	28.620	1.00	0.00
ATOM	8978	HW2	SOL	2618	2.610	21.600	27.400	1.00	0.00
ATOM	8979	OW	SOL	2619	36.230	36.780	43.050	1.00	0.00
ATOM	8980	HW1	SOL	2619	35.620	36.290	43.600	1.00	0.00
ATOM	8981	HW2	SOL	2619	36.530	37.500	43.600	1.00	0.00
ATOM	8982	OW	SOL	2620	15.780	30.730	20.660	1.00	0.00
ATOM	8983	HW1	SOL	2620	15.060	31.280	20.990	1.00	0.00
ATOM	8984	HW2	SOL	2620	15.980	31.100	19.800	1.00	0.00
ATOM	8985	OW	SOL	2621	30.700	41.630	16.280	1.00	0.00
ATOM	8986	HW1	SOL	2621	30.740	40.750	15.910	1.00	0.00
ATOM	8987	HW2	SOL	2621	29.840	41.660	16.700	1.00	0.00
ATOM	8988	OW	SOL	2622	36.670	0.670	35.660	1.00	0.00
ATOM	8989	HW1	SOL	2622	37.270	0.970	36.340	1.00	0.00
ATOM	8990	HW2	SOL	2622	35.900	1.240	35.750	1.00	0.00
ATOM	8991	OW	SOL	2623	8.780	33.070	2.970	1.00	0.00
ATOM	8992	HW1	SOL	2623	8.950	33.480	3.820	1.00	0.00
ATOM	8993	HW2	SOL	2623	9.510	32.450	2.860	1.00	0.00
ATOM	8994	OW	SOL	2624	20.630	17.120	52.880	1.00	0.00
ATOM	8995	HW1	SOL	2624	21.350	17.670	52.570	1.00	0.00
ATOM	8996	HW2	SOL	2624	20.930	16.220	52.750	1.00	0.00
ATOM	8997	OW	SOL	2625	11.000	15.540	7.810	1.00	0.00
ATOM	8998	HW1	SOL	2625	11.440	15.200	7.030	1.00	0.00
ATOM	8999	HW2	SOL	2625	11.020	14.820	8.430	1.00	0.00
ATOM	9000	OW	SOL	2626	17.950	18.170	23.820	1.00	0.00
ATOM	9001	HW1	SOL	2626	18.540	18.810	23.420	1.00	0.00
ATOM	9002	HW2	SOL	2626	18.160	18.200	24.750	1.00	0.00
ATOM	9003	OW	SOL	2627	21.260	36.730	29.020	1.00	0.00
ATOM	9004	HW1	SOL	2627	22.080	36.550	29.490	1.00	0.00
ATOM	9005	HW2	SOL	2627	21.200	37.680	28.980	1.00	0.00
ATOM	9006	OW	SOL	2628	52.880	25.740	15.720	1.00	0.00
ATOM	9007	HW1	SOL	2628	53.400	24.980	15.450	1.00	0.00
ATOM	9008	HW2	SOL	2628	52.280	25.400	16.390	1.00	0.00
ATOM	9009	OW	SOL	2629	54.480	1.230	30.420	1.00	0.00
ATOM	9010	HW1	SOL	2629	55.070	0.530	30.120	1.00	0.00
ATOM	9011	HW2	SOL	2629	53.920	0.800	31.070	1.00	0.00
ATOM	9012	OW	SOL	2630	37.300	16.660	41.810	1.00	0.00
ATOM	9013	HW1	SOL	2630	36.460	17.070	42.010	1.00	0.00
ATOM	9014	HW2	SOL	2630	37.910	17.020	42.460	1.00	0.00
ATOM	9015	OW	SOL	2631	4.160	49.260	4.940	1.00	0.00
ATOM	9016	HW1	SOL	2631	4.170	48.830	4.090	1.00	0.00
ATOM	9017	HW2	SOL	2631	4.580	50.110	4.800	1.00	0.00
ATOM	9018	OW	SOL	2632	29.970	13.330	8.240	1.00	0.00
ATOM	9019	HW1	SOL	2632	29.860	14.280	8.230	1.00	0.00

ATOM	9020	HW2	SOL	2632	30.910	13.190	8.200	1.00	0.00
ATOM	9021	OW	SOL	2633	39.850	10.110	48.910	1.00	0.00
ATOM	9022	HW1	SOL	2633	38.990	9.850	49.250	1.00	0.00
ATOM	9023	HW2	SOL	2633	40.350	10.350	49.680	1.00	0.00
ATOM	9024	OW	SOL	2634	46.670	24.130	47.280	1.00	0.00
ATOM	9025	HW1	SOL	2634	46.550	23.280	47.720	1.00	0.00
ATOM	9026	HW2	SOL	2634	46.660	24.770	48.000	1.00	0.00
ATOM	9027	OW	SOL	2635	11.350	34.520	24.690	1.00	0.00
ATOM	9028	HW1	SOL	2635	11.960	35.260	24.660	1.00	0.00
ATOM	9029	HW2	SOL	2635	11.900	33.770	24.900	1.00	0.00
ATOM	9030	OW	SOL	2636	15.130	16.010	31.520	1.00	0.00
ATOM	9031	HW1	SOL	2636	16.050	16.190	31.720	1.00	0.00
ATOM	9032	HW2	SOL	2636	15.100	15.050	31.430	1.00	0.00
ATOM	9033	OW	SOL	2637	11.280	2.070	36.600	1.00	0.00
ATOM	9034	HW1	SOL	2637	11.090	1.230	37.010	1.00	0.00
ATOM	9035	HW2	SOL	2637	12.230	2.090	36.500	1.00	0.00
ATOM	9036	OW	SOL	2638	16.840	9.440	46.840	1.00	0.00
ATOM	9037	HW1	SOL	2638	17.600	9.640	46.300	1.00	0.00
ATOM	9038	HW2	SOL	2638	16.940	10.010	47.610	1.00	0.00
ATOM	9039	OW	SOL	2639	18.400	30.860	47.250	1.00	0.00
ATOM	9040	HW1	SOL	2639	19.030	31.570	47.390	1.00	0.00
ATOM	9041	HW2	SOL	2639	17.930	30.780	48.080	1.00	0.00
ATOM	9042	OW	SOL	2640	21.900	44.720	8.110	1.00	0.00
ATOM	9043	HW1	SOL	2640	22.420	43.980	7.800	1.00	0.00
ATOM	9044	HW2	SOL	2640	21.870	45.320	7.360	1.00	0.00
ATOM	9045	OW	SOL	2641	5.440	42.750	27.910	1.00	0.00
ATOM	9046	HW1	SOL	2641	4.540	42.530	28.150	1.00	0.00
ATOM	9047	HW2	SOL	2641	5.400	43.680	27.650	1.00	0.00
ATOM	9048	OW	SOL	2642	28.760	45.070	34.480	1.00	0.00
ATOM	9049	HW1	SOL	2642	29.010	45.260	33.570	1.00	0.00
ATOM	9050	HW2	SOL	2642	29.550	44.680	34.870	1.00	0.00
ATOM	9051	OW	SOL	2643	0.300	2.470	4.920	1.00	0.00
ATOM	9052	HW1	SOL	2643	0.840	3.190	5.260	1.00	0.00
ATOM	9053	HW2	SOL	2643	0.930	1.780	4.700	1.00	0.00
ATOM	9054	OW	SOL	2644	54.890	31.860	19.240	1.00	0.00
ATOM	9055	HW1	SOL	2644	54.980	32.700	19.670	1.00	0.00
ATOM	9056	HW2	SOL	2644	54.310	31.350	19.810	1.00	0.00
ATOM	9057	OW	SOL	2645	42.180	11.060	55.400	1.00	0.00
ATOM	9058	HW1	SOL	2645	41.250	10.980	55.230	1.00	0.00
ATOM	9059	HW2	SOL	2645	42.590	11.010	54.540	1.00	0.00
ATOM	9060	OW	SOL	2646	35.000	14.640	20.950	1.00	0.00
ATOM	9061	HW1	SOL	2646	34.530	13.960	20.470	1.00	0.00
ATOM	9062	HW2	SOL	2646	34.310	15.210	21.300	1.00	0.00
ATOM	9063	OW	SOL	2647	19.770	18.020	14.270	1.00	0.00

ATOM	9064	HW1	SOL	2647	19.210	18.660	14.700	1.00	0.00
ATOM	9065	HW2	SOL	2647	19.410	17.170	14.510	1.00	0.00
ATOM	9066	OW	SOL	2648	1.490	49.230	40.870	1.00	0.00
ATOM	9067	HW1	SOL	2648	1.590	48.850	40.000	1.00	0.00
ATOM	9068	HW2	SOL	2648	1.430	50.170	40.720	1.00	0.00
ATOM	9069	OW	SOL	2649	20.870	30.640	6.900	1.00	0.00
ATOM	9070	HW1	SOL	2649	21.110	30.220	6.070	1.00	0.00
ATOM	9071	HW2	SOL	2649	21.700	30.980	7.240	1.00	0.00
ATOM	9072	OW	SOL	2650	40.310	19.670	5.960	1.00	0.00
ATOM	9073	HW1	SOL	2650	39.850	18.950	6.380	1.00	0.00
ATOM	9074	HW2	SOL	2650	39.810	19.860	5.170	1.00	0.00
ATOM	9075	OW	SOL	2651	38.730	3.640	43.830	1.00	0.00
ATOM	9076	HW1	SOL	2651	39.670	3.750	44.020	1.00	0.00
ATOM	9077	HW2	SOL	2651	38.590	2.700	43.890	1.00	0.00
ATOM	9078	OW	SOL	2652	29.850	41.310	10.360	1.00	0.00
ATOM	9079	HW1	SOL	2652	29.430	42.140	10.600	1.00	0.00
ATOM	9080	HW2	SOL	2652	29.310	40.970	9.650	1.00	0.00
ATOM	9081	OW	SOL	2653	28.480	48.820	6.880	1.00	0.00
ATOM	9082	HW1	SOL	2653	28.650	49.320	6.080	1.00	0.00
ATOM	9083	HW2	SOL	2653	29.210	48.200	6.930	1.00	0.00
ATOM	9084	OW	SOL	2654	22.810	20.320	3.460	1.00	0.00
ATOM	9085	HW1	SOL	2654	22.200	19.710	3.870	1.00	0.00
ATOM	9086	HW2	SOL	2654	22.270	21.050	3.170	1.00	0.00
ATOM	9087	OW	SOL	2655	10.870	27.050	34.750	1.00	0.00
ATOM	9088	HW1	SOL	2655	11.030	27.870	34.290	1.00	0.00
ATOM	9089	HW2	SOL	2655	11.200	26.370	34.160	1.00	0.00
ATOM	9090	OW	SOL	2656	54.450	0.790	11.920	1.00	0.00
ATOM	9091	HW1	SOL	2656	54.570	0.430	12.800	1.00	0.00
ATOM	9092	HW2	SOL	2656	55.340	0.990	11.620	1.00	0.00
ATOM	9093	OW	SOL	2657	1.760	31.270	28.250	1.00	0.00
ATOM	9094	HW1	SOL	2657	2.650	31.600	28.070	1.00	0.00
ATOM	9095	HW2	SOL	2657	1.750	31.120	29.190	1.00	0.00
ATOM	9096	OW	SOL	2658	45.870	43.330	28.150	1.00	0.00
ATOM	9097	HW1	SOL	2658	45.930	43.660	27.250	1.00	0.00
ATOM	9098	HW2	SOL	2658	46.580	42.690	28.210	1.00	0.00
ATOM	9099	OW	SOL	2659	39.640	11.880	37.480	1.00	0.00
ATOM	9100	HW1	SOL	2659	39.580	12.390	38.280	1.00	0.00
ATOM	9101	HW2	SOL	2659	40.500	11.460	37.520	1.00	0.00
ATOM	9102	OW	SOL	2660	35.050	15.580	30.640	1.00	0.00
ATOM	9103	HW1	SOL	2660	35.150	15.070	31.440	1.00	0.00
ATOM	9104	HW2	SOL	2660	35.210	14.940	29.940	1.00	0.00
ATOM	9105	OW	SOL	2661	23.700	48.670	7.610	1.00	0.00
ATOM	9106	HW1	SOL	2661	23.820	48.540	8.550	1.00	0.00
ATOM	9107	HW2	SOL	2661	23.180	47.920	7.330	1.00	0.00

ATOM	9108	OW	SOL	2662	35.450	18.070	43.140	1.00	0.00
ATOM	9109	HW1	SOL	2662	36.210	18.240	43.710	1.00	0.00
ATOM	9110	HW2	SOL	2662	35.130	17.210	43.420	1.00	0.00
ATOM	9111	OW	SOL	2663	54.170	33.650	55.330	1.00	0.00
ATOM	9112	HW1	SOL	2663	53.530	33.610	56.040	1.00	0.00
ATOM	9113	HW2	SOL	2663	53.950	34.450	54.860	1.00	0.00
ATOM	9114	OW	SOL	2664	44.690	46.970	15.450	1.00	0.00
ATOM	9115	HW1	SOL	2664	43.790	47.120	15.770	1.00	0.00
ATOM	9116	HW2	SOL	2664	44.890	46.070	15.710	1.00	0.00
ATOM	9117	OW	SOL	2665	43.860	28.960	0.480	1.00	0.00
ATOM	9118	HW1	SOL	2665	44.700	28.650	0.830	1.00	0.00
ATOM	9119	HW2	SOL	2665	43.270	28.930	1.230	1.00	0.00
ATOM	9120	OW	SOL	2666	54.660	3.390	2.720	1.00	0.00
ATOM	9121	HW1	SOL	2666	54.880	3.390	3.660	1.00	0.00
ATOM	9122	HW2	SOL	2666	54.370	4.290	2.550	1.00	0.00
ATOM	9123	OW	SOL	2667	53.850	25.140	29.300	1.00	0.00
ATOM	9124	HW1	SOL	2667	54.530	25.600	28.820	1.00	0.00
ATOM	9125	HW2	SOL	2667	54.320	24.620	29.950	1.00	0.00
ATOM	9126	OW	SOL	2668	32.480	21.410	1.200	1.00	0.00
ATOM	9127	HW1	SOL	2668	32.300	21.250	0.270	1.00	0.00
ATOM	9128	HW2	SOL	2668	32.460	22.360	1.290	1.00	0.00
ATOM	9129	OW	SOL	2669	7.580	4.680	10.300	1.00	0.00
ATOM	9130	HW1	SOL	2669	7.000	5.400	10.560	1.00	0.00
ATOM	9131	HW2	SOL	2669	7.060	4.180	9.670	1.00	0.00
ATOM	9132	OW	SOL	2670	13.010	20.250	37.730	1.00	0.00
ATOM	9133	HW1	SOL	2670	12.080	20.010	37.750	1.00	0.00
ATOM	9134	HW2	SOL	2670	13.010	21.200	37.650	1.00	0.00
ATOM	9135	OW	SOL	2671	2.740	2.650	36.450	1.00	0.00
ATOM	9136	HW1	SOL	2671	1.960	3.210	36.400	1.00	0.00
ATOM	9137	HW2	SOL	2671	2.640	2.040	35.730	1.00	0.00
ATOM	9138	OW	SOL	2672	5.940	33.900	45.780	1.00	0.00
ATOM	9139	HW1	SOL	2672	5.790	34.840	45.920	1.00	0.00
ATOM	9140	HW2	SOL	2672	6.880	33.790	45.890	1.00	0.00
ATOM	9141	OW	SOL	2673	15.650	15.100	40.810	1.00	0.00
ATOM	9142	HW1	SOL	2673	16.410	15.500	40.380	1.00	0.00
ATOM	9143	HW2	SOL	2673	15.980	14.830	41.670	1.00	0.00
ATOM	9144	OW	SOL	2674	46.270	14.260	45.410	1.00	0.00
ATOM	9145	HW1	SOL	2674	45.740	13.540	45.760	1.00	0.00
ATOM	9146	HW2	SOL	2674	46.100	14.250	44.470	1.00	0.00
ATOM	9147	OW	SOL	2675	5.870	36.930	45.920	1.00	0.00
ATOM	9148	HW1	SOL	2675	5.500	37.560	46.530	1.00	0.00
ATOM	9149	HW2	SOL	2675	6.530	37.430	45.430	1.00	0.00
ATOM	9150	OW	SOL	2676	48.400	45.760	16.230	1.00	0.00
ATOM	9151	HW1	SOL	2676	49.130	45.420	16.750	1.00	0.00

ATOM	9152	HW2	SOL	2676	48.560	46.700	16.180	1.00	0.00
ATOM	9153	OW	SOL	2677	49.520	28.430	17.640	1.00	0.00
ATOM	9154	HW1	SOL	2677	49.040	29.260	17.610	1.00	0.00
ATOM	9155	HW2	SOL	2677	48.850	27.760	17.710	1.00	0.00
ATOM	9156	OW	SOL	2678	34.910	53.310	4.920	1.00	0.00
ATOM	9157	HW1	SOL	2678	35.720	53.650	5.300	1.00	0.00
ATOM	9158	HW2	SOL	2678	34.650	53.970	4.270	1.00	0.00
ATOM	9159	OW	SOL	2679	54.380	31.020	1.860	1.00	0.00
ATOM	9160	HW1	SOL	2679	55.240	31.270	2.210	1.00	0.00
ATOM	9161	HW2	SOL	2679	53.850	31.810	1.950	1.00	0.00
ATOM	9162	OW	SOL	2680	8.750	51.360	22.140	1.00	0.00
ATOM	9163	HW1	SOL	2680	7.940	51.850	22.260	1.00	0.00
ATOM	9164	HW2	SOL	2680	9.430	52.040	22.130	1.00	0.00
ATOM	9165	OW	SOL	2681	6.430	23.150	12.750	1.00	0.00
ATOM	9166	HW1	SOL	2681	5.660	23.120	13.320	1.00	0.00
ATOM	9167	HW2	SOL	2681	6.200	23.770	12.060	1.00	0.00
ATOM	9168	OW	SOL	2682	38.860	23.210	20.800	1.00	0.00
ATOM	9169	HW1	SOL	2682	38.740	22.270	20.690	1.00	0.00
ATOM	9170	HW2	SOL	2682	38.050	23.600	20.490	1.00	0.00
ATOM	9171	OW	SOL	2683	53.010	32.500	13.640	1.00	0.00
ATOM	9172	HW1	SOL	2683	53.390	32.270	14.490	1.00	0.00
ATOM	9173	HW2	SOL	2683	53.750	32.790	13.110	1.00	0.00
ATOM	9174	OW	SOL	2684	20.570	22.880	11.950	1.00	0.00
ATOM	9175	HW1	SOL	2684	20.880	22.520	12.780	1.00	0.00
ATOM	9176	HW2	SOL	2684	21.370	23.080	11.470	1.00	0.00
ATOM	9177	OW	SOL	2685	31.910	1.420	27.090	1.00	0.00
ATOM	9178	HW1	SOL	2685	32.730	1.350	26.600	1.00	0.00
ATOM	9179	HW2	SOL	2685	31.420	0.630	26.850	1.00	0.00
ATOM	9180	OW	SOL	2686	32.270	36.940	9.100	1.00	0.00
ATOM	9181	HW1	SOL	2686	32.770	37.350	8.400	1.00	0.00
ATOM	9182	HW2	SOL	2686	32.450	36.000	9.010	1.00	0.00
ATOM	9183	OW	SOL	2687	32.560	18.520	55.310	1.00	0.00
ATOM	9184	HW1	SOL	2687	31.730	18.200	54.930	1.00	0.00
ATOM	9185	HW2	SOL	2687	32.330	18.780	56.200	1.00	0.00
ATOM	9186	OW	SOL	2688	50.180	44.860	40.960	1.00	0.00
ATOM	9187	HW1	SOL	2688	51.030	44.410	40.900	1.00	0.00
ATOM	9188	HW2	SOL	2688	50.410	45.780	41.040	1.00	0.00
ATOM	9189	OW	SOL	2689	49.690	15.210	55.590	1.00	0.00
ATOM	9190	HW1	SOL	2689	48.800	14.880	55.440	1.00	0.00
ATOM	9191	HW2	SOL	2689	50.240	14.420	55.620	1.00	0.00
ATOM	9192	OW	SOL	2690	11.140	42.840	42.580	1.00	0.00
ATOM	9193	HW1	SOL	2690	11.290	43.690	42.160	1.00	0.00
ATOM	9194	HW2	SOL	2690	11.660	42.220	42.070	1.00	0.00
ATOM	9195	OW	SOL	2691	53.570	4.900	22.630	1.00	0.00

ATOM	9196	HW1	SOL	2691	53.700	4.200	21.990	1.00	0.00
ATOM	9197	HW2	SOL	2691	52.910	5.470	22.230	1.00	0.00
ATOM	9198	OW	SOL	2692	43.630	43.730	41.820	1.00	0.00
ATOM	9199	HW1	SOL	2692	44.350	44.000	41.250	1.00	0.00
ATOM	9200	HW2	SOL	2692	42.860	43.710	41.250	1.00	0.00
ATOM	9201	OW	SOL	2693	10.850	26.050	15.440	1.00	0.00
ATOM	9202	HW1	SOL	2693	10.060	26.230	14.930	1.00	0.00
ATOM	9203	HW2	SOL	2693	11.570	26.250	14.840	1.00	0.00
ATOM	9204	OW	SOL	2694	21.200	43.630	47.740	1.00	0.00
ATOM	9205	HW1	SOL	2694	21.990	43.300	48.160	1.00	0.00
ATOM	9206	HW2	SOL	2694	21.100	43.080	46.960	1.00	0.00
ATOM	9207	OW	SOL	2695	55.510	43.600	53.240	1.00	0.00
ATOM	9208	HW1	SOL	2695	54.920	44.340	53.360	1.00	0.00
ATOM	9209	HW2	SOL	2695	55.030	42.990	52.680	1.00	0.00
ATOM	9210	OW	SOL	2696	51.250	11.340	33.130	1.00	0.00
ATOM	9211	HW1	SOL	2696	51.630	11.890	32.440	1.00	0.00
ATOM	9212	HW2	SOL	2696	50.720	11.950	33.650	1.00	0.00
ATOM	9213	OW	SOL	2697	52.010	30.980	11.400	1.00	0.00
ATOM	9214	HW1	SOL	2697	52.490	30.160	11.530	1.00	0.00
ATOM	9215	HW2	SOL	2697	52.190	31.490	12.190	1.00	0.00
ATOM	9216	OW	SOL	2698	37.000	29.800	8.420	1.00	0.00
ATOM	9217	HW1	SOL	2698	36.220	30.100	7.950	1.00	0.00
ATOM	9218	HW2	SOL	2698	37.130	30.460	9.100	1.00	0.00
ATOM	9219	OW	SOL	2699	44.580	17.400	3.070	1.00	0.00
ATOM	9220	HW1	SOL	2699	44.400	17.820	3.910	1.00	0.00
ATOM	9221	HW2	SOL	2699	43.910	17.740	2.480	1.00	0.00
ATOM	9222	OW	SOL	2700	40.460	30.860	9.800	1.00	0.00
ATOM	9223	HW1	SOL	2700	40.890	30.010	9.680	1.00	0.00
ATOM	9224	HW2	SOL	2700	40.640	31.330	8.980	1.00	0.00
ATOM	9225	OW	SOL	2701	12.890	16.520	47.310	1.00	0.00
ATOM	9226	HW1	SOL	2701	13.740	16.860	47.590	1.00	0.00
ATOM	9227	HW2	SOL	2701	12.690	17.010	46.510	1.00	0.00
ATOM	9228	OW	SOL	2702	17.960	2.270	1.350	1.00	0.00
ATOM	9229	HW1	SOL	2702	18.810	1.860	1.230	1.00	0.00
ATOM	9230	HW2	SOL	2702	18.140	3.210	1.310	1.00	0.00
ATOM	9231	OW	SOL	2703	30.060	27.570	19.040	1.00	0.00
ATOM	9232	HW1	SOL	2703	29.610	26.770	18.780	1.00	0.00
ATOM	9233	HW2	SOL	2703	29.360	28.220	19.160	1.00	0.00
ATOM	9234	OW	SOL	2704	42.480	11.160	48.200	1.00	0.00
ATOM	9235	HW1	SOL	2704	42.990	10.550	48.740	1.00	0.00
ATOM	9236	HW2	SOL	2704	41.580	10.880	48.320	1.00	0.00
ATOM	9237	OW	SOL	2705	32.540	22.690	38.440	1.00	0.00
ATOM	9238	HW1	SOL	2705	31.690	22.990	38.750	1.00	0.00
ATOM	9239	HW2	SOL	2705	32.590	21.770	38.700	1.00	0.00

ATOM	9240	OW	SOL	2706	45.520	6.440	30.830	1.00	0.00
ATOM	9241	HW1	SOL	2706	45.320	7.340	31.100	1.00	0.00
ATOM	9242	HW2	SOL	2706	45.980	6.540	29.990	1.00	0.00
ATOM	9243	OW	SOL	2707	40.550	1.020	13.110	1.00	0.00
ATOM	9244	HW1	SOL	2707	40.890	1.820	12.720	1.00	0.00
ATOM	9245	HW2	SOL	2707	40.490	0.410	12.370	1.00	0.00
ATOM	9246	OW	SOL	2708	18.110	33.280	37.710	1.00	0.00
ATOM	9247	HW1	SOL	2708	18.540	32.710	38.350	1.00	0.00
ATOM	9248	HW2	SOL	2708	18.680	33.260	36.940	1.00	0.00
ATOM	9249	OW	SOL	2709	25.530	43.350	50.840	1.00	0.00
ATOM	9250	HW1	SOL	2709	25.590	42.880	51.670	1.00	0.00
ATOM	9251	HW2	SOL	2709	24.820	43.980	50.970	1.00	0.00
ATOM	9252	OW	SOL	2710	36.560	5.360	23.330	1.00	0.00
ATOM	9253	HW1	SOL	2710	35.950	5.840	22.780	1.00	0.00
ATOM	9254	HW2	SOL	2710	37.320	5.200	22.760	1.00	0.00
ATOM	9255	OW	SOL	2711	13.310	47.080	20.610	1.00	0.00
ATOM	9256	HW1	SOL	2711	13.650	46.400	20.030	1.00	0.00
ATOM	9257	HW2	SOL	2711	12.560	47.440	20.150	1.00	0.00
ATOM	9258	OW	SOL	2712	14.670	9.940	54.100	1.00	0.00
ATOM	9259	HW1	SOL	2712	14.200	9.300	54.650	1.00	0.00
ATOM	9260	HW2	SOL	2712	15.530	10.010	54.500	1.00	0.00
ATOM	9261	OW	SOL	2713	1.720	37.080	23.050	1.00	0.00
ATOM	9262	HW1	SOL	2713	2.440	37.100	23.670	1.00	0.00
ATOM	9263	HW2	SOL	2713	1.360	36.200	23.120	1.00	0.00
ATOM	9264	OW	SOL	2714	36.150	55.780	43.210	1.00	0.00
ATOM	9265	HW1	SOL	2714	35.630	55.320	42.550	1.00	0.00
ATOM	9266	HW2	SOL	2714	35.600	56.510	43.480	1.00	0.00
ATOM	9267	OW	SOL	2715	14.850	45.330	18.960	1.00	0.00
ATOM	9268	HW1	SOL	2715	15.550	45.080	19.550	1.00	0.00
ATOM	9269	HW2	SOL	2715	15.070	46.220	18.680	1.00	0.00
ATOM	9270	OW	SOL	2716	4.730	28.560	3.200	1.00	0.00
ATOM	9271	HW1	SOL	2716	3.790	28.430	3.310	1.00	0.00
ATOM	9272	HW2	SOL	2716	5.110	28.350	4.050	1.00	0.00
ATOM	9273	OW	SOL	2717	17.490	36.160	2.980	1.00	0.00
ATOM	9274	HW1	SOL	2717	18.080	35.820	3.650	1.00	0.00
ATOM	9275	HW2	SOL	2717	17.630	35.590	2.220	1.00	0.00
ATOM	9276	OW	SOL	2718	16.750	45.910	52.640	1.00	0.00
ATOM	9277	HW1	SOL	2718	16.160	46.310	53.280	1.00	0.00
ATOM	9278	HW2	SOL	2718	16.330	45.090	52.400	1.00	0.00
ATOM	9279	OW	SOL	2719	31.320	52.270	27.010	1.00	0.00
ATOM	9280	HW1	SOL	2719	30.700	51.570	27.230	1.00	0.00
ATOM	9281	HW2	SOL	2719	32.150	51.980	27.390	1.00	0.00
ATOM	9282	OW	SOL	2720	2.910	33.100	50.400	1.00	0.00
ATOM	9283	HW1	SOL	2720	2.880	34.040	50.220	1.00	0.00

ATOM	9284	HW2	SOL	2720	3.070	32.700	49.550	1.00	0.00
ATOM	9285	OW	SOL	2721	39.440	12.980	44.700	1.00	0.00
ATOM	9286	HW1	SOL	2721	40.230	13.530	44.750	1.00	0.00
ATOM	9287	HW2	SOL	2721	39.620	12.370	43.990	1.00	0.00
ATOM	9288	OW	SOL	2722	13.710	9.040	30.370	1.00	0.00
ATOM	9289	HW1	SOL	2722	14.210	8.230	30.330	1.00	0.00
ATOM	9290	HW2	SOL	2722	13.690	9.350	29.460	1.00	0.00
ATOM	9291	OW	SOL	2723	17.450	26.010	24.780	1.00	0.00
ATOM	9292	HW1	SOL	2723	17.610	25.990	23.840	1.00	0.00
ATOM	9293	HW2	SOL	2723	17.780	26.860	25.060	1.00	0.00
ATOM	9294	OW	SOL	2724	4.490	23.350	49.740	1.00	0.00
ATOM	9295	HW1	SOL	2724	4.050	22.750	49.150	1.00	0.00
ATOM	9296	HW2	SOL	2724	3.810	23.630	50.350	1.00	0.00
ATOM	9297	OW	SOL	2725	28.950	49.830	37.080	1.00	0.00
ATOM	9298	HW1	SOL	2725	28.310	49.870	37.790	1.00	0.00
ATOM	9299	HW2	SOL	2725	29.620	50.470	37.330	1.00	0.00
ATOM	9300	OW	SOL	2726	27.680	8.980	38.540	1.00	0.00
ATOM	9301	HW1	SOL	2726	28.640	8.900	38.500	1.00	0.00
ATOM	9302	HW2	SOL	2726	27.400	8.850	37.630	1.00	0.00
ATOM	9303	OW	SOL	2727	21.940	29.060	38.320	1.00	0.00
ATOM	9304	HW1	SOL	2727	22.560	29.140	39.050	1.00	0.00
ATOM	9305	HW2	SOL	2727	22.020	28.140	38.050	1.00	0.00
ATOM	9306	OW	SOL	2728	22.990	31.780	27.050	1.00	0.00
ATOM	9307	HW1	SOL	2728	23.870	31.840	27.410	1.00	0.00
ATOM	9308	HW2	SOL	2728	23.120	31.650	26.110	1.00	0.00
ATOM	9309	OW	SOL	2729	2.860	36.870	52.670	1.00	0.00
ATOM	9310	HW1	SOL	2729	2.720	37.740	52.300	1.00	0.00
ATOM	9311	HW2	SOL	2729	3.450	37.020	53.410	1.00	0.00
ATOM	9312	OW	SOL	2730	4.200	21.980	35.410	1.00	0.00
ATOM	9313	HW1	SOL	2730	3.980	22.060	34.480	1.00	0.00
ATOM	9314	HW2	SOL	2730	5.140	22.150	35.450	1.00	0.00
ATOM	9315	OW	SOL	2731	3.880	22.820	39.560	1.00	0.00
ATOM	9316	HW1	SOL	2731	3.190	22.190	39.760	1.00	0.00
ATOM	9317	HW2	SOL	2731	3.600	23.630	39.980	1.00	0.00
ATOM	9318	OW	SOL	2732	8.450	9.190	45.310	1.00	0.00
ATOM	9319	HW1	SOL	2732	8.890	8.700	44.620	1.00	0.00
ATOM	9320	HW2	SOL	2732	8.670	8.730	46.110	1.00	0.00
ATOM	9321	OW	SOL	2733	16.350	36.780	16.210	1.00	0.00
ATOM	9322	HW1	SOL	2733	15.690	36.400	15.640	1.00	0.00
ATOM	9323	HW2	SOL	2733	16.770	37.450	15.680	1.00	0.00
ATOM	9324	OW	SOL	2734	10.760	39.520	16.750	1.00	0.00
ATOM	9325	HW1	SOL	2734	10.430	40.370	16.450	1.00	0.00
ATOM	9326	HW2	SOL	2734	11.580	39.730	17.210	1.00	0.00
ATOM	9327	OW	SOL	2735	19.360	45.350	31.120	1.00	0.00

ATOM	9328	HW1	SOL	2735	19.060	45.590	32.000	1.00	0.00
ATOM	9329	HW2	SOL	2735	18.650	44.810	30.770	1.00	0.00
ATOM	9330	OW	SOL	2736	11.400	48.170	24.030	1.00	0.00
ATOM	9331	HW1	SOL	2736	12.030	47.570	23.620	1.00	0.00
ATOM	9332	HW2	SOL	2736	11.880	48.560	24.760	1.00	0.00
ATOM	9333	OW	SOL	2737	22.310	36.870	45.230	1.00	0.00
ATOM	9334	HW1	SOL	2737	22.500	35.940	45.100	1.00	0.00
ATOM	9335	HW2	SOL	2737	22.620	37.050	46.120	1.00	0.00
ATOM	9336	OW	SOL	2738	15.480	9.800	41.420	1.00	0.00
ATOM	9337	HW1	SOL	2738	15.850	9.010	41.820	1.00	0.00
ATOM	9338	HW2	SOL	2738	15.070	10.260	42.150	1.00	0.00
ATOM	9339	OW	SOL	2739	30.060	12.540	41.480	1.00	0.00
ATOM	9340	HW1	SOL	2739	29.350	13.180	41.420	1.00	0.00
ATOM	9341	HW2	SOL	2739	30.710	12.850	40.850	1.00	0.00
ATOM	9342	OW	SOL	2740	3.430	49.560	43.360	1.00	0.00
ATOM	9343	HW1	SOL	2740	3.140	49.160	42.540	1.00	0.00
ATOM	9344	HW2	SOL	2740	2.670	50.070	43.660	1.00	0.00
ATOM	9345	OW	SOL	2741	5.920	39.580	8.570	1.00	0.00
ATOM	9346	HW1	SOL	2741	5.240	39.670	7.900	1.00	0.00
ATOM	9347	HW2	SOL	2741	6.670	39.210	8.100	1.00	0.00
ATOM	9348	OW	SOL	2742	0.530	54.520	6.990	1.00	0.00
ATOM	9349	HW1	SOL	2742	-0.030	54.850	7.700	1.00	0.00
ATOM	9350	HW2	SOL	2742	1.300	55.080	7.020	1.00	0.00
ATOM	9351	OW	SOL	2743	13.330	27.550	48.210	1.00	0.00
ATOM	9352	HW1	SOL	2743	13.260	26.680	47.810	1.00	0.00
ATOM	9353	HW2	SOL	2743	12.480	27.950	48.050	1.00	0.00
ATOM	9354	OW	SOL	2744	39.060	17.470	22.920	1.00	0.00
ATOM	9355	HW1	SOL	2744	39.640	16.810	23.320	1.00	0.00
ATOM	9356	HW2	SOL	2744	39.450	17.620	22.050	1.00	0.00
ATOM	9357	OW	SOL	2745	4.580	51.310	14.010	1.00	0.00
ATOM	9358	HW1	SOL	2745	5.250	50.710	13.670	1.00	0.00
ATOM	9359	HW2	SOL	2745	4.000	50.740	14.530	1.00	0.00
ATOM	9360	OW	SOL	2746	46.910	46.490	38.090	1.00	0.00
ATOM	9361	HW1	SOL	2746	47.690	45.990	38.340	1.00	0.00
ATOM	9362	HW2	SOL	2746	46.620	46.100	37.270	1.00	0.00
ATOM	9363	OW	SOL	2747	14.280	2.130	35.290	1.00	0.00
ATOM	9364	HW1	SOL	2747	14.590	3.020	35.090	1.00	0.00
ATOM	9365	HW2	SOL	2747	14.210	1.710	34.440	1.00	0.00
ATOM	9366	OW	SOL	2748	39.580	38.730	41.030	1.00	0.00
ATOM	9367	HW1	SOL	2748	38.850	38.810	40.420	1.00	0.00
ATOM	9368	HW2	SOL	2748	39.910	37.850	40.900	1.00	0.00
ATOM	9369	OW	SOL	2749	4.420	12.230	30.230	1.00	0.00
ATOM	9370	HW1	SOL	2749	5.010	12.580	30.900	1.00	0.00
ATOM	9371	HW2	SOL	2749	4.910	12.320	29.410	1.00	0.00

ATOM	9372	OW	SOL	2750	1.360	21.180	39.610	1.00	0.00
ATOM	9373	HW1	SOL	2750	1.780	20.850	38.820	1.00	0.00
ATOM	9374	HW2	SOL	2750	0.540	20.680	39.680	1.00	0.00
ATOM	9375	OW	SOL	2751	16.280	38.950	45.760	1.00	0.00
ATOM	9376	HW1	SOL	2751	16.310	38.780	46.700	1.00	0.00
ATOM	9377	HW2	SOL	2751	15.540	38.420	45.440	1.00	0.00
ATOM	9378	OW	SOL	2752	23.440	50.910	46.780	1.00	0.00
ATOM	9379	HW1	SOL	2752	24.210	51.020	47.330	1.00	0.00
ATOM	9380	HW2	SOL	2752	23.200	49.990	46.880	1.00	0.00
ATOM	9381	OW	SOL	2753	40.450	34.580	14.980	1.00	0.00
ATOM	9382	HW1	SOL	2753	40.180	34.970	14.150	1.00	0.00
ATOM	9383	HW2	SOL	2753	40.800	33.720	14.740	1.00	0.00
ATOM	9384	OW	SOL	2754	51.390	25.910	8.320	1.00	0.00
ATOM	9385	HW1	SOL	2754	51.890	25.840	7.510	1.00	0.00
ATOM	9386	HW2	SOL	2754	50.600	25.390	8.160	1.00	0.00
ATOM	9387	OW	SOL	2755	50.880	47.410	42.040	1.00	0.00
ATOM	9388	HW1	SOL	2755	50.160	47.780	41.530	1.00	0.00
ATOM	9389	HW2	SOL	2755	50.600	47.510	42.950	1.00	0.00
ATOM	9390	OW	SOL	2756	2.740	24.840	14.680	1.00	0.00
ATOM	9391	HW1	SOL	2756	2.370	25.360	15.390	1.00	0.00
ATOM	9392	HW2	SOL	2756	2.780	23.950	15.030	1.00	0.00
ATOM	9393	OW	SOL	2757	51.370	1.070	14.720	1.00	0.00
ATOM	9394	HW1	SOL	2757	51.960	1.700	14.310	1.00	0.00
ATOM	9395	HW2	SOL	2757	51.580	0.240	14.290	1.00	0.00
ATOM	9396	OW	SOL	2758	5.820	32.140	32.820	1.00	0.00
ATOM	9397	HW1	SOL	2758	5.930	33.090	32.750	1.00	0.00
ATOM	9398	HW2	SOL	2758	5.030	31.960	32.310	1.00	0.00
ATOM	9399	OW	SOL	2759	50.030	53.950	55.720	1.00	0.00
ATOM	9400	HW1	SOL	2759	50.920	54.270	55.850	1.00	0.00
ATOM	9401	HW2	SOL	2759	49.470	54.640	56.060	1.00	0.00
ATOM	9402	OW	SOL	2760	13.590	46.850	23.170	1.00	0.00
ATOM	9403	HW1	SOL	2760	14.510	46.660	23.370	1.00	0.00
ATOM	9404	HW2	SOL	2760	13.560	46.900	22.220	1.00	0.00
ATOM	9405	OW	SOL	2761	10.240	37.950	55.110	1.00	0.00
ATOM	9406	HW1	SOL	2761	10.660	37.780	55.960	1.00	0.00
ATOM	9407	HW2	SOL	2761	9.950	37.090	54.820	1.00	0.00
ATOM	9408	OW	SOL	2762	36.560	34.280	51.390	1.00	0.00
ATOM	9409	HW1	SOL	2762	35.730	34.270	50.900	1.00	0.00
ATOM	9410	HW2	SOL	2762	36.640	35.180	51.700	1.00	0.00
ATOM	9411	OW	SOL	2763	38.570	1.640	37.140	1.00	0.00
ATOM	9412	HW1	SOL	2763	39.340	1.240	36.730	1.00	0.00
ATOM	9413	HW2	SOL	2763	38.900	2.040	37.940	1.00	0.00
ATOM	9414	OW	SOL	2764	23.160	15.560	13.200	1.00	0.00
ATOM	9415	HW1	SOL	2764	23.860	14.990	12.870	1.00	0.00

ATOM	9416	HW2	SOL	2764	22.940	15.200	14.060	1.00	0.00
ATOM	9417	OW	SOL	2765	31.320	13.160	49.810	1.00	0.00
ATOM	9418	HW1	SOL	2765	30.890	12.360	50.100	1.00	0.00
ATOM	9419	HW2	SOL	2765	31.410	13.060	48.860	1.00	0.00
ATOM	9420	OW	SOL	2766	9.180	20.050	6.510	1.00	0.00
ATOM	9421	HW1	SOL	2766	9.450	20.890	6.130	1.00	0.00
ATOM	9422	HW2	SOL	2766	8.730	19.600	5.790	1.00	0.00
ATOM	9423	OW	SOL	2767	53.100	8.870	19.530	1.00	0.00
ATOM	9424	HW1	SOL	2767	53.700	9.050	20.250	1.00	0.00
ATOM	9425	HW2	SOL	2767	53.670	8.810	18.760	1.00	0.00
ATOM	9426	OW	SOL	2768	2.770	43.400	12.280	1.00	0.00
ATOM	9427	HW1	SOL	2768	3.500	42.780	12.350	1.00	0.00
ATOM	9428	HW2	SOL	2768	2.360	43.390	13.140	1.00	0.00
ATOM	9429	OW	SOL	2769	5.830	43.850	48.510	1.00	0.00
ATOM	9430	HW1	SOL	2769	5.610	43.100	47.950	1.00	0.00
ATOM	9431	HW2	SOL	2769	6.770	43.970	48.380	1.00	0.00
ATOM	9432	OW	SOL	2770	14.840	46.560	33.660	1.00	0.00
ATOM	9433	HW1	SOL	2770	14.320	47.300	33.970	1.00	0.00
ATOM	9434	HW2	SOL	2770	15.030	46.770	32.740	1.00	0.00
ATOM	9435	OW	SOL	2771	18.620	10.850	24.880	1.00	0.00
ATOM	9436	HW1	SOL	2771	17.740	10.480	24.810	1.00	0.00
ATOM	9437	HW2	SOL	2771	18.480	11.800	24.860	1.00	0.00
ATOM	9438	OW	SOL	2772	31.110	44.110	46.920	1.00	0.00
ATOM	9439	HW1	SOL	2772	31.680	43.340	46.990	1.00	0.00
ATOM	9440	HW2	SOL	2772	31.720	44.850	46.890	1.00	0.00
ATOM	9441	OW	SOL	2773	13.800	39.740	49.410	1.00	0.00
ATOM	9442	HW1	SOL	2773	13.870	40.220	48.590	1.00	0.00
ATOM	9443	HW2	SOL	2773	13.470	38.880	49.170	1.00	0.00
ATOM	9444	OW	SOL	2774	35.840	4.410	51.960	1.00	0.00
ATOM	9445	HW1	SOL	2774	36.510	5.070	52.150	1.00	0.00
ATOM	9446	HW2	SOL	2774	36.000	4.170	51.050	1.00	0.00
ATOM	9447	OW	SOL	2775	13.090	52.600	44.960	1.00	0.00
ATOM	9448	HW1	SOL	2775	13.700	52.110	45.510	1.00	0.00
ATOM	9449	HW2	SOL	2775	13.630	53.270	44.540	1.00	0.00
ATOM	9450	OW	SOL	2776	26.550	2.590	24.530	1.00	0.00
ATOM	9451	HW1	SOL	2776	26.160	2.350	23.690	1.00	0.00
ATOM	9452	HW2	SOL	2776	26.810	3.510	24.430	1.00	0.00
ATOM	9453	OW	SOL	2777	25.370	15.740	29.940	1.00	0.00
ATOM	9454	HW1	SOL	2777	25.330	15.470	29.020	1.00	0.00
ATOM	9455	HW2	SOL	2777	25.650	16.650	29.910	1.00	0.00
ATOM	9456	OW	SOL	2778	54.690	55.670	3.650	1.00	0.00
ATOM	9457	HW1	SOL	2778	54.400	56.140	2.870	1.00	0.00
ATOM	9458	HW2	SOL	2778	55.230	56.290	4.130	1.00	0.00
ATOM	9459	OW	SOL	2779	13.290	51.500	29.060	1.00	0.00

ATOM	9460	HW1	SOL	2779	14.120	51.530	29.530	1.00	0.00
ATOM	9461	HW2	SOL	2779	13.320	52.260	28.480	1.00	0.00
ATOM	9462	OW	SOL	2780	13.020	49.600	53.170	1.00	0.00
ATOM	9463	HW1	SOL	2780	13.190	50.130	53.950	1.00	0.00
ATOM	9464	HW2	SOL	2780	12.170	49.920	52.850	1.00	0.00
ATOM	9465	OW	SOL	2781	2.040	47.520	13.250	1.00	0.00
ATOM	9466	HW1	SOL	2781	2.390	48.170	13.860	1.00	0.00
ATOM	9467	HW2	SOL	2781	2.810	47.150	12.820	1.00	0.00
ATOM	9468	OW	SOL	2782	24.250	11.860	8.520	1.00	0.00
ATOM	9469	HW1	SOL	2782	23.630	11.570	7.850	1.00	0.00
ATOM	9470	HW2	SOL	2782	24.950	11.210	8.510	1.00	0.00
ATOM	9471	OW	SOL	2783	11.300	55.230	41.630	1.00	0.00
ATOM	9472	HW1	SOL	2783	11.700	55.850	42.240	1.00	0.00
ATOM	9473	HW2	SOL	2783	10.400	55.540	41.540	1.00	0.00
ATOM	9474	OW	SOL	2784	43.650	37.020	18.710	1.00	0.00
ATOM	9475	HW1	SOL	2784	43.020	37.530	18.190	1.00	0.00
ATOM	9476	HW2	SOL	2784	44.180	37.670	19.160	1.00	0.00
ATOM	9477	OW	SOL	2785	36.190	3.570	6.810	1.00	0.00
ATOM	9478	HW1	SOL	2785	36.590	3.700	7.670	1.00	0.00
ATOM	9479	HW2	SOL	2785	35.350	3.140	7.010	1.00	0.00
ATOM	9480	OW	SOL	2786	0.190	21.040	1.920	1.00	0.00
ATOM	9481	HW1	SOL	2786	0.890	21.530	2.360	1.00	0.00
ATOM	9482	HW2	SOL	2786	-0.190	21.680	1.310	1.00	0.00
ATOM	9483	OW	SOL	2787	32.590	48.080	16.690	1.00	0.00
ATOM	9484	HW1	SOL	2787	33.510	48.230	16.930	1.00	0.00
ATOM	9485	HW2	SOL	2787	32.230	47.610	17.440	1.00	0.00
ATOM	9486	OW	SOL	2788	30.200	39.960	31.930	1.00	0.00
ATOM	9487	HW1	SOL	2788	30.610	39.110	32.070	1.00	0.00
ATOM	9488	HW2	SOL	2788	30.930	40.580	31.950	1.00	0.00
ATOM	9489	OW	SOL	2789	55.230	27.550	23.390	1.00	0.00
ATOM	9490	HW1	SOL	2789	55.930	27.250	23.970	1.00	0.00
ATOM	9491	HW2	SOL	2789	54.540	27.850	23.970	1.00	0.00
ATOM	9492	OW	SOL	2790	0.770	26.370	29.120	1.00	0.00
ATOM	9493	HW1	SOL	2790	1.260	25.650	29.530	1.00	0.00
ATOM	9494	HW2	SOL	2790	0.610	26.980	29.840	1.00	0.00
ATOM	9495	OW	SOL	2791	23.280	31.430	23.800	1.00	0.00
ATOM	9496	HW1	SOL	2791	23.130	32.170	23.200	1.00	0.00
ATOM	9497	HW2	SOL	2791	22.490	30.900	23.740	1.00	0.00
ATOM	9498	OW	SOL	2792	38.110	54.510	37.350	1.00	0.00
ATOM	9499	HW1	SOL	2792	37.660	53.680	37.500	1.00	0.00
ATOM	9500	HW2	SOL	2792	38.260	54.540	36.410	1.00	0.00
ATOM	9501	OW	SOL	2793	3.350	10.070	22.980	1.00	0.00
ATOM	9502	HW1	SOL	2793	3.370	9.720	23.870	1.00	0.00
ATOM	9503	HW2	SOL	2793	3.360	11.020	23.090	1.00	0.00

ATOM	9504	OW	SOL	2794	31.880	32.830	4.890	1.00	0.00
ATOM	9505	HW1	SOL	2794	31.930	32.460	4.010	1.00	0.00
ATOM	9506	HW2	SOL	2794	31.600	32.090	5.440	1.00	0.00
ATOM	9507	OW	SOL	2795	23.130	35.640	14.800	1.00	0.00
ATOM	9508	HW1	SOL	2795	22.220	35.420	15.000	1.00	0.00
ATOM	9509	HW2	SOL	2795	23.640	34.960	15.240	1.00	0.00
ATOM	9510	OW	SOL	2796	19.090	51.240	55.340	1.00	0.00
ATOM	9511	HW1	SOL	2796	18.870	51.420	54.430	1.00	0.00
ATOM	9512	HW2	SOL	2796	19.460	50.360	55.330	1.00	0.00
ATOM	9513	OW	SOL	2797	47.730	17.500	24.400	1.00	0.00
ATOM	9514	HW1	SOL	2797	48.360	17.420	23.690	1.00	0.00
ATOM	9515	HW2	SOL	2797	46.880	17.580	23.960	1.00	0.00
ATOM	9516	OW	SOL	2798	40.020	41.490	14.160	1.00	0.00
ATOM	9517	HW1	SOL	2798	39.870	41.470	15.110	1.00	0.00
ATOM	9518	HW2	SOL	2798	39.200	41.830	13.800	1.00	0.00
ATOM	9519	OW	SOL	2799	25.100	25.880	11.380	1.00	0.00
ATOM	9520	HW1	SOL	2799	25.150	25.100	10.810	1.00	0.00
ATOM	9521	HW2	SOL	2799	24.520	25.620	12.090	1.00	0.00
ATOM	9522	OW	SOL	2800	41.970	48.150	41.440	1.00	0.00
ATOM	9523	HW1	SOL	2800	41.210	48.380	40.920	1.00	0.00
ATOM	9524	HW2	SOL	2800	41.630	48.060	42.330	1.00	0.00
ATOM	9525	OW	SOL	2801	19.240	55.690	21.700	1.00	0.00
ATOM	9526	HW1	SOL	2801	19.180	55.820	20.750	1.00	0.00
ATOM	9527	HW2	SOL	2801	18.580	56.270	22.070	1.00	0.00
ATOM	9528	OW	SOL	2802	0.960	4.840	43.190	1.00	0.00
ATOM	9529	HW1	SOL	2802	1.810	4.410	43.150	1.00	0.00
ATOM	9530	HW2	SOL	2802	1.150	5.780	43.140	1.00	0.00
ATOM	9531	OW	SOL	2803	50.940	23.190	32.080	1.00	0.00
ATOM	9532	HW1	SOL	2803	50.730	23.270	31.150	1.00	0.00
ATOM	9533	HW2	SOL	2803	51.370	22.340	32.160	1.00	0.00
ATOM	9534	OW	SOL	2804	49.400	37.710	4.880	1.00	0.00
ATOM	9535	HW1	SOL	2804	50.180	38.250	4.720	1.00	0.00
ATOM	9536	HW2	SOL	2804	48.720	38.100	4.330	1.00	0.00
ATOM	9537	OW	SOL	2805	3.940	23.870	29.650	1.00	0.00
ATOM	9538	HW1	SOL	2805	3.100	24.080	30.060	1.00	0.00
ATOM	9539	HW2	SOL	2805	3.710	23.570	28.770	1.00	0.00
ATOM	9540	OW	SOL	2806	7.100	7.610	21.560	1.00	0.00
ATOM	9541	HW1	SOL	2806	6.230	7.780	21.200	1.00	0.00
ATOM	9542	HW2	SOL	2806	7.690	8.130	21.010	1.00	0.00
ATOM	9543	OW	SOL	2807	16.510	8.740	38.050	1.00	0.00
ATOM	9544	HW1	SOL	2807	16.360	9.000	38.960	1.00	0.00
ATOM	9545	HW2	SOL	2807	17.020	7.930	38.120	1.00	0.00
ATOM	9546	OW	SOL	2808	8.570	28.270	50.790	1.00	0.00
ATOM	9547	HW1	SOL	2808	7.840	27.690	50.590	1.00	0.00

ATOM	9548	HW2	SOL	2808	9.350	27.790	50.510	1.00	0.00
ATOM	9549	OW	SOL	2809	48.070	37.370	46.790	1.00	0.00
ATOM	9550	HW1	SOL	2809	47.670	37.560	45.930	1.00	0.00
ATOM	9551	HW2	SOL	2809	47.340	37.080	47.330	1.00	0.00
ATOM	9552	OW	SOL	2810	29.170	26.610	8.840	1.00	0.00
ATOM	9553	HW1	SOL	2810	28.790	26.720	9.710	1.00	0.00
ATOM	9554	HW2	SOL	2810	28.850	25.760	8.550	1.00	0.00
ATOM	9555	OW	SOL	2811	42.730	55.160	43.850	1.00	0.00
ATOM	9556	HW1	SOL	2811	42.480	55.910	44.380	1.00	0.00
ATOM	9557	HW2	SOL	2811	43.570	54.880	44.210	1.00	0.00
ATOM	9558	OW	SOL	2812	2.900	52.060	50.020	1.00	0.00
ATOM	9559	HW1	SOL	2812	2.610	51.830	50.910	1.00	0.00
ATOM	9560	HW2	SOL	2812	3.820	51.790	50.000	1.00	0.00
ATOM	9561	OW	SOL	2813	11.080	10.750	20.930	1.00	0.00
ATOM	9562	HW1	SOL	2813	11.670	11.170	21.550	1.00	0.00
ATOM	9563	HW2	SOL	2813	11.650	10.460	20.220	1.00	0.00
ATOM	9564	OW	SOL	2814	13.910	19.710	27.620	1.00	0.00
ATOM	9565	HW1	SOL	2814	13.150	20.230	27.900	1.00	0.00
ATOM	9566	HW2	SOL	2814	14.670	20.170	27.980	1.00	0.00
ATOM	9567	OW	SOL	2815	43.220	47.850	31.930	1.00	0.00
ATOM	9568	HW1	SOL	2815	42.440	47.720	31.400	1.00	0.00
ATOM	9569	HW2	SOL	2815	43.650	48.610	31.540	1.00	0.00
ATOM	9570	OW	SOL	2816	6.270	52.390	22.500	1.00	0.00
ATOM	9571	HW1	SOL	2816	5.800	52.380	21.660	1.00	0.00
ATOM	9572	HW2	SOL	2816	5.570	52.350	23.160	1.00	0.00
ATOM	9573	OW	SOL	2817	27.940	54.390	44.180	1.00	0.00
ATOM	9574	HW1	SOL	2817	27.370	53.660	43.960	1.00	0.00
ATOM	9575	HW2	SOL	2817	28.770	54.200	43.750	1.00	0.00
ATOM	9576	OW	SOL	2818	26.530	4.460	47.450	1.00	0.00
ATOM	9577	HW1	SOL	2818	26.690	4.450	46.510	1.00	0.00
ATOM	9578	HW2	SOL	2818	26.430	3.530	47.680	1.00	0.00
ATOM	9579	OW	SOL	2819	19.770	2.040	32.050	1.00	0.00
ATOM	9580	HW1	SOL	2819	20.110	2.900	32.310	1.00	0.00
ATOM	9581	HW2	SOL	2819	20.550	1.540	31.820	1.00	0.00
ATOM	9582	OW	SOL	2820	35.430	24.500	7.690	1.00	0.00
ATOM	9583	HW1	SOL	2820	34.670	24.200	7.180	1.00	0.00
ATOM	9584	HW2	SOL	2820	35.310	24.100	8.550	1.00	0.00
ATOM	9585	OW	SOL	2821	49.090	48.130	39.950	1.00	0.00
ATOM	9586	HW1	SOL	2821	48.180	48.240	39.700	1.00	0.00
ATOM	9587	HW2	SOL	2821	49.460	47.550	39.280	1.00	0.00
ATOM	9588	OW	SOL	2822	27.300	9.860	7.800	1.00	0.00
ATOM	9589	HW1	SOL	2822	27.950	10.130	7.150	1.00	0.00
ATOM	9590	HW2	SOL	2822	27.230	10.610	8.390	1.00	0.00
ATOM	9591	OW	SOL	2823	5.770	19.300	22.270	1.00	0.00

ATOM	9592	HW1	SOL	2823	5.660	18.510	21.750	1.00	0.00
ATOM	9593	HW2	SOL	2823	6.510	19.110	22.840	1.00	0.00
ATOM	9594	OW	SOL	2824	34.020	26.990	50.920	1.00	0.00
ATOM	9595	HW1	SOL	2824	34.160	27.840	50.500	1.00	0.00
ATOM	9596	HW2	SOL	2824	33.370	27.170	51.600	1.00	0.00
ATOM	9597	OW	SOL	2825	21.330	25.220	27.770	1.00	0.00
ATOM	9598	HW1	SOL	2825	22.160	25.360	27.310	1.00	0.00
ATOM	9599	HW2	SOL	2825	21.570	24.690	28.530	1.00	0.00
ATOM	9600	OW	SOL	2826	23.070	32.360	11.390	1.00	0.00
ATOM	9601	HW1	SOL	2826	22.550	33.000	11.870	1.00	0.00
ATOM	9602	HW2	SOL	2826	23.870	32.830	11.150	1.00	0.00
ATOM	9603	OW	SOL	2827	32.040	52.520	13.190	1.00	0.00
ATOM	9604	HW1	SOL	2827	31.890	51.630	13.500	1.00	0.00
ATOM	9605	HW2	SOL	2827	32.410	52.970	13.950	1.00	0.00
ATOM	9606	OW	SOL	2828	54.430	23.700	14.950	1.00	0.00
ATOM	9607	HW1	SOL	2828	54.440	23.420	14.040	1.00	0.00
ATOM	9608	HW2	SOL	2828	54.320	22.890	15.450	1.00	0.00
ATOM	9609	OW	SOL	2829	51.200	50.570	0.520	1.00	0.00
ATOM	9610	HW1	SOL	2829	51.070	51.050	-0.300	1.00	0.00
ATOM	9611	HW2	SOL	2829	51.610	51.200	1.110	1.00	0.00
ATOM	9612	OW	SOL	2830	42.520	8.800	42.910	1.00	0.00
ATOM	9613	HW1	SOL	2830	41.960	8.800	42.140	1.00	0.00
ATOM	9614	HW2	SOL	2830	42.190	9.530	43.440	1.00	0.00
ATOM	9615	OW	SOL	2831	45.320	38.900	20.210	1.00	0.00
ATOM	9616	HW1	SOL	2831	45.450	39.430	19.420	1.00	0.00
ATOM	9617	HW2	SOL	2831	45.330	39.520	20.930	1.00	0.00
ATOM	9618	OW	SOL	2832	14.930	50.980	55.000	1.00	0.00
ATOM	9619	HW1	SOL	2832	15.520	51.630	55.370	1.00	0.00
ATOM	9620	HW2	SOL	2832	15.490	50.440	54.430	1.00	0.00
ATOM	9621	OW	SOL	2833	2.610	54.510	28.040	1.00	0.00
ATOM	9622	HW1	SOL	2833	3.120	54.930	27.350	1.00	0.00
ATOM	9623	HW2	SOL	2833	2.870	53.590	28.000	1.00	0.00
ATOM	9624	OW	SOL	2834	36.930	43.010	46.250	1.00	0.00
ATOM	9625	HW1	SOL	2834	36.780	43.130	47.190	1.00	0.00
ATOM	9626	HW2	SOL	2834	37.500	42.240	46.200	1.00	0.00
ATOM	9627	OW	SOL	2835	21.370	20.910	14.130	1.00	0.00
ATOM	9628	HW1	SOL	2835	20.640	20.390	13.800	1.00	0.00
ATOM	9629	HW2	SOL	2835	22.090	20.700	13.540	1.00	0.00
ATOM	9630	OW	SOL	2836	0.940	32.770	1.900	1.00	0.00
ATOM	9631	HW1	SOL	2836	1.580	33.350	1.480	1.00	0.00
ATOM	9632	HW2	SOL	2836	0.610	33.280	2.650	1.00	0.00
ATOM	9633	OW	SOL	2837	45.640	49.270	14.550	1.00	0.00
ATOM	9634	HW1	SOL	2837	45.200	48.450	14.790	1.00	0.00
ATOM	9635	HW2	SOL	2837	46.070	49.550	15.360	1.00	0.00

ATOM	9636	OW	SOL	2838	10.850	11.790	30.420	1.00	0.00
ATOM	9637	HW1	SOL	2838	10.880	12.740	30.270	1.00	0.00
ATOM	9638	HW2	SOL	2838	10.350	11.450	29.690	1.00	0.00
ATOM	9639	OW	SOL	2839	25.620	46.130	44.220	1.00	0.00
ATOM	9640	HW1	SOL	2839	26.440	45.640	44.170	1.00	0.00
ATOM	9641	HW2	SOL	2839	25.880	47.040	44.140	1.00	0.00
ATOM	9642	OW	SOL	2840	19.370	7.350	45.500	1.00	0.00
ATOM	9643	HW1	SOL	2840	19.310	8.300	45.410	1.00	0.00
ATOM	9644	HW2	SOL	2840	18.450	7.060	45.530	1.00	0.00
ATOM	9645	OW	SOL	2841	55.550	3.310	29.000	1.00	0.00
ATOM	9646	HW1	SOL	2841	55.060	2.580	29.370	1.00	0.00
ATOM	9647	HW2	SOL	2841	55.290	4.060	29.530	1.00	0.00
ATOM	9648	OW	SOL	2842	49.960	7.480	31.480	1.00	0.00
ATOM	9649	HW1	SOL	2842	50.830	7.090	31.380	1.00	0.00
ATOM	9650	HW2	SOL	2842	49.880	8.060	30.730	1.00	0.00
ATOM	9651	OW	SOL	2843	17.050	1.740	26.880	1.00	0.00
ATOM	9652	HW1	SOL	2843	16.320	2.140	26.400	1.00	0.00
ATOM	9653	HW2	SOL	2843	17.750	2.390	26.850	1.00	0.00
ATOM	9654	OW	SOL	2844	24.740	38.520	3.760	1.00	0.00
ATOM	9655	HW1	SOL	2844	25.480	38.010	3.430	1.00	0.00
ATOM	9656	HW2	SOL	2844	24.250	37.900	4.310	1.00	0.00
ATOM	9657	OW	SOL	2845	50.720	50.420	7.080	1.00	0.00
ATOM	9658	HW1	SOL	2845	51.140	49.670	6.650	1.00	0.00
ATOM	9659	HW2	SOL	2845	49.970	50.630	6.530	1.00	0.00
ATOM	9660	OW	SOL	2846	2.690	51.120	47.320	1.00	0.00
ATOM	9661	HW1	SOL	2846	3.020	51.460	48.160	1.00	0.00
ATOM	9662	HW2	SOL	2846	3.350	50.490	47.050	1.00	0.00
ATOM	9663	OW	SOL	2847	29.300	1.580	36.240	1.00	0.00
ATOM	9664	HW1	SOL	2847	28.980	1.240	35.400	1.00	0.00
ATOM	9665	HW2	SOL	2847	29.810	2.350	36.010	1.00	0.00
ATOM	9666	OW	SOL	2848	33.560	9.350	21.240	1.00	0.00
ATOM	9667	HW1	SOL	2848	33.030	10.150	21.270	1.00	0.00
ATOM	9668	HW2	SOL	2848	32.930	8.640	21.360	1.00	0.00
ATOM	9669	OW	SOL	2849	12.040	0.940	8.160	1.00	0.00
ATOM	9670	HW1	SOL	2849	12.620	1.090	7.410	1.00	0.00
ATOM	9671	HW2	SOL	2849	12.580	1.130	8.920	1.00	0.00
ATOM	9672	OW	SOL	2850	41.590	10.260	18.320	1.00	0.00
ATOM	9673	HW1	SOL	2850	40.870	10.880	18.440	1.00	0.00
ATOM	9674	HW2	SOL	2850	41.530	10.000	17.400	1.00	0.00
ATOM	9675	OW	SOL	2851	26.790	16.050	45.910	1.00	0.00
ATOM	9676	HW1	SOL	2851	26.160	16.660	45.530	1.00	0.00
ATOM	9677	HW2	SOL	2851	27.550	16.590	46.120	1.00	0.00
ATOM	9678	OW	SOL	2852	27.810	6.610	9.690	1.00	0.00
ATOM	9679	HW1	SOL	2852	27.720	7.470	10.090	1.00	0.00

ATOM	9680	HW2	SOL	2852	28.560	6.220	10.150	1.00	0.00
ATOM	9681	OW	SOL	2853	2.520	7.940	2.720	1.00	0.00
ATOM	9682	HW1	SOL	2853	3.370	7.860	2.300	1.00	0.00
ATOM	9683	HW2	SOL	2853	2.590	8.740	3.240	1.00	0.00
ATOM	9684	OW	SOL	2854	33.350	20.430	24.480	1.00	0.00
ATOM	9685	HW1	SOL	2854	33.120	20.570	25.400	1.00	0.00
ATOM	9686	HW2	SOL	2854	34.280	20.640	24.440	1.00	0.00
ATOM	9687	OW	SOL	2855	41.760	21.610	3.650	1.00	0.00
ATOM	9688	HW1	SOL	2855	42.260	22.140	3.030	1.00	0.00
ATOM	9689	HW2	SOL	2855	42.350	20.900	3.890	1.00	0.00
ATOM	9690	OW	SOL	2856	5.460	51.530	0.710	1.00	0.00
ATOM	9691	HW1	SOL	2856	5.750	50.620	0.600	1.00	0.00
ATOM	9692	HW2	SOL	2856	6.130	52.050	0.280	1.00	0.00
ATOM	9693	OW	SOL	2857	12.540	34.810	17.510	1.00	0.00
ATOM	9694	HW1	SOL	2857	12.250	35.510	16.920	1.00	0.00
ATOM	9695	HW2	SOL	2857	13.160	35.230	18.100	1.00	0.00
ATOM	9696	OW	SOL	2858	38.350	53.740	10.270	1.00	0.00
ATOM	9697	HW1	SOL	2858	37.440	53.990	10.400	1.00	0.00
ATOM	9698	HW2	SOL	2858	38.310	53.000	9.650	1.00	0.00
ATOM	9699	OW	SOL	2859	6.170	12.150	28.090	1.00	0.00
ATOM	9700	HW1	SOL	2859	6.840	12.650	28.550	1.00	0.00
ATOM	9701	HW2	SOL	2859	6.440	11.240	28.160	1.00	0.00
ATOM	9702	OW	SOL	2860	29.780	4.790	44.400	1.00	0.00
ATOM	9703	HW1	SOL	2860	28.970	5.260	44.570	1.00	0.00
ATOM	9704	HW2	SOL	2860	30.400	5.470	44.120	1.00	0.00
ATOM	9705	OW	SOL	2861	44.330	32.780	12.290	1.00	0.00
ATOM	9706	HW1	SOL	2861	45.200	32.850	11.910	1.00	0.00
ATOM	9707	HW2	SOL	2861	43.730	32.820	11.540	1.00	0.00
ATOM	9708	OW	SOL	2862	48.570	15.400	3.660	1.00	0.00
ATOM	9709	HW1	SOL	2862	47.640	15.300	3.450	1.00	0.00
ATOM	9710	HW2	SOL	2862	48.750	14.670	4.250	1.00	0.00
ATOM	9711	OW	SOL	2863	14.700	2.680	13.830	1.00	0.00
ATOM	9712	HW1	SOL	2863	15.240	2.150	14.420	1.00	0.00
ATOM	9713	HW2	SOL	2863	14.040	3.070	14.400	1.00	0.00
ATOM	9714	OW	SOL	2864	41.660	7.070	34.630	1.00	0.00
ATOM	9715	HW1	SOL	2864	41.520	8.010	34.630	1.00	0.00
ATOM	9716	HW2	SOL	2864	41.640	6.820	33.700	1.00	0.00
ATOM	9717	OW	SOL	2865	27.010	14.130	47.670	1.00	0.00
ATOM	9718	HW1	SOL	2865	26.790	14.750	46.980	1.00	0.00
ATOM	9719	HW2	SOL	2865	27.620	13.520	47.260	1.00	0.00
ATOM	9720	OW	SOL	2866	30.800	49.840	40.580	1.00	0.00
ATOM	9721	HW1	SOL	2866	30.100	49.400	40.090	1.00	0.00
ATOM	9722	HW2	SOL	2866	31.550	49.260	40.510	1.00	0.00
ATOM	9723	OW	SOL	2867	38.330	1.510	24.440	1.00	0.00

ATOM	9724	HW1	SOL	2867	39.120	1.690	24.940	1.00	0.00
ATOM	9725	HW2	SOL	2867	38.110	0.600	24.660	1.00	0.00
ATOM	9726	OW	SOL	2868	53.150	24.390	4.020	1.00	0.00
ATOM	9727	HW1	SOL	2868	53.310	25.250	3.650	1.00	0.00
ATOM	9728	HW2	SOL	2868	52.210	24.240	3.880	1.00	0.00
ATOM	9729	OW	SOL	2869	34.530	4.920	44.070	1.00	0.00
ATOM	9730	HW1	SOL	2869	34.200	5.160	44.940	1.00	0.00
ATOM	9731	HW2	SOL	2869	35.030	5.680	43.790	1.00	0.00
ATOM	9732	OW	SOL	2870	29.220	18.430	29.090	1.00	0.00
ATOM	9733	HW1	SOL	2870	28.420	18.070	28.700	1.00	0.00
ATOM	9734	HW2	SOL	2870	29.670	18.850	28.360	1.00	0.00
ATOM	9735	OW	SOL	2871	18.400	41.360	46.120	1.00	0.00
ATOM	9736	HW1	SOL	2871	18.010	40.580	45.720	1.00	0.00
ATOM	9737	HW2	SOL	2871	18.440	41.150	47.050	1.00	0.00
ATOM	9738	OW	SOL	2872	18.460	7.180	7.390	1.00	0.00
ATOM	9739	HW1	SOL	2872	17.820	7.750	6.960	1.00	0.00
ATOM	9740	HW2	SOL	2872	19.010	6.860	6.680	1.00	0.00
ATOM	9741	OW	SOL	2873	30.170	9.840	16.320	1.00	0.00
ATOM	9742	HW1	SOL	2873	29.710	10.100	17.120	1.00	0.00
ATOM	9743	HW2	SOL	2873	30.160	10.630	15.770	1.00	0.00
ATOM	9744	OW	SOL	2874	53.920	55.820	46.910	1.00	0.00
ATOM	9745	HW1	SOL	2874	53.170	55.310	46.620	1.00	0.00
ATOM	9746	HW2	SOL	2874	54.330	56.130	46.100	1.00	0.00
ATOM	9747	OW	SOL	2875	31.580	35.700	21.200	1.00	0.00
ATOM	9748	HW1	SOL	2875	30.990	36.310	20.750	1.00	0.00
ATOM	9749	HW2	SOL	2875	32.360	35.670	20.640	1.00	0.00
ATOM	9750	OW	SOL	2876	36.540	49.230	39.820	1.00	0.00
ATOM	9751	HW1	SOL	2876	36.490	48.580	40.530	1.00	0.00
ATOM	9752	HW2	SOL	2876	36.430	50.070	40.260	1.00	0.00
ATOM	9753	OW	SOL	2877	1.220	42.200	43.080	1.00	0.00
ATOM	9754	HW1	SOL	2877	0.570	41.490	43.150	1.00	0.00
ATOM	9755	HW2	SOL	2877	0.970	42.820	43.770	1.00	0.00
ATOM	9756	OW	SOL	2878	50.650	22.660	44.220	1.00	0.00
ATOM	9757	HW1	SOL	2878	50.350	23.340	44.830	1.00	0.00
ATOM	9758	HW2	SOL	2878	50.960	21.950	44.790	1.00	0.00
ATOM	9759	OW	SOL	2879	21.740	18.040	7.980	1.00	0.00
ATOM	9760	HW1	SOL	2879	21.330	18.610	8.630	1.00	0.00
ATOM	9761	HW2	SOL	2879	22.220	18.640	7.410	1.00	0.00
ATOM	9762	OW	SOL	2880	44.250	6.820	22.140	1.00	0.00
ATOM	9763	HW1	SOL	2880	44.910	7.380	21.720	1.00	0.00
ATOM	9764	HW2	SOL	2880	43.550	6.750	21.490	1.00	0.00
ATOM	9765	OW	SOL	2881	7.840	11.910	48.660	1.00	0.00
ATOM	9766	HW1	SOL	2881	8.730	11.580	48.550	1.00	0.00
ATOM	9767	HW2	SOL	2881	7.880	12.810	48.320	1.00	0.00

ATOM	9768	OW	SOL	2882	24.660	45.090	22.460	1.00	0.00
ATOM	9769	HW1	SOL	2882	24.760	45.390	23.360	1.00	0.00
ATOM	9770	HW2	SOL	2882	24.260	45.830	22.010	1.00	0.00
ATOM	9771	OW	SOL	2883	23.310	15.540	36.460	1.00	0.00
ATOM	9772	HW1	SOL	2883	23.920	14.970	35.990	1.00	0.00
ATOM	9773	HW2	SOL	2883	23.810	16.340	36.630	1.00	0.00
ATOM	9774	OW	SOL	2884	4.380	49.070	28.330	1.00	0.00
ATOM	9775	HW1	SOL	2884	3.920	48.290	28.640	1.00	0.00
ATOM	9776	HW2	SOL	2884	4.160	49.130	27.400	1.00	0.00
ATOM	9777	OW	SOL	2885	35.230	41.300	8.760	1.00	0.00
ATOM	9778	HW1	SOL	2885	35.170	40.530	9.330	1.00	0.00
ATOM	9779	HW2	SOL	2885	34.930	40.990	7.910	1.00	0.00
ATOM	9780	OW	SOL	2886	47.330	55.350	54.110	1.00	0.00
ATOM	9781	HW1	SOL	2886	47.880	55.990	54.540	1.00	0.00
ATOM	9782	HW2	SOL	2886	47.780	55.150	53.290	1.00	0.00
ATOM	9783	OW	SOL	2887	22.940	25.040	12.820	1.00	0.00
ATOM	9784	HW1	SOL	2887	22.410	24.450	13.340	1.00	0.00
ATOM	9785	HW2	SOL	2887	22.750	24.800	11.910	1.00	0.00
ATOM	9786	OW	SOL	2888	18.360	41.970	8.150	1.00	0.00
ATOM	9787	HW1	SOL	2888	18.100	41.180	7.670	1.00	0.00
ATOM	9788	HW2	SOL	2888	17.730	42.030	8.860	1.00	0.00
ATOM	9789	OW	SOL	2889	45.930	3.960	47.840	1.00	0.00
ATOM	9790	HW1	SOL	2889	46.360	4.780	47.610	1.00	0.00
ATOM	9791	HW2	SOL	2889	45.480	4.150	48.670	1.00	0.00
ATOM	9792	OW	SOL	2890	26.330	54.700	50.160	1.00	0.00
ATOM	9793	HW1	SOL	2890	25.460	55.090	50.100	1.00	0.00
ATOM	9794	HW2	SOL	2890	26.370	54.100	49.410	1.00	0.00
ATOM	9795	OW	SOL	2891	51.690	6.200	44.040	1.00	0.00
ATOM	9796	HW1	SOL	2891	50.940	5.630	43.860	1.00	0.00
ATOM	9797	HW2	SOL	2891	52.290	6.070	43.310	1.00	0.00
ATOM	9798	OW	SOL	2892	38.780	46.870	24.660	1.00	0.00
ATOM	9799	HW1	SOL	2892	38.690	47.500	25.380	1.00	0.00
ATOM	9800	HW2	SOL	2892	38.690	46.020	25.070	1.00	0.00
ATOM	9801	OW	SOL	2893	33.030	4.370	35.020	1.00	0.00
ATOM	9802	HW1	SOL	2893	33.030	4.290	34.070	1.00	0.00
ATOM	9803	HW2	SOL	2893	33.260	5.280	35.200	1.00	0.00
ATOM	9804	OW	SOL	2894	2.640	12.970	32.890	1.00	0.00
ATOM	9805	HW1	SOL	2894	3.470	12.890	32.400	1.00	0.00
ATOM	9806	HW2	SOL	2894	2.100	12.260	32.550	1.00	0.00
ATOM	9807	OW	SOL	2895	22.460	10.420	55.150	1.00	0.00
ATOM	9808	HW1	SOL	2895	23.160	11.070	55.130	1.00	0.00
ATOM	9809	HW2	SOL	2895	22.580	9.920	54.340	1.00	0.00
ATOM	9810	OW	SOL	2896	18.550	3.730	35.800	1.00	0.00
ATOM	9811	HW1	SOL	2896	18.960	4.250	35.110	1.00	0.00

ATOM	9812	HW2	SOL	2896	17.870	4.310	36.160	1.00	0.00
ATOM	9813	OW	SOL	2897	24.390	47.900	50.580	1.00	0.00
ATOM	9814	HW1	SOL	2897	25.040	48.070	49.900	1.00	0.00
ATOM	9815	HW2	SOL	2897	24.900	47.610	51.330	1.00	0.00
ATOM	9816	OW	SOL	2898	29.550	12.780	31.920	1.00	0.00
ATOM	9817	HW1	SOL	2898	29.470	12.750	32.870	1.00	0.00
ATOM	9818	HW2	SOL	2898	28.850	13.360	31.630	1.00	0.00
ATOM	9819	OW	SOL	2899	38.070	12.720	50.180	1.00	0.00
ATOM	9820	HW1	SOL	2899	37.740	11.850	49.990	1.00	0.00
ATOM	9821	HW2	SOL	2899	38.530	12.990	49.390	1.00	0.00
ATOM	9822	OW	SOL	2900	23.750	37.830	1.300	1.00	0.00
ATOM	9823	HW1	SOL	2900	22.800	37.750	1.430	1.00	0.00
ATOM	9824	HW2	SOL	2900	24.070	38.210	2.110	1.00	0.00
ATOM	9825	OW	SOL	2901	51.280	4.790	16.040	1.00	0.00
ATOM	9826	HW1	SOL	2901	50.970	5.610	15.660	1.00	0.00
ATOM	9827	HW2	SOL	2901	51.400	4.210	15.290	1.00	0.00
ATOM	9828	OW	SOL	2902	53.530	45.250	30.010	1.00	0.00
ATOM	9829	HW1	SOL	2902	53.090	44.410	30.110	1.00	0.00
ATOM	9830	HW2	SOL	2902	54.310	45.050	29.490	1.00	0.00
ATOM	9831	OW	SOL	2903	40.170	18.660	25.510	1.00	0.00
ATOM	9832	HW1	SOL	2903	41.110	18.640	25.670	1.00	0.00
ATOM	9833	HW2	SOL	2903	39.870	17.780	25.740	1.00	0.00
ATOM	9834	OW	SOL	2904	42.280	41.360	54.090	1.00	0.00
ATOM	9835	HW1	SOL	2904	42.660	41.130	53.240	1.00	0.00
ATOM	9836	HW2	SOL	2904	42.160	40.510	54.530	1.00	0.00
ATOM	9837	OW	SOL	2905	14.050	47.420	49.700	1.00	0.00
ATOM	9838	HW1	SOL	2905	14.320	48.320	49.510	1.00	0.00
ATOM	9839	HW2	SOL	2905	14.510	46.890	49.050	1.00	0.00
ATOM	9840	OW	SOL	2906	54.520	28.450	53.350	1.00	0.00
ATOM	9841	HW1	SOL	2906	55.450	28.230	53.420	1.00	0.00
ATOM	9842	HW2	SOL	2906	54.520	29.390	53.190	1.00	0.00
ATOM	9843	OW	SOL	2907	27.610	14.290	41.050	1.00	0.00
ATOM	9844	HW1	SOL	2907	27.540	15.210	40.780	1.00	0.00
ATOM	9845	HW2	SOL	2907	26.730	14.080	41.370	1.00	0.00
ATOM	9846	OW	SOL	2908	25.840	12.950	22.520	1.00	0.00
ATOM	9847	HW1	SOL	2908	25.580	12.980	23.440	1.00	0.00
ATOM	9848	HW2	SOL	2908	26.300	13.770	22.370	1.00	0.00
ATOM	9849	OW	SOL	2909	3.790	39.260	6.790	1.00	0.00
ATOM	9850	HW1	SOL	2909	3.870	38.920	5.900	1.00	0.00
ATOM	9851	HW2	SOL	2909	2.860	39.150	7.000	1.00	0.00
ATOM	9852	OW	SOL	2910	10.550	54.580	17.600	1.00	0.00
ATOM	9853	HW1	SOL	2910	10.630	55.540	17.670	1.00	0.00
ATOM	9854	HW2	SOL	2910	10.420	54.420	16.670	1.00	0.00
ATOM	9855	OW	SOL	2911	13.460	3.000	16.300	1.00	0.00

ATOM	9856	HW1	SOL	2911	12.980	3.820	16.410	1.00	0.00
ATOM	9857	HW2	SOL	2911	13.850	2.830	17.160	1.00	0.00
ATOM	9858	OW	SOL	2912	42.110	44.710	13.680	1.00	0.00
ATOM	9859	HW1	SOL	2912	41.320	44.790	14.220	1.00	0.00
ATOM	9860	HW2	SOL	2912	42.450	43.840	13.880	1.00	0.00
ATOM	9861	OW	SOL	2913	50.500	42.010	6.530	1.00	0.00
ATOM	9862	HW1	SOL	2913	50.090	41.500	7.230	1.00	0.00
ATOM	9863	HW2	SOL	2913	51.170	41.440	6.170	1.00	0.00
ATOM	9864	OW	SOL	2914	21.710	44.860	12.190	1.00	0.00
ATOM	9865	HW1	SOL	2914	21.480	44.580	13.070	1.00	0.00
ATOM	9866	HW2	SOL	2914	20.950	44.630	11.660	1.00	0.00
ATOM	9867	OW	SOL	2915	44.550	17.730	47.550	1.00	0.00
ATOM	9868	HW1	SOL	2915	44.840	18.490	47.040	1.00	0.00
ATOM	9869	HW2	SOL	2915	43.830	18.060	48.080	1.00	0.00
ATOM	9870	OW	SOL	2916	5.310	32.130	1.410	1.00	0.00
ATOM	9871	HW1	SOL	2916	5.580	33.050	1.390	1.00	0.00
ATOM	9872	HW2	SOL	2916	4.700	32.050	0.670	1.00	0.00
ATOM	9873	OW	SOL	2917	16.210	14.080	1.160	1.00	0.00
ATOM	9874	HW1	SOL	2917	16.230	14.350	0.240	1.00	0.00
ATOM	9875	HW2	SOL	2917	16.730	13.280	1.190	1.00	0.00
ATOM	9876	OW	SOL	2918	22.630	18.050	51.580	1.00	0.00
ATOM	9877	HW1	SOL	2918	23.220	18.420	52.240	1.00	0.00
ATOM	9878	HW2	SOL	2918	23.200	17.760	50.870	1.00	0.00
ATOM	9879	OW	SOL	2919	0.890	18.560	50.020	1.00	0.00
ATOM	9880	HW1	SOL	2919	0.530	18.950	50.820	1.00	0.00
ATOM	9881	HW2	SOL	2919	1.780	18.930	49.950	1.00	0.00
ATOM	9882	OW	SOL	2920	43.470	55.070	47.900	1.00	0.00
ATOM	9883	HW1	SOL	2920	43.000	54.230	47.890	1.00	0.00
ATOM	9884	HW2	SOL	2920	44.180	54.950	48.530	1.00	0.00
ATOM	9885	OW	SOL	2921	21.870	50.810	15.730	1.00	0.00
ATOM	9886	HW1	SOL	2921	21.690	49.870	15.610	1.00	0.00
ATOM	9887	HW2	SOL	2921	22.070	51.130	14.850	1.00	0.00
ATOM	9888	OW	SOL	2922	41.980	31.990	7.410	1.00	0.00
ATOM	9889	HW1	SOL	2922	42.160	31.400	6.670	1.00	0.00
ATOM	9890	HW2	SOL	2922	41.650	32.790	7.000	1.00	0.00
ATOM	9891	OW	SOL	2923	19.300	30.360	44.680	1.00	0.00
ATOM	9892	HW1	SOL	2923	18.480	30.410	45.160	1.00	0.00
ATOM	9893	HW2	SOL	2923	19.300	29.470	44.310	1.00	0.00
ATOM	9894	OW	SOL	2924	11.790	21.390	27.850	1.00	0.00
ATOM	9895	HW1	SOL	2924	11.050	20.880	28.190	1.00	0.00
ATOM	9896	HW2	SOL	2924	11.770	22.200	28.360	1.00	0.00
ATOM	9897	OW	SOL	2925	5.150	6.210	40.350	1.00	0.00
ATOM	9898	HW1	SOL	2925	4.460	6.690	39.890	1.00	0.00
ATOM	9899	HW2	SOL	2925	4.730	5.910	41.160	1.00	0.00

ATOM	9900	OW	SOL	2926	11.830	1.010	47.660	1.00	0.00
ATOM	9901	HW1	SOL	2926	11.710	1.960	47.600	1.00	0.00
ATOM	9902	HW2	SOL	2926	11.210	0.730	48.340	1.00	0.00
ATOM	9903	OW	SOL	2927	26.050	37.950	46.300	1.00	0.00
ATOM	9904	HW1	SOL	2927	26.290	38.840	46.570	1.00	0.00
ATOM	9905	HW2	SOL	2927	26.430	37.380	46.980	1.00	0.00
ATOM	9906	OW	SOL	2928	54.200	17.280	21.690	1.00	0.00
ATOM	9907	HW1	SOL	2928	54.730	16.830	22.350	1.00	0.00
ATOM	9908	HW2	SOL	2928	54.090	18.170	22.040	1.00	0.00
ATOM	9909	OW	SOL	2929	4.790	55.790	9.320	1.00	0.00
ATOM	9910	HW1	SOL	2929	5.580	55.630	8.810	1.00	0.00
ATOM	9911	HW2	SOL	2929	4.500	54.920	9.580	1.00	0.00
ATOM	9912	OW	SOL	2930	31.250	14.120	13.690	1.00	0.00
ATOM	9913	HW1	SOL	2930	31.630	13.540	13.030	1.00	0.00
ATOM	9914	HW2	SOL	2930	31.880	14.130	14.400	1.00	0.00
ATOM	9915	OW	SOL	2931	26.280	24.490	54.860	1.00	0.00
ATOM	9916	HW1	SOL	2931	25.370	24.800	54.840	1.00	0.00
ATOM	9917	HW2	SOL	2931	26.530	24.550	55.780	1.00	0.00
ATOM	9918	OW	SOL	2932	35.310	24.360	16.940	1.00	0.00
ATOM	9919	HW1	SOL	2932	34.480	24.820	17.050	1.00	0.00
ATOM	9920	HW2	SOL	2932	35.120	23.460	17.210	1.00	0.00
ATOM	9921	OW	SOL	2933	6.020	8.120	17.430	1.00	0.00
ATOM	9922	HW1	SOL	2933	5.220	8.140	17.950	1.00	0.00
ATOM	9923	HW2	SOL	2933	5.940	8.850	16.820	1.00	0.00
ATOM	9924	OW	SOL	2934	17.010	7.560	27.180	1.00	0.00
ATOM	9925	HW1	SOL	2934	17.410	8.360	27.510	1.00	0.00
ATOM	9926	HW2	SOL	2934	17.740	7.030	26.860	1.00	0.00
ATOM	9927	OW	SOL	2935	8.970	42.880	29.170	1.00	0.00
ATOM	9928	HW1	SOL	2935	9.220	43.190	28.300	1.00	0.00
ATOM	9929	HW2	SOL	2935	9.370	43.500	29.770	1.00	0.00
ATOM	9930	OW	SOL	2936	41.220	31.720	0.080	1.00	0.00
ATOM	9931	HW1	SOL	2936	41.890	31.900	-0.570	1.00	0.00
ATOM	9932	HW2	SOL	2936	41.670	31.830	0.920	1.00	0.00
ATOM	9933	OW	SOL	2937	31.500	51.190	31.720	1.00	0.00
ATOM	9934	HW1	SOL	2937	32.370	51.420	31.390	1.00	0.00
ATOM	9935	HW2	SOL	2937	31.630	50.360	32.180	1.00	0.00
ATOM	9936	OW	SOL	2938	52.370	46.660	10.750	1.00	0.00
ATOM	9937	HW1	SOL	2938	51.850	46.660	9.950	1.00	0.00
ATOM	9938	HW2	SOL	2938	52.690	47.550	10.830	1.00	0.00
ATOM	9939	OW	SOL	2939	34.990	55.410	55.750	1.00	0.00
ATOM	9940	HW1	SOL	2939	35.270	55.200	54.860	1.00	0.00
ATOM	9941	HW2	SOL	2939	35.180	56.350	55.850	1.00	0.00
ATOM	9942	OW	SOL	2940	33.090	29.890	55.910	1.00	0.00
ATOM	9943	HW1	SOL	2940	33.470	30.590	56.440	1.00	0.00

ATOM	9944	HW2	SOL	2940	33.750	29.210	55.890	1.00	0.00
ATOM	9945	OW	SOL	2941	7.450	26.210	7.740	1.00	0.00
ATOM	9946	HW1	SOL	2941	6.900	26.680	8.370	1.00	0.00
ATOM	9947	HW2	SOL	2941	7.390	25.290	8.030	1.00	0.00
ATOM	9948	OW	SOL	2942	37.430	17.390	55.650	1.00	0.00
ATOM	9949	HW1	SOL	2942	37.640	16.460	55.660	1.00	0.00
ATOM	9950	HW2	SOL	2942	36.770	17.490	54.960	1.00	0.00
ATOM	9951	OW	SOL	2943	18.700	50.750	34.090	1.00	0.00
ATOM	9952	HW1	SOL	2943	18.120	50.450	34.800	1.00	0.00
ATOM	9953	HW2	SOL	2943	19.470	51.080	34.540	1.00	0.00
ATOM	9954	OW	SOL	2944	17.030	6.840	34.920	1.00	0.00
ATOM	9955	HW1	SOL	2944	17.020	6.600	35.850	1.00	0.00
ATOM	9956	HW2	SOL	2944	17.960	6.820	34.680	1.00	0.00
ATOM	9957	OW	SOL	2945	29.910	0.190	54.100	1.00	0.00
ATOM	9958	HW1	SOL	2945	29.250	0.010	54.770	1.00	0.00
ATOM	9959	HW2	SOL	2945	30.410	0.930	54.460	1.00	0.00
ATOM	9960	OW	SOL	2946	8.410	18.120	32.870	1.00	0.00
ATOM	9961	HW1	SOL	2946	7.830	18.880	32.740	1.00	0.00
ATOM	9962	HW2	SOL	2946	9.240	18.500	33.150	1.00	0.00
ATOM	9963	OW	SOL	2947	39.280	12.940	35.090	1.00	0.00
ATOM	9964	HW1	SOL	2947	40.220	12.890	34.940	1.00	0.00
ATOM	9965	HW2	SOL	2947	39.170	12.660	36.000	1.00	0.00
ATOM	9966	OW	SOL	2948	6.550	26.460	50.460	1.00	0.00
ATOM	9967	HW1	SOL	2948	6.000	25.970	51.070	1.00	0.00
ATOM	9968	HW2	SOL	2948	5.990	26.580	49.690	1.00	0.00
ATOM	9969	OW	SOL	2949	8.060	1.280	2.230	1.00	0.00
ATOM	9970	HW1	SOL	2949	8.310	0.530	1.700	1.00	0.00
ATOM	9971	HW2	SOL	2949	7.130	1.140	2.430	1.00	0.00
ATOM	9972	OW	SOL	2950	27.540	27.110	4.800	1.00	0.00
ATOM	9973	HW1	SOL	2950	27.290	27.860	5.330	1.00	0.00
ATOM	9974	HW2	SOL	2950	26.740	26.600	4.700	1.00	0.00
ATOM	9975	OW	SOL	2951	52.260	55.210	11.060	1.00	0.00
ATOM	9976	HW1	SOL	2951	52.970	55.770	11.360	1.00	0.00
ATOM	9977	HW2	SOL	2951	51.460	55.680	11.290	1.00	0.00
ATOM	9978	OW	SOL	2952	53.620	27.680	7.700	1.00	0.00
ATOM	9979	HW1	SOL	2952	52.900	27.760	8.330	1.00	0.00
ATOM	9980	HW2	SOL	2952	54.270	28.320	8.000	1.00	0.00
ATOM	9981	OW	SOL	2953	22.320	43.840	44.120	1.00	0.00
ATOM	9982	HW1	SOL	2953	21.720	43.250	44.580	1.00	0.00
ATOM	9983	HW2	SOL	2953	22.830	43.270	43.550	1.00	0.00
ATOM	9984	OW	SOL	2954	31.010	6.550	47.220	1.00	0.00
ATOM	9985	HW1	SOL	2954	30.610	7.370	47.510	1.00	0.00
ATOM	9986	HW2	SOL	2954	30.260	5.990	46.990	1.00	0.00
ATOM	9987	OW	SOL	2955	35.900	39.750	51.040	1.00	0.00

ATOM	9988	HW1	SOL	2955	35.100	40.180	51.340	1.00	0.00
ATOM	9989	HW2	SOL	2955	36.210	40.300	50.320	1.00	0.00
ATOM	9990	OW	SOL	2956	9.330	8.750	38.300	1.00	0.00
ATOM	9991	HW1	SOL	2956	8.620	8.500	37.710	1.00	0.00
ATOM	9992	HW2	SOL	2956	9.570	7.940	38.750	1.00	0.00
ATOM	9993	OW	SOL	2957	3.540	0.480	38.460	1.00	0.00
ATOM	9994	HW1	SOL	2957	3.540	-0.160	37.740	1.00	0.00
ATOM	9995	HW2	SOL	2957	3.900	1.270	38.070	1.00	0.00
ATOM	9996	OW	SOL	2958	37.680	53.160	3.010	1.00	0.00
ATOM	9997	HW1	SOL	2958	38.150	53.810	2.480	1.00	0.00
ATOM	9998	HW2	SOL	2958	36.750	53.340	2.840	1.00	0.00
ATOM	9999	OW	SOL	2959	8.540	17.940	18.870	1.00	0.00
ATOM	10000	HW1	SOL	2959	9.420	17.790	18.530	1.00	0.00
ATOM	10001	HW2	SOL	2959	8.120	18.510	18.220	1.00	0.00
ATOM	10002	OW	SOL	2960	4.620	25.250	33.220	1.00	0.00
ATOM	10003	HW1	SOL	2960	3.690	25.020	33.250	1.00	0.00
ATOM	10004	HW2	SOL	2960	4.630	26.200	33.270	1.00	0.00
ATOM	10005	OW	SOL	2961	18.470	23.080	46.500	1.00	0.00
ATOM	10006	HW1	SOL	2961	17.620	23.220	46.920	1.00	0.00
ATOM	10007	HW2	SOL	2961	18.800	23.950	46.300	1.00	0.00
ATOM	10008	OW	SOL	2962	21.930	25.400	18.220	1.00	0.00
ATOM	10009	HW1	SOL	2962	21.660	24.860	17.480	1.00	0.00
ATOM	10010	HW2	SOL	2962	21.710	26.300	17.950	1.00	0.00
ATOM	10011	OW	SOL	2963	47.550	3.070	16.950	1.00	0.00
ATOM	10012	HW1	SOL	2963	47.360	3.990	16.780	1.00	0.00
ATOM	10013	HW2	SOL	2963	47.440	2.970	17.890	1.00	0.00
ATOM	10014	OW	SOL	2964	45.650	21.770	48.130	1.00	0.00
ATOM	10015	HW1	SOL	2964	45.070	21.850	48.890	1.00	0.00
ATOM	10016	HW2	SOL	2964	46.370	21.220	48.430	1.00	0.00
ATOM	10017	OW	SOL	2965	46.900	0.160	40.840	1.00	0.00
ATOM	10018	HW1	SOL	2965	46.720	-0.300	41.660	1.00	0.00
ATOM	10019	HW2	SOL	2965	47.820	0.410	40.910	1.00	0.00
ATOM	10020	OW	SOL	2966	10.510	28.960	25.930	1.00	0.00
ATOM	10021	HW1	SOL	2966	10.670	29.830	26.290	1.00	0.00
ATOM	10022	HW2	SOL	2966	11.100	28.900	25.180	1.00	0.00
ATOM	10023	OW	SOL	2967	25.700	33.930	25.960	1.00	0.00
ATOM	10024	HW1	SOL	2967	25.340	33.700	26.810	1.00	0.00
ATOM	10025	HW2	SOL	2967	24.930	34.090	25.410	1.00	0.00
ATOM	10026	OW	SOL	2968	13.500	42.380	11.730	1.00	0.00
ATOM	10027	HW1	SOL	2968	14.440	42.410	11.550	1.00	0.00
ATOM	10028	HW2	SOL	2968	13.370	43.040	12.400	1.00	0.00
ATOM	10029	OW	SOL	2969	2.050	55.210	17.610	1.00	0.00
ATOM	10030	HW1	SOL	2969	1.130	55.440	17.680	1.00	0.00
ATOM	10031	HW2	SOL	2969	2.120	54.760	16.770	1.00	0.00

ATOM	10032	OW	SOL	2970	11.140	7.600	17.810	1.00	0.00
ATOM	10033	HW1	SOL	2970	11.010	7.790	18.740	1.00	0.00
ATOM	10034	HW2	SOL	2970	10.270	7.730	17.420	1.00	0.00
ATOM	10035	OW	SOL	2971	46.710	46.740	0.240	1.00	0.00
ATOM	10036	HW1	SOL	2971	47.370	47.420	0.280	1.00	0.00
ATOM	10037	HW2	SOL	2971	47.200	45.920	0.310	1.00	0.00
ATOM	10038	OW	SOL	2972	7.550	28.860	17.010	1.00	0.00
ATOM	10039	HW1	SOL	2972	6.850	28.230	17.170	1.00	0.00
ATOM	10040	HW2	SOL	2972	8.350	28.390	17.250	1.00	0.00
ATOM	10041	OW	SOL	2973	24.510	13.650	42.080	1.00	0.00
ATOM	10042	HW1	SOL	2973	25.030	12.860	41.970	1.00	0.00
ATOM	10043	HW2	SOL	2973	23.680	13.460	41.660	1.00	0.00
ATOM	10044	OW	SOL	2974	12.260	20.540	45.790	1.00	0.00
ATOM	10045	HW1	SOL	2974	13.180	20.310	45.810	1.00	0.00
ATOM	10046	HW2	SOL	2974	12.150	21.050	44.990	1.00	0.00
ATOM	10047	OW	SOL	2975	30.610	3.470	15.440	1.00	0.00
ATOM	10048	HW1	SOL	2975	31.230	3.110	14.800	1.00	0.00
ATOM	10049	HW2	SOL	2975	30.390	4.330	15.100	1.00	0.00
ATOM	10050	OW	SOL	2976	52.310	24.150	18.530	1.00	0.00
ATOM	10051	HW1	SOL	2976	51.380	23.930	18.550	1.00	0.00
ATOM	10052	HW2	SOL	2976	52.340	25.090	18.680	1.00	0.00
ATOM	10053	OW	SOL	2977	32.980	27.870	19.630	1.00	0.00
ATOM	10054	HW1	SOL	2977	32.110	27.730	19.270	1.00	0.00
ATOM	10055	HW2	SOL	2977	33.420	28.450	19.000	1.00	0.00
ATOM	10056	OW	SOL	2978	11.550	25.370	38.610	1.00	0.00
ATOM	10057	HW1	SOL	2978	11.870	26.250	38.750	1.00	0.00
ATOM	10058	HW2	SOL	2978	10.980	25.430	37.840	1.00	0.00
ATOM	10059	OW	SOL	2979	43.830	24.540	46.780	1.00	0.00
ATOM	10060	HW1	SOL	2979	44.690	24.130	46.900	1.00	0.00
ATOM	10061	HW2	SOL	2979	43.750	24.670	45.840	1.00	0.00
ATOM	10062	OW	SOL	2980	27.540	27.170	17.430	1.00	0.00
ATOM	10063	HW1	SOL	2980	27.510	28.070	17.760	1.00	0.00
ATOM	10064	HW2	SOL	2980	26.620	26.950	17.270	1.00	0.00
ATOM	10065	OW	SOL	2981	14.290	47.180	40.620	1.00	0.00
ATOM	10066	HW1	SOL	2981	15.020	46.710	41.030	1.00	0.00
ATOM	10067	HW2	SOL	2981	14.190	47.970	41.160	1.00	0.00
ATOM	10068	OW	SOL	2982	54.110	12.840	25.450	1.00	0.00
ATOM	10069	HW1	SOL	2982	54.620	13.110	26.210	1.00	0.00
ATOM	10070	HW2	SOL	2982	54.740	12.760	24.740	1.00	0.00
ATOM	10071	OW	SOL	2983	29.360	29.670	49.430	1.00	0.00
ATOM	10072	HW1	SOL	2983	29.500	29.980	50.330	1.00	0.00
ATOM	10073	HW2	SOL	2983	30.240	29.560	49.080	1.00	0.00
ATOM	10074	OW	SOL	2984	17.520	2.460	8.820	1.00	0.00
ATOM	10075	HW1	SOL	2984	17.490	3.410	8.850	1.00	0.00

ATOM	10076	HW2	SOL	2984	17.880	2.190	9.670	1.00	0.00
ATOM	10077	OW	SOL	2985	19.120	48.640	24.990	1.00	0.00
ATOM	10078	HW1	SOL	2985	18.980	49.180	25.780	1.00	0.00
ATOM	10079	HW2	SOL	2985	20.060	48.720	24.820	1.00	0.00
ATOM	10080	OW	SOL	2986	11.200	8.210	44.760	1.00	0.00
ATOM	10081	HW1	SOL	2986	11.900	8.830	45.000	1.00	0.00
ATOM	10082	HW2	SOL	2986	11.200	7.580	45.490	1.00	0.00
ATOM	10083	OW	SOL	2987	21.750	25.990	34.390	1.00	0.00
ATOM	10084	HW1	SOL	2987	21.580	26.540	35.160	1.00	0.00
ATOM	10085	HW2	SOL	2987	22.120	25.180	34.750	1.00	0.00
ATOM	10086	OW	SOL	2988	21.200	12.060	33.150	1.00	0.00
ATOM	10087	HW1	SOL	2988	21.770	11.440	32.710	1.00	0.00
ATOM	10088	HW2	SOL	2988	20.420	12.110	32.590	1.00	0.00
ATOM	10089	OW	SOL	2989	27.580	34.620	46.440	1.00	0.00
ATOM	10090	HW1	SOL	2989	27.680	34.110	47.240	1.00	0.00
ATOM	10091	HW2	SOL	2989	27.960	35.470	46.650	1.00	0.00
ATOM	10092	OW	SOL	2990	22.500	32.090	49.870	1.00	0.00
ATOM	10093	HW1	SOL	2990	21.610	32.440	49.900	1.00	0.00
ATOM	10094	HW2	SOL	2990	23.050	32.820	50.170	1.00	0.00
ATOM	10095	OW	SOL	2991	27.670	19.940	48.890	1.00	0.00
ATOM	10096	HW1	SOL	2991	27.820	20.180	47.980	1.00	0.00
ATOM	10097	HW2	SOL	2991	28.410	20.300	49.360	1.00	0.00
ATOM	10098	OW	SOL	2992	49.270	26.520	14.360	1.00	0.00
ATOM	10099	HW1	SOL	2992	48.420	26.510	14.810	1.00	0.00
ATOM	10100	HW2	SOL	2992	49.890	26.190	15.020	1.00	0.00
ATOM	10101	OW	SOL	2993	55.090	6.430	3.090	1.00	0.00
ATOM	10102	HW1	SOL	2993	55.960	6.050	3.270	1.00	0.00
ATOM	10103	HW2	SOL	2993	55.200	6.930	2.290	1.00	0.00
ATOM	10104	OW	SOL	2994	45.660	43.390	18.650	1.00	0.00
ATOM	10105	HW1	SOL	2994	45.620	42.430	18.570	1.00	0.00
ATOM	10106	HW2	SOL	2994	45.310	43.570	19.520	1.00	0.00
ATOM	10107	OW	SOL	2995	46.090	17.620	26.820	1.00	0.00
ATOM	10108	HW1	SOL	2995	46.190	17.180	27.670	1.00	0.00
ATOM	10109	HW2	SOL	2995	46.850	17.350	26.320	1.00	0.00
ATOM	10110	OW	SOL	2996	31.730	47.820	30.040	1.00	0.00
ATOM	10111	HW1	SOL	2996	31.810	48.040	30.960	1.00	0.00
ATOM	10112	HW2	SOL	2996	30.800	47.950	29.840	1.00	0.00
ATOM	10113	OW	SOL	2997	40.820	54.460	1.490	1.00	0.00
ATOM	10114	HW1	SOL	2997	41.640	54.050	1.220	1.00	0.00
ATOM	10115	HW2	SOL	2997	40.950	54.670	2.420	1.00	0.00
ATOM	10116	OW	SOL	2998	44.960	45.870	22.530	1.00	0.00
ATOM	10117	HW1	SOL	2998	45.590	46.470	22.930	1.00	0.00
ATOM	10118	HW2	SOL	2998	44.130	46.340	22.550	1.00	0.00
ATOM	10119	OW	SOL	2999	42.160	33.990	2.150	1.00	0.00

ATOM	10120	HW1	SOL	2999	42.170	34.150	3.090	1.00	0.00
ATOM	10121	HW2	SOL	2999	41.240	33.930	1.920	1.00	0.00
ATOM	10122	OW	SOL	3000	24.410	10.440	45.540	1.00	0.00
ATOM	10123	HW1	SOL	3000	24.430	10.800	44.650	1.00	0.00
ATOM	10124	HW2	SOL	3000	24.620	11.200	46.090	1.00	0.00
ATOM	10125	OW	SOL	3001	15.120	18.160	22.770	1.00	0.00
ATOM	10126	HW1	SOL	3001	14.740	17.300	22.930	1.00	0.00
ATOM	10127	HW2	SOL	3001	16.040	18.060	23.010	1.00	0.00
ATOM	10128	OW	SOL	3002	1.490	1.110	43.050	1.00	0.00
ATOM	10129	HW1	SOL	3002	1.720	0.190	43.180	1.00	0.00
ATOM	10130	HW2	SOL	3002	1.350	1.190	42.110	1.00	0.00
ATOM	10131	OW	SOL	3003	10.520	37.720	5.980	1.00	0.00
ATOM	10132	HW1	SOL	3003	9.750	38.010	6.470	1.00	0.00
ATOM	10133	HW2	SOL	3003	11.250	37.870	6.580	1.00	0.00
ATOM	10134	OW	SOL	3004	17.930	43.410	44.290	1.00	0.00
ATOM	10135	HW1	SOL	3004	18.070	44.290	44.640	1.00	0.00
ATOM	10136	HW2	SOL	3004	18.310	42.820	44.950	1.00	0.00
ATOM	10137	OW	SOL	3005	1.250	54.400	10.650	1.00	0.00
ATOM	10138	HW1	SOL	3005	2.100	54.200	10.260	1.00	0.00
ATOM	10139	HW2	SOL	3005	0.750	53.590	10.560	1.00	0.00
ATOM	10140	OW	SOL	3006	25.680	1.410	21.700	1.00	0.00
ATOM	10141	HW1	SOL	3006	25.290	0.920	20.980	1.00	0.00
ATOM	10142	HW2	SOL	3006	26.610	1.160	21.680	1.00	0.00
ATOM	10143	OW	SOL	3007	1.280	11.720	55.180	1.00	0.00
ATOM	10144	HW1	SOL	3007	0.720	11.860	55.950	1.00	0.00
ATOM	10145	HW2	SOL	3007	0.810	11.070	54.660	1.00	0.00
ATOM	10146	OW	SOL	3008	20.590	12.310	52.560	1.00	0.00
ATOM	10147	HW1	SOL	3008	19.960	12.290	53.280	1.00	0.00
ATOM	10148	HW2	SOL	3008	20.530	11.440	52.160	1.00	0.00
ATOM	10149	OW	SOL	3009	37.350	4.440	4.740	1.00	0.00
ATOM	10150	HW1	SOL	3009	37.590	3.760	4.120	1.00	0.00
ATOM	10151	HW2	SOL	3009	37.130	3.970	5.540	1.00	0.00
ATOM	10152	OW	SOL	3010	35.560	36.260	15.770	1.00	0.00
ATOM	10153	HW1	SOL	3010	35.010	35.870	15.090	1.00	0.00
ATOM	10154	HW2	SOL	3010	35.760	37.140	15.440	1.00	0.00
ATOM	10155	OW	SOL	3011	36.530	10.180	43.900	1.00	0.00
ATOM	10156	HW1	SOL	3011	35.940	10.930	43.930	1.00	0.00
ATOM	10157	HW2	SOL	3011	36.500	9.880	42.990	1.00	0.00
ATOM	10158	OW	SOL	3012	44.960	13.030	27.550	1.00	0.00
ATOM	10159	HW1	SOL	3012	44.420	13.610	28.080	1.00	0.00
ATOM	10160	HW2	SOL	3012	44.420	12.850	26.780	1.00	0.00
ATOM	10161	OW	SOL	3013	23.150	53.170	35.990	1.00	0.00
ATOM	10162	HW1	SOL	3013	22.960	53.790	35.280	1.00	0.00
ATOM	10163	HW2	SOL	3013	23.420	52.370	35.530	1.00	0.00

ATOM	10164	OW	SOL	3014	31.930	19.340	35.160	1.00	0.00
ATOM	10165	HW1	SOL	3014	32.680	19.010	35.660	1.00	0.00
ATOM	10166	HW2	SOL	3014	32.050	20.290	35.130	1.00	0.00
ATOM	10167	OW	SOL	3015	33.390	21.330	50.370	1.00	0.00
ATOM	10168	HW1	SOL	3015	33.420	22.130	49.860	1.00	0.00
ATOM	10169	HW2	SOL	3015	33.760	20.660	49.790	1.00	0.00
ATOM	10170	OW	SOL	3016	49.930	43.090	34.370	1.00	0.00
ATOM	10171	HW1	SOL	3016	49.980	43.570	33.540	1.00	0.00
ATOM	10172	HW2	SOL	3016	50.540	42.360	34.260	1.00	0.00
ATOM	10173	OW	SOL	3017	0.100	12.900	17.860	1.00	0.00
ATOM	10174	HW1	SOL	3017	0.440	12.700	18.730	1.00	0.00
ATOM	10175	HW2	SOL	3017	-0.040	13.840	17.860	1.00	0.00
ATOM	10176	OW	SOL	3018	14.060	34.710	46.640	1.00	0.00
ATOM	10177	HW1	SOL	3018	13.310	34.130	46.550	1.00	0.00
ATOM	10178	HW2	SOL	3018	14.640	34.260	47.250	1.00	0.00
ATOM	10179	OW	SOL	3019	47.230	33.770	4.430	1.00	0.00
ATOM	10180	HW1	SOL	3019	47.250	32.840	4.650	1.00	0.00
ATOM	10181	HW2	SOL	3019	46.310	34.000	4.410	1.00	0.00
ATOM	10182	OW	SOL	3020	3.790	8.200	10.200	1.00	0.00
ATOM	10183	HW1	SOL	3020	3.180	7.610	9.750	1.00	0.00
ATOM	10184	HW2	SOL	3020	3.230	8.770	10.720	1.00	0.00
ATOM	10185	OW	SOL	3021	34.090	55.670	36.520	1.00	0.00
ATOM	10186	HW1	SOL	3021	35.000	55.810	36.260	1.00	0.00
ATOM	10187	HW2	SOL	3021	33.760	56.540	36.730	1.00	0.00
ATOM	10188	OW	SOL	3022	26.180	22.370	27.960	1.00	0.00
ATOM	10189	HW1	SOL	3022	25.930	21.570	28.420	1.00	0.00
ATOM	10190	HW2	SOL	3022	25.450	22.960	28.090	1.00	0.00
ATOM	10191	OW	SOL	3023	46.200	7.340	50.820	1.00	0.00
ATOM	10192	HW1	SOL	3023	47.100	7.650	50.860	1.00	0.00
ATOM	10193	HW2	SOL	3023	46.280	6.380	50.800	1.00	0.00
ATOM	10194	OW	SOL	3024	34.940	2.900	29.350	1.00	0.00
ATOM	10195	HW1	SOL	3024	34.480	3.360	28.650	1.00	0.00
ATOM	10196	HW2	SOL	3024	34.270	2.330	29.740	1.00	0.00
ATOM	10197	OW	SOL	3025	20.780	22.980	21.360	1.00	0.00
ATOM	10198	HW1	SOL	3025	21.120	22.380	22.020	1.00	0.00
ATOM	10199	HW2	SOL	3025	21.130	22.650	20.530	1.00	0.00
ATOM	10200	OW	SOL	3026	53.780	37.160	24.270	1.00	0.00
ATOM	10201	HW1	SOL	3026	53.620	36.530	23.560	1.00	0.00
ATOM	10202	HW2	SOL	3026	52.970	37.650	24.350	1.00	0.00
ATOM	10203	OW	SOL	3027	12.290	33.640	13.890	1.00	0.00
ATOM	10204	HW1	SOL	3027	12.340	33.590	14.850	1.00	0.00
ATOM	10205	HW2	SOL	3027	12.790	34.430	13.670	1.00	0.00
ATOM	10206	OW	SOL	3028	45.930	0.780	50.350	1.00	0.00
ATOM	10207	HW1	SOL	3028	45.090	1.160	50.590	1.00	0.00

ATOM	10208	HW2	SOL	3028	45.720	-0.100	50.040	1.00	0.00
ATOM	10209	OW	SOL	3029	31.400	46.270	25.860	1.00	0.00
ATOM	10210	HW1	SOL	3029	30.900	45.930	25.110	1.00	0.00
ATOM	10211	HW2	SOL	3029	31.730	45.500	26.300	1.00	0.00
ATOM	10212	OW	SOL	3030	41.230	43.760	10.450	1.00	0.00
ATOM	10213	HW1	SOL	3030	42.060	44.190	10.220	1.00	0.00
ATOM	10214	HW2	SOL	3030	41.010	43.230	9.690	1.00	0.00
ATOM	10215	OW	SOL	3031	26.540	49.430	55.220	1.00	0.00
ATOM	10216	HW1	SOL	3031	26.890	50.220	55.620	1.00	0.00
ATOM	10217	HW2	SOL	3031	27.310	48.930	54.940	1.00	0.00
ATOM	10218	OW	SOL	3032	47.930	33.830	1.590	1.00	0.00
ATOM	10219	HW1	SOL	3032	48.010	33.820	2.550	1.00	0.00
ATOM	10220	HW2	SOL	3032	48.650	33.290	1.280	1.00	0.00
ATOM	10221	OW	SOL	3033	11.670	36.250	1.590	1.00	0.00
ATOM	10222	HW1	SOL	3033	12.560	36.580	1.730	1.00	0.00
ATOM	10223	HW2	SOL	3033	11.270	36.260	2.460	1.00	0.00
ATOM	10224	OW	SOL	3034	22.860	9.220	41.400	1.00	0.00
ATOM	10225	HW1	SOL	3034	22.750	8.980	40.480	1.00	0.00
ATOM	10226	HW2	SOL	3034	22.280	8.630	41.880	1.00	0.00
ATOM	10227	OW	SOL	3035	40.190	34.400	7.050	1.00	0.00
ATOM	10228	HW1	SOL	3035	40.270	34.770	7.930	1.00	0.00
ATOM	10229	HW2	SOL	3035	39.330	33.980	7.050	1.00	0.00
ATOM	10230	OW	SOL	3036	12.930	30.990	38.960	1.00	0.00
ATOM	10231	HW1	SOL	3036	13.570	30.280	38.870	1.00	0.00
ATOM	10232	HW2	SOL	3036	13.190	31.640	38.300	1.00	0.00
ATOM	10233	OW	SOL	3037	5.260	15.230	24.400	1.00	0.00
ATOM	10234	HW1	SOL	3037	5.830	14.730	23.820	1.00	0.00
ATOM	10235	HW2	SOL	3037	5.080	14.640	25.140	1.00	0.00
ATOM	10236	OW	SOL	3038	19.230	55.600	25.220	1.00	0.00
ATOM	10237	HW1	SOL	3038	18.670	56.040	24.580	1.00	0.00
ATOM	10238	HW2	SOL	3038	18.660	55.430	25.970	1.00	0.00
ATOM	10239	OW	SOL	3039	19.090	10.090	50.890	1.00	0.00
ATOM	10240	HW1	SOL	3039	18.600	10.530	50.200	1.00	0.00
ATOM	10241	HW2	SOL	3039	19.970	9.970	50.540	1.00	0.00
ATOM	10242	OW	SOL	3040	32.820	11.160	39.660	1.00	0.00
ATOM	10243	HW1	SOL	3040	32.990	10.610	40.420	1.00	0.00
ATOM	10244	HW2	SOL	3040	33.500	11.840	39.690	1.00	0.00
ATOM	10245	OW	SOL	3041	13.600	13.110	0.440	1.00	0.00
ATOM	10246	HW1	SOL	3041	14.230	13.100	1.160	1.00	0.00
ATOM	10247	HW2	SOL	3041	12.910	12.510	0.710	1.00	0.00
ATOM	10248	OW	SOL	3042	30.450	30.130	46.320	1.00	0.00
ATOM	10249	HW1	SOL	3042	30.640	29.470	45.660	1.00	0.00
ATOM	10250	HW2	SOL	3042	30.480	29.660	47.150	1.00	0.00
ATOM	10251	OW	SOL	3043	46.170	15.380	2.240	1.00	0.00

ATOM	10252	HW1	SOL	3043	46.640	15.800	1.520	1.00	0.00
ATOM	10253	HW2	SOL	3043	45.570	16.050	2.570	1.00	0.00
ATOM	10254	OW	SOL	3044	11.310	17.930	9.250	1.00	0.00
ATOM	10255	HW1	SOL	3044	11.780	17.490	9.960	1.00	0.00
ATOM	10256	HW2	SOL	3044	11.520	17.420	8.470	1.00	0.00
ATOM	10257	OW	SOL	3045	18.070	6.560	37.540	1.00	0.00
ATOM	10258	HW1	SOL	3045	18.000	5.760	38.060	1.00	0.00
ATOM	10259	HW2	SOL	3045	18.830	7.020	37.900	1.00	0.00
ATOM	10260	OW	SOL	3046	30.810	4.930	55.030	1.00	0.00
ATOM	10261	HW1	SOL	3046	31.680	4.830	54.620	1.00	0.00
ATOM	10262	HW2	SOL	3046	30.710	4.130	55.550	1.00	0.00
ATOM	10263	OW	SOL	3047	16.730	31.050	49.690	1.00	0.00
ATOM	10264	HW1	SOL	3047	16.200	31.840	49.600	1.00	0.00
ATOM	10265	HW2	SOL	3047	16.110	30.370	49.940	1.00	0.00
ATOM	10266	OW	SOL	3048	29.400	16.500	3.020	1.00	0.00
ATOM	10267	HW1	SOL	3048	29.930	16.440	2.220	1.00	0.00
ATOM	10268	HW2	SOL	3048	28.920	15.670	3.050	1.00	0.00
ATOM	10269	OW	SOL	3049	12.910	53.660	9.590	1.00	0.00
ATOM	10270	HW1	SOL	3049	13.410	52.960	9.170	1.00	0.00
ATOM	10271	HW2	SOL	3049	13.160	54.460	9.120	1.00	0.00
ATOM	10272	OW	SOL	3050	50.380	32.240	19.190	1.00	0.00
ATOM	10273	HW1	SOL	3050	49.510	32.040	19.540	1.00	0.00
ATOM	10274	HW2	SOL	3050	50.300	33.120	18.850	1.00	0.00
ATOM	10275	OW	SOL	3051	14.150	27.850	8.670	1.00	0.00
ATOM	10276	HW1	SOL	3051	14.190	27.480	7.780	1.00	0.00
ATOM	10277	HW2	SOL	3051	15.030	28.210	8.810	1.00	0.00
ATOM	10278	OW	SOL	3052	48.220	22.230	42.890	1.00	0.00
ATOM	10279	HW1	SOL	3052	49.120	22.320	43.220	1.00	0.00
ATOM	10280	HW2	SOL	3052	47.730	22.920	43.330	1.00	0.00
ATOM	10281	OW	SOL	3053	14.250	18.830	42.650	1.00	0.00
ATOM	10282	HW1	SOL	3053	14.710	19.600	42.980	1.00	0.00
ATOM	10283	HW2	SOL	3053	14.390	18.860	41.700	1.00	0.00
ATOM	10284	OW	SOL	3054	1.510	14.240	41.900	1.00	0.00
ATOM	10285	HW1	SOL	3054	0.850	14.920	42.030	1.00	0.00
ATOM	10286	HW2	SOL	3054	2.350	14.690	42.050	1.00	0.00
ATOM	10287	OW	SOL	3055	17.660	21.280	25.530	1.00	0.00
ATOM	10288	HW1	SOL	3055	17.860	22.220	25.610	1.00	0.00
ATOM	10289	HW2	SOL	3055	17.370	21.180	24.620	1.00	0.00
ATOM	10290	OW	SOL	3056	33.420	47.100	43.510	1.00	0.00
ATOM	10291	HW1	SOL	3056	33.110	47.990	43.340	1.00	0.00
ATOM	10292	HW2	SOL	3056	34.250	47.210	43.970	1.00	0.00
ATOM	10293	OW	SOL	3057	41.330	35.080	53.040	1.00	0.00
ATOM	10294	HW1	SOL	3057	40.840	35.080	53.870	1.00	0.00
ATOM	10295	HW2	SOL	3057	41.570	36.000	52.910	1.00	0.00

ATOM	10296	OW	SOL	3058	41.810	1.650	45.440	1.00	0.00
ATOM	10297	HW1	SOL	3058	41.450	2.500	45.190	1.00	0.00
ATOM	10298	HW2	SOL	3058	41.050	1.140	45.700	1.00	0.00
ATOM	10299	OW	SOL	3059	28.220	50.280	12.290	1.00	0.00
ATOM	10300	HW1	SOL	3059	27.800	49.450	12.050	1.00	0.00
ATOM	10301	HW2	SOL	3059	29.130	50.050	12.480	1.00	0.00
ATOM	10302	OW	SOL	3060	4.110	21.700	23.980	1.00	0.00
ATOM	10303	HW1	SOL	3060	4.010	22.600	24.280	1.00	0.00
ATOM	10304	HW2	SOL	3060	3.660	21.170	24.650	1.00	0.00
ATOM	10305	OW	SOL	3061	53.250	35.890	21.560	1.00	0.00
ATOM	10306	HW1	SOL	3061	53.830	35.150	21.360	1.00	0.00
ATOM	10307	HW2	SOL	3061	53.570	36.590	20.990	1.00	0.00
ATOM	10308	OW	SOL	3062	7.180	40.770	15.560	1.00	0.00
ATOM	10309	HW1	SOL	3062	7.210	41.300	16.360	1.00	0.00
ATOM	10310	HW2	SOL	3062	6.610	40.030	15.780	1.00	0.00
ATOM	10311	OW	SOL	3063	28.280	16.720	17.600	1.00	0.00
ATOM	10312	HW1	SOL	3063	28.440	16.490	18.520	1.00	0.00
ATOM	10313	HW2	SOL	3063	28.910	17.420	17.420	1.00	0.00
ATOM	10314	OW	SOL	3064	14.720	20.970	9.720	1.00	0.00
ATOM	10315	HW1	SOL	3064	14.060	20.800	9.050	1.00	0.00
ATOM	10316	HW2	SOL	3064	14.220	21.260	10.480	1.00	0.00
ATOM	10317	OW	SOL	3065	4.610	42.910	6.290	1.00	0.00
ATOM	10318	HW1	SOL	3065	5.530	42.800	6.540	1.00	0.00
ATOM	10319	HW2	SOL	3065	4.120	42.490	6.990	1.00	0.00
ATOM	10320	OW	SOL	3066	29.150	5.220	40.380	1.00	0.00
ATOM	10321	HW1	SOL	3066	28.900	4.960	39.490	1.00	0.00
ATOM	10322	HW2	SOL	3066	29.090	4.400	40.890	1.00	0.00
ATOM	10323	OW	SOL	3067	51.040	52.440	4.040	1.00	0.00
ATOM	10324	HW1	SOL	3067	51.640	52.950	4.590	1.00	0.00
ATOM	10325	HW2	SOL	3067	51.570	52.200	3.270	1.00	0.00
ATOM	10326	OW	SOL	3068	5.860	42.720	41.840	1.00	0.00
ATOM	10327	HW1	SOL	3068	6.220	42.370	41.030	1.00	0.00
ATOM	10328	HW2	SOL	3068	6.610	42.860	42.410	1.00	0.00
ATOM	10329	OW	SOL	3069	7.230	42.840	7.100	1.00	0.00
ATOM	10330	HW1	SOL	3069	7.400	43.050	8.020	1.00	0.00
ATOM	10331	HW2	SOL	3069	8.070	42.540	6.760	1.00	0.00
ATOM	10332	OW	SOL	3070	41.320	46.650	8.650	1.00	0.00
ATOM	10333	HW1	SOL	3070	41.090	45.800	8.270	1.00	0.00
ATOM	10334	HW2	SOL	3070	40.660	47.250	8.300	1.00	0.00
ATOM	10335	OW	SOL	3071	55.180	30.350	10.210	1.00	0.00
ATOM	10336	HW1	SOL	3071	55.270	29.460	10.560	1.00	0.00
ATOM	10337	HW2	SOL	3071	55.530	30.310	9.330	1.00	0.00
ATOM	10338	OW	SOL	3072	48.480	4.730	40.940	1.00	0.00
ATOM	10339	HW1	SOL	3072	49.240	4.200	40.690	1.00	0.00

ATOM	10340	HW2	SOL	3072	47.740	4.320	40.490	1.00	0.00
ATOM	10341	OW	SOL	3073	17.410	26.070	9.890	1.00	0.00
ATOM	10342	HW1	SOL	3073	17.180	25.150	10.040	1.00	0.00
ATOM	10343	HW2	SOL	3073	17.610	26.410	10.760	1.00	0.00
ATOM	10344	OW	SOL	3074	24.770	32.270	55.000	1.00	0.00
ATOM	10345	HW1	SOL	3074	24.480	31.530	55.540	1.00	0.00
ATOM	10346	HW2	SOL	3074	23.960	32.600	54.600	1.00	0.00
ATOM	10347	OW	SOL	3075	18.740	48.300	36.490	1.00	0.00
ATOM	10348	HW1	SOL	3075	17.950	47.930	36.090	1.00	0.00
ATOM	10349	HW2	SOL	3075	18.520	49.210	36.680	1.00	0.00
ATOM	10350	OW	SOL	3076	18.930	38.380	2.730	1.00	0.00
ATOM	10351	HW1	SOL	3076	18.460	38.940	2.120	1.00	0.00
ATOM	10352	HW2	SOL	3076	18.400	37.580	2.780	1.00	0.00
ATOM	10353	OW	SOL	3077	34.880	1.590	16.400	1.00	0.00
ATOM	10354	HW1	SOL	3077	34.610	1.920	15.540	1.00	0.00
ATOM	10355	HW2	SOL	3077	35.630	2.130	16.640	1.00	0.00
ATOM	10356	OW	SOL	3078	6.810	51.920	11.930	1.00	0.00
ATOM	10357	HW1	SOL	3078	7.500	51.930	12.590	1.00	0.00
ATOM	10358	HW2	SOL	3078	7.270	51.940	11.090	1.00	0.00
ATOM	10359	OW	SOL	3079	14.520	5.600	39.590	1.00	0.00
ATOM	10360	HW1	SOL	3079	14.310	4.810	39.090	1.00	0.00
ATOM	10361	HW2	SOL	3079	14.340	6.320	38.990	1.00	0.00
ATOM	10362	OW	SOL	3080	10.310	42.330	16.920	1.00	0.00
ATOM	10363	HW1	SOL	3080	10.210	43.120	17.460	1.00	0.00
ATOM	10364	HW2	SOL	3080	9.950	42.580	16.070	1.00	0.00
ATOM	10365	OW	SOL	3081	37.750	22.840	7.850	1.00	0.00
ATOM	10366	HW1	SOL	3081	36.980	23.390	7.750	1.00	0.00
ATOM	10367	HW2	SOL	3081	38.330	23.350	8.420	1.00	0.00
ATOM	10368	OW	SOL	3082	28.430	0.630	26.350	1.00	0.00
ATOM	10369	HW1	SOL	3082	29.110	-0.030	26.490	1.00	0.00
ATOM	10370	HW2	SOL	3082	28.770	1.170	25.630	1.00	0.00
ATOM	10371	OW	SOL	3083	26.790	47.690	41.010	1.00	0.00
ATOM	10372	HW1	SOL	3083	27.440	48.270	41.420	1.00	0.00
ATOM	10373	HW2	SOL	3083	27.260	46.890	40.820	1.00	0.00
ATOM	10374	OW	SOL	3084	30.050	10.290	49.550	1.00	0.00
ATOM	10375	HW1	SOL	3084	30.150	10.300	48.600	1.00	0.00
ATOM	10376	HW2	SOL	3084	30.800	9.790	49.860	1.00	0.00
ATOM	10377	OW	SOL	3085	25.280	47.810	0.950	1.00	0.00
ATOM	10378	HW1	SOL	3085	25.700	48.530	0.480	1.00	0.00
ATOM	10379	HW2	SOL	3085	25.410	47.050	0.380	1.00	0.00
ATOM	10380	OW	SOL	3086	0.280	24.610	35.810	1.00	0.00
ATOM	10381	HW1	SOL	3086	-0.120	24.580	36.680	1.00	0.00
ATOM	10382	HW2	SOL	3086	1.210	24.720	35.980	1.00	0.00
ATOM	10383	OW	SOL	3087	19.290	42.310	41.780	1.00	0.00

ATOM	10384	HW1	SOL	3087	18.920	42.610	42.610	1.00	0.00
ATOM	10385	HW2	SOL	3087	19.500	41.390	41.930	1.00	0.00
ATOM	10386	OW	SOL	3088	21.210	22.820	40.170	1.00	0.00
ATOM	10387	HW1	SOL	3088	20.610	23.350	40.690	1.00	0.00
ATOM	10388	HW2	SOL	3088	21.080	21.930	40.490	1.00	0.00
ATOM	10389	OW	SOL	3089	10.820	25.470	55.310	1.00	0.00
ATOM	10390	HW1	SOL	3089	10.290	25.290	54.530	1.00	0.00
ATOM	10391	HW2	SOL	3089	10.430	24.920	55.990	1.00	0.00
ATOM	10392	OW	SOL	3090	7.380	38.800	44.430	1.00	0.00
ATOM	10393	HW1	SOL	3090	7.320	39.740	44.600	1.00	0.00
ATOM	10394	HW2	SOL	3090	8.050	38.710	43.760	1.00	0.00
ATOM	10395	OW	SOL	3091	33.860	5.100	9.420	1.00	0.00
ATOM	10396	HW1	SOL	3091	33.270	4.970	8.670	1.00	0.00
ATOM	10397	HW2	SOL	3091	34.450	4.340	9.400	1.00	0.00
ATOM	10398	OW	SOL	3092	1.220	2.600	33.110	1.00	0.00
ATOM	10399	HW1	SOL	3092	0.790	2.930	33.890	1.00	0.00
ATOM	10400	HW2	SOL	3092	0.770	3.030	32.380	1.00	0.00
ATOM	10401	OW	SOL	3093	23.830	44.640	41.690	1.00	0.00
ATOM	10402	HW1	SOL	3093	23.370	45.110	42.390	1.00	0.00
ATOM	10403	HW2	SOL	3093	24.290	45.320	41.200	1.00	0.00
ATOM	10404	OW	SOL	3094	35.130	51.920	51.620	1.00	0.00
ATOM	10405	HW1	SOL	3094	35.620	51.730	52.420	1.00	0.00
ATOM	10406	HW2	SOL	3094	35.610	52.630	51.200	1.00	0.00
ATOM	10407	OW	SOL	3095	30.650	9.660	47.090	1.00	0.00
ATOM	10408	HW1	SOL	3095	31.500	9.630	46.660	1.00	0.00
ATOM	10409	HW2	SOL	3095	30.020	9.400	46.420	1.00	0.00
ATOM	10410	OW	SOL	3096	21.990	48.070	49.280	1.00	0.00
ATOM	10411	HW1	SOL	3096	22.260	48.180	48.370	1.00	0.00
ATOM	10412	HW2	SOL	3096	22.810	48.020	49.770	1.00	0.00
ATOM	10413	OW	SOL	3097	6.010	3.700	24.000	1.00	0.00
ATOM	10414	HW1	SOL	3097	5.800	2.870	24.420	1.00	0.00
ATOM	10415	HW2	SOL	3097	5.700	4.370	24.620	1.00	0.00
ATOM	10416	OW	SOL	3098	51.070	43.300	19.710	1.00	0.00
ATOM	10417	HW1	SOL	3098	51.510	43.320	18.860	1.00	0.00
ATOM	10418	HW2	SOL	3098	50.370	42.660	19.610	1.00	0.00
ATOM	10419	OW	SOL	3099	24.000	48.320	10.150	1.00	0.00
ATOM	10420	HW1	SOL	3099	23.760	48.530	11.050	1.00	0.00
ATOM	10421	HW2	SOL	3099	23.970	47.360	10.120	1.00	0.00
ATOM	10422	OW	SOL	3100	39.180	31.000	12.070	1.00	0.00
ATOM	10423	HW1	SOL	3100	39.690	30.930	11.260	1.00	0.00
ATOM	10424	HW2	SOL	3100	38.910	30.110	12.260	1.00	0.00
ATOM	10425	OW	SOL	3101	11.300	15.350	39.110	1.00	0.00
ATOM	10426	HW1	SOL	3101	12.250	15.470	39.020	1.00	0.00
ATOM	10427	HW2	SOL	3101	10.950	15.550	38.250	1.00	0.00

ATOM	10428	OW	SOL	3102	10.010	20.200	14.360	1.00	0.00
ATOM	10429	HW1	SOL	3102	10.060	19.570	13.640	1.00	0.00
ATOM	10430	HW2	SOL	3102	9.790	21.030	13.940	1.00	0.00
ATOM	10431	OW	SOL	3103	29.770	12.070	4.000	1.00	0.00
ATOM	10432	HW1	SOL	3103	29.010	12.460	3.560	1.00	0.00
ATOM	10433	HW2	SOL	3103	30.250	12.810	4.340	1.00	0.00
ATOM	10434	OW	SOL	3104	30.380	54.790	30.740	1.00	0.00
ATOM	10435	HW1	SOL	3104	29.990	55.660	30.850	1.00	0.00
ATOM	10436	HW2	SOL	3104	30.270	54.370	31.590	1.00	0.00
ATOM	10437	OW	SOL	3105	50.080	30.090	51.640	1.00	0.00
ATOM	10438	HW1	SOL	3105	49.260	30.160	51.130	1.00	0.00
ATOM	10439	HW2	SOL	3105	49.990	30.750	52.320	1.00	0.00
ATOM	10440	OW	SOL	3106	37.150	22.690	37.290	1.00	0.00
ATOM	10441	HW1	SOL	3106	36.600	21.950	37.530	1.00	0.00
ATOM	10442	HW2	SOL	3106	36.900	22.890	36.390	1.00	0.00
ATOM	10443	OW	SOL	3107	52.920	10.620	15.510	1.00	0.00
ATOM	10444	HW1	SOL	3107	53.810	10.880	15.270	1.00	0.00
ATOM	10445	HW2	SOL	3107	52.550	10.250	14.710	1.00	0.00
ATOM	10446	OW	SOL	3108	11.650	40.970	10.010	1.00	0.00
ATOM	10447	HW1	SOL	3108	12.150	41.080	9.200	1.00	0.00
ATOM	10448	HW2	SOL	3108	12.180	41.390	10.680	1.00	0.00
ATOM	10449	OW	SOL	3109	26.820	52.390	55.540	1.00	0.00
ATOM	10450	HW1	SOL	3109	27.280	53.020	56.090	1.00	0.00
ATOM	10451	HW2	SOL	3109	26.400	52.920	54.860	1.00	0.00
ATOM	10452	OW	SOL	3110	9.030	29.530	14.780	1.00	0.00
ATOM	10453	HW1	SOL	3110	8.520	29.180	15.520	1.00	0.00
ATOM	10454	HW2	SOL	3110	9.480	30.290	15.130	1.00	0.00
ATOM	10455	OW	SOL	3111	24.860	10.920	42.680	1.00	0.00
ATOM	10456	HW1	SOL	3111	25.380	10.930	41.870	1.00	0.00
ATOM	10457	HW2	SOL	3111	24.070	10.420	42.450	1.00	0.00
ATOM	10458	OW	SOL	3112	20.620	23.610	37.620	1.00	0.00
ATOM	10459	HW1	SOL	3112	21.090	24.420	37.420	1.00	0.00
ATOM	10460	HW2	SOL	3112	21.020	23.300	38.430	1.00	0.00
ATOM	10461	OW	SOL	3113	23.950	46.310	13.570	1.00	0.00
ATOM	10462	HW1	SOL	3113	23.300	46.430	12.880	1.00	0.00
ATOM	10463	HW2	SOL	3113	23.610	45.590	14.100	1.00	0.00
ATOM	10464	OW	SOL	3114	50.740	16.930	5.710	1.00	0.00
ATOM	10465	HW1	SOL	3114	50.510	17.560	6.390	1.00	0.00
ATOM	10466	HW2	SOL	3114	51.550	17.270	5.330	1.00	0.00
ATOM	10467	OW	SOL	3115	47.590	5.320	32.320	1.00	0.00
ATOM	10468	HW1	SOL	3115	46.950	5.640	31.680	1.00	0.00
ATOM	10469	HW2	SOL	3115	48.270	4.910	31.800	1.00	0.00
ATOM	10470	OW	SOL	3116	4.320	0.050	26.200	1.00	0.00
ATOM	10471	HW1	SOL	3116	5.130	0.510	26.430	1.00	0.00

ATOM	10472	HW2	SOL	3116	4.200	0.220	25.270	1.00	0.00
ATOM	10473	OW	SOL	3117	25.170	19.830	1.930	1.00	0.00
ATOM	10474	HW1	SOL	3117	24.300	19.860	2.340	1.00	0.00
ATOM	10475	HW2	SOL	3117	25.160	19.020	1.420	1.00	0.00
ATOM	10476	OW	SOL	3118	5.770	53.780	17.250	1.00	0.00
ATOM	10477	HW1	SOL	3118	5.390	54.630	17.450	1.00	0.00
ATOM	10478	HW2	SOL	3118	5.800	53.750	16.300	1.00	0.00
ATOM	10479	OW	SOL	3119	9.550	16.660	43.300	1.00	0.00
ATOM	10480	HW1	SOL	3119	10.070	15.960	42.910	1.00	0.00
ATOM	10481	HW2	SOL	3119	9.950	17.470	43.000	1.00	0.00
ATOM	10482	OW	SOL	3120	44.340	48.400	25.340	1.00	0.00
ATOM	10483	HW1	SOL	3120	44.490	48.340	26.280	1.00	0.00
ATOM	10484	HW2	SOL	3120	43.450	48.090	25.210	1.00	0.00
ATOM	10485	OW	SOL	3121	39.890	37.940	24.860	1.00	0.00
ATOM	10486	HW1	SOL	3121	40.520	37.290	24.530	1.00	0.00
ATOM	10487	HW2	SOL	3121	39.710	37.670	25.760	1.00	0.00
ATOM	10488	OW	SOL	3122	18.530	34.720	16.750	1.00	0.00
ATOM	10489	HW1	SOL	3122	19.430	34.400	16.740	1.00	0.00
ATOM	10490	HW2	SOL	3122	18.580	35.580	17.150	1.00	0.00
ATOM	10491	OW	SOL	3123	20.770	15.940	26.350	1.00	0.00
ATOM	10492	HW1	SOL	3123	20.120	15.650	26.990	1.00	0.00
ATOM	10493	HW2	SOL	3123	20.280	16.490	25.740	1.00	0.00
ATOM	10494	OW	SOL	3124	28.980	46.560	42.650	1.00	0.00
ATOM	10495	HW1	SOL	3124	28.800	46.710	43.580	1.00	0.00
ATOM	10496	HW2	SOL	3124	28.760	45.640	42.510	1.00	0.00
ATOM	10497	OW	SOL	3125	15.770	35.240	11.610	1.00	0.00
ATOM	10498	HW1	SOL	3125	15.400	35.190	12.490	1.00	0.00
ATOM	10499	HW2	SOL	3125	16.360	35.990	11.630	1.00	0.00
ATOM	10500	OW	SOL	3126	11.050	0.290	2.330	1.00	0.00
ATOM	10501	HW1	SOL	3126	11.710	0.980	2.280	1.00	0.00
ATOM	10502	HW2	SOL	3126	10.920	0.140	3.260	1.00	0.00
ATOM	10503	OW	SOL	3127	35.210	28.700	18.270	1.00	0.00
ATOM	10504	HW1	SOL	3127	36.140	28.490	18.230	1.00	0.00
ATOM	10505	HW2	SOL	3127	34.900	28.580	17.370	1.00	0.00
ATOM	10506	OW	SOL	3128	6.350	53.260	14.800	1.00	0.00
ATOM	10507	HW1	SOL	3128	7.270	53.100	14.590	1.00	0.00
ATOM	10508	HW2	SOL	3128	5.880	52.560	14.360	1.00	0.00
ATOM	10509	OW	SOL	3129	19.060	23.770	26.570	1.00	0.00
ATOM	10510	HW1	SOL	3129	18.830	24.320	25.830	1.00	0.00
ATOM	10511	HW2	SOL	3129	19.770	24.240	27.010	1.00	0.00
ATOM	10512	OW	SOL	3130	21.390	44.790	24.280	1.00	0.00
ATOM	10513	HW1	SOL	3130	21.790	44.970	23.430	1.00	0.00
ATOM	10514	HW2	SOL	3130	21.650	45.540	24.820	1.00	0.00
ATOM	10515	OW	SOL	3131	43.780	44.940	48.760	1.00	0.00

ATOM	10516	HW1	SOL	3131	42.890	44.850	48.410	1.00	0.00
ATOM	10517	HW2	SOL	3131	44.150	44.060	48.710	1.00	0.00
ATOM	10518	OW	SOL	3132	51.440	37.730	54.040	1.00	0.00
ATOM	10519	HW1	SOL	3132	51.570	38.140	53.190	1.00	0.00
ATOM	10520	HW2	SOL	3132	50.530	37.430	54.030	1.00	0.00
ATOM	10521	OW	SOL	3133	54.650	13.210	10.020	1.00	0.00
ATOM	10522	HW1	SOL	3133	54.760	13.950	9.430	1.00	0.00
ATOM	10523	HW2	SOL	3133	54.690	13.600	10.900	1.00	0.00
ATOM	10524	OW	SOL	3134	33.240	4.350	53.680	1.00	0.00
ATOM	10525	HW1	SOL	3134	32.810	3.600	53.260	1.00	0.00
ATOM	10526	HW2	SOL	3134	34.060	4.460	53.190	1.00	0.00
ATOM	10527	OW	SOL	3135	17.470	12.810	19.880	1.00	0.00
ATOM	10528	HW1	SOL	3135	17.550	11.900	19.590	1.00	0.00
ATOM	10529	HW2	SOL	3135	18.360	13.070	20.120	1.00	0.00
ATOM	10530	OW	SOL	3136	11.300	41.000	45.660	1.00	0.00
ATOM	10531	HW1	SOL	3136	11.580	41.890	45.870	1.00	0.00
ATOM	10532	HW2	SOL	3136	12.060	40.610	45.220	1.00	0.00
ATOM	10533	OW	SOL	3137	26.550	48.340	18.360	1.00	0.00
ATOM	10534	HW1	SOL	3137	26.020	48.080	17.610	1.00	0.00
ATOM	10535	HW2	SOL	3137	27.420	47.970	18.180	1.00	0.00
ATOM	10536	OW	SOL	3138	12.880	41.200	41.360	1.00	0.00
ATOM	10537	HW1	SOL	3138	13.640	41.710	41.650	1.00	0.00
ATOM	10538	HW2	SOL	3138	12.960	40.370	41.830	1.00	0.00
ATOM	10539	OW	SOL	3139	49.870	28.150	24.380	1.00	0.00
ATOM	10540	HW1	SOL	3139	49.430	28.990	24.260	1.00	0.00
ATOM	10541	HW2	SOL	3139	49.730	27.930	25.300	1.00	0.00
ATOM	10542	OW	SOL	3140	50.420	3.230	51.610	1.00	0.00
ATOM	10543	HW1	SOL	3140	51.040	3.050	50.900	1.00	0.00
ATOM	10544	HW2	SOL	3140	49.620	2.780	51.350	1.00	0.00
ATOM	10545	OW	SOL	3141	53.200	39.040	35.910	1.00	0.00
ATOM	10546	HW1	SOL	3141	53.050	38.840	36.830	1.00	0.00
ATOM	10547	HW2	SOL	3141	52.380	39.460	35.620	1.00	0.00
ATOM	10548	OW	SOL	3142	52.220	43.290	8.230	1.00	0.00
ATOM	10549	HW1	SOL	3142	52.040	42.850	9.060	1.00	0.00
ATOM	10550	HW2	SOL	3142	51.590	42.930	7.620	1.00	0.00
ATOM	10551	OW	SOL	3143	37.660	0.980	6.930	1.00	0.00
ATOM	10552	HW1	SOL	3143	37.150	1.780	6.990	1.00	0.00
ATOM	10553	HW2	SOL	3143	38.560	1.250	7.120	1.00	0.00
ATOM	10554	OW	SOL	3144	26.120	5.190	24.210	1.00	0.00
ATOM	10555	HW1	SOL	3144	25.290	5.070	23.750	1.00	0.00
ATOM	10556	HW2	SOL	3144	26.770	5.310	23.520	1.00	0.00
ATOM	10557	OW	SOL	3145	41.270	15.250	1.040	1.00	0.00
ATOM	10558	HW1	SOL	3145	40.570	14.720	1.430	1.00	0.00
ATOM	10559	HW2	SOL	3145	41.940	14.620	0.790	1.00	0.00

ATOM	10560	OW	SOL	3146	43.680	50.500	42.050	1.00	0.00
ATOM	10561	HW1	SOL	3146	43.260	49.960	41.390	1.00	0.00
ATOM	10562	HW2	SOL	3146	43.920	51.300	41.590	1.00	0.00
ATOM	10563	OW	SOL	3147	55.260	20.070	14.900	1.00	0.00
ATOM	10564	HW1	SOL	3147	54.930	19.270	15.290	1.00	0.00
ATOM	10565	HW2	SOL	3147	54.540	20.400	14.360	1.00	0.00
ATOM	10566	OW	SOL	3148	3.640	16.110	21.250	1.00	0.00
ATOM	10567	HW1	SOL	3148	3.650	16.340	22.180	1.00	0.00
ATOM	10568	HW2	SOL	3148	3.450	16.940	20.810	1.00	0.00
ATOM	10569	OW	SOL	3149	23.020	50.730	20.980	1.00	0.00
ATOM	10570	HW1	SOL	3149	23.850	50.480	21.400	1.00	0.00
ATOM	10571	HW2	SOL	3149	22.830	50.020	20.380	1.00	0.00
ATOM	10572	OW	SOL	3150	53.270	17.190	51.820	1.00	0.00
ATOM	10573	HW1	SOL	3150	53.780	16.390	51.710	1.00	0.00
ATOM	10574	HW2	SOL	3150	52.440	16.900	52.200	1.00	0.00
ATOM	10575	OW	SOL	3151	53.630	27.090	34.370	1.00	0.00
ATOM	10576	HW1	SOL	3151	52.760	27.470	34.200	1.00	0.00
ATOM	10577	HW2	SOL	3151	53.660	26.310	33.820	1.00	0.00
ATOM	10578	OW	SOL	3152	33.140	22.410	42.720	1.00	0.00
ATOM	10579	HW1	SOL	3152	33.870	22.630	43.300	1.00	0.00
ATOM	10580	HW2	SOL	3152	33.450	22.660	41.850	1.00	0.00
ATOM	10581	OW	SOL	3153	22.470	18.230	46.540	1.00	0.00
ATOM	10582	HW1	SOL	3153	21.670	17.710	46.660	1.00	0.00
ATOM	10583	HW2	SOL	3153	22.340	18.990	47.100	1.00	0.00
ATOM	10584	OW	SOL	3154	42.110	52.510	43.550	1.00	0.00
ATOM	10585	HW1	SOL	3154	42.350	53.430	43.560	1.00	0.00
ATOM	10586	HW2	SOL	3154	42.830	52.070	43.100	1.00	0.00
ATOM	10587	OW	SOL	3155	29.620	47.410	10.480	1.00	0.00
ATOM	10588	HW1	SOL	3155	29.550	46.980	9.620	1.00	0.00
ATOM	10589	HW2	SOL	3155	30.510	47.750	10.500	1.00	0.00
ATOM	10590	OW	SOL	3156	31.620	28.670	48.460	1.00	0.00
ATOM	10591	HW1	SOL	3156	32.440	28.760	47.970	1.00	0.00
ATOM	10592	HW2	SOL	3156	31.630	27.770	48.780	1.00	0.00
ATOM	10593	OW	SOL	3157	42.980	21.750	55.420	1.00	0.00
ATOM	10594	HW1	SOL	3157	43.580	21.030	55.220	1.00	0.00
ATOM	10595	HW2	SOL	3157	42.290	21.690	54.760	1.00	0.00
ATOM	10596	OW	SOL	3158	10.410	31.680	1.170	1.00	0.00
ATOM	10597	HW1	SOL	3158	9.540	31.400	0.880	1.00	0.00
ATOM	10598	HW2	SOL	3158	10.510	32.560	0.800	1.00	0.00
ATOM	10599	OW	SOL	3159	13.790	32.760	16.250	1.00	0.00
ATOM	10600	HW1	SOL	3159	13.410	33.450	16.800	1.00	0.00
ATOM	10601	HW2	SOL	3159	13.590	31.950	16.710	1.00	0.00
ATOM	10602	OW	SOL	3160	3.980	35.270	25.120	1.00	0.00
ATOM	10603	HW1	SOL	3160	4.000	34.660	24.390	1.00	0.00

ATOM	10604	HW2	SOL	3160	3.220	35.010	25.630	1.00	0.00
ATOM	10605	OW	SOL	3161	26.680	11.640	26.010	1.00	0.00
ATOM	10606	HW1	SOL	3161	26.000	10.980	25.930	1.00	0.00
ATOM	10607	HW2	SOL	3161	27.080	11.480	26.870	1.00	0.00
ATOM	10608	OW	SOL	3162	21.810	46.060	45.720	1.00	0.00
ATOM	10609	HW1	SOL	3162	22.310	46.660	45.170	1.00	0.00
ATOM	10610	HW2	SOL	3162	21.960	45.200	45.320	1.00	0.00
ATOM	10611	OW	SOL	3163	15.240	6.320	7.830	1.00	0.00
ATOM	10612	HW1	SOL	3163	15.930	5.870	8.330	1.00	0.00
ATOM	10613	HW2	SOL	3163	15.640	7.150	7.580	1.00	0.00
ATOM	10614	OW	SOL	3164	36.620	39.070	44.460	1.00	0.00
ATOM	10615	HW1	SOL	3164	37.360	39.560	44.810	1.00	0.00
ATOM	10616	HW2	SOL	3164	35.950	39.130	45.150	1.00	0.00
ATOM	10617	OW	SOL	3165	10.290	39.410	42.530	1.00	0.00
ATOM	10618	HW1	SOL	3165	10.570	40.150	43.060	1.00	0.00
ATOM	10619	HW2	SOL	3165	11.080	38.870	42.440	1.00	0.00
ATOM	10620	OW	SOL	3166	35.530	16.660	51.630	1.00	0.00
ATOM	10621	HW1	SOL	3166	34.900	16.040	51.260	1.00	0.00
ATOM	10622	HW2	SOL	3166	35.040	17.480	51.700	1.00	0.00
ATOM	10623	OW	SOL	3167	16.130	53.820	39.630	1.00	0.00
ATOM	10624	HW1	SOL	3167	15.910	54.610	39.130	1.00	0.00
ATOM	10625	HW2	SOL	3167	17.010	53.990	39.970	1.00	0.00
ATOM	10626	OW	SOL	3168	9.200	28.810	53.510	1.00	0.00
ATOM	10627	HW1	SOL	3168	9.000	28.890	52.580	1.00	0.00
ATOM	10628	HW2	SOL	3168	9.050	27.880	53.710	1.00	0.00
ATOM	10629	OW	SOL	3169	24.850	38.260	14.450	1.00	0.00
ATOM	10630	HW1	SOL	3169	24.180	37.580	14.400	1.00	0.00
ATOM	10631	HW2	SOL	3169	25.270	38.250	13.590	1.00	0.00
ATOM	10632	OW	SOL	3170	48.640	18.250	4.020	1.00	0.00
ATOM	10633	HW1	SOL	3170	49.220	18.460	4.750	1.00	0.00
ATOM	10634	HW2	SOL	3170	48.460	17.320	4.110	1.00	0.00
ATOM	10635	OW	SOL	3171	20.230	9.470	0.670	1.00	0.00
ATOM	10636	HW1	SOL	3171	20.540	9.690	1.550	1.00	0.00
ATOM	10637	HW2	SOL	3171	21.010	9.570	0.120	1.00	0.00
ATOM	10638	OW	SOL	3172	0.560	50.250	49.960	1.00	0.00
ATOM	10639	HW1	SOL	3172	0.280	50.710	50.750	1.00	0.00
ATOM	10640	HW2	SOL	3172	1.230	50.810	49.580	1.00	0.00
ATOM	10641	OW	SOL	3173	46.370	36.450	20.230	1.00	0.00
ATOM	10642	HW1	SOL	3173	46.010	36.050	19.440	1.00	0.00
ATOM	10643	HW2	SOL	3173	46.020	37.340	20.240	1.00	0.00
ATOM	10644	OW	SOL	3174	3.000	24.750	36.880	1.00	0.00
ATOM	10645	HW1	SOL	3174	3.440	24.050	36.390	1.00	0.00
ATOM	10646	HW2	SOL	3174	3.360	24.690	37.760	1.00	0.00
ATOM	10647	OW	SOL	3175	52.190	54.600	29.140	1.00	0.00

ATOM	10648	HW1	SOL	3175	53.070	54.640	28.760	1.00	0.00
ATOM	10649	HW2	SOL	3175	51.730	55.340	28.750	1.00	0.00
ATOM	10650	OW	SOL	3176	8.380	33.900	5.640	1.00	0.00
ATOM	10651	HW1	SOL	3176	7.650	34.420	5.980	1.00	0.00
ATOM	10652	HW2	SOL	3176	8.690	33.400	6.390	1.00	0.00
ATOM	10653	OW	SOL	3177	51.980	27.370	13.530	1.00	0.00
ATOM	10654	HW1	SOL	3177	52.290	26.740	14.180	1.00	0.00
ATOM	10655	HW2	SOL	3177	52.110	28.220	13.930	1.00	0.00
ATOM	10656	OW	SOL	3178	15.260	33.300	49.580	1.00	0.00
ATOM	10657	HW1	SOL	3178	14.350	33.590	49.560	1.00	0.00
ATOM	10658	HW2	SOL	3178	15.770	34.100	49.450	1.00	0.00
ATOM	10659	OW	SOL	3179	5.560	24.700	10.780	1.00	0.00
ATOM	10660	HW1	SOL	3179	5.890	24.600	9.890	1.00	0.00
ATOM	10661	HW2	SOL	3179	4.610	24.700	10.690	1.00	0.00
ATOM	10662	OW	SOL	3180	15.310	6.670	47.500	1.00	0.00
ATOM	10663	HW1	SOL	3180	16.020	6.780	48.130	1.00	0.00
ATOM	10664	HW2	SOL	3180	14.980	7.560	47.350	1.00	0.00
ATOM	10665	OW	SOL	3181	12.580	41.170	33.630	1.00	0.00
ATOM	10666	HW1	SOL	3181	13.240	41.320	32.950	1.00	0.00
ATOM	10667	HW2	SOL	3181	12.840	40.340	34.040	1.00	0.00
ATOM	10668	OW	SOL	3182	33.730	7.490	27.550	1.00	0.00
ATOM	10669	HW1	SOL	3182	34.210	6.850	27.020	1.00	0.00
ATOM	10670	HW2	SOL	3182	32.840	7.480	27.180	1.00	0.00
ATOM	10671	OW	SOL	3183	44.520	22.620	31.670	1.00	0.00
ATOM	10672	HW1	SOL	3183	44.080	21.790	31.500	1.00	0.00
ATOM	10673	HW2	SOL	3183	45.220	22.410	32.290	1.00	0.00
ATOM	10674	OW	SOL	3184	28.250	10.260	45.270	1.00	0.00
ATOM	10675	HW1	SOL	3184	28.730	10.280	44.440	1.00	0.00
ATOM	10676	HW2	SOL	3184	28.230	11.170	45.550	1.00	0.00
ATOM	10677	OW	SOL	3185	36.060	53.200	49.620	1.00	0.00
ATOM	10678	HW1	SOL	3185	36.260	52.680	48.840	1.00	0.00
ATOM	10679	HW2	SOL	3185	35.300	53.720	49.370	1.00	0.00
ATOM	10680	OW	SOL	3186	14.860	0.720	11.890	1.00	0.00
ATOM	10681	HW1	SOL	3186	15.070	1.490	12.420	1.00	0.00
ATOM	10682	HW2	SOL	3186	14.560	1.070	11.050	1.00	0.00
ATOM	10683	OW	SOL	3187	36.510	50.690	23.280	1.00	0.00
ATOM	10684	HW1	SOL	3187	36.320	51.520	22.850	1.00	0.00
ATOM	10685	HW2	SOL	3187	36.150	50.790	24.160	1.00	0.00
ATOM	10686	OW	SOL	3188	53.640	31.570	16.320	1.00	0.00
ATOM	10687	HW1	SOL	3188	53.040	31.170	16.950	1.00	0.00
ATOM	10688	HW2	SOL	3188	54.420	31.780	16.830	1.00	0.00
ATOM	10689	OW	SOL	3189	31.780	49.570	9.940	1.00	0.00
ATOM	10690	HW1	SOL	3189	32.120	48.720	9.660	1.00	0.00
ATOM	10691	HW2	SOL	3189	31.740	50.090	9.140	1.00	0.00

ATOM	10692	OW	SOL	3190	46.870	32.190	51.860	1.00	0.00
ATOM	10693	HW1	SOL	3190	45.960	32.460	52.020	1.00	0.00
ATOM	10694	HW2	SOL	3190	46.850	31.240	51.980	1.00	0.00
ATOM	10695	OW	SOL	3191	14.520	11.110	49.080	1.00	0.00
ATOM	10696	HW1	SOL	3191	14.520	10.320	49.610	1.00	0.00
ATOM	10697	HW2	SOL	3191	14.060	11.760	49.620	1.00	0.00
ATOM	10698	OW	SOL	3192	48.260	27.730	0.480	1.00	0.00
ATOM	10699	HW1	SOL	3192	47.580	28.350	0.740	1.00	0.00
ATOM	10700	HW2	SOL	3192	48.910	28.260	0.020	1.00	0.00
ATOM	10701	OW	SOL	3193	39.360	29.750	31.140	1.00	0.00
ATOM	10702	HW1	SOL	3193	40.140	30.280	31.240	1.00	0.00
ATOM	10703	HW2	SOL	3193	39.430	29.080	31.820	1.00	0.00
ATOM	10704	OW	SOL	3194	27.910	19.350	21.090	1.00	0.00
ATOM	10705	HW1	SOL	3194	28.110	18.460	20.820	1.00	0.00
ATOM	10706	HW2	SOL	3194	28.250	19.900	20.390	1.00	0.00
ATOM	10707	OW	SOL	3195	12.590	43.900	47.670	1.00	0.00
ATOM	10708	HW1	SOL	3195	13.470	44.280	47.770	1.00	0.00
ATOM	10709	HW2	SOL	3195	12.340	44.110	46.770	1.00	0.00
ATOM	10710	OW	SOL	3196	6.440	36.710	50.280	1.00	0.00
ATOM	10711	HW1	SOL	3196	5.880	37.170	50.890	1.00	0.00
ATOM	10712	HW2	SOL	3196	6.560	37.320	49.550	1.00	0.00
ATOM	10713	OW	SOL	3197	34.800	34.210	43.690	1.00	0.00
ATOM	10714	HW1	SOL	3197	35.110	33.840	42.860	1.00	0.00
ATOM	10715	HW2	SOL	3197	35.380	33.820	44.350	1.00	0.00
ATOM	10716	OW	SOL	3198	34.730	44.050	34.720	1.00	0.00
ATOM	10717	HW1	SOL	3198	35.460	43.570	35.130	1.00	0.00
ATOM	10718	HW2	SOL	3198	34.060	44.100	35.400	1.00	0.00
ATOM	10719	OW	SOL	3199	55.500	55.110	51.170	1.00	0.00
ATOM	10720	HW1	SOL	3199	55.340	54.220	51.480	1.00	0.00
ATOM	10721	HW2	SOL	3199	54.640	55.520	51.170	1.00	0.00
ATOM	10722	OW	SOL	3200	42.450	21.160	7.000	1.00	0.00
ATOM	10723	HW1	SOL	3200	41.660	20.620	6.930	1.00	0.00
ATOM	10724	HW2	SOL	3200	42.210	21.870	7.590	1.00	0.00
ATOM	10725	OW	SOL	3201	47.490	43.390	11.000	1.00	0.00
ATOM	10726	HW1	SOL	3201	46.940	43.620	11.740	1.00	0.00
ATOM	10727	HW2	SOL	3201	48.270	43.940	11.100	1.00	0.00
ATOM	10728	OW	SOL	3202	45.940	12.320	18.920	1.00	0.00
ATOM	10729	HW1	SOL	3202	46.580	11.710	19.290	1.00	0.00
ATOM	10730	HW2	SOL	3202	45.100	12.000	19.250	1.00	0.00
ATOM	10731	OW	SOL	3203	48.790	33.560	50.760	1.00	0.00
ATOM	10732	HW1	SOL	3203	48.920	33.280	49.850	1.00	0.00
ATOM	10733	HW2	SOL	3203	48.000	33.100	51.040	1.00	0.00
ATOM	10734	OW	SOL	3204	14.060	48.550	2.510	1.00	0.00
ATOM	10735	HW1	SOL	3204	14.680	49.080	3.010	1.00	0.00

ATOM	10736	HW2	SOL	3204	14.470	48.440	1.650	1.00	0.00
ATOM	10737	OW	SOL	3205	16.640	47.090	4.460	1.00	0.00
ATOM	10738	HW1	SOL	3205	16.590	48.020	4.250	1.00	0.00
ATOM	10739	HW2	SOL	3205	16.420	46.650	3.630	1.00	0.00
ATOM	10740	OW	SOL	3206	23.710	21.820	45.330	1.00	0.00
ATOM	10741	HW1	SOL	3206	23.530	22.540	45.930	1.00	0.00
ATOM	10742	HW2	SOL	3206	23.610	22.200	44.460	1.00	0.00
ATOM	10743	OW	SOL	3207	31.030	44.180	24.020	1.00	0.00
ATOM	10744	HW1	SOL	3207	31.080	43.710	23.190	1.00	0.00
ATOM	10745	HW2	SOL	3207	31.230	43.520	24.680	1.00	0.00
ATOM	10746	OW	SOL	3208	25.720	11.130	16.520	1.00	0.00
ATOM	10747	HW1	SOL	3208	26.320	11.870	16.530	1.00	0.00
ATOM	10748	HW2	SOL	3208	25.540	10.960	17.450	1.00	0.00
ATOM	10749	OW	SOL	3209	38.420	27.320	54.940	1.00	0.00
ATOM	10750	HW1	SOL	3209	38.660	28.060	55.500	1.00	0.00
ATOM	10751	HW2	SOL	3209	38.900	26.580	55.310	1.00	0.00
ATOM	10752	OW	SOL	3210	2.100	12.780	48.440	1.00	0.00
ATOM	10753	HW1	SOL	3210	1.220	12.510	48.710	1.00	0.00
ATOM	10754	HW2	SOL	3210	2.600	12.810	49.260	1.00	0.00
ATOM	10755	OW	SOL	3211	52.370	45.550	49.180	1.00	0.00
ATOM	10756	HW1	SOL	3211	53.140	46.110	49.210	1.00	0.00
ATOM	10757	HW2	SOL	3211	51.630	46.160	49.090	1.00	0.00
ATOM	10758	OW	SOL	3212	42.400	14.840	42.900	1.00	0.00
ATOM	10759	HW1	SOL	3212	42.460	15.330	42.080	1.00	0.00
ATOM	10760	HW2	SOL	3212	43.300	14.570	43.080	1.00	0.00
ATOM	10761	OW	SOL	3213	6.370	55.500	13.110	1.00	0.00
ATOM	10762	HW1	SOL	3213	5.910	54.760	13.520	1.00	0.00
ATOM	10763	HW2	SOL	3213	5.670	56.020	12.700	1.00	0.00
ATOM	10764	OW	SOL	3214	30.090	16.390	30.590	1.00	0.00
ATOM	10765	HW1	SOL	3214	30.810	15.790	30.450	1.00	0.00
ATOM	10766	HW2	SOL	3214	30.110	16.980	29.840	1.00	0.00
ATOM	10767	OW	SOL	3215	35.730	33.870	11.210	1.00	0.00
ATOM	10768	HW1	SOL	3215	35.980	34.580	11.790	1.00	0.00
ATOM	10769	HW2	SOL	3215	34.780	33.930	11.150	1.00	0.00
ATOM	10770	OW	SOL	3216	11.970	21.220	40.750	1.00	0.00
ATOM	10771	HW1	SOL	3216	11.160	21.740	40.740	1.00	0.00
ATOM	10772	HW2	SOL	3216	11.670	20.320	40.910	1.00	0.00
ATOM	10773	OW	SOL	3217	9.480	53.450	38.230	1.00	0.00
ATOM	10774	HW1	SOL	3217	9.600	52.780	38.900	1.00	0.00
ATOM	10775	HW2	SOL	3217	8.740	53.150	37.710	1.00	0.00
ATOM	10776	OW	SOL	3218	30.550	53.540	43.730	1.00	0.00
ATOM	10777	HW1	SOL	3218	30.870	53.120	44.540	1.00	0.00
ATOM	10778	HW2	SOL	3218	31.200	53.300	43.070	1.00	0.00
ATOM	10779	OW	SOL	3219	44.830	11.910	46.710	1.00	0.00

ATOM	10780	HW1	SOL	3219	44.990	11.130	46.180	1.00	0.00
ATOM	10781	HW2	SOL	3219	44.080	11.680	47.250	1.00	0.00
ATOM	10782	OW	SOL	3220	19.320	44.960	52.450	1.00	0.00
ATOM	10783	HW1	SOL	3220	19.730	45.360	51.680	1.00	0.00
ATOM	10784	HW2	SOL	3220	18.500	45.440	52.570	1.00	0.00
ATOM	10785	OW	SOL	3221	19.910	29.600	27.210	1.00	0.00
ATOM	10786	HW1	SOL	3221	19.510	28.740	27.330	1.00	0.00
ATOM	10787	HW2	SOL	3221	20.340	29.550	26.360	1.00	0.00
ATOM	10788	OW	SOL	3222	39.060	3.940	20.050	1.00	0.00
ATOM	10789	HW1	SOL	3222	39.300	3.900	19.130	1.00	0.00
ATOM	10790	HW2	SOL	3222	39.830	4.300	20.490	1.00	0.00
ATOM	10791	OW	SOL	3223	11.190	48.200	32.820	1.00	0.00
ATOM	10792	HW1	SOL	3223	10.560	48.780	32.390	1.00	0.00
ATOM	10793	HW2	SOL	3223	10.810	48.040	33.690	1.00	0.00
ATOM	10794	OW	SOL	3224	50.660	35.290	4.790	1.00	0.00
ATOM	10795	HW1	SOL	3224	50.100	36.060	4.810	1.00	0.00
ATOM	10796	HW2	SOL	3224	51.200	35.360	5.570	1.00	0.00
ATOM	10797	OW	SOL	3225	25.960	31.810	27.910	1.00	0.00
ATOM	10798	HW1	SOL	3225	26.760	32.170	27.540	1.00	0.00
ATOM	10799	HW2	SOL	3225	26.240	31.010	28.360	1.00	0.00
ATOM	10800	OW	SOL	3226	16.320	49.600	25.720	1.00	0.00
ATOM	10801	HW1	SOL	3226	15.370	49.640	25.870	1.00	0.00
ATOM	10802	HW2	SOL	3226	16.430	49.880	24.820	1.00	0.00
ATOM	10803	OW	SOL	3227	2.780	36.070	29.400	1.00	0.00
ATOM	10804	HW1	SOL	3227	2.640	36.800	30.000	1.00	0.00
ATOM	10805	HW2	SOL	3227	2.840	36.470	28.540	1.00	0.00
ATOM	10806	OW	SOL	3228	36.650	8.060	0.810	1.00	0.00
ATOM	10807	HW1	SOL	3228	36.620	8.980	1.070	1.00	0.00
ATOM	10808	HW2	SOL	3228	36.290	7.580	1.560	1.00	0.00
ATOM	10809	OW	SOL	3229	39.340	34.860	9.780	1.00	0.00
ATOM	10810	HW1	SOL	3229	38.440	35.050	9.530	1.00	0.00
ATOM	10811	HW2	SOL	3229	39.270	34.250	10.510	1.00	0.00
ATOM	10812	OW	SOL	3230	38.100	45.840	48.840	1.00	0.00
ATOM	10813	HW1	SOL	3230	38.670	46.340	49.430	1.00	0.00
ATOM	10814	HW2	SOL	3230	38.600	45.780	48.030	1.00	0.00
ATOM	10815	OW	SOL	3231	23.720	6.370	51.930	1.00	0.00
ATOM	10816	HW1	SOL	3231	23.650	5.440	52.150	1.00	0.00
ATOM	10817	HW2	SOL	3231	23.180	6.470	51.150	1.00	0.00
ATOM	10818	OW	SOL	3232	45.260	47.010	51.750	1.00	0.00
ATOM	10819	HW1	SOL	3232	45.610	47.420	50.950	1.00	0.00
ATOM	10820	HW2	SOL	3232	45.090	46.100	51.490	1.00	0.00
ATOM	10821	OW	SOL	3233	31.990	22.710	46.480	1.00	0.00
ATOM	10822	HW1	SOL	3233	32.800	22.370	46.090	1.00	0.00
ATOM	10823	HW2	SOL	3233	32.280	23.310	47.170	1.00	0.00

ATOM	10824	OW	SOL	3234	28.360	9.500	55.570	1.00	0.00
ATOM	10825	HW1	SOL	3234	27.810	9.200	56.290	1.00	0.00
ATOM	10826	HW2	SOL	3234	28.740	8.710	55.210	1.00	0.00
ATOM	10827	OW	SOL	3235	11.280	28.080	39.600	1.00	0.00
ATOM	10828	HW1	SOL	3235	11.400	28.850	40.150	1.00	0.00
ATOM	10829	HW2	SOL	3235	11.350	28.410	38.700	1.00	0.00
ATOM	10830	OW	SOL	3236	28.800	3.190	51.430	1.00	0.00
ATOM	10831	HW1	SOL	3236	28.190	3.700	51.960	1.00	0.00
ATOM	10832	HW2	SOL	3236	28.260	2.550	50.980	1.00	0.00
ATOM	10833	OW	SOL	3237	11.370	20.870	30.960	1.00	0.00
ATOM	10834	HW1	SOL	3237	12.310	21.030	30.850	1.00	0.00
ATOM	10835	HW2	SOL	3237	11.020	21.690	31.320	1.00	0.00
ATOM	10836	OW	SOL	3238	11.630	43.490	1.490	1.00	0.00
ATOM	10837	HW1	SOL	3238	12.420	43.490	2.040	1.00	0.00
ATOM	10838	HW2	SOL	3238	11.580	42.590	1.150	1.00	0.00
ATOM	10839	OW	SOL	3239	6.230	47.960	47.310	1.00	0.00
ATOM	10840	HW1	SOL	3239	6.480	47.760	46.410	1.00	0.00
ATOM	10841	HW2	SOL	3239	6.640	48.810	47.490	1.00	0.00
ATOM	10842	OW	SOL	3240	17.870	10.410	36.550	1.00	0.00
ATOM	10843	HW1	SOL	3240	17.150	9.990	37.010	1.00	0.00
ATOM	10844	HW2	SOL	3240	17.840	11.320	36.840	1.00	0.00
ATOM	10845	OW	SOL	3241	45.780	8.710	20.300	1.00	0.00
ATOM	10846	HW1	SOL	3241	45.320	9.120	19.570	1.00	0.00
ATOM	10847	HW2	SOL	3241	46.690	8.990	20.200	1.00	0.00
ATOM	10848	OW	SOL	3242	18.180	49.260	10.640	1.00	0.00
ATOM	10849	HW1	SOL	3242	18.190	48.620	9.930	1.00	0.00
ATOM	10850	HW2	SOL	3242	19.090	49.300	10.940	1.00	0.00
ATOM	10851	OW	SOL	3243	8.770	46.120	42.930	1.00	0.00
ATOM	10852	HW1	SOL	3243	8.160	46.510	42.300	1.00	0.00
ATOM	10853	HW2	SOL	3243	8.420	45.240	43.090	1.00	0.00
ATOM	10854	OW	SOL	3244	38.370	14.560	40.460	1.00	0.00
ATOM	10855	HW1	SOL	3244	37.930	14.520	39.610	1.00	0.00
ATOM	10856	HW2	SOL	3244	38.050	15.370	40.860	1.00	0.00
ATOM	10857	OW	SOL	3245	34.780	8.230	19.080	1.00	0.00
ATOM	10858	HW1	SOL	3245	34.750	8.940	18.430	1.00	0.00
ATOM	10859	HW2	SOL	3245	34.480	8.640	19.890	1.00	0.00
ATOM	10860	OW	SOL	3246	39.490	51.580	26.650	1.00	0.00
ATOM	10861	HW1	SOL	3246	39.900	52.300	27.130	1.00	0.00
ATOM	10862	HW2	SOL	3246	38.570	51.600	26.920	1.00	0.00
ATOM	10863	OW	SOL	3247	27.950	21.580	34.420	1.00	0.00
ATOM	10864	HW1	SOL	3247	27.880	20.750	33.960	1.00	0.00
ATOM	10865	HW2	SOL	3247	28.780	21.960	34.110	1.00	0.00
ATOM	10866	OW	SOL	3248	20.940	45.740	34.130	1.00	0.00
ATOM	10867	HW1	SOL	3248	20.000	45.550	34.100	1.00	0.00

ATOM	10868	HW2	SOL	3248	21.110	45.950	35.040	1.00	0.00
ATOM	10869	OW	SOL	3249	0.720	16.800	42.410	1.00	0.00
ATOM	10870	HW1	SOL	3249	0.530	17.520	41.800	1.00	0.00
ATOM	10871	HW2	SOL	3249	1.430	17.130	42.950	1.00	0.00
ATOM	10872	OW	SOL	3250	3.080	23.960	17.400	1.00	0.00
ATOM	10873	HW1	SOL	3250	2.480	23.360	17.840	1.00	0.00
ATOM	10874	HW2	SOL	3250	2.930	24.800	17.840	1.00	0.00
ATOM	10875	OW	SOL	3251	43.650	46.690	3.710	1.00	0.00
ATOM	10876	HW1	SOL	3251	43.740	47.390	3.070	1.00	0.00
ATOM	10877	HW2	SOL	3251	42.710	46.560	3.790	1.00	0.00
ATOM	10878	OW	SOL	3252	8.020	15.570	34.280	1.00	0.00
ATOM	10879	HW1	SOL	3252	8.280	16.280	33.690	1.00	0.00
ATOM	10880	HW2	SOL	3252	7.750	14.860	33.690	1.00	0.00
ATOM	10881	OW	SOL	3253	52.220	36.820	2.280	1.00	0.00
ATOM	10882	HW1	SOL	3253	51.860	36.100	2.790	1.00	0.00
ATOM	10883	HW2	SOL	3253	53.010	37.080	2.750	1.00	0.00
ATOM	10884	OW	SOL	3254	51.590	49.840	29.720	1.00	0.00
ATOM	10885	HW1	SOL	3254	51.090	50.650	29.660	1.00	0.00
ATOM	10886	HW2	SOL	3254	52.500	50.120	29.760	1.00	0.00
ATOM	10887	OW	SOL	3255	5.420	42.580	0.370	1.00	0.00
ATOM	10888	HW1	SOL	3255	5.460	42.470	1.320	1.00	0.00
ATOM	10889	HW2	SOL	3255	5.960	43.350	0.190	1.00	0.00
ATOM	10890	OW	SOL	3256	52.370	36.740	46.430	1.00	0.00
ATOM	10891	HW1	SOL	3256	51.910	37.500	46.760	1.00	0.00
ATOM	10892	HW2	SOL	3256	52.320	36.820	45.480	1.00	0.00
ATOM	10893	OW	SOL	3257	46.820	9.770	3.530	1.00	0.00
ATOM	10894	HW1	SOL	3257	46.880	9.450	2.630	1.00	0.00
ATOM	10895	HW2	SOL	3257	46.880	8.990	4.070	1.00	0.00
ATOM	10896	OW	SOL	3258	7.590	49.870	19.990	1.00	0.00
ATOM	10897	HW1	SOL	3258	8.180	50.230	20.660	1.00	0.00
ATOM	10898	HW2	SOL	3258	7.800	50.360	19.200	1.00	0.00
ATOM	10899	OW	SOL	3259	11.420	35.220	44.740	1.00	0.00
ATOM	10900	HW1	SOL	3259	10.510	35.350	45.000	1.00	0.00
ATOM	10901	HW2	SOL	3259	11.780	34.650	45.410	1.00	0.00
ATOM	10902	OW	SOL	3260	5.940	29.230	8.060	1.00	0.00
ATOM	10903	HW1	SOL	3260	6.040	30.000	8.620	1.00	0.00
ATOM	10904	HW2	SOL	3260	5.790	28.510	8.670	1.00	0.00
ATOM	10905	OW	SOL	3261	5.020	0.270	18.130	1.00	0.00
ATOM	10906	HW1	SOL	3261	4.960	1.150	17.770	1.00	0.00
ATOM	10907	HW2	SOL	3261	4.170	0.120	18.540	1.00	0.00
ATOM	10908	OW	SOL	3262	7.380	6.680	38.380	1.00	0.00
ATOM	10909	HW1	SOL	3262	6.940	6.850	39.210	1.00	0.00
ATOM	10910	HW2	SOL	3262	6.760	6.160	37.870	1.00	0.00
ATOM	10911	OW	SOL	3263	21.800	14.520	52.110	1.00	0.00

ATOM	10912	HW1	SOL	3263	22.590	14.670	52.620	1.00	0.00
ATOM	10913	HW2	SOL	3263	21.500	13.650	52.380	1.00	0.00
ATOM	10914	OW	SOL	3264	16.700	19.610	33.570	1.00	0.00
ATOM	10915	HW1	SOL	3264	17.370	19.320	32.950	1.00	0.00
ATOM	10916	HW2	SOL	3264	16.590	20.540	33.390	1.00	0.00
ATOM	10917	OW	SOL	3265	27.020	28.840	2.190	1.00	0.00
ATOM	10918	HW1	SOL	3265	26.630	28.300	2.880	1.00	0.00
ATOM	10919	HW2	SOL	3265	27.370	28.210	1.560	1.00	0.00
ATOM	10920	OW	SOL	3266	6.940	21.530	9.960	1.00	0.00
ATOM	10921	HW1	SOL	3266	7.650	20.880	9.980	1.00	0.00
ATOM	10922	HW2	SOL	3266	6.150	21.030	10.150	1.00	0.00
ATOM	10923	OW	SOL	3267	11.760	15.220	20.760	1.00	0.00
ATOM	10924	HW1	SOL	3267	12.310	15.460	20.010	1.00	0.00
ATOM	10925	HW2	SOL	3267	10.870	15.420	20.470	1.00	0.00
ATOM	10926	OW	SOL	3268	3.130	43.100	54.960	1.00	0.00
ATOM	10927	HW1	SOL	3268	3.920	42.920	55.470	1.00	0.00
ATOM	10928	HW2	SOL	3268	3.010	44.050	55.050	1.00	0.00
ATOM	10929	OW	SOL	3269	28.110	51.200	53.180	1.00	0.00
ATOM	10930	HW1	SOL	3269	28.310	52.130	53.100	1.00	0.00
ATOM	10931	HW2	SOL	3269	27.480	51.150	53.900	1.00	0.00
ATOM	10932	OW	SOL	3270	3.100	55.320	46.210	1.00	0.00
ATOM	10933	HW1	SOL	3270	3.400	54.900	45.410	1.00	0.00
ATOM	10934	HW2	SOL	3270	2.620	54.630	46.670	1.00	0.00
ATOM	10935	OW	SOL	3271	0.220	55.760	53.830	1.00	0.00
ATOM	10936	HW1	SOL	3271	-0.310	55.120	53.370	1.00	0.00
ATOM	10937	HW2	SOL	3271	1.070	55.730	53.390	1.00	0.00
ATOM	10938	OW	SOL	3272	11.520	14.890	51.430	1.00	0.00
ATOM	10939	HW1	SOL	3272	11.380	14.010	51.780	1.00	0.00
ATOM	10940	HW2	SOL	3272	10.930	14.940	50.680	1.00	0.00
ATOM	10941	OW	SOL	3273	23.760	35.510	55.440	1.00	0.00
ATOM	10942	HW1	SOL	3273	23.760	36.360	55.880	1.00	0.00
ATOM	10943	HW2	SOL	3273	24.050	34.890	56.100	1.00	0.00
ATOM	10944	OW	SOL	3274	51.400	8.520	9.420	1.00	0.00
ATOM	10945	HW1	SOL	3274	52.020	9.020	9.950	1.00	0.00
ATOM	10946	HW2	SOL	3274	51.320	9.030	8.610	1.00	0.00
ATOM	10947	OW	SOL	3275	44.110	52.800	40.940	1.00	0.00
ATOM	10948	HW1	SOL	3275	43.550	53.570	40.930	1.00	0.00
ATOM	10949	HW2	SOL	3275	44.240	52.580	40.020	1.00	0.00
ATOM	10950	OW	SOL	3276	44.410	22.530	50.420	1.00	0.00
ATOM	10951	HW1	SOL	3276	44.860	23.370	50.490	1.00	0.00
ATOM	10952	HW2	SOL	3276	44.410	22.190	51.320	1.00	0.00
ATOM	10953	OW	SOL	3277	25.310	12.330	3.010	1.00	0.00
ATOM	10954	HW1	SOL	3277	25.760	11.510	3.210	1.00	0.00
ATOM	10955	HW2	SOL	3277	24.750	12.490	3.770	1.00	0.00

ATOM	10956	OW	SOL	3278	15.860	17.840	4.930	1.00	0.00
ATOM	10957	HW1	SOL	3278	15.180	17.920	5.600	1.00	0.00
ATOM	10958	HW2	SOL	3278	15.390	17.940	4.100	1.00	0.00
ATOM	10959	OW	SOL	3279	12.340	12.920	6.890	1.00	0.00
ATOM	10960	HW1	SOL	3279	11.660	12.380	6.470	1.00	0.00
ATOM	10961	HW2	SOL	3279	12.550	13.590	6.250	1.00	0.00
ATOM	10962	OW	SOL	3280	50.700	50.980	9.840	1.00	0.00
ATOM	10963	HW1	SOL	3280	50.750	50.680	8.930	1.00	0.00
ATOM	10964	HW2	SOL	3280	50.610	51.930	9.780	1.00	0.00
ATOM	10965	OW	SOL	3281	19.870	55.480	53.470	1.00	0.00
ATOM	10966	HW1	SOL	3281	20.380	54.720	53.750	1.00	0.00
ATOM	10967	HW2	SOL	3281	19.000	55.330	53.840	1.00	0.00
ATOM	10968	OW	SOL	3282	35.910	10.650	21.790	1.00	0.00
ATOM	10969	HW1	SOL	3282	35.050	10.220	21.750	1.00	0.00
ATOM	10970	HW2	SOL	3282	36.400	10.160	22.450	1.00	0.00
ATOM	10971	OW	SOL	3283	44.800	38.090	5.670	1.00	0.00
ATOM	10972	HW1	SOL	3283	43.850	37.980	5.790	1.00	0.00
ATOM	10973	HW2	SOL	3283	44.900	39.000	5.370	1.00	0.00
ATOM	10974	OW	SOL	3284	17.580	42.380	53.830	1.00	0.00
ATOM	10975	HW1	SOL	3284	18.090	42.940	54.420	1.00	0.00
ATOM	10976	HW2	SOL	3284	17.300	42.970	53.130	1.00	0.00
ATOM	10977	OW	SOL	3285	30.780	25.150	52.000	1.00	0.00
ATOM	10978	HW1	SOL	3285	31.080	24.700	52.790	1.00	0.00
ATOM	10979	HW2	SOL	3285	31.100	26.050	52.110	1.00	0.00
ATOM	10980	OW	SOL	3286	25.670	26.150	45.840	1.00	0.00
ATOM	10981	HW1	SOL	3286	24.980	25.840	46.430	1.00	0.00
ATOM	10982	HW2	SOL	3286	25.830	27.050	46.110	1.00	0.00
ATOM	10983	OW	SOL	3287	28.640	52.720	40.490	1.00	0.00
ATOM	10984	HW1	SOL	3287	27.920	53.190	40.060	1.00	0.00
ATOM	10985	HW2	SOL	3287	29.370	53.340	40.480	1.00	0.00
ATOM	10986	OW	SOL	3288	18.110	43.450	23.610	1.00	0.00
ATOM	10987	HW1	SOL	3288	18.940	42.980	23.690	1.00	0.00
ATOM	10988	HW2	SOL	3288	18.330	44.360	23.820	1.00	0.00
ATOM	10989	OW	SOL	3289	50.180	27.680	2.990	1.00	0.00
ATOM	10990	HW1	SOL	3289	49.810	27.530	2.120	1.00	0.00
ATOM	10991	HW2	SOL	3289	50.690	28.480	2.910	1.00	0.00
ATOM	10992	OW	SOL	3290	39.530	14.230	20.020	1.00	0.00
ATOM	10993	HW1	SOL	3290	39.990	15.070	19.970	1.00	0.00
ATOM	10994	HW2	SOL	3290	38.820	14.380	20.640	1.00	0.00
ATOM	10995	OW	SOL	3291	20.640	30.980	22.730	1.00	0.00
ATOM	10996	HW1	SOL	3291	19.710	31.170	22.580	1.00	0.00
ATOM	10997	HW2	SOL	3291	21.100	31.710	22.310	1.00	0.00
ATOM	10998	OW	SOL	3292	8.030	43.900	38.710	1.00	0.00
ATOM	10999	HW1	SOL	3292	7.660	43.560	37.900	1.00	0.00

ATOM	11000	HW2	SOL	3292	7.780	43.260	39.380	1.00	0.00
ATOM	11001	OW	SOL	3293	10.570	29.590	48.340	1.00	0.00
ATOM	11002	HW1	SOL	3293	10.960	29.510	49.210	1.00	0.00
ATOM	11003	HW2	SOL	3293	10.060	28.790	48.230	1.00	0.00
ATOM	11004	OW	SOL	3294	38.630	17.180	3.800	1.00	0.00
ATOM	11005	HW1	SOL	3294	37.970	17.030	4.480	1.00	0.00
ATOM	11006	HW2	SOL	3294	38.120	17.420	3.030	1.00	0.00
ATOM	11007	OW	SOL	3295	20.360	55.300	45.190	1.00	0.00
ATOM	11008	HW1	SOL	3295	20.050	54.760	44.470	1.00	0.00
ATOM	11009	HW2	SOL	3295	20.960	54.740	45.680	1.00	0.00
ATOM	11010	OW	SOL	3296	41.870	30.500	3.660	1.00	0.00
ATOM	11011	HW1	SOL	3296	41.830	29.550	3.760	1.00	0.00
ATOM	11012	HW2	SOL	3296	41.260	30.840	4.310	1.00	0.00
ATOM	11013	OW	SOL	3297	8.340	49.210	3.210	1.00	0.00
ATOM	11014	HW1	SOL	3297	9.230	49.010	3.500	1.00	0.00
ATOM	11015	HW2	SOL	3297	7.910	48.350	3.170	1.00	0.00
ATOM	11016	OW	SOL	3298	30.610	22.370	43.690	1.00	0.00
ATOM	11017	HW1	SOL	3298	30.210	22.430	42.830	1.00	0.00
ATOM	11018	HW2	SOL	3298	31.550	22.450	43.530	1.00	0.00
ATOM	11019	OW	SOL	3299	0.690	48.710	47.090	1.00	0.00
ATOM	11020	HW1	SOL	3299	0.430	49.390	47.700	1.00	0.00
ATOM	11021	HW2	SOL	3299	1.650	48.770	47.050	1.00	0.00
ATOM	11022	OW	SOL	3300	0.440	54.100	32.130	1.00	0.00
ATOM	11023	HW1	SOL	3300	0.370	53.160	32.260	1.00	0.00
ATOM	11024	HW2	SOL	3300	1.390	54.270	32.120	1.00	0.00
ATOM	11025	OW	SOL	3301	16.720	32.640	53.920	1.00	0.00
ATOM	11026	HW1	SOL	3301	16.860	33.500	53.530	1.00	0.00
ATOM	11027	HW2	SOL	3301	17.550	32.420	54.340	1.00	0.00
ATOM	11028	OW	SOL	3302	23.230	43.420	19.540	1.00	0.00
ATOM	11029	HW1	SOL	3302	24.070	43.850	19.420	1.00	0.00
ATOM	11030	HW2	SOL	3302	22.860	43.360	18.650	1.00	0.00
ATOM	11031	OW	SOL	3303	1.390	44.510	31.710	1.00	0.00
ATOM	11032	HW1	SOL	3303	0.870	43.710	31.740	1.00	0.00
ATOM	11033	HW2	SOL	3303	0.760	45.220	31.830	1.00	0.00
ATOM	11034	OW	SOL	3304	54.630	18.340	36.140	1.00	0.00
ATOM	11035	HW1	SOL	3304	55.320	17.780	36.500	1.00	0.00
ATOM	11036	HW2	SOL	3304	55.020	19.210	36.110	1.00	0.00
ATOM	11037	OW	SOL	3305	33.280	43.790	54.160	1.00	0.00
ATOM	11038	HW1	SOL	3305	33.020	42.870	54.140	1.00	0.00
ATOM	11039	HW2	SOL	3305	32.530	44.260	53.810	1.00	0.00
ATOM	11040	OW	SOL	3306	54.270	40.070	16.270	1.00	0.00
ATOM	11041	HW1	SOL	3306	54.130	39.860	15.350	1.00	0.00
ATOM	11042	HW2	SOL	3306	54.660	40.940	16.260	1.00	0.00
ATOM	11043	OW	SOL	3307	20.000	13.440	20.460	1.00	0.00

ATOM	11044	HW1	SOL	3307	20.130	14.350	20.180	1.00	0.00
ATOM	11045	HW2	SOL	3307	20.770	12.980	20.120	1.00	0.00
ATOM	11046	OW	SOL	3308	46.540	50.190	16.870	1.00	0.00
ATOM	11047	HW1	SOL	3308	46.600	51.080	17.220	1.00	0.00
ATOM	11048	HW2	SOL	3308	45.970	49.730	17.480	1.00	0.00
ATOM	11049	OW	SOL	3309	21.470	43.170	17.340	1.00	0.00
ATOM	11050	HW1	SOL	3309	21.340	42.320	16.930	1.00	0.00
ATOM	11051	HW2	SOL	3309	20.590	43.490	17.520	1.00	0.00
ATOM	11052	OW	SOL	3310	12.850	4.660	23.390	1.00	0.00
ATOM	11053	HW1	SOL	3310	12.200	4.970	24.020	1.00	0.00
ATOM	11054	HW2	SOL	3310	13.690	4.900	23.770	1.00	0.00
ATOM	11055	OW	SOL	3311	32.120	49.690	13.650	1.00	0.00
ATOM	11056	HW1	SOL	3311	32.360	49.030	13.000	1.00	0.00
ATOM	11057	HW2	SOL	3311	32.540	49.410	14.450	1.00	0.00
ATOM	11058	OW	SOL	3312	47.970	41.500	19.640	1.00	0.00
ATOM	11059	HW1	SOL	3312	48.630	40.840	19.830	1.00	0.00
ATOM	11060	HW2	SOL	3312	47.620	41.750	20.490	1.00	0.00
ATOM	11061	OW	SOL	3313	49.880	14.820	39.290	1.00	0.00
ATOM	11062	HW1	SOL	3313	49.810	15.310	40.110	1.00	0.00
ATOM	11063	HW2	SOL	3313	50.770	14.470	39.300	1.00	0.00
ATOM	11064	OW	SOL	3314	3.600	18.360	54.920	1.00	0.00
ATOM	11065	HW1	SOL	3314	2.680	18.620	54.910	1.00	0.00
ATOM	11066	HW2	SOL	3314	3.590	17.510	55.360	1.00	0.00
ATOM	11067	OW	SOL	3315	13.160	30.730	17.960	1.00	0.00
ATOM	11068	HW1	SOL	3315	12.550	30.520	18.670	1.00	0.00
ATOM	11069	HW2	SOL	3315	13.190	29.940	17.430	1.00	0.00
ATOM	11070	OW	SOL	3316	4.750	11.840	3.090	1.00	0.00
ATOM	11071	HW1	SOL	3316	4.820	11.200	2.380	1.00	0.00
ATOM	11072	HW2	SOL	3316	4.020	11.510	3.620	1.00	0.00
ATOM	11073	OW	SOL	3317	6.030	46.210	5.860	1.00	0.00
ATOM	11074	HW1	SOL	3317	5.900	45.450	5.300	1.00	0.00
ATOM	11075	HW2	SOL	3317	6.980	46.290	5.940	1.00	0.00
ATOM	11076	OW	SOL	3318	14.870	16.140	44.270	1.00	0.00
ATOM	11077	HW1	SOL	3318	14.970	16.650	43.470	1.00	0.00
ATOM	11078	HW2	SOL	3318	14.080	15.620	44.140	1.00	0.00
ATOM	11079	OW	SOL	3319	41.290	26.290	16.380	1.00	0.00
ATOM	11080	HW1	SOL	3319	42.180	25.970	16.240	1.00	0.00
ATOM	11081	HW2	SOL	3319	40.960	25.780	17.110	1.00	0.00
ATOM	11082	OW	SOL	3320	40.710	53.580	22.410	1.00	0.00
ATOM	11083	HW1	SOL	3320	41.650	53.670	22.230	1.00	0.00
ATOM	11084	HW2	SOL	3320	40.600	53.960	23.280	1.00	0.00
ATOM	11085	OW	SOL	3321	28.090	42.880	52.560	1.00	0.00
ATOM	11086	HW1	SOL	3321	27.760	42.370	51.820	1.00	0.00
ATOM	11087	HW2	SOL	3321	27.470	42.710	53.270	1.00	0.00

ATOM	11088	OW	SOL	3322	15.890	17.140	54.220	1.00	0.00
ATOM	11089	HW1	SOL	3322	15.330	17.510	53.540	1.00	0.00
ATOM	11090	HW2	SOL	3322	15.610	16.230	54.300	1.00	0.00
ATOM	11091	OW	SOL	3323	53.590	19.270	27.100	1.00	0.00
ATOM	11092	HW1	SOL	3323	53.220	19.050	27.950	1.00	0.00
ATOM	11093	HW2	SOL	3323	53.590	20.220	27.070	1.00	0.00
ATOM	11094	OW	SOL	3324	15.620	39.900	22.840	1.00	0.00
ATOM	11095	HW1	SOL	3324	15.390	39.000	23.070	1.00	0.00
ATOM	11096	HW2	SOL	3324	15.170	40.430	23.490	1.00	0.00
ATOM	11097	OW	SOL	3325	54.940	3.700	41.390	1.00	0.00
ATOM	11098	HW1	SOL	3325	55.310	3.280	40.620	1.00	0.00
ATOM	11099	HW2	SOL	3325	55.700	4.060	41.860	1.00	0.00
ATOM	11100	OW	SOL	3326	41.630	39.740	39.530	1.00	0.00
ATOM	11101	HW1	SOL	3326	41.040	39.460	38.830	1.00	0.00
ATOM	11102	HW2	SOL	3326	41.130	39.600	40.330	1.00	0.00
ATOM	11103	OW	SOL	3327	16.460	33.170	45.600	1.00	0.00
ATOM	11104	HW1	SOL	3327	16.830	33.580	46.380	1.00	0.00
ATOM	11105	HW2	SOL	3327	15.780	32.580	45.930	1.00	0.00
ATOM	11106	OW	SOL	3328	31.000	27.770	15.710	1.00	0.00
ATOM	11107	HW1	SOL	3328	30.600	27.730	16.580	1.00	0.00
ATOM	11108	HW2	SOL	3328	30.850	28.670	15.420	1.00	0.00
ATOM	11109	OW	SOL	3329	13.980	50.670	50.650	1.00	0.00
ATOM	11110	HW1	SOL	3329	13.430	51.450	50.760	1.00	0.00
ATOM	11111	HW2	SOL	3329	13.830	50.150	51.440	1.00	0.00
ATOM	11112	OW	SOL	3330	11.240	52.310	4.950	1.00	0.00
ATOM	11113	HW1	SOL	3330	10.830	51.950	4.170	1.00	0.00
ATOM	11114	HW2	SOL	3330	12.170	52.340	4.740	1.00	0.00
ATOM	11115	OW	SOL	3331	6.520	19.540	1.810	1.00	0.00
ATOM	11116	HW1	SOL	3331	5.580	19.710	1.770	1.00	0.00
ATOM	11117	HW2	SOL	3331	6.620	18.660	1.440	1.00	0.00
ATOM	11118	OW	SOL	3332	42.670	47.650	52.670	1.00	0.00
ATOM	11119	HW1	SOL	3332	43.110	47.280	53.430	1.00	0.00
ATOM	11120	HW2	SOL	3332	43.380	47.860	52.060	1.00	0.00
ATOM	11121	OW	SOL	3333	2.860	0.790	34.020	1.00	0.00
ATOM	11122	HW1	SOL	3333	3.130	0.010	33.530	1.00	0.00
ATOM	11123	HW2	SOL	3333	2.340	1.300	33.400	1.00	0.00
ATOM	11124	OW	SOL	3334	0.630	19.810	24.610	1.00	0.00
ATOM	11125	HW1	SOL	3334	1.230	19.750	25.350	1.00	0.00
ATOM	11126	HW2	SOL	3334	0.690	18.950	24.190	1.00	0.00
ATOM	11127	OW	SOL	3335	47.510	12.940	28.300	1.00	0.00
ATOM	11128	HW1	SOL	3335	46.620	12.940	27.940	1.00	0.00
ATOM	11129	HW2	SOL	3335	47.410	13.300	29.180	1.00	0.00
ATOM	11130	OW	SOL	3336	47.410	6.550	28.690	1.00	0.00
ATOM	11131	HW1	SOL	3336	48.130	5.920	28.680	1.00	0.00

ATOM	11132	HW2	SOL	3336	47.830	7.380	28.910	1.00	0.00
ATOM	11133	OW	SOL	3337	11.270	30.390	41.050	1.00	0.00
ATOM	11134	HW1	SOL	3337	12.060	30.590	40.550	1.00	0.00
ATOM	11135	HW2	SOL	3337	11.410	30.820	41.900	1.00	0.00
ATOM	11136	OW	SOL	3338	29.130	47.160	16.870	1.00	0.00
ATOM	11137	HW1	SOL	3338	29.820	47.000	17.520	1.00	0.00
ATOM	11138	HW2	SOL	3338	29.050	46.330	16.410	1.00	0.00
ATOM	11139	OW	SOL	3339	12.630	26.020	11.230	1.00	0.00
ATOM	11140	HW1	SOL	3339	12.540	25.180	10.780	1.00	0.00
ATOM	11141	HW2	SOL	3339	13.100	26.580	10.610	1.00	0.00
ATOM	11142	OW	SOL	3340	13.130	7.650	11.880	1.00	0.00
ATOM	11143	HW1	SOL	3340	12.410	7.340	12.430	1.00	0.00
ATOM	11144	HW2	SOL	3340	13.150	8.600	12.010	1.00	0.00
ATOM	11145	OW	SOL	3341	18.700	26.630	12.290	1.00	0.00
ATOM	11146	HW1	SOL	3341	19.340	27.290	12.520	1.00	0.00
ATOM	11147	HW2	SOL	3341	19.120	25.800	12.500	1.00	0.00
ATOM	11148	OW	SOL	3342	52.940	14.780	28.300	1.00	0.00
ATOM	11149	HW1	SOL	3342	52.170	14.230	28.450	1.00	0.00
ATOM	11150	HW2	SOL	3342	52.780	15.580	28.790	1.00	0.00
ATOM	11151	OW	SOL	3343	31.560	4.130	7.860	1.00	0.00
ATOM	11152	HW1	SOL	3343	30.730	3.770	8.160	1.00	0.00
ATOM	11153	HW2	SOL	3343	31.370	5.050	7.660	1.00	0.00
ATOM	11154	OW	SOL	3344	42.690	49.700	27.790	1.00	0.00
ATOM	11155	HW1	SOL	3344	43.240	48.930	27.730	1.00	0.00
ATOM	11156	HW2	SOL	3344	41.910	49.480	27.270	1.00	0.00
ATOM	11157	OW	SOL	3345	53.500	19.930	52.290	1.00	0.00
ATOM	11158	HW1	SOL	3345	54.450	20.030	52.350	1.00	0.00
ATOM	11159	HW2	SOL	3345	53.350	18.990	52.350	1.00	0.00
ATOM	11160	OW	SOL	3346	9.010	30.480	29.510	1.00	0.00
ATOM	11161	HW1	SOL	3346	8.100	30.300	29.310	1.00	0.00
ATOM	11162	HW2	SOL	3346	9.210	31.280	29.040	1.00	0.00
ATOM	11163	OW	SOL	3347	48.510	38.850	10.690	1.00	0.00
ATOM	11164	HW1	SOL	3347	47.640	39.050	10.330	1.00	0.00
ATOM	11165	HW2	SOL	3347	48.360	38.720	11.620	1.00	0.00
ATOM	11166	OW	SOL	3348	40.420	15.130	23.850	1.00	0.00
ATOM	11167	HW1	SOL	3348	39.570	15.020	24.270	1.00	0.00
ATOM	11168	HW2	SOL	3348	40.260	14.950	22.920	1.00	0.00
ATOM	11169	OW	SOL	3349	9.990	54.840	14.960	1.00	0.00
ATOM	11170	HW1	SOL	3349	10.650	55.520	14.820	1.00	0.00
ATOM	11171	HW2	SOL	3349	9.150	55.270	14.770	1.00	0.00
ATOM	11172	OW	SOL	3350	43.180	15.610	22.700	1.00	0.00
ATOM	11173	HW1	SOL	3350	42.780	15.960	21.910	1.00	0.00
ATOM	11174	HW2	SOL	3350	42.540	14.980	23.040	1.00	0.00
ATOM	11175	OW	SOL	3351	47.090	38.090	44.390	1.00	0.00

ATOM	11176	HW1	SOL	3351	46.440	38.650	43.970	1.00	0.00
ATOM	11177	HW2	SOL	3351	47.760	37.950	43.710	1.00	0.00
ATOM	11178	OW	SOL	3352	54.570	45.500	12.120	1.00	0.00
ATOM	11179	HW1	SOL	3352	54.040	45.690	11.340	1.00	0.00
ATOM	11180	HW2	SOL	3352	54.170	46.030	12.810	1.00	0.00
ATOM	11181	OW	SOL	3353	5.810	24.390	17.510	1.00	0.00
ATOM	11182	HW1	SOL	3353	4.940	24.040	17.310	1.00	0.00
ATOM	11183	HW2	SOL	3353	5.830	24.430	18.470	1.00	0.00
ATOM	11184	OW	SOL	3354	43.100	0.420	33.370	1.00	0.00
ATOM	11185	HW1	SOL	3354	43.290	1.250	33.800	1.00	0.00
ATOM	11186	HW2	SOL	3354	43.610	0.430	32.570	1.00	0.00
ATOM	11187	OW	SOL	3355	7.300	22.500	42.640	1.00	0.00
ATOM	11188	HW1	SOL	3355	8.110	22.320	43.100	1.00	0.00
ATOM	11189	HW2	SOL	3355	6.640	22.570	43.320	1.00	0.00
ATOM	11190	OW	SOL	3356	39.870	1.530	4.150	1.00	0.00
ATOM	11191	HW1	SOL	3356	40.800	1.650	3.940	1.00	0.00
ATOM	11192	HW2	SOL	3356	39.460	2.350	3.890	1.00	0.00
ATOM	11193	OW	SOL	3357	30.410	23.790	39.750	1.00	0.00
ATOM	11194	HW1	SOL	3357	31.030	23.900	40.460	1.00	0.00
ATOM	11195	HW2	SOL	3357	29.730	23.220	40.110	1.00	0.00
ATOM	11196	OW	SOL	3358	19.840	28.100	24.650	1.00	0.00
ATOM	11197	HW1	SOL	3358	20.740	27.920	24.910	1.00	0.00
ATOM	11198	HW2	SOL	3358	19.920	28.550	23.810	1.00	0.00
ATOM	11199	OW	SOL	3359	39.820	41.650	25.520	1.00	0.00
ATOM	11200	HW1	SOL	3359	39.780	41.190	24.680	1.00	0.00
ATOM	11201	HW2	SOL	3359	39.230	42.400	25.410	1.00	0.00
ATOM	11202	OW	SOL	3360	49.170	5.110	9.450	1.00	0.00
ATOM	11203	HW1	SOL	3360	49.380	4.890	10.360	1.00	0.00
ATOM	11204	HW2	SOL	3360	50.030	5.230	9.030	1.00	0.00
ATOM	11205	OW	SOL	3361	34.230	5.220	4.260	1.00	0.00
ATOM	11206	HW1	SOL	3361	35.140	5.220	4.570	1.00	0.00
ATOM	11207	HW2	SOL	3361	33.910	6.100	4.470	1.00	0.00
ATOM	11208	OW	SOL	3362	7.660	51.320	52.770	1.00	0.00
ATOM	11209	HW1	SOL	3362	7.690	51.790	51.940	1.00	0.00
ATOM	11210	HW2	SOL	3362	8.310	50.630	52.680	1.00	0.00
ATOM	11211	OW	SOL	3363	55.170	23.910	6.310	1.00	0.00
ATOM	11212	HW1	SOL	3363	55.520	24.740	6.640	1.00	0.00
ATOM	11213	HW2	SOL	3363	54.790	24.140	5.460	1.00	0.00
ATOM	11214	OW	SOL	3364	23.940	42.250	22.470	1.00	0.00
ATOM	11215	HW1	SOL	3364	24.490	41.820	21.820	1.00	0.00
ATOM	11216	HW2	SOL	3364	23.860	43.150	22.170	1.00	0.00
ATOM	11217	OW	SOL	3365	13.200	8.470	39.830	1.00	0.00
ATOM	11218	HW1	SOL	3365	13.730	9.080	40.330	1.00	0.00
ATOM	11219	HW2	SOL	3365	13.450	7.610	40.170	1.00	0.00

ATOM	11220	OW	SOL	3366	16.120	41.840	27.080	1.00	0.00
ATOM	11221	HW1	SOL	3366	15.710	42.450	27.690	1.00	0.00
ATOM	11222	HW2	SOL	3366	16.640	41.260	27.640	1.00	0.00
ATOM	11223	OW	SOL	3367	55.240	32.450	30.050	1.00	0.00
ATOM	11224	HW1	SOL	3367	55.240	31.500	30.140	1.00	0.00
ATOM	11225	HW2	SOL	3367	54.550	32.630	29.410	1.00	0.00
ATOM	11226	OW	SOL	3368	37.690	7.000	41.140	1.00	0.00
ATOM	11227	HW1	SOL	3368	37.390	7.900	41.220	1.00	0.00
ATOM	11228	HW2	SOL	3368	36.900	6.490	40.990	1.00	0.00
ATOM	11229	OW	SOL	3369	8.790	30.800	10.240	1.00	0.00
ATOM	11230	HW1	SOL	3369	8.800	30.010	9.710	1.00	0.00
ATOM	11231	HW2	SOL	3369	9.570	31.290	9.960	1.00	0.00
ATOM	11232	OW	SOL	3370	13.140	37.950	21.530	1.00	0.00
ATOM	11233	HW1	SOL	3370	13.120	37.990	22.480	1.00	0.00
ATOM	11234	HW2	SOL	3370	13.230	38.860	21.260	1.00	0.00
ATOM	11235	OW	SOL	3371	30.730	13.030	54.060	1.00	0.00
ATOM	11236	HW1	SOL	3371	30.600	13.620	53.320	1.00	0.00
ATOM	11237	HW2	SOL	3371	31.640	13.190	54.340	1.00	0.00
ATOM	11238	OW	SOL	3372	20.870	16.120	47.730	1.00	0.00
ATOM	11239	HW1	SOL	3372	20.270	15.910	48.450	1.00	0.00
ATOM	11240	HW2	SOL	3372	20.690	15.450	47.070	1.00	0.00
ATOM	11241	OW	SOL	3373	31.090	20.200	52.520	1.00	0.00
ATOM	11242	HW1	SOL	3373	30.910	19.290	52.290	1.00	0.00
ATOM	11243	HW2	SOL	3373	30.640	20.330	53.360	1.00	0.00
ATOM	11244	OW	SOL	3374	19.140	12.490	43.460	1.00	0.00
ATOM	11245	HW1	SOL	3374	20.080	12.350	43.440	1.00	0.00
ATOM	11246	HW2	SOL	3374	19.020	13.220	44.070	1.00	0.00
ATOM	11247	OW	SOL	3375	7.550	50.340	27.850	1.00	0.00
ATOM	11248	HW1	SOL	3375	8.240	49.730	27.600	1.00	0.00
ATOM	11249	HW2	SOL	3375	6.750	49.810	27.810	1.00	0.00
ATOM	11250	OW	SOL	3376	19.310	43.990	54.950	1.00	0.00
ATOM	11251	HW1	SOL	3376	20.040	44.350	55.460	1.00	0.00
ATOM	11252	HW2	SOL	3376	19.340	44.470	54.120	1.00	0.00
ATOM	11253	OW	SOL	3377	2.860	9.380	51.720	1.00	0.00
ATOM	11254	HW1	SOL	3377	2.330	8.590	51.840	1.00	0.00
ATOM	11255	HW2	SOL	3377	3.620	9.090	51.210	1.00	0.00
ATOM	11256	OW	SOL	3378	5.550	39.240	51.330	1.00	0.00
ATOM	11257	HW1	SOL	3378	6.110	39.800	51.860	1.00	0.00
ATOM	11258	HW2	SOL	3378	4.660	39.400	51.650	1.00	0.00
ATOM	11259	OW	SOL	3379	24.060	10.130	36.930	1.00	0.00
ATOM	11260	HW1	SOL	3379	23.240	10.310	37.380	1.00	0.00
ATOM	11261	HW2	SOL	3379	23.950	10.530	36.060	1.00	0.00
ATOM	11262	OW	SOL	3380	25.160	52.680	37.610	1.00	0.00
ATOM	11263	HW1	SOL	3380	24.950	51.770	37.760	1.00	0.00

ATOM	11264	HW2	SOL	3380	24.380	53.050	37.200	1.00	0.00
ATOM	11265	OW	SOL	3381	14.780	36.830	45.030	1.00	0.00
ATOM	11266	HW1	SOL	3381	14.210	36.520	44.330	1.00	0.00
ATOM	11267	HW2	SOL	3381	14.550	36.300	45.780	1.00	0.00
ATOM	11268	OW	SOL	3382	8.880	24.390	33.960	1.00	0.00
ATOM	11269	HW1	SOL	3382	9.560	25.020	33.730	1.00	0.00
ATOM	11270	HW2	SOL	3382	8.720	23.900	33.150	1.00	0.00
ATOM	11271	OW	SOL	3383	44.130	10.010	1.330	1.00	0.00
ATOM	11272	HW1	SOL	3383	44.970	10.470	1.280	1.00	0.00
ATOM	11273	HW2	SOL	3383	43.550	10.520	0.760	1.00	0.00
ATOM	11274	OW	SOL	3384	34.490	7.410	50.320	1.00	0.00
ATOM	11275	HW1	SOL	3384	33.590	7.130	50.490	1.00	0.00
ATOM	11276	HW2	SOL	3384	34.920	6.640	49.950	1.00	0.00
ATOM	11277	OW	SOL	3385	36.360	20.320	31.010	1.00	0.00
ATOM	11278	HW1	SOL	3385	36.590	19.390	31.030	1.00	0.00
ATOM	11279	HW2	SOL	3385	36.180	20.550	31.920	1.00	0.00
ATOM	11280	OW	SOL	3386	31.320	27.250	45.720	1.00	0.00
ATOM	11281	HW1	SOL	3386	31.620	27.240	46.630	1.00	0.00
ATOM	11282	HW2	SOL	3386	30.390	27.030	45.760	1.00	0.00
ATOM	11283	OW	SOL	3387	2.020	2.290	55.230	1.00	0.00
ATOM	11284	HW1	SOL	3387	1.860	2.810	56.010	1.00	0.00
ATOM	11285	HW2	SOL	3387	1.850	1.380	55.500	1.00	0.00
ATOM	11286	OW	SOL	3388	49.860	47.420	48.850	1.00	0.00
ATOM	11287	HW1	SOL	3388	49.100	47.900	48.520	1.00	0.00
ATOM	11288	HW2	SOL	3388	49.500	46.650	49.280	1.00	0.00
ATOM	11289	OW	SOL	3389	51.870	15.020	25.690	1.00	0.00
ATOM	11290	HW1	SOL	3389	52.730	14.690	25.970	1.00	0.00
ATOM	11291	HW2	SOL	3389	52.040	15.920	25.400	1.00	0.00
ATOM	11292	OW	SOL	3390	33.750	36.250	45.190	1.00	0.00
ATOM	11293	HW1	SOL	3390	34.140	35.600	44.600	1.00	0.00
ATOM	11294	HW2	SOL	3390	34.040	35.990	46.060	1.00	0.00
ATOM	11295	OW	SOL	3391	10.510	10.910	35.230	1.00	0.00
ATOM	11296	HW1	SOL	3391	10.480	11.640	35.840	1.00	0.00
ATOM	11297	HW2	SOL	3391	10.820	10.170	35.750	1.00	0.00
ATOM	11298	OW	SOL	3392	24.610	38.490	26.340	1.00	0.00
ATOM	11299	HW1	SOL	3392	24.170	38.300	27.170	1.00	0.00
ATOM	11300	HW2	SOL	3392	24.770	39.430	26.360	1.00	0.00
ATOM	11301	OW	SOL	3393	3.850	53.000	4.560	1.00	0.00
ATOM	11302	HW1	SOL	3393	4.440	52.320	4.220	1.00	0.00
ATOM	11303	HW2	SOL	3393	3.930	53.710	3.920	1.00	0.00
ATOM	11304	OW	SOL	3394	49.820	21.190	7.860	1.00	0.00
ATOM	11305	HW1	SOL	3394	50.650	21.510	7.500	1.00	0.00
ATOM	11306	HW2	SOL	3394	49.800	21.520	8.750	1.00	0.00
ATOM	11307	OW	SOL	3395	23.590	48.670	30.550	1.00	0.00

ATOM	11308	HW1	SOL	3395	23.180	48.330	31.350	1.00	0.00
ATOM	11309	HW2	SOL	3395	23.700	49.600	30.720	1.00	0.00
ATOM	11310	OW	SOL	3396	6.080	9.340	35.580	1.00	0.00
ATOM	11311	HW1	SOL	3396	6.910	8.870	35.520	1.00	0.00
ATOM	11312	HW2	SOL	3396	5.610	8.930	36.300	1.00	0.00
ATOM	11313	OW	SOL	3397	21.530	47.140	20.900	1.00	0.00
ATOM	11314	HW1	SOL	3397	22.210	47.490	20.320	1.00	0.00
ATOM	11315	HW2	SOL	3397	21.730	47.520	21.760	1.00	0.00
ATOM	11316	OW	SOL	3398	52.870	9.310	27.880	1.00	0.00
ATOM	11317	HW1	SOL	3398	53.200	9.180	26.990	1.00	0.00
ATOM	11318	HW2	SOL	3398	53.260	10.140	28.150	1.00	0.00
ATOM	11319	OW	SOL	3399	18.770	12.300	29.340	1.00	0.00
ATOM	11320	HW1	SOL	3399	18.860	12.400	30.290	1.00	0.00
ATOM	11321	HW2	SOL	3399	17.990	11.760	29.230	1.00	0.00
ATOM	11322	OW	SOL	3400	42.190	13.070	34.460	1.00	0.00
ATOM	11323	HW1	SOL	3400	42.360	13.350	35.360	1.00	0.00
ATOM	11324	HW2	SOL	3400	42.200	13.880	33.960	1.00	0.00
ATOM	11325	OW	SOL	3401	8.560	31.740	7.360	1.00	0.00
ATOM	11326	HW1	SOL	3401	8.860	30.910	7.750	1.00	0.00
ATOM	11327	HW2	SOL	3401	7.610	31.700	7.430	1.00	0.00
ATOM	11328	OW	SOL	3402	21.100	19.680	44.070	1.00	0.00
ATOM	11329	HW1	SOL	3402	20.510	20.170	44.640	1.00	0.00
ATOM	11330	HW2	SOL	3402	21.970	19.820	44.450	1.00	0.00
ATOM	11331	OW	SOL	3403	47.760	26.540	49.990	1.00	0.00
ATOM	11332	HW1	SOL	3403	47.150	26.590	49.250	1.00	0.00
ATOM	11333	HW2	SOL	3403	48.530	27.020	49.690	1.00	0.00
ATOM	11334	OW	SOL	3404	43.590	46.300	54.890	1.00	0.00
ATOM	11335	HW1	SOL	3404	43.040	45.690	55.390	1.00	0.00
ATOM	11336	HW2	SOL	3404	44.430	46.300	55.350	1.00	0.00
ATOM	11337	OW	SOL	3405	48.470	33.460	44.890	1.00	0.00
ATOM	11338	HW1	SOL	3405	48.770	32.870	45.580	1.00	0.00
ATOM	11339	HW2	SOL	3405	49.180	34.100	44.790	1.00	0.00
ATOM	11340	OW	SOL	3406	10.670	14.970	32.400	1.00	0.00
ATOM	11341	HW1	SOL	3406	9.840	14.510	32.540	1.00	0.00
ATOM	11342	HW2	SOL	3406	10.670	15.160	31.460	1.00	0.00
ATOM	11343	OW	SOL	3407	26.470	24.840	1.640	1.00	0.00
ATOM	11344	HW1	SOL	3407	25.820	25.310	2.170	1.00	0.00
ATOM	11345	HW2	SOL	3407	26.390	23.930	1.920	1.00	0.00
ATOM	11346	OW	SOL	3408	37.850	22.590	43.270	1.00	0.00
ATOM	11347	HW1	SOL	3408	36.900	22.690	43.310	1.00	0.00
ATOM	11348	HW2	SOL	3408	38.030	21.780	43.750	1.00	0.00
ATOM	11349	OW	SOL	3409	3.000	18.530	20.010	1.00	0.00
ATOM	11350	HW1	SOL	3409	2.050	18.510	20.090	1.00	0.00
ATOM	11351	HW2	SOL	3409	3.260	19.350	20.430	1.00	0.00

ATOM	11352	OW	SOL	3410	47.290	48.480	19.850	1.00	0.00
ATOM	11353	HW1	SOL	3410	46.480	48.800	19.460	1.00	0.00
ATOM	11354	HW2	SOL	3410	47.370	47.580	19.550	1.00	0.00
ATOM	11355	OW	SOL	3411	40.200	4.380	17.570	1.00	0.00
ATOM	11356	HW1	SOL	3411	41.150	4.440	17.590	1.00	0.00
ATOM	11357	HW2	SOL	3411	39.990	4.280	16.640	1.00	0.00
ATOM	11358	OW	SOL	3412	18.350	43.940	47.660	1.00	0.00
ATOM	11359	HW1	SOL	3412	18.320	43.140	48.180	1.00	0.00
ATOM	11360	HW2	SOL	3412	19.280	44.130	47.570	1.00	0.00
ATOM	11361	OW	SOL	3413	37.190	15.790	46.840	1.00	0.00
ATOM	11362	HW1	SOL	3413	37.900	15.430	47.360	1.00	0.00
ATOM	11363	HW2	SOL	3413	36.460	15.880	47.450	1.00	0.00
ATOM	11364	OW	SOL	3414	23.720	24.560	2.610	1.00	0.00
ATOM	11365	HW1	SOL	3414	23.530	24.170	1.760	1.00	0.00
ATOM	11366	HW2	SOL	3414	23.180	24.080	3.230	1.00	0.00
ATOM	11367	OW	SOL	3415	5.360	17.580	8.790	1.00	0.00
ATOM	11368	HW1	SOL	3415	5.540	16.770	8.300	1.00	0.00
ATOM	11369	HW2	SOL	3415	5.360	18.270	8.120	1.00	0.00
ATOM	11370	OW	SOL	3416	40.370	8.880	41.250	1.00	0.00
ATOM	11371	HW1	SOL	3416	39.970	8.020	41.420	1.00	0.00
ATOM	11372	HW2	SOL	3416	40.730	8.800	40.360	1.00	0.00
ATOM	11373	OW	SOL	3417	23.310	14.560	45.040	1.00	0.00
ATOM	11374	HW1	SOL	3417	23.750	14.370	44.200	1.00	0.00
ATOM	11375	HW2	SOL	3417	22.830	15.370	44.880	1.00	0.00
ATOM	11376	OW	SOL	3418	42.970	0.360	30.340	1.00	0.00
ATOM	11377	HW1	SOL	3418	42.490	0.400	29.520	1.00	0.00
ATOM	11378	HW2	SOL	3418	43.620	1.070	30.270	1.00	0.00
ATOM	11379	OW	SOL	3419	36.370	23.750	50.430	1.00	0.00
ATOM	11380	HW1	SOL	3419	36.930	24.200	51.070	1.00	0.00
ATOM	11381	HW2	SOL	3419	35.500	23.750	50.830	1.00	0.00
ATOM	11382	OW	SOL	3420	44.090	31.240	54.870	1.00	0.00
ATOM	11383	HW1	SOL	3420	43.950	30.450	55.390	1.00	0.00
ATOM	11384	HW2	SOL	3420	44.930	31.090	54.430	1.00	0.00
ATOM	11385	OW	SOL	3421	45.290	17.190	22.760	1.00	0.00
ATOM	11386	HW1	SOL	3421	44.430	16.790	22.680	1.00	0.00
ATOM	11387	HW2	SOL	3421	45.780	16.860	22.010	1.00	0.00
ATOM	11388	OW	SOL	3422	30.400	52.630	22.920	1.00	0.00
ATOM	11389	HW1	SOL	3422	30.720	51.880	23.420	1.00	0.00
ATOM	11390	HW2	SOL	3422	30.370	52.320	22.020	1.00	0.00
ATOM	11391	OW	SOL	3423	37.750	8.970	11.120	1.00	0.00
ATOM	11392	HW1	SOL	3423	38.390	9.430	10.580	1.00	0.00
ATOM	11393	HW2	SOL	3423	38.100	9.030	12.010	1.00	0.00
ATOM	11394	OW	SOL	3424	18.800	45.350	41.900	1.00	0.00
ATOM	11395	HW1	SOL	3424	18.980	45.570	42.810	1.00	0.00

ATOM	11396	HW2	SOL	3424	19.520	44.750	41.660	1.00	0.00
ATOM	11397	OW	SOL	3425	31.420	44.940	40.850	1.00	0.00
ATOM	11398	HW1	SOL	3425	31.090	44.220	41.390	1.00	0.00
ATOM	11399	HW2	SOL	3425	31.110	45.730	41.290	1.00	0.00
ATOM	11400	OW	SOL	3426	39.790	35.800	20.850	1.00	0.00
ATOM	11401	HW1	SOL	3426	39.170	36.490	20.640	1.00	0.00
ATOM	11402	HW2	SOL	3426	39.250	35.120	21.270	1.00	0.00
ATOM	11403	OW	SOL	3427	6.260	17.290	14.510	1.00	0.00
ATOM	11404	HW1	SOL	3427	5.360	17.580	14.330	1.00	0.00
ATOM	11405	HW2	SOL	3427	6.800	18.060	14.340	1.00	0.00
ATOM	11406	OW	SOL	3428	10.710	36.700	21.150	1.00	0.00
ATOM	11407	HW1	SOL	3428	11.660	36.820	21.130	1.00	0.00
ATOM	11408	HW2	SOL	3428	10.380	37.470	21.620	1.00	0.00
ATOM	11409	OW	SOL	3429	20.220	12.620	35.970	1.00	0.00
ATOM	11410	HW1	SOL	3429	20.220	12.920	35.060	1.00	0.00
ATOM	11411	HW2	SOL	3429	19.550	13.150	36.390	1.00	0.00
ATOM	11412	OW	SOL	3430	19.610	4.840	16.900	1.00	0.00
ATOM	11413	HW1	SOL	3430	19.420	4.280	17.660	1.00	0.00
ATOM	11414	HW2	SOL	3430	20.320	5.410	17.200	1.00	0.00
ATOM	11415	OW	SOL	3431	32.690	17.240	25.190	1.00	0.00
ATOM	11416	HW1	SOL	3431	33.190	17.780	25.810	1.00	0.00
ATOM	11417	HW2	SOL	3431	31.780	17.430	25.390	1.00	0.00
ATOM	11418	OW	SOL	3432	27.310	20.510	52.680	1.00	0.00
ATOM	11419	HW1	SOL	3432	26.490	20.550	52.180	1.00	0.00
ATOM	11420	HW2	SOL	3432	27.410	19.580	52.890	1.00	0.00
ATOM	11421	OW	SOL	3433	7.710	35.900	29.300	1.00	0.00
ATOM	11422	HW1	SOL	3433	7.580	36.260	30.170	1.00	0.00
ATOM	11423	HW2	SOL	3433	7.000	36.280	28.770	1.00	0.00
ATOM	11424	OW	SOL	3434	44.770	36.830	51.440	1.00	0.00
ATOM	11425	HW1	SOL	3434	45.100	37.730	51.400	1.00	0.00
ATOM	11426	HW2	SOL	3434	44.700	36.650	52.380	1.00	0.00
ATOM	11427	OW	SOL	3435	15.110	9.200	15.100	1.00	0.00
ATOM	11428	HW1	SOL	3435	15.990	9.020	14.780	1.00	0.00
ATOM	11429	HW2	SOL	3435	14.680	8.350	15.100	1.00	0.00
ATOM	11430	OW	SOL	3436	44.770	45.460	31.350	1.00	0.00
ATOM	11431	HW1	SOL	3436	44.380	45.080	30.560	1.00	0.00
ATOM	11432	HW2	SOL	3436	44.320	46.300	31.450	1.00	0.00
ATOM	11433	OW	SOL	3437	36.030	8.470	52.650	1.00	0.00
ATOM	11434	HW1	SOL	3437	35.810	8.270	51.740	1.00	0.00
ATOM	11435	HW2	SOL	3437	35.310	8.090	53.160	1.00	0.00
ATOM	11436	OW	SOL	3438	30.130	37.830	4.310	1.00	0.00
ATOM	11437	HW1	SOL	3438	29.230	38.120	4.460	1.00	0.00
ATOM	11438	HW2	SOL	3438	30.350	38.170	3.440	1.00	0.00
ATOM	11439	OW	SOL	3439	19.810	35.630	14.390	1.00	0.00

ATOM	11440	HW1	SOL	3439	19.420	35.540	15.260	1.00	0.00
ATOM	11441	HW2	SOL	3439	19.560	36.510	14.110	1.00	0.00
ATOM	11442	OW	SOL	3440	30.460	4.250	25.250	1.00	0.00
ATOM	11443	HW1	SOL	3440	31.360	4.410	24.940	1.00	0.00
ATOM	11444	HW2	SOL	3440	29.970	4.030	24.460	1.00	0.00
ATOM	11445	OW	SOL	3441	19.760	15.230	14.640	1.00	0.00
ATOM	11446	HW1	SOL	3441	19.940	15.730	15.440	1.00	0.00
ATOM	11447	HW2	SOL	3441	20.330	14.470	14.690	1.00	0.00
ATOM	11448	OW	SOL	3442	32.230	26.790	36.400	1.00	0.00
ATOM	11449	HW1	SOL	3442	31.380	26.360	36.310	1.00	0.00
ATOM	11450	HW2	SOL	3442	32.030	27.590	36.900	1.00	0.00
ATOM	11451	OW	SOL	3443	23.800	35.740	30.550	1.00	0.00
ATOM	11452	HW1	SOL	3443	24.190	36.460	30.050	1.00	0.00
ATOM	11453	HW2	SOL	3443	24.060	34.950	30.070	1.00	0.00
ATOM	11454	OW	SOL	3444	22.440	28.930	25.460	1.00	0.00
ATOM	11455	HW1	SOL	3444	23.200	29.480	25.280	1.00	0.00
ATOM	11456	HW2	SOL	3444	22.740	28.330	26.140	1.00	0.00
ATOM	11457	OW	SOL	3445	1.130	11.270	51.080	1.00	0.00
ATOM	11458	HW1	SOL	3445	1.800	10.670	51.410	1.00	0.00
ATOM	11459	HW2	SOL	3445	0.860	10.880	50.240	1.00	0.00
ATOM	11460	OW	SOL	3446	48.360	38.540	16.940	1.00	0.00
ATOM	11461	HW1	SOL	3446	47.650	37.910	16.870	1.00	0.00
ATOM	11462	HW2	SOL	3446	48.690	38.450	17.830	1.00	0.00
ATOM	11463	OW	SOL	3447	36.800	42.570	41.360	1.00	0.00
ATOM	11464	HW1	SOL	3447	36.830	43.230	42.050	1.00	0.00
ATOM	11465	HW2	SOL	3447	36.310	41.850	41.740	1.00	0.00
ATOM	11466	OW	SOL	3448	39.360	49.010	41.190	1.00	0.00
ATOM	11467	HW1	SOL	3448	39.440	48.420	40.430	1.00	0.00
ATOM	11468	HW2	SOL	3448	38.950	48.470	41.860	1.00	0.00
ATOM	11469	OW	SOL	3449	52.720	52.080	26.460	1.00	0.00
ATOM	11470	HW1	SOL	3449	51.850	52.250	26.100	1.00	0.00
ATOM	11471	HW2	SOL	3449	52.880	51.150	26.270	1.00	0.00
ATOM	11472	OW	SOL	3450	50.020	41.100	2.490	1.00	0.00
ATOM	11473	HW1	SOL	3450	50.530	41.040	1.680	1.00	0.00
ATOM	11474	HW2	SOL	3450	49.570	41.940	2.430	1.00	0.00
ATOM	11475	OW	SOL	3451	2.270	5.230	27.690	1.00	0.00
ATOM	11476	HW1	SOL	3451	2.890	5.950	27.800	1.00	0.00
ATOM	11477	HW2	SOL	3451	2.510	4.840	26.850	1.00	0.00
ATOM	11478	OW	SOL	3452	52.760	6.230	12.010	1.00	0.00
ATOM	11479	HW1	SOL	3452	51.840	6.470	12.060	1.00	0.00
ATOM	11480	HW2	SOL	3452	52.780	5.480	11.420	1.00	0.00
ATOM	11481	OW	SOL	3453	3.500	34.440	16.770	1.00	0.00
ATOM	11482	HW1	SOL	3453	4.130	34.810	16.150	1.00	0.00
ATOM	11483	HW2	SOL	3453	3.910	34.550	17.630	1.00	0.00

ATOM	11484	OW	SOL	3454	40.700	6.510	22.850	1.00	0.00
ATOM	11485	HW1	SOL	3454	41.150	7.000	23.530	1.00	0.00
ATOM	11486	HW2	SOL	3454	40.060	7.130	22.500	1.00	0.00
ATOM	11487	OW	SOL	3455	38.760	35.770	42.800	1.00	0.00
ATOM	11488	HW1	SOL	3455	39.340	36.400	43.230	1.00	0.00
ATOM	11489	HW2	SOL	3455	37.930	36.230	42.720	1.00	0.00
ATOM	11490	OW	SOL	3456	45.730	37.920	23.350	1.00	0.00
ATOM	11491	HW1	SOL	3456	45.640	36.980	23.250	1.00	0.00
ATOM	11492	HW2	SOL	3456	45.760	38.260	22.450	1.00	0.00
ATOM	11493	OW	SOL	3457	23.540	11.610	34.900	1.00	0.00
ATOM	11494	HW1	SOL	3457	22.780	11.860	34.370	1.00	0.00
ATOM	11495	HW2	SOL	3457	24.110	12.370	34.880	1.00	0.00
ATOM	11496	OW	SOL	3458	7.350	52.430	33.920	1.00	0.00
ATOM	11497	HW1	SOL	3458	8.200	52.210	34.310	1.00	0.00
ATOM	11498	HW2	SOL	3458	7.430	53.350	33.660	1.00	0.00
ATOM	11499	OW	SOL	3459	35.600	0.460	13.530	1.00	0.00
ATOM	11500	HW1	SOL	3459	35.930	-0.210	14.130	1.00	0.00
ATOM	11501	HW2	SOL	3459	34.800	0.790	13.960	1.00	0.00
ATOM	11502	OW	SOL	3460	0.480	49.020	54.290	1.00	0.00
ATOM	11503	HW1	SOL	3460	-0.410	48.780	54.020	1.00	0.00
ATOM	11504	HW2	SOL	3460	1.050	48.620	53.620	1.00	0.00
ATOM	11505	OW	SOL	3461	19.770	47.520	13.750	1.00	0.00
ATOM	11506	HW1	SOL	3461	19.220	46.790	13.470	1.00	0.00
ATOM	11507	HW2	SOL	3461	20.000	47.980	12.950	1.00	0.00
ATOM	11508	OW	SOL	3462	41.470	43.440	5.950	1.00	0.00
ATOM	11509	HW1	SOL	3462	42.260	43.770	6.360	1.00	0.00
ATOM	11510	HW2	SOL	3462	41.660	43.440	5.010	1.00	0.00
ATOM	11511	OW	SOL	3463	41.690	46.410	17.810	1.00	0.00
ATOM	11512	HW1	SOL	3463	41.700	46.690	16.900	1.00	0.00
ATOM	11513	HW2	SOL	3463	42.580	46.580	18.120	1.00	0.00
ATOM	11514	OW	SOL	3464	0.170	29.440	42.630	1.00	0.00
ATOM	11515	HW1	SOL	3464	0.680	30.110	42.180	1.00	0.00
ATOM	11516	HW2	SOL	3464	0.100	29.760	43.530	1.00	0.00
ATOM	11517	OW	SOL	3465	30.950	47.320	5.940	1.00	0.00
ATOM	11518	HW1	SOL	3465	31.060	46.410	5.700	1.00	0.00
ATOM	11519	HW2	SOL	3465	31.840	47.660	6.020	1.00	0.00
ATOM	11520	OW	SOL	3466	32.750	4.190	27.760	1.00	0.00
ATOM	11521	HW1	SOL	3466	32.310	3.350	27.910	1.00	0.00
ATOM	11522	HW2	SOL	3466	32.310	4.560	27.000	1.00	0.00
ATOM	11523	OW	SOL	3467	36.860	3.880	9.380	1.00	0.00
ATOM	11524	HW1	SOL	3467	36.790	4.840	9.380	1.00	0.00
ATOM	11525	HW2	SOL	3467	37.780	3.700	9.530	1.00	0.00
ATOM	11526	OW	SOL	3468	53.230	10.250	10.310	1.00	0.00
ATOM	11527	HW1	SOL	3468	53.390	10.450	11.230	1.00	0.00

ATOM	11528	HW2	SOL	3468	54.050	10.460	9.870	1.00	0.00
ATOM	11529	OW	SOL	3469	46.580	26.280	3.070	1.00	0.00
ATOM	11530	HW1	SOL	3469	47.460	26.040	3.330	1.00	0.00
ATOM	11531	HW2	SOL	3469	46.030	26.030	3.820	1.00	0.00
ATOM	11532	OW	SOL	3470	6.050	17.870	45.440	1.00	0.00
ATOM	11533	HW1	SOL	3470	5.270	17.920	44.880	1.00	0.00
ATOM	11534	HW2	SOL	3470	6.560	18.640	45.190	1.00	0.00
ATOM	11535	OW	SOL	3471	37.900	55.040	15.660	1.00	0.00
ATOM	11536	HW1	SOL	3471	37.120	55.070	16.210	1.00	0.00
ATOM	11537	HW2	SOL	3471	38.000	54.110	15.460	1.00	0.00
ATOM	11538	OW	SOL	3472	50.860	5.610	33.310	1.00	0.00
ATOM	11539	HW1	SOL	3472	50.540	6.390	32.870	1.00	0.00
ATOM	11540	HW2	SOL	3472	50.570	4.890	32.750	1.00	0.00
ATOM	11541	OW	SOL	3473	19.170	4.840	1.650	1.00	0.00
ATOM	11542	HW1	SOL	3473	19.940	4.650	2.180	1.00	0.00
ATOM	11543	HW2	SOL	3473	19.420	5.610	1.140	1.00	0.00
ATOM	11544	OW	SOL	3474	24.160	0.760	24.200	1.00	0.00
ATOM	11545	HW1	SOL	3474	24.240	1.420	23.510	1.00	0.00
ATOM	11546	HW2	SOL	3474	23.330	0.320	24.020	1.00	0.00
ATOM	11547	OW	SOL	3475	25.910	54.870	11.960	1.00	0.00
ATOM	11548	HW1	SOL	3475	26.390	54.620	11.170	1.00	0.00
ATOM	11549	HW2	SOL	3475	25.010	54.570	11.800	1.00	0.00
ATOM	11550	OW	SOL	3476	55.870	28.930	15.450	1.00	0.00
ATOM	11551	HW1	SOL	3476	55.060	29.040	15.950	1.00	0.00
ATOM	11552	HW2	SOL	3476	56.080	29.810	15.150	1.00	0.00
ATOM	11553	OW	SOL	3477	48.280	18.510	10.280	1.00	0.00
ATOM	11554	HW1	SOL	3477	47.480	18.930	10.600	1.00	0.00
ATOM	11555	HW2	SOL	3477	48.490	17.850	10.940	1.00	0.00
ATOM	11556	OW	SOL	3478	15.550	27.930	23.860	1.00	0.00
ATOM	11557	HW1	SOL	3478	16.130	28.680	23.900	1.00	0.00
ATOM	11558	HW2	SOL	3478	16.120	27.180	23.690	1.00	0.00
ATOM	11559	OW	SOL	3479	39.020	29.950	0.340	1.00	0.00
ATOM	11560	HW1	SOL	3479	39.130	29.780	1.270	1.00	0.00
ATOM	11561	HW2	SOL	3479	39.700	30.580	0.120	1.00	0.00
ATOM	11562	OW	SOL	3480	7.630	33.690	22.670	1.00	0.00
ATOM	11563	HW1	SOL	3480	8.490	33.700	22.240	1.00	0.00
ATOM	11564	HW2	SOL	3480	7.550	32.800	23.010	1.00	0.00
ATOM	11565	OW	SOL	3481	53.440	19.420	8.090	1.00	0.00
ATOM	11566	HW1	SOL	3481	53.210	19.000	7.260	1.00	0.00
ATOM	11567	HW2	SOL	3481	53.940	20.200	7.830	1.00	0.00
ATOM	11568	OW	SOL	3482	24.860	18.680	15.040	1.00	0.00
ATOM	11569	HW1	SOL	3482	24.530	18.690	14.140	1.00	0.00
ATOM	11570	HW2	SOL	3482	25.790	18.900	14.950	1.00	0.00
ATOM	11571	OW	SOL	3483	35.560	35.470	23.780	1.00	0.00

ATOM	11572	HW1	SOL	3483	36.190	36.180	23.910	1.00	0.00
ATOM	11573	HW2	SOL	3483	35.680	34.900	24.540	1.00	0.00
ATOM	11574	OW	SOL	3484	52.750	18.920	44.070	1.00	0.00
ATOM	11575	HW1	SOL	3484	51.930	18.560	44.420	1.00	0.00
ATOM	11576	HW2	SOL	3484	52.900	19.720	44.580	1.00	0.00
ATOM	11577	OW	SOL	3485	16.400	53.510	9.940	1.00	0.00
ATOM	11578	HW1	SOL	3485	15.590	53.230	10.390	1.00	0.00
ATOM	11579	HW2	SOL	3485	17.060	52.880	10.240	1.00	0.00
ATOM	11580	OW	SOL	3486	54.950	25.810	21.200	1.00	0.00
ATOM	11581	HW1	SOL	3486	55.400	26.460	20.670	1.00	0.00
ATOM	11582	HW2	SOL	3486	54.700	26.280	22.000	1.00	0.00
ATOM	11583	OW	SOL	3487	19.530	54.650	33.890	1.00	0.00
ATOM	11584	HW1	SOL	3487	19.290	55.520	34.210	1.00	0.00
ATOM	11585	HW2	SOL	3487	19.290	54.650	32.970	1.00	0.00
ATOM	11586	OW	SOL	3488	18.950	20.600	45.570	1.00	0.00
ATOM	11587	HW1	SOL	3488	19.060	21.540	45.740	1.00	0.00
ATOM	11588	HW2	SOL	3488	18.850	20.210	46.430	1.00	0.00
ATOM	11589	OW	SOL	3489	19.750	16.540	55.610	1.00	0.00
ATOM	11590	HW1	SOL	3489	20.450	15.890	55.700	1.00	0.00
ATOM	11591	HW2	SOL	3489	19.910	16.950	54.760	1.00	0.00
ATOM	11592	OW	SOL	3490	17.500	5.280	8.840	1.00	0.00
ATOM	11593	HW1	SOL	3490	17.500	5.910	9.560	1.00	0.00
ATOM	11594	HW2	SOL	3490	17.960	5.720	8.120	1.00	0.00
ATOM	11595	OW	SOL	3491	27.270	23.070	21.280	1.00	0.00
ATOM	11596	HW1	SOL	3491	27.540	22.400	20.660	1.00	0.00
ATOM	11597	HW2	SOL	3491	28.090	23.480	21.570	1.00	0.00
ATOM	11598	OW	SOL	3492	30.320	17.750	41.910	1.00	0.00
ATOM	11599	HW1	SOL	3492	29.640	17.570	42.560	1.00	0.00
ATOM	11600	HW2	SOL	3492	29.970	18.490	41.400	1.00	0.00
ATOM	11601	OW	SOL	3493	4.520	3.980	54.530	1.00	0.00
ATOM	11602	HW1	SOL	3493	3.950	3.290	54.880	1.00	0.00
ATOM	11603	HW2	SOL	3493	4.270	4.060	53.610	1.00	0.00
ATOM	11604	OW	SOL	3494	5.110	8.910	32.960	1.00	0.00
ATOM	11605	HW1	SOL	3494	5.660	8.170	32.710	1.00	0.00
ATOM	11606	HW2	SOL	3494	5.430	9.150	33.840	1.00	0.00
ATOM	11607	OW	SOL	3495	6.490	49.620	7.020	1.00	0.00
ATOM	11608	HW1	SOL	3495	7.360	49.220	7.050	1.00	0.00
ATOM	11609	HW2	SOL	3495	6.660	50.570	7.040	1.00	0.00
ATOM	11610	OW	SOL	3496	34.280	7.800	2.330	1.00	0.00
ATOM	11611	HW1	SOL	3496	33.540	7.760	2.940	1.00	0.00
ATOM	11612	HW2	SOL	3496	34.320	8.720	2.060	1.00	0.00
ATOM	11613	OW	SOL	3497	12.800	27.200	24.100	1.00	0.00
ATOM	11614	HW1	SOL	3497	13.730	27.410	24.190	1.00	0.00
ATOM	11615	HW2	SOL	3497	12.800	26.330	23.690	1.00	0.00

ATOM	11616	OW	SOL	3498	49.300	35.330	20.730	1.00	0.00
ATOM	11617	HW1	SOL	3498	48.500	35.730	20.380	1.00	0.00
ATOM	11618	HW2	SOL	3498	49.750	34.980	19.970	1.00	0.00
ATOM	11619	OW	SOL	3499	28.190	53.990	1.150	1.00	0.00
ATOM	11620	HW1	SOL	3499	27.690	54.020	1.970	1.00	0.00
ATOM	11621	HW2	SOL	3499	28.960	53.460	1.360	1.00	0.00
ATOM	11622	OW	SOL	3500	49.940	3.910	31.150	1.00	0.00
ATOM	11623	HW1	SOL	3500	49.840	3.000	31.420	1.00	0.00
ATOM	11624	HW2	SOL	3500	50.850	3.970	30.840	1.00	0.00
ATOM	11625	OW	SOL	3501	29.590	20.820	13.500	1.00	0.00
ATOM	11626	HW1	SOL	3501	30.140	21.240	14.160	1.00	0.00
ATOM	11627	HW2	SOL	3501	29.940	21.140	12.670	1.00	0.00
ATOM	11628	OW	SOL	3502	29.380	51.350	2.550	1.00	0.00
ATOM	11629	HW1	SOL	3502	28.500	51.070	2.820	1.00	0.00
ATOM	11630	HW2	SOL	3502	29.970	50.690	2.920	1.00	0.00
ATOM	11631	OW	SOL	3503	22.250	47.050	32.300	1.00	0.00
ATOM	11632	HW1	SOL	3503	21.790	46.450	32.880	1.00	0.00
ATOM	11633	HW2	SOL	3503	22.310	46.590	31.470	1.00	0.00
ATOM	11634	OW	SOL	3504	12.890	15.650	18.480	1.00	0.00
ATOM	11635	HW1	SOL	3504	12.870	14.780	18.080	1.00	0.00
ATOM	11636	HW2	SOL	3504	13.740	16.010	18.230	1.00	0.00
ATOM	11637	OW	SOL	3505	5.300	35.960	4.030	1.00	0.00
ATOM	11638	HW1	SOL	3505	5.740	35.620	4.810	1.00	0.00
ATOM	11639	HW2	SOL	3505	5.520	36.890	4.020	1.00	0.00
ATOM	11640	OW	SOL	3506	36.610	39.450	54.100	1.00	0.00
ATOM	11641	HW1	SOL	3506	36.030	39.650	54.840	1.00	0.00
ATOM	11642	HW2	SOL	3506	36.020	39.120	53.420	1.00	0.00
ATOM	11643	OW	SOL	3507	43.640	28.660	16.880	1.00	0.00
ATOM	11644	HW1	SOL	3507	43.870	28.150	17.660	1.00	0.00
ATOM	11645	HW2	SOL	3507	44.440	28.690	16.360	1.00	0.00
ATOM	11646	OW	SOL	3508	55.480	0.690	44.700	1.00	0.00
ATOM	11647	HW1	SOL	3508	55.390	-0.220	44.430	1.00	0.00
ATOM	11648	HW2	SOL	3508	56.160	1.050	44.120	1.00	0.00
ATOM	11649	OW	SOL	3509	11.440	37.940	26.140	1.00	0.00
ATOM	11650	HW1	SOL	3509	10.840	37.220	25.950	1.00	0.00
ATOM	11651	HW2	SOL	3509	10.920	38.730	26.000	1.00	0.00
ATOM	11652	OW	SOL	3510	33.060	8.000	53.840	1.00	0.00
ATOM	11653	HW1	SOL	3510	32.680	7.520	53.110	1.00	0.00
ATOM	11654	HW2	SOL	3510	33.280	8.860	53.470	1.00	0.00
ATOM	11655	OW	SOL	3511	2.250	4.230	30.300	1.00	0.00
ATOM	11656	HW1	SOL	3511	2.260	4.490	29.380	1.00	0.00
ATOM	11657	HW2	SOL	3511	1.960	3.310	30.290	1.00	0.00
ATOM	11658	OW	SOL	3512	15.370	33.950	27.910	1.00	0.00
ATOM	11659	HW1	SOL	3512	15.000	34.780	27.600	1.00	0.00

ATOM	11660	HW2	SOL	3512	15.810	34.170	28.730	1.00	0.00
ATOM	11661	OW	SOL	3513	44.940	46.940	10.410	1.00	0.00
ATOM	11662	HW1	SOL	3513	45.620	46.350	10.090	1.00	0.00
ATOM	11663	HW2	SOL	3513	44.460	47.200	9.630	1.00	0.00
ATOM	11664	OW	SOL	3514	11.980	40.990	38.770	1.00	0.00
ATOM	11665	HW1	SOL	3514	12.560	41.050	39.530	1.00	0.00
ATOM	11666	HW2	SOL	3514	11.130	40.730	39.130	1.00	0.00
ATOM	11667	OW	SOL	3515	18.850	40.640	19.170	1.00	0.00
ATOM	11668	HW1	SOL	3515	18.760	40.590	20.120	1.00	0.00
ATOM	11669	HW2	SOL	3515	18.340	41.400	18.910	1.00	0.00
ATOM	11670	OW	SOL	3516	52.450	8.510	51.270	1.00	0.00
ATOM	11671	HW1	SOL	3516	51.930	9.170	50.810	1.00	0.00
ATOM	11672	HW2	SOL	3516	51.980	7.690	51.120	1.00	0.00
ATOM	11673	OW	SOL	3517	8.090	6.060	13.990	1.00	0.00
ATOM	11674	HW1	SOL	3517	8.010	6.900	13.550	1.00	0.00
ATOM	11675	HW2	SOL	3517	9.040	5.940	14.080	1.00	0.00
ATOM	11676	OW	SOL	3518	28.570	25.540	42.500	1.00	0.00
ATOM	11677	HW1	SOL	3518	29.080	25.900	43.220	1.00	0.00
ATOM	11678	HW2	SOL	3518	27.890	25.020	42.920	1.00	0.00
ATOM	11679	OW	SOL	3519	10.300	13.250	26.990	1.00	0.00
ATOM	11680	HW1	SOL	3519	10.410	13.940	26.330	1.00	0.00
ATOM	11681	HW2	SOL	3519	9.720	13.640	27.650	1.00	0.00
ATOM	11682	OW	SOL	3520	0.710	8.750	13.900	1.00	0.00
ATOM	11683	HW1	SOL	3520	1.210	9.160	13.200	1.00	0.00
ATOM	11684	HW2	SOL	3520	0.530	9.460	14.520	1.00	0.00
ATOM	11685	OW	SOL	3521	10.510	1.390	17.420	1.00	0.00
ATOM	11686	HW1	SOL	3521	10.760	1.220	16.510	1.00	0.00
ATOM	11687	HW2	SOL	3521	9.570	1.580	17.380	1.00	0.00
ATOM	11688	OW	SOL	3522	23.090	42.490	49.020	1.00	0.00
ATOM	11689	HW1	SOL	3522	23.610	41.680	49.070	1.00	0.00
ATOM	11690	HW2	SOL	3522	23.270	42.940	49.840	1.00	0.00
ATOM	11691	OW	SOL	3523	36.620	51.630	32.930	1.00	0.00
ATOM	11692	HW1	SOL	3523	36.240	51.760	33.800	1.00	0.00
ATOM	11693	HW2	SOL	3523	36.550	50.690	32.780	1.00	0.00
ATOM	11694	OW	SOL	3524	15.210	13.610	36.580	1.00	0.00
ATOM	11695	HW1	SOL	3524	14.830	12.830	36.980	1.00	0.00
ATOM	11696	HW2	SOL	3524	15.170	13.450	35.640	1.00	0.00
ATOM	11697	OW	SOL	3525	37.510	10.480	7.890	1.00	0.00
ATOM	11698	HW1	SOL	3525	37.870	10.370	7.010	1.00	0.00
ATOM	11699	HW2	SOL	3525	38.280	10.470	8.460	1.00	0.00
ATOM	11700	OW	SOL	3526	52.370	44.060	51.390	1.00	0.00
ATOM	11701	HW1	SOL	3526	52.730	44.670	52.030	1.00	0.00
ATOM	11702	HW2	SOL	3526	52.350	44.560	50.570	1.00	0.00
ATOM	11703	OW	SOL	3527	30.890	38.940	14.680	1.00	0.00

ATOM	11704	HW1	SOL	3527	30.890	38.890	13.720	1.00	0.00
ATOM	11705	HW2	SOL	3527	30.170	38.370	14.950	1.00	0.00
ATOM	11706	OW	SOL	3528	33.650	31.590	44.870	1.00	0.00
ATOM	11707	HW1	SOL	3528	33.190	32.420	45.010	1.00	0.00
ATOM	11708	HW2	SOL	3528	34.570	31.790	45.050	1.00	0.00
ATOM	11709	OW	SOL	3529	15.330	6.950	41.500	1.00	0.00
ATOM	11710	HW1	SOL	3529	16.050	6.530	41.970	1.00	0.00
ATOM	11711	HW2	SOL	3529	14.960	6.260	40.960	1.00	0.00
ATOM	11712	OW	SOL	3530	37.680	5.870	17.320	1.00	0.00
ATOM	11713	HW1	SOL	3530	38.440	5.590	17.830	1.00	0.00
ATOM	11714	HW2	SOL	3530	37.980	6.630	16.830	1.00	0.00
ATOM	11715	OW	SOL	3531	24.830	27.880	26.620	1.00	0.00
ATOM	11716	HW1	SOL	3531	25.520	28.190	26.040	1.00	0.00
ATOM	11717	HW2	SOL	3531	24.440	27.140	26.150	1.00	0.00
ATOM	11718	OW	SOL	3532	37.440	25.430	18.210	1.00	0.00
ATOM	11719	HW1	SOL	3532	36.670	25.100	17.760	1.00	0.00
ATOM	11720	HW2	SOL	3532	37.200	25.450	19.140	1.00	0.00
ATOM	11721	OW	SOL	3533	55.370	50.090	4.140	1.00	0.00
ATOM	11722	HW1	SOL	3533	55.880	50.810	4.500	1.00	0.00
ATOM	11723	HW2	SOL	3533	55.960	49.340	4.180	1.00	0.00
ATOM	11724	OW	SOL	3534	36.780	31.130	4.400	1.00	0.00
ATOM	11725	HW1	SOL	3534	37.080	30.310	4.790	1.00	0.00
ATOM	11726	HW2	SOL	3534	37.490	31.750	4.590	1.00	0.00
ATOM	11727	OW	SOL	3535	36.190	20.560	44.720	1.00	0.00
ATOM	11728	HW1	SOL	3535	36.540	19.810	45.210	1.00	0.00
ATOM	11729	HW2	SOL	3535	35.680	20.150	44.020	1.00	0.00
ATOM	11730	OW	SOL	3536	49.930	22.280	52.530	1.00	0.00
ATOM	11731	HW1	SOL	3536	48.980	22.300	52.610	1.00	0.00
ATOM	11732	HW2	SOL	3536	50.250	22.330	53.440	1.00	0.00
ATOM	11733	OW	SOL	3537	11.600	18.600	42.420	1.00	0.00
ATOM	11734	HW1	SOL	3537	12.540	18.800	42.360	1.00	0.00
ATOM	11735	HW2	SOL	3537	11.340	18.430	41.510	1.00	0.00
ATOM	11736	OW	SOL	3538	15.300	8.750	2.310	1.00	0.00
ATOM	11737	HW1	SOL	3538	14.500	8.290	2.550	1.00	0.00
ATOM	11738	HW2	SOL	3538	16.000	8.300	2.780	1.00	0.00
ATOM	11739	OW	SOL	3539	34.730	12.230	50.700	1.00	0.00
ATOM	11740	HW1	SOL	3539	35.080	12.010	49.830	1.00	0.00
ATOM	11741	HW2	SOL	3539	34.550	13.160	50.650	1.00	0.00
ATOM	11742	OW	SOL	3540	16.330	3.930	17.420	1.00	0.00
ATOM	11743	HW1	SOL	3540	17.130	3.410	17.430	1.00	0.00
ATOM	11744	HW2	SOL	3540	16.480	4.610	16.760	1.00	0.00
ATOM	11745	OW	SOL	3541	27.030	50.450	3.230	1.00	0.00
ATOM	11746	HW1	SOL	3541	26.920	49.540	3.490	1.00	0.00
ATOM	11747	HW2	SOL	3541	27.130	50.930	4.050	1.00	0.00

ATOM	11748	OW	SOL	3542	3.630	21.620	47.720	1.00	0.00
ATOM	11749	HW1	SOL	3542	3.620	20.870	47.120	1.00	0.00
ATOM	11750	HW2	SOL	3542	3.090	22.280	47.290	1.00	0.00
ATOM	11751	OW	SOL	3543	12.440	6.040	46.530	1.00	0.00
ATOM	11752	HW1	SOL	3543	13.250	6.310	46.960	1.00	0.00
ATOM	11753	HW2	SOL	3543	12.700	5.850	45.630	1.00	0.00
ATOM	11754	OW	SOL	3544	14.270	7.050	37.370	1.00	0.00
ATOM	11755	HW1	SOL	3544	14.900	7.720	37.640	1.00	0.00
ATOM	11756	HW2	SOL	3544	14.010	7.310	36.490	1.00	0.00
ATOM	11757	OW	SOL	3545	35.970	8.070	4.650	1.00	0.00
ATOM	11758	HW1	SOL	3545	35.750	8.150	3.720	1.00	0.00
ATOM	11759	HW2	SOL	3545	35.130	8.170	5.100	1.00	0.00
ATOM	11760	OW	SOL	3546	22.570	10.160	16.290	1.00	0.00
ATOM	11761	HW1	SOL	3546	23.500	10.370	16.180	1.00	0.00
ATOM	11762	HW2	SOL	3546	22.290	9.830	15.440	1.00	0.00
ATOM	11763	OW	SOL	3547	28.810	19.060	0.690	1.00	0.00
ATOM	11764	HW1	SOL	3547	28.910	19.500	1.540	1.00	0.00
ATOM	11765	HW2	SOL	3547	27.890	18.810	0.660	1.00	0.00
ATOM	11766	OW	SOL	3548	24.510	1.230	9.080	1.00	0.00
ATOM	11767	HW1	SOL	3548	24.470	0.280	9.120	1.00	0.00
ATOM	11768	HW2	SOL	3548	23.750	1.530	9.580	1.00	0.00
ATOM	11769	OW	SOL	3549	5.830	23.960	22.350	1.00	0.00
ATOM	11770	HW1	SOL	3549	5.020	24.110	22.860	1.00	0.00
ATOM	11771	HW2	SOL	3549	6.480	24.520	22.780	1.00	0.00
ATOM	11772	OW	SOL	3550	43.730	16.880	5.770	1.00	0.00
ATOM	11773	HW1	SOL	3550	44.490	16.410	6.120	1.00	0.00
ATOM	11774	HW2	SOL	3550	43.060	16.210	5.670	1.00	0.00
ATOM	11775	OW	SOL	3551	50.060	45.880	18.840	1.00	0.00
ATOM	11776	HW1	SOL	3551	50.360	45.100	19.330	1.00	0.00
ATOM	11777	HW2	SOL	3551	49.130	45.940	19.040	1.00	0.00
ATOM	11778	OW	SOL	3552	6.190	10.830	52.880	1.00	0.00
ATOM	11779	HW1	SOL	3552	5.790	10.880	52.010	1.00	0.00
ATOM	11780	HW2	SOL	3552	5.460	10.930	53.480	1.00	0.00
ATOM	11781	OW	SOL	3553	53.140	39.310	7.920	1.00	0.00
ATOM	11782	HW1	SOL	3553	52.860	40.230	7.920	1.00	0.00
ATOM	11783	HW2	SOL	3553	54.050	39.340	8.240	1.00	0.00
ATOM	11784	OW	SOL	3554	14.540	53.490	21.220	1.00	0.00
ATOM	11785	HW1	SOL	3554	14.270	52.630	20.890	1.00	0.00
ATOM	11786	HW2	SOL	3554	15.480	53.540	21.010	1.00	0.00
ATOM	11787	OW	SOL	3555	42.620	3.770	9.780	1.00	0.00
ATOM	11788	HW1	SOL	3555	42.870	3.440	8.920	1.00	0.00
ATOM	11789	HW2	SOL	3555	43.110	3.230	10.400	1.00	0.00
ATOM	11790	OW	SOL	3556	18.410	1.840	5.850	1.00	0.00
ATOM	11791	HW1	SOL	3556	17.820	1.730	6.600	1.00	0.00

ATOM	11792	HW2	SOL	3556	17.820	1.930	5.100	1.00	0.00
ATOM	11793	OW	SOL	3557	8.200	40.980	3.660	1.00	0.00
ATOM	11794	HW1	SOL	3557	8.670	40.150	3.670	1.00	0.00
ATOM	11795	HW2	SOL	3557	8.850	41.620	3.950	1.00	0.00
ATOM	11796	OW	SOL	3558	29.450	38.970	49.550	1.00	0.00
ATOM	11797	HW1	SOL	3558	29.820	38.710	50.390	1.00	0.00
ATOM	11798	HW2	SOL	3558	30.180	39.410	49.100	1.00	0.00
ATOM	11799	OW	SOL	3559	8.320	15.920	22.720	1.00	0.00
ATOM	11800	HW1	SOL	3559	8.530	15.660	21.820	1.00	0.00
ATOM	11801	HW2	SOL	3559	7.860	16.760	22.630	1.00	0.00
ATOM	11802	OW	SOL	3560	2.350	52.050	52.670	1.00	0.00
ATOM	11803	HW1	SOL	3560	3.170	51.680	52.990	1.00	0.00
ATOM	11804	HW2	SOL	3560	1.720	51.860	53.370	1.00	0.00
ATOM	11805	OW	SOL	3561	49.760	31.360	1.580	1.00	0.00
ATOM	11806	HW1	SOL	3561	49.720	30.940	2.450	1.00	0.00
ATOM	11807	HW2	SOL	3561	50.560	31.020	1.190	1.00	0.00
ATOM	11808	OW	SOL	3562	0.940	15.930	45.610	1.00	0.00
ATOM	11809	HW1	SOL	3562	0.770	16.800	45.980	1.00	0.00
ATOM	11810	HW2	SOL	3562	1.510	16.100	44.860	1.00	0.00
ATOM	11811	OW	SOL	3563	10.990	47.550	19.180	1.00	0.00
ATOM	11812	HW1	SOL	3563	10.410	47.470	18.420	1.00	0.00
ATOM	11813	HW2	SOL	3563	10.500	48.100	19.800	1.00	0.00
ATOM	11814	OW	SOL	3564	36.770	14.570	38.290	1.00	0.00
ATOM	11815	HW1	SOL	3564	37.070	15.340	37.820	1.00	0.00
ATOM	11816	HW2	SOL	3564	35.840	14.500	38.080	1.00	0.00
ATOM	11817	OW	SOL	3565	55.330	52.270	26.110	1.00	0.00
ATOM	11818	HW1	SOL	3565	54.440	52.230	26.470	1.00	0.00
ATOM	11819	HW2	SOL	3565	55.860	52.620	26.830	1.00	0.00
ATOM	11820	OW	SOL	3566	42.600	20.350	51.460	1.00	0.00
ATOM	11821	HW1	SOL	3566	42.110	19.610	51.820	1.00	0.00
ATOM	11822	HW2	SOL	3566	42.180	21.120	51.830	1.00	0.00
ATOM	11823	OW	SOL	3567	24.760	47.760	36.400	1.00	0.00
ATOM	11824	HW1	SOL	3567	25.350	47.310	35.800	1.00	0.00
ATOM	11825	HW2	SOL	3567	24.060	48.100	35.850	1.00	0.00
ATOM	11826	OW	SOL	3568	0.590	53.900	2.430	1.00	0.00
ATOM	11827	HW1	SOL	3568	-0.040	54.340	3.000	1.00	0.00
ATOM	11828	HW2	SOL	3568	0.800	53.090	2.900	1.00	0.00
ATOM	11829	OW	SOL	3569	3.930	45.370	32.930	1.00	0.00
ATOM	11830	HW1	SOL	3569	3.710	45.280	33.860	1.00	0.00
ATOM	11831	HW2	SOL	3569	3.200	44.950	32.470	1.00	0.00
ATOM	11832	OW	SOL	3570	11.190	50.390	45.000	1.00	0.00
ATOM	11833	HW1	SOL	3570	10.780	50.040	44.210	1.00	0.00
ATOM	11834	HW2	SOL	3570	11.520	51.250	44.740	1.00	0.00
ATOM	11835	OW	SOL	3571	15.370	45.200	47.380	1.00	0.00

ATOM	11836	HW1	SOL	3571	15.510	46.140	47.350	1.00	0.00
ATOM	11837	HW2	SOL	3571	16.240	44.820	47.330	1.00	0.00
ATOM	11838	OW	SOL	3572	38.150	19.410	13.890	1.00	0.00
ATOM	11839	HW1	SOL	3572	38.720	18.740	13.520	1.00	0.00
ATOM	11840	HW2	SOL	3572	37.710	19.800	13.130	1.00	0.00
ATOM	11841	OW	SOL	3573	20.630	45.290	1.580	1.00	0.00
ATOM	11842	HW1	SOL	3573	21.080	45.600	0.800	1.00	0.00
ATOM	11843	HW2	SOL	3573	20.830	45.960	2.250	1.00	0.00
ATOM	11844	OW	SOL	3574	6.560	12.900	43.370	1.00	0.00
ATOM	11845	HW1	SOL	3574	6.190	12.050	43.160	1.00	0.00
ATOM	11846	HW2	SOL	3574	6.810	13.270	42.530	1.00	0.00
ATOM	11847	OW	SOL	3575	2.810	20.260	13.120	1.00	0.00
ATOM	11848	HW1	SOL	3575	2.440	19.440	12.790	1.00	0.00
ATOM	11849	HW2	SOL	3575	3.710	20.050	13.360	1.00	0.00
ATOM	11850	OW	SOL	3576	27.080	42.840	21.690	1.00	0.00
ATOM	11851	HW1	SOL	3576	26.840	41.940	21.460	1.00	0.00
ATOM	11852	HW2	SOL	3576	26.400	43.380	21.290	1.00	0.00
ATOM	11853	OW	SOL	3577	6.100	19.740	6.920	1.00	0.00
ATOM	11854	HW1	SOL	3577	6.270	19.110	6.220	1.00	0.00
ATOM	11855	HW2	SOL	3577	6.960	19.920	7.290	1.00	0.00
ATOM	11856	OW	SOL	3578	42.760	41.450	49.320	1.00	0.00
ATOM	11857	HW1	SOL	3578	42.010	41.390	49.920	1.00	0.00
ATOM	11858	HW2	SOL	3578	42.450	42.010	48.610	1.00	0.00
ATOM	11859	OW	SOL	3579	4.770	39.590	33.810	1.00	0.00
ATOM	11860	HW1	SOL	3579	3.920	39.980	34.030	1.00	0.00
ATOM	11861	HW2	SOL	3579	5.050	40.050	33.020	1.00	0.00
ATOM	11862	OW	SOL	3580	51.470	4.220	39.880	1.00	0.00
ATOM	11863	HW1	SOL	3580	51.910	3.450	40.250	1.00	0.00
ATOM	11864	HW2	SOL	3580	50.920	3.870	39.180	1.00	0.00
ATOM	11865	OW	SOL	3581	45.090	43.870	13.120	1.00	0.00
ATOM	11866	HW1	SOL	3581	45.720	43.920	13.850	1.00	0.00
ATOM	11867	HW2	SOL	3581	44.540	43.120	13.330	1.00	0.00
ATOM	11868	OW	SOL	3582	9.830	21.430	35.310	1.00	0.00
ATOM	11869	HW1	SOL	3582	9.370	22.270	35.330	1.00	0.00
ATOM	11870	HW2	SOL	3582	10.750	21.660	35.210	1.00	0.00
ATOM	11871	OW	SOL	3583	9.950	41.210	36.460	1.00	0.00
ATOM	11872	HW1	SOL	3583	10.780	41.530	36.810	1.00	0.00
ATOM	11873	HW2	SOL	3583	9.410	41.070	37.240	1.00	0.00
ATOM	11874	OW	SOL	3584	43.850	11.140	34.050	1.00	0.00
ATOM	11875	HW1	SOL	3584	44.260	11.150	34.910	1.00	0.00
ATOM	11876	HW2	SOL	3584	43.090	11.720	34.140	1.00	0.00
ATOM	11877	OW	SOL	3585	28.840	12.330	0.040	1.00	0.00
ATOM	11878	HW1	SOL	3585	28.800	11.370	0.030	1.00	0.00
ATOM	11879	HW2	SOL	3585	29.630	12.540	-0.450	1.00	0.00

ATOM	11880	OW	SOL	3586	35.910	9.680	47.150	1.00	0.00
ATOM	11881	HW1	SOL	3586	35.220	9.540	46.500	1.00	0.00
ATOM	11882	HW2	SOL	3586	36.270	10.540	46.930	1.00	0.00
ATOM	11883	OW	SOL	3587	23.210	20.050	38.250	1.00	0.00
ATOM	11884	HW1	SOL	3587	23.870	19.430	37.930	1.00	0.00
ATOM	11885	HW2	SOL	3587	22.380	19.600	38.100	1.00	0.00
ATOM	11886	OW	SOL	3588	27.360	0.960	8.550	1.00	0.00
ATOM	11887	HW1	SOL	3588	26.540	1.370	8.270	1.00	0.00
ATOM	11888	HW2	SOL	3588	27.150	0.030	8.620	1.00	0.00
ATOM	11889	OW	SOL	3589	4.940	37.180	18.520	1.00	0.00
ATOM	11890	HW1	SOL	3589	4.080	37.430	18.850	1.00	0.00
ATOM	11891	HW2	SOL	3589	5.320	36.650	19.230	1.00	0.00
ATOM	11892	OW	SOL	3590	30.880	3.860	4.160	1.00	0.00
ATOM	11893	HW1	SOL	3590	31.660	3.310	4.150	1.00	0.00
ATOM	11894	HW2	SOL	3590	31.130	4.660	3.700	1.00	0.00
ATOM	11895	OW	SOL	3591	9.880	13.990	46.570	1.00	0.00
ATOM	11896	HW1	SOL	3591	9.650	13.450	45.810	1.00	0.00
ATOM	11897	HW2	SOL	3591	9.340	14.770	46.480	1.00	0.00
ATOM	11898	OW	SOL	3592	10.570	51.050	29.260	1.00	0.00
ATOM	11899	HW1	SOL	3592	11.520	51.000	29.200	1.00	0.00
ATOM	11900	HW2	SOL	3592	10.400	51.630	30.000	1.00	0.00
ATOM	11901	OW	SOL	3593	53.890	26.730	2.840	1.00	0.00
ATOM	11902	HW1	SOL	3593	53.580	27.350	3.490	1.00	0.00
ATOM	11903	HW2	SOL	3593	54.330	27.270	2.180	1.00	0.00
ATOM	11904	OW	SOL	3594	55.640	7.040	23.120	1.00	0.00
ATOM	11905	HW1	SOL	3594	56.380	6.490	22.840	1.00	0.00
ATOM	11906	HW2	SOL	3594	54.900	6.430	23.170	1.00	0.00
ATOM	11907	OW	SOL	3595	50.120	2.540	47.580	1.00	0.00
ATOM	11908	HW1	SOL	3595	50.310	1.870	46.920	1.00	0.00
ATOM	11909	HW2	SOL	3595	49.250	2.310	47.910	1.00	0.00
ATOM	11910	OW	SOL	3596	6.120	23.430	52.340	1.00	0.00
ATOM	11911	HW1	SOL	3596	5.530	23.400	51.590	1.00	0.00
ATOM	11912	HW2	SOL	3596	6.790	22.780	52.160	1.00	0.00
ATOM	11913	OW	SOL	3597	10.540	38.360	49.350	1.00	0.00
ATOM	11914	HW1	SOL	3597	10.800	39.280	49.400	1.00	0.00
ATOM	11915	HW2	SOL	3597	11.370	37.880	49.270	1.00	0.00
ATOM	11916	OW	SOL	3598	20.510	5.370	34.650	1.00	0.00
ATOM	11917	HW1	SOL	3598	20.880	6.090	35.150	1.00	0.00
ATOM	11918	HW2	SOL	3598	21.260	4.890	34.310	1.00	0.00
ATOM	11919	OW	SOL	3599	41.410	50.360	46.050	1.00	0.00
ATOM	11920	HW1	SOL	3599	41.180	51.180	45.610	1.00	0.00
ATOM	11921	HW2	SOL	3599	42.040	49.950	45.460	1.00	0.00
ATOM	11922	OW	SOL	3600	5.220	1.560	55.800	1.00	0.00
ATOM	11923	HW1	SOL	3600	4.630	1.840	56.490	1.00	0.00

ATOM	11924	HW2	SOL	3600	4.800	0.790	55.420	1.00	0.00
ATOM	11925	OW	SOL	3601	30.220	45.380	38.180	1.00	0.00
ATOM	11926	HW1	SOL	3601	30.570	45.390	39.070	1.00	0.00
ATOM	11927	HW2	SOL	3601	30.720	44.700	37.730	1.00	0.00
ATOM	11928	OW	SOL	3602	29.340	35.890	22.950	1.00	0.00
ATOM	11929	HW1	SOL	3602	28.630	35.990	22.310	1.00	0.00
ATOM	11930	HW2	SOL	3602	30.130	35.780	22.430	1.00	0.00
ATOM	11931	OW	SOL	3603	0.930	38.240	6.250	1.00	0.00
ATOM	11932	HW1	SOL	3603	0.350	39.000	6.330	1.00	0.00
ATOM	11933	HW2	SOL	3603	1.040	38.130	5.310	1.00	0.00
ATOM	11934	OW	SOL	3604	20.910	18.300	38.550	1.00	0.00
ATOM	11935	HW1	SOL	3604	21.220	18.550	39.420	1.00	0.00
ATOM	11936	HW2	SOL	3604	20.260	18.970	38.330	1.00	0.00
ATOM	11937	OW	SOL	3605	49.330	4.390	28.430	1.00	0.00
ATOM	11938	HW1	SOL	3605	50.120	4.250	27.900	1.00	0.00
ATOM	11939	HW2	SOL	3605	49.650	4.370	29.330	1.00	0.00
ATOM	11940	OW	SOL	3606	21.260	21.020	1.360	1.00	0.00
ATOM	11941	HW1	SOL	3606	21.240	21.380	0.470	1.00	0.00
ATOM	11942	HW2	SOL	3606	20.930	20.130	1.260	1.00	0.00
ATOM	11943	OW	SOL	3607	1.970	11.840	43.690	1.00	0.00
ATOM	11944	HW1	SOL	3607	2.930	11.870	43.730	1.00	0.00
ATOM	11945	HW2	SOL	3607	1.750	12.550	43.080	1.00	0.00
ATOM	11946	OW	SOL	3608	15.980	41.850	17.600	1.00	0.00
ATOM	11947	HW1	SOL	3608	15.270	42.340	17.200	1.00	0.00
ATOM	11948	HW2	SOL	3608	15.720	41.740	18.510	1.00	0.00
ATOM	11949	OW	SOL	3609	13.760	32.920	37.250	1.00	0.00
ATOM	11950	HW1	SOL	3609	13.400	33.120	36.380	1.00	0.00
ATOM	11951	HW2	SOL	3609	14.370	33.630	37.430	1.00	0.00
ATOM	11952	OW	SOL	3610	34.000	23.790	0.530	1.00	0.00
ATOM	11953	HW1	SOL	3610	33.830	24.520	-0.070	1.00	0.00
ATOM	11954	HW2	SOL	3610	34.550	23.190	0.040	1.00	0.00
ATOM	11955	OW	SOL	3611	12.500	46.640	3.290	1.00	0.00
ATOM	11956	HW1	SOL	3611	13.030	45.860	3.430	1.00	0.00
ATOM	11957	HW2	SOL	3611	13.110	47.280	2.920	1.00	0.00
ATOM	11958	OW	SOL	3612	39.610	25.650	1.020	1.00	0.00
ATOM	11959	HW1	SOL	3612	40.290	26.100	1.530	1.00	0.00
ATOM	11960	HW2	SOL	3612	39.930	24.750	0.930	1.00	0.00
ATOM	11961	OW	SOL	3613	40.570	37.540	9.810	1.00	0.00
ATOM	11962	HW1	SOL	3613	39.690	37.510	9.440	1.00	0.00
ATOM	11963	HW2	SOL	3613	40.680	36.690	10.250	1.00	0.00
ATOM	11964	OW	SOL	3614	17.780	23.530	37.420	1.00	0.00
ATOM	11965	HW1	SOL	3614	18.730	23.620	37.510	1.00	0.00
ATOM	11966	HW2	SOL	3614	17.560	22.840	38.050	1.00	0.00
ATOM	11967	OW	SOL	3615	52.500	16.080	19.870	1.00	0.00

ATOM	11968	HW1	SOL	3615	53.010	16.600	20.490	1.00	0.00
ATOM	11969	HW2	SOL	3615	52.170	15.350	20.390	1.00	0.00
ATOM	11970	OW	SOL	3616	54.470	14.060	5.080	1.00	0.00
ATOM	11971	HW1	SOL	3616	54.560	15.020	5.050	1.00	0.00
ATOM	11972	HW2	SOL	3616	54.770	13.770	4.220	1.00	0.00
ATOM	11973	OW	SOL	3617	29.720	51.020	46.600	1.00	0.00
ATOM	11974	HW1	SOL	3617	30.580	50.640	46.440	1.00	0.00
ATOM	11975	HW2	SOL	3617	29.900	51.790	47.140	1.00	0.00
ATOM	11976	OW	SOL	3618	47.090	8.550	43.720	1.00	0.00
ATOM	11977	HW1	SOL	3618	47.860	7.990	43.790	1.00	0.00
ATOM	11978	HW2	SOL	3618	47.040	8.790	42.800	1.00	0.00
ATOM	11979	OW	SOL	3619	7.510	12.380	5.500	1.00	0.00
ATOM	11980	HW1	SOL	3619	7.290	13.230	5.900	1.00	0.00
ATOM	11981	HW2	SOL	3619	6.950	11.750	5.960	1.00	0.00
ATOM	11982	OW	SOL	3620	3.200	21.450	43.040	1.00	0.00
ATOM	11983	HW1	SOL	3620	2.350	21.460	43.490	1.00	0.00
ATOM	11984	HW2	SOL	3620	3.090	22.060	42.310	1.00	0.00
ATOM	11985	OW	SOL	3621	36.390	53.870	45.240	1.00	0.00
ATOM	11986	HW1	SOL	3621	35.690	53.680	45.860	1.00	0.00
ATOM	11987	HW2	SOL	3621	36.060	54.610	44.720	1.00	0.00
ATOM	11988	OW	SOL	3622	36.820	55.040	33.040	1.00	0.00
ATOM	11989	HW1	SOL	3622	37.580	55.550	32.760	1.00	0.00
ATOM	11990	HW2	SOL	3622	36.690	55.290	33.960	1.00	0.00
ATOM	11991	OW	SOL	3623	24.000	10.160	13.000	1.00	0.00
ATOM	11992	HW1	SOL	3623	23.670	9.270	12.880	1.00	0.00
ATOM	11993	HW2	SOL	3623	24.940	10.050	13.140	1.00	0.00
ATOM	11994	OW	SOL	3624	8.990	27.690	22.620	1.00	0.00
ATOM	11995	HW1	SOL	3624	8.740	26.970	23.190	1.00	0.00
ATOM	11996	HW2	SOL	3624	8.380	28.390	22.840	1.00	0.00
ATOM	11997	OW	SOL	3625	0.170	15.630	30.270	1.00	0.00
ATOM	11998	HW1	SOL	3625	0.200	15.220	29.410	1.00	0.00
ATOM	11999	HW2	SOL	3625	-0.300	15.000	30.820	1.00	0.00
ATOM	12000	OW	SOL	3626	52.900	49.540	25.190	1.00	0.00
ATOM	12001	HW1	SOL	3626	52.830	49.430	24.240	1.00	0.00
ATOM	12002	HW2	SOL	3626	53.840	49.560	25.360	1.00	0.00
ATOM	12003	OW	SOL	3627	39.060	0.370	27.700	1.00	0.00
ATOM	12004	HW1	SOL	3627	38.380	0.410	28.360	1.00	0.00
ATOM	12005	HW2	SOL	3627	39.520	-0.450	27.860	1.00	0.00
ATOM	12006	OW	SOL	3628	23.080	13.350	26.580	1.00	0.00
ATOM	12007	HW1	SOL	3628	22.220	13.150	26.950	1.00	0.00
ATOM	12008	HW2	SOL	3628	22.900	13.640	25.690	1.00	0.00
ATOM	12009	OW	SOL	3629	37.670	1.810	49.450	1.00	0.00
ATOM	12010	HW1	SOL	3629	36.720	1.870	49.570	1.00	0.00
ATOM	12011	HW2	SOL	3629	38.000	2.660	49.750	1.00	0.00

ATOM	12012	OW	SOL	3630	52.420	41.800	36.920	1.00	0.00
ATOM	12013	HW1	SOL	3630	52.460	41.270	36.120	1.00	0.00
ATOM	12014	HW2	SOL	3630	53.250	42.280	36.930	1.00	0.00
ATOM	12015	OW	SOL	3631	15.530	16.450	14.420	1.00	0.00
ATOM	12016	HW1	SOL	3631	15.360	17.100	13.750	1.00	0.00
ATOM	12017	HW2	SOL	3631	14.710	15.950	14.480	1.00	0.00
ATOM	12018	OW	SOL	3632	31.270	7.990	26.690	1.00	0.00
ATOM	12019	HW1	SOL	3632	30.690	7.960	25.930	1.00	0.00
ATOM	12020	HW2	SOL	3632	31.360	8.920	26.890	1.00	0.00
ATOM	12021	OW	SOL	3633	40.120	37.100	3.750	1.00	0.00
ATOM	12022	HW1	SOL	3633	39.250	37.500	3.680	1.00	0.00
ATOM	12023	HW2	SOL	3633	40.730	37.810	3.550	1.00	0.00
ATOM	12024	OW	SOL	3634	28.730	2.440	31.440	1.00	0.00
ATOM	12025	HW1	SOL	3634	28.690	3.390	31.560	1.00	0.00
ATOM	12026	HW2	SOL	3634	28.730	2.320	30.490	1.00	0.00
ATOM	12027	OW	SOL	3635	34.570	26.550	35.400	1.00	0.00
ATOM	12028	HW1	SOL	3635	35.030	26.990	36.110	1.00	0.00
ATOM	12029	HW2	SOL	3635	33.660	26.510	35.700	1.00	0.00
ATOM	12030	OW	SOL	3636	42.340	21.220	46.110	1.00	0.00
ATOM	12031	HW1	SOL	3636	42.370	21.270	47.070	1.00	0.00
ATOM	12032	HW2	SOL	3636	43.200	20.870	45.870	1.00	0.00
ATOM	12033	OW	SOL	3637	50.190	33.850	53.610	1.00	0.00
ATOM	12034	HW1	SOL	3637	49.750	33.700	52.770	1.00	0.00
ATOM	12035	HW2	SOL	3637	50.180	32.990	54.030	1.00	0.00
ATOM	12036	OW	SOL	3638	2.800	49.500	15.210	1.00	0.00
ATOM	12037	HW1	SOL	3638	2.890	48.900	15.960	1.00	0.00
ATOM	12038	HW2	SOL	3638	1.860	49.520	15.040	1.00	0.00
ATOM	12039	OW	SOL	3639	8.290	42.920	51.330	1.00	0.00
ATOM	12040	HW1	SOL	3639	8.740	42.700	50.520	1.00	0.00
ATOM	12041	HW2	SOL	3639	8.610	43.790	51.570	1.00	0.00
ATOM	12042	OW	SOL	3640	32.260	22.010	34.980	1.00	0.00
ATOM	12043	HW1	SOL	3640	32.390	22.100	35.930	1.00	0.00
ATOM	12044	HW2	SOL	3640	32.300	22.910	34.660	1.00	0.00
ATOM	12045	OW	SOL	3641	24.070	29.730	15.260	1.00	0.00
ATOM	12046	HW1	SOL	3641	23.540	30.390	14.820	1.00	0.00
ATOM	12047	HW2	SOL	3641	23.560	28.930	15.200	1.00	0.00
ATOM	12048	OW	SOL	3642	2.170	51.650	30.860	1.00	0.00
ATOM	12049	HW1	SOL	3642	1.780	51.440	31.700	1.00	0.00
ATOM	12050	HW2	SOL	3642	1.560	51.300	30.210	1.00	0.00
ATOM	12051	OW	SOL	3643	45.640	15.900	12.340	1.00	0.00
ATOM	12052	HW1	SOL	3643	45.330	15.370	13.070	1.00	0.00
ATOM	12053	HW2	SOL	3643	45.510	16.800	12.620	1.00	0.00
ATOM	12054	OW	SOL	3644	32.960	1.750	3.320	1.00	0.00
ATOM	12055	HW1	SOL	3644	33.420	2.350	2.740	1.00	0.00

ATOM	12056	HW2	SOL	3644	33.420	1.820	4.160	1.00	0.00
ATOM	12057	OW	SOL	3645	17.780	20.810	41.870	1.00	0.00
ATOM	12058	HW1	SOL	3645	18.660	20.650	42.200	1.00	0.00
ATOM	12059	HW2	SOL	3645	17.680	20.200	41.140	1.00	0.00
ATOM	12060	OW	SOL	3646	34.160	15.500	40.650	1.00	0.00
ATOM	12061	HW1	SOL	3646	33.230	15.340	40.800	1.00	0.00
ATOM	12062	HW2	SOL	3646	34.180	16.090	39.890	1.00	0.00
ATOM	12063	OW	SOL	3647	40.670	9.560	34.710	1.00	0.00
ATOM	12064	HW1	SOL	3647	40.770	10.150	33.960	1.00	0.00
ATOM	12065	HW2	SOL	3647	40.030	10.000	35.270	1.00	0.00
ATOM	12066	OW	SOL	3648	35.080	47.000	53.660	1.00	0.00
ATOM	12067	HW1	SOL	3648	35.930	47.440	53.690	1.00	0.00
ATOM	12068	HW2	SOL	3648	34.970	46.740	52.740	1.00	0.00
ATOM	12069	OW	SOL	3649	45.610	27.170	9.030	1.00	0.00
ATOM	12070	HW1	SOL	3649	45.510	26.870	9.930	1.00	0.00
ATOM	12071	HW2	SOL	3649	45.950	26.400	8.560	1.00	0.00
ATOM	12072	OW	SOL	3650	19.300	37.580	16.750	1.00	0.00
ATOM	12073	HW1	SOL	3650	20.150	38.010	16.800	1.00	0.00
ATOM	12074	HW2	SOL	3650	18.670	38.290	16.670	1.00	0.00
ATOM	12075	OW	SOL	3651	31.270	39.590	44.790	1.00	0.00
ATOM	12076	HW1	SOL	3651	31.600	38.980	44.130	1.00	0.00
ATOM	12077	HW2	SOL	3651	30.350	39.720	44.560	1.00	0.00
ATOM	12078	OW	SOL	3652	5.860	3.810	6.570	1.00	0.00
ATOM	12079	HW1	SOL	3652	6.130	2.940	6.290	1.00	0.00
ATOM	12080	HW2	SOL	3652	6.400	4.410	6.060	1.00	0.00
ATOM	12081	OW	SOL	3653	30.240	23.180	11.290	1.00	0.00
ATOM	12082	HW1	SOL	3653	30.780	23.920	11.560	1.00	0.00
ATOM	12083	HW2	SOL	3653	30.810	22.660	10.720	1.00	0.00
ATOM	12084	OW	SOL	3654	32.910	5.660	30.320	1.00	0.00
ATOM	12085	HW1	SOL	3654	32.600	6.440	29.850	1.00	0.00
ATOM	12086	HW2	SOL	3654	32.920	4.970	29.670	1.00	0.00
ATOM	12087	OW	SOL	3655	31.380	22.980	15.750	1.00	0.00
ATOM	12088	HW1	SOL	3655	31.600	23.910	15.680	1.00	0.00
ATOM	12089	HW2	SOL	3655	32.200	22.520	15.550	1.00	0.00
ATOM	12090	OW	SOL	3656	40.090	7.650	18.210	1.00	0.00
ATOM	12091	HW1	SOL	3656	40.730	8.340	18.080	1.00	0.00
ATOM	12092	HW2	SOL	3656	40.470	6.880	17.780	1.00	0.00
ATOM	12093	OW	SOL	3657	55.420	22.910	20.520	1.00	0.00
ATOM	12094	HW1	SOL	3657	55.560	22.600	21.410	1.00	0.00
ATOM	12095	HW2	SOL	3657	55.250	23.850	20.610	1.00	0.00
ATOM	12096	OW	SOL	3658	10.780	50.180	13.650	1.00	0.00
ATOM	12097	HW1	SOL	3658	11.640	50.090	14.050	1.00	0.00
ATOM	12098	HW2	SOL	3658	10.950	50.310	12.720	1.00	0.00
ATOM	12099	OW	SOL	3659	32.550	51.140	35.990	1.00	0.00

ATOM	12100	HW1	SOL	3659	32.090	50.850	36.780	1.00	0.00
ATOM	12101	HW2	SOL	3659	31.850	51.280	35.350	1.00	0.00
ATOM	12102	OW	SOL	3660	44.800	13.350	43.180	1.00	0.00
ATOM	12103	HW1	SOL	3660	44.080	12.740	43.010	1.00	0.00
ATOM	12104	HW2	SOL	3660	45.440	13.160	42.490	1.00	0.00
ATOM	12105	OW	SOL	3661	18.590	13.120	31.900	1.00	0.00
ATOM	12106	HW1	SOL	3661	18.770	14.050	32.090	1.00	0.00
ATOM	12107	HW2	SOL	3661	18.040	12.840	32.630	1.00	0.00
ATOM	12108	OW	SOL	3662	4.280	33.790	19.570	1.00	0.00
ATOM	12109	HW1	SOL	3662	4.510	32.880	19.390	1.00	0.00
ATOM	12110	HW2	SOL	3662	4.870	34.060	20.280	1.00	0.00
ATOM	12111	OW	SOL	3663	44.140	1.870	16.110	1.00	0.00
ATOM	12112	HW1	SOL	3663	43.890	1.370	16.880	1.00	0.00
ATOM	12113	HW2	SOL	3663	43.660	1.450	15.390	1.00	0.00
ATOM	12114	OW	SOL	3664	21.740	30.380	54.380	1.00	0.00
ATOM	12115	HW1	SOL	3664	22.080	30.050	53.550	1.00	0.00
ATOM	12116	HW2	SOL	3664	22.210	29.880	55.050	1.00	0.00
ATOM	12117	OW	SOL	3665	46.130	26.970	47.750	1.00	0.00
ATOM	12118	HW1	SOL	3665	45.270	27.260	48.040	1.00	0.00
ATOM	12119	HW2	SOL	3665	46.090	27.000	46.800	1.00	0.00
ATOM	12120	OW	SOL	3666	43.420	49.120	44.720	1.00	0.00
ATOM	12121	HW1	SOL	3666	43.680	48.230	44.930	1.00	0.00
ATOM	12122	HW2	SOL	3666	43.550	49.190	43.770	1.00	0.00
ATOM	12123	OW	SOL	3667	27.850	18.870	14.640	1.00	0.00
ATOM	12124	HW1	SOL	3667	28.640	19.110	14.150	1.00	0.00
ATOM	12125	HW2	SOL	3667	27.710	19.610	15.230	1.00	0.00
ATOM	12126	OW	SOL	3668	20.830	38.030	51.240	1.00	0.00
ATOM	12127	HW1	SOL	3668	20.490	38.780	51.730	1.00	0.00
ATOM	12128	HW2	SOL	3668	20.190	37.880	50.550	1.00	0.00
ATOM	12129	OW	SOL	3669	49.110	12.100	42.150	1.00	0.00
ATOM	12130	HW1	SOL	3669	49.870	11.990	41.580	1.00	0.00
ATOM	12131	HW2	SOL	3669	48.350	12.000	41.570	1.00	0.00
ATOM	12132	OW	SOL	3670	19.390	21.020	55.120	1.00	0.00
ATOM	12133	HW1	SOL	3670	18.710	20.930	55.790	1.00	0.00
ATOM	12134	HW2	SOL	3670	19.160	20.360	54.460	1.00	0.00
ATOM	12135	OW	SOL	3671	43.210	18.640	8.130	1.00	0.00
ATOM	12136	HW1	SOL	3671	43.890	18.070	7.750	1.00	0.00
ATOM	12137	HW2	SOL	3671	42.770	19.020	7.370	1.00	0.00
ATOM	12138	OW	SOL	3672	15.830	34.180	39.690	1.00	0.00
ATOM	12139	HW1	SOL	3672	16.420	33.700	39.120	1.00	0.00
ATOM	12140	HW2	SOL	3672	16.410	34.650	40.290	1.00	0.00
ATOM	12141	OW	SOL	3673	0.810	25.250	55.290	1.00	0.00
ATOM	12142	HW1	SOL	3673	0.600	24.750	54.500	1.00	0.00
ATOM	12143	HW2	SOL	3673	0.600	24.660	56.010	1.00	0.00

ATOM	12144	OW	SOL	3674	30.060	25.740	37.520	1.00	0.00
ATOM	12145	HW1	SOL	3674	29.930	26.650	37.790	1.00	0.00
ATOM	12146	HW2	SOL	3674	30.380	25.290	38.300	1.00	0.00
ATOM	12147	OW	SOL	3675	40.110	53.150	47.860	1.00	0.00
ATOM	12148	HW1	SOL	3675	39.910	53.340	46.940	1.00	0.00
ATOM	12149	HW2	SOL	3675	39.980	53.980	48.310	1.00	0.00
ATOM	12150	OW	SOL	3676	32.420	1.440	54.820	1.00	0.00
ATOM	12151	HW1	SOL	3676	31.910	2.170	55.180	1.00	0.00
ATOM	12152	HW2	SOL	3676	32.750	0.980	55.590	1.00	0.00
ATOM	12153	OW	SOL	3677	55.500	5.580	26.920	1.00	0.00
ATOM	12154	HW1	SOL	3677	56.100	5.400	27.650	1.00	0.00
ATOM	12155	HW2	SOL	3677	55.640	6.500	26.720	1.00	0.00
ATOM	12156	OW	SOL	3678	22.850	29.440	28.420	1.00	0.00
ATOM	12157	HW1	SOL	3678	23.060	30.110	27.760	1.00	0.00
ATOM	12158	HW2	SOL	3678	21.940	29.200	28.230	1.00	0.00
ATOM	12159	OW	SOL	3679	24.140	4.150	10.390	1.00	0.00
ATOM	12160	HW1	SOL	3679	23.450	3.490	10.320	1.00	0.00
ATOM	12161	HW2	SOL	3679	23.700	4.980	10.190	1.00	0.00
ATOM	12162	OW	SOL	3680	19.610	35.720	34.370	1.00	0.00
ATOM	12163	HW1	SOL	3680	19.990	35.630	33.500	1.00	0.00
ATOM	12164	HW2	SOL	3680	18.670	35.830	34.210	1.00	0.00
ATOM	12165	OW	SOL	3681	44.410	9.540	18.060	1.00	0.00
ATOM	12166	HW1	SOL	3681	44.320	9.160	17.180	1.00	0.00
ATOM	12167	HW2	SOL	3681	43.520	9.810	18.290	1.00	0.00
ATOM	12168	OW	SOL	3682	45.340	16.880	54.770	1.00	0.00
ATOM	12169	HW1	SOL	3682	46.090	16.410	55.150	1.00	0.00
ATOM	12170	HW2	SOL	3682	44.790	16.190	54.400	1.00	0.00
ATOM	12171	OW	SOL	3683	16.770	31.320	29.260	1.00	0.00
ATOM	12172	HW1	SOL	3683	16.390	31.800	28.520	1.00	0.00
ATOM	12173	HW2	SOL	3683	16.110	30.670	29.490	1.00	0.00
ATOM	12174	OW	SOL	3684	45.740	20.580	26.360	1.00	0.00
ATOM	12175	HW1	SOL	3684	46.470	21.020	26.800	1.00	0.00
ATOM	12176	HW2	SOL	3684	45.710	19.710	26.760	1.00	0.00
ATOM	12177	OW	SOL	3685	48.850	4.520	22.990	1.00	0.00
ATOM	12178	HW1	SOL	3685	48.990	5.460	23.020	1.00	0.00
ATOM	12179	HW2	SOL	3685	49.590	4.150	23.480	1.00	0.00
ATOM	12180	OW	SOL	3686	24.500	26.740	16.850	1.00	0.00
ATOM	12181	HW1	SOL	3686	24.590	25.830	17.120	1.00	0.00
ATOM	12182	HW2	SOL	3686	23.740	26.750	16.270	1.00	0.00
ATOM	12183	OW	SOL	3687	3.300	52.950	37.420	1.00	0.00
ATOM	12184	HW1	SOL	3687	2.930	53.730	37.010	1.00	0.00
ATOM	12185	HW2	SOL	3687	3.150	52.250	36.780	1.00	0.00
ATOM	12186	OW	SOL	3688	50.110	46.820	24.060	1.00	0.00
ATOM	12187	HW1	SOL	3688	51.030	46.760	23.800	1.00	0.00

ATOM	12188	HW2	SOL	3688	50.140	46.940	25.010	1.00	0.00
ATOM	12189	OW	SOL	3689	3.050	47.900	32.460	1.00	0.00
ATOM	12190	HW1	SOL	3689	2.650	48.010	33.320	1.00	0.00
ATOM	12191	HW2	SOL	3689	3.370	47.000	32.450	1.00	0.00
ATOM	12192	OW	SOL	3690	18.440	0.250	50.800	1.00	0.00
ATOM	12193	HW1	SOL	3690	17.950	0.470	50.010	1.00	0.00
ATOM	12194	HW2	SOL	3690	18.370	1.030	51.350	1.00	0.00
ATOM	12195	OW	SOL	3691	52.270	41.320	4.240	1.00	0.00
ATOM	12196	HW1	SOL	3691	53.130	41.490	3.850	1.00	0.00
ATOM	12197	HW2	SOL	3691	51.660	41.380	3.510	1.00	0.00
ATOM	12198	OW	SOL	3692	3.110	45.880	35.550	1.00	0.00
ATOM	12199	HW1	SOL	3692	3.850	45.490	36.000	1.00	0.00
ATOM	12200	HW2	SOL	3692	3.010	46.740	35.960	1.00	0.00
ATOM	12201	OW	SOL	3693	20.160	52.550	40.880	1.00	0.00
ATOM	12202	HW1	SOL	3693	20.590	52.670	40.030	1.00	0.00
ATOM	12203	HW2	SOL	3693	19.340	52.100	40.670	1.00	0.00
ATOM	12204	OW	SOL	3694	10.220	22.350	9.260	1.00	0.00
ATOM	12205	HW1	SOL	3694	10.790	23.120	9.370	1.00	0.00
ATOM	12206	HW2	SOL	3694	9.850	22.200	10.120	1.00	0.00
ATOM	12207	OW	SOL	3695	28.280	0.800	22.070	1.00	0.00
ATOM	12208	HW1	SOL	3695	28.790	0.240	21.490	1.00	0.00
ATOM	12209	HW2	SOL	3695	28.820	0.890	22.850	1.00	0.00
ATOM	12210	OW	SOL	3696	32.440	41.120	47.720	1.00	0.00
ATOM	12211	HW1	SOL	3696	31.800	41.090	48.420	1.00	0.00
ATOM	12212	HW2	SOL	3696	32.190	40.420	47.120	1.00	0.00
ATOM	12213	OW	SOL	3697	37.910	46.650	0.670	1.00	0.00
ATOM	12214	HW1	SOL	3697	37.790	46.830	1.600	1.00	0.00
ATOM	12215	HW2	SOL	3697	37.390	45.860	0.500	1.00	0.00
ATOM	12216	OW	SOL	3698	41.130	48.200	30.180	1.00	0.00
ATOM	12217	HW1	SOL	3698	41.530	48.540	29.380	1.00	0.00
ATOM	12218	HW2	SOL	3698	40.440	48.830	30.390	1.00	0.00
ATOM	12219	OW	SOL	3699	19.770	32.760	50.400	1.00	0.00
ATOM	12220	HW1	SOL	3699	18.840	32.710	50.180	1.00	0.00
ATOM	12221	HW2	SOL	3699	19.790	33.030	51.320	1.00	0.00
ATOM	12222	OW	SOL	3700	39.910	20.860	24.030	1.00	0.00
ATOM	12223	HW1	SOL	3700	39.750	20.120	24.610	1.00	0.00
ATOM	12224	HW2	SOL	3700	40.180	20.460	23.200	1.00	0.00
ATOM	12225	OW	SOL	3701	45.290	11.450	36.730	1.00	0.00
ATOM	12226	HW1	SOL	3701	45.020	10.530	36.720	1.00	0.00
ATOM	12227	HW2	SOL	3701	46.040	11.500	36.140	1.00	0.00
ATOM	12228	OW	SOL	3702	35.050	18.140	39.830	1.00	0.00
ATOM	12229	HW1	SOL	3702	35.700	17.710	40.380	1.00	0.00
ATOM	12230	HW2	SOL	3702	34.460	18.560	40.460	1.00	0.00
ATOM	12231	OW	SOL	3703	37.240	7.380	47.860	1.00	0.00

ATOM	12232	HW1	SOL	3703	36.980	8.200	47.440	1.00	0.00
ATOM	12233	HW2	SOL	3703	37.380	6.780	47.130	1.00	0.00
ATOM	12234	OW	SOL	3704	37.130	13.410	7.090	1.00	0.00
ATOM	12235	HW1	SOL	3704	37.990	13.260	6.690	1.00	0.00
ATOM	12236	HW2	SOL	3704	37.330	13.850	7.920	1.00	0.00
ATOM	12237	OW	SOL	3705	6.650	32.940	27.060	1.00	0.00
ATOM	12238	HW1	SOL	3705	7.560	32.910	26.770	1.00	0.00
ATOM	12239	HW2	SOL	3705	6.340	33.800	26.780	1.00	0.00
ATOM	12240	OW	SOL	3706	31.000	14.810	37.870	1.00	0.00
ATOM	12241	HW1	SOL	3706	30.170	14.340	37.960	1.00	0.00
ATOM	12242	HW2	SOL	3706	31.210	15.100	38.760	1.00	0.00
ATOM	12243	OW	SOL	3707	14.590	29.190	50.270	1.00	0.00
ATOM	12244	HW1	SOL	3707	13.940	28.940	50.930	1.00	0.00
ATOM	12245	HW2	SOL	3707	14.510	28.520	49.590	1.00	0.00
ATOM	12246	OW	SOL	3708	1.160	31.250	51.760	1.00	0.00
ATOM	12247	HW1	SOL	3708	1.190	31.580	52.660	1.00	0.00
ATOM	12248	HW2	SOL	3708	1.800	31.780	51.290	1.00	0.00
ATOM	12249	OW	SOL	3709	1.300	30.550	24.680	1.00	0.00
ATOM	12250	HW1	SOL	3709	1.970	31.150	25.010	1.00	0.00
ATOM	12251	HW2	SOL	3709	0.540	30.720	25.230	1.00	0.00
ATOM	12252	OW	SOL	3710	7.710	0.210	15.870	1.00	0.00
ATOM	12253	HW1	SOL	3710	7.020	-0.170	15.330	1.00	0.00
ATOM	12254	HW2	SOL	3710	7.490	-0.050	16.760	1.00	0.00
ATOM	12255	OW	SOL	3711	42.860	13.340	15.500	1.00	0.00
ATOM	12256	HW1	SOL	3711	43.000	13.470	16.430	1.00	0.00
ATOM	12257	HW2	SOL	3711	42.760	12.400	15.400	1.00	0.00
ATOM	12258	OW	SOL	3712	13.030	39.050	24.080	1.00	0.00
ATOM	12259	HW1	SOL	3712	12.600	38.590	24.800	1.00	0.00
ATOM	12260	HW2	SOL	3712	13.560	39.720	24.520	1.00	0.00
ATOM	12261	OW	SOL	3713	25.820	48.060	23.410	1.00	0.00
ATOM	12262	HW1	SOL	3713	26.530	47.680	22.890	1.00	0.00
ATOM	12263	HW2	SOL	3713	25.900	49.000	23.280	1.00	0.00
ATOM	12264	OW	SOL	3714	54.960	22.360	48.680	1.00	0.00
ATOM	12265	HW1	SOL	3714	54.540	21.690	49.220	1.00	0.00
ATOM	12266	HW2	SOL	3714	54.680	23.190	49.080	1.00	0.00
ATOM	12267	OW	SOL	3715	46.330	41.080	9.760	1.00	0.00
ATOM	12268	HW1	SOL	3715	46.470	41.130	8.820	1.00	0.00
ATOM	12269	HW2	SOL	3715	46.680	41.900	10.100	1.00	0.00
ATOM	12270	OW	SOL	3716	55.100	12.970	32.420	1.00	0.00
ATOM	12271	HW1	SOL	3716	54.210	12.850	32.100	1.00	0.00
ATOM	12272	HW2	SOL	3716	55.650	12.570	31.740	1.00	0.00
ATOM	12273	OW	SOL	3717	39.490	32.280	2.880	1.00	0.00
ATOM	12274	HW1	SOL	3717	39.530	31.360	2.620	1.00	0.00
ATOM	12275	HW2	SOL	3717	39.060	32.720	2.140	1.00	0.00

ATOM	12276	OW	SOL	3718	26.560	41.710	9.730	1.00	0.00
ATOM	12277	HW1	SOL	3718	27.180	41.120	9.290	1.00	0.00
ATOM	12278	HW2	SOL	3718	27.120	42.390	10.110	1.00	0.00
ATOM	12279	OW	SOL	3719	49.460	18.470	7.550	1.00	0.00
ATOM	12280	HW1	SOL	3719	49.750	19.370	7.390	1.00	0.00
ATOM	12281	HW2	SOL	3719	49.220	18.470	8.480	1.00	0.00
ATOM	12282	OW	SOL	3720	49.540	25.790	31.770	1.00	0.00
ATOM	12283	HW1	SOL	3720	49.620	25.810	30.820	1.00	0.00
ATOM	12284	HW2	SOL	3720	49.920	24.950	32.020	1.00	0.00
ATOM	12285	OW	SOL	3721	45.750	16.070	29.580	1.00	0.00
ATOM	12286	HW1	SOL	3721	45.220	15.470	30.100	1.00	0.00
ATOM	12287	HW2	SOL	3721	45.230	16.880	29.530	1.00	0.00
ATOM	12288	OW	SOL	3722	37.480	20.030	16.680	1.00	0.00
ATOM	12289	HW1	SOL	3722	38.070	19.300	16.850	1.00	0.00
ATOM	12290	HW2	SOL	3722	37.550	20.180	15.740	1.00	0.00
ATOM	12291	OW	SOL	3723	37.400	45.060	14.070	1.00	0.00
ATOM	12292	HW1	SOL	3723	37.670	44.360	13.480	1.00	0.00
ATOM	12293	HW2	SOL	3723	37.580	45.870	13.580	1.00	0.00
ATOM	12294	OW	SOL	3724	52.160	53.950	13.640	1.00	0.00
ATOM	12295	HW1	SOL	3724	52.510	54.170	12.780	1.00	0.00
ATOM	12296	HW2	SOL	3724	51.950	53.020	13.580	1.00	0.00
ATOM	12297	OW	SOL	3725	23.900	14.530	6.830	1.00	0.00
ATOM	12298	HW1	SOL	3725	24.750	14.960	6.760	1.00	0.00
ATOM	12299	HW2	SOL	3725	23.860	14.240	7.740	1.00	0.00
ATOM	12300	OW	SOL	3726	55.200	37.370	14.770	1.00	0.00
ATOM	12301	HW1	SOL	3726	55.920	37.610	14.180	1.00	0.00
ATOM	12302	HW2	SOL	3726	55.560	37.500	15.650	1.00	0.00
ATOM	12303	OW	SOL	3727	18.360	18.890	19.150	1.00	0.00
ATOM	12304	HW1	SOL	3727	17.930	18.830	18.290	1.00	0.00
ATOM	12305	HW2	SOL	3727	19.190	19.320	18.980	1.00	0.00
ATOM	12306	OW	SOL	3728	39.180	34.430	50.270	1.00	0.00
ATOM	12307	HW1	SOL	3728	39.830	33.760	50.080	1.00	0.00
ATOM	12308	HW2	SOL	3728	38.470	33.960	50.710	1.00	0.00
ATOM	12309	OW	SOL	3729	30.410	51.790	20.340	1.00	0.00
ATOM	12310	HW1	SOL	3729	30.710	52.360	19.630	1.00	0.00
ATOM	12311	HW2	SOL	3729	30.750	50.930	20.110	1.00	0.00
ATOM	12312	OW	SOL	3730	27.310	24.260	8.150	1.00	0.00
ATOM	12313	HW1	SOL	3730	27.970	23.890	7.560	1.00	0.00
ATOM	12314	HW2	SOL	3730	26.720	24.750	7.580	1.00	0.00
ATOM	12315	OW	SOL	3731	3.690	40.630	46.290	1.00	0.00
ATOM	12316	HW1	SOL	3731	3.110	39.910	46.030	1.00	0.00
ATOM	12317	HW2	SOL	3731	3.910	40.450	47.200	1.00	0.00
ATOM	12318	OW	SOL	3732	31.180	53.230	18.000	1.00	0.00
ATOM	12319	HW1	SOL	3732	31.470	52.490	17.470	1.00	0.00

ATOM	12320	HW2	SOL	3732	30.900	53.890	17.360	1.00	0.00
ATOM	12321	OW	SOL	3733	48.910	2.970	14.710	1.00	0.00
ATOM	12322	HW1	SOL	3733	48.310	2.920	15.450	1.00	0.00
ATOM	12323	HW2	SOL	3733	49.660	2.440	14.970	1.00	0.00
ATOM	12324	OW	SOL	3734	15.350	54.010	48.170	1.00	0.00
ATOM	12325	HW1	SOL	3734	16.220	53.920	47.770	1.00	0.00
ATOM	12326	HW2	SOL	3734	15.010	54.830	47.830	1.00	0.00
ATOM	12327	OW	SOL	3735	38.060	3.140	2.140	1.00	0.00
ATOM	12328	HW1	SOL	3735	38.290	2.560	1.410	1.00	0.00
ATOM	12329	HW2	SOL	3735	37.610	3.880	1.730	1.00	0.00
ATOM	12330	OW	SOL	3736	34.450	21.040	8.370	1.00	0.00
ATOM	12331	HW1	SOL	3736	35.130	20.880	7.720	1.00	0.00
ATOM	12332	HW2	SOL	3736	33.820	21.610	7.920	1.00	0.00
ATOM	12333	OW	SOL	3737	24.890	15.250	27.190	1.00	0.00
ATOM	12334	HW1	SOL	3737	24.190	14.630	27.390	1.00	0.00
ATOM	12335	HW2	SOL	3737	24.480	15.900	26.630	1.00	0.00
ATOM	12336	OW	SOL	3738	24.810	23.390	25.200	1.00	0.00
ATOM	12337	HW1	SOL	3738	24.470	22.510	25.040	1.00	0.00
ATOM	12338	HW2	SOL	3738	25.400	23.290	25.960	1.00	0.00
ATOM	12339	OW	SOL	3739	53.210	13.070	41.780	1.00	0.00
ATOM	12340	HW1	SOL	3739	52.410	13.220	42.290	1.00	0.00
ATOM	12341	HW2	SOL	3739	53.750	12.490	42.320	1.00	0.00
ATOM	12342	OW	SOL	3740	5.500	43.190	51.420	1.00	0.00
ATOM	12343	HW1	SOL	3740	5.290	43.070	50.500	1.00	0.00
ATOM	12344	HW2	SOL	3740	6.450	43.320	51.440	1.00	0.00
ATOM	12345	OW	SOL	3741	23.800	33.180	29.520	1.00	0.00
ATOM	12346	HW1	SOL	3741	23.900	32.430	30.110	1.00	0.00
ATOM	12347	HW2	SOL	3741	23.040	32.960	28.980	1.00	0.00
ATOM	12348	OW	SOL	3742	32.430	21.520	21.290	1.00	0.00
ATOM	12349	HW1	SOL	3742	32.640	21.330	20.380	1.00	0.00
ATOM	12350	HW2	SOL	3742	32.500	20.680	21.730	1.00	0.00
ATOM	12351	OW	SOL	3743	36.660	30.900	13.950	1.00	0.00
ATOM	12352	HW1	SOL	3743	36.610	31.840	14.090	1.00	0.00
ATOM	12353	HW2	SOL	3743	37.460	30.630	14.400	1.00	0.00
ATOM	12354	OW	SOL	3744	22.380	55.240	34.200	1.00	0.00
ATOM	12355	HW1	SOL	3744	21.430	55.270	34.090	1.00	0.00
ATOM	12356	HW2	SOL	3744	22.730	55.400	33.330	1.00	0.00
ATOM	12357	OW	SOL	3745	23.680	4.170	34.730	1.00	0.00
ATOM	12358	HW1	SOL	3745	23.490	3.440	35.310	1.00	0.00
ATOM	12359	HW2	SOL	3745	24.600	4.040	34.470	1.00	0.00
ATOM	12360	OW	SOL	3746	26.120	36.850	48.650	1.00	0.00
ATOM	12361	HW1	SOL	3746	25.210	36.890	48.370	1.00	0.00
ATOM	12362	HW2	SOL	3746	26.130	36.170	49.330	1.00	0.00
ATOM	12363	OW	SOL	3747	15.120	7.970	18.200	1.00	0.00

ATOM	12364	HW1	SOL	3747	15.370	8.390	17.370	1.00	0.00
ATOM	12365	HW2	SOL	3747	15.940	7.610	18.540	1.00	0.00
ATOM	12366	OW	SOL	3748	37.980	50.890	10.500	1.00	0.00
ATOM	12367	HW1	SOL	3748	37.290	51.270	11.040	1.00	0.00
ATOM	12368	HW2	SOL	3748	38.540	50.420	11.120	1.00	0.00
ATOM	12369	OW	SOL	3749	9.760	24.890	28.520	1.00	0.00
ATOM	12370	HW1	SOL	3749	9.560	25.750	28.140	1.00	0.00
ATOM	12371	HW2	SOL	3749	10.710	24.800	28.400	1.00	0.00
ATOM	12372	OW	SOL	3750	46.120	7.720	5.050	1.00	0.00
ATOM	12373	HW1	SOL	3750	45.720	8.200	5.780	1.00	0.00
ATOM	12374	HW2	SOL	3750	45.400	7.200	4.690	1.00	0.00
ATOM	12375	OW	SOL	3751	10.980	14.000	16.310	1.00	0.00
ATOM	12376	HW1	SOL	3751	10.400	14.570	15.810	1.00	0.00
ATOM	12377	HW2	SOL	3751	10.890	14.310	17.210	1.00	0.00
ATOM	12378	OW	SOL	3752	47.850	22.070	27.750	1.00	0.00
ATOM	12379	HW1	SOL	3752	47.710	22.710	27.050	1.00	0.00
ATOM	12380	HW2	SOL	3752	47.740	22.580	28.560	1.00	0.00
ATOM	12381	OW	SOL	3753	47.410	48.610	29.240	1.00	0.00
ATOM	12382	HW1	SOL	3753	47.030	49.150	29.930	1.00	0.00
ATOM	12383	HW2	SOL	3753	47.860	49.230	28.670	1.00	0.00
ATOM	12384	OW	SOL	3754	37.970	30.140	50.360	1.00	0.00
ATOM	12385	HW1	SOL	3754	37.710	29.300	49.970	1.00	0.00
ATOM	12386	HW2	SOL	3754	38.610	30.500	49.750	1.00	0.00
ATOM	12387	OW	SOL	3755	48.300	19.320	1.680	1.00	0.00
ATOM	12388	HW1	SOL	3755	47.510	19.640	2.120	1.00	0.00
ATOM	12389	HW2	SOL	3755	48.830	18.960	2.390	1.00	0.00
ATOM	12390	OW	SOL	3756	49.980	18.750	45.180	1.00	0.00
ATOM	12391	HW1	SOL	3756	49.080	18.530	44.930	1.00	0.00
ATOM	12392	HW2	SOL	3756	49.960	18.800	46.140	1.00	0.00
ATOM	12393	OW	SOL	3757	43.120	15.000	36.180	1.00	0.00
ATOM	12394	HW1	SOL	3757	42.900	15.320	37.050	1.00	0.00
ATOM	12395	HW2	SOL	3757	43.360	15.780	35.680	1.00	0.00
ATOM	12396	OW	SOL	3758	6.500	9.260	40.220	1.00	0.00
ATOM	12397	HW1	SOL	3758	5.850	8.840	39.670	1.00	0.00
ATOM	12398	HW2	SOL	3758	7.000	9.820	39.630	1.00	0.00
ATOM	12399	OW	SOL	3759	14.920	39.840	35.380	1.00	0.00
ATOM	12400	HW1	SOL	3759	14.530	39.110	35.860	1.00	0.00
ATOM	12401	HW2	SOL	3759	15.000	40.540	36.030	1.00	0.00
ATOM	12402	OW	SOL	3760	32.450	38.610	0.250	1.00	0.00
ATOM	12403	HW1	SOL	3760	32.860	39.000	1.010	1.00	0.00
ATOM	12404	HW2	SOL	3760	32.580	37.670	0.350	1.00	0.00
ATOM	12405	OW	SOL	3761	47.030	30.680	48.400	1.00	0.00
ATOM	12406	HW1	SOL	3761	47.030	31.630	48.480	1.00	0.00
ATOM	12407	HW2	SOL	3761	46.620	30.510	47.550	1.00	0.00

ATOM	12408	OW	SOL	3762	19.100	35.280	28.730	1.00	0.00
ATOM	12409	HW1	SOL	3762	19.600	34.560	28.340	1.00	0.00
ATOM	12410	HW2	SOL	3762	19.770	35.870	29.070	1.00	0.00
ATOM	12411	OW	SOL	3763	47.270	33.990	53.950	1.00	0.00
ATOM	12412	HW1	SOL	3763	48.190	34.060	54.180	1.00	0.00
ATOM	12413	HW2	SOL	3763	47.210	33.150	53.480	1.00	0.00
ATOM	12414	OW	SOL	3764	39.780	44.280	15.490	1.00	0.00
ATOM	12415	HW1	SOL	3764	39.710	43.670	16.230	1.00	0.00
ATOM	12416	HW2	SOL	3764	38.870	44.420	15.210	1.00	0.00
ATOM	12417	OW	SOL	3765	4.200	26.240	26.850	1.00	0.00
ATOM	12418	HW1	SOL	3765	3.350	26.640	26.670	1.00	0.00
ATOM	12419	HW2	SOL	3765	4.500	26.660	27.660	1.00	0.00
ATOM	12420	OW	SOL	3766	5.650	13.390	32.210	1.00	0.00
ATOM	12421	HW1	SOL	3766	5.590	14.340	32.190	1.00	0.00
ATOM	12422	HW2	SOL	3766	5.430	13.150	33.110	1.00	0.00
ATOM	12423	OW	SOL	3767	38.090	14.390	55.320	1.00	0.00
ATOM	12424	HW1	SOL	3767	38.310	14.440	56.250	1.00	0.00
ATOM	12425	HW2	SOL	3767	38.280	13.490	55.070	1.00	0.00
ATOM	12426	OW	SOL	3768	21.400	6.600	17.260	1.00	0.00
ATOM	12427	HW1	SOL	3768	22.110	6.440	17.890	1.00	0.00
ATOM	12428	HW2	SOL	3768	20.930	7.360	17.610	1.00	0.00
ATOM	12429	OW	SOL	3769	8.490	16.580	53.940	1.00	0.00
ATOM	12430	HW1	SOL	3769	8.800	17.320	53.420	1.00	0.00
ATOM	12431	HW2	SOL	3769	7.730	16.250	53.460	1.00	0.00
ATOM	12432	OW	SOL	3770	43.970	5.130	53.250	1.00	0.00
ATOM	12433	HW1	SOL	3770	44.570	5.690	53.750	1.00	0.00
ATOM	12434	HW2	SOL	3770	44.510	4.370	53.000	1.00	0.00
ATOM	12435	OW	SOL	3771	46.420	28.890	15.450	1.00	0.00
ATOM	12436	HW1	SOL	3771	46.910	29.590	15.900	1.00	0.00
ATOM	12437	HW2	SOL	3771	46.120	29.300	14.640	1.00	0.00
ATOM	12438	OW	SOL	3772	2.120	38.690	44.880	1.00	0.00
ATOM	12439	HW1	SOL	3772	2.290	37.850	44.460	1.00	0.00
ATOM	12440	HW2	SOL	3772	2.050	39.310	44.150	1.00	0.00
ATOM	12441	OW	SOL	3773	30.460	13.590	21.870	1.00	0.00
ATOM	12442	HW1	SOL	3773	29.550	13.800	21.700	1.00	0.00
ATOM	12443	HW2	SOL	3773	30.860	14.430	22.090	1.00	0.00
ATOM	12444	OW	SOL	3774	43.550	47.900	12.850	1.00	0.00
ATOM	12445	HW1	SOL	3774	44.340	48.060	13.360	1.00	0.00
ATOM	12446	HW2	SOL	3774	43.120	48.760	12.790	1.00	0.00
ATOM	12447	OW	SOL	3775	35.910	26.680	2.620	1.00	0.00
ATOM	12448	HW1	SOL	3775	35.970	25.960	1.980	1.00	0.00
ATOM	12449	HW2	SOL	3775	36.720	26.620	3.120	1.00	0.00
ATOM	12450	OW	SOL	3776	8.120	17.530	12.200	1.00	0.00
ATOM	12451	HW1	SOL	3776	7.450	16.880	12.000	1.00	0.00

ATOM	12452	HW2	SOL	3776	8.660	17.120	12.870	1.00	0.00
ATOM	12453	OW	SOL	3777	8.630	5.050	20.550	1.00	0.00
ATOM	12454	HW1	SOL	3777	9.170	4.440	21.040	1.00	0.00
ATOM	12455	HW2	SOL	3777	8.450	5.760	21.170	1.00	0.00
ATOM	12456	OW	SOL	3778	14.490	45.090	55.330	1.00	0.00
ATOM	12457	HW1	SOL	3778	14.510	45.740	54.620	1.00	0.00
ATOM	12458	HW2	SOL	3778	14.910	45.530	56.070	1.00	0.00
ATOM	12459	OW	SOL	3779	55.590	30.800	35.540	1.00	0.00
ATOM	12460	HW1	SOL	3779	54.770	30.560	35.970	1.00	0.00
ATOM	12461	HW2	SOL	3779	55.340	31.420	34.850	1.00	0.00
ATOM	12462	OW	SOL	3780	37.490	43.090	24.630	1.00	0.00
ATOM	12463	HW1	SOL	3780	36.830	42.400	24.720	1.00	0.00
ATOM	12464	HW2	SOL	3780	37.470	43.320	23.700	1.00	0.00
ATOM	12465	OW	SOL	3781	34.490	36.270	7.470	1.00	0.00
ATOM	12466	HW1	SOL	3781	34.130	35.590	6.900	1.00	0.00
ATOM	12467	HW2	SOL	3781	34.850	36.920	6.860	1.00	0.00
ATOM	12468	OW	SOL	3782	14.810	38.890	52.620	1.00	0.00
ATOM	12469	HW1	SOL	3782	14.820	39.480	53.370	1.00	0.00
ATOM	12470	HW2	SOL	3782	13.950	38.470	52.650	1.00	0.00
ATOM	12471	OW	SOL	3783	34.950	12.530	43.730	1.00	0.00
ATOM	12472	HW1	SOL	3783	34.790	12.900	42.860	1.00	0.00
ATOM	12473	HW2	SOL	3783	35.130	13.290	44.290	1.00	0.00
ATOM	12474	OW	SOL	3784	50.770	3.380	6.680	1.00	0.00
ATOM	12475	HW1	SOL	3784	51.230	3.250	5.840	1.00	0.00
ATOM	12476	HW2	SOL	3784	50.480	4.300	6.650	1.00	0.00
ATOM	12477	OW	SOL	3785	42.490	31.370	16.970	1.00	0.00
ATOM	12478	HW1	SOL	3785	42.660	31.670	16.080	1.00	0.00
ATOM	12479	HW2	SOL	3785	41.710	30.820	16.890	1.00	0.00
ATOM	12480	OW	SOL	3786	13.630	55.410	17.750	1.00	0.00
ATOM	12481	HW1	SOL	3786	12.720	55.260	17.490	1.00	0.00
ATOM	12482	HW2	SOL	3786	14.140	54.920	17.110	1.00	0.00
ATOM	12483	OW	SOL	3787	1.560	51.990	8.320	1.00	0.00
ATOM	12484	HW1	SOL	3787	1.540	52.550	7.550	1.00	0.00
ATOM	12485	HW2	SOL	3787	0.660	51.680	8.420	1.00	0.00
ATOM	12486	OW	SOL	3788	47.350	43.670	45.240	1.00	0.00
ATOM	12487	HW1	SOL	3788	46.660	44.260	44.910	1.00	0.00
ATOM	12488	HW2	SOL	3788	48.020	43.680	44.550	1.00	0.00
ATOM	12489	OW	SOL	3789	20.990	55.010	50.340	1.00	0.00
ATOM	12490	HW1	SOL	3789	20.280	55.590	50.600	1.00	0.00
ATOM	12491	HW2	SOL	3789	21.790	55.500	50.540	1.00	0.00
ATOM	12492	OW	SOL	3790	22.580	15.140	20.310	1.00	0.00
ATOM	12493	HW1	SOL	3790	22.490	15.910	19.740	1.00	0.00
ATOM	12494	HW2	SOL	3790	23.360	15.320	20.830	1.00	0.00
ATOM	12495	OW	SOL	3791	50.510	20.920	3.670	1.00	0.00

ATOM	12496	HW1	SOL	3791	50.820	21.050	2.770	1.00	0.00
ATOM	12497	HW2	SOL	3791	49.680	21.400	3.710	1.00	0.00
ATOM	12498	OW	SOL	3792	45.960	53.880	22.920	1.00	0.00
ATOM	12499	HW1	SOL	3792	46.380	54.090	23.760	1.00	0.00
ATOM	12500	HW2	SOL	3792	45.950	52.920	22.890	1.00	0.00
ATOM	12501	OW	SOL	3793	3.310	47.780	47.690	1.00	0.00
ATOM	12502	HW1	SOL	3793	3.380	47.110	48.370	1.00	0.00
ATOM	12503	HW2	SOL	3793	4.200	48.080	47.550	1.00	0.00
ATOM	12504	OW	SOL	3794	3.390	54.140	14.020	1.00	0.00
ATOM	12505	HW1	SOL	3794	3.960	53.390	14.190	1.00	0.00
ATOM	12506	HW2	SOL	3794	2.540	53.760	13.780	1.00	0.00
ATOM	12507	OW	SOL	3795	52.460	45.180	33.520	1.00	0.00
ATOM	12508	HW1	SOL	3795	53.070	44.600	33.070	1.00	0.00
ATOM	12509	HW2	SOL	3795	51.630	45.060	33.080	1.00	0.00
ATOM	12510	OW	SOL	3796	6.970	22.230	36.460	1.00	0.00
ATOM	12511	HW1	SOL	3796	6.950	23.110	36.090	1.00	0.00
ATOM	12512	HW2	SOL	3796	6.680	22.340	37.360	1.00	0.00
ATOM	12513	OW	SOL	3797	35.120	39.450	46.530	1.00	0.00
ATOM	12514	HW1	SOL	3797	34.830	38.820	47.190	1.00	0.00
ATOM	12515	HW2	SOL	3797	34.760	40.290	46.820	1.00	0.00
ATOM	12516	OW	SOL	3798	9.040	22.310	44.650	1.00	0.00
ATOM	12517	HW1	SOL	3798	9.850	21.810	44.600	1.00	0.00
ATOM	12518	HW2	SOL	3798	9.280	23.110	45.110	1.00	0.00
ATOM	12519	OW	SOL	3799	18.730	4.920	39.400	1.00	0.00
ATOM	12520	HW1	SOL	3799	18.820	3.980	39.250	1.00	0.00
ATOM	12521	HW2	SOL	3799	19.630	5.230	39.540	1.00	0.00
ATOM	12522	OW	SOL	3800	32.670	32.490	25.130	1.00	0.00
ATOM	12523	HW1	SOL	3800	32.600	33.410	24.840	1.00	0.00
ATOM	12524	HW2	SOL	3800	32.710	31.990	24.310	1.00	0.00
ATOM	12525	OW	SOL	3801	20.750	0.570	15.180	1.00	0.00
ATOM	12526	HW1	SOL	3801	21.260	1.360	14.990	1.00	0.00
ATOM	12527	HW2	SOL	3801	21.140	0.210	15.970	1.00	0.00
ATOM	12528	OW	SOL	3802	3.020	54.870	2.030	1.00	0.00
ATOM	12529	HW1	SOL	3802	3.140	54.910	1.080	1.00	0.00
ATOM	12530	HW2	SOL	3802	2.150	54.500	2.150	1.00	0.00
ATOM	12531	OW	SOL	3803	26.650	54.330	9.110	1.00	0.00
ATOM	12532	HW1	SOL	3803	25.710	54.180	9.170	1.00	0.00
ATOM	12533	HW2	SOL	3803	27.040	53.470	9.280	1.00	0.00
ATOM	12534	OW	SOL	3804	52.670	52.420	1.990	1.00	0.00
ATOM	12535	HW1	SOL	3804	52.590	53.260	1.530	1.00	0.00
ATOM	12536	HW2	SOL	3804	53.590	52.360	2.240	1.00	0.00
ATOM	12537	OW	SOL	3805	18.410	47.320	39.910	1.00	0.00
ATOM	12538	HW1	SOL	3805	18.410	46.540	40.470	1.00	0.00
ATOM	12539	HW2	SOL	3805	19.310	47.380	39.590	1.00	0.00

ATOM	12540	OW	SOL	3806	17.000	29.980	39.970	1.00	0.00
ATOM	12541	HW1	SOL	3806	16.690	30.200	39.090	1.00	0.00
ATOM	12542	HW2	SOL	3806	17.820	30.470	40.060	1.00	0.00
ATOM	12543	OW	SOL	3807	21.880	28.090	2.150	1.00	0.00
ATOM	12544	HW1	SOL	3807	22.600	28.180	1.530	1.00	0.00
ATOM	12545	HW2	SOL	3807	21.140	27.780	1.620	1.00	0.00
ATOM	12546	OW	SOL	3808	22.420	20.510	31.020	1.00	0.00
ATOM	12547	HW1	SOL	3808	22.630	20.680	31.940	1.00	0.00
ATOM	12548	HW2	SOL	3808	21.490	20.710	30.940	1.00	0.00
ATOM	12549	OW	SOL	3809	15.680	37.340	8.560	1.00	0.00
ATOM	12550	HW1	SOL	3809	16.380	36.700	8.410	1.00	0.00
ATOM	12551	HW2	SOL	3809	15.630	37.430	9.510	1.00	0.00
ATOM	12552	OW	SOL	3810	7.230	55.430	51.770	1.00	0.00
ATOM	12553	HW1	SOL	3810	6.710	56.210	51.950	1.00	0.00
ATOM	12554	HW2	SOL	3810	7.720	55.270	52.580	1.00	0.00
ATOM	12555	OW	SOL	3811	35.640	20.170	20.220	1.00	0.00
ATOM	12556	HW1	SOL	3811	35.480	20.520	21.100	1.00	0.00
ATOM	12557	HW2	SOL	3811	36.160	19.380	20.360	1.00	0.00
ATOM	12558	OW	SOL	3812	37.670	12.100	29.630	1.00	0.00
ATOM	12559	HW1	SOL	3812	38.100	11.510	29.020	1.00	0.00
ATOM	12560	HW2	SOL	3812	37.640	11.620	30.450	1.00	0.00
ATOM	12561	OW	SOL	3813	33.690	53.740	18.940	1.00	0.00
ATOM	12562	HW1	SOL	3813	33.420	54.240	19.710	1.00	0.00
ATOM	12563	HW2	SOL	3813	33.110	52.970	18.940	1.00	0.00
ATOM	12564	OW	SOL	3814	7.960	46.410	30.680	1.00	0.00
ATOM	12565	HW1	SOL	3814	7.640	45.510	30.750	1.00	0.00
ATOM	12566	HW2	SOL	3814	8.810	46.330	30.250	1.00	0.00
ATOM	12567	OW	SOL	3815	14.000	21.270	31.200	1.00	0.00
ATOM	12568	HW1	SOL	3815	14.180	21.230	32.140	1.00	0.00
ATOM	12569	HW2	SOL	3815	14.260	20.400	30.880	1.00	0.00
ATOM	12570	OW	SOL	3816	18.620	10.680	53.910	1.00	0.00
ATOM	12571	HW1	SOL	3816	17.950	10.270	54.450	1.00	0.00
ATOM	12572	HW2	SOL	3816	18.740	10.080	53.180	1.00	0.00
ATOM	12573	OW	SOL	3817	16.440	50.240	49.210	1.00	0.00
ATOM	12574	HW1	SOL	3817	15.780	50.500	49.850	1.00	0.00
ATOM	12575	HW2	SOL	3817	16.850	49.460	49.590	1.00	0.00
ATOM	12576	OW	SOL	3818	43.230	3.500	50.040	1.00	0.00
ATOM	12577	HW1	SOL	3818	42.600	3.050	50.590	1.00	0.00
ATOM	12578	HW2	SOL	3818	43.410	2.900	49.320	1.00	0.00
ATOM	12579	OW	SOL	3819	6.550	25.190	20.310	1.00	0.00
ATOM	12580	HW1	SOL	3819	6.320	24.710	21.100	1.00	0.00
ATOM	12581	HW2	SOL	3819	7.470	25.410	20.410	1.00	0.00
ATOM	12582	OW	SOL	3820	21.100	29.730	49.790	1.00	0.00
ATOM	12583	HW1	SOL	3820	21.700	30.480	49.790	1.00	0.00

ATOM	12584	HW2	SOL	3820	20.270	30.090	50.120	1.00	0.00
ATOM	12585	OW	SOL	3821	1.560	6.660	51.690	1.00	0.00
ATOM	12586	HW1	SOL	3821	0.830	7.150	51.290	1.00	0.00
ATOM	12587	HW2	SOL	3821	1.300	5.740	51.630	1.00	0.00
ATOM	12588	OW	SOL	3822	31.250	15.430	40.680	1.00	0.00
ATOM	12589	HW1	SOL	3822	31.070	16.300	41.050	1.00	0.00
ATOM	12590	HW2	SOL	3822	31.030	14.820	41.380	1.00	0.00
ATOM	12591	OW	SOL	3823	51.910	43.210	43.530	1.00	0.00
ATOM	12592	HW1	SOL	3823	52.300	43.790	42.870	1.00	0.00
ATOM	12593	HW2	SOL	3823	51.220	43.740	43.930	1.00	0.00
ATOM	12594	OW	SOL	3824	7.510	38.540	37.600	1.00	0.00
ATOM	12595	HW1	SOL	3824	7.560	38.970	36.750	1.00	0.00
ATOM	12596	HW2	SOL	3824	8.090	37.780	37.520	1.00	0.00
ATOM	12597	OW	SOL	3825	54.070	43.950	47.580	1.00	0.00
ATOM	12598	HW1	SOL	3825	53.280	44.400	47.880	1.00	0.00
ATOM	12599	HW2	SOL	3825	54.760	44.280	48.170	1.00	0.00
ATOM	12600	OW	SOL	3826	35.170	12.160	8.740	1.00	0.00
ATOM	12601	HW1	SOL	3826	35.580	12.480	7.940	1.00	0.00
ATOM	12602	HW2	SOL	3826	35.540	11.290	8.870	1.00	0.00
ATOM	12603	OW	SOL	3827	19.350	50.780	31.440	1.00	0.00
ATOM	12604	HW1	SOL	3827	19.140	50.920	32.370	1.00	0.00
ATOM	12605	HW2	SOL	3827	19.040	49.900	31.260	1.00	0.00
ATOM	12606	OW	SOL	3828	43.410	53.150	21.430	1.00	0.00
ATOM	12607	HW1	SOL	3828	43.970	53.170	20.650	1.00	0.00
ATOM	12608	HW2	SOL	3828	43.320	52.220	21.640	1.00	0.00
ATOM	12609	OW	SOL	3829	55.890	16.930	1.620	1.00	0.00
ATOM	12610	HW1	SOL	3829	56.220	16.060	1.390	1.00	0.00
ATOM	12611	HW2	SOL	3829	54.940	16.800	1.730	1.00	0.00
ATOM	12612	OW	SOL	3830	37.440	46.510	40.770	1.00	0.00
ATOM	12613	HW1	SOL	3830	37.500	45.590	40.490	1.00	0.00
ATOM	12614	HW2	SOL	3830	38.120	46.960	40.260	1.00	0.00
ATOM	12615	OW	SOL	3831	0.560	13.870	52.280	1.00	0.00
ATOM	12616	HW1	SOL	3831	0.720	12.970	51.990	1.00	0.00
ATOM	12617	HW2	SOL	3831	0.940	14.410	51.580	1.00	0.00
ATOM	12618	OW	SOL	3832	47.210	50.520	21.650	1.00	0.00
ATOM	12619	HW1	SOL	3832	47.450	50.330	22.550	1.00	0.00
ATOM	12620	HW2	SOL	3832	47.380	49.710	21.180	1.00	0.00
ATOM	12621	OW	SOL	3833	29.520	45.770	8.290	1.00	0.00
ATOM	12622	HW1	SOL	3833	30.110	45.050	8.520	1.00	0.00
ATOM	12623	HW2	SOL	3833	30.020	46.310	7.690	1.00	0.00
ATOM	12624	OW	SOL	3834	15.920	5.710	21.640	1.00	0.00
ATOM	12625	HW1	SOL	3834	16.310	4.850	21.800	1.00	0.00
ATOM	12626	HW2	SOL	3834	15.140	5.520	21.110	1.00	0.00
ATOM	12627	OW	SOL	3835	36.400	11.670	4.970	1.00	0.00

ATOM	12628	HW1	SOL	3835	35.770	11.030	5.310	1.00	0.00
ATOM	12629	HW2	SOL	3835	36.410	12.360	5.630	1.00	0.00
ATOM	12630	OW	SOL	3836	28.760	13.540	38.630	1.00	0.00
ATOM	12631	HW1	SOL	3836	28.280	13.920	39.370	1.00	0.00
ATOM	12632	HW2	SOL	3836	28.230	12.780	38.370	1.00	0.00
ATOM	12633	OW	SOL	3837	40.770	49.390	22.120	1.00	0.00
ATOM	12634	HW1	SOL	3837	39.890	49.100	22.320	1.00	0.00
ATOM	12635	HW2	SOL	3837	40.940	50.100	22.730	1.00	0.00
ATOM	12636	OW	SOL	3838	11.400	45.770	36.320	1.00	0.00
ATOM	12637	HW1	SOL	3838	10.840	45.810	37.100	1.00	0.00
ATOM	12638	HW2	SOL	3838	11.630	44.840	36.240	1.00	0.00
ATOM	12639	OW	SOL	3839	45.130	2.130	30.230	1.00	0.00
ATOM	12640	HW1	SOL	3839	46.030	2.430	30.070	1.00	0.00
ATOM	12641	HW2	SOL	3839	44.670	2.920	30.510	1.00	0.00
ATOM	12642	OW	SOL	3840	51.820	30.020	14.660	1.00	0.00
ATOM	12643	HW1	SOL	3840	51.450	30.900	14.590	1.00	0.00
ATOM	12644	HW2	SOL	3840	52.730	30.160	14.940	1.00	0.00
ATOM	12645	OW	SOL	3841	41.550	15.880	15.520	1.00	0.00
ATOM	12646	HW1	SOL	3841	40.680	15.640	15.850	1.00	0.00
ATOM	12647	HW2	SOL	3841	42.010	15.050	15.430	1.00	0.00
ATOM	12648	OW	SOL	3842	40.070	54.870	8.120	1.00	0.00
ATOM	12649	HW1	SOL	3842	39.340	55.020	7.530	1.00	0.00
ATOM	12650	HW2	SOL	3842	39.850	54.050	8.570	1.00	0.00
ATOM	12651	OW	SOL	3843	33.850	31.290	16.770	1.00	0.00
ATOM	12652	HW1	SOL	3843	34.490	30.600	16.950	1.00	0.00
ATOM	12653	HW2	SOL	3843	34.060	31.970	17.420	1.00	0.00
ATOM	12654	OW	SOL	3844	40.970	6.740	43.870	1.00	0.00
ATOM	12655	HW1	SOL	3844	41.500	7.480	43.550	1.00	0.00
ATOM	12656	HW2	SOL	3844	40.310	7.150	44.430	1.00	0.00
ATOM	12657	OW	SOL	3845	47.710	11.490	14.340	1.00	0.00
ATOM	12658	HW1	SOL	3845	47.640	12.200	14.980	1.00	0.00
ATOM	12659	HW2	SOL	3845	47.910	11.940	13.510	1.00	0.00
ATOM	12660	OW	SOL	3846	26.320	36.390	27.260	1.00	0.00
ATOM	12661	HW1	SOL	3846	25.950	37.150	26.820	1.00	0.00
ATOM	12662	HW2	SOL	3846	26.090	35.640	26.700	1.00	0.00
ATOM	12663	OW	SOL	3847	3.450	15.850	14.620	1.00	0.00
ATOM	12664	HW1	SOL	3847	3.690	15.030	15.040	1.00	0.00
ATOM	12665	HW2	SOL	3847	3.070	16.380	15.320	1.00	0.00
ATOM	12666	OW	SOL	3848	43.280	12.880	4.080	1.00	0.00
ATOM	12667	HW1	SOL	3848	43.750	12.850	3.250	1.00	0.00
ATOM	12668	HW2	SOL	3848	43.950	13.070	4.740	1.00	0.00
ATOM	12669	OW	SOL	3849	17.380	20.810	4.020	1.00	0.00
ATOM	12670	HW1	SOL	3849	16.460	20.880	4.260	1.00	0.00
ATOM	12671	HW2	SOL	3849	17.670	19.980	4.400	1.00	0.00

ATOM	12672	OW	SOL	3850	45.500	24.860	50.840	1.00	0.00
ATOM	12673	HW1	SOL	3850	46.410	24.990	51.080	1.00	0.00
ATOM	12674	HW2	SOL	3850	45.010	25.470	51.400	1.00	0.00
ATOM	12675	OW	SOL	3851	52.900	31.730	32.090	1.00	0.00
ATOM	12676	HW1	SOL	3851	52.960	32.220	31.270	1.00	0.00
ATOM	12677	HW2	SOL	3851	53.030	30.820	31.840	1.00	0.00
ATOM	12678	OW	SOL	3852	19.180	3.300	27.270	1.00	0.00
ATOM	12679	HW1	SOL	3852	19.840	3.540	27.920	1.00	0.00
ATOM	12680	HW2	SOL	3852	19.650	2.750	26.640	1.00	0.00
ATOM	12681	OW	SOL	3853	43.100	38.980	12.440	1.00	0.00
ATOM	12682	HW1	SOL	3853	42.140	39.010	12.490	1.00	0.00
ATOM	12683	HW2	SOL	3853	43.290	38.080	12.170	1.00	0.00
ATOM	12684	OW	SOL	3854	42.750	7.180	17.020	1.00	0.00
ATOM	12685	HW1	SOL	3854	42.500	7.160	16.090	1.00	0.00
ATOM	12686	HW2	SOL	3854	43.160	6.330	17.180	1.00	0.00
ATOM	12687	OW	SOL	3855	38.370	48.260	19.600	1.00	0.00
ATOM	12688	HW1	SOL	3855	38.900	48.840	19.060	1.00	0.00
ATOM	12689	HW2	SOL	3855	38.190	48.770	20.390	1.00	0.00
ATOM	12690	OW	SOL	3856	25.610	41.060	26.870	1.00	0.00
ATOM	12691	HW1	SOL	3856	25.510	41.800	26.270	1.00	0.00
ATOM	12692	HW2	SOL	3856	26.520	40.790	26.770	1.00	0.00
ATOM	12693	OW	SOL	3857	30.290	2.620	45.990	1.00	0.00
ATOM	12694	HW1	SOL	3857	30.810	2.300	45.250	1.00	0.00
ATOM	12695	HW2	SOL	3857	30.060	3.520	45.750	1.00	0.00
ATOM	12696	OW	SOL	3858	3.210	12.340	13.330	1.00	0.00
ATOM	12697	HW1	SOL	3858	3.480	13.090	13.870	1.00	0.00
ATOM	12698	HW2	SOL	3858	3.270	12.670	12.430	1.00	0.00
ATOM	12699	OW	SOL	3859	55.540	15.330	8.530	1.00	0.00
ATOM	12700	HW1	SOL	3859	56.130	16.080	8.490	1.00	0.00
ATOM	12701	HW2	SOL	3859	55.570	14.950	7.650	1.00	0.00
ATOM	12702	OW	SOL	3860	45.040	35.560	54.050	1.00	0.00
ATOM	12703	HW1	SOL	3860	44.620	35.430	54.900	1.00	0.00
ATOM	12704	HW2	SOL	3860	45.790	34.960	54.060	1.00	0.00
ATOM	12705	OW	SOL	3861	43.160	2.180	23.590	1.00	0.00
ATOM	12706	HW1	SOL	3861	43.760	1.570	23.180	1.00	0.00
ATOM	12707	HW2	SOL	3861	43.450	3.040	23.300	1.00	0.00
ATOM	12708	OW	SOL	3862	34.270	1.150	20.210	1.00	0.00
ATOM	12709	HW1	SOL	3862	35.130	0.770	20.020	1.00	0.00
ATOM	12710	HW2	SOL	3862	34.470	2.030	20.540	1.00	0.00
ATOM	12711	OW	SOL	3863	23.260	19.780	27.330	1.00	0.00
ATOM	12712	HW1	SOL	3863	23.830	19.980	28.070	1.00	0.00
ATOM	12713	HW2	SOL	3863	22.440	20.220	27.540	1.00	0.00
ATOM	12714	OW	SOL	3864	41.900	39.420	8.250	1.00	0.00
ATOM	12715	HW1	SOL	3864	42.730	39.450	8.730	1.00	0.00

ATOM	12716	HW2	SOL	3864	41.500	38.590	8.500	1.00	0.00
ATOM	12717	OW	SOL	3865	30.930	44.720	4.760	1.00	0.00
ATOM	12718	HW1	SOL	3865	30.370	45.010	4.050	1.00	0.00
ATOM	12719	HW2	SOL	3865	31.480	44.040	4.380	1.00	0.00
ATOM	12720	OW	SOL	3866	19.190	27.750	31.300	1.00	0.00
ATOM	12721	HW1	SOL	3866	20.120	27.700	31.520	1.00	0.00
ATOM	12722	HW2	SOL	3866	19.170	27.650	30.350	1.00	0.00
ATOM	12723	OW	SOL	3867	20.050	6.800	55.830	1.00	0.00
ATOM	12724	HW1	SOL	3867	20.130	7.730	56.030	1.00	0.00
ATOM	12725	HW2	SOL	3867	20.910	6.550	55.510	1.00	0.00
ATOM	12726	OW	SOL	3868	34.310	10.650	17.560	1.00	0.00
ATOM	12727	HW1	SOL	3868	33.600	11.240	17.320	1.00	0.00
ATOM	12728	HW2	SOL	3868	34.870	11.160	18.150	1.00	0.00
ATOM	12729	OW	SOL	3869	2.000	23.430	46.410	1.00	0.00
ATOM	12730	HW1	SOL	3869	1.550	24.170	46.820	1.00	0.00
ATOM	12731	HW2	SOL	3869	2.330	23.780	45.580	1.00	0.00
ATOM	12732	OW	SOL	3870	45.980	30.060	53.400	1.00	0.00
ATOM	12733	HW1	SOL	3870	46.390	29.560	52.690	1.00	0.00
ATOM	12734	HW2	SOL	3870	46.550	29.910	54.160	1.00	0.00
ATOM	12735	OW	SOL	3871	38.960	53.610	12.880	1.00	0.00
ATOM	12736	HW1	SOL	3871	38.390	54.260	13.310	1.00	0.00
ATOM	12737	HW2	SOL	3871	38.730	53.680	11.950	1.00	0.00
ATOM	12738	OW	SOL	3872	8.650	29.910	3.710	1.00	0.00
ATOM	12739	HW1	SOL	3872	7.870	29.770	3.180	1.00	0.00
ATOM	12740	HW2	SOL	3872	8.740	30.860	3.760	1.00	0.00
ATOM	12741	OW	SOL	3873	15.990	14.280	18.090	1.00	0.00
ATOM	12742	HW1	SOL	3873	15.150	13.830	18.070	1.00	0.00
ATOM	12743	HW2	SOL	3873	16.480	13.830	18.780	1.00	0.00
ATOM	12744	OW	SOL	3874	23.310	34.650	24.220	1.00	0.00
ATOM	12745	HW1	SOL	3874	23.230	35.600	24.300	1.00	0.00
ATOM	12746	HW2	SOL	3874	22.410	34.330	24.290	1.00	0.00
ATOM	12747	OW	SOL	3875	14.860	4.400	1.580	1.00	0.00
ATOM	12748	HW1	SOL	3875	15.390	4.890	0.950	1.00	0.00
ATOM	12749	HW2	SOL	3875	14.850	4.950	2.360	1.00	0.00
ATOM	12750	OW	SOL	3876	5.290	45.960	10.320	1.00	0.00
ATOM	12751	HW1	SOL	3876	6.220	46.040	10.100	1.00	0.00
ATOM	12752	HW2	SOL	3876	5.260	46.100	11.270	1.00	0.00
ATOM	12753	OW	SOL	3877	15.270	25.880	49.460	1.00	0.00
ATOM	12754	HW1	SOL	3877	14.400	26.170	49.720	1.00	0.00
ATOM	12755	HW2	SOL	3877	15.150	25.490	48.590	1.00	0.00
ATOM	12756	OW	SOL	3878	2.900	42.990	29.510	1.00	0.00
ATOM	12757	HW1	SOL	3878	2.250	43.300	28.870	1.00	0.00
ATOM	12758	HW2	SOL	3878	2.650	43.430	30.330	1.00	0.00
ATOM	12759	OW	SOL	3879	22.240	26.710	15.090	1.00	0.00

ATOM	12760	HW1	SOL	3879	21.320	26.710	15.360	1.00	0.00
ATOM	12761	HW2	SOL	3879	22.290	26.010	14.430	1.00	0.00
ATOM	12762	OW	SOL	3880	52.800	10.690	55.650	1.00	0.00
ATOM	12763	HW1	SOL	3880	52.950	10.280	54.790	1.00	0.00
ATOM	12764	HW2	SOL	3880	52.660	9.950	56.240	1.00	0.00
ATOM	12765	OW	SOL	3881	26.830	16.590	39.650	1.00	0.00
ATOM	12766	HW1	SOL	3881	26.550	17.090	38.880	1.00	0.00
ATOM	12767	HW2	SOL	3881	26.010	16.400	40.110	1.00	0.00
ATOM	12768	OW	SOL	3882	41.060	35.950	23.390	1.00	0.00
ATOM	12769	HW1	SOL	3882	40.210	35.820	22.970	1.00	0.00
ATOM	12770	HW2	SOL	3882	41.700	35.610	22.760	1.00	0.00
ATOM	12771	OW	SOL	3883	8.570	36.410	42.910	1.00	0.00
ATOM	12772	HW1	SOL	3883	7.730	35.980	42.780	1.00	0.00
ATOM	12773	HW2	SOL	3883	8.560	36.670	43.830	1.00	0.00
ATOM	12774	OW	SOL	3884	3.330	4.200	6.160	1.00	0.00
ATOM	12775	HW1	SOL	3884	4.220	3.930	6.410	1.00	0.00
ATOM	12776	HW2	SOL	3884	2.810	4.060	6.950	1.00	0.00
ATOM	12777	OW	SOL	3885	22.030	31.230	45.300	1.00	0.00
ATOM	12778	HW1	SOL	3885	22.350	30.420	44.920	1.00	0.00
ATOM	12779	HW2	SOL	3885	21.080	31.160	45.270	1.00	0.00
ATOM	12780	OW	SOL	3886	53.670	0.600	33.020	1.00	0.00
ATOM	12781	HW1	SOL	3886	53.080	0.480	33.770	1.00	0.00
ATOM	12782	HW2	SOL	3886	53.700	-0.260	32.600	1.00	0.00
ATOM	12783	OW	SOL	3887	25.880	31.600	45.710	1.00	0.00
ATOM	12784	HW1	SOL	3887	26.660	31.340	46.210	1.00	0.00
ATOM	12785	HW2	SOL	3887	25.170	31.550	46.350	1.00	0.00
ATOM	12786	OW	SOL	3888	39.810	30.610	5.930	1.00	0.00
ATOM	12787	HW1	SOL	3888	39.750	29.680	6.120	1.00	0.00
ATOM	12788	HW2	SOL	3888	39.130	31.010	6.470	1.00	0.00
ATOM	12789	OW	SOL	3889	12.990	36.500	36.300	1.00	0.00
ATOM	12790	HW1	SOL	3889	12.220	35.930	36.300	1.00	0.00
ATOM	12791	HW2	SOL	3889	13.720	35.920	36.530	1.00	0.00
ATOM	12792	OW	SOL	3890	41.500	6.700	31.980	1.00	0.00
ATOM	12793	HW1	SOL	3890	41.370	5.810	31.670	1.00	0.00
ATOM	12794	HW2	SOL	3890	42.060	7.100	31.310	1.00	0.00
ATOM	12795	OW	SOL	3891	24.760	49.940	38.020	1.00	0.00
ATOM	12796	HW1	SOL	3891	25.620	49.920	38.430	1.00	0.00
ATOM	12797	HW2	SOL	3891	24.690	49.110	37.550	1.00	0.00
ATOM	12798	OW	SOL	3892	38.510	12.510	53.170	1.00	0.00
ATOM	12799	HW1	SOL	3892	38.740	12.770	52.280	1.00	0.00
ATOM	12800	HW2	SOL	3892	37.580	12.260	53.110	1.00	0.00
ATOM	12801	OW	SOL	3893	44.190	35.010	15.830	1.00	0.00
ATOM	12802	HW1	SOL	3893	43.850	34.170	15.530	1.00	0.00
ATOM	12803	HW2	SOL	3893	43.770	35.150	16.680	1.00	0.00

ATOM	12804	OW	SOL	3894	48.870	41.180	16.840	1.00	0.00
ATOM	12805	HW1	SOL	3894	48.410	41.370	17.660	1.00	0.00
ATOM	12806	HW2	SOL	3894	48.660	40.270	16.650	1.00	0.00
ATOM	12807	OW	SOL	3895	44.870	20.410	46.010	1.00	0.00
ATOM	12808	HW1	SOL	3895	45.560	20.550	45.370	1.00	0.00
ATOM	12809	HW2	SOL	3895	45.150	20.900	46.790	1.00	0.00
ATOM	12810	OW	SOL	3896	6.330	17.360	20.610	1.00	0.00
ATOM	12811	HW1	SOL	3896	7.120	17.630	20.130	1.00	0.00
ATOM	12812	HW2	SOL	3896	5.840	16.840	19.970	1.00	0.00
ATOM	12813	OW	SOL	3897	17.530	12.640	8.570	1.00	0.00
ATOM	12814	HW1	SOL	3897	17.770	11.960	9.200	1.00	0.00
ATOM	12815	HW2	SOL	3897	17.790	13.460	8.980	1.00	0.00
ATOM	12816	OW	SOL	3898	11.730	24.730	45.010	1.00	0.00
ATOM	12817	HW1	SOL	3898	12.500	25.280	44.840	1.00	0.00
ATOM	12818	HW2	SOL	3898	10.990	25.340	44.990	1.00	0.00
ATOM	12819	OW	SOL	3899	2.570	51.420	34.840	1.00	0.00
ATOM	12820	HW1	SOL	3899	1.760	51.600	34.370	1.00	0.00
ATOM	12821	HW2	SOL	3899	3.240	51.930	34.380	1.00	0.00
ATOM	12822	OW	SOL	3900	15.150	34.220	22.950	1.00	0.00
ATOM	12823	HW1	SOL	3900	15.080	33.690	22.160	1.00	0.00
ATOM	12824	HW2	SOL	3900	16.100	34.340	23.070	1.00	0.00
ATOM	12825	OW	SOL	3901	47.120	22.690	52.780	1.00	0.00
ATOM	12826	HW1	SOL	3901	47.200	23.640	52.670	1.00	0.00
ATOM	12827	HW2	SOL	3901	46.290	22.580	53.240	1.00	0.00
ATOM	12828	OW	SOL	3902	42.100	37.030	0.680	1.00	0.00
ATOM	12829	HW1	SOL	3902	42.210	36.080	0.790	1.00	0.00
ATOM	12830	HW2	SOL	3902	41.290	37.110	0.160	1.00	0.00
ATOM	12831	OW	SOL	3903	5.420	16.300	5.910	1.00	0.00
ATOM	12832	HW1	SOL	3903	5.000	15.460	5.750	1.00	0.00
ATOM	12833	HW2	SOL	3903	5.150	16.850	5.170	1.00	0.00
ATOM	12834	OW	SOL	3904	1.390	30.980	4.000	1.00	0.00
ATOM	12835	HW1	SOL	3904	1.750	30.260	3.480	1.00	0.00
ATOM	12836	HW2	SOL	3904	1.200	31.660	3.350	1.00	0.00
ATOM	12837	OW	SOL	3905	54.040	55.250	8.710	1.00	0.00
ATOM	12838	HW1	SOL	3905	53.490	54.810	8.060	1.00	0.00
ATOM	12839	HW2	SOL	3905	53.570	55.140	9.540	1.00	0.00
ATOM	12840	OW	SOL	3906	13.350	16.970	52.620	1.00	0.00
ATOM	12841	HW1	SOL	3906	13.520	16.160	53.100	1.00	0.00
ATOM	12842	HW2	SOL	3906	12.740	16.720	51.930	1.00	0.00
ATOM	12843	OW	SOL	3907	33.480	3.190	6.050	1.00	0.00
ATOM	12844	HW1	SOL	3907	33.760	3.930	5.500	1.00	0.00
ATOM	12845	HW2	SOL	3907	32.870	3.580	6.680	1.00	0.00
ATOM	12846	OW	SOL	3908	16.920	11.160	4.380	1.00	0.00
ATOM	12847	HW1	SOL	3908	16.840	10.490	5.060	1.00	0.00

ATOM	12848	HW2	SOL	3908	17.470	10.750	3.700	1.00	0.00
ATOM	12849	OW	SOL	3909	4.660	52.280	33.190	1.00	0.00
ATOM	12850	HW1	SOL	3909	5.600	52.430	33.240	1.00	0.00
ATOM	12851	HW2	SOL	3909	4.570	51.470	32.690	1.00	0.00
ATOM	12852	OW	SOL	3910	10.800	55.030	5.120	1.00	0.00
ATOM	12853	HW1	SOL	3910	10.720	54.100	4.890	1.00	0.00
ATOM	12854	HW2	SOL	3910	10.630	55.050	6.060	1.00	0.00
ATOM	12855	OW	SOL	3911	15.080	23.230	5.590	1.00	0.00
ATOM	12856	HW1	SOL	3911	15.200	22.560	4.920	1.00	0.00
ATOM	12857	HW2	SOL	3911	15.920	23.700	5.610	1.00	0.00
ATOM	12858	OW	SOL	3912	52.960	43.890	40.840	1.00	0.00
ATOM	12859	HW1	SOL	3912	53.400	43.060	40.680	1.00	0.00
ATOM	12860	HW2	SOL	3912	53.680	44.510	41.000	1.00	0.00
ATOM	12861	OW	SOL	3913	10.520	46.780	30.330	1.00	0.00
ATOM	12862	HW1	SOL	3913	10.530	47.050	31.250	1.00	0.00
ATOM	12863	HW2	SOL	3913	11.320	46.270	30.220	1.00	0.00
ATOM	12864	OW	SOL	3914	49.790	44.460	5.640	1.00	0.00
ATOM	12865	HW1	SOL	3914	49.620	44.420	4.700	1.00	0.00
ATOM	12866	HW2	SOL	3914	50.090	43.580	5.870	1.00	0.00
ATOM	12867	OW	SOL	3915	4.840	47.490	40.960	1.00	0.00
ATOM	12868	HW1	SOL	3915	4.340	47.060	41.650	1.00	0.00
ATOM	12869	HW2	SOL	3915	4.440	47.190	40.140	1.00	0.00
ATOM	12870	OW	SOL	3916	38.220	47.330	12.800	1.00	0.00
ATOM	12871	HW1	SOL	3916	39.160	47.210	12.640	1.00	0.00
ATOM	12872	HW2	SOL	3916	37.820	47.260	11.940	1.00	0.00
ATOM	12873	OW	SOL	3917	18.490	14.180	54.840	1.00	0.00
ATOM	12874	HW1	SOL	3917	18.690	15.030	55.240	1.00	0.00
ATOM	12875	HW2	SOL	3917	19.110	13.570	55.250	1.00	0.00
ATOM	12876	OW	SOL	3918	45.410	5.190	26.760	1.00	0.00
ATOM	12877	HW1	SOL	3918	45.260	6.030	27.190	1.00	0.00
ATOM	12878	HW2	SOL	3918	46.350	5.170	26.590	1.00	0.00
ATOM	12879	OW	SOL	3919	55.510	27.220	11.350	1.00	0.00
ATOM	12880	HW1	SOL	3919	55.620	26.960	12.270	1.00	0.00
ATOM	12881	HW2	SOL	3919	56.390	27.390	11.040	1.00	0.00
ATOM	12882	OW	SOL	3920	55.000	4.340	51.240	1.00	0.00
ATOM	12883	HW1	SOL	3920	54.570	3.960	52.010	1.00	0.00
ATOM	12884	HW2	SOL	3920	55.910	4.020	51.300	1.00	0.00
ATOM	12885	OW	SOL	3921	41.690	2.840	52.420	1.00	0.00
ATOM	12886	HW1	SOL	3921	41.290	3.610	52.820	1.00	0.00
ATOM	12887	HW2	SOL	3921	42.130	2.400	53.150	1.00	0.00
ATOM	12888	OW	SOL	3922	49.960	27.880	48.910	1.00	0.00
ATOM	12889	HW1	SOL	3922	50.860	27.820	49.230	1.00	0.00
ATOM	12890	HW2	SOL	3922	50.040	28.310	48.060	1.00	0.00
ATOM	12891	OW	SOL	3923	40.370	46.050	11.300	1.00	0.00

ATOM	12892	HW1	SOL	3923	40.580	45.130	11.440	1.00	0.00
ATOM	12893	HW2	SOL	3923	40.570	46.210	10.380	1.00	0.00
ATOM	12894	OW	SOL	3924	8.410	46.070	3.230	1.00	0.00
ATOM	12895	HW1	SOL	3924	8.780	46.120	2.340	1.00	0.00
ATOM	12896	HW2	SOL	3924	7.500	45.800	3.090	1.00	0.00
ATOM	12897	OW	SOL	3925	4.640	1.280	11.700	1.00	0.00
ATOM	12898	HW1	SOL	3925	3.730	1.540	11.840	1.00	0.00
ATOM	12899	HW2	SOL	3925	4.650	0.900	10.830	1.00	0.00
ATOM	12900	OW	SOL	3926	16.520	8.280	21.960	1.00	0.00
ATOM	12901	HW1	SOL	3926	15.600	8.530	21.890	1.00	0.00
ATOM	12902	HW2	SOL	3926	16.510	7.320	21.900	1.00	0.00
ATOM	12903	OW	SOL	3927	46.510	46.700	24.670	1.00	0.00
ATOM	12904	HW1	SOL	3927	45.890	47.410	24.860	1.00	0.00
ATOM	12905	HW2	SOL	3927	47.120	46.710	25.410	1.00	0.00
ATOM	12906	OW	SOL	3928	2.750	18.780	33.780	1.00	0.00
ATOM	12907	HW1	SOL	3928	2.540	19.630	33.400	1.00	0.00
ATOM	12908	HW2	SOL	3928	2.520	18.150	33.110	1.00	0.00
ATOM	12909	OW	SOL	3929	19.230	2.470	38.320	1.00	0.00
ATOM	12910	HW1	SOL	3929	19.240	2.810	37.430	1.00	0.00
ATOM	12911	HW2	SOL	3929	20.150	2.270	38.510	1.00	0.00
ATOM	12912	OW	SOL	3930	16.060	23.270	10.030	1.00	0.00
ATOM	12913	HW1	SOL	3930	16.850	23.000	9.560	1.00	0.00
ATOM	12914	HW2	SOL	3930	15.540	22.470	10.100	1.00	0.00
ATOM	12915	OW	SOL	3931	40.340	51.440	51.560	1.00	0.00
ATOM	12916	HW1	SOL	3931	40.110	51.670	50.660	1.00	0.00
ATOM	12917	HW2	SOL	3931	40.670	52.260	51.930	1.00	0.00
ATOM	12918	OW	SOL	3932	49.890	25.140	16.910	1.00	0.00
ATOM	12919	HW1	SOL	3932	49.890	24.400	17.520	1.00	0.00
ATOM	12920	HW2	SOL	3932	49.000	25.470	16.930	1.00	0.00
ATOM	12921	OW	SOL	3933	18.550	18.160	4.190	1.00	0.00
ATOM	12922	HW1	SOL	3933	17.740	18.110	4.690	1.00	0.00
ATOM	12923	HW2	SOL	3933	18.390	17.630	3.410	1.00	0.00
ATOM	12924	OW	SOL	3934	16.870	52.230	4.160	1.00	0.00
ATOM	12925	HW1	SOL	3934	17.320	52.100	4.990	1.00	0.00
ATOM	12926	HW2	SOL	3934	17.560	52.190	3.500	1.00	0.00
ATOM	12927	OW	SOL	3935	53.350	2.470	45.440	1.00	0.00
ATOM	12928	HW1	SOL	3935	53.610	3.380	45.290	1.00	0.00
ATOM	12929	HW2	SOL	3935	54.050	1.950	45.040	1.00	0.00
ATOM	12930	OW	SOL	3936	53.380	54.310	24.050	1.00	0.00
ATOM	12931	HW1	SOL	3936	53.220	54.070	23.140	1.00	0.00
ATOM	12932	HW2	SOL	3936	53.220	53.500	24.540	1.00	0.00
ATOM	12933	OW	SOL	3937	11.730	55.220	11.770	1.00	0.00
ATOM	12934	HW1	SOL	3937	11.910	54.960	10.870	1.00	0.00
ATOM	12935	HW2	SOL	3937	10.810	55.490	11.750	1.00	0.00

ATOM	12936	OW	SOL	3938	44.690	30.870	3.350	1.00	0.00
ATOM	12937	HW1	SOL	3938	44.880	31.160	2.450	1.00	0.00
ATOM	12938	HW2	SOL	3938	43.750	31.010	3.450	1.00	0.00
ATOM	12939	OW	SOL	3939	28.990	5.390	27.250	1.00	0.00
ATOM	12940	HW1	SOL	3939	28.970	4.500	27.620	1.00	0.00
ATOM	12941	HW2	SOL	3939	29.650	5.340	26.550	1.00	0.00
ATOM	12942	OW	SOL	3940	47.400	9.490	39.260	1.00	0.00
ATOM	12943	HW1	SOL	3940	47.210	8.890	38.540	1.00	0.00
ATOM	12944	HW2	SOL	3940	47.740	8.930	39.960	1.00	0.00
ATOM	12945	OW	SOL	3941	31.000	44.930	52.910	1.00	0.00
ATOM	12946	HW1	SOL	3941	30.100	45.210	52.720	1.00	0.00
ATOM	12947	HW2	SOL	3941	31.430	44.920	52.060	1.00	0.00
ATOM	12948	OW	SOL	3942	54.180	37.920	53.490	1.00	0.00
ATOM	12949	HW1	SOL	3942	54.070	37.570	52.610	1.00	0.00
ATOM	12950	HW2	SOL	3942	53.290	38.040	53.820	1.00	0.00
ATOM	12951	OW	SOL	3943	2.540	0.320	21.650	1.00	0.00
ATOM	12952	HW1	SOL	3943	2.490	1.250	21.870	1.00	0.00
ATOM	12953	HW2	SOL	3943	1.920	-0.100	22.240	1.00	0.00
ATOM	12954	OW	SOL	3944	1.410	15.710	50.550	1.00	0.00
ATOM	12955	HW1	SOL	3944	1.100	16.580	50.810	1.00	0.00
ATOM	12956	HW2	SOL	3944	1.900	15.860	49.750	1.00	0.00
ATOM	12957	OW	SOL	3945	5.020	10.940	0.510	1.00	0.00
ATOM	12958	HW1	SOL	3945	5.590	10.200	0.750	1.00	0.00
ATOM	12959	HW2	SOL	3945	4.900	10.840	-0.430	1.00	0.00
ATOM	12960	OW	SOL	3946	1.760	10.180	20.780	1.00	0.00
ATOM	12961	HW1	SOL	3946	2.480	10.000	21.390	1.00	0.00
ATOM	12962	HW2	SOL	3946	1.980	11.020	20.380	1.00	0.00
ATOM	12963	OW	SOL	3947	6.510	29.030	52.640	1.00	0.00
ATOM	12964	HW1	SOL	3947	6.710	28.500	53.420	1.00	0.00
ATOM	12965	HW2	SOL	3947	7.180	28.770	52.000	1.00	0.00
ATOM	12966	OW	SOL	3948	13.140	50.280	18.600	1.00	0.00
ATOM	12967	HW1	SOL	3948	13.350	49.800	17.800	1.00	0.00
ATOM	12968	HW2	SOL	3948	13.550	51.140	18.480	1.00	0.00
ATOM	12969	OW	SOL	3949	53.400	42.850	1.880	1.00	0.00
ATOM	12970	HW1	SOL	3949	53.730	41.970	1.720	1.00	0.00
ATOM	12971	HW2	SOL	3949	54.140	43.430	1.680	1.00	0.00
ATOM	12972	OW	SOL	3950	41.160	28.330	9.350	1.00	0.00
ATOM	12973	HW1	SOL	3950	41.910	28.620	8.830	1.00	0.00
ATOM	12974	HW2	SOL	3950	40.960	27.450	9.000	1.00	0.00
ATOM	12975	OW	SOL	3951	33.520	51.630	11.120	1.00	0.00
ATOM	12976	HW1	SOL	3951	32.880	51.210	10.540	1.00	0.00
ATOM	12977	HW2	SOL	3951	33.000	51.970	11.850	1.00	0.00
ATOM	12978	OW	SOL	3952	16.930	37.130	28.490	1.00	0.00
ATOM	12979	HW1	SOL	3952	17.620	36.470	28.540	1.00	0.00

ATOM	12980	HW2	SOL	3952	17.300	37.840	27.970	1.00	0.00
ATOM	12981	OW	SOL	3953	15.920	3.180	24.920	1.00	0.00
ATOM	12982	HW1	SOL	3953	15.410	2.640	24.310	1.00	0.00
ATOM	12983	HW2	SOL	3953	15.490	4.030	24.900	1.00	0.00
ATOM	12984	OW	SOL	3954	44.320	1.550	4.600	1.00	0.00
ATOM	12985	HW1	SOL	3954	43.780	2.330	4.500	1.00	0.00
ATOM	12986	HW2	SOL	3954	45.140	1.770	4.150	1.00	0.00
ATOM	12987	OW	SOL	3955	39.990	3.830	54.800	1.00	0.00
ATOM	12988	HW1	SOL	3955	40.560	3.590	55.530	1.00	0.00
ATOM	12989	HW2	SOL	3955	39.160	3.390	54.990	1.00	0.00
ATOM	12990	OW	SOL	3956	32.930	35.830	0.880	1.00	0.00
ATOM	12991	HW1	SOL	3956	33.150	34.900	0.860	1.00	0.00
ATOM	12992	HW2	SOL	3956	32.230	35.900	1.530	1.00	0.00
ATOM	12993	OW	SOL	3957	33.750	17.850	37.210	1.00	0.00
ATOM	12994	HW1	SOL	3957	33.030	18.330	37.620	1.00	0.00
ATOM	12995	HW2	SOL	3957	34.470	17.900	37.830	1.00	0.00
ATOM	12996	OW	SOL	3958	2.290	40.980	35.220	1.00	0.00
ATOM	12997	HW1	SOL	3958	1.450	40.620	35.520	1.00	0.00
ATOM	12998	HW2	SOL	3958	2.630	41.460	35.980	1.00	0.00
ATOM	12999	OW	SOL	3959	2.140	22.190	11.350	1.00	0.00
ATOM	13000	HW1	SOL	3959	2.510	21.670	12.060	1.00	0.00
ATOM	13001	HW2	SOL	3959	1.400	21.660	11.030	1.00	0.00
ATOM	13002	OW	SOL	3960	2.170	28.250	3.220	1.00	0.00
ATOM	13003	HW1	SOL	3960	1.310	28.180	2.810	1.00	0.00
ATOM	13004	HW2	SOL	3960	2.080	27.790	4.050	1.00	0.00
ATOM	13005	OW	SOL	3961	22.390	4.730	7.740	1.00	0.00
ATOM	13006	HW1	SOL	3961	23.170	5.150	7.380	1.00	0.00
ATOM	13007	HW2	SOL	3961	21.920	5.440	8.190	1.00	0.00
ATOM	13008	OW	SOL	3962	33.550	31.490	31.470	1.00	0.00
ATOM	13009	HW1	SOL	3962	33.730	32.160	30.800	1.00	0.00
ATOM	13010	HW2	SOL	3962	32.650	31.210	31.280	1.00	0.00
ATOM	13011	OW	SOL	3963	20.200	6.640	28.390	1.00	0.00
ATOM	13012	HW1	SOL	3963	19.380	6.270	28.710	1.00	0.00
ATOM	13013	HW2	SOL	3963	20.360	7.390	28.960	1.00	0.00
ATOM	13014	OW	SOL	3964	15.750	34.150	32.310	1.00	0.00
ATOM	13015	HW1	SOL	3964	15.100	34.020	33.000	1.00	0.00
ATOM	13016	HW2	SOL	3964	15.840	33.290	31.900	1.00	0.00
ATOM	13017	OW	SOL	3965	35.490	45.660	47.580	1.00	0.00
ATOM	13018	HW1	SOL	3965	36.360	45.750	47.960	1.00	0.00
ATOM	13019	HW2	SOL	3965	34.920	46.170	48.170	1.00	0.00
ATOM	13020	OW	SOL	3966	7.910	19.290	4.180	1.00	0.00
ATOM	13021	HW1	SOL	3966	8.280	18.430	3.970	1.00	0.00
ATOM	13022	HW2	SOL	3966	7.210	19.420	3.540	1.00	0.00
ATOM	13023	OW	SOL	3967	27.400	26.150	24.860	1.00	0.00

ATOM	13024	HW1	SOL	3967	27.930	26.630	24.220	1.00	0.00
ATOM	13025	HW2	SOL	3967	26.770	25.660	24.330	1.00	0.00
ATOM	13026	OW	SOL	3968	17.630	3.650	21.750	1.00	0.00
ATOM	13027	HW1	SOL	3968	17.070	3.530	20.980	1.00	0.00
ATOM	13028	HW2	SOL	3968	18.510	3.770	21.390	1.00	0.00
ATOM	13029	OW	SOL	3969	45.610	8.060	27.370	1.00	0.00
ATOM	13030	HW1	SOL	3969	45.630	8.790	27.990	1.00	0.00
ATOM	13031	HW2	SOL	3969	46.270	7.450	27.690	1.00	0.00
ATOM	13032	OW	SOL	3970	24.910	10.190	18.850	1.00	0.00
ATOM	13033	HW1	SOL	3970	25.040	10.100	19.800	1.00	0.00
ATOM	13034	HW2	SOL	3970	23.990	10.420	18.750	1.00	0.00
ATOM	13035	OW	SOL	3971	5.480	29.660	21.110	1.00	0.00
ATOM	13036	HW1	SOL	3971	5.320	28.730	20.930	1.00	0.00
ATOM	13037	HW2	SOL	3971	4.680	30.100	20.810	1.00	0.00
ATOM	13038	OW	SOL	3972	43.370	27.220	5.950	1.00	0.00
ATOM	13039	HW1	SOL	3972	44.070	26.680	5.590	1.00	0.00
ATOM	13040	HW2	SOL	3972	42.710	27.250	5.260	1.00	0.00
ATOM	13041	OW	SOL	3973	24.120	27.140	29.170	1.00	0.00
ATOM	13042	HW1	SOL	3973	23.560	27.610	29.780	1.00	0.00
ATOM	13043	HW2	SOL	3973	24.330	27.780	28.490	1.00	0.00
ATOM	13044	OW	SOL	3974	16.540	32.840	18.740	1.00	0.00
ATOM	13045	HW1	SOL	3974	16.920	33.360	19.440	1.00	0.00
ATOM	13046	HW2	SOL	3974	16.770	33.310	17.940	1.00	0.00
ATOM	13047	OW	SOL	3975	42.460	50.300	13.150	1.00	0.00
ATOM	13048	HW1	SOL	3975	42.240	51.230	13.220	1.00	0.00
ATOM	13049	HW2	SOL	3975	42.000	49.900	13.890	1.00	0.00
ATOM	13050	OW	SOL	3976	37.090	24.470	1.500	1.00	0.00
ATOM	13051	HW1	SOL	3976	37.130	23.600	1.110	1.00	0.00
ATOM	13052	HW2	SOL	3976	37.980	24.810	1.430	1.00	0.00
ATOM	13053	OW	SOL	3977	34.940	15.360	7.800	1.00	0.00
ATOM	13054	HW1	SOL	3977	35.440	15.210	7.000	1.00	0.00
ATOM	13055	HW2	SOL	3977	35.590	15.320	8.500	1.00	0.00
ATOM	13056	OW	SOL	3978	40.680	18.290	51.790	1.00	0.00
ATOM	13057	HW1	SOL	3978	39.810	18.550	52.070	1.00	0.00
ATOM	13058	HW2	SOL	3978	40.530	17.560	51.180	1.00	0.00
ATOM	13059	OW	SOL	3979	1.270	2.950	52.530	1.00	0.00
ATOM	13060	HW1	SOL	3979	1.180	2.220	51.920	1.00	0.00
ATOM	13061	HW2	SOL	3979	1.460	2.540	53.380	1.00	0.00
ATOM	13062	OW	SOL	3980	6.640	7.610	53.110	1.00	0.00
ATOM	13063	HW1	SOL	3980	6.210	8.320	52.630	1.00	0.00
ATOM	13064	HW2	SOL	3980	7.570	7.740	52.960	1.00	0.00
ATOM	13065	OW	SOL	3981	43.500	29.040	8.260	1.00	0.00
ATOM	13066	HW1	SOL	3981	44.310	28.890	8.740	1.00	0.00
ATOM	13067	HW2	SOL	3981	43.570	28.480	7.490	1.00	0.00

ATOM	13068	OW	SOL	3982	43.740	10.990	14.320	1.00	0.00
ATOM	13069	HW1	SOL	3982	44.640	10.730	14.510	1.00	0.00
ATOM	13070	HW2	SOL	3982	43.720	11.110	13.370	1.00	0.00
ATOM	13071	OW	SOL	3983	39.340	10.850	42.810	1.00	0.00
ATOM	13072	HW1	SOL	3983	39.830	10.220	42.270	1.00	0.00
ATOM	13073	HW2	SOL	3983	38.510	10.420	42.990	1.00	0.00
ATOM	13074	OW	SOL	3984	2.900	6.830	38.530	1.00	0.00
ATOM	13075	HW1	SOL	3984	1.960	6.890	38.710	1.00	0.00
ATOM	13076	HW2	SOL	3984	2.950	6.440	37.650	1.00	0.00
ATOM	13077	OW	SOL	3985	51.250	10.710	3.220	1.00	0.00
ATOM	13078	HW1	SOL	3985	50.410	11.090	3.490	1.00	0.00
ATOM	13079	HW2	SOL	3985	51.660	11.410	2.710	1.00	0.00
ATOM	13080	OW	SOL	3986	17.480	48.270	50.840	1.00	0.00
ATOM	13081	HW1	SOL	3986	18.410	48.060	50.730	1.00	0.00
ATOM	13082	HW2	SOL	3986	17.130	47.550	51.360	1.00	0.00
ATOM	13083	OW	SOL	3987	0.460	8.100	38.250	1.00	0.00
ATOM	13084	HW1	SOL	3987	-0.230	8.080	37.600	1.00	0.00
ATOM	13085	HW2	SOL	3987	1.040	8.810	37.970	1.00	0.00
ATOM	13086	OW	SOL	3988	10.790	28.070	0.630	1.00	0.00
ATOM	13087	HW1	SOL	3988	9.930	27.940	1.050	1.00	0.00
ATOM	13088	HW2	SOL	3988	10.840	27.380	-0.030	1.00	0.00
ATOM	13089	OW	SOL	3989	46.020	51.790	38.850	1.00	0.00
ATOM	13090	HW1	SOL	3989	46.140	50.840	38.930	1.00	0.00
ATOM	13091	HW2	SOL	3989	46.330	52.140	39.680	1.00	0.00
ATOM	13092	OW	SOL	3990	0.000	42.140	30.990	1.00	0.00
ATOM	13093	HW1	SOL	3990	0.220	41.840	30.110	1.00	0.00
ATOM	13094	HW2	SOL	3990	0.370	41.470	31.570	1.00	0.00
ATOM	13095	OW	SOL	3991	41.710	15.230	32.660	1.00	0.00
ATOM	13096	HW1	SOL	3991	41.790	16.160	32.900	1.00	0.00
ATOM	13097	HW2	SOL	3991	40.970	15.210	32.050	1.00	0.00
ATOM	13098	OW	SOL	3992	39.850	30.740	48.290	1.00	0.00
ATOM	13099	HW1	SOL	3992	40.470	30.040	48.480	1.00	0.00
ATOM	13100	HW2	SOL	3992	40.170	31.490	48.790	1.00	0.00
ATOM	13101	OW	SOL	3993	33.200	41.320	43.910	1.00	0.00
ATOM	13102	HW1	SOL	3993	32.730	40.570	44.270	1.00	0.00
ATOM	13103	HW2	SOL	3993	33.370	41.880	44.670	1.00	0.00
ATOM	13104	OW	SOL	3994	13.850	11.410	15.810	1.00	0.00
ATOM	13105	HW1	SOL	3994	13.190	11.140	15.160	1.00	0.00
ATOM	13106	HW2	SOL	3994	14.510	10.720	15.770	1.00	0.00
ATOM	13107	OW	SOL	3995	47.030	44.400	47.890	1.00	0.00
ATOM	13108	HW1	SOL	3995	47.340	43.990	47.090	1.00	0.00
ATOM	13109	HW2	SOL	3995	46.500	43.730	48.320	1.00	0.00
ATOM	13110	OW	SOL	3996	21.210	15.330	11.070	1.00	0.00
ATOM	13111	HW1	SOL	3996	21.610	15.600	11.890	1.00	0.00

ATOM	13112	HW2	SOL	3996	21.950	15.180	10.480	1.00	0.00
ATOM	13113	OW	SOL	3997	55.490	32.620	11.710	1.00	0.00
ATOM	13114	HW1	SOL	3997	56.280	33.010	11.350	1.00	0.00
ATOM	13115	HW2	SOL	3997	55.460	31.740	11.340	1.00	0.00
ATOM	13116	OW	SOL	3998	32.170	12.130	16.570	1.00	0.00
ATOM	13117	HW1	SOL	3998	31.600	12.220	17.330	1.00	0.00
ATOM	13118	HW2	SOL	3998	32.030	12.920	16.060	1.00	0.00
ATOM	13119	OW	SOL	3999	16.390	18.210	35.650	1.00	0.00
ATOM	13120	HW1	SOL	3999	15.610	17.690	35.460	1.00	0.00
ATOM	13121	HW2	SOL	3999	16.590	18.650	34.830	1.00	0.00
ATOM	13122	OW	SOL	4000	6.890	39.500	11.150	1.00	0.00
ATOM	13123	HW1	SOL	4000	6.630	39.440	10.230	1.00	0.00
ATOM	13124	HW2	SOL	4000	6.830	38.600	11.470	1.00	0.00
ATOM	13125	OW	SOL	4001	26.990	52.450	14.590	1.00	0.00
ATOM	13126	HW1	SOL	4001	26.540	52.340	15.420	1.00	0.00
ATOM	13127	HW2	SOL	4001	26.940	51.590	14.170	1.00	0.00
ATOM	13128	OW	SOL	4002	33.020	44.970	0.750	1.00	0.00
ATOM	13129	HW1	SOL	4002	33.040	45.880	0.430	1.00	0.00
ATOM	13130	HW2	SOL	4002	33.220	44.440	-0.020	1.00	0.00
ATOM	13131	OW	SOL	4003	47.070	20.050	29.650	1.00	0.00
ATOM	13132	HW1	SOL	4003	47.800	19.430	29.690	1.00	0.00
ATOM	13133	HW2	SOL	4003	47.410	20.790	29.140	1.00	0.00
ATOM	13134	OW	SOL	4004	52.550	1.840	27.600	1.00	0.00
ATOM	13135	HW1	SOL	4004	52.990	2.510	27.080	1.00	0.00
ATOM	13136	HW2	SOL	4004	53.210	1.150	27.720	1.00	0.00
ATOM	13137	OW	SOL	4005	30.010	27.800	43.310	1.00	0.00
ATOM	13138	HW1	SOL	4005	30.240	28.720	43.130	1.00	0.00
ATOM	13139	HW2	SOL	4005	30.520	27.580	44.090	1.00	0.00
ATOM	13140	OW	SOL	4006	47.730	19.410	20.800	1.00	0.00
ATOM	13141	HW1	SOL	4006	46.910	18.930	20.690	1.00	0.00
ATOM	13142	HW2	SOL	4006	47.510	20.310	20.560	1.00	0.00
ATOM	13143	OW	SOL	4007	4.490	31.210	3.890	1.00	0.00
ATOM	13144	HW1	SOL	4007	4.930	30.370	4.020	1.00	0.00
ATOM	13145	HW2	SOL	4007	4.890	31.570	3.100	1.00	0.00
ATOM	13146	OW	SOL	4008	26.170	10.400	40.440	1.00	0.00
ATOM	13147	HW1	SOL	4008	26.460	9.590	40.010	1.00	0.00
ATOM	13148	HW2	SOL	4008	26.060	11.020	39.720	1.00	0.00
ATOM	13149	OW	SOL	4009	18.080	42.590	14.660	1.00	0.00
ATOM	13150	HW1	SOL	4009	17.140	42.570	14.830	1.00	0.00
ATOM	13151	HW2	SOL	4009	18.160	42.250	13.760	1.00	0.00
ATOM	13152	OW	SOL	4010	34.700	28.970	13.390	1.00	0.00
ATOM	13153	HW1	SOL	4010	33.800	29.250	13.260	1.00	0.00
ATOM	13154	HW2	SOL	4010	35.210	29.780	13.430	1.00	0.00
ATOM	13155	OW	SOL	4011	51.100	5.580	36.180	1.00	0.00

ATOM	13156	HW1	SOL	4011	51.250	4.650	36.350	1.00	0.00
ATOM	13157	HW2	SOL	4011	51.110	5.660	35.230	1.00	0.00
ATOM	13158	OW	SOL	4012	11.750	53.580	48.220	1.00	0.00
ATOM	13159	HW1	SOL	4012	10.870	53.240	48.050	1.00	0.00
ATOM	13160	HW2	SOL	4012	11.840	54.310	47.620	1.00	0.00
ATOM	13161	OW	SOL	4013	23.530	45.650	10.020	1.00	0.00
ATOM	13162	HW1	SOL	4013	22.990	45.250	9.340	1.00	0.00
ATOM	13163	HW2	SOL	4013	22.930	45.780	10.750	1.00	0.00
ATOM	13164	OW	SOL	4014	50.360	8.820	43.910	1.00	0.00
ATOM	13165	HW1	SOL	4014	50.870	8.030	44.120	1.00	0.00
ATOM	13166	HW2	SOL	4014	50.370	8.860	42.950	1.00	0.00
ATOM	13167	OW	SOL	4015	0.410	44.150	7.060	1.00	0.00
ATOM	13168	HW1	SOL	4015	1.070	43.490	6.850	1.00	0.00
ATOM	13169	HW2	SOL	4015	0.910	44.930	7.290	1.00	0.00
ATOM	13170	OW	SOL	4016	7.990	51.200	42.210	1.00	0.00
ATOM	13171	HW1	SOL	4016	7.540	51.580	42.960	1.00	0.00
ATOM	13172	HW2	SOL	4016	7.290	51.010	41.580	1.00	0.00
ATOM	13173	OW	SOL	4017	8.340	21.490	31.940	1.00	0.00
ATOM	13174	HW1	SOL	4017	7.430	21.750	31.800	1.00	0.00
ATOM	13175	HW2	SOL	4017	8.860	22.200	31.570	1.00	0.00
ATOM	13176	OW	SOL	4018	31.490	12.310	11.330	1.00	0.00
ATOM	13177	HW1	SOL	4018	31.640	11.360	11.290	1.00	0.00
ATOM	13178	HW2	SOL	4018	30.560	12.410	11.110	1.00	0.00
ATOM	13179	OW	SOL	4019	41.940	48.250	47.940	1.00	0.00
ATOM	13180	HW1	SOL	4019	41.810	49.030	47.390	1.00	0.00
ATOM	13181	HW2	SOL	4019	42.870	48.270	48.180	1.00	0.00
ATOM	13182	OW	SOL	4020	55.410	36.460	44.000	1.00	0.00
ATOM	13183	HW1	SOL	4020	55.810	35.650	44.300	1.00	0.00
ATOM	13184	HW2	SOL	4020	55.290	36.980	44.790	1.00	0.00
ATOM	13185	OW	SOL	4021	33.490	43.030	46.250	1.00	0.00
ATOM	13186	HW1	SOL	4021	34.250	43.540	46.530	1.00	0.00
ATOM	13187	HW2	SOL	4021	33.350	42.400	46.960	1.00	0.00
ATOM	13188	OW	SOL	4022	6.210	21.140	19.150	1.00	0.00
ATOM	13189	HW1	SOL	4022	6.590	21.510	18.360	1.00	0.00
ATOM	13190	HW2	SOL	4022	5.810	20.320	18.860	1.00	0.00
ATOM	13191	OW	SOL	4023	12.370	37.850	30.110	1.00	0.00
ATOM	13192	HW1	SOL	4023	11.770	37.330	29.580	1.00	0.00
ATOM	13193	HW2	SOL	4023	13.170	37.320	30.150	1.00	0.00
ATOM	13194	OW	SOL	4024	50.620	35.920	9.690	1.00	0.00
ATOM	13195	HW1	SOL	4024	49.730	35.560	9.770	1.00	0.00
ATOM	13196	HW2	SOL	4024	51.080	35.580	10.460	1.00	0.00
ATOM	13197	OW	SOL	4025	2.480	50.090	21.860	1.00	0.00
ATOM	13198	HW1	SOL	4025	2.270	50.030	22.790	1.00	0.00
ATOM	13199	HW2	SOL	4025	2.290	49.220	21.510	1.00	0.00

ATOM	13200	OW	SOL	4026	34.800	26.610	23.230	1.00	0.00
ATOM	13201	HW1	SOL	4026	34.660	27.530	23.020	1.00	0.00
ATOM	13202	HW2	SOL	4026	33.950	26.200	23.060	1.00	0.00
ATOM	13203	OW	SOL	4027	43.650	53.660	0.840	1.00	0.00
ATOM	13204	HW1	SOL	4027	43.450	53.460	-0.070	1.00	0.00
ATOM	13205	HW2	SOL	4027	44.600	53.800	0.850	1.00	0.00
ATOM	13206	OW	SOL	4028	50.030	14.620	7.730	1.00	0.00
ATOM	13207	HW1	SOL	4028	50.640	14.900	7.050	1.00	0.00
ATOM	13208	HW2	SOL	4028	50.580	14.210	8.400	1.00	0.00
ATOM	13209	OW	SOL	4029	11.270	5.650	25.240	1.00	0.00
ATOM	13210	HW1	SOL	4029	11.130	5.850	26.170	1.00	0.00
ATOM	13211	HW2	SOL	4029	10.630	6.190	24.780	1.00	0.00
ATOM	13212	OW	SOL	4030	19.710	28.100	40.490	1.00	0.00
ATOM	13213	HW1	SOL	4030	18.950	28.470	40.040	1.00	0.00
ATOM	13214	HW2	SOL	4030	19.500	28.180	41.420	1.00	0.00
ATOM	13215	OW	SOL	4031	1.720	8.650	42.010	1.00	0.00
ATOM	13216	HW1	SOL	4031	1.790	9.430	41.460	1.00	0.00
ATOM	13217	HW2	SOL	4031	2.480	8.700	42.590	1.00	0.00
ATOM	13218	OW	SOL	4032	44.560	53.500	17.850	1.00	0.00
ATOM	13219	HW1	SOL	4032	45.100	54.230	17.570	1.00	0.00
ATOM	13220	HW2	SOL	4032	45.150	52.950	18.370	1.00	0.00
ATOM	13221	OW	SOL	4033	27.530	48.290	21.030	1.00	0.00
ATOM	13222	HW1	SOL	4033	27.820	49.180	21.220	1.00	0.00
ATOM	13223	HW2	SOL	4033	27.080	48.360	20.190	1.00	0.00
ATOM	13224	OW	SOL	4034	55.170	34.420	3.630	1.00	0.00
ATOM	13225	HW1	SOL	4034	54.950	34.890	2.820	1.00	0.00
ATOM	13226	HW2	SOL	4034	54.650	34.860	4.310	1.00	0.00
ATOM	13227	OW	SOL	4035	10.530	23.400	48.670	1.00	0.00
ATOM	13228	HW1	SOL	4035	9.830	22.890	48.270	1.00	0.00
ATOM	13229	HW2	SOL	4035	11.280	22.800	48.680	1.00	0.00
ATOM	13230	OW	SOL	4036	0.440	13.750	24.080	1.00	0.00
ATOM	13231	HW1	SOL	4036	1.300	13.410	23.860	1.00	0.00
ATOM	13232	HW2	SOL	4036	0.450	14.660	23.770	1.00	0.00
ATOM	13233	OW	SOL	4037	55.690	37.450	40.510	1.00	0.00
ATOM	13234	HW1	SOL	4037	55.220	37.050	39.780	1.00	0.00
ATOM	13235	HW2	SOL	4037	56.610	37.250	40.350	1.00	0.00
ATOM	13236	OW	SOL	4038	33.290	24.690	2.980	1.00	0.00
ATOM	13237	HW1	SOL	4038	33.760	24.390	2.200	1.00	0.00
ATOM	13238	HW2	SOL	4038	33.850	25.380	3.350	1.00	0.00
ATOM	13239	OW	SOL	4039	14.610	44.860	12.770	1.00	0.00
ATOM	13240	HW1	SOL	4039	15.340	44.740	12.170	1.00	0.00
ATOM	13241	HW2	SOL	4039	14.670	45.780	13.030	1.00	0.00
ATOM	13242	OW	SOL	4040	27.090	54.380	36.660	1.00	0.00
ATOM	13243	HW1	SOL	4040	28.010	54.110	36.690	1.00	0.00

ATOM	13244	HW2	SOL	4040	26.600	53.590	36.900	1.00	0.00
ATOM	13245	OW	SOL	4041	0.570	19.930	11.340	1.00	0.00
ATOM	13246	HW1	SOL	4041	0.010	19.350	10.830	1.00	0.00
ATOM	13247	HW2	SOL	4041	1.450	19.560	11.240	1.00	0.00
ATOM	13248	OW	SOL	4042	17.400	1.120	23.190	1.00	0.00
ATOM	13249	HW1	SOL	4042	17.500	2.000	22.830	1.00	0.00
ATOM	13250	HW2	SOL	4042	16.480	1.050	23.420	1.00	0.00
ATOM	13251	OW	SOL	4043	44.210	24.640	16.610	1.00	0.00
ATOM	13252	HW1	SOL	4043	43.720	23.910	16.990	1.00	0.00
ATOM	13253	HW2	SOL	4043	44.480	24.330	15.750	1.00	0.00
ATOM	13254	OW	SOL	4044	19.150	49.140	43.150	1.00	0.00
ATOM	13255	HW1	SOL	4044	19.110	48.200	42.980	1.00	0.00
ATOM	13256	HW2	SOL	4044	20.000	49.410	42.810	1.00	0.00
ATOM	13257	OW	SOL	4045	11.570	5.920	36.810	1.00	0.00
ATOM	13258	HW1	SOL	4045	12.260	6.400	36.360	1.00	0.00
ATOM	13259	HW2	SOL	4045	11.510	6.340	37.670	1.00	0.00
ATOM	13260	OW	SOL	4046	12.280	26.030	27.030	1.00	0.00
ATOM	13261	HW1	SOL	4046	13.200	26.280	27.150	1.00	0.00
ATOM	13262	HW2	SOL	4046	12.080	26.290	26.130	1.00	0.00
ATOM	13263	OW	SOL	4047	43.740	24.450	13.640	1.00	0.00
ATOM	13264	HW1	SOL	4047	44.130	24.790	12.840	1.00	0.00
ATOM	13265	HW2	SOL	4047	42.910	24.050	13.360	1.00	0.00
ATOM	13266	OW	SOL	4048	11.870	30.250	9.370	1.00	0.00
ATOM	13267	HW1	SOL	4048	12.210	30.440	10.240	1.00	0.00
ATOM	13268	HW2	SOL	4048	12.120	29.340	9.200	1.00	0.00
ATOM	13269	OW	SOL	4049	36.170	24.420	24.520	1.00	0.00
ATOM	13270	HW1	SOL	4049	35.890	25.310	24.300	1.00	0.00
ATOM	13271	HW2	SOL	4049	37.100	24.490	24.710	1.00	0.00
ATOM	13272	OW	SOL	4050	5.450	10.920	49.710	1.00	0.00
ATOM	13273	HW1	SOL	4050	6.330	11.260	49.540	1.00	0.00
ATOM	13274	HW2	SOL	4050	5.560	9.980	49.780	1.00	0.00
ATOM	13275	OW	SOL	4051	38.540	46.360	53.940	1.00	0.00
ATOM	13276	HW1	SOL	4051	38.650	46.380	54.890	1.00	0.00
ATOM	13277	HW2	SOL	4051	38.750	47.240	53.650	1.00	0.00
ATOM	13278	OW	SOL	4052	23.570	46.870	27.490	1.00	0.00
ATOM	13279	HW1	SOL	4052	24.070	46.700	28.290	1.00	0.00
ATOM	13280	HW2	SOL	4052	22.660	46.770	27.740	1.00	0.00
ATOM	13281	OW	SOL	4053	10.380	49.210	42.510	1.00	0.00
ATOM	13282	HW1	SOL	4053	10.390	48.650	41.740	1.00	0.00
ATOM	13283	HW2	SOL	4053	9.740	49.890	42.310	1.00	0.00
ATOM	13284	OW	SOL	4054	35.190	5.790	25.780	1.00	0.00
ATOM	13285	HW1	SOL	4054	35.540	5.830	24.890	1.00	0.00
ATOM	13286	HW2	SOL	4054	35.460	4.930	26.110	1.00	0.00
ATOM	13287	OW	SOL	4055	50.710	11.450	40.030	1.00	0.00

ATOM	13288	HW1	SOL	4055	51.350	12.130	39.820	1.00	0.00
ATOM	13289	HW2	SOL	4055	51.250	10.670	40.210	1.00	0.00
ATOM	13290	OW	SOL	4056	1.600	36.630	8.720	1.00	0.00
ATOM	13291	HW1	SOL	4056	2.380	37.080	9.040	1.00	0.00
ATOM	13292	HW2	SOL	4056	1.310	37.140	7.970	1.00	0.00
ATOM	13293	OW	SOL	4057	52.240	13.250	55.350	1.00	0.00
ATOM	13294	HW1	SOL	4057	52.390	12.320	55.560	1.00	0.00
ATOM	13295	HW2	SOL	4057	52.320	13.300	54.400	1.00	0.00
ATOM	13296	OW	SOL	4058	44.180	9.410	49.790	1.00	0.00
ATOM	13297	HW1	SOL	4058	44.410	8.750	49.130	1.00	0.00
ATOM	13298	HW2	SOL	4058	45.010	9.850	49.980	1.00	0.00
ATOM	13299	OW	SOL	4059	8.480	50.850	9.690	1.00	0.00
ATOM	13300	HW1	SOL	4059	9.260	50.630	10.200	1.00	0.00
ATOM	13301	HW2	SOL	4059	8.450	50.200	8.990	1.00	0.00
ATOM	13302	OW	SOL	4060	10.690	19.380	22.900	1.00	0.00
ATOM	13303	HW1	SOL	4060	11.530	19.440	22.450	1.00	0.00
ATOM	13304	HW2	SOL	4060	10.540	20.250	23.260	1.00	0.00
ATOM	13305	OW	SOL	4061	21.660	46.950	5.910	1.00	0.00
ATOM	13306	HW1	SOL	4061	20.720	46.840	6.080	1.00	0.00
ATOM	13307	HW2	SOL	4061	21.840	46.390	5.160	1.00	0.00
ATOM	13308	OW	SOL	4062	37.580	41.110	6.060	1.00	0.00
ATOM	13309	HW1	SOL	4062	37.310	41.360	6.940	1.00	0.00
ATOM	13310	HW2	SOL	4062	38.490	40.820	6.150	1.00	0.00
ATOM	13311	OW	SOL	4063	4.830	5.760	25.580	1.00	0.00
ATOM	13312	HW1	SOL	4063	4.100	6.370	25.610	1.00	0.00
ATOM	13313	HW2	SOL	4063	5.580	6.260	25.910	1.00	0.00
ATOM	13314	OW	SOL	4064	11.630	44.810	54.880	1.00	0.00
ATOM	13315	HW1	SOL	4064	11.320	44.250	55.590	1.00	0.00
ATOM	13316	HW2	SOL	4064	12.560	44.950	55.070	1.00	0.00
ATOM	13317	OW	SOL	4065	55.610	3.080	38.970	1.00	0.00
ATOM	13318	HW1	SOL	4065	55.620	4.030	38.820	1.00	0.00
ATOM	13319	HW2	SOL	4065	55.160	2.720	38.200	1.00	0.00
ATOM	13320	OW	SOL	4066	7.770	22.070	2.240	1.00	0.00
ATOM	13321	HW1	SOL	4066	7.280	21.250	2.110	1.00	0.00
ATOM	13322	HW2	SOL	4066	8.040	22.320	1.360	1.00	0.00
ATOM	13323	OW	SOL	4067	35.200	49.390	19.700	1.00	0.00
ATOM	13324	HW1	SOL	4067	35.550	49.160	20.560	1.00	0.00
ATOM	13325	HW2	SOL	4067	34.250	49.340	19.810	1.00	0.00
ATOM	13326	OW	SOL	4068	18.640	18.020	44.040	1.00	0.00
ATOM	13327	HW1	SOL	4068	17.950	17.930	44.700	1.00	0.00
ATOM	13328	HW2	SOL	4068	19.110	18.810	44.290	1.00	0.00
ATOM	13329	OW	SOL	4069	15.130	5.400	33.280	1.00	0.00
ATOM	13330	HW1	SOL	4069	15.890	5.200	32.730	1.00	0.00
ATOM	13331	HW2	SOL	4069	15.430	6.110	33.860	1.00	0.00

ATOM	13332	OW	SOL	4070	20.840	12.730	6.640	1.00	0.00
ATOM	13333	HW1	SOL	4070	20.370	13.370	6.100	1.00	0.00
ATOM	13334	HW2	SOL	4070	20.330	11.930	6.570	1.00	0.00
ATOM	13335	OW	SOL	4071	12.570	33.440	5.320	1.00	0.00
ATOM	13336	HW1	SOL	4071	13.420	33.280	4.910	1.00	0.00
ATOM	13337	HW2	SOL	4071	12.200	32.560	5.450	1.00	0.00
ATOM	13338	OW	SOL	4072	22.630	21.790	36.210	1.00	0.00
ATOM	13339	HW1	SOL	4072	22.990	21.460	37.040	1.00	0.00
ATOM	13340	HW2	SOL	4072	21.800	22.190	36.460	1.00	0.00
ATOM	13341	OW	SOL	4073	37.500	37.960	0.570	1.00	0.00
ATOM	13342	HW1	SOL	4073	37.310	37.030	0.470	1.00	0.00
ATOM	13343	HW2	SOL	4073	37.390	38.330	-0.310	1.00	0.00
ATOM	13344	OW	SOL	4074	2.250	24.590	43.660	1.00	0.00
ATOM	13345	HW1	SOL	4074	2.510	25.510	43.530	1.00	0.00
ATOM	13346	HW2	SOL	4074	2.210	24.230	42.780	1.00	0.00
ATOM	13347	OW	SOL	4075	38.590	0.790	43.240	1.00	0.00
ATOM	13348	HW1	SOL	4075	38.450	0.690	44.180	1.00	0.00
ATOM	13349	HW2	SOL	4075	37.790	0.430	42.840	1.00	0.00
ATOM	13350	OW	SOL	4076	4.520	5.190	18.470	1.00	0.00
ATOM	13351	HW1	SOL	4076	4.770	5.190	19.390	1.00	0.00
ATOM	13352	HW2	SOL	4076	4.760	4.320	18.150	1.00	0.00
ATOM	13353	OW	SOL	4077	3.990	9.610	13.680	1.00	0.00
ATOM	13354	HW1	SOL	4077	3.620	9.060	14.370	1.00	0.00
ATOM	13355	HW2	SOL	4077	3.330	10.290	13.540	1.00	0.00
ATOM	13356	OW	SOL	4078	39.720	26.830	14.310	1.00	0.00
ATOM	13357	HW1	SOL	4078	38.900	26.520	14.680	1.00	0.00
ATOM	13358	HW2	SOL	4078	40.330	26.850	15.060	1.00	0.00
ATOM	13359	OW	SOL	4079	43.070	25.640	20.070	1.00	0.00
ATOM	13360	HW1	SOL	4079	42.540	24.900	19.780	1.00	0.00
ATOM	13361	HW2	SOL	4079	42.740	25.840	20.940	1.00	0.00
ATOM	13362	OW	SOL	4080	8.250	47.720	0.010	1.00	0.00
ATOM	13363	HW1	SOL	4080	9.090	47.320	-0.230	1.00	0.00
ATOM	13364	HW2	SOL	4080	8.480	48.470	0.560	1.00	0.00
ATOM	13365	OW	SOL	4081	34.770	2.610	39.150	1.00	0.00
ATOM	13366	HW1	SOL	4081	34.040	2.780	39.740	1.00	0.00
ATOM	13367	HW2	SOL	4081	34.890	1.660	39.160	1.00	0.00
ATOM	13368	OW	SOL	4082	15.930	7.210	52.690	1.00	0.00
ATOM	13369	HW1	SOL	4082	16.460	6.440	52.500	1.00	0.00
ATOM	13370	HW2	SOL	4082	16.550	7.830	53.090	1.00	0.00
ATOM	13371	OW	SOL	4083	18.390	36.020	24.280	1.00	0.00
ATOM	13372	HW1	SOL	4083	18.830	36.650	23.700	1.00	0.00
ATOM	13373	HW2	SOL	4083	19.100	35.640	24.800	1.00	0.00
ATOM	13374	OW	SOL	4084	35.010	31.140	7.080	1.00	0.00
ATOM	13375	HW1	SOL	4084	35.380	31.420	6.240	1.00	0.00

ATOM	13376	HW2	SOL	4084	34.430	31.850	7.340	1.00	0.00
ATOM	13377	OW	SOL	4085	10.980	33.280	52.660	1.00	0.00
ATOM	13378	HW1	SOL	4085	10.150	33.270	53.150	1.00	0.00
ATOM	13379	HW2	SOL	4085	10.890	34.020	52.060	1.00	0.00
ATOM	13380	OW	SOL	4086	29.660	3.420	22.290	1.00	0.00
ATOM	13381	HW1	SOL	4086	28.940	3.870	21.850	1.00	0.00
ATOM	13382	HW2	SOL	4086	29.750	2.590	21.800	1.00	0.00
ATOM	13383	OW	SOL	4087	20.730	20.190	9.660	1.00	0.00
ATOM	13384	HW1	SOL	4087	20.300	19.660	10.320	1.00	0.00
ATOM	13385	HW2	SOL	4087	21.570	20.430	10.050	1.00	0.00
ATOM	13386	OW	SOL	4088	43.950	55.630	22.330	1.00	0.00
ATOM	13387	HW1	SOL	4088	43.680	54.810	21.920	1.00	0.00
ATOM	13388	HW2	SOL	4088	44.900	55.580	22.380	1.00	0.00
ATOM	13389	OW	SOL	4089	30.320	21.130	55.290	1.00	0.00
ATOM	13390	HW1	SOL	4089	29.580	21.740	55.340	1.00	0.00
ATOM	13391	HW2	SOL	4089	29.980	20.310	55.650	1.00	0.00
ATOM	13392	OW	SOL	4090	36.400	32.350	23.220	1.00	0.00
ATOM	13393	HW1	SOL	4090	35.880	32.900	22.630	1.00	0.00
ATOM	13394	HW2	SOL	4090	36.100	31.460	23.050	1.00	0.00
ATOM	13395	OW	SOL	4091	27.250	1.200	18.250	1.00	0.00
ATOM	13396	HW1	SOL	4091	28.140	1.280	17.930	1.00	0.00
ATOM	13397	HW2	SOL	4091	27.120	1.970	18.800	1.00	0.00
ATOM	13398	OW	SOL	4092	36.060	5.110	20.430	1.00	0.00
ATOM	13399	HW1	SOL	4092	36.810	4.540	20.210	1.00	0.00
ATOM	13400	HW2	SOL	4092	36.160	5.870	19.870	1.00	0.00
ATOM	13401	OW	SOL	4093	50.070	27.390	21.430	1.00	0.00
ATOM	13402	HW1	SOL	4093	50.420	28.180	21.830	1.00	0.00
ATOM	13403	HW2	SOL	4093	49.120	27.480	21.500	1.00	0.00
ATOM	13404	OW	SOL	4094	31.130	27.420	6.550	1.00	0.00
ATOM	13405	HW1	SOL	4094	30.790	26.630	6.130	1.00	0.00
ATOM	13406	HW2	SOL	4094	30.570	27.550	7.320	1.00	0.00
ATOM	13407	OW	SOL	4095	38.660	20.810	9.420	1.00	0.00
ATOM	13408	HW1	SOL	4095	37.840	20.960	8.950	1.00	0.00
ATOM	13409	HW2	SOL	4095	39.300	21.340	8.950	1.00	0.00
ATOM	13410	OW	SOL	4096	33.680	52.950	45.420	1.00	0.00
ATOM	13411	HW1	SOL	4096	33.720	52.100	44.980	1.00	0.00
ATOM	13412	HW2	SOL	4096	33.530	53.570	44.710	1.00	0.00
ATOM	13413	OW	SOL	4097	41.120	40.560	19.320	1.00	0.00
ATOM	13414	HW1	SOL	4097	41.500	40.260	18.500	1.00	0.00
ATOM	13415	HW2	SOL	4097	40.340	40.020	19.440	1.00	0.00
ATOM	13416	OW	SOL	4098	23.040	20.230	12.140	1.00	0.00
ATOM	13417	HW1	SOL	4098	23.240	20.390	11.220	1.00	0.00
ATOM	13418	HW2	SOL	4098	23.510	19.420	12.350	1.00	0.00
ATOM	13419	OW	SOL	4099	38.700	10.380	1.140	1.00	0.00

ATOM	13420	HW1	SOL	4099	39.030	10.120	0.270	1.00	0.00
ATOM	13421	HW2	SOL	4099	38.700	9.570	1.650	1.00	0.00
ATOM	13422	OW	SOL	4100	53.280	3.250	32.660	1.00	0.00
ATOM	13423	HW1	SOL	4100	53.610	2.400	32.940	1.00	0.00
ATOM	13424	HW2	SOL	4100	52.860	3.090	31.820	1.00	0.00
ATOM	13425	OW	SOL	4101	42.820	19.530	10.760	1.00	0.00
ATOM	13426	HW1	SOL	4101	42.300	20.310	10.570	1.00	0.00
ATOM	13427	HW2	SOL	4101	43.040	19.170	9.900	1.00	0.00
ATOM	13428	OW	SOL	4102	34.500	7.800	37.830	1.00	0.00
ATOM	13429	HW1	SOL	4102	35.400	7.490	37.770	1.00	0.00
ATOM	13430	HW2	SOL	4102	34.360	7.990	38.750	1.00	0.00
ATOM	13431	OW	SOL	4103	13.890	33.190	25.070	1.00	0.00
ATOM	13432	HW1	SOL	4103	14.110	33.760	25.810	1.00	0.00
ATOM	13433	HW2	SOL	4103	14.360	33.580	24.330	1.00	0.00
ATOM	13434	OW	SOL	4104	38.170	23.440	12.880	1.00	0.00
ATOM	13435	HW1	SOL	4104	38.330	22.500	12.960	1.00	0.00
ATOM	13436	HW2	SOL	4104	37.240	23.540	13.070	1.00	0.00
ATOM	13437	OW	SOL	4105	11.970	54.250	21.940	1.00	0.00
ATOM	13438	HW1	SOL	4105	11.990	54.170	22.900	1.00	0.00
ATOM	13439	HW2	SOL	4105	12.810	53.880	21.660	1.00	0.00
ATOM	13440	OW	SOL	4106	30.790	17.460	51.780	1.00	0.00
ATOM	13441	HW1	SOL	4106	30.350	17.340	50.930	1.00	0.00
ATOM	13442	HW2	SOL	4106	30.830	16.580	52.150	1.00	0.00
ATOM	13443	OW	SOL	4107	23.480	22.650	42.560	1.00	0.00
ATOM	13444	HW1	SOL	4107	23.580	23.440	42.030	1.00	0.00
ATOM	13445	HW2	SOL	4107	22.980	22.050	42.010	1.00	0.00
ATOM	13446	OW	SOL	4108	34.020	52.670	27.740	1.00	0.00
ATOM	13447	HW1	SOL	4108	34.380	53.080	26.950	1.00	0.00
ATOM	13448	HW2	SOL	4108	34.120	53.330	28.420	1.00	0.00
ATOM	13449	OW	SOL	4109	38.530	3.540	27.180	1.00	0.00
ATOM	13450	HW1	SOL	4109	38.360	2.720	27.650	1.00	0.00
ATOM	13451	HW2	SOL	4109	37.760	3.660	26.630	1.00	0.00
ATOM	13452	OW	SOL	4110	0.800	8.680	31.870	1.00	0.00
ATOM	13453	HW1	SOL	4110	1.550	9.250	31.680	1.00	0.00
ATOM	13454	HW2	SOL	4110	0.070	9.280	31.990	1.00	0.00
ATOM	13455	OW	SOL	4111	51.320	2.250	9.060	1.00	0.00
ATOM	13456	HW1	SOL	4111	51.160	2.710	8.240	1.00	0.00
ATOM	13457	HW2	SOL	4111	50.470	1.930	9.340	1.00	0.00
ATOM	13458	OW	SOL	4112	27.480	50.890	26.500	1.00	0.00
ATOM	13459	HW1	SOL	4112	26.690	50.470	26.840	1.00	0.00
ATOM	13460	HW2	SOL	4112	27.180	51.370	25.730	1.00	0.00
ATOM	13461	OW	SOL	4113	0.440	48.960	30.610	1.00	0.00
ATOM	13462	HW1	SOL	4113	0.370	48.020	30.770	1.00	0.00
ATOM	13463	HW2	SOL	4113	0.350	49.360	31.480	1.00	0.00

ATOM	13464	OW	SOL	4114	45.280	55.350	45.600	1.00	0.00
ATOM	13465	HW1	SOL	4114	44.850	55.760	46.340	1.00	0.00
ATOM	13466	HW2	SOL	4114	45.620	56.090	45.080	1.00	0.00
ATOM	13467	OW	SOL	4115	37.780	18.480	52.590	1.00	0.00
ATOM	13468	HW1	SOL	4115	37.240	18.850	53.280	1.00	0.00
ATOM	13469	HW2	SOL	4115	37.160	18.140	51.950	1.00	0.00
ATOM	13470	OW	SOL	4116	13.430	53.530	50.330	1.00	0.00
ATOM	13471	HW1	SOL	4116	14.240	53.500	49.810	1.00	0.00
ATOM	13472	HW2	SOL	4116	12.730	53.550	49.680	1.00	0.00
ATOM	13473	OW	SOL	4117	16.930	18.760	16.710	1.00	0.00
ATOM	13474	HW1	SOL	4117	17.500	19.200	16.080	1.00	0.00
ATOM	13475	HW2	SOL	4117	16.250	18.360	16.170	1.00	0.00
ATOM	13476	OW	SOL	4118	42.920	0.270	8.930	1.00	0.00
ATOM	13477	HW1	SOL	4118	42.670	0.320	9.850	1.00	0.00
ATOM	13478	HW2	SOL	4118	42.160	-0.130	8.500	1.00	0.00
ATOM	13479	OW	SOL	4119	51.880	50.960	41.350	1.00	0.00
ATOM	13480	HW1	SOL	4119	51.170	50.590	40.820	1.00	0.00
ATOM	13481	HW2	SOL	4119	51.850	51.900	41.190	1.00	0.00
ATOM	13482	OW	SOL	4120	1.370	25.360	33.590	1.00	0.00
ATOM	13483	HW1	SOL	4120	0.780	24.830	34.130	1.00	0.00
ATOM	13484	HW2	SOL	4120	1.070	26.260	33.710	1.00	0.00
ATOM	13485	OW	SOL	4121	15.480	43.600	51.980	1.00	0.00
ATOM	13486	HW1	SOL	4121	14.990	42.800	52.160	1.00	0.00
ATOM	13487	HW2	SOL	4121	15.490	43.670	51.030	1.00	0.00
ATOM	13488	OW	SOL	4122	42.560	16.990	29.790	1.00	0.00
ATOM	13489	HW1	SOL	4122	42.720	16.200	30.300	1.00	0.00
ATOM	13490	HW2	SOL	4122	43.260	17.590	30.050	1.00	0.00
ATOM	13491	OW	SOL	4123	32.030	37.170	43.300	1.00	0.00
ATOM	13492	HW1	SOL	4123	31.150	37.180	43.700	1.00	0.00
ATOM	13493	HW2	SOL	4123	32.600	36.820	43.990	1.00	0.00
ATOM	13494	OW	SOL	4124	4.290	46.320	0.890	1.00	0.00
ATOM	13495	HW1	SOL	4124	3.660	46.280	0.170	1.00	0.00
ATOM	13496	HW2	SOL	4124	5.050	45.810	0.580	1.00	0.00
ATOM	13497	OW	SOL	4125	30.290	51.830	34.390	1.00	0.00
ATOM	13498	HW1	SOL	4125	29.820	51.140	33.930	1.00	0.00
ATOM	13499	HW2	SOL	4125	30.250	52.590	33.810	1.00	0.00
ATOM	13500	OW	SOL	4126	46.210	3.270	39.960	1.00	0.00
ATOM	13501	HW1	SOL	4126	46.120	3.050	40.890	1.00	0.00
ATOM	13502	HW2	SOL	4126	45.370	3.680	39.730	1.00	0.00
ATOM	13503	OW	SOL	4127	34.420	49.800	15.160	1.00	0.00
ATOM	13504	HW1	SOL	4127	34.850	49.150	15.720	1.00	0.00
ATOM	13505	HW2	SOL	4127	34.900	49.780	14.340	1.00	0.00
ATOM	13506	OW	SOL	4128	19.320	31.070	29.560	1.00	0.00
ATOM	13507	HW1	SOL	4128	19.370	30.400	28.880	1.00	0.00

ATOM	13508	HW2	SOL	4128	18.390	31.310	29.590	1.00	0.00
ATOM	13509	OW	SOL	4129	20.830	49.320	11.590	1.00	0.00
ATOM	13510	HW1	SOL	4129	21.600	49.220	12.150	1.00	0.00
ATOM	13511	HW2	SOL	4129	20.960	50.180	11.180	1.00	0.00
ATOM	13512	OW	SOL	4130	6.920	40.300	47.230	1.00	0.00
ATOM	13513	HW1	SOL	4130	6.150	40.020	47.730	1.00	0.00
ATOM	13514	HW2	SOL	4130	6.560	40.620	46.400	1.00	0.00
ATOM	13515	OW	SOL	4131	19.090	16.240	19.790	1.00	0.00
ATOM	13516	HW1	SOL	4131	18.960	15.890	20.670	1.00	0.00
ATOM	13517	HW2	SOL	4131	18.700	17.120	19.830	1.00	0.00
ATOM	13518	OW	SOL	4132	13.580	43.980	3.600	1.00	0.00
ATOM	13519	HW1	SOL	4132	14.340	44.210	3.060	1.00	0.00
ATOM	13520	HW2	SOL	4132	13.940	43.850	4.480	1.00	0.00
ATOM	13521	OW	SOL	4133	21.710	20.320	41.310	1.00	0.00
ATOM	13522	HW1	SOL	4133	21.580	19.990	42.190	1.00	0.00
ATOM	13523	HW2	SOL	4133	22.080	19.580	40.830	1.00	0.00
ATOM	13524	OW	SOL	4134	49.260	38.170	49.090	1.00	0.00
ATOM	13525	HW1	SOL	4134	50.060	38.620	48.810	1.00	0.00
ATOM	13526	HW2	SOL	4134	48.820	37.930	48.280	1.00	0.00
ATOM	13527	OW	SOL	4135	37.420	15.330	33.830	1.00	0.00
ATOM	13528	HW1	SOL	4135	36.780	14.730	33.450	1.00	0.00
ATOM	13529	HW2	SOL	4135	38.180	14.770	34.030	1.00	0.00
ATOM	13530	OW	SOL	4136	31.810	45.100	44.040	1.00	0.00
ATOM	13531	HW1	SOL	4136	32.440	45.790	43.810	1.00	0.00
ATOM	13532	HW2	SOL	4136	31.490	45.340	44.910	1.00	0.00
ATOM	13533	OW	SOL	4137	39.910	18.300	46.950	1.00	0.00
ATOM	13534	HW1	SOL	4137	39.790	17.720	47.700	1.00	0.00
ATOM	13535	HW2	SOL	4137	39.550	19.140	47.230	1.00	0.00
ATOM	13536	OW	SOL	4138	21.870	41.930	53.970	1.00	0.00
ATOM	13537	HW1	SOL	4138	21.550	42.020	54.860	1.00	0.00
ATOM	13538	HW2	SOL	4138	22.520	41.230	54.020	1.00	0.00
ATOM	13539	OW	SOL	4139	48.390	45.310	22.840	1.00	0.00
ATOM	13540	HW1	SOL	4139	49.120	45.600	23.400	1.00	0.00
ATOM	13541	HW2	SOL	4139	47.630	45.790	23.180	1.00	0.00
ATOM	13542	OW	SOL	4140	5.380	22.810	44.420	1.00	0.00
ATOM	13543	HW1	SOL	4140	4.530	22.640	44.000	1.00	0.00
ATOM	13544	HW2	SOL	4140	5.420	22.180	45.140	1.00	0.00
ATOM	13545	OW	SOL	4141	37.560	5.830	45.350	1.00	0.00
ATOM	13546	HW1	SOL	4141	37.860	5.090	44.820	1.00	0.00
ATOM	13547	HW2	SOL	4141	37.030	6.350	44.750	1.00	0.00
ATOM	13548	OW	SOL	4142	46.050	43.640	25.430	1.00	0.00
ATOM	13549	HW1	SOL	4142	46.340	44.330	24.830	1.00	0.00
ATOM	13550	HW2	SOL	4142	45.490	43.080	24.890	1.00	0.00
ATOM	13551	OW	SOL	4143	20.170	49.850	6.850	1.00	0.00

ATOM	13552	HW1	SOL	4143	20.920	50.430	6.670	1.00	0.00
ATOM	13553	HW2	SOL	4143	20.490	48.970	6.640	1.00	0.00
ATOM	13554	OW	SOL	4144	0.570	6.810	30.250	1.00	0.00
ATOM	13555	HW1	SOL	4144	1.240	6.190	30.520	1.00	0.00
ATOM	13556	HW2	SOL	4144	0.660	7.540	30.860	1.00	0.00
ATOM	13557	OW	SOL	4145	25.760	20.160	43.980	1.00	0.00
ATOM	13558	HW1	SOL	4145	26.050	20.570	43.160	1.00	0.00
ATOM	13559	HW2	SOL	4145	25.240	20.840	44.420	1.00	0.00
ATOM	13560	OW	SOL	4146	17.380	55.210	54.680	1.00	0.00
ATOM	13561	HW1	SOL	4146	16.870	55.970	54.400	1.00	0.00
ATOM	13562	HW2	SOL	4146	16.840	54.460	54.450	1.00	0.00
ATOM	13563	OW	SOL	4147	34.940	51.630	47.490	1.00	0.00
ATOM	13564	HW1	SOL	4147	34.380	51.920	46.760	1.00	0.00
ATOM	13565	HW2	SOL	4147	34.340	51.520	48.230	1.00	0.00
ATOM	13566	OW	SOL	4148	15.730	39.670	14.000	1.00	0.00
ATOM	13567	HW1	SOL	4148	15.200	40.420	14.280	1.00	0.00
ATOM	13568	HW2	SOL	4148	15.130	39.120	13.510	1.00	0.00
ATOM	13569	OW	SOL	4149	46.760	32.480	10.930	1.00	0.00
ATOM	13570	HW1	SOL	4149	47.640	32.200	10.660	1.00	0.00
ATOM	13571	HW2	SOL	4149	46.170	31.990	10.350	1.00	0.00
ATOM	13572	OW	SOL	4150	6.050	53.820	40.150	1.00	0.00
ATOM	13573	HW1	SOL	4150	5.220	54.250	40.380	1.00	0.00
ATOM	13574	HW2	SOL	4150	6.060	53.810	39.200	1.00	0.00
ATOM	13575	OW	SOL	4151	1.320	35.260	41.640	1.00	0.00
ATOM	13576	HW1	SOL	4151	0.890	35.990	42.080	1.00	0.00
ATOM	13577	HW2	SOL	4151	1.520	34.640	42.340	1.00	0.00
ATOM	13578	OW	SOL	4152	8.500	24.980	16.930	1.00	0.00
ATOM	13579	HW1	SOL	4152	7.550	24.830	16.990	1.00	0.00
ATOM	13580	HW2	SOL	4152	8.620	25.370	16.060	1.00	0.00
ATOM	13581	OW	SOL	4153	3.100	10.350	31.410	1.00	0.00
ATOM	13582	HW1	SOL	4153	3.530	11.130	31.060	1.00	0.00
ATOM	13583	HW2	SOL	4153	3.790	9.870	31.870	1.00	0.00
ATOM	13584	OW	SOL	4154	26.480	54.300	26.030	1.00	0.00
ATOM	13585	HW1	SOL	4154	27.230	54.210	25.440	1.00	0.00
ATOM	13586	HW2	SOL	4154	25.970	53.510	25.890	1.00	0.00
ATOM	13587	OW	SOL	4155	33.920	44.470	24.250	1.00	0.00
ATOM	13588	HW1	SOL	4155	34.050	44.950	23.430	1.00	0.00
ATOM	13589	HW2	SOL	4155	33.000	44.610	24.470	1.00	0.00
ATOM	13590	OW	SOL	4156	50.830	24.460	26.950	1.00	0.00
ATOM	13591	HW1	SOL	4156	50.480	24.830	26.150	1.00	0.00
ATOM	13592	HW2	SOL	4156	51.040	23.560	26.730	1.00	0.00
ATOM	13593	OW	SOL	4157	12.200	43.940	34.060	1.00	0.00
ATOM	13594	HW1	SOL	4157	13.020	44.090	33.590	1.00	0.00
ATOM	13595	HW2	SOL	4157	12.250	43.030	34.330	1.00	0.00

ATOM	13596	OW	SOL	4158	39.710	46.710	34.510	1.00	0.00
ATOM	13597	HW1	SOL	4158	40.130	46.170	33.850	1.00	0.00
ATOM	13598	HW2	SOL	4158	40.380	47.350	34.760	1.00	0.00
ATOM	13599	OW	SOL	4159	35.460	2.320	55.500	1.00	0.00
ATOM	13600	HW1	SOL	4159	36.300	2.700	55.240	1.00	0.00
ATOM	13601	HW2	SOL	4159	35.170	2.860	56.230	1.00	0.00
ATOM	13602	OW	SOL	4160	27.590	22.250	11.360	1.00	0.00
ATOM	13603	HW1	SOL	4160	27.130	22.200	12.200	1.00	0.00
ATOM	13604	HW2	SOL	4160	28.450	22.620	11.580	1.00	0.00
ATOM	13605	OW	SOL	4161	41.120	29.420	43.950	1.00	0.00
ATOM	13606	HW1	SOL	4161	40.700	30.040	44.550	1.00	0.00
ATOM	13607	HW2	SOL	4161	40.840	29.710	43.080	1.00	0.00
ATOM	13608	OW	SOL	4162	45.650	7.700	48.220	1.00	0.00
ATOM	13609	HW1	SOL	4162	45.970	7.390	49.060	1.00	0.00
ATOM	13610	HW2	SOL	4162	46.250	8.410	47.980	1.00	0.00
ATOM	13611	OW	SOL	4163	15.920	8.780	9.090	1.00	0.00
ATOM	13612	HW1	SOL	4163	15.330	8.730	9.850	1.00	0.00
ATOM	13613	HW2	SOL	4163	16.800	8.730	9.470	1.00	0.00
ATOM	13614	OW	SOL	4164	27.000	53.700	3.690	1.00	0.00
ATOM	13615	HW1	SOL	4164	27.310	53.120	4.380	1.00	0.00
ATOM	13616	HW2	SOL	4164	26.960	54.560	4.100	1.00	0.00
ATOM	13617	OW	SOL	4165	27.260	20.450	16.750	1.00	0.00
ATOM	13618	HW1	SOL	4165	27.480	21.170	16.150	1.00	0.00
ATOM	13619	HW2	SOL	4165	26.420	20.700	17.130	1.00	0.00
ATOM	13620	OW	SOL	4166	5.180	23.440	0.610	1.00	0.00
ATOM	13621	HW1	SOL	4166	6.030	23.240	0.220	1.00	0.00
ATOM	13622	HW2	SOL	4166	5.310	24.260	1.080	1.00	0.00
ATOM	13623	OW	SOL	4167	46.250	51.380	29.080	1.00	0.00
ATOM	13624	HW1	SOL	4167	46.980	51.990	28.990	1.00	0.00
ATOM	13625	HW2	SOL	4167	45.630	51.640	28.400	1.00	0.00
ATOM	13626	OW	SOL	4168	9.520	4.440	17.140	1.00	0.00
ATOM	13627	HW1	SOL	4168	9.330	4.210	16.230	1.00	0.00
ATOM	13628	HW2	SOL	4168	9.000	3.830	17.660	1.00	0.00
ATOM	13629	OW	SOL	4169	54.780	44.180	23.060	1.00	0.00
ATOM	13630	HW1	SOL	4169	55.160	43.310	23.170	1.00	0.00
ATOM	13631	HW2	SOL	4169	55.390	44.770	23.490	1.00	0.00
ATOM	13632	OW	SOL	4170	32.480	11.590	1.150	1.00	0.00
ATOM	13633	HW1	SOL	4170	33.090	10.900	0.890	1.00	0.00
ATOM	13634	HW2	SOL	4170	32.190	11.980	0.320	1.00	0.00
ATOM	13635	OW	SOL	4171	52.200	53.700	41.830	1.00	0.00
ATOM	13636	HW1	SOL	4171	52.830	54.380	41.610	1.00	0.00
ATOM	13637	HW2	SOL	4171	51.540	54.130	42.370	1.00	0.00
ATOM	13638	OW	SOL	4172	52.690	54.220	5.120	1.00	0.00
ATOM	13639	HW1	SOL	4172	53.460	54.560	4.670	1.00	0.00

ATOM	13640	HW2	SOL	4172	52.190	54.990	5.370	1.00	0.00
ATOM	13641	OW	SOL	4173	0.660	41.970	55.150	1.00	0.00
ATOM	13642	HW1	SOL	4173	0.180	42.660	54.700	1.00	0.00
ATOM	13643	HW2	SOL	4173	1.580	42.120	54.920	1.00	0.00
ATOM	13644	OW	SOL	4174	7.450	32.970	13.800	1.00	0.00
ATOM	13645	HW1	SOL	4174	8.100	32.310	13.560	1.00	0.00
ATOM	13646	HW2	SOL	4174	6.640	32.470	13.920	1.00	0.00
ATOM	13647	OW	SOL	4175	28.830	12.320	14.950	1.00	0.00
ATOM	13648	HW1	SOL	4175	28.530	12.810	15.720	1.00	0.00
ATOM	13649	HW2	SOL	4175	29.230	12.990	14.390	1.00	0.00
ATOM	13650	OW	SOL	4176	6.890	53.770	37.030	1.00	0.00
ATOM	13651	HW1	SOL	4176	6.480	53.930	36.180	1.00	0.00
ATOM	13652	HW2	SOL	4176	6.620	52.880	37.270	1.00	0.00
ATOM	13653	OW	SOL	4177	0.170	4.170	36.050	1.00	0.00
ATOM	13654	HW1	SOL	4177	-0.470	3.480	36.240	1.00	0.00
ATOM	13655	HW2	SOL	4177	-0.350	4.970	36.010	1.00	0.00
ATOM	13656	OW	SOL	4178	43.380	11.910	19.960	1.00	0.00
ATOM	13657	HW1	SOL	4178	43.020	12.230	19.140	1.00	0.00
ATOM	13658	HW2	SOL	4178	42.660	11.440	20.380	1.00	0.00
ATOM	13659	OW	SOL	4179	24.150	6.060	44.690	1.00	0.00
ATOM	13660	HW1	SOL	4179	24.480	6.950	44.760	1.00	0.00
ATOM	13661	HW2	SOL	4179	23.990	5.790	45.600	1.00	0.00
ATOM	13662	OW	SOL	4180	50.110	42.700	28.030	1.00	0.00
ATOM	13663	HW1	SOL	4180	49.540	42.540	27.280	1.00	0.00
ATOM	13664	HW2	SOL	4180	49.560	42.570	28.790	1.00	0.00
ATOM	13665	OW	SOL	4181	53.190	31.500	5.140	1.00	0.00
ATOM	13666	HW1	SOL	4181	52.840	32.010	5.870	1.00	0.00
ATOM	13667	HW2	SOL	4181	54.120	31.390	5.340	1.00	0.00
ATOM	13668	OW	SOL	4182	27.420	0.970	38.300	1.00	0.00
ATOM	13669	HW1	SOL	4182	28.240	1.130	37.830	1.00	0.00
ATOM	13670	HW2	SOL	4182	27.050	0.200	37.880	1.00	0.00
ATOM	13671	OW	SOL	4183	48.830	44.430	0.140	1.00	0.00
ATOM	13672	HW1	SOL	4183	49.150	44.540	-0.760	1.00	0.00
ATOM	13673	HW2	SOL	4183	49.610	44.170	0.630	1.00	0.00
ATOM	13674	OW	SOL	4184	32.930	23.150	7.450	1.00	0.00
ATOM	13675	HW1	SOL	4184	32.170	23.400	7.970	1.00	0.00
ATOM	13676	HW2	SOL	4184	32.660	23.280	6.540	1.00	0.00
ATOM	13677	OW	SOL	4185	43.320	35.080	50.130	1.00	0.00
ATOM	13678	HW1	SOL	4185	43.890	35.680	50.610	1.00	0.00
ATOM	13679	HW2	SOL	4185	42.610	34.890	50.740	1.00	0.00
ATOM	13680	OW	SOL	4186	38.570	20.250	26.970	1.00	0.00
ATOM	13681	HW1	SOL	4186	37.980	20.430	26.240	1.00	0.00
ATOM	13682	HW2	SOL	4186	39.270	19.720	26.580	1.00	0.00
ATOM	13683	OW	SOL	4187	35.790	43.950	9.610	1.00	0.00

ATOM	13684	HW1	SOL	4187	36.090	43.920	8.700	1.00	0.00
ATOM	13685	HW2	SOL	4187	35.270	43.160	9.720	1.00	0.00
ATOM	13686	OW	SOL	4188	39.370	4.860	40.780	1.00	0.00
ATOM	13687	HW1	SOL	4188	38.920	5.670	41.020	1.00	0.00
ATOM	13688	HW2	SOL	4188	38.670	4.210	40.710	1.00	0.00
ATOM	13689	OW	SOL	4189	47.350	16.460	8.570	1.00	0.00
ATOM	13690	HW1	SOL	4189	47.630	17.190	9.120	1.00	0.00
ATOM	13691	HW2	SOL	4189	47.990	15.770	8.750	1.00	0.00
ATOM	13692	OW	SOL	4190	18.870	31.940	0.020	1.00	0.00
ATOM	13693	HW1	SOL	4190	18.600	31.060	0.280	1.00	0.00
ATOM	13694	HW2	SOL	4190	19.550	31.790	-0.640	1.00	0.00
ATOM	13695	OW	SOL	4191	9.140	53.400	9.950	1.00	0.00
ATOM	13696	HW1	SOL	4191	9.660	53.670	9.200	1.00	0.00
ATOM	13697	HW2	SOL	4191	9.020	52.460	9.840	1.00	0.00
ATOM	13698	OW	SOL	4192	6.240	35.230	21.080	1.00	0.00
ATOM	13699	HW1	SOL	4192	6.600	34.710	21.790	1.00	0.00
ATOM	13700	HW2	SOL	4192	6.990	35.420	20.510	1.00	0.00
ATOM	13701	OW	SOL	4193	48.440	5.120	36.360	1.00	0.00
ATOM	13702	HW1	SOL	4193	49.360	5.200	36.080	1.00	0.00
ATOM	13703	HW2	SOL	4193	48.210	4.210	36.160	1.00	0.00
ATOM	13704	OW	SOL	4194	50.030	12.070	35.530	1.00	0.00
ATOM	13705	HW1	SOL	4194	49.930	11.550	36.320	1.00	0.00
ATOM	13706	HW2	SOL	4194	49.950	12.970	35.820	1.00	0.00
ATOM	13707	OW	SOL	4195	0.870	9.440	18.270	1.00	0.00
ATOM	13708	HW1	SOL	4195	0.750	8.510	18.080	1.00	0.00
ATOM	13709	HW2	SOL	4195	1.360	9.460	19.090	1.00	0.00
ATOM	13710	OW	SOL	4196	36.970	27.360	25.560	1.00	0.00
ATOM	13711	HW1	SOL	4196	36.610	26.610	26.040	1.00	0.00
ATOM	13712	HW2	SOL	4196	36.350	28.070	25.720	1.00	0.00
ATOM	13713	OW	SOL	4197	43.690	52.610	52.100	1.00	0.00
ATOM	13714	HW1	SOL	4197	43.330	53.500	52.100	1.00	0.00
ATOM	13715	HW2	SOL	4197	44.310	52.600	52.830	1.00	0.00
ATOM	13716	OW	SOL	4198	19.580	15.580	39.130	1.00	0.00
ATOM	13717	HW1	SOL	4198	20.010	15.760	38.290	1.00	0.00
ATOM	13718	HW2	SOL	4198	18.680	15.870	39.000	1.00	0.00
ATOM	13719	OW	SOL	4199	5.080	3.990	31.220	1.00	0.00
ATOM	13720	HW1	SOL	4199	4.150	4.060	31.000	1.00	0.00
ATOM	13721	HW2	SOL	4199	5.080	3.770	32.150	1.00	0.00
ATOM	13722	OW	SOL	4200	15.540	40.350	54.800	1.00	0.00
ATOM	13723	HW1	SOL	4200	16.390	40.730	54.580	1.00	0.00
ATOM	13724	HW2	SOL	4200	15.010	41.090	55.090	1.00	0.00
ATOM	13725	OW	SOL	4201	25.690	40.920	4.710	1.00	0.00
ATOM	13726	HW1	SOL	4201	25.350	40.070	4.420	1.00	0.00
ATOM	13727	HW2	SOL	4201	24.930	41.380	5.070	1.00	0.00

ATOM	13728	OW	SOL	4202	6.750	45.390	55.820	1.00	0.00
ATOM	13729	HW1	SOL	4202	7.380	46.110	55.910	1.00	0.00
ATOM	13730	HW2	SOL	4202	6.630	45.300	54.880	1.00	0.00
ATOM	13731	OW	SOL	4203	44.360	35.130	4.280	1.00	0.00
ATOM	13732	HW1	SOL	4203	43.430	34.910	4.280	1.00	0.00
ATOM	13733	HW2	SOL	4203	44.390	36.060	4.050	1.00	0.00
ATOM	13734	OW	SOL	4204	55.560	20.890	54.970	1.00	0.00
ATOM	13735	HW1	SOL	4204	56.230	20.620	55.600	1.00	0.00
ATOM	13736	HW2	SOL	4204	54.970	20.130	54.910	1.00	0.00
ATOM	13737	OW	SOL	4205	20.780	20.910	27.690	1.00	0.00
ATOM	13738	HW1	SOL	4205	20.580	21.570	27.020	1.00	0.00
ATOM	13739	HW2	SOL	4205	20.270	21.200	28.450	1.00	0.00
ATOM	13740	OW	SOL	4206	53.530	51.820	43.800	1.00	0.00
ATOM	13741	HW1	SOL	4206	53.400	51.020	44.300	1.00	0.00
ATOM	13742	HW2	SOL	4206	52.810	51.840	43.170	1.00	0.00
ATOM	13743	OW	SOL	4207	24.850	9.440	25.900	1.00	0.00
ATOM	13744	HW1	SOL	4207	23.940	9.730	25.950	1.00	0.00
ATOM	13745	HW2	SOL	4207	24.840	8.540	26.230	1.00	0.00
ATOM	13746	OW	SOL	4208	28.820	30.860	22.880	1.00	0.00
ATOM	13747	HW1	SOL	4208	28.040	31.140	22.410	1.00	0.00
ATOM	13748	HW2	SOL	4208	28.950	31.520	23.560	1.00	0.00
ATOM	13749	OW	SOL	4209	51.560	41.250	41.310	1.00	0.00
ATOM	13750	HW1	SOL	4209	52.400	41.370	40.850	1.00	0.00
ATOM	13751	HW2	SOL	4209	51.620	41.840	42.060	1.00	0.00
ATOM	13752	OW	SOL	4210	39.870	1.940	22.070	1.00	0.00
ATOM	13753	HW1	SOL	4210	39.650	2.820	21.760	1.00	0.00
ATOM	13754	HW2	SOL	4210	39.470	1.890	22.950	1.00	0.00
ATOM	13755	OW	SOL	4211	46.860	3.280	8.900	1.00	0.00
ATOM	13756	HW1	SOL	4211	47.550	3.910	9.100	1.00	0.00
ATOM	13757	HW2	SOL	4211	46.900	3.180	7.940	1.00	0.00
ATOM	13758	OW	SOL	4212	17.370	55.730	30.100	1.00	0.00
ATOM	13759	HW1	SOL	4212	18.160	55.610	30.630	1.00	0.00
ATOM	13760	HW2	SOL	4212	17.210	56.680	30.130	1.00	0.00
ATOM	13761	OW	SOL	4213	49.440	41.110	12.900	1.00	0.00
ATOM	13762	HW1	SOL	4213	48.590	41.120	13.330	1.00	0.00
ATOM	13763	HW2	SOL	4213	49.510	40.230	12.520	1.00	0.00
ATOM	13764	OW	SOL	4214	52.980	49.330	49.440	1.00	0.00
ATOM	13765	HW1	SOL	4214	52.920	49.680	48.560	1.00	0.00
ATOM	13766	HW2	SOL	4214	52.110	49.470	49.820	1.00	0.00
ATOM	13767	OW	SOL	4215	43.080	16.740	38.600	1.00	0.00
ATOM	13768	HW1	SOL	4215	42.430	16.530	39.270	1.00	0.00
ATOM	13769	HW2	SOL	4215	43.920	16.490	39.000	1.00	0.00
ATOM	13770	OW	SOL	4216	18.480	44.080	2.590	1.00	0.00
ATOM	13771	HW1	SOL	4216	19.280	44.370	2.140	1.00	0.00

ATOM	13772	HW2	SOL	4216	18.190	43.320	2.090	1.00	0.00
ATOM	13773	OW	SOL	4217	17.070	12.340	13.770	1.00	0.00
ATOM	13774	HW1	SOL	4217	17.360	12.600	14.640	1.00	0.00
ATOM	13775	HW2	SOL	4217	17.090	13.150	13.250	1.00	0.00
ATOM	13776	OW	SOL	4218	37.380	6.650	13.100	1.00	0.00
ATOM	13777	HW1	SOL	4218	36.740	7.060	12.520	1.00	0.00
ATOM	13778	HW2	SOL	4218	38.130	6.460	12.540	1.00	0.00
ATOM	13779	OW	SOL	4219	24.810	21.370	40.040	1.00	0.00
ATOM	13780	HW1	SOL	4219	24.930	22.070	39.400	1.00	0.00
ATOM	13781	HW2	SOL	4219	24.150	20.790	39.650	1.00	0.00
ATOM	13782	OW	SOL	4220	5.560	1.670	52.660	1.00	0.00
ATOM	13783	HW1	SOL	4220	5.940	2.180	53.390	1.00	0.00
ATOM	13784	HW2	SOL	4220	4.950	2.280	52.240	1.00	0.00
ATOM	13785	OW	SOL	4221	2.680	2.430	26.930	1.00	0.00
ATOM	13786	HW1	SOL	4221	2.150	2.470	27.730	1.00	0.00
ATOM	13787	HW2	SOL	4221	3.250	1.670	27.060	1.00	0.00
ATOM	13788	OW	SOL	4222	49.840	23.110	9.910	1.00	0.00
ATOM	13789	HW1	SOL	4222	50.200	23.910	10.290	1.00	0.00
ATOM	13790	HW2	SOL	4222	48.950	23.060	10.270	1.00	0.00
ATOM	13791	OW	SOL	4223	54.150	52.340	28.750	1.00	0.00
ATOM	13792	HW1	SOL	4223	53.920	51.990	29.620	1.00	0.00
ATOM	13793	HW2	SOL	4223	53.310	52.580	28.360	1.00	0.00
ATOM	13794	OW	SOL	4224	30.620	16.240	35.590	1.00	0.00
ATOM	13795	HW1	SOL	4224	30.570	17.140	35.920	1.00	0.00
ATOM	13796	HW2	SOL	4224	31.020	15.740	36.300	1.00	0.00
ATOM	13797	OW	SOL	4225	0.400	39.860	32.700	1.00	0.00
ATOM	13798	HW1	SOL	4225	0.430	38.960	33.040	1.00	0.00
ATOM	13799	HW2	SOL	4225	1.070	40.330	33.200	1.00	0.00
ATOM	13800	OW	SOL	4226	25.210	52.490	19.200	1.00	0.00
ATOM	13801	HW1	SOL	4226	25.790	53.240	19.080	1.00	0.00
ATOM	13802	HW2	SOL	4226	24.520	52.820	19.770	1.00	0.00
ATOM	13803	OW	SOL	4227	36.950	42.160	49.500	1.00	0.00
ATOM	13804	HW1	SOL	4227	36.600	42.950	49.910	1.00	0.00
ATOM	13805	HW2	SOL	4227	37.890	42.220	49.620	1.00	0.00
ATOM	13806	OW	SOL	4228	44.750	0.790	36.730	1.00	0.00
ATOM	13807	HW1	SOL	4228	45.480	1.120	37.260	1.00	0.00
ATOM	13808	HW2	SOL	4228	44.620	1.460	36.060	1.00	0.00
ATOM	13809	OW	SOL	4229	11.950	23.830	16.620	1.00	0.00
ATOM	13810	HW1	SOL	4229	11.550	24.650	16.340	1.00	0.00
ATOM	13811	HW2	SOL	4229	11.210	23.280	16.890	1.00	0.00
ATOM	13812	OW	SOL	4230	49.620	55.210	12.170	1.00	0.00
ATOM	13813	HW1	SOL	4230	48.730	55.470	12.420	1.00	0.00
ATOM	13814	HW2	SOL	4230	49.500	54.440	11.610	1.00	0.00
ATOM	13815	OW	SOL	4231	12.190	10.410	12.240	1.00	0.00

ATOM	13816	HW1	SOL	4231	13.080	10.670	12.000	1.00	0.00
ATOM	13817	HW2	SOL	4231	12.000	10.880	13.050	1.00	0.00
ATOM	13818	OW	SOL	4232	22.450	24.260	5.390	1.00	0.00
ATOM	13819	HW1	SOL	4232	21.830	23.570	5.140	1.00	0.00
ATOM	13820	HW2	SOL	4232	22.090	24.630	6.200	1.00	0.00
ATOM	13821	OW	SOL	4233	54.370	4.670	7.280	1.00	0.00
ATOM	13822	HW1	SOL	4233	54.400	3.720	7.400	1.00	0.00
ATOM	13823	HW2	SOL	4233	54.690	5.020	8.120	1.00	0.00
ATOM	13824	OW	SOL	4234	37.210	10.000	49.710	1.00	0.00
ATOM	13825	HW1	SOL	4234	37.300	9.120	50.070	1.00	0.00
ATOM	13826	HW2	SOL	4234	36.650	9.890	48.940	1.00	0.00
ATOM	13827	OW	SOL	4235	48.960	2.430	25.410	1.00	0.00
ATOM	13828	HW1	SOL	4235	49.890	2.460	25.180	1.00	0.00
ATOM	13829	HW2	SOL	4235	48.790	1.500	25.590	1.00	0.00
ATOM	13830	OW	SOL	4236	11.310	50.490	7.120	1.00	0.00
ATOM	13831	HW1	SOL	4236	11.180	51.110	7.830	1.00	0.00
ATOM	13832	HW2	SOL	4236	11.430	51.050	6.340	1.00	0.00
ATOM	13833	OW	SOL	4237	16.670	8.600	55.540	1.00	0.00
ATOM	13834	HW1	SOL	4237	16.400	8.810	56.430	1.00	0.00
ATOM	13835	HW2	SOL	4237	17.370	7.950	55.640	1.00	0.00
ATOM	13836	OW	SOL	4238	42.890	42.230	13.820	1.00	0.00
ATOM	13837	HW1	SOL	4238	42.970	41.840	12.950	1.00	0.00
ATOM	13838	HW2	SOL	4238	42.070	41.880	14.160	1.00	0.00
ATOM	13839	OW	SOL	4239	26.000	1.660	31.120	1.00	0.00
ATOM	13840	HW1	SOL	4239	25.330	2.320	30.900	1.00	0.00
ATOM	13841	HW2	SOL	4239	26.810	2.160	31.170	1.00	0.00
ATOM	13842	OW	SOL	4240	50.880	16.120	52.760	1.00	0.00
ATOM	13843	HW1	SOL	4240	51.180	15.240	53.000	1.00	0.00
ATOM	13844	HW2	SOL	4240	49.940	16.110	52.960	1.00	0.00
ATOM	13845	OW	SOL	4241	25.260	29.450	21.110	1.00	0.00
ATOM	13846	HW1	SOL	4241	24.970	30.180	21.660	1.00	0.00
ATOM	13847	HW2	SOL	4241	24.560	29.340	20.460	1.00	0.00
ATOM	13848	OW	SOL	4242	53.180	23.350	10.480	1.00	0.00
ATOM	13849	HW1	SOL	4242	52.580	23.990	10.870	1.00	0.00
ATOM	13850	HW2	SOL	4242	52.650	22.900	9.830	1.00	0.00
ATOM	13851	OW	SOL	4243	4.700	37.190	32.690	1.00	0.00
ATOM	13852	HW1	SOL	4243	4.940	37.920	33.270	1.00	0.00
ATOM	13853	HW2	SOL	4243	3.950	37.530	32.190	1.00	0.00
ATOM	13854	OW	SOL	4244	1.460	27.640	26.590	1.00	0.00
ATOM	13855	HW1	SOL	4244	0.950	28.440	26.700	1.00	0.00
ATOM	13856	HW2	SOL	4244	1.130	27.050	27.260	1.00	0.00
ATOM	13857	OW	SOL	4245	25.390	35.680	22.640	1.00	0.00
ATOM	13858	HW1	SOL	4245	24.650	35.320	23.130	1.00	0.00
ATOM	13859	HW2	SOL	4245	25.090	35.700	21.730	1.00	0.00

ATOM	13860	OW	SOL	4246	47.850	19.390	13.700	1.00	0.00
ATOM	13861	HW1	SOL	4246	47.560	20.000	13.020	1.00	0.00
ATOM	13862	HW2	SOL	4246	48.210	19.940	14.390	1.00	0.00
ATOM	13863	OW	SOL	4247	17.140	49.460	45.210	1.00	0.00
ATOM	13864	HW1	SOL	4247	16.530	50.190	45.280	1.00	0.00
ATOM	13865	HW2	SOL	4247	17.810	49.760	44.600	1.00	0.00
ATOM	13866	OW	SOL	4248	16.420	2.240	49.530	1.00	0.00
ATOM	13867	HW1	SOL	4248	15.840	2.740	48.960	1.00	0.00
ATOM	13868	HW2	SOL	4248	16.850	1.620	48.950	1.00	0.00
ATOM	13869	OW	SOL	4249	34.750	44.640	27.540	1.00	0.00
ATOM	13870	HW1	SOL	4249	34.590	44.880	28.450	1.00	0.00
ATOM	13871	HW2	SOL	4249	33.890	44.640	27.130	1.00	0.00
ATOM	13872	OW	SOL	4250	13.950	41.910	8.400	1.00	0.00
ATOM	13873	HW1	SOL	4250	14.670	42.400	8.780	1.00	0.00
ATOM	13874	HW2	SOL	4250	14.360	41.360	7.730	1.00	0.00
ATOM	13875	OW	SOL	4251	50.840	28.680	55.200	1.00	0.00
ATOM	13876	HW1	SOL	4251	51.450	28.050	54.820	1.00	0.00
ATOM	13877	HW2	SOL	4251	51.410	29.390	55.530	1.00	0.00
ATOM	13878	OW	SOL	4252	38.620	41.060	45.360	1.00	0.00
ATOM	13879	HW1	SOL	4252	38.990	41.030	44.480	1.00	0.00
ATOM	13880	HW2	SOL	4252	39.380	41.090	45.940	1.00	0.00
ATOM	13881	OW	SOL	4253	54.350	45.620	42.520	1.00	0.00
ATOM	13882	HW1	SOL	4253	54.750	46.470	42.330	1.00	0.00
ATOM	13883	HW2	SOL	4253	54.880	45.250	43.220	1.00	0.00
ATOM	13884	OW	SOL	4254	47.920	41.220	46.980	1.00	0.00
ATOM	13885	HW1	SOL	4254	48.660	40.770	46.570	1.00	0.00
ATOM	13886	HW2	SOL	4254	48.060	42.150	46.780	1.00	0.00
ATOM	13887	OW	SOL	4255	31.110	39.150	51.780	1.00	0.00
ATOM	13888	HW1	SOL	4255	31.890	39.700	51.670	1.00	0.00
ATOM	13889	HW2	SOL	4255	30.530	39.660	52.350	1.00	0.00
ATOM	13890	OW	SOL	4256	47.460	20.750	55.450	1.00	0.00
ATOM	13891	HW1	SOL	4256	46.730	20.320	55.020	1.00	0.00
ATOM	13892	HW2	SOL	4256	47.680	20.180	56.190	1.00	0.00
ATOM	13893	OW	SOL	4257	17.040	25.300	1.970	1.00	0.00
ATOM	13894	HW1	SOL	4257	17.000	24.680	1.240	1.00	0.00
ATOM	13895	HW2	SOL	4257	17.810	25.040	2.470	1.00	0.00
ATOM	13896	OW	SOL	4258	6.500	41.510	44.620	1.00	0.00
ATOM	13897	HW1	SOL	4258	5.610	41.390	44.300	1.00	0.00
ATOM	13898	HW2	SOL	4258	6.880	42.170	44.040	1.00	0.00
ATOM	13899	OW	SOL	4259	24.490	47.200	16.560	1.00	0.00
ATOM	13900	HW1	SOL	4259	25.140	47.320	15.870	1.00	0.00
ATOM	13901	HW2	SOL	4259	23.650	47.200	16.100	1.00	0.00
ATOM	13902	OW	SOL	4260	34.330	31.450	9.900	1.00	0.00
ATOM	13903	HW1	SOL	4260	35.270	31.600	9.980	1.00	0.00

ATOM	13904	HW2	SOL	4260	34.000	31.510	10.790	1.00	0.00
ATOM	13905	OW	SOL	4261	8.370	31.310	20.360	1.00	0.00
ATOM	13906	HW1	SOL	4261	8.010	31.100	21.220	1.00	0.00
ATOM	13907	HW2	SOL	4261	7.730	30.960	19.740	1.00	0.00
ATOM	13908	OW	SOL	4262	22.160	52.790	17.880	1.00	0.00
ATOM	13909	HW1	SOL	4262	22.980	52.970	18.340	1.00	0.00
ATOM	13910	HW2	SOL	4262	22.390	52.170	17.190	1.00	0.00
ATOM	13911	OW	SOL	4263	4.470	21.930	7.080	1.00	0.00
ATOM	13912	HW1	SOL	4263	3.990	21.680	7.860	1.00	0.00
ATOM	13913	HW2	SOL	4263	5.030	21.180	6.890	1.00	0.00
ATOM	13914	OW	SOL	4264	35.420	29.560	52.040	1.00	0.00
ATOM	13915	HW1	SOL	4264	35.790	30.220	51.460	1.00	0.00
ATOM	13916	HW2	SOL	4264	36.080	28.870	52.080	1.00	0.00
ATOM	13917	OW	SOL	4265	3.610	36.190	43.920	1.00	0.00
ATOM	13918	HW1	SOL	4265	3.980	35.940	43.070	1.00	0.00
ATOM	13919	HW2	SOL	4265	4.250	35.890	44.560	1.00	0.00
ATOM	13920	OW	SOL	4266	0.560	51.670	19.790	1.00	0.00
ATOM	13921	HW1	SOL	4266	0.170	51.700	20.660	1.00	0.00
ATOM	13922	HW2	SOL	4266	1.490	51.510	19.940	1.00	0.00
ATOM	13923	OW	SOL	4267	40.210	20.760	43.690	1.00	0.00
ATOM	13924	HW1	SOL	4267	41.080	21.150	43.610	1.00	0.00
ATOM	13925	HW2	SOL	4267	40.260	20.240	44.500	1.00	0.00
ATOM	13926	OW	SOL	4268	27.220	5.310	16.710	1.00	0.00
ATOM	13927	HW1	SOL	4268	27.360	5.680	15.840	1.00	0.00
ATOM	13928	HW2	SOL	4268	26.480	5.800	17.060	1.00	0.00
ATOM	13929	OW	SOL	4269	50.310	34.730	18.170	1.00	0.00
ATOM	13930	HW1	SOL	4269	50.810	35.540	18.070	1.00	0.00
ATOM	13931	HW2	SOL	4269	49.700	34.730	17.430	1.00	0.00
ATOM	13932	OW	SOL	4270	37.480	50.140	13.210	1.00	0.00
ATOM	13933	HW1	SOL	4270	37.070	50.930	12.870	1.00	0.00
ATOM	13934	HW2	SOL	4270	36.790	49.480	13.190	1.00	0.00
ATOM	13935	OW	SOL	4271	19.720	30.650	33.960	1.00	0.00
ATOM	13936	HW1	SOL	4271	19.840	31.510	34.370	1.00	0.00
ATOM	13937	HW2	SOL	4271	20.050	30.760	33.070	1.00	0.00
ATOM	13938	OW	SOL	4272	1.300	34.610	39.050	1.00	0.00
ATOM	13939	HW1	SOL	4272	1.510	34.780	39.960	1.00	0.00
ATOM	13940	HW2	SOL	4272	0.960	33.710	39.040	1.00	0.00
ATOM	13941	OW	SOL	4273	43.560	23.430	6.370	1.00	0.00
ATOM	13942	HW1	SOL	4273	43.390	22.490	6.410	1.00	0.00
ATOM	13943	HW2	SOL	4273	42.800	23.790	5.920	1.00	0.00
ATOM	13944	OW	SOL	4274	30.030	49.920	26.810	1.00	0.00
ATOM	13945	HW1	SOL	4274	29.140	50.260	26.740	1.00	0.00
ATOM	13946	HW2	SOL	4274	29.940	49.140	27.360	1.00	0.00
ATOM	13947	OW	SOL	4275	31.520	38.180	47.510	1.00	0.00

ATOM	13948	HW1	SOL	4275	31.820	38.120	46.600	1.00	0.00
ATOM	13949	HW2	SOL	4275	30.810	37.540	47.560	1.00	0.00
ATOM	13950	OW	SOL	4276	53.720	24.010	54.950	1.00	0.00
ATOM	13951	HW1	SOL	4276	54.170	23.700	55.730	1.00	0.00
ATOM	13952	HW2	SOL	4276	53.200	23.260	54.660	1.00	0.00
ATOM	13953	OW	SOL	4277	6.790	48.960	10.900	1.00	0.00
ATOM	13954	HW1	SOL	4277	5.840	48.980	11.040	1.00	0.00
ATOM	13955	HW2	SOL	4277	6.980	49.770	10.430	1.00	0.00
ATOM	13956	OW	SOL	4278	32.720	19.290	11.940	1.00	0.00
ATOM	13957	HW1	SOL	4278	32.720	18.910	12.810	1.00	0.00
ATOM	13958	HW2	SOL	4278	32.930	18.560	11.350	1.00	0.00
ATOM	13959	OW	SOL	4279	54.790	55.360	37.560	1.00	0.00
ATOM	13960	HW1	SOL	4279	54.890	54.950	36.700	1.00	0.00
ATOM	13961	HW2	SOL	4279	55.350	54.840	38.140	1.00	0.00
ATOM	13962	OW	SOL	4280	41.490	34.990	4.580	1.00	0.00
ATOM	13963	HW1	SOL	4280	41.050	34.890	5.420	1.00	0.00
ATOM	13964	HW2	SOL	4280	41.100	35.770	4.190	1.00	0.00
ATOM	13965	OW	SOL	4281	36.990	48.770	55.080	1.00	0.00
ATOM	13966	HW1	SOL	4281	37.150	48.080	55.720	1.00	0.00
ATOM	13967	HW2	SOL	4281	36.810	49.550	55.610	1.00	0.00
ATOM	13968	OW	SOL	4282	8.810	47.520	11.750	1.00	0.00
ATOM	13969	HW1	SOL	4282	8.940	47.490	12.700	1.00	0.00
ATOM	13970	HW2	SOL	4282	8.080	48.130	11.630	1.00	0.00
ATOM	13971	OW	SOL	4283	22.150	36.490	7.770	1.00	0.00
ATOM	13972	HW1	SOL	4283	22.470	37.390	7.850	1.00	0.00
ATOM	13973	HW2	SOL	4283	22.270	36.120	8.640	1.00	0.00
ATOM	13974	OW	SOL	4284	55.300	20.560	32.770	1.00	0.00
ATOM	13975	HW1	SOL	4284	55.260	19.920	32.060	1.00	0.00
ATOM	13976	HW2	SOL	4284	54.590	20.310	33.360	1.00	0.00
ATOM	13977	OW	SOL	4285	19.160	6.760	32.830	1.00	0.00
ATOM	13978	HW1	SOL	4285	19.850	7.410	32.670	1.00	0.00
ATOM	13979	HW2	SOL	4285	19.510	6.210	33.530	1.00	0.00
ATOM	13980	OW	SOL	4286	0.950	53.220	46.520	1.00	0.00
ATOM	13981	HW1	SOL	4286	1.290	52.330	46.560	1.00	0.00
ATOM	13982	HW2	SOL	4286	0.870	53.490	47.430	1.00	0.00
ATOM	13983	OW	SOL	4287	43.640	17.280	25.280	1.00	0.00
ATOM	13984	HW1	SOL	4287	43.280	17.040	24.430	1.00	0.00
ATOM	13985	HW2	SOL	4287	44.580	17.170	25.180	1.00	0.00
ATOM	13986	OW	SOL	4288	4.700	17.210	2.760	1.00	0.00
ATOM	13987	HW1	SOL	4288	4.230	16.380	2.680	1.00	0.00
ATOM	13988	HW2	SOL	4288	4.180	17.730	3.370	1.00	0.00
ATOM	13989	OW	SOL	4289	46.580	26.180	12.260	1.00	0.00
ATOM	13990	HW1	SOL	4289	47.180	26.890	12.020	1.00	0.00
ATOM	13991	HW2	SOL	4289	45.860	26.620	12.710	1.00	0.00

ATOM	13992	OW	SOL	4290	42.750	40.910	41.670	1.00	0.00
ATOM	13993	HW1	SOL	4290	42.800	40.430	40.840	1.00	0.00
ATOM	13994	HW2	SOL	4290	42.330	41.740	41.440	1.00	0.00
ATOM	13995	OW	SOL	4291	4.030	18.340	36.290	1.00	0.00
ATOM	13996	HW1	SOL	4291	4.930	18.010	36.200	1.00	0.00
ATOM	13997	HW2	SOL	4291	3.760	18.550	35.400	1.00	0.00
ATOM	13998	OW	SOL	4292	3.210	51.780	27.680	1.00	0.00
ATOM	13999	HW1	SOL	4292	2.670	51.120	27.240	1.00	0.00
ATOM	14000	HW2	SOL	4292	3.910	51.280	28.080	1.00	0.00
ATOM	14001	OW	SOL	4293	13.230	48.780	30.760	1.00	0.00
ATOM	14002	HW1	SOL	4293	13.160	49.690	30.450	1.00	0.00
ATOM	14003	HW2	SOL	4293	12.500	48.680	31.370	1.00	0.00
ATOM	14004	OW	SOL	4294	27.660	5.950	42.390	1.00	0.00
ATOM	14005	HW1	SOL	4294	28.190	5.850	41.600	1.00	0.00
ATOM	14006	HW2	SOL	4294	27.020	6.620	42.170	1.00	0.00
ATOM	14007	OW	SOL	4295	6.440	23.560	8.430	1.00	0.00
ATOM	14008	HW1	SOL	4295	6.690	22.920	9.100	1.00	0.00
ATOM	14009	HW2	SOL	4295	5.950	23.050	7.790	1.00	0.00
ATOM	14010	OW	SOL	4296	52.760	50.930	52.180	1.00	0.00
ATOM	14011	HW1	SOL	4296	52.670	51.400	51.350	1.00	0.00
ATOM	14012	HW2	SOL	4296	52.070	51.290	52.730	1.00	0.00
ATOM	14013	OW	SOL	4297	42.210	35.580	11.050	1.00	0.00
ATOM	14014	HW1	SOL	4297	43.080	35.880	11.300	1.00	0.00
ATOM	14015	HW2	SOL	4297	42.350	34.860	10.450	1.00	0.00
ATOM	14016	OW	SOL	4298	20.680	6.400	9.080	1.00	0.00
ATOM	14017	HW1	SOL	4298	21.140	7.210	9.280	1.00	0.00
ATOM	14018	HW2	SOL	4298	19.820	6.680	8.760	1.00	0.00
ATOM	14019	OW	SOL	4299	54.350	15.700	38.350	1.00	0.00
ATOM	14020	HW1	SOL	4299	54.740	15.980	37.520	1.00	0.00
ATOM	14021	HW2	SOL	4299	54.060	16.510	38.760	1.00	0.00
ATOM	14022	OW	SOL	4300	36.650	17.540	20.950	1.00	0.00
ATOM	14023	HW1	SOL	4300	36.830	17.800	21.850	1.00	0.00
ATOM	14024	HW2	SOL	4300	35.750	17.210	20.960	1.00	0.00
ATOM	14025	OW	SOL	4301	47.590	45.340	19.960	1.00	0.00
ATOM	14026	HW1	SOL	4301	46.890	44.780	19.630	1.00	0.00
ATOM	14027	HW2	SOL	4301	47.660	45.120	20.890	1.00	0.00
ATOM	14028	OW	SOL	4302	52.240	28.500	50.550	1.00	0.00
ATOM	14029	HW1	SOL	4302	53.020	28.230	51.030	1.00	0.00
ATOM	14030	HW2	SOL	4302	51.660	28.850	51.230	1.00	0.00
ATOM	14031	OW	SOL	4303	13.160	35.190	39.520	1.00	0.00
ATOM	14032	HW1	SOL	4303	14.010	34.840	39.250	1.00	0.00
ATOM	14033	HW2	SOL	4303	13.280	36.140	39.540	1.00	0.00
ATOM	14034	OW	SOL	4304	55.550	28.670	1.380	1.00	0.00
ATOM	14035	HW1	SOL	4304	55.190	29.510	1.670	1.00	0.00

ATOM	14036	HW2	SOL	4304	55.570	28.730	0.430	1.00	0.00
ATOM	14037	OW	SOL	4305	1.220	43.560	27.310	1.00	0.00
ATOM	14038	HW1	SOL	4305	0.310	43.480	27.030	1.00	0.00
ATOM	14039	HW2	SOL	4305	1.690	42.910	26.790	1.00	0.00
ATOM	14040	OW	SOL	4306	37.180	18.440	45.990	1.00	0.00
ATOM	14041	HW1	SOL	4306	37.080	17.650	46.520	1.00	0.00
ATOM	14042	HW2	SOL	4306	37.900	18.240	45.390	1.00	0.00
ATOM	14043	OW	SOL	4307	8.450	16.930	2.760	1.00	0.00
ATOM	14044	HW1	SOL	4307	8.180	16.030	2.900	1.00	0.00
ATOM	14045	HW2	SOL	4307	8.080	17.170	1.910	1.00	0.00
ATOM	14046	OW	SOL	4308	25.970	24.730	23.220	1.00	0.00
ATOM	14047	HW1	SOL	4308	25.610	24.140	23.880	1.00	0.00
ATOM	14048	HW2	SOL	4308	26.120	24.170	22.460	1.00	0.00
ATOM	14049	OW	SOL	4309	22.540	15.470	33.680	1.00	0.00
ATOM	14050	HW1	SOL	4309	22.840	15.290	34.570	1.00	0.00
ATOM	14051	HW2	SOL	4309	23.300	15.280	33.130	1.00	0.00
ATOM	14052	OW	SOL	4310	48.060	49.170	46.950	1.00	0.00
ATOM	14053	HW1	SOL	4310	47.400	49.200	47.640	1.00	0.00
ATOM	14054	HW2	SOL	4310	47.940	50.000	46.480	1.00	0.00
ATOM	14055	OW	SOL	4311	28.890	37.280	7.480	1.00	0.00
ATOM	14056	HW1	SOL	4311	28.540	36.460	7.100	1.00	0.00
ATOM	14057	HW2	SOL	4311	29.750	37.030	7.820	1.00	0.00
ATOM	14058	OW	SOL	4312	18.780	10.150	32.730	1.00	0.00
ATOM	14059	HW1	SOL	4312	19.440	9.960	33.390	1.00	0.00
ATOM	14060	HW2	SOL	4312	18.250	10.840	33.120	1.00	0.00
ATOM	14061	OW	SOL	4313	47.830	55.810	48.060	1.00	0.00
ATOM	14062	HW1	SOL	4313	47.700	56.460	47.380	1.00	0.00
ATOM	14063	HW2	SOL	4313	47.800	56.300	48.880	1.00	0.00
ATOM	14064	OW	SOL	4314	11.030	45.730	22.020	1.00	0.00
ATOM	14065	HW1	SOL	4314	11.190	46.480	21.450	1.00	0.00
ATOM	14066	HW2	SOL	4314	11.910	45.400	22.230	1.00	0.00
ATOM	14067	OW	SOL	4315	54.920	7.140	6.130	1.00	0.00
ATOM	14068	HW1	SOL	4315	55.060	6.950	5.200	1.00	0.00
ATOM	14069	HW2	SOL	4315	54.610	6.310	6.490	1.00	0.00
ATOM	14070	OW	SOL	4316	15.220	49.990	6.310	1.00	0.00
ATOM	14071	HW1	SOL	4316	15.950	49.400	6.510	1.00	0.00
ATOM	14072	HW2	SOL	4316	14.480	49.410	6.120	1.00	0.00
ATOM	14073	OW	SOL	4317	53.230	42.820	11.650	1.00	0.00
ATOM	14074	HW1	SOL	4317	53.590	43.480	12.250	1.00	0.00
ATOM	14075	HW2	SOL	4317	53.860	42.790	10.930	1.00	0.00
ATOM	14076	OW	SOL	4318	2.540	3.660	24.640	1.00	0.00
ATOM	14077	HW1	SOL	4318	1.600	3.780	24.530	1.00	0.00
ATOM	14078	HW2	SOL	4318	2.630	3.130	25.430	1.00	0.00
ATOM	14079	OW	SOL	4319	33.180	13.540	54.960	1.00	0.00

ATOM	14080	HW1	SOL	4319	33.900	12.910	55.080	1.00	0.00
ATOM	14081	HW2	SOL	4319	33.600	14.290	54.530	1.00	0.00
ATOM	14082	OW	SOL	4320	34.780	41.630	28.910	1.00	0.00
ATOM	14083	HW1	SOL	4320	35.090	40.940	28.320	1.00	0.00
ATOM	14084	HW2	SOL	4320	34.750	42.420	28.360	1.00	0.00
ATOM	14085	OW	SOL	4321	37.330	6.480	28.560	1.00	0.00
ATOM	14086	HW1	SOL	4321	36.980	5.720	29.020	1.00	0.00
ATOM	14087	HW2	SOL	4321	37.040	7.230	29.070	1.00	0.00
ATOM	14088	OW	SOL	4322	17.360	35.240	7.740	1.00	0.00
ATOM	14089	HW1	SOL	4322	18.320	35.250	7.660	1.00	0.00
ATOM	14090	HW2	SOL	4322	17.130	34.320	7.820	1.00	0.00
ATOM	14091	OW	SOL	4323	27.430	15.150	35.440	1.00	0.00
ATOM	14092	HW1	SOL	4323	27.970	14.660	36.060	1.00	0.00
ATOM	14093	HW2	SOL	4323	28.040	15.410	34.740	1.00	0.00
ATOM	14094	OW	SOL	4324	31.430	14.680	43.320	1.00	0.00
ATOM	14095	HW1	SOL	4324	31.320	13.890	43.850	1.00	0.00
ATOM	14096	HW2	SOL	4324	30.820	15.310	43.710	1.00	0.00
ATOM	14097	OW	SOL	4325	22.650	13.270	18.170	1.00	0.00
ATOM	14098	HW1	SOL	4325	22.590	13.840	18.940	1.00	0.00
ATOM	14099	HW2	SOL	4325	22.260	12.440	18.450	1.00	0.00
ATOM	14100	OW	SOL	4326	13.270	35.670	26.530	1.00	0.00
ATOM	14101	HW1	SOL	4326	12.810	36.410	26.930	1.00	0.00
ATOM	14102	HW2	SOL	4326	13.410	35.950	25.620	1.00	0.00
ATOM	14103	OW	SOL	4327	0.320	25.240	3.260	1.00	0.00
ATOM	14104	HW1	SOL	4327	-0.430	25.810	3.110	1.00	0.00
ATOM	14105	HW2	SOL	4327	0.710	25.570	4.070	1.00	0.00
ATOM	14106	OW	SOL	4328	24.900	45.660	47.180	1.00	0.00
ATOM	14107	HW1	SOL	4328	24.460	45.820	46.350	1.00	0.00
ATOM	14108	HW2	SOL	4328	24.950	44.700	47.240	1.00	0.00
ATOM	14109	OW	SOL	4329	51.020	34.190	14.800	1.00	0.00
ATOM	14110	HW1	SOL	4329	51.550	33.790	14.110	1.00	0.00
ATOM	14111	HW2	SOL	4329	51.400	35.060	14.920	1.00	0.00
ATOM	14112	OW	SOL	4330	46.420	51.410	11.830	1.00	0.00
ATOM	14113	HW1	SOL	4330	46.130	51.050	12.670	1.00	0.00
ATOM	14114	HW2	SOL	4330	46.250	50.720	11.200	1.00	0.00
ATOM	14115	OW	SOL	4331	11.530	28.040	21.450	1.00	0.00
ATOM	14116	HW1	SOL	4331	12.120	27.910	22.190	1.00	0.00
ATOM	14117	HW2	SOL	4331	10.650	27.950	21.820	1.00	0.00
ATOM	14118	OW	SOL	4332	17.100	30.020	24.660	1.00	0.00
ATOM	14119	HW1	SOL	4332	17.440	30.400	23.850	1.00	0.00
ATOM	14120	HW2	SOL	4332	17.050	30.750	25.280	1.00	0.00
ATOM	14121	OW	SOL	4333	49.350	25.140	0.650	1.00	0.00
ATOM	14122	HW1	SOL	4333	49.150	26.060	0.470	1.00	0.00
ATOM	14123	HW2	SOL	4333	48.510	24.690	0.570	1.00	0.00

ATOM	14124	OW	SOL	4334	28.370	2.330	28.400	1.00	0.00
ATOM	14125	HW1	SOL	4334	28.290	1.770	27.630	1.00	0.00
ATOM	14126	HW2	SOL	4334	29.270	2.220	28.690	1.00	0.00
ATOM	14127	OW	SOL	4335	11.170	14.830	29.610	1.00	0.00
ATOM	14128	HW1	SOL	4335	11.820	14.320	29.120	1.00	0.00
ATOM	14129	HW2	SOL	4335	11.170	15.690	29.180	1.00	0.00
ATOM	14130	OW	SOL	4336	45.120	42.480	49.170	1.00	0.00
ATOM	14131	HW1	SOL	4336	44.430	41.820	49.190	1.00	0.00
ATOM	14132	HW2	SOL	4336	45.900	42.030	49.480	1.00	0.00
ATOM	14133	OW	SOL	4337	10.050	48.220	27.760	1.00	0.00
ATOM	14134	HW1	SOL	4337	10.350	49.120	27.890	1.00	0.00
ATOM	14135	HW2	SOL	4337	10.170	47.790	28.600	1.00	0.00
ATOM	14136	OW	SOL	4338	42.170	17.670	1.590	1.00	0.00
ATOM	14137	HW1	SOL	4338	41.890	17.780	2.500	1.00	0.00
ATOM	14138	HW2	SOL	4338	41.790	16.830	1.320	1.00	0.00
ATOM	14139	OW	SOL	4339	48.940	1.840	10.610	1.00	0.00
ATOM	14140	HW1	SOL	4339	48.940	0.930	10.910	1.00	0.00
ATOM	14141	HW2	SOL	4339	48.190	1.900	10.010	1.00	0.00
ATOM	14142	OW	SOL	4340	25.080	39.530	43.560	1.00	0.00
ATOM	14143	HW1	SOL	4340	24.970	40.380	43.130	1.00	0.00
ATOM	14144	HW2	SOL	4340	24.820	38.890	42.900	1.00	0.00
ATOM	14145	OW	SOL	4341	34.750	55.550	3.260	1.00	0.00
ATOM	14146	HW1	SOL	4341	35.310	56.280	3.530	1.00	0.00
ATOM	14147	HW2	SOL	4341	33.970	55.970	2.900	1.00	0.00
ATOM	14148	OW	SOL	4342	32.200	54.690	24.000	1.00	0.00
ATOM	14149	HW1	SOL	4342	33.040	54.560	24.420	1.00	0.00
ATOM	14150	HW2	SOL	4342	32.050	53.900	23.490	1.00	0.00
ATOM	14151	OW	SOL	4343	14.660	24.000	17.540	1.00	0.00
ATOM	14152	HW1	SOL	4343	14.550	24.690	18.200	1.00	0.00
ATOM	14153	HW2	SOL	4343	13.770	23.680	17.390	1.00	0.00
ATOM	14154	OW	SOL	4344	21.570	49.060	42.070	1.00	0.00
ATOM	14155	HW1	SOL	4344	22.260	48.680	42.610	1.00	0.00
ATOM	14156	HW2	SOL	4344	22.020	49.680	41.500	1.00	0.00
ATOM	14157	OW	SOL	4345	54.420	35.780	38.210	1.00	0.00
ATOM	14158	HW1	SOL	4345	53.970	34.960	38.010	1.00	0.00
ATOM	14159	HW2	SOL	4345	55.350	35.540	38.260	1.00	0.00
ATOM	14160	OW	SOL	4346	11.100	35.640	11.150	1.00	0.00
ATOM	14161	HW1	SOL	4346	11.010	36.260	10.430	1.00	0.00
ATOM	14162	HW2	SOL	4346	11.980	35.290	11.050	1.00	0.00
ATOM	14163	OW	SOL	4347	5.440	12.080	34.730	1.00	0.00
ATOM	14164	HW1	SOL	4347	5.290	12.720	35.430	1.00	0.00
ATOM	14165	HW2	SOL	4347	5.720	11.290	35.190	1.00	0.00
ATOM	14166	OW	SOL	4348	48.330	45.180	29.600	1.00	0.00
ATOM	14167	HW1	SOL	4348	48.400	44.270	29.890	1.00	0.00

ATOM	14168	HW2	SOL	4348	47.910	45.630	30.330	1.00	0.00
ATOM	14169	OW	SOL	4349	33.170	44.510	36.950	1.00	0.00
ATOM	14170	HW1	SOL	4349	33.820	44.340	37.630	1.00	0.00
ATOM	14171	HW2	SOL	4349	32.900	45.410	37.090	1.00	0.00
ATOM	14172	OW	SOL	4350	51.920	13.250	52.540	1.00	0.00
ATOM	14173	HW1	SOL	4350	52.780	13.500	52.200	1.00	0.00
ATOM	14174	HW2	SOL	4350	51.430	12.960	51.770	1.00	0.00
ATOM	14175	OW	SOL	4351	15.920	45.350	2.340	1.00	0.00
ATOM	14176	HW1	SOL	4351	16.840	45.070	2.370	1.00	0.00
ATOM	14177	HW2	SOL	4351	15.880	45.960	1.610	1.00	0.00
ATOM	14178	OW	SOL	4352	6.400	29.980	46.030	1.00	0.00
ATOM	14179	HW1	SOL	4352	6.470	30.610	46.750	1.00	0.00
ATOM	14180	HW2	SOL	4352	7.030	30.280	45.380	1.00	0.00
ATOM	14181	OW	SOL	4353	23.090	54.940	42.630	1.00	0.00
ATOM	14182	HW1	SOL	4353	23.820	54.320	42.570	1.00	0.00
ATOM	14183	HW2	SOL	4353	23.110	55.250	43.540	1.00	0.00
ATOM	14184	OW	SOL	4354	12.220	38.050	53.170	1.00	0.00
ATOM	14185	HW1	SOL	4354	11.790	37.300	52.760	1.00	0.00
ATOM	14186	HW2	SOL	4354	11.850	38.080	54.050	1.00	0.00
ATOM	14187	OW	SOL	4355	42.830	24.780	10.810	1.00	0.00
ATOM	14188	HW1	SOL	4355	42.170	24.990	11.470	1.00	0.00
ATOM	14189	HW2	SOL	4355	42.380	24.910	9.970	1.00	0.00
ATOM	14190	OW	SOL	4356	24.680	16.700	55.400	1.00	0.00
ATOM	14191	HW1	SOL	4356	25.320	16.900	56.080	1.00	0.00
ATOM	14192	HW2	SOL	4356	23.880	16.480	55.880	1.00	0.00
ATOM	14193	OW	SOL	4357	24.200	2.330	17.930	1.00	0.00
ATOM	14194	HW1	SOL	4357	24.640	1.790	17.280	1.00	0.00
ATOM	14195	HW2	SOL	4357	24.070	1.760	18.680	1.00	0.00
ATOM	14196	OW	SOL	4358	0.610	47.120	0.750	1.00	0.00
ATOM	14197	HW1	SOL	4358	0.020	47.180	1.510	1.00	0.00
ATOM	14198	HW2	SOL	4358	0.440	47.920	0.260	1.00	0.00
ATOM	14199	OW	SOL	4359	5.650	45.350	45.000	1.00	0.00
ATOM	14200	HW1	SOL	4359	6.310	44.670	45.140	1.00	0.00
ATOM	14201	HW2	SOL	4359	6.150	46.160	44.880	1.00	0.00
ATOM	14202	OW	SOL	4360	38.660	42.190	21.950	1.00	0.00
ATOM	14203	HW1	SOL	4360	39.090	41.480	22.420	1.00	0.00
ATOM	14204	HW2	SOL	4360	39.210	42.960	22.110	1.00	0.00
ATOM	14205	OW	SOL	4361	14.510	3.990	6.340	1.00	0.00
ATOM	14206	HW1	SOL	4361	14.630	4.710	6.960	1.00	0.00
ATOM	14207	HW2	SOL	4361	14.180	4.410	5.550	1.00	0.00
ATOM	14208	OW	SOL	4362	46.560	54.430	43.040	1.00	0.00
ATOM	14209	HW1	SOL	4362	46.590	53.570	42.610	1.00	0.00
ATOM	14210	HW2	SOL	4362	46.390	54.230	43.960	1.00	0.00
ATOM	14211	OW	SOL	4363	13.620	8.600	20.990	1.00	0.00

ATOM	14212	HW1	SOL	4363	13.830	8.240	20.120	1.00	0.00
ATOM	14213	HW2	SOL	4363	12.740	8.270	21.180	1.00	0.00
ATOM	14214	OW	SOL	4364	53.620	28.330	4.980	1.00	0.00
ATOM	14215	HW1	SOL	4364	53.640	27.960	5.870	1.00	0.00
ATOM	14216	HW2	SOL	4364	53.270	29.210	5.100	1.00	0.00
ATOM	14217	OW	SOL	4365	23.970	49.470	26.770	1.00	0.00
ATOM	14218	HW1	SOL	4365	23.580	49.990	27.470	1.00	0.00
ATOM	14219	HW2	SOL	4365	23.950	48.570	27.100	1.00	0.00
ATOM	14220	OW	SOL	4366	31.010	30.190	12.160	1.00	0.00
ATOM	14221	HW1	SOL	4366	31.480	29.490	11.710	1.00	0.00
ATOM	14222	HW2	SOL	4366	31.570	30.400	12.910	1.00	0.00
ATOM	14223	OW	SOL	4367	11.160	19.240	33.050	1.00	0.00
ATOM	14224	HW1	SOL	4367	11.970	18.770	32.820	1.00	0.00
ATOM	14225	HW2	SOL	4367	11.110	19.960	32.430	1.00	0.00
ATOM	14226	OW	SOL	4368	15.990	48.100	38.660	1.00	0.00
ATOM	14227	HW1	SOL	4368	16.880	48.170	39.020	1.00	0.00
ATOM	14228	HW2	SOL	4368	15.440	47.900	39.420	1.00	0.00
ATOM	14229	OW	SOL	4369	42.070	29.330	53.740	1.00	0.00
ATOM	14230	HW1	SOL	4369	42.130	29.040	54.650	1.00	0.00
ATOM	14231	HW2	SOL	4369	42.590	30.130	53.700	1.00	0.00
ATOM	14232	OW	SOL	4370	40.820	21.410	53.880	1.00	0.00
ATOM	14233	HW1	SOL	4370	39.950	21.490	53.480	1.00	0.00
ATOM	14234	HW2	SOL	4370	40.860	20.510	54.210	1.00	0.00
ATOM	14235	OW	SOL	4371	51.630	23.940	22.170	1.00	0.00
ATOM	14236	HW1	SOL	4371	51.590	23.630	21.270	1.00	0.00
ATOM	14237	HW2	SOL	4371	52.450	24.420	22.230	1.00	0.00
ATOM	14238	OW	SOL	4372	35.450	26.250	40.210	1.00	0.00
ATOM	14239	HW1	SOL	4372	36.070	26.470	40.900	1.00	0.00
ATOM	14240	HW2	SOL	4372	35.830	25.480	39.780	1.00	0.00
ATOM	14241	OW	SOL	4373	38.070	20.780	47.180	1.00	0.00
ATOM	14242	HW1	SOL	4373	38.110	21.730	47.100	1.00	0.00
ATOM	14243	HW2	SOL	4373	37.200	20.550	46.870	1.00	0.00
ATOM	14244	OW	SOL	4374	12.510	44.780	9.660	1.00	0.00
ATOM	14245	HW1	SOL	4374	12.850	43.980	9.250	1.00	0.00
ATOM	14246	HW2	SOL	4374	11.830	44.480	10.250	1.00	0.00
ATOM	14247	OW	SOL	4375	0.300	39.220	36.230	1.00	0.00
ATOM	14248	HW1	SOL	4375	-0.630	39.070	36.380	1.00	0.00
ATOM	14249	HW2	SOL	4375	0.720	38.980	37.050	1.00	0.00
ATOM	14250	OW	SOL	4376	43.740	52.140	36.830	1.00	0.00
ATOM	14251	HW1	SOL	4376	44.540	52.210	37.340	1.00	0.00
ATOM	14252	HW2	SOL	4376	44.020	51.780	35.990	1.00	0.00
ATOM	14253	OW	SOL	4377	2.520	16.100	48.110	1.00	0.00
ATOM	14254	HW1	SOL	4377	1.700	16.260	47.640	1.00	0.00
ATOM	14255	HW2	SOL	4377	2.650	15.150	48.030	1.00	0.00

ATOM	14256	OW	SOL	4378	22.640	39.630	10.190	1.00	0.00
ATOM	14257	HW1	SOL	4378	21.960	39.770	10.840	1.00	0.00
ATOM	14258	HW2	SOL	4378	22.410	40.220	9.470	1.00	0.00
ATOM	14259	OW	SOL	4379	40.880	17.060	27.850	1.00	0.00
ATOM	14260	HW1	SOL	4379	41.460	17.060	28.610	1.00	0.00
ATOM	14261	HW2	SOL	4379	41.240	16.380	27.280	1.00	0.00
ATOM	14262	OW	SOL	4380	7.220	13.460	22.860	1.00	0.00
ATOM	14263	HW1	SOL	4380	7.850	12.750	22.920	1.00	0.00
ATOM	14264	HW2	SOL	4380	7.720	14.200	22.520	1.00	0.00
ATOM	14265	OW	SOL	4381	5.210	44.770	36.950	1.00	0.00
ATOM	14266	HW1	SOL	4381	5.620	45.590	36.650	1.00	0.00
ATOM	14267	HW2	SOL	4381	5.920	44.130	36.910	1.00	0.00
ATOM	14268	OW	SOL	4382	33.310	47.610	55.700	1.00	0.00
ATOM	14269	HW1	SOL	4382	33.930	47.650	54.970	1.00	0.00
ATOM	14270	HW2	SOL	4382	33.740	48.100	56.400	1.00	0.00
ATOM	14271	OW	SOL	4383	30.260	1.030	4.750	1.00	0.00
ATOM	14272	HW1	SOL	4383	29.900	0.830	5.610	1.00	0.00
ATOM	14273	HW2	SOL	4383	29.620	1.630	4.370	1.00	0.00
ATOM	14274	OW	SOL	4384	12.870	21.920	25.210	1.00	0.00
ATOM	14275	HW1	SOL	4384	13.790	22.130	25.390	1.00	0.00
ATOM	14276	HW2	SOL	4384	12.440	21.940	26.060	1.00	0.00
ATOM	14277	OW	SOL	4385	21.840	45.080	4.080	1.00	0.00
ATOM	14278	HW1	SOL	4385	21.420	44.220	4.130	1.00	0.00
ATOM	14279	HW2	SOL	4385	22.760	44.890	3.940	1.00	0.00
ATOM	14280	OW	SOL	4386	8.380	30.550	44.130	1.00	0.00
ATOM	14281	HW1	SOL	4386	8.480	29.690	43.730	1.00	0.00
ATOM	14282	HW2	SOL	4386	8.360	31.160	43.390	1.00	0.00
ATOM	14283	OW	SOL	4387	17.090	14.100	48.430	1.00	0.00
ATOM	14284	HW1	SOL	4387	16.480	14.190	49.160	1.00	0.00
ATOM	14285	HW2	SOL	4387	17.950	14.010	48.840	1.00	0.00
ATOM	14286	OW	SOL	4388	48.530	54.030	45.500	1.00	0.00
ATOM	14287	HW1	SOL	4388	47.950	54.570	46.030	1.00	0.00
ATOM	14288	HW2	SOL	4388	49.410	54.290	45.770	1.00	0.00
ATOM	14289	OW	SOL	4389	10.130	55.210	34.630	1.00	0.00
ATOM	14290	HW1	SOL	4389	9.220	55.070	34.370	1.00	0.00
ATOM	14291	HW2	SOL	4389	10.130	55.080	35.580	1.00	0.00
ATOM	14292	OW	SOL	4390	34.910	27.730	38.020	1.00	0.00
ATOM	14293	HW1	SOL	4390	34.090	28.210	37.870	1.00	0.00
ATOM	14294	HW2	SOL	4390	34.710	27.130	38.740	1.00	0.00
ATOM	14295	OW	SOL	4391	45.430	13.930	9.650	1.00	0.00
ATOM	14296	HW1	SOL	4391	45.110	13.590	10.490	1.00	0.00
ATOM	14297	HW2	SOL	4391	46.030	14.640	9.890	1.00	0.00
ATOM	14298	OW	SOL	4392	49.600	54.770	8.260	1.00	0.00
ATOM	14299	HW1	SOL	4392	49.320	55.360	7.560	1.00	0.00

ATOM	14300	HW2	SOL	4392	49.010	54.020	8.200	1.00	0.00
ATOM	14301	OW	SOL	4393	12.230	43.910	23.930	1.00	0.00
ATOM	14302	HW1	SOL	4393	11.430	43.670	24.410	1.00	0.00
ATOM	14303	HW2	SOL	4393	12.350	44.840	24.140	1.00	0.00
ATOM	14304	OW	SOL	4394	15.430	1.920	0.090	1.00	0.00
ATOM	14305	HW1	SOL	4394	16.310	2.260	0.230	1.00	0.00
ATOM	14306	HW2	SOL	4394	14.860	2.530	0.550	1.00	0.00
ATOM	14307	OW	SOL	4395	0.860	16.050	11.310	1.00	0.00
ATOM	14308	HW1	SOL	4395	0.180	16.720	11.390	1.00	0.00
ATOM	14309	HW2	SOL	4395	0.550	15.470	10.620	1.00	0.00
ATOM	14310	OW	SOL	4396	40.140	11.360	25.980	1.00	0.00
ATOM	14311	HW1	SOL	4396	40.670	12.160	25.940	1.00	0.00
ATOM	14312	HW2	SOL	4396	40.370	10.880	25.180	1.00	0.00
ATOM	14313	OW	SOL	4397	29.850	47.650	35.350	1.00	0.00
ATOM	14314	HW1	SOL	4397	29.360	46.860	35.580	1.00	0.00
ATOM	14315	HW2	SOL	4397	29.400	48.350	35.820	1.00	0.00
ATOM	14316	OW	SOL	4398	17.740	48.760	27.840	1.00	0.00
ATOM	14317	HW1	SOL	4398	17.190	49.020	27.110	1.00	0.00
ATOM	14318	HW2	SOL	4398	17.710	47.800	27.840	1.00	0.00
ATOM	14319	OW	SOL	4399	42.990	18.080	42.770	1.00	0.00
ATOM	14320	HW1	SOL	4399	43.330	18.910	42.460	1.00	0.00
ATOM	14321	HW2	SOL	4399	42.520	18.290	43.570	1.00	0.00
ATOM	14322	OW	SOL	4400	55.630	31.400	5.930	1.00	0.00
ATOM	14323	HW1	SOL	4400	56.320	31.090	5.340	1.00	0.00
ATOM	14324	HW2	SOL	4400	56.080	32.010	6.510	1.00	0.00
ATOM	14325	OW	SOL	4401	9.290	27.140	11.200	1.00	0.00
ATOM	14326	HW1	SOL	4401	8.880	27.940	11.520	1.00	0.00
ATOM	14327	HW2	SOL	4401	10.200	27.390	11.030	1.00	0.00
ATOM	14328	OW	SOL	4402	16.480	8.860	33.260	1.00	0.00
ATOM	14329	HW1	SOL	4402	17.340	9.170	32.990	1.00	0.00
ATOM	14330	HW2	SOL	4402	16.640	8.390	34.080	1.00	0.00
ATOM	14331	OW	SOL	4403	53.160	49.520	10.310	1.00	0.00
ATOM	14332	HW1	SOL	4403	53.610	49.890	11.070	1.00	0.00
ATOM	14333	HW2	SOL	4403	52.320	49.970	10.280	1.00	0.00
ATOM	14334	OW	SOL	4404	31.240	55.630	37.330	1.00	0.00
ATOM	14335	HW1	SOL	4404	30.770	56.080	38.030	1.00	0.00
ATOM	14336	HW2	SOL	4404	30.610	55.010	36.980	1.00	0.00
ATOM	14337	OW	SOL	4405	7.130	25.630	24.280	1.00	0.00
ATOM	14338	HW1	SOL	4405	6.460	25.900	24.900	1.00	0.00
ATOM	14339	HW2	SOL	4405	7.600	24.920	24.720	1.00	0.00
ATOM	14340	OW	SOL	4406	53.050	41.730	24.520	1.00	0.00
ATOM	14341	HW1	SOL	4406	52.620	40.880	24.500	1.00	0.00
ATOM	14342	HW2	SOL	4406	53.180	41.950	23.600	1.00	0.00
ATOM	14343	OW	SOL	4407	46.300	20.440	43.820	1.00	0.00

ATOM	14344	HW1	SOL	4407	45.580	20.400	43.180	1.00	0.00
ATOM	14345	HW2	SOL	4407	47.010	20.870	43.350	1.00	0.00
ATOM	14346	OW	SOL	4408	41.550	24.260	4.800	1.00	0.00
ATOM	14347	HW1	SOL	4408	41.530	25.110	4.370	1.00	0.00
ATOM	14348	HW2	SOL	4408	41.100	23.670	4.200	1.00	0.00
ATOM	14349	OW	SOL	4409	37.390	5.480	32.730	1.00	0.00
ATOM	14350	HW1	SOL	4409	37.910	4.690	32.580	1.00	0.00
ATOM	14351	HW2	SOL	4409	36.620	5.370	32.170	1.00	0.00
ATOM	14352	OW	SOL	4410	20.230	34.830	20.010	1.00	0.00
ATOM	14353	HW1	SOL	4410	20.680	34.620	19.200	1.00	0.00
ATOM	14354	HW2	SOL	4410	20.150	35.780	20.000	1.00	0.00
ATOM	14355	OW	SOL	4411	46.390	52.010	31.650	1.00	0.00
ATOM	14356	HW1	SOL	4411	47.280	52.350	31.730	1.00	0.00
ATOM	14357	HW2	SOL	4411	46.340	51.690	30.750	1.00	0.00
ATOM	14358	OW	SOL	4412	41.420	21.120	14.270	1.00	0.00
ATOM	14359	HW1	SOL	4412	40.620	20.830	14.710	1.00	0.00
ATOM	14360	HW2	SOL	4412	41.440	22.070	14.410	1.00	0.00
ATOM	14361	OW	SOL	4413	53.160	11.650	12.650	1.00	0.00
ATOM	14362	HW1	SOL	4413	53.500	12.490	12.340	1.00	0.00
ATOM	14363	HW2	SOL	4413	52.470	11.890	13.270	1.00	0.00
ATOM	14364	OW	SOL	4414	38.750	17.060	44.080	1.00	0.00
ATOM	14365	HW1	SOL	4414	39.300	16.700	44.780	1.00	0.00
ATOM	14366	HW2	SOL	4414	39.360	17.590	43.560	1.00	0.00
ATOM	14367	OW	SOL	4415	27.280	14.160	54.440	1.00	0.00
ATOM	14368	HW1	SOL	4415	27.900	13.520	54.790	1.00	0.00
ATOM	14369	HW2	SOL	4415	26.490	14.060	54.970	1.00	0.00
ATOM	14370	OW	SOL	4416	27.680	6.490	36.370	1.00	0.00
ATOM	14371	HW1	SOL	4416	27.060	7.210	36.370	1.00	0.00
ATOM	14372	HW2	SOL	4416	28.380	6.780	35.780	1.00	0.00
ATOM	14373	OW	SOL	4417	19.970	6.350	4.780	1.00	0.00
ATOM	14374	HW1	SOL	4417	19.970	5.500	5.220	1.00	0.00
ATOM	14375	HW2	SOL	4417	20.890	6.610	4.750	1.00	0.00
ATOM	14376	OW	SOL	4418	8.870	31.610	33.850	1.00	0.00
ATOM	14377	HW1	SOL	4418	8.810	32.200	33.100	1.00	0.00
ATOM	14378	HW2	SOL	4418	9.040	30.750	33.480	1.00	0.00
ATOM	14379	OW	SOL	4419	5.630	51.300	49.400	1.00	0.00
ATOM	14380	HW1	SOL	4419	6.200	51.690	50.070	1.00	0.00
ATOM	14381	HW2	SOL	4419	6.220	51.100	48.670	1.00	0.00
ATOM	14382	OW	SOL	4420	10.050	7.530	50.480	1.00	0.00
ATOM	14383	HW1	SOL	4420	9.920	7.470	51.420	1.00	0.00
ATOM	14384	HW2	SOL	4420	10.740	6.890	50.290	1.00	0.00
ATOM	14385	OW	SOL	4421	43.810	24.380	29.650	1.00	0.00
ATOM	14386	HW1	SOL	4421	43.700	23.890	30.470	1.00	0.00
ATOM	14387	HW2	SOL	4421	43.990	23.720	28.990	1.00	0.00

ATOM	14388	OW	SOL	4422	43.840	20.250	33.350	1.00	0.00
ATOM	14389	HW1	SOL	4422	44.720	20.230	32.960	1.00	0.00
ATOM	14390	HW2	SOL	4422	43.980	20.580	34.240	1.00	0.00
ATOM	14391	OW	SOL	4423	39.310	29.130	2.910	1.00	0.00
ATOM	14392	HW1	SOL	4423	38.790	28.630	3.540	1.00	0.00
ATOM	14393	HW2	SOL	4423	40.190	28.760	2.980	1.00	0.00
ATOM	14394	OW	SOL	4424	8.700	11.270	12.990	1.00	0.00
ATOM	14395	HW1	SOL	4424	7.770	11.240	13.190	1.00	0.00
ATOM	14396	HW2	SOL	4424	8.760	11.100	12.050	1.00	0.00
ATOM	14397	OW	SOL	4425	43.800	4.460	31.150	1.00	0.00
ATOM	14398	HW1	SOL	4425	43.770	4.160	32.060	1.00	0.00
ATOM	14399	HW2	SOL	4425	44.310	5.270	31.190	1.00	0.00
ATOM	14400	OW	SOL	4426	24.190	26.560	51.760	1.00	0.00
ATOM	14401	HW1	SOL	4426	25.000	26.090	51.600	1.00	0.00
ATOM	14402	HW2	SOL	4426	24.120	26.610	52.710	1.00	0.00
ATOM	14403	OW	SOL	4427	24.640	31.990	4.420	1.00	0.00
ATOM	14404	HW1	SOL	4427	23.880	31.430	4.560	1.00	0.00
ATOM	14405	HW2	SOL	4427	25.080	31.620	3.660	1.00	0.00
ATOM	14406	OW	SOL	4428	55.410	11.010	42.960	1.00	0.00
ATOM	14407	HW1	SOL	4428	56.100	11.280	43.570	1.00	0.00
ATOM	14408	HW2	SOL	4428	55.830	10.400	42.370	1.00	0.00
ATOM	14409	OW	SOL	4429	41.740	26.990	12.400	1.00	0.00
ATOM	14410	HW1	SOL	4429	41.180	27.070	13.180	1.00	0.00
ATOM	14411	HW2	SOL	4429	41.300	27.500	11.730	1.00	0.00
ATOM	14412	OW	SOL	4430	50.140	8.580	47.200	1.00	0.00
ATOM	14413	HW1	SOL	4430	50.380	7.700	47.500	1.00	0.00
ATOM	14414	HW2	SOL	4430	50.440	8.620	46.290	1.00	0.00
ATOM	14415	OW	SOL	4431	18.720	53.060	11.480	1.00	0.00
ATOM	14416	HW1	SOL	4431	19.280	53.450	10.800	1.00	0.00
ATOM	14417	HW2	SOL	4431	18.410	53.810	11.980	1.00	0.00
ATOM	14418	OW	SOL	4432	32.680	37.140	49.830	1.00	0.00
ATOM	14419	HW1	SOL	4432	31.820	36.990	50.220	1.00	0.00
ATOM	14420	HW2	SOL	4432	32.490	37.460	48.950	1.00	0.00
ATOM	14421	OW	SOL	4433	39.450	51.830	14.800	1.00	0.00
ATOM	14422	HW1	SOL	4433	40.020	52.360	15.370	1.00	0.00
ATOM	14423	HW2	SOL	4433	39.210	52.420	14.090	1.00	0.00
ATOM	14424	OW	SOL	4434	37.950	22.650	25.590	1.00	0.00
ATOM	14425	HW1	SOL	4434	38.400	22.260	26.330	1.00	0.00
ATOM	14426	HW2	SOL	4434	38.450	22.370	24.820	1.00	0.00
ATOM	14427	OW	SOL	4435	25.750	35.940	44.660	1.00	0.00
ATOM	14428	HW1	SOL	4435	26.390	35.420	45.140	1.00	0.00
ATOM	14429	HW2	SOL	4435	25.950	36.840	44.910	1.00	0.00
ATOM	14430	OW	SOL	4436	50.510	0.380	27.180	1.00	0.00
ATOM	14431	HW1	SOL	4436	51.190	1.010	27.400	1.00	0.00

ATOM	14432	HW2	SOL	4436	50.420	0.440	26.230	1.00	0.00
ATOM	14433	OW	SOL	4437	40.120	53.660	54.360	1.00	0.00
ATOM	14434	HW1	SOL	4437	39.700	54.250	53.750	1.00	0.00
ATOM	14435	HW2	SOL	4437	40.250	54.180	55.160	1.00	0.00
ATOM	14436	OW	SOL	4438	21.080	32.390	41.190	1.00	0.00
ATOM	14437	HW1	SOL	4438	20.630	32.600	42.010	1.00	0.00
ATOM	14438	HW2	SOL	4438	21.950	32.780	41.270	1.00	0.00
ATOM	14439	OW	SOL	4439	17.880	23.250	31.400	1.00	0.00
ATOM	14440	HW1	SOL	4439	17.110	23.000	31.910	1.00	0.00
ATOM	14441	HW2	SOL	4439	18.130	24.110	31.750	1.00	0.00
ATOM	14442	OW	SOL	4440	51.660	25.750	24.320	1.00	0.00
ATOM	14443	HW1	SOL	4440	51.550	25.190	23.550	1.00	0.00
ATOM	14444	HW2	SOL	4440	51.070	26.490	24.170	1.00	0.00
ATOM	14445	OW	SOL	4441	27.810	50.940	21.220	1.00	0.00
ATOM	14446	HW1	SOL	4441	27.570	51.760	21.660	1.00	0.00
ATOM	14447	HW2	SOL	4441	28.610	51.160	20.740	1.00	0.00
ATOM	14448	OW	SOL	4442	36.760	37.730	24.340	1.00	0.00
ATOM	14449	HW1	SOL	4442	37.390	38.190	24.910	1.00	0.00
ATOM	14450	HW2	SOL	4442	36.540	38.370	23.670	1.00	0.00
ATOM	14451	OW	SOL	4443	3.200	46.150	43.000	1.00	0.00
ATOM	14452	HW1	SOL	4443	2.440	45.990	43.560	1.00	0.00
ATOM	14453	HW2	SOL	4443	3.950	45.900	43.540	1.00	0.00
ATOM	14454	OW	SOL	4444	32.870	8.850	32.960	1.00	0.00
ATOM	14455	HW1	SOL	4444	33.100	9.650	33.440	1.00	0.00
ATOM	14456	HW2	SOL	4444	33.400	8.900	32.160	1.00	0.00
ATOM	14457	OW	SOL	4445	48.250	1.210	28.890	1.00	0.00
ATOM	14458	HW1	SOL	4445	47.810	1.800	28.270	1.00	0.00
ATOM	14459	HW2	SOL	4445	49.070	0.970	28.440	1.00	0.00
ATOM	14460	OW	SOL	4446	10.900	18.310	39.790	1.00	0.00
ATOM	14461	HW1	SOL	4446	10.840	18.500	38.860	1.00	0.00
ATOM	14462	HW2	SOL	4446	10.180	17.690	39.960	1.00	0.00
ATOM	14463	OW	SOL	4447	27.240	48.880	46.280	1.00	0.00
ATOM	14464	HW1	SOL	4447	26.750	49.550	46.760	1.00	0.00
ATOM	14465	HW2	SOL	4447	26.860	48.900	45.400	1.00	0.00
ATOM	14466	OW	SOL	4448	42.890	54.950	27.600	1.00	0.00
ATOM	14467	HW1	SOL	4448	42.680	55.870	27.740	1.00	0.00
ATOM	14468	HW2	SOL	4448	43.310	54.930	26.740	1.00	0.00
ATOM	14469	OW	SOL	4449	2.190	3.330	11.460	1.00	0.00
ATOM	14470	HW1	SOL	4449	1.790	4.090	11.880	1.00	0.00
ATOM	14471	HW2	SOL	4449	1.480	2.690	11.410	1.00	0.00
ATOM	14472	OW	SOL	4450	36.680	51.570	43.160	1.00	0.00
ATOM	14473	HW1	SOL	4450	37.630	51.630	43.170	1.00	0.00
ATOM	14474	HW2	SOL	4450	36.380	52.350	43.630	1.00	0.00
ATOM	14475	OW	SOL	4451	14.080	26.140	2.390	1.00	0.00

ATOM	14476	HW1	SOL	4451	14.950	25.790	2.200	1.00	0.00
ATOM	14477	HW2	SOL	4451	13.620	25.430	2.830	1.00	0.00
ATOM	14478	OW	SOL	4452	29.450	32.370	13.490	1.00	0.00
ATOM	14479	HW1	SOL	4452	29.860	31.800	12.840	1.00	0.00
ATOM	14480	HW2	SOL	4452	28.660	32.690	13.070	1.00	0.00
ATOM	14481	OW	SOL	4453	6.010	6.280	29.970	1.00	0.00
ATOM	14482	HW1	SOL	4453	5.480	5.610	30.410	1.00	0.00
ATOM	14483	HW2	SOL	4453	6.610	6.590	30.640	1.00	0.00
ATOM	14484	OW	SOL	4454	31.870	54.540	7.920	1.00	0.00
ATOM	14485	HW1	SOL	4454	32.250	55.200	7.340	1.00	0.00
ATOM	14486	HW2	SOL	4454	32.220	54.750	8.780	1.00	0.00
ATOM	14487	OW	SOL	4455	47.410	28.460	7.300	1.00	0.00
ATOM	14488	HW1	SOL	4455	48.200	28.500	7.830	1.00	0.00
ATOM	14489	HW2	SOL	4455	46.780	27.990	7.840	1.00	0.00
ATOM	14490	OW	SOL	4456	29.700	35.070	17.170	1.00	0.00
ATOM	14491	HW1	SOL	4456	28.840	34.900	16.790	1.00	0.00
ATOM	14492	HW2	SOL	4456	30.000	34.210	17.470	1.00	0.00
ATOM	14493	OW	SOL	4457	18.900	42.010	0.740	1.00	0.00
ATOM	14494	HW1	SOL	4457	19.740	41.560	0.860	1.00	0.00
ATOM	14495	HW2	SOL	4457	19.070	42.630	0.030	1.00	0.00
ATOM	14496	OW	SOL	4458	24.090	39.900	16.580	1.00	0.00
ATOM	14497	HW1	SOL	4458	24.470	39.300	15.940	1.00	0.00
ATOM	14498	HW2	SOL	4458	23.800	40.650	16.070	1.00	0.00
ATOM	14499	OW	SOL	4459	27.860	11.470	36.380	1.00	0.00
ATOM	14500	HW1	SOL	4459	27.810	10.890	35.610	1.00	0.00
ATOM	14501	HW2	SOL	4459	28.520	12.120	36.150	1.00	0.00
ATOM	14502	OW	SOL	4460	44.330	46.290	34.730	1.00	0.00
ATOM	14503	HW1	SOL	4460	44.630	47.170	34.950	1.00	0.00
ATOM	14504	HW2	SOL	4460	43.620	46.430	34.100	1.00	0.00
ATOM	14505	OW	SOL	4461	8.010	13.820	15.690	1.00	0.00
ATOM	14506	HW1	SOL	4461	8.590	13.060	15.590	1.00	0.00
ATOM	14507	HW2	SOL	4461	7.680	13.970	14.810	1.00	0.00
ATOM	14508	OW	SOL	4462	19.030	4.070	24.290	1.00	0.00
ATOM	14509	HW1	SOL	4462	18.530	3.780	23.530	1.00	0.00
ATOM	14510	HW2	SOL	4462	18.430	3.970	25.030	1.00	0.00
ATOM	14511	OW	SOL	4463	54.990	14.000	48.630	1.00	0.00
ATOM	14512	HW1	SOL	4463	55.020	13.040	48.660	1.00	0.00
ATOM	14513	HW2	SOL	4463	55.220	14.210	47.720	1.00	0.00
ATOM	14514	OW	SOL	4464	5.730	34.100	6.180	1.00	0.00
ATOM	14515	HW1	SOL	4464	5.840	33.590	5.380	1.00	0.00
ATOM	14516	HW2	SOL	4464	4.820	33.940	6.440	1.00	0.00
ATOM	14517	OW	SOL	4465	12.910	7.960	3.030	1.00	0.00
ATOM	14518	HW1	SOL	4465	12.340	8.280	3.730	1.00	0.00
ATOM	14519	HW2	SOL	4465	12.340	7.920	2.260	1.00	0.00

ATOM	14520	OW	SOL	4466	46.910	6.810	34.370	1.00	0.00
ATOM	14521	HW1	SOL	4466	47.380	6.540	35.160	1.00	0.00
ATOM	14522	HW2	SOL	4466	47.310	6.310	33.660	1.00	0.00
ATOM	14523	OW	SOL	4467	47.810	54.110	20.210	1.00	0.00
ATOM	14524	HW1	SOL	4467	47.860	55.020	19.930	1.00	0.00
ATOM	14525	HW2	SOL	4467	47.300	54.130	21.010	1.00	0.00
ATOM	14526	OW	SOL	4468	18.250	19.730	37.260	1.00	0.00
ATOM	14527	HW1	SOL	4468	18.000	19.610	38.170	1.00	0.00
ATOM	14528	HW2	SOL	4468	17.600	19.230	36.760	1.00	0.00
ATOM	14529	OW	SOL	4469	10.660	31.620	26.930	1.00	0.00
ATOM	14530	HW1	SOL	4469	11.550	31.590	27.290	1.00	0.00
ATOM	14531	HW2	SOL	4469	10.330	32.480	27.170	1.00	0.00
ATOM	14532	OW	SOL	4470	30.100	13.610	34.540	1.00	0.00
ATOM	14533	HW1	SOL	4470	30.350	14.520	34.710	1.00	0.00
ATOM	14534	HW2	SOL	4470	30.750	13.310	33.900	1.00	0.00
ATOM	14535	OW	SOL	4471	25.960	41.950	53.940	1.00	0.00
ATOM	14536	HW1	SOL	4471	25.450	42.610	54.410	1.00	0.00
ATOM	14537	HW2	SOL	4471	25.920	41.170	54.500	1.00	0.00
ATOM	14538	OW	SOL	4472	31.690	44.200	10.830	1.00	0.00
ATOM	14539	HW1	SOL	4472	31.390	43.660	10.100	1.00	0.00
ATOM	14540	HW2	SOL	4472	30.890	44.430	11.300	1.00	0.00
ATOM	14541	OW	SOL	4473	20.010	26.920	0.610	1.00	0.00
ATOM	14542	HW1	SOL	4473	20.370	26.670	-0.240	1.00	0.00
ATOM	14543	HW2	SOL	4473	19.930	26.090	1.090	1.00	0.00
ATOM	14544	OW	SOL	4474	33.730	20.660	30.020	1.00	0.00
ATOM	14545	HW1	SOL	4474	34.500	20.800	30.570	1.00	0.00
ATOM	14546	HW2	SOL	4474	33.200	20.020	30.490	1.00	0.00
ATOM	14547	OW	SOL	4475	19.720	15.380	34.200	1.00	0.00
ATOM	14548	HW1	SOL	4475	20.530	15.300	33.700	1.00	0.00
ATOM	14549	HW2	SOL	4475	20.010	15.540	35.100	1.00	0.00
ATOM	14550	OW	SOL	4476	42.140	10.210	23.990	1.00	0.00
ATOM	14551	HW1	SOL	4476	42.670	9.410	23.910	1.00	0.00
ATOM	14552	HW2	SOL	4476	41.700	10.280	23.150	1.00	0.00
ATOM	14553	OW	SOL	4477	32.080	9.580	12.080	1.00	0.00
ATOM	14554	HW1	SOL	4477	32.790	9.090	11.650	1.00	0.00
ATOM	14555	HW2	SOL	4477	32.400	9.740	12.970	1.00	0.00
ATOM	14556	OW	SOL	4478	42.490	51.980	47.840	1.00	0.00
ATOM	14557	HW1	SOL	4478	41.610	52.350	47.980	1.00	0.00
ATOM	14558	HW2	SOL	4478	42.350	51.240	47.270	1.00	0.00
ATOM	14559	OW	SOL	4479	5.710	32.390	37.990	1.00	0.00
ATOM	14560	HW1	SOL	4479	6.020	32.820	38.790	1.00	0.00
ATOM	14561	HW2	SOL	4479	6.040	32.930	37.280	1.00	0.00
ATOM	14562	OW	SOL	4480	17.830	46.030	12.590	1.00	0.00
ATOM	14563	HW1	SOL	4480	17.180	46.450	12.020	1.00	0.00

ATOM	14564	HW2	SOL	4480	17.910	45.150	12.240	1.00	0.00
ATOM	14565	OW	SOL	4481	13.470	32.560	9.150	1.00	0.00
ATOM	14566	HW1	SOL	4481	13.590	32.120	9.990	1.00	0.00
ATOM	14567	HW2	SOL	4481	12.910	31.970	8.650	1.00	0.00
ATOM	14568	OW	SOL	4482	28.770	17.330	50.000	1.00	0.00
ATOM	14569	HW1	SOL	4482	28.400	18.210	50.000	1.00	0.00
ATOM	14570	HW2	SOL	4482	28.030	16.760	50.220	1.00	0.00
ATOM	14571	OW	SOL	4483	33.460	45.780	30.270	1.00	0.00
ATOM	14572	HW1	SOL	4483	32.860	45.070	30.490	1.00	0.00
ATOM	14573	HW2	SOL	4483	32.890	46.550	30.180	1.00	0.00
ATOM	14574	OW	SOL	4484	8.530	22.100	51.430	1.00	0.00
ATOM	14575	HW1	SOL	4484	9.000	21.400	51.880	1.00	0.00
ATOM	14576	HW2	SOL	4484	8.850	22.070	50.530	1.00	0.00
ATOM	14577	OW	SOL	4485	17.460	53.750	46.060	1.00	0.00
ATOM	14578	HW1	SOL	4485	18.040	53.980	46.790	1.00	0.00
ATOM	14579	HW2	SOL	4485	18.020	53.240	45.470	1.00	0.00
ATOM	14580	OW	SOL	4486	25.460	1.300	35.100	1.00	0.00
ATOM	14581	HW1	SOL	4486	26.130	0.660	35.360	1.00	0.00
ATOM	14582	HW2	SOL	4486	25.920	2.150	35.130	1.00	0.00
ATOM	14583	OW	SOL	4487	17.120	38.130	38.080	1.00	0.00
ATOM	14584	HW1	SOL	4487	17.440	37.860	38.940	1.00	0.00
ATOM	14585	HW2	SOL	4487	16.580	38.900	38.250	1.00	0.00
ATOM	14586	OW	SOL	4488	8.130	43.750	9.760	1.00	0.00
ATOM	14587	HW1	SOL	4488	7.930	44.670	9.590	1.00	0.00
ATOM	14588	HW2	SOL	4488	8.310	43.710	10.700	1.00	0.00
ATOM	14589	OW	SOL	4489	9.890	31.860	12.770	1.00	0.00
ATOM	14590	HW1	SOL	4489	10.840	31.750	12.770	1.00	0.00
ATOM	14591	HW2	SOL	4489	9.590	31.350	12.020	1.00	0.00
ATOM	14592	OW	SOL	4490	53.260	46.650	0.250	1.00	0.00
ATOM	14593	HW1	SOL	4490	53.720	46.410	1.060	1.00	0.00
ATOM	14594	HW2	SOL	4490	52.330	46.620	0.480	1.00	0.00
ATOM	14595	OW	SOL	4491	10.950	38.730	32.180	1.00	0.00
ATOM	14596	HW1	SOL	4491	11.580	38.650	32.890	1.00	0.00
ATOM	14597	HW2	SOL	4491	11.460	38.630	31.390	1.00	0.00
ATOM	14598	OW	SOL	4492	13.820	11.680	42.640	1.00	0.00
ATOM	14599	HW1	SOL	4492	12.870	11.790	42.620	1.00	0.00
ATOM	14600	HW2	SOL	4492	14.070	11.860	43.540	1.00	0.00
ATOM	14601	OW	SOL	4493	7.960	13.900	29.180	1.00	0.00
ATOM	14602	HW1	SOL	4493	8.050	13.930	30.130	1.00	0.00
ATOM	14603	HW2	SOL	4493	7.710	14.790	28.930	1.00	0.00
ATOM	14604	OW	SOL	4494	20.420	51.070	26.670	1.00	0.00
ATOM	14605	HW1	SOL	4494	20.030	51.820	26.220	1.00	0.00
ATOM	14606	HW2	SOL	4494	20.850	50.580	25.970	1.00	0.00
ATOM	14607	OW	SOL	4495	44.220	6.640	46.040	1.00	0.00

ATOM	14608	HW1	SOL	4495	43.400	7.120	45.960	1.00	0.00
ATOM	14609	HW2	SOL	4495	44.560	6.880	46.900	1.00	0.00
ATOM	14610	OW	SOL	4496	28.760	44.610	15.800	1.00	0.00
ATOM	14611	HW1	SOL	4496	28.580	43.700	16.020	1.00	0.00
ATOM	14612	HW2	SOL	4496	28.150	44.810	15.100	1.00	0.00
ATOM	14613	OW	SOL	4497	52.420	24.820	39.350	1.00	0.00
ATOM	14614	HW1	SOL	4497	51.550	25.210	39.350	1.00	0.00
ATOM	14615	HW2	SOL	4497	52.340	24.050	39.910	1.00	0.00
ATOM	14616	OW	SOL	4498	4.370	37.230	12.900	1.00	0.00
ATOM	14617	HW1	SOL	4498	4.290	37.380	13.840	1.00	0.00
ATOM	14618	HW2	SOL	4498	5.280	37.440	12.690	1.00	0.00
ATOM	14619	OW	SOL	4499	33.770	54.740	29.420	1.00	0.00
ATOM	14620	HW1	SOL	4499	32.870	55.000	29.230	1.00	0.00
ATOM	14621	HW2	SOL	4499	33.920	55.010	30.330	1.00	0.00
ATOM	14622	OW	SOL	4500	26.360	49.820	7.950	1.00	0.00
ATOM	14623	HW1	SOL	4500	25.630	49.240	7.740	1.00	0.00
ATOM	14624	HW2	SOL	4500	27.130	49.400	7.550	1.00	0.00
ATOM	14625	OW	SOL	4501	12.470	19.760	17.460	1.00	0.00
ATOM	14626	HW1	SOL	4501	12.010	18.930	17.580	1.00	0.00
ATOM	14627	HW2	SOL	4501	12.620	19.810	16.510	1.00	0.00
ATOM	14628	OW	SOL	4502	37.750	27.620	23.110	1.00	0.00
ATOM	14629	HW1	SOL	4502	37.380	27.570	23.990	1.00	0.00
ATOM	14630	HW2	SOL	4502	38.240	28.450	23.100	1.00	0.00
ATOM	14631	OW	SOL	4503	1.840	26.390	5.370	1.00	0.00
ATOM	14632	HW1	SOL	4503	1.600	26.580	6.280	1.00	0.00
ATOM	14633	HW2	SOL	4503	2.480	25.680	5.430	1.00	0.00
ATOM	14634	OW	SOL	4504	36.620	25.230	20.910	1.00	0.00
ATOM	14635	HW1	SOL	4504	35.720	24.960	21.100	1.00	0.00
ATOM	14636	HW2	SOL	4504	36.950	25.550	21.750	1.00	0.00
ATOM	14637	OW	SOL	4505	12.010	5.390	41.260	1.00	0.00
ATOM	14638	HW1	SOL	4505	12.840	5.310	40.800	1.00	0.00
ATOM	14639	HW2	SOL	4505	11.420	5.800	40.620	1.00	0.00
ATOM	14640	OW	SOL	4506	34.370	40.430	6.240	1.00	0.00
ATOM	14641	HW1	SOL	4506	33.470	40.300	5.950	1.00	0.00
ATOM	14642	HW2	SOL	4506	34.880	39.830	5.690	1.00	0.00
ATOM	14643	OW	SOL	4507	22.690	55.490	39.700	1.00	0.00
ATOM	14644	HW1	SOL	4507	22.660	55.320	40.640	1.00	0.00
ATOM	14645	HW2	SOL	4507	23.590	55.780	39.540	1.00	0.00
ATOM	14646	OW	SOL	4508	5.530	50.610	44.890	1.00	0.00
ATOM	14647	HW1	SOL	4508	5.050	50.170	44.180	1.00	0.00
ATOM	14648	HW2	SOL	4508	5.390	51.540	44.720	1.00	0.00
ATOM	14649	OW	SOL	4509	24.880	24.320	48.530	1.00	0.00
ATOM	14650	HW1	SOL	4509	24.160	23.700	48.620	1.00	0.00
ATOM	14651	HW2	SOL	4509	24.620	25.060	49.080	1.00	0.00

ATOM	14652	OW	SOL	4510	37.220	1.920	19.520	1.00	0.00
ATOM	14653	HW1	SOL	4510	37.180	2.400	18.700	1.00	0.00
ATOM	14654	HW2	SOL	4510	37.880	2.390	20.040	1.00	0.00
ATOM	14655	OW	SOL	4511	18.530	14.380	10.580	1.00	0.00
ATOM	14656	HW1	SOL	4511	17.800	14.550	11.170	1.00	0.00
ATOM	14657	HW2	SOL	4511	19.300	14.700	11.040	1.00	0.00
ATOM	14658	OW	SOL	4512	14.660	4.290	48.530	1.00	0.00
ATOM	14659	HW1	SOL	4512	15.080	5.100	48.230	1.00	0.00
ATOM	14660	HW2	SOL	4512	13.880	4.580	49.000	1.00	0.00
ATOM	14661	OW	SOL	4513	3.200	22.040	32.680	1.00	0.00
ATOM	14662	HW1	SOL	4513	3.680	22.330	31.910	1.00	0.00
ATOM	14663	HW2	SOL	4513	2.400	22.570	32.690	1.00	0.00
ATOM	14664	OW	SOL	4514	6.350	14.090	50.480	1.00	0.00
ATOM	14665	HW1	SOL	4514	5.540	13.640	50.700	1.00	0.00
ATOM	14666	HW2	SOL	4514	6.400	14.060	49.530	1.00	0.00
ATOM	14667	OW	SOL	4515	8.910	28.280	42.900	1.00	0.00
ATOM	14668	HW1	SOL	4515	9.670	27.990	42.400	1.00	0.00
ATOM	14669	HW2	SOL	4515	8.170	28.190	42.310	1.00	0.00
ATOM	14670	OW	SOL	4516	5.100	48.590	19.780	1.00	0.00
ATOM	14671	HW1	SOL	4516	5.960	48.890	20.070	1.00	0.00
ATOM	14672	HW2	SOL	4516	4.510	49.320	19.960	1.00	0.00
ATOM	14673	OW	SOL	4517	8.970	26.330	5.330	1.00	0.00
ATOM	14674	HW1	SOL	4517	8.690	26.090	6.220	1.00	0.00
ATOM	14675	HW2	SOL	4517	8.490	25.720	4.770	1.00	0.00
ATOM	14676	OW	SOL	4518	15.190	10.470	12.190	1.00	0.00
ATOM	14677	HW1	SOL	4518	15.510	9.720	12.680	1.00	0.00
ATOM	14678	HW2	SOL	4518	15.600	11.230	12.610	1.00	0.00
ATOM	14679	OW	SOL	4519	7.920	16.580	30.490	1.00	0.00
ATOM	14680	HW1	SOL	4519	7.020	16.900	30.540	1.00	0.00
ATOM	14681	HW2	SOL	4519	8.360	16.970	31.250	1.00	0.00
ATOM	14682	OW	SOL	4520	38.150	1.550	31.730	1.00	0.00
ATOM	14683	HW1	SOL	4520	38.360	2.480	31.700	1.00	0.00
ATOM	14684	HW2	SOL	4520	38.990	1.120	31.910	1.00	0.00
ATOM	14685	OW	SOL	4521	14.800	31.660	31.640	1.00	0.00
ATOM	14686	HW1	SOL	4521	15.180	31.000	31.060	1.00	0.00
ATOM	14687	HW2	SOL	4521	13.960	31.890	31.240	1.00	0.00
ATOM	14688	OW	SOL	4522	19.610	27.790	52.490	1.00	0.00
ATOM	14689	HW1	SOL	4522	19.330	28.640	52.160	1.00	0.00
ATOM	14690	HW2	SOL	4522	19.130	27.160	51.950	1.00	0.00
ATOM	14691	OW	SOL	4523	18.340	26.990	35.460	1.00	0.00
ATOM	14692	HW1	SOL	4523	17.880	27.820	35.440	1.00	0.00
ATOM	14693	HW2	SOL	4523	19.230	27.220	35.740	1.00	0.00
ATOM	14694	OW	SOL	4524	21.030	47.690	3.250	1.00	0.00
ATOM	14695	HW1	SOL	4524	21.930	47.930	3.470	1.00	0.00

ATOM	14696	HW2	SOL	4524	20.530	47.900	4.040	1.00	0.00
ATOM	14697	OW	SOL	4525	14.240	36.170	14.270	1.00	0.00
ATOM	14698	HW1	SOL	4525	14.240	36.610	13.420	1.00	0.00
ATOM	14699	HW2	SOL	4525	13.560	36.620	14.770	1.00	0.00
ATOM	14700	OW	SOL	4526	21.610	3.230	50.940	1.00	0.00
ATOM	14701	HW1	SOL	4526	22.250	3.400	51.630	1.00	0.00
ATOM	14702	HW2	SOL	4526	22.130	2.900	50.200	1.00	0.00
ATOM	14703	OW	SOL	4527	36.640	10.460	2.640	1.00	0.00
ATOM	14704	HW1	SOL	4527	37.310	10.890	2.110	1.00	0.00
ATOM	14705	HW2	SOL	4527	36.540	11.030	3.410	1.00	0.00
ATOM	14706	OW	SOL	4528	31.380	42.850	21.730	1.00	0.00
ATOM	14707	HW1	SOL	4528	31.020	42.050	21.350	1.00	0.00
ATOM	14708	HW2	SOL	4528	31.420	43.470	21.000	1.00	0.00
ATOM	14709	OW	SOL	4529	42.650	11.770	42.230	1.00	0.00
ATOM	14710	HW1	SOL	4529	42.110	11.470	42.960	1.00	0.00
ATOM	14711	HW2	SOL	4529	42.060	12.320	41.710	1.00	0.00
ATOM	14712	OW	SOL	4530	14.650	12.620	45.190	1.00	0.00
ATOM	14713	HW1	SOL	4530	13.950	13.220	45.450	1.00	0.00
ATOM	14714	HW2	SOL	4530	15.460	13.100	45.370	1.00	0.00
ATOM	14715	OW	SOL	4531	17.250	10.140	18.860	1.00	0.00
ATOM	14716	HW1	SOL	4531	16.310	10.050	19.000	1.00	0.00
ATOM	14717	HW2	SOL	4531	17.650	9.540	19.490	1.00	0.00
ATOM	14718	OW	SOL	4532	8.570	13.300	18.670	1.00	0.00
ATOM	14719	HW1	SOL	4532	8.220	13.430	17.790	1.00	0.00
ATOM	14720	HW2	SOL	4532	8.700	12.350	18.740	1.00	0.00
ATOM	14721	OW	SOL	4533	16.450	40.870	4.270	1.00	0.00
ATOM	14722	HW1	SOL	4533	16.680	39.960	4.120	1.00	0.00
ATOM	14723	HW2	SOL	4533	15.750	41.050	3.640	1.00	0.00
ATOM	14724	OW	SOL	4534	48.590	50.770	52.980	1.00	0.00
ATOM	14725	HW1	SOL	4534	48.690	49.830	52.850	1.00	0.00
ATOM	14726	HW2	SOL	4534	47.650	50.920	52.960	1.00	0.00
ATOM	14727	OW	SOL	4535	9.060	3.200	26.740	1.00	0.00
ATOM	14728	HW1	SOL	4535	8.600	4.050	26.770	1.00	0.00
ATOM	14729	HW2	SOL	4535	9.820	3.320	27.310	1.00	0.00
ATOM	14730	OW	SOL	4536	25.870	38.300	23.020	1.00	0.00
ATOM	14731	HW1	SOL	4536	26.490	38.450	23.740	1.00	0.00
ATOM	14732	HW2	SOL	4536	25.520	37.420	23.180	1.00	0.00
ATOM	14733	OW	SOL	4537	43.020	0.970	54.930	1.00	0.00
ATOM	14734	HW1	SOL	4537	42.630	1.540	55.590	1.00	0.00
ATOM	14735	HW2	SOL	4537	43.850	0.690	55.320	1.00	0.00
ATOM	14736	OW	SOL	4538	40.940	23.940	13.610	1.00	0.00
ATOM	14737	HW1	SOL	4538	41.120	24.740	14.100	1.00	0.00
ATOM	14738	HW2	SOL	4538	40.000	23.970	13.440	1.00	0.00
ATOM	14739	OW	SOL	4539	40.030	51.230	1.340	1.00	0.00

ATOM	14740	HW1	SOL	4539	39.950	51.900	2.020	1.00	0.00
ATOM	14741	HW2	SOL	4539	39.370	51.470	0.690	1.00	0.00
ATOM	14742	OW	SOL	4540	16.460	20.480	20.570	1.00	0.00
ATOM	14743	HW1	SOL	4540	17.250	20.110	20.180	1.00	0.00
ATOM	14744	HW2	SOL	4540	16.760	20.880	21.390	1.00	0.00
ATOM	14745	OW	SOL	4541	49.780	43.740	47.400	1.00	0.00
ATOM	14746	HW1	SOL	4541	50.070	44.240	46.640	1.00	0.00
ATOM	14747	HW2	SOL	4541	49.110	44.290	47.800	1.00	0.00
ATOM	14748	OW	SOL	4542	7.240	19.710	41.080	1.00	0.00
ATOM	14749	HW1	SOL	4542	7.160	20.490	41.640	1.00	0.00
ATOM	14750	HW2	SOL	4542	7.290	20.060	40.190	1.00	0.00
ATOM	14751	OW	SOL	4543	50.500	22.590	29.410	1.00	0.00
ATOM	14752	HW1	SOL	4543	51.140	22.260	28.770	1.00	0.00
ATOM	14753	HW2	SOL	4543	49.660	22.530	28.960	1.00	0.00
ATOM	14754	OW	SOL	4544	52.940	3.570	53.000	1.00	0.00
ATOM	14755	HW1	SOL	4544	52.230	4.040	53.440	1.00	0.00
ATOM	14756	HW2	SOL	4544	52.500	3.060	52.320	1.00	0.00
ATOM	14757	OW	SOL	4545	20.800	1.860	53.610	1.00	0.00
ATOM	14758	HW1	SOL	4545	20.500	0.950	53.580	1.00	0.00
ATOM	14759	HW2	SOL	4545	21.190	2.010	52.750	1.00	0.00
ATOM	14760	OW	SOL	4546	15.630	51.970	37.470	1.00	0.00
ATOM	14761	HW1	SOL	4546	14.700	51.820	37.320	1.00	0.00
ATOM	14762	HW2	SOL	4546	15.670	52.580	38.210	1.00	0.00
ATOM	14763	OW	SOL	4547	50.430	41.660	48.980	1.00	0.00
ATOM	14764	HW1	SOL	4547	49.760	41.870	49.640	1.00	0.00
ATOM	14765	HW2	SOL	4547	50.180	42.180	48.220	1.00	0.00
ATOM	14766	OW	SOL	4548	8.240	34.030	47.230	1.00	0.00
ATOM	14767	HW1	SOL	4548	7.980	34.080	48.150	1.00	0.00
ATOM	14768	HW2	SOL	4548	8.390	33.100	47.070	1.00	0.00
ATOM	14769	OW	SOL	4549	29.130	3.030	8.580	1.00	0.00
ATOM	14770	HW1	SOL	4549	28.740	2.160	8.500	1.00	0.00
ATOM	14771	HW2	SOL	4549	28.390	3.630	8.550	1.00	0.00
ATOM	14772	OW	SOL	4550	32.780	49.190	27.210	1.00	0.00
ATOM	14773	HW1	SOL	4550	32.470	48.870	28.050	1.00	0.00
ATOM	14774	HW2	SOL	4550	32.000	49.200	26.650	1.00	0.00
ATOM	14775	OW	SOL	4551	40.700	12.170	13.920	1.00	0.00
ATOM	14776	HW1	SOL	4551	40.130	12.580	13.270	1.00	0.00
ATOM	14777	HW2	SOL	4551	41.460	12.750	13.990	1.00	0.00
ATOM	14778	OW	SOL	4552	32.450	30.350	14.510	1.00	0.00
ATOM	14779	HW1	SOL	4552	31.630	30.540	14.980	1.00	0.00
ATOM	14780	HW2	SOL	4552	33.140	30.620	15.110	1.00	0.00
ATOM	14781	OW	SOL	4553	11.230	51.700	11.110	1.00	0.00
ATOM	14782	HW1	SOL	4553	11.920	52.280	11.430	1.00	0.00
ATOM	14783	HW2	SOL	4553	10.460	52.260	11.040	1.00	0.00

ATOM	14784	OW	SOL	4554	1.120	30.300	18.230	1.00	0.00
ATOM	14785	HW1	SOL	4554	0.530	31.040	18.080	1.00	0.00
ATOM	14786	HW2	SOL	4554	1.820	30.420	17.580	1.00	0.00
ATOM	14787	OW	SOL	4555	6.230	11.400	10.380	1.00	0.00
ATOM	14788	HW1	SOL	4555	5.880	10.680	9.850	1.00	0.00
ATOM	14789	HW2	SOL	4555	7.180	11.270	10.360	1.00	0.00
ATOM	14790	OW	SOL	4556	11.060	41.060	48.580	1.00	0.00
ATOM	14791	HW1	SOL	4556	10.750	41.080	47.670	1.00	0.00
ATOM	14792	HW2	SOL	4556	12.000	41.230	48.520	1.00	0.00
ATOM	14793	OW	SOL	4557	9.930	19.990	2.300	1.00	0.00
ATOM	14794	HW1	SOL	4557	9.320	19.740	3.000	1.00	0.00
ATOM	14795	HW2	SOL	4557	10.140	20.910	2.480	1.00	0.00
ATOM	14796	OW	SOL	4558	34.410	1.730	52.880	1.00	0.00
ATOM	14797	HW1	SOL	4558	33.500	1.560	53.100	1.00	0.00
ATOM	14798	HW2	SOL	4558	34.790	2.060	53.690	1.00	0.00
ATOM	14799	OW	SOL	4559	9.610	27.490	17.900	1.00	0.00
ATOM	14800	HW1	SOL	4559	10.560	27.560	18.040	1.00	0.00
ATOM	14801	HW2	SOL	4559	9.450	26.550	17.890	1.00	0.00
ATOM	14802	OW	SOL	4560	6.070	9.120	8.720	1.00	0.00
ATOM	14803	HW1	SOL	4560	5.300	8.700	9.100	1.00	0.00
ATOM	14804	HW2	SOL	4560	6.780	8.490	8.870	1.00	0.00
ATOM	14805	OW	SOL	4561	48.370	3.420	19.860	1.00	0.00
ATOM	14806	HW1	SOL	4561	49.250	3.670	19.570	1.00	0.00
ATOM	14807	HW2	SOL	4561	48.370	3.600	20.800	1.00	0.00
ATOM	14808	OW	SOL	4562	0.940	34.070	23.210	1.00	0.00
ATOM	14809	HW1	SOL	4562	0.760	33.960	24.140	1.00	0.00
ATOM	14810	HW2	SOL	4562	1.460	33.300	22.970	1.00	0.00
ATOM	14811	OW	SOL	4563	33.860	35.550	19.720	1.00	0.00
ATOM	14812	HW1	SOL	4563	33.790	34.680	19.320	1.00	0.00
ATOM	14813	HW2	SOL	4563	33.700	36.160	19.000	1.00	0.00
ATOM	14814	OW	SOL	4564	9.420	25.290	52.390	1.00	0.00
ATOM	14815	HW1	SOL	4564	10.220	24.770	52.370	1.00	0.00
ATOM	14816	HW2	SOL	4564	9.260	25.520	51.480	1.00	0.00
ATOM	14817	OW	SOL	4565	53.650	9.770	3.890	1.00	0.00
ATOM	14818	HW1	SOL	4565	52.730	10.060	3.880	1.00	0.00
ATOM	14819	HW2	SOL	4565	53.900	9.820	4.810	1.00	0.00
ATOM	14820	OW	SOL	4566	4.670	39.730	48.600	1.00	0.00
ATOM	14821	HW1	SOL	4566	4.860	39.740	49.540	1.00	0.00
ATOM	14822	HW2	SOL	4566	4.180	38.920	48.470	1.00	0.00
ATOM	14823	OW	SOL	4567	29.290	53.700	7.360	1.00	0.00
ATOM	14824	HW1	SOL	4567	28.780	54.500	7.500	1.00	0.00
ATOM	14825	HW2	SOL	4567	30.170	53.940	7.650	1.00	0.00
ATOM	14826	OW	SOL	4568	21.360	18.990	20.330	1.00	0.00
ATOM	14827	HW1	SOL	4568	21.440	18.460	19.540	1.00	0.00

ATOM	14828	HW2	SOL	4568	22.100	18.720	20.870	1.00	0.00
ATOM	14829	OW	SOL	4569	12.840	6.750	49.620	1.00	0.00
ATOM	14830	HW1	SOL	4569	13.020	7.670	49.460	1.00	0.00
ATOM	14831	HW2	SOL	4569	13.190	6.590	50.500	1.00	0.00
ATOM	14832	OW	SOL	4570	55.150	29.930	30.480	1.00	0.00
ATOM	14833	HW1	SOL	4570	54.370	29.400	30.650	1.00	0.00
ATOM	14834	HW2	SOL	4570	55.860	29.440	30.900	1.00	0.00
ATOM	14835	OW	SOL	4571	3.750	15.610	27.810	1.00	0.00
ATOM	14836	HW1	SOL	4571	3.250	16.280	27.340	1.00	0.00
ATOM	14837	HW2	SOL	4571	4.060	16.060	28.600	1.00	0.00
ATOM	14838	OW	SOL	4572	47.730	9.070	33.050	1.00	0.00
ATOM	14839	HW1	SOL	4572	48.530	8.690	32.680	1.00	0.00
ATOM	14840	HW2	SOL	4572	47.390	8.380	33.630	1.00	0.00
ATOM	14841	OW	SOL	4573	18.200	10.920	16.300	1.00	0.00
ATOM	14842	HW1	SOL	4573	18.350	11.860	16.170	1.00	0.00
ATOM	14843	HW2	SOL	4573	17.910	10.850	17.210	1.00	0.00
ATOM	14844	OW	SOL	4574	29.310	47.070	22.950	1.00	0.00
ATOM	14845	HW1	SOL	4574	30.050	47.670	22.890	1.00	0.00
ATOM	14846	HW2	SOL	4574	28.840	47.180	22.120	1.00	0.00
ATOM	14847	OW	SOL	4575	13.480	35.280	9.730	1.00	0.00
ATOM	14848	HW1	SOL	4575	13.170	34.450	9.360	1.00	0.00
ATOM	14849	HW2	SOL	4575	14.400	35.120	9.930	1.00	0.00
ATOM	14850	OW	SOL	4576	51.820	42.070	14.430	1.00	0.00
ATOM	14851	HW1	SOL	4576	50.910	42.090	14.140	1.00	0.00
ATOM	14852	HW2	SOL	4576	51.950	41.180	14.760	1.00	0.00
ATOM	14853	OW	SOL	4577	29.570	8.860	24.760	1.00	0.00
ATOM	14854	HW1	SOL	4577	28.850	8.660	24.160	1.00	0.00
ATOM	14855	HW2	SOL	4577	29.740	9.790	24.640	1.00	0.00
ATOM	14856	OW	SOL	4578	43.360	6.230	6.450	1.00	0.00
ATOM	14857	HW1	SOL	4578	43.390	7.010	5.910	1.00	0.00
ATOM	14858	HW2	SOL	4578	42.460	6.190	6.770	1.00	0.00
ATOM	14859	OW	SOL	4579	18.890	52.760	29.480	1.00	0.00
ATOM	14860	HW1	SOL	4579	18.210	52.620	28.820	1.00	0.00
ATOM	14861	HW2	SOL	4579	18.960	51.920	29.930	1.00	0.00
ATOM	14862	OW	SOL	4580	47.910	8.020	53.600	1.00	0.00
ATOM	14863	HW1	SOL	4580	48.140	8.950	53.720	1.00	0.00
ATOM	14864	HW2	SOL	4580	47.050	8.050	53.190	1.00	0.00
ATOM	14865	OW	SOL	4581	17.560	5.270	41.930	1.00	0.00
ATOM	14866	HW1	SOL	4581	17.850	5.310	41.020	1.00	0.00
ATOM	14867	HW2	SOL	4581	18.370	5.310	42.440	1.00	0.00
ATOM	14868	OW	SOL	4582	8.740	52.290	14.090	1.00	0.00
ATOM	14869	HW1	SOL	4582	9.050	51.420	14.320	1.00	0.00
ATOM	14870	HW2	SOL	4582	9.510	52.850	14.170	1.00	0.00
ATOM	14871	OW	SOL	4583	17.860	26.620	21.990	1.00	0.00

ATOM	14872	HW1	SOL	4583	17.920	25.780	21.550	1.00	0.00
ATOM	14873	HW2	SOL	4583	18.620	27.110	21.670	1.00	0.00
ATOM	14874	OW	SOL	4584	54.620	23.670	1.600	1.00	0.00
ATOM	14875	HW1	SOL	4584	53.840	23.470	2.130	1.00	0.00
ATOM	14876	HW2	SOL	4584	55.250	24.010	2.230	1.00	0.00
ATOM	14877	OW	SOL	4585	50.440	54.850	32.640	1.00	0.00
ATOM	14878	HW1	SOL	4585	51.280	54.900	32.180	1.00	0.00
ATOM	14879	HW2	SOL	4585	49.960	55.620	32.320	1.00	0.00
ATOM	14880	OW	SOL	4586	55.720	3.070	21.910	1.00	0.00
ATOM	14881	HW1	SOL	4586	55.060	2.600	21.410	1.00	0.00
ATOM	14882	HW2	SOL	4586	55.490	2.890	22.830	1.00	0.00
ATOM	14883	OW	SOL	4587	30.970	15.900	0.760	1.00	0.00
ATOM	14884	HW1	SOL	4587	31.880	15.630	0.820	1.00	0.00
ATOM	14885	HW2	SOL	4587	30.830	16.080	-0.170	1.00	0.00
ATOM	14886	OW	SOL	4588	16.830	6.720	45.410	1.00	0.00
ATOM	14887	HW1	SOL	4588	16.350	6.820	46.240	1.00	0.00
ATOM	14888	HW2	SOL	4588	16.140	6.630	44.750	1.00	0.00
ATOM	14889	OW	SOL	4589	25.960	28.120	8.880	1.00	0.00
ATOM	14890	HW1	SOL	4589	25.140	27.850	9.280	1.00	0.00
ATOM	14891	HW2	SOL	4589	26.640	27.730	9.440	1.00	0.00
ATOM	14892	OW	SOL	4590	45.430	40.000	17.360	1.00	0.00
ATOM	14893	HW1	SOL	4590	46.100	39.930	16.680	1.00	0.00
ATOM	14894	HW2	SOL	4590	44.610	39.840	16.910	1.00	0.00
ATOM	14895	OW	SOL	4591	36.060	49.040	32.140	1.00	0.00
ATOM	14896	HW1	SOL	4591	35.540	48.860	31.360	1.00	0.00
ATOM	14897	HW2	SOL	4591	35.810	48.360	32.760	1.00	0.00
ATOM	14898	OW	SOL	4592	20.620	48.760	51.800	1.00	0.00
ATOM	14899	HW1	SOL	4592	21.160	48.030	52.100	1.00	0.00
ATOM	14900	HW2	SOL	4592	21.020	49.040	50.970	1.00	0.00
ATOM	14901	OW	SOL	4593	36.360	40.160	22.880	1.00	0.00
ATOM	14902	HW1	SOL	4593	36.000	39.790	22.070	1.00	0.00
ATOM	14903	HW2	SOL	4593	36.420	41.100	22.700	1.00	0.00
ATOM	14904	OW	SOL	4594	5.430	19.060	33.280	1.00	0.00
ATOM	14905	HW1	SOL	4594	4.480	19.130	33.430	1.00	0.00
ATOM	14906	HW2	SOL	4594	5.820	19.620	33.950	1.00	0.00
ATOM	14907	OW	SOL	4595	52.980	3.360	20.380	1.00	0.00
ATOM	14908	HW1	SOL	4595	52.210	3.760	19.980	1.00	0.00
ATOM	14909	HW2	SOL	4595	53.680	3.470	19.730	1.00	0.00
ATOM	14910	OW	SOL	4596	39.520	20.860	50.140	1.00	0.00
ATOM	14911	HW1	SOL	4596	40.430	20.950	49.860	1.00	0.00
ATOM	14912	HW2	SOL	4596	39.020	21.330	49.480	1.00	0.00
ATOM	14913	OW	SOL	4597	1.450	5.940	11.890	1.00	0.00
ATOM	14914	HW1	SOL	4597	1.960	6.060	12.690	1.00	0.00
ATOM	14915	HW2	SOL	4597	0.620	6.390	12.070	1.00	0.00

ATOM	14916	OW	SOL	4598	43.550	47.710	37.030	1.00	0.00
ATOM	14917	HW1	SOL	4598	42.710	47.930	36.620	1.00	0.00
ATOM	14918	HW2	SOL	4598	43.980	47.130	36.400	1.00	0.00
ATOM	14919	OW	SOL	4599	33.240	41.240	17.760	1.00	0.00
ATOM	14920	HW1	SOL	4599	32.690	41.920	17.380	1.00	0.00
ATOM	14921	HW2	SOL	4599	33.210	40.520	17.130	1.00	0.00
ATOM	14922	OW	SOL	4600	21.740	11.100	19.510	1.00	0.00
ATOM	14923	HW1	SOL	4600	21.160	10.410	19.180	1.00	0.00
ATOM	14924	HW2	SOL	4600	21.910	10.850	20.420	1.00	0.00
ATOM	14925	OW	SOL	4601	15.380	25.180	15.030	1.00	0.00
ATOM	14926	HW1	SOL	4601	15.250	24.860	15.920	1.00	0.00
ATOM	14927	HW2	SOL	4601	16.310	25.010	14.850	1.00	0.00
ATOM	14928	OW	SOL	4602	52.210	20.170	37.680	1.00	0.00
ATOM	14929	HW1	SOL	4602	52.540	20.760	37.000	1.00	0.00
ATOM	14930	HW2	SOL	4602	51.900	20.760	38.370	1.00	0.00
ATOM	14931	OW	SOL	4603	19.880	40.260	52.560	1.00	0.00
ATOM	14932	HW1	SOL	4603	19.000	40.410	52.890	1.00	0.00
ATOM	14933	HW2	SOL	4603	20.450	40.760	53.150	1.00	0.00
ATOM	14934	OW	SOL	4604	16.830	23.360	0.060	1.00	0.00
ATOM	14935	HW1	SOL	4604	16.980	23.560	-0.860	1.00	0.00
ATOM	14936	HW2	SOL	4604	17.060	22.440	0.160	1.00	0.00
ATOM	14937	OW	SOL	4605	48.520	48.380	15.990	1.00	0.00
ATOM	14938	HW1	SOL	4605	49.030	49.070	15.570	1.00	0.00
ATOM	14939	HW2	SOL	4605	47.850	48.830	16.490	1.00	0.00
ATOM	14940	OW	SOL	4606	33.140	28.800	32.490	1.00	0.00
ATOM	14941	HW1	SOL	4606	32.590	28.630	31.730	1.00	0.00
ATOM	14942	HW2	SOL	4606	33.320	29.740	32.450	1.00	0.00
ATOM	14943	OW	SOL	4607	55.270	18.720	40.640	1.00	0.00
ATOM	14944	HW1	SOL	4607	54.690	18.580	39.900	1.00	0.00
ATOM	14945	HW2	SOL	4607	54.830	19.400	41.150	1.00	0.00
ATOM	14946	OW	SOL	4608	22.820	33.670	53.590	1.00	0.00
ATOM	14947	HW1	SOL	4608	23.000	34.450	54.110	1.00	0.00
ATOM	14948	HW2	SOL	4608	22.870	33.970	52.680	1.00	0.00
ATOM	14949	OW	SOL	4609	43.270	2.430	7.400	1.00	0.00
ATOM	14950	HW1	SOL	4609	43.900	2.020	6.810	1.00	0.00
ATOM	14951	HW2	SOL	4609	43.150	1.790	8.100	1.00	0.00
ATOM	14952	OW	SOL	4610	21.880	12.480	44.000	1.00	0.00
ATOM	14953	HW1	SOL	4610	22.280	13.020	44.690	1.00	0.00
ATOM	14954	HW2	SOL	4610	22.550	12.400	43.330	1.00	0.00
ATOM	14955	OW	SOL	4611	32.940	13.470	7.750	1.00	0.00
ATOM	14956	HW1	SOL	4611	33.500	12.830	8.190	1.00	0.00
ATOM	14957	HW2	SOL	4611	33.500	14.240	7.640	1.00	0.00
ATOM	14958	OW	SOL	4612	42.480	55.170	36.770	1.00	0.00
ATOM	14959	HW1	SOL	4612	43.300	55.630	36.590	1.00	0.00

ATOM	14960	HW2	SOL	4612	42.700	54.240	36.660	1.00	0.00
ATOM	14961	OW	SOL	4613	25.480	20.610	50.410	1.00	0.00
ATOM	14962	HW1	SOL	4613	25.970	21.350	50.060	1.00	0.00
ATOM	14963	HW2	SOL	4613	25.790	19.850	49.920	1.00	0.00
ATOM	14964	OW	SOL	4614	39.540	30.540	52.710	1.00	0.00
ATOM	14965	HW1	SOL	4614	39.040	30.000	52.100	1.00	0.00
ATOM	14966	HW2	SOL	4614	40.270	29.980	53.000	1.00	0.00
ATOM	14967	OW	SOL	4615	28.840	48.240	30.860	1.00	0.00
ATOM	14968	HW1	SOL	4615	29.030	49.170	30.940	1.00	0.00
ATOM	14969	HW2	SOL	4615	28.250	48.170	30.110	1.00	0.00
ATOM	14970	OW	SOL	4616	2.910	1.100	3.960	1.00	0.00
ATOM	14971	HW1	SOL	4616	3.450	1.820	3.620	1.00	0.00
ATOM	14972	HW2	SOL	4616	3.050	0.390	3.340	1.00	0.00
ATOM	14973	OW	SOL	4617	36.350	54.430	23.030	1.00	0.00
ATOM	14974	HW1	SOL	4617	36.820	55.020	22.440	1.00	0.00
ATOM	14975	HW2	SOL	4617	36.070	53.710	22.470	1.00	0.00
ATOM	14976	OW	SOL	4618	39.250	14.020	47.860	1.00	0.00
ATOM	14977	HW1	SOL	4618	40.180	14.150	48.030	1.00	0.00
ATOM	14978	HW2	SOL	4618	39.190	13.880	46.920	1.00	0.00
ATOM	14979	OW	SOL	4619	24.370	34.800	20.310	1.00	0.00
ATOM	14980	HW1	SOL	4619	24.170	35.460	19.640	1.00	0.00
ATOM	14981	HW2	SOL	4619	24.840	34.120	19.830	1.00	0.00
ATOM	14982	OW	SOL	4620	48.100	39.020	2.520	1.00	0.00
ATOM	14983	HW1	SOL	4620	48.620	39.640	2.020	1.00	0.00
ATOM	14984	HW2	SOL	4620	47.250	39.440	2.630	1.00	0.00
ATOM	14985	OW	SOL	4621	14.730	47.710	13.320	1.00	0.00
ATOM	14986	HW1	SOL	4621	13.830	47.450	13.130	1.00	0.00
ATOM	14987	HW2	SOL	4621	14.650	48.510	13.840	1.00	0.00
ATOM	14988	OW	SOL	4622	30.820	5.690	13.890	1.00	0.00
ATOM	14989	HW1	SOL	4622	31.520	5.460	13.280	1.00	0.00
ATOM	14990	HW2	SOL	4622	30.990	6.600	14.130	1.00	0.00
ATOM	14991	OW	SOL	4623	14.650	0.980	32.820	1.00	0.00
ATOM	14992	HW1	SOL	4623	15.480	1.300	32.470	1.00	0.00
ATOM	14993	HW2	SOL	4623	14.750	0.030	32.870	1.00	0.00
ATOM	14994	OW	SOL	4624	52.660	11.290	37.400	1.00	0.00
ATOM	14995	HW1	SOL	4624	53.050	12.070	37.800	1.00	0.00
ATOM	14996	HW2	SOL	4624	52.500	11.540	36.490	1.00	0.00
ATOM	14997	OW	SOL	4625	2.800	5.940	14.100	1.00	0.00
ATOM	14998	HW1	SOL	4625	3.740	6.090	14.120	1.00	0.00
ATOM	14999	HW2	SOL	4625	2.520	6.050	15.010	1.00	0.00
ATOM	15000	OW	SOL	4626	13.250	38.760	0.020	1.00	0.00
ATOM	15001	HW1	SOL	4626	14.100	39.070	-0.310	1.00	0.00
ATOM	15002	HW2	SOL	4626	13.370	37.820	0.130	1.00	0.00
ATOM	15003	OW	SOL	4627	9.650	3.400	1.050	1.00	0.00

ATOM	15004	HW1	SOL	4627	9.250	2.710	1.570	1.00	0.00
ATOM	15005	HW2	SOL	4627	8.910	3.930	0.740	1.00	0.00
ATOM	15006	OW	SOL	4628	9.860	16.050	25.220	1.00	0.00
ATOM	15007	HW1	SOL	4628	9.570	16.300	26.090	1.00	0.00
ATOM	15008	HW2	SOL	4628	9.050	15.930	24.720	1.00	0.00
ATOM	15009	OW	SOL	4629	0.850	10.930	40.580	1.00	0.00
ATOM	15010	HW1	SOL	4629	1.610	11.150	40.040	1.00	0.00
ATOM	15011	HW2	SOL	4629	0.480	11.770	40.840	1.00	0.00
ATOM	15012	OW	SOL	4630	1.740	6.900	54.470	1.00	0.00
ATOM	15013	HW1	SOL	4630	2.580	7.160	54.840	1.00	0.00
ATOM	15014	HW2	SOL	4630	1.920	6.720	53.550	1.00	0.00
ATOM	15015	OW	SOL	4631	1.170	23.540	25.520	1.00	0.00
ATOM	15016	HW1	SOL	4631	1.510	23.240	26.360	1.00	0.00
ATOM	15017	HW2	SOL	4631	0.220	23.510	25.620	1.00	0.00
ATOM	15018	OW	SOL	4632	31.630	48.720	32.670	1.00	0.00
ATOM	15019	HW1	SOL	4632	32.120	48.640	33.490	1.00	0.00
ATOM	15020	HW2	SOL	4632	30.930	48.070	32.740	1.00	0.00
ATOM	15021	OW	SOL	4633	36.490	17.470	15.020	1.00	0.00
ATOM	15022	HW1	SOL	4633	37.190	18.050	14.710	1.00	0.00
ATOM	15023	HW2	SOL	4633	36.060	17.150	14.230	1.00	0.00
ATOM	15024	OW	SOL	4634	29.780	6.760	34.600	1.00	0.00
ATOM	15025	HW1	SOL	4634	30.450	7.170	35.150	1.00	0.00
ATOM	15026	HW2	SOL	4634	30.140	6.800	33.710	1.00	0.00
ATOM	15027	OW	SOL	4635	53.850	10.510	31.170	1.00	0.00
ATOM	15028	HW1	SOL	4635	54.630	10.640	30.630	1.00	0.00
ATOM	15029	HW2	SOL	4635	53.270	11.230	30.920	1.00	0.00
ATOM	15030	OW	SOL	4636	41.650	37.970	52.380	1.00	0.00
ATOM	15031	HW1	SOL	4636	42.110	38.110	53.210	1.00	0.00
ATOM	15032	HW2	SOL	4636	42.030	38.620	51.780	1.00	0.00
ATOM	15033	OW	SOL	4637	21.140	1.260	7.770	1.00	0.00
ATOM	15034	HW1	SOL	4637	20.480	0.920	7.160	1.00	0.00
ATOM	15035	HW2	SOL	4637	21.960	1.240	7.270	1.00	0.00
ATOM	15036	OW	SOL	4638	29.940	18.420	10.220	1.00	0.00
ATOM	15037	HW1	SOL	4638	30.680	18.620	10.790	1.00	0.00
ATOM	15038	HW2	SOL	4638	29.320	17.960	10.790	1.00	0.00
ATOM	15039	OW	SOL	4639	25.840	54.150	53.760	1.00	0.00
ATOM	15040	HW1	SOL	4639	25.740	55.020	54.150	1.00	0.00
ATOM	15041	HW2	SOL	4639	26.730	54.140	53.420	1.00	0.00
ATOM	15042	OW	SOL	4640	13.870	33.050	55.030	1.00	0.00
ATOM	15043	HW1	SOL	4640	14.720	32.630	54.930	1.00	0.00
ATOM	15044	HW2	SOL	4640	13.570	32.780	55.900	1.00	0.00
ATOM	15045	OW	SOL	4641	37.830	52.530	30.300	1.00	0.00
ATOM	15046	HW1	SOL	4641	37.590	53.450	30.160	1.00	0.00
ATOM	15047	HW2	SOL	4641	37.660	52.380	31.230	1.00	0.00

ATOM	15048	OW	SOL	4642	6.760	30.130	38.820	1.00	0.00
ATOM	15049	HW1	SOL	4642	6.110	30.770	38.520	1.00	0.00
ATOM	15050	HW2	SOL	4642	7.600	30.560	38.640	1.00	0.00
ATOM	15051	OW	SOL	4643	5.090	3.000	48.940	1.00	0.00
ATOM	15052	HW1	SOL	4643	4.350	2.390	48.940	1.00	0.00
ATOM	15053	HW2	SOL	4643	5.820	2.490	49.270	1.00	0.00
ATOM	15054	OW	SOL	4644	37.120	44.270	2.360	1.00	0.00
ATOM	15055	HW1	SOL	4644	36.600	44.670	1.650	1.00	0.00
ATOM	15056	HW2	SOL	4644	36.680	44.560	3.160	1.00	0.00
ATOM	15057	OW	SOL	4645	6.830	6.330	5.620	1.00	0.00
ATOM	15058	HW1	SOL	4645	7.570	6.260	6.220	1.00	0.00
ATOM	15059	HW2	SOL	4645	6.650	7.270	5.570	1.00	0.00
ATOM	15060	OW	SOL	4646	9.210	11.680	1.820	1.00	0.00
ATOM	15061	HW1	SOL	4646	9.210	11.030	2.520	1.00	0.00
ATOM	15062	HW2	SOL	4646	8.500	12.270	2.040	1.00	0.00
ATOM	15063	OW	SOL	4647	40.690	18.420	15.980	1.00	0.00
ATOM	15064	HW1	SOL	4647	40.780	17.520	15.680	1.00	0.00
ATOM	15065	HW2	SOL	4647	41.390	18.900	15.530	1.00	0.00
ATOM	15066	OW	SOL	4648	51.840	26.920	29.670	1.00	0.00
ATOM	15067	HW1	SOL	4648	52.520	26.250	29.630	1.00	0.00
ATOM	15068	HW2	SOL	4648	51.010	26.440	29.620	1.00	0.00
ATOM	15069	OW	SOL	4649	38.900	52.920	18.180	1.00	0.00
ATOM	15070	HW1	SOL	4649	39.650	53.510	18.060	1.00	0.00
ATOM	15071	HW2	SOL	4649	38.710	52.970	19.120	1.00	0.00
ATOM	15072	OW	SOL	4650	2.140	41.290	21.030	1.00	0.00
ATOM	15073	HW1	SOL	4650	1.200	41.350	21.240	1.00	0.00
ATOM	15074	HW2	SOL	4650	2.470	42.180	21.180	1.00	0.00
ATOM	15075	OW	SOL	4651	9.040	49.210	31.350	1.00	0.00
ATOM	15076	HW1	SOL	4651	8.620	48.360	31.240	1.00	0.00
ATOM	15077	HW2	SOL	4651	9.340	49.450	30.480	1.00	0.00
ATOM	15078	OW	SOL	4652	32.970	54.270	15.270	1.00	0.00
ATOM	15079	HW1	SOL	4652	33.590	54.140	15.980	1.00	0.00
ATOM	15080	HW2	SOL	4652	32.150	54.500	15.700	1.00	0.00
ATOM	15081	OW	SOL	4653	3.770	2.810	22.090	1.00	0.00
ATOM	15082	HW1	SOL	4653	4.270	3.500	21.650	1.00	0.00
ATOM	15083	HW2	SOL	4653	3.530	3.200	22.930	1.00	0.00
ATOM	15084	OW	SOL	4654	39.660	17.690	32.180	1.00	0.00
ATOM	15085	HW1	SOL	4654	40.560	17.750	32.500	1.00	0.00
ATOM	15086	HW2	SOL	4654	39.760	17.520	31.240	1.00	0.00
ATOM	15087	OW	SOL	4655	18.080	3.200	53.780	1.00	0.00
ATOM	15088	HW1	SOL	4655	18.890	2.690	53.870	1.00	0.00
ATOM	15089	HW2	SOL	4655	17.390	2.540	53.730	1.00	0.00
ATOM	15090	OW	SOL	4656	52.420	12.870	31.180	1.00	0.00
ATOM	15091	HW1	SOL	4656	51.620	12.870	30.660	1.00	0.00

ATOM	15092	HW2	SOL	4656	52.590	13.800	31.360	1.00	0.00
ATOM	15093	OW	SOL	4657	32.800	55.330	10.450	1.00	0.00
ATOM	15094	HW1	SOL	4657	32.300	54.950	11.170	1.00	0.00
ATOM	15095	HW2	SOL	4657	32.560	56.260	10.460	1.00	0.00
ATOM	15096	OW	SOL	4658	1.300	7.640	34.660	1.00	0.00
ATOM	15097	HW1	SOL	4658	0.530	7.190	35.010	1.00	0.00
ATOM	15098	HW2	SOL	4658	1.070	7.820	33.740	1.00	0.00
ATOM	15099	OW	SOL	4659	5.050	31.400	14.050	1.00	0.00
ATOM	15100	HW1	SOL	4659	5.020	30.460	13.870	1.00	0.00
ATOM	15101	HW2	SOL	4659	4.270	31.750	13.620	1.00	0.00
ATOM	15102	OW	SOL	4660	50.570	16.440	34.850	1.00	0.00
ATOM	15103	HW1	SOL	4660	50.290	17.200	35.370	1.00	0.00
ATOM	15104	HW2	SOL	4660	50.260	15.680	35.340	1.00	0.00
ATOM	15105	OW	SOL	4661	52.150	6.110	4.090	1.00	0.00
ATOM	15106	HW1	SOL	4661	51.780	6.980	4.260	1.00	0.00
ATOM	15107	HW2	SOL	4661	53.050	6.160	4.410	1.00	0.00
ATOM	15108	OW	SOL	4662	48.690	24.490	24.930	1.00	0.00
ATOM	15109	HW1	SOL	4662	48.980	23.950	24.200	1.00	0.00
ATOM	15110	HW2	SOL	4662	48.580	25.360	24.560	1.00	0.00
ATOM	15111	OW	SOL	4663	22.160	2.340	48.070	1.00	0.00
ATOM	15112	HW1	SOL	4663	21.280	2.090	47.800	1.00	0.00
ATOM	15113	HW2	SOL	4663	22.350	3.120	47.550	1.00	0.00
ATOM	15114	OW	SOL	4664	6.030	8.800	4.840	1.00	0.00
ATOM	15115	HW1	SOL	4664	5.550	8.140	4.340	1.00	0.00
ATOM	15116	HW2	SOL	4664	5.350	9.340	5.240	1.00	0.00
ATOM	15117	OW	SOL	4665	16.960	22.580	17.720	1.00	0.00
ATOM	15118	HW1	SOL	4665	16.250	21.940	17.640	1.00	0.00
ATOM	15119	HW2	SOL	4665	16.520	23.430	17.630	1.00	0.00
ATOM	15120	OW	SOL	4666	41.530	3.990	29.750	1.00	0.00
ATOM	15121	HW1	SOL	4666	41.500	4.570	28.990	1.00	0.00
ATOM	15122	HW2	SOL	4666	42.370	4.180	30.160	1.00	0.00
ATOM	15123	OW	SOL	4667	51.540	7.100	26.270	1.00	0.00
ATOM	15124	HW1	SOL	4667	51.630	7.810	26.900	1.00	0.00
ATOM	15125	HW2	SOL	4667	51.100	7.500	25.520	1.00	0.00
ATOM	15126	OW	SOL	4668	14.050	0.980	19.930	1.00	0.00
ATOM	15127	HW1	SOL	4668	14.060	0.330	19.240	1.00	0.00
ATOM	15128	HW2	SOL	4668	13.180	1.360	19.900	1.00	0.00
ATOM	15129	OW	SOL	4669	38.450	23.330	3.630	1.00	0.00
ATOM	15130	HW1	SOL	4669	37.750	23.650	3.080	1.00	0.00
ATOM	15131	HW2	SOL	4669	38.300	22.390	3.690	1.00	0.00
ATOM	15132	OW	SOL	4670	44.950	9.490	45.230	1.00	0.00
ATOM	15133	HW1	SOL	4670	44.290	8.950	44.810	1.00	0.00
ATOM	15134	HW2	SOL	4670	45.770	9.270	44.790	1.00	0.00
ATOM	15135	OW	SOL	4671	50.000	48.090	30.990	1.00	0.00

ATOM	15136	HW1	SOL	4671	50.310	48.690	30.310	1.00	0.00
ATOM	15137	HW2	SOL	4671	49.050	48.050	30.880	1.00	0.00
ATOM	15138	OW	SOL	4672	33.360	1.720	24.780	1.00	0.00
ATOM	15139	HW1	SOL	4672	32.980	2.390	24.200	1.00	0.00
ATOM	15140	HW2	SOL	4672	34.300	1.900	24.770	1.00	0.00
ATOM	15141	OW	SOL	4673	34.890	2.250	8.860	1.00	0.00
ATOM	15142	HW1	SOL	4673	35.690	2.620	9.230	1.00	0.00
ATOM	15143	HW2	SOL	4673	34.910	1.320	9.110	1.00	0.00
ATOM	15144	OW	SOL	4674	19.630	7.910	15.100	1.00	0.00
ATOM	15145	HW1	SOL	4674	19.820	8.490	15.850	1.00	0.00
ATOM	15146	HW2	SOL	4674	18.920	7.350	15.410	1.00	0.00
ATOM	15147	OW	SOL	4675	4.520	6.870	54.610	1.00	0.00
ATOM	15148	HW1	SOL	4675	5.260	7.110	54.050	1.00	0.00
ATOM	15149	HW2	SOL	4675	4.620	5.930	54.740	1.00	0.00
ATOM	15150	OW	SOL	4676	31.220	16.000	23.100	1.00	0.00
ATOM	15151	HW1	SOL	4676	31.020	15.820	24.010	1.00	0.00
ATOM	15152	HW2	SOL	4676	31.740	16.800	23.110	1.00	0.00
ATOM	15153	OW	SOL	4677	3.100	1.190	48.300	1.00	0.00
ATOM	15154	HW1	SOL	4677	3.010	0.610	47.540	1.00	0.00
ATOM	15155	HW2	SOL	4677	2.600	0.760	48.990	1.00	0.00
ATOM	15156	OW	SOL	4678	39.300	33.490	22.270	1.00	0.00
ATOM	15157	HW1	SOL	4678	38.580	32.890	22.440	1.00	0.00
ATOM	15158	HW2	SOL	4678	39.950	33.270	22.950	1.00	0.00
ATOM	15159	OW	SOL	4679	33.300	18.350	46.670	1.00	0.00
ATOM	15160	HW1	SOL	4679	32.540	18.880	46.880	1.00	0.00
ATOM	15161	HW2	SOL	4679	33.300	18.280	45.720	1.00	0.00
ATOM	15162	OW	SOL	4680	18.990	51.000	52.810	1.00	0.00
ATOM	15163	HW1	SOL	4680	18.970	51.820	52.320	1.00	0.00
ATOM	15164	HW2	SOL	4680	19.380	50.370	52.200	1.00	0.00
ATOM	15165	OW	SOL	4681	29.290	45.690	32.080	1.00	0.00
ATOM	15166	HW1	SOL	4681	29.650	45.010	31.510	1.00	0.00
ATOM	15167	HW2	SOL	4681	29.090	46.410	31.490	1.00	0.00
ATOM	15168	OW	SOL	4682	7.340	27.670	40.660	1.00	0.00
ATOM	15169	HW1	SOL	4682	7.680	28.470	40.260	1.00	0.00
ATOM	15170	HW2	SOL	4682	7.480	26.990	40.000	1.00	0.00
ATOM	15171	OW	SOL	4683	55.850	36.500	0.600	1.00	0.00
ATOM	15172	HW1	SOL	4683	55.460	37.310	0.290	1.00	0.00
ATOM	15173	HW2	SOL	4683	56.060	36.010	-0.200	1.00	0.00
ATOM	15174	OW	SOL	4684	8.730	2.840	14.730	1.00	0.00
ATOM	15175	HW1	SOL	4684	8.280	3.380	14.080	1.00	0.00
ATOM	15176	HW2	SOL	4684	8.200	2.040	14.770	1.00	0.00
ATOM	15177	OW	SOL	4685	35.890	36.250	9.870	1.00	0.00
ATOM	15178	HW1	SOL	4685	35.900	35.300	9.900	1.00	0.00
ATOM	15179	HW2	SOL	4685	35.230	36.460	9.210	1.00	0.00

ATOM	15180	OW	SOL	4686	35.120	26.910	32.790	1.00	0.00
ATOM	15181	HW1	SOL	4686	34.570	27.680	32.670	1.00	0.00
ATOM	15182	HW2	SOL	4686	35.120	26.760	33.730	1.00	0.00
ATOM	15183	OW	SOL	4687	49.220	7.450	3.780	1.00	0.00
ATOM	15184	HW1	SOL	4687	49.510	8.290	3.430	1.00	0.00
ATOM	15185	HW2	SOL	4687	48.360	7.310	3.400	1.00	0.00
ATOM	15186	OW	SOL	4688	20.970	46.380	28.150	1.00	0.00
ATOM	15187	HW1	SOL	4688	20.380	47.110	27.950	1.00	0.00
ATOM	15188	HW2	SOL	4688	20.730	46.110	29.040	1.00	0.00
ATOM	15189	OW	SOL	4689	40.100	50.210	17.850	1.00	0.00
ATOM	15190	HW1	SOL	4689	39.930	51.150	17.810	1.00	0.00
ATOM	15191	HW2	SOL	4689	40.080	49.920	16.940	1.00	0.00
ATOM	15192	OW	SOL	4690	5.190	53.280	47.020	1.00	0.00
ATOM	15193	HW1	SOL	4690	4.870	52.410	47.220	1.00	0.00
ATOM	15194	HW2	SOL	4690	5.570	53.600	47.840	1.00	0.00
ATOM	15195	OW	SOL	4691	18.980	54.130	6.300	1.00	0.00
ATOM	15196	HW1	SOL	4691	18.650	53.270	6.570	1.00	0.00
ATOM	15197	HW2	SOL	4691	18.710	54.720	7.010	1.00	0.00
ATOM	15198	OW	SOL	4692	9.740	10.170	23.300	1.00	0.00
ATOM	15199	HW1	SOL	4692	10.420	10.490	23.900	1.00	0.00
ATOM	15200	HW2	SOL	4692	10.060	10.420	22.430	1.00	0.00
ATOM	15201	OW	SOL	4693	35.190	37.740	48.980	1.00	0.00
ATOM	15202	HW1	SOL	4693	34.360	37.470	49.360	1.00	0.00
ATOM	15203	HW2	SOL	4693	35.640	38.200	49.690	1.00	0.00
ATOM	15204	OW	SOL	4694	24.000	8.720	32.900	1.00	0.00
ATOM	15205	HW1	SOL	4694	24.900	9.040	32.830	1.00	0.00
ATOM	15206	HW2	SOL	4694	23.750	8.910	33.810	1.00	0.00
ATOM	15207	OW	SOL	4695	26.650	7.560	0.720	1.00	0.00
ATOM	15208	HW1	SOL	4695	27.360	6.960	0.920	1.00	0.00
ATOM	15209	HW2	SOL	4695	26.450	7.400	-0.200	1.00	0.00
ATOM	15210	OW	SOL	4696	47.330	17.800	44.610	1.00	0.00
ATOM	15211	HW1	SOL	4696	47.020	18.700	44.610	1.00	0.00
ATOM	15212	HW2	SOL	4696	46.680	17.320	44.100	1.00	0.00
ATOM	15213	OW	SOL	4697	24.000	3.640	52.680	1.00	0.00
ATOM	15214	HW1	SOL	4697	24.940	3.700	52.850	1.00	0.00
ATOM	15215	HW2	SOL	4697	23.650	3.190	53.450	1.00	0.00
ATOM	15216	OW	SOL	4698	41.650	24.310	8.420	1.00	0.00
ATOM	15217	HW1	SOL	4698	40.990	24.920	8.080	1.00	0.00
ATOM	15218	HW2	SOL	4698	42.350	24.340	7.770	1.00	0.00
ATOM	15219	OW	SOL	4699	55.560	14.830	27.460	1.00	0.00
ATOM	15220	HW1	SOL	4699	55.710	15.750	27.280	1.00	0.00
ATOM	15221	HW2	SOL	4699	54.630	14.770	27.700	1.00	0.00
ATOM	15222	OW	SOL	4700	14.920	31.320	43.090	1.00	0.00
ATOM	15223	HW1	SOL	4700	15.750	31.730	43.310	1.00	0.00

ATOM	15224	HW2	SOL	4700	14.570	31.860	42.380	1.00	0.00
ATOM	15225	OW	SOL	4701	13.940	34.170	34.200	1.00	0.00
ATOM	15226	HW1	SOL	4701	13.620	34.930	33.730	1.00	0.00
ATOM	15227	HW2	SOL	4701	13.470	33.430	33.820	1.00	0.00
ATOM	15228	OW	SOL	4702	1.270	47.750	21.170	1.00	0.00
ATOM	15229	HW1	SOL	4702	0.410	47.480	21.470	1.00	0.00
ATOM	15230	HW2	SOL	4702	1.250	47.610	20.220	1.00	0.00
ATOM	15231	OW	SOL	4703	14.720	46.580	9.640	1.00	0.00
ATOM	15232	HW1	SOL	4703	13.970	45.980	9.740	1.00	0.00
ATOM	15233	HW2	SOL	4703	14.400	47.420	9.950	1.00	0.00
ATOM	15234	OW	SOL	4704	41.700	9.590	50.780	1.00	0.00
ATOM	15235	HW1	SOL	4704	42.620	9.630	50.530	1.00	0.00
ATOM	15236	HW2	SOL	4704	41.500	8.650	50.790	1.00	0.00
ATOM	15237	OW	SOL	4705	16.950	36.950	19.950	1.00	0.00
ATOM	15238	HW1	SOL	4705	16.480	37.570	19.390	1.00	0.00
ATOM	15239	HW2	SOL	4705	17.700	37.450	20.280	1.00	0.00
ATOM	15240	OW	SOL	4706	41.450	19.460	21.630	1.00	0.00
ATOM	15241	HW1	SOL	4706	42.240	19.460	22.180	1.00	0.00
ATOM	15242	HW2	SOL	4706	41.520	18.660	21.110	1.00	0.00
ATOM	15243	OW	SOL	4707	6.410	24.940	41.420	1.00	0.00
ATOM	15244	HW1	SOL	4707	6.530	24.070	41.800	1.00	0.00
ATOM	15245	HW2	SOL	4707	6.640	25.550	42.120	1.00	0.00
ATOM	15246	OW	SOL	4708	10.930	27.270	7.960	1.00	0.00
ATOM	15247	HW1	SOL	4708	11.880	27.140	8.030	1.00	0.00
ATOM	15248	HW2	SOL	4708	10.570	26.740	8.670	1.00	0.00
ATOM	15249	OW	SOL	4709	34.400	37.460	25.970	1.00	0.00
ATOM	15250	HW1	SOL	4709	33.810	36.900	25.470	1.00	0.00
ATOM	15251	HW2	SOL	4709	35.010	37.820	25.320	1.00	0.00
ATOM	15252	OW	SOL	4710	26.780	5.060	44.870	1.00	0.00
ATOM	15253	HW1	SOL	4710	25.870	5.350	44.850	1.00	0.00
ATOM	15254	HW2	SOL	4710	27.190	5.510	44.130	1.00	0.00
ATOM	15255	OW	SOL	4711	2.910	42.110	4.260	1.00	0.00
ATOM	15256	HW1	SOL	4711	3.410	42.660	4.870	1.00	0.00
ATOM	15257	HW2	SOL	4711	3.030	41.220	4.590	1.00	0.00
ATOM	15258	OW	SOL	4712	11.700	18.150	35.620	1.00	0.00
ATOM	15259	HW1	SOL	4712	11.630	18.710	34.850	1.00	0.00
ATOM	15260	HW2	SOL	4712	11.400	18.690	36.350	1.00	0.00
ATOM	15261	OW	SOL	4713	35.790	53.700	14.190	1.00	0.00
ATOM	15262	HW1	SOL	4713	35.010	53.800	14.730	1.00	0.00
ATOM	15263	HW2	SOL	4713	36.340	53.070	14.660	1.00	0.00
ATOM	15264	OW	SOL	4714	15.430	21.150	43.720	1.00	0.00
ATOM	15265	HW1	SOL	4714	16.370	21.080	43.550	1.00	0.00
ATOM	15266	HW2	SOL	4714	15.310	20.730	44.580	1.00	0.00
ATOM	15267	OW	SOL	4715	37.310	48.770	52.210	1.00	0.00

ATOM	15268	HW1	SOL	4715	38.220	48.970	51.970	1.00	0.00
ATOM	15269	HW2	SOL	4715	37.280	48.920	53.150	1.00	0.00
ATOM	15270	OW	SOL	4716	27.410	29.340	19.460	1.00	0.00
ATOM	15271	HW1	SOL	4716	27.520	30.270	19.280	1.00	0.00
ATOM	15272	HW2	SOL	4716	26.720	29.310	20.130	1.00	0.00
ATOM	15273	OW	SOL	4717	50.280	7.710	11.890	1.00	0.00
ATOM	15274	HW1	SOL	4717	49.560	8.230	12.240	1.00	0.00
ATOM	15275	HW2	SOL	4717	50.400	8.040	11.000	1.00	0.00
ATOM	15276	OW	SOL	4718	4.820	49.650	36.050	1.00	0.00
ATOM	15277	HW1	SOL	4718	5.310	50.240	36.610	1.00	0.00
ATOM	15278	HW2	SOL	4718	5.440	49.410	35.350	1.00	0.00
ATOM	15279	OW	SOL	4719	53.050	24.740	32.730	1.00	0.00
ATOM	15280	HW1	SOL	4719	52.300	24.310	32.310	1.00	0.00
ATOM	15281	HW2	SOL	4719	53.170	24.250	33.550	1.00	0.00
ATOM	15282	OW	SOL	4720	3.860	53.440	9.800	1.00	0.00
ATOM	15283	HW1	SOL	4720	4.030	52.660	9.270	1.00	0.00
ATOM	15284	HW2	SOL	4720	4.200	53.210	10.670	1.00	0.00
ATOM	15285	OW	SOL	4721	39.160	18.890	42.070	1.00	0.00
ATOM	15286	HW1	SOL	4721	39.430	19.560	42.690	1.00	0.00
ATOM	15287	HW2	SOL	4721	38.780	19.380	41.330	1.00	0.00
ATOM	15288	OW	SOL	4722	25.190	13.870	34.750	1.00	0.00
ATOM	15289	HW1	SOL	4722	25.220	14.310	33.900	1.00	0.00
ATOM	15290	HW2	SOL	4722	26.000	14.130	35.180	1.00	0.00
ATOM	15291	OW	SOL	4723	32.660	51.680	7.950	1.00	0.00
ATOM	15292	HW1	SOL	4723	32.620	51.570	7.000	1.00	0.00
ATOM	15293	HW2	SOL	4723	31.980	52.320	8.150	1.00	0.00
ATOM	15294	OW	SOL	4724	47.760	9.030	12.590	1.00	0.00
ATOM	15295	HW1	SOL	4724	47.580	9.680	13.280	1.00	0.00
ATOM	15296	HW2	SOL	4724	47.890	9.560	11.800	1.00	0.00
ATOM	15297	OW	SOL	4725	33.360	39.660	2.730	1.00	0.00
ATOM	15298	HW1	SOL	4725	32.690	39.830	3.390	1.00	0.00
ATOM	15299	HW2	SOL	4725	34.140	39.440	3.230	1.00	0.00
ATOM	15300	OW	SOL	4726	19.190	9.930	7.550	1.00	0.00
ATOM	15301	HW1	SOL	4726	18.450	9.700	7.000	1.00	0.00
ATOM	15302	HW2	SOL	4726	18.950	9.620	8.430	1.00	0.00
ATOM	15303	OW	SOL	4727	48.120	12.020	46.770	1.00	0.00
ATOM	15304	HW1	SOL	4727	48.700	11.910	46.020	1.00	0.00
ATOM	15305	HW2	SOL	4727	47.680	12.850	46.630	1.00	0.00
ATOM	15306	OW	SOL	4728	11.970	2.460	19.310	1.00	0.00
ATOM	15307	HW1	SOL	4728	11.350	2.210	18.620	1.00	0.00
ATOM	15308	HW2	SOL	4728	11.460	2.420	20.110	1.00	0.00
ATOM	15309	OW	SOL	4729	14.920	42.480	36.580	1.00	0.00
ATOM	15310	HW1	SOL	4729	15.390	42.990	35.920	1.00	0.00
ATOM	15311	HW2	SOL	4729	15.140	42.900	37.410	1.00	0.00

ATOM	15312	OW	SOL	4730	2.840	44.200	49.950	1.00	0.00
ATOM	15313	HW1	SOL	4730	3.480	44.870	49.720	1.00	0.00
ATOM	15314	HW2	SOL	4730	3.290	43.370	49.810	1.00	0.00
ATOM	15315	OW	SOL	4731	45.630	11.350	5.180	1.00	0.00
ATOM	15316	HW1	SOL	4731	46.200	10.920	4.540	1.00	0.00
ATOM	15317	HW2	SOL	4731	44.770	10.970	5.030	1.00	0.00
ATOM	15318	OW	SOL	4732	54.550	32.010	47.500	1.00	0.00
ATOM	15319	HW1	SOL	4732	54.760	31.830	48.420	1.00	0.00
ATOM	15320	HW2	SOL	4732	53.920	31.330	47.250	1.00	0.00
ATOM	15321	OW	SOL	4733	46.340	26.460	54.540	1.00	0.00
ATOM	15322	HW1	SOL	4733	46.960	25.870	54.110	1.00	0.00
ATOM	15323	HW2	SOL	4733	46.810	26.780	55.310	1.00	0.00
ATOM	15324	OW	SOL	4734	22.780	23.670	10.340	1.00	0.00
ATOM	15325	HW1	SOL	4734	23.660	23.350	10.110	1.00	0.00
ATOM	15326	HW2	SOL	4734	22.570	24.300	9.650	1.00	0.00
ATOM	15327	OW	SOL	4735	14.280	1.210	41.740	1.00	0.00
ATOM	15328	HW1	SOL	4735	14.710	0.480	41.300	1.00	0.00
ATOM	15329	HW2	SOL	4735	14.120	0.900	42.630	1.00	0.00
ATOM	15330	OW	SOL	4736	54.080	2.160	36.950	1.00	0.00
ATOM	15331	HW1	SOL	4736	53.200	2.370	36.650	1.00	0.00
ATOM	15332	HW2	SOL	4736	54.060	1.220	37.130	1.00	0.00
ATOM	15333	OW	SOL	4737	43.100	32.830	14.730	1.00	0.00
ATOM	15334	HW1	SOL	4737	43.500	32.840	13.870	1.00	0.00
ATOM	15335	HW2	SOL	4737	42.230	32.440	14.590	1.00	0.00
ATOM	15336	OW	SOL	4738	48.600	18.500	48.010	1.00	0.00
ATOM	15337	HW1	SOL	4738	48.300	17.720	47.550	1.00	0.00
ATOM	15338	HW2	SOL	4738	49.000	18.170	48.820	1.00	0.00
ATOM	15339	OW	SOL	4739	50.950	2.750	36.360	1.00	0.00
ATOM	15340	HW1	SOL	4739	50.740	2.550	37.270	1.00	0.00
ATOM	15341	HW2	SOL	4739	50.120	3.010	35.970	1.00	0.00
ATOM	15342	OW	SOL	4740	9.390	25.500	36.380	1.00	0.00
ATOM	15343	HW1	SOL	4740	8.840	25.030	35.760	1.00	0.00
ATOM	15344	HW2	SOL	4740	9.960	26.040	35.830	1.00	0.00
ATOM	15345	OW	SOL	4741	19.490	4.330	14.060	1.00	0.00
ATOM	15346	HW1	SOL	4741	19.960	3.710	14.620	1.00	0.00
ATOM	15347	HW2	SOL	4741	19.090	4.940	14.680	1.00	0.00
ATOM	15348	OW	SOL	4742	2.970	17.160	24.560	1.00	0.00
ATOM	15349	HW1	SOL	4742	3.720	16.590	24.420	1.00	0.00
ATOM	15350	HW2	SOL	4742	3.200	17.690	25.330	1.00	0.00
ATOM	15351	OW	SOL	4743	12.370	43.160	37.030	1.00	0.00
ATOM	15352	HW1	SOL	4743	13.220	42.990	36.620	1.00	0.00
ATOM	15353	HW2	SOL	4743	12.370	42.610	37.810	1.00	0.00
ATOM	15354	OW	SOL	4744	25.120	0.550	28.190	1.00	0.00
ATOM	15355	HW1	SOL	4744	25.610	0.690	29.000	1.00	0.00

ATOM	15356	HW2	SOL	4744	24.230	0.380	28.470	1.00	0.00
ATOM	15357	OW	SOL	4745	6.530	27.510	10.140	1.00	0.00
ATOM	15358	HW1	SOL	4745	7.010	28.070	10.750	1.00	0.00
ATOM	15359	HW2	SOL	4745	6.240	26.770	10.680	1.00	0.00
ATOM	15360	OW	SOL	4746	29.120	21.890	41.400	1.00	0.00
ATOM	15361	HW1	SOL	4746	28.440	22.200	42.010	1.00	0.00
ATOM	15362	HW2	SOL	4746	28.640	21.530	40.660	1.00	0.00
ATOM	15363	OW	SOL	4747	13.000	12.360	3.600	1.00	0.00
ATOM	15364	HW1	SOL	4747	12.970	11.640	4.230	1.00	0.00
ATOM	15365	HW2	SOL	4747	13.690	12.120	2.980	1.00	0.00
ATOM	15366	OW	SOL	4748	31.750	21.280	10.130	1.00	0.00
ATOM	15367	HW1	SOL	4748	32.000	20.670	10.830	1.00	0.00
ATOM	15368	HW2	SOL	4748	32.560	21.450	9.660	1.00	0.00
ATOM	15369	OW	SOL	4749	48.360	10.190	23.660	1.00	0.00
ATOM	15370	HW1	SOL	4749	47.720	9.550	23.980	1.00	0.00
ATOM	15371	HW2	SOL	4749	48.230	10.200	22.710	1.00	0.00
ATOM	15372	OW	SOL	4750	39.520	46.100	30.220	1.00	0.00
ATOM	15373	HW1	SOL	4750	40.210	46.760	30.110	1.00	0.00
ATOM	15374	HW2	SOL	4750	39.280	46.160	31.150	1.00	0.00
ATOM	15375	OW	SOL	4751	42.940	11.990	11.410	1.00	0.00
ATOM	15376	HW1	SOL	4751	42.260	11.520	10.920	1.00	0.00
ATOM	15377	HW2	SOL	4751	42.560	12.850	11.570	1.00	0.00
ATOM	15378	OW	SOL	4752	44.650	47.530	48.220	1.00	0.00
ATOM	15379	HW1	SOL	4752	44.290	46.740	48.620	1.00	0.00
ATOM	15380	HW2	SOL	4752	44.700	47.320	47.280	1.00	0.00
ATOM	15381	OW	SOL	4753	43.040	52.090	6.090	1.00	0.00
ATOM	15382	HW1	SOL	4753	43.290	53.020	6.110	1.00	0.00
ATOM	15383	HW2	SOL	4753	42.430	51.990	6.820	1.00	0.00
ATOM	15384	OW	SOL	4754	31.740	4.880	19.630	1.00	0.00
ATOM	15385	HW1	SOL	4754	32.140	5.330	18.880	1.00	0.00
ATOM	15386	HW2	SOL	4754	30.870	4.610	19.310	1.00	0.00
ATOM	15387	OW	SOL	4755	35.390	40.430	42.450	1.00	0.00
ATOM	15388	HW1	SOL	4755	35.850	39.960	43.150	1.00	0.00
ATOM	15389	HW2	SOL	4755	34.700	40.920	42.900	1.00	0.00
ATOM	15390	OW	SOL	4756	15.360	14.080	50.880	1.00	0.00
ATOM	15391	HW1	SOL	4756	14.600	14.370	50.380	1.00	0.00
ATOM	15392	HW2	SOL	4756	15.020	13.400	51.470	1.00	0.00
ATOM	15393	OW	SOL	4757	14.200	16.910	35.430	1.00	0.00
ATOM	15394	HW1	SOL	4757	13.930	16.140	34.930	1.00	0.00
ATOM	15395	HW2	SOL	4757	13.390	17.380	35.610	1.00	0.00
ATOM	15396	OW	SOL	4758	27.130	19.360	9.540	1.00	0.00
ATOM	15397	HW1	SOL	4758	26.990	20.240	9.900	1.00	0.00
ATOM	15398	HW2	SOL	4758	28.070	19.310	9.390	1.00	0.00
ATOM	15399	OW	SOL	4759	38.670	1.770	14.980	1.00	0.00

ATOM	15400	HW1	SOL	4759	38.370	0.980	15.430	1.00	0.00
ATOM	15401	HW2	SOL	4759	39.410	1.480	14.440	1.00	0.00
ATOM	15402	OW	SOL	4760	26.860	47.090	48.870	1.00	0.00
ATOM	15403	HW1	SOL	4760	26.090	46.610	48.570	1.00	0.00
ATOM	15404	HW2	SOL	4760	27.600	46.550	48.590	1.00	0.00
ATOM	15405	OW	SOL	4761	35.490	15.810	23.700	1.00	0.00
ATOM	15406	HW1	SOL	4761	35.250	14.900	23.510	1.00	0.00
ATOM	15407	HW2	SOL	4761	35.410	15.880	24.650	1.00	0.00
ATOM	15408	OW	SOL	4762	7.450	2.770	54.590	1.00	0.00
ATOM	15409	HW1	SOL	4762	6.630	2.420	54.930	1.00	0.00
ATOM	15410	HW2	SOL	4762	7.260	3.690	54.390	1.00	0.00
ATOM	15411	OW	SOL	4763	4.470	28.090	41.190	1.00	0.00
ATOM	15412	HW1	SOL	4763	4.340	28.470	42.060	1.00	0.00
ATOM	15413	HW2	SOL	4763	5.420	27.990	41.110	1.00	0.00
ATOM	15414	OW	SOL	4764	41.980	54.490	18.510	1.00	0.00
ATOM	15415	HW1	SOL	4764	42.810	54.160	18.150	1.00	0.00
ATOM	15416	HW2	SOL	4764	42.250	55.160	19.140	1.00	0.00
ATOM	15417	OW	SOL	4765	27.390	40.670	51.090	1.00	0.00
ATOM	15418	HW1	SOL	4765	26.800	39.960	51.350	1.00	0.00
ATOM	15419	HW2	SOL	4765	28.130	40.230	50.680	1.00	0.00
ATOM	15420	OW	SOL	4766	6.220	27.600	54.930	1.00	0.00
ATOM	15421	HW1	SOL	4766	5.340	27.760	55.280	1.00	0.00
ATOM	15422	HW2	SOL	4766	6.210	26.680	54.680	1.00	0.00
ATOM	15423	OW	SOL	4767	53.580	1.380	1.450	1.00	0.00
ATOM	15424	HW1	SOL	4767	53.700	1.560	0.520	1.00	0.00
ATOM	15425	HW2	SOL	4767	53.790	2.210	1.880	1.00	0.00
ATOM	15426	OW	SOL	4768	49.620	18.960	25.670	1.00	0.00
ATOM	15427	HW1	SOL	4768	48.970	18.460	25.180	1.00	0.00
ATOM	15428	HW2	SOL	4768	49.680	18.520	26.520	1.00	0.00
ATOM	15429	OW	SOL	4769	4.630	41.080	11.670	1.00	0.00
ATOM	15430	HW1	SOL	4769	5.480	40.730	11.410	1.00	0.00
ATOM	15431	HW2	SOL	4769	4.170	41.240	10.840	1.00	0.00
ATOM	15432	OW	SOL	4770	23.190	6.310	55.410	1.00	0.00
ATOM	15433	HW1	SOL	4770	24.080	6.410	55.070	1.00	0.00
ATOM	15434	HW2	SOL	4770	22.920	5.430	55.120	1.00	0.00
ATOM	15435	OW	SOL	4771	34.140	7.640	23.560	1.00	0.00
ATOM	15436	HW1	SOL	4771	34.050	8.330	22.910	1.00	0.00
ATOM	15437	HW2	SOL	4771	33.320	7.130	23.490	1.00	0.00
ATOM	15438	OW	SOL	4772	14.540	29.450	0.020	1.00	0.00
ATOM	15439	HW1	SOL	4772	15.070	29.590	-0.770	1.00	0.00
ATOM	15440	HW2	SOL	4772	15.120	29.020	0.630	1.00	0.00
ATOM	15441	OW	SOL	4773	32.730	47.410	34.990	1.00	0.00
ATOM	15442	HW1	SOL	4773	32.790	46.450	34.920	1.00	0.00
ATOM	15443	HW2	SOL	4773	31.790	47.580	35.020	1.00	0.00

ATOM	15444	OW	SOL	4774	35.260	9.060	8.390	1.00	0.00
ATOM	15445	HW1	SOL	4774	36.130	9.400	8.200	1.00	0.00
ATOM	15446	HW2	SOL	4774	34.720	9.380	7.660	1.00	0.00
ATOM	15447	OW	SOL	4775	26.280	8.280	17.450	1.00	0.00
ATOM	15448	HW1	SOL	4775	26.820	8.710	16.790	1.00	0.00
ATOM	15449	HW2	SOL	4775	25.830	9.000	17.900	1.00	0.00
ATOM	15450	OW	SOL	4776	51.590	12.990	9.430	1.00	0.00
ATOM	15451	HW1	SOL	4776	52.320	12.440	9.730	1.00	0.00
ATOM	15452	HW2	SOL	4776	50.970	12.370	9.030	1.00	0.00
ATOM	15453	OW	SOL	4777	26.590	17.300	1.780	1.00	0.00
ATOM	15454	HW1	SOL	4777	25.920	16.850	2.290	1.00	0.00
ATOM	15455	HW2	SOL	4777	27.390	17.210	2.300	1.00	0.00
ATOM	15456	OW	SOL	4778	48.900	5.440	43.750	1.00	0.00
ATOM	15457	HW1	SOL	4778	48.230	5.080	44.320	1.00	0.00
ATOM	15458	HW2	SOL	4778	48.810	4.950	42.930	1.00	0.00
ATOM	15459	OW	SOL	4779	48.550	42.630	30.440	1.00	0.00
ATOM	15460	HW1	SOL	4779	47.720	42.730	30.900	1.00	0.00
ATOM	15461	HW2	SOL	4779	49.220	42.740	31.120	1.00	0.00
ATOM	15462	OW	SOL	4780	49.550	31.130	54.100	1.00	0.00
ATOM	15463	HW1	SOL	4780	48.790	31.120	54.680	1.00	0.00
ATOM	15464	HW2	SOL	4780	50.100	30.420	54.410	1.00	0.00
ATOM	15465	OW	SOL	4781	41.690	52.230	30.420	1.00	0.00
ATOM	15466	HW1	SOL	4781	41.810	51.330	30.100	1.00	0.00
ATOM	15467	HW2	SOL	4781	41.210	52.120	31.240	1.00	0.00
ATOM	15468	OW	SOL	4782	51.140	27.650	33.500	1.00	0.00
ATOM	15469	HW1	SOL	4782	50.540	27.850	34.220	1.00	0.00
ATOM	15470	HW2	SOL	4782	50.680	27.010	32.970	1.00	0.00
ATOM	15471	OW	SOL	4783	18.840	25.570	32.800	1.00	0.00
ATOM	15472	HW1	SOL	4783	18.980	25.980	33.650	1.00	0.00
ATOM	15473	HW2	SOL	4783	18.850	26.300	32.180	1.00	0.00
ATOM	15474	OW	SOL	4784	11.350	14.340	12.170	1.00	0.00
ATOM	15475	HW1	SOL	4784	11.950	13.790	12.680	1.00	0.00
ATOM	15476	HW2	SOL	4784	11.430	14.020	11.270	1.00	0.00
ATOM	15477	OW	SOL	4785	1.180	34.390	15.030	1.00	0.00
ATOM	15478	HW1	SOL	4785	1.850	34.660	15.650	1.00	0.00
ATOM	15479	HW2	SOL	4785	0.350	34.590	15.470	1.00	0.00
ATOM	15480	OW	SOL	4786	43.680	24.970	55.220	1.00	0.00
ATOM	15481	HW1	SOL	4786	43.740	25.340	56.100	1.00	0.00
ATOM	15482	HW2	SOL	4786	44.190	25.580	54.670	1.00	0.00
ATOM	15483	OW	SOL	4787	44.990	8.320	11.310	1.00	0.00
ATOM	15484	HW1	SOL	4787	45.700	8.710	11.820	1.00	0.00
ATOM	15485	HW2	SOL	4787	45.440	7.760	10.680	1.00	0.00
ATOM	15486	OW	SOL	4788	5.440	6.140	11.230	1.00	0.00
ATOM	15487	HW1	SOL	4788	4.870	5.400	11.440	1.00	0.00

ATOM	15488	HW2	SOL	4788	4.840	6.860	11.060	1.00	0.00
ATOM	15489	OW	SOL	4789	29.150	48.610	55.540	1.00	0.00
ATOM	15490	HW1	SOL	4789	29.710	48.110	54.950	1.00	0.00
ATOM	15491	HW2	SOL	4789	29.490	48.400	56.420	1.00	0.00
ATOM	15492	OW	SOL	4790	36.580	14.690	9.710	1.00	0.00
ATOM	15493	HW1	SOL	4790	36.700	13.810	10.050	1.00	0.00
ATOM	15494	HW2	SOL	4790	36.880	15.270	10.410	1.00	0.00
ATOM	15495	OW	SOL	4791	35.620	55.290	27.550	1.00	0.00
ATOM	15496	HW1	SOL	4791	35.020	54.850	28.150	1.00	0.00
ATOM	15497	HW2	SOL	4791	36.290	55.670	28.120	1.00	0.00
ATOM	15498	OW	SOL	4792	23.050	41.480	14.510	1.00	0.00
ATOM	15499	HW1	SOL	4792	22.140	41.700	14.720	1.00	0.00
ATOM	15500	HW2	SOL	4792	23.130	41.650	13.570	1.00	0.00
ATOM	15501	OW	SOL	4793	4.800	3.170	38.020	1.00	0.00
ATOM	15502	HW1	SOL	4793	3.950	3.130	37.590	1.00	0.00
ATOM	15503	HW2	SOL	4793	5.100	4.070	37.870	1.00	0.00
ATOM	15504	OW	SOL	4794	24.480	51.770	4.670	1.00	0.00
ATOM	15505	HW1	SOL	4794	23.840	51.740	3.960	1.00	0.00
ATOM	15506	HW2	SOL	4794	25.230	52.230	4.290	1.00	0.00
ATOM	15507	OW	SOL	4795	0.950	28.050	54.720	1.00	0.00
ATOM	15508	HW1	SOL	4795	1.360	28.320	53.900	1.00	0.00
ATOM	15509	HW2	SOL	4795	0.920	27.100	54.670	1.00	0.00
ATOM	15510	OW	SOL	4796	39.070	53.980	45.440	1.00	0.00
ATOM	15511	HW1	SOL	4796	39.140	53.270	44.800	1.00	0.00
ATOM	15512	HW2	SOL	4796	38.150	53.980	45.710	1.00	0.00
ATOM	15513	OW	SOL	4797	31.850	0.940	41.550	1.00	0.00
ATOM	15514	HW1	SOL	4797	31.920	1.350	42.410	1.00	0.00
ATOM	15515	HW2	SOL	4797	31.990	1.650	40.930	1.00	0.00
ATOM	15516	OW	SOL	4798	22.930	32.060	8.340	1.00	0.00
ATOM	15517	HW1	SOL	4798	23.230	32.330	9.210	1.00	0.00
ATOM	15518	HW2	SOL	4798	23.680	31.600	7.960	1.00	0.00
ATOM	15519	OW	SOL	4799	12.580	10.050	28.130	1.00	0.00
ATOM	15520	HW1	SOL	4799	11.660	10.010	28.380	1.00	0.00
ATOM	15521	HW2	SOL	4799	12.710	9.300	27.550	1.00	0.00
ATOM	15522	OW	SOL	4800	51.930	13.270	44.400	1.00	0.00
ATOM	15523	HW1	SOL	4800	51.130	12.750	44.480	1.00	0.00
ATOM	15524	HW2	SOL	4800	51.850	13.930	45.090	1.00	0.00
ATOM	15525	OW	SOL	4801	22.190	47.090	53.440	1.00	0.00
ATOM	15526	HW1	SOL	4801	22.280	46.320	54.000	1.00	0.00
ATOM	15527	HW2	SOL	4801	23.060	47.490	53.430	1.00	0.00
ATOM	15528	OW	SOL	4802	32.560	55.170	21.260	1.00	0.00
ATOM	15529	HW1	SOL	4802	32.980	55.960	20.920	1.00	0.00
ATOM	15530	HW2	SOL	4802	32.780	55.170	22.190	1.00	0.00
ATOM	15531	OW	SOL	4803	31.050	46.460	18.760	1.00	0.00

ATOM	15532	HW1	SOL	4803	30.950	46.300	19.700	1.00	0.00
ATOM	15533	HW2	SOL	4803	31.520	45.690	18.430	1.00	0.00
ATOM	15534	OW	SOL	4804	14.520	53.220	1.320	1.00	0.00
ATOM	15535	HW1	SOL	4804	14.350	53.290	2.260	1.00	0.00
ATOM	15536	HW2	SOL	4804	14.280	54.070	0.970	1.00	0.00
ATOM	15537	OW	SOL	4805	10.220	50.950	18.460	1.00	0.00
ATOM	15538	HW1	SOL	4805	11.110	50.590	18.440	1.00	0.00
ATOM	15539	HW2	SOL	4805	10.290	51.740	19.000	1.00	0.00
ATOM	15540	OW	SOL	4806	28.200	50.110	42.290	1.00	0.00
ATOM	15541	HW1	SOL	4806	28.330	51.030	42.070	1.00	0.00
ATOM	15542	HW2	SOL	4806	28.860	49.930	42.960	1.00	0.00
ATOM	15543	OW	SOL	4807	33.460	25.670	29.930	1.00	0.00
ATOM	15544	HW1	SOL	4807	32.730	26.290	29.950	1.00	0.00
ATOM	15545	HW2	SOL	4807	33.070	24.830	30.150	1.00	0.00
ATOM	15546	OW	SOL	4808	35.440	5.020	48.980	1.00	0.00
ATOM	15547	HW1	SOL	4808	35.460	4.170	48.530	1.00	0.00
ATOM	15548	HW2	SOL	4808	35.530	5.670	48.280	1.00	0.00
ATOM	15549	OW	SOL	4809	50.830	10.890	19.240	1.00	0.00
ATOM	15550	HW1	SOL	4809	50.740	11.090	20.170	1.00	0.00
ATOM	15551	HW2	SOL	4809	51.490	11.510	18.920	1.00	0.00
ATOM	15552	OW	SOL	4810	48.690	15.060	30.370	1.00	0.00
ATOM	15553	HW1	SOL	4810	49.050	15.480	31.160	1.00	0.00
ATOM	15554	HW2	SOL	4810	48.020	15.670	30.070	1.00	0.00
ATOM	15555	OW	SOL	4811	49.010	9.410	6.700	1.00	0.00
ATOM	15556	HW1	SOL	4811	48.730	9.010	7.530	1.00	0.00
ATOM	15557	HW2	SOL	4811	48.210	9.500	6.190	1.00	0.00
ATOM	15558	OW	SOL	4812	36.460	18.280	23.520	1.00	0.00
ATOM	15559	HW1	SOL	4812	36.090	17.410	23.660	1.00	0.00
ATOM	15560	HW2	SOL	4812	37.410	18.140	23.530	1.00	0.00
ATOM	15561	OW	SOL	4813	35.000	41.550	19.730	1.00	0.00
ATOM	15562	HW1	SOL	4813	34.240	41.300	19.200	1.00	0.00
ATOM	15563	HW2	SOL	4813	35.490	42.140	19.170	1.00	0.00
ATOM	15564	OW	SOL	4814	55.550	46.470	32.330	1.00	0.00
ATOM	15565	HW1	SOL	4814	55.580	46.360	33.280	1.00	0.00
ATOM	15566	HW2	SOL	4814	54.620	46.600	32.130	1.00	0.00
ATOM	15567	OW	SOL	4815	12.580	16.310	24.840	1.00	0.00
ATOM	15568	HW1	SOL	4815	11.630	16.250	24.950	1.00	0.00
ATOM	15569	HW2	SOL	4815	12.920	16.460	25.720	1.00	0.00
ATOM	15570	OW	SOL	4816	48.740	43.920	43.070	1.00	0.00
ATOM	15571	HW1	SOL	4816	48.210	44.700	42.910	1.00	0.00
ATOM	15572	HW2	SOL	4816	49.270	43.830	42.280	1.00	0.00
ATOM	15573	OW	SOL	4817	14.890	3.700	43.080	1.00	0.00
ATOM	15574	HW1	SOL	4817	15.770	4.050	42.920	1.00	0.00
ATOM	15575	HW2	SOL	4817	14.770	3.050	42.380	1.00	0.00

ATOM	15576	OW	SOL	4818	37.670	0.730	9.800	1.00	0.00
ATOM	15577	HW1	SOL	4818	36.970	0.310	10.300	1.00	0.00
ATOM	15578	HW2	SOL	4818	37.530	0.440	8.900	1.00	0.00
ATOM	15579	OW	SOL	4819	53.400	1.040	51.530	1.00	0.00
ATOM	15580	HW1	SOL	4819	53.670	1.910	51.820	1.00	0.00
ATOM	15581	HW2	SOL	4819	52.660	1.210	50.940	1.00	0.00
ATOM	15582	OW	SOL	4820	12.690	5.290	29.860	1.00	0.00
ATOM	15583	HW1	SOL	4820	12.520	5.300	30.800	1.00	0.00
ATOM	15584	HW2	SOL	4820	12.620	6.200	29.590	1.00	0.00
ATOM	15585	OW	SOL	4821	8.480	11.260	39.230	1.00	0.00
ATOM	15586	HW1	SOL	4821	9.020	11.900	38.760	1.00	0.00
ATOM	15587	HW2	SOL	4821	8.870	10.420	39.030	1.00	0.00
ATOM	15588	OW	SOL	4822	15.630	14.320	25.960	1.00	0.00
ATOM	15589	HW1	SOL	4822	15.010	13.870	26.540	1.00	0.00
ATOM	15590	HW2	SOL	4822	15.750	15.190	26.350	1.00	0.00
ATOM	15591	OW	SOL	4823	39.820	51.600	8.380	1.00	0.00
ATOM	15592	HW1	SOL	4823	40.780	51.540	8.390	1.00	0.00
ATOM	15593	HW2	SOL	4823	39.550	51.130	9.170	1.00	0.00
ATOM	15594	OW	SOL	4824	3.930	54.800	43.630	1.00	0.00
ATOM	15595	HW1	SOL	4824	3.700	53.960	43.230	1.00	0.00
ATOM	15596	HW2	SOL	4824	4.880	54.740	43.790	1.00	0.00
ATOM	15597	OW	SOL	4825	39.350	49.860	49.060	1.00	0.00
ATOM	15598	HW1	SOL	4825	39.870	49.820	48.260	1.00	0.00
ATOM	15599	HW2	SOL	4825	39.390	48.970	49.420	1.00	0.00
ATOM	15600	OW	SOL	4826	9.030	9.220	15.250	1.00	0.00
ATOM	15601	HW1	SOL	4826	8.130	9.480	15.060	1.00	0.00
ATOM	15602	HW2	SOL	4826	8.940	8.420	15.770	1.00	0.00
ATOM	15603	OW	SOL	4827	35.920	20.720	24.880	1.00	0.00
ATOM	15604	HW1	SOL	4827	36.510	21.360	24.480	1.00	0.00
ATOM	15605	HW2	SOL	4827	35.950	19.970	24.300	1.00	0.00
ATOM	15606	OW	SOL	4828	12.070	24.420	20.220	1.00	0.00
ATOM	15607	HW1	SOL	4828	11.120	24.580	20.140	1.00	0.00
ATOM	15608	HW2	SOL	4828	12.200	23.580	19.790	1.00	0.00
ATOM	15609	OW	SOL	4829	22.040	17.600	23.410	1.00	0.00
ATOM	15610	HW1	SOL	4829	22.770	17.570	22.790	1.00	0.00
ATOM	15611	HW2	SOL	4829	22.350	17.080	24.160	1.00	0.00
ATOM	15612	OW	SOL	4830	4.100	12.100	39.930	1.00	0.00
ATOM	15613	HW1	SOL	4830	3.740	12.920	40.270	1.00	0.00
ATOM	15614	HW2	SOL	4830	4.550	11.710	40.680	1.00	0.00
ATOM	15615	OW	SOL	4831	38.420	7.010	54.760	1.00	0.00
ATOM	15616	HW1	SOL	4831	39.190	7.300	55.250	1.00	0.00
ATOM	15617	HW2	SOL	4831	37.680	7.340	55.270	1.00	0.00
ATOM	15618	OW	SOL	4832	23.960	55.260	51.560	1.00	0.00
ATOM	15619	HW1	SOL	4832	23.200	54.960	52.050	1.00	0.00

ATOM	15620	HW2	SOL	4832	24.700	54.770	51.930	1.00	0.00
ATOM	15621	OW	SOL	4833	41.670	15.730	8.240	1.00	0.00
ATOM	15622	HW1	SOL	4833	42.480	16.250	8.190	1.00	0.00
ATOM	15623	HW2	SOL	4833	41.890	14.910	7.800	1.00	0.00
ATOM	15624	OW	SOL	4834	39.690	6.700	11.850	1.00	0.00
ATOM	15625	HW1	SOL	4834	39.820	6.710	10.900	1.00	0.00
ATOM	15626	HW2	SOL	4834	40.100	7.500	12.160	1.00	0.00
ATOM	15627	OW	SOL	4835	1.370	46.670	8.760	1.00	0.00
ATOM	15628	HW1	SOL	4835	0.570	47.190	8.760	1.00	0.00
ATOM	15629	HW2	SOL	4835	1.170	45.910	9.300	1.00	0.00
ATOM	15630	OW	SOL	4836	25.280	5.890	12.430	1.00	0.00
ATOM	15631	HW1	SOL	4836	26.190	5.730	12.670	1.00	0.00
ATOM	15632	HW2	SOL	4836	24.880	5.020	12.440	1.00	0.00
ATOM	15633	OW	SOL	4837	49.460	9.390	1.270	1.00	0.00
ATOM	15634	HW1	SOL	4837	50.080	9.840	1.840	1.00	0.00
ATOM	15635	HW2	SOL	4837	49.940	8.620	0.960	1.00	0.00
ATOM	15636	OW	SOL	4838	13.260	31.510	27.670	1.00	0.00
ATOM	15637	HW1	SOL	4838	13.760	32.090	27.090	1.00	0.00
ATOM	15638	HW2	SOL	4838	13.490	30.630	27.380	1.00	0.00
ATOM	15639	OW	SOL	4839	32.650	10.880	3.870	1.00	0.00
ATOM	15640	HW1	SOL	4839	32.810	11.470	3.130	1.00	0.00
ATOM	15641	HW2	SOL	4839	31.710	10.940	4.030	1.00	0.00
ATOM	15642	OW	SOL	4840	28.420	45.550	52.710	1.00	0.00
ATOM	15643	HW1	SOL	4840	28.230	44.610	52.670	1.00	0.00
ATOM	15644	HW2	SOL	4840	27.560	45.970	52.630	1.00	0.00
ATOM	15645	OW	SOL	4841	18.150	13.580	25.080	1.00	0.00
ATOM	15646	HW1	SOL	4841	18.230	14.210	24.370	1.00	0.00
ATOM	15647	HW2	SOL	4841	17.250	13.680	25.400	1.00	0.00
ATOM	15648	OW	SOL	4842	17.700	16.390	32.630	1.00	0.00
ATOM	15649	HW1	SOL	4842	18.310	15.950	33.230	1.00	0.00
ATOM	15650	HW2	SOL	4842	18.030	17.290	32.580	1.00	0.00
ATOM	15651	OW	SOL	4843	46.050	15.770	16.720	1.00	0.00
ATOM	15652	HW1	SOL	4843	45.110	15.930	16.690	1.00	0.00
ATOM	15653	HW2	SOL	4843	46.320	16.090	17.580	1.00	0.00
ATOM	15654	OW	SOL	4844	9.760	21.840	24.820	1.00	0.00
ATOM	15655	HW1	SOL	4844	9.430	21.420	25.620	1.00	0.00
ATOM	15656	HW2	SOL	4844	10.350	22.520	25.150	1.00	0.00
ATOM	15657	OW	SOL	4845	28.880	20.040	5.940	1.00	0.00
ATOM	15658	HW1	SOL	4845	29.560	20.160	6.590	1.00	0.00
ATOM	15659	HW2	SOL	4845	28.340	19.330	6.290	1.00	0.00
ATOM	15660	OW	SOL	4846	34.050	10.740	14.460	1.00	0.00
ATOM	15661	HW1	SOL	4846	34.810	10.400	14.930	1.00	0.00
ATOM	15662	HW2	SOL	4846	33.390	10.900	15.150	1.00	0.00
ATOM	15663	OW	SOL	4847	23.630	0.060	20.010	1.00	0.00

ATOM	15664	HW1	SOL	4847	23.090	-0.010	19.220	1.00	0.00
ATOM	15665	HW2	SOL	4847	23.510	-0.770	20.460	1.00	0.00
ATOM	15666	OW	SOL	4848	22.310	39.810	22.270	1.00	0.00
ATOM	15667	HW1	SOL	4848	22.870	40.540	22.530	1.00	0.00
ATOM	15668	HW2	SOL	4848	22.400	39.770	21.320	1.00	0.00
ATOM	15669	OW	SOL	4849	10.430	36.460	28.810	1.00	0.00
ATOM	15670	HW1	SOL	4849	10.270	36.220	27.900	1.00	0.00
ATOM	15671	HW2	SOL	4849	9.560	36.580	29.190	1.00	0.00
ATOM	15672	OW	SOL	4850	31.910	23.700	4.980	1.00	0.00
ATOM	15673	HW1	SOL	4850	32.230	24.030	4.150	1.00	0.00
ATOM	15674	HW2	SOL	4850	31.060	24.130	5.100	1.00	0.00
ATOM	15675	OW	SOL	4851	4.480	21.590	2.560	1.00	0.00
ATOM	15676	HW1	SOL	4851	5.400	21.620	2.840	1.00	0.00
ATOM	15677	HW2	SOL	4851	4.400	22.300	1.930	1.00	0.00
ATOM	15678	OW	SOL	4852	32.880	14.490	26.070	1.00	0.00
ATOM	15679	HW1	SOL	4852	33.790	14.240	26.250	1.00	0.00
ATOM	15680	HW2	SOL	4852	32.940	15.370	25.690	1.00	0.00
ATOM	15681	OW	SOL	4853	43.300	13.340	0.460	1.00	0.00
ATOM	15682	HW1	SOL	4853	44.090	13.050	0.920	1.00	0.00
ATOM	15683	HW2	SOL	4853	42.910	12.540	0.120	1.00	0.00
ATOM	15684	OW	SOL	4854	6.830	2.370	36.140	1.00	0.00
ATOM	15685	HW1	SOL	4854	6.030	2.560	36.640	1.00	0.00
ATOM	15686	HW2	SOL	4854	7.510	2.880	36.580	1.00	0.00
ATOM	15687	OW	SOL	4855	52.150	8.990	13.310	1.00	0.00
ATOM	15688	HW1	SOL	4855	52.020	8.270	12.690	1.00	0.00
ATOM	15689	HW2	SOL	4855	52.350	9.740	12.750	1.00	0.00
ATOM	15690	OW	SOL	4856	53.460	41.710	45.400	1.00	0.00
ATOM	15691	HW1	SOL	4856	53.660	42.330	46.090	1.00	0.00
ATOM	15692	HW2	SOL	4856	52.680	42.070	44.970	1.00	0.00
ATOM	15693	OW	SOL	4857	23.510	42.340	5.120	1.00	0.00
ATOM	15694	HW1	SOL	4857	23.540	42.860	5.920	1.00	0.00
ATOM	15695	HW2	SOL	4857	22.600	42.050	5.050	1.00	0.00
ATOM	15696	OW	SOL	4858	24.810	21.530	4.930	1.00	0.00
ATOM	15697	HW1	SOL	4858	24.180	21.010	4.430	1.00	0.00
ATOM	15698	HW2	SOL	4858	25.530	21.700	4.320	1.00	0.00
ATOM	15699	OW	SOL	4859	38.570	1.600	55.850	1.00	0.00
ATOM	15700	HW1	SOL	4859	37.910	1.530	55.170	1.00	0.00
ATOM	15701	HW2	SOL	4859	39.220	0.930	55.630	1.00	0.00
ATOM	15702	OW	SOL	4860	0.830	40.850	25.530	1.00	0.00
ATOM	15703	HW1	SOL	4860	0.390	40.800	24.680	1.00	0.00
ATOM	15704	HW2	SOL	4860	0.140	40.640	26.160	1.00	0.00
ATOM	15705	OW	SOL	4861	48.080	46.570	27.000	1.00	0.00
ATOM	15706	HW1	SOL	4861	48.830	47.070	27.330	1.00	0.00
ATOM	15707	HW2	SOL	4861	48.000	45.830	27.600	1.00	0.00

ATOM	15708	OW	SOL	4862	6.970	47.790	44.450	1.00	0.00
ATOM	15709	HW1	SOL	4862	7.670	47.830	43.800	1.00	0.00
ATOM	15710	HW2	SOL	4862	6.570	48.660	44.430	1.00	0.00
ATOM	15711	OW	SOL	4863	29.500	2.320	42.970	1.00	0.00
ATOM	15712	HW1	SOL	4863	28.790	1.870	42.520	1.00	0.00
ATOM	15713	HW2	SOL	4863	29.070	3.020	43.460	1.00	0.00
ATOM	15714	OW	SOL	4864	7.460	52.990	55.080	1.00	0.00
ATOM	15715	HW1	SOL	4864	7.730	53.820	54.690	1.00	0.00
ATOM	15716	HW2	SOL	4864	7.210	52.450	54.330	1.00	0.00
ATOM	15717	OW	SOL	4865	32.630	32.920	18.960	1.00	0.00
ATOM	15718	HW1	SOL	4865	32.100	32.570	19.670	1.00	0.00
ATOM	15719	HW2	SOL	4865	32.210	32.590	18.160	1.00	0.00
ATOM	15720	OW	SOL	4866	26.260	3.460	37.700	1.00	0.00
ATOM	15721	HW1	SOL	4866	26.590	2.590	37.920	1.00	0.00
ATOM	15722	HW2	SOL	4866	27.010	4.040	37.810	1.00	0.00
ATOM	15723	OW	SOL	4867	9.170	21.990	20.410	1.00	0.00
ATOM	15724	HW1	SOL	4867	8.680	21.200	20.170	1.00	0.00
ATOM	15725	HW2	SOL	4867	8.890	22.190	21.300	1.00	0.00
ATOM	15726	OW	SOL	4868	7.010	23.500	38.840	1.00	0.00
ATOM	15727	HW1	SOL	4868	6.200	23.440	39.350	1.00	0.00
ATOM	15728	HW2	SOL	4868	7.190	24.440	38.790	1.00	0.00
ATOM	15729	OW	SOL	4869	24.350	18.650	53.800	1.00	0.00
ATOM	15730	HW1	SOL	4869	24.370	17.890	54.380	1.00	0.00
ATOM	15731	HW2	SOL	4869	24.080	19.370	54.360	1.00	0.00
ATOM	15732	OW	SOL	4870	6.030	24.730	35.620	1.00	0.00
ATOM	15733	HW1	SOL	4870	5.550	24.950	34.830	1.00	0.00
ATOM	15734	HW2	SOL	4870	6.010	25.530	36.150	1.00	0.00
ATOM	15735	OW	SOL	4871	38.840	14.220	1.940	1.00	0.00
ATOM	15736	HW1	SOL	4871	39.460	13.880	2.580	1.00	0.00
ATOM	15737	HW2	SOL	4871	38.030	14.340	2.440	1.00	0.00
ATOM	15738	OW	SOL	4872	16.320	16.330	27.670	1.00	0.00
ATOM	15739	HW1	SOL	4872	15.390	16.360	27.890	1.00	0.00
ATOM	15740	HW2	SOL	4872	16.640	17.220	27.850	1.00	0.00
ATOM	15741	OW	SOL	4873	11.690	29.050	33.010	1.00	0.00
ATOM	15742	HW1	SOL	4873	10.810	29.260	32.690	1.00	0.00
ATOM	15743	HW2	SOL	4873	11.900	28.210	32.610	1.00	0.00
ATOM	15744	OW	SOL	4874	14.760	36.050	30.180	1.00	0.00
ATOM	15745	HW1	SOL	4874	15.470	36.500	29.730	1.00	0.00
ATOM	15746	HW2	SOL	4874	15.190	35.550	30.880	1.00	0.00
ATOM	15747	OW	SOL	4875	9.380	3.360	50.410	1.00	0.00
ATOM	15748	HW1	SOL	4875	8.560	3.380	49.920	1.00	0.00
ATOM	15749	HW2	SOL	4875	10.030	3.720	49.800	1.00	0.00
ATOM	15750	OW	SOL	4876	14.190	6.750	54.920	1.00	0.00
ATOM	15751	HW1	SOL	4876	14.510	6.960	54.040	1.00	0.00

ATOM	15752	HW2	SOL	4876	14.950	6.380	55.360	1.00	0.00
ATOM	15753	OW	SOL	4877	18.300	40.500	22.190	1.00	0.00
ATOM	15754	HW1	SOL	4877	18.470	41.350	22.600	1.00	0.00
ATOM	15755	HW2	SOL	4877	17.350	40.480	22.070	1.00	0.00
ATOM	15756	OW	SOL	4878	46.620	54.550	34.990	1.00	0.00
ATOM	15757	HW1	SOL	4878	47.520	54.220	35.070	1.00	0.00
ATOM	15758	HW2	SOL	4878	46.070	53.770	34.980	1.00	0.00
ATOM	15759	OW	SOL	4879	12.680	46.030	51.780	1.00	0.00
ATOM	15760	HW1	SOL	4879	12.130	45.290	51.530	1.00	0.00
ATOM	15761	HW2	SOL	4879	12.940	46.420	50.940	1.00	0.00
ATOM	15762	OW	SOL	4880	48.200	31.460	13.720	1.00	0.00
ATOM	15763	HW1	SOL	4880	47.330	31.360	13.340	1.00	0.00
ATOM	15764	HW2	SOL	4880	48.650	30.640	13.510	1.00	0.00
ATOM	15765	OW	SOL	4881	3.750	52.960	23.730	1.00	0.00
ATOM	15766	HW1	SOL	4881	3.710	53.720	24.300	1.00	0.00
ATOM	15767	HW2	SOL	4881	2.870	52.880	23.380	1.00	0.00
ATOM	15768	OW	SOL	4882	10.250	0.690	27.970	1.00	0.00
ATOM	15769	HW1	SOL	4882	9.580	1.040	27.390	1.00	0.00
ATOM	15770	HW2	SOL	4882	10.700	0.030	27.440	1.00	0.00
ATOM	15771	OW	SOL	4883	38.060	14.980	24.910	1.00	0.00
ATOM	15772	HW1	SOL	4883	37.800	14.050	24.930	1.00	0.00
ATOM	15773	HW2	SOL	4883	37.430	15.410	25.490	1.00	0.00
ATOM	15774	OW	SOL	4884	38.270	53.140	21.060	1.00	0.00
ATOM	15775	HW1	SOL	4884	38.090	54.070	20.960	1.00	0.00
ATOM	15776	HW2	SOL	4884	39.120	53.110	21.490	1.00	0.00
ATOM	15777	OW	SOL	4885	47.110	41.710	38.240	1.00	0.00
ATOM	15778	HW1	SOL	4885	46.300	41.980	37.820	1.00	0.00
ATOM	15779	HW2	SOL	4885	47.540	42.530	38.500	1.00	0.00
ATOM	15780	OW	SOL	4886	5.000	25.530	4.740	1.00	0.00
ATOM	15781	HW1	SOL	4886	5.730	24.950	4.510	1.00	0.00
ATOM	15782	HW2	SOL	4886	5.410	26.340	5.040	1.00	0.00
ATOM	15783	OW	SOL	4887	20.320	45.880	49.890	1.00	0.00
ATOM	15784	HW1	SOL	4887	20.680	45.260	49.260	1.00	0.00
ATOM	15785	HW2	SOL	4887	20.690	46.730	49.630	1.00	0.00
ATOM	15786	OW	SOL	4888	21.010	7.570	47.520	1.00	0.00
ATOM	15787	HW1	SOL	4888	20.400	7.430	46.800	1.00	0.00
ATOM	15788	HW2	SOL	4888	21.490	8.360	47.280	1.00	0.00
ATOM	15789	OW	SOL	4889	25.170	42.830	47.040	1.00	0.00
ATOM	15790	HW1	SOL	4889	24.770	42.280	46.370	1.00	0.00
ATOM	15791	HW2	SOL	4889	24.750	42.570	47.860	1.00	0.00
ATOM	15792	OW	SOL	4890	47.030	13.670	23.770	1.00	0.00
ATOM	15793	HW1	SOL	4890	46.350	13.030	23.570	1.00	0.00
ATOM	15794	HW2	SOL	4890	46.600	14.520	23.670	1.00	0.00
ATOM	15795	OW	SOL	4891	27.410	7.090	7.030	1.00	0.00

ATOM	15796	HW1	SOL	4891	27.460	7.040	7.980	1.00	0.00
ATOM	15797	HW2	SOL	4891	27.050	7.960	6.860	1.00	0.00
ATOM	15798	OW	SOL	4892	33.700	48.820	47.930	1.00	0.00
ATOM	15799	HW1	SOL	4892	34.620	48.890	47.690	1.00	0.00
ATOM	15800	HW2	SOL	4892	33.550	49.560	48.530	1.00	0.00
ATOM	15801	OW	SOL	4893	43.110	9.360	26.730	1.00	0.00
ATOM	15802	HW1	SOL	4893	43.960	8.920	26.810	1.00	0.00
ATOM	15803	HW2	SOL	4893	42.750	9.040	25.910	1.00	0.00
ATOM	15804	OW	SOL	4894	32.040	3.040	39.900	1.00	0.00
ATOM	15805	HW1	SOL	4894	32.370	3.720	39.300	1.00	0.00
ATOM	15806	HW2	SOL	4894	31.250	3.420	40.280	1.00	0.00
ATOM	15807	OW	SOL	4895	49.730	40.800	44.950	1.00	0.00
ATOM	15808	HW1	SOL	4895	50.260	41.450	44.490	1.00	0.00
ATOM	15809	HW2	SOL	4895	48.960	40.670	44.390	1.00	0.00
ATOM	15810	OW	SOL	4896	37.800	42.110	12.830	1.00	0.00
ATOM	15811	HW1	SOL	4896	37.800	42.240	11.890	1.00	0.00
ATOM	15812	HW2	SOL	4896	36.880	42.050	13.070	1.00	0.00
ATOM	15813	OW	SOL	4897	24.380	39.070	19.330	1.00	0.00
ATOM	15814	HW1	SOL	4897	24.160	39.570	18.540	1.00	0.00
ATOM	15815	HW2	SOL	4897	23.930	38.230	19.210	1.00	0.00
ATOM	15816	OW	SOL	4898	41.390	44.930	55.900	1.00	0.00
ATOM	15817	HW1	SOL	4898	41.100	45.830	56.090	1.00	0.00
ATOM	15818	HW2	SOL	4898	40.750	44.370	56.340	1.00	0.00
ATOM	15819	OW	SOL	4899	6.940	42.010	31.130	1.00	0.00
ATOM	15820	HW1	SOL	4899	6.570	42.890	31.170	1.00	0.00
ATOM	15821	HW2	SOL	4899	7.490	42.020	30.350	1.00	0.00
ATOM	15822	OW	SOL	4900	1.300	35.410	36.360	1.00	0.00
ATOM	15823	HW1	SOL	4900	1.200	35.320	37.310	1.00	0.00
ATOM	15824	HW2	SOL	4900	1.740	34.610	36.090	1.00	0.00
ATOM	15825	OW	SOL	4901	15.370	35.400	36.750	1.00	0.00
ATOM	15826	HW1	SOL	4901	15.890	35.660	35.990	1.00	0.00
ATOM	15827	HW2	SOL	4901	15.830	35.800	37.490	1.00	0.00
ATOM	15828	OW	SOL	4902	50.010	50.460	44.260	1.00	0.00
ATOM	15829	HW1	SOL	4902	49.290	51.020	44.570	1.00	0.00
ATOM	15830	HW2	SOL	4902	50.270	50.860	43.430	1.00	0.00
ATOM	15831	OW	SOL	4903	39.800	51.580	40.310	1.00	0.00
ATOM	15832	HW1	SOL	4903	40.470	51.510	39.630	1.00	0.00
ATOM	15833	HW2	SOL	4903	39.500	50.680	40.440	1.00	0.00
ATOM	15834	OW	SOL	4904	53.660	4.330	0.300	1.00	0.00
ATOM	15835	HW1	SOL	4904	54.050	3.570	-0.130	1.00	0.00
ATOM	15836	HW2	SOL	4904	53.850	5.070	-0.280	1.00	0.00
ATOM	15837	OW	SOL	4905	25.030	20.540	29.700	1.00	0.00
ATOM	15838	HW1	SOL	4905	24.220	20.470	30.210	1.00	0.00
ATOM	15839	HW2	SOL	4905	25.730	20.530	30.350	1.00	0.00

ATOM	15840	OW	SOL	4906	23.670	11.710	28.790	1.00	0.00
ATOM	15841	HW1	SOL	4906	23.530	12.330	28.080	1.00	0.00
ATOM	15842	HW2	SOL	4906	24.600	11.820	29.020	1.00	0.00
ATOM	15843	OW	SOL	4907	10.080	44.100	6.670	1.00	0.00
ATOM	15844	HW1	SOL	4907	9.750	44.030	7.570	1.00	0.00
ATOM	15845	HW2	SOL	4907	11.030	44.120	6.750	1.00	0.00
ATOM	15846	OW	SOL	4908	21.700	14.480	55.640	1.00	0.00
ATOM	15847	HW1	SOL	4908	21.900	14.350	56.570	1.00	0.00
ATOM	15848	HW2	SOL	4908	21.250	13.680	55.380	1.00	0.00
ATOM	15849	OW	SOL	4909	52.950	15.440	31.530	1.00	0.00
ATOM	15850	HW1	SOL	4909	52.870	16.330	31.200	1.00	0.00
ATOM	15851	HW2	SOL	4909	53.130	15.550	32.470	1.00	0.00
ATOM	15852	OW	SOL	4910	36.980	11.890	45.720	1.00	0.00
ATOM	15853	HW1	SOL	4910	37.830	12.270	45.520	1.00	0.00
ATOM	15854	HW2	SOL	4910	36.850	11.230	45.040	1.00	0.00
ATOM	15855	OW	SOL	4911	27.870	14.660	30.400	1.00	0.00
ATOM	15856	HW1	SOL	4911	27.020	15.070	30.590	1.00	0.00
ATOM	15857	HW2	SOL	4911	28.510	15.360	30.520	1.00	0.00
ATOM	15858	OW	SOL	4912	26.160	16.850	51.010	1.00	0.00
ATOM	15859	HW1	SOL	4912	25.940	15.950	50.770	1.00	0.00
ATOM	15860	HW2	SOL	4912	25.590	17.390	50.460	1.00	0.00
ATOM	15861	OW	SOL	4913	53.330	35.410	14.110	1.00	0.00
ATOM	15862	HW1	SOL	4913	53.820	36.230	14.100	1.00	0.00
ATOM	15863	HW2	SOL	4913	53.400	35.100	15.010	1.00	0.00
ATOM	15864	OW	SOL	4914	31.720	22.540	23.730	1.00	0.00
ATOM	15865	HW1	SOL	4914	31.650	22.460	22.780	1.00	0.00
ATOM	15866	HW2	SOL	4914	32.230	21.780	24.010	1.00	0.00
ATOM	15867	OW	SOL	4915	2.540	17.590	12.600	1.00	0.00
ATOM	15868	HW1	SOL	4915	1.890	16.930	12.350	1.00	0.00
ATOM	15869	HW2	SOL	4915	3.150	17.130	13.170	1.00	0.00
ATOM	15870	OW	SOL	4916	44.490	12.900	22.650	1.00	0.00
ATOM	15871	HW1	SOL	4916	43.620	13.170	22.940	1.00	0.00
ATOM	15872	HW2	SOL	4916	44.450	12.970	21.690	1.00	0.00
ATOM	15873	OW	SOL	4917	34.490	37.980	17.670	1.00	0.00
ATOM	15874	HW1	SOL	4917	34.220	38.750	17.170	1.00	0.00
ATOM	15875	HW2	SOL	4917	34.790	37.350	17.010	1.00	0.00
ATOM	15876	OW	SOL	4918	45.570	7.160	55.320	1.00	0.00
ATOM	15877	HW1	SOL	4918	46.530	7.130	55.300	1.00	0.00
ATOM	15878	HW2	SOL	4918	45.330	7.770	54.630	1.00	0.00
ATOM	15879	OW	SOL	4919	1.200	20.030	22.000	1.00	0.00
ATOM	15880	HW1	SOL	4919	0.300	20.300	21.870	1.00	0.00
ATOM	15881	HW2	SOL	4919	1.390	20.240	22.920	1.00	0.00
ATOM	15882	OW	SOL	4920	12.890	38.210	34.210	1.00	0.00
ATOM	15883	HW1	SOL	4920	12.890	37.710	35.030	1.00	0.00

ATOM	15884	HW2	SOL	4920	13.650	37.890	33.730	1.00	0.00
ATOM	15885	OW	SOL	4921	13.790	18.560	20.200	1.00	0.00
ATOM	15886	HW1	SOL	4921	13.960	18.000	19.440	1.00	0.00
ATOM	15887	HW2	SOL	4921	14.250	18.130	20.920	1.00	0.00
ATOM	15888	OW	SOL	4922	12.540	21.660	48.270	1.00	0.00
ATOM	15889	HW1	SOL	4922	13.000	20.950	48.730	1.00	0.00
ATOM	15890	HW2	SOL	4922	12.290	21.270	47.430	1.00	0.00
ATOM	15891	OW	SOL	4923	17.350	39.810	16.300	1.00	0.00
ATOM	15892	HW1	SOL	4923	17.120	39.840	15.370	1.00	0.00
ATOM	15893	HW2	SOL	4923	17.150	40.690	16.630	1.00	0.00
ATOM	15894	OW	SOL	4924	36.100	35.830	3.330	1.00	0.00
ATOM	15895	HW1	SOL	4924	36.810	35.210	3.540	1.00	0.00
ATOM	15896	HW2	SOL	4924	36.500	36.690	3.430	1.00	0.00
ATOM	15897	OW	SOL	4925	4.470	16.880	30.220	1.00	0.00
ATOM	15898	HW1	SOL	4925	4.700	16.730	31.140	1.00	0.00
ATOM	15899	HW2	SOL	4925	3.930	17.670	30.230	1.00	0.00
ATOM	15900	OW	SOL	4926	18.860	30.160	51.620	1.00	0.00
ATOM	15901	HW1	SOL	4926	19.140	30.870	52.200	1.00	0.00
ATOM	15902	HW2	SOL	4926	18.030	30.470	51.250	1.00	0.00
ATOM	15903	OW	SOL	4927	32.130	38.410	19.140	1.00	0.00
ATOM	15904	HW1	SOL	4927	32.800	37.950	18.640	1.00	0.00
ATOM	15905	HW2	SOL	4927	31.360	37.840	19.100	1.00	0.00
ATOM	15906	OW	SOL	4928	38.910	36.750	49.160	1.00	0.00
ATOM	15907	HW1	SOL	4928	38.950	36.000	49.750	1.00	0.00
ATOM	15908	HW2	SOL	4928	38.930	37.510	49.740	1.00	0.00
ATOM	15909	OW	SOL	4929	15.220	42.740	42.010	1.00	0.00
ATOM	15910	HW1	SOL	4929	15.480	43.650	42.130	1.00	0.00
ATOM	15911	HW2	SOL	4929	15.180	42.380	42.900	1.00	0.00
ATOM	15912	OW	SOL	4930	55.020	6.160	54.620	1.00	0.00
ATOM	15913	HW1	SOL	4930	54.800	6.730	53.890	1.00	0.00
ATOM	15914	HW2	SOL	4930	55.910	6.410	54.870	1.00	0.00
ATOM	15915	OW	SOL	4931	44.100	43.180	2.560	1.00	0.00
ATOM	15916	HW1	SOL	4931	43.810	42.770	1.750	1.00	0.00
ATOM	15917	HW2	SOL	4931	43.330	43.650	2.880	1.00	0.00
ATOM	15918	OW	SOL	4932	9.350	3.270	5.930	1.00	0.00
ATOM	15919	HW1	SOL	4932	10.230	3.560	6.200	1.00	0.00
ATOM	15920	HW2	SOL	4932	9.170	2.520	6.490	1.00	0.00
ATOM	15921	OW	SOL	4933	54.740	52.820	52.340	1.00	0.00
ATOM	15922	HW1	SOL	4933	54.030	52.190	52.370	1.00	0.00
ATOM	15923	HW2	SOL	4933	55.290	52.600	53.100	1.00	0.00
ATOM	15924	OW	SOL	4934	26.010	2.410	11.420	1.00	0.00
ATOM	15925	HW1	SOL	4934	25.640	3.220	11.060	1.00	0.00
ATOM	15926	HW2	SOL	4934	25.870	1.760	10.740	1.00	0.00
ATOM	15927	OW	SOL	4935	45.870	41.820	53.090	1.00	0.00

ATOM	15928	HW1	SOL	4935	46.430	41.290	53.660	1.00	0.00
ATOM	15929	HW2	SOL	4935	44.980	41.650	53.420	1.00	0.00
ATOM	15930	OW	SOL	4936	8.630	37.850	17.260	1.00	0.00
ATOM	15931	HW1	SOL	4936	9.550	38.100	17.370	1.00	0.00
ATOM	15932	HW2	SOL	4936	8.380	37.500	18.120	1.00	0.00
ATOM	15933	OW	SOL	4937	12.970	48.210	35.590	1.00	0.00
ATOM	15934	HW1	SOL	4937	12.620	48.220	34.700	1.00	0.00
ATOM	15935	HW2	SOL	4937	12.510	47.480	36.020	1.00	0.00
ATOM	15936	OW	SOL	4938	15.720	18.350	0.940	1.00	0.00
ATOM	15937	HW1	SOL	4938	16.110	17.860	0.210	1.00	0.00
ATOM	15938	HW2	SOL	4938	15.510	17.680	1.590	1.00	0.00
ATOM	15939	OW	SOL	4939	9.360	26.250	44.670	1.00	0.00
ATOM	15940	HW1	SOL	4939	9.190	26.930	44.010	1.00	0.00
ATOM	15941	HW2	SOL	4939	8.500	25.870	44.850	1.00	0.00
ATOM	15942	OW	SOL	4940	22.770	34.080	37.690	1.00	0.00
ATOM	15943	HW1	SOL	4940	23.080	34.960	37.470	1.00	0.00
ATOM	15944	HW2	SOL	4940	22.410	34.170	38.580	1.00	0.00
ATOM	15945	OW	SOL	4941	0.950	44.850	10.840	1.00	0.00
ATOM	15946	HW1	SOL	4941	1.530	44.440	11.480	1.00	0.00
ATOM	15947	HW2	SOL	4941	0.310	45.330	11.360	1.00	0.00
ATOM	15948	OW	SOL	4942	17.850	34.010	20.780	1.00	0.00
ATOM	15949	HW1	SOL	4942	17.440	34.860	20.980	1.00	0.00
ATOM	15950	HW2	SOL	4942	18.710	34.240	20.410	1.00	0.00
ATOM	15951	OW	SOL	4943	15.350	44.440	24.070	1.00	0.00
ATOM	15952	HW1	SOL	4943	14.730	43.890	23.590	1.00	0.00
ATOM	15953	HW2	SOL	4943	16.210	44.110	23.810	1.00	0.00
ATOM	15954	OW	SOL	4944	30.990	0.190	2.110	1.00	0.00
ATOM	15955	HW1	SOL	4944	31.310	0.840	2.740	1.00	0.00
ATOM	15956	HW2	SOL	4944	31.740	-0.370	1.930	1.00	0.00
ATOM	15957	OW	SOL	4945	13.850	26.110	6.050	1.00	0.00
ATOM	15958	HW1	SOL	4945	14.480	25.380	6.040	1.00	0.00
ATOM	15959	HW2	SOL	4945	13.450	26.100	5.190	1.00	0.00
ATOM	15960	OW	SOL	4946	6.210	50.950	17.250	1.00	0.00
ATOM	15961	HW1	SOL	4946	6.050	50.860	18.190	1.00	0.00
ATOM	15962	HW2	SOL	4946	5.550	51.570	16.950	1.00	0.00
ATOM	15963	OW	SOL	4947	24.750	10.490	21.540	1.00	0.00
ATOM	15964	HW1	SOL	4947	23.920	10.880	21.810	1.00	0.00
ATOM	15965	HW2	SOL	4947	25.410	11.120	21.800	1.00	0.00
ATOM	15966	OW	SOL	4948	36.980	15.350	18.200	1.00	0.00
ATOM	15967	HW1	SOL	4948	37.910	15.170	18.030	1.00	0.00
ATOM	15968	HW2	SOL	4948	36.990	15.910	18.980	1.00	0.00
ATOM	15969	OW	SOL	4949	35.570	18.220	11.060	1.00	0.00
ATOM	15970	HW1	SOL	4949	35.340	17.460	11.590	1.00	0.00
ATOM	15971	HW2	SOL	4949	34.940	18.210	10.340	1.00	0.00

ATOM	15972	OW	SOL	4950	11.690	47.230	10.040	1.00	0.00
ATOM	15973	HW1	SOL	4950	11.340	47.060	10.910	1.00	0.00
ATOM	15974	HW2	SOL	4950	12.100	46.410	9.780	1.00	0.00
ATOM	15975	OW	SOL	4951	40.930	54.990	4.400	1.00	0.00
ATOM	15976	HW1	SOL	4951	40.430	55.810	4.460	1.00	0.00
ATOM	15977	HW2	SOL	4951	40.330	54.330	4.760	1.00	0.00
ATOM	15978	OW	SOL	4952	22.420	51.930	13.170	1.00	0.00
ATOM	15979	HW1	SOL	4952	23.310	52.210	12.970	1.00	0.00
ATOM	15980	HW2	SOL	4952	21.970	51.950	12.320	1.00	0.00
ATOM	15981	OW	SOL	4953	17.480	12.180	34.230	1.00	0.00
ATOM	15982	HW1	SOL	4953	17.580	11.910	35.140	1.00	0.00
ATOM	15983	HW2	SOL	4953	16.710	12.750	34.240	1.00	0.00
ATOM	15984	OW	SOL	4954	13.790	0.020	52.000	1.00	0.00
ATOM	15985	HW1	SOL	4954	13.910	-0.800	51.530	1.00	0.00
ATOM	15986	HW2	SOL	4954	12.890	-0.030	52.340	1.00	0.00
ATOM	15987	OW	SOL	4955	8.130	18.740	23.410	1.00	0.00
ATOM	15988	HW1	SOL	4955	8.240	18.940	24.340	1.00	0.00
ATOM	15989	HW2	SOL	4955	9.020	18.550	23.100	1.00	0.00
ATOM	15990	OW	SOL	4956	0.770	51.570	11.410	1.00	0.00
ATOM	15991	HW1	SOL	4956	0.340	50.720	11.320	1.00	0.00
ATOM	15992	HW2	SOL	4956	1.440	51.570	10.720	1.00	0.00
ATOM	15993	OW	SOL	4957	9.010	29.230	32.140	1.00	0.00
ATOM	15994	HW1	SOL	4957	8.800	29.580	31.270	1.00	0.00
ATOM	15995	HW2	SOL	4957	8.350	28.550	32.290	1.00	0.00
ATOM	15996	OW	SOL	4958	48.790	4.270	1.480	1.00	0.00
ATOM	15997	HW1	SOL	4958	49.660	4.010	1.180	1.00	0.00
ATOM	15998	HW2	SOL	4958	48.410	3.470	1.840	1.00	0.00
ATOM	15999	OW	SOL	4959	2.940	31.650	16.520	1.00	0.00
ATOM	16000	HW1	SOL	4959	3.180	32.560	16.380	1.00	0.00
ATOM	16001	HW2	SOL	4959	3.770	31.180	16.530	1.00	0.00
ATOM	16002	OW	SOL	4960	40.910	37.550	47.200	1.00	0.00
ATOM	16003	HW1	SOL	4960	40.090	37.310	46.760	1.00	0.00
ATOM	16004	HW2	SOL	4960	40.960	36.970	47.960	1.00	0.00
ATOM	16005	OW	SOL	4961	9.450	2.540	10.670	1.00	0.00
ATOM	16006	HW1	SOL	4961	8.940	3.210	11.130	1.00	0.00
ATOM	16007	HW2	SOL	4961	9.000	1.720	10.870	1.00	0.00
ATOM	16008	OW	SOL	4962	24.750	24.150	39.290	1.00	0.00
ATOM	16009	HW1	SOL	4962	24.420	24.890	38.790	1.00	0.00
ATOM	16010	HW2	SOL	4962	24.420	24.290	40.180	1.00	0.00
ATOM	16011	OW	SOL	4963	49.890	39.430	19.700	1.00	0.00
ATOM	16012	HW1	SOL	4963	50.520	39.500	18.980	1.00	0.00
ATOM	16013	HW2	SOL	4963	50.430	39.390	20.490	1.00	0.00
ATOM	16014	OW	SOL	4964	8.190	14.150	3.770	1.00	0.00
ATOM	16015	HW1	SOL	4964	8.240	13.410	4.380	1.00	0.00

ATOM	16016	HW2	SOL	4964	9.100	14.330	3.540	1.00	0.00
ATOM	16017	OW	SOL	4965	22.450	20.360	48.480	1.00	0.00
ATOM	16018	HW1	SOL	4965	22.740	20.860	47.710	1.00	0.00
ATOM	16019	HW2	SOL	4965	22.810	20.850	49.220	1.00	0.00
ATOM	16020	OW	SOL	4966	13.680	5.730	52.010	1.00	0.00
ATOM	16021	HW1	SOL	4966	14.090	4.880	51.830	1.00	0.00
ATOM	16022	HW2	SOL	4966	14.410	6.340	52.040	1.00	0.00
ATOM	16023	OW	SOL	4967	12.670	44.610	6.790	1.00	0.00
ATOM	16024	HW1	SOL	4967	13.220	43.820	6.790	1.00	0.00
ATOM	16025	HW2	SOL	4967	13.120	45.220	6.220	1.00	0.00
ATOM	16026	OW	SOL	4968	1.120	13.860	14.910	1.00	0.00
ATOM	16027	HW1	SOL	4968	1.310	13.620	15.820	1.00	0.00
ATOM	16028	HW2	SOL	4968	1.830	13.460	14.410	1.00	0.00
ATOM	16029	OW	SOL	4969	53.510	51.380	31.130	1.00	0.00
ATOM	16030	HW1	SOL	4969	54.230	51.240	31.750	1.00	0.00
ATOM	16031	HW2	SOL	4969	52.750	50.980	31.550	1.00	0.00
ATOM	16032	OW	SOL	4970	23.120	36.340	18.550	1.00	0.00
ATOM	16033	HW1	SOL	4970	22.680	37.000	18.010	1.00	0.00
ATOM	16034	HW2	SOL	4970	22.790	35.500	18.220	1.00	0.00
ATOM	16035	OW	SOL	4971	48.840	48.800	11.250	1.00	0.00
ATOM	16036	HW1	SOL	4971	48.510	49.340	10.530	1.00	0.00
ATOM	16037	HW2	SOL	4971	49.790	48.920	11.220	1.00	0.00
ATOM	16038	OW	SOL	4972	41.020	26.950	49.370	1.00	0.00
ATOM	16039	HW1	SOL	4972	41.810	26.560	48.990	1.00	0.00
ATOM	16040	HW2	SOL	4972	41.120	27.890	49.230	1.00	0.00
ATOM	16041	OW	SOL	4973	22.950	1.080	36.290	1.00	0.00
ATOM	16042	HW1	SOL	4973	22.550	0.490	35.660	1.00	0.00
ATOM	16043	HW2	SOL	4973	23.880	1.070	36.070	1.00	0.00
ATOM	16044	OW	SOL	4974	45.000	13.790	54.400	1.00	0.00
ATOM	16045	HW1	SOL	4974	44.470	13.670	55.190	1.00	0.00
ATOM	16046	HW2	SOL	4974	44.360	13.930	53.700	1.00	0.00
ATOM	16047	OW	SOL	4975	26.060	53.710	40.020	1.00	0.00
ATOM	16048	HW1	SOL	4975	25.920	53.540	39.090	1.00	0.00
ATOM	16049	HW2	SOL	4975	25.800	54.620	40.140	1.00	0.00
ATOM	16050	OW	SOL	4976	48.410	2.400	35.800	1.00	0.00
ATOM	16051	HW1	SOL	4976	47.820	2.050	36.470	1.00	0.00
ATOM	16052	HW2	SOL	4976	48.050	2.090	34.970	1.00	0.00
ATOM	16053	OW	SOL	4977	9.140	47.580	14.640	1.00	0.00
ATOM	16054	HW1	SOL	4977	10.010	47.280	14.920	1.00	0.00
ATOM	16055	HW2	SOL	4977	8.990	48.380	15.130	1.00	0.00
ATOM	16056	OW	SOL	4978	51.500	49.560	32.930	1.00	0.00
ATOM	16057	HW1	SOL	4978	51.040	49.150	32.200	1.00	0.00
ATOM	16058	HW2	SOL	4978	52.240	48.980	33.100	1.00	0.00
ATOM	16059	OW	SOL	4979	24.860	51.920	25.370	1.00	0.00

ATOM	16060	HW1	SOL	4979	24.620	51.110	25.830	1.00	0.00
ATOM	16061	HW2	SOL	4979	24.140	52.520	25.540	1.00	0.00
ATOM	16062	OW	SOL	4980	37.610	27.460	52.380	1.00	0.00
ATOM	16063	HW1	SOL	4980	37.850	26.550	52.230	1.00	0.00
ATOM	16064	HW2	SOL	4980	38.000	27.680	53.230	1.00	0.00
ATOM	16065	OW	SOL	4981	13.050	23.930	3.770	1.00	0.00
ATOM	16066	HW1	SOL	4981	12.150	23.970	3.450	1.00	0.00
ATOM	16067	HW2	SOL	4981	13.500	23.370	3.130	1.00	0.00
ATOM	16068	OW	SOL	4982	25.510	24.680	4.990	1.00	0.00
ATOM	16069	HW1	SOL	4982	24.620	24.330	4.980	1.00	0.00
ATOM	16070	HW2	SOL	4982	25.730	24.790	4.070	1.00	0.00
ATOM	16071	OW	SOL	4983	55.640	36.890	28.800	1.00	0.00
ATOM	16072	HW1	SOL	4983	55.510	36.090	29.320	1.00	0.00
ATOM	16073	HW2	SOL	4983	55.550	37.600	29.420	1.00	0.00
ATOM	16074	OW	SOL	4984	52.100	52.720	16.260	1.00	0.00
ATOM	16075	HW1	SOL	4984	52.730	52.080	15.920	1.00	0.00
ATOM	16076	HW2	SOL	4984	52.290	53.530	15.790	1.00	0.00
ATOM	16077	OW	SOL	4985	3.610	32.490	54.940	1.00	0.00
ATOM	16078	HW1	SOL	4985	4.020	31.650	54.750	1.00	0.00
ATOM	16079	HW2	SOL	4985	3.880	33.060	54.210	1.00	0.00
ATOM	16080	OW	SOL	4986	32.570	51.620	0.150	1.00	0.00
ATOM	16081	HW1	SOL	4986	32.200	51.160	-0.610	1.00	0.00
ATOM	16082	HW2	SOL	4986	32.680	52.520	-0.140	1.00	0.00
ATOM	16083	OW	SOL	4987	43.920	41.840	0.270	1.00	0.00
ATOM	16084	HW1	SOL	4987	44.710	41.320	0.090	1.00	0.00
ATOM	16085	HW2	SOL	4987	43.400	41.790	-0.530	1.00	0.00
ATOM	16086	OW	SOL	4988	22.430	5.240	26.960	1.00	0.00
ATOM	16087	HW1	SOL	4988	21.840	5.960	27.180	1.00	0.00
ATOM	16088	HW2	SOL	4988	22.120	4.500	27.470	1.00	0.00
ATOM	16089	OW	SOL	4989	16.040	44.610	35.270	1.00	0.00
ATOM	16090	HW1	SOL	4989	15.840	45.400	34.770	1.00	0.00
ATOM	16091	HW2	SOL	4989	15.950	44.880	36.190	1.00	0.00
ATOM	16092	OW	SOL	4990	34.100	32.120	53.960	1.00	0.00
ATOM	16093	HW1	SOL	4990	33.580	31.660	53.290	1.00	0.00
ATOM	16094	HW2	SOL	4990	35.010	31.980	53.690	1.00	0.00
ATOM	16095	OW	SOL	4991	47.420	10.690	35.230	1.00	0.00
ATOM	16096	HW1	SOL	4991	47.460	10.160	34.430	1.00	0.00
ATOM	16097	HW2	SOL	4991	48.070	11.380	35.100	1.00	0.00
ATOM	16098	OW	SOL	4992	27.490	36.700	20.790	1.00	0.00
ATOM	16099	HW1	SOL	4992	27.070	36.260	21.530	1.00	0.00
ATOM	16100	HW2	SOL	4992	27.180	37.600	20.840	1.00	0.00
ATOM	16101	OW	SOL	4993	21.900	4.530	24.190	1.00	0.00
ATOM	16102	HW1	SOL	4993	20.960	4.340	24.160	1.00	0.00
ATOM	16103	HW2	SOL	4993	22.070	4.770	25.100	1.00	0.00

ATOM	16104	OW	SOL	4994	21.050	46.660	39.190	1.00	0.00
ATOM	16105	HW1	SOL	4994	21.740	47.000	39.770	1.00	0.00
ATOM	16106	HW2	SOL	4994	21.020	45.720	39.380	1.00	0.00
ATOM	16107	OW	SOL	4995	28.310	31.520	5.450	1.00	0.00
ATOM	16108	HW1	SOL	4995	29.190	31.180	5.610	1.00	0.00
ATOM	16109	HW2	SOL	4995	27.730	30.890	5.880	1.00	0.00
ATOM	16110	OW	SOL	4996	33.330	18.680	6.280	1.00	0.00
ATOM	16111	HW1	SOL	4996	33.880	19.360	5.900	1.00	0.00
ATOM	16112	HW2	SOL	4996	32.650	18.530	5.630	1.00	0.00
ATOM	16113	OW	SOL	4997	26.810	24.360	44.350	1.00	0.00
ATOM	16114	HW1	SOL	4997	26.170	25.010	44.610	1.00	0.00
ATOM	16115	HW2	SOL	4997	27.300	24.160	45.150	1.00	0.00
ATOM	16116	OW	SOL	4998	20.340	32.870	27.590	1.00	0.00
ATOM	16117	HW1	SOL	4998	20.080	32.150	28.150	1.00	0.00
ATOM	16118	HW2	SOL	4998	21.060	32.520	27.060	1.00	0.00
ATOM	16119	OW	SOL	4999	52.820	26.570	54.370	1.00	0.00
ATOM	16120	HW1	SOL	4999	53.410	27.180	53.920	1.00	0.00
ATOM	16121	HW2	SOL	4999	53.370	25.800	54.550	1.00	0.00
ATOM	16122	OW	SOL	5000	52.140	48.540	15.940	1.00	0.00
ATOM	16123	HW1	SOL	5000	51.730	49.320	15.590	1.00	0.00
ATOM	16124	HW2	SOL	5000	51.420	48.020	16.300	1.00	0.00
ATOM	16125	OW	SOL	5001	16.570	55.750	4.920	1.00	0.00
ATOM	16126	HW1	SOL	5001	17.390	55.460	5.310	1.00	0.00
ATOM	16127	HW2	SOL	5001	16.690	55.640	3.970	1.00	0.00
ATOM	16128	OW	SOL	5002	17.150	41.150	34.250	1.00	0.00
ATOM	16129	HW1	SOL	5002	16.440	40.910	34.850	1.00	0.00
ATOM	16130	HW2	SOL	5002	17.920	40.690	34.590	1.00	0.00
ATOM	16131	OW	SOL	5003	7.940	40.690	21.170	1.00	0.00
ATOM	16132	HW1	SOL	5003	8.680	40.220	21.560	1.00	0.00
ATOM	16133	HW2	SOL	5003	7.660	40.130	20.450	1.00	0.00
ATOM	16134	OW	SOL	5004	10.610	18.440	48.650	1.00	0.00
ATOM	16135	HW1	SOL	5004	11.240	18.470	47.930	1.00	0.00
ATOM	16136	HW2	SOL	5004	9.820	18.050	48.270	1.00	0.00
ATOM	16137	OW	SOL	5005	10.590	34.220	33.850	1.00	0.00
ATOM	16138	HW1	SOL	5005	10.420	33.470	33.280	1.00	0.00
ATOM	16139	HW2	SOL	5005	10.230	34.970	33.370	1.00	0.00
ATOM	16140	OW	SOL	5006	29.620	15.020	25.360	1.00	0.00
ATOM	16141	HW1	SOL	5006	29.990	14.150	25.190	1.00	0.00
ATOM	16142	HW2	SOL	5006	28.940	14.860	26.020	1.00	0.00
ATOM	16143	OW	SOL	5007	46.430	34.070	17.490	1.00	0.00
ATOM	16144	HW1	SOL	5007	45.850	34.120	16.730	1.00	0.00
ATOM	16145	HW2	SOL	5007	45.870	33.760	18.200	1.00	0.00
ATOM	16146	OW	SOL	5008	40.070	2.160	7.530	1.00	0.00
ATOM	16147	HW1	SOL	5008	40.990	2.270	7.310	1.00	0.00

ATOM	16148	HW2	SOL	5008	39.910	2.800	8.230	1.00	0.00
ATOM	16149	OW	SOL	5009	21.370	51.810	50.540	1.00	0.00
ATOM	16150	HW1	SOL	5009	20.760	51.250	50.060	1.00	0.00
ATOM	16151	HW2	SOL	5009	21.020	52.690	50.430	1.00	0.00
ATOM	16152	OW	SOL	5010	0.570	39.540	12.330	1.00	0.00
ATOM	16153	HW1	SOL	5010	1.180	39.940	12.950	1.00	0.00
ATOM	16154	HW2	SOL	5010	0.960	38.700	12.110	1.00	0.00
ATOM	16155	OW	SOL	5011	46.460	42.050	7.370	1.00	0.00
ATOM	16156	HW1	SOL	5011	46.660	42.970	7.200	1.00	0.00
ATOM	16157	HW2	SOL	5011	46.960	41.570	6.710	1.00	0.00
ATOM	16158	OW	SOL	5012	33.950	28.200	3.970	1.00	0.00
ATOM	16159	HW1	SOL	5012	34.500	27.690	3.380	1.00	0.00
ATOM	16160	HW2	SOL	5012	34.160	27.860	4.850	1.00	0.00
ATOM	16161	OW	SOL	5013	49.180	17.320	12.590	1.00	0.00
ATOM	16162	HW1	SOL	5013	50.130	17.340	12.720	1.00	0.00
ATOM	16163	HW2	SOL	5013	48.830	17.910	13.260	1.00	0.00
ATOM	16164	OW	SOL	5014	54.440	40.390	1.630	1.00	0.00
ATOM	16165	HW1	SOL	5014	55.120	39.840	1.250	1.00	0.00
ATOM	16166	HW2	SOL	5014	53.620	40.020	1.290	1.00	0.00
ATOM	16167	OW	SOL	5015	50.490	50.540	15.190	1.00	0.00
ATOM	16168	HW1	SOL	5015	50.550	50.780	14.270	1.00	0.00
ATOM	16169	HW2	SOL	5015	50.720	51.340	15.660	1.00	0.00
ATOM	16170	OW	SOL	5016	0.340	16.940	24.220	1.00	0.00
ATOM	16171	HW1	SOL	5016	0.130	16.990	25.150	1.00	0.00
ATOM	16172	HW2	SOL	5016	1.290	16.830	24.200	1.00	0.00
ATOM	16173	OW	SOL	5017	6.770	14.300	47.600	1.00	0.00
ATOM	16174	HW1	SOL	5017	7.090	15.120	47.210	1.00	0.00
ATOM	16175	HW2	SOL	5017	5.950	14.120	47.130	1.00	0.00
ATOM	16176	OW	SOL	5018	39.970	42.410	50.590	1.00	0.00
ATOM	16177	HW1	SOL	5018	39.790	42.530	51.530	1.00	0.00
ATOM	16178	HW2	SOL	5018	40.360	43.240	50.320	1.00	0.00
ATOM	16179	OW	SOL	5019	14.820	25.900	19.510	1.00	0.00
ATOM	16180	HW1	SOL	5019	13.920	26.160	19.310	1.00	0.00
ATOM	16181	HW2	SOL	5019	15.190	26.640	20.000	1.00	0.00
ATOM	16182	OW	SOL	5020	3.580	31.150	9.070	1.00	0.00
ATOM	16183	HW1	SOL	5020	3.360	31.390	9.970	1.00	0.00
ATOM	16184	HW2	SOL	5020	4.530	31.210	9.040	1.00	0.00
ATOM	16185	OW	SOL	5021	23.320	2.270	6.690	1.00	0.00
ATOM	16186	HW1	SOL	5021	23.750	1.900	7.460	1.00	0.00
ATOM	16187	HW2	SOL	5021	23.080	3.150	6.950	1.00	0.00
ATOM	16188	OW	SOL	5022	7.240	13.040	55.080	1.00	0.00
ATOM	16189	HW1	SOL	5022	8.190	13.190	55.050	1.00	0.00
ATOM	16190	HW2	SOL	5022	7.030	13.080	56.020	1.00	0.00
ATOM	16191	OW	SOL	5023	31.670	18.900	31.430	1.00	0.00

ATOM	16192	HW1	SOL	5023	31.540	18.060	31.870	1.00	0.00
ATOM	16193	HW2	SOL	5023	31.030	18.900	30.720	1.00	0.00
ATOM	16194	OW	SOL	5024	53.600	5.370	49.070	1.00	0.00
ATOM	16195	HW1	SOL	5024	54.160	5.490	48.290	1.00	0.00
ATOM	16196	HW2	SOL	5024	54.210	5.140	49.770	1.00	0.00
ATOM	16197	OW	SOL	5025	35.480	15.600	54.250	1.00	0.00
ATOM	16198	HW1	SOL	5025	35.420	15.840	53.330	1.00	0.00
ATOM	16199	HW2	SOL	5025	36.390	15.330	54.370	1.00	0.00
ATOM	16200	OW	SOL	5026	46.810	50.430	2.840	1.00	0.00
ATOM	16201	HW1	SOL	5026	46.880	49.490	3.000	1.00	0.00
ATOM	16202	HW2	SOL	5026	47.180	50.550	1.960	1.00	0.00
ATOM	16203	OW	SOL	5027	12.030	5.560	16.520	1.00	0.00
ATOM	16204	HW1	SOL	5027	11.260	5.040	16.750	1.00	0.00
ATOM	16205	HW2	SOL	5027	11.850	6.420	16.900	1.00	0.00
ATOM	16206	OW	SOL	5028	19.070	38.390	47.420	1.00	0.00
ATOM	16207	HW1	SOL	5028	19.040	37.430	47.400	1.00	0.00
ATOM	16208	HW2	SOL	5028	18.690	38.620	48.270	1.00	0.00
ATOM	16209	OW	SOL	5029	11.110	5.200	8.670	1.00	0.00
ATOM	16210	HW1	SOL	5029	11.310	4.340	9.010	1.00	0.00
ATOM	16211	HW2	SOL	5029	10.780	5.690	9.420	1.00	0.00
ATOM	16212	OW	SOL	5030	53.520	44.120	14.680	1.00	0.00
ATOM	16213	HW1	SOL	5030	53.120	43.250	14.650	1.00	0.00
ATOM	16214	HW2	SOL	5030	52.870	44.700	14.280	1.00	0.00
ATOM	16215	OW	SOL	5031	5.930	46.290	17.010	1.00	0.00
ATOM	16216	HW1	SOL	5031	5.980	45.730	17.790	1.00	0.00
ATOM	16217	HW2	SOL	5031	5.200	46.880	17.180	1.00	0.00
ATOM	16218	OW	SOL	5032	26.280	34.840	50.510	1.00	0.00
ATOM	16219	HW1	SOL	5032	26.620	33.940	50.570	1.00	0.00
ATOM	16220	HW2	SOL	5032	26.210	35.130	51.420	1.00	0.00
ATOM	16221	OW	SOL	5033	28.420	17.790	43.930	1.00	0.00
ATOM	16222	HW1	SOL	5033	28.870	18.530	44.340	1.00	0.00
ATOM	16223	HW2	SOL	5033	27.610	18.160	43.590	1.00	0.00
ATOM	16224	OW	SOL	5034	52.330	35.010	7.540	1.00	0.00
ATOM	16225	HW1	SOL	5034	51.470	35.190	7.920	1.00	0.00
ATOM	16226	HW2	SOL	5034	52.720	34.360	8.120	1.00	0.00
ATOM	16227	OW	SOL	5035	43.210	51.480	16.180	1.00	0.00
ATOM	16228	HW1	SOL	5035	43.680	51.860	16.920	1.00	0.00
ATOM	16229	HW2	SOL	5035	43.790	51.640	15.420	1.00	0.00
ATOM	16230	OW	SOL	5036	11.790	28.550	36.890	1.00	0.00
ATOM	16231	HW1	SOL	5036	11.690	29.420	36.510	1.00	0.00
ATOM	16232	HW2	SOL	5036	11.360	27.960	36.270	1.00	0.00
ATOM	16233	OW	SOL	5037	16.210	37.990	4.920	1.00	0.00
ATOM	16234	HW1	SOL	5037	16.840	37.720	4.260	1.00	0.00
ATOM	16235	HW2	SOL	5037	15.820	37.170	5.230	1.00	0.00

ATOM	16236	OW	SOL	5038	12.230	20.450	8.580	1.00	0.00
ATOM	16237	HW1	SOL	5038	11.770	19.650	8.810	1.00	0.00
ATOM	16238	HW2	SOL	5038	11.540	21.110	8.500	1.00	0.00
ATOM	16239	OW	SOL	5039	35.140	9.630	26.510	1.00	0.00
ATOM	16240	HW1	SOL	5039	34.700	8.800	26.720	1.00	0.00
ATOM	16241	HW2	SOL	5039	36.000	9.360	26.180	1.00	0.00
ATOM	16242	OW	SOL	5040	21.800	39.310	28.610	1.00	0.00
ATOM	16243	HW1	SOL	5040	21.720	40.210	28.290	1.00	0.00
ATOM	16244	HW2	SOL	5040	22.500	39.350	29.260	1.00	0.00
ATOM	16245	OW	SOL	5041	7.590	19.960	34.930	1.00	0.00
ATOM	16246	HW1	SOL	5041	7.150	20.690	35.380	1.00	0.00
ATOM	16247	HW2	SOL	5041	8.520	20.170	34.980	1.00	0.00
ATOM	16248	OW	SOL	5042	17.510	6.260	11.450	1.00	0.00
ATOM	16249	HW1	SOL	5042	17.030	6.210	12.280	1.00	0.00
ATOM	16250	HW2	SOL	5042	18.260	6.820	11.640	1.00	0.00
ATOM	16251	OW	SOL	5043	37.330	45.000	26.930	1.00	0.00
ATOM	16252	HW1	SOL	5043	37.650	44.180	26.570	1.00	0.00
ATOM	16253	HW2	SOL	5043	36.420	44.820	27.180	1.00	0.00
ATOM	16254	OW	SOL	5044	52.630	42.450	29.030	1.00	0.00
ATOM	16255	HW1	SOL	5044	53.170	41.850	28.520	1.00	0.00
ATOM	16256	HW2	SOL	5044	51.770	42.410	28.620	1.00	0.00
ATOM	16257	OW	SOL	5045	15.930	36.290	41.940	1.00	0.00
ATOM	16258	HW1	SOL	5045	15.840	36.880	42.690	1.00	0.00
ATOM	16259	HW2	SOL	5045	15.460	36.730	41.230	1.00	0.00
ATOM	16260	OW	SOL	5046	54.750	40.930	51.790	1.00	0.00
ATOM	16261	HW1	SOL	5046	54.600	40.070	52.190	1.00	0.00
ATOM	16262	HW2	SOL	5046	55.430	40.770	51.130	1.00	0.00
ATOM	16263	OW	SOL	5047	11.760	30.750	51.490	1.00	0.00
ATOM	16264	HW1	SOL	5047	11.430	31.490	51.990	1.00	0.00
ATOM	16265	HW2	SOL	5047	11.770	31.060	50.580	1.00	0.00
ATOM	16266	OW	SOL	5048	28.220	29.550	11.260	1.00	0.00
ATOM	16267	HW1	SOL	5048	28.990	30.030	11.550	1.00	0.00
ATOM	16268	HW2	SOL	5048	27.480	30.120	11.460	1.00	0.00
ATOM	16269	OW	SOL	5049	5.460	34.530	9.140	1.00	0.00
ATOM	16270	HW1	SOL	5049	6.370	34.570	8.830	1.00	0.00
ATOM	16271	HW2	SOL	5049	4.950	34.870	8.400	1.00	0.00
ATOM	16272	OW	SOL	5050	53.390	16.570	2.490	1.00	0.00
ATOM	16273	HW1	SOL	5050	53.720	17.020	3.260	1.00	0.00
ATOM	16274	HW2	SOL	5050	52.440	16.680	2.530	1.00	0.00
ATOM	16275	OW	SOL	5051	21.930	55.250	4.930	1.00	0.00
ATOM	16276	HW1	SOL	5051	22.110	54.590	5.600	1.00	0.00
ATOM	16277	HW2	SOL	5051	20.980	55.360	4.950	1.00	0.00
ATOM	16278	OW	SOL	5052	34.040	34.670	13.940	1.00	0.00
ATOM	16279	HW1	SOL	5052	34.590	33.970	14.280	1.00	0.00

ATOM	16280	HW2	SOL	5052	33.140	34.360	14.100	1.00	0.00
ATOM	16281	OW	SOL	5053	16.720	49.620	53.410	1.00	0.00
ATOM	16282	HW1	SOL	5053	17.600	50.000	53.350	1.00	0.00
ATOM	16283	HW2	SOL	5053	16.580	49.200	52.560	1.00	0.00
ATOM	16284	OW	SOL	5054	30.520	33.980	52.380	1.00	0.00
ATOM	16285	HW1	SOL	5054	31.330	34.340	52.750	1.00	0.00
ATOM	16286	HW2	SOL	5054	30.110	34.710	51.930	1.00	0.00
ATOM	16287	OW	SOL	5055	55.540	22.350	46.060	1.00	0.00
ATOM	16288	HW1	SOL	5055	56.400	22.780	46.050	1.00	0.00
ATOM	16289	HW2	SOL	5055	55.340	22.260	46.990	1.00	0.00
ATOM	16290	OW	SOL	5056	16.040	32.650	8.180	1.00	0.00
ATOM	16291	HW1	SOL	5056	15.130	32.430	8.350	1.00	0.00
ATOM	16292	HW2	SOL	5056	16.530	32.200	8.870	1.00	0.00
ATOM	16293	OW	SOL	5057	30.960	5.910	2.450	1.00	0.00
ATOM	16294	HW1	SOL	5057	30.760	6.640	3.030	1.00	0.00
ATOM	16295	HW2	SOL	5057	31.720	6.220	1.940	1.00	0.00
ATOM	16296	OW	SOL	5058	8.100	14.520	9.940	1.00	0.00
ATOM	16297	HW1	SOL	5058	8.500	13.960	10.590	1.00	0.00
ATOM	16298	HW2	SOL	5058	8.740	14.570	9.230	1.00	0.00
ATOM	16299	OW	SOL	5059	1.010	36.260	26.630	1.00	0.00
ATOM	16300	HW1	SOL	5059	1.700	36.920	26.620	1.00	0.00
ATOM	16301	HW2	SOL	5059	0.540	36.410	27.450	1.00	0.00
ATOM	16302	OW	SOL	5060	14.880	12.130	8.800	1.00	0.00
ATOM	16303	HW1	SOL	5060	15.790	12.370	8.620	1.00	0.00
ATOM	16304	HW2	SOL	5060	14.570	11.760	7.970	1.00	0.00
ATOM	16305	OW	SOL	5061	12.820	11.010	47.070	1.00	0.00
ATOM	16306	HW1	SOL	5061	13.500	11.130	47.740	1.00	0.00
ATOM	16307	HW2	SOL	5061	12.640	11.900	46.760	1.00	0.00
ATOM	16308	OW	SOL	5062	23.990	4.040	30.760	1.00	0.00
ATOM	16309	HW1	SOL	5062	23.800	4.970	30.910	1.00	0.00
ATOM	16310	HW2	SOL	5062	24.820	4.040	30.280	1.00	0.00
ATOM	16311	OW	SOL	5063	36.490	3.020	47.090	1.00	0.00
ATOM	16312	HW1	SOL	5063	36.710	2.240	47.600	1.00	0.00
ATOM	16313	HW2	SOL	5063	37.280	3.550	47.110	1.00	0.00
ATOM	16314	OW	SOL	5064	24.020	15.190	10.070	1.00	0.00
ATOM	16315	HW1	SOL	5064	24.460	15.900	9.600	1.00	0.00
ATOM	16316	HW2	SOL	5064	24.700	14.520	10.160	1.00	0.00
ATOM	16317	OW	SOL	5065	21.330	3.250	29.020	1.00	0.00
ATOM	16318	HW1	SOL	5065	21.500	2.320	28.920	1.00	0.00
ATOM	16319	HW2	SOL	5065	21.840	3.510	29.790	1.00	0.00
ATOM	16320	OW	SOL	5066	38.390	48.850	26.370	1.00	0.00
ATOM	16321	HW1	SOL	5066	38.900	49.650	26.190	1.00	0.00
ATOM	16322	HW2	SOL	5066	38.070	48.970	27.260	1.00	0.00
ATOM	16323	OW	SOL	5067	37.650	22.090	0.270	1.00	0.00

ATOM	16324	HW1	SOL	5067	36.920	21.980	-0.340	1.00	0.00
ATOM	16325	HW2	SOL	5067	37.930	21.190	0.460	1.00	0.00
ATOM	16326	OW	SOL	5068	0.610	23.040	53.510	1.00	0.00
ATOM	16327	HW1	SOL	5068	1.230	22.460	53.060	1.00	0.00
ATOM	16328	HW2	SOL	5068	-0.030	22.440	53.890	1.00	0.00
ATOM	16329	OW	SOL	5069	21.560	10.590	2.790	1.00	0.00
ATOM	16330	HW1	SOL	5069	22.280	9.980	2.610	1.00	0.00
ATOM	16331	HW2	SOL	5069	21.850	11.410	2.400	1.00	0.00
ATOM	16332	OW	SOL	5070	8.610	43.640	46.140	1.00	0.00
ATOM	16333	HW1	SOL	5070	8.520	43.060	46.900	1.00	0.00
ATOM	16334	HW2	SOL	5070	9.190	44.340	46.440	1.00	0.00
ATOM	16335	OW	SOL	5071	7.710	41.440	12.970	1.00	0.00
ATOM	16336	HW1	SOL	5071	7.700	41.220	13.900	1.00	0.00
ATOM	16337	HW2	SOL	5071	7.390	40.660	12.520	1.00	0.00
ATOM	16338	OW	SOL	5072	37.870	55.870	21.350	1.00	0.00
ATOM	16339	HW1	SOL	5072	38.720	56.190	21.670	1.00	0.00
ATOM	16340	HW2	SOL	5072	37.610	56.500	20.680	1.00	0.00
ATOM	16341	OW	SOL	5073	55.740	4.230	24.480	1.00	0.00
ATOM	16342	HW1	SOL	5073	55.190	4.560	23.770	1.00	0.00
ATOM	16343	HW2	SOL	5073	55.390	4.630	25.270	1.00	0.00
ATOM	16344	OW	SOL	5074	29.890	19.010	37.150	1.00	0.00
ATOM	16345	HW1	SOL	5074	30.690	19.100	36.640	1.00	0.00
ATOM	16346	HW2	SOL	5074	29.260	19.580	36.710	1.00	0.00
ATOM	16347	OW	SOL	5075	13.360	14.140	14.600	1.00	0.00
ATOM	16348	HW1	SOL	5075	12.570	13.850	15.050	1.00	0.00
ATOM	16349	HW2	SOL	5075	14.030	13.510	14.870	1.00	0.00
ATOM	16350	OW	SOL	5076	32.390	16.370	33.350	1.00	0.00
ATOM	16351	HW1	SOL	5076	32.000	16.190	34.200	1.00	0.00
ATOM	16352	HW2	SOL	5076	33.210	16.810	33.550	1.00	0.00
ATOM	16353	OW	SOL	5077	49.620	22.820	18.290	1.00	0.00
ATOM	16354	HW1	SOL	5077	49.930	21.940	18.110	1.00	0.00
ATOM	16355	HW2	SOL	5077	48.920	22.700	18.930	1.00	0.00
ATOM	16356	OW	SOL	5078	34.540	46.400	2.780	1.00	0.00
ATOM	16357	HW1	SOL	5078	34.480	47.220	2.300	1.00	0.00
ATOM	16358	HW2	SOL	5078	34.110	45.760	2.220	1.00	0.00
ATOM	16359	OW	SOL	5079	55.320	35.270	50.270	1.00	0.00
ATOM	16360	HW1	SOL	5079	55.140	35.320	49.330	1.00	0.00
ATOM	16361	HW2	SOL	5079	54.860	34.480	50.560	1.00	0.00
ATOM	16362	OW	SOL	5080	12.430	23.400	6.750	1.00	0.00
ATOM	16363	HW1	SOL	5080	11.990	23.460	5.900	1.00	0.00
ATOM	16364	HW2	SOL	5080	13.360	23.530	6.550	1.00	0.00
ATOM	16365	OW	SOL	5081	41.770	10.460	32.030	1.00	0.00
ATOM	16366	HW1	SOL	5081	42.660	10.270	32.330	1.00	0.00
ATOM	16367	HW2	SOL	5081	41.780	10.250	31.090	1.00	0.00

ATOM	16368	OW	SOL	5082	25.440	38.840	52.020	1.00	0.00
ATOM	16369	HW1	SOL	5082	24.620	38.570	51.610	1.00	0.00
ATOM	16370	HW2	SOL	5082	25.890	38.020	52.220	1.00	0.00
ATOM	16371	OW	SOL	5083	0.230	11.040	3.820	1.00	0.00
ATOM	16372	HW1	SOL	5083	-0.510	10.460	4.020	1.00	0.00
ATOM	16373	HW2	SOL	5083	0.100	11.280	2.900	1.00	0.00
ATOM	16374	OW	SOL	5084	10.630	34.110	21.940	1.00	0.00
ATOM	16375	HW1	SOL	5084	11.140	34.120	22.750	1.00	0.00
ATOM	16376	HW2	SOL	5084	10.790	34.960	21.530	1.00	0.00
ATOM	16377	OW	SOL	5085	29.960	19.920	44.870	1.00	0.00
ATOM	16378	HW1	SOL	5085	30.790	19.460	44.910	1.00	0.00
ATOM	16379	HW2	SOL	5085	30.190	20.820	44.660	1.00	0.00
ATOM	16380	OW	SOL	5086	25.720	27.460	37.860	1.00	0.00
ATOM	16381	HW1	SOL	5086	25.880	28.320	38.230	1.00	0.00
ATOM	16382	HW2	SOL	5086	26.350	26.880	38.290	1.00	0.00
ATOM	16383	OW	SOL	5087	5.570	6.110	14.910	1.00	0.00
ATOM	16384	HW1	SOL	5087	5.760	6.490	15.770	1.00	0.00
ATOM	16385	HW2	SOL	5087	6.430	5.890	14.550	1.00	0.00
ATOM	16386	OW	SOL	5088	28.280	3.070	4.010	1.00	0.00
ATOM	16387	HW1	SOL	5088	29.090	3.510	4.280	1.00	0.00
ATOM	16388	HW2	SOL	5088	27.630	3.350	4.650	1.00	0.00
ATOM	16389	OW	SOL	5089	11.330	27.990	50.660	1.00	0.00
ATOM	16390	HW1	SOL	5089	11.730	28.470	51.390	1.00	0.00
ATOM	16391	HW2	SOL	5089	11.620	27.090	50.770	1.00	0.00
ATOM	16392	OW	SOL	5090	37.240	35.710	12.750	1.00	0.00
ATOM	16393	HW1	SOL	5090	38.150	35.890	12.980	1.00	0.00
ATOM	16394	HW2	SOL	5090	36.850	36.570	12.630	1.00	0.00
ATOM	16395	OW	SOL	5091	44.540	3.570	44.560	1.00	0.00
ATOM	16396	HW1	SOL	5091	43.700	3.540	45.010	1.00	0.00
ATOM	16397	HW2	SOL	5091	45.050	4.230	45.030	1.00	0.00
ATOM	16398	OW	SOL	5092	37.650	37.330	20.060	1.00	0.00
ATOM	16399	HW1	SOL	5092	37.040	38.040	20.240	1.00	0.00
ATOM	16400	HW2	SOL	5092	37.240	36.830	19.350	1.00	0.00
ATOM	16401	OW	SOL	5093	48.720	51.720	48.600	1.00	0.00
ATOM	16402	HW1	SOL	5093	48.190	51.590	47.820	1.00	0.00
ATOM	16403	HW2	SOL	5093	49.190	50.880	48.710	1.00	0.00
ATOM	16404	OW	SOL	5094	47.360	22.960	30.180	1.00	0.00
ATOM	16405	HW1	SOL	5094	47.030	22.200	30.650	1.00	0.00
ATOM	16406	HW2	SOL	5094	47.170	23.700	30.760	1.00	0.00
ATOM	16407	OW	SOL	5095	53.560	2.890	13.870	1.00	0.00
ATOM	16408	HW1	SOL	5095	54.400	2.500	14.120	1.00	0.00
ATOM	16409	HW2	SOL	5095	53.620	3.000	12.920	1.00	0.00
ATOM	16410	OW	SOL	5096	37.680	7.200	38.150	1.00	0.00
ATOM	16411	HW1	SOL	5096	38.040	7.640	38.920	1.00	0.00

ATOM	16412	HW2	SOL	5096	38.060	6.320	38.190	1.00	0.00
ATOM	16413	OW	SOL	5097	13.140	13.040	28.210	1.00	0.00
ATOM	16414	HW1	SOL	5097	13.780	12.420	27.860	1.00	0.00
ATOM	16415	HW2	SOL	5097	12.300	12.750	27.850	1.00	0.00
ATOM	16416	OW	SOL	5098	24.380	17.530	44.540	1.00	0.00
ATOM	16417	HW1	SOL	5098	23.660	17.470	45.170	1.00	0.00
ATOM	16418	HW2	SOL	5098	24.750	18.400	44.680	1.00	0.00
ATOM	16419	OW	SOL	5099	14.790	1.270	37.730	1.00	0.00
ATOM	16420	HW1	SOL	5099	14.160	1.840	38.160	1.00	0.00
ATOM	16421	HW2	SOL	5099	14.870	1.610	36.840	1.00	0.00
ATOM	16422	OW	SOL	5100	40.600	8.100	55.870	1.00	0.00
ATOM	16423	HW1	SOL	5100	40.610	8.650	55.090	1.00	0.00
ATOM	16424	HW2	SOL	5100	41.520	8.000	56.120	1.00	0.00
ATOM	16425	OW	SOL	5101	32.670	26.100	40.770	1.00	0.00
ATOM	16426	HW1	SOL	5101	33.550	25.980	40.420	1.00	0.00
ATOM	16427	HW2	SOL	5101	32.770	25.980	41.710	1.00	0.00
ATOM	16428	OW	SOL	5102	13.190	31.320	12.160	1.00	0.00
ATOM	16429	HW1	SOL	5102	13.080	30.560	12.720	1.00	0.00
ATOM	16430	HW2	SOL	5102	13.220	32.060	12.770	1.00	0.00
ATOM	16431	OW	SOL	5103	41.730	32.290	52.980	1.00	0.00
ATOM	16432	HW1	SOL	5103	41.470	33.190	53.150	1.00	0.00
ATOM	16433	HW2	SOL	5103	40.910	31.790	52.980	1.00	0.00
ATOM	16434	OW	SOL	5104	4.910	17.430	47.980	1.00	0.00
ATOM	16435	HW1	SOL	5104	4.160	16.840	48.010	1.00	0.00
ATOM	16436	HW2	SOL	5104	5.200	17.410	47.070	1.00	0.00
ATOM	16437	OW	SOL	5105	23.590	25.990	37.770	1.00	0.00
ATOM	16438	HW1	SOL	5105	24.320	26.600	37.740	1.00	0.00
ATOM	16439	HW2	SOL	5105	23.680	25.460	36.970	1.00	0.00
ATOM	16440	OW	SOL	5106	17.920	40.730	12.600	1.00	0.00
ATOM	16441	HW1	SOL	5106	17.540	41.160	11.840	1.00	0.00
ATOM	16442	HW2	SOL	5106	17.170	40.330	13.050	1.00	0.00
ATOM	16443	OW	SOL	5107	30.140	5.410	10.430	1.00	0.00
ATOM	16444	HW1	SOL	5107	29.700	4.560	10.450	1.00	0.00
ATOM	16445	HW2	SOL	5107	31.070	5.190	10.390	1.00	0.00
ATOM	16446	OW	SOL	5108	17.370	0.450	39.040	1.00	0.00
ATOM	16447	HW1	SOL	5108	17.930	1.220	38.890	1.00	0.00
ATOM	16448	HW2	SOL	5108	16.840	0.390	38.240	1.00	0.00
ATOM	16449	OW	SOL	5109	36.600	48.340	6.180	1.00	0.00
ATOM	16450	HW1	SOL	5109	36.230	48.730	6.960	1.00	0.00
ATOM	16451	HW2	SOL	5109	36.980	49.070	5.690	1.00	0.00
ATOM	16452	OW	SOL	5110	53.790	19.910	3.000	1.00	0.00
ATOM	16453	HW1	SOL	5110	53.120	20.530	2.710	1.00	0.00
ATOM	16454	HW2	SOL	5110	54.620	20.320	2.740	1.00	0.00
ATOM	16455	OW	SOL	5111	51.380	13.770	2.900	1.00	0.00

ATOM	16456	HW1	SOL	5111	51.110	14.680	2.790	1.00	0.00
ATOM	16457	HW2	SOL	5111	52.150	13.670	2.330	1.00	0.00
ATOM	16458	OW	SOL	5112	17.920	13.290	5.750	1.00	0.00
ATOM	16459	HW1	SOL	5112	17.990	13.040	6.670	1.00	0.00
ATOM	16460	HW2	SOL	5112	17.460	12.560	5.330	1.00	0.00
ATOM	16461	OW	SOL	5113	26.250	51.060	47.810	1.00	0.00
ATOM	16462	HW1	SOL	5113	26.440	51.920	47.440	1.00	0.00
ATOM	16463	HW2	SOL	5113	26.490	51.140	48.730	1.00	0.00
ATOM	16464	OW	SOL	5114	33.340	0.200	39.130	1.00	0.00
ATOM	16465	HW1	SOL	5114	32.970	-0.210	38.350	1.00	0.00
ATOM	16466	HW2	SOL	5114	32.690	0.030	39.820	1.00	0.00
ATOM	16467	OW	SOL	5115	6.060	47.140	36.100	1.00	0.00
ATOM	16468	HW1	SOL	5115	6.380	46.960	35.220	1.00	0.00
ATOM	16469	HW2	SOL	5115	5.290	47.690	35.960	1.00	0.00
ATOM	16470	OW	SOL	5116	22.980	6.440	36.520	1.00	0.00
ATOM	16471	HW1	SOL	5116	23.090	6.130	37.420	1.00	0.00
ATOM	16472	HW2	SOL	5116	23.580	5.900	36.010	1.00	0.00
ATOM	16473	OW	SOL	5117	34.780	18.410	27.190	1.00	0.00
ATOM	16474	HW1	SOL	5117	34.920	18.680	28.090	1.00	0.00
ATOM	16475	HW2	SOL	5117	35.030	19.180	26.670	1.00	0.00
ATOM	16476	OW	SOL	5118	36.220	44.780	55.600	1.00	0.00
ATOM	16477	HW1	SOL	5118	35.830	45.050	54.770	1.00	0.00
ATOM	16478	HW2	SOL	5118	36.420	43.850	55.480	1.00	0.00
ATOM	16479	OW	SOL	5119	45.100	26.540	18.580	1.00	0.00
ATOM	16480	HW1	SOL	5119	44.890	25.790	18.030	1.00	0.00
ATOM	16481	HW2	SOL	5119	44.500	26.460	19.330	1.00	0.00
ATOM	16482	OW	SOL	5120	23.480	6.780	31.290	1.00	0.00
ATOM	16483	HW1	SOL	5120	23.670	7.480	31.910	1.00	0.00
ATOM	16484	HW2	SOL	5120	22.530	6.840	31.150	1.00	0.00
ATOM	16485	OW	SOL	5121	14.250	41.490	24.780	1.00	0.00
ATOM	16486	HW1	SOL	5121	14.580	41.710	25.650	1.00	0.00
ATOM	16487	HW2	SOL	5121	13.850	42.300	24.460	1.00	0.00
ATOM	16488	OW	SOL	5122	50.590	17.400	23.030	1.00	0.00
ATOM	16489	HW1	SOL	5122	51.170	17.640	23.760	1.00	0.00
ATOM	16490	HW2	SOL	5122	50.510	16.450	23.100	1.00	0.00
ATOM	16491	OW	SOL	5123	50.610	45.200	45.340	1.00	0.00
ATOM	16492	HW1	SOL	5123	49.940	45.850	45.130	1.00	0.00
ATOM	16493	HW2	SOL	5123	51.380	45.720	45.580	1.00	0.00
ATOM	16494	OW	SOL	5124	14.360	51.810	24.900	1.00	0.00
ATOM	16495	HW1	SOL	5124	13.440	51.590	24.770	1.00	0.00
ATOM	16496	HW2	SOL	5124	14.420	52.060	25.820	1.00	0.00
ATOM	16497	OW	SOL	5125	8.730	16.780	27.560	1.00	0.00
ATOM	16498	HW1	SOL	5125	7.850	17.090	27.340	1.00	0.00
ATOM	16499	HW2	SOL	5125	8.690	16.590	28.500	1.00	0.00

ATOM	16500	OW	SOL	5126	32.660	25.750	15.330	1.00	0.00
ATOM	16501	HW1	SOL	5126	33.370	26.350	15.560	1.00	0.00
ATOM	16502	HW2	SOL	5126	31.860	26.260	15.450	1.00	0.00
ATOM	16503	OW	SOL	5127	47.100	23.910	0.700	1.00	0.00
ATOM	16504	HW1	SOL	5127	46.910	22.990	0.550	1.00	0.00
ATOM	16505	HW2	SOL	5127	46.250	24.320	0.820	1.00	0.00
ATOM	16506	OW	SOL	5128	41.860	9.560	21.140	1.00	0.00
ATOM	16507	HW1	SOL	5128	40.970	9.220	21.120	1.00	0.00
ATOM	16508	HW2	SOL	5128	42.330	9.060	20.470	1.00	0.00
ATOM	16509	OW	SOL	5129	34.180	55.220	49.300	1.00	0.00
ATOM	16510	HW1	SOL	5129	33.580	55.740	49.840	1.00	0.00
ATOM	16511	HW2	SOL	5129	34.630	55.860	48.740	1.00	0.00
ATOM	16512	OW	SOL	5130	44.470	22.870	53.360	1.00	0.00
ATOM	16513	HW1	SOL	5130	43.920	23.480	52.880	1.00	0.00
ATOM	16514	HW2	SOL	5130	43.910	22.540	54.060	1.00	0.00
ATOM	16515	OW	SOL	5131	22.160	2.210	10.530	1.00	0.00
ATOM	16516	HW1	SOL	5131	21.830	1.650	11.230	1.00	0.00
ATOM	16517	HW2	SOL	5131	21.630	1.990	9.770	1.00	0.00
ATOM	16518	OW	SOL	5132	17.490	44.760	20.590	1.00	0.00
ATOM	16519	HW1	SOL	5132	18.020	44.110	21.050	1.00	0.00
ATOM	16520	HW2	SOL	5132	17.710	45.590	21.020	1.00	0.00
ATOM	16521	OW	SOL	5133	54.820	22.580	12.600	1.00	0.00
ATOM	16522	HW1	SOL	5133	54.430	22.630	11.730	1.00	0.00
ATOM	16523	HW2	SOL	5133	55.620	22.060	12.470	1.00	0.00
ATOM	16524	OW	SOL	5134	54.870	4.210	18.530	1.00	0.00
ATOM	16525	HW1	SOL	5134	54.930	5.020	19.040	1.00	0.00
ATOM	16526	HW2	SOL	5134	54.810	4.490	17.620	1.00	0.00
ATOM	16527	OW	SOL	5135	45.040	9.860	23.030	1.00	0.00
ATOM	16528	HW1	SOL	5135	44.890	9.440	22.190	1.00	0.00
ATOM	16529	HW2	SOL	5135	44.770	10.770	22.900	1.00	0.00
ATOM	16530	OW	SOL	5136	37.030	27.370	49.560	1.00	0.00
ATOM	16531	HW1	SOL	5136	37.700	27.000	48.980	1.00	0.00
ATOM	16532	HW2	SOL	5136	37.350	27.190	50.440	1.00	0.00
ATOM	16533	OW	SOL	5137	34.190	6.870	34.540	1.00	0.00
ATOM	16534	HW1	SOL	5137	35.130	7.020	34.590	1.00	0.00
ATOM	16535	HW2	SOL	5137	33.850	7.610	34.030	1.00	0.00
ATOM	16536	OW	SOL	5138	36.090	20.150	4.210	1.00	0.00
ATOM	16537	HW1	SOL	5138	35.280	20.650	4.110	1.00	0.00
ATOM	16538	HW2	SOL	5138	36.460	20.130	3.320	1.00	0.00
ATOM	16539	OW	SOL	5139	42.070	44.540	26.960	1.00	0.00
ATOM	16540	HW1	SOL	5139	41.990	43.600	26.840	1.00	0.00
ATOM	16541	HW2	SOL	5139	41.240	44.800	27.360	1.00	0.00
ATOM	16542	OW	SOL	5140	24.500	3.410	26.580	1.00	0.00
ATOM	16543	HW1	SOL	5140	24.000	4.170	26.880	1.00	0.00

ATOM	16544	HW2	SOL	5140	25.130	3.760	25.950	1.00	0.00
ATOM	16545	OW	SOL	5141	39.660	14.460	29.570	1.00	0.00
ATOM	16546	HW1	SOL	5141	39.220	13.620	29.550	1.00	0.00
ATOM	16547	HW2	SOL	5141	38.960	15.110	29.620	1.00	0.00
ATOM	16548	OW	SOL	5142	3.600	43.560	21.290	1.00	0.00
ATOM	16549	HW1	SOL	5142	3.810	44.300	21.860	1.00	0.00
ATOM	16550	HW2	SOL	5142	4.410	43.050	21.250	1.00	0.00
ATOM	16551	OW	SOL	5143	48.260	5.010	26.060	1.00	0.00
ATOM	16552	HW1	SOL	5143	48.610	4.830	26.930	1.00	0.00
ATOM	16553	HW2	SOL	5143	48.360	4.180	25.590	1.00	0.00
ATOM	16554	OW	SOL	5144	24.930	15.560	3.120	1.00	0.00
ATOM	16555	HW1	SOL	5144	24.480	15.660	3.950	1.00	0.00
ATOM	16556	HW2	SOL	5144	25.260	14.660	3.120	1.00	0.00
ATOM	16557	OW	SOL	5145	42.860	7.580	24.570	1.00	0.00
ATOM	16558	HW1	SOL	5145	42.660	6.700	24.900	1.00	0.00
ATOM	16559	HW2	SOL	5145	43.540	7.430	23.900	1.00	0.00
ATOM	16560	OW	SOL	5146	15.850	42.940	39.290	1.00	0.00
ATOM	16561	HW1	SOL	5146	16.290	43.790	39.300	1.00	0.00
ATOM	16562	HW2	SOL	5146	15.530	42.820	40.190	1.00	0.00
ATOM	16563	OW	SOL	5147	8.120	27.500	1.230	1.00	0.00
ATOM	16564	HW1	SOL	5147	7.630	27.360	0.430	1.00	0.00
ATOM	16565	HW2	SOL	5147	7.600	28.130	1.730	1.00	0.00
ATOM	16566	OW	SOL	5148	42.890	29.580	22.150	1.00	0.00
ATOM	16567	HW1	SOL	5148	42.730	29.560	21.200	1.00	0.00
ATOM	16568	HW2	SOL	5148	42.090	29.950	22.520	1.00	0.00
ATOM	16569	OW	SOL	5149	20.470	33.330	35.680	1.00	0.00
ATOM	16570	HW1	SOL	5149	21.030	33.560	36.420	1.00	0.00
ATOM	16571	HW2	SOL	5149	20.440	34.130	35.150	1.00	0.00
ATOM	16572	OW	SOL	5150	15.450	24.020	52.990	1.00	0.00
ATOM	16573	HW1	SOL	5150	14.560	23.810	53.290	1.00	0.00
ATOM	16574	HW2	SOL	5150	15.550	24.950	53.170	1.00	0.00
ATOM	16575	OW	SOL	5151	13.710	7.830	34.960	1.00	0.00
ATOM	16576	HW1	SOL	5151	13.610	8.750	34.720	1.00	0.00
ATOM	16577	HW2	SOL	5151	13.590	7.350	34.140	1.00	0.00
ATOM	16578	OW	SOL	5152	15.990	28.440	18.110	1.00	0.00
ATOM	16579	HW1	SOL	5152	16.330	28.440	19.000	1.00	0.00
ATOM	16580	HW2	SOL	5152	15.100	28.780	18.190	1.00	0.00
ATOM	16581	OW	SOL	5153	24.700	40.540	48.780	1.00	0.00
ATOM	16582	HW1	SOL	5153	24.670	40.350	47.840	1.00	0.00
ATOM	16583	HW2	SOL	5153	25.540	40.190	49.060	1.00	0.00
ATOM	16584	OW	SOL	5154	12.170	26.740	19.070	1.00	0.00
ATOM	16585	HW1	SOL	5154	11.830	27.370	19.710	1.00	0.00
ATOM	16586	HW2	SOL	5154	12.010	25.890	19.470	1.00	0.00
ATOM	16587	OW	SOL	5155	47.490	46.620	31.780	1.00	0.00

ATOM	16588	HW1	SOL	5155	47.170	47.510	31.970	1.00	0.00
ATOM	16589	HW2	SOL	5155	46.690	46.090	31.740	1.00	0.00
ATOM	16590	OW	SOL	5156	17.160	20.900	1.190	1.00	0.00
ATOM	16591	HW1	SOL	5156	17.340	20.830	2.130	1.00	0.00
ATOM	16592	HW2	SOL	5156	16.710	20.080	0.970	1.00	0.00
ATOM	16593	OW	SOL	5157	40.490	53.980	28.640	1.00	0.00
ATOM	16594	HW1	SOL	5157	41.310	54.250	28.240	1.00	0.00
ATOM	16595	HW2	SOL	5157	40.740	53.260	29.230	1.00	0.00
ATOM	16596	OW	SOL	5158	54.560	17.840	11.390	1.00	0.00
ATOM	16597	HW1	SOL	5158	53.870	17.270	11.060	1.00	0.00
ATOM	16598	HW2	SOL	5158	54.110	18.660	11.590	1.00	0.00
ATOM	16599	OW	SOL	5159	33.050	25.790	43.780	1.00	0.00
ATOM	16600	HW1	SOL	5159	32.480	26.110	44.480	1.00	0.00
ATOM	16601	HW2	SOL	5159	33.930	25.800	44.170	1.00	0.00
ATOM	16602	OW	SOL	5160	37.070	3.080	16.880	1.00	0.00
ATOM	16603	HW1	SOL	5160	37.540	3.910	16.920	1.00	0.00
ATOM	16604	HW2	SOL	5160	37.610	2.530	16.310	1.00	0.00
ATOM	16605	OW	SOL	5161	30.660	7.870	31.940	1.00	0.00
ATOM	16606	HW1	SOL	5161	31.420	8.220	32.410	1.00	0.00
ATOM	16607	HW2	SOL	5161	30.860	8.000	31.020	1.00	0.00
ATOM	16608	OW	SOL	5162	40.460	14.150	4.330	1.00	0.00
ATOM	16609	HW1	SOL	5162	40.300	14.900	4.900	1.00	0.00
ATOM	16610	HW2	SOL	5162	41.410	14.160	4.170	1.00	0.00
ATOM	16611	OW	SOL	5163	51.060	6.770	19.880	1.00	0.00
ATOM	16612	HW1	SOL	5163	51.740	7.420	19.680	1.00	0.00
ATOM	16613	HW2	SOL	5163	50.900	6.860	20.820	1.00	0.00
ATOM	16614	OW	SOL	5164	20.770	40.730	7.400	1.00	0.00
ATOM	16615	HW1	SOL	5164	20.530	40.050	6.780	1.00	0.00
ATOM	16616	HW2	SOL	5164	20.020	41.320	7.420	1.00	0.00
ATOM	16617	OW	SOL	5165	4.470	33.490	40.380	1.00	0.00
ATOM	16618	HW1	SOL	5165	4.480	32.600	40.020	1.00	0.00
ATOM	16619	HW2	SOL	5165	3.620	33.840	40.120	1.00	0.00
ATOM	16620	OW	SOL	5166	29.660	40.800	53.590	1.00	0.00
ATOM	16621	HW1	SOL	5166	29.810	41.730	53.770	1.00	0.00
ATOM	16622	HW2	SOL	5166	28.770	40.760	53.260	1.00	0.00
ATOM	16623	OW	SOL	5167	12.190	4.120	43.820	1.00	0.00
ATOM	16624	HW1	SOL	5167	11.900	4.630	43.070	1.00	0.00
ATOM	16625	HW2	SOL	5167	13.150	4.120	43.750	1.00	0.00
ATOM	16626	OW	SOL	5168	7.010	45.800	14.450	1.00	0.00
ATOM	16627	HW1	SOL	5168	7.720	46.440	14.430	1.00	0.00
ATOM	16628	HW2	SOL	5168	6.660	45.850	15.340	1.00	0.00
ATOM	16629	OW	SOL	5169	16.190	47.670	18.160	1.00	0.00
ATOM	16630	HW1	SOL	5169	17.080	47.610	17.810	1.00	0.00
ATOM	16631	HW2	SOL	5169	16.110	48.580	18.440	1.00	0.00

ATOM	16632	OW	SOL	5170	8.200	1.590	46.450	1.00	0.00
ATOM	16633	HW1	SOL	5170	7.440	2.170	46.410	1.00	0.00
ATOM	16634	HW2	SOL	5170	8.840	1.990	45.860	1.00	0.00
ATOM	16635	OW	SOL	5171	4.620	36.560	38.360	1.00	0.00
ATOM	16636	HW1	SOL	5171	4.760	37.500	38.210	1.00	0.00
ATOM	16637	HW2	SOL	5171	4.930	36.410	39.250	1.00	0.00
ATOM	16638	OW	SOL	5172	40.070	25.300	18.510	1.00	0.00
ATOM	16639	HW1	SOL	5172	39.130	25.270	18.330	1.00	0.00
ATOM	16640	HW2	SOL	5172	40.140	25.830	19.310	1.00	0.00
ATOM	16641	OW	SOL	5173	29.950	36.900	19.580	1.00	0.00
ATOM	16642	HW1	SOL	5173	29.660	36.170	19.030	1.00	0.00
ATOM	16643	HW2	SOL	5173	29.170	37.440	19.700	1.00	0.00
ATOM	16644	OW	SOL	5174	49.320	47.910	44.780	1.00	0.00
ATOM	16645	HW1	SOL	5174	48.750	48.220	45.480	1.00	0.00
ATOM	16646	HW2	SOL	5174	49.760	48.700	44.460	1.00	0.00
ATOM	16647	OW	SOL	5175	9.240	27.900	28.210	1.00	0.00
ATOM	16648	HW1	SOL	5175	9.130	28.780	28.580	1.00	0.00
ATOM	16649	HW2	SOL	5175	9.860	28.020	27.490	1.00	0.00
ATOM	16650	OW	SOL	5176	41.500	47.770	25.550	1.00	0.00
ATOM	16651	HW1	SOL	5176	40.870	47.530	24.880	1.00	0.00
ATOM	16652	HW2	SOL	5176	41.190	47.330	26.340	1.00	0.00
ATOM	16653	OW	SOL	5177	29.760	36.850	44.720	1.00	0.00
ATOM	16654	HW1	SOL	5177	29.080	37.380	44.290	1.00	0.00
ATOM	16655	HW2	SOL	5177	29.390	36.620	45.570	1.00	0.00
ATOM	16656	OW	SOL	5178	32.570	52.110	41.820	1.00	0.00
ATOM	16657	HW1	SOL	5178	32.660	52.070	40.870	1.00	0.00
ATOM	16658	HW2	SOL	5178	32.900	51.270	42.130	1.00	0.00
ATOM	16659	OW	SOL	5179	45.170	50.740	24.400	1.00	0.00
ATOM	16660	HW1	SOL	5179	44.820	50.010	24.900	1.00	0.00
ATOM	16661	HW2	SOL	5179	44.680	50.720	23.570	1.00	0.00
ATOM	16662	OW	SOL	5180	29.890	22.860	17.990	1.00	0.00
ATOM	16663	HW1	SOL	5180	29.610	23.720	17.660	1.00	0.00
ATOM	16664	HW2	SOL	5180	30.580	22.590	17.380	1.00	0.00
ATOM	16665	OW	SOL	5181	30.760	24.270	8.610	1.00	0.00
ATOM	16666	HW1	SOL	5181	30.310	25.090	8.800	1.00	0.00
ATOM	16667	HW2	SOL	5181	30.430	23.650	9.260	1.00	0.00
ATOM	16668	OW	SOL	5182	0.860	15.410	18.340	1.00	0.00
ATOM	16669	HW1	SOL	5182	1.340	15.880	17.660	1.00	0.00
ATOM	16670	HW2	SOL	5182	0.570	16.100	18.940	1.00	0.00
ATOM	16671	OW	SOL	5183	21.760	44.800	15.160	1.00	0.00
ATOM	16672	HW1	SOL	5183	21.970	44.190	15.870	1.00	0.00
ATOM	16673	HW2	SOL	5183	21.360	45.550	15.600	1.00	0.00
ATOM	16674	OW	SOL	5184	50.550	51.480	12.740	1.00	0.00
ATOM	16675	HW1	SOL	5184	50.300	51.250	11.840	1.00	0.00

ATOM	16676	HW2	SOL	5184	49.970	52.200	12.970	1.00	0.00
ATOM	16677	OW	SOL	5185	48.530	53.680	3.540	1.00	0.00
ATOM	16678	HW1	SOL	5185	49.390	53.260	3.600	1.00	0.00
ATOM	16679	HW2	SOL	5185	47.920	53.030	3.880	1.00	0.00
ATOM	16680	OW	SOL	5186	7.480	24.240	4.410	1.00	0.00
ATOM	16681	HW1	SOL	5186	7.610	23.730	3.610	1.00	0.00
ATOM	16682	HW2	SOL	5186	7.780	23.660	5.120	1.00	0.00
ATOM	16683	OW	SOL	5187	34.920	21.250	11.050	1.00	0.00
ATOM	16684	HW1	SOL	5187	34.520	20.470	11.430	1.00	0.00
ATOM	16685	HW2	SOL	5187	34.930	21.070	10.110	1.00	0.00
ATOM	16686	OW	SOL	5188	48.810	12.980	51.630	1.00	0.00
ATOM	16687	HW1	SOL	5188	48.170	12.800	50.940	1.00	0.00
ATOM	16688	HW2	SOL	5188	49.560	13.370	51.170	1.00	0.00
ATOM	16689	OW	SOL	5189	20.370	9.300	17.550	1.00	0.00
ATOM	16690	HW1	SOL	5189	21.220	9.650	17.280	1.00	0.00
ATOM	16691	HW2	SOL	5189	19.750	10.000	17.360	1.00	0.00
ATOM	16692	OW	SOL	5190	47.730	7.720	8.910	1.00	0.00
ATOM	16693	HW1	SOL	5190	47.860	7.020	8.270	1.00	0.00
ATOM	16694	HW2	SOL	5190	47.250	7.310	9.620	1.00	0.00
ATOM	16695	OW	SOL	5191	19.670	26.410	16.190	1.00	0.00
ATOM	16696	HW1	SOL	5191	19.310	25.680	15.700	1.00	0.00
ATOM	16697	HW2	SOL	5191	19.010	27.100	16.100	1.00	0.00
ATOM	16698	OW	SOL	5192	33.990	37.180	4.520	1.00	0.00
ATOM	16699	HW1	SOL	5192	33.110	36.880	4.290	1.00	0.00
ATOM	16700	HW2	SOL	5192	34.570	36.730	3.910	1.00	0.00
ATOM	16701	OW	SOL	5193	36.870	26.100	15.550	1.00	0.00
ATOM	16702	HW1	SOL	5193	37.410	26.220	16.340	1.00	0.00
ATOM	16703	HW2	SOL	5193	36.320	25.340	15.750	1.00	0.00
ATOM	16704	OW	SOL	5194	55.850	27.840	18.940	1.00	0.00
ATOM	16705	HW1	SOL	5194	56.180	28.740	18.870	1.00	0.00
ATOM	16706	HW2	SOL	5194	55.140	27.790	18.300	1.00	0.00
ATOM	16707	OW	SOL	5195	18.450	20.120	48.330	1.00	0.00
ATOM	16708	HW1	SOL	5195	19.000	20.840	48.650	1.00	0.00
ATOM	16709	HW2	SOL	5195	18.740	19.370	48.840	1.00	0.00
ATOM	16710	OW	SOL	5196	19.230	53.040	25.240	1.00	0.00
ATOM	16711	HW1	SOL	5196	19.090	53.960	25.010	1.00	0.00
ATOM	16712	HW2	SOL	5196	19.000	52.560	24.440	1.00	0.00
ATOM	16713	OW	SOL	5197	14.880	10.770	20.500	1.00	0.00
ATOM	16714	HW1	SOL	5197	14.310	10.110	20.900	1.00	0.00
ATOM	16715	HW2	SOL	5197	14.790	11.540	21.060	1.00	0.00
ATOM	16716	OW	SOL	5198	15.190	41.280	20.390	1.00	0.00
ATOM	16717	HW1	SOL	5198	15.500	40.760	21.130	1.00	0.00
ATOM	16718	HW2	SOL	5198	14.260	41.420	20.560	1.00	0.00
ATOM	16719	OW	SOL	5199	6.860	4.680	0.670	1.00	0.00

ATOM	16720	HW1	SOL	5199	6.460	5.450	1.060	1.00	0.00
ATOM	16721	HW2	SOL	5199	6.130	4.180	0.310	1.00	0.00
ATOM	16722	OW	SOL	5200	21.980	23.570	29.970	1.00	0.00
ATOM	16723	HW1	SOL	5200	22.020	23.750	30.910	1.00	0.00
ATOM	16724	HW2	SOL	5200	21.970	22.620	29.910	1.00	0.00
ATOM	16725	OW	SOL	5201	6.270	25.590	44.530	1.00	0.00
ATOM	16726	HW1	SOL	5201	5.760	24.810	44.720	1.00	0.00
ATOM	16727	HW2	SOL	5201	6.140	26.160	45.290	1.00	0.00
ATOM	16728	OW	SOL	5202	51.240	17.500	55.010	1.00	0.00
ATOM	16729	HW1	SOL	5202	50.770	16.850	55.540	1.00	0.00
ATOM	16730	HW2	SOL	5202	51.180	17.160	54.120	1.00	0.00
ATOM	16731	OW	SOL	5203	51.660	30.790	49.400	1.00	0.00
ATOM	16732	HW1	SOL	5203	50.920	30.570	49.960	1.00	0.00
ATOM	16733	HW2	SOL	5203	52.360	30.190	49.670	1.00	0.00
ATOM	16734	OW	SOL	5204	51.580	3.240	4.060	1.00	0.00
ATOM	16735	HW1	SOL	5204	51.210	4.110	3.930	1.00	0.00
ATOM	16736	HW2	SOL	5204	52.320	3.190	3.450	1.00	0.00
ATOM	16737	OW	SOL	5205	47.860	48.560	51.100	1.00	0.00
ATOM	16738	HW1	SOL	5205	48.020	47.640	50.920	1.00	0.00
ATOM	16739	HW2	SOL	5205	47.530	48.920	50.270	1.00	0.00
ATOM	16740	OW	SOL	5206	20.470	42.230	23.230	1.00	0.00
ATOM	16741	HW1	SOL	5206	20.820	42.970	22.740	1.00	0.00
ATOM	16742	HW2	SOL	5206	21.120	41.530	23.110	1.00	0.00
ATOM	16743	OW	SOL	5207	9.950	42.940	4.100	1.00	0.00
ATOM	16744	HW1	SOL	5207	10.320	43.420	3.350	1.00	0.00
ATOM	16745	HW2	SOL	5207	9.930	43.590	4.800	1.00	0.00
ATOM	16746	OW	SOL	5208	54.520	35.890	47.730	1.00	0.00
ATOM	16747	HW1	SOL	5208	53.640	36.170	47.490	1.00	0.00
ATOM	16748	HW2	SOL	5208	55.100	36.510	47.280	1.00	0.00
ATOM	16749	OW	SOL	5209	38.190	43.840	19.540	1.00	0.00
ATOM	16750	HW1	SOL	5209	39.140	43.890	19.420	1.00	0.00
ATOM	16751	HW2	SOL	5209	38.070	43.200	20.240	1.00	0.00
ATOM	16752	OW	SOL	5210	27.950	53.710	47.620	1.00	0.00
ATOM	16753	HW1	SOL	5210	27.780	53.750	46.680	1.00	0.00
ATOM	16754	HW2	SOL	5210	28.880	53.530	47.690	1.00	0.00
ATOM	16755	OW	SOL	5211	24.650	52.530	9.980	1.00	0.00
ATOM	16756	HW1	SOL	5211	25.230	51.780	9.820	1.00	0.00
ATOM	16757	HW2	SOL	5211	24.610	52.590	10.940	1.00	0.00
ATOM	16758	OW	SOL	5212	6.100	10.020	15.550	1.00	0.00
ATOM	16759	HW1	SOL	5212	5.160	9.830	15.490	1.00	0.00
ATOM	16760	HW2	SOL	5212	6.240	10.760	14.950	1.00	0.00
ATOM	16761	OW	SOL	5213	32.940	11.460	47.620	1.00	0.00
ATOM	16762	HW1	SOL	5213	33.220	10.760	47.030	1.00	0.00
ATOM	16763	HW2	SOL	5213	32.820	11.030	48.470	1.00	0.00

ATOM	16764	OW	SOL	5214	40.020	46.320	46.870	1.00	0.00
ATOM	16765	HW1	SOL	5214	40.560	47.100	46.970	1.00	0.00
ATOM	16766	HW2	SOL	5214	40.120	46.070	45.950	1.00	0.00
ATOM	16767	OW	SOL	5215	25.760	40.340	21.210	1.00	0.00
ATOM	16768	HW1	SOL	5215	25.880	39.860	22.030	1.00	0.00
ATOM	16769	HW2	SOL	5215	25.190	39.780	20.690	1.00	0.00
ATOM	16770	OW	SOL	5216	0.280	9.500	53.620	1.00	0.00
ATOM	16771	HW1	SOL	5216	-0.630	9.350	53.890	1.00	0.00
ATOM	16772	HW2	SOL	5216	0.230	9.690	52.690	1.00	0.00
ATOM	16773	OW	SOL	5217	4.270	37.590	55.040	1.00	0.00
ATOM	16774	HW1	SOL	5217	3.460	37.640	55.540	1.00	0.00
ATOM	16775	HW2	SOL	5217	4.800	38.320	55.360	1.00	0.00
ATOM	16776	OW	SOL	5218	33.960	20.340	33.290	1.00	0.00
ATOM	16777	HW1	SOL	5218	33.630	21.090	33.790	1.00	0.00
ATOM	16778	HW2	SOL	5218	33.200	19.770	33.170	1.00	0.00
ATOM	16779	OW	SOL	5219	50.640	14.110	46.910	1.00	0.00
ATOM	16780	HW1	SOL	5219	49.860	14.650	46.780	1.00	0.00
ATOM	16781	HW2	SOL	5219	51.330	14.730	47.140	1.00	0.00
ATOM	16782	OW	SOL	5220	26.700	5.440	32.390	1.00	0.00
ATOM	16783	HW1	SOL	5220	25.900	5.530	31.870	1.00	0.00
ATOM	16784	HW2	SOL	5220	26.570	6.010	33.140	1.00	0.00
ATOM	16785	OW	SOL	5221	47.360	24.850	27.300	1.00	0.00
ATOM	16786	HW1	SOL	5221	47.920	24.470	26.620	1.00	0.00
ATOM	16787	HW2	SOL	5221	46.770	25.430	26.820	1.00	0.00
ATOM	16788	OW	SOL	5222	31.820	17.170	14.110	1.00	0.00
ATOM	16789	HW1	SOL	5222	32.330	16.370	14.220	1.00	0.00
ATOM	16790	HW2	SOL	5222	31.070	16.900	13.580	1.00	0.00
ATOM	16791	OW	SOL	5223	33.170	34.180	10.710	1.00	0.00
ATOM	16792	HW1	SOL	5223	32.640	34.010	9.930	1.00	0.00
ATOM	16793	HW2	SOL	5223	32.540	34.450	11.380	1.00	0.00
ATOM	16794	OW	SOL	5224	52.990	15.690	47.300	1.00	0.00
ATOM	16795	HW1	SOL	5224	53.470	15.160	47.940	1.00	0.00
ATOM	16796	HW2	SOL	5224	53.300	16.580	47.440	1.00	0.00
ATOM	16797	OW	SOL	5225	53.620	33.600	51.890	1.00	0.00
ATOM	16798	HW1	SOL	5225	52.730	33.690	51.550	1.00	0.00
ATOM	16799	HW2	SOL	5225	53.620	34.090	52.700	1.00	0.00
ATOM	16800	OW	SOL	5226	20.010	35.690	52.590	1.00	0.00
ATOM	16801	HW1	SOL	5226	20.170	36.040	53.460	1.00	0.00
ATOM	16802	HW2	SOL	5226	20.310	36.380	52.000	1.00	0.00
ATOM	16803	OW	SOL	5227	44.540	4.100	21.890	1.00	0.00
ATOM	16804	HW1	SOL	5227	44.770	4.940	22.290	1.00	0.00
ATOM	16805	HW2	SOL	5227	44.700	4.220	20.950	1.00	0.00
ATOM	16806	OW	SOL	5228	13.440	16.120	22.440	1.00	0.00
ATOM	16807	HW1	SOL	5228	12.760	15.870	21.800	1.00	0.00

ATOM	16808	HW2	SOL	5228	12.960	16.310	23.240	1.00	0.00
ATOM	16809	OW	SOL	5229	30.160	30.800	16.110	1.00	0.00
ATOM	16810	HW1	SOL	5229	29.940	31.210	16.950	1.00	0.00
ATOM	16811	HW2	SOL	5229	29.490	31.130	15.510	1.00	0.00
ATOM	16812	OW	SOL	5230	43.480	9.410	3.980	1.00	0.00
ATOM	16813	HW1	SOL	5230	43.840	8.530	3.960	1.00	0.00
ATOM	16814	HW2	SOL	5230	43.680	9.770	3.110	1.00	0.00
ATOM	16815	OW	SOL	5231	31.480	8.000	0.320	1.00	0.00
ATOM	16816	HW1	SOL	5231	30.790	7.390	0.040	1.00	0.00
ATOM	16817	HW2	SOL	5231	31.920	8.250	-0.500	1.00	0.00
ATOM	16818	OW	SOL	5232	28.260	8.330	21.960	1.00	0.00
ATOM	16819	HW1	SOL	5232	28.300	7.990	21.070	1.00	0.00
ATOM	16820	HW2	SOL	5232	28.220	9.280	21.860	1.00	0.00
ATOM	16821	OW	SOL	5233	9.550	23.320	40.790	1.00	0.00
ATOM	16822	HW1	SOL	5233	9.910	24.200	40.680	1.00	0.00
ATOM	16823	HW2	SOL	5233	8.630	23.470	41.000	1.00	0.00
ATOM	16824	OW	SOL	5234	38.570	9.580	5.410	1.00	0.00
ATOM	16825	HW1	SOL	5234	39.260	10.060	4.950	1.00	0.00
ATOM	16826	HW2	SOL	5234	37.780	9.760	4.910	1.00	0.00
ATOM	16827	OW	SOL	5235	40.460	0.390	10.570	1.00	0.00
ATOM	16828	HW1	SOL	5235	40.280	-0.490	10.230	1.00	0.00
ATOM	16829	HW2	SOL	5235	39.650	0.880	10.410	1.00	0.00
ATOM	16830	OW	SOL	5236	1.590	12.780	27.370	1.00	0.00
ATOM	16831	HW1	SOL	5236	2.070	12.770	26.540	1.00	0.00
ATOM	16832	HW2	SOL	5236	1.070	13.580	27.330	1.00	0.00
ATOM	16833	OW	SOL	5237	22.390	8.500	9.230	1.00	0.00
ATOM	16834	HW1	SOL	5237	23.100	8.160	9.790	1.00	0.00
ATOM	16835	HW2	SOL	5237	21.930	9.120	9.800	1.00	0.00
ATOM	16836	OW	SOL	5238	40.990	6.990	50.680	1.00	0.00
ATOM	16837	HW1	SOL	5238	41.110	6.540	51.520	1.00	0.00
ATOM	16838	HW2	SOL	5238	41.830	6.890	50.230	1.00	0.00
ATOM	16839	OW	SOL	5239	54.330	20.990	42.150	1.00	0.00
ATOM	16840	HW1	SOL	5239	53.880	21.710	41.720	1.00	0.00
ATOM	16841	HW2	SOL	5239	53.630	20.440	42.510	1.00	0.00
ATOM	16842	OW	SOL	5240	31.950	6.120	51.680	1.00	0.00
ATOM	16843	HW1	SOL	5240	32.130	5.190	51.830	1.00	0.00
ATOM	16844	HW2	SOL	5240	31.010	6.170	51.530	1.00	0.00
ATOM	16845	OW	SOL	5241	2.390	17.190	16.630	1.00	0.00
ATOM	16846	HW1	SOL	5241	2.920	17.190	17.420	1.00	0.00
ATOM	16847	HW2	SOL	5241	2.230	18.110	16.440	1.00	0.00
ATOM	16848	OW	SOL	5242	54.190	5.330	30.230	1.00	0.00
ATOM	16849	HW1	SOL	5242	53.490	5.960	30.390	1.00	0.00
ATOM	16850	HW2	SOL	5242	55.000	5.850	30.250	1.00	0.00
ATOM	16851	OW	SOL	5243	14.800	7.700	5.280	1.00	0.00

ATOM	16852	HW1	SOL	5243	14.090	7.960	4.700	1.00	0.00
ATOM	16853	HW2	SOL	5243	14.990	6.790	5.040	1.00	0.00
ATOM	16854	OW	SOL	5244	43.880	38.450	47.870	1.00	0.00
ATOM	16855	HW1	SOL	5244	43.210	38.860	48.420	1.00	0.00
ATOM	16856	HW2	SOL	5244	43.770	38.850	47.010	1.00	0.00
ATOM	16857	OW	SOL	5245	26.570	29.690	29.400	1.00	0.00
ATOM	16858	HW1	SOL	5245	26.240	28.790	29.470	1.00	0.00
ATOM	16859	HW2	SOL	5245	26.560	30.020	30.300	1.00	0.00
ATOM	16860	OW	SOL	5246	24.700	12.980	13.000	1.00	0.00
ATOM	16861	HW1	SOL	5246	24.860	12.080	13.280	1.00	0.00
ATOM	16862	HW2	SOL	5246	23.810	12.960	12.630	1.00	0.00
ATOM	16863	OW	SOL	5247	28.000	38.350	24.750	1.00	0.00
ATOM	16864	HW1	SOL	5247	28.260	38.810	25.560	1.00	0.00
ATOM	16865	HW2	SOL	5247	28.770	38.400	24.190	1.00	0.00
ATOM	16866	OW	SOL	5248	17.010	19.300	28.840	1.00	0.00
ATOM	16867	HW1	SOL	5248	17.850	18.840	28.900	1.00	0.00
ATOM	16868	HW2	SOL	5248	17.260	20.210	28.680	1.00	0.00
ATOM	16869	OW	SOL	5249	28.960	32.700	49.860	1.00	0.00
ATOM	16870	HW1	SOL	5249	28.260	32.120	50.150	1.00	0.00
ATOM	16871	HW2	SOL	5249	29.530	32.780	50.620	1.00	0.00
ATOM	16872	OW	SOL	5250	29.900	37.510	10.350	1.00	0.00
ATOM	16873	HW1	SOL	5250	29.700	38.420	10.130	1.00	0.00
ATOM	16874	HW2	SOL	5250	30.760	37.350	9.980	1.00	0.00
ATOM	16875	OW	SOL	5251	19.050	9.260	27.710	1.00	0.00
ATOM	16876	HW1	SOL	5251	19.320	9.100	28.610	1.00	0.00
ATOM	16877	HW2	SOL	5251	19.510	10.060	27.460	1.00	0.00
ATOM	16878	OW	SOL	5252	13.180	11.580	23.270	1.00	0.00
ATOM	16879	HW1	SOL	5252	13.470	12.490	23.220	1.00	0.00
ATOM	16880	HW2	SOL	5252	12.360	11.620	23.770	1.00	0.00
ATOM	16881	OW	SOL	5253	24.640	32.630	18.480	1.00	0.00
ATOM	16882	HW1	SOL	5253	23.910	33.100	18.080	1.00	0.00
ATOM	16883	HW2	SOL	5253	24.360	31.710	18.480	1.00	0.00
ATOM	16884	OW	SOL	5254	40.750	35.990	40.470	1.00	0.00
ATOM	16885	HW1	SOL	5254	40.910	35.730	41.370	1.00	0.00
ATOM	16886	HW2	SOL	5254	41.530	35.690	39.990	1.00	0.00
ATOM	16887	OW	SOL	5255	34.050	14.790	50.260	1.00	0.00
ATOM	16888	HW1	SOL	5255	33.130	15.020	50.120	1.00	0.00
ATOM	16889	HW2	SOL	5255	34.460	14.930	49.410	1.00	0.00
ATOM	16890	OW	SOL	5256	18.770	23.120	34.630	1.00	0.00
ATOM	16891	HW1	SOL	5256	18.900	22.170	34.720	1.00	0.00
ATOM	16892	HW2	SOL	5256	18.500	23.410	35.510	1.00	0.00
ATOM	16893	OW	SOL	5257	39.980	46.530	20.250	1.00	0.00
ATOM	16894	HW1	SOL	5257	39.400	47.220	19.920	1.00	0.00
ATOM	16895	HW2	SOL	5257	40.700	46.500	19.630	1.00	0.00

ATOM	16896	OW	SOL	5258	2.100	55.250	36.280	1.00	0.00
ATOM	16897	HW1	SOL	5258	2.440	55.690	35.500	1.00	0.00
ATOM	16898	HW2	SOL	5258	1.180	55.100	36.090	1.00	0.00
ATOM	16899	OW	SOL	5259	30.500	35.320	2.470	1.00	0.00
ATOM	16900	HW1	SOL	5259	30.130	34.520	2.840	1.00	0.00
ATOM	16901	HW2	SOL	5259	30.060	36.030	2.930	1.00	0.00
ATOM	16902	OW	SOL	5260	37.620	52.880	6.980	1.00	0.00
ATOM	16903	HW1	SOL	5260	36.720	52.780	7.290	1.00	0.00
ATOM	16904	HW2	SOL	5260	38.150	52.350	7.580	1.00	0.00
ATOM	16905	OW	SOL	5261	35.410	51.560	35.250	1.00	0.00
ATOM	16906	HW1	SOL	5261	35.680	50.770	35.700	1.00	0.00
ATOM	16907	HW2	SOL	5261	34.450	51.530	35.270	1.00	0.00
ATOM	16908	OW	SOL	5262	44.410	46.700	45.570	1.00	0.00
ATOM	16909	HW1	SOL	5262	45.300	46.640	45.230	1.00	0.00
ATOM	16910	HW2	SOL	5262	44.060	45.810	45.500	1.00	0.00
ATOM	16911	OW	SOL	5263	21.230	36.030	10.480	1.00	0.00
ATOM	16912	HW1	SOL	5263	20.410	36.500	10.330	1.00	0.00
ATOM	16913	HW2	SOL	5263	20.990	35.330	11.090	1.00	0.00
ATOM	16914	OW	SOL	5264	34.040	8.120	40.460	1.00	0.00
ATOM	16915	HW1	SOL	5264	34.020	7.270	40.890	1.00	0.00
ATOM	16916	HW2	SOL	5264	33.560	8.700	41.050	1.00	0.00
ATOM	16917	OW	SOL	5265	0.990	18.370	3.610	1.00	0.00
ATOM	16918	HW1	SOL	5265	1.380	19.230	3.450	1.00	0.00
ATOM	16919	HW2	SOL	5265	0.690	18.080	2.750	1.00	0.00
ATOM	16920	OW	SOL	5266	21.580	55.570	23.910	1.00	0.00
ATOM	16921	HW1	SOL	5266	20.720	55.780	24.290	1.00	0.00
ATOM	16922	HW2	SOL	5266	21.380	55.030	23.150	1.00	0.00
ATOM	16923	OW	SOL	5267	13.400	38.900	37.680	1.00	0.00
ATOM	16924	HW1	SOL	5267	12.920	38.100	37.450	1.00	0.00
ATOM	16925	HW2	SOL	5267	12.740	39.590	37.690	1.00	0.00
ATOM	16926	OW	SOL	5268	18.150	51.690	6.620	1.00	0.00
ATOM	16927	HW1	SOL	5268	17.490	51.420	7.260	1.00	0.00
ATOM	16928	HW2	SOL	5268	18.820	51.010	6.660	1.00	0.00
ATOM	16929	OW	SOL	5269	21.870	23.670	0.310	1.00	0.00
ATOM	16930	HW1	SOL	5269	21.100	23.910	0.810	1.00	0.00
ATOM	16931	HW2	SOL	5269	21.570	23.620	-0.600	1.00	0.00
ATOM	16932	OW	SOL	5270	11.150	0.130	53.380	1.00	0.00
ATOM	16933	HW1	SOL	5270	10.430	0.760	53.260	1.00	0.00
ATOM	16934	HW2	SOL	5270	11.180	-0.020	54.320	1.00	0.00
ATOM	16935	OW	SOL	5271	28.300	6.340	48.130	1.00	0.00
ATOM	16936	HW1	SOL	5271	27.770	7.120	48.270	1.00	0.00
ATOM	16937	HW2	SOL	5271	27.670	5.660	47.910	1.00	0.00
ATOM	16938	OW	SOL	5272	47.340	10.330	10.220	1.00	0.00
ATOM	16939	HW1	SOL	5272	47.560	9.630	9.610	1.00	0.00

ATOM	16940	HW2	SOL	5272	46.520	10.690	9.890	1.00	0.00
ATOM	16941	OW	SOL	5273	36.600	49.330	49.400	1.00	0.00
ATOM	16942	HW1	SOL	5273	37.430	49.760	49.190	1.00	0.00
ATOM	16943	HW2	SOL	5273	36.350	49.680	50.260	1.00	0.00
ATOM	16944	OW	SOL	5274	26.660	47.770	4.940	1.00	0.00
ATOM	16945	HW1	SOL	5274	27.110	46.960	4.700	1.00	0.00
ATOM	16946	HW2	SOL	5274	27.180	48.120	5.670	1.00	0.00
ATOM	16947	OW	SOL	5275	33.670	28.970	46.770	1.00	0.00
ATOM	16948	HW1	SOL	5275	33.260	29.400	46.020	1.00	0.00
ATOM	16949	HW2	SOL	5275	34.610	29.060	46.610	1.00	0.00
ATOM	16950	OW	SOL	5276	11.430	3.860	34.500	1.00	0.00
ATOM	16951	HW1	SOL	5276	11.380	3.650	35.430	1.00	0.00
ATOM	16952	HW2	SOL	5276	11.240	3.040	34.050	1.00	0.00
ATOM	16953	OW	SOL	5277	12.410	33.780	49.340	1.00	0.00
ATOM	16954	HW1	SOL	5277	12.520	33.500	48.430	1.00	0.00
ATOM	16955	HW2	SOL	5277	11.560	33.440	49.600	1.00	0.00
ATOM	16956	OW	SOL	5278	47.330	26.530	16.970	1.00	0.00
ATOM	16957	HW1	SOL	5278	46.650	26.240	17.580	1.00	0.00
ATOM	16958	HW2	SOL	5278	46.920	27.230	16.470	1.00	0.00
ATOM	16959	OW	SOL	5279	33.920	21.860	4.150	1.00	0.00
ATOM	16960	HW1	SOL	5279	33.270	22.400	4.590	1.00	0.00
ATOM	16961	HW2	SOL	5279	33.940	22.180	3.250	1.00	0.00
ATOM	16962	OW	SOL	5280	24.780	26.350	20.110	1.00	0.00
ATOM	16963	HW1	SOL	5280	24.080	25.780	20.420	1.00	0.00
ATOM	16964	HW2	SOL	5280	24.390	27.220	20.090	1.00	0.00
ATOM	16965	OW	SOL	5281	28.470	46.730	45.330	1.00	0.00
ATOM	16966	HW1	SOL	5281	28.880	46.190	46.000	1.00	0.00
ATOM	16967	HW2	SOL	5281	28.020	47.420	45.820	1.00	0.00
ATOM	16968	OW	SOL	5282	55.580	17.480	27.040	1.00	0.00
ATOM	16969	HW1	SOL	5282	54.800	18.040	27.040	1.00	0.00
ATOM	16970	HW2	SOL	5282	56.260	18.020	27.430	1.00	0.00
ATOM	16971	OW	SOL	5283	25.790	28.720	46.950	1.00	0.00
ATOM	16972	HW1	SOL	5283	25.040	29.240	46.640	1.00	0.00
ATOM	16973	HW2	SOL	5283	26.460	29.370	47.150	1.00	0.00
ATOM	16974	OW	SOL	5284	2.380	47.710	52.350	1.00	0.00
ATOM	16975	HW1	SOL	5284	2.850	46.980	52.750	1.00	0.00
ATOM	16976	HW2	SOL	5284	3.060	48.190	51.870	1.00	0.00
ATOM	16977	OW	SOL	5285	2.170	33.030	34.770	1.00	0.00
ATOM	16978	HW1	SOL	5285	1.720	32.230	35.060	1.00	0.00
ATOM	16979	HW2	SOL	5285	1.940	33.120	33.850	1.00	0.00
ATOM	16980	OW	SOL	5286	35.650	23.010	13.200	1.00	0.00
ATOM	16981	HW1	SOL	5286	35.150	22.470	12.590	1.00	0.00
ATOM	16982	HW2	SOL	5286	35.160	22.960	14.020	1.00	0.00
ATOM	16983	OW	SOL	5287	37.880	8.690	26.510	1.00	0.00

ATOM	16984	HW1	SOL	5287	38.290	9.270	27.140	1.00	0.00
ATOM	16985	HW2	SOL	5287	37.660	7.900	27.010	1.00	0.00
ATOM	16986	OW	SOL	5288	14.540	18.650	39.720	1.00	0.00
ATOM	16987	HW1	SOL	5288	14.180	17.760	39.720	1.00	0.00
ATOM	16988	HW2	SOL	5288	13.850	19.190	39.350	1.00	0.00
ATOM	16989	OW	SOL	5289	11.600	5.740	6.090	1.00	0.00
ATOM	16990	HW1	SOL	5289	11.450	5.450	6.990	1.00	0.00
ATOM	16991	HW2	SOL	5289	10.730	5.720	5.680	1.00	0.00
ATOM	16992	OW	SOL	5290	46.660	30.930	5.240	1.00	0.00
ATOM	16993	HW1	SOL	5290	46.420	30.060	5.550	1.00	0.00
ATOM	16994	HW2	SOL	5290	46.040	31.110	4.530	1.00	0.00
ATOM	16995	OW	SOL	5291	22.900	53.520	25.660	1.00	0.00
ATOM	16996	HW1	SOL	5291	22.240	53.740	26.310	1.00	0.00
ATOM	16997	HW2	SOL	5291	22.790	54.180	24.980	1.00	0.00
ATOM	16998	OW	SOL	5292	6.490	31.160	22.810	1.00	0.00
ATOM	16999	HW1	SOL	5292	6.220	30.420	22.270	1.00	0.00
ATOM	17000	HW2	SOL	5292	6.860	30.760	23.590	1.00	0.00
ATOM	17001	OW	SOL	5293	37.660	35.960	17.640	1.00	0.00
ATOM	17002	HW1	SOL	5293	37.050	35.760	16.920	1.00	0.00
ATOM	17003	HW2	SOL	5293	38.040	35.110	17.870	1.00	0.00
ATOM	17004	OW	SOL	5294	27.930	32.540	0.800	1.00	0.00
ATOM	17005	HW1	SOL	5294	28.400	32.590	1.630	1.00	0.00
ATOM	17006	HW2	SOL	5294	28.610	32.690	0.130	1.00	0.00
ATOM	17007	OW	SOL	5295	55.180	7.820	40.630	1.00	0.00
ATOM	17008	HW1	SOL	5295	55.480	7.960	39.730	1.00	0.00
ATOM	17009	HW2	SOL	5295	55.990	7.700	41.130	1.00	0.00
ATOM	17010	OW	SOL	5296	32.620	51.930	5.140	1.00	0.00
ATOM	17011	HW1	SOL	5296	33.480	52.300	5.330	1.00	0.00
ATOM	17012	HW2	SOL	5296	32.120	52.650	4.770	1.00	0.00
ATOM	17013	OW	SOL	5297	50.560	19.010	36.010	1.00	0.00
ATOM	17014	HW1	SOL	5297	49.690	19.380	36.160	1.00	0.00
ATOM	17015	HW2	SOL	5297	51.080	19.330	36.750	1.00	0.00
ATOM	17016	OW	SOL	5298	5.300	31.310	18.400	1.00	0.00
ATOM	17017	HW1	SOL	5298	5.950	31.830	17.910	1.00	0.00
ATOM	17018	HW2	SOL	5298	5.690	30.440	18.460	1.00	0.00
ATOM	17019	OW	SOL	5299	32.590	54.110	55.040	1.00	0.00
ATOM	17020	HW1	SOL	5299	32.290	54.040	54.130	1.00	0.00
ATOM	17021	HW2	SOL	5299	33.190	54.850	55.040	1.00	0.00
ATOM	17022	OW	SOL	5300	38.100	5.820	52.370	1.00	0.00
ATOM	17023	HW1	SOL	5300	38.450	6.330	53.090	1.00	0.00
ATOM	17024	HW2	SOL	5300	37.950	6.460	51.670	1.00	0.00
ATOM	17025	OW	SOL	5301	4.740	42.260	14.930	1.00	0.00
ATOM	17026	HW1	SOL	5301	5.110	43.100	15.200	1.00	0.00
ATOM	17027	HW2	SOL	5301	5.070	41.630	15.560	1.00	0.00

ATOM	17028	OW	SOL	5302	53.400	1.730	42.650	1.00	0.00
ATOM	17029	HW1	SOL	5302	53.920	2.430	42.250	1.00	0.00
ATOM	17030	HW2	SOL	5302	53.680	0.930	42.200	1.00	0.00
ATOM	17031	OW	SOL	5303	49.640	0.900	21.760	1.00	0.00
ATOM	17032	HW1	SOL	5303	49.890	0.730	20.850	1.00	0.00
ATOM	17033	HW2	SOL	5303	50.440	0.730	22.260	1.00	0.00
ATOM	17034	OW	SOL	5304	22.950	53.380	53.930	1.00	0.00
ATOM	17035	HW1	SOL	5304	23.660	53.610	54.530	1.00	0.00
ATOM	17036	HW2	SOL	5304	22.150	53.530	54.440	1.00	0.00
ATOM	17037	OW	SOL	5305	40.290	6.190	46.850	1.00	0.00
ATOM	17038	HW1	SOL	5305	40.570	5.500	47.450	1.00	0.00
ATOM	17039	HW2	SOL	5305	39.370	5.990	46.670	1.00	0.00
ATOM	17040	OW	SOL	5306	49.020	0.660	0.240	1.00	0.00
ATOM	17041	HW1	SOL	5306	48.760	1.040	1.080	1.00	0.00
ATOM	17042	HW2	SOL	5306	48.960	1.390	-0.380	1.00	0.00
ATOM	17043	OW	SOL	5307	39.600	44.680	22.140	1.00	0.00
ATOM	17044	HW1	SOL	5307	39.770	45.320	21.440	1.00	0.00
ATOM	17045	HW2	SOL	5307	40.100	45.010	22.880	1.00	0.00
ATOM	17046	OW	SOL	5308	34.520	21.950	46.790	1.00	0.00
ATOM	17047	HW1	SOL	5308	35.010	21.840	45.980	1.00	0.00
ATOM	17048	HW2	SOL	5308	34.400	21.060	47.120	1.00	0.00
ATOM	17049	OW	SOL	5309	27.860	13.100	2.480	1.00	0.00
ATOM	17050	HW1	SOL	5309	26.920	12.920	2.450	1.00	0.00
ATOM	17051	HW2	SOL	5309	28.170	12.960	1.590	1.00	0.00
ATOM	17052	OW	SOL	5310	52.670	48.460	38.500	1.00	0.00
ATOM	17053	HW1	SOL	5310	53.460	47.950	38.330	1.00	0.00
ATOM	17054	HW2	SOL	5310	52.400	48.210	39.390	1.00	0.00
ATOM	17055	OW	SOL	5311	50.360	25.240	54.000	1.00	0.00
ATOM	17056	HW1	SOL	5311	51.110	25.800	54.210	1.00	0.00
ATOM	17057	HW2	SOL	5311	50.020	24.970	54.850	1.00	0.00
ATOM	17058	OW	SOL	5312	5.570	48.950	55.130	1.00	0.00
ATOM	17059	HW1	SOL	5312	6.360	48.400	55.120	1.00	0.00
ATOM	17060	HW2	SOL	5312	5.080	48.650	55.890	1.00	0.00
ATOM	17061	OW	SOL	5313	44.570	8.780	36.780	1.00	0.00
ATOM	17062	HW1	SOL	5313	43.940	8.120	37.050	1.00	0.00
ATOM	17063	HW2	SOL	5313	44.960	8.440	35.980	1.00	0.00
ATOM	17064	OW	SOL	5314	26.380	45.010	13.940	1.00	0.00
ATOM	17065	HW1	SOL	5314	25.700	45.680	13.960	1.00	0.00
ATOM	17066	HW2	SOL	5314	26.260	44.570	13.090	1.00	0.00
ATOM	17067	OW	SOL	5315	0.980	26.370	50.550	1.00	0.00
ATOM	17068	HW1	SOL	5315	0.320	26.090	51.170	1.00	0.00
ATOM	17069	HW2	SOL	5315	1.240	27.250	50.840	1.00	0.00
ATOM	17070	OW	SOL	5316	32.490	24.700	12.580	1.00	0.00
ATOM	17071	HW1	SOL	5316	33.290	24.760	12.050	1.00	0.00

ATOM	17072	HW2	SOL	5316	32.720	25.140	13.400	1.00	0.00
ATOM	17073	OW	SOL	5317	41.930	42.880	20.900	1.00	0.00
ATOM	17074	HW1	SOL	5317	41.540	42.160	20.400	1.00	0.00
ATOM	17075	HW2	SOL	5317	41.690	42.700	21.810	1.00	0.00
ATOM	17076	OW	SOL	5318	46.550	43.620	31.980	1.00	0.00
ATOM	17077	HW1	SOL	5318	45.790	44.110	31.670	1.00	0.00
ATOM	17078	HW2	SOL	5318	46.310	43.310	32.850	1.00	0.00
ATOM	17079	OW	SOL	5319	40.720	7.060	6.280	1.00	0.00
ATOM	17080	HW1	SOL	5319	40.050	6.660	5.740	1.00	0.00
ATOM	17081	HW2	SOL	5319	40.260	7.740	6.770	1.00	0.00
ATOM	17082	OW	SOL	5320	42.300	23.200	23.150	1.00	0.00
ATOM	17083	HW1	SOL	5320	42.070	22.750	23.960	1.00	0.00
ATOM	17084	HW2	SOL	5320	42.200	24.130	23.350	1.00	0.00
ATOM	17085	OW	SOL	5321	22.170	55.750	17.670	1.00	0.00
ATOM	17086	HW1	SOL	5321	21.760	54.900	17.830	1.00	0.00
ATOM	17087	HW2	SOL	5321	22.900	55.550	17.080	1.00	0.00
ATOM	17088	OW	SOL	5322	6.790	21.870	26.090	1.00	0.00
ATOM	17089	HW1	SOL	5322	6.820	21.150	26.710	1.00	0.00
ATOM	17090	HW2	SOL	5322	6.220	21.570	25.380	1.00	0.00
ATOM	17091	OW	SOL	5323	43.200	0.210	2.350	1.00	0.00
ATOM	17092	HW1	SOL	5323	43.280	-0.540	1.770	1.00	0.00
ATOM	17093	HW2	SOL	5323	43.900	0.100	2.990	1.00	0.00
ATOM	17094	OW	SOL	5324	0.260	27.710	33.560	1.00	0.00
ATOM	17095	HW1	SOL	5324	0.380	28.480	34.110	1.00	0.00
ATOM	17096	HW2	SOL	5324	-0.650	27.430	33.730	1.00	0.00
ATOM	17097	OW	SOL	5325	0.770	20.590	44.220	1.00	0.00
ATOM	17098	HW1	SOL	5325	-0.030	20.560	43.710	1.00	0.00
ATOM	17099	HW2	SOL	5325	0.530	21.030	45.040	1.00	0.00
ATOM	17100	NA	NA	5326	48.370	0.030	4.360	1.00	0.00
ATOM	17101	NA	NA	5327	4.850	47.920	6.750	1.00	0.00
ATOM	17102	NA	NA	5328	52.400	16.870	15.040	1.00	0.00
ATOM	17103	NA	NA	5329	12.140	30.060	0.770	1.00	0.00
ATOM	17104	NA	NA	5330	5.530	21.520	21.680	1.00	0.00
ATOM	17105	NA	NA	5331	43.780	45.660	12.120	1.00	0.00
ATOM	17106	NA	NA	5332	51.300	38.090	8.940	1.00	0.00
ATOM	17107	NA	NA	5333	55.280	13.860	34.590	1.00	0.00
ATOM	17108	NA	NA	5334	25.720	0.930	25.960	1.00	0.00
ATOM	17109	NA	NA	5335	14.050	20.920	20.650	1.00	0.00
ATOM	17110	NA	NA	5336	42.140	0.650	39.840	1.00	0.00
ATOM	17111	CL	CL	5337	21.620	33.710	17.460	1.00	0.00
ATOM	17112	CL	CL	5338	46.970	1.770	2.510	1.00	0.00
ATOM	17113	CL	CL	5339	28.070	14.750	20.110	1.00	0.00
ATOM	17114	CL	CL	5340	33.190	9.470	45.390	1.00	0.00
ATOM	17115	CL	CL	5341	55.740	51.130	33.220	1.00	0.00

ATOM	17116	CL	CL	5342	41.710	26.990	3.260	1.00	0.00
ATOM	17117	CL	CL	5343	3.240	19.620	26.700	1.00	0.00
ATOM	17118	CL	CL	5344	16.350	42.500	10.480	1.00	0.00
ATOM	17119	CL	CL	5345	25.570	44.740	0.080	1.00	0.00
ATOM	17120	CL	CL	5346	52.070	18.310	29.880	1.00	0.00
ATOM	17121	CL	CL	5347	41.640	16.560	20.050	1.00	0.00

PDB file of hIAPP$_{20\text{-}29}$+Mel system：

ATOM	1	O1	MOL	13	37.310	35.920	19.970	1.00	0.00
ATOM	2	O2	MOL	13	36.870	28.690	24.330	1.00	0.00
ATOM	3	N1	MOL	13	41.370	35.220	23.600	1.00	0.00
ATOM	4	N2	MOL	13	38.280	30.300	23.690	1.00	0.00
ATOM	5	C1	MOL	13	40.490	33.320	22.940	1.00	0.00
ATOM	6	C2	MOL	13	39.730	34.370	22.280	1.00	0.00
ATOM	7	C3	MOL	13	40.120	31.870	23.130	1.00	0.00
ATOM	8	C4	MOL	13	40.350	35.540	22.720	1.00	0.00
ATOM	9	C5	MOL	13	41.290	33.870	23.910	1.00	0.00
ATOM	10	C6	MOL	13	38.770	31.670	23.840	1.00	0.00
ATOM	11	C7	MOL	13	38.680	34.410	21.360	1.00	0.00
ATOM	12	C8	MOL	13	39.980	36.810	22.270	1.00	0.00
ATOM	13	C9	MOL	13	38.290	35.670	20.900	1.00	0.00
ATOM	14	C10	MOL	13	38.950	36.830	21.330	1.00	0.00
ATOM	15	C11	MOL	13	37.040	29.880	24.120	1.00	0.00
ATOM	16	C12	MOL	13	35.900	30.850	24.410	1.00	0.00
ATOM	17	C13	MOL	13	36.810	34.830	19.190	1.00	0.00
ATOM	18	H1	MOL	13	40.890	31.340	23.690	1.00	0.00
ATOM	19	H2	MOL	13	40.130	31.380	22.150	1.00	0.00
ATOM	20	H3	MOL	13	41.810	33.340	24.700	1.00	0.00
ATOM	21	H4	MOL	13	38.070	32.320	23.290	1.00	0.00
ATOM	22	H5	MOL	13	38.930	31.980	24.880	1.00	0.00
ATOM	23	H6	MOL	13	42.000	35.900	24.010	1.00	0.00
ATOM	24	H7	MOL	13	38.310	33.500	20.890	1.00	0.00
ATOM	25	H8	MOL	13	40.540	37.700	22.530	1.00	0.00
ATOM	26	H9	MOL	13	38.540	37.720	20.850	1.00	0.00
ATOM	27	H10	MOL	13	38.990	29.580	23.780	1.00	0.00
ATOM	28	H11	MOL	13	36.130	31.480	25.280	1.00	0.00
ATOM	29	H12	MOL	13	34.980	30.290	24.610	1.00	0.00
ATOM	30	H13	MOL	13	35.700	31.560	23.600	1.00	0.00
ATOM	31	H14	MOL	13	37.660	34.220	18.870	1.00	0.00
ATOM	32	H15	MOL	13	36.430	35.210	18.230	1.00	0.00
ATOM	33	H16	MOL	13	36.070	34.310	19.800	1.00	0.00
ATOM	34	CH3	ACE	1	20.210	36.060	16.050	1.00	0.00
ATOM	35	1HH3	ACE	1	19.630	35.680	15.200	1.00	0.00
ATOM	36	2HH3	ACE	1	20.670	35.130	16.380	1.00	0.00
ATOM	37	3HH3	ACE	1	20.910	36.760	15.590	1.00	0.00
ATOM	38	C	ACE	1	19.250	36.650	17.070	1.00	0.00
ATOM	39	O	ACE	1	18.740	35.850	17.850	1.00	0.00
ATOM	40	N	SER	2	19.060	37.970	17.150	1.00	0.00
ATOM	41	H	SER	2	19.620	38.580	16.570	1.00	0.00
ATOM	42	CA	SER	2	17.940	38.540	17.860	1.00	0.00
ATOM	43	HA	SER	2	17.780	37.930	18.740	1.00	0.00

ATOM	44	CB	SER	2	18.400	39.970	18.160	1.00	0.00
ATOM	45	HB1	SER	2	17.530	40.380	18.660	1.00	0.00
ATOM	46	HB2	SER	2	18.620	40.490	17.220	1.00	0.00
ATOM	47	OG	SER	2	19.390	39.850	19.150	1.00	0.00
ATOM	48	HG	SER	2	19.350	40.670	19.650	1.00	0.00
ATOM	49	C	SER	2	16.580	38.560	17.180	1.00	0.00
ATOM	50	O	SER	2	16.560	38.890	15.990	1.00	0.00
ATOM	51	N	ASN	3	15.470	38.170	17.820	1.00	0.00
ATOM	52	H	ASN	3	14.630	38.220	17.250	1.00	0.00
ATOM	53	CA	ASN	3	15.320	37.900	19.230	1.00	0.00
ATOM	54	HA	ASN	3	16.220	37.340	19.510	1.00	0.00
ATOM	55	CB	ASN	3	14.100	37.000	19.380	1.00	0.00
ATOM	56	HB1	ASN	3	14.210	36.170	18.690	1.00	0.00
ATOM	57	HB2	ASN	3	13.200	37.610	19.320	1.00	0.00
ATOM	58	CG	ASN	3	14.160	36.240	20.690	1.00	0.00
ATOM	59	OD1	ASN	3	14.990	35.360	20.900	1.00	0.00
ATOM	60	ND2	ASN	3	13.270	36.540	21.650	1.00	0.00
ATOM	61	1HD2	ASN	3	13.270	36.000	22.500	1.00	0.00
ATOM	62	2HD2	ASN	3	12.720	37.380	21.610	1.00	0.00
ATOM	63	C	ASN	3	15.280	39.200	20.020	1.00	0.00
ATOM	64	O	ASN	3	14.830	40.210	19.470	1.00	0.00
ATOM	65	N	ASN	4	15.650	39.150	21.300	1.00	0.00
ATOM	66	H	ASN	4	16.070	38.310	21.660	1.00	0.00
ATOM	67	CA	ASN	4	15.680	40.290	22.200	1.00	0.00
ATOM	68	HA	ASN	4	14.860	40.930	21.880	1.00	0.00
ATOM	69	CB	ASN	4	17.010	40.990	21.970	1.00	0.00
ATOM	70	HB1	ASN	4	17.100	41.280	20.930	1.00	0.00
ATOM	71	HB2	ASN	4	17.790	40.320	22.330	1.00	0.00
ATOM	72	CG	ASN	4	17.080	42.240	22.850	1.00	0.00
ATOM	73	OD1	ASN	4	17.750	42.330	23.880	1.00	0.00
ATOM	74	ND2	ASN	4	16.310	43.280	22.530	1.00	0.00
ATOM	75	1HD2	ASN	4	16.370	44.130	23.070	1.00	0.00
ATOM	76	2HD2	ASN	4	15.900	43.360	21.610	1.00	0.00
ATOM	77	C	ASN	4	15.260	39.840	23.600	1.00	0.00
ATOM	78	O	ASN	4	15.250	38.670	23.950	1.00	0.00
ATOM	79	N	PHE	5	15.050	40.910	24.380	1.00	0.00
ATOM	80	H	PHE	5	15.350	41.790	23.990	1.00	0.00
ATOM	81	CA	PHE	5	14.340	40.960	25.640	1.00	0.00
ATOM	82	HA	PHE	5	13.470	40.340	25.450	1.00	0.00
ATOM	83	CB	PHE	5	13.890	42.410	25.850	1.00	0.00
ATOM	84	HB1	PHE	5	13.460	42.700	26.800	1.00	0.00
ATOM	85	HB2	PHE	5	14.790	43.020	25.880	1.00	0.00
ATOM	86	CG	PHE	5	12.980	42.980	24.790	1.00	0.00
ATOM	87	CD1	PHE	5	13.190	44.320	24.430	1.00	0.00

ATOM	88	HD1	PHE	5	13.970	44.890	24.900	1.00	0.00
ATOM	89	CE1	PHE	5	12.330	44.950	23.530	1.00	0.00
ATOM	90	HE1	PHE	5	12.430	45.980	23.220	1.00	0.00
ATOM	91	CZ	PHE	5	11.280	44.230	22.940	1.00	0.00
ATOM	92	HZ	PHE	5	10.580	44.780	22.330	1.00	0.00
ATOM	93	CE2	PHE	5	11.150	42.860	23.210	1.00	0.00
ATOM	94	HE2	PHE	5	10.390	42.280	22.710	1.00	0.00
ATOM	95	CD2	PHE	5	11.930	42.260	24.210	1.00	0.00
ATOM	96	HD2	PHE	5	11.800	41.210	24.410	1.00	0.00
ATOM	97	C	PHE	5	15.170	40.410	26.800	1.00	0.00
ATOM	98	O	PHE	5	16.300	39.940	26.700	1.00	0.00
ATOM	99	N	GLY	6	14.520	40.520	27.960	1.00	0.00
ATOM	100	H	GLY	6	13.550	40.800	27.870	1.00	0.00
ATOM	101	CA	GLY	6	15.140	40.210	29.230	1.00	0.00
ATOM	102	HA1	GLY	6	16.110	39.740	29.130	1.00	0.00
ATOM	103	HA2	GLY	6	14.390	39.590	29.730	1.00	0.00
ATOM	104	C	GLY	6	15.360	41.420	30.130	1.00	0.00
ATOM	105	O	GLY	6	15.200	42.540	29.650	1.00	0.00
ATOM	106	N	ALA	7	15.610	41.330	31.440	1.00	0.00
ATOM	107	H	ALA	7	15.740	42.150	32.010	1.00	0.00
ATOM	108	CA	ALA	7	16.030	40.130	32.130	1.00	0.00
ATOM	109	HA	ALA	7	16.090	39.390	31.340	1.00	0.00
ATOM	110	CB	ALA	7	17.410	40.440	32.710	1.00	0.00
ATOM	111	HB1	ALA	7	17.600	39.590	33.350	1.00	0.00
ATOM	112	HB2	ALA	7	18.210	40.320	31.980	1.00	0.00
ATOM	113	HB3	ALA	7	17.470	41.290	33.400	1.00	0.00
ATOM	114	C	ALA	7	15.020	39.830	33.230	1.00	0.00
ATOM	115	O	ALA	7	14.690	40.770	33.960	1.00	0.00
ATOM	116	N	ILE	8	14.610	38.580	33.460	1.00	0.00
ATOM	117	H	ILE	8	13.980	38.440	34.240	1.00	0.00
ATOM	118	CA	ILE	8	14.910	37.410	32.660	1.00	0.00
ATOM	119	HA	ILE	8	14.890	37.730	31.620	1.00	0.00
ATOM	120	CB	ILE	8	13.820	36.350	32.830	1.00	0.00
ATOM	121	HB	ILE	8	12.860	36.840	32.680	1.00	0.00
ATOM	122	CG2	ILE	8	13.770	35.340	31.690	1.00	0.00
ATOM	123	1HG2	ILE	8	14.530	34.570	31.840	1.00	0.00
ATOM	124	2HG2	ILE	8	12.810	34.830	31.730	1.00	0.00
ATOM	125	3HG2	ILE	8	13.890	35.860	30.740	1.00	0.00
ATOM	126	CG1	ILE	8	13.920	35.680	34.190	1.00	0.00
ATOM	127	1HG1	ILE	8	14.920	35.270	34.350	1.00	0.00
ATOM	128	2HG1	ILE	8	13.730	36.520	34.860	1.00	0.00
ATOM	129	CD	ILE	8	12.800	34.700	34.560	1.00	0.00
ATOM	130	HD1	ILE	8	12.910	33.670	34.230	1.00	0.00
ATOM	131	HD2	ILE	8	11.800	35.000	34.240	1.00	0.00

ATOM	132	HD3	ILE	8	12.710	34.820	35.640	1.00	0.00
ATOM	133	C	ILE	8	16.340	36.950	32.880	1.00	0.00
ATOM	134	O	ILE	8	16.880	37.170	33.960	1.00	0.00
ATOM	135	N	LEU	9	16.910	36.310	31.860	1.00	0.00
ATOM	136	H	LEU	9	16.450	36.270	30.960	1.00	0.00
ATOM	137	CA	LEU	9	18.320	35.980	31.800	1.00	0.00
ATOM	138	HA	LEU	9	18.820	36.310	32.720	1.00	0.00
ATOM	139	CB	LEU	9	18.390	34.460	31.740	1.00	0.00
ATOM	140	HB1	LEU	9	17.950	34.030	30.840	1.00	0.00
ATOM	141	HB2	LEU	9	19.420	34.090	31.790	1.00	0.00
ATOM	142	CG	LEU	9	17.720	33.750	32.920	1.00	0.00
ATOM	143	HG	LEU	9	16.650	33.940	32.950	1.00	0.00
ATOM	144	CD1	LEU	9	18.300	34.030	34.300	1.00	0.00
ATOM	145	1HD1	LEU	9	19.290	33.580	34.430	1.00	0.00
ATOM	146	2HD1	LEU	9	17.680	33.490	35.020	1.00	0.00
ATOM	147	3HD1	LEU	9	18.330	35.090	34.540	1.00	0.00
ATOM	148	CD2	LEU	9	17.650	32.230	32.760	1.00	0.00
ATOM	149	1HD2	LEU	9	18.620	31.730	32.880	1.00	0.00
ATOM	150	2HD2	LEU	9	17.340	32.040	31.740	1.00	0.00
ATOM	151	3HD2	LEU	9	17.040	31.870	33.580	1.00	0.00
ATOM	152	C	LEU	9	19.050	36.720	30.690	1.00	0.00
ATOM	153	O	LEU	9	18.560	37.760	30.240	1.00	0.00
ATOM	154	N	SER	10	20.240	36.260	30.310	1.00	0.00
ATOM	155	H	SER	10	20.730	35.510	30.760	1.00	0.00
ATOM	156	CA	SER	10	20.780	36.800	29.070	1.00	0.00
ATOM	157	HA	SER	10	19.970	37.090	28.390	1.00	0.00
ATOM	158	CB	SER	10	21.680	37.960	29.480	1.00	0.00
ATOM	159	HB1	SER	10	22.520	37.610	30.070	1.00	0.00
ATOM	160	HB2	SER	10	22.090	38.510	28.630	1.00	0.00
ATOM	161	OG	SER	10	20.960	38.990	30.110	1.00	0.00
ATOM	162	HG	SER	10	20.040	38.730	30.180	1.00	0.00
ATOM	163	C	SER	10	21.620	35.820	28.260	1.00	0.00
ATOM	164	O	SER	10	21.940	34.740	28.750	1.00	0.00
ATOM	165	N	SER	11	22.000	36.280	27.070	1.00	0.00
ATOM	166	H	SER	11	21.780	37.210	26.760	1.00	0.00
ATOM	167	CA	SER	11	22.790	35.530	26.110	1.00	0.00
ATOM	168	HA	SER	11	23.590	34.940	26.550	1.00	0.00
ATOM	169	CB	SER	11	22.010	34.340	25.540	1.00	0.00
ATOM	170	HB1	SER	11	22.680	33.750	24.920	1.00	0.00
ATOM	171	HB2	SER	11	21.710	33.750	26.410	1.00	0.00
ATOM	172	OG	SER	11	20.830	34.660	24.850	1.00	0.00
ATOM	173	HG	SER	11	20.230	33.940	25.040	1.00	0.00
ATOM	174	C	SER	11	23.490	36.290	24.990	1.00	0.00
ATOM	175	O	SER	11	24.430	35.720	24.450	1.00	0.00

ATOM	176	N	NH2	12	23.020	37.490	24.650	1.00	0.00
ATOM	177	H1	NH2	12	23.440	37.920	23.840	1.00	0.00
ATOM	178	H2	NH2	12	22.290	37.930	25.190	1.00	0.00
ATOM	179	CH3	ACE	1	12.620	37.040	25.930	1.00	0.00
ATOM	180	1HH3	ACE	1	12.480	37.960	26.490	1.00	0.00
ATOM	181	2HH3	ACE	1	11.790	36.330	25.990	1.00	0.00
ATOM	182	3HH3	ACE	1	12.840	37.150	24.870	1.00	0.00
ATOM	183	C	ACE	1	13.840	36.510	26.680	1.00	0.00
ATOM	184	O	ACE	1	13.590	35.680	27.540	1.00	0.00
ATOM	185	N	SER	2	15.060	36.860	26.280	1.00	0.00
ATOM	186	H	SER	2	15.140	37.550	25.540	1.00	0.00
ATOM	187	CA	SER	2	16.260	36.190	26.730	1.00	0.00
ATOM	188	HA	SER	2	16.030	35.130	26.810	1.00	0.00
ATOM	189	CB	SER	2	16.670	36.620	28.130	1.00	0.00
ATOM	190	HB1	SER	2	16.820	37.700	28.130	1.00	0.00
ATOM	191	HB2	SER	2	17.630	36.150	28.350	1.00	0.00
ATOM	192	OG	SER	2	15.700	36.340	29.110	1.00	0.00
ATOM	193	HG	SER	2	14.870	36.130	28.660	1.00	0.00
ATOM	194	C	SER	2	17.500	36.220	25.850	1.00	0.00
ATOM	195	O	SER	2	18.280	35.280	25.970	1.00	0.00
ATOM	196	N	ASN	3	17.750	37.220	24.990	1.00	0.00
ATOM	197	H	ASN	3	17.030	37.930	25.060	1.00	0.00
ATOM	198	CA	ASN	3	19.020	37.570	24.370	1.00	0.00
ATOM	199	HA	ASN	3	19.780	36.870	24.710	1.00	0.00
ATOM	200	CB	ASN	3	19.370	38.990	24.800	1.00	0.00
ATOM	201	HB1	ASN	3	20.180	39.290	24.140	1.00	0.00
ATOM	202	HB2	ASN	3	18.510	39.650	24.720	1.00	0.00
ATOM	203	CG	ASN	3	19.930	39.000	26.210	1.00	0.00
ATOM	204	OD1	ASN	3	21.130	38.940	26.460	1.00	0.00
ATOM	205	ND2	ASN	3	19.050	39.220	27.190	1.00	0.00
ATOM	206	1HD2	ASN	3	19.310	39.260	28.160	1.00	0.00
ATOM	207	2HD2	ASN	3	18.110	39.510	26.940	1.00	0.00
ATOM	208	C	ASN	3	18.900	37.410	22.860	1.00	0.00
ATOM	209	O	ASN	3	17.990	37.780	22.130	1.00	0.00
ATOM	210	N	ASN	4	20.020	36.880	22.370	1.00	0.00
ATOM	211	H	ASN	4	20.620	36.520	23.100	1.00	0.00
ATOM	212	CA	ASN	4	20.350	36.610	20.980	1.00	0.00
ATOM	213	HA	ASN	4	19.730	37.220	20.330	1.00	0.00
ATOM	214	CB	ASN	4	20.250	35.110	20.720	1.00	0.00
ATOM	215	HB1	ASN	4	20.960	34.590	21.370	1.00	0.00
ATOM	216	HB2	ASN	4	20.410	34.920	19.650	1.00	0.00
ATOM	217	CG	ASN	4	18.870	34.590	21.070	1.00	0.00
ATOM	218	OD1	ASN	4	18.710	33.880	22.060	1.00	0.00
ATOM	219	ND2	ASN	4	17.820	34.900	20.300	1.00	0.00

ATOM	220	1HD2	ASN	4	16.940	34.470	20.530	1.00	0.00
ATOM	221	2HD2	ASN	4	17.910	35.480	19.470	1.00	0.00
ATOM	222	C	ASN	4	21.720	37.140	20.580	1.00	0.00
ATOM	223	O	ASN	4	22.700	36.910	21.280	1.00	0.00
ATOM	224	N	PHE	5	21.860	37.650	19.350	1.00	0.00
ATOM	225	H	PHE	5	21.030	37.800	18.780	1.00	0.00
ATOM	226	CA	PHE	5	23.050	38.280	18.840	1.00	0.00
ATOM	227	HA	PHE	5	23.890	37.710	19.240	1.00	0.00
ATOM	228	CB	PHE	5	23.150	39.770	19.190	1.00	0.00
ATOM	229	HB1	PHE	5	23.960	40.260	18.660	1.00	0.00
ATOM	230	HB2	PHE	5	22.190	40.140	18.840	1.00	0.00
ATOM	231	CG	PHE	5	23.540	40.210	20.580	1.00	0.00
ATOM	232	CD1	PHE	5	24.830	40.210	21.110	1.00	0.00
ATOM	233	HD1	PHE	5	25.650	39.950	20.450	1.00	0.00
ATOM	234	CE1	PHE	5	25.040	40.510	22.460	1.00	0.00
ATOM	235	HE1	PHE	5	26.060	40.460	22.820	1.00	0.00
ATOM	236	CZ	PHE	5	23.940	40.590	23.320	1.00	0.00
ATOM	237	HZ	PHE	5	24.090	40.640	24.390	1.00	0.00
ATOM	238	CE2	PHE	5	22.630	40.580	22.810	1.00	0.00
ATOM	239	HE2	PHE	5	21.790	40.690	23.470	1.00	0.00
ATOM	240	CD2	PHE	5	22.450	40.420	21.430	1.00	0.00
ATOM	241	HD2	PHE	5	21.430	40.380	21.080	1.00	0.00
ATOM	242	C	PHE	5	23.050	38.190	17.320	1.00	0.00
ATOM	243	O	PHE	5	22.120	38.670	16.680	1.00	0.00
ATOM	244	N	GLY	6	24.080	37.640	16.670	1.00	0.00
ATOM	245	H	GLY	6	24.220	37.910	15.700	1.00	0.00
ATOM	246	CA	GLY	6	25.040	36.700	17.210	1.00	0.00
ATOM	247	HA1	GLY	6	25.520	36.130	16.410	1.00	0.00
ATOM	248	HA2	GLY	6	24.640	35.950	17.890	1.00	0.00
ATOM	249	C	GLY	6	26.260	37.320	17.890	1.00	0.00
ATOM	250	O	GLY	6	26.610	38.480	17.670	1.00	0.00
ATOM	251	N	ALA	7	26.750	36.560	18.870	1.00	0.00
ATOM	252	H	ALA	7	26.430	35.620	18.990	1.00	0.00
ATOM	253	CA	ALA	7	28.040	36.750	19.500	1.00	0.00
ATOM	254	HA	ALA	7	28.390	37.770	19.300	1.00	0.00
ATOM	255	CB	ALA	7	27.840	36.610	21.010	1.00	0.00
ATOM	256	HB1	ALA	7	28.580	37.210	21.530	1.00	0.00
ATOM	257	HB2	ALA	7	26.840	36.960	21.250	1.00	0.00
ATOM	258	HB3	ALA	7	27.770	35.570	21.330	1.00	0.00
ATOM	259	C	ALA	7	29.070	35.750	19.000	1.00	0.00
ATOM	260	O	ALA	7	28.750	34.630	18.610	1.00	0.00
ATOM	261	N	ILE	8	30.360	36.080	19.070	1.00	0.00
ATOM	262	H	ILE	8	30.920	35.310	18.740	1.00	0.00
ATOM	263	CA	ILE	8	31.040	37.280	19.510	1.00	0.00

ATOM	264	HA	ILE	8	30.450	37.760	20.280	1.00	0.00
ATOM	265	CB	ILE	8	32.390	36.970	20.160	1.00	0.00
ATOM	266	HB	ILE	8	32.160	36.220	20.910	1.00	0.00
ATOM	267	CG2	ILE	8	32.980	38.200	20.860	1.00	0.00
ATOM	268	1HG2	ILE	8	32.210	38.710	21.440	1.00	0.00
ATOM	269	2HG2	ILE	8	33.780	37.910	21.540	1.00	0.00
ATOM	270	3HG2	ILE	8	33.410	38.930	20.180	1.00	0.00
ATOM	271	CG1	ILE	8	33.460	36.510	19.180	1.00	0.00
ATOM	272	1HG1	ILE	8	33.740	37.330	18.510	1.00	0.00
ATOM	273	2HG1	ILE	8	34.390	36.150	19.610	1.00	0.00
ATOM	274	CD	ILE	8	33.150	35.230	18.410	1.00	0.00
ATOM	275	HD1	ILE	8	34.010	34.710	17.990	1.00	0.00
ATOM	276	HD2	ILE	8	32.600	35.500	17.510	1.00	0.00
ATOM	277	HD3	ILE	8	32.530	34.510	18.940	1.00	0.00
ATOM	278	C	ILE	8	31.060	38.280	18.360	1.00	0.00
ATOM	279	O	ILE	8	31.420	39.440	18.570	1.00	0.00
ATOM	280	N	LEU	9	30.710	37.900	17.130	1.00	0.00
ATOM	281	H	LEU	9	30.260	37.000	17.010	1.00	0.00
ATOM	282	CA	LEU	9	30.670	38.790	15.990	1.00	0.00
ATOM	283	HA	LEU	9	30.110	38.320	15.180	1.00	0.00
ATOM	284	CB	LEU	9	32.080	38.950	15.440	1.00	0.00
ATOM	285	HB1	LEU	9	32.640	39.640	16.070	1.00	0.00
ATOM	286	HB2	LEU	9	32.080	39.420	14.460	1.00	0.00
ATOM	287	CG	LEU	9	32.870	37.650	15.350	1.00	0.00
ATOM	288	HG	LEU	9	33.010	37.120	16.300	1.00	0.00
ATOM	289	CD1	LEU	9	34.230	38.000	14.740	1.00	0.00
ATOM	290	1HD1	LEU	9	34.040	38.440	13.760	1.00	0.00
ATOM	291	2HD1	LEU	9	34.720	38.740	15.380	1.00	0.00
ATOM	292	3HD1	LEU	9	34.830	37.110	14.930	1.00	0.00
ATOM	293	CD2	LEU	9	32.220	36.610	14.440	1.00	0.00
ATOM	294	1HD2	LEU	9	32.910	35.780	14.330	1.00	0.00
ATOM	295	2HD2	LEU	9	31.270	36.150	14.730	1.00	0.00
ATOM	296	3HD2	LEU	9	32.030	37.110	13.490	1.00	0.00
ATOM	297	C	LEU	9	29.920	40.120	16.090	1.00	0.00
ATOM	298	O	LEU	9	30.170	41.070	15.350	1.00	0.00
ATOM	299	N	SER	10	28.990	40.230	17.040	1.00	0.00
ATOM	300	H	SER	10	28.810	39.500	17.720	1.00	0.00
ATOM	301	CA	SER	10	28.360	41.510	17.300	1.00	0.00
ATOM	302	HA	SER	10	29.020	42.350	17.140	1.00	0.00
ATOM	303	CB	SER	10	27.250	41.720	16.270	1.00	0.00
ATOM	304	HB1	SER	10	27.490	41.290	15.300	1.00	0.00
ATOM	305	HB2	SER	10	27.000	42.760	16.100	1.00	0.00
ATOM	306	OG	SER	10	26.120	41.050	16.790	1.00	0.00
ATOM	307	HG	SER	10	26.300	40.120	16.920	1.00	0.00

ATOM	308	C	SER	10	27.960	41.610	18.770	1.00	0.00
ATOM	309	O	SER	10	28.140	40.710	19.590	1.00	0.00
ATOM	310	N	SER	11	27.380	42.760	19.100	1.00	0.00
ATOM	311	H	SER	11	27.260	43.460	18.380	1.00	0.00
ATOM	312	CA	SER	11	26.720	43.170	20.320	1.00	0.00
ATOM	313	HA	SER	11	26.490	42.320	20.960	1.00	0.00
ATOM	314	CB	SER	11	27.650	44.170	21.010	1.00	0.00
ATOM	315	HB1	SER	11	28.550	43.600	21.260	1.00	0.00
ATOM	316	HB2	SER	11	27.750	44.980	20.300	1.00	0.00
ATOM	317	OG	SER	11	27.260	44.670	22.270	1.00	0.00
ATOM	318	HG	SER	11	28.060	45.010	22.670	1.00	0.00
ATOM	319	C	SER	11	25.420	43.910	19.990	1.00	0.00
ATOM	320	O	SER	11	25.200	44.290	18.850	1.00	0.00
ATOM	321	N	NH2	12	24.480	44.050	20.920	1.00	0.00
ATOM	322	H1	NH2	12	23.580	44.440	20.680	1.00	0.00
ATOM	323	H2	NH2	12	24.800	43.670	21.800	1.00	0.00
ATOM	324	CH3	ACE	1	23.610	53.320	21.540	1.00	0.00
ATOM	325	1HH3	ACE	1	24.670	53.080	21.470	1.00	0.00
ATOM	326	2HH3	ACE	1	23.300	52.760	22.420	1.00	0.00
ATOM	327	3HH3	ACE	1	23.470	54.360	21.810	1.00	0.00
ATOM	328	C	ACE	1	22.930	52.840	20.260	1.00	0.00
ATOM	329	O	ACE	1	23.220	51.760	19.760	1.00	0.00
ATOM	330	N	SER	2	21.940	53.580	19.770	1.00	0.00
ATOM	331	H	SER	2	21.770	54.430	20.290	1.00	0.00
ATOM	332	CA	SER	2	21.100	53.310	18.610	1.00	0.00
ATOM	333	HA	SER	2	21.500	52.420	18.120	1.00	0.00
ATOM	334	CB	SER	2	21.250	54.490	17.660	1.00	0.00
ATOM	335	HB1	SER	2	20.910	55.420	18.110	1.00	0.00
ATOM	336	HB2	SER	2	20.570	54.210	16.850	1.00	0.00
ATOM	337	OG	SER	2	22.550	54.790	17.180	1.00	0.00
ATOM	338	HG	SER	2	22.440	55.290	16.370	1.00	0.00
ATOM	339	C	SER	2	19.620	53.060	18.840	1.00	0.00
ATOM	340	O	SER	2	18.930	53.870	19.460	1.00	0.00
ATOM	341	N	ASN	3	19.090	51.850	18.630	1.00	0.00
ATOM	342	H	ASN	3	18.100	51.890	18.800	1.00	0.00
ATOM	343	CA	ASN	3	19.700	50.620	18.160	1.00	0.00
ATOM	344	HA	ASN	3	20.590	50.860	17.570	1.00	0.00
ATOM	345	CB	ASN	3	18.750	50.100	17.090	1.00	0.00
ATOM	346	HB1	ASN	3	17.830	49.690	17.500	1.00	0.00
ATOM	347	HB2	ASN	3	19.210	49.200	16.680	1.00	0.00
ATOM	348	CG	ASN	3	18.450	51.130	16.000	1.00	0.00
ATOM	349	OD1	ASN	3	19.280	51.460	15.160	1.00	0.00
ATOM	350	ND2	ASN	3	17.360	51.890	16.070	1.00	0.00
ATOM	351	1HD2	ASN	3	17.330	52.720	15.500	1.00	0.00

ATOM	352	2HD2	ASN	3	16.610	51.630	16.700	1.00	0.00
ATOM	353	C	ASN	3	20.050	49.640	19.270	1.00	0.00
ATOM	354	O	ASN	3	19.220	48.840	19.690	1.00	0.00
ATOM	355	N	ASN	4	21.260	49.750	19.830	1.00	0.00
ATOM	356	H	ASN	4	21.940	50.400	19.470	1.00	0.00
ATOM	357	CA	ASN	4	21.700	48.900	20.920	1.00	0.00
ATOM	358	HA	ASN	4	20.890	48.220	21.170	1.00	0.00
ATOM	359	CB	ASN	4	22.060	49.670	22.200	1.00	0.00
ATOM	360	HB1	ASN	4	22.740	50.480	21.950	1.00	0.00
ATOM	361	HB2	ASN	4	22.450	48.990	22.950	1.00	0.00
ATOM	362	CG	ASN	4	20.840	50.380	22.770	1.00	0.00
ATOM	363	OD1	ASN	4	20.720	51.600	22.750	1.00	0.00
ATOM	364	ND2	ASN	4	19.800	49.660	23.200	1.00	0.00
ATOM	365	1HD2	ASN	4	19.850	48.660	23.270	1.00	0.00
ATOM	366	2HD2	ASN	4	18.990	50.160	23.550	1.00	0.00
ATOM	367	C	ASN	4	22.760	47.870	20.570	1.00	0.00
ATOM	368	O	ASN	4	22.490	46.680	20.500	1.00	0.00
ATOM	369	N	PHE	5	23.810	48.450	19.980	1.00	0.00
ATOM	370	H	PHE	5	23.800	49.450	19.860	1.00	0.00
ATOM	371	CA	PHE	5	25.000	47.710	19.590	1.00	0.00
ATOM	372	HA	PHE	5	24.660	46.690	19.410	1.00	0.00
ATOM	373	CB	PHE	5	26.000	47.710	20.740	1.00	0.00
ATOM	374	HB1	PHE	5	25.630	47.060	21.530	1.00	0.00
ATOM	375	HB2	PHE	5	26.960	47.310	20.410	1.00	0.00
ATOM	376	CG	PHE	5	26.320	49.080	21.300	1.00	0.00
ATOM	377	CD1	PHE	5	25.640	49.560	22.420	1.00	0.00
ATOM	378	HD1	PHE	5	24.750	49.050	22.770	1.00	0.00
ATOM	379	CE1	PHE	5	26.060	50.670	23.170	1.00	0.00
ATOM	380	HE1	PHE	5	25.610	51.000	24.090	1.00	0.00
ATOM	381	CZ	PHE	5	27.120	51.420	22.650	1.00	0.00
ATOM	382	HZ	PHE	5	27.490	52.280	23.190	1.00	0.00
ATOM	383	CE2	PHE	5	27.740	51.040	21.460	1.00	0.00
ATOM	384	HE2	PHE	5	28.620	51.620	21.200	1.00	0.00
ATOM	385	CD2	PHE	5	27.280	49.930	20.750	1.00	0.00
ATOM	386	HD2	PHE	5	27.890	49.530	19.950	1.00	0.00
ATOM	387	C	PHE	5	25.650	48.040	18.250	1.00	0.00
ATOM	388	O	PHE	5	25.490	49.110	17.670	1.00	0.00
ATOM	389	N	GLY	6	26.480	47.150	17.710	1.00	0.00
ATOM	390	H	GLY	6	26.460	46.310	18.260	1.00	0.00
ATOM	391	CA	GLY	6	27.060	47.110	16.380	1.00	0.00
ATOM	392	HA1	GLY	6	27.500	48.090	16.230	1.00	0.00
ATOM	393	HA2	GLY	6	26.310	46.940	15.610	1.00	0.00
ATOM	394	C	GLY	6	28.120	46.010	16.260	1.00	0.00
ATOM	395	O	GLY	6	28.100	45.110	17.090	1.00	0.00

ATOM	396	N	ALA	7	28.970	46.020	15.230	1.00	0.00
ATOM	397	H	ALA	7	28.830	46.810	14.610	1.00	0.00
ATOM	398	CA	ALA	7	29.870	44.950	14.870	1.00	0.00
ATOM	399	HA	ALA	7	30.240	44.330	15.690	1.00	0.00
ATOM	400	CB	ALA	7	29.110	44.030	13.920	1.00	0.00
ATOM	401	HB1	ALA	7	29.670	43.100	13.830	1.00	0.00
ATOM	402	HB2	ALA	7	28.210	43.720	14.460	1.00	0.00
ATOM	403	HB3	ALA	7	29.030	44.480	12.930	1.00	0.00
ATOM	404	C	ALA	7	31.120	45.490	14.210	1.00	0.00
ATOM	405	O	ALA	7	31.330	46.700	14.150	1.00	0.00
ATOM	406	N	ILE	8	32.050	44.580	13.900	1.00	0.00
ATOM	407	H	ILE	8	31.810	43.630	14.120	1.00	0.00
ATOM	408	CA	ILE	8	33.300	44.920	13.240	1.00	0.00
ATOM	409	HA	ILE	8	33.920	45.580	13.840	1.00	0.00
ATOM	410	CB	ILE	8	34.230	43.730	13.000	1.00	0.00
ATOM	411	HB	ILE	8	33.640	43.050	12.390	1.00	0.00
ATOM	412	CG2	ILE	8	35.500	44.240	12.340	1.00	0.00
ATOM	413	1HG2	ILE	8	35.260	44.690	11.370	1.00	0.00
ATOM	414	2HG2	ILE	8	36.080	43.350	12.080	1.00	0.00
ATOM	415	3HG2	ILE	8	36.120	44.850	13.010	1.00	0.00
ATOM	416	CG1	ILE	8	34.570	43.000	14.300	1.00	0.00
ATOM	417	1HG1	ILE	8	34.940	43.720	15.030	1.00	0.00
ATOM	418	2HG1	ILE	8	33.660	42.550	14.690	1.00	0.00
ATOM	419	CD	ILE	8	35.660	41.930	14.190	1.00	0.00
ATOM	420	HD1	ILE	8	36.640	42.310	13.890	1.00	0.00
ATOM	421	HD2	ILE	8	35.300	41.100	13.590	1.00	0.00
ATOM	422	HD3	ILE	8	35.810	41.500	15.180	1.00	0.00
ATOM	423	C	ILE	8	32.890	45.640	11.960	1.00	0.00
ATOM	424	O	ILE	8	32.210	45.050	11.140	1.00	0.00
ATOM	425	N	LEU	9	33.410	46.860	11.810	1.00	0.00
ATOM	426	H	LEU	9	33.720	47.260	12.680	1.00	0.00
ATOM	427	CA	LEU	9	33.470	47.700	10.620	1.00	0.00
ATOM	428	HA	LEU	9	33.720	47.140	9.720	1.00	0.00
ATOM	429	CB	LEU	9	34.460	48.820	10.950	1.00	0.00
ATOM	430	HB1	LEU	9	34.110	49.500	11.730	1.00	0.00
ATOM	431	HB2	LEU	9	34.520	49.510	10.110	1.00	0.00
ATOM	432	CG	LEU	9	35.870	48.400	11.340	1.00	0.00
ATOM	433	HG	LEU	9	35.850	47.800	12.250	1.00	0.00
ATOM	434	CD1	LEU	9	36.800	49.580	11.620	1.00	0.00
ATOM	435	1HD1	LEU	9	36.440	50.070	12.530	1.00	0.00
ATOM	436	2HD1	LEU	9	37.810	49.220	11.790	1.00	0.00
ATOM	437	3HD1	LEU	9	36.840	50.200	10.720	1.00	0.00
ATOM	438	CD2	LEU	9	36.500	47.480	10.290	1.00	0.00
ATOM	439	1HD2	LEU	9	36.040	46.490	10.240	1.00	0.00

ATOM	440	2HD2	LEU	9	36.280	47.860	9.300	1.00	0.00
ATOM	441	3HD2	LEU	9	37.560	47.380	10.490	1.00	0.00
ATOM	442	C	LEU	9	32.150	48.380	10.270	1.00	0.00
ATOM	443	O	LEU	9	32.110	48.900	9.150	1.00	0.00
ATOM	444	N	SER	10	31.100	48.340	11.090	1.00	0.00
ATOM	445	H	SER	10	31.190	47.880	11.980	1.00	0.00
ATOM	446	CA	SER	10	29.790	48.850	10.760	1.00	0.00
ATOM	447	HA	SER	10	29.490	48.760	9.710	1.00	0.00
ATOM	448	CB	SER	10	29.630	50.350	11.010	1.00	0.00
ATOM	449	HB1	SER	10	28.600	50.610	10.740	1.00	0.00
ATOM	450	HB2	SER	10	30.480	50.870	10.580	1.00	0.00
ATOM	451	OG	SER	10	29.700	50.630	12.390	1.00	0.00
ATOM	452	HG	SER	10	30.430	50.130	12.760	1.00	0.00
ATOM	453	C	SER	10	28.710	48.160	11.580	1.00	0.00
ATOM	454	O	SER	10	28.820	47.720	12.720	1.00	0.00
ATOM	455	N	SER	11	27.550	48.100	10.910	1.00	0.00
ATOM	456	H	SER	11	27.420	48.520	10.000	1.00	0.00
ATOM	457	CA	SER	11	26.350	47.360	11.260	1.00	0.00
ATOM	458	HA	SER	11	26.510	46.720	12.130	1.00	0.00
ATOM	459	CB	SER	11	25.190	48.310	11.530	1.00	0.00
ATOM	460	HB1	SER	11	24.950	49.030	10.750	1.00	0.00
ATOM	461	HB2	SER	11	24.380	47.610	11.720	1.00	0.00
ATOM	462	OG	SER	11	25.460	49.010	12.730	1.00	0.00
ATOM	463	HG	SER	11	25.850	49.890	12.680	1.00	0.00
ATOM	464	C	SER	11	25.970	46.270	10.270	1.00	0.00
ATOM	465	O	SER	11	25.770	46.530	9.090	1.00	0.00
ATOM	466	N	NH2	12	25.840	45.030	10.750	1.00	0.00
ATOM	467	H1	NH2	12	25.480	44.270	10.180	1.00	0.00
ATOM	468	H2	NH2	12	25.970	44.770	11.720	1.00	0.00
ATOM	469	CH3	ACE	1	16.780	0.790	28.840	1.00	0.00
ATOM	470	1HH3	ACE	1	15.850	1.360	28.820	1.00	0.00
ATOM	471	2HH3	ACE	1	17.310	0.930	27.900	1.00	0.00
ATOM	472	3HH3	ACE	1	17.310	1.320	29.630	1.00	0.00
ATOM	473	C	ACE	1	16.600	-0.660	29.260	1.00	0.00
ATOM	474	O	ACE	1	15.640	-1.270	28.810	1.00	0.00
ATOM	475	N	SER	2	17.470	-1.170	30.140	1.00	0.00
ATOM	476	H	SER	2	18.080	-0.560	30.660	1.00	0.00
ATOM	477	CA	SER	2	17.410	-2.540	30.600	1.00	0.00
ATOM	478	HA	SER	2	17.000	-3.090	29.750	1.00	0.00
ATOM	479	CB	SER	2	16.410	-2.540	31.750	1.00	0.00
ATOM	480	HB1	SER	2	16.370	-3.500	32.280	1.00	0.00
ATOM	481	HB2	SER	2	15.410	-2.290	31.400	1.00	0.00
ATOM	482	OG	SER	2	16.740	-1.550	32.700	1.00	0.00
ATOM	483	HG	SER	2	16.950	-2.040	33.500	1.00	0.00

ATOM	484	C	SER	2	18.780	-3.160	30.820	1.00	0.00
ATOM	485	O	SER	2	19.290	-3.460	31.900	1.00	0.00
ATOM	486	N	ASN	3	19.380	-3.340	29.640	1.00	0.00
ATOM	487	H	ASN	3	18.820	-3.080	28.840	1.00	0.00
ATOM	488	CA	ASN	3	20.800	-3.580	29.520	1.00	0.00
ATOM	489	HA	ASN	3	21.310	-2.760	30.030	1.00	0.00
ATOM	490	CB	ASN	3	21.250	-3.490	28.070	1.00	0.00
ATOM	491	HB1	ASN	3	22.340	-3.470	28.030	1.00	0.00
ATOM	492	HB2	ASN	3	20.880	-4.330	27.480	1.00	0.00
ATOM	493	CG	ASN	3	20.760	-2.180	27.460	1.00	0.00
ATOM	494	OD1	ASN	3	21.280	-1.080	27.580	1.00	0.00
ATOM	495	ND2	ASN	3	19.570	-2.240	26.850	1.00	0.00
ATOM	496	1HD2	ASN	3	19.240	-1.440	26.330	1.00	0.00
ATOM	497	2HD2	ASN	3	19.150	-3.160	26.790	1.00	0.00
ATOM	498	C	ASN	3	21.240	-4.940	30.050	1.00	0.00
ATOM	499	O	ASN	3	20.470	-5.890	29.900	1.00	0.00
ATOM	500	N	ASN	4	22.410	-5.010	30.690	1.00	0.00
ATOM	501	H	ASN	4	23.090	-4.260	30.660	1.00	0.00
ATOM	502	CA	ASN	4	22.860	-6.200	31.370	1.00	0.00
ATOM	503	HA	ASN	4	22.030	-6.790	31.760	1.00	0.00
ATOM	504	CB	ASN	4	23.660	-5.820	32.620	1.00	0.00
ATOM	505	HB1	ASN	4	23.000	-5.280	33.290	1.00	0.00
ATOM	506	HB2	ASN	4	24.500	-5.180	32.370	1.00	0.00
ATOM	507	CG	ASN	4	24.310	-6.990	33.330	1.00	0.00
ATOM	508	OD1	ASN	4	24.710	-7.960	32.700	1.00	0.00
ATOM	509	ND2	ASN	4	24.250	-7.060	34.670	1.00	0.00
ATOM	510	1HD2	ASN	4	23.850	-6.290	35.180	1.00	0.00
ATOM	511	2HD2	ASN	4	24.880	-7.680	35.170	1.00	0.00
ATOM	512	C	ASN	4	23.630	-7.060	30.370	1.00	0.00
ATOM	513	O	ASN	4	24.550	-6.670	29.660	1.00	0.00
ATOM	514	N	PHE	5	23.110	-8.270	30.170	1.00	0.00
ATOM	515	H	PHE	5	22.240	-8.460	30.650	1.00	0.00
ATOM	516	CA	PHE	5	23.760	-9.240	29.310	1.00	0.00
ATOM	517	HA	PHE	5	24.700	-8.860	28.900	1.00	0.00
ATOM	518	CB	PHE	5	22.830	-9.530	28.140	1.00	0.00
ATOM	519	HB1	PHE	5	23.240	-10.250	27.440	1.00	0.00
ATOM	520	HB2	PHE	5	21.910	-9.810	28.660	1.00	0.00
ATOM	521	CG	PHE	5	22.490	-8.270	27.380	1.00	0.00
ATOM	522	CD1	PHE	5	21.190	-7.750	27.360	1.00	0.00
ATOM	523	HD1	PHE	5	20.450	-8.160	28.030	1.00	0.00
ATOM	524	CE1	PHE	5	20.920	-6.660	26.530	1.00	0.00
ATOM	525	HE1	PHE	5	19.930	-6.240	26.370	1.00	0.00
ATOM	526	CZ	PHE	5	21.950	-5.960	25.880	1.00	0.00
ATOM	527	HZ	PHE	5	21.780	-5.050	25.330	1.00	0.00

ATOM	528	CE2	PHE	5	23.270	-6.430	25.940	1.00	0.00
ATOM	529	HE2	PHE	5	24.100	-5.910	25.490	1.00	0.00
ATOM	530	CD2	PHE	5	23.490	-7.630	26.620	1.00	0.00
ATOM	531	HD2	PHE	5	24.510	-7.950	26.750	1.00	0.00
ATOM	532	C	PHE	5	24.310	-10.480	29.990	1.00	0.00
ATOM	533	O	PHE	5	24.670	-11.420	29.280	1.00	0.00
ATOM	534	N	GLY	6	24.260	-10.500	31.330	1.00	0.00
ATOM	535	H	GLY	6	23.850	-9.720	31.810	1.00	0.00
ATOM	536	CA	GLY	6	24.760	-11.580	32.170	1.00	0.00
ATOM	537	HA1	GLY	6	25.840	-11.540	32.040	1.00	0.00
ATOM	538	HA2	GLY	6	24.400	-12.490	31.690	1.00	0.00
ATOM	539	C	GLY	6	24.400	-11.700	33.640	1.00	0.00
ATOM	540	O	GLY	6	25.170	-12.260	34.420	1.00	0.00
ATOM	541	N	ALA	7	23.320	-11.050	34.090	1.00	0.00
ATOM	542	H	ALA	7	22.780	-10.550	33.400	1.00	0.00
ATOM	543	CA	ALA	7	22.680	-11.260	35.370	1.00	0.00
ATOM	544	HA	ALA	7	22.870	-12.310	35.620	1.00	0.00
ATOM	545	CB	ALA	7	23.180	-10.270	36.420	1.00	0.00
ATOM	546	HB1	ALA	7	22.940	-9.230	36.210	1.00	0.00
ATOM	547	HB2	ALA	7	22.760	-10.370	37.420	1.00	0.00
ATOM	548	HB3	ALA	7	24.250	-10.370	36.590	1.00	0.00
ATOM	549	C	ALA	7	21.190	-11.130	35.130	1.00	0.00
ATOM	550	O	ALA	7	20.750	-10.360	34.280	1.00	0.00
ATOM	551	N	ILE	8	20.420	-12.000	35.790	1.00	0.00
ATOM	552	H	ILE	8	20.800	-12.650	36.470	1.00	0.00
ATOM	553	CA	ILE	8	18.990	-12.130	35.640	1.00	0.00
ATOM	554	HA	ILE	8	18.870	-12.360	34.580	1.00	0.00
ATOM	555	CB	ILE	8	18.570	-13.330	36.480	1.00	0.00
ATOM	556	HB	ILE	8	19.100	-14.150	36.010	1.00	0.00
ATOM	557	CG2	ILE	8	19.160	-13.240	37.890	1.00	0.00
ATOM	558	1HG2	ILE	8	20.190	-13.570	37.920	1.00	0.00
ATOM	559	2HG2	ILE	8	18.680	-13.960	38.550	1.00	0.00
ATOM	560	3HG2	ILE	8	19.110	-12.270	38.380	1.00	0.00
ATOM	561	CG1	ILE	8	17.060	-13.490	36.380	1.00	0.00
ATOM	562	1HG1	ILE	8	16.640	-12.690	37.000	1.00	0.00
ATOM	563	2HG1	ILE	8	16.730	-13.310	35.350	1.00	0.00
ATOM	564	CD	ILE	8	16.590	-14.900	36.730	1.00	0.00
ATOM	565	HD1	ILE	8	15.510	-15.000	36.580	1.00	0.00
ATOM	566	HD2	ILE	8	16.850	-15.100	37.770	1.00	0.00
ATOM	567	HD3	ILE	8	16.970	-15.630	36.020	1.00	0.00
ATOM	568	C	ILE	8	18.310	-10.810	36.000	1.00	0.00
ATOM	569	O	ILE	8	18.520	-10.290	37.090	1.00	0.00
ATOM	570	N	LEU	9	17.430	-10.370	35.100	1.00	0.00
ATOM	571	H	LEU	9	17.340	-10.890	34.240	1.00	0.00

ATOM	572	CA	LEU	9	16.710	-9.120	35.130	1.00	0.00
ATOM	573	HA	LEU	9	16.600	-8.880	34.070	1.00	0.00
ATOM	574	CB	LEU	9	15.400	-9.340	35.890	1.00	0.00
ATOM	575	HB1	LEU	9	14.900	-10.190	35.440	1.00	0.00
ATOM	576	HB2	LEU	9	15.760	-9.720	36.840	1.00	0.00
ATOM	577	CG	LEU	9	14.370	-8.290	36.310	1.00	0.00
ATOM	578	HG	LEU	9	14.810	-7.460	36.870	1.00	0.00
ATOM	579	CD1	LEU	9	13.730	-7.720	35.050	1.00	0.00
ATOM	580	1HD1	LEU	9	14.430	-7.060	34.530	1.00	0.00
ATOM	581	2HD1	LEU	9	13.410	-8.580	34.450	1.00	0.00
ATOM	582	3HD1	LEU	9	12.910	-7.110	35.440	1.00	0.00
ATOM	583	CD2	LEU	9	13.220	-8.890	37.110	1.00	0.00
ATOM	584	1HD2	LEU	9	12.730	-8.130	37.720	1.00	0.00
ATOM	585	2HD2	LEU	9	13.410	-9.660	37.860	1.00	0.00
ATOM	586	3HD2	LEU	9	12.400	-9.190	36.460	1.00	0.00
ATOM	587	C	LEU	9	17.590	-7.940	35.530	1.00	0.00
ATOM	588	O	LEU	9	17.200	-6.910	36.070	1.00	0.00
ATOM	589	N	SER	10	18.870	-8.090	35.190	1.00	0.00
ATOM	590	H	SER	10	19.030	-8.990	34.770	1.00	0.00
ATOM	591	CA	SER	10	19.920	-7.100	35.340	1.00	0.00
ATOM	592	HA	SER	10	20.870	-7.530	35.040	1.00	0.00
ATOM	593	CB	SER	10	19.690	-5.950	34.360	1.00	0.00
ATOM	594	HB1	SER	10	20.480	-5.210	34.440	1.00	0.00
ATOM	595	HB2	SER	10	18.750	-5.460	34.610	1.00	0.00
ATOM	596	OG	SER	10	19.700	-6.420	33.030	1.00	0.00
ATOM	597	HG	SER	10	19.580	-5.770	32.340	1.00	0.00
ATOM	598	C	SER	10	20.380	-6.690	36.740	1.00	0.00
ATOM	599	O	SER	10	20.900	-5.590	36.890	1.00	0.00
ATOM	600	N	SER	11	20.250	-7.530	37.770	1.00	0.00
ATOM	601	H	SER	11	19.800	-8.400	37.510	1.00	0.00
ATOM	602	CA	SER	11	20.550	-7.420	39.180	1.00	0.00
ATOM	603	HA	SER	11	20.020	-6.530	39.520	1.00	0.00
ATOM	604	CB	SER	11	19.900	-8.580	39.920	1.00	0.00
ATOM	605	HB1	SER	11	20.240	-8.630	40.960	1.00	0.00
ATOM	606	HB2	SER	11	18.810	-8.470	39.960	1.00	0.00
ATOM	607	OG	SER	11	20.200	-9.790	39.260	1.00	0.00
ATOM	608	HG	SER	11	19.510	-9.960	38.620	1.00	0.00
ATOM	609	C	SER	11	22.040	-7.310	39.500	1.00	0.00
ATOM	610	O	SER	11	22.410	-6.830	40.570	1.00	0.00
ATOM	611	N	NH2	12	22.850	-7.720	38.530	1.00	0.00
ATOM	612	H1	NH2	12	23.840	-7.530	38.620	1.00	0.00
ATOM	613	H2	NH2	12	22.560	-8.260	37.720	1.00	0.00
ATOM	614	CH3	ACE	1	21.960	2.890	19.310	1.00	0.00
ATOM	615	1HH3	ACE	1	22.650	3.730	19.370	1.00	0.00

ATOM	616	2HH3	ACE	1	21.210	3.030	20.090	1.00	0.00
ATOM	617	3HH3	ACE	1	21.570	2.800	18.300	1.00	0.00
ATOM	618	C	ACE	1	22.740	1.620	19.620	1.00	0.00
ATOM	619	O	ACE	1	22.240	0.690	20.270	1.00	0.00
ATOM	620	N	SER	2	23.920	1.540	19.010	1.00	0.00
ATOM	621	H	SER	2	24.180	2.310	18.410	1.00	0.00
ATOM	622	CA	SER	2	24.860	0.470	19.250	1.00	0.00
ATOM	623	HA	SER	2	24.270	-0.430	19.380	1.00	0.00
ATOM	624	CB	SER	2	25.700	0.730	20.500	1.00	0.00
ATOM	625	HB1	SER	2	25.000	0.870	21.320	1.00	0.00
ATOM	626	HB2	SER	2	26.290	1.640	20.480	1.00	0.00
ATOM	627	OG	SER	2	26.550	-0.370	20.750	1.00	0.00
ATOM	628	HG	SER	2	27.180	-0.120	21.430	1.00	0.00
ATOM	629	C	SER	2	25.730	0.260	18.020	1.00	0.00
ATOM	630	O	SER	2	26.180	1.300	17.540	1.00	0.00
ATOM	631	N	ASN	3	26.000	-0.930	17.480	1.00	0.00
ATOM	632	H	ASN	3	26.220	-0.790	16.510	1.00	0.00
ATOM	633	CA	ASN	3	25.450	-2.230	17.830	1.00	0.00
ATOM	634	HA	ASN	3	24.530	-2.150	18.390	1.00	0.00
ATOM	635	CB	ASN	3	26.460	-3.070	18.610	1.00	0.00
ATOM	636	HB1	ASN	3	26.890	-2.600	19.500	1.00	0.00
ATOM	637	HB2	ASN	3	25.960	-3.990	18.880	1.00	0.00
ATOM	638	CG	ASN	3	27.620	-3.620	17.800	1.00	0.00
ATOM	639	OD1	ASN	3	28.720	-3.060	17.800	1.00	0.00
ATOM	640	ND2	ASN	3	27.460	-4.770	17.130	1.00	0.00
ATOM	641	1HD2	ASN	3	28.240	-5.320	16.790	1.00	0.00
ATOM	642	2HD2	ASN	3	26.620	-5.330	17.150	1.00	0.00
ATOM	643	C	ASN	3	25.120	-3.000	16.550	1.00	0.00
ATOM	644	O	ASN	3	25.680	-2.730	15.490	1.00	0.00
ATOM	645	N	ASN	4	24.140	-3.880	16.710	1.00	0.00
ATOM	646	H	ASN	4	23.850	-4.290	17.590	1.00	0.00
ATOM	647	CA	ASN	4	23.700	-4.670	15.570	1.00	0.00
ATOM	648	HA	ASN	4	24.580	-4.900	14.970	1.00	0.00
ATOM	649	CB	ASN	4	22.630	-3.880	14.830	1.00	0.00
ATOM	650	HB1	ASN	4	23.010	-2.880	14.620	1.00	0.00
ATOM	651	HB2	ASN	4	21.760	-3.860	15.500	1.00	0.00
ATOM	652	CG	ASN	4	22.350	-4.660	13.550	1.00	0.00
ATOM	653	OD1	ASN	4	23.180	-4.810	12.650	1.00	0.00
ATOM	654	ND2	ASN	4	21.150	-5.190	13.330	1.00	0.00
ATOM	655	1HD2	ASN	4	20.430	-5.090	14.040	1.00	0.00
ATOM	656	2HD2	ASN	4	21.050	-5.750	12.500	1.00	0.00
ATOM	657	C	ASN	4	23.150	-5.980	16.090	1.00	0.00
ATOM	658	O	ASN	4	22.420	-6.010	17.090	1.00	0.00
ATOM	659	N	PHE	5	23.430	-7.130	15.470	1.00	0.00

ATOM	660	H	PHE	5	24.010	-7.080	14.650	1.00	0.00
ATOM	661	CA	PHE	5	22.810	-8.400	15.800	1.00	0.00
ATOM	662	HA	PHE	5	23.070	-8.610	16.840	1.00	0.00
ATOM	663	CB	PHE	5	23.520	-9.350	14.840	1.00	0.00
ATOM	664	HB1	PHE	5	23.240	-9.160	13.810	1.00	0.00
ATOM	665	HB2	PHE	5	24.560	-9.190	15.120	1.00	0.00
ATOM	666	CG	PHE	5	23.210	-10.800	15.100	1.00	0.00
ATOM	667	CD1	PHE	5	22.480	-11.560	14.170	1.00	0.00
ATOM	668	HD1	PHE	5	22.260	-11.250	13.160	1.00	0.00
ATOM	669	CE1	PHE	5	22.220	-12.860	14.590	1.00	0.00
ATOM	670	HE1	PHE	5	21.450	-13.450	14.110	1.00	0.00
ATOM	671	CZ	PHE	5	22.820	-13.440	15.720	1.00	0.00
ATOM	672	HZ	PHE	5	22.670	-14.490	15.900	1.00	0.00
ATOM	673	CE2	PHE	5	23.530	-12.680	16.650	1.00	0.00
ATOM	674	HE2	PHE	5	24.070	-13.100	17.480	1.00	0.00
ATOM	675	CD2	PHE	5	23.630	-11.320	16.340	1.00	0.00
ATOM	676	HD2	PHE	5	24.300	-10.670	16.890	1.00	0.00
ATOM	677	C	PHE	5	21.310	-8.460	15.550	1.00	0.00
ATOM	678	O	PHE	5	20.870	-7.870	14.560	1.00	0.00
ATOM	679	N	GLY	6	20.450	-9.200	16.250	1.00	0.00
ATOM	680	H	GLY	6	19.560	-9.390	15.820	1.00	0.00
ATOM	681	CA	GLY	6	20.730	-9.810	17.540	1.00	0.00
ATOM	682	HA1	GLY	6	20.270	-9.230	18.340	1.00	0.00
ATOM	683	HA2	GLY	6	21.780	-9.940	17.830	1.00	0.00
ATOM	684	C	GLY	6	20.040	-11.160	17.570	1.00	0.00
ATOM	685	O	GLY	6	19.920	-11.780	16.510	1.00	0.00
ATOM	686	N	ALA	7	19.600	-11.650	18.730	1.00	0.00
ATOM	687	H	ALA	7	19.270	-12.600	18.700	1.00	0.00
ATOM	688	CA	ALA	7	19.700	-11.110	20.080	1.00	0.00
ATOM	689	HA	ALA	7	20.580	-10.470	20.130	1.00	0.00
ATOM	690	CB	ALA	7	18.420	-10.310	20.300	1.00	0.00
ATOM	691	HB1	ALA	7	17.600	-11.030	20.300	1.00	0.00
ATOM	692	HB2	ALA	7	18.330	-9.580	19.490	1.00	0.00
ATOM	693	HB3	ALA	7	18.350	-9.800	21.260	1.00	0.00
ATOM	694	C	ALA	7	19.910	-12.270	21.030	1.00	0.00
ATOM	695	O	ALA	7	19.500	-13.400	20.760	1.00	0.00
ATOM	696	N	ILE	8	20.370	-12.100	22.280	1.00	0.00
ATOM	697	H	ILE	8	20.280	-12.940	22.830	1.00	0.00
ATOM	698	CA	ILE	8	20.960	-10.910	22.860	1.00	0.00
ATOM	699	HA	ILE	8	21.420	-10.310	22.080	1.00	0.00
ATOM	700	CB	ILE	8	22.150	-11.290	23.730	1.00	0.00
ATOM	701	HB	ILE	8	22.700	-12.000	23.120	1.00	0.00
ATOM	702	CG2	ILE	8	21.880	-12.030	25.040	1.00	0.00
ATOM	703	1HG2	ILE	8	21.300	-12.930	24.880	1.00	0.00

ATOM	704	2HG2	ILE	8	21.320	-11.400	25.740	1.00	0.00
ATOM	705	3HG2	ILE	8	22.800	-12.210	25.590	1.00	0.00
ATOM	706	CG1	ILE	8	22.960	-10.040	24.110	1.00	0.00
ATOM	707	1HG1	ILE	8	22.830	-9.350	23.280	1.00	0.00
ATOM	708	2HG1	ILE	8	22.440	-9.590	24.950	1.00	0.00
ATOM	709	CD	ILE	8	24.430	-10.210	24.460	1.00	0.00
ATOM	710	HD1	ILE	8	24.970	-9.380	24.000	1.00	0.00
ATOM	711	HD2	ILE	8	24.770	-11.200	24.160	1.00	0.00
ATOM	712	HD3	ILE	8	24.650	-10.090	25.520	1.00	0.00
ATOM	713	C	ILE	8	20.020	-10.040	23.690	1.00	0.00
ATOM	714	O	ILE	8	20.260	-8.840	23.740	1.00	0.00
ATOM	715	N	LEU	9	18.940	-10.600	24.240	1.00	0.00
ATOM	716	H	LEU	9	18.850	-11.590	24.100	1.00	0.00
ATOM	717	CA	LEU	9	18.000	-9.930	25.120	1.00	0.00
ATOM	718	HA	LEU	9	18.560	-9.200	25.690	1.00	0.00
ATOM	719	CB	LEU	9	17.440	-11.050	25.990	1.00	0.00
ATOM	720	HB1	LEU	9	16.940	-11.840	25.440	1.00	0.00
ATOM	721	HB2	LEU	9	16.690	-10.510	26.570	1.00	0.00
ATOM	722	CG	LEU	9	18.420	-11.740	26.940	1.00	0.00
ATOM	723	HG	LEU	9	19.080	-12.440	26.430	1.00	0.00
ATOM	724	CD1	LEU	9	17.650	-12.660	27.890	1.00	0.00
ATOM	725	1HD1	LEU	9	16.980	-13.260	27.280	1.00	0.00
ATOM	726	2HD1	LEU	9	18.320	-13.360	28.390	1.00	0.00
ATOM	727	3HD1	LEU	9	17.110	-11.990	28.560	1.00	0.00
ATOM	728	CD2	LEU	9	19.140	-10.730	27.830	1.00	0.00
ATOM	729	1HD2	LEU	9	19.790	-10.190	27.150	1.00	0.00
ATOM	730	2HD2	LEU	9	18.410	-10.270	28.500	1.00	0.00
ATOM	731	3HD2	LEU	9	19.860	-11.270	28.440	1.00	0.00
ATOM	732	C	LEU	9	16.880	-9.220	24.360	1.00	0.00
ATOM	733	O	LEU	9	16.320	-9.760	23.420	1.00	0.00
ATOM	734	N	SER	10	16.590	-8.020	24.870	1.00	0.00
ATOM	735	H	SER	10	17.110	-7.720	25.680	1.00	0.00
ATOM	736	CA	SER	10	15.620	-7.060	24.380	1.00	0.00
ATOM	737	HA	SER	10	15.030	-7.450	23.560	1.00	0.00
ATOM	738	CB	SER	10	14.540	-6.890	25.440	1.00	0.00
ATOM	739	HB1	SER	10	14.870	-6.370	26.340	1.00	0.00
ATOM	740	HB2	SER	10	13.730	-6.310	24.990	1.00	0.00
ATOM	741	OG	SER	10	13.960	-8.110	25.830	1.00	0.00
ATOM	742	HG	SER	10	14.200	-8.220	26.750	1.00	0.00
ATOM	743	C	SER	10	16.260	-5.780	23.840	1.00	0.00
ATOM	744	O	SER	10	17.270	-5.450	24.470	1.00	0.00
ATOM	745	N	SER	11	15.730	-5.010	22.890	1.00	0.00
ATOM	746	H	SER	11	14.890	-5.390	22.480	1.00	0.00
ATOM	747	CA	SER	11	16.190	-3.680	22.530	1.00	0.00

ATOM	748	HA	SER	11	16.460	-3.130	23.430	1.00	0.00
ATOM	749	CB	SER	11	17.420	-3.750	21.640	1.00	0.00
ATOM	750	HB1	SER	11	17.820	-2.740	21.510	1.00	0.00
ATOM	751	HB2	SER	11	18.250	-4.230	22.150	1.00	0.00
ATOM	752	OG	SER	11	17.020	-4.420	20.460	1.00	0.00
ATOM	753	HG	SER	11	16.820	-5.350	20.580	1.00	0.00
ATOM	754	C	SER	11	15.090	-2.820	21.930	1.00	0.00
ATOM	755	O	SER	11	14.060	-3.310	21.460	1.00	0.00
ATOM	756	N	NH2	12	15.340	-1.510	21.910	1.00	0.00
ATOM	757	H1	NH2	12	14.610	-0.930	21.540	1.00	0.00
ATOM	758	H2	NH2	12	16.130	-1.060	22.370	1.00	0.00
ATOM	759	CH3	ACE	1	36.540	53.850	27.200	1.00	0.00
ATOM	760	1HH3	ACE	1	37.260	54.510	27.690	1.00	0.00
ATOM	761	2HH3	ACE	1	36.980	52.860	27.060	1.00	0.00
ATOM	762	3HH3	ACE	1	35.690	53.680	27.860	1.00	0.00
ATOM	763	C	ACE	1	36.050	54.550	25.940	1.00	0.00
ATOM	764	O	ACE	1	36.410	55.700	25.680	1.00	0.00
ATOM	765	N	SER	2	35.320	53.830	25.090	1.00	0.00
ATOM	766	H	SER	2	35.400	52.830	25.060	1.00	0.00
ATOM	767	CA	SER	2	34.690	54.310	23.870	1.00	0.00
ATOM	768	HA	SER	2	35.040	55.330	23.740	1.00	0.00
ATOM	769	CB	SER	2	33.180	54.340	24.090	1.00	0.00
ATOM	770	HB1	SER	2	32.860	55.030	24.880	1.00	0.00
ATOM	771	HB2	SER	2	32.730	53.410	24.450	1.00	0.00
ATOM	772	OG	SER	2	32.570	54.980	23.000	1.00	0.00
ATOM	773	HG	SER	2	32.420	54.360	22.280	1.00	0.00
ATOM	774	C	SER	2	35.070	53.530	22.620	1.00	0.00
ATOM	775	O	SER	2	35.030	52.310	22.620	1.00	0.00
ATOM	776	N	ASN	3	35.290	54.130	21.450	1.00	0.00
ATOM	777	H	ASN	3	35.420	53.430	20.730	1.00	0.00
ATOM	778	CA	ASN	3	35.350	55.550	21.140	1.00	0.00
ATOM	779	HA	ASN	3	34.420	55.950	21.550	1.00	0.00
ATOM	780	CB	ASN	3	35.330	55.700	19.620	1.00	0.00
ATOM	781	HB1	ASN	3	36.210	55.180	19.230	1.00	0.00
ATOM	782	HB2	ASN	3	35.280	56.750	19.350	1.00	0.00
ATOM	783	CG	ASN	3	34.120	55.010	19.010	1.00	0.00
ATOM	784	OD1	ASN	3	34.100	53.810	18.740	1.00	0.00
ATOM	785	ND2	ASN	3	32.990	55.690	18.790	1.00	0.00
ATOM	786	1HD2	ASN	3	32.210	55.090	18.540	1.00	0.00
ATOM	787	2HD2	ASN	3	32.860	56.660	19.040	1.00	0.00
ATOM	788	C	ASN	3	36.540	56.320	21.680	1.00	0.00
ATOM	789	O	ASN	3	36.420	57.520	21.920	1.00	0.00
ATOM	790	N	ASN	4	37.600	55.620	22.090	1.00	0.00
ATOM	791	H	ASN	4	37.470	54.690	21.720	1.00	0.00

ATOM	792	CA	ASN	4	38.710	56.000	22.940	1.00	0.00
ATOM	793	HA	ASN	4	38.300	56.730	23.630	1.00	0.00
ATOM	794	CB	ASN	4	39.820	56.650	22.120	1.00	0.00
ATOM	795	HB1	ASN	4	40.630	57.080	22.700	1.00	0.00
ATOM	796	HB2	ASN	4	39.390	57.440	21.490	1.00	0.00
ATOM	797	CG	ASN	4	40.580	55.590	21.340	1.00	0.00
ATOM	798	OD1	ASN	4	41.560	55.020	21.820	1.00	0.00
ATOM	799	ND2	ASN	4	40.300	55.390	20.050	1.00	0.00
ATOM	800	1HD2	ASN	4	39.540	55.930	19.650	1.00	0.00
ATOM	801	2HD2	ASN	4	41.060	54.960	19.540	1.00	0.00
ATOM	802	C	ASN	4	39.150	54.790	23.760	1.00	0.00
ATOM	803	O	ASN	4	38.710	53.660	23.590	1.00	0.00
ATOM	804	N	PHE	5	39.980	54.940	24.790	1.00	0.00
ATOM	805	H	PHE	5	40.340	55.880	24.890	1.00	0.00
ATOM	806	CA	PHE	5	40.710	53.850	25.380	1.00	0.00
ATOM	807	HA	PHE	5	40.860	53.090	24.610	1.00	0.00
ATOM	808	CB	PHE	5	40.020	53.350	26.650	1.00	0.00
ATOM	809	HB1	PHE	5	40.400	54.010	27.430	1.00	0.00
ATOM	810	HB2	PHE	5	38.990	53.710	26.580	1.00	0.00
ATOM	811	CG	PHE	5	40.020	51.900	27.070	1.00	0.00
ATOM	812	CD1	PHE	5	39.180	50.970	26.450	1.00	0.00
ATOM	813	HD1	PHE	5	38.470	51.300	25.720	1.00	0.00
ATOM	814	CE1	PHE	5	39.080	49.700	27.050	1.00	0.00
ATOM	815	HE1	PHE	5	38.430	48.980	26.570	1.00	0.00
ATOM	816	CZ	PHE	5	39.940	49.250	28.050	1.00	0.00
ATOM	817	HZ	PHE	5	39.970	48.240	28.450	1.00	0.00
ATOM	818	CE2	PHE	5	40.810	50.200	28.600	1.00	0.00
ATOM	819	HE2	PHE	5	41.370	49.950	29.490	1.00	0.00
ATOM	820	CD2	PHE	5	40.850	51.520	28.140	1.00	0.00
ATOM	821	HD2	PHE	5	41.470	52.230	28.650	1.00	0.00
ATOM	822	C	PHE	5	42.150	54.280	25.620	1.00	0.00
ATOM	823	O	PHE	5	42.430	55.480	25.670	1.00	0.00
ATOM	824	N	GLY	6	43.090	53.330	25.620	1.00	0.00
ATOM	825	H	GLY	6	42.830	52.390	25.350	1.00	0.00
ATOM	826	CA	GLY	6	44.490	53.560	25.900	1.00	0.00
ATOM	827	HA1	GLY	6	44.770	54.520	25.460	1.00	0.00
ATOM	828	HA2	GLY	6	44.990	52.720	25.410	1.00	0.00
ATOM	829	C	GLY	6	44.850	53.540	27.380	1.00	0.00
ATOM	830	O	GLY	6	44.140	52.970	28.200	1.00	0.00
ATOM	831	N	ALA	7	45.980	54.150	27.720	1.00	0.00
ATOM	832	H	ALA	7	46.530	54.670	27.040	1.00	0.00
ATOM	833	CA	ALA	7	46.620	54.200	29.020	1.00	0.00
ATOM	834	HA	ALA	7	46.330	53.320	29.590	1.00	0.00
ATOM	835	CB	ALA	7	48.140	54.110	28.850	1.00	0.00

ATOM	836	HB1	ALA	7	48.430	53.290	28.190	1.00	0.00
ATOM	837	HB2	ALA	7	48.470	54.990	28.300	1.00	0.00
ATOM	838	HB3	ALA	7	48.580	54.050	29.840	1.00	0.00
ATOM	839	C	ALA	7	46.100	55.350	29.870	1.00	0.00
ATOM	840	O	ALA	7	46.060	56.530	29.540	1.00	0.00
ATOM	841	N	ILE	8	45.730	54.960	31.100	1.00	0.00
ATOM	842	H	ILE	8	46.050	54.050	31.390	1.00	0.00
ATOM	843	CA	ILE	8	44.920	55.730	32.020	1.00	0.00
ATOM	844	HA	ILE	8	44.940	56.800	31.800	1.00	0.00
ATOM	845	CB	ILE	8	43.490	55.220	31.900	1.00	0.00
ATOM	846	HB	ILE	8	42.910	55.860	32.570	1.00	0.00
ATOM	847	CG2	ILE	8	42.890	55.620	30.550	1.00	0.00
ATOM	848	1HG2	ILE	8	41.860	55.260	30.530	1.00	0.00
ATOM	849	2HG2	ILE	8	43.040	56.680	30.350	1.00	0.00
ATOM	850	3HG2	ILE	8	43.420	55.000	29.830	1.00	0.00
ATOM	851	CG1	ILE	8	43.320	53.740	32.240	1.00	0.00
ATOM	852	1HG1	ILE	8	43.830	53.160	31.470	1.00	0.00
ATOM	853	2HG1	ILE	8	43.880	53.560	33.160	1.00	0.00
ATOM	854	CD	ILE	8	41.840	53.390	32.290	1.00	0.00
ATOM	855	HD1	ILE	8	41.610	52.680	33.090	1.00	0.00
ATOM	856	HD2	ILE	8	41.450	53.080	31.320	1.00	0.00
ATOM	857	HD3	ILE	8	41.320	54.280	32.650	1.00	0.00
ATOM	858	C	ILE	8	45.540	55.590	33.400	1.00	0.00
ATOM	859	O	ILE	8	46.160	54.570	33.680	1.00	0.00
ATOM	860	N	LEU	9	45.400	56.620	34.230	1.00	0.00
ATOM	861	H	LEU	9	44.980	57.460	33.860	1.00	0.00
ATOM	862	CA	LEU	9	45.980	56.760	35.550	1.00	0.00
ATOM	863	HA	LEU	9	45.800	55.780	36.000	1.00	0.00
ATOM	864	CB	LEU	9	45.090	57.650	36.420	1.00	0.00
ATOM	865	HB1	LEU	9	45.040	58.670	36.040	1.00	0.00
ATOM	866	HB2	LEU	9	45.590	57.680	37.390	1.00	0.00
ATOM	867	CG	LEU	9	43.650	57.210	36.670	1.00	0.00
ATOM	868	HG	LEU	9	43.120	57.150	35.720	1.00	0.00
ATOM	869	CD1	LEU	9	43.570	55.870	37.400	1.00	0.00
ATOM	870	1HD1	LEU	9	44.360	55.840	38.150	1.00	0.00
ATOM	871	2HD1	LEU	9	43.830	55.050	36.730	1.00	0.00
ATOM	872	3HD1	LEU	9	42.620	55.720	37.910	1.00	0.00
ATOM	873	CD2	LEU	9	43.150	58.380	37.510	1.00	0.00
ATOM	874	1HD2	LEU	9	43.370	59.390	37.160	1.00	0.00
ATOM	875	2HD2	LEU	9	43.490	58.280	38.540	1.00	0.00
ATOM	876	3HD2	LEU	9	42.070	58.210	37.600	1.00	0.00
ATOM	877	C	LEU	9	47.480	56.930	35.720	1.00	0.00
ATOM	878	O	LEU	9	48.000	57.650	36.570	1.00	0.00
ATOM	879	N	SER	10	48.160	56.220	34.820	1.00	0.00

ATOM	880	H	SER	10	47.650	55.600	34.200	1.00	0.00
ATOM	881	CA	SER	10	49.550	56.480	34.490	1.00	0.00
ATOM	882	HA	SER	10	49.790	57.530	34.610	1.00	0.00
ATOM	883	CB	SER	10	49.770	56.130	33.020	1.00	0.00
ATOM	884	HB1	SER	10	50.780	56.400	32.740	1.00	0.00
ATOM	885	HB2	SER	10	49.510	55.080	32.890	1.00	0.00
ATOM	886	OG	SER	10	48.870	56.970	32.330	1.00	0.00
ATOM	887	HG	SER	10	49.130	57.890	32.280	1.00	0.00
ATOM	888	C	SER	10	50.460	55.710	35.440	1.00	0.00
ATOM	889	O	SER	10	50.470	54.480	35.360	1.00	0.00
ATOM	890	N	SER	11	51.210	56.420	36.290	1.00	0.00
ATOM	891	H	SER	11	51.120	57.420	36.150	1.00	0.00
ATOM	892	CA	SER	11	52.190	56.020	37.270	1.00	0.00
ATOM	893	HA	SER	11	52.730	55.160	36.890	1.00	0.00
ATOM	894	CB	SER	11	53.200	57.150	37.480	1.00	0.00
ATOM	895	HB1	SER	11	52.620	57.890	38.020	1.00	0.00
ATOM	896	HB2	SER	11	54.040	56.820	38.100	1.00	0.00
ATOM	897	OG	SER	11	53.680	57.580	36.230	1.00	0.00
ATOM	898	HG	SER	11	53.660	58.540	36.230	1.00	0.00
ATOM	899	C	SER	11	51.470	55.540	38.530	1.00	0.00
ATOM	900	O	SER	11	50.340	55.930	38.840	1.00	0.00
ATOM	901	N	NH2	12	52.160	54.690	39.270	1.00	0.00
ATOM	902	H1	NH2	12	51.620	54.340	40.050	1.00	0.00
ATOM	903	H2	NH2	12	53.150	54.510	39.170	1.00	0.00
ATOM	904	CH3	ACE	1	23.540	39.150	41.880	1.00	0.00
ATOM	905	1HH3	ACE	1	24.280	38.540	41.360	1.00	0.00
ATOM	906	2HH3	ACE	1	22.560	38.880	41.510	1.00	0.00
ATOM	907	3HH3	ACE	1	23.640	38.940	42.950	1.00	0.00
ATOM	908	C	ACE	1	23.880	40.630	41.730	1.00	0.00
ATOM	909	O	ACE	1	24.780	41.090	42.430	1.00	0.00
ATOM	910	N	SER	2	23.130	41.330	40.880	1.00	0.00
ATOM	911	H	SER	2	22.320	40.890	40.470	1.00	0.00
ATOM	912	CA	SER	2	23.380	42.730	40.610	1.00	0.00
ATOM	913	HA	SER	2	23.980	43.120	41.430	1.00	0.00
ATOM	914	CB	SER	2	22.140	43.590	40.830	1.00	0.00
ATOM	915	HB1	SER	2	21.450	43.350	40.010	1.00	0.00
ATOM	916	HB2	SER	2	22.380	44.650	40.870	1.00	0.00
ATOM	917	OG	SER	2	21.580	43.220	42.070	1.00	0.00
ATOM	918	HG	SER	2	21.070	43.950	42.430	1.00	0.00
ATOM	919	C	SER	2	23.960	43.010	39.230	1.00	0.00
ATOM	920	O	SER	2	23.280	42.830	38.220	1.00	0.00
ATOM	921	N	ASN	3	25.090	43.710	39.120	1.00	0.00
ATOM	922	H	ASN	3	25.490	43.930	38.220	1.00	0.00
ATOM	923	CA	ASN	3	26.080	43.870	40.170	1.00	0.00

ATOM	924	HA	ASN	3	25.650	44.130	41.130	1.00	0.00
ATOM	925	CB	ASN	3	26.940	45.080	39.820	1.00	0.00
ATOM	926	HB1	ASN	3	27.450	44.930	38.870	1.00	0.00
ATOM	927	HB2	ASN	3	27.820	45.120	40.460	1.00	0.00
ATOM	928	CG	ASN	3	26.140	46.380	39.810	1.00	0.00
ATOM	929	OD1	ASN	3	26.210	47.150	38.860	1.00	0.00
ATOM	930	ND2	ASN	3	25.430	46.720	40.890	1.00	0.00
ATOM	931	1HD2	ASN	3	25.010	47.640	40.840	1.00	0.00
ATOM	932	2HD2	ASN	3	25.560	46.210	41.750	1.00	0.00
ATOM	933	C	ASN	3	26.890	42.610	40.450	1.00	0.00
ATOM	934	O	ASN	3	27.520	42.450	41.490	1.00	0.00
ATOM	935	N	ASN	4	26.880	41.740	39.440	1.00	0.00
ATOM	936	H	ASN	4	26.390	41.960	38.580	1.00	0.00
ATOM	937	CA	ASN	4	27.700	40.540	39.350	1.00	0.00
ATOM	938	HA	ASN	4	28.260	40.330	40.270	1.00	0.00
ATOM	939	CB	ASN	4	28.790	40.770	38.310	1.00	0.00
ATOM	940	HB1	ASN	4	28.410	41.110	37.350	1.00	0.00
ATOM	941	HB2	ASN	4	29.250	39.780	38.220	1.00	0.00
ATOM	942	CG	ASN	4	29.750	41.890	38.700	1.00	0.00
ATOM	943	OD1	ASN	4	29.740	42.640	39.670	1.00	0.00
ATOM	944	ND2	ASN	4	30.920	41.920	38.060	1.00	0.00
ATOM	945	1HD2	ASN	4	31.130	41.280	37.300	1.00	0.00
ATOM	946	2HD2	ASN	4	31.630	42.500	38.480	1.00	0.00
ATOM	947	C	ASN	4	26.810	39.330	39.110	1.00	0.00
ATOM	948	O	ASN	4	25.680	39.420	38.630	1.00	0.00
ATOM	949	N	PHE	5	27.260	38.090	39.300	1.00	0.00
ATOM	950	H	PHE	5	28.100	37.970	39.860	1.00	0.00
ATOM	951	CA	PHE	5	26.560	36.890	38.900	1.00	0.00
ATOM	952	HA	PHE	5	26.360	37.040	37.840	1.00	0.00
ATOM	953	CB	PHE	5	27.530	35.710	38.880	1.00	0.00
ATOM	954	HB1	PHE	5	28.460	36.010	38.410	1.00	0.00
ATOM	955	HB2	PHE	5	27.680	35.390	39.920	1.00	0.00
ATOM	956	CG	PHE	5	27.050	34.470	38.160	1.00	0.00
ATOM	957	CD1	PHE	5	26.800	34.530	36.790	1.00	0.00
ATOM	958	HD1	PHE	5	26.880	35.510	36.350	1.00	0.00
ATOM	959	CE1	PHE	5	26.460	33.390	36.050	1.00	0.00
ATOM	960	HE1	PHE	5	26.300	33.400	34.980	1.00	0.00
ATOM	961	CZ	PHE	5	26.380	32.180	36.750	1.00	0.00
ATOM	962	HZ	PHE	5	26.080	31.250	36.270	1.00	0.00
ATOM	963	CE2	PHE	5	26.560	32.130	38.130	1.00	0.00
ATOM	964	HE2	PHE	5	26.340	31.160	38.570	1.00	0.00
ATOM	965	CD2	PHE	5	26.860	33.280	38.870	1.00	0.00
ATOM	966	HD2	PHE	5	27.180	33.150	39.900	1.00	0.00
ATOM	967	C	PHE	5	25.310	36.600	39.720	1.00	0.00

ATOM	968	O	PHE	5	25.390	36.370	40.930	1.00	0.00
ATOM	969	N	GLY	6	24.160	36.520	39.060	1.00	0.00
ATOM	970	H	GLY	6	24.290	36.460	38.060	1.00	0.00
ATOM	971	CA	GLY	6	22.890	36.440	39.760	1.00	0.00
ATOM	972	HA1	GLY	6	22.160	36.920	39.110	1.00	0.00
ATOM	973	HA2	GLY	6	22.890	36.930	40.730	1.00	0.00
ATOM	974	C	GLY	6	22.400	35.050	40.140	1.00	0.00
ATOM	975	O	GLY	6	23.190	34.110	40.100	1.00	0.00
ATOM	976	N	ALA	7	21.100	34.810	40.310	1.00	0.00
ATOM	977	H	ALA	7	20.930	33.910	40.750	1.00	0.00
ATOM	978	CA	ALA	7	19.940	35.680	40.360	1.00	0.00
ATOM	979	HA	ALA	7	19.830	36.240	39.430	1.00	0.00
ATOM	980	CB	ALA	7	18.720	34.760	40.350	1.00	0.00
ATOM	981	HB1	ALA	7	18.740	34.020	39.550	1.00	0.00
ATOM	982	HB2	ALA	7	18.820	34.330	41.350	1.00	0.00
ATOM	983	HB3	ALA	7	17.900	35.460	40.190	1.00	0.00
ATOM	984	C	ALA	7	19.820	36.730	41.450	1.00	0.00
ATOM	985	O	ALA	7	20.600	36.800	42.400	1.00	0.00
ATOM	986	N	ILE	8	18.860	37.630	41.270	1.00	0.00
ATOM	987	H	ILE	8	18.310	37.480	40.440	1.00	0.00
ATOM	988	CA	ILE	8	18.690	38.800	42.110	1.00	0.00
ATOM	989	HA	ILE	8	18.860	38.490	43.140	1.00	0.00
ATOM	990	CB	ILE	8	17.300	39.410	41.990	1.00	0.00
ATOM	991	HB	ILE	8	17.250	40.480	42.210	1.00	0.00
ATOM	992	CG2	ILE	8	16.590	39.100	40.670	1.00	0.00
ATOM	993	1HG2	ILE	8	15.550	39.400	40.610	1.00	0.00
ATOM	994	2HG2	ILE	8	17.040	39.670	39.850	1.00	0.00
ATOM	995	3HG2	ILE	8	16.670	38.060	40.360	1.00	0.00
ATOM	996	CG1	ILE	8	16.470	38.800	43.120	1.00	0.00
ATOM	997	1HG1	ILE	8	16.340	37.730	43.000	1.00	0.00
ATOM	998	2HG1	ILE	8	16.960	38.980	44.080	1.00	0.00
ATOM	999	CD	ILE	8	15.120	39.500	43.280	1.00	0.00
ATOM	1000	HD1	ILE	8	14.390	39.250	42.510	1.00	0.00
ATOM	1001	HD2	ILE	8	15.260	40.580	43.300	1.00	0.00
ATOM	1002	HD3	ILE	8	14.690	39.210	44.240	1.00	0.00
ATOM	1003	C	ILE	8	19.710	39.910	41.900	1.00	0.00
ATOM	1004	O	ILE	8	20.200	39.990	40.770	1.00	0.00
ATOM	1005	N	LEU	9	20.110	40.740	42.870	1.00	0.00
ATOM	1006	H	LEU	9	20.820	41.400	42.590	1.00	0.00
ATOM	1007	CA	LEU	9	19.600	40.810	44.220	1.00	0.00
ATOM	1008	HA	LEU	9	19.160	39.870	44.540	1.00	0.00
ATOM	1009	CB	LEU	9	20.790	41.100	45.150	1.00	0.00
ATOM	1010	HB1	LEU	9	20.460	41.170	46.180	1.00	0.00
ATOM	1011	HB2	LEU	9	21.160	42.080	44.850	1.00	0.00

ATOM	1012	CG	LEU	9	21.850	40.010	45.050	1.00	0.00
ATOM	1013	HG	LEU	9	22.240	40.030	44.030	1.00	0.00
ATOM	1014	CD1	LEU	9	21.200	38.690	45.470	1.00	0.00
ATOM	1015	1HD1	LEU	9	21.970	38.050	45.900	1.00	0.00
ATOM	1016	2HD1	LEU	9	20.490	38.830	46.280	1.00	0.00
ATOM	1017	3HD1	LEU	9	20.770	38.190	44.600	1.00	0.00
ATOM	1018	CD2	LEU	9	23.000	40.260	46.020	1.00	0.00
ATOM	1019	1HD2	LEU	9	23.910	39.720	45.760	1.00	0.00
ATOM	1020	2HD2	LEU	9	22.570	40.210	47.020	1.00	0.00
ATOM	1021	3HD2	LEU	9	23.120	41.330	45.870	1.00	0.00
ATOM	1022	C	LEU	9	18.470	41.810	44.370	1.00	0.00
ATOM	1023	O	LEU	9	18.350	42.710	43.550	1.00	0.00
ATOM	1024	N	SER	10	17.670	41.740	45.440	1.00	0.00
ATOM	1025	H	SER	10	17.820	40.940	46.040	1.00	0.00
ATOM	1026	CA	SER	10	16.630	42.700	45.740	1.00	0.00
ATOM	1027	HA	SER	10	16.130	42.970	44.810	1.00	0.00
ATOM	1028	CB	SER	10	15.650	41.990	46.670	1.00	0.00
ATOM	1029	HB1	SER	10	15.390	41.010	46.260	1.00	0.00
ATOM	1030	HB2	SER	10	14.710	42.480	46.910	1.00	0.00
ATOM	1031	OG	SER	10	16.320	41.700	47.870	1.00	0.00
ATOM	1032	HG	SER	10	15.740	41.110	48.340	1.00	0.00
ATOM	1033	C	SER	10	17.190	43.950	46.410	1.00	0.00
ATOM	1034	O	SER	10	16.400	44.820	46.770	1.00	0.00
ATOM	1035	N	SER	11	18.510	44.100	46.540	1.00	0.00
ATOM	1036	H	SER	11	19.130	43.350	46.260	1.00	0.00
ATOM	1037	CA	SER	11	19.180	45.300	46.980	1.00	0.00
ATOM	1038	HA	SER	11	19.020	46.080	46.240	1.00	0.00
ATOM	1039	CB	SER	11	20.700	45.140	46.970	1.00	0.00
ATOM	1040	HB1	SER	11	21.120	46.140	46.900	1.00	0.00
ATOM	1041	HB2	SER	11	21.020	44.800	47.960	1.00	0.00
ATOM	1042	OG	SER	11	21.150	44.330	45.910	1.00	0.00
ATOM	1043	HG	SER	11	22.110	44.260	45.980	1.00	0.00
ATOM	1044	C	SER	11	18.920	45.680	48.430	1.00	0.00
ATOM	1045	O	SER	11	19.430	46.720	48.860	1.00	0.00
ATOM	1046	N	NH2	12	18.250	44.890	49.280	1.00	0.00
ATOM	1047	H1	NH2	12	18.140	45.180	50.240	1.00	0.00
ATOM	1048	H2	NH2	12	17.810	44.030	49.000	1.00	0.00
ATOM	1049	CH3	ACE	1	25.430	3.900	29.890	1.00	0.00
ATOM	1050	1HH3	ACE	1	24.640	3.150	29.810	1.00	0.00
ATOM	1051	2HH3	ACE	1	25.660	4.450	28.980	1.00	0.00
ATOM	1052	3HH3	ACE	1	24.900	4.680	30.430	1.00	0.00
ATOM	1053	C	ACE	1	26.780	3.680	30.560	1.00	0.00
ATOM	1054	O	ACE	1	27.040	4.230	31.620	1.00	0.00
ATOM	1055	N	SER	2	27.610	2.930	29.840	1.00	0.00

ATOM	1056	H	SER	2	27.420	2.680	28.880	1.00	0.00
ATOM	1057	CA	SER	2	29.010	2.660	30.100	1.00	0.00
ATOM	1058	HA	SER	2	29.060	2.350	31.150	1.00	0.00
ATOM	1059	CB	SER	2	29.520	1.500	29.250	1.00	0.00
ATOM	1060	HB1	SER	2	30.490	1.310	29.700	1.00	0.00
ATOM	1061	HB2	SER	2	29.510	1.790	28.200	1.00	0.00
ATOM	1062	OG	SER	2	28.670	0.380	29.310	1.00	0.00
ATOM	1063	HG	SER	2	27.970	0.640	28.700	1.00	0.00
ATOM	1064	C	SER	2	29.870	3.900	29.930	1.00	0.00
ATOM	1065	O	SER	2	29.820	4.620	28.930	1.00	0.00
ATOM	1066	N	ASN	3	30.760	4.190	30.880	1.00	0.00
ATOM	1067	H	ASN	3	31.320	4.990	30.600	1.00	0.00
ATOM	1068	CA	ASN	3	31.080	3.480	32.100	1.00	0.00
ATOM	1069	HA	ASN	3	30.810	2.420	32.060	1.00	0.00
ATOM	1070	CB	ASN	3	30.310	4.080	33.270	1.00	0.00
ATOM	1071	HB1	ASN	3	30.680	5.100	33.320	1.00	0.00
ATOM	1072	HB2	ASN	3	29.240	4.190	33.050	1.00	0.00
ATOM	1073	CG	ASN	3	30.490	3.300	34.560	1.00	0.00
ATOM	1074	OD1	ASN	3	30.750	2.100	34.700	1.00	0.00
ATOM	1075	ND2	ASN	3	30.420	4.050	35.660	1.00	0.00
ATOM	1076	1HD2	ASN	3	30.910	3.800	36.520	1.00	0.00
ATOM	1077	2HD2	ASN	3	30.120	5.000	35.550	1.00	0.00
ATOM	1078	C	ASN	3	32.580	3.530	32.330	1.00	0.00
ATOM	1079	O	ASN	3	33.350	4.230	31.670	1.00	0.00
ATOM	1080	N	ASN	4	33.110	2.610	33.140	1.00	0.00
ATOM	1081	H	ASN	4	32.480	2.130	33.770	1.00	0.00
ATOM	1082	CA	ASN	4	34.520	2.570	33.490	1.00	0.00
ATOM	1083	HA	ASN	4	34.920	3.540	33.180	1.00	0.00
ATOM	1084	CB	ASN	4	35.010	1.470	32.550	1.00	0.00
ATOM	1085	HB1	ASN	4	34.730	1.740	31.540	1.00	0.00
ATOM	1086	HB2	ASN	4	34.550	0.530	32.840	1.00	0.00
ATOM	1087	CG	ASN	4	36.470	1.110	32.750	1.00	0.00
ATOM	1088	OD1	ASN	4	37.380	1.650	32.120	1.00	0.00
ATOM	1089	ND2	ASN	4	36.770	0.110	33.600	1.00	0.00
ATOM	1090	1HD2	ASN	4	35.990	-0.380	34.010	1.00	0.00
ATOM	1091	2HD2	ASN	4	37.730	-0.050	33.860	1.00	0.00
ATOM	1092	C	ASN	4	34.770	2.240	34.950	1.00	0.00
ATOM	1093	O	ASN	4	33.920	1.560	35.520	1.00	0.00
ATOM	1094	N	PHE	5	35.820	2.700	35.640	1.00	0.00
ATOM	1095	H	PHE	5	35.870	2.420	36.610	1.00	0.00
ATOM	1096	CA	PHE	5	36.870	3.600	35.230	1.00	0.00
ATOM	1097	HA	PHE	5	36.780	3.690	34.150	1.00	0.00
ATOM	1098	CB	PHE	5	36.590	4.990	35.800	1.00	0.00
ATOM	1099	HB1	PHE	5	36.810	4.900	36.870	1.00	0.00

ATOM	1100	HB2	PHE	5	37.280	5.680	35.320	1.00	0.00
ATOM	1101	CG	PHE	5	35.150	5.440	35.750	1.00	0.00
ATOM	1102	CD1	PHE	5	34.600	5.790	34.510	1.00	0.00
ATOM	1103	HD1	PHE	5	35.180	5.790	33.600	1.00	0.00
ATOM	1104	CE1	PHE	5	33.320	6.370	34.480	1.00	0.00
ATOM	1105	HE1	PHE	5	33.100	6.960	33.600	1.00	0.00
ATOM	1106	CZ	PHE	5	32.480	6.410	35.600	1.00	0.00
ATOM	1107	HZ	PHE	5	31.470	6.780	35.540	1.00	0.00
ATOM	1108	CE2	PHE	5	33.030	5.920	36.790	1.00	0.00
ATOM	1109	HE2	PHE	5	32.420	6.000	37.680	1.00	0.00
ATOM	1110	CD2	PHE	5	34.370	5.540	36.910	1.00	0.00
ATOM	1111	HD2	PHE	5	34.710	5.220	37.880	1.00	0.00
ATOM	1112	C	PHE	5	38.300	3.150	35.520	1.00	0.00
ATOM	1113	O	PHE	5	39.320	3.740	35.200	1.00	0.00
ATOM	1114	N	GLY	6	38.430	2.060	36.270	1.00	0.00
ATOM	1115	H	GLY	6	37.640	1.510	36.570	1.00	0.00
ATOM	1116	CA	GLY	6	39.660	1.350	36.570	1.00	0.00
ATOM	1117	HA1	GLY	6	39.850	1.260	37.640	1.00	0.00
ATOM	1118	HA2	GLY	6	40.540	1.910	36.260	1.00	0.00
ATOM	1119	C	GLY	6	39.740	0.040	35.800	1.00	0.00
ATOM	1120	O	GLY	6	39.460	0.070	34.600	1.00	0.00
ATOM	1121	N	ALA	7	40.100	-1.060	36.460	1.00	0.00
ATOM	1122	H	ALA	7	40.410	-0.920	37.410	1.00	0.00
ATOM	1123	CA	ALA	7	39.910	-2.440	36.050	1.00	0.00
ATOM	1124	HA	ALA	7	40.250	-2.510	35.010	1.00	0.00
ATOM	1125	CB	ALA	7	40.960	-3.220	36.840	1.00	0.00
ATOM	1126	HB1	ALA	7	40.920	-4.270	36.550	1.00	0.00
ATOM	1127	HB2	ALA	7	41.960	-2.860	36.600	1.00	0.00
ATOM	1128	HB3	ALA	7	40.860	-3.100	37.920	1.00	0.00
ATOM	1129	C	ALA	7	38.520	-2.990	36.350	1.00	0.00
ATOM	1130	O	ALA	7	37.750	-2.330	37.050	1.00	0.00
ATOM	1131	N	ILE	8	38.190	-4.170	35.820	1.00	0.00
ATOM	1132	H	ILE	8	38.920	-4.610	35.280	1.00	0.00
ATOM	1133	CA	ILE	8	36.950	-4.910	35.940	1.00	0.00
ATOM	1134	HA	ILE	8	36.250	-4.220	36.410	1.00	0.00
ATOM	1135	CB	ILE	8	37.160	-6.120	36.850	1.00	0.00
ATOM	1136	HB	ILE	8	36.320	-6.810	36.770	1.00	0.00
ATOM	1137	CG2	ILE	8	38.350	-7.000	36.470	1.00	0.00
ATOM	1138	1HG2	ILE	8	38.180	-7.990	36.880	1.00	0.00
ATOM	1139	2HG2	ILE	8	38.360	-7.130	35.390	1.00	0.00
ATOM	1140	3HG2	ILE	8	39.260	-6.520	36.840	1.00	0.00
ATOM	1141	CG1	ILE	8	37.280	-5.770	38.330	1.00	0.00
ATOM	1142	1HG1	ILE	8	37.660	-6.660	38.850	1.00	0.00
ATOM	1143	2HG1	ILE	8	38.080	-5.040	38.390	1.00	0.00

ATOM	1144	CD	ILE	8	36.020	-5.270	39.020	1.00	0.00
ATOM	1145	HD1	ILE	8	35.750	-4.270	38.660	1.00	0.00
ATOM	1146	HD2	ILE	8	36.250	-5.220	40.090	1.00	0.00
ATOM	1147	HD3	ILE	8	35.250	-6.020	38.870	1.00	0.00
ATOM	1148	C	ILE	8	36.300	-5.160	34.590	1.00	0.00
ATOM	1149	O	ILE	8	35.980	-6.300	34.280	1.00	0.00
ATOM	1150	N	LEU	9	36.110	-4.090	33.810	1.00	0.00
ATOM	1151	H	LEU	9	36.280	-3.170	34.210	1.00	0.00
ATOM	1152	CA	LEU	9	35.680	-4.220	32.440	1.00	0.00
ATOM	1153	HA	LEU	9	36.010	-5.190	32.060	1.00	0.00
ATOM	1154	CB	LEU	9	36.370	-3.140	31.610	1.00	0.00
ATOM	1155	HB1	LEU	9	35.960	-3.080	30.610	1.00	0.00
ATOM	1156	HB2	LEU	9	36.080	-2.200	32.080	1.00	0.00
ATOM	1157	CG	LEU	9	37.900	-3.070	31.690	1.00	0.00
ATOM	1158	HG	LEU	9	38.170	-2.740	32.690	1.00	0.00
ATOM	1159	CD1	LEU	9	38.540	-4.430	31.390	1.00	0.00
ATOM	1160	1HD1	LEU	9	38.470	-5.140	32.210	1.00	0.00
ATOM	1161	2HD1	LEU	9	38.430	-4.830	30.380	1.00	0.00
ATOM	1162	3HD1	LEU	9	39.610	-4.230	31.370	1.00	0.00
ATOM	1163	CD2	LEU	9	38.180	-2.040	30.600	1.00	0.00
ATOM	1164	1HD2	LEU	9	37.960	-2.490	29.640	1.00	0.00
ATOM	1165	2HD2	LEU	9	37.500	-1.190	30.710	1.00	0.00
ATOM	1166	3HD2	LEU	9	39.230	-1.750	30.650	1.00	0.00
ATOM	1167	C	LEU	9	34.170	-4.240	32.270	1.00	0.00
ATOM	1168	O	LEU	9	33.620	-3.850	31.240	1.00	0.00
ATOM	1169	N	SER	10	33.360	-4.540	33.290	1.00	0.00
ATOM	1170	H	SER	10	33.910	-4.730	34.110	1.00	0.00
ATOM	1171	CA	SER	10	31.920	-4.460	33.420	1.00	0.00
ATOM	1172	HA	SER	10	31.460	-3.650	32.860	1.00	0.00
ATOM	1173	CB	SER	10	31.270	-5.710	32.830	1.00	0.00
ATOM	1174	HB1	SER	10	30.210	-5.480	32.840	1.00	0.00
ATOM	1175	HB2	SER	10	31.700	-5.830	31.830	1.00	0.00
ATOM	1176	OG	SER	10	31.350	-6.850	33.660	1.00	0.00
ATOM	1177	HG	SER	10	32.240	-7.180	33.800	1.00	0.00
ATOM	1178	C	SER	10	31.530	-4.310	34.880	1.00	0.00
ATOM	1179	O	SER	10	32.200	-4.850	35.760	1.00	0.00
ATOM	1180	N	SER	11	30.310	-3.840	35.180	1.00	0.00
ATOM	1181	H	SER	11	29.860	-3.320	34.440	1.00	0.00
ATOM	1182	CA	SER	11	29.770	-3.860	36.520	1.00	0.00
ATOM	1183	HA	SER	11	30.530	-4.130	37.250	1.00	0.00
ATOM	1184	CB	SER	11	29.460	-2.490	37.110	1.00	0.00
ATOM	1185	HB1	SER	11	29.350	-2.650	38.190	1.00	0.00
ATOM	1186	HB2	SER	11	28.530	-2.140	36.670	1.00	0.00
ATOM	1187	OG	SER	11	30.500	-1.610	36.740	1.00	0.00

ATOM	1188	HG	SER	11	30.130	-0.750	36.970	1.00	0.00
ATOM	1189	C	SER	11	28.560	-4.760	36.730	1.00	0.00
ATOM	1190	O	SER	11	27.740	-4.460	37.590	1.00	0.00
ATOM	1191	N	NH2	12	28.270	-5.770	35.900	1.00	0.00
ATOM	1192	H1	NH2	12	27.570	-6.440	36.210	1.00	0.00
ATOM	1193	H2	NH2	12	28.940	-6.050	35.200	1.00	0.00
ATOM	1194	O1	MOL	14	32.050	45.460	25.640	1.00	0.00
ATOM	1195	O2	MOL	14	29.100	40.430	29.420	1.00	0.00
ATOM	1196	N1	MOL	14	34.510	40.870	27.300	1.00	0.00
ATOM	1197	N2	MOL	14	30.560	39.230	28.260	1.00	0.00
ATOM	1198	C1	MOL	14	32.450	40.550	26.440	1.00	0.00
ATOM	1199	C2	MOL	14	32.730	41.980	26.480	1.00	0.00
ATOM	1200	C3	MOL	14	31.270	39.770	25.960	1.00	0.00
ATOM	1201	C4	MOL	14	34.010	42.130	27.040	1.00	0.00
ATOM	1202	C5	MOL	14	33.590	39.930	26.890	1.00	0.00
ATOM	1203	C6	MOL	14	30.180	39.910	27.030	1.00	0.00
ATOM	1204	C7	MOL	14	32.040	43.130	26.090	1.00	0.00
ATOM	1205	C8	MOL	14	34.610	43.370	27.290	1.00	0.00
ATOM	1206	C9	MOL	14	32.680	44.370	26.180	1.00	0.00
ATOM	1207	C10	MOL	14	33.930	44.490	26.790	1.00	0.00
ATOM	1208	C11	MOL	14	29.710	39.380	29.340	1.00	0.00
ATOM	1209	C12	MOL	14	29.720	38.220	30.330	1.00	0.00
ATOM	1210	C13	MOL	14	32.440	46.830	25.780	1.00	0.00
ATOM	1211	H1	MOL	14	31.530	38.720	25.750	1.00	0.00
ATOM	1212	H2	MOL	14	30.870	40.130	25.000	1.00	0.00
ATOM	1213	H3	MOL	14	33.770	38.860	26.890	1.00	0.00
ATOM	1214	H4	MOL	14	29.220	39.460	26.740	1.00	0.00
ATOM	1215	H5	MOL	14	30.090	41.000	27.150	1.00	0.00
ATOM	1216	H6	MOL	14	35.450	40.620	27.570	1.00	0.00
ATOM	1217	H7	MOL	14	31.030	43.020	25.710	1.00	0.00
ATOM	1218	H8	MOL	14	35.500	43.460	27.900	1.00	0.00
ATOM	1219	H9	MOL	14	34.420	45.450	26.940	1.00	0.00
ATOM	1220	H10	MOL	14	31.100	38.370	28.300	1.00	0.00
ATOM	1221	H11	MOL	14	30.030	38.590	31.310	1.00	0.00
ATOM	1222	H12	MOL	14	28.700	37.880	30.580	1.00	0.00
ATOM	1223	H13	MOL	14	30.230	37.310	30.000	1.00	0.00
ATOM	1224	H14	MOL	14	31.900	47.570	25.180	1.00	0.00
ATOM	1225	H15	MOL	14	32.490	47.050	26.850	1.00	0.00
ATOM	1226	H16	MOL	14	33.440	46.920	25.330	1.00	0.00
ATOM	1227	O1	MOL	15	25.490	38.610	27.070	1.00	0.00
ATOM	1228	O2	MOL	15	28.110	47.280	28.880	1.00	0.00
ATOM	1229	N1	MOL	15	28.810	41.390	23.700	1.00	0.00
ATOM	1230	N2	MOL	15	27.580	45.340	27.910	1.00	0.00
ATOM	1231	C1	MOL	15	27.810	42.700	25.260	1.00	0.00

ATOM	1232	C2	MOL	15	27.440	41.310	25.490	1.00	0.00
ATOM	1233	C3	MOL	15	27.340	44.010	25.830	1.00	0.00
ATOM	1234	C4	MOL	15	28.050	40.550	24.500	1.00	0.00
ATOM	1235	C5	MOL	15	28.710	42.660	24.220	1.00	0.00
ATOM	1236	C6	MOL	15	27.940	44.100	27.240	1.00	0.00
ATOM	1237	C7	MOL	15	26.540	40.730	26.390	1.00	0.00
ATOM	1238	C8	MOL	15	27.900	39.160	24.400	1.00	0.00
ATOM	1239	C9	MOL	15	26.290	39.360	26.240	1.00	0.00
ATOM	1240	C10	MOL	15	27.050	38.600	25.350	1.00	0.00
ATOM	1241	C11	MOL	15	28.500	46.330	28.220	1.00	0.00
ATOM	1242	C12	MOL	15	29.890	46.340	27.600	1.00	0.00
ATOM	1243	C13	MOL	15	26.020	38.330	28.370	1.00	0.00
ATOM	1244	H1	MOL	15	26.280	43.830	26.070	1.00	0.00
ATOM	1245	H2	MOL	15	27.360	44.890	25.170	1.00	0.00
ATOM	1246	H3	MOL	15	29.320	43.450	23.800	1.00	0.00
ATOM	1247	H4	MOL	15	29.040	44.070	27.140	1.00	0.00
ATOM	1248	H5	MOL	15	27.530	43.330	27.900	1.00	0.00
ATOM	1249	H6	MOL	15	29.430	41.150	22.940	1.00	0.00
ATOM	1250	H7	MOL	15	25.910	41.300	27.060	1.00	0.00
ATOM	1251	H8	MOL	15	28.460	38.630	23.640	1.00	0.00
ATOM	1252	H9	MOL	15	26.870	37.520	25.360	1.00	0.00
ATOM	1253	H10	MOL	15	26.660	45.420	28.310	1.00	0.00
ATOM	1254	H11	MOL	15	30.420	45.550	28.140	1.00	0.00
ATOM	1255	H12	MOL	15	29.870	46.250	26.500	1.00	0.00
ATOM	1256	H13	MOL	15	30.390	47.280	27.830	1.00	0.00
ATOM	1257	H14	MOL	15	26.440	39.260	28.770	1.00	0.00
ATOM	1258	H15	MOL	15	25.200	37.950	28.990	1.00	0.00
ATOM	1259	H16	MOL	15	26.900	37.680	28.470	1.00	0.00
ATOM	1260	O1	MOL	16	38.000	46.240	19.120	1.00	0.00
ATOM	1261	O2	MOL	16	46.140	47.550	20.900	1.00	0.00
ATOM	1262	N1	MOL	16	42.880	44.100	20.250	1.00	0.00
ATOM	1263	N2	MOL	16	44.880	48.000	19.110	1.00	0.00
ATOM	1264	C1	MOL	16	42.860	45.660	18.590	1.00	0.00
ATOM	1265	C2	MOL	16	41.490	45.350	18.970	1.00	0.00
ATOM	1266	C3	MOL	16	43.380	46.730	17.670	1.00	0.00
ATOM	1267	C4	MOL	16	41.560	44.400	20.000	1.00	0.00
ATOM	1268	C5	MOL	16	43.640	44.860	19.380	1.00	0.00
ATOM	1269	C6	MOL	16	43.610	48.070	18.380	1.00	0.00
ATOM	1270	C7	MOL	16	40.320	46.050	18.680	1.00	0.00
ATOM	1271	C8	MOL	16	40.450	43.890	20.680	1.00	0.00
ATOM	1272	C9	MOL	16	39.180	45.590	19.350	1.00	0.00
ATOM	1273	C10	MOL	16	39.280	44.550	20.290	1.00	0.00
ATOM	1274	C11	MOL	16	45.030	47.770	20.460	1.00	0.00
ATOM	1275	C12	MOL	16	43.830	47.960	21.380	1.00	0.00

ATOM	1276	C13	MOL	16	37.670	46.970	17.930	1.00	0.00
ATOM	1277	H1	MOL	16	42.700	46.940	16.830	1.00	0.00
ATOM	1278	H2	MOL	16	44.330	46.370	17.250	1.00	0.00
ATOM	1279	H3	MOL	16	44.720	44.830	19.520	1.00	0.00
ATOM	1280	H4	MOL	16	42.780	48.210	19.080	1.00	0.00
ATOM	1281	H5	MOL	16	43.670	48.990	17.770	1.00	0.00
ATOM	1282	H6	MOL	16	43.180	43.590	21.070	1.00	0.00
ATOM	1283	H7	MOL	16	40.190	46.800	17.900	1.00	0.00
ATOM	1284	H8	MOL	16	40.500	43.060	21.370	1.00	0.00
ATOM	1285	H9	MOL	16	38.310	44.240	20.680	1.00	0.00
ATOM	1286	H10	MOL	16	45.720	48.010	18.550	1.00	0.00
ATOM	1287	H11	MOL	16	44.250	47.920	22.390	1.00	0.00
ATOM	1288	H12	MOL	16	43.240	48.870	21.220	1.00	0.00
ATOM	1289	H13	MOL	16	43.180	47.080	21.340	1.00	0.00
ATOM	1290	H14	MOL	16	37.910	46.370	17.040	1.00	0.00
ATOM	1291	H15	MOL	16	36.610	47.250	17.910	1.00	0.00
ATOM	1292	H16	MOL	16	38.330	47.840	17.830	1.00	0.00
ATOM	1293	O1	MOL	17	34.680	36.830	41.110	1.00	0.00
ATOM	1294	O2	MOL	17	34.390	29.810	40.810	1.00	0.00
ATOM	1295	N1	MOL	17	29.820	35.000	42.950	1.00	0.00
ATOM	1296	N2	MOL	17	32.580	31.060	41.050	1.00	0.00
ATOM	1297	C1	MOL	17	30.690	33.770	41.260	1.00	0.00
ATOM	1298	C2	MOL	17	31.620	34.840	41.570	1.00	0.00
ATOM	1299	C3	MOL	17	30.810	32.640	40.280	1.00	0.00
ATOM	1300	C4	MOL	17	31.050	35.550	42.640	1.00	0.00
ATOM	1301	C5	MOL	17	29.570	34.020	42.010	1.00	0.00
ATOM	1302	C6	MOL	17	31.140	31.250	40.870	1.00	0.00
ATOM	1303	C7	MOL	17	32.880	35.230	41.110	1.00	0.00
ATOM	1304	C8	MOL	17	31.720	36.580	43.300	1.00	0.00
ATOM	1305	C9	MOL	17	33.520	36.350	41.650	1.00	0.00
ATOM	1306	C10	MOL	17	32.950	36.980	42.760	1.00	0.00
ATOM	1307	C11	MOL	17	33.220	29.840	41.140	1.00	0.00
ATOM	1308	C12	MOL	17	32.420	28.710	41.770	1.00	0.00
ATOM	1309	C13	MOL	17	34.510	38.040	40.380	1.00	0.00
ATOM	1310	H1	MOL	17	31.520	32.880	39.490	1.00	0.00
ATOM	1311	H2	MOL	17	29.820	32.480	39.820	1.00	0.00
ATOM	1312	H3	MOL	17	28.700	33.370	42.100	1.00	0.00
ATOM	1313	H4	MOL	17	30.770	30.460	40.200	1.00	0.00
ATOM	1314	H5	MOL	17	30.620	31.080	41.820	1.00	0.00
ATOM	1315	H6	MOL	17	29.240	35.250	43.740	1.00	0.00
ATOM	1316	H7	MOL	17	33.370	34.810	40.230	1.00	0.00
ATOM	1317	H8	MOL	17	31.500	36.890	44.310	1.00	0.00
ATOM	1318	H9	MOL	17	33.540	37.720	43.290	1.00	0.00
ATOM	1319	H10	MOL	17	33.150	31.770	40.610	1.00	0.00

ATOM	1320	H11	MOL	17	31.560	28.450	41.140	1.00	0.00
ATOM	1321	H12	MOL	17	33.010	27.790	41.940	1.00	0.00
ATOM	1322	H13	MOL	17	31.940	29.040	42.690	1.00	0.00
ATOM	1323	H14	MOL	17	34.090	38.850	40.990	1.00	0.00
ATOM	1324	H15	MOL	17	35.400	38.300	39.790	1.00	0.00
ATOM	1325	H16	MOL	17	33.710	37.930	39.630	1.00	0.00
ATOM	1326	O1	MOL	18	33.150	1.250	28.520	1.00	0.00
ATOM	1327	O2	MOL	18	38.380	3.540	24.460	1.00	0.00
ATOM	1328	N1	MOL	18	37.020	5.140	28.800	1.00	0.00
ATOM	1329	N2	MOL	18	39.110	2.250	26.150	1.00	0.00
ATOM	1330	C1	MOL	18	37.790	3.020	28.580	1.00	0.00
ATOM	1331	C2	MOL	18	36.340	3.030	28.570	1.00	0.00
ATOM	1332	C3	MOL	18	38.860	1.970	28.530	1.00	0.00
ATOM	1333	C4	MOL	18	35.890	4.350	28.720	1.00	0.00
ATOM	1334	C5	MOL	18	38.140	4.340	28.750	1.00	0.00
ATOM	1335	C6	MOL	18	39.870	2.180	27.390	1.00	0.00
ATOM	1336	C7	MOL	18	35.450	1.950	28.530	1.00	0.00
ATOM	1337	C8	MOL	18	34.540	4.670	28.540	1.00	0.00
ATOM	1338	C9	MOL	18	34.080	2.260	28.530	1.00	0.00
ATOM	1339	C10	MOL	18	33.660	3.580	28.460	1.00	0.00
ATOM	1340	C11	MOL	18	39.280	3.270	25.240	1.00	0.00
ATOM	1341	C12	MOL	18	40.700	3.680	24.880	1.00	0.00
ATOM	1342	C13	MOL	18	32.920	0.390	29.630	1.00	0.00
ATOM	1343	H1	MOL	18	38.360	0.990	28.440	1.00	0.00
ATOM	1344	H2	MOL	18	39.400	1.930	29.490	1.00	0.00
ATOM	1345	H3	MOL	18	39.140	4.750	28.870	1.00	0.00
ATOM	1346	H4	MOL	18	40.700	1.460	27.480	1.00	0.00
ATOM	1347	H5	MOL	18	40.350	3.160	27.480	1.00	0.00
ATOM	1348	H6	MOL	18	37.060	6.140	28.890	1.00	0.00
ATOM	1349	H7	MOL	18	35.660	0.890	28.490	1.00	0.00
ATOM	1350	H8	MOL	18	34.320	5.730	28.480	1.00	0.00
ATOM	1351	H9	MOL	18	32.600	3.840	28.480	1.00	0.00
ATOM	1352	H10	MOL	18	38.150	1.950	26.210	1.00	0.00
ATOM	1353	H11	MOL	18	41.300	2.770	24.740	1.00	0.00
ATOM	1354	H12	MOL	18	40.740	4.050	23.850	1.00	0.00
ATOM	1355	H13	MOL	18	41.170	4.300	25.650	1.00	0.00
ATOM	1356	H14	MOL	18	32.170	-0.360	29.340	1.00	0.00
ATOM	1357	H15	MOL	18	33.840	-0.160	29.880	1.00	0.00
ATOM	1358	H16	MOL	18	32.700	1.020	30.500	1.00	0.00
ATOM	1359	O1	MOL	19	15.770	44.590	39.540	1.00	0.00
ATOM	1360	O2	MOL	19	13.870	50.960	37.130	1.00	0.00
ATOM	1361	N1	MOL	19	14.860	49.050	42.480	1.00	0.00
ATOM	1362	N2	MOL	19	15.720	50.580	38.350	1.00	0.00
ATOM	1363	C1	MOL	19	16.440	49.300	40.790	1.00	0.00

ATOM	1364	C2	MOL	19	15.900	47.950	40.840	1.00	0.00
ATOM	1365	C3	MOL	19	17.470	49.990	39.940	1.00	0.00
ATOM	1366	C4	MOL	19	14.900	47.840	41.810	1.00	0.00
ATOM	1367	C5	MOL	19	15.770	49.870	41.840	1.00	0.00
ATOM	1368	C6	MOL	19	16.840	51.130	39.130	1.00	0.00
ATOM	1369	C7	MOL	19	16.160	46.910	39.930	1.00	0.00
ATOM	1370	C8	MOL	19	14.250	46.630	42.070	1.00	0.00
ATOM	1371	C9	MOL	19	15.500	45.730	40.260	1.00	0.00
ATOM	1372	C10	MOL	19	14.600	45.510	41.310	1.00	0.00
ATOM	1373	C11	MOL	19	14.630	51.340	38.000	1.00	0.00
ATOM	1374	C12	MOL	19	14.400	52.690	38.670	1.00	0.00
ATOM	1375	C13	MOL	19	16.460	43.480	40.110	1.00	0.00
ATOM	1376	H1	MOL	19	17.840	49.270	39.190	1.00	0.00
ATOM	1377	H2	MOL	19	18.310	50.270	40.580	1.00	0.00
ATOM	1378	H3	MOL	19	15.860	50.890	42.210	1.00	0.00
ATOM	1379	H4	MOL	19	17.550	51.550	38.410	1.00	0.00
ATOM	1380	H5	MOL	19	16.490	51.880	39.850	1.00	0.00
ATOM	1381	H6	MOL	19	14.400	49.140	43.370	1.00	0.00
ATOM	1382	H7	MOL	19	16.750	47.080	39.040	1.00	0.00
ATOM	1383	H8	MOL	19	13.520	46.530	42.870	1.00	0.00
ATOM	1384	H9	MOL	19	14.120	44.560	41.520	1.00	0.00
ATOM	1385	H10	MOL	19	15.760	49.670	37.910	1.00	0.00
ATOM	1386	H11	MOL	19	13.370	53.020	38.530	1.00	0.00
ATOM	1387	H12	MOL	19	14.570	52.710	39.760	1.00	0.00
ATOM	1388	H13	MOL	19	15.020	53.400	38.110	1.00	0.00
ATOM	1389	H14	MOL	19	15.730	43.000	40.780	1.00	0.00
ATOM	1390	H15	MOL	19	16.770	42.720	39.380	1.00	0.00
ATOM	1391	H16	MOL	19	17.310	43.770	40.740	1.00	0.00
ATOM	1392	O1	MOL	20	22.060	34.710	32.220	1.00	0.00
ATOM	1393	O2	MOL	20	20.840	43.630	31.710	1.00	0.00
ATOM	1394	N1	MOL	20	24.840	39.430	32.440	1.00	0.00
ATOM	1395	N2	MOL	20	21.040	41.400	31.980	1.00	0.00
ATOM	1396	C1	MOL	20	22.820	39.440	33.360	1.00	0.00
ATOM	1397	C2	MOL	20	23.060	38.140	32.750	1.00	0.00
ATOM	1398	C3	MOL	20	21.670	40.050	34.100	1.00	0.00
ATOM	1399	C4	MOL	20	24.310	38.200	32.110	1.00	0.00
ATOM	1400	C5	MOL	20	23.990	40.150	33.260	1.00	0.00
ATOM	1401	C6	MOL	20	21.050	41.290	33.440	1.00	0.00
ATOM	1402	C7	MOL	20	22.270	36.980	32.720	1.00	0.00
ATOM	1403	C8	MOL	20	24.850	37.010	31.610	1.00	0.00
ATOM	1404	C9	MOL	20	22.800	35.860	32.080	1.00	0.00
ATOM	1405	C10	MOL	20	24.090	35.840	31.530	1.00	0.00
ATOM	1406	C11	MOL	20	21.000	42.540	31.200	1.00	0.00
ATOM	1407	C12	MOL	20	21.250	42.420	29.700	1.00	0.00

ATOM	1408	C13	MOL	20	22.490	33.350	32.130	1.00	0.00
ATOM	1409	H1	MOL	20	21.940	40.240	35.140	1.00	0.00
ATOM	1410	H2	MOL	20	20.860	39.300	34.040	1.00	0.00
ATOM	1411	H3	MOL	20	24.380	41.040	33.750	1.00	0.00
ATOM	1412	H4	MOL	20	21.630	42.160	33.760	1.00	0.00
ATOM	1413	H5	MOL	20	20.060	41.520	33.840	1.00	0.00
ATOM	1414	H6	MOL	20	25.710	39.830	32.120	1.00	0.00
ATOM	1415	H7	MOL	20	21.280	36.980	33.150	1.00	0.00
ATOM	1416	H8	MOL	20	25.830	37.010	31.150	1.00	0.00
ATOM	1417	H9	MOL	20	24.470	34.950	31.030	1.00	0.00
ATOM	1418	H10	MOL	20	21.210	40.530	31.500	1.00	0.00
ATOM	1419	H11	MOL	20	22.280	42.040	29.660	1.00	0.00
ATOM	1420	H12	MOL	20	20.610	41.690	29.180	1.00	0.00
ATOM	1421	H13	MOL	20	21.370	43.440	29.320	1.00	0.00
ATOM	1422	H14	MOL	20	23.480	33.080	32.500	1.00	0.00
ATOM	1423	H15	MOL	20	21.600	32.880	32.550	1.00	0.00
ATOM	1424	H16	MOL	20	22.470	32.990	31.090	1.00	0.00
ATOM	1425	O1	MOL	21	33.950	46.300	30.750	1.00	0.00
ATOM	1426	O2	MOL	21	28.480	45.130	36.220	1.00	0.00
ATOM	1427	N1	MOL	21	29.460	43.110	30.310	1.00	0.00
ATOM	1428	N2	MOL	21	29.390	46.140	34.370	1.00	0.00
ATOM	1429	C1	MOL	21	29.120	45.070	31.380	1.00	0.00
ATOM	1430	C2	MOL	21	30.550	44.930	31.160	1.00	0.00
ATOM	1431	C3	MOL	21	28.350	46.190	32.020	1.00	0.00
ATOM	1432	C4	MOL	21	30.680	43.770	30.390	1.00	0.00
ATOM	1433	C5	MOL	21	28.510	43.930	30.890	1.00	0.00
ATOM	1434	C6	MOL	21	28.190	46.030	33.540	1.00	0.00
ATOM	1435	C7	MOL	21	31.650	45.790	31.270	1.00	0.00
ATOM	1436	C8	MOL	21	31.910	43.320	29.920	1.00	0.00
ATOM	1437	C9	MOL	21	32.910	45.410	30.800	1.00	0.00
ATOM	1438	C10	MOL	21	32.980	44.220	30.070	1.00	0.00
ATOM	1439	C11	MOL	21	29.330	45.850	35.720	1.00	0.00
ATOM	1440	C12	MOL	21	30.480	46.490	36.490	1.00	0.00
ATOM	1441	C13	MOL	21	33.890	47.730	30.650	1.00	0.00
ATOM	1442	H1	MOL	21	27.350	46.310	31.580	1.00	0.00
ATOM	1443	H2	MOL	21	28.910	47.070	31.690	1.00	0.00
ATOM	1444	H3	MOL	21	27.470	43.660	30.830	1.00	0.00
ATOM	1445	H4	MOL	21	27.560	45.150	33.740	1.00	0.00
ATOM	1446	H5	MOL	21	27.540	46.840	33.890	1.00	0.00
ATOM	1447	H6	MOL	21	29.320	42.200	29.900	1.00	0.00
ATOM	1448	H7	MOL	21	31.560	46.700	31.850	1.00	0.00
ATOM	1449	H8	MOL	21	31.930	42.350	29.440	1.00	0.00
ATOM	1450	H9	MOL	21	33.980	43.960	29.730	1.00	0.00
ATOM	1451	H10	MOL	21	30.220	46.590	34.010	1.00	0.00

ATOM	1452	H11	MOL	21	30.570	45.850	37.380	1.00	0.00
ATOM	1453	H12	MOL	21	31.430	46.440	35.960	1.00	0.00
ATOM	1454	H13	MOL	21	30.320	47.570	36.630	1.00	0.00
ATOM	1455	H14	MOL	21	34.870	48.180	30.430	1.00	0.00
ATOM	1456	H15	MOL	21	33.270	47.990	29.790	1.00	0.00
ATOM	1457	H16	MOL	21	33.550	48.200	31.580	1.00	0.00
ATOM	1458	O1	MOL	22	3.380	8.690	40.110	1.00	0.00
ATOM	1459	O2	MOL	22	7.880	9.080	33.200	1.00	0.00
ATOM	1460	N1	MOL	22	5.610	5.000	36.620	1.00	0.00
ATOM	1461	N2	MOL	22	9.420	8.900	34.900	1.00	0.00
ATOM	1462	C1	MOL	22	6.660	6.970	36.720	1.00	0.00
ATOM	1463	C2	MOL	22	5.450	7.110	37.520	1.00	0.00
ATOM	1464	C3	MOL	22	7.660	8.010	36.300	1.00	0.00
ATOM	1465	C4	MOL	22	4.820	5.870	37.370	1.00	0.00
ATOM	1466	C5	MOL	22	6.750	5.680	36.250	1.00	0.00
ATOM	1467	C6	MOL	22	8.910	7.650	35.470	1.00	0.00
ATOM	1468	C7	MOL	22	5.020	8.070	38.430	1.00	0.00
ATOM	1469	C8	MOL	22	3.640	5.610	38.070	1.00	0.00
ATOM	1470	C9	MOL	22	3.870	7.810	39.180	1.00	0.00
ATOM	1471	C10	MOL	22	3.160	6.630	38.900	1.00	0.00
ATOM	1472	C11	MOL	22	8.930	9.410	33.710	1.00	0.00
ATOM	1473	C12	MOL	22	9.890	10.490	33.210	1.00	0.00
ATOM	1474	C13	MOL	22	4.120	9.840	40.540	1.00	0.00
ATOM	1475	H1	MOL	22	8.110	8.490	37.180	1.00	0.00
ATOM	1476	H2	MOL	22	7.100	8.730	35.700	1.00	0.00
ATOM	1477	H3	MOL	22	7.440	5.210	35.550	1.00	0.00
ATOM	1478	H4	MOL	22	8.710	6.970	34.640	1.00	0.00
ATOM	1479	H5	MOL	22	9.650	7.110	36.060	1.00	0.00
ATOM	1480	H6	MOL	22	5.350	4.090	36.270	1.00	0.00
ATOM	1481	H7	MOL	22	5.450	9.070	38.400	1.00	0.00
ATOM	1482	H8	MOL	22	3.050	4.710	37.920	1.00	0.00
ATOM	1483	H9	MOL	22	2.250	6.520	39.470	1.00	0.00
ATOM	1484	H10	MOL	22	10.310	9.280	35.180	1.00	0.00
ATOM	1485	H11	MOL	22	9.310	11.180	32.600	1.00	0.00
ATOM	1486	H12	MOL	22	10.320	11.020	34.070	1.00	0.00
ATOM	1487	H13	MOL	22	10.600	10.040	32.500	1.00	0.00
ATOM	1488	H14	MOL	22	3.550	10.540	41.150	1.00	0.00
ATOM	1489	H15	MOL	22	5.110	9.620	40.960	1.00	0.00
ATOM	1490	H16	MOL	22	4.270	10.400	39.610	1.00	0.00
ATOM	1491	O1	MOL	23	42.460	40.010	38.430	1.00	0.00
ATOM	1492	O2	MOL	23	38.570	44.450	40.790	1.00	0.00
ATOM	1493	N1	MOL	23	44.110	42.030	43.290	1.00	0.00
ATOM	1494	N2	MOL	23	40.710	44.410	41.520	1.00	0.00
ATOM	1495	C1	MOL	23	41.910	42.110	42.920	1.00	0.00

ATOM	1496	C2	MOL	23	42.570	41.530	41.760	1.00	0.00
ATOM	1497	C3	MOL	23	40.520	42.610	43.200	1.00	0.00
ATOM	1498	C4	MOL	23	43.950	41.550	42.000	1.00	0.00
ATOM	1499	C5	MOL	23	42.880	42.270	43.880	1.00	0.00
ATOM	1500	C6	MOL	23	40.480	44.120	42.930	1.00	0.00
ATOM	1501	C7	MOL	23	42.020	40.990	40.590	1.00	0.00
ATOM	1502	C8	MOL	23	44.820	41.060	41.030	1.00	0.00
ATOM	1503	C9	MOL	23	42.900	40.470	39.640	1.00	0.00
ATOM	1504	C10	MOL	23	44.290	40.530	39.850	1.00	0.00
ATOM	1505	C11	MOL	23	39.750	44.560	40.540	1.00	0.00
ATOM	1506	C12	MOL	23	40.270	44.940	39.160	1.00	0.00
ATOM	1507	C13	MOL	23	43.300	39.590	37.350	1.00	0.00
ATOM	1508	H1	MOL	23	40.010	42.290	44.110	1.00	0.00
ATOM	1509	H2	MOL	23	39.840	42.230	42.430	1.00	0.00
ATOM	1510	H3	MOL	23	42.710	42.760	44.830	1.00	0.00
ATOM	1511	H4	MOL	23	41.100	44.710	43.630	1.00	0.00
ATOM	1512	H5	MOL	23	39.420	44.350	43.120	1.00	0.00
ATOM	1513	H6	MOL	23	45.030	42.140	43.680	1.00	0.00
ATOM	1514	H7	MOL	23	40.980	40.840	40.360	1.00	0.00
ATOM	1515	H8	MOL	23	45.890	41.050	41.200	1.00	0.00
ATOM	1516	H9	MOL	23	44.990	40.160	39.110	1.00	0.00
ATOM	1517	H10	MOL	23	41.670	44.630	41.290	1.00	0.00
ATOM	1518	H11	MOL	23	40.800	44.080	38.730	1.00	0.00
ATOM	1519	H12	MOL	23	39.440	45.290	38.530	1.00	0.00
ATOM	1520	H13	MOL	23	40.990	45.770	39.160	1.00	0.00
ATOM	1521	H14	MOL	23	43.750	38.670	37.740	1.00	0.00
ATOM	1522	H15	MOL	23	42.710	39.340	36.460	1.00	0.00
ATOM	1523	H16	MOL	23	44.040	40.370	37.130	1.00	0.00
ATOM	1524	O1	MOL	24	34.820	43.220	35.080	1.00	0.00
ATOM	1525	O2	MOL	24	44.270	46.230	34.180	1.00	0.00
ATOM	1526	N1	MOL	24	39.280	41.510	37.780	1.00	0.00
ATOM	1527	N2	MOL	24	42.470	45.060	34.720	1.00	0.00
ATOM	1528	C1	MOL	24	39.710	42.700	35.920	1.00	0.00
ATOM	1529	C2	MOL	24	38.260	42.650	36.050	1.00	0.00
ATOM	1530	C3	MOL	24	40.570	43.480	34.960	1.00	0.00
ATOM	1531	C4	MOL	24	38.070	41.880	37.200	1.00	0.00
ATOM	1532	C5	MOL	24	40.270	42.120	37.030	1.00	0.00
ATOM	1533	C6	MOL	24	41.290	44.710	35.530	1.00	0.00
ATOM	1534	C7	MOL	24	37.190	43.060	35.260	1.00	0.00
ATOM	1535	C8	MOL	24	36.820	41.390	37.580	1.00	0.00
ATOM	1536	C9	MOL	24	35.920	42.730	35.740	1.00	0.00
ATOM	1537	C10	MOL	24	35.740	41.850	36.810	1.00	0.00
ATOM	1538	C11	MOL	24	43.370	46.070	34.980	1.00	0.00
ATOM	1539	C12	MOL	24	43.110	46.930	36.210	1.00	0.00

ATOM	1540	C13	MOL	24	34.650	44.600	34.740	1.00	0.00
ATOM	1541	H1	MOL	24	40.000	43.810	34.090	1.00	0.00
ATOM	1542	H2	MOL	24	41.220	42.690	34.560	1.00	0.00
ATOM	1543	H3	MOL	24	41.290	41.910	37.310	1.00	0.00
ATOM	1544	H4	MOL	24	41.700	44.510	36.520	1.00	0.00
ATOM	1545	H5	MOL	24	40.640	45.600	35.520	1.00	0.00
ATOM	1546	H6	MOL	24	39.440	40.860	38.530	1.00	0.00
ATOM	1547	H7	MOL	24	37.390	43.620	34.350	1.00	0.00
ATOM	1548	H8	MOL	24	36.760	40.640	38.360	1.00	0.00
ATOM	1549	H9	MOL	24	34.700	41.650	37.040	1.00	0.00
ATOM	1550	H10	MOL	24	42.850	44.360	34.090	1.00	0.00
ATOM	1551	H11	MOL	24	44.010	47.540	36.310	1.00	0.00
ATOM	1552	H12	MOL	24	42.170	47.490	36.110	1.00	0.00
ATOM	1553	H13	MOL	24	42.980	46.260	37.070	1.00	0.00
ATOM	1554	H14	MOL	24	33.700	44.790	34.230	1.00	0.00
ATOM	1555	H15	MOL	24	34.650	45.290	35.590	1.00	0.00
ATOM	1556	H16	MOL	24	35.480	44.960	34.120	1.00	0.00
ATOM	1557	O1	MOL	25	44.730	51.900	21.500	1.00	0.00
ATOM	1558	O2	MOL	25	46.090	48.640	29.550	1.00	0.00
ATOM	1559	N1	MOL	25	42.120	49.760	25.720	1.00	0.00
ATOM	1560	N2	MOL	25	45.480	50.470	28.310	1.00	0.00
ATOM	1561	C1	MOL	25	44.360	49.510	25.770	1.00	0.00
ATOM	1562	C2	MOL	25	43.970	50.130	24.510	1.00	0.00
ATOM	1563	C3	MOL	25	45.740	49.170	26.270	1.00	0.00
ATOM	1564	C4	MOL	25	42.570	50.160	24.480	1.00	0.00
ATOM	1565	C5	MOL	25	43.200	49.350	26.480	1.00	0.00
ATOM	1566	C6	MOL	25	46.380	50.220	27.190	1.00	0.00
ATOM	1567	C7	MOL	25	44.650	50.790	23.490	1.00	0.00
ATOM	1568	C8	MOL	25	41.840	50.840	23.500	1.00	0.00
ATOM	1569	C9	MOL	25	43.910	51.340	22.440	1.00	0.00
ATOM	1570	C10	MOL	25	42.520	51.470	22.450	1.00	0.00
ATOM	1571	C11	MOL	25	45.460	49.680	29.450	1.00	0.00
ATOM	1572	C12	MOL	25	44.400	50.180	30.420	1.00	0.00
ATOM	1573	C13	MOL	25	44.450	51.750	20.100	1.00	0.00
ATOM	1574	H1	MOL	25	46.460	49.110	25.440	1.00	0.00
ATOM	1575	H2	MOL	25	45.750	48.170	26.710	1.00	0.00
ATOM	1576	H3	MOL	25	43.070	49.140	27.540	1.00	0.00
ATOM	1577	H4	MOL	25	46.580	51.140	26.640	1.00	0.00
ATOM	1578	H5	MOL	25	47.340	49.820	27.540	1.00	0.00
ATOM	1579	H6	MOL	25	41.180	49.900	26.050	1.00	0.00
ATOM	1580	H7	MOL	25	45.730	50.910	23.550	1.00	0.00
ATOM	1581	H8	MOL	25	40.780	51.040	23.580	1.00	0.00
ATOM	1582	H9	MOL	25	41.940	52.010	21.700	1.00	0.00
ATOM	1583	H10	MOL	25	44.780	51.210	28.260	1.00	0.00

ATOM	1584	H11	MOL	25	44.860	50.090	31.420	1.00	0.00
ATOM	1585	H12	MOL	25	44.240	51.220	30.140	1.00	0.00
ATOM	1586	H13	MOL	25	43.480	49.580	30.300	1.00	0.00
ATOM	1587	H14	MOL	25	45.280	52.110	19.490	1.00	0.00
ATOM	1588	H15	MOL	25	44.150	50.710	19.920	1.00	0.00
ATOM	1589	H16	MOL	25	43.590	52.410	19.890	1.00	0.00
ATOM	1590	O1	MOL	26	39.290	39.810	33.100	1.00	0.00
ATOM	1591	O2	MOL	26	31.780	41.280	35.360	1.00	0.00
ATOM	1592	N1	MOL	26	35.020	37.850	30.310	1.00	0.00
ATOM	1593	N2	MOL	26	32.120	41.870	33.240	1.00	0.00
ATOM	1594	C1	MOL	26	34.560	39.760	31.420	1.00	0.00
ATOM	1595	C2	MOL	26	35.960	39.430	31.610	1.00	0.00
ATOM	1596	C3	MOL	26	33.800	40.990	31.840	1.00	0.00
ATOM	1597	C4	MOL	26	36.190	38.210	30.960	1.00	0.00
ATOM	1598	C5	MOL	26	34.060	38.810	30.570	1.00	0.00
ATOM	1599	C6	MOL	26	32.910	40.660	33.050	1.00	0.00
ATOM	1600	C7	MOL	26	37.000	40.030	32.330	1.00	0.00
ATOM	1601	C8	MOL	26	37.390	37.500	30.990	1.00	0.00
ATOM	1602	C9	MOL	26	38.210	39.320	32.420	1.00	0.00
ATOM	1603	C10	MOL	26	38.390	38.100	31.760	1.00	0.00
ATOM	1604	C11	MOL	26	31.610	42.130	34.500	1.00	0.00
ATOM	1605	C12	MOL	26	30.770	43.400	34.630	1.00	0.00
ATOM	1606	C13	MOL	26	39.400	39.670	34.520	1.00	0.00
ATOM	1607	H1	MOL	26	33.080	41.330	31.080	1.00	0.00
ATOM	1608	H2	MOL	26	34.390	41.830	32.210	1.00	0.00
ATOM	1609	H3	MOL	26	33.090	38.620	30.130	1.00	0.00
ATOM	1610	H4	MOL	26	33.510	40.390	33.930	1.00	0.00
ATOM	1611	H5	MOL	26	32.270	39.790	32.920	1.00	0.00
ATOM	1612	H6	MOL	26	34.890	37.060	29.700	1.00	0.00
ATOM	1613	H7	MOL	26	36.870	40.970	32.850	1.00	0.00
ATOM	1614	H8	MOL	26	37.450	36.540	30.490	1.00	0.00
ATOM	1615	H9	MOL	26	39.330	37.580	31.940	1.00	0.00
ATOM	1616	H10	MOL	26	32.010	42.560	32.500	1.00	0.00
ATOM	1617	H11	MOL	26	30.590	43.560	35.700	1.00	0.00
ATOM	1618	H12	MOL	26	31.420	44.230	34.360	1.00	0.00
ATOM	1619	H13	MOL	26	29.880	43.460	33.990	1.00	0.00
ATOM	1620	H14	MOL	26	39.540	38.600	34.720	1.00	0.00
ATOM	1621	H15	MOL	26	40.160	40.310	34.980	1.00	0.00
ATOM	1622	H16	MOL	26	38.480	39.970	35.040	1.00	0.00
ATOM	1623	O1	MOL	27	34.010	37.550	24.560	1.00	0.00
ATOM	1624	O2	MOL	27	25.510	32.790	23.690	1.00	0.00
ATOM	1625	N1	MOL	27	33.110	32.990	21.620	1.00	0.00
ATOM	1626	N2	MOL	27	27.720	32.950	23.470	1.00	0.00
ATOM	1627	C1	MOL	27	31.370	33.710	22.820	1.00	0.00

ATOM	1628	C2	MOL	27	32.520	34.560	23.090	1.00	0.00
ATOM	1629	C3	MOL	27	29.980	33.760	23.400	1.00	0.00
ATOM	1630	C4	MOL	27	33.570	34.120	22.280	1.00	0.00
ATOM	1631	C5	MOL	27	31.750	32.860	21.810	1.00	0.00
ATOM	1632	C6	MOL	27	29.010	32.700	22.850	1.00	0.00
ATOM	1633	C7	MOL	27	32.650	35.690	23.910	1.00	0.00
ATOM	1634	C8	MOL	27	34.850	34.660	22.420	1.00	0.00
ATOM	1635	C9	MOL	27	33.840	36.410	23.820	1.00	0.00
ATOM	1636	C10	MOL	27	34.940	35.820	23.180	1.00	0.00
ATOM	1637	C11	MOL	27	26.480	32.570	22.990	1.00	0.00
ATOM	1638	C12	MOL	27	26.400	31.780	21.690	1.00	0.00
ATOM	1639	C13	MOL	27	34.420	38.780	23.950	1.00	0.00
ATOM	1640	H1	MOL	27	29.560	34.770	23.420	1.00	0.00
ATOM	1641	H2	MOL	27	30.120	33.440	24.440	1.00	0.00
ATOM	1642	H3	MOL	27	31.220	32.100	21.250	1.00	0.00
ATOM	1643	H4	MOL	27	29.350	31.730	23.240	1.00	0.00
ATOM	1644	H5	MOL	27	28.960	32.770	21.750	1.00	0.00
ATOM	1645	H6	MOL	27	33.670	32.480	20.960	1.00	0.00
ATOM	1646	H7	MOL	27	31.860	35.950	24.610	1.00	0.00
ATOM	1647	H8	MOL	27	35.710	34.200	21.930	1.00	0.00
ATOM	1648	H9	MOL	27	35.920	36.270	23.330	1.00	0.00
ATOM	1649	H10	MOL	27	27.670	33.320	24.410	1.00	0.00
ATOM	1650	H11	MOL	27	26.820	32.440	20.930	1.00	0.00
ATOM	1651	H12	MOL	27	27.070	30.920	21.830	1.00	0.00
ATOM	1652	H13	MOL	27	25.370	31.540	21.400	1.00	0.00
ATOM	1653	H14	MOL	27	33.590	39.400	23.600	1.00	0.00
ATOM	1654	H15	MOL	27	34.970	39.300	24.740	1.00	0.00
ATOM	1655	H16	MOL	27	35.190	38.640	23.180	1.00	0.00
ATOM	1656	O1	MOL	28	43.870	53.450	41.420	1.00	0.00
ATOM	1657	O2	MOL	28	47.410	51.750	31.900	1.00	0.00
ATOM	1658	N1	MOL	28	48.100	54.280	38.110	1.00	0.00
ATOM	1659	N2	MOL	28	46.810	51.920	34.120	1.00	0.00
ATOM	1660	C1	MOL	28	46.500	53.110	37.200	1.00	0.00
ATOM	1661	C2	MOL	28	46.120	53.390	38.580	1.00	0.00
ATOM	1662	C3	MOL	28	45.560	52.540	36.170	1.00	0.00
ATOM	1663	C4	MOL	28	47.180	54.130	39.130	1.00	0.00
ATOM	1664	C5	MOL	28	47.750	53.620	36.950	1.00	0.00
ATOM	1665	C6	MOL	28	46.210	51.410	35.350	1.00	0.00
ATOM	1666	C7	MOL	28	44.970	53.150	39.340	1.00	0.00
ATOM	1667	C8	MOL	28	47.210	54.510	40.470	1.00	0.00
ATOM	1668	C9	MOL	28	44.980	53.630	40.650	1.00	0.00
ATOM	1669	C10	MOL	28	46.080	54.250	41.250	1.00	0.00
ATOM	1670	C11	MOL	28	47.100	51.240	32.960	1.00	0.00
ATOM	1671	C12	MOL	28	46.980	49.720	33.000	1.00	0.00

ATOM	1672	C13	MOL	28	43.020	54.590	41.600	1.00	0.00
ATOM	1673	H1	MOL	28	45.170	53.300	35.490	1.00	0.00
ATOM	1674	H2	MOL	28	44.690	52.050	36.630	1.00	0.00
ATOM	1675	H3	MOL	28	48.300	53.620	36.020	1.00	0.00
ATOM	1676	H4	MOL	28	45.400	50.680	35.170	1.00	0.00
ATOM	1677	H5	MOL	28	46.950	50.900	35.970	1.00	0.00
ATOM	1678	H6	MOL	28	48.800	55.010	38.220	1.00	0.00
ATOM	1679	H7	MOL	28	44.060	52.690	38.960	1.00	0.00
ATOM	1680	H8	MOL	28	48.050	55.100	40.850	1.00	0.00
ATOM	1681	H9	MOL	28	46.050	54.630	42.270	1.00	0.00
ATOM	1682	H10	MOL	28	46.620	52.910	33.980	1.00	0.00
ATOM	1683	H11	MOL	28	47.490	49.360	33.900	1.00	0.00
ATOM	1684	H12	MOL	28	45.970	49.280	33.050	1.00	0.00
ATOM	1685	H13	MOL	28	47.530	49.230	32.190	1.00	0.00
ATOM	1686	H14	MOL	28	42.970	55.200	40.690	1.00	0.00
ATOM	1687	H15	MOL	28	42.030	54.310	41.970	1.00	0.00
ATOM	1688	H16	MOL	28	43.370	55.130	42.490	1.00	0.00
ATOM	1689	O1	MOL	29	27.640	52.800	34.090	1.00	0.00
ATOM	1690	O2	MOL	29	33.590	51.740	26.420	1.00	0.00
ATOM	1691	N1	MOL	29	28.900	49.920	29.590	1.00	0.00
ATOM	1692	N2	MOL	29	32.720	52.140	28.450	1.00	0.00
ATOM	1693	C1	MOL	29	29.950	51.840	29.750	1.00	0.00
ATOM	1694	C2	MOL	29	28.990	51.730	30.830	1.00	0.00
ATOM	1695	C3	MOL	29	30.710	53.080	29.370	1.00	0.00
ATOM	1696	C4	MOL	29	28.360	50.480	30.730	1.00	0.00
ATOM	1697	C5	MOL	29	29.910	50.670	29.020	1.00	0.00
ATOM	1698	C6	MOL	29	31.560	52.960	28.090	1.00	0.00
ATOM	1699	C7	MOL	29	28.690	52.470	31.980	1.00	0.00
ATOM	1700	C8	MOL	29	27.450	50.030	31.700	1.00	0.00
ATOM	1701	C9	MOL	29	27.830	52.010	32.980	1.00	0.00
ATOM	1702	C10	MOL	29	27.130	50.810	32.800	1.00	0.00
ATOM	1703	C11	MOL	29	33.540	51.440	27.600	1.00	0.00
ATOM	1704	C12	MOL	29	34.360	50.310	28.190	1.00	0.00
ATOM	1705	C13	MOL	29	26.400	52.780	34.810	1.00	0.00
ATOM	1706	H1	MOL	29	31.220	53.410	30.280	1.00	0.00
ATOM	1707	H2	MOL	29	30.010	53.900	29.160	1.00	0.00
ATOM	1708	H3	MOL	29	30.400	50.470	28.080	1.00	0.00
ATOM	1709	H4	MOL	29	31.880	53.950	27.730	1.00	0.00
ATOM	1710	H5	MOL	29	31.070	52.480	27.240	1.00	0.00
ATOM	1711	H6	MOL	29	28.450	49.160	29.110	1.00	0.00
ATOM	1712	H7	MOL	29	29.260	53.380	32.100	1.00	0.00
ATOM	1713	H8	MOL	29	26.930	49.090	31.500	1.00	0.00
ATOM	1714	H9	MOL	29	26.530	50.280	33.530	1.00	0.00
ATOM	1715	H10	MOL	29	32.710	51.670	29.350	1.00	0.00

ATOM	1716	H11	MOL	29	35.280	50.380	27.590	1.00	0.00
ATOM	1717	H12	MOL	29	34.570	50.570	29.240	1.00	0.00
ATOM	1718	H13	MOL	29	33.930	49.330	27.960	1.00	0.00
ATOM	1719	H14	MOL	29	25.640	53.200	34.140	1.00	0.00
ATOM	1720	H15	MOL	29	26.310	51.750	35.170	1.00	0.00
ATOM	1721	H16	MOL	29	26.540	53.430	35.680	1.00	0.00
ATOM	1722	O1	MOL	30	39.970	42.000	29.910	1.00	0.00
ATOM	1723	O2	MOL	30	40.800	49.810	34.630	1.00	0.00
ATOM	1724	N1	MOL	30	36.600	45.890	31.840	1.00	0.00
ATOM	1725	N2	MOL	30	41.480	48.970	32.610	1.00	0.00
ATOM	1726	C1	MOL	30	38.800	46.220	32.310	1.00	0.00
ATOM	1727	C2	MOL	30	38.670	44.990	31.550	1.00	0.00
ATOM	1728	C3	MOL	30	40.070	46.870	32.800	1.00	0.00
ATOM	1729	C4	MOL	30	37.320	44.900	31.190	1.00	0.00
ATOM	1730	C5	MOL	30	37.520	46.680	32.500	1.00	0.00
ATOM	1731	C6	MOL	30	40.220	48.310	32.280	1.00	0.00
ATOM	1732	C7	MOL	30	39.640	44.080	31.110	1.00	0.00
ATOM	1733	C8	MOL	30	36.830	43.750	30.570	1.00	0.00
ATOM	1734	C9	MOL	30	39.150	43.000	30.370	1.00	0.00
ATOM	1735	C10	MOL	30	37.800	42.880	30.050	1.00	0.00
ATOM	1736	C11	MOL	30	41.680	49.640	33.800	1.00	0.00
ATOM	1737	C12	MOL	30	43.120	50.030	34.090	1.00	0.00
ATOM	1738	C13	MOL	30	41.160	41.580	30.580	1.00	0.00
ATOM	1739	H1	MOL	30	40.970	46.330	32.470	1.00	0.00
ATOM	1740	H2	MOL	30	40.100	46.860	33.890	1.00	0.00
ATOM	1741	H3	MOL	30	37.110	47.490	33.090	1.00	0.00
ATOM	1742	H4	MOL	30	39.400	48.920	32.670	1.00	0.00
ATOM	1743	H5	MOL	30	40.170	48.240	31.190	1.00	0.00
ATOM	1744	H6	MOL	30	35.650	46.210	31.690	1.00	0.00
ATOM	1745	H7	MOL	30	40.640	44.120	31.520	1.00	0.00
ATOM	1746	H8	MOL	30	35.800	43.440	30.510	1.00	0.00
ATOM	1747	H9	MOL	30	37.550	41.960	29.520	1.00	0.00
ATOM	1748	H10	MOL	30	42.320	48.800	32.050	1.00	0.00
ATOM	1749	H11	MOL	30	43.740	49.140	34.260	1.00	0.00
ATOM	1750	H12	MOL	30	43.570	50.710	33.360	1.00	0.00
ATOM	1751	H13	MOL	30	43.230	50.580	35.030	1.00	0.00
ATOM	1752	H14	MOL	30	41.080	41.620	31.670	1.00	0.00
ATOM	1753	H15	MOL	30	41.420	40.570	30.220	1.00	0.00
ATOM	1754	H16	MOL	30	41.940	42.330	30.380	1.00	0.00
ATOM	1755	O1	MOL	31	38.590	42.390	17.740	1.00	0.00
ATOM	1756	O2	MOL	31	31.450	48.210	16.850	1.00	0.00
ATOM	1757	N1	MOL	31	33.110	41.880	17.970	1.00	0.00
ATOM	1758	N2	MOL	31	32.200	46.510	18.240	1.00	0.00
ATOM	1759	C1	MOL	31	33.880	43.940	17.470	1.00	0.00

ATOM	1760	C2	MOL	31	34.990	43.040	17.740	1.00	0.00
ATOM	1761	C3	MOL	31	33.850	45.290	16.800	1.00	0.00
ATOM	1762	C4	MOL	31	34.490	41.770	18.040	1.00	0.00
ATOM	1763	C5	MOL	31	32.760	43.190	17.700	1.00	0.00
ATOM	1764	C6	MOL	31	33.600	46.380	17.850	1.00	0.00
ATOM	1765	C7	MOL	31	36.360	43.270	17.600	1.00	0.00
ATOM	1766	C8	MOL	31	35.320	40.660	18.160	1.00	0.00
ATOM	1767	C9	MOL	31	37.240	42.200	17.800	1.00	0.00
ATOM	1768	C10	MOL	31	36.690	40.950	18.110	1.00	0.00
ATOM	1769	C11	MOL	31	31.340	47.520	17.850	1.00	0.00
ATOM	1770	C12	MOL	31	30.140	47.610	18.790	1.00	0.00
ATOM	1771	C13	MOL	31	39.190	43.120	16.670	1.00	0.00
ATOM	1772	H1	MOL	31	33.080	45.410	16.030	1.00	0.00
ATOM	1773	H2	MOL	31	34.830	45.470	16.340	1.00	0.00
ATOM	1774	H3	MOL	31	31.760	43.560	17.490	1.00	0.00
ATOM	1775	H4	MOL	31	33.910	47.360	17.480	1.00	0.00
ATOM	1776	H5	MOL	31	34.260	46.270	18.720	1.00	0.00
ATOM	1777	H6	MOL	31	32.500	41.090	18.100	1.00	0.00
ATOM	1778	H7	MOL	31	36.690	44.290	17.410	1.00	0.00
ATOM	1779	H8	MOL	31	34.920	39.660	18.280	1.00	0.00
ATOM	1780	H9	MOL	31	37.290	40.040	18.130	1.00	0.00
ATOM	1781	H10	MOL	31	31.900	45.880	18.980	1.00	0.00
ATOM	1782	H11	MOL	31	30.110	46.710	19.410	1.00	0.00
ATOM	1783	H12	MOL	31	30.260	48.520	19.380	1.00	0.00
ATOM	1784	H13	MOL	31	29.200	47.670	18.240	1.00	0.00
ATOM	1785	H14	MOL	31	38.880	44.170	16.630	1.00	0.00
ATOM	1786	H15	MOL	31	40.280	43.010	16.740	1.00	0.00
ATOM	1787	H16	MOL	31	38.920	42.750	15.670	1.00	0.00
ATOM	1788	O1	MOL	32	25.580	55.480	25.530	1.00	0.00
ATOM	1789	O2	MOL	32	28.030	56.640	33.900	1.00	0.00
ATOM	1790	N1	MOL	32	24.850	52.240	29.910	1.00	0.00
ATOM	1791	N2	MOL	32	26.090	57.190	32.820	1.00	0.00
ATOM	1792	C1	MOL	32	24.920	54.480	30.380	1.00	0.00
ATOM	1793	C2	MOL	32	25.140	54.290	28.950	1.00	0.00
ATOM	1794	C3	MOL	32	24.740	55.730	31.210	1.00	0.00
ATOM	1795	C4	MOL	32	25.000	52.920	28.720	1.00	0.00
ATOM	1796	C5	MOL	32	24.670	53.220	30.870	1.00	0.00
ATOM	1797	C6	MOL	32	25.900	55.880	32.200	1.00	0.00
ATOM	1798	C7	MOL	32	25.360	55.170	27.900	1.00	0.00
ATOM	1799	C8	MOL	32	25.120	52.380	27.430	1.00	0.00
ATOM	1800	C9	MOL	32	25.350	54.650	26.600	1.00	0.00
ATOM	1801	C10	MOL	32	25.300	53.270	26.360	1.00	0.00
ATOM	1802	C11	MOL	32	27.050	57.350	33.800	1.00	0.00
ATOM	1803	C12	MOL	32	26.780	58.520	34.730	1.00	0.00

ATOM	1804	C13	MOL	32	26.660	55.250	24.620	1.00	0.00
ATOM	1805	H1	MOL	32	23.770	55.620	31.710	1.00	0.00
ATOM	1806	H2	MOL	32	24.700	56.590	30.530	1.00	0.00
ATOM	1807	H3	MOL	32	24.470	52.980	31.900	1.00	0.00
ATOM	1808	H4	MOL	32	26.830	55.620	31.660	1.00	0.00
ATOM	1809	H5	MOL	32	25.680	55.200	33.030	1.00	0.00
ATOM	1810	H6	MOL	32	25.110	51.270	29.990	1.00	0.00
ATOM	1811	H7	MOL	32	25.550	56.230	28.050	1.00	0.00
ATOM	1812	H8	MOL	32	25.080	51.330	27.190	1.00	0.00
ATOM	1813	H9	MOL	32	25.190	53.010	25.320	1.00	0.00
ATOM	1814	H10	MOL	32	25.260	57.770	32.850	1.00	0.00
ATOM	1815	H11	MOL	32	26.890	59.400	34.080	1.00	0.00
ATOM	1816	H12	MOL	32	25.730	58.590	35.050	1.00	0.00
ATOM	1817	H13	MOL	32	27.470	58.520	35.580	1.00	0.00
ATOM	1818	H14	MOL	32	26.760	56.150	24.000	1.00	0.00
ATOM	1819	H15	MOL	32	27.580	55.060	25.190	1.00	0.00
ATOM	1820	H16	MOL	32	26.410	54.380	23.990	1.00	0.00
ATOM	1821	O1	MOL	33	16.980	37.270	37.820	1.00	0.00
ATOM	1822	O2	MOL	33	23.190	30.530	34.400	1.00	0.00
ATOM	1823	N1	MOL	33	22.290	37.340	36.310	1.00	0.00
ATOM	1824	N2	MOL	33	22.580	31.630	36.270	1.00	0.00
ATOM	1825	C1	MOL	33	21.410	35.290	36.350	1.00	0.00
ATOM	1826	C2	MOL	33	20.370	36.250	36.690	1.00	0.00
ATOM	1827	C3	MOL	33	21.370	33.790	36.490	1.00	0.00
ATOM	1828	C4	MOL	33	20.950	37.520	36.630	1.00	0.00
ATOM	1829	C5	MOL	33	22.550	36.010	36.080	1.00	0.00
ATOM	1830	C6	MOL	33	22.310	32.920	35.640	1.00	0.00
ATOM	1831	C7	MOL	33	19.020	36.140	37.020	1.00	0.00
ATOM	1832	C8	MOL	33	20.270	38.650	37.080	1.00	0.00
ATOM	1833	C9	MOL	33	18.310	37.260	37.460	1.00	0.00
ATOM	1834	C10	MOL	33	18.960	38.500	37.520	1.00	0.00
ATOM	1835	C11	MOL	33	23.050	30.510	35.610	1.00	0.00
ATOM	1836	C12	MOL	33	23.260	29.230	36.410	1.00	0.00
ATOM	1837	C13	MOL	33	16.150	36.140	37.520	1.00	0.00
ATOM	1838	H1	MOL	33	20.410	33.280	36.610	1.00	0.00
ATOM	1839	H2	MOL	33	21.790	33.630	37.500	1.00	0.00
ATOM	1840	H3	MOL	33	23.540	35.630	35.860	1.00	0.00
ATOM	1841	H4	MOL	33	23.220	33.510	35.510	1.00	0.00
ATOM	1842	H5	MOL	33	21.820	32.790	34.660	1.00	0.00
ATOM	1843	H6	MOL	33	22.950	38.100	36.200	1.00	0.00
ATOM	1844	H7	MOL	33	18.620	35.130	36.950	1.00	0.00
ATOM	1845	H8	MOL	33	20.770	39.600	37.280	1.00	0.00
ATOM	1846	H9	MOL	33	18.450	39.300	38.050	1.00	0.00
ATOM	1847	H10	MOL	33	22.430	31.510	37.260	1.00	0.00

ATOM	1848	H11	MOL	33	22.870	28.420	35.790	1.00	0.00
ATOM	1849	H12	MOL	33	24.340	29.060	36.520	1.00	0.00
ATOM	1850	H13	MOL	33	22.760	29.190	37.390	1.00	0.00
ATOM	1851	H14	MOL	33	16.590	35.180	37.810	1.00	0.00
ATOM	1852	H15	MOL	33	15.200	36.250	38.060	1.00	0.00
ATOM	1853	H16	MOL	33	15.870	36.160	36.460	1.00	0.00
ATOM	1854	O1	MOL	34	37.380	41.800	22.890	1.00	0.00
ATOM	1855	O2	MOL	34	35.550	35.530	28.400	1.00	0.00
ATOM	1856	N1	MOL	34	38.860	40.330	27.900	1.00	0.00
ATOM	1857	N2	MOL	34	37.150	36.200	26.990	1.00	0.00
ATOM	1858	C1	MOL	34	38.810	38.640	26.430	1.00	0.00
ATOM	1859	C2	MOL	34	38.490	39.890	25.760	1.00	0.00
ATOM	1860	C3	MOL	34	39.090	37.280	25.840	1.00	0.00
ATOM	1861	C4	MOL	34	38.580	40.940	26.690	1.00	0.00
ATOM	1862	C5	MOL	34	39.070	38.980	27.730	1.00	0.00
ATOM	1863	C6	MOL	34	37.990	36.210	25.800	1.00	0.00
ATOM	1864	C7	MOL	34	38.150	40.140	24.430	1.00	0.00
ATOM	1865	C8	MOL	34	38.220	42.250	26.370	1.00	0.00
ATOM	1866	C9	MOL	34	37.820	41.470	24.150	1.00	0.00
ATOM	1867	C10	MOL	34	37.810	42.530	25.070	1.00	0.00
ATOM	1868	C11	MOL	34	36.180	35.300	27.380	1.00	0.00
ATOM	1869	C12	MOL	34	35.760	34.200	26.400	1.00	0.00
ATOM	1870	C13	MOL	34	37.800	41.220	21.650	1.00	0.00
ATOM	1871	H1	MOL	34	39.830	36.810	26.490	1.00	0.00
ATOM	1872	H2	MOL	34	39.470	37.280	24.810	1.00	0.00
ATOM	1873	H3	MOL	34	39.360	38.360	28.570	1.00	0.00
ATOM	1874	H4	MOL	34	38.470	35.220	25.780	1.00	0.00
ATOM	1875	H5	MOL	34	37.370	36.310	24.900	1.00	0.00
ATOM	1876	H6	MOL	34	39.120	40.810	28.750	1.00	0.00
ATOM	1877	H7	MOL	34	37.980	39.340	23.720	1.00	0.00
ATOM	1878	H8	MOL	34	38.260	43.010	27.150	1.00	0.00
ATOM	1879	H9	MOL	34	37.450	43.510	24.730	1.00	0.00
ATOM	1880	H10	MOL	34	37.430	36.890	27.680	1.00	0.00
ATOM	1881	H11	MOL	34	34.680	34.060	26.530	1.00	0.00
ATOM	1882	H12	MOL	34	35.900	34.560	25.370	1.00	0.00
ATOM	1883	H13	MOL	34	36.270	33.260	26.630	1.00	0.00
ATOM	1884	H14	MOL	34	38.880	41.050	21.670	1.00	0.00
ATOM	1885	H15	MOL	34	37.330	40.230	21.530	1.00	0.00
ATOM	1886	H16	MOL	34	37.550	41.870	20.810	1.00	0.00
ATOM	1887	O1	MOL	35	37.160	36.300	37.730	1.00	0.00
ATOM	1888	O2	MOL	35	30.440	30.980	37.250	1.00	0.00
ATOM	1889	N1	MOL	35	32.400	38.540	36.280	1.00	0.00
ATOM	1890	N2	MOL	35	30.260	33.230	36.990	1.00	0.00
ATOM	1891	C1	MOL	35	32.220	36.540	37.210	1.00	0.00

ATOM	1892	C2	MOL	35	33.640	36.770	36.950	1.00	0.00
ATOM	1893	C3	MOL	35	31.520	35.290	37.650	1.00	0.00
ATOM	1894	C4	MOL	35	33.680	38.030	36.340	1.00	0.00
ATOM	1895	C5	MOL	35	31.500	37.620	36.770	1.00	0.00
ATOM	1896	C6	MOL	35	30.680	34.570	36.570	1.00	0.00
ATOM	1897	C7	MOL	35	34.760	36.090	37.420	1.00	0.00
ATOM	1898	C8	MOL	35	34.930	38.600	36.070	1.00	0.00
ATOM	1899	C9	MOL	35	35.980	36.730	37.180	1.00	0.00
ATOM	1900	C10	MOL	35	36.090	37.950	36.510	1.00	0.00
ATOM	1901	C11	MOL	35	30.900	32.020	36.810	1.00	0.00
ATOM	1902	C12	MOL	35	32.070	31.950	35.840	1.00	0.00
ATOM	1903	C13	MOL	35	37.340	35.720	39.030	1.00	0.00
ATOM	1904	H1	MOL	35	30.840	35.600	38.450	1.00	0.00
ATOM	1905	H2	MOL	35	32.280	34.600	38.040	1.00	0.00
ATOM	1906	H3	MOL	35	30.430	37.780	36.750	1.00	0.00
ATOM	1907	H4	MOL	35	31.320	34.430	35.690	1.00	0.00
ATOM	1908	H5	MOL	35	29.830	35.160	36.220	1.00	0.00
ATOM	1909	H6	MOL	35	32.130	39.450	35.930	1.00	0.00
ATOM	1910	H7	MOL	35	34.750	35.230	38.090	1.00	0.00
ATOM	1911	H8	MOL	35	35.020	39.560	35.580	1.00	0.00
ATOM	1912	H9	MOL	35	37.100	38.320	36.370	1.00	0.00
ATOM	1913	H10	MOL	35	29.430	33.150	37.560	1.00	0.00
ATOM	1914	H11	MOL	35	32.720	32.830	35.910	1.00	0.00
ATOM	1915	H12	MOL	35	32.630	31.040	36.110	1.00	0.00
ATOM	1916	H13	MOL	35	31.770	31.740	34.810	1.00	0.00
ATOM	1917	H14	MOL	35	36.910	34.710	39.000	1.00	0.00
ATOM	1918	H15	MOL	35	38.400	35.740	39.330	1.00	0.00
ATOM	1919	H16	MOL	35	36.750	36.290	39.760	1.00	0.00
ATOM	1920	O1	MOL	36	12.060	38.830	30.420	1.00	0.00
ATOM	1921	O2	MOL	36	16.500	43.800	32.780	1.00	0.00
ATOM	1922	N1	MOL	36	10.740	43.990	28.990	1.00	0.00
ATOM	1923	N2	MOL	36	14.510	44.810	32.080	1.00	0.00
ATOM	1924	C1	MOL	36	11.700	43.790	30.980	1.00	0.00
ATOM	1925	C2	MOL	36	11.600	42.450	30.410	1.00	0.00
ATOM	1926	C3	MOL	36	12.090	44.330	32.330	1.00	0.00
ATOM	1927	C4	MOL	36	11.070	42.650	29.140	1.00	0.00
ATOM	1928	C5	MOL	36	10.990	44.640	30.180	1.00	0.00
ATOM	1929	C6	MOL	36	13.530	43.970	32.750	1.00	0.00
ATOM	1930	C7	MOL	36	11.860	41.160	30.870	1.00	0.00
ATOM	1931	C8	MOL	36	10.920	41.570	28.260	1.00	0.00
ATOM	1932	C9	MOL	36	11.610	40.070	30.030	1.00	0.00
ATOM	1933	C10	MOL	36	11.200	40.280	28.710	1.00	0.00
ATOM	1934	C11	MOL	36	15.880	44.610	32.110	1.00	0.00
ATOM	1935	C12	MOL	36	16.610	45.580	31.180	1.00	0.00

ATOM	1936	C13	MOL	36	11.420	37.660	29.890	1.00	0.00
ATOM	1937	H1	MOL	36	11.390	43.970	33.090	1.00	0.00
ATOM	1938	H2	MOL	36	11.970	45.420	32.360	1.00	0.00
ATOM	1939	H3	MOL	36	10.800	45.680	30.410	1.00	0.00
ATOM	1940	H4	MOL	36	13.700	42.890	32.710	1.00	0.00
ATOM	1941	H5	MOL	36	13.670	44.260	33.800	1.00	0.00
ATOM	1942	H6	MOL	36	10.230	44.380	28.210	1.00	0.00
ATOM	1943	H7	MOL	36	12.070	40.980	31.930	1.00	0.00
ATOM	1944	H8	MOL	36	10.560	41.730	27.250	1.00	0.00
ATOM	1945	H9	MOL	36	11.010	39.460	28.020	1.00	0.00
ATOM	1946	H10	MOL	36	14.250	45.630	31.530	1.00	0.00
ATOM	1947	H11	MOL	36	16.290	46.570	31.530	1.00	0.00
ATOM	1948	H12	MOL	36	17.690	45.530	31.350	1.00	0.00
ATOM	1949	H13	MOL	36	16.370	45.330	30.140	1.00	0.00
ATOM	1950	H14	MOL	36	11.840	36.810	30.440	1.00	0.00
ATOM	1951	H15	MOL	36	10.370	37.540	30.160	1.00	0.00
ATOM	1952	H16	MOL	36	11.420	37.470	28.810	1.00	0.00
ATOM	1953	O1	MOL	37	28.370	39.730	34.760	1.00	0.00
ATOM	1954	O2	MOL	37	35.730	33.840	34.060	1.00	0.00
ATOM	1955	N1	MOL	37	29.270	34.770	32.540	1.00	0.00
ATOM	1956	N2	MOL	37	34.980	35.610	32.950	1.00	0.00
ATOM	1957	C1	MOL	37	31.160	35.990	32.800	1.00	0.00
ATOM	1958	C2	MOL	37	30.030	36.760	33.320	1.00	0.00
ATOM	1959	C3	MOL	37	32.600	36.380	32.590	1.00	0.00
ATOM	1960	C4	MOL	37	28.970	35.850	33.350	1.00	0.00
ATOM	1961	C5	MOL	37	30.600	34.880	32.220	1.00	0.00
ATOM	1962	C6	MOL	37	33.570	35.380	33.250	1.00	0.00
ATOM	1963	C7	MOL	37	29.850	38.060	33.800	1.00	0.00
ATOM	1964	C8	MOL	37	27.680	36.310	33.650	1.00	0.00
ATOM	1965	C9	MOL	37	28.590	38.490	34.220	1.00	0.00
ATOM	1966	C10	MOL	37	27.520	37.600	34.170	1.00	0.00
ATOM	1967	C11	MOL	37	35.950	34.990	33.710	1.00	0.00
ATOM	1968	C12	MOL	37	37.240	35.720	34.070	1.00	0.00
ATOM	1969	C13	MOL	37	28.280	40.880	33.910	1.00	0.00
ATOM	1970	H1	MOL	37	32.690	37.360	33.060	1.00	0.00
ATOM	1971	H2	MOL	37	32.890	36.300	31.540	1.00	0.00
ATOM	1972	H3	MOL	37	31.090	34.070	31.690	1.00	0.00
ATOM	1973	H4	MOL	37	33.340	34.340	33.020	1.00	0.00
ATOM	1974	H5	MOL	37	33.450	35.540	34.330	1.00	0.00
ATOM	1975	H6	MOL	37	28.660	34.010	32.260	1.00	0.00
ATOM	1976	H7	MOL	37	30.750	38.660	33.850	1.00	0.00
ATOM	1977	H8	MOL	37	26.770	35.740	33.460	1.00	0.00
ATOM	1978	H9	MOL	37	26.630	38.040	34.600	1.00	0.00
ATOM	1979	H10	MOL	37	35.160	36.600	32.880	1.00	0.00

ATOM	1980	H11	MOL	37	37.010	36.780	34.230	1.00	0.00
ATOM	1981	H12	MOL	37	37.450	35.270	35.050	1.00	0.00
ATOM	1982	H13	MOL	37	38.000	35.600	33.290	1.00	0.00
ATOM	1983	H14	MOL	37	28.530	41.770	34.500	1.00	0.00
ATOM	1984	H15	MOL	37	28.920	40.830	33.020	1.00	0.00
ATOM	1985	H16	MOL	37	27.220	40.870	33.630	1.00	0.00
ATOM	1986	O1	MOL	38	36.370	50.040	18.990	1.00	0.00
ATOM	1987	O2	MOL	38	32.470	42.370	21.400	1.00	0.00
ATOM	1988	N1	MOL	38	32.220	47.600	21.640	1.00	0.00
ATOM	1989	N2	MOL	38	34.040	43.930	21.230	1.00	0.00
ATOM	1990	C1	MOL	38	34.220	46.700	22.040	1.00	0.00
ATOM	1991	C2	MOL	38	34.430	47.880	21.200	1.00	0.00
ATOM	1992	C3	MOL	38	35.280	45.730	22.480	1.00	0.00
ATOM	1993	C4	MOL	38	33.150	48.410	21.010	1.00	0.00
ATOM	1994	C5	MOL	38	32.890	46.620	22.350	1.00	0.00
ATOM	1995	C6	MOL	38	35.350	44.480	21.590	1.00	0.00
ATOM	1996	C7	MOL	38	35.560	48.460	20.630	1.00	0.00
ATOM	1997	C8	MOL	38	32.960	49.540	20.210	1.00	0.00
ATOM	1998	C9	MOL	38	35.350	49.500	19.720	1.00	0.00
ATOM	1999	C10	MOL	38	34.090	50.090	19.590	1.00	0.00
ATOM	2000	C11	MOL	38	33.500	42.820	21.850	1.00	0.00
ATOM	2001	C12	MOL	38	34.020	42.370	23.210	1.00	0.00
ATOM	2002	C13	MOL	38	36.100	50.220	17.590	1.00	0.00
ATOM	2003	H1	MOL	38	34.880	45.340	23.430	1.00	0.00
ATOM	2004	H2	MOL	38	36.280	46.140	22.680	1.00	0.00
ATOM	2005	H3	MOL	38	32.390	45.930	23.010	1.00	0.00
ATOM	2006	H4	MOL	38	36.040	43.730	21.990	1.00	0.00
ATOM	2007	H5	MOL	38	35.800	44.850	20.670	1.00	0.00
ATOM	2008	H6	MOL	38	31.270	47.920	21.800	1.00	0.00
ATOM	2009	H7	MOL	38	36.560	48.060	20.770	1.00	0.00
ATOM	2010	H8	MOL	38	31.930	49.800	19.980	1.00	0.00
ATOM	2011	H9	MOL	38	34.080	51.030	19.050	1.00	0.00
ATOM	2012	H10	MOL	38	33.640	43.990	20.300	1.00	0.00
ATOM	2013	H11	MOL	38	35.100	42.150	23.230	1.00	0.00
ATOM	2014	H12	MOL	38	33.890	43.130	23.990	1.00	0.00
ATOM	2015	H13	MOL	38	33.440	41.450	23.400	1.00	0.00
ATOM	2016	H14	MOL	38	35.590	49.360	17.130	1.00	0.00
ATOM	2017	H15	MOL	38	35.520	51.150	17.570	1.00	0.00
ATOM	2018	H16	MOL	38	37.080	50.350	17.110	1.00	0.00
ATOM	2019	O1	MOL	39	19.690	0.630	31.460	1.00	0.00
ATOM	2020	O2	MOL	39	27.200	2.200	26.980	1.00	0.00
ATOM	2021	N1	MOL	39	22.070	5.070	29.330	1.00	0.00
ATOM	2022	N2	MOL	39	24.990	2.640	26.770	1.00	0.00
ATOM	2023	C1	MOL	39	22.270	3.280	28.020	1.00	0.00

ATOM	2024	C2	MOL	39	21.530	2.840	29.200	1.00	0.00
ATOM	2025	C3	MOL	39	22.450	2.530	26.740	1.00	0.00
ATOM	2026	C4	MOL	39	21.570	3.980	30.020	1.00	0.00
ATOM	2027	C5	MOL	39	22.430	4.640	28.070	1.00	0.00
ATOM	2028	C6	MOL	39	23.770	2.700	25.960	1.00	0.00
ATOM	2029	C7	MOL	39	20.920	1.670	29.650	1.00	0.00
ATOM	2030	C8	MOL	39	20.890	4.020	31.240	1.00	0.00
ATOM	2031	C9	MOL	39	20.430	1.670	30.960	1.00	0.00
ATOM	2032	C10	MOL	39	20.400	2.820	31.760	1.00	0.00
ATOM	2033	C11	MOL	39	26.270	2.560	26.280	1.00	0.00
ATOM	2034	C12	MOL	39	26.540	3.000	24.840	1.00	0.00
ATOM	2035	C13	MOL	39	20.250	-0.260	32.440	1.00	0.00
ATOM	2036	H1	MOL	39	21.610	2.670	26.050	1.00	0.00
ATOM	2037	H2	MOL	39	22.330	1.470	27.000	1.00	0.00
ATOM	2038	H3	MOL	39	22.840	5.290	27.320	1.00	0.00
ATOM	2039	H4	MOL	39	23.730	3.640	25.400	1.00	0.00
ATOM	2040	H5	MOL	39	23.730	1.930	25.190	1.00	0.00
ATOM	2041	H6	MOL	39	21.950	6.030	29.630	1.00	0.00
ATOM	2042	H7	MOL	39	20.900	0.750	29.070	1.00	0.00
ATOM	2043	H8	MOL	39	20.780	4.960	31.760	1.00	0.00
ATOM	2044	H9	MOL	39	19.860	2.820	32.700	1.00	0.00
ATOM	2045	H10	MOL	39	24.830	2.510	27.760	1.00	0.00
ATOM	2046	H11	MOL	39	25.800	2.640	24.110	1.00	0.00
ATOM	2047	H12	MOL	39	26.470	4.090	24.720	1.00	0.00
ATOM	2048	H13	MOL	39	27.550	2.640	24.580	1.00	0.00
ATOM	2049	H14	MOL	39	20.800	0.270	33.230	1.00	0.00
ATOM	2050	H15	MOL	39	19.420	-0.790	32.910	1.00	0.00
ATOM	2051	H16	MOL	39	20.990	-0.950	32.030	1.00	0.00
ATOM	2052	O1	MOL	40	35.870	47.370	26.930	1.00	0.00
ATOM	2053	O2	MOL	40	42.880	43.620	27.600	1.00	0.00
ATOM	2054	N1	MOL	40	38.880	48.640	22.610	1.00	0.00
ATOM	2055	N2	MOL	40	41.500	44.550	25.950	1.00	0.00
ATOM	2056	C1	MOL	40	39.460	46.720	23.600	1.00	0.00
ATOM	2057	C2	MOL	40	38.240	47.220	24.220	1.00	0.00
ATOM	2058	C3	MOL	40	40.280	45.520	24.000	1.00	0.00
ATOM	2059	C4	MOL	40	37.900	48.410	23.570	1.00	0.00
ATOM	2060	C5	MOL	40	39.750	47.570	22.570	1.00	0.00
ATOM	2061	C6	MOL	40	40.450	45.470	25.520	1.00	0.00
ATOM	2062	C7	MOL	40	37.470	46.840	25.320	1.00	0.00
ATOM	2063	C8	MOL	40	36.880	49.280	23.980	1.00	0.00
ATOM	2064	C9	MOL	40	36.400	47.660	25.710	1.00	0.00
ATOM	2065	C10	MOL	40	36.070	48.830	25.020	1.00	0.00
ATOM	2066	C11	MOL	40	42.070	44.440	27.200	1.00	0.00
ATOM	2067	C12	MOL	40	41.730	45.560	28.180	1.00	0.00

ATOM	2068	C13	MOL	40	36.650	47.070	28.100	1.00	0.00
ATOM	2069	H1	MOL	40	41.190	45.570	23.380	1.00	0.00
ATOM	2070	H2	MOL	40	39.760	44.580	23.810	1.00	0.00
ATOM	2071	H3	MOL	40	40.550	47.560	21.850	1.00	0.00
ATOM	2072	H4	MOL	40	39.550	45.330	26.120	1.00	0.00
ATOM	2073	H5	MOL	40	40.870	46.450	25.790	1.00	0.00
ATOM	2074	H6	MOL	40	38.790	49.320	21.870	1.00	0.00
ATOM	2075	H7	MOL	40	37.660	45.960	25.930	1.00	0.00
ATOM	2076	H8	MOL	40	36.770	50.210	23.430	1.00	0.00
ATOM	2077	H9	MOL	40	35.240	49.450	25.350	1.00	0.00
ATOM	2078	H10	MOL	40	41.760	43.760	25.370	1.00	0.00
ATOM	2079	H11	MOL	40	40.640	45.650	28.150	1.00	0.00
ATOM	2080	H12	MOL	40	41.980	45.360	29.230	1.00	0.00
ATOM	2081	H13	MOL	40	42.300	46.460	27.920	1.00	0.00
ATOM	2082	H14	MOL	40	37.180	46.120	27.930	1.00	0.00
ATOM	2083	H15	MOL	40	37.430	47.830	28.210	1.00	0.00
ATOM	2084	H16	MOL	40	36.080	46.880	29.020	1.00	0.00
ATOM	2085	O1	MOL	41	43.530	45.740	41.400	1.00	0.00
ATOM	2086	O2	MOL	41	51.020	49.160	40.050	1.00	0.00
ATOM	2087	N1	MOL	41	46.480	45.800	36.750	1.00	0.00
ATOM	2088	N2	MOL	41	48.920	49.500	39.300	1.00	0.00
ATOM	2089	C1	MOL	41	46.520	47.710	37.950	1.00	0.00
ATOM	2090	C2	MOL	41	45.800	46.680	38.690	1.00	0.00
ATOM	2091	C3	MOL	41	46.600	49.180	38.230	1.00	0.00
ATOM	2092	C4	MOL	41	45.770	45.530	37.910	1.00	0.00
ATOM	2093	C5	MOL	41	46.900	47.110	36.770	1.00	0.00
ATOM	2094	C6	MOL	41	48.040	49.720	38.160	1.00	0.00
ATOM	2095	C7	MOL	41	45.070	46.780	39.880	1.00	0.00
ATOM	2096	C8	MOL	41	45.020	44.400	38.260	1.00	0.00
ATOM	2097	C9	MOL	41	44.230	45.720	40.230	1.00	0.00
ATOM	2098	C10	MOL	41	44.210	44.600	39.390	1.00	0.00
ATOM	2099	C11	MOL	41	50.260	49.820	39.370	1.00	0.00
ATOM	2100	C12	MOL	41	50.640	51.170	38.760	1.00	0.00
ATOM	2101	C13	MOL	41	44.240	45.860	42.640	1.00	0.00
ATOM	2102	H1	MOL	41	46.160	49.470	39.200	1.00	0.00
ATOM	2103	H2	MOL	41	45.910	49.630	37.510	1.00	0.00
ATOM	2104	H3	MOL	41	47.320	47.590	35.890	1.00	0.00
ATOM	2105	H4	MOL	41	47.920	50.810	38.070	1.00	0.00
ATOM	2106	H5	MOL	41	48.610	49.370	37.290	1.00	0.00
ATOM	2107	H6	MOL	41	46.440	45.240	35.910	1.00	0.00
ATOM	2108	H7	MOL	41	45.040	47.690	40.480	1.00	0.00
ATOM	2109	H8	MOL	41	44.860	43.500	37.670	1.00	0.00
ATOM	2110	H9	MOL	41	43.450	43.910	39.740	1.00	0.00
ATOM	2111	H10	MOL	41	48.660	48.710	39.880	1.00	0.00

ATOM	2112	H11	MOL	41	50.600	51.160	37.660	1.00	0.00
ATOM	2113	H12	MOL	41	49.860	51.860	39.120	1.00	0.00
ATOM	2114	H13	MOL	41	51.570	51.570	39.170	1.00	0.00
ATOM	2115	H14	MOL	41	43.500	45.870	43.450	1.00	0.00
ATOM	2116	H15	MOL	41	45.040	45.120	42.750	1.00	0.00
ATOM	2117	H16	MOL	41	44.690	46.860	42.740	1.00	0.00
ATOM	2118	O1	MOL	42	31.050	36.380	27.940	1.00	0.00
ATOM	2119	O2	MOL	42	30.270	31.460	31.960	1.00	0.00
ATOM	2120	N1	MOL	42	26.250	34.250	26.640	1.00	0.00
ATOM	2121	N2	MOL	42	28.170	32.120	31.870	1.00	0.00
ATOM	2122	C1	MOL	42	26.940	33.780	28.760	1.00	0.00
ATOM	2123	C2	MOL	42	28.020	34.420	28.020	1.00	0.00
ATOM	2124	C3	MOL	42	26.910	33.310	30.180	1.00	0.00
ATOM	2125	C4	MOL	42	27.560	34.670	26.730	1.00	0.00
ATOM	2126	C5	MOL	42	25.890	33.740	27.870	1.00	0.00
ATOM	2127	C6	MOL	42	28.150	32.430	30.440	1.00	0.00
ATOM	2128	C7	MOL	42	29.260	34.950	28.410	1.00	0.00
ATOM	2129	C8	MOL	42	28.280	35.410	25.780	1.00	0.00
ATOM	2130	C9	MOL	42	29.990	35.670	27.460	1.00	0.00
ATOM	2131	C10	MOL	42	29.550	35.880	26.150	1.00	0.00
ATOM	2132	C11	MOL	42	29.210	31.540	32.560	1.00	0.00
ATOM	2133	C12	MOL	42	28.900	31.010	33.960	1.00	0.00
ATOM	2134	C13	MOL	42	32.200	35.640	28.350	1.00	0.00
ATOM	2135	H1	MOL	42	26.010	32.760	30.490	1.00	0.00
ATOM	2136	H2	MOL	42	26.980	34.250	30.750	1.00	0.00
ATOM	2137	H3	MOL	42	24.870	33.430	28.050	1.00	0.00
ATOM	2138	H4	MOL	42	29.140	32.850	30.230	1.00	0.00
ATOM	2139	H5	MOL	42	27.950	31.490	29.920	1.00	0.00
ATOM	2140	H6	MOL	42	25.720	34.430	25.800	1.00	0.00
ATOM	2141	H7	MOL	42	29.560	34.850	29.450	1.00	0.00
ATOM	2142	H8	MOL	42	27.820	35.700	24.840	1.00	0.00
ATOM	2143	H9	MOL	42	30.210	36.470	25.520	1.00	0.00
ATOM	2144	H10	MOL	42	27.290	31.810	32.270	1.00	0.00
ATOM	2145	H11	MOL	42	29.660	30.310	34.330	1.00	0.00
ATOM	2146	H12	MOL	42	28.880	31.820	34.700	1.00	0.00
ATOM	2147	H13	MOL	42	27.900	30.560	33.990	1.00	0.00
ATOM	2148	H14	MOL	42	32.830	35.450	27.470	1.00	0.00
ATOM	2149	H15	MOL	42	32.860	36.280	28.950	1.00	0.00
ATOM	2150	H16	MOL	42	31.940	34.710	28.870	1.00	0.00
ATOM	2151	O1	MOL	43	50.380	36.890	45.140	1.00	0.00
ATOM	2152	O2	MOL	43	51.380	34.780	36.980	1.00	0.00
ATOM	2153	N1	MOL	43	53.120	39.660	41.200	1.00	0.00
ATOM	2154	N2	MOL	43	49.840	36.020	37.980	1.00	0.00
ATOM	2155	C1	MOL	43	51.380	38.400	40.520	1.00	0.00

ATOM	2156	C2	MOL	43	51.560	38.180	41.940	1.00	0.00
ATOM	2157	C3	MOL	43	50.280	37.900	39.620	1.00	0.00
ATOM	2158	C4	MOL	43	52.590	39.050	42.330	1.00	0.00
ATOM	2159	C5	MOL	43	52.270	39.390	40.160	1.00	0.00
ATOM	2160	C6	MOL	43	50.810	36.670	38.860	1.00	0.00
ATOM	2161	C7	MOL	43	50.810	37.390	42.830	1.00	0.00
ATOM	2162	C8	MOL	43	52.910	39.200	43.680	1.00	0.00
ATOM	2163	C9	MOL	43	51.080	37.590	44.180	1.00	0.00
ATOM	2164	C10	MOL	43	52.150	38.410	44.550	1.00	0.00
ATOM	2165	C11	MOL	43	50.220	35.150	36.980	1.00	0.00
ATOM	2166	C12	MOL	43	49.170	34.600	36.020	1.00	0.00
ATOM	2167	C13	MOL	43	49.490	37.410	46.130	1.00	0.00
ATOM	2168	H1	MOL	43	50.010	38.690	38.910	1.00	0.00
ATOM	2169	H2	MOL	43	49.330	37.660	40.110	1.00	0.00
ATOM	2170	H3	MOL	43	52.350	40.040	39.300	1.00	0.00
ATOM	2171	H4	MOL	43	51.180	35.930	39.580	1.00	0.00
ATOM	2172	H5	MOL	43	51.700	36.930	38.270	1.00	0.00
ATOM	2173	H6	MOL	43	53.840	40.370	41.230	1.00	0.00
ATOM	2174	H7	MOL	43	50.020	36.780	42.410	1.00	0.00
ATOM	2175	H8	MOL	43	53.710	39.860	43.990	1.00	0.00
ATOM	2176	H9	MOL	43	52.400	38.500	45.600	1.00	0.00
ATOM	2177	H10	MOL	43	48.880	36.290	38.100	1.00	0.00
ATOM	2178	H11	MOL	43	48.730	35.480	35.530	1.00	0.00
ATOM	2179	H12	MOL	43	49.490	33.820	35.320	1.00	0.00
ATOM	2180	H13	MOL	43	48.400	34.110	36.620	1.00	0.00
ATOM	2181	H14	MOL	43	48.890	38.240	45.760	1.00	0.00
ATOM	2182	H15	MOL	43	48.840	36.640	46.560	1.00	0.00
ATOM	2183	H16	MOL	43	50.120	37.800	46.940	1.00	0.00
ATOM	2184	O1	MOL	44	18.140	30.600	37.720	1.00	0.00
ATOM	2185	O2	MOL	44	16.160	24.290	35.670	1.00	0.00
ATOM	2186	N1	MOL	44	20.990	27.340	34.340	1.00	0.00
ATOM	2187	N2	MOL	44	17.620	25.390	37.100	1.00	0.00
ATOM	2188	C1	MOL	44	20.390	26.340	36.270	1.00	0.00
ATOM	2189	C2	MOL	44	19.940	27.730	36.300	1.00	0.00
ATOM	2190	C3	MOL	44	20.110	25.290	37.310	1.00	0.00
ATOM	2191	C4	MOL	44	20.350	28.310	35.090	1.00	0.00
ATOM	2192	C5	MOL	44	21.010	26.160	35.060	1.00	0.00
ATOM	2193	C6	MOL	44	18.800	24.530	37.040	1.00	0.00
ATOM	2194	C7	MOL	44	19.090	28.450	37.140	1.00	0.00
ATOM	2195	C8	MOL	44	20.040	29.630	34.750	1.00	0.00
ATOM	2196	C9	MOL	44	18.800	29.780	36.840	1.00	0.00
ATOM	2197	C10	MOL	44	19.260	30.360	35.650	1.00	0.00
ATOM	2198	C11	MOL	44	16.390	25.110	36.540	1.00	0.00
ATOM	2199	C12	MOL	44	15.290	25.950	37.190	1.00	0.00

ATOM	2200	C13	MOL	44	17.220	31.650	37.410	1.00	0.00
ATOM	2201	H1	MOL	44	20.110	25.810	38.280	1.00	0.00
ATOM	2202	H2	MOL	44	20.910	24.540	37.370	1.00	0.00
ATOM	2203	H3	MOL	44	21.340	25.260	34.560	1.00	0.00
ATOM	2204	H4	MOL	44	18.640	23.780	37.820	1.00	0.00
ATOM	2205	H5	MOL	44	18.880	24.040	36.060	1.00	0.00
ATOM	2206	H6	MOL	44	21.550	27.600	33.540	1.00	0.00
ATOM	2207	H7	MOL	44	18.750	27.980	38.060	1.00	0.00
ATOM	2208	H8	MOL	44	20.280	30.020	33.760	1.00	0.00
ATOM	2209	H9	MOL	44	19.060	31.410	35.460	1.00	0.00
ATOM	2210	H10	MOL	44	17.640	25.910	37.960	1.00	0.00
ATOM	2211	H11	MOL	44	15.630	26.980	37.330	1.00	0.00
ATOM	2212	H12	MOL	44	14.450	25.980	36.490	1.00	0.00
ATOM	2213	H13	MOL	44	14.950	25.450	38.100	1.00	0.00
ATOM	2214	H14	MOL	44	16.890	31.510	36.380	1.00	0.00
ATOM	2215	H15	MOL	44	16.320	31.480	38.020	1.00	0.00
ATOM	2216	H16	MOL	44	17.700	32.610	37.640	1.00	0.00
ATOM	2217	OW	SOL	45	50.980	35.340	2.180	1.00	0.00
ATOM	2218	HW1	SOL	45	51.620	34.730	2.550	1.00	0.00
ATOM	2219	HW2	SOL	45	50.800	35.000	1.310	1.00	0.00
ATOM	2220	OW	SOL	46	54.700	18.590	7.480	1.00	0.00
ATOM	2221	HW1	SOL	46	55.180	18.810	8.280	1.00	0.00
ATOM	2222	HW2	SOL	46	54.230	19.390	7.250	1.00	0.00
ATOM	2223	OW	SOL	47	8.730	14.050	1.090	1.00	0.00
ATOM	2224	HW1	SOL	47	9.600	14.190	1.460	1.00	0.00
ATOM	2225	HW2	SOL	47	8.320	14.910	1.100	1.00	0.00
ATOM	2226	OW	SOL	48	21.940	30.440	15.270	1.00	0.00
ATOM	2227	HW1	SOL	48	22.570	31.010	14.850	1.00	0.00
ATOM	2228	HW2	SOL	48	22.290	29.560	15.140	1.00	0.00
ATOM	2229	OW	SOL	49	13.910	19.080	12.200	1.00	0.00
ATOM	2230	HW1	SOL	49	14.650	18.600	12.570	1.00	0.00
ATOM	2231	HW2	SOL	49	14.210	19.990	12.160	1.00	0.00
ATOM	2232	OW	SOL	50	28.550	18.450	35.760	1.00	0.00
ATOM	2233	HW1	SOL	50	27.960	17.700	35.850	1.00	0.00
ATOM	2234	HW2	SOL	50	28.030	19.100	35.290	1.00	0.00
ATOM	2235	OW	SOL	51	17.770	2.950	11.540	1.00	0.00
ATOM	2236	HW1	SOL	51	18.180	3.050	12.400	1.00	0.00
ATOM	2237	HW2	SOL	51	18.450	2.550	11.000	1.00	0.00
ATOM	2238	OW	SOL	52	21.310	21.960	53.510	1.00	0.00
ATOM	2239	HW1	SOL	52	21.310	21.030	53.280	1.00	0.00
ATOM	2240	HW2	SOL	52	21.190	22.420	52.680	1.00	0.00
ATOM	2241	OW	SOL	53	1.420	49.320	44.650	1.00	0.00
ATOM	2242	HW1	SOL	53	2.110	49.230	43.990	1.00	0.00
ATOM	2243	HW2	SOL	53	1.530	48.570	45.230	1.00	0.00

ATOM	2244	OW	SOL	54	4.650	51.700	1.530	1.00	0.00
ATOM	2245	HW1	SOL	54	5.540	52.030	1.610	1.00	0.00
ATOM	2246	HW2	SOL	54	4.110	52.370	1.940	1.00	0.00
ATOM	2247	OW	SOL	55	0.660	20.750	50.710	1.00	0.00
ATOM	2248	HW1	SOL	55	-0.020	20.180	50.340	1.00	0.00
ATOM	2249	HW2	SOL	55	1.470	20.270	50.570	1.00	0.00
ATOM	2250	OW	SOL	56	16.750	27.600	17.400	1.00	0.00
ATOM	2251	HW1	SOL	56	17.310	28.360	17.290	1.00	0.00
ATOM	2252	HW2	SOL	56	16.860	27.110	16.580	1.00	0.00
ATOM	2253	OW	SOL	57	47.030	43.270	18.090	1.00	0.00
ATOM	2254	HW1	SOL	57	46.380	42.750	18.560	1.00	0.00
ATOM	2255	HW2	SOL	57	46.530	43.700	17.400	1.00	0.00
ATOM	2256	OW	SOL	58	46.760	10.600	13.560	1.00	0.00
ATOM	2257	HW1	SOL	58	46.100	11.120	13.110	1.00	0.00
ATOM	2258	HW2	SOL	58	46.290	9.830	13.880	1.00	0.00
ATOM	2259	OW	SOL	59	5.500	27.120	15.470	1.00	0.00
ATOM	2260	HW1	SOL	59	5.700	26.320	14.980	1.00	0.00
ATOM	2261	HW2	SOL	59	5.330	27.780	14.800	1.00	0.00
ATOM	2262	OW	SOL	60	46.690	40.260	34.030	1.00	0.00
ATOM	2263	HW1	SOL	60	47.220	40.200	33.230	1.00	0.00
ATOM	2264	HW2	SOL	60	46.800	41.160	34.320	1.00	0.00
ATOM	2265	OW	SOL	61	14.440	31.340	33.120	1.00	0.00
ATOM	2266	HW1	SOL	61	14.420	31.000	32.230	1.00	0.00
ATOM	2267	HW2	SOL	61	14.030	32.210	33.050	1.00	0.00
ATOM	2268	OW	SOL	62	11.070	2.360	33.490	1.00	0.00
ATOM	2269	HW1	SOL	62	11.650	2.840	32.890	1.00	0.00
ATOM	2270	HW2	SOL	62	10.190	2.590	33.200	1.00	0.00
ATOM	2271	OW	SOL	63	48.620	4.350	35.580	1.00	0.00
ATOM	2272	HW1	SOL	63	48.390	3.560	36.070	1.00	0.00
ATOM	2273	HW2	SOL	63	47.790	4.820	35.480	1.00	0.00
ATOM	2274	OW	SOL	64	36.310	19.580	7.680	1.00	0.00
ATOM	2275	HW1	SOL	64	35.870	20.200	7.080	1.00	0.00
ATOM	2276	HW2	SOL	64	36.910	19.100	7.120	1.00	0.00
ATOM	2277	OW	SOL	65	33.890	43.620	46.830	1.00	0.00
ATOM	2278	HW1	SOL	65	34.770	43.750	46.480	1.00	0.00
ATOM	2279	HW2	SOL	65	34.020	43.400	47.750	1.00	0.00
ATOM	2280	OW	SOL	66	5.660	47.560	38.750	1.00	0.00
ATOM	2281	HW1	SOL	66	5.330	47.240	39.590	1.00	0.00
ATOM	2282	HW2	SOL	66	5.430	46.870	38.120	1.00	0.00
ATOM	2283	OW	SOL	67	36.510	9.920	18.990	1.00	0.00
ATOM	2284	HW1	SOL	67	36.770	10.670	18.460	1.00	0.00
ATOM	2285	HW2	SOL	67	37.260	9.330	18.950	1.00	0.00
ATOM	2286	OW	SOL	68	23.520	0.670	53.380	1.00	0.00
ATOM	2287	HW1	SOL	68	22.920	-0.060	53.280	1.00	0.00

ATOM	2288	HW2	SOL	68	23.460	1.140	52.550	1.00	0.00
ATOM	2289	OW	SOL	69	15.250	0.120	55.650	1.00	0.00
ATOM	2290	HW1	SOL	69	15.670	0.930	55.380	1.00	0.00
ATOM	2291	HW2	SOL	69	14.930	0.300	56.540	1.00	0.00
ATOM	2292	OW	SOL	70	46.990	33.280	20.970	1.00	0.00
ATOM	2293	HW1	SOL	70	47.090	33.290	20.020	1.00	0.00
ATOM	2294	HW2	SOL	70	47.440	34.070	21.260	1.00	0.00
ATOM	2295	OW	SOL	71	46.030	9.160	18.330	1.00	0.00
ATOM	2296	HW1	SOL	71	45.120	9.110	18.600	1.00	0.00
ATOM	2297	HW2	SOL	71	46.000	9.160	17.370	1.00	0.00
ATOM	2298	OW	SOL	72	43.360	4.320	54.240	1.00	0.00
ATOM	2299	HW1	SOL	72	44.290	4.490	54.100	1.00	0.00
ATOM	2300	HW2	SOL	72	42.930	4.710	53.480	1.00	0.00
ATOM	2301	OW	SOL	73	13.380	48.560	4.100	1.00	0.00
ATOM	2302	HW1	SOL	73	14.230	48.560	3.660	1.00	0.00
ATOM	2303	HW2	SOL	73	12.960	49.370	3.810	1.00	0.00
ATOM	2304	OW	SOL	74	53.820	51.530	29.860	1.00	0.00
ATOM	2305	HW1	SOL	74	54.250	52.240	30.350	1.00	0.00
ATOM	2306	HW2	SOL	74	54.390	50.780	29.970	1.00	0.00
ATOM	2307	OW	SOL	75	47.940	6.160	21.180	1.00	0.00
ATOM	2308	HW1	SOL	75	47.490	6.660	21.860	1.00	0.00
ATOM	2309	HW2	SOL	75	47.850	5.250	21.450	1.00	0.00
ATOM	2310	OW	SOL	76	0.720	29.550	51.340	1.00	0.00
ATOM	2311	HW1	SOL	76	0.800	30.470	51.600	1.00	0.00
ATOM	2312	HW2	SOL	76	1.490	29.380	50.800	1.00	0.00
ATOM	2313	OW	SOL	77	29.350	53.990	48.020	1.00	0.00
ATOM	2314	HW1	SOL	77	30.310	53.990	48.110	1.00	0.00
ATOM	2315	HW2	SOL	77	29.030	53.660	48.860	1.00	0.00
ATOM	2316	OW	SOL	78	50.480	35.480	9.210	1.00	0.00
ATOM	2317	HW1	SOL	78	49.710	35.370	8.650	1.00	0.00
ATOM	2318	HW2	SOL	78	50.280	34.990	10.010	1.00	0.00
ATOM	2319	OW	SOL	79	0.730	16.030	22.960	1.00	0.00
ATOM	2320	HW1	SOL	79	1.250	16.700	23.400	1.00	0.00
ATOM	2321	HW2	SOL	79	0.800	15.260	23.530	1.00	0.00
ATOM	2322	OW	SOL	80	42.370	53.010	1.670	1.00	0.00
ATOM	2323	HW1	SOL	80	41.700	53.580	1.300	1.00	0.00
ATOM	2324	HW2	SOL	80	42.620	52.430	0.950	1.00	0.00
ATOM	2325	OW	SOL	81	19.740	3.090	36.930	1.00	0.00
ATOM	2326	HW1	SOL	81	19.020	3.530	36.480	1.00	0.00
ATOM	2327	HW2	SOL	81	19.540	3.200	37.860	1.00	0.00
ATOM	2328	OW	SOL	82	4.000	13.960	22.150	1.00	0.00
ATOM	2329	HW1	SOL	82	3.430	13.930	21.380	1.00	0.00
ATOM	2330	HW2	SOL	82	3.630	14.650	22.700	1.00	0.00
ATOM	2331	OW	SOL	83	44.230	29.700	45.720	1.00	0.00

ATOM	2332	HW1	SOL	83	43.600	29.700	45.000	1.00	0.00
ATOM	2333	HW2	SOL	83	43.800	30.200	46.420	1.00	0.00
ATOM	2334	OW	SOL	84	3.090	33.240	0.480	1.00	0.00
ATOM	2335	HW1	SOL	84	2.170	33.410	0.280	1.00	0.00
ATOM	2336	HW2	SOL	84	3.230	32.330	0.220	1.00	0.00
ATOM	2337	OW	SOL	85	52.460	40.170	1.180	1.00	0.00
ATOM	2338	HW1	SOL	85	51.940	39.560	1.710	1.00	0.00
ATOM	2339	HW2	SOL	85	53.070	40.570	1.790	1.00	0.00
ATOM	2340	OW	SOL	86	13.480	29.550	21.120	1.00	0.00
ATOM	2341	HW1	SOL	86	12.670	29.120	21.380	1.00	0.00
ATOM	2342	HW2	SOL	86	13.680	29.200	20.260	1.00	0.00
ATOM	2343	OW	SOL	87	40.450	28.130	24.070	1.00	0.00
ATOM	2344	HW1	SOL	87	39.730	27.830	24.630	1.00	0.00
ATOM	2345	HW2	SOL	87	41.040	28.590	24.660	1.00	0.00
ATOM	2346	OW	SOL	88	14.060	23.510	2.460	1.00	0.00
ATOM	2347	HW1	SOL	88	13.570	23.230	3.240	1.00	0.00
ATOM	2348	HW2	SOL	88	13.940	22.800	1.830	1.00	0.00
ATOM	2349	OW	SOL	89	11.530	52.990	51.360	1.00	0.00
ATOM	2350	HW1	SOL	89	10.610	52.830	51.560	1.00	0.00
ATOM	2351	HW2	SOL	89	12.000	52.820	52.180	1.00	0.00
ATOM	2352	OW	SOL	90	6.810	47.200	11.520	1.00	0.00
ATOM	2353	HW1	SOL	90	7.350	46.410	11.530	1.00	0.00
ATOM	2354	HW2	SOL	90	7.450	47.920	11.560	1.00	0.00
ATOM	2355	OW	SOL	91	46.690	36.090	18.210	1.00	0.00
ATOM	2356	HW1	SOL	91	45.740	35.950	18.130	1.00	0.00
ATOM	2357	HW2	SOL	91	46.850	36.060	19.150	1.00	0.00
ATOM	2358	OW	SOL	92	20.440	1.960	13.240	1.00	0.00
ATOM	2359	HW1	SOL	92	19.700	2.260	13.770	1.00	0.00
ATOM	2360	HW2	SOL	92	20.580	2.670	12.610	1.00	0.00
ATOM	2361	OW	SOL	93	52.470	11.770	26.110	1.00	0.00
ATOM	2362	HW1	SOL	93	51.960	12.360	25.550	1.00	0.00
ATOM	2363	HW2	SOL	93	52.280	10.900	25.770	1.00	0.00
ATOM	2364	OW	SOL	94	51.310	43.160	37.890	1.00	0.00
ATOM	2365	HW1	SOL	94	51.100	43.740	37.160	1.00	0.00
ATOM	2366	HW2	SOL	94	51.680	42.380	37.480	1.00	0.00
ATOM	2367	OW	SOL	95	5.560	16.100	3.900	1.00	0.00
ATOM	2368	HW1	SOL	95	4.830	15.670	3.470	1.00	0.00
ATOM	2369	HW2	SOL	95	6.270	15.460	3.870	1.00	0.00
ATOM	2370	OW	SOL	96	9.000	21.070	5.930	1.00	0.00
ATOM	2371	HW1	SOL	96	9.690	20.880	5.290	1.00	0.00
ATOM	2372	HW2	SOL	96	8.210	20.700	5.530	1.00	0.00
ATOM	2373	OW	SOL	97	24.490	35.120	10.650	1.00	0.00
ATOM	2374	HW1	SOL	97	24.930	35.420	9.850	1.00	0.00
ATOM	2375	HW2	SOL	97	23.560	35.130	10.440	1.00	0.00

ATOM	2376	OW	SOL	98	54.290	15.810	16.280	1.00	0.00
ATOM	2377	HW1	SOL	98	54.390	15.000	16.780	1.00	0.00
ATOM	2378	HW2	SOL	98	54.470	16.510	16.910	1.00	0.00
ATOM	2379	OW	SOL	99	3.770	26.350	21.140	1.00	0.00
ATOM	2380	HW1	SOL	99	3.960	27.070	20.540	1.00	0.00
ATOM	2381	HW2	SOL	99	4.210	25.590	20.760	1.00	0.00
ATOM	2382	OW	SOL	100	41.840	41.190	13.270	1.00	0.00
ATOM	2383	HW1	SOL	100	41.710	41.520	12.380	1.00	0.00
ATOM	2384	HW2	SOL	100	42.630	40.650	13.220	1.00	0.00
ATOM	2385	OW	SOL	101	2.660	20.220	20.630	1.00	0.00
ATOM	2386	HW1	SOL	101	3.620	20.210	20.540	1.00	0.00
ATOM	2387	HW2	SOL	101	2.520	20.160	21.570	1.00	0.00
ATOM	2388	OW	SOL	102	11.380	0.690	51.990	1.00	0.00
ATOM	2389	HW1	SOL	102	12.110	0.640	52.600	1.00	0.00
ATOM	2390	HW2	SOL	102	10.680	0.200	52.410	1.00	0.00
ATOM	2391	OW	SOL	103	8.810	23.630	50.130	1.00	0.00
ATOM	2392	HW1	SOL	103	8.520	24.170	49.400	1.00	0.00
ATOM	2393	HW2	SOL	103	8.400	22.780	49.980	1.00	0.00
ATOM	2394	OW	SOL	104	54.360	50.630	43.730	1.00	0.00
ATOM	2395	HW1	SOL	104	54.920	50.190	44.370	1.00	0.00
ATOM	2396	HW2	SOL	104	53.510	50.170	43.800	1.00	0.00
ATOM	2397	OW	SOL	105	41.620	36.530	33.500	1.00	0.00
ATOM	2398	HW1	SOL	105	41.800	36.420	34.430	1.00	0.00
ATOM	2399	HW2	SOL	105	41.330	35.670	33.210	1.00	0.00
ATOM	2400	OW	SOL	106	16.740	40.060	9.110	1.00	0.00
ATOM	2401	HW1	SOL	106	15.880	39.670	9.300	1.00	0.00
ATOM	2402	HW2	SOL	106	17.370	39.490	9.550	1.00	0.00
ATOM	2403	OW	SOL	107	6.530	25.530	5.520	1.00	0.00
ATOM	2404	HW1	SOL	107	6.520	24.820	6.160	1.00	0.00
ATOM	2405	HW2	SOL	107	5.770	25.350	4.960	1.00	0.00
ATOM	2406	OW	SOL	108	7.490	36.850	30.260	1.00	0.00
ATOM	2407	HW1	SOL	108	6.710	37.160	30.710	1.00	0.00
ATOM	2408	HW2	SOL	108	7.150	36.290	29.550	1.00	0.00
ATOM	2409	OW	SOL	109	44.320	44.080	23.270	1.00	0.00
ATOM	2410	HW1	SOL	109	45.130	44.220	22.780	1.00	0.00
ATOM	2411	HW2	SOL	109	44.110	44.930	23.650	1.00	0.00
ATOM	2412	OW	SOL	110	5.680	6.730	4.440	1.00	0.00
ATOM	2413	HW1	SOL	110	6.270	6.030	4.180	1.00	0.00
ATOM	2414	HW2	SOL	110	6.260	7.430	4.750	1.00	0.00
ATOM	2415	OW	SOL	111	19.440	38.580	13.970	1.00	0.00
ATOM	2416	HW1	SOL	111	19.110	37.850	13.450	1.00	0.00
ATOM	2417	HW2	SOL	111	20.310	38.760	13.610	1.00	0.00
ATOM	2418	OW	SOL	112	33.270	1.980	25.430	1.00	0.00
ATOM	2419	HW1	SOL	112	33.370	1.490	26.250	1.00	0.00

ATOM	2420	HW2	SOL	112	34.160	2.070	25.090	1.00	0.00
ATOM	2421	OW	SOL	113	5.310	9.250	46.540	1.00	0.00
ATOM	2422	HW1	SOL	113	5.090	8.680	47.280	1.00	0.00
ATOM	2423	HW2	SOL	113	5.390	8.650	45.800	1.00	0.00
ATOM	2424	OW	SOL	114	4.420	6.590	16.680	1.00	0.00
ATOM	2425	HW1	SOL	114	4.220	5.650	16.620	1.00	0.00
ATOM	2426	HW2	SOL	114	4.910	6.780	15.880	1.00	0.00
ATOM	2427	OW	SOL	115	5.330	33.980	18.490	1.00	0.00
ATOM	2428	HW1	SOL	115	4.530	34.490	18.350	1.00	0.00
ATOM	2429	HW2	SOL	115	5.560	33.640	17.630	1.00	0.00
ATOM	2430	OW	SOL	116	35.100	29.170	11.500	1.00	0.00
ATOM	2431	HW1	SOL	116	36.010	29.070	11.190	1.00	0.00
ATOM	2432	HW2	SOL	116	34.750	29.880	10.970	1.00	0.00
ATOM	2433	OW	SOL	117	46.920	7.410	45.710	1.00	0.00
ATOM	2434	HW1	SOL	117	46.110	7.610	45.240	1.00	0.00
ATOM	2435	HW2	SOL	117	46.760	6.550	46.110	1.00	0.00
ATOM	2436	OW	SOL	118	55.140	9.620	7.440	1.00	0.00
ATOM	2437	HW1	SOL	118	55.200	10.360	6.830	1.00	0.00
ATOM	2438	HW2	SOL	118	54.340	9.780	7.940	1.00	0.00
ATOM	2439	OW	SOL	119	10.250	51.780	14.610	1.00	0.00
ATOM	2440	HW1	SOL	119	10.870	51.090	14.850	1.00	0.00
ATOM	2441	HW2	SOL	119	10.440	51.950	13.690	1.00	0.00
ATOM	2442	OW	SOL	120	23.200	48.780	51.320	1.00	0.00
ATOM	2443	HW1	SOL	120	23.050	48.430	52.200	1.00	0.00
ATOM	2444	HW2	SOL	120	24.150	48.880	51.270	1.00	0.00
ATOM	2445	OW	SOL	121	16.150	48.380	15.760	1.00	0.00
ATOM	2446	HW1	SOL	121	15.430	48.720	16.290	1.00	0.00
ATOM	2447	HW2	SOL	121	16.050	48.810	14.910	1.00	0.00
ATOM	2448	OW	SOL	122	40.340	24.820	32.680	1.00	0.00
ATOM	2449	HW1	SOL	122	41.190	24.860	32.230	1.00	0.00
ATOM	2450	HW2	SOL	122	39.720	25.130	32.020	1.00	0.00
ATOM	2451	OW	SOL	123	36.820	29.540	39.550	1.00	0.00
ATOM	2452	HW1	SOL	123	36.660	30.450	39.280	1.00	0.00
ATOM	2453	HW2	SOL	123	36.070	29.330	40.110	1.00	0.00
ATOM	2454	OW	SOL	124	7.320	38.420	13.140	1.00	0.00
ATOM	2455	HW1	SOL	124	7.530	37.930	13.930	1.00	0.00
ATOM	2456	HW2	SOL	124	7.700	39.290	13.280	1.00	0.00
ATOM	2457	OW	SOL	125	47.610	23.400	37.470	1.00	0.00
ATOM	2458	HW1	SOL	125	47.860	24.010	38.160	1.00	0.00
ATOM	2459	HW2	SOL	125	47.120	23.940	36.850	1.00	0.00
ATOM	2460	OW	SOL	126	42.250	32.050	14.530	1.00	0.00
ATOM	2461	HW1	SOL	126	41.580	32.710	14.710	1.00	0.00
ATOM	2462	HW2	SOL	126	41.810	31.430	13.950	1.00	0.00
ATOM	2463	OW	SOL	127	18.110	24.620	5.290	1.00	0.00

ATOM	2464	HW1	SOL	127	17.750	23.920	5.840	1.00	0.00
ATOM	2465	HW2	SOL	127	17.570	24.590	4.490	1.00	0.00
ATOM	2466	OW	SOL	128	0.740	39.240	1.520	1.00	0.00
ATOM	2467	HW1	SOL	128	1.650	39.220	1.210	1.00	0.00
ATOM	2468	HW2	SOL	128	0.220	39.050	0.740	1.00	0.00
ATOM	2469	OW	SOL	129	16.820	21.410	35.680	1.00	0.00
ATOM	2470	HW1	SOL	129	16.590	22.340	35.790	1.00	0.00
ATOM	2471	HW2	SOL	129	17.430	21.400	34.950	1.00	0.00
ATOM	2472	OW	SOL	130	55.590	5.100	38.060	1.00	0.00
ATOM	2473	HW1	SOL	130	55.080	5.840	38.390	1.00	0.00
ATOM	2474	HW2	SOL	130	55.410	5.090	37.120	1.00	0.00
ATOM	2475	OW	SOL	131	2.150	53.150	30.810	1.00	0.00
ATOM	2476	HW1	SOL	131	2.910	52.590	30.950	1.00	0.00
ATOM	2477	HW2	SOL	131	1.880	52.980	29.910	1.00	0.00
ATOM	2478	OW	SOL	132	17.890	20.240	7.050	1.00	0.00
ATOM	2479	HW1	SOL	132	18.320	19.400	6.920	1.00	0.00
ATOM	2480	HW2	SOL	132	17.910	20.380	8.000	1.00	0.00
ATOM	2481	OW	SOL	133	8.130	39.400	29.430	1.00	0.00
ATOM	2482	HW1	SOL	133	7.920	38.510	29.710	1.00	0.00
ATOM	2483	HW2	SOL	133	7.300	39.760	29.130	1.00	0.00
ATOM	2484	OW	SOL	134	5.040	30.510	8.060	1.00	0.00
ATOM	2485	HW1	SOL	134	4.530	31.310	7.920	1.00	0.00
ATOM	2486	HW2	SOL	134	5.380	30.300	7.190	1.00	0.00
ATOM	2487	OW	SOL	135	23.700	2.190	2.440	1.00	0.00
ATOM	2488	HW1	SOL	135	22.840	1.980	2.800	1.00	0.00
ATOM	2489	HW2	SOL	135	24.300	1.590	2.890	1.00	0.00
ATOM	2490	OW	SOL	136	8.870	37.370	42.020	1.00	0.00
ATOM	2491	HW1	SOL	136	8.550	38.270	42.010	1.00	0.00
ATOM	2492	HW2	SOL	136	8.320	36.930	42.670	1.00	0.00
ATOM	2493	OW	SOL	137	6.150	51.730	35.960	1.00	0.00
ATOM	2494	HW1	SOL	137	5.610	50.940	35.980	1.00	0.00
ATOM	2495	HW2	SOL	137	6.660	51.680	36.770	1.00	0.00
ATOM	2496	OW	SOL	138	1.850	51.760	46.330	1.00	0.00
ATOM	2497	HW1	SOL	138	2.730	51.990	46.600	1.00	0.00
ATOM	2498	HW2	SOL	138	1.850	50.810	46.280	1.00	0.00
ATOM	2499	OW	SOL	139	4.270	49.630	36.830	1.00	0.00
ATOM	2500	HW1	SOL	139	4.520	48.880	37.370	1.00	0.00
ATOM	2501	HW2	SOL	139	3.980	49.240	36.000	1.00	0.00
ATOM	2502	OW	SOL	140	10.010	23.930	14.580	1.00	0.00
ATOM	2503	HW1	SOL	140	10.300	23.210	15.140	1.00	0.00
ATOM	2504	HW2	SOL	140	10.080	23.580	13.690	1.00	0.00
ATOM	2505	OW	SOL	141	8.870	35.470	26.870	1.00	0.00
ATOM	2506	HW1	SOL	141	7.950	35.340	27.100	1.00	0.00
ATOM	2507	HW2	SOL	141	9.050	36.380	27.140	1.00	0.00

ATOM	2508	OW	SOL	142	22.550	28.990	50.150	1.00	0.00
ATOM	2509	HW1	SOL	142	22.250	29.800	49.720	1.00	0.00
ATOM	2510	HW2	SOL	142	23.190	29.290	50.800	1.00	0.00
ATOM	2511	OW	SOL	143	9.120	21.520	33.560	1.00	0.00
ATOM	2512	HW1	SOL	143	8.420	22.150	33.720	1.00	0.00
ATOM	2513	HW2	SOL	143	9.000	21.250	32.650	1.00	0.00
ATOM	2514	OW	SOL	144	43.590	33.220	52.500	1.00	0.00
ATOM	2515	HW1	SOL	144	44.380	33.350	51.970	1.00	0.00
ATOM	2516	HW2	SOL	144	42.980	33.890	52.180	1.00	0.00
ATOM	2517	OW	SOL	145	48.420	3.610	54.140	1.00	0.00
ATOM	2518	HW1	SOL	145	49.230	3.180	53.860	1.00	0.00
ATOM	2519	HW2	SOL	145	48.720	4.320	54.710	1.00	0.00
ATOM	2520	OW	SOL	146	4.930	53.230	45.410	1.00	0.00
ATOM	2521	HW1	SOL	146	4.820	52.280	45.310	1.00	0.00
ATOM	2522	HW2	SOL	146	4.680	53.400	46.320	1.00	0.00
ATOM	2523	OW	SOL	147	18.410	8.460	9.130	1.00	0.00
ATOM	2524	HW1	SOL	147	18.790	9.260	8.780	1.00	0.00
ATOM	2525	HW2	SOL	147	17.480	8.530	8.930	1.00	0.00
ATOM	2526	OW	SOL	148	22.980	47.290	44.620	1.00	0.00
ATOM	2527	HW1	SOL	148	23.480	46.500	44.830	1.00	0.00
ATOM	2528	HW2	SOL	148	22.920	47.770	45.440	1.00	0.00
ATOM	2529	OW	SOL	149	33.950	52.860	48.700	1.00	0.00
ATOM	2530	HW1	SOL	149	34.880	52.930	48.940	1.00	0.00
ATOM	2531	HW2	SOL	149	33.510	52.670	49.530	1.00	0.00
ATOM	2532	OW	SOL	150	20.510	28.440	20.440	1.00	0.00
ATOM	2533	HW1	SOL	150	21.200	27.880	20.790	1.00	0.00
ATOM	2534	HW2	SOL	150	20.960	29.020	19.820	1.00	0.00
ATOM	2535	OW	SOL	151	33.590	42.730	5.280	1.00	0.00
ATOM	2536	HW1	SOL	151	33.770	43.680	5.250	1.00	0.00
ATOM	2537	HW2	SOL	151	34.140	42.420	6.000	1.00	0.00
ATOM	2538	OW	SOL	152	25.980	30.780	41.500	1.00	0.00
ATOM	2539	HW1	SOL	152	26.470	31.600	41.570	1.00	0.00
ATOM	2540	HW2	SOL	152	25.120	31.030	41.160	1.00	0.00
ATOM	2541	OW	SOL	153	51.140	3.090	52.860	1.00	0.00
ATOM	2542	HW1	SOL	153	51.420	2.530	52.130	1.00	0.00
ATOM	2543	HW2	SOL	153	51.380	3.970	52.580	1.00	0.00
ATOM	2544	OW	SOL	154	27.200	2.470	44.180	1.00	0.00
ATOM	2545	HW1	SOL	154	26.820	1.730	44.660	1.00	0.00
ATOM	2546	HW2	SOL	154	26.580	2.630	43.460	1.00	0.00
ATOM	2547	OW	SOL	155	5.910	11.140	50.430	1.00	0.00
ATOM	2548	HW1	SOL	155	6.730	10.670	50.550	1.00	0.00
ATOM	2549	HW2	SOL	155	6.120	12.040	50.680	1.00	0.00
ATOM	2550	OW	SOL	156	17.750	32.080	42.600	1.00	0.00
ATOM	2551	HW1	SOL	156	17.510	31.160	42.560	1.00	0.00

ATOM	2552	HW2	SOL	156	16.970	32.550	42.300	1.00	0.00
ATOM	2553	OW	SOL	157	18.510	43.570	53.430	1.00	0.00
ATOM	2554	HW1	SOL	157	18.090	44.420	53.290	1.00	0.00
ATOM	2555	HW2	SOL	157	18.110	43.240	54.230	1.00	0.00
ATOM	2556	OW	SOL	158	13.330	9.930	53.520	1.00	0.00
ATOM	2557	HW1	SOL	158	13.210	10.720	52.990	1.00	0.00
ATOM	2558	HW2	SOL	158	12.770	10.060	54.280	1.00	0.00
ATOM	2559	OW	SOL	159	35.560	34.780	44.570	1.00	0.00
ATOM	2560	HW1	SOL	159	34.950	35.330	45.070	1.00	0.00
ATOM	2561	HW2	SOL	159	35.000	34.110	44.170	1.00	0.00
ATOM	2562	OW	SOL	160	31.780	54.570	48.310	1.00	0.00
ATOM	2563	HW1	SOL	160	32.050	55.300	48.860	1.00	0.00
ATOM	2564	HW2	SOL	160	32.560	54.020	48.240	1.00	0.00
ATOM	2565	OW	SOL	161	3.700	14.690	28.730	1.00	0.00
ATOM	2566	HW1	SOL	161	4.060	14.060	29.350	1.00	0.00
ATOM	2567	HW2	SOL	161	4.370	15.370	28.660	1.00	0.00
ATOM	2568	OW	SOL	162	34.870	12.890	25.930	1.00	0.00
ATOM	2569	HW1	SOL	162	35.430	12.900	25.150	1.00	0.00
ATOM	2570	HW2	SOL	162	34.720	11.960	26.100	1.00	0.00
ATOM	2571	OW	SOL	163	9.390	20.980	21.070	1.00	0.00
ATOM	2572	HW1	SOL	163	10.010	21.580	20.670	1.00	0.00
ATOM	2573	HW2	SOL	163	9.810	20.710	21.890	1.00	0.00
ATOM	2574	OW	SOL	164	17.300	14.600	15.110	1.00	0.00
ATOM	2575	HW1	SOL	164	17.860	15.160	14.570	1.00	0.00
ATOM	2576	HW2	SOL	164	16.500	15.110	15.230	1.00	0.00
ATOM	2577	OW	SOL	165	42.580	48.580	44.250	1.00	0.00
ATOM	2578	HW1	SOL	165	43.050	49.100	44.900	1.00	0.00
ATOM	2579	HW2	SOL	165	41.820	48.240	44.720	1.00	0.00
ATOM	2580	OW	SOL	166	35.930	26.460	29.360	1.00	0.00
ATOM	2581	HW1	SOL	166	36.330	27.320	29.510	1.00	0.00
ATOM	2582	HW2	SOL	166	35.730	26.140	30.240	1.00	0.00
ATOM	2583	OW	SOL	167	0.120	45.130	21.990	1.00	0.00
ATOM	2584	HW1	SOL	167	-0.170	46.040	22.010	1.00	0.00
ATOM	2585	HW2	SOL	167	1.070	45.170	21.940	1.00	0.00
ATOM	2586	OW	SOL	168	29.470	41.150	49.210	1.00	0.00
ATOM	2587	HW1	SOL	168	28.560	41.410	49.370	1.00	0.00
ATOM	2588	HW2	SOL	168	29.860	41.900	48.770	1.00	0.00
ATOM	2589	OW	SOL	169	39.440	50.480	2.430	1.00	0.00
ATOM	2590	HW1	SOL	169	39.920	51.040	3.040	1.00	0.00
ATOM	2591	HW2	SOL	169	38.650	50.230	2.910	1.00	0.00
ATOM	2592	OW	SOL	170	11.130	49.520	26.120	1.00	0.00
ATOM	2593	HW1	SOL	170	11.660	50.130	26.640	1.00	0.00
ATOM	2594	HW2	SOL	170	10.240	49.880	26.150	1.00	0.00
ATOM	2595	OW	SOL	171	5.890	51.850	23.570	1.00	0.00

ATOM	2596	HW1	SOL	171	6.670	51.310	23.470	1.00	0.00
ATOM	2597	HW2	SOL	171	5.980	52.260	24.430	1.00	0.00
ATOM	2598	OW	SOL	172	30.810	24.680	15.660	1.00	0.00
ATOM	2599	HW1	SOL	172	31.230	23.840	15.870	1.00	0.00
ATOM	2600	HW2	SOL	172	31.390	25.070	15.010	1.00	0.00
ATOM	2601	OW	SOL	173	13.240	3.840	9.950	1.00	0.00
ATOM	2602	HW1	SOL	173	12.540	4.460	10.160	1.00	0.00
ATOM	2603	HW2	SOL	173	13.890	4.360	9.470	1.00	0.00
ATOM	2604	OW	SOL	174	17.250	5.490	16.450	1.00	0.00
ATOM	2605	HW1	SOL	174	16.610	4.800	16.280	1.00	0.00
ATOM	2606	HW2	SOL	174	16.930	6.250	15.960	1.00	0.00
ATOM	2607	OW	SOL	175	7.550	50.920	12.820	1.00	0.00
ATOM	2608	HW1	SOL	175	7.480	50.040	13.180	1.00	0.00
ATOM	2609	HW2	SOL	175	7.710	51.480	13.580	1.00	0.00
ATOM	2610	OW	SOL	176	13.990	24.140	43.110	1.00	0.00
ATOM	2611	HW1	SOL	176	13.770	23.470	43.760	1.00	0.00
ATOM	2612	HW2	SOL	176	14.690	24.650	43.510	1.00	0.00
ATOM	2613	OW	SOL	177	14.980	9.860	38.940	1.00	0.00
ATOM	2614	HW1	SOL	177	15.790	9.610	39.390	1.00	0.00
ATOM	2615	HW2	SOL	177	14.830	9.160	38.300	1.00	0.00
ATOM	2616	OW	SOL	178	7.260	40.580	17.280	1.00	0.00
ATOM	2617	HW1	SOL	178	6.710	39.990	16.770	1.00	0.00
ATOM	2618	HW2	SOL	178	6.640	41.200	17.670	1.00	0.00
ATOM	2619	OW	SOL	179	8.770	43.150	25.910	1.00	0.00
ATOM	2620	HW1	SOL	179	8.080	43.240	25.250	1.00	0.00
ATOM	2621	HW2	SOL	179	9.360	43.890	25.740	1.00	0.00
ATOM	2622	OW	SOL	180	36.890	14.240	38.490	1.00	0.00
ATOM	2623	HW1	SOL	180	37.150	13.410	38.890	1.00	0.00
ATOM	2624	HW2	SOL	180	35.980	14.120	38.230	1.00	0.00
ATOM	2625	OW	SOL	181	12.430	15.050	27.130	1.00	0.00
ATOM	2626	HW1	SOL	181	13.360	15.110	27.340	1.00	0.00
ATOM	2627	HW2	SOL	181	12.310	15.650	26.400	1.00	0.00
ATOM	2628	OW	SOL	182	12.240	17.330	14.330	1.00	0.00
ATOM	2629	HW1	SOL	182	12.620	17.730	15.120	1.00	0.00
ATOM	2630	HW2	SOL	182	12.990	17.220	13.740	1.00	0.00
ATOM	2631	OW	SOL	183	54.900	27.390	50.220	1.00	0.00
ATOM	2632	HW1	SOL	183	55.340	28.170	50.560	1.00	0.00
ATOM	2633	HW2	SOL	183	55.470	27.080	49.510	1.00	0.00
ATOM	2634	OW	SOL	184	39.350	47.900	42.630	1.00	0.00
ATOM	2635	HW1	SOL	184	40.090	47.890	42.030	1.00	0.00
ATOM	2636	HW2	SOL	184	39.160	48.830	42.760	1.00	0.00
ATOM	2637	OW	SOL	185	34.980	28.990	46.590	1.00	0.00
ATOM	2638	HW1	SOL	185	35.600	29.010	45.860	1.00	0.00
ATOM	2639	HW2	SOL	185	34.120	28.910	46.180	1.00	0.00

ATOM	2640	OW	SOL	186	8.610	28.780	22.660	1.00	0.00
ATOM	2641	HW1	SOL	186	8.750	29.700	22.470	1.00	0.00
ATOM	2642	HW2	SOL	186	8.790	28.700	23.600	1.00	0.00
ATOM	2643	OW	SOL	187	9.200	25.910	6.930	1.00	0.00
ATOM	2644	HW1	SOL	187	8.290	26.030	6.660	1.00	0.00
ATOM	2645	HW2	SOL	187	9.240	25.020	7.270	1.00	0.00
ATOM	2646	OW	SOL	188	48.580	30.380	4.540	1.00	0.00
ATOM	2647	HW1	SOL	188	49.530	30.370	4.590	1.00	0.00
ATOM	2648	HW2	SOL	188	48.310	29.540	4.920	1.00	0.00
ATOM	2649	OW	SOL	189	21.050	53.940	35.490	1.00	0.00
ATOM	2650	HW1	SOL	189	20.800	54.860	35.440	1.00	0.00
ATOM	2651	HW2	SOL	189	20.670	53.630	36.320	1.00	0.00
ATOM	2652	OW	SOL	190	16.550	7.830	15.320	1.00	0.00
ATOM	2653	HW1	SOL	190	15.920	8.130	14.670	1.00	0.00
ATOM	2654	HW2	SOL	190	17.200	8.540	15.370	1.00	0.00
ATOM	2655	OW	SOL	191	45.680	22.490	18.100	1.00	0.00
ATOM	2656	HW1	SOL	191	44.860	22.970	18.180	1.00	0.00
ATOM	2657	HW2	SOL	191	46.190	23.000	17.470	1.00	0.00
ATOM	2658	OW	SOL	192	54.550	46.090	34.780	1.00	0.00
ATOM	2659	HW1	SOL	192	54.630	45.230	35.190	1.00	0.00
ATOM	2660	HW2	SOL	192	54.730	46.710	35.480	1.00	0.00
ATOM	2661	OW	SOL	193	38.860	7.870	11.590	1.00	0.00
ATOM	2662	HW1	SOL	193	39.440	7.990	10.840	1.00	0.00
ATOM	2663	HW2	SOL	193	38.500	6.990	11.480	1.00	0.00
ATOM	2664	OW	SOL	194	40.190	17.750	6.040	1.00	0.00
ATOM	2665	HW1	SOL	194	40.000	16.990	5.490	1.00	0.00
ATOM	2666	HW2	SOL	194	41.130	17.730	6.170	1.00	0.00
ATOM	2667	OW	SOL	195	17.620	18.340	52.060	1.00	0.00
ATOM	2668	HW1	SOL	195	17.980	18.860	52.790	1.00	0.00
ATOM	2669	HW2	SOL	195	17.910	18.790	51.270	1.00	0.00
ATOM	2670	OW	SOL	196	10.920	10.630	25.800	1.00	0.00
ATOM	2671	HW1	SOL	196	10.550	10.770	26.680	1.00	0.00
ATOM	2672	HW2	SOL	196	10.360	11.140	25.220	1.00	0.00
ATOM	2673	OW	SOL	197	20.060	55.260	48.640	1.00	0.00
ATOM	2674	HW1	SOL	197	19.840	55.590	47.770	1.00	0.00
ATOM	2675	HW2	SOL	197	20.980	55.500	48.760	1.00	0.00
ATOM	2676	OW	SOL	198	24.830	30.720	54.670	1.00	0.00
ATOM	2677	HW1	SOL	198	23.890	30.510	54.720	1.00	0.00
ATOM	2678	HW2	SOL	198	25.250	30.070	55.230	1.00	0.00
ATOM	2679	OW	SOL	199	51.450	18.750	13.050	1.00	0.00
ATOM	2680	HW1	SOL	199	50.660	19.270	13.190	1.00	0.00
ATOM	2681	HW2	SOL	199	51.740	18.510	13.940	1.00	0.00
ATOM	2682	OW	SOL	200	8.120	5.670	3.470	1.00	0.00
ATOM	2683	HW1	SOL	200	8.340	5.940	4.360	1.00	0.00

ATOM	2684	HW2	SOL	200	7.930	4.730	3.550	1.00	0.00
ATOM	2685	OW	SOL	201	14.150	0.910	26.770	1.00	0.00
ATOM	2686	HW1	SOL	201	14.990	1.340	26.610	1.00	0.00
ATOM	2687	HW2	SOL	201	13.490	1.550	26.520	1.00	0.00
ATOM	2688	OW	SOL	202	38.370	45.810	55.220	1.00	0.00
ATOM	2689	HW1	SOL	202	38.090	46.730	55.260	1.00	0.00
ATOM	2690	HW2	SOL	202	39.250	45.860	54.850	1.00	0.00
ATOM	2691	OW	SOL	203	33.450	5.310	20.930	1.00	0.00
ATOM	2692	HW1	SOL	203	33.100	4.560	20.440	1.00	0.00
ATOM	2693	HW2	SOL	203	33.370	5.050	21.850	1.00	0.00
ATOM	2694	OW	SOL	204	52.880	42.750	26.900	1.00	0.00
ATOM	2695	HW1	SOL	204	53.700	43.230	26.780	1.00	0.00
ATOM	2696	HW2	SOL	204	53.130	41.970	27.390	1.00	0.00
ATOM	2697	OW	SOL	205	37.070	9.730	12.660	1.00	0.00
ATOM	2698	HW1	SOL	205	36.760	9.310	13.460	1.00	0.00
ATOM	2699	HW2	SOL	205	37.570	9.040	12.210	1.00	0.00
ATOM	2700	OW	SOL	206	46.120	16.610	24.830	1.00	0.00
ATOM	2701	HW1	SOL	206	45.470	16.590	24.120	1.00	0.00
ATOM	2702	HW2	SOL	206	46.850	17.110	24.460	1.00	0.00
ATOM	2703	OW	SOL	207	0.690	29.690	30.220	1.00	0.00
ATOM	2704	HW1	SOL	207	0.510	28.880	30.690	1.00	0.00
ATOM	2705	HW2	SOL	207	0.760	29.430	29.300	1.00	0.00
ATOM	2706	OW	SOL	208	46.680	19.360	42.620	1.00	0.00
ATOM	2707	HW1	SOL	208	47.580	19.050	42.690	1.00	0.00
ATOM	2708	HW2	SOL	208	46.460	19.660	43.500	1.00	0.00
ATOM	2709	OW	SOL	209	27.130	14.390	55.600	1.00	0.00
ATOM	2710	HW1	SOL	209	27.340	13.460	55.730	1.00	0.00
ATOM	2711	HW2	SOL	209	27.720	14.670	54.900	1.00	0.00
ATOM	2712	OW	SOL	210	55.650	20.910	21.810	1.00	0.00
ATOM	2713	HW1	SOL	210	56.560	21.020	22.090	1.00	0.00
ATOM	2714	HW2	SOL	210	55.210	21.710	22.100	1.00	0.00
ATOM	2715	OW	SOL	211	44.930	24.630	5.260	1.00	0.00
ATOM	2716	HW1	SOL	211	45.780	24.230	5.060	1.00	0.00
ATOM	2717	HW2	SOL	211	45.150	25.480	5.650	1.00	0.00
ATOM	2718	OW	SOL	212	32.280	23.230	43.680	1.00	0.00
ATOM	2719	HW1	SOL	212	32.320	23.770	42.890	1.00	0.00
ATOM	2720	HW2	SOL	212	32.340	23.850	44.400	1.00	0.00
ATOM	2721	OW	SOL	213	27.770	4.210	21.580	1.00	0.00
ATOM	2722	HW1	SOL	213	28.590	4.620	21.840	1.00	0.00
ATOM	2723	HW2	SOL	213	27.530	4.650	20.770	1.00	0.00
ATOM	2724	OW	SOL	214	37.170	18.660	15.360	1.00	0.00
ATOM	2725	HW1	SOL	214	36.490	19.280	15.630	1.00	0.00
ATOM	2726	HW2	SOL	214	37.920	18.870	15.910	1.00	0.00
ATOM	2727	OW	SOL	215	22.530	9.120	16.900	1.00	0.00

ATOM	2728	HW1	SOL	215	23.420	9.460	17.010	1.00	0.00
ATOM	2729	HW2	SOL	215	22.550	8.680	16.040	1.00	0.00
ATOM	2730	OW	SOL	216	32.270	31.250	48.000	1.00	0.00
ATOM	2731	HW1	SOL	216	31.380	31.600	48.030	1.00	0.00
ATOM	2732	HW2	SOL	216	32.180	30.340	48.270	1.00	0.00
ATOM	2733	OW	SOL	217	23.530	12.220	52.020	1.00	0.00
ATOM	2734	HW1	SOL	217	23.670	12.190	52.970	1.00	0.00
ATOM	2735	HW2	SOL	217	24.260	12.720	51.680	1.00	0.00
ATOM	2736	OW	SOL	218	3.340	16.870	44.290	1.00	0.00
ATOM	2737	HW1	SOL	218	3.540	15.980	44.010	1.00	0.00
ATOM	2738	HW2	SOL	218	2.490	17.060	43.880	1.00	0.00
ATOM	2739	OW	SOL	219	19.770	10.750	8.410	1.00	0.00
ATOM	2740	HW1	SOL	219	20.080	10.680	9.310	1.00	0.00
ATOM	2741	HW2	SOL	219	19.440	11.640	8.330	1.00	0.00
ATOM	2742	OW	SOL	220	15.770	9.930	6.040	1.00	0.00
ATOM	2743	HW1	SOL	220	16.180	9.120	5.750	1.00	0.00
ATOM	2744	HW2	SOL	220	16.490	10.560	6.100	1.00	0.00
ATOM	2745	OW	SOL	221	55.400	48.850	54.440	1.00	0.00
ATOM	2746	HW1	SOL	221	55.900	48.770	53.630	1.00	0.00
ATOM	2747	HW2	SOL	221	55.850	48.270	55.060	1.00	0.00
ATOM	2748	OW	SOL	222	52.970	16.810	10.880	1.00	0.00
ATOM	2749	HW1	SOL	222	52.580	17.180	10.080	1.00	0.00
ATOM	2750	HW2	SOL	222	52.740	17.440	11.570	1.00	0.00
ATOM	2751	OW	SOL	223	23.430	53.440	45.430	1.00	0.00
ATOM	2752	HW1	SOL	223	24.060	53.630	46.130	1.00	0.00
ATOM	2753	HW2	SOL	223	23.300	54.280	44.990	1.00	0.00
ATOM	2754	OW	SOL	224	19.630	6.990	23.900	1.00	0.00
ATOM	2755	HW1	SOL	224	19.020	6.260	23.870	1.00	0.00
ATOM	2756	HW2	SOL	224	19.090	7.760	23.720	1.00	0.00
ATOM	2757	OW	SOL	225	33.750	54.230	41.340	1.00	0.00
ATOM	2758	HW1	SOL	225	33.210	54.630	42.020	1.00	0.00
ATOM	2759	HW2	SOL	225	33.290	53.430	41.110	1.00	0.00
ATOM	2760	OW	SOL	226	7.020	37.090	55.250	1.00	0.00
ATOM	2761	HW1	SOL	226	6.160	36.680	55.320	1.00	0.00
ATOM	2762	HW2	SOL	226	6.880	38.000	55.530	1.00	0.00
ATOM	2763	OW	SOL	227	1.220	54.140	37.140	1.00	0.00
ATOM	2764	HW1	SOL	227	1.190	54.630	36.320	1.00	0.00
ATOM	2765	HW2	SOL	227	0.940	54.770	37.810	1.00	0.00
ATOM	2766	OW	SOL	228	15.810	21.050	48.630	1.00	0.00
ATOM	2767	HW1	SOL	228	15.770	20.140	48.340	1.00	0.00
ATOM	2768	HW2	SOL	228	14.890	21.310	48.720	1.00	0.00
ATOM	2769	OW	SOL	229	54.530	35.840	39.850	1.00	0.00
ATOM	2770	HW1	SOL	229	54.240	36.320	40.630	1.00	0.00
ATOM	2771	HW2	SOL	229	54.450	34.910	40.100	1.00	0.00

ATOM	2772	OW	SOL	230	55.620	9.620	24.100	1.00	0.00
ATOM	2773	HW1	SOL	230	55.850	10.540	24.260	1.00	0.00
ATOM	2774	HW2	SOL	230	55.540	9.240	24.970	1.00	0.00
ATOM	2775	OW	SOL	231	6.330	48.040	8.350	1.00	0.00
ATOM	2776	HW1	SOL	231	6.100	47.120	8.210	1.00	0.00
ATOM	2777	HW2	SOL	231	7.240	48.110	8.080	1.00	0.00
ATOM	2778	OW	SOL	232	33.680	21.880	50.920	1.00	0.00
ATOM	2779	HW1	SOL	232	33.530	21.420	50.090	1.00	0.00
ATOM	2780	HW2	SOL	232	33.180	22.690	50.840	1.00	0.00
ATOM	2781	OW	SOL	233	27.940	23.220	45.780	1.00	0.00
ATOM	2782	HW1	SOL	233	27.500	22.400	46.000	1.00	0.00
ATOM	2783	HW2	SOL	233	28.490	23.010	45.020	1.00	0.00
ATOM	2784	OW	SOL	234	24.540	29.730	20.050	1.00	0.00
ATOM	2785	HW1	SOL	234	23.930	30.130	20.670	1.00	0.00
ATOM	2786	HW2	SOL	234	24.520	28.800	20.260	1.00	0.00
ATOM	2787	OW	SOL	235	46.260	42.340	49.370	1.00	0.00
ATOM	2788	HW1	SOL	235	45.980	43.160	48.980	1.00	0.00
ATOM	2789	HW2	SOL	235	47.210	42.330	49.260	1.00	0.00
ATOM	2790	OW	SOL	236	38.950	20.480	10.040	1.00	0.00
ATOM	2791	HW1	SOL	236	38.140	20.940	10.270	1.00	0.00
ATOM	2792	HW2	SOL	236	39.010	19.770	10.670	1.00	0.00
ATOM	2793	OW	SOL	237	1.490	24.620	21.140	1.00	0.00
ATOM	2794	HW1	SOL	237	1.020	24.120	20.470	1.00	0.00
ATOM	2795	HW2	SOL	237	2.350	24.790	20.750	1.00	0.00
ATOM	2796	OW	SOL	238	52.370	18.620	15.540	1.00	0.00
ATOM	2797	HW1	SOL	238	52.990	18.110	16.080	1.00	0.00
ATOM	2798	HW2	SOL	238	52.710	19.510	15.560	1.00	0.00
ATOM	2799	OW	SOL	239	27.790	10.980	17.520	1.00	0.00
ATOM	2800	HW1	SOL	239	27.460	11.800	17.140	1.00	0.00
ATOM	2801	HW2	SOL	239	27.650	10.330	16.840	1.00	0.00
ATOM	2802	OW	SOL	240	9.600	39.300	16.900	1.00	0.00
ATOM	2803	HW1	SOL	240	9.020	40.050	17.000	1.00	0.00
ATOM	2804	HW2	SOL	240	9.950	39.140	17.780	1.00	0.00
ATOM	2805	OW	SOL	241	27.200	35.340	47.620	1.00	0.00
ATOM	2806	HW1	SOL	241	28.140	35.280	47.790	1.00	0.00
ATOM	2807	HW2	SOL	241	26.880	34.440	47.710	1.00	0.00
ATOM	2808	OW	SOL	242	38.980	30.740	7.450	1.00	0.00
ATOM	2809	HW1	SOL	242	38.840	31.560	7.920	1.00	0.00
ATOM	2810	HW2	SOL	242	38.100	30.380	7.330	1.00	0.00
ATOM	2811	OW	SOL	243	3.590	22.200	28.040	1.00	0.00
ATOM	2812	HW1	SOL	243	3.760	22.810	28.760	1.00	0.00
ATOM	2813	HW2	SOL	243	3.510	22.760	27.270	1.00	0.00
ATOM	2814	OW	SOL	244	7.150	26.950	43.160	1.00	0.00
ATOM	2815	HW1	SOL	244	7.350	26.090	42.790	1.00	0.00

ATOM	2816	HW2	SOL	244	6.530	26.770	43.860	1.00	0.00
ATOM	2817	OW	SOL	245	43.410	15.890	22.570	1.00	0.00
ATOM	2818	HW1	SOL	245	43.110	15.060	22.930	1.00	0.00
ATOM	2819	HW2	SOL	245	43.820	15.660	21.740	1.00	0.00
ATOM	2820	OW	SOL	246	40.300	23.190	9.000	1.00	0.00
ATOM	2821	HW1	SOL	246	39.710	23.940	9.090	1.00	0.00
ATOM	2822	HW2	SOL	246	39.750	22.420	9.140	1.00	0.00
ATOM	2823	OW	SOL	247	4.290	39.810	52.100	1.00	0.00
ATOM	2824	HW1	SOL	247	4.080	40.440	51.410	1.00	0.00
ATOM	2825	HW2	SOL	247	5.170	39.510	51.900	1.00	0.00
ATOM	2826	OW	SOL	248	42.900	18.390	39.250	1.00	0.00
ATOM	2827	HW1	SOL	248	43.460	18.020	38.570	1.00	0.00
ATOM	2828	HW2	SOL	248	43.510	18.780	39.880	1.00	0.00
ATOM	2829	OW	SOL	249	49.790	51.150	26.560	1.00	0.00
ATOM	2830	HW1	SOL	249	49.730	50.310	27.020	1.00	0.00
ATOM	2831	HW2	SOL	249	49.960	51.790	27.260	1.00	0.00
ATOM	2832	OW	SOL	250	54.460	32.840	29.000	1.00	0.00
ATOM	2833	HW1	SOL	250	54.020	32.000	29.150	1.00	0.00
ATOM	2834	HW2	SOL	250	54.600	32.870	28.050	1.00	0.00
ATOM	2835	OW	SOL	251	46.670	17.150	30.720	1.00	0.00
ATOM	2836	HW1	SOL	251	47.500	17.030	31.190	1.00	0.00
ATOM	2837	HW2	SOL	251	46.000	17.010	31.380	1.00	0.00
ATOM	2838	OW	SOL	252	9.850	13.070	50.880	1.00	0.00
ATOM	2839	HW1	SOL	252	10.320	13.680	50.310	1.00	0.00
ATOM	2840	HW2	SOL	252	9.440	13.620	51.540	1.00	0.00
ATOM	2841	OW	SOL	253	1.900	31.730	49.070	1.00	0.00
ATOM	2842	HW1	SOL	253	1.190	31.720	49.710	1.00	0.00
ATOM	2843	HW2	SOL	253	2.030	32.660	48.880	1.00	0.00
ATOM	2844	OW	SOL	254	9.840	45.220	53.540	1.00	0.00
ATOM	2845	HW1	SOL	254	9.250	45.860	53.940	1.00	0.00
ATOM	2846	HW2	SOL	254	10.140	45.640	52.740	1.00	0.00
ATOM	2847	OW	SOL	255	11.230	22.090	46.210	1.00	0.00
ATOM	2848	HW1	SOL	255	10.350	22.380	46.460	1.00	0.00
ATOM	2849	HW2	SOL	255	11.080	21.350	45.630	1.00	0.00
ATOM	2850	OW	SOL	256	0.200	43.150	27.430	1.00	0.00
ATOM	2851	HW1	SOL	256	0.470	42.250	27.600	1.00	0.00
ATOM	2852	HW2	SOL	256	-0.050	43.490	28.290	1.00	0.00
ATOM	2853	OW	SOL	257	51.880	52.240	25.120	1.00	0.00
ATOM	2854	HW1	SOL	257	51.440	51.910	25.900	1.00	0.00
ATOM	2855	HW2	SOL	257	51.260	52.840	24.720	1.00	0.00
ATOM	2856	OW	SOL	258	28.840	14.530	4.960	1.00	0.00
ATOM	2857	HW1	SOL	258	28.030	14.330	5.430	1.00	0.00
ATOM	2858	HW2	SOL	258	28.580	14.520	4.030	1.00	0.00
ATOM	2859	OW	SOL	259	48.290	28.840	14.060	1.00	0.00

ATOM	2860	HW1	SOL	259	49.240	28.750	14.140	1.00	0.00
ATOM	2861	HW2	SOL	259	47.990	29.070	14.940	1.00	0.00
ATOM	2862	OW	SOL	260	13.100	8.410	31.850	1.00	0.00
ATOM	2863	HW1	SOL	260	13.050	9.280	31.460	1.00	0.00
ATOM	2864	HW2	SOL	260	12.680	7.840	31.200	1.00	0.00
ATOM	2865	OW	SOL	261	28.260	44.740	54.100	1.00	0.00
ATOM	2866	HW1	SOL	261	27.340	44.560	53.930	1.00	0.00
ATOM	2867	HW2	SOL	261	28.280	45.650	54.400	1.00	0.00
ATOM	2868	OW	SOL	262	48.380	21.340	7.800	1.00	0.00
ATOM	2869	HW1	SOL	262	47.480	21.130	8.040	1.00	0.00
ATOM	2870	HW2	SOL	262	48.750	20.510	7.490	1.00	0.00
ATOM	2871	OW	SOL	263	19.950	15.750	5.990	1.00	0.00
ATOM	2872	HW1	SOL	263	20.240	15.020	5.440	1.00	0.00
ATOM	2873	HW2	SOL	263	19.300	16.210	5.460	1.00	0.00
ATOM	2874	OW	SOL	264	53.850	22.650	36.010	1.00	0.00
ATOM	2875	HW1	SOL	264	54.340	22.420	35.220	1.00	0.00
ATOM	2876	HW2	SOL	264	54.100	21.970	36.640	1.00	0.00
ATOM	2877	OW	SOL	265	49.210	45.380	1.020	1.00	0.00
ATOM	2878	HW1	SOL	265	49.100	45.480	1.960	1.00	0.00
ATOM	2879	HW2	SOL	265	48.810	46.160	0.640	1.00	0.00
ATOM	2880	OW	SOL	266	11.330	35.790	1.700	1.00	0.00
ATOM	2881	HW1	SOL	266	10.430	35.970	1.430	1.00	0.00
ATOM	2882	HW2	SOL	266	11.330	35.930	2.640	1.00	0.00
ATOM	2883	OW	SOL	267	41.930	39.010	44.910	1.00	0.00
ATOM	2884	HW1	SOL	267	41.170	39.420	45.320	1.00	0.00
ATOM	2885	HW2	SOL	267	42.680	39.490	45.260	1.00	0.00
ATOM	2886	OW	SOL	268	2.520	51.650	52.150	1.00	0.00
ATOM	2887	HW1	SOL	268	2.550	51.340	51.240	1.00	0.00
ATOM	2888	HW2	SOL	268	2.770	52.570	52.100	1.00	0.00
ATOM	2889	OW	SOL	269	25.380	51.960	49.760	1.00	0.00
ATOM	2890	HW1	SOL	269	24.860	51.670	50.500	1.00	0.00
ATOM	2891	HW2	SOL	269	25.160	51.350	49.050	1.00	0.00
ATOM	2892	OW	SOL	270	50.410	11.430	21.550	1.00	0.00
ATOM	2893	HW1	SOL	270	50.750	12.240	21.150	1.00	0.00
ATOM	2894	HW2	SOL	270	50.750	11.450	22.440	1.00	0.00
ATOM	2895	OW	SOL	271	12.670	53.490	49.090	1.00	0.00
ATOM	2896	HW1	SOL	271	12.190	53.150	49.850	1.00	0.00
ATOM	2897	HW2	SOL	271	13.060	54.300	49.400	1.00	0.00
ATOM	2898	OW	SOL	272	9.840	38.880	22.590	1.00	0.00
ATOM	2899	HW1	SOL	272	9.590	38.080	23.040	1.00	0.00
ATOM	2900	HW2	SOL	272	9.230	38.940	21.850	1.00	0.00
ATOM	2901	OW	SOL	273	32.410	45.480	52.160	1.00	0.00
ATOM	2902	HW1	SOL	273	32.360	45.450	51.200	1.00	0.00
ATOM	2903	HW2	SOL	273	31.730	46.120	52.410	1.00	0.00

ATOM	2904	OW	SOL	274	34.430	52.340	12.150	1.00	0.00
ATOM	2905	HW1	SOL	274	33.780	53.040	12.160	1.00	0.00
ATOM	2906	HW2	SOL	274	35.130	52.670	11.600	1.00	0.00
ATOM	2907	OW	SOL	275	49.810	49.420	10.850	1.00	0.00
ATOM	2908	HW1	SOL	275	49.690	48.510	11.130	1.00	0.00
ATOM	2909	HW2	SOL	275	49.880	49.360	9.890	1.00	0.00
ATOM	2910	OW	SOL	276	16.180	1.540	43.400	1.00	0.00
ATOM	2911	HW1	SOL	276	16.110	1.800	44.320	1.00	0.00
ATOM	2912	HW2	SOL	276	17.060	1.180	43.310	1.00	0.00
ATOM	2913	OW	SOL	277	45.440	21.130	7.550	1.00	0.00
ATOM	2914	HW1	SOL	277	44.920	21.920	7.660	1.00	0.00
ATOM	2915	HW2	SOL	277	45.260	20.840	6.660	1.00	0.00
ATOM	2916	OW	SOL	278	31.190	29.140	49.190	1.00	0.00
ATOM	2917	HW1	SOL	278	31.140	28.330	48.680	1.00	0.00
ATOM	2918	HW2	SOL	278	30.320	29.540	49.090	1.00	0.00
ATOM	2919	OW	SOL	279	9.290	33.880	34.040	1.00	0.00
ATOM	2920	HW1	SOL	279	9.320	34.160	34.950	1.00	0.00
ATOM	2921	HW2	SOL	279	9.310	34.690	33.530	1.00	0.00
ATOM	2922	OW	SOL	280	5.380	45.580	36.600	1.00	0.00
ATOM	2923	HW1	SOL	280	6.220	45.910	36.280	1.00	0.00
ATOM	2924	HW2	SOL	280	5.580	44.710	36.960	1.00	0.00
ATOM	2925	OW	SOL	281	38.250	15.170	22.860	1.00	0.00
ATOM	2926	HW1	SOL	281	37.760	14.660	23.500	1.00	0.00
ATOM	2927	HW2	SOL	281	39.160	15.140	23.180	1.00	0.00
ATOM	2928	OW	SOL	282	9.920	48.240	49.260	1.00	0.00
ATOM	2929	HW1	SOL	282	9.760	49.100	48.860	1.00	0.00
ATOM	2930	HW2	SOL	282	9.800	47.620	48.540	1.00	0.00
ATOM	2931	OW	SOL	283	48.270	19.740	27.570	1.00	0.00
ATOM	2932	HW1	SOL	283	48.390	19.910	26.640	1.00	0.00
ATOM	2933	HW2	SOL	283	48.680	20.490	28.000	1.00	0.00
ATOM	2934	OW	SOL	284	15.700	29.980	30.440	1.00	0.00
ATOM	2935	HW1	SOL	284	15.550	30.920	30.490	1.00	0.00
ATOM	2936	HW2	SOL	284	16.470	29.890	29.880	1.00	0.00
ATOM	2937	OW	SOL	285	4.550	25.030	2.220	1.00	0.00
ATOM	2938	HW1	SOL	285	4.610	24.400	2.940	1.00	0.00
ATOM	2939	HW2	SOL	285	3.880	25.650	2.510	1.00	0.00
ATOM	2940	OW	SOL	286	38.470	33.210	37.180	1.00	0.00
ATOM	2941	HW1	SOL	286	38.050	34.050	37.340	1.00	0.00
ATOM	2942	HW2	SOL	286	38.440	32.750	38.020	1.00	0.00
ATOM	2943	OW	SOL	287	0.360	54.840	7.400	1.00	0.00
ATOM	2944	HW1	SOL	287	-0.300	54.400	7.940	1.00	0.00
ATOM	2945	HW2	SOL	287	1.060	54.190	7.320	1.00	0.00
ATOM	2946	OW	SOL	288	38.400	47.020	5.540	1.00	0.00
ATOM	2947	HW1	SOL	288	37.530	46.870	5.180	1.00	0.00

ATOM	2948	HW2	SOL	288	38.590	46.230	6.060	1.00	0.00
ATOM	2949	OW	SOL	289	42.100	53.520	17.330	1.00	0.00
ATOM	2950	HW1	SOL	289	42.590	54.330	17.220	1.00	0.00
ATOM	2951	HW2	SOL	289	42.420	52.960	16.620	1.00	0.00
ATOM	2952	OW	SOL	290	17.860	10.370	15.430	1.00	0.00
ATOM	2953	HW1	SOL	290	17.890	11.240	15.820	1.00	0.00
ATOM	2954	HW2	SOL	290	18.510	9.870	15.920	1.00	0.00
ATOM	2955	OW	SOL	291	4.810	25.400	9.230	1.00	0.00
ATOM	2956	HW1	SOL	291	4.250	25.190	8.490	1.00	0.00
ATOM	2957	HW2	SOL	291	4.910	26.350	9.190	1.00	0.00
ATOM	2958	OW	SOL	292	50.260	6.140	49.530	1.00	0.00
ATOM	2959	HW1	SOL	292	49.800	6.200	48.690	1.00	0.00
ATOM	2960	HW2	SOL	292	50.350	7.050	49.820	1.00	0.00
ATOM	2961	OW	SOL	293	9.710	5.410	5.880	1.00	0.00
ATOM	2962	HW1	SOL	293	9.770	5.890	6.700	1.00	0.00
ATOM	2963	HW2	SOL	293	9.390	4.540	6.140	1.00	0.00
ATOM	2964	OW	SOL	294	41.210	11.560	48.340	1.00	0.00
ATOM	2965	HW1	SOL	294	40.490	12.180	48.280	1.00	0.00
ATOM	2966	HW2	SOL	294	41.210	11.110	47.490	1.00	0.00
ATOM	2967	OW	SOL	295	40.230	1.700	32.610	1.00	0.00
ATOM	2968	HW1	SOL	295	40.070	1.380	33.490	1.00	0.00
ATOM	2969	HW2	SOL	295	39.380	1.680	32.180	1.00	0.00
ATOM	2970	OW	SOL	296	7.850	5.830	17.360	1.00	0.00
ATOM	2971	HW1	SOL	296	7.280	6.200	18.030	1.00	0.00
ATOM	2972	HW2	SOL	296	8.570	6.460	17.280	1.00	0.00
ATOM	2973	OW	SOL	297	44.730	14.580	39.090	1.00	0.00
ATOM	2974	HW1	SOL	297	44.170	13.830	38.920	1.00	0.00
ATOM	2975	HW2	SOL	297	45.620	14.250	38.990	1.00	0.00
ATOM	2976	OW	SOL	298	40.000	23.580	48.820	1.00	0.00
ATOM	2977	HW1	SOL	298	39.190	23.810	48.350	1.00	0.00
ATOM	2978	HW2	SOL	298	40.290	24.410	49.210	1.00	0.00
ATOM	2979	OW	SOL	299	4.940	7.420	25.150	1.00	0.00
ATOM	2980	HW1	SOL	299	5.440	8.190	25.420	1.00	0.00
ATOM	2981	HW2	SOL	299	4.030	7.660	25.350	1.00	0.00
ATOM	2982	OW	SOL	300	6.430	35.510	8.080	1.00	0.00
ATOM	2983	HW1	SOL	300	7.060	36.020	8.590	1.00	0.00
ATOM	2984	HW2	SOL	300	6.920	35.250	7.300	1.00	0.00
ATOM	2985	OW	SOL	301	44.850	17.450	44.850	1.00	0.00
ATOM	2986	HW1	SOL	301	44.240	17.470	45.580	1.00	0.00
ATOM	2987	HW2	SOL	301	44.440	18.010	44.180	1.00	0.00
ATOM	2988	OW	SOL	302	3.490	5.900	5.950	1.00	0.00
ATOM	2989	HW1	SOL	302	2.620	5.920	5.560	1.00	0.00
ATOM	2990	HW2	SOL	302	4.060	6.270	5.270	1.00	0.00
ATOM	2991	OW	SOL	303	2.480	20.820	17.940	1.00	0.00

ATOM	2992	HW1	SOL	303	2.650	20.210	18.660	1.00	0.00
ATOM	2993	HW2	SOL	303	2.720	20.330	17.150	1.00	0.00
ATOM	2994	OW	SOL	304	3.990	44.820	10.760	1.00	0.00
ATOM	2995	HW1	SOL	304	3.080	45.120	10.700	1.00	0.00
ATOM	2996	HW2	SOL	304	4.060	44.140	10.080	1.00	0.00
ATOM	2997	OW	SOL	305	49.540	45.130	45.670	1.00	0.00
ATOM	2998	HW1	SOL	305	50.320	45.180	46.220	1.00	0.00
ATOM	2999	HW2	SOL	305	49.870	44.910	44.800	1.00	0.00
ATOM	3000	OW	SOL	306	54.580	20.670	11.270	1.00	0.00
ATOM	3001	HW1	SOL	306	54.960	21.400	10.790	1.00	0.00
ATOM	3002	HW2	SOL	306	53.670	20.640	10.990	1.00	0.00
ATOM	3003	OW	SOL	307	22.980	54.880	13.090	1.00	0.00
ATOM	3004	HW1	SOL	307	22.780	55.180	13.980	1.00	0.00
ATOM	3005	HW2	SOL	307	23.920	54.990	13.000	1.00	0.00
ATOM	3006	OW	SOL	308	30.170	10.750	8.680	1.00	0.00
ATOM	3007	HW1	SOL	308	31.080	10.520	8.910	1.00	0.00
ATOM	3008	HW2	SOL	308	30.260	11.320	7.920	1.00	0.00
ATOM	3009	OW	SOL	309	47.710	35.530	54.930	1.00	0.00
ATOM	3010	HW1	SOL	309	47.050	35.990	55.450	1.00	0.00
ATOM	3011	HW2	SOL	309	48.150	36.230	54.430	1.00	0.00
ATOM	3012	OW	SOL	310	35.290	8.880	49.910	1.00	0.00
ATOM	3013	HW1	SOL	310	35.540	8.260	50.600	1.00	0.00
ATOM	3014	HW2	SOL	310	35.430	8.410	49.100	1.00	0.00
ATOM	3015	OW	SOL	311	41.630	21.170	48.920	1.00	0.00
ATOM	3016	HW1	SOL	311	41.330	22.070	48.840	1.00	0.00
ATOM	3017	HW2	SOL	311	40.830	20.660	49.050	1.00	0.00
ATOM	3018	OW	SOL	312	40.980	25.190	22.990	1.00	0.00
ATOM	3019	HW1	SOL	312	40.770	24.300	22.730	1.00	0.00
ATOM	3020	HW2	SOL	312	40.150	25.570	23.270	1.00	0.00
ATOM	3021	OW	SOL	313	28.900	17.330	47.050	1.00	0.00
ATOM	3022	HW1	SOL	313	29.170	16.670	47.690	1.00	0.00
ATOM	3023	HW2	SOL	313	29.570	18.020	47.130	1.00	0.00
ATOM	3024	OW	SOL	314	31.630	32.330	0.540	1.00	0.00
ATOM	3025	HW1	SOL	314	32.000	33.190	0.350	1.00	0.00
ATOM	3026	HW2	SOL	314	32.310	31.710	0.270	1.00	0.00
ATOM	3027	OW	SOL	315	51.840	4.750	20.500	1.00	0.00
ATOM	3028	HW1	SOL	315	51.940	4.010	21.090	1.00	0.00
ATOM	3029	HW2	SOL	315	52.670	5.230	20.580	1.00	0.00
ATOM	3030	OW	SOL	316	23.020	24.940	44.870	1.00	0.00
ATOM	3031	HW1	SOL	316	23.290	24.420	45.630	1.00	0.00
ATOM	3032	HW2	SOL	316	22.060	24.990	44.950	1.00	0.00
ATOM	3033	OW	SOL	317	4.320	18.480	17.580	1.00	0.00
ATOM	3034	HW1	SOL	317	4.470	17.550	17.720	1.00	0.00
ATOM	3035	HW2	SOL	317	4.710	18.650	16.720	1.00	0.00

ATOM	3036	OW	SOL	318	50.180	47.630	20.730	1.00	0.00
ATOM	3037	HW1	SOL	318	50.300	46.680	20.690	1.00	0.00
ATOM	3038	HW2	SOL	318	49.700	47.850	19.940	1.00	0.00
ATOM	3039	OW	SOL	319	25.980	23.680	14.050	1.00	0.00
ATOM	3040	HW1	SOL	319	25.100	23.970	13.800	1.00	0.00
ATOM	3041	HW2	SOL	319	26.280	23.160	13.310	1.00	0.00
ATOM	3042	OW	SOL	320	8.840	54.140	44.750	1.00	0.00
ATOM	3043	HW1	SOL	320	9.240	53.770	43.970	1.00	0.00
ATOM	3044	HW2	SOL	320	9.000	55.080	44.680	1.00	0.00
ATOM	3045	OW	SOL	321	6.510	2.630	42.700	1.00	0.00
ATOM	3046	HW1	SOL	321	5.920	2.080	43.220	1.00	0.00
ATOM	3047	HW2	SOL	321	6.050	2.750	41.870	1.00	0.00
ATOM	3048	OW	SOL	322	46.490	46.230	24.740	1.00	0.00
ATOM	3049	HW1	SOL	322	46.660	45.660	23.990	1.00	0.00
ATOM	3050	HW2	SOL	322	47.290	46.740	24.840	1.00	0.00
ATOM	3051	OW	SOL	323	29.520	16.460	55.020	1.00	0.00
ATOM	3052	HW1	SOL	323	29.990	16.220	54.220	1.00	0.00
ATOM	3053	HW2	SOL	323	28.590	16.350	54.790	1.00	0.00
ATOM	3054	OW	SOL	324	50.710	48.490	5.660	1.00	0.00
ATOM	3055	HW1	SOL	324	51.630	48.330	5.440	1.00	0.00
ATOM	3056	HW2	SOL	324	50.220	48.120	4.930	1.00	0.00
ATOM	3057	OW	SOL	325	46.580	49.750	55.590	1.00	0.00
ATOM	3058	HW1	SOL	325	45.900	50.160	56.130	1.00	0.00
ATOM	3059	HW2	SOL	325	46.190	49.680	54.720	1.00	0.00
ATOM	3060	OW	SOL	326	2.160	35.430	50.890	1.00	0.00
ATOM	3061	HW1	SOL	326	1.910	34.680	50.340	1.00	0.00
ATOM	3062	HW2	SOL	326	1.480	35.470	51.560	1.00	0.00
ATOM	3063	OW	SOL	327	44.210	11.120	8.730	1.00	0.00
ATOM	3064	HW1	SOL	327	44.590	12.000	8.850	1.00	0.00
ATOM	3065	HW2	SOL	327	44.040	10.820	9.620	1.00	0.00
ATOM	3066	OW	SOL	328	17.850	3.900	34.660	1.00	0.00
ATOM	3067	HW1	SOL	328	17.410	3.220	34.140	1.00	0.00
ATOM	3068	HW2	SOL	328	17.150	4.310	35.160	1.00	0.00
ATOM	3069	OW	SOL	329	21.850	25.260	15.120	1.00	0.00
ATOM	3070	HW1	SOL	329	22.500	24.650	15.490	1.00	0.00
ATOM	3071	HW2	SOL	329	21.020	24.790	15.200	1.00	0.00
ATOM	3072	OW	SOL	330	47.180	37.550	49.480	1.00	0.00
ATOM	3073	HW1	SOL	330	47.050	37.240	48.590	1.00	0.00
ATOM	3074	HW2	SOL	330	47.330	36.760	49.990	1.00	0.00
ATOM	3075	OW	SOL	331	18.240	4.780	6.110	1.00	0.00
ATOM	3076	HW1	SOL	331	18.160	4.050	6.710	1.00	0.00
ATOM	3077	HW2	SOL	331	19.180	4.980	6.090	1.00	0.00
ATOM	3078	OW	SOL	332	47.650	30.920	24.940	1.00	0.00
ATOM	3079	HW1	SOL	332	47.750	30.970	23.990	1.00	0.00

ATOM	3080	HW2	SOL	332	46.930	31.520	25.130	1.00	0.00
ATOM	3081	OW	SOL	333	22.330	9.200	19.890	1.00	0.00
ATOM	3082	HW1	SOL	333	22.670	8.370	20.220	1.00	0.00
ATOM	3083	HW2	SOL	333	22.400	9.130	18.940	1.00	0.00
ATOM	3084	OW	SOL	334	13.390	6.360	42.370	1.00	0.00
ATOM	3085	HW1	SOL	334	13.180	6.880	43.150	1.00	0.00
ATOM	3086	HW2	SOL	334	13.610	5.500	42.720	1.00	0.00
ATOM	3087	OW	SOL	335	55.440	31.480	6.120	1.00	0.00
ATOM	3088	HW1	SOL	335	54.760	30.980	5.670	1.00	0.00
ATOM	3089	HW2	SOL	335	55.060	32.350	6.240	1.00	0.00
ATOM	3090	OW	SOL	336	5.680	30.610	26.030	1.00	0.00
ATOM	3091	HW1	SOL	336	4.780	30.340	25.870	1.00	0.00
ATOM	3092	HW2	SOL	336	6.010	30.870	25.170	1.00	0.00
ATOM	3093	OW	SOL	337	48.000	7.430	36.890	1.00	0.00
ATOM	3094	HW1	SOL	337	48.620	8.060	37.260	1.00	0.00
ATOM	3095	HW2	SOL	337	47.440	7.960	36.320	1.00	0.00
ATOM	3096	OW	SOL	338	14.410	16.220	22.610	1.00	0.00
ATOM	3097	HW1	SOL	338	13.630	16.290	22.050	1.00	0.00
ATOM	3098	HW2	SOL	338	14.610	15.290	22.630	1.00	0.00
ATOM	3099	OW	SOL	339	40.900	52.490	14.460	1.00	0.00
ATOM	3100	HW1	SOL	339	40.270	53.200	14.330	1.00	0.00
ATOM	3101	HW2	SOL	339	40.590	52.040	15.240	1.00	0.00
ATOM	3102	OW	SOL	340	21.400	7.850	30.530	1.00	0.00
ATOM	3103	HW1	SOL	340	20.480	7.620	30.390	1.00	0.00
ATOM	3104	HW2	SOL	340	21.790	7.810	29.660	1.00	0.00
ATOM	3105	OW	SOL	341	54.510	46.040	0.390	1.00	0.00
ATOM	3106	HW1	SOL	341	53.900	45.330	0.220	1.00	0.00
ATOM	3107	HW2	SOL	341	54.410	46.230	1.320	1.00	0.00
ATOM	3108	OW	SOL	342	44.500	8.430	47.780	1.00	0.00
ATOM	3109	HW1	SOL	342	44.470	7.660	48.360	1.00	0.00
ATOM	3110	HW2	SOL	342	43.800	8.280	47.150	1.00	0.00
ATOM	3111	OW	SOL	343	5.040	26.410	52.880	1.00	0.00
ATOM	3112	HW1	SOL	343	5.770	26.660	52.310	1.00	0.00
ATOM	3113	HW2	SOL	343	4.670	27.250	53.170	1.00	0.00
ATOM	3114	OW	SOL	344	6.340	5.230	53.180	1.00	0.00
ATOM	3115	HW1	SOL	344	6.320	5.380	52.230	1.00	0.00
ATOM	3116	HW2	SOL	344	7.100	4.660	53.310	1.00	0.00
ATOM	3117	OW	SOL	345	25.610	54.930	13.380	1.00	0.00
ATOM	3118	HW1	SOL	345	25.470	54.250	14.040	1.00	0.00
ATOM	3119	HW2	SOL	345	26.420	54.670	12.930	1.00	0.00
ATOM	3120	OW	SOL	346	43.260	10.320	15.600	1.00	0.00
ATOM	3121	HW1	SOL	346	42.360	10.010	15.710	1.00	0.00
ATOM	3122	HW2	SOL	346	43.400	10.300	14.650	1.00	0.00
ATOM	3123	OW	SOL	347	8.520	34.890	43.380	1.00	0.00

ATOM	3124	HW1	SOL	347	9.140	34.430	43.950	1.00	0.00
ATOM	3125	HW2	SOL	347	7.670	34.500	43.580	1.00	0.00
ATOM	3126	OW	SOL	348	28.280	54.050	12.320	1.00	0.00
ATOM	3127	HW1	SOL	348	27.990	53.180	12.590	1.00	0.00
ATOM	3128	HW2	SOL	348	28.120	54.080	11.370	1.00	0.00
ATOM	3129	OW	SOL	349	55.700	34.230	18.520	1.00	0.00
ATOM	3130	HW1	SOL	349	54.940	34.110	19.100	1.00	0.00
ATOM	3131	HW2	SOL	349	55.340	34.670	17.750	1.00	0.00
ATOM	3132	OW	SOL	350	47.350	9.350	9.900	1.00	0.00
ATOM	3133	HW1	SOL	350	48.060	9.220	10.530	1.00	0.00
ATOM	3134	HW2	SOL	350	47.790	9.630	9.100	1.00	0.00
ATOM	3135	OW	SOL	351	17.840	4.410	48.050	1.00	0.00
ATOM	3136	HW1	SOL	351	18.210	5.280	47.990	1.00	0.00
ATOM	3137	HW2	SOL	351	17.610	4.170	47.150	1.00	0.00
ATOM	3138	OW	SOL	352	54.210	51.210	2.900	1.00	0.00
ATOM	3139	HW1	SOL	352	55.080	51.280	2.500	1.00	0.00
ATOM	3140	HW2	SOL	352	53.740	50.600	2.340	1.00	0.00
ATOM	3141	OW	SOL	353	37.160	17.830	20.910	1.00	0.00
ATOM	3142	HW1	SOL	353	36.570	17.310	21.440	1.00	0.00
ATOM	3143	HW2	SOL	353	37.390	17.270	20.170	1.00	0.00
ATOM	3144	OW	SOL	354	43.210	37.700	0.940	1.00	0.00
ATOM	3145	HW1	SOL	354	43.360	37.010	0.300	1.00	0.00
ATOM	3146	HW2	SOL	354	43.160	37.240	1.780	1.00	0.00
ATOM	3147	OW	SOL	355	40.710	13.130	15.600	1.00	0.00
ATOM	3148	HW1	SOL	355	41.330	13.750	15.210	1.00	0.00
ATOM	3149	HW2	SOL	355	40.920	13.130	16.530	1.00	0.00
ATOM	3150	OW	SOL	356	31.180	20.720	43.720	1.00	0.00
ATOM	3151	HW1	SOL	356	31.720	21.510	43.660	1.00	0.00
ATOM	3152	HW2	SOL	356	31.740	20.030	43.380	1.00	0.00
ATOM	3153	OW	SOL	357	52.580	46.230	24.490	1.00	0.00
ATOM	3154	HW1	SOL	357	51.840	45.690	24.750	1.00	0.00
ATOM	3155	HW2	SOL	357	53.350	45.770	24.830	1.00	0.00
ATOM	3156	OW	SOL	358	49.140	14.310	54.450	1.00	0.00
ATOM	3157	HW1	SOL	358	48.250	13.950	54.470	1.00	0.00
ATOM	3158	HW2	SOL	358	49.700	13.570	54.670	1.00	0.00
ATOM	3159	OW	SOL	359	19.280	9.610	27.700	1.00	0.00
ATOM	3160	HW1	SOL	359	18.350	9.670	27.460	1.00	0.00
ATOM	3161	HW2	SOL	359	19.300	9.010	28.450	1.00	0.00
ATOM	3162	OW	SOL	360	17.320	53.360	43.730	1.00	0.00
ATOM	3163	HW1	SOL	360	17.040	53.360	44.650	1.00	0.00
ATOM	3164	HW2	SOL	360	18.270	53.330	43.770	1.00	0.00
ATOM	3165	OW	SOL	361	40.230	35.700	28.720	1.00	0.00
ATOM	3166	HW1	SOL	361	40.400	36.490	29.240	1.00	0.00
ATOM	3167	HW2	SOL	361	40.940	35.680	28.080	1.00	0.00

ATOM	3168	OW	SOL	362	4.460	46.030	40.930	1.00	0.00
ATOM	3169	HW1	SOL	362	3.660	45.540	40.770	1.00	0.00
ATOM	3170	HW2	SOL	362	4.220	46.660	41.620	1.00	0.00
ATOM	3171	OW	SOL	363	21.000	27.470	2.710	1.00	0.00
ATOM	3172	HW1	SOL	363	20.340	26.780	2.830	1.00	0.00
ATOM	3173	HW2	SOL	363	20.760	28.130	3.360	1.00	0.00
ATOM	3174	OW	SOL	364	2.870	3.200	42.560	1.00	0.00
ATOM	3175	HW1	SOL	364	2.710	2.380	43.030	1.00	0.00
ATOM	3176	HW2	SOL	364	3.330	2.930	41.760	1.00	0.00
ATOM	3177	OW	SOL	365	55.190	44.780	18.750	1.00	0.00
ATOM	3178	HW1	SOL	365	55.860	45.250	18.240	1.00	0.00
ATOM	3179	HW2	SOL	365	55.690	44.290	19.400	1.00	0.00
ATOM	3180	OW	SOL	366	41.600	31.120	1.910	1.00	0.00
ATOM	3181	HW1	SOL	366	42.010	31.610	1.190	1.00	0.00
ATOM	3182	HW2	SOL	366	40.910	30.610	1.480	1.00	0.00
ATOM	3183	OW	SOL	367	17.810	29.200	0.370	1.00	0.00
ATOM	3184	HW1	SOL	367	18.760	29.070	0.410	1.00	0.00
ATOM	3185	HW2	SOL	367	17.540	29.270	1.280	1.00	0.00
ATOM	3186	OW	SOL	368	11.510	40.820	4.650	1.00	0.00
ATOM	3187	HW1	SOL	368	11.610	39.870	4.700	1.00	0.00
ATOM	3188	HW2	SOL	368	12.370	41.140	4.370	1.00	0.00
ATOM	3189	OW	SOL	369	41.860	39.100	32.290	1.00	0.00
ATOM	3190	HW1	SOL	369	41.970	38.210	32.630	1.00	0.00
ATOM	3191	HW2	SOL	369	41.150	39.470	32.830	1.00	0.00
ATOM	3192	OW	SOL	370	21.650	34.880	48.910	1.00	0.00
ATOM	3193	HW1	SOL	370	20.810	35.320	49.020	1.00	0.00
ATOM	3194	HW2	SOL	370	22.030	35.280	48.130	1.00	0.00
ATOM	3195	OW	SOL	371	5.840	23.580	53.660	1.00	0.00
ATOM	3196	HW1	SOL	371	5.270	24.340	53.520	1.00	0.00
ATOM	3197	HW2	SOL	371	5.630	23.280	54.540	1.00	0.00
ATOM	3198	OW	SOL	372	34.840	42.140	49.590	1.00	0.00
ATOM	3199	HW1	SOL	372	34.510	41.800	50.420	1.00	0.00
ATOM	3200	HW2	SOL	372	35.250	42.980	49.820	1.00	0.00
ATOM	3201	OW	SOL	373	41.290	37.900	29.970	1.00	0.00
ATOM	3202	HW1	SOL	373	41.390	38.510	30.700	1.00	0.00
ATOM	3203	HW2	SOL	373	41.550	37.050	30.330	1.00	0.00
ATOM	3204	OW	SOL	374	44.750	3.580	32.930	1.00	0.00
ATOM	3205	HW1	SOL	374	43.810	3.620	32.740	1.00	0.00
ATOM	3206	HW2	SOL	374	45.170	3.860	32.120	1.00	0.00
ATOM	3207	OW	SOL	375	29.000	46.680	8.030	1.00	0.00
ATOM	3208	HW1	SOL	375	29.810	46.940	7.590	1.00	0.00
ATOM	3209	HW2	SOL	375	29.290	46.180	8.790	1.00	0.00
ATOM	3210	OW	SOL	376	43.980	4.570	6.460	1.00	0.00
ATOM	3211	HW1	SOL	376	44.750	5.070	6.170	1.00	0.00

ATOM	3212	HW2	SOL	376	43.370	5.240	6.780	1.00	0.00
ATOM	3213	OW	SOL	377	13.480	31.340	23.300	1.00	0.00
ATOM	3214	HW1	SOL	377	14.180	30.870	23.750	1.00	0.00
ATOM	3215	HW2	SOL	377	13.300	30.820	22.520	1.00	0.00
ATOM	3216	OW	SOL	378	2.390	37.110	39.380	1.00	0.00
ATOM	3217	HW1	SOL	378	1.620	36.780	38.930	1.00	0.00
ATOM	3218	HW2	SOL	378	2.060	37.450	40.210	1.00	0.00
ATOM	3219	OW	SOL	379	16.920	51.060	7.760	1.00	0.00
ATOM	3220	HW1	SOL	379	16.610	51.960	7.820	1.00	0.00
ATOM	3221	HW2	SOL	379	17.790	51.140	7.380	1.00	0.00
ATOM	3222	OW	SOL	380	3.110	8.140	48.200	1.00	0.00
ATOM	3223	HW1	SOL	380	2.410	8.590	47.740	1.00	0.00
ATOM	3224	HW2	SOL	380	3.100	8.500	49.080	1.00	0.00
ATOM	3225	OW	SOL	381	50.080	51.060	51.330	1.00	0.00
ATOM	3226	HW1	SOL	381	50.860	51.230	50.790	1.00	0.00
ATOM	3227	HW2	SOL	381	50.340	51.350	52.200	1.00	0.00
ATOM	3228	OW	SOL	382	20.090	18.700	21.700	1.00	0.00
ATOM	3229	HW1	SOL	382	20.230	17.780	21.470	1.00	0.00
ATOM	3230	HW2	SOL	382	20.960	19.040	21.910	1.00	0.00
ATOM	3231	OW	SOL	383	53.920	42.610	43.250	1.00	0.00
ATOM	3232	HW1	SOL	383	54.480	42.370	43.980	1.00	0.00
ATOM	3233	HW2	SOL	383	53.040	42.640	43.620	1.00	0.00
ATOM	3234	OW	SOL	384	45.440	26.280	16.480	1.00	0.00
ATOM	3235	HW1	SOL	384	45.900	25.480	16.750	1.00	0.00
ATOM	3236	HW2	SOL	384	44.960	26.030	15.690	1.00	0.00
ATOM	3237	OW	SOL	385	43.830	20.610	27.790	1.00	0.00
ATOM	3238	HW1	SOL	385	44.180	21.010	26.990	1.00	0.00
ATOM	3239	HW2	SOL	385	44.600	20.240	28.230	1.00	0.00
ATOM	3240	OW	SOL	386	1.970	17.840	36.880	1.00	0.00
ATOM	3241	HW1	SOL	386	2.280	18.460	37.540	1.00	0.00
ATOM	3242	HW2	SOL	386	2.620	17.890	36.180	1.00	0.00
ATOM	3243	OW	SOL	387	42.400	34.780	26.980	1.00	0.00
ATOM	3244	HW1	SOL	387	42.290	33.930	27.400	1.00	0.00
ATOM	3245	HW2	SOL	387	42.570	34.580	26.060	1.00	0.00
ATOM	3246	OW	SOL	388	4.610	34.460	46.170	1.00	0.00
ATOM	3247	HW1	SOL	388	3.970	34.470	45.460	1.00	0.00
ATOM	3248	HW2	SOL	388	4.100	34.650	46.960	1.00	0.00
ATOM	3249	OW	SOL	389	52.640	37.460	8.490	1.00	0.00
ATOM	3250	HW1	SOL	389	52.840	36.530	8.610	1.00	0.00
ATOM	3251	HW2	SOL	389	53.170	37.910	9.140	1.00	0.00
ATOM	3252	OW	SOL	390	45.240	29.140	21.680	1.00	0.00
ATOM	3253	HW1	SOL	390	44.930	29.740	21.010	1.00	0.00
ATOM	3254	HW2	SOL	390	45.680	28.440	21.200	1.00	0.00
ATOM	3255	OW	SOL	391	0.390	35.000	24.700	1.00	0.00

ATOM	3256	HW1	SOL	391	1.200	34.750	25.150	1.00	0.00
ATOM	3257	HW2	SOL	391	0.460	34.590	23.840	1.00	0.00
ATOM	3258	OW	SOL	392	25.430	55.650	9.310	1.00	0.00
ATOM	3259	HW1	SOL	392	24.480	55.560	9.380	1.00	0.00
ATOM	3260	HW2	SOL	392	25.770	54.880	9.770	1.00	0.00
ATOM	3261	OW	SOL	393	43.570	42.280	1.660	1.00	0.00
ATOM	3262	HW1	SOL	393	43.220	42.570	2.500	1.00	0.00
ATOM	3263	HW2	SOL	393	43.010	41.540	1.410	1.00	0.00
ATOM	3264	OW	SOL	394	36.940	53.160	46.480	1.00	0.00
ATOM	3265	HW1	SOL	394	37.570	52.570	46.060	1.00	0.00
ATOM	3266	HW2	SOL	394	36.790	52.760	47.340	1.00	0.00
ATOM	3267	OW	SOL	395	11.260	42.700	12.120	1.00	0.00
ATOM	3268	HW1	SOL	395	11.550	41.920	12.590	1.00	0.00
ATOM	3269	HW2	SOL	395	11.560	42.580	11.230	1.00	0.00
ATOM	3270	OW	SOL	396	32.200	16.620	50.210	1.00	0.00
ATOM	3271	HW1	SOL	396	32.710	17.090	49.550	1.00	0.00
ATOM	3272	HW2	SOL	396	32.450	17.030	51.040	1.00	0.00
ATOM	3273	OW	SOL	397	53.690	31.970	35.250	1.00	0.00
ATOM	3274	HW1	SOL	397	53.250	32.160	36.080	1.00	0.00
ATOM	3275	HW2	SOL	397	54.620	32.030	35.460	1.00	0.00
ATOM	3276	OW	SOL	398	27.810	12.660	38.120	1.00	0.00
ATOM	3277	HW1	SOL	398	28.760	12.560	38.180	1.00	0.00
ATOM	3278	HW2	SOL	398	27.590	12.380	37.240	1.00	0.00
ATOM	3279	OW	SOL	399	24.560	30.680	29.980	1.00	0.00
ATOM	3280	HW1	SOL	399	24.110	30.640	29.140	1.00	0.00
ATOM	3281	HW2	SOL	399	25.200	29.970	29.950	1.00	0.00
ATOM	3282	OW	SOL	400	40.080	47.670	2.940	1.00	0.00
ATOM	3283	HW1	SOL	400	40.580	48.450	2.730	1.00	0.00
ATOM	3284	HW2	SOL	400	39.840	47.770	3.860	1.00	0.00
ATOM	3285	OW	SOL	401	18.760	23.520	43.100	1.00	0.00
ATOM	3286	HW1	SOL	401	19.140	22.700	42.800	1.00	0.00
ATOM	3287	HW2	SOL	401	19.150	24.190	42.530	1.00	0.00
ATOM	3288	OW	SOL	402	21.330	29.670	4.610	1.00	0.00
ATOM	3289	HW1	SOL	402	20.840	30.200	3.980	1.00	0.00
ATOM	3290	HW2	SOL	402	22.220	30.010	4.550	1.00	0.00
ATOM	3291	OW	SOL	403	1.010	50.500	37.520	1.00	0.00
ATOM	3292	HW1	SOL	403	1.140	49.870	38.220	1.00	0.00
ATOM	3293	HW2	SOL	403	0.400	50.070	36.920	1.00	0.00
ATOM	3294	OW	SOL	404	19.220	1.850	17.320	1.00	0.00
ATOM	3295	HW1	SOL	404	19.470	1.190	16.680	1.00	0.00
ATOM	3296	HW2	SOL	404	18.450	1.490	17.760	1.00	0.00
ATOM	3297	OW	SOL	405	54.020	23.070	54.910	1.00	0.00
ATOM	3298	HW1	SOL	405	53.940	23.340	55.830	1.00	0.00
ATOM	3299	HW2	SOL	405	54.700	23.650	54.550	1.00	0.00

ATOM	3300	OW	SOL	406	1.760	30.970	22.670	1.00	0.00
ATOM	3301	HW1	SOL	406	2.620	30.560	22.730	1.00	0.00
ATOM	3302	HW2	SOL	406	1.160	30.360	23.100	1.00	0.00
ATOM	3303	OW	SOL	407	29.220	6.460	39.730	1.00	0.00
ATOM	3304	HW1	SOL	407	29.720	5.800	39.250	1.00	0.00
ATOM	3305	HW2	SOL	407	29.540	6.410	40.630	1.00	0.00
ATOM	3306	OW	SOL	408	38.300	9.330	43.270	1.00	0.00
ATOM	3307	HW1	SOL	408	37.970	9.580	44.130	1.00	0.00
ATOM	3308	HW2	SOL	408	39.140	8.900	43.450	1.00	0.00
ATOM	3309	OW	SOL	409	55.520	50.940	49.620	1.00	0.00
ATOM	3310	HW1	SOL	409	55.400	51.550	50.350	1.00	0.00
ATOM	3311	HW2	SOL	409	56.470	50.900	49.490	1.00	0.00
ATOM	3312	OW	SOL	410	5.280	34.160	41.150	1.00	0.00
ATOM	3313	HW1	SOL	410	5.220	35.020	41.570	1.00	0.00
ATOM	3314	HW2	SOL	410	5.260	33.540	41.880	1.00	0.00
ATOM	3315	OW	SOL	411	0.030	44.560	15.390	1.00	0.00
ATOM	3316	HW1	SOL	411	0.930	44.440	15.080	1.00	0.00
ATOM	3317	HW2	SOL	411	0.060	45.410	15.840	1.00	0.00
ATOM	3318	OW	SOL	412	25.050	20.440	26.060	1.00	0.00
ATOM	3319	HW1	SOL	412	24.360	20.770	26.630	1.00	0.00
ATOM	3320	HW2	SOL	412	24.620	19.810	25.490	1.00	0.00
ATOM	3321	OW	SOL	413	50.430	42.540	8.540	1.00	0.00
ATOM	3322	HW1	SOL	413	49.830	42.630	9.280	1.00	0.00
ATOM	3323	HW2	SOL	413	50.030	41.880	7.980	1.00	0.00
ATOM	3324	OW	SOL	414	51.920	13.730	42.800	1.00	0.00
ATOM	3325	HW1	SOL	414	52.070	14.060	43.690	1.00	0.00
ATOM	3326	HW2	SOL	414	51.040	13.350	42.830	1.00	0.00
ATOM	3327	OW	SOL	415	52.530	23.520	47.380	1.00	0.00
ATOM	3328	HW1	SOL	415	52.190	23.760	48.240	1.00	0.00
ATOM	3329	HW2	SOL	415	53.260	22.930	47.560	1.00	0.00
ATOM	3330	OW	SOL	416	40.580	8.220	43.950	1.00	0.00
ATOM	3331	HW1	SOL	416	40.420	7.740	44.770	1.00	0.00
ATOM	3332	HW2	SOL	416	41.160	7.650	43.450	1.00	0.00
ATOM	3333	OW	SOL	417	55.450	25.740	4.860	1.00	0.00
ATOM	3334	HW1	SOL	417	55.880	25.130	4.260	1.00	0.00
ATOM	3335	HW2	SOL	417	54.600	25.900	4.460	1.00	0.00
ATOM	3336	OW	SOL	418	2.700	35.240	12.290	1.00	0.00
ATOM	3337	HW1	SOL	418	1.810	35.510	12.500	1.00	0.00
ATOM	3338	HW2	SOL	418	3.240	36.010	12.500	1.00	0.00
ATOM	3339	OW	SOL	419	6.050	0.440	30.580	1.00	0.00
ATOM	3340	HW1	SOL	419	6.170	-0.370	31.080	1.00	0.00
ATOM	3341	HW2	SOL	419	6.540	0.300	29.770	1.00	0.00
ATOM	3342	OW	SOL	420	23.910	31.870	13.730	1.00	0.00
ATOM	3343	HW1	SOL	420	24.800	31.980	14.060	1.00	0.00

ATOM	3344	HW2	SOL	420	23.650	32.740	13.440	1.00	0.00
ATOM	3345	OW	SOL	421	11.480	14.500	0.890	1.00	0.00
ATOM	3346	HW1	SOL	421	12.290	14.970	1.100	1.00	0.00
ATOM	3347	HW2	SOL	421	11.540	14.350	-0.060	1.00	0.00
ATOM	3348	OW	SOL	422	3.170	18.710	50.580	1.00	0.00
ATOM	3349	HW1	SOL	422	3.990	18.290	50.820	1.00	0.00
ATOM	3350	HW2	SOL	422	2.500	18.040	50.710	1.00	0.00
ATOM	3351	OW	SOL	423	31.540	20.450	31.470	1.00	0.00
ATOM	3352	HW1	SOL	423	31.360	21.360	31.210	1.00	0.00
ATOM	3353	HW2	SOL	423	30.690	20.020	31.440	1.00	0.00
ATOM	3354	OW	SOL	424	11.510	40.810	15.210	1.00	0.00
ATOM	3355	HW1	SOL	424	12.300	41.000	15.720	1.00	0.00
ATOM	3356	HW2	SOL	424	10.790	41.000	15.810	1.00	0.00
ATOM	3357	OW	SOL	425	40.290	37.250	52.230	1.00	0.00
ATOM	3358	HW1	SOL	425	40.040	36.620	52.900	1.00	0.00
ATOM	3359	HW2	SOL	425	40.180	38.110	52.650	1.00	0.00
ATOM	3360	OW	SOL	426	12.700	49.140	0.640	1.00	0.00
ATOM	3361	HW1	SOL	426	12.530	48.850	-0.260	1.00	0.00
ATOM	3362	HW2	SOL	426	13.410	48.580	0.940	1.00	0.00
ATOM	3363	OW	SOL	427	25.270	11.440	44.450	1.00	0.00
ATOM	3364	HW1	SOL	427	25.810	10.660	44.400	1.00	0.00
ATOM	3365	HW2	SOL	427	25.890	12.170	44.380	1.00	0.00
ATOM	3366	OW	SOL	428	54.270	35.590	16.530	1.00	0.00
ATOM	3367	HW1	SOL	428	54.510	35.400	15.630	1.00	0.00
ATOM	3368	HW2	SOL	428	53.630	36.300	16.460	1.00	0.00
ATOM	3369	OW	SOL	429	54.210	37.120	2.760	1.00	0.00
ATOM	3370	HW1	SOL	429	54.590	36.250	2.650	1.00	0.00
ATOM	3371	HW2	SOL	429	54.970	37.720	2.760	1.00	0.00
ATOM	3372	OW	SOL	430	6.180	8.300	43.040	1.00	0.00
ATOM	3373	HW1	SOL	430	5.770	9.150	42.970	1.00	0.00
ATOM	3374	HW2	SOL	430	6.860	8.300	42.360	1.00	0.00
ATOM	3375	OW	SOL	431	3.200	38.630	8.700	1.00	0.00
ATOM	3376	HW1	SOL	431	3.660	38.900	9.500	1.00	0.00
ATOM	3377	HW2	SOL	431	2.290	38.550	8.970	1.00	0.00
ATOM	3378	OW	SOL	432	55.390	48.310	18.900	1.00	0.00
ATOM	3379	HW1	SOL	432	55.520	48.000	19.800	1.00	0.00
ATOM	3380	HW2	SOL	432	55.320	49.260	18.980	1.00	0.00
ATOM	3381	OW	SOL	433	40.640	34.050	33.240	1.00	0.00
ATOM	3382	HW1	SOL	433	39.780	33.840	32.870	1.00	0.00
ATOM	3383	HW2	SOL	433	41.260	33.540	32.720	1.00	0.00
ATOM	3384	OW	SOL	434	36.760	1.410	39.120	1.00	0.00
ATOM	3385	HW1	SOL	434	36.820	0.660	38.530	1.00	0.00
ATOM	3386	HW2	SOL	434	35.840	1.690	39.050	1.00	0.00
ATOM	3387	OW	SOL	435	45.020	31.890	13.900	1.00	0.00

ATOM	3388	HW1	SOL	435	44.070	32.000	13.970	1.00	0.00
ATOM	3389	HW2	SOL	435	45.180	31.700	12.980	1.00	0.00
ATOM	3390	OW	SOL	436	46.910	22.490	4.030	1.00	0.00
ATOM	3391	HW1	SOL	436	47.270	23.080	4.700	1.00	0.00
ATOM	3392	HW2	SOL	436	47.400	22.700	3.240	1.00	0.00
ATOM	3393	OW	SOL	437	45.630	46.030	11.900	1.00	0.00
ATOM	3394	HW1	SOL	437	44.920	46.260	11.300	1.00	0.00
ATOM	3395	HW2	SOL	437	45.300	45.290	12.390	1.00	0.00
ATOM	3396	OW	SOL	438	26.670	42.860	4.690	1.00	0.00
ATOM	3397	HW1	SOL	438	26.450	43.170	5.560	1.00	0.00
ATOM	3398	HW2	SOL	438	27.410	43.400	4.420	1.00	0.00
ATOM	3399	OW	SOL	439	53.000	40.340	14.780	1.00	0.00
ATOM	3400	HW1	SOL	439	52.920	41.280	14.900	1.00	0.00
ATOM	3401	HW2	SOL	439	53.920	40.150	14.950	1.00	0.00
ATOM	3402	OW	SOL	440	40.220	9.300	21.650	1.00	0.00
ATOM	3403	HW1	SOL	440	41.150	9.530	21.670	1.00	0.00
ATOM	3404	HW2	SOL	440	40.000	9.090	22.560	1.00	0.00
ATOM	3405	OW	SOL	441	15.250	28.420	45.580	1.00	0.00
ATOM	3406	HW1	SOL	441	15.690	29.240	45.790	1.00	0.00
ATOM	3407	HW2	SOL	441	14.600	28.320	46.280	1.00	0.00
ATOM	3408	OW	SOL	442	41.340	23.220	53.930	1.00	0.00
ATOM	3409	HW1	SOL	442	41.460	23.250	54.880	1.00	0.00
ATOM	3410	HW2	SOL	442	41.820	23.970	53.600	1.00	0.00
ATOM	3411	OW	SOL	443	21.220	19.750	2.200	1.00	0.00
ATOM	3412	HW1	SOL	443	20.310	19.920	2.480	1.00	0.00
ATOM	3413	HW2	SOL	443	21.150	19.560	1.270	1.00	0.00
ATOM	3414	OW	SOL	444	10.530	48.370	23.080	1.00	0.00
ATOM	3415	HW1	SOL	444	11.350	48.780	22.800	1.00	0.00
ATOM	3416	HW2	SOL	444	10.730	48.000	23.940	1.00	0.00
ATOM	3417	OW	SOL	445	43.740	29.860	30.860	1.00	0.00
ATOM	3418	HW1	SOL	445	44.370	30.570	31.000	1.00	0.00
ATOM	3419	HW2	SOL	445	43.610	29.840	29.910	1.00	0.00
ATOM	3420	OW	SOL	446	35.010	23.190	12.690	1.00	0.00
ATOM	3421	HW1	SOL	446	34.870	23.950	12.130	1.00	0.00
ATOM	3422	HW2	SOL	446	35.640	23.480	13.340	1.00	0.00
ATOM	3423	OW	SOL	447	32.520	19.030	7.630	1.00	0.00
ATOM	3424	HW1	SOL	447	32.960	19.840	7.910	1.00	0.00
ATOM	3425	HW2	SOL	447	31.600	19.180	7.840	1.00	0.00
ATOM	3426	OW	SOL	448	0.970	0.570	49.840	1.00	0.00
ATOM	3427	HW1	SOL	448	1.170	1.250	50.480	1.00	0.00
ATOM	3428	HW2	SOL	448	0.020	0.630	49.710	1.00	0.00
ATOM	3429	OW	SOL	449	19.030	3.630	1.570	1.00	0.00
ATOM	3430	HW1	SOL	449	19.500	2.810	1.410	1.00	0.00
ATOM	3431	HW2	SOL	449	19.170	4.150	0.790	1.00	0.00

ATOM	3432	OW	SOL	450	9.950	28.260	2.970	1.00	0.00
ATOM	3433	HW1	SOL	450	9.250	27.650	2.730	1.00	0.00
ATOM	3434	HW2	SOL	450	10.730	27.720	3.060	1.00	0.00
ATOM	3435	OW	SOL	451	12.040	5.930	4.480	1.00	0.00
ATOM	3436	HW1	SOL	451	11.910	6.240	3.580	1.00	0.00
ATOM	3437	HW2	SOL	451	11.160	5.780	4.820	1.00	0.00
ATOM	3438	OW	SOL	452	45.940	29.910	34.620	1.00	0.00
ATOM	3439	HW1	SOL	452	45.660	29.260	35.270	1.00	0.00
ATOM	3440	HW2	SOL	452	46.440	29.410	33.980	1.00	0.00
ATOM	3441	OW	SOL	453	52.080	52.390	35.080	1.00	0.00
ATOM	3442	HW1	SOL	453	51.340	52.970	34.900	1.00	0.00
ATOM	3443	HW2	SOL	453	52.260	52.510	36.010	1.00	0.00
ATOM	3444	OW	SOL	454	12.120	40.590	41.450	1.00	0.00
ATOM	3445	HW1	SOL	454	11.910	41.290	42.060	1.00	0.00
ATOM	3446	HW2	SOL	454	12.400	39.860	42.000	1.00	0.00
ATOM	3447	OW	SOL	455	36.570	7.760	1.440	1.00	0.00
ATOM	3448	HW1	SOL	455	35.940	7.900	0.730	1.00	0.00
ATOM	3449	HW2	SOL	455	37.400	7.600	0.990	1.00	0.00
ATOM	3450	OW	SOL	456	16.970	13.770	55.440	1.00	0.00
ATOM	3451	HW1	SOL	456	17.560	14.320	54.930	1.00	0.00
ATOM	3452	HW2	SOL	456	17.350	12.900	55.390	1.00	0.00
ATOM	3453	OW	SOL	457	34.690	18.250	4.120	1.00	0.00
ATOM	3454	HW1	SOL	457	34.770	18.570	5.020	1.00	0.00
ATOM	3455	HW2	SOL	457	34.900	17.320	4.180	1.00	0.00
ATOM	3456	OW	SOL	458	1.670	41.120	10.800	1.00	0.00
ATOM	3457	HW1	SOL	458	2.130	40.300	10.940	1.00	0.00
ATOM	3458	HW2	SOL	458	1.050	40.940	10.090	1.00	0.00
ATOM	3459	OW	SOL	459	15.330	17.160	32.790	1.00	0.00
ATOM	3460	HW1	SOL	459	15.660	17.710	33.500	1.00	0.00
ATOM	3461	HW2	SOL	459	15.660	17.560	31.990	1.00	0.00
ATOM	3462	OW	SOL	460	35.310	46.880	6.580	1.00	0.00
ATOM	3463	HW1	SOL	460	34.600	46.760	5.950	1.00	0.00
ATOM	3464	HW2	SOL	460	35.190	46.180	7.210	1.00	0.00
ATOM	3465	OW	SOL	461	47.010	22.760	43.150	1.00	0.00
ATOM	3466	HW1	SOL	461	47.690	23.420	43.140	1.00	0.00
ATOM	3467	HW2	SOL	461	46.920	22.520	44.080	1.00	0.00
ATOM	3468	OW	SOL	462	47.730	13.850	1.490	1.00	0.00
ATOM	3469	HW1	SOL	462	47.340	13.460	0.710	1.00	0.00
ATOM	3470	HW2	SOL	462	47.070	13.740	2.180	1.00	0.00
ATOM	3471	OW	SOL	463	7.830	30.680	10.140	1.00	0.00
ATOM	3472	HW1	SOL	463	7.460	31.550	10.010	1.00	0.00
ATOM	3473	HW2	SOL	463	7.180	30.080	9.770	1.00	0.00
ATOM	3474	OW	SOL	464	12.790	29.820	51.940	1.00	0.00
ATOM	3475	HW1	SOL	464	13.360	29.850	52.700	1.00	0.00

ATOM	3476	HW2	SOL	464	11.990	29.390	52.260	1.00	0.00
ATOM	3477	OW	SOL	465	7.000	40.110	8.870	1.00	0.00
ATOM	3478	HW1	SOL	465	7.840	40.100	8.410	1.00	0.00
ATOM	3479	HW2	SOL	465	6.950	40.980	9.260	1.00	0.00
ATOM	3480	OW	SOL	466	27.780	52.800	3.860	1.00	0.00
ATOM	3481	HW1	SOL	466	28.400	52.090	3.690	1.00	0.00
ATOM	3482	HW2	SOL	466	28.190	53.570	3.470	1.00	0.00
ATOM	3483	OW	SOL	467	2.000	26.240	15.890	1.00	0.00
ATOM	3484	HW1	SOL	467	2.710	26.850	16.120	1.00	0.00
ATOM	3485	HW2	SOL	467	1.710	26.530	15.030	1.00	0.00
ATOM	3486	OW	SOL	468	39.910	35.260	18.140	1.00	0.00
ATOM	3487	HW1	SOL	468	39.170	35.290	17.540	1.00	0.00
ATOM	3488	HW2	SOL	468	39.890	34.370	18.500	1.00	0.00
ATOM	3489	OW	SOL	469	27.860	7.400	10.140	1.00	0.00
ATOM	3490	HW1	SOL	469	27.350	6.860	10.740	1.00	0.00
ATOM	3491	HW2	SOL	469	27.570	7.120	9.270	1.00	0.00
ATOM	3492	OW	SOL	470	1.080	51.070	6.420	1.00	0.00
ATOM	3493	HW1	SOL	470	1.010	51.330	7.330	1.00	0.00
ATOM	3494	HW2	SOL	470	1.990	50.770	6.320	1.00	0.00
ATOM	3495	OW	SOL	471	52.100	31.980	30.860	1.00	0.00
ATOM	3496	HW1	SOL	471	51.900	31.940	31.800	1.00	0.00
ATOM	3497	HW2	SOL	471	52.410	32.870	30.720	1.00	0.00
ATOM	3498	OW	SOL	472	16.690	15.450	41.400	1.00	0.00
ATOM	3499	HW1	SOL	472	16.240	15.180	42.200	1.00	0.00
ATOM	3500	HW2	SOL	472	16.490	16.380	41.310	1.00	0.00
ATOM	3501	OW	SOL	473	53.870	37.740	37.080	1.00	0.00
ATOM	3502	HW1	SOL	473	53.910	37.760	38.040	1.00	0.00
ATOM	3503	HW2	SOL	473	54.350	36.940	36.840	1.00	0.00
ATOM	3504	OW	SOL	474	11.320	22.820	20.590	1.00	0.00
ATOM	3505	HW1	SOL	474	11.880	22.340	19.980	1.00	0.00
ATOM	3506	HW2	SOL	474	11.830	22.880	21.400	1.00	0.00
ATOM	3507	OW	SOL	475	11.010	39.470	10.900	1.00	0.00
ATOM	3508	HW1	SOL	475	11.630	39.870	11.510	1.00	0.00
ATOM	3509	HW2	SOL	475	10.710	38.680	11.350	1.00	0.00
ATOM	3510	OW	SOL	476	12.430	28.300	36.380	1.00	0.00
ATOM	3511	HW1	SOL	476	11.680	28.000	36.890	1.00	0.00
ATOM	3512	HW2	SOL	476	12.170	29.140	36.020	1.00	0.00
ATOM	3513	OW	SOL	477	45.790	20.330	0.920	1.00	0.00
ATOM	3514	HW1	SOL	477	46.390	19.920	0.300	1.00	0.00
ATOM	3515	HW2	SOL	477	45.160	19.650	1.140	1.00	0.00
ATOM	3516	OW	SOL	478	22.110	22.210	49.290	1.00	0.00
ATOM	3517	HW1	SOL	478	22.800	22.850	49.100	1.00	0.00
ATOM	3518	HW2	SOL	478	21.920	21.800	48.450	1.00	0.00
ATOM	3519	OW	SOL	479	16.300	55.150	16.190	1.00	0.00

ATOM	3520	HW1	SOL	479	17.000	55.430	16.770	1.00	0.00
ATOM	3521	HW2	SOL	479	16.330	55.780	15.460	1.00	0.00
ATOM	3522	OW	SOL	480	11.450	11.360	48.820	1.00	0.00
ATOM	3523	HW1	SOL	480	10.750	11.930	48.500	1.00	0.00
ATOM	3524	HW2	SOL	480	12.100	11.960	49.170	1.00	0.00
ATOM	3525	OW	SOL	481	3.620	26.830	31.930	1.00	0.00
ATOM	3526	HW1	SOL	481	4.110	27.370	31.320	1.00	0.00
ATOM	3527	HW2	SOL	481	2.750	27.240	31.980	1.00	0.00
ATOM	3528	OW	SOL	482	23.350	55.160	39.500	1.00	0.00
ATOM	3529	HW1	SOL	482	24.230	55.020	39.880	1.00	0.00
ATOM	3530	HW2	SOL	482	23.120	54.310	39.130	1.00	0.00
ATOM	3531	OW	SOL	483	18.850	17.790	43.110	1.00	0.00
ATOM	3532	HW1	SOL	483	18.630	17.710	42.180	1.00	0.00
ATOM	3533	HW2	SOL	483	18.380	17.060	43.520	1.00	0.00
ATOM	3534	OW	SOL	484	45.840	27.670	52.400	1.00	0.00
ATOM	3535	HW1	SOL	484	46.690	27.770	52.830	1.00	0.00
ATOM	3536	HW2	SOL	484	45.790	28.400	51.790	1.00	0.00
ATOM	3537	OW	SOL	485	26.980	54.230	1.110	1.00	0.00
ATOM	3538	HW1	SOL	485	27.320	54.630	0.310	1.00	0.00
ATOM	3539	HW2	SOL	485	27.460	54.650	1.820	1.00	0.00
ATOM	3540	OW	SOL	486	2.340	13.940	6.450	1.00	0.00
ATOM	3541	HW1	SOL	486	3.020	13.660	7.070	1.00	0.00
ATOM	3542	HW2	SOL	486	1.530	13.570	6.800	1.00	0.00
ATOM	3543	OW	SOL	487	46.110	22.680	31.230	1.00	0.00
ATOM	3544	HW1	SOL	487	46.710	23.410	31.050	1.00	0.00
ATOM	3545	HW2	SOL	487	46.640	21.900	31.160	1.00	0.00
ATOM	3546	OW	SOL	488	41.880	36.520	19.550	1.00	0.00
ATOM	3547	HW1	SOL	488	41.150	36.120	19.080	1.00	0.00
ATOM	3548	HW2	SOL	488	42.110	35.880	20.220	1.00	0.00
ATOM	3549	OW	SOL	489	51.730	24.850	27.610	1.00	0.00
ATOM	3550	HW1	SOL	489	50.940	25.390	27.560	1.00	0.00
ATOM	3551	HW2	SOL	489	52.440	25.480	27.730	1.00	0.00
ATOM	3552	OW	SOL	490	9.050	14.100	32.500	1.00	0.00
ATOM	3553	HW1	SOL	490	9.540	14.820	32.880	1.00	0.00
ATOM	3554	HW2	SOL	490	8.650	13.650	33.240	1.00	0.00
ATOM	3555	OW	SOL	491	29.330	41.340	54.100	1.00	0.00
ATOM	3556	HW1	SOL	491	29.750	42.140	53.780	1.00	0.00
ATOM	3557	HW2	SOL	491	29.370	40.730	53.360	1.00	0.00
ATOM	3558	OW	SOL	492	53.420	37.540	53.690	1.00	0.00
ATOM	3559	HW1	SOL	492	53.270	37.000	54.470	1.00	0.00
ATOM	3560	HW2	SOL	492	52.620	38.070	53.610	1.00	0.00
ATOM	3561	OW	SOL	493	4.400	21.830	41.160	1.00	0.00
ATOM	3562	HW1	SOL	493	4.130	21.240	41.870	1.00	0.00
ATOM	3563	HW2	SOL	493	3.610	21.990	40.660	1.00	0.00

ATOM	3564	OW	SOL	494	30.110	24.220	55.330	1.00	0.00
ATOM	3565	HW1	SOL	494	30.590	24.480	56.110	1.00	0.00
ATOM	3566	HW2	SOL	494	29.490	23.560	55.630	1.00	0.00
ATOM	3567	OW	SOL	495	42.420	29.610	25.170	1.00	0.00
ATOM	3568	HW1	SOL	495	43.320	29.820	24.940	1.00	0.00
ATOM	3569	HW2	SOL	495	42.340	29.860	26.090	1.00	0.00
ATOM	3570	OW	SOL	496	46.010	54.430	22.230	1.00	0.00
ATOM	3571	HW1	SOL	496	46.220	53.520	22.450	1.00	0.00
ATOM	3572	HW2	SOL	496	45.720	54.400	21.320	1.00	0.00
ATOM	3573	OW	SOL	497	18.810	14.410	51.570	1.00	0.00
ATOM	3574	HW1	SOL	497	19.500	14.950	51.950	1.00	0.00
ATOM	3575	HW2	SOL	497	19.270	13.680	51.170	1.00	0.00
ATOM	3576	OW	SOL	498	42.700	12.130	0.170	1.00	0.00
ATOM	3577	HW1	SOL	498	41.870	12.040	-0.300	1.00	0.00
ATOM	3578	HW2	SOL	498	43.300	12.490	-0.470	1.00	0.00
ATOM	3579	OW	SOL	499	2.630	26.680	3.360	1.00	0.00
ATOM	3580	HW1	SOL	499	1.770	26.860	3.000	1.00	0.00
ATOM	3581	HW2	SOL	499	2.480	26.560	4.300	1.00	0.00
ATOM	3582	OW	SOL	500	54.130	18.990	49.370	1.00	0.00
ATOM	3583	HW1	SOL	500	54.180	18.040	49.250	1.00	0.00
ATOM	3584	HW2	SOL	500	53.530	19.110	50.100	1.00	0.00
ATOM	3585	OW	SOL	501	46.750	47.330	8.970	1.00	0.00
ATOM	3586	HW1	SOL	501	46.890	46.750	9.720	1.00	0.00
ATOM	3587	HW2	SOL	501	47.060	46.820	8.220	1.00	0.00
ATOM	3588	OW	SOL	502	3.610	0.560	3.540	1.00	0.00
ATOM	3589	HW1	SOL	502	3.520	0.450	4.490	1.00	0.00
ATOM	3590	HW2	SOL	502	4.030	1.420	3.430	1.00	0.00
ATOM	3591	OW	SOL	503	50.750	22.090	54.910	1.00	0.00
ATOM	3592	HW1	SOL	503	50.870	22.520	54.070	1.00	0.00
ATOM	3593	HW2	SOL	503	50.610	22.800	55.530	1.00	0.00
ATOM	3594	OW	SOL	504	31.330	24.190	46.990	1.00	0.00
ATOM	3595	HW1	SOL	504	31.740	24.250	47.860	1.00	0.00
ATOM	3596	HW2	SOL	504	30.470	24.580	47.110	1.00	0.00
ATOM	3597	OW	SOL	505	1.360	42.210	19.920	1.00	0.00
ATOM	3598	HW1	SOL	505	1.910	42.620	20.590	1.00	0.00
ATOM	3599	HW2	SOL	505	0.490	42.160	20.320	1.00	0.00
ATOM	3600	OW	SOL	506	54.460	33.170	20.840	1.00	0.00
ATOM	3601	HW1	SOL	506	53.820	32.840	20.210	1.00	0.00
ATOM	3602	HW2	SOL	506	54.060	33.970	21.180	1.00	0.00
ATOM	3603	OW	SOL	507	27.830	27.350	32.220	1.00	0.00
ATOM	3604	HW1	SOL	507	28.650	27.700	32.580	1.00	0.00
ATOM	3605	HW2	SOL	507	27.950	26.400	32.220	1.00	0.00
ATOM	3606	OW	SOL	508	34.500	14.100	54.670	1.00	0.00
ATOM	3607	HW1	SOL	508	34.440	14.990	54.320	1.00	0.00

ATOM	3608	HW2	SOL	508	34.220	14.180	55.580	1.00	0.00
ATOM	3609	OW	SOL	509	38.140	27.100	21.670	1.00	0.00
ATOM	3610	HW1	SOL	509	37.230	26.950	21.410	1.00	0.00
ATOM	3611	HW2	SOL	509	38.240	26.610	22.480	1.00	0.00
ATOM	3612	OW	SOL	510	47.290	12.050	43.700	1.00	0.00
ATOM	3613	HW1	SOL	510	47.860	11.350	43.380	1.00	0.00
ATOM	3614	HW2	SOL	510	46.690	12.230	42.980	1.00	0.00
ATOM	3615	OW	SOL	511	50.600	39.080	13.870	1.00	0.00
ATOM	3616	HW1	SOL	511	50.370	38.280	14.350	1.00	0.00
ATOM	3617	HW2	SOL	511	51.540	39.180	14.020	1.00	0.00
ATOM	3618	OW	SOL	512	45.840	40.800	1.150	1.00	0.00
ATOM	3619	HW1	SOL	512	45.030	41.280	1.330	1.00	0.00
ATOM	3620	HW2	SOL	512	46.520	41.320	1.580	1.00	0.00
ATOM	3621	OW	SOL	513	50.260	14.000	33.360	1.00	0.00
ATOM	3622	HW1	SOL	513	50.630	13.500	32.630	1.00	0.00
ATOM	3623	HW2	SOL	513	51.010	14.460	33.740	1.00	0.00
ATOM	3624	OW	SOL	514	54.920	6.400	52.740	1.00	0.00
ATOM	3625	HW1	SOL	514	54.110	5.930	52.560	1.00	0.00
ATOM	3626	HW2	SOL	514	54.960	7.090	52.080	1.00	0.00
ATOM	3627	OW	SOL	515	25.080	4.620	36.910	1.00	0.00
ATOM	3628	HW1	SOL	515	24.460	5.120	37.440	1.00	0.00
ATOM	3629	HW2	SOL	515	24.690	4.600	36.040	1.00	0.00
ATOM	3630	OW	SOL	516	12.190	5.700	24.870	1.00	0.00
ATOM	3631	HW1	SOL	516	11.610	6.010	24.180	1.00	0.00
ATOM	3632	HW2	SOL	516	11.950	6.230	25.640	1.00	0.00
ATOM	3633	OW	SOL	517	33.260	31.220	9.320	1.00	0.00
ATOM	3634	HW1	SOL	517	32.710	30.820	8.650	1.00	0.00
ATOM	3635	HW2	SOL	517	33.490	32.080	8.970	1.00	0.00
ATOM	3636	OW	SOL	518	53.550	21.060	16.090	1.00	0.00
ATOM	3637	HW1	SOL	518	52.920	21.500	16.660	1.00	0.00
ATOM	3638	HW2	SOL	518	54.030	21.780	15.670	1.00	0.00
ATOM	3639	OW	SOL	519	43.950	35.580	17.870	1.00	0.00
ATOM	3640	HW1	SOL	519	43.820	35.770	16.940	1.00	0.00
ATOM	3641	HW2	SOL	519	43.110	35.830	18.270	1.00	0.00
ATOM	3642	OW	SOL	520	38.980	23.860	0.690	1.00	0.00
ATOM	3643	HW1	SOL	520	39.350	24.370	1.420	1.00	0.00
ATOM	3644	HW2	SOL	520	39.200	24.360	-0.090	1.00	0.00
ATOM	3645	OW	SOL	521	43.800	0.140	22.630	1.00	0.00
ATOM	3646	HW1	SOL	521	42.940	-0.250	22.460	1.00	0.00
ATOM	3647	HW2	SOL	521	44.410	-0.600	22.640	1.00	0.00
ATOM	3648	OW	SOL	522	23.500	3.070	32.770	1.00	0.00
ATOM	3649	HW1	SOL	522	23.170	2.220	33.040	1.00	0.00
ATOM	3650	HW2	SOL	522	22.840	3.400	32.150	1.00	0.00
ATOM	3651	OW	SOL	523	46.150	27.900	8.520	1.00	0.00

ATOM	3652	HW1	SOL	523	46.590	27.140	8.890	1.00	0.00
ATOM	3653	HW2	SOL	523	46.590	28.650	8.920	1.00	0.00
ATOM	3654	OW	SOL	524	52.840	34.030	8.530	1.00	0.00
ATOM	3655	HW1	SOL	524	51.970	34.340	8.770	1.00	0.00
ATOM	3656	HW2	SOL	524	52.900	33.160	8.920	1.00	0.00
ATOM	3657	OW	SOL	525	21.030	15.690	21.570	1.00	0.00
ATOM	3658	HW1	SOL	525	20.850	15.570	20.640	1.00	0.00
ATOM	3659	HW2	SOL	525	20.200	15.460	22.000	1.00	0.00
ATOM	3660	OW	SOL	526	5.300	32.830	27.550	1.00	0.00
ATOM	3661	HW1	SOL	526	5.370	32.030	27.030	1.00	0.00
ATOM	3662	HW2	SOL	526	5.200	32.510	28.450	1.00	0.00
ATOM	3663	OW	SOL	527	50.450	27.330	55.220	1.00	0.00
ATOM	3664	HW1	SOL	527	51.310	27.620	54.920	1.00	0.00
ATOM	3665	HW2	SOL	527	50.210	27.970	55.890	1.00	0.00
ATOM	3666	OW	SOL	528	47.780	23.060	1.440	1.00	0.00
ATOM	3667	HW1	SOL	528	46.940	22.610	1.580	1.00	0.00
ATOM	3668	HW2	SOL	528	47.530	23.960	1.260	1.00	0.00
ATOM	3669	OW	SOL	529	6.120	43.870	23.900	1.00	0.00
ATOM	3670	HW1	SOL	529	6.420	44.450	23.200	1.00	0.00
ATOM	3671	HW2	SOL	529	6.500	43.020	23.680	1.00	0.00
ATOM	3672	OW	SOL	530	14.940	3.840	16.320	1.00	0.00
ATOM	3673	HW1	SOL	530	14.240	4.330	16.760	1.00	0.00
ATOM	3674	HW2	SOL	530	15.230	3.200	16.980	1.00	0.00
ATOM	3675	OW	SOL	531	4.820	37.740	13.320	1.00	0.00
ATOM	3676	HW1	SOL	531	5.730	38.010	13.400	1.00	0.00
ATOM	3677	HW2	SOL	531	4.660	37.210	14.100	1.00	0.00
ATOM	3678	OW	SOL	532	20.440	12.100	54.820	1.00	0.00
ATOM	3679	HW1	SOL	532	19.920	12.080	54.020	1.00	0.00
ATOM	3680	HW2	SOL	532	21.060	11.380	54.720	1.00	0.00
ATOM	3681	OW	SOL	533	11.830	19.710	37.200	1.00	0.00
ATOM	3682	HW1	SOL	533	11.130	19.160	37.550	1.00	0.00
ATOM	3683	HW2	SOL	533	11.480	20.060	36.380	1.00	0.00
ATOM	3684	OW	SOL	534	17.560	5.070	23.930	1.00	0.00
ATOM	3685	HW1	SOL	534	16.600	5.020	23.870	1.00	0.00
ATOM	3686	HW2	SOL	534	17.860	4.680	23.110	1.00	0.00
ATOM	3687	OW	SOL	535	0.910	18.600	12.770	1.00	0.00
ATOM	3688	HW1	SOL	535	1.320	18.450	13.620	1.00	0.00
ATOM	3689	HW2	SOL	535	0.020	18.240	12.870	1.00	0.00
ATOM	3690	OW	SOL	536	8.620	50.360	26.620	1.00	0.00
ATOM	3691	HW1	SOL	536	8.580	50.940	27.370	1.00	0.00
ATOM	3692	HW2	SOL	536	7.730	50.380	26.250	1.00	0.00
ATOM	3693	OW	SOL	537	50.980	44.770	40.310	1.00	0.00
ATOM	3694	HW1	SOL	537	51.170	44.270	39.520	1.00	0.00
ATOM	3695	HW2	SOL	537	50.040	44.950	40.260	1.00	0.00

ATOM	3696	OW	SOL	538	1.420	24.520	9.570	1.00	0.00
ATOM	3697	HW1	SOL	538	1.890	25.350	9.600	1.00	0.00
ATOM	3698	HW2	SOL	538	1.860	23.960	10.210	1.00	0.00
ATOM	3699	OW	SOL	539	19.260	17.210	25.180	1.00	0.00
ATOM	3700	HW1	SOL	539	18.440	16.860	24.840	1.00	0.00
ATOM	3701	HW2	SOL	539	19.350	16.820	26.040	1.00	0.00
ATOM	3702	OW	SOL	540	8.210	25.230	47.170	1.00	0.00
ATOM	3703	HW1	SOL	540	7.890	25.780	46.450	1.00	0.00
ATOM	3704	HW2	SOL	540	8.770	25.810	47.680	1.00	0.00
ATOM	3705	OW	SOL	541	49.280	10.880	28.590	1.00	0.00
ATOM	3706	HW1	SOL	541	49.170	11.220	27.700	1.00	0.00
ATOM	3707	HW2	SOL	541	48.400	10.630	28.870	1.00	0.00
ATOM	3708	OW	SOL	542	31.250	25.340	32.470	1.00	0.00
ATOM	3709	HW1	SOL	542	32.170	25.390	32.220	1.00	0.00
ATOM	3710	HW2	SOL	542	31.250	24.900	33.320	1.00	0.00
ATOM	3711	OW	SOL	543	54.340	45.560	50.680	1.00	0.00
ATOM	3712	HW1	SOL	543	54.230	45.510	51.640	1.00	0.00
ATOM	3713	HW2	SOL	543	54.580	46.470	50.510	1.00	0.00
ATOM	3714	OW	SOL	544	40.170	5.290	7.600	1.00	0.00
ATOM	3715	HW1	SOL	544	39.270	5.590	7.740	1.00	0.00
ATOM	3716	HW2	SOL	544	40.710	6.050	7.820	1.00	0.00
ATOM	3717	OW	SOL	545	17.430	54.910	23.930	1.00	0.00
ATOM	3718	HW1	SOL	545	17.630	55.700	24.430	1.00	0.00
ATOM	3719	HW2	SOL	545	18.280	54.500	23.760	1.00	0.00
ATOM	3720	OW	SOL	546	48.090	38.380	23.960	1.00	0.00
ATOM	3721	HW1	SOL	546	48.180	39.150	24.530	1.00	0.00
ATOM	3722	HW2	SOL	546	47.690	37.720	24.520	1.00	0.00
ATOM	3723	OW	SOL	547	50.850	8.690	27.940	1.00	0.00
ATOM	3724	HW1	SOL	547	50.550	9.520	28.310	1.00	0.00
ATOM	3725	HW2	SOL	547	50.320	8.030	28.390	1.00	0.00
ATOM	3726	OW	SOL	548	1.960	46.210	37.190	1.00	0.00
ATOM	3727	HW1	SOL	548	1.080	46.100	36.820	1.00	0.00
ATOM	3728	HW2	SOL	548	2.410	46.750	36.540	1.00	0.00
ATOM	3729	OW	SOL	549	53.100	31.170	23.260	1.00	0.00
ATOM	3730	HW1	SOL	549	53.910	30.750	22.960	1.00	0.00
ATOM	3731	HW2	SOL	549	52.980	30.850	24.150	1.00	0.00
ATOM	3732	OW	SOL	550	7.200	3.230	17.600	1.00	0.00
ATOM	3733	HW1	SOL	550	7.960	2.770	17.240	1.00	0.00
ATOM	3734	HW2	SOL	550	7.450	4.160	17.570	1.00	0.00
ATOM	3735	OW	SOL	551	53.810	34.130	53.420	1.00	0.00
ATOM	3736	HW1	SOL	551	53.290	33.390	53.100	1.00	0.00
ATOM	3737	HW2	SOL	551	53.180	34.700	53.870	1.00	0.00
ATOM	3738	OW	SOL	552	4.060	9.180	4.480	1.00	0.00
ATOM	3739	HW1	SOL	552	4.510	8.340	4.490	1.00	0.00

ATOM	3740	HW2	SOL	552	3.130	8.950	4.370	1.00	0.00
ATOM	3741	OW	SOL	553	44.760	49.480	42.480	1.00	0.00
ATOM	3742	HW1	SOL	553	45.520	49.570	43.060	1.00	0.00
ATOM	3743	HW2	SOL	553	44.070	49.120	43.040	1.00	0.00
ATOM	3744	OW	SOL	554	52.240	9.720	32.050	1.00	0.00
ATOM	3745	HW1	SOL	554	52.410	10.480	31.480	1.00	0.00
ATOM	3746	HW2	SOL	554	53.090	9.300	32.140	1.00	0.00
ATOM	3747	OW	SOL	555	31.770	18.230	52.820	1.00	0.00
ATOM	3748	HW1	SOL	555	32.240	17.640	53.400	1.00	0.00
ATOM	3749	HW2	SOL	555	31.860	19.100	53.230	1.00	0.00
ATOM	3750	OW	SOL	556	29.370	31.040	1.460	1.00	0.00
ATOM	3751	HW1	SOL	556	29.810	30.260	1.790	1.00	0.00
ATOM	3752	HW2	SOL	556	30.090	31.590	1.140	1.00	0.00
ATOM	3753	OW	SOL	557	44.660	6.600	31.270	1.00	0.00
ATOM	3754	HW1	SOL	557	44.160	6.660	30.460	1.00	0.00
ATOM	3755	HW2	SOL	557	45.550	6.840	31.010	1.00	0.00
ATOM	3756	OW	SOL	558	46.630	34.290	52.620	1.00	0.00
ATOM	3757	HW1	SOL	558	47.010	33.420	52.710	1.00	0.00
ATOM	3758	HW2	SOL	558	47.210	34.850	53.140	1.00	0.00
ATOM	3759	OW	SOL	559	26.970	23.120	16.570	1.00	0.00
ATOM	3760	HW1	SOL	559	26.730	23.340	15.670	1.00	0.00
ATOM	3761	HW2	SOL	559	26.140	22.940	17.000	1.00	0.00
ATOM	3762	OW	SOL	560	54.840	6.480	11.840	1.00	0.00
ATOM	3763	HW1	SOL	560	54.540	6.500	12.750	1.00	0.00
ATOM	3764	HW2	SOL	560	55.090	7.380	11.650	1.00	0.00
ATOM	3765	OW	SOL	561	33.210	28.010	33.120	1.00	0.00
ATOM	3766	HW1	SOL	561	33.700	28.830	33.120	1.00	0.00
ATOM	3767	HW2	SOL	561	33.430	27.600	33.960	1.00	0.00
ATOM	3768	OW	SOL	562	0.510	12.710	17.610	1.00	0.00
ATOM	3769	HW1	SOL	562	-0.440	12.700	17.600	1.00	0.00
ATOM	3770	HW2	SOL	562	0.760	11.820	17.350	1.00	0.00
ATOM	3771	OW	SOL	563	50.360	23.290	52.540	1.00	0.00
ATOM	3772	HW1	SOL	563	50.160	22.620	51.890	1.00	0.00
ATOM	3773	HW2	SOL	563	49.880	24.070	52.250	1.00	0.00
ATOM	3774	OW	SOL	564	19.420	10.090	33.330	1.00	0.00
ATOM	3775	HW1	SOL	564	19.220	10.270	34.250	1.00	0.00
ATOM	3776	HW2	SOL	564	20.190	9.510	33.370	1.00	0.00
ATOM	3777	OW	SOL	565	35.060	11.050	42.100	1.00	0.00
ATOM	3778	HW1	SOL	565	35.740	10.470	41.760	1.00	0.00
ATOM	3779	HW2	SOL	565	34.360	10.990	41.440	1.00	0.00
ATOM	3780	OW	SOL	566	37.760	9.140	40.550	1.00	0.00
ATOM	3781	HW1	SOL	566	38.200	9.300	41.380	1.00	0.00
ATOM	3782	HW2	SOL	566	38.260	8.440	40.140	1.00	0.00
ATOM	3783	OW	SOL	567	48.200	44.150	25.870	1.00	0.00

ATOM	3784	HW1	SOL	567	47.690	44.780	25.360	1.00	0.00
ATOM	3785	HW2	SOL	567	47.540	43.560	26.240	1.00	0.00
ATOM	3786	OW	SOL	568	28.030	0.500	40.120	1.00	0.00
ATOM	3787	HW1	SOL	568	27.740	1.410	40.030	1.00	0.00
ATOM	3788	HW2	SOL	568	28.850	0.460	39.640	1.00	0.00
ATOM	3789	OW	SOL	569	33.410	30.550	55.160	1.00	0.00
ATOM	3790	HW1	SOL	569	32.530	30.770	54.870	1.00	0.00
ATOM	3791	HW2	SOL	569	33.900	30.380	54.360	1.00	0.00
ATOM	3792	OW	SOL	570	10.830	20.160	43.980	1.00	0.00
ATOM	3793	HW1	SOL	570	10.490	19.400	43.510	1.00	0.00
ATOM	3794	HW2	SOL	570	11.400	20.590	43.350	1.00	0.00
ATOM	3795	OW	SOL	571	7.160	32.950	44.990	1.00	0.00
ATOM	3796	HW1	SOL	571	7.770	32.450	45.540	1.00	0.00
ATOM	3797	HW2	SOL	571	6.310	32.830	45.420	1.00	0.00
ATOM	3798	OW	SOL	572	53.180	52.350	51.410	1.00	0.00
ATOM	3799	HW1	SOL	572	52.750	52.990	51.990	1.00	0.00
ATOM	3800	HW2	SOL	572	53.180	51.540	51.910	1.00	0.00
ATOM	3801	OW	SOL	573	41.160	45.020	13.730	1.00	0.00
ATOM	3802	HW1	SOL	573	41.810	44.490	14.190	1.00	0.00
ATOM	3803	HW2	SOL	573	41.250	45.900	14.110	1.00	0.00
ATOM	3804	OW	SOL	574	5.410	43.910	46.210	1.00	0.00
ATOM	3805	HW1	SOL	574	5.410	43.290	46.940	1.00	0.00
ATOM	3806	HW2	SOL	574	6.050	43.550	45.590	1.00	0.00
ATOM	3807	OW	SOL	575	19.620	5.040	51.990	1.00	0.00
ATOM	3808	HW1	SOL	575	18.740	5.060	51.620	1.00	0.00
ATOM	3809	HW2	SOL	575	19.920	4.140	51.860	1.00	0.00
ATOM	3810	OW	SOL	576	50.690	55.460	1.250	1.00	0.00
ATOM	3811	HW1	SOL	576	50.500	56.290	1.690	1.00	0.00
ATOM	3812	HW2	SOL	576	51.080	54.910	1.920	1.00	0.00
ATOM	3813	OW	SOL	577	48.200	44.970	39.630	1.00	0.00
ATOM	3814	HW1	SOL	577	47.770	44.870	40.480	1.00	0.00
ATOM	3815	HW2	SOL	577	47.850	44.260	39.100	1.00	0.00
ATOM	3816	OW	SOL	578	41.050	2.540	18.400	1.00	0.00
ATOM	3817	HW1	SOL	578	41.260	3.390	18.000	1.00	0.00
ATOM	3818	HW2	SOL	578	41.800	2.360	18.980	1.00	0.00
ATOM	3819	OW	SOL	579	8.090	39.450	25.100	1.00	0.00
ATOM	3820	HW1	SOL	579	8.260	38.670	24.570	1.00	0.00
ATOM	3821	HW2	SOL	579	8.170	40.180	24.490	1.00	0.00
ATOM	3822	OW	SOL	580	5.760	19.690	39.680	1.00	0.00
ATOM	3823	HW1	SOL	580	5.250	20.410	40.060	1.00	0.00
ATOM	3824	HW2	SOL	580	5.230	18.910	39.840	1.00	0.00
ATOM	3825	OW	SOL	581	1.170	12.120	1.660	1.00	0.00
ATOM	3826	HW1	SOL	581	0.730	11.290	1.850	1.00	0.00
ATOM	3827	HW2	SOL	581	1.990	12.080	2.150	1.00	0.00

ATOM	3828	OW	SOL	582	34.410	47.360	46.760	1.00	0.00
ATOM	3829	HW1	SOL	582	34.910	46.790	46.180	1.00	0.00
ATOM	3830	HW2	SOL	582	35.060	47.770	47.330	1.00	0.00
ATOM	3831	OW	SOL	583	44.570	16.100	55.240	1.00	0.00
ATOM	3832	HW1	SOL	583	44.550	16.930	55.710	1.00	0.00
ATOM	3833	HW2	SOL	583	43.670	15.970	54.940	1.00	0.00
ATOM	3834	OW	SOL	584	14.310	16.750	46.060	1.00	0.00
ATOM	3835	HW1	SOL	584	13.550	17.160	45.650	1.00	0.00
ATOM	3836	HW2	SOL	584	14.170	15.810	45.940	1.00	0.00
ATOM	3837	OW	SOL	585	29.920	30.070	52.610	1.00	0.00
ATOM	3838	HW1	SOL	585	29.710	29.970	51.680	1.00	0.00
ATOM	3839	HW2	SOL	585	29.070	30.250	53.020	1.00	0.00
ATOM	3840	OW	SOL	586	27.470	55.610	54.480	1.00	0.00
ATOM	3841	HW1	SOL	586	27.900	56.420	54.190	1.00	0.00
ATOM	3842	HW2	SOL	586	26.770	55.470	53.840	1.00	0.00
ATOM	3843	OW	SOL	587	40.720	36.680	42.840	1.00	0.00
ATOM	3844	HW1	SOL	587	41.580	36.280	42.960	1.00	0.00
ATOM	3845	HW2	SOL	587	40.550	36.620	41.900	1.00	0.00
ATOM	3846	OW	SOL	588	40.700	11.380	54.120	1.00	0.00
ATOM	3847	HW1	SOL	588	41.190	11.660	53.340	1.00	0.00
ATOM	3848	HW2	SOL	588	39.790	11.390	53.840	1.00	0.00
ATOM	3849	OW	SOL	589	36.170	30.980	29.020	1.00	0.00
ATOM	3850	HW1	SOL	589	37.050	30.780	28.710	1.00	0.00
ATOM	3851	HW2	SOL	589	36.230	31.880	29.330	1.00	0.00
ATOM	3852	OW	SOL	590	13.830	18.810	16.200	1.00	0.00
ATOM	3853	HW1	SOL	590	14.420	18.530	16.890	1.00	0.00
ATOM	3854	HW2	SOL	590	13.030	19.060	16.650	1.00	0.00
ATOM	3855	OW	SOL	591	7.490	5.670	14.560	1.00	0.00
ATOM	3856	HW1	SOL	591	7.260	4.780	14.320	1.00	0.00
ATOM	3857	HW2	SOL	591	7.640	5.630	15.510	1.00	0.00
ATOM	3858	OW	SOL	592	27.760	54.900	43.130	1.00	0.00
ATOM	3859	HW1	SOL	592	27.020	55.300	43.580	1.00	0.00
ATOM	3860	HW2	SOL	592	27.790	55.330	42.280	1.00	0.00
ATOM	3861	OW	SOL	593	4.640	3.920	16.780	1.00	0.00
ATOM	3862	HW1	SOL	593	5.490	3.470	16.810	1.00	0.00
ATOM	3863	HW2	SOL	593	3.990	3.220	16.830	1.00	0.00
ATOM	3864	OW	SOL	594	1.650	32.600	45.570	1.00	0.00
ATOM	3865	HW1	SOL	594	0.850	32.960	45.190	1.00	0.00
ATOM	3866	HW2	SOL	594	2.340	32.870	44.960	1.00	0.00
ATOM	3867	OW	SOL	595	41.720	21.500	39.200	1.00	0.00
ATOM	3868	HW1	SOL	595	42.460	20.910	39.130	1.00	0.00
ATOM	3869	HW2	SOL	595	41.070	21.020	39.710	1.00	0.00
ATOM	3870	OW	SOL	596	38.790	4.580	43.990	1.00	0.00
ATOM	3871	HW1	SOL	596	38.970	5.030	43.160	1.00	0.00

ATOM	3872	HW2	SOL	596	37.840	4.490	44.010	1.00	0.00
ATOM	3873	OW	SOL	597	51.090	3.740	36.590	1.00	0.00
ATOM	3874	HW1	SOL	597	50.420	3.930	35.940	1.00	0.00
ATOM	3875	HW2	SOL	597	50.630	3.730	37.420	1.00	0.00
ATOM	3876	OW	SOL	598	9.780	32.000	5.690	1.00	0.00
ATOM	3877	HW1	SOL	598	9.210	32.690	5.350	1.00	0.00
ATOM	3878	HW2	SOL	598	9.850	32.180	6.630	1.00	0.00
ATOM	3879	OW	SOL	599	3.470	40.400	3.700	1.00	0.00
ATOM	3880	HW1	SOL	599	3.510	40.240	2.760	1.00	0.00
ATOM	3881	HW2	SOL	599	4.380	40.340	3.990	1.00	0.00
ATOM	3882	OW	SOL	600	43.470	3.590	23.140	1.00	0.00
ATOM	3883	HW1	SOL	600	42.800	3.370	22.490	1.00	0.00
ATOM	3884	HW2	SOL	600	44.290	3.530	22.650	1.00	0.00
ATOM	3885	OW	SOL	601	55.570	55.580	45.530	1.00	0.00
ATOM	3886	HW1	SOL	601	55.440	54.720	45.940	1.00	0.00
ATOM	3887	HW2	SOL	601	54.690	55.830	45.240	1.00	0.00
ATOM	3888	OW	SOL	602	39.050	11.620	11.870	1.00	0.00
ATOM	3889	HW1	SOL	602	38.450	11.050	12.360	1.00	0.00
ATOM	3890	HW2	SOL	602	39.300	12.300	12.500	1.00	0.00
ATOM	3891	OW	SOL	603	2.070	16.350	20.250	1.00	0.00
ATOM	3892	HW1	SOL	603	1.240	16.100	20.670	1.00	0.00
ATOM	3893	HW2	SOL	603	2.010	15.970	19.370	1.00	0.00
ATOM	3894	OW	SOL	604	48.600	25.650	52.080	1.00	0.00
ATOM	3895	HW1	SOL	604	48.760	26.320	52.750	1.00	0.00
ATOM	3896	HW2	SOL	604	48.790	26.090	51.260	1.00	0.00
ATOM	3897	OW	SOL	605	24.190	23.170	39.220	1.00	0.00
ATOM	3898	HW1	SOL	605	23.710	23.010	40.030	1.00	0.00
ATOM	3899	HW2	SOL	605	23.760	23.930	38.830	1.00	0.00
ATOM	3900	OW	SOL	606	25.480	25.260	51.390	1.00	0.00
ATOM	3901	HW1	SOL	606	26.290	25.220	51.900	1.00	0.00
ATOM	3902	HW2	SOL	606	24.900	25.810	51.920	1.00	0.00
ATOM	3903	OW	SOL	607	12.900	6.310	12.360	1.00	0.00
ATOM	3904	HW1	SOL	607	13.170	5.550	12.880	1.00	0.00
ATOM	3905	HW2	SOL	607	13.580	6.380	11.680	1.00	0.00
ATOM	3906	OW	SOL	608	4.450	43.550	33.020	1.00	0.00
ATOM	3907	HW1	SOL	608	3.960	43.670	33.830	1.00	0.00
ATOM	3908	HW2	SOL	608	4.540	42.600	32.940	1.00	0.00
ATOM	3909	OW	SOL	609	38.820	7.310	38.760	1.00	0.00
ATOM	3910	HW1	SOL	609	38.090	7.370	38.140	1.00	0.00
ATOM	3911	HW2	SOL	609	39.450	6.730	38.340	1.00	0.00
ATOM	3912	OW	SOL	610	4.370	1.000	33.670	1.00	0.00
ATOM	3913	HW1	SOL	610	4.640	0.190	33.220	1.00	0.00
ATOM	3914	HW2	SOL	610	4.760	1.700	33.160	1.00	0.00
ATOM	3915	OW	SOL	611	15.410	20.230	22.480	1.00	0.00

ATOM	3916	HW1	SOL	611	15.850	20.430	23.300	1.00	0.00
ATOM	3917	HW2	SOL	611	16.100	19.900	21.910	1.00	0.00
ATOM	3918	OW	SOL	612	28.170	23.240	24.400	1.00	0.00
ATOM	3919	HW1	SOL	612	28.700	23.630	23.710	1.00	0.00
ATOM	3920	HW2	SOL	612	28.360	23.770	25.170	1.00	0.00
ATOM	3921	OW	SOL	613	54.510	38.600	6.380	1.00	0.00
ATOM	3922	HW1	SOL	613	54.610	39.050	5.540	1.00	0.00
ATOM	3923	HW2	SOL	613	53.620	38.240	6.350	1.00	0.00
ATOM	3924	OW	SOL	614	44.170	36.860	49.300	1.00	0.00
ATOM	3925	HW1	SOL	614	44.390	37.670	49.760	1.00	0.00
ATOM	3926	HW2	SOL	614	43.260	36.990	49.010	1.00	0.00
ATOM	3927	OW	SOL	615	17.100	20.700	41.090	1.00	0.00
ATOM	3928	HW1	SOL	615	16.510	20.010	40.800	1.00	0.00
ATOM	3929	HW2	SOL	615	17.550	20.980	40.290	1.00	0.00
ATOM	3930	OW	SOL	616	27.960	16.010	22.320	1.00	0.00
ATOM	3931	HW1	SOL	616	28.440	15.180	22.360	1.00	0.00
ATOM	3932	HW2	SOL	616	27.070	15.750	22.070	1.00	0.00
ATOM	3933	OW	SOL	617	7.620	14.530	7.870	1.00	0.00
ATOM	3934	HW1	SOL	617	8.460	14.170	8.140	1.00	0.00
ATOM	3935	HW2	SOL	617	7.000	13.810	7.990	1.00	0.00
ATOM	3936	OW	SOL	618	44.450	6.450	17.290	1.00	0.00
ATOM	3937	HW1	SOL	618	44.530	5.970	16.470	1.00	0.00
ATOM	3938	HW2	SOL	618	43.520	6.710	17.330	1.00	0.00
ATOM	3939	OW	SOL	619	53.500	50.770	5.970	1.00	0.00
ATOM	3940	HW1	SOL	619	54.000	51.570	6.150	1.00	0.00
ATOM	3941	HW2	SOL	619	52.870	51.030	5.290	1.00	0.00
ATOM	3942	OW	SOL	620	17.330	1.840	33.030	1.00	0.00
ATOM	3943	HW1	SOL	620	16.870	1.290	33.670	1.00	0.00
ATOM	3944	HW2	SOL	620	18.000	1.270	32.660	1.00	0.00
ATOM	3945	OW	SOL	621	47.340	10.650	38.980	1.00	0.00
ATOM	3946	HW1	SOL	621	46.820	10.380	38.220	1.00	0.00
ATOM	3947	HW2	SOL	621	46.970	10.170	39.710	1.00	0.00
ATOM	3948	OW	SOL	622	11.280	55.680	14.280	1.00	0.00
ATOM	3949	HW1	SOL	622	10.540	55.190	13.920	1.00	0.00
ATOM	3950	HW2	SOL	622	11.910	55.740	13.560	1.00	0.00
ATOM	3951	OW	SOL	623	10.930	13.320	28.730	1.00	0.00
ATOM	3952	HW1	SOL	623	11.520	13.830	28.170	1.00	0.00
ATOM	3953	HW2	SOL	623	11.270	12.430	28.670	1.00	0.00
ATOM	3954	OW	SOL	624	30.920	18.970	47.100	1.00	0.00
ATOM	3955	HW1	SOL	624	31.780	18.980	46.670	1.00	0.00
ATOM	3956	HW2	SOL	624	30.920	19.760	47.640	1.00	0.00
ATOM	3957	OW	SOL	625	2.170	43.130	53.540	1.00	0.00
ATOM	3958	HW1	SOL	625	2.920	43.460	54.030	1.00	0.00
ATOM	3959	HW2	SOL	625	1.490	42.980	54.190	1.00	0.00

ATOM	3960	OW	SOL	626	54.770	16.740	13.490	1.00	0.00
ATOM	3961	HW1	SOL	626	54.230	16.110	13.010	1.00	0.00
ATOM	3962	HW2	SOL	626	54.640	16.510	14.420	1.00	0.00
ATOM	3963	OW	SOL	627	42.640	41.960	24.350	1.00	0.00
ATOM	3964	HW1	SOL	627	42.470	41.700	23.450	1.00	0.00
ATOM	3965	HW2	SOL	627	43.210	42.720	24.290	1.00	0.00
ATOM	3966	OW	SOL	628	21.750	11.270	34.910	1.00	0.00
ATOM	3967	HW1	SOL	628	20.820	11.270	34.650	1.00	0.00
ATOM	3968	HW2	SOL	628	22.230	11.370	34.090	1.00	0.00
ATOM	3969	OW	SOL	629	35.430	5.470	9.160	1.00	0.00
ATOM	3970	HW1	SOL	629	35.220	6.280	8.700	1.00	0.00
ATOM	3971	HW2	SOL	629	34.640	5.260	9.660	1.00	0.00
ATOM	3972	OW	SOL	630	6.450	48.150	51.870	1.00	0.00
ATOM	3973	HW1	SOL	630	7.360	48.450	51.900	1.00	0.00
ATOM	3974	HW2	SOL	630	6.160	48.350	50.980	1.00	0.00
ATOM	3975	OW	SOL	631	27.640	5.970	37.320	1.00	0.00
ATOM	3976	HW1	SOL	631	27.870	5.850	38.240	1.00	0.00
ATOM	3977	HW2	SOL	631	26.970	5.300	37.160	1.00	0.00
ATOM	3978	OW	SOL	632	28.530	39.310	2.780	1.00	0.00
ATOM	3979	HW1	SOL	632	27.580	39.180	2.840	1.00	0.00
ATOM	3980	HW2	SOL	632	28.640	40.260	2.710	1.00	0.00
ATOM	3981	OW	SOL	633	52.740	24.910	18.130	1.00	0.00
ATOM	3982	HW1	SOL	633	53.450	25.330	17.650	1.00	0.00
ATOM	3983	HW2	SOL	633	53.130	24.140	18.530	1.00	0.00
ATOM	3984	OW	SOL	634	35.620	16.920	13.880	1.00	0.00
ATOM	3985	HW1	SOL	634	34.720	17.220	13.720	1.00	0.00
ATOM	3986	HW2	SOL	634	35.910	17.430	14.630	1.00	0.00
ATOM	3987	OW	SOL	635	44.750	4.890	40.030	1.00	0.00
ATOM	3988	HW1	SOL	635	43.870	4.540	40.200	1.00	0.00
ATOM	3989	HW2	SOL	635	44.670	5.320	39.180	1.00	0.00
ATOM	3990	OW	SOL	636	21.210	17.100	15.670	1.00	0.00
ATOM	3991	HW1	SOL	636	20.570	17.750	15.390	1.00	0.00
ATOM	3992	HW2	SOL	636	20.760	16.260	15.610	1.00	0.00
ATOM	3993	OW	SOL	637	3.930	52.020	27.090	1.00	0.00
ATOM	3994	HW1	SOL	637	3.090	51.710	27.430	1.00	0.00
ATOM	3995	HW2	SOL	637	3.740	52.890	26.750	1.00	0.00
ATOM	3996	OW	SOL	638	36.560	7.810	37.250	1.00	0.00
ATOM	3997	HW1	SOL	638	35.940	8.340	36.750	1.00	0.00
ATOM	3998	HW2	SOL	638	36.200	7.780	38.140	1.00	0.00
ATOM	3999	OW	SOL	639	8.230	44.180	49.340	1.00	0.00
ATOM	4000	HW1	SOL	639	7.930	43.370	48.920	1.00	0.00
ATOM	4001	HW2	SOL	639	7.500	44.790	49.220	1.00	0.00
ATOM	4002	OW	SOL	640	25.700	49.390	51.470	1.00	0.00
ATOM	4003	HW1	SOL	640	25.690	49.880	52.290	1.00	0.00

ATOM	4004	HW2	SOL	640	26.540	49.610	51.070	1.00	0.00
ATOM	4005	OW	SOL	641	24.320	27.400	45.180	1.00	0.00
ATOM	4006	HW1	SOL	641	24.560	27.180	46.080	1.00	0.00
ATOM	4007	HW2	SOL	641	23.880	26.620	44.850	1.00	0.00
ATOM	4008	OW	SOL	642	11.110	52.200	12.030	1.00	0.00
ATOM	4009	HW1	SOL	642	10.970	51.350	11.620	1.00	0.00
ATOM	4010	HW2	SOL	642	12.040	52.390	11.860	1.00	0.00
ATOM	4011	OW	SOL	643	43.550	9.690	19.190	1.00	0.00
ATOM	4012	HW1	SOL	643	42.860	9.170	18.780	1.00	0.00
ATOM	4013	HW2	SOL	643	43.460	10.560	18.780	1.00	0.00
ATOM	4014	OW	SOL	644	37.860	3.270	53.250	1.00	0.00
ATOM	4015	HW1	SOL	644	38.320	3.680	53.980	1.00	0.00
ATOM	4016	HW2	SOL	644	38.210	2.380	53.210	1.00	0.00
ATOM	4017	OW	SOL	645	5.500	36.140	22.890	1.00	0.00
ATOM	4018	HW1	SOL	645	4.920	36.230	22.140	1.00	0.00
ATOM	4019	HW2	SOL	645	5.520	35.200	23.070	1.00	0.00
ATOM	4020	OW	SOL	646	5.400	30.300	45.580	1.00	0.00
ATOM	4021	HW1	SOL	646	6.280	29.920	45.550	1.00	0.00
ATOM	4022	HW2	SOL	646	5.290	30.580	46.480	1.00	0.00
ATOM	4023	OW	SOL	647	5.740	29.620	5.500	1.00	0.00
ATOM	4024	HW1	SOL	647	6.700	29.570	5.490	1.00	0.00
ATOM	4025	HW2	SOL	647	5.540	30.340	4.900	1.00	0.00
ATOM	4026	OW	SOL	648	10.670	2.630	42.710	1.00	0.00
ATOM	4027	HW1	SOL	648	10.820	3.400	42.160	1.00	0.00
ATOM	4028	HW2	SOL	648	9.860	2.250	42.390	1.00	0.00
ATOM	4029	OW	SOL	649	54.770	44.840	25.580	1.00	0.00
ATOM	4030	HW1	SOL	649	55.270	44.270	26.160	1.00	0.00
ATOM	4031	HW2	SOL	649	55.270	45.660	25.570	1.00	0.00
ATOM	4032	OW	SOL	650	36.750	21.000	49.940	1.00	0.00
ATOM	4033	HW1	SOL	650	37.660	20.930	50.220	1.00	0.00
ATOM	4034	HW2	SOL	650	36.800	21.400	49.070	1.00	0.00
ATOM	4035	OW	SOL	651	25.260	10.890	36.690	1.00	0.00
ATOM	4036	HW1	SOL	651	25.610	10.070	36.340	1.00	0.00
ATOM	4037	HW2	SOL	651	24.810	11.300	35.950	1.00	0.00
ATOM	4038	OW	SOL	652	8.080	55.010	14.890	1.00	0.00
ATOM	4039	HW1	SOL	652	7.120	55.000	14.910	1.00	0.00
ATOM	4040	HW2	SOL	652	8.300	54.570	14.070	1.00	0.00
ATOM	4041	OW	SOL	653	34.960	45.360	43.780	1.00	0.00
ATOM	4042	HW1	SOL	653	35.440	44.570	44.040	1.00	0.00
ATOM	4043	HW2	SOL	653	35.510	45.760	43.100	1.00	0.00
ATOM	4044	OW	SOL	654	15.620	52.770	10.540	1.00	0.00
ATOM	4045	HW1	SOL	654	16.550	52.850	10.310	1.00	0.00
ATOM	4046	HW2	SOL	654	15.170	52.790	9.710	1.00	0.00
ATOM	4047	OW	SOL	655	37.180	1.380	48.650	1.00	0.00

ATOM	4048	HW1	SOL	655	36.300	1.240	48.300	1.00	0.00
ATOM	4049	HW2	SOL	655	37.140	2.230	49.080	1.00	0.00
ATOM	4050	OW	SOL	656	13.310	34.710	15.700	1.00	0.00
ATOM	4051	HW1	SOL	656	12.680	34.450	15.030	1.00	0.00
ATOM	4052	HW2	SOL	656	12.780	35.130	16.380	1.00	0.00
ATOM	4053	OW	SOL	657	54.580	38.320	10.850	1.00	0.00
ATOM	4054	HW1	SOL	657	54.870	37.920	11.660	1.00	0.00
ATOM	4055	HW2	SOL	657	54.180	39.150	11.110	1.00	0.00
ATOM	4056	OW	SOL	658	45.810	23.720	14.210	1.00	0.00
ATOM	4057	HW1	SOL	658	46.010	22.940	13.690	1.00	0.00
ATOM	4058	HW2	SOL	658	44.870	23.670	14.380	1.00	0.00
ATOM	4059	OW	SOL	659	32.020	32.390	17.740	1.00	0.00
ATOM	4060	HW1	SOL	659	32.930	32.360	17.450	1.00	0.00
ATOM	4061	HW2	SOL	659	31.520	32.450	16.920	1.00	0.00
ATOM	4062	OW	SOL	660	36.600	11.500	28.820	1.00	0.00
ATOM	4063	HW1	SOL	660	36.490	10.710	29.360	1.00	0.00
ATOM	4064	HW2	SOL	660	37.000	12.140	29.400	1.00	0.00
ATOM	4065	OW	SOL	661	41.850	0.370	2.940	1.00	0.00
ATOM	4066	HW1	SOL	661	42.660	0.070	3.350	1.00	0.00
ATOM	4067	HW2	SOL	661	42.120	0.740	2.100	1.00	0.00
ATOM	4068	OW	SOL	662	45.450	21.080	12.830	1.00	0.00
ATOM	4069	HW1	SOL	662	46.270	20.730	12.490	1.00	0.00
ATOM	4070	HW2	SOL	662	44.880	21.160	12.060	1.00	0.00
ATOM	4071	OW	SOL	663	3.740	36.120	7.880	1.00	0.00
ATOM	4072	HW1	SOL	663	4.660	35.840	7.830	1.00	0.00
ATOM	4073	HW2	SOL	663	3.780	36.990	8.290	1.00	0.00
ATOM	4074	OW	SOL	664	19.400	13.230	7.400	1.00	0.00
ATOM	4075	HW1	SOL	664	19.670	12.760	6.610	1.00	0.00
ATOM	4076	HW2	SOL	664	19.340	14.150	7.130	1.00	0.00
ATOM	4077	OW	SOL	665	43.720	30.590	3.760	1.00	0.00
ATOM	4078	HW1	SOL	665	43.150	31.070	3.160	1.00	0.00
ATOM	4079	HW2	SOL	665	43.590	29.670	3.530	1.00	0.00
ATOM	4080	OW	SOL	666	44.030	2.210	25.510	1.00	0.00
ATOM	4081	HW1	SOL	666	43.780	2.650	24.700	1.00	0.00
ATOM	4082	HW2	SOL	666	43.520	1.400	25.510	1.00	0.00
ATOM	4083	OW	SOL	667	16.250	34.410	0.910	1.00	0.00
ATOM	4084	HW1	SOL	667	15.310	34.370	0.760	1.00	0.00
ATOM	4085	HW2	SOL	667	16.540	33.500	0.850	1.00	0.00
ATOM	4086	OW	SOL	668	32.620	24.470	50.500	1.00	0.00
ATOM	4087	HW1	SOL	668	31.700	24.450	50.750	1.00	0.00
ATOM	4088	HW2	SOL	668	33.050	24.990	51.180	1.00	0.00
ATOM	4089	OW	SOL	669	34.670	46.850	53.430	1.00	0.00
ATOM	4090	HW1	SOL	669	33.930	46.390	53.060	1.00	0.00
ATOM	4091	HW2	SOL	669	35.280	46.960	52.700	1.00	0.00

ATOM	4092	OW	SOL	670	26.040	24.560	23.280	1.00	0.00
ATOM	4093	HW1	SOL	670	26.550	24.010	23.870	1.00	0.00
ATOM	4094	HW2	SOL	670	26.160	25.450	23.600	1.00	0.00
ATOM	4095	OW	SOL	671	14.870	53.620	42.460	1.00	0.00
ATOM	4096	HW1	SOL	671	15.720	53.470	42.860	1.00	0.00
ATOM	4097	HW2	SOL	671	15.050	54.210	41.730	1.00	0.00
ATOM	4098	OW	SOL	672	1.190	37.790	41.620	1.00	0.00
ATOM	4099	HW1	SOL	672	0.670	38.520	41.290	1.00	0.00
ATOM	4100	HW2	SOL	672	1.580	38.110	42.430	1.00	0.00
ATOM	4101	OW	SOL	673	26.800	30.910	2.280	1.00	0.00
ATOM	4102	HW1	SOL	673	27.680	30.700	1.960	1.00	0.00
ATOM	4103	HW2	SOL	673	26.680	31.830	2.080	1.00	0.00
ATOM	4104	OW	SOL	674	19.620	20.990	42.000	1.00	0.00
ATOM	4105	HW1	SOL	674	18.720	20.770	41.750	1.00	0.00
ATOM	4106	HW2	SOL	674	20.080	21.060	41.170	1.00	0.00
ATOM	4107	OW	SOL	675	27.360	21.190	28.390	1.00	0.00
ATOM	4108	HW1	SOL	675	27.340	20.240	28.430	1.00	0.00
ATOM	4109	HW2	SOL	675	26.450	21.450	28.290	1.00	0.00
ATOM	4110	OW	SOL	676	53.630	19.610	29.840	1.00	0.00
ATOM	4111	HW1	SOL	676	54.290	19.220	29.260	1.00	0.00
ATOM	4112	HW2	SOL	676	53.120	20.190	29.280	1.00	0.00
ATOM	4113	OW	SOL	677	20.350	21.970	37.780	1.00	0.00
ATOM	4114	HW1	SOL	677	21.010	22.110	37.100	1.00	0.00
ATOM	4115	HW2	SOL	677	20.860	21.670	38.540	1.00	0.00
ATOM	4116	OW	SOL	678	54.390	28.990	15.350	1.00	0.00
ATOM	4117	HW1	SOL	678	53.720	29.340	14.750	1.00	0.00
ATOM	4118	HW2	SOL	678	55.110	29.620	15.300	1.00	0.00
ATOM	4119	OW	SOL	679	54.770	1.370	12.460	1.00	0.00
ATOM	4120	HW1	SOL	679	53.810	1.330	12.470	1.00	0.00
ATOM	4121	HW2	SOL	679	54.970	2.270	12.200	1.00	0.00
ATOM	4122	OW	SOL	680	14.420	37.290	13.670	1.00	0.00
ATOM	4123	HW1	SOL	680	13.980	37.400	14.510	1.00	0.00
ATOM	4124	HW2	SOL	680	14.950	36.490	13.780	1.00	0.00
ATOM	4125	OW	SOL	681	22.380	27.040	21.720	1.00	0.00
ATOM	4126	HW1	SOL	681	22.250	26.870	22.660	1.00	0.00
ATOM	4127	HW2	SOL	681	23.330	27.010	21.600	1.00	0.00
ATOM	4128	OW	SOL	682	46.830	55.460	14.920	1.00	0.00
ATOM	4129	HW1	SOL	682	47.650	55.020	15.140	1.00	0.00
ATOM	4130	HW2	SOL	682	46.170	54.770	14.950	1.00	0.00
ATOM	4131	OW	SOL	683	52.920	11.450	30.120	1.00	0.00
ATOM	4132	HW1	SOL	683	52.880	12.290	29.650	1.00	0.00
ATOM	4133	HW2	SOL	683	53.680	11.010	29.750	1.00	0.00
ATOM	4134	OW	SOL	684	16.420	20.190	10.170	1.00	0.00
ATOM	4135	HW1	SOL	684	16.170	20.820	10.850	1.00	0.00

ATOM	4136	HW2	SOL	684	16.550	19.370	10.640	1.00	0.00
ATOM	4137	OW	SOL	685	39.090	40.320	52.210	1.00	0.00
ATOM	4138	HW1	SOL	685	39.620	39.860	51.560	1.00	0.00
ATOM	4139	HW2	SOL	685	39.560	41.140	52.360	1.00	0.00
ATOM	4140	OW	SOL	686	50.370	8.450	33.390	1.00	0.00
ATOM	4141	HW1	SOL	686	51.050	8.820	32.830	1.00	0.00
ATOM	4142	HW2	SOL	686	50.340	9.030	34.140	1.00	0.00
ATOM	4143	OW	SOL	687	15.680	3.660	52.240	1.00	0.00
ATOM	4144	HW1	SOL	687	14.890	3.900	51.760	1.00	0.00
ATOM	4145	HW2	SOL	687	15.800	2.730	52.040	1.00	0.00
ATOM	4146	OW	SOL	688	26.040	3.710	16.290	1.00	0.00
ATOM	4147	HW1	SOL	688	26.680	3.430	15.640	1.00	0.00
ATOM	4148	HW2	SOL	688	26.120	3.080	17.010	1.00	0.00
ATOM	4149	OW	SOL	689	8.980	7.710	24.510	1.00	0.00
ATOM	4150	HW1	SOL	689	9.210	8.620	24.320	1.00	0.00
ATOM	4151	HW2	SOL	689	8.930	7.680	25.470	1.00	0.00
ATOM	4152	OW	SOL	690	5.400	16.300	8.520	1.00	0.00
ATOM	4153	HW1	SOL	690	5.450	17.020	7.890	1.00	0.00
ATOM	4154	HW2	SOL	690	6.270	15.890	8.480	1.00	0.00
ATOM	4155	OW	SOL	691	29.540	14.450	16.100	1.00	0.00
ATOM	4156	HW1	SOL	691	30.230	14.170	15.510	1.00	0.00
ATOM	4157	HW2	SOL	691	28.820	13.850	15.930	1.00	0.00
ATOM	4158	OW	SOL	692	22.060	48.430	54.070	1.00	0.00
ATOM	4159	HW1	SOL	692	22.010	47.580	54.510	1.00	0.00
ATOM	4160	HW2	SOL	692	21.180	48.810	54.190	1.00	0.00
ATOM	4161	OW	SOL	693	13.170	1.600	7.380	1.00	0.00
ATOM	4162	HW1	SOL	693	12.620	1.940	6.680	1.00	0.00
ATOM	4163	HW2	SOL	693	13.070	2.240	8.090	1.00	0.00
ATOM	4164	OW	SOL	694	25.300	6.220	51.670	1.00	0.00
ATOM	4165	HW1	SOL	694	25.560	5.980	52.550	1.00	0.00
ATOM	4166	HW2	SOL	694	25.250	7.170	51.680	1.00	0.00
ATOM	4167	OW	SOL	695	53.650	4.170	1.690	1.00	0.00
ATOM	4168	HW1	SOL	695	54.230	4.050	0.930	1.00	0.00
ATOM	4169	HW2	SOL	695	53.100	4.920	1.450	1.00	0.00
ATOM	4170	OW	SOL	696	33.740	54.140	0.490	1.00	0.00
ATOM	4171	HW1	SOL	696	33.280	54.340	1.310	1.00	0.00
ATOM	4172	HW2	SOL	696	34.090	53.270	0.620	1.00	0.00
ATOM	4173	OW	SOL	697	53.100	32.690	11.100	1.00	0.00
ATOM	4174	HW1	SOL	697	53.050	32.640	12.060	1.00	0.00
ATOM	4175	HW2	SOL	697	52.690	31.880	10.800	1.00	0.00
ATOM	4176	OW	SOL	698	47.960	53.640	4.960	1.00	0.00
ATOM	4177	HW1	SOL	698	48.790	53.820	5.400	1.00	0.00
ATOM	4178	HW2	SOL	698	47.320	54.140	5.460	1.00	0.00
ATOM	4179	OW	SOL	699	3.430	48.620	47.770	1.00	0.00

ATOM	4180	HW1	SOL	699	2.750	48.150	47.290	1.00	0.00
ATOM	4181	HW2	SOL	699	3.890	49.130	47.110	1.00	0.00
ATOM	4182	OW	SOL	700	23.390	37.040	3.360	1.00	0.00
ATOM	4183	HW1	SOL	700	22.490	37.300	3.150	1.00	0.00
ATOM	4184	HW2	SOL	700	23.400	36.970	4.310	1.00	0.00
ATOM	4185	OW	SOL	701	28.860	23.110	37.310	1.00	0.00
ATOM	4186	HW1	SOL	701	28.270	23.690	37.790	1.00	0.00
ATOM	4187	HW2	SOL	701	29.050	23.580	36.490	1.00	0.00
ATOM	4188	OW	SOL	702	2.320	16.460	5.160	1.00	0.00
ATOM	4189	HW1	SOL	702	1.390	16.580	4.950	1.00	0.00
ATOM	4190	HW2	SOL	702	2.360	15.590	5.560	1.00	0.00
ATOM	4191	OW	SOL	703	17.580	41.040	51.890	1.00	0.00
ATOM	4192	HW1	SOL	703	16.890	41.670	51.680	1.00	0.00
ATOM	4193	HW2	SOL	703	17.880	41.300	52.770	1.00	0.00
ATOM	4194	OW	SOL	704	3.370	24.760	26.900	1.00	0.00
ATOM	4195	HW1	SOL	704	4.260	24.690	27.250	1.00	0.00
ATOM	4196	HW2	SOL	704	3.490	25.000	25.980	1.00	0.00
ATOM	4197	OW	SOL	705	15.110	30.990	3.050	1.00	0.00
ATOM	4198	HW1	SOL	705	14.550	31.640	3.490	1.00	0.00
ATOM	4199	HW2	SOL	705	15.810	30.830	3.680	1.00	0.00
ATOM	4200	OW	SOL	706	17.100	46.110	16.610	1.00	0.00
ATOM	4201	HW1	SOL	706	17.260	45.710	15.750	1.00	0.00
ATOM	4202	HW2	SOL	706	16.900	47.020	16.410	1.00	0.00
ATOM	4203	OW	SOL	707	50.130	24.560	17.570	1.00	0.00
ATOM	4204	HW1	SOL	707	49.600	24.300	18.320	1.00	0.00
ATOM	4205	HW2	SOL	707	51.030	24.470	17.870	1.00	0.00
ATOM	4206	OW	SOL	708	29.420	10.400	27.370	1.00	0.00
ATOM	4207	HW1	SOL	708	30.380	10.420	27.370	1.00	0.00
ATOM	4208	HW2	SOL	708	29.180	10.510	28.290	1.00	0.00
ATOM	4209	OW	SOL	709	10.060	22.550	28.110	1.00	0.00
ATOM	4210	HW1	SOL	709	9.590	21.810	28.490	1.00	0.00
ATOM	4211	HW2	SOL	709	10.080	23.210	28.800	1.00	0.00
ATOM	4212	OW	SOL	710	24.720	20.560	32.370	1.00	0.00
ATOM	4213	HW1	SOL	710	23.780	20.620	32.560	1.00	0.00
ATOM	4214	HW2	SOL	710	24.850	19.660	32.080	1.00	0.00
ATOM	4215	OW	SOL	711	5.770	5.100	30.470	1.00	0.00
ATOM	4216	HW1	SOL	711	5.980	4.970	29.550	1.00	0.00
ATOM	4217	HW2	SOL	711	6.620	5.270	30.890	1.00	0.00
ATOM	4218	OW	SOL	712	46.620	55.520	6.220	1.00	0.00
ATOM	4219	HW1	SOL	712	46.040	55.430	6.970	1.00	0.00
ATOM	4220	HW2	SOL	712	46.930	56.430	6.260	1.00	0.00
ATOM	4221	OW	SOL	713	19.380	27.980	50.440	1.00	0.00
ATOM	4222	HW1	SOL	713	19.800	28.840	50.380	1.00	0.00
ATOM	4223	HW2	SOL	713	20.020	27.370	50.090	1.00	0.00

ATOM	4224	OW	SOL	714	10.020	51.000	54.870	1.00	0.00
ATOM	4225	HW1	SOL	714	9.880	51.480	55.680	1.00	0.00
ATOM	4226	HW2	SOL	714	10.230	50.110	55.150	1.00	0.00
ATOM	4227	OW	SOL	715	2.630	44.730	26.790	1.00	0.00
ATOM	4228	HW1	SOL	715	2.690	44.700	25.840	1.00	0.00
ATOM	4229	HW2	SOL	715	1.740	44.430	26.990	1.00	0.00
ATOM	4230	OW	SOL	716	5.120	33.860	5.890	1.00	0.00
ATOM	4231	HW1	SOL	716	4.770	34.730	5.690	1.00	0.00
ATOM	4232	HW2	SOL	716	4.360	33.330	6.120	1.00	0.00
ATOM	4233	OW	SOL	717	20.550	43.910	51.470	1.00	0.00
ATOM	4234	HW1	SOL	717	20.350	43.460	50.650	1.00	0.00
ATOM	4235	HW2	SOL	717	19.970	43.500	52.110	1.00	0.00
ATOM	4236	OW	SOL	718	21.900	32.240	0.980	1.00	0.00
ATOM	4237	HW1	SOL	718	22.720	32.660	0.710	1.00	0.00
ATOM	4238	HW2	SOL	718	22.080	31.300	0.920	1.00	0.00
ATOM	4239	OW	SOL	719	11.330	11.360	2.390	1.00	0.00
ATOM	4240	HW1	SOL	719	11.100	10.920	3.200	1.00	0.00
ATOM	4241	HW2	SOL	719	12.160	11.800	2.580	1.00	0.00
ATOM	4242	OW	SOL	720	14.510	47.390	28.560	1.00	0.00
ATOM	4243	HW1	SOL	720	14.260	47.860	29.350	1.00	0.00
ATOM	4244	HW2	SOL	720	14.040	46.560	28.610	1.00	0.00
ATOM	4245	OW	SOL	721	51.720	18.030	8.660	1.00	0.00
ATOM	4246	HW1	SOL	721	51.630	18.830	9.180	1.00	0.00
ATOM	4247	HW2	SOL	721	52.040	18.320	7.810	1.00	0.00
ATOM	4248	OW	SOL	722	14.810	36.300	4.410	1.00	0.00
ATOM	4249	HW1	SOL	722	14.340	36.690	5.150	1.00	0.00
ATOM	4250	HW2	SOL	722	14.540	35.380	4.420	1.00	0.00
ATOM	4251	OW	SOL	723	53.520	16.760	43.490	1.00	0.00
ATOM	4252	HW1	SOL	723	53.430	15.950	43.990	1.00	0.00
ATOM	4253	HW2	SOL	723	54.230	17.230	43.920	1.00	0.00
ATOM	4254	OW	SOL	724	49.010	45.750	31.580	1.00	0.00
ATOM	4255	HW1	SOL	724	48.320	45.970	30.950	1.00	0.00
ATOM	4256	HW2	SOL	724	49.810	46.090	31.180	1.00	0.00
ATOM	4257	OW	SOL	725	1.890	35.860	15.900	1.00	0.00
ATOM	4258	HW1	SOL	725	1.460	35.830	15.050	1.00	0.00
ATOM	4259	HW2	SOL	725	1.320	36.410	16.440	1.00	0.00
ATOM	4260	OW	SOL	726	33.510	21.180	9.040	1.00	0.00
ATOM	4261	HW1	SOL	726	33.870	21.670	8.310	1.00	0.00
ATOM	4262	HW2	SOL	726	34.270	20.770	9.460	1.00	0.00
ATOM	4263	OW	SOL	727	44.910	9.420	10.830	1.00	0.00
ATOM	4264	HW1	SOL	727	44.660	8.590	10.430	1.00	0.00
ATOM	4265	HW2	SOL	727	45.840	9.520	10.630	1.00	0.00
ATOM	4266	OW	SOL	728	34.240	21.620	23.900	1.00	0.00
ATOM	4267	HW1	SOL	728	33.310	21.860	23.850	1.00	0.00

ATOM	4268	HW2	SOL	728	34.590	22.180	24.590	1.00	0.00
ATOM	4269	OW	SOL	729	34.930	0.870	42.420	1.00	0.00
ATOM	4270	HW1	SOL	729	34.640	0.120	41.900	1.00	0.00
ATOM	4271	HW2	SOL	729	34.980	0.530	43.310	1.00	0.00
ATOM	4272	OW	SOL	730	25.220	20.370	15.150	1.00	0.00
ATOM	4273	HW1	SOL	730	24.430	19.860	15.310	1.00	0.00
ATOM	4274	HW2	SOL	730	25.200	21.050	15.830	1.00	0.00
ATOM	4275	OW	SOL	731	53.830	0.340	10.200	1.00	0.00
ATOM	4276	HW1	SOL	731	54.350	0.690	10.920	1.00	0.00
ATOM	4277	HW2	SOL	731	52.990	0.810	10.270	1.00	0.00
ATOM	4278	OW	SOL	732	18.190	24.240	28.030	1.00	0.00
ATOM	4279	HW1	SOL	732	19.090	23.990	28.220	1.00	0.00
ATOM	4280	HW2	SOL	732	17.780	23.440	27.720	1.00	0.00
ATOM	4281	OW	SOL	733	11.410	37.430	50.570	1.00	0.00
ATOM	4282	HW1	SOL	733	10.810	37.990	50.080	1.00	0.00
ATOM	4283	HW2	SOL	733	11.620	36.720	49.970	1.00	0.00
ATOM	4284	OW	SOL	734	54.240	35.580	21.710	1.00	0.00
ATOM	4285	HW1	SOL	734	53.640	36.300	21.510	1.00	0.00
ATOM	4286	HW2	SOL	734	55.110	35.950	21.560	1.00	0.00
ATOM	4287	OW	SOL	735	40.200	28.980	30.560	1.00	0.00
ATOM	4288	HW1	SOL	735	40.350	29.590	31.270	1.00	0.00
ATOM	4289	HW2	SOL	735	39.640	28.300	30.930	1.00	0.00
ATOM	4290	OW	SOL	736	34.990	40.520	44.400	1.00	0.00
ATOM	4291	HW1	SOL	736	35.150	39.580	44.460	1.00	0.00
ATOM	4292	HW2	SOL	736	34.660	40.770	45.260	1.00	0.00
ATOM	4293	OW	SOL	737	1.420	1.450	55.590	1.00	0.00
ATOM	4294	HW1	SOL	737	2.180	1.650	55.040	1.00	0.00
ATOM	4295	HW2	SOL	737	1.210	0.530	55.390	1.00	0.00
ATOM	4296	OW	SOL	738	32.770	54.980	2.950	1.00	0.00
ATOM	4297	HW1	SOL	738	32.240	55.250	3.700	1.00	0.00
ATOM	4298	HW2	SOL	738	33.630	54.770	3.330	1.00	0.00
ATOM	4299	OW	SOL	739	22.990	5.930	37.970	1.00	0.00
ATOM	4300	HW1	SOL	739	23.290	6.550	38.630	1.00	0.00
ATOM	4301	HW2	SOL	739	22.070	6.150	37.830	1.00	0.00
ATOM	4302	OW	SOL	740	44.850	13.840	24.940	1.00	0.00
ATOM	4303	HW1	SOL	740	45.370	14.620	25.130	1.00	0.00
ATOM	4304	HW2	SOL	740	45.390	13.350	24.310	1.00	0.00
ATOM	4305	OW	SOL	741	27.170	22.060	21.500	1.00	0.00
ATOM	4306	HW1	SOL	741	26.660	22.190	22.290	1.00	0.00
ATOM	4307	HW2	SOL	741	27.830	21.410	21.730	1.00	0.00
ATOM	4308	OW	SOL	742	34.240	7.040	18.040	1.00	0.00
ATOM	4309	HW1	SOL	742	33.660	6.650	17.380	1.00	0.00
ATOM	4310	HW2	SOL	742	34.690	6.310	18.440	1.00	0.00
ATOM	4311	OW	SOL	743	26.880	45.310	43.780	1.00	0.00

ATOM	4312	HW1	SOL	743	27.730	44.900	43.940	1.00	0.00
ATOM	4313	HW2	SOL	743	26.630	45.660	44.640	1.00	0.00
ATOM	4314	OW	SOL	744	43.070	15.110	27.790	1.00	0.00
ATOM	4315	HW1	SOL	744	43.590	15.870	27.530	1.00	0.00
ATOM	4316	HW2	SOL	744	42.240	15.220	27.330	1.00	0.00
ATOM	4317	OW	SOL	745	45.880	40.940	28.410	1.00	0.00
ATOM	4318	HW1	SOL	745	45.940	40.370	27.650	1.00	0.00
ATOM	4319	HW2	SOL	745	45.830	40.340	29.160	1.00	0.00
ATOM	4320	OW	SOL	746	16.610	2.300	54.630	1.00	0.00
ATOM	4321	HW1	SOL	746	17.210	3.010	54.850	1.00	0.00
ATOM	4322	HW2	SOL	746	16.330	2.500	53.730	1.00	0.00
ATOM	4323	OW	SOL	747	24.360	15.770	38.440	1.00	0.00
ATOM	4324	HW1	SOL	747	24.260	15.450	37.540	1.00	0.00
ATOM	4325	HW2	SOL	747	25.280	15.640	38.640	1.00	0.00
ATOM	4326	OW	SOL	748	4.650	24.710	43.350	1.00	0.00
ATOM	4327	HW1	SOL	748	5.110	23.970	43.750	1.00	0.00
ATOM	4328	HW2	SOL	748	4.880	25.460	43.900	1.00	0.00
ATOM	4329	OW	SOL	749	35.820	4.480	45.360	1.00	0.00
ATOM	4330	HW1	SOL	749	36.270	5.300	45.160	1.00	0.00
ATOM	4331	HW2	SOL	749	36.000	4.320	46.280	1.00	0.00
ATOM	4332	OW	SOL	750	26.260	17.830	46.510	1.00	0.00
ATOM	4333	HW1	SOL	750	26.280	17.090	45.910	1.00	0.00
ATOM	4334	HW2	SOL	750	27.160	17.930	46.820	1.00	0.00
ATOM	4335	OW	SOL	751	18.490	27.920	32.300	1.00	0.00
ATOM	4336	HW1	SOL	751	17.540	27.880	32.260	1.00	0.00
ATOM	4337	HW2	SOL	751	18.680	28.770	32.700	1.00	0.00
ATOM	4338	OW	SOL	752	47.430	42.930	29.530	1.00	0.00
ATOM	4339	HW1	SOL	752	47.190	42.110	29.110	1.00	0.00
ATOM	4340	HW2	SOL	752	47.910	43.420	28.860	1.00	0.00
ATOM	4341	OW	SOL	753	2.820	51.610	22.790	1.00	0.00
ATOM	4342	HW1	SOL	753	2.560	52.520	22.640	1.00	0.00
ATOM	4343	HW2	SOL	753	3.670	51.680	23.240	1.00	0.00
ATOM	4344	OW	SOL	754	46.590	21.480	49.250	1.00	0.00
ATOM	4345	HW1	SOL	754	47.090	20.780	49.670	1.00	0.00
ATOM	4346	HW2	SOL	754	47.130	22.260	49.350	1.00	0.00
ATOM	4347	OW	SOL	755	40.950	13.730	32.590	1.00	0.00
ATOM	4348	HW1	SOL	755	41.430	13.150	32.010	1.00	0.00
ATOM	4349	HW2	SOL	755	40.730	14.490	32.050	1.00	0.00
ATOM	4350	OW	SOL	756	47.940	40.850	25.310	1.00	0.00
ATOM	4351	HW1	SOL	756	48.620	40.880	25.980	1.00	0.00
ATOM	4352	HW2	SOL	756	47.120	40.960	25.790	1.00	0.00
ATOM	4353	OW	SOL	757	0.560	37.710	17.650	1.00	0.00
ATOM	4354	HW1	SOL	757	-0.030	38.090	17.000	1.00	0.00
ATOM	4355	HW2	SOL	757	0.040	37.680	18.450	1.00	0.00

ATOM	4356	OW	SOL	758	42.550	50.830	48.850	1.00	0.00
ATOM	4357	HW1	SOL	758	41.670	51.210	48.880	1.00	0.00
ATOM	4358	HW2	SOL	758	42.770	50.790	47.920	1.00	0.00
ATOM	4359	OW	SOL	759	39.480	24.710	35.260	1.00	0.00
ATOM	4360	HW1	SOL	759	39.590	24.700	34.300	1.00	0.00
ATOM	4361	HW2	SOL	759	40.140	24.100	35.580	1.00	0.00
ATOM	4362	OW	SOL	760	9.090	2.750	6.780	1.00	0.00
ATOM	4363	HW1	SOL	760	8.750	3.000	7.630	1.00	0.00
ATOM	4364	HW2	SOL	760	9.410	1.860	6.890	1.00	0.00
ATOM	4365	OW	SOL	761	16.700	24.440	3.100	1.00	0.00
ATOM	4366	HW1	SOL	761	16.950	23.610	2.700	1.00	0.00
ATOM	4367	HW2	SOL	761	15.750	24.480	3.020	1.00	0.00
ATOM	4368	OW	SOL	762	48.430	28.970	45.690	1.00	0.00
ATOM	4369	HW1	SOL	762	48.920	28.350	46.230	1.00	0.00
ATOM	4370	HW2	SOL	762	47.700	29.240	46.250	1.00	0.00
ATOM	4371	OW	SOL	763	48.720	2.840	10.030	1.00	0.00
ATOM	4372	HW1	SOL	763	48.080	2.420	10.600	1.00	0.00
ATOM	4373	HW2	SOL	763	48.400	3.730	9.910	1.00	0.00
ATOM	4374	OW	SOL	764	20.970	31.990	28.560	1.00	0.00
ATOM	4375	HW1	SOL	764	20.080	32.320	28.410	1.00	0.00
ATOM	4376	HW2	SOL	764	21.510	32.780	28.610	1.00	0.00
ATOM	4377	OW	SOL	765	18.760	25.740	25.620	1.00	0.00
ATOM	4378	HW1	SOL	765	18.950	26.650	25.860	1.00	0.00
ATOM	4379	HW2	SOL	765	18.420	25.340	26.420	1.00	0.00
ATOM	4380	OW	SOL	766	55.080	49.120	29.230	1.00	0.00
ATOM	4381	HW1	SOL	766	55.390	48.920	30.110	1.00	0.00
ATOM	4382	HW2	SOL	766	54.710	48.290	28.920	1.00	0.00
ATOM	4383	OW	SOL	767	9.920	51.970	31.300	1.00	0.00
ATOM	4384	HW1	SOL	767	10.000	52.850	31.660	1.00	0.00
ATOM	4385	HW2	SOL	767	9.690	51.420	32.060	1.00	0.00
ATOM	4386	OW	SOL	768	8.370	51.910	6.530	1.00	0.00
ATOM	4387	HW1	SOL	768	7.720	51.280	6.810	1.00	0.00
ATOM	4388	HW2	SOL	768	8.080	52.750	6.900	1.00	0.00
ATOM	4389	OW	SOL	769	3.450	1.550	53.200	1.00	0.00
ATOM	4390	HW1	SOL	769	2.850	1.920	52.550	1.00	0.00
ATOM	4391	HW2	SOL	769	4.300	1.540	52.750	1.00	0.00
ATOM	4392	OW	SOL	770	41.450	26.260	15.820	1.00	0.00
ATOM	4393	HW1	SOL	770	40.650	26.620	16.190	1.00	0.00
ATOM	4394	HW2	SOL	770	41.840	26.990	15.330	1.00	0.00
ATOM	4395	OW	SOL	771	15.660	43.360	52.600	1.00	0.00
ATOM	4396	HW1	SOL	771	14.860	43.550	52.120	1.00	0.00
ATOM	4397	HW2	SOL	771	16.130	44.190	52.640	1.00	0.00
ATOM	4398	OW	SOL	772	14.870	14.440	29.920	1.00	0.00
ATOM	4399	HW1	SOL	772	15.740	14.760	29.670	1.00	0.00

ATOM	4400	HW2	SOL	772	14.850	14.530	30.870	1.00	0.00
ATOM	4401	OW	SOL	773	33.630	24.480	21.560	1.00	0.00
ATOM	4402	HW1	SOL	773	34.000	23.640	21.270	1.00	0.00
ATOM	4403	HW2	SOL	773	33.810	25.080	20.840	1.00	0.00
ATOM	4404	OW	SOL	774	4.770	23.990	19.980	1.00	0.00
ATOM	4405	HW1	SOL	774	5.240	23.320	20.470	1.00	0.00
ATOM	4406	HW2	SOL	774	4.550	23.570	19.150	1.00	0.00
ATOM	4407	OW	SOL	775	52.400	5.400	33.650	1.00	0.00
ATOM	4408	HW1	SOL	775	52.180	5.660	34.540	1.00	0.00
ATOM	4409	HW2	SOL	775	51.780	4.700	33.440	1.00	0.00
ATOM	4410	OW	SOL	776	9.790	13.650	47.980	1.00	0.00
ATOM	4411	HW1	SOL	776	9.920	14.390	48.580	1.00	0.00
ATOM	4412	HW2	SOL	776	8.850	13.690	47.770	1.00	0.00
ATOM	4413	OW	SOL	777	55.220	12.130	36.910	1.00	0.00
ATOM	4414	HW1	SOL	777	54.400	11.700	36.690	1.00	0.00
ATOM	4415	HW2	SOL	777	55.120	13.030	36.590	1.00	0.00
ATOM	4416	OW	SOL	778	36.230	42.890	44.210	1.00	0.00
ATOM	4417	HW1	SOL	778	35.700	42.090	44.230	1.00	0.00
ATOM	4418	HW2	SOL	778	36.510	42.970	43.300	1.00	0.00
ATOM	4419	OW	SOL	779	20.100	26.320	30.300	1.00	0.00
ATOM	4420	HW1	SOL	779	19.670	26.380	29.440	1.00	0.00
ATOM	4421	HW2	SOL	779	19.710	27.030	30.810	1.00	0.00
ATOM	4422	OW	SOL	780	46.500	30.720	46.880	1.00	0.00
ATOM	4423	HW1	SOL	780	45.620	30.410	46.640	1.00	0.00
ATOM	4424	HW2	SOL	780	46.740	31.310	46.170	1.00	0.00
ATOM	4425	OW	SOL	781	6.340	28.790	17.530	1.00	0.00
ATOM	4426	HW1	SOL	781	7.260	28.740	17.780	1.00	0.00
ATOM	4427	HW2	SOL	781	6.210	28.060	16.930	1.00	0.00
ATOM	4428	OW	SOL	782	12.250	42.290	49.150	1.00	0.00
ATOM	4429	HW1	SOL	782	12.560	41.520	49.630	1.00	0.00
ATOM	4430	HW2	SOL	782	12.280	42.030	48.230	1.00	0.00
ATOM	4431	OW	SOL	783	16.670	48.660	20.820	1.00	0.00
ATOM	4432	HW1	SOL	783	17.540	48.390	20.530	1.00	0.00
ATOM	4433	HW2	SOL	783	16.240	47.850	21.080	1.00	0.00
ATOM	4434	OW	SOL	784	12.710	9.630	42.780	1.00	0.00
ATOM	4435	HW1	SOL	784	13.330	9.070	43.250	1.00	0.00
ATOM	4436	HW2	SOL	784	12.240	9.030	42.200	1.00	0.00
ATOM	4437	OW	SOL	785	41.840	8.190	29.780	1.00	0.00
ATOM	4438	HW1	SOL	785	41.650	7.630	29.020	1.00	0.00
ATOM	4439	HW2	SOL	785	40.980	8.460	30.090	1.00	0.00
ATOM	4440	OW	SOL	786	16.260	15.890	52.320	1.00	0.00
ATOM	4441	HW1	SOL	786	16.850	15.250	51.920	1.00	0.00
ATOM	4442	HW2	SOL	786	16.610	16.740	52.060	1.00	0.00
ATOM	4443	OW	SOL	787	49.070	10.000	44.370	1.00	0.00

ATOM	4444	HW1	SOL	787	49.120	10.760	44.950	1.00	0.00
ATOM	4445	HW2	SOL	787	48.530	9.370	44.860	1.00	0.00
ATOM	4446	OW	SOL	788	8.270	12.690	34.910	1.00	0.00
ATOM	4447	HW1	SOL	788	7.390	13.040	35.080	1.00	0.00
ATOM	4448	HW2	SOL	788	8.560	12.370	35.770	1.00	0.00
ATOM	4449	OW	SOL	789	43.080	1.890	20.450	1.00	0.00
ATOM	4450	HW1	SOL	789	43.200	1.050	20.900	1.00	0.00
ATOM	4451	HW2	SOL	789	43.910	2.350	20.580	1.00	0.00
ATOM	4452	OW	SOL	790	9.360	4.230	52.500	1.00	0.00
ATOM	4453	HW1	SOL	790	9.450	3.450	53.050	1.00	0.00
ATOM	4454	HW2	SOL	790	9.870	4.040	51.720	1.00	0.00
ATOM	4455	OW	SOL	791	35.880	20.880	42.690	1.00	0.00
ATOM	4456	HW1	SOL	791	36.760	21.040	43.010	1.00	0.00
ATOM	4457	HW2	SOL	791	35.990	20.650	41.770	1.00	0.00
ATOM	4458	OW	SOL	792	17.910	49.670	4.270	1.00	0.00
ATOM	4459	HW1	SOL	792	18.600	50.310	4.420	1.00	0.00
ATOM	4460	HW2	SOL	792	17.740	49.280	5.130	1.00	0.00
ATOM	4461	OW	SOL	793	36.060	23.060	18.410	1.00	0.00
ATOM	4462	HW1	SOL	793	36.730	22.390	18.560	1.00	0.00
ATOM	4463	HW2	SOL	793	36.180	23.320	17.500	1.00	0.00
ATOM	4464	OW	SOL	794	26.390	37.240	6.140	1.00	0.00
ATOM	4465	HW1	SOL	794	26.730	36.440	5.740	1.00	0.00
ATOM	4466	HW2	SOL	794	27.130	37.580	6.660	1.00	0.00
ATOM	4467	OW	SOL	795	46.830	36.430	21.250	1.00	0.00
ATOM	4468	HW1	SOL	795	47.660	36.300	21.710	1.00	0.00
ATOM	4469	HW2	SOL	795	46.910	37.300	20.850	1.00	0.00
ATOM	4470	OW	SOL	796	45.590	20.250	3.790	1.00	0.00
ATOM	4471	HW1	SOL	796	46.150	20.980	4.040	1.00	0.00
ATOM	4472	HW2	SOL	796	46.200	19.540	3.570	1.00	0.00
ATOM	4473	OW	SOL	797	50.640	29.420	36.990	1.00	0.00
ATOM	4474	HW1	SOL	797	49.750	29.760	37.070	1.00	0.00
ATOM	4475	HW2	SOL	797	51.110	29.810	37.720	1.00	0.00
ATOM	4476	OW	SOL	798	45.370	53.110	14.590	1.00	0.00
ATOM	4477	HW1	SOL	798	45.480	52.160	14.560	1.00	0.00
ATOM	4478	HW2	SOL	798	44.710	53.300	13.930	1.00	0.00
ATOM	4479	OW	SOL	799	52.880	27.050	47.750	1.00	0.00
ATOM	4480	HW1	SOL	799	53.580	27.400	47.190	1.00	0.00
ATOM	4481	HW2	SOL	799	53.340	26.570	48.440	1.00	0.00
ATOM	4482	OW	SOL	800	50.650	47.210	50.760	1.00	0.00
ATOM	4483	HW1	SOL	800	50.310	46.320	50.640	1.00	0.00
ATOM	4484	HW2	SOL	800	51.120	47.180	51.600	1.00	0.00
ATOM	4485	OW	SOL	801	42.630	21.270	16.190	1.00	0.00
ATOM	4486	HW1	SOL	801	43.510	20.890	16.230	1.00	0.00
ATOM	4487	HW2	SOL	801	42.730	22.040	15.620	1.00	0.00

ATOM	4488	OW	SOL	802	36.940	43.670	3.880	1.00	0.00
ATOM	4489	HW1	SOL	802	37.490	44.430	3.690	1.00	0.00
ATOM	4490	HW2	SOL	802	36.250	43.710	3.220	1.00	0.00
ATOM	4491	OW	SOL	803	21.990	15.170	24.030	1.00	0.00
ATOM	4492	HW1	SOL	803	21.500	15.330	23.220	1.00	0.00
ATOM	4493	HW2	SOL	803	22.310	14.270	23.950	1.00	0.00
ATOM	4494	OW	SOL	804	6.320	9.580	17.060	1.00	0.00
ATOM	4495	HW1	SOL	804	6.750	9.840	16.240	1.00	0.00
ATOM	4496	HW2	SOL	804	7.000	9.660	17.720	1.00	0.00
ATOM	4497	OW	SOL	805	34.640	31.680	33.190	1.00	0.00
ATOM	4498	HW1	SOL	805	34.920	32.420	33.740	1.00	0.00
ATOM	4499	HW2	SOL	805	34.130	32.090	32.490	1.00	0.00
ATOM	4500	OW	SOL	806	50.920	51.730	14.910	1.00	0.00
ATOM	4501	HW1	SOL	806	50.590	50.840	14.960	1.00	0.00
ATOM	4502	HW2	SOL	806	50.730	52.010	14.010	1.00	0.00
ATOM	4503	OW	SOL	807	46.550	12.370	23.120	1.00	0.00
ATOM	4504	HW1	SOL	807	46.270	12.090	22.250	1.00	0.00
ATOM	4505	HW2	SOL	807	46.230	11.680	23.700	1.00	0.00
ATOM	4506	OW	SOL	808	29.810	26.980	3.320	1.00	0.00
ATOM	4507	HW1	SOL	808	30.400	27.680	3.040	1.00	0.00
ATOM	4508	HW2	SOL	808	29.120	27.430	3.800	1.00	0.00
ATOM	4509	OW	SOL	809	47.720	31.490	53.460	1.00	0.00
ATOM	4510	HW1	SOL	809	47.480	30.910	54.190	1.00	0.00
ATOM	4511	HW2	SOL	809	47.810	30.900	52.710	1.00	0.00
ATOM	4512	OW	SOL	810	17.620	29.100	42.790	1.00	0.00
ATOM	4513	HW1	SOL	810	17.040	28.500	43.270	1.00	0.00
ATOM	4514	HW2	SOL	810	18.270	28.530	42.390	1.00	0.00
ATOM	4515	OW	SOL	811	48.530	18.580	7.250	1.00	0.00
ATOM	4516	HW1	SOL	811	47.610	18.510	7.000	1.00	0.00
ATOM	4517	HW2	SOL	811	48.770	17.700	7.550	1.00	0.00
ATOM	4518	OW	SOL	812	16.260	29.290	20.330	1.00	0.00
ATOM	4519	HW1	SOL	812	15.850	29.800	19.630	1.00	0.00
ATOM	4520	HW2	SOL	812	15.840	28.430	20.280	1.00	0.00
ATOM	4521	OW	SOL	813	40.660	29.310	49.410	1.00	0.00
ATOM	4522	HW1	SOL	813	41.130	30.140	49.410	1.00	0.00
ATOM	4523	HW2	SOL	813	39.910	29.460	48.830	1.00	0.00
ATOM	4524	OW	SOL	814	7.620	23.830	42.410	1.00	0.00
ATOM	4525	HW1	SOL	814	6.890	23.970	41.800	1.00	0.00
ATOM	4526	HW2	SOL	814	8.200	23.220	41.940	1.00	0.00
ATOM	4527	OW	SOL	815	51.120	31.340	54.410	1.00	0.00
ATOM	4528	HW1	SOL	815	51.510	30.810	55.110	1.00	0.00
ATOM	4529	HW2	SOL	815	50.560	31.970	54.880	1.00	0.00
ATOM	4530	OW	SOL	816	42.290	25.420	25.260	1.00	0.00
ATOM	4531	HW1	SOL	816	41.850	25.260	24.430	1.00	0.00

ATOM	4532	HW2	SOL	816	41.940	26.260	25.560	1.00	0.00
ATOM	4533	OW	SOL	817	13.300	16.110	10.320	1.00	0.00
ATOM	4534	HW1	SOL	817	12.370	15.870	10.290	1.00	0.00
ATOM	4535	HW2	SOL	817	13.300	17.070	10.330	1.00	0.00
ATOM	4536	OW	SOL	818	7.650	51.520	38.290	1.00	0.00
ATOM	4537	HW1	SOL	818	7.520	52.240	38.900	1.00	0.00
ATOM	4538	HW2	SOL	818	8.250	50.930	38.740	1.00	0.00
ATOM	4539	OW	SOL	819	31.260	48.520	38.840	1.00	0.00
ATOM	4540	HW1	SOL	819	31.760	48.030	39.490	1.00	0.00
ATOM	4541	HW2	SOL	819	30.610	49.010	39.350	1.00	0.00
ATOM	4542	OW	SOL	820	26.570	5.610	11.880	1.00	0.00
ATOM	4543	HW1	SOL	820	25.910	5.890	12.520	1.00	0.00
ATOM	4544	HW2	SOL	820	26.080	5.070	11.260	1.00	0.00
ATOM	4545	OW	SOL	821	19.870	28.350	13.750	1.00	0.00
ATOM	4546	HW1	SOL	821	20.180	28.740	12.930	1.00	0.00
ATOM	4547	HW2	SOL	821	20.670	28.150	14.240	1.00	0.00
ATOM	4548	OW	SOL	822	43.610	25.100	0.260	1.00	0.00
ATOM	4549	HW1	SOL	822	44.100	25.910	0.420	1.00	0.00
ATOM	4550	HW2	SOL	822	42.940	25.080	0.940	1.00	0.00
ATOM	4551	OW	SOL	823	22.080	51.560	4.620	1.00	0.00
ATOM	4552	HW1	SOL	823	21.260	51.460	5.110	1.00	0.00
ATOM	4553	HW2	SOL	823	22.630	50.830	4.920	1.00	0.00
ATOM	4554	OW	SOL	824	51.710	9.710	9.740	1.00	0.00
ATOM	4555	HW1	SOL	824	52.620	9.620	10.030	1.00	0.00
ATOM	4556	HW2	SOL	824	51.770	10.140	8.890	1.00	0.00
ATOM	4557	OW	SOL	825	45.660	17.440	6.880	1.00	0.00
ATOM	4558	HW1	SOL	825	45.860	17.320	5.960	1.00	0.00
ATOM	4559	HW2	SOL	825	46.300	16.910	7.350	1.00	0.00
ATOM	4560	OW	SOL	826	43.750	24.510	18.300	1.00	0.00
ATOM	4561	HW1	SOL	826	43.770	25.260	18.880	1.00	0.00
ATOM	4562	HW2	SOL	826	42.900	24.560	17.860	1.00	0.00
ATOM	4563	OW	SOL	827	1.970	48.820	15.230	1.00	0.00
ATOM	4564	HW1	SOL	827	1.120	48.400	15.350	1.00	0.00
ATOM	4565	HW2	SOL	827	2.540	48.120	14.910	1.00	0.00
ATOM	4566	OW	SOL	828	47.370	53.220	24.940	1.00	0.00
ATOM	4567	HW1	SOL	828	48.090	53.060	25.550	1.00	0.00
ATOM	4568	HW2	SOL	828	47.690	52.880	24.100	1.00	0.00
ATOM	4569	OW	SOL	829	9.340	3.240	20.050	1.00	0.00
ATOM	4570	HW1	SOL	829	8.860	4.030	20.290	1.00	0.00
ATOM	4571	HW2	SOL	829	10.070	3.210	20.660	1.00	0.00
ATOM	4572	OW	SOL	830	17.010	7.450	5.890	1.00	0.00
ATOM	4573	HW1	SOL	830	17.760	8.000	6.140	1.00	0.00
ATOM	4574	HW2	SOL	830	17.370	6.560	5.870	1.00	0.00
ATOM	4575	OW	SOL	831	48.310	35.490	3.170	1.00	0.00

ATOM	4576	HW1	SOL	831	49.230	35.680	3.000	1.00	0.00
ATOM	4577	HW2	SOL	831	47.840	35.890	2.440	1.00	0.00
ATOM	4578	OW	SOL	832	9.130	46.570	47.140	1.00	0.00
ATOM	4579	HW1	SOL	832	8.550	46.080	46.550	1.00	0.00
ATOM	4580	HW2	SOL	832	9.420	45.920	47.790	1.00	0.00
ATOM	4581	OW	SOL	833	53.540	53.480	14.080	1.00	0.00
ATOM	4582	HW1	SOL	833	53.540	52.960	13.270	1.00	0.00
ATOM	4583	HW2	SOL	833	54.320	54.020	14.020	1.00	0.00
ATOM	4584	OW	SOL	834	5.260	51.510	10.610	1.00	0.00
ATOM	4585	HW1	SOL	834	5.860	51.100	11.240	1.00	0.00
ATOM	4586	HW2	SOL	834	4.740	50.790	10.270	1.00	0.00
ATOM	4587	OW	SOL	835	4.420	19.600	28.720	1.00	0.00
ATOM	4588	HW1	SOL	835	4.220	20.510	28.480	1.00	0.00
ATOM	4589	HW2	SOL	835	5.280	19.430	28.320	1.00	0.00
ATOM	4590	OW	SOL	836	28.810	22.590	7.460	1.00	0.00
ATOM	4591	HW1	SOL	836	28.280	22.800	6.690	1.00	0.00
ATOM	4592	HW2	SOL	836	28.310	22.950	8.190	1.00	0.00
ATOM	4593	OW	SOL	837	26.790	20.070	34.450	1.00	0.00
ATOM	4594	HW1	SOL	837	25.950	19.670	34.220	1.00	0.00
ATOM	4595	HW2	SOL	837	26.560	20.940	34.770	1.00	0.00
ATOM	4596	OW	SOL	838	55.570	8.090	50.590	1.00	0.00
ATOM	4597	HW1	SOL	838	55.740	8.930	50.180	1.00	0.00
ATOM	4598	HW2	SOL	838	55.860	7.440	49.950	1.00	0.00
ATOM	4599	OW	SOL	839	5.500	20.940	23.270	1.00	0.00
ATOM	4600	HW1	SOL	839	5.710	21.750	23.730	1.00	0.00
ATOM	4601	HW2	SOL	839	5.980	20.260	23.740	1.00	0.00
ATOM	4602	OW	SOL	840	5.330	45.520	7.690	1.00	0.00
ATOM	4603	HW1	SOL	840	5.680	44.880	7.060	1.00	0.00
ATOM	4604	HW2	SOL	840	4.390	45.390	7.670	1.00	0.00
ATOM	4605	OW	SOL	841	37.810	14.070	20.310	1.00	0.00
ATOM	4606	HW1	SOL	841	36.880	13.870	20.370	1.00	0.00
ATOM	4607	HW2	SOL	841	38.030	14.440	21.160	1.00	0.00
ATOM	4608	OW	SOL	842	0.370	31.030	25.830	1.00	0.00
ATOM	4609	HW1	SOL	842	0.010	30.580	26.590	1.00	0.00
ATOM	4610	HW2	SOL	842	-0.100	31.860	25.790	1.00	0.00
ATOM	4611	OW	SOL	843	10.950	41.190	34.200	1.00	0.00
ATOM	4612	HW1	SOL	843	11.720	41.750	34.300	1.00	0.00
ATOM	4613	HW2	SOL	843	10.310	41.540	34.820	1.00	0.00
ATOM	4614	OW	SOL	844	48.170	19.430	13.180	1.00	0.00
ATOM	4615	HW1	SOL	844	48.260	20.280	13.610	1.00	0.00
ATOM	4616	HW2	SOL	844	47.790	18.860	13.850	1.00	0.00
ATOM	4617	OW	SOL	845	38.470	52.410	9.390	1.00	0.00
ATOM	4618	HW1	SOL	845	39.170	52.130	9.970	1.00	0.00
ATOM	4619	HW2	SOL	845	38.780	53.240	9.020	1.00	0.00

ATOM	4620	OW	SOL	846	17.280	6.460	38.530	1.00	0.00
ATOM	4621	HW1	SOL	846	17.100	7.090	39.230	1.00	0.00
ATOM	4622	HW2	SOL	846	17.910	6.900	37.960	1.00	0.00
ATOM	4623	OW	SOL	847	33.800	31.690	3.790	1.00	0.00
ATOM	4624	HW1	SOL	847	34.180	31.920	2.940	1.00	0.00
ATOM	4625	HW2	SOL	847	34.350	30.980	4.120	1.00	0.00
ATOM	4626	OW	SOL	848	25.000	0.990	37.160	1.00	0.00
ATOM	4627	HW1	SOL	848	24.500	1.810	37.130	1.00	0.00
ATOM	4628	HW2	SOL	848	25.540	1.060	37.940	1.00	0.00
ATOM	4629	OW	SOL	849	45.590	17.310	52.790	1.00	0.00
ATOM	4630	HW1	SOL	849	44.930	17.960	52.520	1.00	0.00
ATOM	4631	HW2	SOL	849	45.210	16.910	53.580	1.00	0.00
ATOM	4632	OW	SOL	850	35.020	27.390	36.760	1.00	0.00
ATOM	4633	HW1	SOL	850	35.600	27.280	37.520	1.00	0.00
ATOM	4634	HW2	SOL	850	35.270	28.240	36.400	1.00	0.00
ATOM	4635	OW	SOL	851	6.700	50.560	47.800	1.00	0.00
ATOM	4636	HW1	SOL	851	7.610	50.410	47.570	1.00	0.00
ATOM	4637	HW2	SOL	851	6.690	51.420	48.230	1.00	0.00
ATOM	4638	OW	SOL	852	39.250	20.410	7.110	1.00	0.00
ATOM	4639	HW1	SOL	852	40.060	19.960	6.850	1.00	0.00
ATOM	4640	HW2	SOL	852	39.260	20.370	8.070	1.00	0.00
ATOM	4641	OW	SOL	853	14.380	39.930	3.970	1.00	0.00
ATOM	4642	HW1	SOL	853	15.030	39.570	3.370	1.00	0.00
ATOM	4643	HW2	SOL	853	13.630	39.330	3.900	1.00	0.00
ATOM	4644	OW	SOL	854	13.250	55.590	12.170	1.00	0.00
ATOM	4645	HW1	SOL	854	13.330	55.300	11.260	1.00	0.00
ATOM	4646	HW2	SOL	854	13.510	56.510	12.150	1.00	0.00
ATOM	4647	OW	SOL	855	8.080	19.120	12.100	1.00	0.00
ATOM	4648	HW1	SOL	855	8.390	19.590	11.320	1.00	0.00
ATOM	4649	HW2	SOL	855	7.260	19.560	12.330	1.00	0.00
ATOM	4650	OW	SOL	856	28.750	6.550	34.900	1.00	0.00
ATOM	4651	HW1	SOL	856	27.960	6.600	34.360	1.00	0.00
ATOM	4652	HW2	SOL	856	28.430	6.320	35.770	1.00	0.00
ATOM	4653	OW	SOL	857	49.710	42.960	55.280	1.00	0.00
ATOM	4654	HW1	SOL	857	49.960	43.360	54.440	1.00	0.00
ATOM	4655	HW2	SOL	857	49.340	43.680	55.780	1.00	0.00
ATOM	4656	OW	SOL	858	28.140	51.710	43.110	1.00	0.00
ATOM	4657	HW1	SOL	858	29.010	51.550	43.460	1.00	0.00
ATOM	4658	HW2	SOL	858	28.050	52.660	43.110	1.00	0.00
ATOM	4659	OW	SOL	859	11.070	22.410	40.870	1.00	0.00
ATOM	4660	HW1	SOL	859	11.350	23.190	41.360	1.00	0.00
ATOM	4661	HW2	SOL	859	10.820	22.750	40.010	1.00	0.00
ATOM	4662	OW	SOL	860	54.940	11.780	5.900	1.00	0.00
ATOM	4663	HW1	SOL	860	54.820	12.730	5.950	1.00	0.00

ATOM	4664	HW2	SOL	860	54.730	11.560	4.990	1.00	0.00
ATOM	4665	OW	SOL	861	43.190	16.590	46.770	1.00	0.00
ATOM	4666	HW1	SOL	861	42.950	17.110	47.540	1.00	0.00
ATOM	4667	HW2	SOL	861	43.870	15.990	47.090	1.00	0.00
ATOM	4668	OW	SOL	862	26.420	51.040	9.520	1.00	0.00
ATOM	4669	HW1	SOL	862	26.910	50.350	9.070	1.00	0.00
ATOM	4670	HW2	SOL	862	25.520	50.940	9.210	1.00	0.00
ATOM	4671	OW	SOL	863	23.000	18.150	13.020	1.00	0.00
ATOM	4672	HW1	SOL	863	23.140	18.350	13.940	1.00	0.00
ATOM	4673	HW2	SOL	863	22.180	17.660	13.000	1.00	0.00
ATOM	4674	OW	SOL	864	34.120	24.950	15.110	1.00	0.00
ATOM	4675	HW1	SOL	864	33.400	25.340	14.610	1.00	0.00
ATOM	4676	HW2	SOL	864	34.510	25.690	15.580	1.00	0.00
ATOM	4677	OW	SOL	865	17.280	45.300	14.090	1.00	0.00
ATOM	4678	HW1	SOL	865	17.690	44.670	13.510	1.00	0.00
ATOM	4679	HW2	SOL	865	16.630	45.740	13.550	1.00	0.00
ATOM	4680	OW	SOL	866	41.770	5.140	49.650	1.00	0.00
ATOM	4681	HW1	SOL	866	41.120	5.630	49.150	1.00	0.00
ATOM	4682	HW2	SOL	866	41.550	4.230	49.490	1.00	0.00
ATOM	4683	OW	SOL	867	6.500	55.160	11.750	1.00	0.00
ATOM	4684	HW1	SOL	867	7.120	54.880	11.070	1.00	0.00
ATOM	4685	HW2	SOL	867	5.810	54.490	11.730	1.00	0.00
ATOM	4686	OW	SOL	868	46.270	53.300	11.020	1.00	0.00
ATOM	4687	HW1	SOL	868	45.770	52.680	10.510	1.00	0.00
ATOM	4688	HW2	SOL	868	47.150	53.280	10.640	1.00	0.00
ATOM	4689	OW	SOL	869	31.060	5.210	44.480	1.00	0.00
ATOM	4690	HW1	SOL	869	31.790	4.590	44.560	1.00	0.00
ATOM	4691	HW2	SOL	869	30.320	4.770	44.880	1.00	0.00
ATOM	4692	OW	SOL	870	45.040	20.570	15.560	1.00	0.00
ATOM	4693	HW1	SOL	870	45.280	19.750	15.130	1.00	0.00
ATOM	4694	HW2	SOL	870	45.270	21.250	14.930	1.00	0.00
ATOM	4695	OW	SOL	871	21.900	49.410	43.210	1.00	0.00
ATOM	4696	HW1	SOL	871	22.310	48.580	43.480	1.00	0.00
ATOM	4697	HW2	SOL	871	21.980	49.410	42.250	1.00	0.00
ATOM	4698	OW	SOL	872	2.490	45.380	6.030	1.00	0.00
ATOM	4699	HW1	SOL	872	2.590	44.580	5.510	1.00	0.00
ATOM	4700	HW2	SOL	872	2.160	45.070	6.880	1.00	0.00
ATOM	4701	OW	SOL	873	23.650	7.780	11.610	1.00	0.00
ATOM	4702	HW1	SOL	873	23.910	7.520	10.720	1.00	0.00
ATOM	4703	HW2	SOL	873	23.110	7.050	11.930	1.00	0.00
ATOM	4704	OW	SOL	874	21.270	13.510	48.080	1.00	0.00
ATOM	4705	HW1	SOL	874	20.900	14.340	48.370	1.00	0.00
ATOM	4706	HW2	SOL	874	22.150	13.730	47.770	1.00	0.00
ATOM	4707	OW	SOL	875	41.830	6.780	27.480	1.00	0.00

ATOM	4708	HW1	SOL	875	42.530	7.090	26.910	1.00	0.00
ATOM	4709	HW2	SOL	875	41.120	6.550	26.880	1.00	0.00
ATOM	4710	OW	SOL	876	47.180	26.150	10.080	1.00	0.00
ATOM	4711	HW1	SOL	876	47.170	26.100	11.040	1.00	0.00
ATOM	4712	HW2	SOL	876	47.880	25.560	9.810	1.00	0.00
ATOM	4713	OW	SOL	877	7.250	14.880	43.750	1.00	0.00
ATOM	4714	HW1	SOL	877	6.700	14.850	42.970	1.00	0.00
ATOM	4715	HW2	SOL	877	8.140	14.730	43.420	1.00	0.00
ATOM	4716	OW	SOL	878	44.650	32.550	22.520	1.00	0.00
ATOM	4717	HW1	SOL	878	45.200	32.720	21.760	1.00	0.00
ATOM	4718	HW2	SOL	878	44.350	31.640	22.400	1.00	0.00
ATOM	4719	OW	SOL	879	53.500	45.420	40.090	1.00	0.00
ATOM	4720	HW1	SOL	879	52.570	45.280	40.270	1.00	0.00
ATOM	4721	HW2	SOL	879	53.580	46.360	39.930	1.00	0.00
ATOM	4722	OW	SOL	880	43.970	42.300	10.190	1.00	0.00
ATOM	4723	HW1	SOL	880	43.990	41.460	10.650	1.00	0.00
ATOM	4724	HW2	SOL	880	44.170	42.950	10.870	1.00	0.00
ATOM	4725	OW	SOL	881	16.020	26.800	48.290	1.00	0.00
ATOM	4726	HW1	SOL	881	15.110	26.620	48.020	1.00	0.00
ATOM	4727	HW2	SOL	881	15.990	26.760	49.240	1.00	0.00
ATOM	4728	OW	SOL	882	52.820	14.000	29.260	1.00	0.00
ATOM	4729	HW1	SOL	882	53.490	14.660	29.410	1.00	0.00
ATOM	4730	HW2	SOL	882	52.290	14.350	28.550	1.00	0.00
ATOM	4731	OW	SOL	883	24.560	38.970	36.240	1.00	0.00
ATOM	4732	HW1	SOL	883	25.340	39.230	35.760	1.00	0.00
ATOM	4733	HW2	SOL	883	24.760	39.190	37.150	1.00	0.00
ATOM	4734	OW	SOL	884	34.990	4.270	40.470	1.00	0.00
ATOM	4735	HW1	SOL	884	35.110	3.420	40.040	1.00	0.00
ATOM	4736	HW2	SOL	884	35.580	4.230	41.220	1.00	0.00
ATOM	4737	OW	SOL	885	47.330	28.260	16.660	1.00	0.00
ATOM	4738	HW1	SOL	885	47.180	28.620	17.540	1.00	0.00
ATOM	4739	HW2	SOL	885	46.670	27.580	16.560	1.00	0.00
ATOM	4740	OW	SOL	886	49.170	40.630	47.350	1.00	0.00
ATOM	4741	HW1	SOL	886	49.350	41.100	46.530	1.00	0.00
ATOM	4742	HW2	SOL	886	48.920	41.320	47.960	1.00	0.00
ATOM	4743	OW	SOL	887	17.770	33.570	16.380	1.00	0.00
ATOM	4744	HW1	SOL	887	17.990	34.370	16.850	1.00	0.00
ATOM	4745	HW2	SOL	887	18.600	33.260	16.020	1.00	0.00
ATOM	4746	OW	SOL	888	7.520	25.260	15.100	1.00	0.00
ATOM	4747	HW1	SOL	888	7.690	24.920	15.980	1.00	0.00
ATOM	4748	HW2	SOL	888	7.990	24.670	14.520	1.00	0.00
ATOM	4749	OW	SOL	889	38.130	18.890	12.370	1.00	0.00
ATOM	4750	HW1	SOL	889	37.390	18.280	12.300	1.00	0.00
ATOM	4751	HW2	SOL	889	38.800	18.400	12.840	1.00	0.00

ATOM	4752	OW	SOL	890	47.470	48.010	5.520	1.00	0.00
ATOM	4753	HW1	SOL	890	47.580	48.860	5.950	1.00	0.00
ATOM	4754	HW2	SOL	890	46.630	47.690	5.830	1.00	0.00
ATOM	4755	OW	SOL	891	52.890	31.900	18.620	1.00	0.00
ATOM	4756	HW1	SOL	891	53.410	31.110	18.500	1.00	0.00
ATOM	4757	HW2	SOL	891	52.850	32.300	17.750	1.00	0.00
ATOM	4758	OW	SOL	892	22.380	32.830	42.920	1.00	0.00
ATOM	4759	HW1	SOL	892	22.550	32.300	42.150	1.00	0.00
ATOM	4760	HW2	SOL	892	21.970	32.230	43.550	1.00	0.00
ATOM	4761	OW	SOL	893	37.750	35.310	53.620	1.00	0.00
ATOM	4762	HW1	SOL	893	37.050	34.780	53.250	1.00	0.00
ATOM	4763	HW2	SOL	893	37.400	36.200	53.620	1.00	0.00
ATOM	4764	OW	SOL	894	33.360	16.600	10.590	1.00	0.00
ATOM	4765	HW1	SOL	894	34.240	16.930	10.760	1.00	0.00
ATOM	4766	HW2	SOL	894	33.460	15.650	10.600	1.00	0.00
ATOM	4767	OW	SOL	895	11.280	12.290	15.920	1.00	0.00
ATOM	4768	HW1	SOL	895	11.170	11.470	15.440	1.00	0.00
ATOM	4769	HW2	SOL	895	10.420	12.710	15.880	1.00	0.00
ATOM	4770	OW	SOL	896	0.500	19.490	40.270	1.00	0.00
ATOM	4771	HW1	SOL	896	-0.320	19.960	40.100	1.00	0.00
ATOM	4772	HW2	SOL	896	0.260	18.830	40.930	1.00	0.00
ATOM	4773	OW	SOL	897	9.610	18.290	14.030	1.00	0.00
ATOM	4774	HW1	SOL	897	9.140	18.310	13.200	1.00	0.00
ATOM	4775	HW2	SOL	897	10.530	18.180	13.790	1.00	0.00
ATOM	4776	OW	SOL	898	33.540	5.160	11.030	1.00	0.00
ATOM	4777	HW1	SOL	898	33.890	4.710	11.800	1.00	0.00
ATOM	4778	HW2	SOL	898	32.590	5.140	11.140	1.00	0.00
ATOM	4779	OW	SOL	899	4.720	44.460	30.580	1.00	0.00
ATOM	4780	HW1	SOL	899	5.520	44.210	30.110	1.00	0.00
ATOM	4781	HW2	SOL	899	4.930	44.300	31.500	1.00	0.00
ATOM	4782	OW	SOL	900	12.110	7.970	10.220	1.00	0.00
ATOM	4783	HW1	SOL	900	11.910	7.350	10.910	1.00	0.00
ATOM	4784	HW2	SOL	900	12.520	8.710	10.660	1.00	0.00
ATOM	4785	OW	SOL	901	47.240	24.100	16.530	1.00	0.00
ATOM	4786	HW1	SOL	901	48.200	24.120	16.450	1.00	0.00
ATOM	4787	HW2	SOL	901	46.940	23.760	15.680	1.00	0.00
ATOM	4788	OW	SOL	902	26.860	24.580	29.080	1.00	0.00
ATOM	4789	HW1	SOL	902	27.490	24.660	29.800	1.00	0.00
ATOM	4790	HW2	SOL	902	26.530	23.680	29.150	1.00	0.00
ATOM	4791	OW	SOL	903	50.950	13.030	24.290	1.00	0.00
ATOM	4792	HW1	SOL	903	51.650	13.520	23.860	1.00	0.00
ATOM	4793	HW2	SOL	903	50.140	13.470	24.040	1.00	0.00
ATOM	4794	OW	SOL	904	40.930	53.100	50.690	1.00	0.00
ATOM	4795	HW1	SOL	904	41.330	53.380	51.510	1.00	0.00

ATOM	4796	HW2	SOL	904	41.190	53.760	50.050	1.00	0.00
ATOM	4797	OW	SOL	905	6.710	2.090	0.400	1.00	0.00
ATOM	4798	HW1	SOL	905	7.580	2.460	0.250	1.00	0.00
ATOM	4799	HW2	SOL	905	6.110	2.820	0.240	1.00	0.00
ATOM	4800	OW	SOL	906	2.290	31.700	31.170	1.00	0.00
ATOM	4801	HW1	SOL	906	1.780	32.490	30.990	1.00	0.00
ATOM	4802	HW2	SOL	906	1.800	30.990	30.730	1.00	0.00
ATOM	4803	OW	SOL	907	36.920	15.940	4.660	1.00	0.00
ATOM	4804	HW1	SOL	907	36.470	15.600	5.430	1.00	0.00
ATOM	4805	HW2	SOL	907	37.850	15.770	4.820	1.00	0.00
ATOM	4806	OW	SOL	908	3.090	29.630	2.170	1.00	0.00
ATOM	4807	HW1	SOL	908	2.850	30.300	2.810	1.00	0.00
ATOM	4808	HW2	SOL	908	3.970	29.360	2.430	1.00	0.00
ATOM	4809	OW	SOL	909	23.600	33.880	6.680	1.00	0.00
ATOM	4810	HW1	SOL	909	23.450	33.190	7.330	1.00	0.00
ATOM	4811	HW2	SOL	909	23.590	33.430	5.840	1.00	0.00
ATOM	4812	OW	SOL	910	26.240	22.420	35.910	1.00	0.00
ATOM	4813	HW1	SOL	910	26.180	21.810	36.650	1.00	0.00
ATOM	4814	HW2	SOL	910	27.180	22.590	35.820	1.00	0.00
ATOM	4815	OW	SOL	911	5.820	5.190	50.250	1.00	0.00
ATOM	4816	HW1	SOL	911	5.780	4.240	50.150	1.00	0.00
ATOM	4817	HW2	SOL	911	5.860	5.530	49.360	1.00	0.00
ATOM	4818	OW	SOL	912	33.320	27.280	11.810	1.00	0.00
ATOM	4819	HW1	SOL	912	33.940	28.010	11.800	1.00	0.00
ATOM	4820	HW2	SOL	912	32.570	27.600	11.310	1.00	0.00
ATOM	4821	OW	SOL	913	1.000	22.850	19.120	1.00	0.00
ATOM	4822	HW1	SOL	913	1.080	21.990	19.520	1.00	0.00
ATOM	4823	HW2	SOL	913	1.620	22.840	18.390	1.00	0.00
ATOM	4824	OW	SOL	914	10.370	45.800	43.540	1.00	0.00
ATOM	4825	HW1	SOL	914	10.560	45.780	42.610	1.00	0.00
ATOM	4826	HW2	SOL	914	10.450	46.720	43.780	1.00	0.00
ATOM	4827	OW	SOL	915	53.350	33.840	49.780	1.00	0.00
ATOM	4828	HW1	SOL	915	52.680	33.260	50.130	1.00	0.00
ATOM	4829	HW2	SOL	915	53.150	34.700	50.140	1.00	0.00
ATOM	4830	OW	SOL	916	27.720	36.210	1.880	1.00	0.00
ATOM	4831	HW1	SOL	916	27.600	35.450	1.300	1.00	0.00
ATOM	4832	HW2	SOL	916	28.230	35.880	2.610	1.00	0.00
ATOM	4833	OW	SOL	917	22.760	37.310	52.800	1.00	0.00
ATOM	4834	HW1	SOL	917	21.830	37.240	52.600	1.00	0.00
ATOM	4835	HW2	SOL	917	22.830	38.090	53.350	1.00	0.00
ATOM	4836	OW	SOL	918	38.930	38.340	15.920	1.00	0.00
ATOM	4837	HW1	SOL	918	39.080	39.280	15.820	1.00	0.00
ATOM	4838	HW2	SOL	918	39.630	37.930	15.410	1.00	0.00
ATOM	4839	OW	SOL	919	53.210	55.010	23.510	1.00	0.00

ATOM	4840	HW1	SOL	919	52.420	54.550	23.790	1.00	0.00
ATOM	4841	HW2	SOL	919	53.430	55.600	24.230	1.00	0.00
ATOM	4842	OW	SOL	920	55.010	45.870	42.440	1.00	0.00
ATOM	4843	HW1	SOL	920	55.790	45.320	42.400	1.00	0.00
ATOM	4844	HW2	SOL	920	54.400	45.470	41.820	1.00	0.00
ATOM	4845	OW	SOL	921	24.590	33.220	0.100	1.00	0.00
ATOM	4846	HW1	SOL	921	25.050	32.520	-0.350	1.00	0.00
ATOM	4847	HW2	SOL	921	24.700	33.990	-0.460	1.00	0.00
ATOM	4848	OW	SOL	922	7.210	54.140	7.410	1.00	0.00
ATOM	4849	HW1	SOL	922	6.500	53.500	7.370	1.00	0.00
ATOM	4850	HW2	SOL	922	6.780	54.970	7.610	1.00	0.00
ATOM	4851	OW	SOL	923	4.100	22.600	50.710	1.00	0.00
ATOM	4852	HW1	SOL	923	3.560	21.890	50.370	1.00	0.00
ATOM	4853	HW2	SOL	923	4.870	22.150	51.080	1.00	0.00
ATOM	4854	OW	SOL	924	55.080	34.210	2.660	1.00	0.00
ATOM	4855	HW1	SOL	924	55.200	33.890	1.770	1.00	0.00
ATOM	4856	HW2	SOL	924	55.910	34.650	2.870	1.00	0.00
ATOM	4857	OW	SOL	925	35.660	14.900	6.870	1.00	0.00
ATOM	4858	HW1	SOL	925	34.900	15.360	7.220	1.00	0.00
ATOM	4859	HW2	SOL	925	35.730	14.110	7.420	1.00	0.00
ATOM	4860	OW	SOL	926	37.590	21.410	47.350	1.00	0.00
ATOM	4861	HW1	SOL	926	37.860	22.310	47.200	1.00	0.00
ATOM	4862	HW2	SOL	926	38.350	20.880	47.080	1.00	0.00
ATOM	4863	OW	SOL	927	52.190	28.780	30.390	1.00	0.00
ATOM	4864	HW1	SOL	927	52.510	28.900	31.280	1.00	0.00
ATOM	4865	HW2	SOL	927	52.280	29.640	29.980	1.00	0.00
ATOM	4866	OW	SOL	928	4.830	36.380	5.040	1.00	0.00
ATOM	4867	HW1	SOL	928	3.970	36.740	5.240	1.00	0.00
ATOM	4868	HW2	SOL	928	5.380	37.150	4.890	1.00	0.00
ATOM	4869	OW	SOL	929	34.310	13.520	4.930	1.00	0.00
ATOM	4870	HW1	SOL	929	33.690	14.010	4.390	1.00	0.00
ATOM	4871	HW2	SOL	929	34.820	14.180	5.390	1.00	0.00
ATOM	4872	OW	SOL	930	46.470	35.380	44.000	1.00	0.00
ATOM	4873	HW1	SOL	930	46.830	35.980	44.660	1.00	0.00
ATOM	4874	HW2	SOL	930	45.620	35.110	44.350	1.00	0.00
ATOM	4875	OW	SOL	931	53.560	0.580	2.230	1.00	0.00
ATOM	4876	HW1	SOL	931	53.060	0.850	1.460	1.00	0.00
ATOM	4877	HW2	SOL	931	53.250	-0.310	2.410	1.00	0.00
ATOM	4878	OW	SOL	932	38.000	19.730	26.230	1.00	0.00
ATOM	4879	HW1	SOL	932	38.520	19.030	26.620	1.00	0.00
ATOM	4880	HW2	SOL	932	37.220	19.300	25.890	1.00	0.00
ATOM	4881	OW	SOL	933	43.980	31.260	54.490	1.00	0.00
ATOM	4882	HW1	SOL	933	44.800	31.050	54.050	1.00	0.00
ATOM	4883	HW2	SOL	933	43.610	31.990	53.980	1.00	0.00

ATOM	4884	OW	SOL	934	6.250	38.920	50.650	1.00	0.00
ATOM	4885	HW1	SOL	934	6.650	39.180	49.820	1.00	0.00
ATOM	4886	HW2	SOL	934	6.920	38.400	51.090	1.00	0.00
ATOM	4887	OW	SOL	935	30.840	12.830	30.140	1.00	0.00
ATOM	4888	HW1	SOL	935	31.210	13.510	30.710	1.00	0.00
ATOM	4889	HW2	SOL	935	31.580	12.540	29.610	1.00	0.00
ATOM	4890	OW	SOL	936	44.390	8.260	53.010	1.00	0.00
ATOM	4891	HW1	SOL	936	44.690	9.050	53.450	1.00	0.00
ATOM	4892	HW2	SOL	936	43.470	8.430	52.820	1.00	0.00
ATOM	4893	OW	SOL	937	5.040	6.390	11.720	1.00	0.00
ATOM	4894	HW1	SOL	937	4.400	7.050	11.460	1.00	0.00
ATOM	4895	HW2	SOL	937	4.530	5.610	11.930	1.00	0.00
ATOM	4896	OW	SOL	938	48.280	15.870	10.780	1.00	0.00
ATOM	4897	HW1	SOL	938	48.490	16.140	11.670	1.00	0.00
ATOM	4898	HW2	SOL	938	47.330	15.760	10.790	1.00	0.00
ATOM	4899	OW	SOL	939	28.490	8.920	7.760	1.00	0.00
ATOM	4900	HW1	SOL	939	29.140	8.250	7.550	1.00	0.00
ATOM	4901	HW2	SOL	939	29.000	9.650	8.100	1.00	0.00
ATOM	4902	OW	SOL	940	34.350	9.870	33.690	1.00	0.00
ATOM	4903	HW1	SOL	940	34.860	9.100	33.430	1.00	0.00
ATOM	4904	HW2	SOL	940	34.310	9.830	34.640	1.00	0.00
ATOM	4905	OW	SOL	941	22.670	9.960	41.750	1.00	0.00
ATOM	4906	HW1	SOL	941	23.440	10.380	42.140	1.00	0.00
ATOM	4907	HW2	SOL	941	22.020	10.660	41.700	1.00	0.00
ATOM	4908	OW	SOL	942	12.860	43.640	4.500	1.00	0.00
ATOM	4909	HW1	SOL	942	12.140	43.910	3.930	1.00	0.00
ATOM	4910	HW2	SOL	942	13.250	44.460	4.810	1.00	0.00
ATOM	4911	OW	SOL	943	2.850	10.510	29.260	1.00	0.00
ATOM	4912	HW1	SOL	943	2.380	11.080	28.650	1.00	0.00
ATOM	4913	HW2	SOL	943	2.990	9.700	28.770	1.00	0.00
ATOM	4914	OW	SOL	944	12.490	29.350	12.120	1.00	0.00
ATOM	4915	HW1	SOL	944	11.700	29.200	11.610	1.00	0.00
ATOM	4916	HW2	SOL	944	13.050	29.870	11.550	1.00	0.00
ATOM	4917	OW	SOL	945	48.090	54.320	8.550	1.00	0.00
ATOM	4918	HW1	SOL	945	47.530	54.980	8.960	1.00	0.00
ATOM	4919	HW2	SOL	945	47.970	54.450	7.610	1.00	0.00
ATOM	4920	OW	SOL	946	10.680	40.850	20.210	1.00	0.00
ATOM	4921	HW1	SOL	946	11.030	41.650	19.830	1.00	0.00
ATOM	4922	HW2	SOL	946	9.750	41.040	20.350	1.00	0.00
ATOM	4923	OW	SOL	947	6.450	10.250	26.610	1.00	0.00
ATOM	4924	HW1	SOL	947	6.680	10.680	25.790	1.00	0.00
ATOM	4925	HW2	SOL	947	6.110	10.950	27.170	1.00	0.00
ATOM	4926	OW	SOL	948	55.000	15.780	55.520	1.00	0.00
ATOM	4927	HW1	SOL	948	54.600	14.920	55.390	1.00	0.00

ATOM	4928	HW2	SOL	948	54.430	16.390	55.050	1.00	0.00
ATOM	4929	OW	SOL	949	4.700	10.750	19.790	1.00	0.00
ATOM	4930	HW1	SOL	949	5.080	9.950	19.430	1.00	0.00
ATOM	4931	HW2	SOL	949	4.500	10.530	20.700	1.00	0.00
ATOM	4932	OW	SOL	950	0.150	12.590	11.990	1.00	0.00
ATOM	4933	HW1	SOL	950	-0.290	13.440	11.890	1.00	0.00
ATOM	4934	HW2	SOL	950	0.450	12.370	11.110	1.00	0.00
ATOM	4935	OW	SOL	951	17.890	16.350	1.550	1.00	0.00
ATOM	4936	HW1	SOL	951	17.220	15.740	1.240	1.00	0.00
ATOM	4937	HW2	SOL	951	18.660	16.150	1.020	1.00	0.00
ATOM	4938	OW	SOL	952	15.490	0.720	52.150	1.00	0.00
ATOM	4939	HW1	SOL	952	15.020	0.430	51.370	1.00	0.00
ATOM	4940	HW2	SOL	952	16.130	0.040	52.320	1.00	0.00
ATOM	4941	OW	SOL	953	8.400	1.170	45.190	1.00	0.00
ATOM	4942	HW1	SOL	953	8.090	1.560	46.000	1.00	0.00
ATOM	4943	HW2	SOL	953	8.140	1.790	44.510	1.00	0.00
ATOM	4944	OW	SOL	954	33.400	29.380	14.700	1.00	0.00
ATOM	4945	HW1	SOL	954	33.200	29.020	15.560	1.00	0.00
ATOM	4946	HW2	SOL	954	34.020	28.760	14.310	1.00	0.00
ATOM	4947	OW	SOL	955	1.650	33.570	10.470	1.00	0.00
ATOM	4948	HW1	SOL	955	2.100	34.170	11.070	1.00	0.00
ATOM	4949	HW2	SOL	955	1.050	34.140	9.970	1.00	0.00
ATOM	4950	OW	SOL	956	13.930	16.430	54.390	1.00	0.00
ATOM	4951	HW1	SOL	956	13.020	16.680	54.550	1.00	0.00
ATOM	4952	HW2	SOL	956	14.290	17.160	53.890	1.00	0.00
ATOM	4953	OW	SOL	957	12.480	31.390	0.730	1.00	0.00
ATOM	4954	HW1	SOL	957	12.620	31.460	1.670	1.00	0.00
ATOM	4955	HW2	SOL	957	12.270	30.470	0.590	1.00	0.00
ATOM	4956	OW	SOL	958	32.150	24.380	23.940	1.00	0.00
ATOM	4957	HW1	SOL	958	31.630	23.600	23.780	1.00	0.00
ATOM	4958	HW2	SOL	958	32.660	24.490	23.140	1.00	0.00
ATOM	4959	OW	SOL	959	1.090	36.090	21.320	1.00	0.00
ATOM	4960	HW1	SOL	959	1.450	35.320	21.760	1.00	0.00
ATOM	4961	HW2	SOL	959	1.180	36.790	21.960	1.00	0.00
ATOM	4962	OW	SOL	960	14.210	36.060	40.800	1.00	0.00
ATOM	4963	HW1	SOL	960	13.420	35.520	40.810	1.00	0.00
ATOM	4964	HW2	SOL	960	13.890	36.950	40.720	1.00	0.00
ATOM	4965	OW	SOL	961	33.170	14.350	42.810	1.00	0.00
ATOM	4966	HW1	SOL	961	32.660	13.880	42.150	1.00	0.00
ATOM	4967	HW2	SOL	961	33.980	14.570	42.370	1.00	0.00
ATOM	4968	OW	SOL	962	43.740	6.810	25.430	1.00	0.00
ATOM	4969	HW1	SOL	962	43.590	6.850	24.480	1.00	0.00
ATOM	4970	HW2	SOL	962	44.660	6.560	25.510	1.00	0.00
ATOM	4971	OW	SOL	963	29.030	28.810	23.270	1.00	0.00

ATOM	4972	HW1	SOL	963	29.870	28.930	23.700	1.00	0.00
ATOM	4973	HW2	SOL	963	28.840	29.660	22.880	1.00	0.00
ATOM	4974	OW	SOL	964	18.000	11.080	19.950	1.00	0.00
ATOM	4975	HW1	SOL	964	18.640	11.690	20.310	1.00	0.00
ATOM	4976	HW2	SOL	964	17.320	11.640	19.570	1.00	0.00
ATOM	4977	OW	SOL	965	32.190	53.390	21.020	1.00	0.00
ATOM	4978	HW1	SOL	965	31.470	52.830	20.720	1.00	0.00
ATOM	4979	HW2	SOL	965	32.870	53.280	20.360	1.00	0.00
ATOM	4980	OW	SOL	966	16.830	22.070	27.040	1.00	0.00
ATOM	4981	HW1	SOL	966	15.990	22.270	27.450	1.00	0.00
ATOM	4982	HW2	SOL	966	16.660	21.280	26.520	1.00	0.00
ATOM	4983	OW	SOL	967	29.140	27.140	52.250	1.00	0.00
ATOM	4984	HW1	SOL	967	29.030	27.720	51.500	1.00	0.00
ATOM	4985	HW2	SOL	967	28.380	27.310	52.810	1.00	0.00
ATOM	4986	OW	SOL	968	14.630	31.690	11.040	1.00	0.00
ATOM	4987	HW1	SOL	968	14.800	32.270	11.780	1.00	0.00
ATOM	4988	HW2	SOL	968	13.680	31.660	10.960	1.00	0.00
ATOM	4989	OW	SOL	969	2.960	0.350	6.620	1.00	0.00
ATOM	4990	HW1	SOL	969	2.970	-0.600	6.750	1.00	0.00
ATOM	4991	HW2	SOL	969	3.730	0.660	7.100	1.00	0.00
ATOM	4992	OW	SOL	970	31.870	21.460	48.030	1.00	0.00
ATOM	4993	HW1	SOL	970	31.620	21.980	47.260	1.00	0.00
ATOM	4994	HW2	SOL	970	32.780	21.200	47.860	1.00	0.00
ATOM	4995	OW	SOL	971	50.760	44.230	35.300	1.00	0.00
ATOM	4996	HW1	SOL	971	50.130	44.730	34.790	1.00	0.00
ATOM	4997	HW2	SOL	971	51.450	44.000	34.680	1.00	0.00
ATOM	4998	OW	SOL	972	23.770	19.050	44.280	1.00	0.00
ATOM	4999	HW1	SOL	972	24.610	18.700	44.000	1.00	0.00
ATOM	5000	HW2	SOL	972	23.700	19.900	43.850	1.00	0.00
ATOM	5001	OW	SOL	973	16.040	1.550	37.860	1.00	0.00
ATOM	5002	HW1	SOL	973	15.940	1.110	38.700	1.00	0.00
ATOM	5003	HW2	SOL	973	16.660	2.260	38.040	1.00	0.00
ATOM	5004	OW	SOL	974	3.040	46.030	14.840	1.00	0.00
ATOM	5005	HW1	SOL	974	2.600	45.640	14.090	1.00	0.00
ATOM	5006	HW2	SOL	974	3.890	46.310	14.510	1.00	0.00
ATOM	5007	OW	SOL	975	2.780	3.190	27.990	1.00	0.00
ATOM	5008	HW1	SOL	975	2.900	4.130	27.830	1.00	0.00
ATOM	5009	HW2	SOL	975	2.710	2.810	27.110	1.00	0.00
ATOM	5010	OW	SOL	976	45.220	43.810	4.140	1.00	0.00
ATOM	5011	HW1	SOL	976	45.640	42.960	4.200	1.00	0.00
ATOM	5012	HW2	SOL	976	44.290	43.610	4.030	1.00	0.00
ATOM	5013	OW	SOL	977	6.840	45.580	21.780	1.00	0.00
ATOM	5014	HW1	SOL	977	6.530	46.480	21.660	1.00	0.00
ATOM	5015	HW2	SOL	977	7.350	45.400	20.990	1.00	0.00

ATOM	5016	OW	SOL	978	3.290	4.190	33.070	1.00	0.00
ATOM	5017	HW1	SOL	978	2.660	3.900	32.410	1.00	0.00
ATOM	5018	HW2	SOL	978	4.060	3.640	32.930	1.00	0.00
ATOM	5019	OW	SOL	979	41.700	13.600	23.990	1.00	0.00
ATOM	5020	HW1	SOL	979	42.210	13.040	24.560	1.00	0.00
ATOM	5021	HW2	SOL	979	40.850	13.670	24.420	1.00	0.00
ATOM	5022	OW	SOL	980	36.450	25.750	50.470	1.00	0.00
ATOM	5023	HW1	SOL	980	36.560	26.460	49.840	1.00	0.00
ATOM	5024	HW2	SOL	980	35.990	26.150	51.200	1.00	0.00
ATOM	5025	OW	SOL	981	10.270	17.730	48.880	1.00	0.00
ATOM	5026	HW1	SOL	981	11.130	18.140	48.950	1.00	0.00
ATOM	5027	HW2	SOL	981	9.670	18.460	48.790	1.00	0.00
ATOM	5028	OW	SOL	982	20.690	45.440	43.340	1.00	0.00
ATOM	5029	HW1	SOL	982	21.080	46.290	43.530	1.00	0.00
ATOM	5030	HW2	SOL	982	20.840	44.920	44.130	1.00	0.00
ATOM	5031	OW	SOL	983	20.310	42.450	4.120	1.00	0.00
ATOM	5032	HW1	SOL	983	20.340	42.040	3.250	1.00	0.00
ATOM	5033	HW2	SOL	983	20.900	41.910	4.650	1.00	0.00
ATOM	5034	OW	SOL	984	17.960	46.140	1.090	1.00	0.00
ATOM	5035	HW1	SOL	984	18.410	45.350	0.820	1.00	0.00
ATOM	5036	HW2	SOL	984	17.630	45.950	1.960	1.00	0.00
ATOM	5037	OW	SOL	985	20.310	41.090	1.700	1.00	0.00
ATOM	5038	HW1	SOL	985	21.150	41.540	1.560	1.00	0.00
ATOM	5039	HW2	SOL	985	20.410	40.270	1.220	1.00	0.00
ATOM	5040	OW	SOL	986	49.310	9.680	7.760	1.00	0.00
ATOM	5041	HW1	SOL	986	49.340	8.930	7.160	1.00	0.00
ATOM	5042	HW2	SOL	986	50.020	10.250	7.470	1.00	0.00
ATOM	5043	OW	SOL	987	25.850	52.410	5.760	1.00	0.00
ATOM	5044	HW1	SOL	987	25.630	51.660	6.310	1.00	0.00
ATOM	5045	HW2	SOL	987	26.630	52.140	5.280	1.00	0.00
ATOM	5046	OW	SOL	988	42.280	1.310	15.370	1.00	0.00
ATOM	5047	HW1	SOL	988	42.350	2.070	15.950	1.00	0.00
ATOM	5048	HW2	SOL	988	42.760	1.560	14.580	1.00	0.00
ATOM	5049	OW	SOL	989	42.790	10.210	21.930	1.00	0.00
ATOM	5050	HW1	SOL	989	43.080	9.950	21.060	1.00	0.00
ATOM	5051	HW2	SOL	989	42.410	11.080	21.800	1.00	0.00
ATOM	5052	OW	SOL	990	5.280	10.660	43.860	1.00	0.00
ATOM	5053	HW1	SOL	990	6.220	10.870	43.830	1.00	0.00
ATOM	5054	HW2	SOL	990	5.100	10.510	44.780	1.00	0.00
ATOM	5055	OW	SOL	991	17.700	30.620	28.520	1.00	0.00
ATOM	5056	HW1	SOL	991	17.630	31.540	28.790	1.00	0.00
ATOM	5057	HW2	SOL	991	17.130	30.560	27.750	1.00	0.00
ATOM	5058	OW	SOL	992	4.410	28.100	9.130	1.00	0.00
ATOM	5059	HW1	SOL	992	3.740	28.220	9.810	1.00	0.00

ATOM	5060	HW2	SOL	992	4.520	28.970	8.740	1.00	0.00
ATOM	5061	OW	SOL	993	38.440	9.260	51.240	1.00	0.00
ATOM	5062	HW1	SOL	993	37.690	8.660	51.270	1.00	0.00
ATOM	5063	HW2	SOL	993	38.380	9.760	52.050	1.00	0.00
ATOM	5064	OW	SOL	994	42.440	14.330	19.760	1.00	0.00
ATOM	5065	HW1	SOL	994	43.400	14.360	19.710	1.00	0.00
ATOM	5066	HW2	SOL	994	42.180	15.230	19.940	1.00	0.00
ATOM	5067	OW	SOL	995	46.790	34.440	5.570	1.00	0.00
ATOM	5068	HW1	SOL	995	46.920	34.970	4.780	1.00	0.00
ATOM	5069	HW2	SOL	995	46.140	34.920	6.080	1.00	0.00
ATOM	5070	OW	SOL	996	6.430	4.850	27.810	1.00	0.00
ATOM	5071	HW1	SOL	996	7.370	5.040	27.830	1.00	0.00
ATOM	5072	HW2	SOL	996	6.220	4.780	26.880	1.00	0.00
ATOM	5073	OW	SOL	997	53.580	30.140	49.270	1.00	0.00
ATOM	5074	HW1	SOL	997	52.700	30.110	48.910	1.00	0.00
ATOM	5075	HW2	SOL	997	54.140	30.400	48.540	1.00	0.00
ATOM	5076	OW	SOL	998	10.560	38.280	19.430	1.00	0.00
ATOM	5077	HW1	SOL	998	9.670	38.000	19.630	1.00	0.00
ATOM	5078	HW2	SOL	998	10.620	39.160	19.790	1.00	0.00
ATOM	5079	OW	SOL	999	28.240	14.690	53.040	1.00	0.00
ATOM	5080	HW1	SOL	999	27.610	14.950	52.360	1.00	0.00
ATOM	5081	HW2	SOL	999	28.990	14.350	52.550	1.00	0.00
ATOM	5082	OW	SOL	1000	30.880	44.440	20.530	1.00	0.00
ATOM	5083	HW1	SOL	1000	31.070	43.580	20.910	1.00	0.00
ATOM	5084	HW2	SOL	1000	31.380	45.050	21.080	1.00	0.00
ATOM	5085	OW	SOL	1001	23.500	13.910	40.430	1.00	0.00
ATOM	5086	HW1	SOL	1001	24.190	13.480	39.930	1.00	0.00
ATOM	5087	HW2	SOL	1001	23.420	14.780	40.020	1.00	0.00
ATOM	5088	OW	SOL	1002	23.070	2.830	55.060	1.00	0.00
ATOM	5089	HW1	SOL	1002	23.080	2.050	54.500	1.00	0.00
ATOM	5090	HW2	SOL	1002	22.870	2.490	55.930	1.00	0.00
ATOM	5091	OW	SOL	1003	39.630	15.480	4.480	1.00	0.00
ATOM	5092	HW1	SOL	1003	39.820	14.550	4.360	1.00	0.00
ATOM	5093	HW2	SOL	1003	39.860	15.880	3.630	1.00	0.00
ATOM	5094	OW	SOL	1004	37.990	3.860	21.240	1.00	0.00
ATOM	5095	HW1	SOL	1004	38.160	4.040	22.170	1.00	0.00
ATOM	5096	HW2	SOL	1004	37.180	3.340	21.240	1.00	0.00
ATOM	5097	OW	SOL	1005	13.620	32.900	8.390	1.00	0.00
ATOM	5098	HW1	SOL	1005	13.390	33.830	8.400	1.00	0.00
ATOM	5099	HW2	SOL	1005	13.240	32.550	9.190	1.00	0.00
ATOM	5100	OW	SOL	1006	29.920	32.090	15.550	1.00	0.00
ATOM	5101	HW1	SOL	1006	29.510	31.290	15.880	1.00	0.00
ATOM	5102	HW2	SOL	1006	29.350	32.790	15.860	1.00	0.00
ATOM	5103	OW	SOL	1007	29.980	0.140	32.160	1.00	0.00

ATOM	5104	HW1	SOL	1007	29.610	0.550	32.950	1.00	0.00
ATOM	5105	HW2	SOL	1007	29.220	-0.160	31.670	1.00	0.00
ATOM	5106	OW	SOL	1008	10.040	23.860	30.640	1.00	0.00
ATOM	5107	HW1	SOL	1008	9.690	24.670	31.020	1.00	0.00
ATOM	5108	HW2	SOL	1008	10.980	23.890	30.830	1.00	0.00
ATOM	5109	OW	SOL	1009	17.290	27.660	12.750	1.00	0.00
ATOM	5110	HW1	SOL	1009	18.070	27.850	13.280	1.00	0.00
ATOM	5111	HW2	SOL	1009	17.570	26.980	12.140	1.00	0.00
ATOM	5112	OW	SOL	1010	47.320	47.150	55.540	1.00	0.00
ATOM	5113	HW1	SOL	1010	47.450	48.100	55.540	1.00	0.00
ATOM	5114	HW2	SOL	1010	46.370	47.030	55.470	1.00	0.00
ATOM	5115	OW	SOL	1011	2.900	44.620	45.340	1.00	0.00
ATOM	5116	HW1	SOL	1011	3.660	44.230	45.770	1.00	0.00
ATOM	5117	HW2	SOL	1011	2.190	44.000	45.510	1.00	0.00
ATOM	5118	OW	SOL	1012	45.990	12.420	28.290	1.00	0.00
ATOM	5119	HW1	SOL	1012	46.440	12.850	27.560	1.00	0.00
ATOM	5120	HW2	SOL	1012	45.060	12.580	28.120	1.00	0.00
ATOM	5121	OW	SOL	1013	13.180	4.150	32.460	1.00	0.00
ATOM	5122	HW1	SOL	1013	13.520	5.000	32.720	1.00	0.00
ATOM	5123	HW2	SOL	1013	13.960	3.620	32.290	1.00	0.00
ATOM	5124	OW	SOL	1014	21.760	47.510	1.590	1.00	0.00
ATOM	5125	HW1	SOL	1014	20.840	47.760	1.490	1.00	0.00
ATOM	5126	HW2	SOL	1014	21.730	46.600	1.900	1.00	0.00
ATOM	5127	OW	SOL	1015	21.560	15.320	26.920	1.00	0.00
ATOM	5128	HW1	SOL	1015	21.580	15.200	25.970	1.00	0.00
ATOM	5129	HW2	SOL	1015	20.940	14.650	27.220	1.00	0.00
ATOM	5130	OW	SOL	1016	53.740	26.800	22.890	1.00	0.00
ATOM	5131	HW1	SOL	1016	53.500	27.630	22.490	1.00	0.00
ATOM	5132	HW2	SOL	1016	53.590	26.150	22.210	1.00	0.00
ATOM	5133	OW	SOL	1017	28.940	42.110	2.670	1.00	0.00
ATOM	5134	HW1	SOL	1017	29.640	41.880	2.070	1.00	0.00
ATOM	5135	HW2	SOL	1017	28.720	43.010	2.450	1.00	0.00
ATOM	5136	OW	SOL	1018	15.350	49.720	13.340	1.00	0.00
ATOM	5137	HW1	SOL	1018	15.240	49.580	12.400	1.00	0.00
ATOM	5138	HW2	SOL	1018	15.200	50.660	13.470	1.00	0.00
ATOM	5139	OW	SOL	1019	39.340	33.340	2.300	1.00	0.00
ATOM	5140	HW1	SOL	1019	39.200	32.790	3.070	1.00	0.00
ATOM	5141	HW2	SOL	1019	39.020	32.810	1.560	1.00	0.00
ATOM	5142	OW	SOL	1020	55.180	48.130	12.080	1.00	0.00
ATOM	5143	HW1	SOL	1020	55.380	47.230	12.330	1.00	0.00
ATOM	5144	HW2	SOL	1020	54.280	48.100	11.770	1.00	0.00
ATOM	5145	OW	SOL	1021	8.210	44.270	36.790	1.00	0.00
ATOM	5146	HW1	SOL	1021	8.090	44.330	35.840	1.00	0.00
ATOM	5147	HW2	SOL	1021	8.560	43.390	36.930	1.00	0.00

ATOM	5148	OW	SOL	1022	44.620	17.310	27.220	1.00	0.00
ATOM	5149	HW1	SOL	1022	45.200	17.200	26.460	1.00	0.00
ATOM	5150	HW2	SOL	1022	44.850	18.160	27.580	1.00	0.00
ATOM	5151	OW	SOL	1023	15.600	49.710	2.620	1.00	0.00
ATOM	5152	HW1	SOL	1023	16.420	49.790	3.100	1.00	0.00
ATOM	5153	HW2	SOL	1023	15.500	50.550	2.180	1.00	0.00
ATOM	5154	OW	SOL	1024	2.240	23.480	15.530	1.00	0.00
ATOM	5155	HW1	SOL	1024	2.180	24.440	15.590	1.00	0.00
ATOM	5156	HW2	SOL	1024	1.480	23.240	14.990	1.00	0.00
ATOM	5157	OW	SOL	1025	46.240	5.150	47.180	1.00	0.00
ATOM	5158	HW1	SOL	1025	45.670	5.020	47.940	1.00	0.00
ATOM	5159	HW2	SOL	1025	45.870	4.590	46.500	1.00	0.00
ATOM	5160	OW	SOL	1026	49.350	48.890	28.600	1.00	0.00
ATOM	5161	HW1	SOL	1026	49.210	49.420	29.380	1.00	0.00
ATOM	5162	HW2	SOL	1026	49.690	48.060	28.920	1.00	0.00
ATOM	5163	OW	SOL	1027	5.230	34.650	0.770	1.00	0.00
ATOM	5164	HW1	SOL	1027	4.440	34.140	0.570	1.00	0.00
ATOM	5165	HW2	SOL	1027	5.900	34.280	0.190	1.00	0.00
ATOM	5166	OW	SOL	1028	1.240	52.710	11.600	1.00	0.00
ATOM	5167	HW1	SOL	1028	0.720	53.340	12.110	1.00	0.00
ATOM	5168	HW2	SOL	1028	1.880	52.370	12.220	1.00	0.00
ATOM	5169	OW	SOL	1029	33.190	35.560	50.030	1.00	0.00
ATOM	5170	HW1	SOL	1029	32.700	36.390	49.990	1.00	0.00
ATOM	5171	HW2	SOL	1029	33.940	35.710	49.460	1.00	0.00
ATOM	5172	OW	SOL	1030	30.030	51.170	51.920	1.00	0.00
ATOM	5173	HW1	SOL	1030	29.170	50.900	51.610	1.00	0.00
ATOM	5174	HW2	SOL	1030	30.280	50.490	52.550	1.00	0.00
ATOM	5175	OW	SOL	1031	6.240	23.430	39.980	1.00	0.00
ATOM	5176	HW1	SOL	1031	6.120	23.170	39.060	1.00	0.00
ATOM	5177	HW2	SOL	1031	5.560	22.950	40.450	1.00	0.00
ATOM	5178	OW	SOL	1032	10.600	17.050	18.630	1.00	0.00
ATOM	5179	HW1	SOL	1032	10.300	17.650	17.940	1.00	0.00
ATOM	5180	HW2	SOL	1032	11.370	16.620	18.260	1.00	0.00
ATOM	5181	OW	SOL	1033	25.310	30.910	25.830	1.00	0.00
ATOM	5182	HW1	SOL	1033	24.770	31.350	26.490	1.00	0.00
ATOM	5183	HW2	SOL	1033	25.280	31.490	25.070	1.00	0.00
ATOM	5184	OW	SOL	1034	38.000	21.320	36.770	1.00	0.00
ATOM	5185	HW1	SOL	1034	38.150	22.250	36.890	1.00	0.00
ATOM	5186	HW2	SOL	1034	38.780	21.000	36.330	1.00	0.00
ATOM	5187	OW	SOL	1035	45.090	27.520	55.330	1.00	0.00
ATOM	5188	HW1	SOL	1035	45.320	27.570	54.400	1.00	0.00
ATOM	5189	HW2	SOL	1035	44.220	27.930	55.380	1.00	0.00
ATOM	5190	OW	SOL	1036	23.420	5.400	4.840	1.00	0.00
ATOM	5191	HW1	SOL	1036	23.480	5.360	3.880	1.00	0.00

ATOM	5192	HW2	SOL	1036	24.150	5.960	5.100	1.00	0.00
ATOM	5193	OW	SOL	1037	36.630	38.130	50.760	1.00	0.00
ATOM	5194	HW1	SOL	1037	37.110	37.890	51.550	1.00	0.00
ATOM	5195	HW2	SOL	1037	35.840	38.580	51.070	1.00	0.00
ATOM	5196	OW	SOL	1038	49.520	26.410	27.260	1.00	0.00
ATOM	5197	HW1	SOL	1038	49.100	27.110	26.760	1.00	0.00
ATOM	5198	HW2	SOL	1038	48.810	25.800	27.480	1.00	0.00
ATOM	5199	OW	SOL	1039	35.420	2.730	54.410	1.00	0.00
ATOM	5200	HW1	SOL	1039	35.470	1.780	54.540	1.00	0.00
ATOM	5201	HW2	SOL	1039	36.300	2.970	54.110	1.00	0.00
ATOM	5202	OW	SOL	1040	18.930	27.660	40.380	1.00	0.00
ATOM	5203	HW1	SOL	1040	19.560	28.260	39.990	1.00	0.00
ATOM	5204	HW2	SOL	1040	18.130	27.780	39.860	1.00	0.00
ATOM	5205	OW	SOL	1041	18.060	25.320	46.880	1.00	0.00
ATOM	5206	HW1	SOL	1041	17.280	25.440	47.430	1.00	0.00
ATOM	5207	HW2	SOL	1041	17.710	25.130	46.010	1.00	0.00
ATOM	5208	OW	SOL	1042	50.040	15.600	2.890	1.00	0.00
ATOM	5209	HW1	SOL	1042	49.210	15.170	2.690	1.00	0.00
ATOM	5210	HW2	SOL	1042	49.960	16.470	2.480	1.00	0.00
ATOM	5211	OW	SOL	1043	33.730	20.140	27.650	1.00	0.00
ATOM	5212	HW1	SOL	1043	33.870	21.020	27.990	1.00	0.00
ATOM	5213	HW2	SOL	1043	32.870	20.180	27.230	1.00	0.00
ATOM	5214	OW	SOL	1044	21.820	15.880	32.690	1.00	0.00
ATOM	5215	HW1	SOL	1044	22.420	15.300	32.200	1.00	0.00
ATOM	5216	HW2	SOL	1044	21.350	15.290	33.280	1.00	0.00
ATOM	5217	OW	SOL	1045	18.560	11.060	53.050	1.00	0.00
ATOM	5218	HW1	SOL	1045	17.720	10.640	52.870	1.00	0.00
ATOM	5219	HW2	SOL	1045	19.180	10.340	53.140	1.00	0.00
ATOM	5220	OW	SOL	1046	29.310	49.460	15.490	1.00	0.00
ATOM	5221	HW1	SOL	1046	29.340	49.300	14.550	1.00	0.00
ATOM	5222	HW2	SOL	1046	30.120	49.060	15.820	1.00	0.00
ATOM	5223	OW	SOL	1047	34.270	31.710	38.700	1.00	0.00
ATOM	5224	HW1	SOL	1047	34.370	30.850	39.100	1.00	0.00
ATOM	5225	HW2	SOL	1047	34.770	32.300	39.270	1.00	0.00
ATOM	5226	OW	SOL	1048	20.180	25.110	7.120	1.00	0.00
ATOM	5227	HW1	SOL	1048	20.510	25.990	6.940	1.00	0.00
ATOM	5228	HW2	SOL	1048	19.640	24.900	6.360	1.00	0.00
ATOM	5229	OW	SOL	1049	0.360	10.860	49.040	1.00	0.00
ATOM	5230	HW1	SOL	1049	0.640	10.670	48.140	1.00	0.00
ATOM	5231	HW2	SOL	1049	0.920	11.580	49.320	1.00	0.00
ATOM	5232	OW	SOL	1050	24.100	19.850	10.820	1.00	0.00
ATOM	5233	HW1	SOL	1050	23.150	19.960	10.870	1.00	0.00
ATOM	5234	HW2	SOL	1050	24.290	19.130	11.430	1.00	0.00
ATOM	5235	OW	SOL	1051	55.530	40.300	30.420	1.00	0.00

ATOM	5236	HW1	SOL	1051	56.360	39.840	30.530	1.00	0.00
ATOM	5237	HW2	SOL	1051	55.630	41.110	30.920	1.00	0.00
ATOM	5238	OW	SOL	1052	49.750	53.810	47.390	1.00	0.00
ATOM	5239	HW1	SOL	1052	49.970	54.330	48.170	1.00	0.00
ATOM	5240	HW2	SOL	1052	50.580	53.420	47.120	1.00	0.00
ATOM	5241	OW	SOL	1053	46.180	38.630	3.980	1.00	0.00
ATOM	5242	HW1	SOL	1053	46.950	38.470	4.530	1.00	0.00
ATOM	5243	HW2	SOL	1053	46.440	38.350	3.110	1.00	0.00
ATOM	5244	OW	SOL	1054	48.880	38.390	29.230	1.00	0.00
ATOM	5245	HW1	SOL	1054	49.740	38.120	28.910	1.00	0.00
ATOM	5246	HW2	SOL	1054	48.470	37.580	29.520	1.00	0.00
ATOM	5247	OW	SOL	1055	47.900	24.190	27.410	1.00	0.00
ATOM	5248	HW1	SOL	1055	48.070	23.380	26.920	1.00	0.00
ATOM	5249	HW2	SOL	1055	46.970	24.150	27.630	1.00	0.00
ATOM	5250	OW	SOL	1056	35.770	39.680	3.870	1.00	0.00
ATOM	5251	HW1	SOL	1056	35.850	38.750	3.650	1.00	0.00
ATOM	5252	HW2	SOL	1056	34.940	39.740	4.340	1.00	0.00
ATOM	5253	OW	SOL	1057	40.290	22.200	14.010	1.00	0.00
ATOM	5254	HW1	SOL	1057	39.460	22.240	13.530	1.00	0.00
ATOM	5255	HW2	SOL	1057	40.040	22.170	14.930	1.00	0.00
ATOM	5256	OW	SOL	1058	13.370	2.280	48.210	1.00	0.00
ATOM	5257	HW1	SOL	1058	13.300	2.120	47.270	1.00	0.00
ATOM	5258	HW2	SOL	1058	12.730	2.980	48.380	1.00	0.00
ATOM	5259	OW	SOL	1059	35.330	54.830	50.930	1.00	0.00
ATOM	5260	HW1	SOL	1059	34.940	55.320	50.210	1.00	0.00
ATOM	5261	HW2	SOL	1059	35.300	55.440	51.670	1.00	0.00
ATOM	5262	OW	SOL	1060	28.690	20.290	10.770	1.00	0.00
ATOM	5263	HW1	SOL	1060	28.410	20.560	11.650	1.00	0.00
ATOM	5264	HW2	SOL	1060	29.620	20.520	10.730	1.00	0.00
ATOM	5265	OW	SOL	1061	30.180	29.450	13.690	1.00	0.00
ATOM	5266	HW1	SOL	1061	29.240	29.590	13.720	1.00	0.00
ATOM	5267	HW2	SOL	1061	30.480	29.600	14.590	1.00	0.00
ATOM	5268	OW	SOL	1062	48.210	42.110	35.740	1.00	0.00
ATOM	5269	HW1	SOL	1062	47.780	42.850	35.320	1.00	0.00
ATOM	5270	HW2	SOL	1062	48.950	41.900	35.170	1.00	0.00
ATOM	5271	OW	SOL	1063	19.230	17.040	8.320	1.00	0.00
ATOM	5272	HW1	SOL	1063	19.380	16.700	7.450	1.00	0.00
ATOM	5273	HW2	SOL	1063	20.030	16.850	8.810	1.00	0.00
ATOM	5274	OW	SOL	1064	48.040	42.610	32.450	1.00	0.00
ATOM	5275	HW1	SOL	1064	47.680	42.900	31.610	1.00	0.00
ATOM	5276	HW2	SOL	1064	48.840	42.150	32.230	1.00	0.00
ATOM	5277	OW	SOL	1065	11.190	1.790	39.000	1.00	0.00
ATOM	5278	HW1	SOL	1065	11.390	2.710	39.190	1.00	0.00
ATOM	5279	HW2	SOL	1065	10.560	1.830	38.270	1.00	0.00

ATOM	5280	OW	SOL	1066	50.370	0.950	6.710	1.00	0.00
ATOM	5281	HW1	SOL	1066	49.760	1.680	6.610	1.00	0.00
ATOM	5282	HW2	SOL	1066	51.220	1.300	6.420	1.00	0.00
ATOM	5283	OW	SOL	1067	45.760	28.410	12.010	1.00	0.00
ATOM	5284	HW1	SOL	1067	46.240	28.360	11.180	1.00	0.00
ATOM	5285	HW2	SOL	1067	46.410	28.680	12.650	1.00	0.00
ATOM	5286	OW	SOL	1068	41.580	12.150	37.720	1.00	0.00
ATOM	5287	HW1	SOL	1068	40.900	12.820	37.700	1.00	0.00
ATOM	5288	HW2	SOL	1068	41.240	11.450	37.170	1.00	0.00
ATOM	5289	OW	SOL	1069	45.890	3.730	30.500	1.00	0.00
ATOM	5290	HW1	SOL	1069	45.720	4.310	29.760	1.00	0.00
ATOM	5291	HW2	SOL	1069	46.330	2.970	30.120	1.00	0.00
ATOM	5292	OW	SOL	1070	42.940	37.310	15.330	1.00	0.00
ATOM	5293	HW1	SOL	1070	42.270	37.630	14.730	1.00	0.00
ATOM	5294	HW2	SOL	1070	43.480	36.730	14.800	1.00	0.00
ATOM	5295	OW	SOL	1071	44.640	35.440	13.980	1.00	0.00
ATOM	5296	HW1	SOL	1071	45.320	35.970	14.400	1.00	0.00
ATOM	5297	HW2	SOL	1071	44.700	34.590	14.400	1.00	0.00
ATOM	5298	OW	SOL	1072	26.850	20.620	4.940	1.00	0.00
ATOM	5299	HW1	SOL	1072	27.690	20.470	4.500	1.00	0.00
ATOM	5300	HW2	SOL	1072	26.540	21.450	4.580	1.00	0.00
ATOM	5301	OW	SOL	1073	18.080	14.570	27.220	1.00	0.00
ATOM	5302	HW1	SOL	1073	18.570	15.350	27.490	1.00	0.00
ATOM	5303	HW2	SOL	1073	18.450	13.860	27.740	1.00	0.00
ATOM	5304	OW	SOL	1074	16.370	46.010	6.350	1.00	0.00
ATOM	5305	HW1	SOL	1074	17.260	46.070	6.700	1.00	0.00
ATOM	5306	HW2	SOL	1074	16.480	45.670	5.460	1.00	0.00
ATOM	5307	OW	SOL	1075	10.710	8.310	41.490	1.00	0.00
ATOM	5308	HW1	SOL	1075	9.770	8.150	41.410	1.00	0.00
ATOM	5309	HW2	SOL	1075	11.120	7.560	41.060	1.00	0.00
ATOM	5310	OW	SOL	1076	11.620	52.130	27.560	1.00	0.00
ATOM	5311	HW1	SOL	1076	12.100	52.960	27.520	1.00	0.00
ATOM	5312	HW2	SOL	1076	10.710	52.380	27.740	1.00	0.00
ATOM	5313	OW	SOL	1077	23.790	14.040	20.120	1.00	0.00
ATOM	5314	HW1	SOL	1077	23.210	14.800	19.990	1.00	0.00
ATOM	5315	HW2	SOL	1077	23.200	13.290	20.070	1.00	0.00
ATOM	5316	OW	SOL	1078	25.150	38.930	0.140	1.00	0.00
ATOM	5317	HW1	SOL	1078	24.490	38.290	-0.100	1.00	0.00
ATOM	5318	HW2	SOL	1078	25.550	38.580	0.940	1.00	0.00
ATOM	5319	OW	SOL	1079	12.860	28.480	24.270	1.00	0.00
ATOM	5320	HW1	SOL	1079	13.770	28.670	24.050	1.00	0.00
ATOM	5321	HW2	SOL	1079	12.600	27.810	23.630	1.00	0.00
ATOM	5322	OW	SOL	1080	27.880	6.390	28.710	1.00	0.00
ATOM	5323	HW1	SOL	1080	28.120	6.920	29.470	1.00	0.00

ATOM	5324	HW2	SOL	1080	28.550	5.700	28.680	1.00	0.00
ATOM	5325	OW	SOL	1081	36.750	6.780	15.990	1.00	0.00
ATOM	5326	HW1	SOL	1081	36.460	6.170	16.660	1.00	0.00
ATOM	5327	HW2	SOL	1081	37.410	6.300	15.500	1.00	0.00
ATOM	5328	OW	SOL	1082	3.170	20.570	15.210	1.00	0.00
ATOM	5329	HW1	SOL	1082	2.340	20.980	14.960	1.00	0.00
ATOM	5330	HW2	SOL	1082	3.810	21.280	15.190	1.00	0.00
ATOM	5331	OW	SOL	1083	53.530	40.250	50.010	1.00	0.00
ATOM	5332	HW1	SOL	1083	53.630	41.090	50.460	1.00	0.00
ATOM	5333	HW2	SOL	1083	53.630	39.600	50.700	1.00	0.00
ATOM	5334	OW	SOL	1084	4.190	47.510	5.760	1.00	0.00
ATOM	5335	HW1	SOL	1084	3.490	46.880	5.640	1.00	0.00
ATOM	5336	HW2	SOL	1084	4.970	47.080	5.410	1.00	0.00
ATOM	5337	OW	SOL	1085	27.770	27.720	36.560	1.00	0.00
ATOM	5338	HW1	SOL	1085	27.270	27.900	35.770	1.00	0.00
ATOM	5339	HW2	SOL	1085	28.570	27.310	36.250	1.00	0.00
ATOM	5340	OW	SOL	1086	0.560	1.340	9.020	1.00	0.00
ATOM	5341	HW1	SOL	1086	-0.300	0.920	9.010	1.00	0.00
ATOM	5342	HW2	SOL	1086	1.100	0.800	8.440	1.00	0.00
ATOM	5343	OW	SOL	1087	49.820	32.810	23.540	1.00	0.00
ATOM	5344	HW1	SOL	1087	49.380	32.030	23.210	1.00	0.00
ATOM	5345	HW2	SOL	1087	50.700	32.780	23.150	1.00	0.00
ATOM	5346	OW	SOL	1088	24.640	49.460	54.250	1.00	0.00
ATOM	5347	HW1	SOL	1088	24.370	50.330	54.540	1.00	0.00
ATOM	5348	HW2	SOL	1088	23.840	48.950	54.220	1.00	0.00
ATOM	5349	OW	SOL	1089	31.680	1.560	49.200	1.00	0.00
ATOM	5350	HW1	SOL	1089	31.250	2.040	48.490	1.00	0.00
ATOM	5351	HW2	SOL	1089	31.060	1.620	49.930	1.00	0.00
ATOM	5352	OW	SOL	1090	8.200	51.280	42.570	1.00	0.00
ATOM	5353	HW1	SOL	1090	7.370	51.240	43.040	1.00	0.00
ATOM	5354	HW2	SOL	1090	8.860	51.080	43.240	1.00	0.00
ATOM	5355	OW	SOL	1091	13.950	21.960	44.860	1.00	0.00
ATOM	5356	HW1	SOL	1091	14.830	21.780	45.190	1.00	0.00
ATOM	5357	HW2	SOL	1091	13.370	21.780	45.600	1.00	0.00
ATOM	5358	OW	SOL	1092	29.610	36.290	54.740	1.00	0.00
ATOM	5359	HW1	SOL	1092	29.180	36.880	55.360	1.00	0.00
ATOM	5360	HW2	SOL	1092	29.590	35.430	55.170	1.00	0.00
ATOM	5361	OW	SOL	1093	25.040	14.940	25.830	1.00	0.00
ATOM	5362	HW1	SOL	1093	24.220	15.350	26.120	1.00	0.00
ATOM	5363	HW2	SOL	1093	24.800	14.030	25.650	1.00	0.00
ATOM	5364	OW	SOL	1094	13.670	35.940	52.420	1.00	0.00
ATOM	5365	HW1	SOL	1094	12.820	35.610	52.120	1.00	0.00
ATOM	5366	HW2	SOL	1094	13.520	36.860	52.630	1.00	0.00
ATOM	5367	OW	SOL	1095	13.930	5.750	22.150	1.00	0.00

ATOM	5368	HW1	SOL	1095	14.670	6.340	22.040	1.00	0.00
ATOM	5369	HW2	SOL	1095	14.300	4.970	22.570	1.00	0.00
ATOM	5370	OW	SOL	1096	43.570	10.450	12.880	1.00	0.00
ATOM	5371	HW1	SOL	1096	42.960	10.970	12.350	1.00	0.00
ATOM	5372	HW2	SOL	1096	44.000	9.870	12.250	1.00	0.00
ATOM	5373	OW	SOL	1097	19.240	14.480	40.550	1.00	0.00
ATOM	5374	HW1	SOL	1097	18.450	14.780	41.000	1.00	0.00
ATOM	5375	HW2	SOL	1097	19.130	14.770	39.650	1.00	0.00
ATOM	5376	OW	SOL	1098	38.080	35.400	15.860	1.00	0.00
ATOM	5377	HW1	SOL	1098	37.150	35.180	15.860	1.00	0.00
ATOM	5378	HW2	SOL	1098	38.110	36.340	15.690	1.00	0.00
ATOM	5379	OW	SOL	1099	50.270	2.120	2.420	1.00	0.00
ATOM	5380	HW1	SOL	1099	49.650	2.850	2.430	1.00	0.00
ATOM	5381	HW2	SOL	1099	50.690	2.150	3.280	1.00	0.00
ATOM	5382	OW	SOL	1100	40.570	0.760	10.860	1.00	0.00
ATOM	5383	HW1	SOL	1100	40.360	1.230	10.050	1.00	0.00
ATOM	5384	HW2	SOL	1100	39.720	0.590	11.260	1.00	0.00
ATOM	5385	OW	SOL	1101	1.060	30.270	15.810	1.00	0.00
ATOM	5386	HW1	SOL	1101	1.580	29.480	15.660	1.00	0.00
ATOM	5387	HW2	SOL	1101	1.460	30.690	16.570	1.00	0.00
ATOM	5388	OW	SOL	1102	6.150	21.140	52.410	1.00	0.00
ATOM	5389	HW1	SOL	1102	6.210	21.940	52.930	1.00	0.00
ATOM	5390	HW2	SOL	1102	5.470	20.620	52.840	1.00	0.00
ATOM	5391	OW	SOL	1103	7.500	54.190	35.320	1.00	0.00
ATOM	5392	HW1	SOL	1103	7.050	53.370	35.530	1.00	0.00
ATOM	5393	HW2	SOL	1103	8.410	54.040	35.590	1.00	0.00
ATOM	5394	OW	SOL	1104	47.910	51.660	49.570	1.00	0.00
ATOM	5395	HW1	SOL	1104	48.620	51.260	50.080	1.00	0.00
ATOM	5396	HW2	SOL	1104	48.150	52.590	49.530	1.00	0.00
ATOM	5397	OW	SOL	1105	20.350	52.440	48.150	1.00	0.00
ATOM	5398	HW1	SOL	1105	20.810	52.790	47.390	1.00	0.00
ATOM	5399	HW2	SOL	1105	19.830	53.180	48.470	1.00	0.00
ATOM	5400	OW	SOL	1106	50.660	18.000	37.630	1.00	0.00
ATOM	5401	HW1	SOL	1106	49.810	18.410	37.510	1.00	0.00
ATOM	5402	HW2	SOL	1106	51.160	18.630	38.160	1.00	0.00
ATOM	5403	OW	SOL	1107	19.760	55.340	7.320	1.00	0.00
ATOM	5404	HW1	SOL	1107	19.900	54.430	7.070	1.00	0.00
ATOM	5405	HW2	SOL	1107	19.620	55.800	6.490	1.00	0.00
ATOM	5406	OW	SOL	1108	2.040	18.000	28.840	1.00	0.00
ATOM	5407	HW1	SOL	1108	2.190	17.290	28.230	1.00	0.00
ATOM	5408	HW2	SOL	1108	2.840	18.520	28.800	1.00	0.00
ATOM	5409	OW	SOL	1109	15.420	33.370	44.690	1.00	0.00
ATOM	5410	HW1	SOL	1109	15.650	33.460	43.770	1.00	0.00
ATOM	5411	HW2	SOL	1109	15.590	34.230	45.070	1.00	0.00

ATOM	5412	OW	SOL	1110	44.730	43.050	30.270	1.00	0.00
ATOM	5413	HW1	SOL	1110	44.560	42.320	29.670	1.00	0.00
ATOM	5414	HW2	SOL	1110	45.620	43.340	30.050	1.00	0.00
ATOM	5415	OW	SOL	1111	30.190	47.080	52.990	1.00	0.00
ATOM	5416	HW1	SOL	1111	30.130	47.180	52.040	1.00	0.00
ATOM	5417	HW2	SOL	1111	30.440	47.950	53.300	1.00	0.00
ATOM	5418	OW	SOL	1112	11.690	44.350	45.300	1.00	0.00
ATOM	5419	HW1	SOL	1112	11.010	44.780	44.780	1.00	0.00
ATOM	5420	HW2	SOL	1112	12.250	45.060	45.600	1.00	0.00
ATOM	5421	OW	SOL	1113	36.690	49.650	7.000	1.00	0.00
ATOM	5422	HW1	SOL	1113	35.990	50.300	6.910	1.00	0.00
ATOM	5423	HW2	SOL	1113	36.230	48.820	7.140	1.00	0.00
ATOM	5424	OW	SOL	1114	16.110	2.800	26.150	1.00	0.00
ATOM	5425	HW1	SOL	1114	16.940	2.950	26.610	1.00	0.00
ATOM	5426	HW2	SOL	1114	15.690	3.660	26.120	1.00	0.00
ATOM	5427	OW	SOL	1115	51.450	25.680	24.240	1.00	0.00
ATOM	5428	HW1	SOL	1115	52.330	25.990	24.020	1.00	0.00
ATOM	5429	HW2	SOL	1115	51.080	26.370	24.790	1.00	0.00
ATOM	5430	OW	SOL	1116	11.730	3.690	45.230	1.00	0.00
ATOM	5431	HW1	SOL	1116	11.700	3.110	44.470	1.00	0.00
ATOM	5432	HW2	SOL	1116	10.810	3.830	45.450	1.00	0.00
ATOM	5433	OW	SOL	1117	31.820	48.460	46.730	1.00	0.00
ATOM	5434	HW1	SOL	1117	32.590	47.920	46.910	1.00	0.00
ATOM	5435	HW2	SOL	1117	32.100	49.060	46.050	1.00	0.00
ATOM	5436	OW	SOL	1118	42.900	34.690	21.030	1.00	0.00
ATOM	5437	HW1	SOL	1118	43.650	34.880	21.600	1.00	0.00
ATOM	5438	HW2	SOL	1118	42.230	34.350	21.610	1.00	0.00
ATOM	5439	OW	SOL	1119	26.860	11.580	11.510	1.00	0.00
ATOM	5440	HW1	SOL	1119	26.330	10.950	12.000	1.00	0.00
ATOM	5441	HW2	SOL	1119	27.750	11.250	11.570	1.00	0.00
ATOM	5442	OW	SOL	1120	40.950	5.470	31.350	1.00	0.00
ATOM	5443	HW1	SOL	1120	41.580	6.170	31.160	1.00	0.00
ATOM	5444	HW2	SOL	1120	41.100	4.810	30.680	1.00	0.00
ATOM	5445	OW	SOL	1121	55.080	37.350	34.250	1.00	0.00
ATOM	5446	HW1	SOL	1121	55.160	37.830	33.430	1.00	0.00
ATOM	5447	HW2	SOL	1121	54.750	38.000	34.880	1.00	0.00
ATOM	5448	OW	SOL	1122	28.680	23.970	12.240	1.00	0.00
ATOM	5449	HW1	SOL	1122	29.350	24.620	12.450	1.00	0.00
ATOM	5450	HW2	SOL	1122	28.640	23.410	13.010	1.00	0.00
ATOM	5451	OW	SOL	1123	31.060	17.000	26.070	1.00	0.00
ATOM	5452	HW1	SOL	1123	30.690	16.430	26.740	1.00	0.00
ATOM	5453	HW2	SOL	1123	30.320	17.540	25.790	1.00	0.00
ATOM	5454	OW	SOL	1124	46.500	18.010	33.370	1.00	0.00
ATOM	5455	HW1	SOL	1124	46.630	18.640	34.080	1.00	0.00

ATOM	5456	HW2	SOL	1124	47.190	17.360	33.500	1.00	0.00
ATOM	5457	OW	SOL	1125	24.880	4.410	42.790	1.00	0.00
ATOM	5458	HW1	SOL	1125	24.130	4.840	42.380	1.00	0.00
ATOM	5459	HW2	SOL	1125	25.650	4.840	42.410	1.00	0.00
ATOM	5460	OW	SOL	1126	17.700	29.770	53.170	1.00	0.00
ATOM	5461	HW1	SOL	1126	17.500	29.100	52.510	1.00	0.00
ATOM	5462	HW2	SOL	1126	17.950	29.280	53.950	1.00	0.00
ATOM	5463	OW	SOL	1127	16.240	12.710	51.860	1.00	0.00
ATOM	5464	HW1	SOL	1127	17.110	12.790	52.240	1.00	0.00
ATOM	5465	HW2	SOL	1127	16.310	11.950	51.270	1.00	0.00
ATOM	5466	OW	SOL	1128	38.230	31.390	27.180	1.00	0.00
ATOM	5467	HW1	SOL	1128	38.910	30.730	27.060	1.00	0.00
ATOM	5468	HW2	SOL	1128	38.590	31.990	27.840	1.00	0.00
ATOM	5469	OW	SOL	1129	50.390	12.450	51.910	1.00	0.00
ATOM	5470	HW1	SOL	1129	50.060	13.310	52.190	1.00	0.00
ATOM	5471	HW2	SOL	1129	49.770	12.150	51.250	1.00	0.00
ATOM	5472	OW	SOL	1130	52.930	51.230	48.410	1.00	0.00
ATOM	5473	HW1	SOL	1130	53.260	50.450	47.970	1.00	0.00
ATOM	5474	HW2	SOL	1130	53.390	51.240	49.250	1.00	0.00
ATOM	5475	OW	SOL	1131	6.490	6.000	9.100	1.00	0.00
ATOM	5476	HW1	SOL	1131	6.390	6.190	10.040	1.00	0.00
ATOM	5477	HW2	SOL	1131	6.890	6.790	8.740	1.00	0.00
ATOM	5478	OW	SOL	1132	20.900	15.700	53.260	1.00	0.00
ATOM	5479	HW1	SOL	1132	21.450	15.380	53.970	1.00	0.00
ATOM	5480	HW2	SOL	1132	20.160	16.120	53.690	1.00	0.00
ATOM	5481	OW	SOL	1133	31.290	33.260	45.020	1.00	0.00
ATOM	5482	HW1	SOL	1133	30.490	33.510	44.540	1.00	0.00
ATOM	5483	HW2	SOL	1133	31.450	32.350	44.760	1.00	0.00
ATOM	5484	OW	SOL	1134	40.700	12.480	51.600	1.00	0.00
ATOM	5485	HW1	SOL	1134	41.080	12.650	50.740	1.00	0.00
ATOM	5486	HW2	SOL	1134	39.910	13.020	51.630	1.00	0.00
ATOM	5487	OW	SOL	1135	42.800	47.870	8.270	1.00	0.00
ATOM	5488	HW1	SOL	1135	42.760	48.390	9.070	1.00	0.00
ATOM	5489	HW2	SOL	1135	41.920	47.930	7.910	1.00	0.00
ATOM	5490	OW	SOL	1136	19.670	0.610	4.600	1.00	0.00
ATOM	5491	HW1	SOL	1136	20.090	-0.070	4.070	1.00	0.00
ATOM	5492	HW2	SOL	1136	19.930	1.440	4.190	1.00	0.00
ATOM	5493	OW	SOL	1137	29.700	6.280	42.320	1.00	0.00
ATOM	5494	HW1	SOL	1137	30.320	5.790	42.870	1.00	0.00
ATOM	5495	HW2	SOL	1137	29.300	6.910	42.920	1.00	0.00
ATOM	5496	OW	SOL	1138	47.920	2.590	6.670	1.00	0.00
ATOM	5497	HW1	SOL	1138	47.490	2.810	5.850	1.00	0.00
ATOM	5498	HW2	SOL	1138	47.360	2.980	7.340	1.00	0.00
ATOM	5499	OW	SOL	1139	17.090	53.150	13.170	1.00	0.00

ATOM	5500	HW1	SOL	1139	17.990	53.290	12.880	1.00	0.00
ATOM	5501	HW2	SOL	1139	16.600	53.000	12.360	1.00	0.00
ATOM	5502	OW	SOL	1140	39.280	25.120	28.890	1.00	0.00
ATOM	5503	HW1	SOL	1140	39.110	24.220	29.170	1.00	0.00
ATOM	5504	HW2	SOL	1140	38.830	25.660	29.530	1.00	0.00
ATOM	5505	OW	SOL	1141	18.700	36.070	1.500	1.00	0.00
ATOM	5506	HW1	SOL	1141	18.510	36.200	0.570	1.00	0.00
ATOM	5507	HW2	SOL	1141	18.120	35.350	1.760	1.00	0.00
ATOM	5508	OW	SOL	1142	37.170	53.880	2.000	1.00	0.00
ATOM	5509	HW1	SOL	1142	38.030	53.870	2.420	1.00	0.00
ATOM	5510	HW2	SOL	1142	37.360	53.960	1.070	1.00	0.00
ATOM	5511	OW	SOL	1143	48.600	12.670	13.370	1.00	0.00
ATOM	5512	HW1	SOL	1143	47.950	11.980	13.510	1.00	0.00
ATOM	5513	HW2	SOL	1143	48.070	13.460	13.250	1.00	0.00
ATOM	5514	OW	SOL	1144	4.830	26.890	48.550	1.00	0.00
ATOM	5515	HW1	SOL	1144	4.200	27.050	49.250	1.00	0.00
ATOM	5516	HW2	SOL	1144	5.370	26.170	48.870	1.00	0.00
ATOM	5517	OW	SOL	1145	50.310	41.880	27.340	1.00	0.00
ATOM	5518	HW1	SOL	1145	50.590	41.620	28.210	1.00	0.00
ATOM	5519	HW2	SOL	1145	51.120	41.960	26.840	1.00	0.00
ATOM	5520	OW	SOL	1146	27.640	9.580	53.140	1.00	0.00
ATOM	5521	HW1	SOL	1146	28.050	10.420	52.930	1.00	0.00
ATOM	5522	HW2	SOL	1146	27.720	9.510	54.090	1.00	0.00
ATOM	5523	OW	SOL	1147	36.860	12.410	1.370	1.00	0.00
ATOM	5524	HW1	SOL	1147	37.040	13.290	1.680	1.00	0.00
ATOM	5525	HW2	SOL	1147	37.330	11.840	1.980	1.00	0.00
ATOM	5526	OW	SOL	1148	32.220	18.220	43.000	1.00	0.00
ATOM	5527	HW1	SOL	1148	33.000	17.760	43.320	1.00	0.00
ATOM	5528	HW2	SOL	1148	31.480	17.720	43.360	1.00	0.00
ATOM	5529	OW	SOL	1149	25.690	17.470	12.240	1.00	0.00
ATOM	5530	HW1	SOL	1149	24.800	17.140	12.170	1.00	0.00
ATOM	5531	HW2	SOL	1149	25.830	17.590	13.180	1.00	0.00
ATOM	5532	OW	SOL	1150	28.640	29.250	28.590	1.00	0.00
ATOM	5533	HW1	SOL	1150	29.290	29.770	28.110	1.00	0.00
ATOM	5534	HW2	SOL	1150	28.040	28.950	27.910	1.00	0.00
ATOM	5535	OW	SOL	1151	9.150	4.200	49.230	1.00	0.00
ATOM	5536	HW1	SOL	1151	9.500	5.080	49.340	1.00	0.00
ATOM	5537	HW2	SOL	1151	9.860	3.620	49.540	1.00	0.00
ATOM	5538	OW	SOL	1152	30.500	42.800	47.320	1.00	0.00
ATOM	5539	HW1	SOL	1152	31.060	43.510	46.990	1.00	0.00
ATOM	5540	HW2	SOL	1152	30.690	42.060	46.750	1.00	0.00
ATOM	5541	OW	SOL	1153	6.720	37.560	10.420	1.00	0.00
ATOM	5542	HW1	SOL	1153	6.670	38.410	10.000	1.00	0.00
ATOM	5543	HW2	SOL	1153	6.170	37.640	11.200	1.00	0.00

ATOM	5544	OW	SOL	1154	45.960	5.550	10.930	1.00	0.00
ATOM	5545	HW1	SOL	1154	46.520	6.300	11.150	1.00	0.00
ATOM	5546	HW2	SOL	1154	45.080	5.920	10.880	1.00	0.00
ATOM	5547	OW	SOL	1155	10.050	55.210	32.310	1.00	0.00
ATOM	5548	HW1	SOL	1155	10.060	56.010	32.830	1.00	0.00
ATOM	5549	HW2	SOL	1155	10.240	54.510	32.930	1.00	0.00
ATOM	5550	OW	SOL	1156	26.400	5.390	8.770	1.00	0.00
ATOM	5551	HW1	SOL	1156	27.110	4.900	8.350	1.00	0.00
ATOM	5552	HW2	SOL	1156	26.160	4.860	9.530	1.00	0.00
ATOM	5553	OW	SOL	1157	31.370	3.330	38.750	1.00	0.00
ATOM	5554	HW1	SOL	1157	30.880	2.510	38.790	1.00	0.00
ATOM	5555	HW2	SOL	1157	31.600	3.520	39.660	1.00	0.00
ATOM	5556	OW	SOL	1158	6.400	35.350	27.630	1.00	0.00
ATOM	5557	HW1	SOL	1158	6.010	34.470	27.550	1.00	0.00
ATOM	5558	HW2	SOL	1158	5.850	35.900	27.080	1.00	0.00
ATOM	5559	OW	SOL	1159	22.470	21.060	46.350	1.00	0.00
ATOM	5560	HW1	SOL	1159	22.150	20.830	45.490	1.00	0.00
ATOM	5561	HW2	SOL	1159	23.180	21.690	46.180	1.00	0.00
ATOM	5562	OW	SOL	1160	17.200	5.500	50.860	1.00	0.00
ATOM	5563	HW1	SOL	1160	16.750	4.790	51.320	1.00	0.00
ATOM	5564	HW2	SOL	1160	16.950	5.380	49.940	1.00	0.00
ATOM	5565	OW	SOL	1161	0.950	35.050	28.060	1.00	0.00
ATOM	5566	HW1	SOL	1161	0.300	34.380	27.860	1.00	0.00
ATOM	5567	HW2	SOL	1161	1.790	34.610	27.960	1.00	0.00
ATOM	5568	OW	SOL	1162	41.140	47.540	14.930	1.00	0.00
ATOM	5569	HW1	SOL	1162	41.990	47.980	14.910	1.00	0.00
ATOM	5570	HW2	SOL	1162	40.520	48.200	15.230	1.00	0.00
ATOM	5571	OW	SOL	1163	22.030	18.840	32.050	1.00	0.00
ATOM	5572	HW1	SOL	1163	22.840	18.640	31.580	1.00	0.00
ATOM	5573	HW2	SOL	1163	21.570	18.010	32.110	1.00	0.00
ATOM	5574	OW	SOL	1164	42.100	51.710	43.410	1.00	0.00
ATOM	5575	HW1	SOL	1164	41.210	51.460	43.170	1.00	0.00
ATOM	5576	HW2	SOL	1164	42.450	52.140	42.630	1.00	0.00
ATOM	5577	OW	SOL	1165	23.080	31.060	27.600	1.00	0.00
ATOM	5578	HW1	SOL	1165	22.300	31.090	28.150	1.00	0.00
ATOM	5579	HW2	SOL	1165	22.870	30.430	26.920	1.00	0.00
ATOM	5580	OW	SOL	1166	31.480	30.300	23.720	1.00	0.00
ATOM	5581	HW1	SOL	1166	31.760	30.300	22.810	1.00	0.00
ATOM	5582	HW2	SOL	1166	31.630	31.200	24.020	1.00	0.00
ATOM	5583	OW	SOL	1167	6.980	16.130	22.090	1.00	0.00
ATOM	5584	HW1	SOL	1167	7.620	16.390	21.430	1.00	0.00
ATOM	5585	HW2	SOL	1167	6.310	16.800	22.050	1.00	0.00
ATOM	5586	OW	SOL	1168	47.130	37.750	1.190	1.00	0.00
ATOM	5587	HW1	SOL	1168	47.410	38.630	0.930	1.00	0.00

ATOM	5588	HW2	SOL	1168	46.360	37.580	0.660	1.00	0.00
ATOM	5589	OW	SOL	1169	16.330	1.700	21.850	1.00	0.00
ATOM	5590	HW1	SOL	1169	15.810	1.160	22.440	1.00	0.00
ATOM	5591	HW2	SOL	1169	17.120	1.180	21.680	1.00	0.00
ATOM	5592	OW	SOL	1170	22.350	0.950	49.040	1.00	0.00
ATOM	5593	HW1	SOL	1170	21.880	1.780	49.010	1.00	0.00
ATOM	5594	HW2	SOL	1170	23.110	1.120	49.590	1.00	0.00
ATOM	5595	OW	SOL	1171	6.130	30.590	20.970	1.00	0.00
ATOM	5596	HW1	SOL	1171	7.030	30.830	21.180	1.00	0.00
ATOM	5597	HW2	SOL	1171	5.650	31.420	20.960	1.00	0.00
ATOM	5598	OW	SOL	1172	37.140	10.820	45.380	1.00	0.00
ATOM	5599	HW1	SOL	1172	37.810	11.490	45.540	1.00	0.00
ATOM	5600	HW2	SOL	1172	36.360	11.320	45.160	1.00	0.00
ATOM	5601	OW	SOL	1173	16.990	23.100	52.530	1.00	0.00
ATOM	5602	HW1	SOL	1173	17.420	23.330	53.350	1.00	0.00
ATOM	5603	HW2	SOL	1173	16.560	23.910	52.250	1.00	0.00
ATOM	5604	OW	SOL	1174	14.550	16.710	18.470	1.00	0.00
ATOM	5605	HW1	SOL	1174	15.210	16.780	19.160	1.00	0.00
ATOM	5606	HW2	SOL	1174	13.880	17.340	18.720	1.00	0.00
ATOM	5607	OW	SOL	1175	38.510	25.240	9.020	1.00	0.00
ATOM	5608	HW1	SOL	1175	38.350	26.050	8.540	1.00	0.00
ATOM	5609	HW2	SOL	1175	38.380	25.490	9.940	1.00	0.00
ATOM	5610	OW	SOL	1176	43.460	31.500	36.240	1.00	0.00
ATOM	5611	HW1	SOL	1176	43.320	31.190	35.350	1.00	0.00
ATOM	5612	HW2	SOL	1176	42.970	32.320	36.280	1.00	0.00
ATOM	5613	OW	SOL	1177	26.310	9.490	19.640	1.00	0.00
ATOM	5614	HW1	SOL	1177	26.590	10.410	19.730	1.00	0.00
ATOM	5615	HW2	SOL	1177	25.540	9.530	19.080	1.00	0.00
ATOM	5616	OW	SOL	1178	43.130	37.980	21.590	1.00	0.00
ATOM	5617	HW1	SOL	1178	42.630	37.510	20.920	1.00	0.00
ATOM	5618	HW2	SOL	1178	43.030	37.450	22.380	1.00	0.00
ATOM	5619	OW	SOL	1179	42.460	27.220	37.190	1.00	0.00
ATOM	5620	HW1	SOL	1179	42.250	27.280	36.260	1.00	0.00
ATOM	5621	HW2	SOL	1179	42.730	26.320	37.330	1.00	0.00
ATOM	5622	OW	SOL	1180	1.530	20.260	25.570	1.00	0.00
ATOM	5623	HW1	SOL	1180	2.160	20.490	26.260	1.00	0.00
ATOM	5624	HW2	SOL	1180	0.780	20.830	25.740	1.00	0.00
ATOM	5625	OW	SOL	1181	43.690	31.120	47.940	1.00	0.00
ATOM	5626	HW1	SOL	1181	42.950	31.620	48.280	1.00	0.00
ATOM	5627	HW2	SOL	1181	44.450	31.680	48.080	1.00	0.00
ATOM	5628	OW	SOL	1182	42.490	37.390	24.800	1.00	0.00
ATOM	5629	HW1	SOL	1182	42.070	37.750	25.590	1.00	0.00
ATOM	5630	HW2	SOL	1182	43.410	37.620	24.890	1.00	0.00
ATOM	5631	OW	SOL	1183	29.070	55.190	2.930	1.00	0.00

ATOM	5632	HW1	SOL	1183	29.370	55.730	2.190	1.00	0.00
ATOM	5633	HW2	SOL	1183	29.130	55.770	3.690	1.00	0.00
ATOM	5634	OW	SOL	1184	52.360	33.440	16.400	1.00	0.00
ATOM	5635	HW1	SOL	1184	53.010	34.110	16.620	1.00	0.00
ATOM	5636	HW2	SOL	1184	52.590	33.160	15.520	1.00	0.00
ATOM	5637	OW	SOL	1185	28.380	25.390	16.700	1.00	0.00
ATOM	5638	HW1	SOL	1185	27.810	24.640	16.530	1.00	0.00
ATOM	5639	HW2	SOL	1185	29.190	25.190	16.230	1.00	0.00
ATOM	5640	OW	SOL	1186	51.910	2.920	4.480	1.00	0.00
ATOM	5641	HW1	SOL	1186	51.290	3.630	4.680	1.00	0.00
ATOM	5642	HW2	SOL	1186	52.300	2.700	5.320	1.00	0.00
ATOM	5643	OW	SOL	1187	32.200	45.950	43.780	1.00	0.00
ATOM	5644	HW1	SOL	1187	31.910	45.480	43.000	1.00	0.00
ATOM	5645	HW2	SOL	1187	33.150	45.940	43.730	1.00	0.00
ATOM	5646	OW	SOL	1188	9.890	42.450	17.100	1.00	0.00
ATOM	5647	HW1	SOL	1188	10.560	42.670	17.750	1.00	0.00
ATOM	5648	HW2	SOL	1188	9.370	43.250	17.020	1.00	0.00
ATOM	5649	OW	SOL	1189	13.720	47.590	31.340	1.00	0.00
ATOM	5650	HW1	SOL	1189	14.350	47.590	32.060	1.00	0.00
ATOM	5651	HW2	SOL	1189	12.950	48.030	31.690	1.00	0.00
ATOM	5652	OW	SOL	1190	40.360	16.250	24.060	1.00	0.00
ATOM	5653	HW1	SOL	1190	39.990	17.040	23.670	1.00	0.00
ATOM	5654	HW2	SOL	1190	41.310	16.350	23.950	1.00	0.00
ATOM	5655	OW	SOL	1191	24.890	7.980	8.770	1.00	0.00
ATOM	5656	HW1	SOL	1191	25.650	8.550	8.630	1.00	0.00
ATOM	5657	HW2	SOL	1191	25.230	7.090	8.650	1.00	0.00
ATOM	5658	OW	SOL	1192	27.330	40.050	42.920	1.00	0.00
ATOM	5659	HW1	SOL	1192	26.460	40.430	42.960	1.00	0.00
ATOM	5660	HW2	SOL	1192	27.860	40.710	42.470	1.00	0.00
ATOM	5661	OW	SOL	1193	0.180	7.920	14.670	1.00	0.00
ATOM	5662	HW1	SOL	1193	0.730	7.550	15.360	1.00	0.00
ATOM	5663	HW2	SOL	1193	-0.340	8.590	15.110	1.00	0.00
ATOM	5664	OW	SOL	1194	12.450	54.400	45.770	1.00	0.00
ATOM	5665	HW1	SOL	1194	12.440	54.600	46.710	1.00	0.00
ATOM	5666	HW2	SOL	1194	12.060	55.170	45.350	1.00	0.00
ATOM	5667	OW	SOL	1195	33.360	18.300	35.750	1.00	0.00
ATOM	5668	HW1	SOL	1195	33.770	18.850	35.090	1.00	0.00
ATOM	5669	HW2	SOL	1195	32.460	18.190	35.450	1.00	0.00
ATOM	5670	OW	SOL	1196	8.360	40.790	31.920	1.00	0.00
ATOM	5671	HW1	SOL	1196	9.200	40.760	32.380	1.00	0.00
ATOM	5672	HW2	SOL	1196	8.520	40.310	31.100	1.00	0.00
ATOM	5673	OW	SOL	1197	54.650	27.440	45.210	1.00	0.00
ATOM	5674	HW1	SOL	1197	54.980	26.570	44.980	1.00	0.00
ATOM	5675	HW2	SOL	1197	55.380	28.030	45.000	1.00	0.00

ATOM	5676	OW	SOL	1198	20.070	18.310	11.960	1.00	0.00
ATOM	5677	HW1	SOL	1198	19.230	18.270	11.500	1.00	0.00
ATOM	5678	HW2	SOL	1198	19.840	18.590	12.840	1.00	0.00
ATOM	5679	OW	SOL	1199	54.320	33.980	47.150	1.00	0.00
ATOM	5680	HW1	SOL	1199	53.500	33.890	46.670	1.00	0.00
ATOM	5681	HW2	SOL	1199	54.080	33.940	48.070	1.00	0.00
ATOM	5682	OW	SOL	1200	6.120	20.280	5.420	1.00	0.00
ATOM	5683	HW1	SOL	1200	5.780	19.380	5.390	1.00	0.00
ATOM	5684	HW2	SOL	1200	5.710	20.660	6.190	1.00	0.00
ATOM	5685	OW	SOL	1201	38.900	17.250	44.030	1.00	0.00
ATOM	5686	HW1	SOL	1201	39.040	17.840	43.290	1.00	0.00
ATOM	5687	HW2	SOL	1201	39.610	17.450	44.640	1.00	0.00
ATOM	5688	OW	SOL	1202	43.120	21.450	23.590	1.00	0.00
ATOM	5689	HW1	SOL	1202	42.460	20.760	23.590	1.00	0.00
ATOM	5690	HW2	SOL	1202	43.160	21.750	22.680	1.00	0.00
ATOM	5691	OW	SOL	1203	19.640	13.380	36.840	1.00	0.00
ATOM	5692	HW1	SOL	1203	19.210	14.040	37.380	1.00	0.00
ATOM	5693	HW2	SOL	1203	20.480	13.240	37.260	1.00	0.00
ATOM	5694	OW	SOL	1204	50.710	18.400	29.130	1.00	0.00
ATOM	5695	HW1	SOL	1204	50.930	17.810	28.410	1.00	0.00
ATOM	5696	HW2	SOL	1204	50.030	18.970	28.760	1.00	0.00
ATOM	5697	OW	SOL	1205	8.300	55.070	28.240	1.00	0.00
ATOM	5698	HW1	SOL	1205	9.190	55.280	28.500	1.00	0.00
ATOM	5699	HW2	SOL	1205	8.050	55.760	27.630	1.00	0.00
ATOM	5700	OW	SOL	1206	35.650	38.380	55.640	1.00	0.00
ATOM	5701	HW1	SOL	1206	35.960	38.560	56.530	1.00	0.00
ATOM	5702	HW2	SOL	1206	35.250	37.510	55.700	1.00	0.00
ATOM	5703	OW	SOL	1207	24.870	49.730	40.580	1.00	0.00
ATOM	5704	HW1	SOL	1207	23.930	49.570	40.530	1.00	0.00
ATOM	5705	HW2	SOL	1207	25.080	50.160	39.750	1.00	0.00
ATOM	5706	OW	SOL	1208	12.320	39.530	55.660	1.00	0.00
ATOM	5707	HW1	SOL	1208	12.770	40.360	55.580	1.00	0.00
ATOM	5708	HW2	SOL	1208	11.520	39.730	56.150	1.00	0.00
ATOM	5709	OW	SOL	1209	12.640	28.860	27.170	1.00	0.00
ATOM	5710	HW1	SOL	1209	12.990	29.650	26.770	1.00	0.00
ATOM	5711	HW2	SOL	1209	12.480	28.270	26.440	1.00	0.00
ATOM	5712	OW	SOL	1210	37.780	18.770	5.400	1.00	0.00
ATOM	5713	HW1	SOL	1210	38.680	18.710	5.720	1.00	0.00
ATOM	5714	HW2	SOL	1210	37.610	17.920	5.000	1.00	0.00
ATOM	5715	OW	SOL	1211	13.590	9.320	49.600	1.00	0.00
ATOM	5716	HW1	SOL	1211	13.170	10.130	49.880	1.00	0.00
ATOM	5717	HW2	SOL	1211	12.910	8.650	49.710	1.00	0.00
ATOM	5718	OW	SOL	1212	51.140	18.590	2.450	1.00	0.00
ATOM	5719	HW1	SOL	1212	51.490	19.360	1.990	1.00	0.00

ATOM	5720	HW2	SOL	1212	51.790	17.900	2.290	1.00	0.00
ATOM	5721	OW	SOL	1213	37.080	14.230	15.720	1.00	0.00
ATOM	5722	HW1	SOL	1213	36.300	14.480	16.230	1.00	0.00
ATOM	5723	HW2	SOL	1213	37.590	15.030	15.650	1.00	0.00
ATOM	5724	OW	SOL	1214	36.750	4.390	17.390	1.00	0.00
ATOM	5725	HW1	SOL	1214	36.010	3.800	17.530	1.00	0.00
ATOM	5726	HW2	SOL	1214	37.400	3.870	16.910	1.00	0.00
ATOM	5727	OW	SOL	1215	6.720	1.880	22.660	1.00	0.00
ATOM	5728	HW1	SOL	1215	6.950	1.890	21.730	1.00	0.00
ATOM	5729	HW2	SOL	1215	5.780	1.700	22.670	1.00	0.00
ATOM	5730	OW	SOL	1216	13.010	23.090	31.260	1.00	0.00
ATOM	5731	HW1	SOL	1216	13.360	23.910	31.610	1.00	0.00
ATOM	5732	HW2	SOL	1216	12.260	22.890	31.820	1.00	0.00
ATOM	5733	OW	SOL	1217	25.660	15.780	20.990	1.00	0.00
ATOM	5734	HW1	SOL	1217	25.840	16.230	20.160	1.00	0.00
ATOM	5735	HW2	SOL	1217	24.880	15.250	20.810	1.00	0.00
ATOM	5736	OW	SOL	1218	13.880	11.280	28.840	1.00	0.00
ATOM	5737	HW1	SOL	1218	13.880	12.240	28.870	1.00	0.00
ATOM	5738	HW2	SOL	1218	13.600	11.010	29.720	1.00	0.00
ATOM	5739	OW	SOL	1219	39.810	14.760	30.030	1.00	0.00
ATOM	5740	HW1	SOL	1219	38.860	14.610	30.050	1.00	0.00
ATOM	5741	HW2	SOL	1219	40.070	14.460	29.160	1.00	0.00
ATOM	5742	OW	SOL	1220	24.030	20.590	21.710	1.00	0.00
ATOM	5743	HW1	SOL	1220	24.310	19.710	21.450	1.00	0.00
ATOM	5744	HW2	SOL	1220	23.710	20.480	22.610	1.00	0.00
ATOM	5745	OW	SOL	1221	54.680	16.400	40.910	1.00	0.00
ATOM	5746	HW1	SOL	1221	54.210	16.270	40.090	1.00	0.00
ATOM	5747	HW2	SOL	1221	54.040	16.200	41.590	1.00	0.00
ATOM	5748	OW	SOL	1222	38.070	45.670	2.390	1.00	0.00
ATOM	5749	HW1	SOL	1222	38.670	46.410	2.500	1.00	0.00
ATOM	5750	HW2	SOL	1222	38.170	45.420	1.470	1.00	0.00
ATOM	5751	OW	SOL	1223	21.700	21.550	40.210	1.00	0.00
ATOM	5752	HW1	SOL	1223	21.740	22.300	40.800	1.00	0.00
ATOM	5753	HW2	SOL	1223	22.300	20.910	40.600	1.00	0.00
ATOM	5754	OW	SOL	1224	17.950	8.430	19.640	1.00	0.00
ATOM	5755	HW1	SOL	1224	18.050	9.380	19.740	1.00	0.00
ATOM	5756	HW2	SOL	1224	18.840	8.090	19.590	1.00	0.00
ATOM	5757	OW	SOL	1225	2.590	47.170	22.020	1.00	0.00
ATOM	5758	HW1	SOL	1225	2.250	47.920	21.530	1.00	0.00
ATOM	5759	HW2	SOL	1225	3.130	47.560	22.710	1.00	0.00
ATOM	5760	OW	SOL	1226	43.250	51.400	13.120	1.00	0.00
ATOM	5761	HW1	SOL	1226	43.660	52.110	12.620	1.00	0.00
ATOM	5762	HW2	SOL	1226	42.510	51.820	13.560	1.00	0.00
ATOM	5763	OW	SOL	1227	51.910	20.270	10.720	1.00	0.00

ATOM	5764	HW1	SOL	1227	51.320	21.030	10.750	1.00	0.00
ATOM	5765	HW2	SOL	1227	51.850	19.900	11.590	1.00	0.00
ATOM	5766	OW	SOL	1228	28.550	38.820	0.110	1.00	0.00
ATOM	5767	HW1	SOL	1228	28.840	39.730	0.120	1.00	0.00
ATOM	5768	HW2	SOL	1228	28.420	38.590	1.030	1.00	0.00
ATOM	5769	OW	SOL	1229	39.360	18.300	22.250	1.00	0.00
ATOM	5770	HW1	SOL	1229	39.280	19.000	22.890	1.00	0.00
ATOM	5771	HW2	SOL	1229	38.480	18.210	21.890	1.00	0.00
ATOM	5772	OW	SOL	1230	0.190	47.360	26.120	1.00	0.00
ATOM	5773	HW1	SOL	1230	0.300	47.240	27.060	1.00	0.00
ATOM	5774	HW2	SOL	1230	1.060	47.630	25.810	1.00	0.00
ATOM	5775	OW	SOL	1231	32.650	24.960	41.180	1.00	0.00
ATOM	5776	HW1	SOL	1231	33.470	25.280	41.560	1.00	0.00
ATOM	5777	HW2	SOL	1231	32.930	24.380	40.470	1.00	0.00
ATOM	5778	OW	SOL	1232	27.680	18.220	41.030	1.00	0.00
ATOM	5779	HW1	SOL	1232	28.140	18.270	40.190	1.00	0.00
ATOM	5780	HW2	SOL	1232	27.550	19.140	41.280	1.00	0.00
ATOM	5781	OW	SOL	1233	41.780	8.720	0.230	1.00	0.00
ATOM	5782	HW1	SOL	1233	41.810	9.160	-0.620	1.00	0.00
ATOM	5783	HW2	SOL	1233	41.440	9.380	0.840	1.00	0.00
ATOM	5784	OW	SOL	1234	8.940	52.220	28.540	1.00	0.00
ATOM	5785	HW1	SOL	1234	8.780	53.160	28.490	1.00	0.00
ATOM	5786	HW2	SOL	1234	9.060	52.040	29.470	1.00	0.00
ATOM	5787	OW	SOL	1235	0.510	10.580	38.830	1.00	0.00
ATOM	5788	HW1	SOL	1235	1.210	11.180	39.100	1.00	0.00
ATOM	5789	HW2	SOL	1235	0.060	11.040	38.120	1.00	0.00
ATOM	5790	OW	SOL	1236	34.750	47.730	14.600	1.00	0.00
ATOM	5791	HW1	SOL	1236	35.140	48.590	14.680	1.00	0.00
ATOM	5792	HW2	SOL	1236	34.050	47.710	15.250	1.00	0.00
ATOM	5793	OW	SOL	1237	29.900	50.010	23.220	1.00	0.00
ATOM	5794	HW1	SOL	1237	29.290	50.280	23.910	1.00	0.00
ATOM	5795	HW2	SOL	1237	30.740	49.910	23.670	1.00	0.00
ATOM	5796	OW	SOL	1238	42.270	2.890	40.860	1.00	0.00
ATOM	5797	HW1	SOL	1238	42.060	3.720	40.450	1.00	0.00
ATOM	5798	HW2	SOL	1238	41.420	2.450	40.970	1.00	0.00
ATOM	5799	OW	SOL	1239	34.030	40.310	47.130	1.00	0.00
ATOM	5800	HW1	SOL	1239	33.170	39.890	47.040	1.00	0.00
ATOM	5801	HW2	SOL	1239	33.980	40.760	47.970	1.00	0.00
ATOM	5802	OW	SOL	1240	46.930	44.380	52.950	1.00	0.00
ATOM	5803	HW1	SOL	1240	47.130	44.010	53.810	1.00	0.00
ATOM	5804	HW2	SOL	1240	47.560	45.100	52.850	1.00	0.00
ATOM	5805	OW	SOL	1241	41.640	28.410	14.020	1.00	0.00
ATOM	5806	HW1	SOL	1241	42.130	28.550	13.210	1.00	0.00
ATOM	5807	HW2	SOL	1241	40.840	28.930	13.900	1.00	0.00

ATOM	5808	OW	SOL	1242	49.600	16.870	26.890	1.00	0.00
ATOM	5809	HW1	SOL	1242	49.060	17.270	27.580	1.00	0.00
ATOM	5810	HW2	SOL	1242	49.300	17.290	26.090	1.00	0.00
ATOM	5811	OW	SOL	1243	6.940	50.210	19.600	1.00	0.00
ATOM	5812	HW1	SOL	1243	6.940	50.990	20.160	1.00	0.00
ATOM	5813	HW2	SOL	1243	7.700	50.320	19.040	1.00	0.00
ATOM	5814	OW	SOL	1244	37.670	9.960	2.640	1.00	0.00
ATOM	5815	HW1	SOL	1244	37.310	9.930	3.520	1.00	0.00
ATOM	5816	HW2	SOL	1244	37.230	9.250	2.170	1.00	0.00
ATOM	5817	OW	SOL	1245	3.440	47.320	35.040	1.00	0.00
ATOM	5818	HW1	SOL	1245	3.150	46.870	34.250	1.00	0.00
ATOM	5819	HW2	SOL	1245	4.150	46.780	35.380	1.00	0.00
ATOM	5820	OW	SOL	1246	5.870	20.730	48.120	1.00	0.00
ATOM	5821	HW1	SOL	1246	5.860	20.180	47.340	1.00	0.00
ATOM	5822	HW2	SOL	1246	5.710	21.620	47.800	1.00	0.00
ATOM	5823	OW	SOL	1247	48.780	44.910	13.400	1.00	0.00
ATOM	5824	HW1	SOL	1247	48.290	45.430	14.040	1.00	0.00
ATOM	5825	HW2	SOL	1247	49.580	44.650	13.860	1.00	0.00
ATOM	5826	OW	SOL	1248	17.330	2.240	50.000	1.00	0.00
ATOM	5827	HW1	SOL	1248	17.220	3.120	49.640	1.00	0.00
ATOM	5828	HW2	SOL	1248	17.240	1.650	49.250	1.00	0.00
ATOM	5829	OW	SOL	1249	30.740	4.050	22.320	1.00	0.00
ATOM	5830	HW1	SOL	1249	30.790	3.110	22.460	1.00	0.00
ATOM	5831	HW2	SOL	1249	31.000	4.430	23.160	1.00	0.00
ATOM	5832	OW	SOL	1250	2.850	3.870	1.350	1.00	0.00
ATOM	5833	HW1	SOL	1250	3.660	3.540	1.740	1.00	0.00
ATOM	5834	HW2	SOL	1250	3.130	4.350	0.570	1.00	0.00
ATOM	5835	OW	SOL	1251	34.910	7.500	7.270	1.00	0.00
ATOM	5836	HW1	SOL	1251	34.500	7.800	6.460	1.00	0.00
ATOM	5837	HW2	SOL	1251	34.960	8.280	7.820	1.00	0.00
ATOM	5838	OW	SOL	1252	50.810	17.920	19.390	1.00	0.00
ATOM	5839	HW1	SOL	1252	50.240	18.230	18.690	1.00	0.00
ATOM	5840	HW2	SOL	1252	50.240	17.350	19.920	1.00	0.00
ATOM	5841	OW	SOL	1253	36.860	27.070	17.930	1.00	0.00
ATOM	5842	HW1	SOL	1253	37.480	26.380	17.710	1.00	0.00
ATOM	5843	HW2	SOL	1253	37.400	27.830	18.140	1.00	0.00
ATOM	5844	OW	SOL	1254	55.290	32.120	50.310	1.00	0.00
ATOM	5845	HW1	SOL	1254	54.670	31.400	50.350	1.00	0.00
ATOM	5846	HW2	SOL	1254	54.770	32.860	49.990	1.00	0.00
ATOM	5847	OW	SOL	1255	6.010	3.340	13.720	1.00	0.00
ATOM	5848	HW1	SOL	1255	5.260	3.920	13.650	1.00	0.00
ATOM	5849	HW2	SOL	1255	5.640	2.500	13.980	1.00	0.00
ATOM	5850	OW	SOL	1256	52.740	36.360	50.470	1.00	0.00
ATOM	5851	HW1	SOL	1256	53.580	36.780	50.660	1.00	0.00

ATOM	5852	HW2	SOL	1256	52.190	37.070	50.120	1.00	0.00
ATOM	5853	OW	SOL	1257	10.150	27.660	29.050	1.00	0.00
ATOM	5854	HW1	SOL	1257	10.920	27.400	28.550	1.00	0.00
ATOM	5855	HW2	SOL	1257	10.290	27.290	29.920	1.00	0.00
ATOM	5856	OW	SOL	1258	51.600	8.080	54.510	1.00	0.00
ATOM	5857	HW1	SOL	1258	50.650	8.020	54.500	1.00	0.00
ATOM	5858	HW2	SOL	1258	51.810	8.760	53.870	1.00	0.00
ATOM	5859	OW	SOL	1259	27.740	22.200	55.560	1.00	0.00
ATOM	5860	HW1	SOL	1259	27.990	21.330	55.240	1.00	0.00
ATOM	5861	HW2	SOL	1259	27.130	22.020	56.280	1.00	0.00
ATOM	5862	OW	SOL	1260	9.720	45.940	34.120	1.00	0.00
ATOM	5863	HW1	SOL	1260	9.220	45.250	33.680	1.00	0.00
ATOM	5864	HW2	SOL	1260	10.090	45.510	34.900	1.00	0.00
ATOM	5865	OW	SOL	1261	38.410	1.060	43.220	1.00	0.00
ATOM	5866	HW1	SOL	1261	39.000	0.890	43.950	1.00	0.00
ATOM	5867	HW2	SOL	1261	38.010	0.210	43.030	1.00	0.00
ATOM	5868	OW	SOL	1262	20.550	49.120	47.760	1.00	0.00
ATOM	5869	HW1	SOL	1262	19.730	48.610	47.740	1.00	0.00
ATOM	5870	HW2	SOL	1262	20.360	49.890	47.210	1.00	0.00
ATOM	5871	OW	SOL	1263	38.160	26.890	55.450	1.00	0.00
ATOM	5872	HW1	SOL	1263	38.560	26.210	54.910	1.00	0.00
ATOM	5873	HW2	SOL	1263	37.830	27.530	54.820	1.00	0.00
ATOM	5874	OW	SOL	1264	24.750	41.880	51.530	1.00	0.00
ATOM	5875	HW1	SOL	1264	24.860	42.040	50.600	1.00	0.00
ATOM	5876	HW2	SOL	1264	24.870	40.930	51.620	1.00	0.00
ATOM	5877	OW	SOL	1265	16.270	23.720	11.610	1.00	0.00
ATOM	5878	HW1	SOL	1265	16.670	23.620	10.740	1.00	0.00
ATOM	5879	HW2	SOL	1265	15.800	24.550	11.550	1.00	0.00
ATOM	5880	OW	SOL	1266	19.760	49.990	54.810	1.00	0.00
ATOM	5881	HW1	SOL	1266	19.690	50.860	54.400	1.00	0.00
ATOM	5882	HW2	SOL	1266	19.760	50.170	55.750	1.00	0.00
ATOM	5883	OW	SOL	1267	37.010	31.240	44.350	1.00	0.00
ATOM	5884	HW1	SOL	1267	36.160	31.440	43.950	1.00	0.00
ATOM	5885	HW2	SOL	1267	37.440	32.090	44.420	1.00	0.00
ATOM	5886	OW	SOL	1268	37.050	15.080	44.270	1.00	0.00
ATOM	5887	HW1	SOL	1268	37.260	14.430	43.600	1.00	0.00
ATOM	5888	HW2	SOL	1268	37.380	15.910	43.920	1.00	0.00
ATOM	5889	OW	SOL	1269	40.690	6.950	47.700	1.00	0.00
ATOM	5890	HW1	SOL	1269	41.270	6.960	46.940	1.00	0.00
ATOM	5891	HW2	SOL	1269	40.880	7.770	48.160	1.00	0.00
ATOM	5892	OW	SOL	1270	1.240	6.120	49.020	1.00	0.00
ATOM	5893	HW1	SOL	1270	1.820	5.840	49.730	1.00	0.00
ATOM	5894	HW2	SOL	1270	1.830	6.460	48.350	1.00	0.00
ATOM	5895	OW	SOL	1271	47.110	36.860	38.890	1.00	0.00

ATOM	5896	HW1	SOL	1271	46.600	36.060	38.750	1.00	0.00
ATOM	5897	HW2	SOL	1271	47.110	36.980	39.840	1.00	0.00
ATOM	5898	OW	SOL	1272	41.400	41.050	10.310	1.00	0.00
ATOM	5899	HW1	SOL	1272	41.000	40.730	9.490	1.00	0.00
ATOM	5900	HW2	SOL	1272	42.030	41.710	10.020	1.00	0.00
ATOM	5901	OW	SOL	1273	36.630	8.750	25.700	1.00	0.00
ATOM	5902	HW1	SOL	1273	35.680	8.840	25.660	1.00	0.00
ATOM	5903	HW2	SOL	1273	36.800	7.870	25.340	1.00	0.00
ATOM	5904	OW	SOL	1274	9.510	33.900	21.430	1.00	0.00
ATOM	5905	HW1	SOL	1274	10.460	33.890	21.330	1.00	0.00
ATOM	5906	HW2	SOL	1274	9.360	34.410	22.230	1.00	0.00
ATOM	5907	OW	SOL	1275	4.340	2.640	35.680	1.00	0.00
ATOM	5908	HW1	SOL	1275	3.840	2.250	36.400	1.00	0.00
ATOM	5909	HW2	SOL	1275	4.230	2.010	34.960	1.00	0.00
ATOM	5910	OW	SOL	1276	7.200	10.700	3.650	1.00	0.00
ATOM	5911	HW1	SOL	1276	7.560	10.750	2.760	1.00	0.00
ATOM	5912	HW2	SOL	1276	6.270	10.530	3.530	1.00	0.00
ATOM	5913	OW	SOL	1277	7.540	45.000	40.560	1.00	0.00
ATOM	5914	HW1	SOL	1277	6.630	45.290	40.550	1.00	0.00
ATOM	5915	HW2	SOL	1277	7.980	45.560	39.910	1.00	0.00
ATOM	5916	OW	SOL	1278	15.090	14.130	43.350	1.00	0.00
ATOM	5917	HW1	SOL	1278	14.980	13.190	43.220	1.00	0.00
ATOM	5918	HW2	SOL	1278	14.690	14.290	44.210	1.00	0.00
ATOM	5919	OW	SOL	1279	12.010	35.530	48.160	1.00	0.00
ATOM	5920	HW1	SOL	1279	12.490	34.970	47.560	1.00	0.00
ATOM	5921	HW2	SOL	1279	11.470	34.930	48.670	1.00	0.00
ATOM	5922	OW	SOL	1280	3.080	9.940	50.690	1.00	0.00
ATOM	5923	HW1	SOL	1280	3.940	10.330	50.540	1.00	0.00
ATOM	5924	HW2	SOL	1280	3.190	9.390	51.460	1.00	0.00
ATOM	5925	OW	SOL	1281	9.570	6.550	50.660	1.00	0.00
ATOM	5926	HW1	SOL	1281	10.090	7.060	51.280	1.00	0.00
ATOM	5927	HW2	SOL	1281	9.140	5.890	51.210	1.00	0.00
ATOM	5928	OW	SOL	1282	12.530	50.100	22.230	1.00	0.00
ATOM	5929	HW1	SOL	1282	11.750	50.570	22.510	1.00	0.00
ATOM	5930	HW2	SOL	1282	13.050	50.770	21.760	1.00	0.00
ATOM	5931	OW	SOL	1283	28.510	27.670	42.220	1.00	0.00
ATOM	5932	HW1	SOL	1283	27.870	27.810	41.520	1.00	0.00
ATOM	5933	HW2	SOL	1283	29.320	27.450	41.760	1.00	0.00
ATOM	5934	OW	SOL	1284	46.320	6.810	41.650	1.00	0.00
ATOM	5935	HW1	SOL	1284	47.260	6.650	41.650	1.00	0.00
ATOM	5936	HW2	SOL	1284	45.940	6.010	41.280	1.00	0.00
ATOM	5937	OW	SOL	1285	12.050	31.310	3.730	1.00	0.00
ATOM	5938	HW1	SOL	1285	12.360	32.070	4.240	1.00	0.00
ATOM	5939	HW2	SOL	1285	11.110	31.290	3.910	1.00	0.00

ATOM	5940	OW	SOL	1286	4.410	53.350	12.480	1.00	0.00
ATOM	5941	HW1	SOL	1286	4.830	52.700	11.910	1.00	0.00
ATOM	5942	HW2	SOL	1286	3.610	52.940	12.770	1.00	0.00
ATOM	5943	OW	SOL	1287	0.120	33.330	0.120	1.00	0.00
ATOM	5944	HW1	SOL	1287	-0.640	33.330	-0.460	1.00	0.00
ATOM	5945	HW2	SOL	1287	-0.080	32.650	0.770	1.00	0.00
ATOM	5946	OW	SOL	1288	37.560	48.460	55.400	1.00	0.00
ATOM	5947	HW1	SOL	1288	36.960	48.900	54.790	1.00	0.00
ATOM	5948	HW2	SOL	1288	38.030	49.170	55.830	1.00	0.00
ATOM	5949	OW	SOL	1289	51.340	25.090	11.970	1.00	0.00
ATOM	5950	HW1	SOL	1289	51.530	25.700	11.250	1.00	0.00
ATOM	5951	HW2	SOL	1289	51.750	25.480	12.740	1.00	0.00
ATOM	5952	OW	SOL	1290	8.160	19.170	29.810	1.00	0.00
ATOM	5953	HW1	SOL	1290	8.870	18.590	30.100	1.00	0.00
ATOM	5954	HW2	SOL	1290	8.470	20.050	30.020	1.00	0.00
ATOM	5955	OW	SOL	1291	20.060	18.930	38.820	1.00	0.00
ATOM	5956	HW1	SOL	1291	20.870	18.460	38.650	1.00	0.00
ATOM	5957	HW2	SOL	1291	20.140	19.240	39.720	1.00	0.00
ATOM	5958	OW	SOL	1292	23.820	4.440	45.290	1.00	0.00
ATOM	5959	HW1	SOL	1292	23.920	4.370	44.340	1.00	0.00
ATOM	5960	HW2	SOL	1292	22.910	4.720	45.410	1.00	0.00
ATOM	5961	OW	SOL	1293	5.020	43.490	15.210	1.00	0.00
ATOM	5962	HW1	SOL	1293	4.440	42.830	14.840	1.00	0.00
ATOM	5963	HW2	SOL	1293	5.450	43.900	14.450	1.00	0.00
ATOM	5964	OW	SOL	1294	36.410	23.480	52.020	1.00	0.00
ATOM	5965	HW1	SOL	1294	36.140	23.830	52.870	1.00	0.00
ATOM	5966	HW2	SOL	1294	36.340	24.220	51.420	1.00	0.00
ATOM	5967	OW	SOL	1295	10.060	7.150	17.890	1.00	0.00
ATOM	5968	HW1	SOL	1295	10.510	6.480	18.400	1.00	0.00
ATOM	5969	HW2	SOL	1295	10.730	7.820	17.720	1.00	0.00
ATOM	5970	OW	SOL	1296	19.180	25.720	52.540	1.00	0.00
ATOM	5971	HW1	SOL	1296	19.630	25.050	52.030	1.00	0.00
ATOM	5972	HW2	SOL	1296	18.790	26.300	51.890	1.00	0.00
ATOM	5973	OW	SOL	1297	8.200	15.320	12.270	1.00	0.00
ATOM	5974	HW1	SOL	1297	8.870	14.770	12.670	1.00	0.00
ATOM	5975	HW2	SOL	1297	7.420	15.180	12.800	1.00	0.00
ATOM	5976	OW	SOL	1298	55.300	52.940	5.210	1.00	0.00
ATOM	5977	HW1	SOL	1298	55.950	53.220	4.570	1.00	0.00
ATOM	5978	HW2	SOL	1298	55.680	52.160	5.620	1.00	0.00
ATOM	5979	OW	SOL	1299	12.260	41.710	38.920	1.00	0.00
ATOM	5980	HW1	SOL	1299	12.110	41.230	39.730	1.00	0.00
ATOM	5981	HW2	SOL	1299	11.420	41.660	38.460	1.00	0.00
ATOM	5982	OW	SOL	1300	54.020	2.740	17.150	1.00	0.00
ATOM	5983	HW1	SOL	1300	54.290	3.570	16.760	1.00	0.00

ATOM	5984	HW2	SOL	1300	53.400	2.370	16.520	1.00	0.00
ATOM	5985	OW	SOL	1301	53.420	11.970	0.180	1.00	0.00
ATOM	5986	HW1	SOL	1301	53.340	12.280	-0.720	1.00	0.00
ATOM	5987	HW2	SOL	1301	53.640	11.040	0.090	1.00	0.00
ATOM	5988	OW	SOL	1302	10.680	20.040	4.050	1.00	0.00
ATOM	5989	HW1	SOL	1302	11.530	19.590	4.070	1.00	0.00
ATOM	5990	HW2	SOL	1302	10.100	19.420	3.610	1.00	0.00
ATOM	5991	OW	SOL	1303	28.800	18.080	1.560	1.00	0.00
ATOM	5992	HW1	SOL	1303	28.980	17.720	0.690	1.00	0.00
ATOM	5993	HW2	SOL	1303	29.590	17.880	2.070	1.00	0.00
ATOM	5994	OW	SOL	1304	12.740	25.840	26.350	1.00	0.00
ATOM	5995	HW1	SOL	1304	12.300	25.860	25.500	1.00	0.00
ATOM	5996	HW2	SOL	1304	12.380	25.070	26.790	1.00	0.00
ATOM	5997	OW	SOL	1305	52.690	6.440	6.190	1.00	0.00
ATOM	5998	HW1	SOL	1305	52.310	6.270	5.330	1.00	0.00
ATOM	5999	HW2	SOL	1305	53.630	6.420	6.050	1.00	0.00
ATOM	6000	OW	SOL	1306	20.130	6.920	18.360	1.00	0.00
ATOM	6001	HW1	SOL	1306	19.620	6.110	18.370	1.00	0.00
ATOM	6002	HW2	SOL	1306	21.010	6.640	18.110	1.00	0.00
ATOM	6003	OW	SOL	1307	25.400	47.030	6.350	1.00	0.00
ATOM	6004	HW1	SOL	1307	25.540	47.250	7.270	1.00	0.00
ATOM	6005	HW2	SOL	1307	25.160	47.870	5.940	1.00	0.00
ATOM	6006	OW	SOL	1308	2.690	41.960	16.870	1.00	0.00
ATOM	6007	HW1	SOL	1308	2.240	42.100	16.040	1.00	0.00
ATOM	6008	HW2	SOL	1308	2.000	42.020	17.530	1.00	0.00
ATOM	6009	OW	SOL	1309	54.420	41.690	8.120	1.00	0.00
ATOM	6010	HW1	SOL	1309	53.860	41.900	7.370	1.00	0.00
ATOM	6011	HW2	SOL	1309	53.940	42.020	8.880	1.00	0.00
ATOM	6012	OW	SOL	1310	5.770	12.390	8.440	1.00	0.00
ATOM	6013	HW1	SOL	1310	5.580	11.850	7.680	1.00	0.00
ATOM	6014	HW2	SOL	1310	6.340	11.840	8.980	1.00	0.00
ATOM	6015	OW	SOL	1311	3.240	4.160	22.910	1.00	0.00
ATOM	6016	HW1	SOL	1311	2.600	3.650	22.410	1.00	0.00
ATOM	6017	HW2	SOL	1311	3.560	4.810	22.300	1.00	0.00
ATOM	6018	OW	SOL	1312	4.960	11.430	2.130	1.00	0.00
ATOM	6019	HW1	SOL	1312	4.840	12.270	1.670	1.00	0.00
ATOM	6020	HW2	SOL	1312	4.540	11.560	2.980	1.00	0.00
ATOM	6021	OW	SOL	1313	26.970	44.860	6.810	1.00	0.00
ATOM	6022	HW1	SOL	1313	26.160	45.320	6.590	1.00	0.00
ATOM	6023	HW2	SOL	1313	27.420	45.460	7.420	1.00	0.00
ATOM	6024	OW	SOL	1314	0.170	54.830	13.290	1.00	0.00
ATOM	6025	HW1	SOL	1314	-0.290	55.510	12.790	1.00	0.00
ATOM	6026	HW2	SOL	1314	0.430	55.270	14.100	1.00	0.00
ATOM	6027	OW	SOL	1315	3.580	54.530	26.220	1.00	0.00

ATOM	6028	HW1	SOL	1315	2.740	54.940	26.450	1.00	0.00
ATOM	6029	HW2	SOL	1315	4.230	55.010	26.740	1.00	0.00
ATOM	6030	OW	SOL	1316	5.120	17.290	51.750	1.00	0.00
ATOM	6031	HW1	SOL	1316	5.920	17.560	52.200	1.00	0.00
ATOM	6032	HW2	SOL	1316	5.330	16.440	51.370	1.00	0.00
ATOM	6033	OW	SOL	1317	2.570	22.670	44.510	1.00	0.00
ATOM	6034	HW1	SOL	1317	2.390	22.030	43.830	1.00	0.00
ATOM	6035	HW2	SOL	1317	2.670	23.500	44.050	1.00	0.00
ATOM	6036	OW	SOL	1318	37.740	40.060	11.820	1.00	0.00
ATOM	6037	HW1	SOL	1318	36.870	39.710	11.620	1.00	0.00
ATOM	6038	HW2	SOL	1318	37.790	40.050	12.780	1.00	0.00
ATOM	6039	OW	SOL	1319	22.170	0.870	15.270	1.00	0.00
ATOM	6040	HW1	SOL	1319	22.810	1.580	15.300	1.00	0.00
ATOM	6041	HW2	SOL	1319	21.420	1.240	14.810	1.00	0.00
ATOM	6042	OW	SOL	1320	11.460	14.310	22.230	1.00	0.00
ATOM	6043	HW1	SOL	1320	10.640	14.250	21.740	1.00	0.00
ATOM	6044	HW2	SOL	1320	11.790	13.410	22.260	1.00	0.00
ATOM	6045	OW	SOL	1321	18.880	46.440	7.690	1.00	0.00
ATOM	6046	HW1	SOL	1321	19.050	47.330	7.990	1.00	0.00
ATOM	6047	HW2	SOL	1321	19.700	45.970	7.850	1.00	0.00
ATOM	6048	OW	SOL	1322	25.950	38.270	2.920	1.00	0.00
ATOM	6049	HW1	SOL	1322	26.470	37.520	2.640	1.00	0.00
ATOM	6050	HW2	SOL	1322	25.180	37.890	3.340	1.00	0.00
ATOM	6051	OW	SOL	1323	29.220	43.940	44.050	1.00	0.00
ATOM	6052	HW1	SOL	1323	28.980	43.110	43.620	1.00	0.00
ATOM	6053	HW2	SOL	1323	30.170	43.890	44.130	1.00	0.00
ATOM	6054	OW	SOL	1324	42.780	29.310	11.660	1.00	0.00
ATOM	6055	HW1	SOL	1324	42.730	30.180	11.260	1.00	0.00
ATOM	6056	HW2	SOL	1324	43.710	29.110	11.690	1.00	0.00
ATOM	6057	OW	SOL	1325	49.340	11.750	25.910	1.00	0.00
ATOM	6058	HW1	SOL	1325	49.870	12.320	25.360	1.00	0.00
ATOM	6059	HW2	SOL	1325	48.730	12.340	26.350	1.00	0.00
ATOM	6060	OW	SOL	1326	39.150	39.720	55.070	1.00	0.00
ATOM	6061	HW1	SOL	1326	38.360	40.090	55.450	1.00	0.00
ATOM	6062	HW2	SOL	1326	39.540	40.450	54.590	1.00	0.00
ATOM	6063	OW	SOL	1327	10.330	20.360	23.540	1.00	0.00
ATOM	6064	HW1	SOL	1327	9.940	21.030	24.090	1.00	0.00
ATOM	6065	HW2	SOL	1327	9.830	19.570	23.720	1.00	0.00
ATOM	6066	OW	SOL	1328	22.310	8.090	14.280	1.00	0.00
ATOM	6067	HW1	SOL	1328	22.590	8.410	13.430	1.00	0.00
ATOM	6068	HW2	SOL	1328	22.180	7.150	14.150	1.00	0.00
ATOM	6069	OW	SOL	1329	39.220	28.250	47.300	1.00	0.00
ATOM	6070	HW1	SOL	1329	39.810	27.800	46.690	1.00	0.00
ATOM	6071	HW2	SOL	1329	38.610	28.720	46.740	1.00	0.00

ATOM	6072	OW	SOL	1330	49.420	46.250	17.470	1.00	0.00
ATOM	6073	HW1	SOL	1330	50.000	45.500	17.610	1.00	0.00
ATOM	6074	HW2	SOL	1330	48.890	46.000	16.710	1.00	0.00
ATOM	6075	OW	SOL	1331	26.710	16.360	35.650	1.00	0.00
ATOM	6076	HW1	SOL	1331	25.870	16.600	36.030	1.00	0.00
ATOM	6077	HW2	SOL	1331	26.490	15.960	34.820	1.00	0.00
ATOM	6078	OW	SOL	1332	55.300	19.090	19.880	1.00	0.00
ATOM	6079	HW1	SOL	1332	56.250	19.210	19.790	1.00	0.00
ATOM	6080	HW2	SOL	1332	55.060	19.620	20.630	1.00	0.00
ATOM	6081	OW	SOL	1333	27.490	27.400	45.550	1.00	0.00
ATOM	6082	HW1	SOL	1333	27.270	26.500	45.790	1.00	0.00
ATOM	6083	HW2	SOL	1333	26.790	27.660	44.950	1.00	0.00
ATOM	6084	OW	SOL	1334	4.880	1.310	27.210	1.00	0.00
ATOM	6085	HW1	SOL	1334	4.280	2.050	27.250	1.00	0.00
ATOM	6086	HW2	SOL	1334	5.700	1.680	26.890	1.00	0.00
ATOM	6087	OW	SOL	1335	44.660	27.380	31.590	1.00	0.00
ATOM	6088	HW1	SOL	1335	44.430	28.280	31.360	1.00	0.00
ATOM	6089	HW2	SOL	1335	43.830	26.970	31.830	1.00	0.00
ATOM	6090	OW	SOL	1336	48.870	41.270	51.940	1.00	0.00
ATOM	6091	HW1	SOL	1336	48.000	41.010	52.220	1.00	0.00
ATOM	6092	HW2	SOL	1336	49.390	40.460	51.980	1.00	0.00
ATOM	6093	OW	SOL	1337	18.590	3.220	27.420	1.00	0.00
ATOM	6094	HW1	SOL	1337	18.960	4.090	27.570	1.00	0.00
ATOM	6095	HW2	SOL	1337	19.330	2.620	27.540	1.00	0.00
ATOM	6096	OW	SOL	1338	9.040	18.030	24.170	1.00	0.00
ATOM	6097	HW1	SOL	1338	9.060	17.210	23.680	1.00	0.00
ATOM	6098	HW2	SOL	1338	8.110	18.140	24.400	1.00	0.00
ATOM	6099	OW	SOL	1339	51.000	6.750	35.460	1.00	0.00
ATOM	6100	HW1	SOL	1339	51.070	7.190	34.610	1.00	0.00
ATOM	6101	HW2	SOL	1339	50.100	6.920	35.740	1.00	0.00
ATOM	6102	OW	SOL	1340	23.380	37.620	8.360	1.00	0.00
ATOM	6103	HW1	SOL	1340	24.060	36.970	8.230	1.00	0.00
ATOM	6104	HW2	SOL	1340	22.980	37.730	7.490	1.00	0.00
ATOM	6105	OW	SOL	1341	30.590	3.190	47.320	1.00	0.00
ATOM	6106	HW1	SOL	1341	30.780	3.960	47.860	1.00	0.00
ATOM	6107	HW2	SOL	1341	29.960	3.510	46.670	1.00	0.00
ATOM	6108	OW	SOL	1342	18.750	48.540	12.860	1.00	0.00
ATOM	6109	HW1	SOL	1342	19.370	48.350	13.560	1.00	0.00
ATOM	6110	HW2	SOL	1342	17.910	48.620	13.310	1.00	0.00
ATOM	6111	OW	SOL	1343	45.870	43.550	27.160	1.00	0.00
ATOM	6112	HW1	SOL	1343	45.110	43.800	26.620	1.00	0.00
ATOM	6113	HW2	SOL	1343	45.500	43.000	27.850	1.00	0.00
ATOM	6114	OW	SOL	1344	55.140	19.480	36.830	1.00	0.00
ATOM	6115	HW1	SOL	1344	55.300	18.570	37.090	1.00	0.00

ATOM	6116	HW2	SOL	1344	54.280	19.690	37.210	1.00	0.00
ATOM	6117	OW	SOL	1345	41.210	18.650	10.810	1.00	0.00
ATOM	6118	HW1	SOL	1345	41.590	19.400	11.270	1.00	0.00
ATOM	6119	HW2	SOL	1345	41.800	18.490	10.070	1.00	0.00
ATOM	6120	OW	SOL	1346	26.090	0.110	45.330	1.00	0.00
ATOM	6121	HW1	SOL	1346	26.510	-0.520	45.920	1.00	0.00
ATOM	6122	HW2	SOL	1346	25.400	0.520	45.860	1.00	0.00
ATOM	6123	OW	SOL	1347	35.250	6.610	48.450	1.00	0.00
ATOM	6124	HW1	SOL	1347	36.010	6.690	47.870	1.00	0.00
ATOM	6125	HW2	SOL	1347	35.380	5.780	48.910	1.00	0.00
ATOM	6126	OW	SOL	1348	5.760	2.520	50.350	1.00	0.00
ATOM	6127	HW1	SOL	1348	5.870	2.180	51.240	1.00	0.00
ATOM	6128	HW2	SOL	1348	5.250	1.850	49.900	1.00	0.00
ATOM	6129	OW	SOL	1349	48.760	14.160	23.650	1.00	0.00
ATOM	6130	HW1	SOL	1349	47.980	13.640	23.450	1.00	0.00
ATOM	6131	HW2	SOL	1349	48.820	14.790	22.930	1.00	0.00
ATOM	6132	OW	SOL	1350	44.250	46.200	51.290	1.00	0.00
ATOM	6133	HW1	SOL	1350	44.390	47.090	50.960	1.00	0.00
ATOM	6134	HW2	SOL	1350	44.840	45.650	50.760	1.00	0.00
ATOM	6135	OW	SOL	1351	32.210	7.780	45.220	1.00	0.00
ATOM	6136	HW1	SOL	1351	32.500	7.970	46.110	1.00	0.00
ATOM	6137	HW2	SOL	1351	31.320	7.460	45.320	1.00	0.00
ATOM	6138	OW	SOL	1352	53.570	12.320	46.940	1.00	0.00
ATOM	6139	HW1	SOL	1352	54.140	11.550	46.880	1.00	0.00
ATOM	6140	HW2	SOL	1352	53.150	12.240	47.800	1.00	0.00
ATOM	6141	OW	SOL	1353	43.310	26.830	22.600	1.00	0.00
ATOM	6142	HW1	SOL	1353	42.430	26.480	22.750	1.00	0.00
ATOM	6143	HW2	SOL	1353	43.220	27.780	22.740	1.00	0.00
ATOM	6144	OW	SOL	1354	30.410	43.510	53.250	1.00	0.00
ATOM	6145	HW1	SOL	1354	31.070	44.200	53.170	1.00	0.00
ATOM	6146	HW2	SOL	1354	29.620	43.970	53.520	1.00	0.00
ATOM	6147	OW	SOL	1355	2.580	42.850	4.410	1.00	0.00
ATOM	6148	HW1	SOL	1355	2.700	41.990	4.010	1.00	0.00
ATOM	6149	HW2	SOL	1355	2.140	43.370	3.730	1.00	0.00
ATOM	6150	OW	SOL	1356	31.600	4.860	7.300	1.00	0.00
ATOM	6151	HW1	SOL	1356	31.730	3.920	7.360	1.00	0.00
ATOM	6152	HW2	SOL	1356	32.200	5.150	6.610	1.00	0.00
ATOM	6153	OW	SOL	1357	26.440	49.010	27.710	1.00	0.00
ATOM	6154	HW1	SOL	1357	25.790	49.090	28.400	1.00	0.00
ATOM	6155	HW2	SOL	1357	26.990	48.270	27.980	1.00	0.00
ATOM	6156	OW	SOL	1358	3.620	28.170	46.130	1.00	0.00
ATOM	6157	HW1	SOL	1358	3.690	28.000	47.070	1.00	0.00
ATOM	6158	HW2	SOL	1358	4.310	28.810	45.950	1.00	0.00
ATOM	6159	OW	SOL	1359	55.650	4.210	8.350	1.00	0.00

ATOM	6160	HW1	SOL	1359	55.990	3.320	8.440	1.00	0.00
ATOM	6161	HW2	SOL	1359	54.700	4.100	8.330	1.00	0.00
ATOM	6162	OW	SOL	1360	13.400	0.450	53.750	1.00	0.00
ATOM	6163	HW1	SOL	1360	14.220	0.660	53.310	1.00	0.00
ATOM	6164	HW2	SOL	1360	13.660	-0.060	54.520	1.00	0.00
ATOM	6165	OW	SOL	1361	34.720	54.600	45.950	1.00	0.00
ATOM	6166	HW1	SOL	1361	34.150	53.920	45.600	1.00	0.00
ATOM	6167	HW2	SOL	1361	35.540	54.150	46.140	1.00	0.00
ATOM	6168	OW	SOL	1362	31.720	9.860	35.620	1.00	0.00
ATOM	6169	HW1	SOL	1362	31.020	9.860	36.280	1.00	0.00
ATOM	6170	HW2	SOL	1362	31.260	9.780	34.790	1.00	0.00
ATOM	6171	OW	SOL	1363	46.240	6.140	25.750	1.00	0.00
ATOM	6172	HW1	SOL	1363	46.700	6.830	26.240	1.00	0.00
ATOM	6173	HW2	SOL	1363	46.830	5.920	25.040	1.00	0.00
ATOM	6174	OW	SOL	1364	0.940	39.630	4.650	1.00	0.00
ATOM	6175	HW1	SOL	1364	1.840	39.910	4.830	1.00	0.00
ATOM	6176	HW2	SOL	1364	0.880	39.590	3.690	1.00	0.00
ATOM	6177	OW	SOL	1365	44.590	26.110	47.630	1.00	0.00
ATOM	6178	HW1	SOL	1365	44.240	26.990	47.740	1.00	0.00
ATOM	6179	HW2	SOL	1365	44.810	25.820	48.510	1.00	0.00
ATOM	6180	OW	SOL	1366	52.940	43.900	55.690	1.00	0.00
ATOM	6181	HW1	SOL	1366	52.930	43.120	55.150	1.00	0.00
ATOM	6182	HW2	SOL	1366	52.550	43.630	56.520	1.00	0.00
ATOM	6183	OW	SOL	1367	15.110	32.110	21.110	1.00	0.00
ATOM	6184	HW1	SOL	1367	14.920	31.180	21.160	1.00	0.00
ATOM	6185	HW2	SOL	1367	14.630	32.420	20.340	1.00	0.00
ATOM	6186	OW	SOL	1368	51.450	1.450	29.050	1.00	0.00
ATOM	6187	HW1	SOL	1368	51.360	0.660	28.520	1.00	0.00
ATOM	6188	HW2	SOL	1368	50.660	1.470	29.580	1.00	0.00
ATOM	6189	OW	SOL	1369	29.570	46.170	23.380	1.00	0.00
ATOM	6190	HW1	SOL	1369	28.960	46.840	23.700	1.00	0.00
ATOM	6191	HW2	SOL	1369	30.150	45.990	24.120	1.00	0.00
ATOM	6192	OW	SOL	1370	1.350	42.310	33.940	1.00	0.00
ATOM	6193	HW1	SOL	1370	2.130	41.950	33.510	1.00	0.00
ATOM	6194	HW2	SOL	1370	1.680	42.850	34.650	1.00	0.00
ATOM	6195	OW	SOL	1371	6.050	52.480	21.000	1.00	0.00
ATOM	6196	HW1	SOL	1371	6.100	52.360	21.950	1.00	0.00
ATOM	6197	HW2	SOL	1371	5.700	53.370	20.890	1.00	0.00
ATOM	6198	OW	SOL	1372	14.060	13.840	53.170	1.00	0.00
ATOM	6199	HW1	SOL	1372	14.260	14.660	53.620	1.00	0.00
ATOM	6200	HW2	SOL	1372	14.910	13.420	53.050	1.00	0.00
ATOM	6201	OW	SOL	1373	19.670	22.800	8.490	1.00	0.00
ATOM	6202	HW1	SOL	1373	19.730	23.570	7.940	1.00	0.00
ATOM	6203	HW2	SOL	1373	20.150	22.120	8.020	1.00	0.00

ATOM	6204	OW	SOL	1374	33.810	50.340	7.070	1.00	0.00
ATOM	6205	HW1	SOL	1374	33.510	50.030	6.210	1.00	0.00
ATOM	6206	HW2	SOL	1374	33.470	49.690	7.690	1.00	0.00
ATOM	6207	OW	SOL	1375	51.470	21.770	13.400	1.00	0.00
ATOM	6208	HW1	SOL	1375	51.950	22.330	14.020	1.00	0.00
ATOM	6209	HW2	SOL	1375	51.650	22.150	12.550	1.00	0.00
ATOM	6210	OW	SOL	1376	43.400	45.400	9.480	1.00	0.00
ATOM	6211	HW1	SOL	1376	43.940	44.840	8.930	1.00	0.00
ATOM	6212	HW2	SOL	1376	43.370	46.240	9.020	1.00	0.00
ATOM	6213	OW	SOL	1377	27.190	51.830	13.430	1.00	0.00
ATOM	6214	HW1	SOL	1377	27.930	51.220	13.400	1.00	0.00
ATOM	6215	HW2	SOL	1377	27.170	52.130	14.340	1.00	0.00
ATOM	6216	OW	SOL	1378	41.580	7.770	18.200	1.00	0.00
ATOM	6217	HW1	SOL	1378	41.550	7.570	17.270	1.00	0.00
ATOM	6218	HW2	SOL	1378	40.820	8.330	18.350	1.00	0.00
ATOM	6219	OW	SOL	1379	49.040	34.060	42.290	1.00	0.00
ATOM	6220	HW1	SOL	1379	48.880	33.300	41.720	1.00	0.00
ATOM	6221	HW2	SOL	1379	48.170	34.290	42.630	1.00	0.00
ATOM	6222	OW	SOL	1380	5.300	31.530	3.050	1.00	0.00
ATOM	6223	HW1	SOL	1380	4.560	32.140	3.140	1.00	0.00
ATOM	6224	HW2	SOL	1380	5.780	31.860	2.300	1.00	0.00
ATOM	6225	OW	SOL	1381	11.390	6.910	29.850	1.00	0.00
ATOM	6226	HW1	SOL	1381	11.240	7.290	28.980	1.00	0.00
ATOM	6227	HW2	SOL	1381	11.790	6.060	29.670	1.00	0.00
ATOM	6228	OW	SOL	1382	15.610	33.700	53.040	1.00	0.00
ATOM	6229	HW1	SOL	1382	16.220	34.100	53.670	1.00	0.00
ATOM	6230	HW2	SOL	1382	15.090	34.430	52.710	1.00	0.00
ATOM	6231	OW	SOL	1383	51.360	52.050	28.770	1.00	0.00
ATOM	6232	HW1	SOL	1383	52.220	51.760	29.100	1.00	0.00
ATOM	6233	HW2	SOL	1383	50.730	51.590	29.320	1.00	0.00
ATOM	6234	OW	SOL	1384	48.450	16.590	43.060	1.00	0.00
ATOM	6235	HW1	SOL	1384	48.690	16.660	43.990	1.00	0.00
ATOM	6236	HW2	SOL	1384	49.290	16.520	42.600	1.00	0.00
ATOM	6237	OW	SOL	1385	51.860	0.970	16.250	1.00	0.00
ATOM	6238	HW1	SOL	1385	52.240	0.220	16.700	1.00	0.00
ATOM	6239	HW2	SOL	1385	51.160	0.600	15.710	1.00	0.00
ATOM	6240	OW	SOL	1386	52.590	41.210	36.570	1.00	0.00
ATOM	6241	HW1	SOL	1386	52.230	40.330	36.650	1.00	0.00
ATOM	6242	HW2	SOL	1386	53.390	41.100	36.060	1.00	0.00
ATOM	6243	OW	SOL	1387	42.840	18.650	30.140	1.00	0.00
ATOM	6244	HW1	SOL	1387	43.250	19.070	29.380	1.00	0.00
ATOM	6245	HW2	SOL	1387	43.410	18.880	30.870	1.00	0.00
ATOM	6246	OW	SOL	1388	15.110	46.360	21.210	1.00	0.00
ATOM	6247	HW1	SOL	1388	14.910	45.540	20.750	1.00	0.00

ATOM	6248	HW2	SOL	1388	15.420	46.090	22.070	1.00	0.00
ATOM	6249	OW	SOL	1389	18.040	19.810	54.530	1.00	0.00
ATOM	6250	HW1	SOL	1389	18.180	20.750	54.440	1.00	0.00
ATOM	6251	HW2	SOL	1389	17.400	19.730	55.240	1.00	0.00
ATOM	6252	OW	SOL	1390	2.490	5.780	26.900	1.00	0.00
ATOM	6253	HW1	SOL	1390	2.680	6.250	27.710	1.00	0.00
ATOM	6254	HW2	SOL	1390	1.570	5.950	26.730	1.00	0.00
ATOM	6255	OW	SOL	1391	26.800	12.030	3.100	1.00	0.00
ATOM	6256	HW1	SOL	1391	27.450	11.540	3.600	1.00	0.00
ATOM	6257	HW2	SOL	1391	26.290	12.500	3.760	1.00	0.00
ATOM	6258	OW	SOL	1392	23.650	15.260	11.160	1.00	0.00
ATOM	6259	HW1	SOL	1392	23.110	15.950	10.770	1.00	0.00
ATOM	6260	HW2	SOL	1392	24.410	15.190	10.590	1.00	0.00
ATOM	6261	OW	SOL	1393	7.980	23.710	17.580	1.00	0.00
ATOM	6262	HW1	SOL	1393	7.520	23.280	18.310	1.00	0.00
ATOM	6263	HW2	SOL	1393	8.900	23.490	17.720	1.00	0.00
ATOM	6264	OW	SOL	1394	50.640	41.570	17.760	1.00	0.00
ATOM	6265	HW1	SOL	1394	51.400	42.140	17.720	1.00	0.00
ATOM	6266	HW2	SOL	1394	50.080	41.980	18.430	1.00	0.00
ATOM	6267	OW	SOL	1395	38.970	0.880	53.350	1.00	0.00
ATOM	6268	HW1	SOL	1395	38.640	0.400	52.590	1.00	0.00
ATOM	6269	HW2	SOL	1395	39.880	1.080	53.130	1.00	0.00
ATOM	6270	OW	SOL	1396	46.720	3.550	26.960	1.00	0.00
ATOM	6271	HW1	SOL	1396	47.250	4.320	27.150	1.00	0.00
ATOM	6272	HW2	SOL	1396	45.950	3.890	26.490	1.00	0.00
ATOM	6273	OW	SOL	1397	28.350	33.660	0.720	1.00	0.00
ATOM	6274	HW1	SOL	1397	28.630	33.730	-0.190	1.00	0.00
ATOM	6275	HW2	SOL	1397	28.880	32.950	1.090	1.00	0.00
ATOM	6276	OW	SOL	1398	19.510	46.120	5.010	1.00	0.00
ATOM	6277	HW1	SOL	1398	20.330	45.810	4.630	1.00	0.00
ATOM	6278	HW2	SOL	1398	19.710	46.250	5.940	1.00	0.00
ATOM	6279	OW	SOL	1399	22.750	42.270	1.710	1.00	0.00
ATOM	6280	HW1	SOL	1399	23.620	42.120	2.080	1.00	0.00
ATOM	6281	HW2	SOL	1399	22.920	42.790	0.920	1.00	0.00
ATOM	6282	OW	SOL	1400	15.390	11.440	42.010	1.00	0.00
ATOM	6283	HW1	SOL	1400	15.900	11.310	42.810	1.00	0.00
ATOM	6284	HW2	SOL	1400	14.850	10.640	41.940	1.00	0.00
ATOM	6285	OW	SOL	1401	21.670	15.390	50.490	1.00	0.00
ATOM	6286	HW1	SOL	1401	21.200	14.580	50.290	1.00	0.00
ATOM	6287	HW2	SOL	1401	21.410	15.600	51.390	1.00	0.00
ATOM	6288	OW	SOL	1402	12.280	42.000	53.320	1.00	0.00
ATOM	6289	HW1	SOL	1402	12.400	42.870	52.950	1.00	0.00
ATOM	6290	HW2	SOL	1402	13.160	41.670	53.480	1.00	0.00
ATOM	6291	OW	SOL	1403	22.900	42.730	53.500	1.00	0.00

ATOM	6292	HW1	SOL	1403	23.740	42.570	53.080	1.00	0.00
ATOM	6293	HW2	SOL	1403	22.320	42.990	52.790	1.00	0.00
ATOM	6294	OW	SOL	1404	53.330	41.200	19.400	1.00	0.00
ATOM	6295	HW1	SOL	1404	53.550	40.410	18.900	1.00	0.00
ATOM	6296	HW2	SOL	1404	52.760	40.890	20.110	1.00	0.00
ATOM	6297	OW	SOL	1405	44.860	23.240	41.780	1.00	0.00
ATOM	6298	HW1	SOL	1405	44.110	23.550	42.290	1.00	0.00
ATOM	6299	HW2	SOL	1405	45.540	23.080	42.440	1.00	0.00
ATOM	6300	OW	SOL	1406	48.540	20.330	24.410	1.00	0.00
ATOM	6301	HW1	SOL	1406	49.190	21.030	24.390	1.00	0.00
ATOM	6302	HW2	SOL	1406	47.720	20.760	24.170	1.00	0.00
ATOM	6303	OW	SOL	1407	50.930	28.900	15.560	1.00	0.00
ATOM	6304	HW1	SOL	1407	50.830	29.850	15.440	1.00	0.00
ATOM	6305	HW2	SOL	1407	51.470	28.620	14.820	1.00	0.00
ATOM	6306	OW	SOL	1408	48.940	38.750	4.620	1.00	0.00
ATOM	6307	HW1	SOL	1408	49.380	38.520	5.430	1.00	0.00
ATOM	6308	HW2	SOL	1408	49.370	38.220	3.950	1.00	0.00
ATOM	6309	OW	SOL	1409	5.610	38.850	34.120	1.00	0.00
ATOM	6310	HW1	SOL	1409	6.260	39.160	34.740	1.00	0.00
ATOM	6311	HW2	SOL	1409	5.410	39.610	33.570	1.00	0.00
ATOM	6312	OW	SOL	1410	6.080	18.260	15.160	1.00	0.00
ATOM	6313	HW1	SOL	1410	6.650	18.580	15.860	1.00	0.00
ATOM	6314	HW2	SOL	1410	6.260	18.840	14.420	1.00	0.00
ATOM	6315	OW	SOL	1411	18.730	4.650	54.740	1.00	0.00
ATOM	6316	HW1	SOL	1411	19.200	4.680	53.910	1.00	0.00
ATOM	6317	HW2	SOL	1411	17.990	5.250	54.610	1.00	0.00
ATOM	6318	OW	SOL	1412	44.730	27.620	6.200	1.00	0.00
ATOM	6319	HW1	SOL	1412	43.990	28.130	6.540	1.00	0.00
ATOM	6320	HW2	SOL	1412	45.390	27.660	6.890	1.00	0.00
ATOM	6321	OW	SOL	1413	49.510	22.440	46.620	1.00	0.00
ATOM	6322	HW1	SOL	1413	50.190	21.900	47.010	1.00	0.00
ATOM	6323	HW2	SOL	1413	49.520	22.210	45.690	1.00	0.00
ATOM	6324	OW	SOL	1414	55.380	25.400	16.910	1.00	0.00
ATOM	6325	HW1	SOL	1414	55.130	25.680	17.790	1.00	0.00
ATOM	6326	HW2	SOL	1414	56.320	25.550	16.870	1.00	0.00
ATOM	6327	OW	SOL	1415	21.760	24.790	54.600	1.00	0.00
ATOM	6328	HW1	SOL	1415	21.720	23.950	54.140	1.00	0.00
ATOM	6329	HW2	SOL	1415	21.250	24.650	55.400	1.00	0.00
ATOM	6330	OW	SOL	1416	2.690	12.570	39.510	1.00	0.00
ATOM	6331	HW1	SOL	1416	2.390	13.280	38.940	1.00	0.00
ATOM	6332	HW2	SOL	1416	3.580	12.830	39.770	1.00	0.00
ATOM	6333	OW	SOL	1417	26.560	31.470	8.580	1.00	0.00
ATOM	6334	HW1	SOL	1417	25.670	31.240	8.320	1.00	0.00
ATOM	6335	HW2	SOL	1417	27.120	31.130	7.880	1.00	0.00

ATOM	6336	OW	SOL	1418	25.460	50.810	43.160	1.00	0.00
ATOM	6337	HW1	SOL	1418	25.320	50.330	42.340	1.00	0.00
ATOM	6338	HW2	SOL	1418	26.390	51.010	43.160	1.00	0.00
ATOM	6339	OW	SOL	1419	42.420	34.790	1.670	1.00	0.00
ATOM	6340	HW1	SOL	1419	43.070	34.150	1.960	1.00	0.00
ATOM	6341	HW2	SOL	1419	42.020	34.390	0.900	1.00	0.00
ATOM	6342	OW	SOL	1420	55.680	18.750	4.650	1.00	0.00
ATOM	6343	HW1	SOL	1420	55.440	18.370	3.810	1.00	0.00
ATOM	6344	HW2	SOL	1420	55.150	18.270	5.290	1.00	0.00
ATOM	6345	OW	SOL	1421	0.240	26.680	38.830	1.00	0.00
ATOM	6346	HW1	SOL	1421	0.460	26.630	39.760	1.00	0.00
ATOM	6347	HW2	SOL	1421	-0.210	27.510	38.730	1.00	0.00
ATOM	6348	OW	SOL	1422	18.770	4.810	41.080	1.00	0.00
ATOM	6349	HW1	SOL	1422	19.450	5.470	40.920	1.00	0.00
ATOM	6350	HW2	SOL	1422	18.120	5.260	41.610	1.00	0.00
ATOM	6351	OW	SOL	1423	22.720	39.880	54.200	1.00	0.00
ATOM	6352	HW1	SOL	1423	23.530	40.000	54.680	1.00	0.00
ATOM	6353	HW2	SOL	1423	22.570	40.710	53.750	1.00	0.00
ATOM	6354	OW	SOL	1424	24.090	44.300	46.040	1.00	0.00
ATOM	6355	HW1	SOL	1424	24.840	44.840	46.280	1.00	0.00
ATOM	6356	HW2	SOL	1424	24.470	43.540	45.610	1.00	0.00
ATOM	6357	OW	SOL	1425	49.500	26.350	7.020	1.00	0.00
ATOM	6358	HW1	SOL	1425	50.330	26.720	6.710	1.00	0.00
ATOM	6359	HW2	SOL	1425	49.740	25.900	7.830	1.00	0.00
ATOM	6360	OW	SOL	1426	8.970	40.560	5.950	1.00	0.00
ATOM	6361	HW1	SOL	1426	9.600	40.420	6.660	1.00	0.00
ATOM	6362	HW2	SOL	1426	9.460	41.030	5.290	1.00	0.00
ATOM	6363	OW	SOL	1427	51.560	45.250	47.270	1.00	0.00
ATOM	6364	HW1	SOL	1427	52.320	45.780	47.030	1.00	0.00
ATOM	6365	HW2	SOL	1427	51.710	45.010	48.180	1.00	0.00
ATOM	6366	OW	SOL	1428	0.130	1.980	26.990	1.00	0.00
ATOM	6367	HW1	SOL	1428	0.650	2.750	27.250	1.00	0.00
ATOM	6368	HW2	SOL	1428	-0.670	2.040	27.510	1.00	0.00
ATOM	6369	OW	SOL	1429	16.220	51.190	47.290	1.00	0.00
ATOM	6370	HW1	SOL	1429	16.990	51.760	47.310	1.00	0.00
ATOM	6371	HW2	SOL	1429	16.390	50.580	46.570	1.00	0.00
ATOM	6372	OW	SOL	1430	15.700	18.340	47.930	1.00	0.00
ATOM	6373	HW1	SOL	1430	15.380	17.790	47.220	1.00	0.00
ATOM	6374	HW2	SOL	1430	15.250	18.020	48.710	1.00	0.00
ATOM	6375	OW	SOL	1431	14.830	2.470	11.800	1.00	0.00
ATOM	6376	HW1	SOL	1431	14.320	2.790	11.050	1.00	0.00
ATOM	6377	HW2	SOL	1431	15.740	2.570	11.540	1.00	0.00
ATOM	6378	OW	SOL	1432	7.970	16.630	0.670	1.00	0.00
ATOM	6379	HW1	SOL	1432	7.810	17.580	0.750	1.00	0.00

ATOM	6380	HW2	SOL	1432	7.670	16.410	-0.210	1.00	0.00
ATOM	6381	OW	SOL	1433	47.710	44.540	42.500	1.00	0.00
ATOM	6382	HW1	SOL	1433	47.890	44.960	43.340	1.00	0.00
ATOM	6383	HW2	SOL	1433	47.390	43.670	42.730	1.00	0.00
ATOM	6384	OW	SOL	1434	7.130	52.860	1.930	1.00	0.00
ATOM	6385	HW1	SOL	1434	8.070	53.030	1.970	1.00	0.00
ATOM	6386	HW2	SOL	1434	6.730	53.740	1.950	1.00	0.00
ATOM	6387	OW	SOL	1435	40.340	42.920	0.990	1.00	0.00
ATOM	6388	HW1	SOL	1435	40.590	43.680	1.510	1.00	0.00
ATOM	6389	HW2	SOL	1435	40.130	42.250	1.650	1.00	0.00
ATOM	6390	OW	SOL	1436	45.350	31.740	38.010	1.00	0.00
ATOM	6391	HW1	SOL	1436	45.580	32.640	37.790	1.00	0.00
ATOM	6392	HW2	SOL	1436	44.670	31.510	37.380	1.00	0.00
ATOM	6393	OW	SOL	1437	52.380	30.840	14.220	1.00	0.00
ATOM	6394	HW1	SOL	1437	51.570	31.340	14.290	1.00	0.00
ATOM	6395	HW2	SOL	1437	53.070	31.510	14.210	1.00	0.00
ATOM	6396	OW	SOL	1438	47.380	18.920	50.680	1.00	0.00
ATOM	6397	HW1	SOL	1438	48.060	18.430	51.150	1.00	0.00
ATOM	6398	HW2	SOL	1438	47.020	18.290	50.070	1.00	0.00
ATOM	6399	OW	SOL	1439	12.150	1.230	16.540	1.00	0.00
ATOM	6400	HW1	SOL	1439	11.870	0.730	15.770	1.00	0.00
ATOM	6401	HW2	SOL	1439	13.050	1.510	16.330	1.00	0.00
ATOM	6402	OW	SOL	1440	14.760	0.390	31.710	1.00	0.00
ATOM	6403	HW1	SOL	1440	14.090	0.350	32.390	1.00	0.00
ATOM	6404	HW2	SOL	1440	15.490	-0.110	32.080	1.00	0.00
ATOM	6405	OW	SOL	1441	0.570	1.580	35.420	1.00	0.00
ATOM	6406	HW1	SOL	1441	-0.370	1.720	35.520	1.00	0.00
ATOM	6407	HW2	SOL	1441	0.930	2.440	35.210	1.00	0.00
ATOM	6408	OW	SOL	1442	2.010	12.170	42.270	1.00	0.00
ATOM	6409	HW1	SOL	1442	2.250	12.620	41.460	1.00	0.00
ATOM	6410	HW2	SOL	1442	1.330	11.550	42.020	1.00	0.00
ATOM	6411	OW	SOL	1443	52.060	31.320	7.090	1.00	0.00
ATOM	6412	HW1	SOL	1443	52.910	31.760	7.110	1.00	0.00
ATOM	6413	HW2	SOL	1443	51.850	31.260	6.160	1.00	0.00
ATOM	6414	OW	SOL	1444	34.640	14.260	33.490	1.00	0.00
ATOM	6415	HW1	SOL	1444	34.350	13.520	34.010	1.00	0.00
ATOM	6416	HW2	SOL	1444	34.970	14.900	34.120	1.00	0.00
ATOM	6417	OW	SOL	1445	43.670	47.230	31.670	1.00	0.00
ATOM	6418	HW1	SOL	1445	44.470	46.950	31.220	1.00	0.00
ATOM	6419	HW2	SOL	1445	43.770	46.890	32.560	1.00	0.00
ATOM	6420	OW	SOL	1446	31.200	51.890	5.690	1.00	0.00
ATOM	6421	HW1	SOL	1446	31.640	52.490	5.090	1.00	0.00
ATOM	6422	HW2	SOL	1446	30.920	51.150	5.140	1.00	0.00
ATOM	6423	OW	SOL	1447	26.800	11.450	32.500	1.00	0.00

ATOM	6424	HW1	SOL	1447	27.610	11.890	32.760	1.00	0.00
ATOM	6425	HW2	SOL	1447	26.430	12.020	31.830	1.00	0.00
ATOM	6426	OW	SOL	1448	34.200	21.720	36.530	1.00	0.00
ATOM	6427	HW1	SOL	1448	33.860	21.280	37.310	1.00	0.00
ATOM	6428	HW2	SOL	1448	34.920	21.150	36.240	1.00	0.00
ATOM	6429	OW	SOL	1449	44.160	27.740	41.170	1.00	0.00
ATOM	6430	HW1	SOL	1449	44.790	27.500	41.850	1.00	0.00
ATOM	6431	HW2	SOL	1449	44.030	28.680	41.300	1.00	0.00
ATOM	6432	OW	SOL	1450	35.490	24.970	54.140	1.00	0.00
ATOM	6433	HW1	SOL	1450	35.660	25.850	53.800	1.00	0.00
ATOM	6434	HW2	SOL	1450	34.870	25.100	54.850	1.00	0.00
ATOM	6435	OW	SOL	1451	44.730	34.180	47.550	1.00	0.00
ATOM	6436	HW1	SOL	1451	44.770	34.140	48.510	1.00	0.00
ATOM	6437	HW2	SOL	1451	45.500	34.690	47.300	1.00	0.00
ATOM	6438	OW	SOL	1452	41.330	4.520	14.440	1.00	0.00
ATOM	6439	HW1	SOL	1452	41.680	3.980	15.140	1.00	0.00
ATOM	6440	HW2	SOL	1452	41.490	4.010	13.640	1.00	0.00
ATOM	6441	OW	SOL	1453	18.840	48.230	53.020	1.00	0.00
ATOM	6442	HW1	SOL	1453	18.000	48.520	52.660	1.00	0.00
ATOM	6443	HW2	SOL	1453	19.000	48.810	53.770	1.00	0.00
ATOM	6444	OW	SOL	1454	46.680	15.280	41.700	1.00	0.00
ATOM	6445	HW1	SOL	1454	47.100	15.250	40.840	1.00	0.00
ATOM	6446	HW2	SOL	1454	47.310	15.750	42.260	1.00	0.00
ATOM	6447	OW	SOL	1455	48.920	0.180	20.450	1.00	0.00
ATOM	6448	HW1	SOL	1455	49.220	1.090	20.400	1.00	0.00
ATOM	6449	HW2	SOL	1455	48.400	0.130	21.250	1.00	0.00
ATOM	6450	OW	SOL	1456	1.070	13.870	24.570	1.00	0.00
ATOM	6451	HW1	SOL	1456	1.500	13.080	24.890	1.00	0.00
ATOM	6452	HW2	SOL	1456	0.130	13.680	24.640	1.00	0.00
ATOM	6453	OW	SOL	1457	40.260	25.410	2.490	1.00	0.00
ATOM	6454	HW1	SOL	1457	40.530	25.040	3.330	1.00	0.00
ATOM	6455	HW2	SOL	1457	40.990	25.980	2.240	1.00	0.00
ATOM	6456	OW	SOL	1458	25.870	44.650	52.760	1.00	0.00
ATOM	6457	HW1	SOL	1458	24.940	44.860	52.650	1.00	0.00
ATOM	6458	HW2	SOL	1458	25.930	43.720	52.570	1.00	0.00
ATOM	6459	OW	SOL	1459	18.250	16.600	34.410	1.00	0.00
ATOM	6460	HW1	SOL	1459	18.260	16.960	35.300	1.00	0.00
ATOM	6461	HW2	SOL	1459	18.620	15.720	34.500	1.00	0.00
ATOM	6462	OW	SOL	1460	16.790	45.720	9.700	1.00	0.00
ATOM	6463	HW1	SOL	1460	17.110	45.030	10.290	1.00	0.00
ATOM	6464	HW2	SOL	1460	17.350	45.650	8.920	1.00	0.00
ATOM	6465	OW	SOL	1461	6.660	22.130	10.040	1.00	0.00
ATOM	6466	HW1	SOL	1461	5.880	21.820	10.490	1.00	0.00
ATOM	6467	HW2	SOL	1461	6.400	22.180	9.120	1.00	0.00

ATOM	6468	OW	SOL	1462	46.630	13.060	54.450	1.00	0.00
ATOM	6469	HW1	SOL	1462	46.640	12.220	53.980	1.00	0.00
ATOM	6470	HW2	SOL	1462	46.230	13.670	53.830	1.00	0.00
ATOM	6471	OW	SOL	1463	54.820	29.270	21.730	1.00	0.00
ATOM	6472	HW1	SOL	1463	55.490	28.590	21.640	1.00	0.00
ATOM	6473	HW2	SOL	1463	54.740	29.640	20.850	1.00	0.00
ATOM	6474	OW	SOL	1464	40.500	5.540	36.980	1.00	0.00
ATOM	6475	HW1	SOL	1464	39.950	4.890	36.540	1.00	0.00
ATOM	6476	HW2	SOL	1464	41.260	5.650	36.400	1.00	0.00
ATOM	6477	OW	SOL	1465	23.680	32.840	20.300	1.00	0.00
ATOM	6478	HW1	SOL	1465	23.940	33.560	20.860	1.00	0.00
ATOM	6479	HW2	SOL	1465	22.790	32.620	20.590	1.00	0.00
ATOM	6480	OW	SOL	1466	9.560	33.240	18.110	1.00	0.00
ATOM	6481	HW1	SOL	1466	9.020	32.890	17.410	1.00	0.00
ATOM	6482	HW2	SOL	1466	9.840	32.480	18.610	1.00	0.00
ATOM	6483	OW	SOL	1467	46.830	36.940	41.600	1.00	0.00
ATOM	6484	HW1	SOL	1467	46.880	36.210	42.230	1.00	0.00
ATOM	6485	HW2	SOL	1467	46.960	37.720	42.140	1.00	0.00
ATOM	6486	OW	SOL	1468	5.340	44.560	53.080	1.00	0.00
ATOM	6487	HW1	SOL	1468	5.850	43.780	52.880	1.00	0.00
ATOM	6488	HW2	SOL	1468	5.420	45.110	52.300	1.00	0.00
ATOM	6489	OW	SOL	1469	6.770	22.970	44.770	1.00	0.00
ATOM	6490	HW1	SOL	1469	6.870	22.160	44.260	1.00	0.00
ATOM	6491	HW2	SOL	1469	7.140	23.650	44.210	1.00	0.00
ATOM	6492	OW	SOL	1470	26.010	43.360	48.870	1.00	0.00
ATOM	6493	HW1	SOL	1470	26.220	42.890	48.060	1.00	0.00
ATOM	6494	HW2	SOL	1470	26.830	43.750	49.140	1.00	0.00
ATOM	6495	OW	SOL	1471	28.470	4.120	14.190	1.00	0.00
ATOM	6496	HW1	SOL	1471	28.000	4.790	13.690	1.00	0.00
ATOM	6497	HW2	SOL	1471	28.990	3.650	13.540	1.00	0.00
ATOM	6498	OW	SOL	1472	2.200	50.060	40.890	1.00	0.00
ATOM	6499	HW1	SOL	1472	1.930	49.180	41.180	1.00	0.00
ATOM	6500	HW2	SOL	1472	3.120	50.120	41.130	1.00	0.00
ATOM	6501	OW	SOL	1473	5.340	44.050	26.600	1.00	0.00
ATOM	6502	HW1	SOL	1473	4.400	44.150	26.750	1.00	0.00
ATOM	6503	HW2	SOL	1473	5.400	43.650	25.740	1.00	0.00
ATOM	6504	OW	SOL	1474	0.740	40.930	52.540	1.00	0.00
ATOM	6505	HW1	SOL	1474	1.320	41.670	52.710	1.00	0.00
ATOM	6506	HW2	SOL	1474	1.110	40.500	51.770	1.00	0.00
ATOM	6507	OW	SOL	1475	50.480	24.760	0.700	1.00	0.00
ATOM	6508	HW1	SOL	1475	49.940	24.800	1.490	1.00	0.00
ATOM	6509	HW2	SOL	1475	50.430	25.640	0.340	1.00	0.00
ATOM	6510	OW	SOL	1476	48.400	18.300	45.780	1.00	0.00
ATOM	6511	HW1	SOL	1476	48.910	18.430	46.570	1.00	0.00

ATOM	6512	HW2	SOL	1476	47.680	17.730	46.050	1.00	0.00
ATOM	6513	OW	SOL	1477	34.360	3.170	16.920	1.00	0.00
ATOM	6514	HW1	SOL	1477	33.700	3.000	17.590	1.00	0.00
ATOM	6515	HW2	SOL	1477	34.070	3.990	16.510	1.00	0.00
ATOM	6516	OW	SOL	1478	25.200	10.590	23.460	1.00	0.00
ATOM	6517	HW1	SOL	1478	24.640	9.840	23.660	1.00	0.00
ATOM	6518	HW2	SOL	1478	25.920	10.530	24.090	1.00	0.00
ATOM	6519	OW	SOL	1479	20.280	31.390	41.440	1.00	0.00
ATOM	6520	HW1	SOL	1479	20.560	30.770	42.110	1.00	0.00
ATOM	6521	HW2	SOL	1479	19.390	31.650	41.700	1.00	0.00
ATOM	6522	OW	SOL	1480	42.600	29.340	16.680	1.00	0.00
ATOM	6523	HW1	SOL	1480	41.950	29.010	16.050	1.00	0.00
ATOM	6524	HW2	SOL	1480	43.430	29.290	16.210	1.00	0.00
ATOM	6525	OW	SOL	1481	0.520	23.730	49.990	1.00	0.00
ATOM	6526	HW1	SOL	1481	0.030	24.150	50.690	1.00	0.00
ATOM	6527	HW2	SOL	1481	0.760	22.870	50.340	1.00	0.00
ATOM	6528	OW	SOL	1482	32.050	16.440	21.560	1.00	0.00
ATOM	6529	HW1	SOL	1482	31.330	17.050	21.720	1.00	0.00
ATOM	6530	HW2	SOL	1482	32.840	16.980	21.590	1.00	0.00
ATOM	6531	OW	SOL	1483	18.420	50.270	10.740	1.00	0.00
ATOM	6532	HW1	SOL	1483	18.460	49.700	11.510	1.00	0.00
ATOM	6533	HW2	SOL	1483	19.000	51.010	10.960	1.00	0.00
ATOM	6534	OW	SOL	1484	25.070	13.610	5.600	1.00	0.00
ATOM	6535	HW1	SOL	1484	24.260	14.080	5.410	1.00	0.00
ATOM	6536	HW2	SOL	1484	25.700	14.310	5.800	1.00	0.00
ATOM	6537	OW	SOL	1485	2.490	51.790	43.410	1.00	0.00
ATOM	6538	HW1	SOL	1485	2.340	50.870	43.190	1.00	0.00
ATOM	6539	HW2	SOL	1485	2.050	51.910	44.250	1.00	0.00
ATOM	6540	OW	SOL	1486	45.220	13.690	5.890	1.00	0.00
ATOM	6541	HW1	SOL	1486	44.800	13.590	6.740	1.00	0.00
ATOM	6542	HW2	SOL	1486	45.880	13.000	5.860	1.00	0.00
ATOM	6543	OW	SOL	1487	29.730	55.050	18.050	1.00	0.00
ATOM	6544	HW1	SOL	1487	29.510	55.270	17.140	1.00	0.00
ATOM	6545	HW2	SOL	1487	29.580	54.110	18.110	1.00	0.00
ATOM	6546	OW	SOL	1488	47.530	31.220	15.520	1.00	0.00
ATOM	6547	HW1	SOL	1488	46.750	31.420	15.000	1.00	0.00
ATOM	6548	HW2	SOL	1488	47.410	31.730	16.320	1.00	0.00
ATOM	6549	OW	SOL	1489	46.480	48.100	14.000	1.00	0.00
ATOM	6550	HW1	SOL	1489	46.010	47.680	13.270	1.00	0.00
ATOM	6551	HW2	SOL	1489	46.740	48.950	13.650	1.00	0.00
ATOM	6552	OW	SOL	1490	28.050	14.610	48.200	1.00	0.00
ATOM	6553	HW1	SOL	1490	27.460	13.850	48.300	1.00	0.00
ATOM	6554	HW2	SOL	1490	27.470	15.370	48.250	1.00	0.00
ATOM	6555	OW	SOL	1491	10.880	19.850	28.320	1.00	0.00

ATOM	6556	HW1	SOL	1491	11.380	20.550	28.730	1.00	0.00
ATOM	6557	HW2	SOL	1491	11.480	19.470	27.680	1.00	0.00
ATOM	6558	OW	SOL	1492	8.040	45.380	17.180	1.00	0.00
ATOM	6559	HW1	SOL	1492	7.260	45.120	16.690	1.00	0.00
ATOM	6560	HW2	SOL	1492	7.870	46.290	17.440	1.00	0.00
ATOM	6561	OW	SOL	1493	5.370	53.010	50.320	1.00	0.00
ATOM	6562	HW1	SOL	1493	5.400	52.390	51.050	1.00	0.00
ATOM	6563	HW2	SOL	1493	6.040	53.660	50.520	1.00	0.00
ATOM	6564	OW	SOL	1494	22.310	29.080	8.760	1.00	0.00
ATOM	6565	HW1	SOL	1494	21.780	28.890	7.980	1.00	0.00
ATOM	6566	HW2	SOL	1494	22.590	28.220	9.080	1.00	0.00
ATOM	6567	OW	SOL	1495	17.190	25.780	19.220	1.00	0.00
ATOM	6568	HW1	SOL	1495	17.150	26.540	18.630	1.00	0.00
ATOM	6569	HW2	SOL	1495	16.340	25.790	19.670	1.00	0.00
ATOM	6570	OW	SOL	1496	27.120	46.280	3.950	1.00	0.00
ATOM	6571	HW1	SOL	1496	26.210	46.000	3.950	1.00	0.00
ATOM	6572	HW2	SOL	1496	27.400	46.170	4.860	1.00	0.00
ATOM	6573	OW	SOL	1497	6.490	23.240	24.960	1.00	0.00
ATOM	6574	HW1	SOL	1497	5.950	23.860	25.450	1.00	0.00
ATOM	6575	HW2	SOL	1497	7.300	23.160	25.470	1.00	0.00
ATOM	6576	OW	SOL	1498	21.150	12.340	41.160	1.00	0.00
ATOM	6577	HW1	SOL	1498	20.450	13.000	41.220	1.00	0.00
ATOM	6578	HW2	SOL	1498	21.930	12.850	40.950	1.00	0.00
ATOM	6579	OW	SOL	1499	24.990	11.730	54.810	1.00	0.00
ATOM	6580	HW1	SOL	1499	25.930	11.620	54.990	1.00	0.00
ATOM	6581	HW2	SOL	1499	24.820	12.640	55.020	1.00	0.00
ATOM	6582	OW	SOL	1500	20.350	24.980	45.250	1.00	0.00
ATOM	6583	HW1	SOL	1500	20.000	24.440	44.540	1.00	0.00
ATOM	6584	HW2	SOL	1500	19.650	24.970	45.910	1.00	0.00
ATOM	6585	OW	SOL	1501	6.580	12.700	13.200	1.00	0.00
ATOM	6586	HW1	SOL	1501	5.700	13.070	13.120	1.00	0.00
ATOM	6587	HW2	SOL	1501	6.750	12.700	14.140	1.00	0.00
ATOM	6588	OW	SOL	1502	13.330	9.390	0.910	1.00	0.00
ATOM	6589	HW1	SOL	1502	12.950	9.300	1.780	1.00	0.00
ATOM	6590	HW2	SOL	1502	12.910	10.170	0.540	1.00	0.00
ATOM	6591	OW	SOL	1503	52.260	1.980	50.490	1.00	0.00
ATOM	6592	HW1	SOL	1503	52.830	2.480	49.900	1.00	0.00
ATOM	6593	HW2	SOL	1503	52.280	1.090	50.140	1.00	0.00
ATOM	6594	OW	SOL	1504	22.700	20.860	24.070	1.00	0.00
ATOM	6595	HW1	SOL	1504	21.990	21.490	23.950	1.00	0.00
ATOM	6596	HW2	SOL	1504	22.340	20.210	24.670	1.00	0.00
ATOM	6597	OW	SOL	1505	48.530	41.670	5.260	1.00	0.00
ATOM	6598	HW1	SOL	1505	48.660	41.170	6.070	1.00	0.00
ATOM	6599	HW2	SOL	1505	47.590	41.600	5.090	1.00	0.00

ATOM	6600	OW	SOL	1506	29.620	12.140	44.870	1.00	0.00
ATOM	6601	HW1	SOL	1506	29.740	11.610	44.070	1.00	0.00
ATOM	6602	HW2	SOL	1506	30.350	12.760	44.850	1.00	0.00
ATOM	6603	OW	SOL	1507	32.630	50.670	23.630	1.00	0.00
ATOM	6604	HW1	SOL	1507	33.380	50.500	23.060	1.00	0.00
ATOM	6605	HW2	SOL	1507	32.760	51.560	23.940	1.00	0.00
ATOM	6606	OW	SOL	1508	51.190	28.250	25.160	1.00	0.00
ATOM	6607	HW1	SOL	1508	51.580	29.030	25.550	1.00	0.00
ATOM	6608	HW2	SOL	1508	50.350	28.150	25.620	1.00	0.00
ATOM	6609	OW	SOL	1509	37.670	3.690	8.980	1.00	0.00
ATOM	6610	HW1	SOL	1509	36.930	4.280	9.050	1.00	0.00
ATOM	6611	HW2	SOL	1509	37.290	2.830	8.830	1.00	0.00
ATOM	6612	OW	SOL	1510	13.050	2.240	19.790	1.00	0.00
ATOM	6613	HW1	SOL	1510	12.850	1.310	19.950	1.00	0.00
ATOM	6614	HW2	SOL	1510	12.530	2.710	20.420	1.00	0.00
ATOM	6615	OW	SOL	1511	18.670	6.450	34.270	1.00	0.00
ATOM	6616	HW1	SOL	1511	18.240	5.620	34.490	1.00	0.00
ATOM	6617	HW2	SOL	1511	17.990	6.960	33.830	1.00	0.00
ATOM	6618	OW	SOL	1512	14.290	13.230	48.310	1.00	0.00
ATOM	6619	HW1	SOL	1512	14.680	12.420	48.640	1.00	0.00
ATOM	6620	HW2	SOL	1512	14.270	13.810	49.070	1.00	0.00
ATOM	6621	OW	SOL	1513	33.070	6.230	40.890	1.00	0.00
ATOM	6622	HW1	SOL	1513	33.660	5.570	40.520	1.00	0.00
ATOM	6623	HW2	SOL	1513	33.320	6.290	41.810	1.00	0.00
ATOM	6624	OW	SOL	1514	35.230	8.470	54.730	1.00	0.00
ATOM	6625	HW1	SOL	1514	34.650	9.040	54.230	1.00	0.00
ATOM	6626	HW2	SOL	1514	35.640	7.910	54.070	1.00	0.00
ATOM	6627	OW	SOL	1515	54.760	42.240	22.010	1.00	0.00
ATOM	6628	HW1	SOL	1515	53.860	42.100	21.720	1.00	0.00
ATOM	6629	HW2	SOL	1515	54.780	43.160	22.280	1.00	0.00
ATOM	6630	OW	SOL	1516	6.220	28.600	2.100	1.00	0.00
ATOM	6631	HW1	SOL	1516	6.340	29.380	2.650	1.00	0.00
ATOM	6632	HW2	SOL	1516	6.590	27.880	2.620	1.00	0.00
ATOM	6633	OW	SOL	1517	36.820	15.550	1.800	1.00	0.00
ATOM	6634	HW1	SOL	1517	37.100	15.850	2.660	1.00	0.00
ATOM	6635	HW2	SOL	1517	36.140	16.180	1.540	1.00	0.00
ATOM	6636	OW	SOL	1518	33.470	25.200	8.200	1.00	0.00
ATOM	6637	HW1	SOL	1518	32.710	24.630	8.060	1.00	0.00
ATOM	6638	HW2	SOL	1518	34.180	24.790	7.700	1.00	0.00
ATOM	6639	OW	SOL	1519	11.770	6.960	52.910	1.00	0.00
ATOM	6640	HW1	SOL	1519	12.610	7.150	53.340	1.00	0.00
ATOM	6641	HW2	SOL	1519	11.240	6.550	53.590	1.00	0.00
ATOM	6642	OW	SOL	1520	48.330	54.200	0.130	1.00	0.00
ATOM	6643	HW1	SOL	1520	49.110	54.730	0.250	1.00	0.00

ATOM	6644	HW2	SOL	1520	48.630	53.300	0.220	1.00	0.00
ATOM	6645	OW	SOL	1521	41.720	40.260	1.520	1.00	0.00
ATOM	6646	HW1	SOL	1521	42.040	39.360	1.550	1.00	0.00
ATOM	6647	HW2	SOL	1521	40.950	40.260	2.080	1.00	0.00
ATOM	6648	OW	SOL	1522	6.790	43.960	28.980	1.00	0.00
ATOM	6649	HW1	SOL	1522	7.640	44.300	28.700	1.00	0.00
ATOM	6650	HW2	SOL	1522	6.470	43.480	28.220	1.00	0.00
ATOM	6651	OW	SOL	1523	32.770	9.740	14.660	1.00	0.00
ATOM	6652	HW1	SOL	1523	32.430	9.020	14.130	1.00	0.00
ATOM	6653	HW2	SOL	1523	33.660	9.480	14.880	1.00	0.00
ATOM	6654	OW	SOL	1524	49.980	9.730	12.600	1.00	0.00
ATOM	6655	HW1	SOL	1524	49.020	9.700	12.660	1.00	0.00
ATOM	6656	HW2	SOL	1524	50.160	10.530	12.120	1.00	0.00
ATOM	6657	OW	SOL	1525	48.980	5.610	39.010	1.00	0.00
ATOM	6658	HW1	SOL	1525	48.250	5.860	38.440	1.00	0.00
ATOM	6659	HW2	SOL	1525	49.220	4.730	38.710	1.00	0.00
ATOM	6660	OW	SOL	1526	13.020	21.420	48.580	1.00	0.00
ATOM	6661	HW1	SOL	1526	13.000	20.490	48.330	1.00	0.00
ATOM	6662	HW2	SOL	1526	12.340	21.830	48.060	1.00	0.00
ATOM	6663	OW	SOL	1527	26.510	24.660	55.210	1.00	0.00
ATOM	6664	HW1	SOL	1527	26.880	23.790	55.310	1.00	0.00
ATOM	6665	HW2	SOL	1527	25.680	24.620	55.690	1.00	0.00
ATOM	6666	OW	SOL	1528	3.300	13.000	19.060	1.00	0.00
ATOM	6667	HW1	SOL	1528	2.590	12.690	18.500	1.00	0.00
ATOM	6668	HW2	SOL	1528	3.810	12.210	19.270	1.00	0.00
ATOM	6669	OW	SOL	1529	4.190	16.170	54.110	1.00	0.00
ATOM	6670	HW1	SOL	1529	3.340	16.150	54.550	1.00	0.00
ATOM	6671	HW2	SOL	1529	3.990	16.480	53.230	1.00	0.00
ATOM	6672	OW	SOL	1530	41.510	51.290	46.240	1.00	0.00
ATOM	6673	HW1	SOL	1530	41.080	50.480	46.510	1.00	0.00
ATOM	6674	HW2	SOL	1530	41.650	51.190	45.300	1.00	0.00
ATOM	6675	OW	SOL	1531	36.750	40.860	47.800	1.00	0.00
ATOM	6676	HW1	SOL	1531	36.240	41.370	48.420	1.00	0.00
ATOM	6677	HW2	SOL	1531	36.110	40.320	47.350	1.00	0.00
ATOM	6678	OW	SOL	1532	47.260	54.680	45.180	1.00	0.00
ATOM	6679	HW1	SOL	1532	47.560	53.780	45.320	1.00	0.00
ATOM	6680	HW2	SOL	1532	47.890	55.040	44.560	1.00	0.00
ATOM	6681	OW	SOL	1533	40.530	28.670	6.950	1.00	0.00
ATOM	6682	HW1	SOL	1533	41.160	28.630	7.680	1.00	0.00
ATOM	6683	HW2	SOL	1533	39.980	29.420	7.150	1.00	0.00
ATOM	6684	OW	SOL	1534	29.860	20.000	8.030	1.00	0.00
ATOM	6685	HW1	SOL	1534	29.460	19.780	8.870	1.00	0.00
ATOM	6686	HW2	SOL	1534	29.450	20.830	7.780	1.00	0.00
ATOM	6687	OW	SOL	1535	38.430	26.410	31.160	1.00	0.00

ATOM	6688	HW1	SOL	1535	37.870	27.160	30.990	1.00	0.00
ATOM	6689	HW2	SOL	1535	38.090	26.030	31.970	1.00	0.00
ATOM	6690	OW	SOL	1536	51.120	51.310	4.650	1.00	0.00
ATOM	6691	HW1	SOL	1536	50.480	51.880	5.080	1.00	0.00
ATOM	6692	HW2	SOL	1536	50.950	50.440	5.010	1.00	0.00
ATOM	6693	OW	SOL	1537	13.580	24.890	22.590	1.00	0.00
ATOM	6694	HW1	SOL	1537	13.470	24.020	22.970	1.00	0.00
ATOM	6695	HW2	SOL	1537	14.260	25.300	23.130	1.00	0.00
ATOM	6696	OW	SOL	1538	0.130	26.460	19.460	1.00	0.00
ATOM	6697	HW1	SOL	1538	-0.770	26.520	19.750	1.00	0.00
ATOM	6698	HW2	SOL	1538	0.570	25.930	20.130	1.00	0.00
ATOM	6699	OW	SOL	1539	13.980	46.170	45.480	1.00	0.00
ATOM	6700	HW1	SOL	1539	14.220	45.300	45.800	1.00	0.00
ATOM	6701	HW2	SOL	1539	14.790	46.540	45.130	1.00	0.00
ATOM	6702	OW	SOL	1540	26.160	32.470	48.580	1.00	0.00
ATOM	6703	HW1	SOL	1540	26.070	31.530	48.400	1.00	0.00
ATOM	6704	HW2	SOL	1540	25.480	32.890	48.050	1.00	0.00
ATOM	6705	OW	SOL	1541	10.560	30.940	20.190	1.00	0.00
ATOM	6706	HW1	SOL	1541	11.010	31.710	20.530	1.00	0.00
ATOM	6707	HW2	SOL	1541	11.130	30.620	19.490	1.00	0.00
ATOM	6708	OW	SOL	1542	5.300	0.090	18.230	1.00	0.00
ATOM	6709	HW1	SOL	1542	4.390	0.060	18.530	1.00	0.00
ATOM	6710	HW2	SOL	1542	5.260	-0.170	17.310	1.00	0.00
ATOM	6711	OW	SOL	1543	23.030	10.880	48.730	1.00	0.00
ATOM	6712	HW1	SOL	1543	22.480	10.770	47.960	1.00	0.00
ATOM	6713	HW2	SOL	1543	23.460	11.720	48.600	1.00	0.00
ATOM	6714	OW	SOL	1544	4.890	35.740	52.060	1.00	0.00
ATOM	6715	HW1	SOL	1544	4.020	35.720	51.650	1.00	0.00
ATOM	6716	HW2	SOL	1544	4.720	35.950	52.970	1.00	0.00
ATOM	6717	OW	SOL	1545	25.200	26.700	28.360	1.00	0.00
ATOM	6718	HW1	SOL	1545	26.010	26.180	28.360	1.00	0.00
ATOM	6719	HW2	SOL	1545	25.350	27.370	29.030	1.00	0.00
ATOM	6720	OW	SOL	1546	55.040	51.320	16.670	1.00	0.00
ATOM	6721	HW1	SOL	1546	55.900	51.340	17.100	1.00	0.00
ATOM	6722	HW2	SOL	1546	55.220	50.990	15.790	1.00	0.00
ATOM	6723	OW	SOL	1547	39.190	38.760	3.610	1.00	0.00
ATOM	6724	HW1	SOL	1547	39.230	37.920	3.170	1.00	0.00
ATOM	6725	HW2	SOL	1547	39.270	38.550	4.540	1.00	0.00
ATOM	6726	OW	SOL	1548	5.820	27.240	29.050	1.00	0.00
ATOM	6727	HW1	SOL	1548	5.810	27.870	29.760	1.00	0.00
ATOM	6728	HW2	SOL	1548	6.180	27.720	28.300	1.00	0.00
ATOM	6729	OW	SOL	1549	27.450	13.570	9.650	1.00	0.00
ATOM	6730	HW1	SOL	1549	27.640	12.980	8.920	1.00	0.00
ATOM	6731	HW2	SOL	1549	27.080	13.010	10.320	1.00	0.00

ATOM	6732	OW	SOL	1550	3.100	44.080	24.260	1.00	0.00
ATOM	6733	HW1	SOL	1550	4.040	44.160	24.110	1.00	0.00
ATOM	6734	HW2	SOL	1550	2.710	44.560	23.520	1.00	0.00
ATOM	6735	OW	SOL	1551	55.670	30.520	47.190	1.00	0.00
ATOM	6736	HW1	SOL	1551	55.850	31.370	46.770	1.00	0.00
ATOM	6737	HW2	SOL	1551	56.290	30.470	47.910	1.00	0.00
ATOM	6738	OW	SOL	1552	9.950	31.160	8.640	1.00	0.00
ATOM	6739	HW1	SOL	1552	10.230	30.410	8.110	1.00	0.00
ATOM	6740	HW2	SOL	1552	9.280	30.810	9.220	1.00	0.00
ATOM	6741	OW	SOL	1553	26.170	5.640	45.920	1.00	0.00
ATOM	6742	HW1	SOL	1553	25.790	6.410	46.340	1.00	0.00
ATOM	6743	HW2	SOL	1553	25.430	5.150	45.580	1.00	0.00
ATOM	6744	OW	SOL	1554	23.210	40.260	27.470	1.00	0.00
ATOM	6745	HW1	SOL	1554	23.930	39.670	27.230	1.00	0.00
ATOM	6746	HW2	SOL	1554	22.480	39.970	26.930	1.00	0.00
ATOM	6747	OW	SOL	1555	29.510	20.440	22.840	1.00	0.00
ATOM	6748	HW1	SOL	1555	29.620	19.690	22.270	1.00	0.00
ATOM	6749	HW2	SOL	1555	28.930	20.140	23.540	1.00	0.00
ATOM	6750	OW	SOL	1556	24.900	38.870	51.650	1.00	0.00
ATOM	6751	HW1	SOL	1556	25.620	38.500	52.150	1.00	0.00
ATOM	6752	HW2	SOL	1556	24.260	38.150	51.580	1.00	0.00
ATOM	6753	OW	SOL	1557	22.980	17.880	27.770	1.00	0.00
ATOM	6754	HW1	SOL	1557	22.670	18.790	27.810	1.00	0.00
ATOM	6755	HW2	SOL	1557	22.290	17.420	27.300	1.00	0.00
ATOM	6756	OW	SOL	1558	10.740	55.390	5.120	1.00	0.00
ATOM	6757	HW1	SOL	1558	10.470	55.750	5.960	1.00	0.00
ATOM	6758	HW2	SOL	1558	11.680	55.570	5.070	1.00	0.00
ATOM	6759	OW	SOL	1559	14.070	45.320	8.510	1.00	0.00
ATOM	6760	HW1	SOL	1559	14.890	45.360	9.010	1.00	0.00
ATOM	6761	HW2	SOL	1559	14.330	45.530	7.610	1.00	0.00
ATOM	6762	OW	SOL	1560	2.860	13.310	32.600	1.00	0.00
ATOM	6763	HW1	SOL	1560	3.490	12.600	32.730	1.00	0.00
ATOM	6764	HW2	SOL	1560	3.300	14.090	32.930	1.00	0.00
ATOM	6765	OW	SOL	1561	9.890	17.450	42.740	1.00	0.00
ATOM	6766	HW1	SOL	1561	9.280	17.670	42.050	1.00	0.00
ATOM	6767	HW2	SOL	1561	9.870	16.500	42.800	1.00	0.00
ATOM	6768	OW	SOL	1562	5.690	8.750	1.360	1.00	0.00
ATOM	6769	HW1	SOL	1562	6.300	8.680	0.620	1.00	0.00
ATOM	6770	HW2	SOL	1562	5.450	9.680	1.380	1.00	0.00
ATOM	6771	OW	SOL	1563	54.490	7.810	41.410	1.00	0.00
ATOM	6772	HW1	SOL	1563	54.880	8.620	41.730	1.00	0.00
ATOM	6773	HW2	SOL	1563	54.620	7.840	40.460	1.00	0.00
ATOM	6774	OW	SOL	1564	49.340	3.870	31.970	1.00	0.00
ATOM	6775	HW1	SOL	1564	49.450	3.760	31.030	1.00	0.00

ATOM	6776	HW2	SOL	1564	48.920	4.720	32.060	1.00	0.00
ATOM	6777	OW	SOL	1565	39.380	29.510	40.540	1.00	0.00
ATOM	6778	HW1	SOL	1565	39.780	29.310	39.700	1.00	0.00
ATOM	6779	HW2	SOL	1565	38.470	29.240	40.450	1.00	0.00
ATOM	6780	OW	SOL	1566	51.830	21.190	21.170	1.00	0.00
ATOM	6781	HW1	SOL	1566	52.460	20.680	21.680	1.00	0.00
ATOM	6782	HW2	SOL	1566	51.000	20.730	21.270	1.00	0.00
ATOM	6783	OW	SOL	1567	51.100	23.010	40.600	1.00	0.00
ATOM	6784	HW1	SOL	1567	51.200	22.230	41.130	1.00	0.00
ATOM	6785	HW2	SOL	1567	50.400	22.790	39.980	1.00	0.00
ATOM	6786	OW	SOL	1568	46.260	17.990	12.030	1.00	0.00
ATOM	6787	HW1	SOL	1568	47.040	18.520	12.210	1.00	0.00
ATOM	6788	HW2	SOL	1568	45.740	18.530	11.420	1.00	0.00
ATOM	6789	OW	SOL	1569	24.520	35.930	49.700	1.00	0.00
ATOM	6790	HW1	SOL	1569	23.720	35.740	50.200	1.00	0.00
ATOM	6791	HW2	SOL	1569	24.380	35.520	48.850	1.00	0.00
ATOM	6792	OW	SOL	1570	8.260	41.300	14.290	1.00	0.00
ATOM	6793	HW1	SOL	1570	7.950	41.950	14.930	1.00	0.00
ATOM	6794	HW2	SOL	1570	9.190	41.220	14.460	1.00	0.00
ATOM	6795	OW	SOL	1571	48.820	12.740	35.150	1.00	0.00
ATOM	6796	HW1	SOL	1571	48.680	13.370	35.870	1.00	0.00
ATOM	6797	HW2	SOL	1571	49.260	13.260	34.470	1.00	0.00
ATOM	6798	OW	SOL	1572	43.370	31.280	6.450	1.00	0.00
ATOM	6799	HW1	SOL	1572	43.460	31.200	5.500	1.00	0.00
ATOM	6800	HW2	SOL	1572	42.520	31.680	6.580	1.00	0.00
ATOM	6801	OW	SOL	1573	3.240	28.710	16.250	1.00	0.00
ATOM	6802	HW1	SOL	1573	4.020	28.630	15.690	1.00	0.00
ATOM	6803	HW2	SOL	1573	3.530	29.230	16.990	1.00	0.00
ATOM	6804	OW	SOL	1574	2.080	45.640	39.740	1.00	0.00
ATOM	6805	HW1	SOL	1574	2.200	46.110	38.910	1.00	0.00
ATOM	6806	HW2	SOL	1574	1.800	44.760	39.470	1.00	0.00
ATOM	6807	OW	SOL	1575	53.320	40.300	24.800	1.00	0.00
ATOM	6808	HW1	SOL	1575	53.560	40.490	25.700	1.00	0.00
ATOM	6809	HW2	SOL	1575	54.100	39.880	24.430	1.00	0.00
ATOM	6810	OW	SOL	1576	16.020	23.920	40.320	1.00	0.00
ATOM	6811	HW1	SOL	1576	16.580	23.380	39.760	1.00	0.00
ATOM	6812	HW2	SOL	1576	15.280	23.350	40.540	1.00	0.00
ATOM	6813	OW	SOL	1577	42.510	25.450	53.070	1.00	0.00
ATOM	6814	HW1	SOL	1577	42.470	26.120	52.390	1.00	0.00
ATOM	6815	HW2	SOL	1577	43.450	25.340	53.230	1.00	0.00
ATOM	6816	OW	SOL	1578	41.030	21.880	18.420	1.00	0.00
ATOM	6817	HW1	SOL	1578	41.560	21.790	17.630	1.00	0.00
ATOM	6818	HW2	SOL	1578	40.600	22.720	18.320	1.00	0.00
ATOM	6819	OW	SOL	1579	36.500	16.980	11.290	1.00	0.00

ATOM	6820	HW1	SOL	1579	36.270	16.800	12.210	1.00	0.00
ATOM	6821	HW2	SOL	1579	37.160	16.320	11.080	1.00	0.00
ATOM	6822	OW	SOL	1580	50.860	12.410	0.350	1.00	0.00
ATOM	6823	HW1	SOL	1580	50.550	12.660	1.220	1.00	0.00
ATOM	6824	HW2	SOL	1580	51.810	12.340	0.450	1.00	0.00
ATOM	6825	OW	SOL	1581	3.950	11.820	47.390	1.00	0.00
ATOM	6826	HW1	SOL	1581	4.510	11.100	47.110	1.00	0.00
ATOM	6827	HW2	SOL	1581	3.830	12.350	46.600	1.00	0.00
ATOM	6828	OW	SOL	1582	31.160	12.440	19.560	1.00	0.00
ATOM	6829	HW1	SOL	1582	31.120	13.180	18.960	1.00	0.00
ATOM	6830	HW2	SOL	1582	31.280	12.840	20.420	1.00	0.00
ATOM	6831	OW	SOL	1583	18.580	21.620	13.590	1.00	0.00
ATOM	6832	HW1	SOL	1583	18.980	22.050	12.830	1.00	0.00
ATOM	6833	HW2	SOL	1583	17.940	21.010	13.220	1.00	0.00
ATOM	6834	OW	SOL	1584	26.480	32.740	44.400	1.00	0.00
ATOM	6835	HW1	SOL	1584	25.960	33.230	43.770	1.00	0.00
ATOM	6836	HW2	SOL	1584	25.900	32.030	44.690	1.00	0.00
ATOM	6837	OW	SOL	1585	12.190	2.700	4.970	1.00	0.00
ATOM	6838	HW1	SOL	1585	12.970	3.130	4.620	1.00	0.00
ATOM	6839	HW2	SOL	1585	11.550	2.730	4.260	1.00	0.00
ATOM	6840	OW	SOL	1586	23.080	27.610	14.620	1.00	0.00
ATOM	6841	HW1	SOL	1586	22.580	26.880	14.980	1.00	0.00
ATOM	6842	HW2	SOL	1586	23.920	27.580	15.080	1.00	0.00
ATOM	6843	OW	SOL	1587	33.010	39.630	8.620	1.00	0.00
ATOM	6844	HW1	SOL	1587	33.150	39.100	9.410	1.00	0.00
ATOM	6845	HW2	SOL	1587	33.880	39.940	8.380	1.00	0.00
ATOM	6846	OW	SOL	1588	54.880	23.730	38.670	1.00	0.00
ATOM	6847	HW1	SOL	1588	55.160	23.560	37.780	1.00	0.00
ATOM	6848	HW2	SOL	1588	55.070	24.660	38.810	1.00	0.00
ATOM	6849	OW	SOL	1589	27.650	41.960	7.730	1.00	0.00
ATOM	6850	HW1	SOL	1589	27.370	42.870	7.680	1.00	0.00
ATOM	6851	HW2	SOL	1589	28.600	42.000	7.670	1.00	0.00
ATOM	6852	OW	SOL	1590	35.220	34.810	7.860	1.00	0.00
ATOM	6853	HW1	SOL	1590	34.870	35.700	7.960	1.00	0.00
ATOM	6854	HW2	SOL	1590	35.020	34.580	6.950	1.00	0.00
ATOM	6855	OW	SOL	1591	31.140	12.900	5.330	1.00	0.00
ATOM	6856	HW1	SOL	1591	30.770	13.630	4.840	1.00	0.00
ATOM	6857	HW2	SOL	1591	31.330	13.260	6.200	1.00	0.00
ATOM	6858	OW	SOL	1592	6.350	51.320	4.280	1.00	0.00
ATOM	6859	HW1	SOL	1592	7.260	51.070	4.450	1.00	0.00
ATOM	6860	HW2	SOL	1592	6.370	51.680	3.400	1.00	0.00
ATOM	6861	OW	SOL	1593	34.920	27.540	26.260	1.00	0.00
ATOM	6862	HW1	SOL	1593	35.620	27.900	25.710	1.00	0.00
ATOM	6863	HW2	SOL	1593	35.260	26.690	26.550	1.00	0.00

ATOM	6864	OW	SOL	1594	19.460	40.930	12.440	1.00	0.00
ATOM	6865	HW1	SOL	1594	19.230	41.620	13.070	1.00	0.00
ATOM	6866	HW2	SOL	1594	19.050	40.140	12.790	1.00	0.00
ATOM	6867	OW	SOL	1595	43.920	52.220	10.270	1.00	0.00
ATOM	6868	HW1	SOL	1595	43.580	52.050	9.390	1.00	0.00
ATOM	6869	HW2	SOL	1595	43.440	52.980	10.570	1.00	0.00
ATOM	6870	OW	SOL	1596	26.570	7.800	22.040	1.00	0.00
ATOM	6871	HW1	SOL	1596	26.400	8.250	21.210	1.00	0.00
ATOM	6872	HW2	SOL	1596	27.500	7.970	22.210	1.00	0.00
ATOM	6873	OW	SOL	1597	17.470	5.740	3.400	1.00	0.00
ATOM	6874	HW1	SOL	1597	17.570	5.440	4.300	1.00	0.00
ATOM	6875	HW2	SOL	1597	18.220	5.350	2.940	1.00	0.00
ATOM	6876	OW	SOL	1598	9.890	44.570	8.580	1.00	0.00
ATOM	6877	HW1	SOL	1598	10.190	44.180	9.400	1.00	0.00
ATOM	6878	HW2	SOL	1598	10.510	44.260	7.920	1.00	0.00
ATOM	6879	OW	SOL	1599	13.490	48.550	20.190	1.00	0.00
ATOM	6880	HW1	SOL	1599	13.930	47.800	20.600	1.00	0.00
ATOM	6881	HW2	SOL	1599	13.140	49.050	20.920	1.00	0.00
ATOM	6882	OW	SOL	1600	11.360	54.710	38.250	1.00	0.00
ATOM	6883	HW1	SOL	1600	11.240	55.630	38.490	1.00	0.00
ATOM	6884	HW2	SOL	1600	11.940	54.360	38.930	1.00	0.00
ATOM	6885	OW	SOL	1601	13.200	34.140	38.200	1.00	0.00
ATOM	6886	HW1	SOL	1601	12.560	33.720	37.630	1.00	0.00
ATOM	6887	HW2	SOL	1601	12.970	35.070	38.170	1.00	0.00
ATOM	6888	OW	SOL	1602	25.090	14.540	51.850	1.00	0.00
ATOM	6889	HW1	SOL	1602	25.890	14.320	51.360	1.00	0.00
ATOM	6890	HW2	SOL	1602	24.900	15.440	51.610	1.00	0.00
ATOM	6891	OW	SOL	1603	31.260	15.450	17.980	1.00	0.00
ATOM	6892	HW1	SOL	1603	30.550	15.120	17.440	1.00	0.00
ATOM	6893	HW2	SOL	1603	31.740	16.060	17.410	1.00	0.00
ATOM	6894	OW	SOL	1604	47.570	14.170	26.680	1.00	0.00
ATOM	6895	HW1	SOL	1604	48.390	14.550	26.380	1.00	0.00
ATOM	6896	HW2	SOL	1604	47.160	14.860	27.200	1.00	0.00
ATOM	6897	OW	SOL	1605	12.170	39.110	35.750	1.00	0.00
ATOM	6898	HW1	SOL	1605	12.490	39.200	36.650	1.00	0.00
ATOM	6899	HW2	SOL	1605	11.690	39.920	35.580	1.00	0.00
ATOM	6900	OW	SOL	1606	25.910	26.880	38.490	1.00	0.00
ATOM	6901	HW1	SOL	1606	26.480	27.270	37.820	1.00	0.00
ATOM	6902	HW2	SOL	1606	26.230	27.240	39.310	1.00	0.00
ATOM	6903	OW	SOL	1607	5.240	10.680	6.400	1.00	0.00
ATOM	6904	HW1	SOL	1607	4.960	10.230	5.600	1.00	0.00
ATOM	6905	HW2	SOL	1607	5.370	9.980	7.040	1.00	0.00
ATOM	6906	OW	SOL	1608	11.310	16.140	29.670	1.00	0.00
ATOM	6907	HW1	SOL	1608	10.860	15.320	29.490	1.00	0.00

ATOM	6908	HW2	SOL	1608	12.050	16.140	29.060	1.00	0.00
ATOM	6909	OW	SOL	1609	13.540	49.190	45.230	1.00	0.00
ATOM	6910	HW1	SOL	1609	13.640	49.830	45.930	1.00	0.00
ATOM	6911	HW2	SOL	1609	13.680	48.350	45.660	1.00	0.00
ATOM	6912	OW	SOL	1610	14.760	17.830	50.420	1.00	0.00
ATOM	6913	HW1	SOL	1610	13.850	17.870	50.690	1.00	0.00
ATOM	6914	HW2	SOL	1610	15.260	17.950	51.230	1.00	0.00
ATOM	6915	OW	SOL	1611	2.360	52.060	14.010	1.00	0.00
ATOM	6916	HW1	SOL	1611	1.870	51.240	14.090	1.00	0.00
ATOM	6917	HW2	SOL	1611	2.940	52.070	14.770	1.00	0.00
ATOM	6918	OW	SOL	1612	15.140	12.380	18.830	1.00	0.00
ATOM	6919	HW1	SOL	1612	14.780	11.630	19.300	1.00	0.00
ATOM	6920	HW2	SOL	1612	15.690	12.830	19.460	1.00	0.00
ATOM	6921	OW	SOL	1613	36.960	8.530	33.410	1.00	0.00
ATOM	6922	HW1	SOL	1613	37.410	8.970	34.130	1.00	0.00
ATOM	6923	HW2	SOL	1613	37.540	7.810	33.170	1.00	0.00
ATOM	6924	OW	SOL	1614	38.570	51.120	51.180	1.00	0.00
ATOM	6925	HW1	SOL	1614	38.060	51.160	51.990	1.00	0.00
ATOM	6926	HW2	SOL	1614	39.180	51.860	51.240	1.00	0.00
ATOM	6927	OW	SOL	1615	29.780	38.560	48.630	1.00	0.00
ATOM	6928	HW1	SOL	1615	28.890	38.330	48.360	1.00	0.00
ATOM	6929	HW2	SOL	1615	29.710	39.460	48.930	1.00	0.00
ATOM	6930	OW	SOL	1616	6.630	52.660	26.010	1.00	0.00
ATOM	6931	HW1	SOL	1616	6.950	53.400	26.520	1.00	0.00
ATOM	6932	HW2	SOL	1616	5.750	52.490	26.360	1.00	0.00
ATOM	6933	OW	SOL	1617	25.600	17.340	18.300	1.00	0.00
ATOM	6934	HW1	SOL	1617	25.730	16.840	17.500	1.00	0.00
ATOM	6935	HW2	SOL	1617	24.650	17.450	18.350	1.00	0.00
ATOM	6936	OW	SOL	1618	7.490	10.620	39.920	1.00	0.00
ATOM	6937	HW1	SOL	1618	7.680	10.830	40.840	1.00	0.00
ATOM	6938	HW2	SOL	1618	7.640	9.680	39.850	1.00	0.00
ATOM	6939	OW	SOL	1619	9.260	35.810	6.880	1.00	0.00
ATOM	6940	HW1	SOL	1619	9.380	36.160	7.770	1.00	0.00
ATOM	6941	HW2	SOL	1619	8.780	36.490	6.420	1.00	0.00
ATOM	6942	OW	SOL	1620	45.420	6.510	37.620	1.00	0.00
ATOM	6943	HW1	SOL	1620	45.510	6.050	36.780	1.00	0.00
ATOM	6944	HW2	SOL	1620	46.170	7.100	37.650	1.00	0.00
ATOM	6945	OW	SOL	1621	31.890	13.730	21.810	1.00	0.00
ATOM	6946	HW1	SOL	1621	31.900	13.380	22.700	1.00	0.00
ATOM	6947	HW2	SOL	1621	31.980	14.680	21.930	1.00	0.00
ATOM	6948	OW	SOL	1622	42.330	51.160	55.080	1.00	0.00
ATOM	6949	HW1	SOL	1622	42.320	50.210	54.960	1.00	0.00
ATOM	6950	HW2	SOL	1622	41.420	51.420	54.950	1.00	0.00
ATOM	6951	OW	SOL	1623	47.060	13.440	38.780	1.00	0.00

ATOM	6952	HW1	SOL	1623	47.580	13.560	37.980	1.00	0.00
ATOM	6953	HW2	SOL	1623	47.130	12.510	38.980	1.00	0.00
ATOM	6954	OW	SOL	1624	4.550	41.780	37.760	1.00	0.00
ATOM	6955	HW1	SOL	1624	4.320	41.570	38.660	1.00	0.00
ATOM	6956	HW2	SOL	1624	5.120	41.070	37.480	1.00	0.00
ATOM	6957	OW	SOL	1625	49.060	42.150	49.550	1.00	0.00
ATOM	6958	HW1	SOL	1625	48.910	41.690	50.380	1.00	0.00
ATOM	6959	HW2	SOL	1625	49.980	42.420	49.600	1.00	0.00
ATOM	6960	OW	SOL	1626	10.930	28.290	53.530	1.00	0.00
ATOM	6961	HW1	SOL	1626	10.670	28.180	54.440	1.00	0.00
ATOM	6962	HW2	SOL	1626	10.110	28.290	53.040	1.00	0.00
ATOM	6963	OW	SOL	1627	20.620	54.280	2.990	1.00	0.00
ATOM	6964	HW1	SOL	1627	21.130	54.130	2.190	1.00	0.00
ATOM	6965	HW2	SOL	1627	20.360	53.400	3.280	1.00	0.00
ATOM	6966	OW	SOL	1628	33.210	22.270	0.050	1.00	0.00
ATOM	6967	HW1	SOL	1628	32.260	22.200	-0.010	1.00	0.00
ATOM	6968	HW2	SOL	1628	33.370	23.210	0.170	1.00	0.00
ATOM	6969	OW	SOL	1629	45.390	13.300	35.490	1.00	0.00
ATOM	6970	HW1	SOL	1629	46.050	13.110	34.830	1.00	0.00
ATOM	6971	HW2	SOL	1629	44.760	13.870	35.050	1.00	0.00
ATOM	6972	OW	SOL	1630	27.120	37.770	14.490	1.00	0.00
ATOM	6973	HW1	SOL	1630	26.770	38.270	13.750	1.00	0.00
ATOM	6974	HW2	SOL	1630	26.600	38.070	15.240	1.00	0.00
ATOM	6975	OW	SOL	1631	5.370	15.350	49.580	1.00	0.00
ATOM	6976	HW1	SOL	1631	4.950	15.220	48.720	1.00	0.00
ATOM	6977	HW2	SOL	1631	6.270	15.600	49.380	1.00	0.00
ATOM	6978	OW	SOL	1632	2.900	1.630	15.800	1.00	0.00
ATOM	6979	HW1	SOL	1632	2.480	2.230	15.190	1.00	0.00
ATOM	6980	HW2	SOL	1632	3.240	0.920	15.260	1.00	0.00
ATOM	6981	OW	SOL	1633	33.350	16.260	27.510	1.00	0.00
ATOM	6982	HW1	SOL	1633	32.480	16.440	27.150	1.00	0.00
ATOM	6983	HW2	SOL	1633	33.900	16.960	27.160	1.00	0.00
ATOM	6984	OW	SOL	1634	18.020	53.770	9.580	1.00	0.00
ATOM	6985	HW1	SOL	1634	18.320	53.770	8.670	1.00	0.00
ATOM	6986	HW2	SOL	1634	18.830	53.770	10.100	1.00	0.00
ATOM	6987	OW	SOL	1635	44.090	34.540	33.650	1.00	0.00
ATOM	6988	HW1	SOL	1635	44.690	33.790	33.700	1.00	0.00
ATOM	6989	HW2	SOL	1635	44.020	34.840	34.550	1.00	0.00
ATOM	6990	OW	SOL	1636	49.080	46.950	11.960	1.00	0.00
ATOM	6991	HW1	SOL	1636	49.000	46.090	12.360	1.00	0.00
ATOM	6992	HW2	SOL	1636	48.330	47.020	11.380	1.00	0.00
ATOM	6993	OW	SOL	1637	24.570	22.580	45.660	1.00	0.00
ATOM	6994	HW1	SOL	1637	25.160	22.040	46.180	1.00	0.00
ATOM	6995	HW2	SOL	1637	24.850	22.450	44.760	1.00	0.00

ATOM	6996	OW	SOL	1638	8.970	21.450	25.600	1.00	0.00
ATOM	6997	HW1	SOL	1638	8.320	20.860	25.980	1.00	0.00
ATOM	6998	HW2	SOL	1638	9.260	21.990	26.340	1.00	0.00
ATOM	6999	OW	SOL	1639	49.720	23.560	31.260	1.00	0.00
ATOM	7000	HW1	SOL	1639	48.990	23.100	30.850	1.00	0.00
ATOM	7001	HW2	SOL	1639	49.450	24.480	31.270	1.00	0.00
ATOM	7002	OW	SOL	1640	16.060	25.150	24.230	1.00	0.00
ATOM	7003	HW1	SOL	1640	16.980	24.880	24.120	1.00	0.00
ATOM	7004	HW2	SOL	1640	16.060	26.090	24.020	1.00	0.00
ATOM	7005	OW	SOL	1641	41.330	19.920	26.910	1.00	0.00
ATOM	7006	HW1	SOL	1641	42.120	20.270	27.330	1.00	0.00
ATOM	7007	HW2	SOL	1641	40.700	19.830	27.630	1.00	0.00
ATOM	7008	OW	SOL	1642	8.510	34.980	40.510	1.00	0.00
ATOM	7009	HW1	SOL	1642	8.500	34.430	41.290	1.00	0.00
ATOM	7010	HW2	SOL	1642	8.370	35.860	40.840	1.00	0.00
ATOM	7011	OW	SOL	1643	2.140	28.230	33.610	1.00	0.00
ATOM	7012	HW1	SOL	1643	2.600	27.410	33.810	1.00	0.00
ATOM	7013	HW2	SOL	1643	2.730	28.920	33.900	1.00	0.00
ATOM	7014	OW	SOL	1644	51.800	50.420	17.640	1.00	0.00
ATOM	7015	HW1	SOL	1644	50.920	50.610	17.950	1.00	0.00
ATOM	7016	HW2	SOL	1644	52.300	50.240	18.430	1.00	0.00
ATOM	7017	OW	SOL	1645	4.010	0.280	13.160	1.00	0.00
ATOM	7018	HW1	SOL	1645	3.540	0.810	12.510	1.00	0.00
ATOM	7019	HW2	SOL	1645	4.100	-0.580	12.750	1.00	0.00
ATOM	7020	OW	SOL	1646	42.870	11.180	25.550	1.00	0.00
ATOM	7021	HW1	SOL	1646	43.320	10.510	26.080	1.00	0.00
ATOM	7022	HW2	SOL	1646	43.050	10.930	24.640	1.00	0.00
ATOM	7023	OW	SOL	1647	8.760	21.930	2.910	1.00	0.00
ATOM	7024	HW1	SOL	1647	8.080	21.420	2.470	1.00	0.00
ATOM	7025	HW2	SOL	1647	8.430	22.830	2.920	1.00	0.00
ATOM	7026	OW	SOL	1648	51.500	4.790	29.600	1.00	0.00
ATOM	7027	HW1	SOL	1648	51.700	3.850	29.520	1.00	0.00
ATOM	7028	HW2	SOL	1648	52.280	5.160	30.020	1.00	0.00
ATOM	7029	OW	SOL	1649	40.580	26.140	48.520	1.00	0.00
ATOM	7030	HW1	SOL	1649	41.150	26.410	49.240	1.00	0.00
ATOM	7031	HW2	SOL	1649	40.070	26.930	48.310	1.00	0.00
ATOM	7032	OW	SOL	1650	8.580	49.690	40.220	1.00	0.00
ATOM	7033	HW1	SOL	1650	9.370	49.170	40.100	1.00	0.00
ATOM	7034	HW2	SOL	1650	8.410	49.660	41.160	1.00	0.00
ATOM	7035	OW	SOL	1651	16.920	6.880	54.160	1.00	0.00
ATOM	7036	HW1	SOL	1651	16.500	6.930	55.010	1.00	0.00
ATOM	7037	HW2	SOL	1651	16.200	6.790	53.530	1.00	0.00
ATOM	7038	OW	SOL	1652	42.080	31.440	38.990	1.00	0.00
ATOM	7039	HW1	SOL	1652	42.490	31.490	38.130	1.00	0.00

ATOM	7040	HW2	SOL	1652	41.560	30.630	38.960	1.00	0.00
ATOM	7041	OW	SOL	1653	52.860	13.650	38.820	1.00	0.00
ATOM	7042	HW1	SOL	1653	53.560	13.450	38.190	1.00	0.00
ATOM	7043	HW2	SOL	1653	52.390	14.390	38.430	1.00	0.00
ATOM	7044	OW	SOL	1654	43.540	22.610	46.150	1.00	0.00
ATOM	7045	HW1	SOL	1654	44.490	22.620	46.090	1.00	0.00
ATOM	7046	HW2	SOL	1654	43.360	22.780	47.080	1.00	0.00
ATOM	7047	OW	SOL	1655	27.460	11.680	0.510	1.00	0.00
ATOM	7048	HW1	SOL	1655	27.350	11.750	1.450	1.00	0.00
ATOM	7049	HW2	SOL	1655	28.230	11.120	0.390	1.00	0.00
ATOM	7050	OW	SOL	1656	19.230	34.010	11.980	1.00	0.00
ATOM	7051	HW1	SOL	1656	19.640	33.980	12.850	1.00	0.00
ATOM	7052	HW2	SOL	1656	19.180	33.100	11.710	1.00	0.00
ATOM	7053	OW	SOL	1657	7.980	3.500	9.080	1.00	0.00
ATOM	7054	HW1	SOL	1657	7.410	4.260	8.980	1.00	0.00
ATOM	7055	HW2	SOL	1657	7.430	2.860	9.560	1.00	0.00
ATOM	7056	OW	SOL	1658	34.190	31.750	16.370	1.00	0.00
ATOM	7057	HW1	SOL	1658	34.610	32.350	15.750	1.00	0.00
ATOM	7058	HW2	SOL	1658	34.090	30.930	15.870	1.00	0.00
ATOM	7059	OW	SOL	1659	12.440	39.380	21.710	1.00	0.00
ATOM	7060	HW1	SOL	1659	12.110	39.990	21.050	1.00	0.00
ATOM	7061	HW2	SOL	1659	11.680	39.180	22.250	1.00	0.00
ATOM	7062	OW	SOL	1660	51.350	45.990	53.550	1.00	0.00
ATOM	7063	HW1	SOL	1660	51.040	45.090	53.450	1.00	0.00
ATOM	7064	HW2	SOL	1660	52.260	45.960	53.260	1.00	0.00
ATOM	7065	OW	SOL	1661	9.110	26.290	22.690	1.00	0.00
ATOM	7066	HW1	SOL	1661	8.730	27.160	22.770	1.00	0.00
ATOM	7067	HW2	SOL	1661	8.470	25.710	23.110	1.00	0.00
ATOM	7068	OW	SOL	1662	43.150	7.290	5.090	1.00	0.00
ATOM	7069	HW1	SOL	1662	42.860	6.790	4.330	1.00	0.00
ATOM	7070	HW2	SOL	1662	42.950	8.200	4.880	1.00	0.00
ATOM	7071	OW	SOL	1663	16.310	5.900	35.880	1.00	0.00
ATOM	7072	HW1	SOL	1663	16.010	6.700	35.440	1.00	0.00
ATOM	7073	HW2	SOL	1663	16.540	6.180	36.760	1.00	0.00
ATOM	7074	OW	SOL	1664	47.620	8.180	23.460	1.00	0.00
ATOM	7075	HW1	SOL	1664	48.460	8.490	23.800	1.00	0.00
ATOM	7076	HW2	SOL	1664	47.480	7.340	23.910	1.00	0.00
ATOM	7077	OW	SOL	1665	43.420	37.060	42.770	1.00	0.00
ATOM	7078	HW1	SOL	1665	44.270	37.030	43.220	1.00	0.00
ATOM	7079	HW2	SOL	1665	43.000	37.850	43.100	1.00	0.00
ATOM	7080	OW	SOL	1666	9.600	31.970	12.150	1.00	0.00
ATOM	7081	HW1	SOL	1666	8.880	31.670	12.710	1.00	0.00
ATOM	7082	HW2	SOL	1666	9.260	31.890	11.260	1.00	0.00
ATOM	7083	OW	SOL	1667	53.170	21.320	52.630	1.00	0.00

ATOM	7084	HW1	SOL	1667	53.440	21.640	53.500	1.00	0.00
ATOM	7085	HW2	SOL	1667	52.990	22.120	52.130	1.00	0.00
ATOM	7086	OW	SOL	1668	20.220	34.680	8.210	1.00	0.00
ATOM	7087	HW1	SOL	1668	19.260	34.580	8.250	1.00	0.00
ATOM	7088	HW2	SOL	1668	20.560	33.800	8.340	1.00	0.00
ATOM	7089	OW	SOL	1669	55.210	19.210	43.940	1.00	0.00
ATOM	7090	HW1	SOL	1669	54.650	19.980	43.840	1.00	0.00
ATOM	7091	HW2	SOL	1669	55.440	19.200	44.870	1.00	0.00
ATOM	7092	OW	SOL	1670	11.330	37.980	42.800	1.00	0.00
ATOM	7093	HW1	SOL	1670	10.370	37.960	42.810	1.00	0.00
ATOM	7094	HW2	SOL	1670	11.570	37.610	41.950	1.00	0.00
ATOM	7095	OW	SOL	1671	8.090	11.270	9.370	1.00	0.00
ATOM	7096	HW1	SOL	1671	8.640	11.560	10.090	1.00	0.00
ATOM	7097	HW2	SOL	1671	8.670	10.750	8.810	1.00	0.00
ATOM	7098	OW	SOL	1672	35.200	12.830	8.730	1.00	0.00
ATOM	7099	HW1	SOL	1672	36.110	13.020	8.990	1.00	0.00
ATOM	7100	HW2	SOL	1672	34.680	13.110	9.480	1.00	0.00
ATOM	7101	OW	SOL	1673	1.720	54.010	22.250	1.00	0.00
ATOM	7102	HW1	SOL	1673	2.340	54.590	22.700	1.00	0.00
ATOM	7103	HW2	SOL	1673	1.560	54.450	21.410	1.00	0.00
ATOM	7104	OW	SOL	1674	0.630	48.360	5.950	1.00	0.00
ATOM	7105	HW1	SOL	1674	0.550	49.300	6.140	1.00	0.00
ATOM	7106	HW2	SOL	1674	0.800	48.310	5.010	1.00	0.00
ATOM	7107	OW	SOL	1675	22.600	18.880	8.080	1.00	0.00
ATOM	7108	HW1	SOL	1675	22.830	19.790	8.260	1.00	0.00
ATOM	7109	HW2	SOL	1675	23.370	18.510	7.660	1.00	0.00
ATOM	7110	OW	SOL	1676	17.610	19.810	20.800	1.00	0.00
ATOM	7111	HW1	SOL	1676	17.850	20.720	20.630	1.00	0.00
ATOM	7112	HW2	SOL	1676	18.430	19.400	21.080	1.00	0.00
ATOM	7113	OW	SOL	1677	4.200	5.190	45.190	1.00	0.00
ATOM	7114	HW1	SOL	1677	4.390	4.330	45.550	1.00	0.00
ATOM	7115	HW2	SOL	1677	3.730	5.650	45.880	1.00	0.00
ATOM	7116	OW	SOL	1678	15.840	40.260	0.570	1.00	0.00
ATOM	7117	HW1	SOL	1678	16.380	40.440	1.340	1.00	0.00
ATOM	7118	HW2	SOL	1678	15.860	39.300	0.490	1.00	0.00
ATOM	7119	OW	SOL	1679	38.810	26.740	41.400	1.00	0.00
ATOM	7120	HW1	SOL	1679	38.770	25.780	41.370	1.00	0.00
ATOM	7121	HW2	SOL	1679	39.750	26.940	41.470	1.00	0.00
ATOM	7122	OW	SOL	1680	28.280	26.910	54.950	1.00	0.00
ATOM	7123	HW1	SOL	1680	29.130	27.320	55.070	1.00	0.00
ATOM	7124	HW2	SOL	1680	28.340	26.090	55.430	1.00	0.00
ATOM	7125	OW	SOL	1681	27.590	52.000	46.800	1.00	0.00
ATOM	7126	HW1	SOL	1681	27.850	51.340	46.150	1.00	0.00
ATOM	7127	HW2	SOL	1681	28.010	52.810	46.500	1.00	0.00

ATOM	7128	OW	SOL	1682	2.990	31.300	33.850	1.00	0.00
ATOM	7129	HW1	SOL	1682	2.470	31.900	34.380	1.00	0.00
ATOM	7130	HW2	SOL	1682	2.580	31.310	32.990	1.00	0.00
ATOM	7131	OW	SOL	1683	46.280	25.150	35.850	1.00	0.00
ATOM	7132	HW1	SOL	1683	45.470	25.540	36.160	1.00	0.00
ATOM	7133	HW2	SOL	1683	46.960	25.780	36.090	1.00	0.00
ATOM	7134	OW	SOL	1684	47.680	44.740	10.490	1.00	0.00
ATOM	7135	HW1	SOL	1684	47.230	45.150	11.230	1.00	0.00
ATOM	7136	HW2	SOL	1684	47.630	43.800	10.660	1.00	0.00
ATOM	7137	OW	SOL	1685	28.950	12.010	35.680	1.00	0.00
ATOM	7138	HW1	SOL	1685	28.810	11.120	36.000	1.00	0.00
ATOM	7139	HW2	SOL	1685	29.140	11.900	34.750	1.00	0.00
ATOM	7140	OW	SOL	1686	38.290	33.020	31.750	1.00	0.00
ATOM	7141	HW1	SOL	1686	38.330	32.340	31.080	1.00	0.00
ATOM	7142	HW2	SOL	1686	37.380	33.300	31.760	1.00	0.00
ATOM	7143	OW	SOL	1687	54.810	4.670	35.380	1.00	0.00
ATOM	7144	HW1	SOL	1687	54.530	4.530	34.470	1.00	0.00
ATOM	7145	HW2	SOL	1687	55.770	4.680	35.330	1.00	0.00
ATOM	7146	OW	SOL	1688	0.360	23.310	34.290	1.00	0.00
ATOM	7147	HW1	SOL	1688	0.480	22.390	34.530	1.00	0.00
ATOM	7148	HW2	SOL	1688	0.870	23.800	34.940	1.00	0.00
ATOM	7149	OW	SOL	1689	13.930	7.320	55.290	1.00	0.00
ATOM	7150	HW1	SOL	1689	13.600	8.110	55.710	1.00	0.00
ATOM	7151	HW2	SOL	1689	14.390	6.850	55.990	1.00	0.00
ATOM	7152	OW	SOL	1690	40.810	14.790	47.340	1.00	0.00
ATOM	7153	HW1	SOL	1690	40.720	15.630	47.790	1.00	0.00
ATOM	7154	HW2	SOL	1690	40.090	14.260	47.670	1.00	0.00
ATOM	7155	OW	SOL	1691	35.580	33.420	30.320	1.00	0.00
ATOM	7156	HW1	SOL	1691	35.130	33.660	31.130	1.00	0.00
ATOM	7157	HW2	SOL	1691	35.370	34.120	29.710	1.00	0.00
ATOM	7158	OW	SOL	1692	34.560	54.090	35.110	1.00	0.00
ATOM	7159	HW1	SOL	1692	34.080	53.680	35.830	1.00	0.00
ATOM	7160	HW2	SOL	1692	33.910	54.640	34.670	1.00	0.00
ATOM	7161	OW	SOL	1693	3.900	38.620	46.750	1.00	0.00
ATOM	7162	HW1	SOL	1693	3.620	38.150	47.530	1.00	0.00
ATOM	7163	HW2	SOL	1693	3.470	39.480	46.820	1.00	0.00
ATOM	7164	OW	SOL	1694	50.770	52.060	8.160	1.00	0.00
ATOM	7165	HW1	SOL	1694	50.140	51.340	8.210	1.00	0.00
ATOM	7166	HW2	SOL	1694	50.320	52.740	7.650	1.00	0.00
ATOM	7167	OW	SOL	1695	25.950	26.150	2.780	1.00	0.00
ATOM	7168	HW1	SOL	1695	25.020	26.070	2.990	1.00	0.00
ATOM	7169	HW2	SOL	1695	26.270	26.850	3.350	1.00	0.00
ATOM	7170	OW	SOL	1696	1.160	17.580	54.560	1.00	0.00
ATOM	7171	HW1	SOL	1696	0.500	17.090	55.050	1.00	0.00

ATOM	7172	HW2	SOL	1696	1.220	17.140	53.720	1.00	0.00
ATOM	7173	OW	SOL	1697	34.560	32.730	48.450	1.00	0.00
ATOM	7174	HW1	SOL	1697	33.840	32.150	48.200	1.00	0.00
ATOM	7175	HW2	SOL	1697	34.350	33.570	48.050	1.00	0.00
ATOM	7176	OW	SOL	1698	49.440	40.000	11.490	1.00	0.00
ATOM	7177	HW1	SOL	1698	49.680	39.410	10.780	1.00	0.00
ATOM	7178	HW2	SOL	1698	49.920	39.670	12.250	1.00	0.00
ATOM	7179	OW	SOL	1699	24.680	13.080	8.740	1.00	0.00
ATOM	7180	HW1	SOL	1699	24.060	13.580	8.210	1.00	0.00
ATOM	7181	HW2	SOL	1699	25.210	13.740	9.180	1.00	0.00
ATOM	7182	OW	SOL	1700	7.990	10.660	15.180	1.00	0.00
ATOM	7183	HW1	SOL	1700	7.940	11.510	15.620	1.00	0.00
ATOM	7184	HW2	SOL	1700	8.900	10.580	14.910	1.00	0.00
ATOM	7185	OW	SOL	1701	50.220	28.170	22.550	1.00	0.00
ATOM	7186	HW1	SOL	1701	50.560	28.320	23.430	1.00	0.00
ATOM	7187	HW2	SOL	1701	49.680	27.380	22.620	1.00	0.00
ATOM	7188	OW	SOL	1702	39.490	10.520	15.760	1.00	0.00
ATOM	7189	HW1	SOL	1702	38.760	10.750	16.330	1.00	0.00
ATOM	7190	HW2	SOL	1702	40.050	11.300	15.750	1.00	0.00
ATOM	7191	OW	SOL	1703	5.250	55.110	15.380	1.00	0.00
ATOM	7192	HW1	SOL	1703	5.030	54.190	15.490	1.00	0.00
ATOM	7193	HW2	SOL	1703	4.870	55.360	14.540	1.00	0.00
ATOM	7194	OW	SOL	1704	54.250	2.460	48.840	1.00	0.00
ATOM	7195	HW1	SOL	1704	54.430	3.390	48.700	1.00	0.00
ATOM	7196	HW2	SOL	1704	54.170	2.090	47.960	1.00	0.00
ATOM	7197	OW	SOL	1705	15.200	8.460	35.050	1.00	0.00
ATOM	7198	HW1	SOL	1705	14.750	8.680	35.860	1.00	0.00
ATOM	7199	HW2	SOL	1705	15.190	9.270	34.540	1.00	0.00
ATOM	7200	OW	SOL	1706	3.350	24.540	7.140	1.00	0.00
ATOM	7201	HW1	SOL	1706	3.300	25.360	6.650	1.00	0.00
ATOM	7202	HW2	SOL	1706	2.550	24.070	6.900	1.00	0.00
ATOM	7203	OW	SOL	1707	47.820	7.060	4.640	1.00	0.00
ATOM	7204	HW1	SOL	1707	48.370	7.400	3.940	1.00	0.00
ATOM	7205	HW2	SOL	1707	48.410	6.990	5.400	1.00	0.00
ATOM	7206	OW	SOL	1708	48.720	41.790	38.380	1.00	0.00
ATOM	7207	HW1	SOL	1708	48.500	41.560	37.480	1.00	0.00
ATOM	7208	HW2	SOL	1708	49.470	42.380	38.300	1.00	0.00
ATOM	7209	OW	SOL	1709	30.390	1.350	44.280	1.00	0.00
ATOM	7210	HW1	SOL	1709	30.410	1.830	43.450	1.00	0.00
ATOM	7211	HW2	SOL	1709	29.460	1.320	44.510	1.00	0.00
ATOM	7212	OW	SOL	1710	51.190	4.170	46.930	1.00	0.00
ATOM	7213	HW1	SOL	1710	51.260	5.000	47.400	1.00	0.00
ATOM	7214	HW2	SOL	1710	50.260	3.970	46.930	1.00	0.00
ATOM	7215	OW	SOL	1711	31.660	6.280	51.570	1.00	0.00

ATOM	7216	HW1	SOL	1711	32.310	6.420	52.250	1.00	0.00
ATOM	7217	HW2	SOL	1711	30.830	6.560	51.970	1.00	0.00
ATOM	7218	OW	SOL	1712	22.170	5.110	48.350	1.00	0.00
ATOM	7219	HW1	SOL	1712	22.270	5.900	48.880	1.00	0.00
ATOM	7220	HW2	SOL	1712	23.040	4.700	48.370	1.00	0.00
ATOM	7221	OW	SOL	1713	41.490	3.070	12.050	1.00	0.00
ATOM	7222	HW1	SOL	1713	41.190	2.200	11.780	1.00	0.00
ATOM	7223	HW2	SOL	1713	41.530	3.580	11.230	1.00	0.00
ATOM	7224	OW	SOL	1714	35.290	31.280	20.230	1.00	0.00
ATOM	7225	HW1	SOL	1714	36.200	31.000	20.260	1.00	0.00
ATOM	7226	HW2	SOL	1714	34.800	30.540	20.590	1.00	0.00
ATOM	7227	OW	SOL	1715	23.500	23.960	6.560	1.00	0.00
ATOM	7228	HW1	SOL	1715	23.710	24.030	7.490	1.00	0.00
ATOM	7229	HW2	SOL	1715	23.160	24.820	6.320	1.00	0.00
ATOM	7230	OW	SOL	1716	30.780	12.980	38.320	1.00	0.00
ATOM	7231	HW1	SOL	1716	30.620	13.930	38.370	1.00	0.00
ATOM	7232	HW2	SOL	1716	30.910	12.820	37.390	1.00	0.00
ATOM	7233	OW	SOL	1717	52.850	26.080	30.920	1.00	0.00
ATOM	7234	HW1	SOL	1717	52.230	25.890	31.620	1.00	0.00
ATOM	7235	HW2	SOL	1717	52.850	27.030	30.850	1.00	0.00
ATOM	7236	OW	SOL	1718	26.150	6.460	15.960	1.00	0.00
ATOM	7237	HW1	SOL	1718	26.150	5.500	16.000	1.00	0.00
ATOM	7238	HW2	SOL	1718	26.680	6.740	16.700	1.00	0.00
ATOM	7239	OW	SOL	1719	0.650	25.950	27.380	1.00	0.00
ATOM	7240	HW1	SOL	1719	1.430	25.470	27.110	1.00	0.00
ATOM	7241	HW2	SOL	1719	0.440	25.600	28.250	1.00	0.00
ATOM	7242	OW	SOL	1720	40.130	30.750	12.820	1.00	0.00
ATOM	7243	HW1	SOL	1720	40.270	31.590	12.390	1.00	0.00
ATOM	7244	HW2	SOL	1720	39.510	30.290	12.250	1.00	0.00
ATOM	7245	OW	SOL	1721	49.420	29.260	20.360	1.00	0.00
ATOM	7246	HW1	SOL	1721	49.780	28.870	21.160	1.00	0.00
ATOM	7247	HW2	SOL	1721	50.030	28.990	19.670	1.00	0.00
ATOM	7248	OW	SOL	1722	23.330	13.800	31.550	1.00	0.00
ATOM	7249	HW1	SOL	1722	23.300	13.920	30.600	1.00	0.00
ATOM	7250	HW2	SOL	1722	24.190	14.140	31.810	1.00	0.00
ATOM	7251	OW	SOL	1723	10.760	24.540	36.260	1.00	0.00
ATOM	7252	HW1	SOL	1723	10.560	24.310	37.160	1.00	0.00
ATOM	7253	HW2	SOL	1723	11.420	25.220	36.320	1.00	0.00
ATOM	7254	OW	SOL	1724	11.860	49.810	16.270	1.00	0.00
ATOM	7255	HW1	SOL	1724	11.530	50.510	16.840	1.00	0.00
ATOM	7256	HW2	SOL	1724	12.510	49.350	16.810	1.00	0.00
ATOM	7257	OW	SOL	1725	15.490	16.760	37.790	1.00	0.00
ATOM	7258	HW1	SOL	1725	15.130	16.130	37.170	1.00	0.00
ATOM	7259	HW2	SOL	1725	15.710	17.520	37.250	1.00	0.00

ATOM	7260	OW	SOL	1726	37.090	4.310	31.690	1.00	0.00
ATOM	7261	HW1	SOL	1726	37.040	4.470	30.750	1.00	0.00
ATOM	7262	HW2	SOL	1726	37.310	3.380	31.770	1.00	0.00
ATOM	7263	OW	SOL	1727	16.520	5.370	19.590	1.00	0.00
ATOM	7264	HW1	SOL	1727	17.350	5.070	19.230	1.00	0.00
ATOM	7265	HW2	SOL	1727	15.910	4.660	19.420	1.00	0.00
ATOM	7266	OW	SOL	1728	19.860	9.340	0.970	1.00	0.00
ATOM	7267	HW1	SOL	1728	19.230	10.010	0.700	1.00	0.00
ATOM	7268	HW2	SOL	1728	20.700	9.810	0.990	1.00	0.00
ATOM	7269	OW	SOL	1729	29.000	24.340	20.960	1.00	0.00
ATOM	7270	HW1	SOL	1729	28.840	24.750	21.820	1.00	0.00
ATOM	7271	HW2	SOL	1729	28.440	23.570	20.960	1.00	0.00
ATOM	7272	OW	SOL	1730	17.030	45.830	52.980	1.00	0.00
ATOM	7273	HW1	SOL	1730	16.530	46.430	52.420	1.00	0.00
ATOM	7274	HW2	SOL	1730	16.940	46.200	53.860	1.00	0.00
ATOM	7275	OW	SOL	1731	45.090	40.110	31.110	1.00	0.00
ATOM	7276	HW1	SOL	1731	44.170	39.830	31.120	1.00	0.00
ATOM	7277	HW2	SOL	1731	45.480	39.640	31.850	1.00	0.00
ATOM	7278	OW	SOL	1732	3.190	34.710	25.710	1.00	0.00
ATOM	7279	HW1	SOL	1732	3.440	33.880	26.100	1.00	0.00
ATOM	7280	HW2	SOL	1732	3.410	34.610	24.780	1.00	0.00
ATOM	7281	OW	SOL	1733	23.890	34.880	53.710	1.00	0.00
ATOM	7282	HW1	SOL	1733	23.540	35.740	53.470	1.00	0.00
ATOM	7283	HW2	SOL	1733	23.170	34.270	53.520	1.00	0.00
ATOM	7284	OW	SOL	1734	13.620	4.520	29.640	1.00	0.00
ATOM	7285	HW1	SOL	1734	14.010	4.110	30.410	1.00	0.00
ATOM	7286	HW2	SOL	1734	13.620	3.840	28.970	1.00	0.00
ATOM	7287	OW	SOL	1735	22.130	36.290	6.040	1.00	0.00
ATOM	7288	HW1	SOL	1735	21.220	36.520	6.210	1.00	0.00
ATOM	7289	HW2	SOL	1735	22.210	35.380	6.330	1.00	0.00
ATOM	7290	OW	SOL	1736	48.130	2.400	44.740	1.00	0.00
ATOM	7291	HW1	SOL	1736	47.220	2.110	44.810	1.00	0.00
ATOM	7292	HW2	SOL	1736	48.600	1.880	45.390	1.00	0.00
ATOM	7293	OW	SOL	1737	12.800	52.180	43.620	1.00	0.00
ATOM	7294	HW1	SOL	1737	13.490	52.700	43.210	1.00	0.00
ATOM	7295	HW2	SOL	1737	12.640	52.610	44.460	1.00	0.00
ATOM	7296	OW	SOL	1738	25.680	32.310	11.020	1.00	0.00
ATOM	7297	HW1	SOL	1738	25.980	32.050	10.150	1.00	0.00
ATOM	7298	HW2	SOL	1738	25.090	33.050	10.860	1.00	0.00
ATOM	7299	OW	SOL	1739	3.280	48.420	54.120	1.00	0.00
ATOM	7300	HW1	SOL	1739	2.870	48.630	53.280	1.00	0.00
ATOM	7301	HW2	SOL	1739	3.760	49.210	54.360	1.00	0.00
ATOM	7302	OW	SOL	1740	28.720	9.230	36.260	1.00	0.00
ATOM	7303	HW1	SOL	1740	27.820	9.020	36.010	1.00	0.00

ATOM	7304	HW2	SOL	1740	28.750	9.070	37.200	1.00	0.00
ATOM	7305	OW	SOL	1741	55.080	40.010	35.470	1.00	0.00
ATOM	7306	HW1	SOL	1741	55.370	39.350	36.100	1.00	0.00
ATOM	7307	HW2	SOL	1741	55.870	40.500	35.250	1.00	0.00
ATOM	7308	OW	SOL	1742	9.100	42.430	41.770	1.00	0.00
ATOM	7309	HW1	SOL	1742	8.690	43.270	41.940	1.00	0.00
ATOM	7310	HW2	SOL	1742	9.330	42.450	40.840	1.00	0.00
ATOM	7311	OW	SOL	1743	38.280	16.220	32.830	1.00	0.00
ATOM	7312	HW1	SOL	1743	38.280	16.000	33.760	1.00	0.00
ATOM	7313	HW2	SOL	1743	39.120	16.660	32.680	1.00	0.00
ATOM	7314	OW	SOL	1744	12.440	55.320	23.120	1.00	0.00
ATOM	7315	HW1	SOL	1744	12.420	55.440	22.170	1.00	0.00
ATOM	7316	HW2	SOL	1744	11.610	55.700	23.430	1.00	0.00
ATOM	7317	OW	SOL	1745	49.700	44.410	28.100	1.00	0.00
ATOM	7318	HW1	SOL	1745	49.790	43.470	27.930	1.00	0.00
ATOM	7319	HW2	SOL	1745	49.180	44.740	27.370	1.00	0.00
ATOM	7320	OW	SOL	1746	53.170	17.600	54.640	1.00	0.00
ATOM	7321	HW1	SOL	1746	52.370	17.120	54.870	1.00	0.00
ATOM	7322	HW2	SOL	1746	53.300	18.220	55.360	1.00	0.00
ATOM	7323	OW	SOL	1747	11.710	13.110	40.580	1.00	0.00
ATOM	7324	HW1	SOL	1747	12.160	13.900	40.270	1.00	0.00
ATOM	7325	HW2	SOL	1747	12.130	12.910	41.420	1.00	0.00
ATOM	7326	OW	SOL	1748	37.920	13.090	9.730	1.00	0.00
ATOM	7327	HW1	SOL	1748	38.240	13.990	9.830	1.00	0.00
ATOM	7328	HW2	SOL	1748	38.300	12.620	10.480	1.00	0.00
ATOM	7329	OW	SOL	1749	19.570	8.580	6.250	1.00	0.00
ATOM	7330	HW1	SOL	1749	19.570	9.450	6.650	1.00	0.00
ATOM	7331	HW2	SOL	1749	20.460	8.260	6.360	1.00	0.00
ATOM	7332	OW	SOL	1750	3.370	6.780	29.410	1.00	0.00
ATOM	7333	HW1	SOL	1750	3.790	5.990	29.730	1.00	0.00
ATOM	7334	HW2	SOL	1750	4.020	7.190	28.840	1.00	0.00
ATOM	7335	OW	SOL	1751	52.780	21.960	42.760	1.00	0.00
ATOM	7336	HW1	SOL	1751	52.430	22.730	43.220	1.00	0.00
ATOM	7337	HW2	SOL	1751	52.730	21.250	43.400	1.00	0.00
ATOM	7338	OW	SOL	1752	3.490	6.680	2.340	1.00	0.00
ATOM	7339	HW1	SOL	1752	3.480	6.800	1.390	1.00	0.00
ATOM	7340	HW2	SOL	1752	4.410	6.800	2.590	1.00	0.00
ATOM	7341	OW	SOL	1753	54.400	14.290	5.280	1.00	0.00
ATOM	7342	HW1	SOL	1753	53.770	15.000	5.420	1.00	0.00
ATOM	7343	HW2	SOL	1753	54.680	14.400	4.370	1.00	0.00
ATOM	7344	OW	SOL	1754	45.090	29.400	15.390	1.00	0.00
ATOM	7345	HW1	SOL	1754	45.100	30.340	15.250	1.00	0.00
ATOM	7346	HW2	SOL	1754	45.940	29.200	15.780	1.00	0.00
ATOM	7347	OW	SOL	1755	32.550	33.100	50.670	1.00	0.00

ATOM	7348	HW1	SOL	1755	32.440	34.040	50.790	1.00	0.00
ATOM	7349	HW2	SOL	1755	33.040	33.020	49.860	1.00	0.00
ATOM	7350	OW	SOL	1756	20.660	39.080	55.690	1.00	0.00
ATOM	7351	HW1	SOL	1756	21.330	39.410	55.080	1.00	0.00
ATOM	7352	HW2	SOL	1756	19.980	38.710	55.120	1.00	0.00
ATOM	7353	OW	SOL	1757	23.170	7.800	45.250	1.00	0.00
ATOM	7354	HW1	SOL	1757	23.040	8.720	45.470	1.00	0.00
ATOM	7355	HW2	SOL	1757	23.810	7.490	45.880	1.00	0.00
ATOM	7356	OW	SOL	1758	30.820	14.220	47.630	1.00	0.00
ATOM	7357	HW1	SOL	1758	29.870	14.230	47.660	1.00	0.00
ATOM	7358	HW2	SOL	1758	31.030	14.220	46.700	1.00	0.00
ATOM	7359	OW	SOL	1759	39.110	38.290	10.310	1.00	0.00
ATOM	7360	HW1	SOL	1759	38.650	37.490	10.050	1.00	0.00
ATOM	7361	HW2	SOL	1759	38.590	38.650	11.020	1.00	0.00
ATOM	7362	OW	SOL	1760	15.660	8.090	25.720	1.00	0.00
ATOM	7363	HW1	SOL	1760	14.830	8.570	25.700	1.00	0.00
ATOM	7364	HW2	SOL	1760	16.170	8.520	26.400	1.00	0.00
ATOM	7365	OW	SOL	1761	52.350	32.240	52.050	1.00	0.00
ATOM	7366	HW1	SOL	1761	52.640	31.430	51.640	1.00	0.00
ATOM	7367	HW2	SOL	1761	51.980	31.970	52.880	1.00	0.00
ATOM	7368	OW	SOL	1762	43.300	0.970	48.000	1.00	0.00
ATOM	7369	HW1	SOL	1762	44.110	1.050	48.510	1.00	0.00
ATOM	7370	HW2	SOL	1762	43.560	1.180	47.100	1.00	0.00
ATOM	7371	OW	SOL	1763	48.830	13.900	45.380	1.00	0.00
ATOM	7372	HW1	SOL	1763	49.380	13.130	45.530	1.00	0.00
ATOM	7373	HW2	SOL	1763	48.100	13.570	44.850	1.00	0.00
ATOM	7374	OW	SOL	1764	22.900	19.630	49.980	1.00	0.00
ATOM	7375	HW1	SOL	1764	23.110	19.240	49.130	1.00	0.00
ATOM	7376	HW2	SOL	1764	22.670	20.540	49.770	1.00	0.00
ATOM	7377	OW	SOL	1765	53.310	1.860	31.590	1.00	0.00
ATOM	7378	HW1	SOL	1765	52.440	1.730	31.210	1.00	0.00
ATOM	7379	HW2	SOL	1765	53.320	1.310	32.370	1.00	0.00
ATOM	7380	OW	SOL	1766	16.720	36.420	6.830	1.00	0.00
ATOM	7381	HW1	SOL	1766	15.840	36.780	6.820	1.00	0.00
ATOM	7382	HW2	SOL	1766	17.110	36.700	6.000	1.00	0.00
ATOM	7383	OW	SOL	1767	52.190	6.660	44.640	1.00	0.00
ATOM	7384	HW1	SOL	1767	52.250	6.630	45.600	1.00	0.00
ATOM	7385	HW2	SOL	1767	52.140	7.590	44.430	1.00	0.00
ATOM	7386	OW	SOL	1768	19.350	27.390	44.460	1.00	0.00
ATOM	7387	HW1	SOL	1768	19.410	27.140	45.380	1.00	0.00
ATOM	7388	HW2	SOL	1768	18.970	28.270	44.470	1.00	0.00
ATOM	7389	OW	SOL	1769	1.860	39.010	33.980	1.00	0.00
ATOM	7390	HW1	SOL	1769	1.230	38.860	34.690	1.00	0.00
ATOM	7391	HW2	SOL	1769	1.580	38.420	33.290	1.00	0.00

ATOM	7392	OW	SOL	1770	14.060	28.680	18.330	1.00	0.00
ATOM	7393	HW1	SOL	1770	14.890	28.530	17.880	1.00	0.00
ATOM	7394	HW2	SOL	1770	13.560	27.880	18.170	1.00	0.00
ATOM	7395	OW	SOL	1771	54.790	33.120	40.250	1.00	0.00
ATOM	7396	HW1	SOL	1771	55.390	32.500	39.830	1.00	0.00
ATOM	7397	HW2	SOL	1771	53.930	32.720	40.170	1.00	0.00
ATOM	7398	OW	SOL	1772	4.460	4.300	7.720	1.00	0.00
ATOM	7399	HW1	SOL	1772	5.050	4.820	8.260	1.00	0.00
ATOM	7400	HW2	SOL	1772	4.260	4.860	6.970	1.00	0.00
ATOM	7401	OW	SOL	1773	23.330	10.090	29.980	1.00	0.00
ATOM	7402	HW1	SOL	1773	24.170	10.330	29.580	1.00	0.00
ATOM	7403	HW2	SOL	1773	23.150	9.210	29.650	1.00	0.00
ATOM	7404	OW	SOL	1774	55.300	5.100	15.940	1.00	0.00
ATOM	7405	HW1	SOL	1774	54.850	5.320	15.130	1.00	0.00
ATOM	7406	HW2	SOL	1774	55.900	5.830	16.090	1.00	0.00
ATOM	7407	OW	SOL	1775	19.620	10.810	47.850	1.00	0.00
ATOM	7408	HW1	SOL	1775	19.890	11.480	47.210	1.00	0.00
ATOM	7409	HW2	SOL	1775	20.380	10.250	47.940	1.00	0.00
ATOM	7410	OW	SOL	1776	24.050	17.060	51.670	1.00	0.00
ATOM	7411	HW1	SOL	1776	23.990	17.430	52.550	1.00	0.00
ATOM	7412	HW2	SOL	1776	23.620	17.700	51.110	1.00	0.00
ATOM	7413	OW	SOL	1777	24.170	50.950	45.600	1.00	0.00
ATOM	7414	HW1	SOL	1777	24.600	50.840	44.760	1.00	0.00
ATOM	7415	HW2	SOL	1777	23.910	51.870	45.620	1.00	0.00
ATOM	7416	OW	SOL	1778	16.420	37.900	2.950	1.00	0.00
ATOM	7417	HW1	SOL	1778	16.460	37.400	2.130	1.00	0.00
ATOM	7418	HW2	SOL	1778	16.040	37.280	3.570	1.00	0.00
ATOM	7419	OW	SOL	1779	50.070	32.180	14.840	1.00	0.00
ATOM	7420	HW1	SOL	1779	49.790	33.080	14.690	1.00	0.00
ATOM	7421	HW2	SOL	1779	49.260	31.690	14.970	1.00	0.00
ATOM	7422	OW	SOL	1780	25.930	20.500	46.950	1.00	0.00
ATOM	7423	HW1	SOL	1780	25.950	19.580	46.710	1.00	0.00
ATOM	7424	HW2	SOL	1780	26.010	20.490	47.910	1.00	0.00
ATOM	7425	OW	SOL	1781	13.900	52.530	12.820	1.00	0.00
ATOM	7426	HW1	SOL	1781	14.490	52.290	12.110	1.00	0.00
ATOM	7427	HW2	SOL	1781	13.920	53.490	12.840	1.00	0.00
ATOM	7428	OW	SOL	1782	23.630	23.410	23.080	1.00	0.00
ATOM	7429	HW1	SOL	1782	23.680	22.530	22.710	1.00	0.00
ATOM	7430	HW2	SOL	1782	24.520	23.760	22.990	1.00	0.00
ATOM	7431	OW	SOL	1783	22.730	19.440	15.620	1.00	0.00
ATOM	7432	HW1	SOL	1783	21.890	18.980	15.560	1.00	0.00
ATOM	7433	HW2	SOL	1783	22.570	20.140	16.250	1.00	0.00
ATOM	7434	OW	SOL	1784	11.150	0.750	44.850	1.00	0.00
ATOM	7435	HW1	SOL	1784	10.210	0.900	44.960	1.00	0.00

ATOM	7436	HW2	SOL	1784	11.390	1.270	44.080	1.00	0.00
ATOM	7437	OW	SOL	1785	21.120	21.700	16.710	1.00	0.00
ATOM	7438	HW1	SOL	1785	20.250	21.880	17.060	1.00	0.00
ATOM	7439	HW2	SOL	1785	21.560	22.550	16.720	1.00	0.00
ATOM	7440	OW	SOL	1786	9.590	54.390	12.450	1.00	0.00
ATOM	7441	HW1	SOL	1786	10.220	53.670	12.410	1.00	0.00
ATOM	7442	HW2	SOL	1786	9.690	54.830	11.600	1.00	0.00
ATOM	7443	OW	SOL	1787	40.820	36.610	39.430	1.00	0.00
ATOM	7444	HW1	SOL	1787	40.750	35.660	39.550	1.00	0.00
ATOM	7445	HW2	SOL	1787	41.680	36.830	39.780	1.00	0.00
ATOM	7446	OW	SOL	1788	4.630	53.310	5.290	1.00	0.00
ATOM	7447	HW1	SOL	1788	4.930	52.410	5.130	1.00	0.00
ATOM	7448	HW2	SOL	1788	4.140	53.550	4.500	1.00	0.00
ATOM	7449	OW	SOL	1789	15.610	26.560	40.680	1.00	0.00
ATOM	7450	HW1	SOL	1789	15.840	26.620	41.610	1.00	0.00
ATOM	7451	HW2	SOL	1789	15.750	25.630	40.470	1.00	0.00
ATOM	7452	OW	SOL	1790	2.060	55.320	34.530	1.00	0.00
ATOM	7453	HW1	SOL	1790	1.410	55.970	34.790	1.00	0.00
ATOM	7454	HW2	SOL	1790	2.820	55.840	34.260	1.00	0.00
ATOM	7455	OW	SOL	1791	18.720	36.600	4.790	1.00	0.00
ATOM	7456	HW1	SOL	1791	18.940	37.330	4.220	1.00	0.00
ATOM	7457	HW2	SOL	1791	18.870	36.940	5.680	1.00	0.00
ATOM	7458	OW	SOL	1792	40.910	8.880	34.460	1.00	0.00
ATOM	7459	HW1	SOL	1792	41.440	9.610	34.140	1.00	0.00
ATOM	7460	HW2	SOL	1792	41.520	8.150	34.510	1.00	0.00
ATOM	7461	OW	SOL	1793	30.110	40.520	21.370	1.00	0.00
ATOM	7462	HW1	SOL	1793	29.360	40.310	20.820	1.00	0.00
ATOM	7463	HW2	SOL	1793	30.780	40.830	20.750	1.00	0.00
ATOM	7464	OW	SOL	1794	49.870	54.470	18.290	1.00	0.00
ATOM	7465	HW1	SOL	1794	50.800	54.300	18.190	1.00	0.00
ATOM	7466	HW2	SOL	1794	49.810	55.080	19.020	1.00	0.00
ATOM	7467	OW	SOL	1795	5.360	6.660	48.120	1.00	0.00
ATOM	7468	HW1	SOL	1795	6.030	7.260	48.440	1.00	0.00
ATOM	7469	HW2	SOL	1795	4.530	7.100	48.310	1.00	0.00
ATOM	7470	OW	SOL	1796	26.070	38.120	11.700	1.00	0.00
ATOM	7471	HW1	SOL	1796	25.540	37.320	11.630	1.00	0.00
ATOM	7472	HW2	SOL	1796	25.500	38.810	11.370	1.00	0.00
ATOM	7473	OW	SOL	1797	8.890	13.270	16.300	1.00	0.00
ATOM	7474	HW1	SOL	1797	8.610	14.180	16.190	1.00	0.00
ATOM	7475	HW2	SOL	1797	8.330	12.930	17.000	1.00	0.00
ATOM	7476	OW	SOL	1798	15.180	14.320	32.940	1.00	0.00
ATOM	7477	HW1	SOL	1798	15.320	15.250	33.060	1.00	0.00
ATOM	7478	HW2	SOL	1798	16.040	13.920	33.050	1.00	0.00
ATOM	7479	OW	SOL	1799	9.330	46.010	38.690	1.00	0.00

ATOM	7480	HW1	SOL	1799	9.480	46.910	38.390	1.00	0.00
ATOM	7481	HW2	SOL	1799	9.120	45.530	37.890	1.00	0.00
ATOM	7482	OW	SOL	1800	55.670	24.860	29.770	1.00	0.00
ATOM	7483	HW1	SOL	1800	54.880	24.870	30.300	1.00	0.00
ATOM	7484	HW2	SOL	1800	56.170	24.110	30.100	1.00	0.00
ATOM	7485	OW	SOL	1801	37.360	36.140	9.860	1.00	0.00
ATOM	7486	HW1	SOL	1801	36.550	36.490	10.240	1.00	0.00
ATOM	7487	HW2	SOL	1801	37.520	35.330	10.360	1.00	0.00
ATOM	7488	OW	SOL	1802	41.390	1.870	52.820	1.00	0.00
ATOM	7489	HW1	SOL	1802	42.100	1.360	53.210	1.00	0.00
ATOM	7490	HW2	SOL	1802	41.320	2.650	53.380	1.00	0.00
ATOM	7491	OW	SOL	1803	13.750	33.210	48.300	1.00	0.00
ATOM	7492	HW1	SOL	1803	13.870	34.100	48.610	1.00	0.00
ATOM	7493	HW2	SOL	1803	14.640	32.890	48.150	1.00	0.00
ATOM	7494	OW	SOL	1804	42.310	20.300	13.060	1.00	0.00
ATOM	7495	HW1	SOL	1804	42.630	20.840	12.340	1.00	0.00
ATOM	7496	HW2	SOL	1804	41.540	20.780	13.390	1.00	0.00
ATOM	7497	OW	SOL	1805	30.710	16.310	6.420	1.00	0.00
ATOM	7498	HW1	SOL	1805	30.170	17.080	6.610	1.00	0.00
ATOM	7499	HW2	SOL	1805	30.090	15.660	6.080	1.00	0.00
ATOM	7500	OW	SOL	1806	50.320	51.770	21.330	1.00	0.00
ATOM	7501	HW1	SOL	1806	50.000	51.380	20.510	1.00	0.00
ATOM	7502	HW2	SOL	1806	50.910	51.110	21.690	1.00	0.00
ATOM	7503	OW	SOL	1807	44.850	19.770	18.710	1.00	0.00
ATOM	7504	HW1	SOL	1807	44.570	19.940	17.810	1.00	0.00
ATOM	7505	HW2	SOL	1807	45.430	20.510	18.920	1.00	0.00
ATOM	7506	OW	SOL	1808	53.970	29.100	32.640	1.00	0.00
ATOM	7507	HW1	SOL	1808	54.750	29.420	32.190	1.00	0.00
ATOM	7508	HW2	SOL	1808	53.980	29.540	33.490	1.00	0.00
ATOM	7509	OW	SOL	1809	14.100	4.350	13.740	1.00	0.00
ATOM	7510	HW1	SOL	1809	14.590	4.300	14.560	1.00	0.00
ATOM	7511	HW2	SOL	1809	14.210	3.490	13.340	1.00	0.00
ATOM	7512	OW	SOL	1810	26.980	24.230	39.080	1.00	0.00
ATOM	7513	HW1	SOL	1810	26.170	23.720	39.060	1.00	0.00
ATOM	7514	HW2	SOL	1810	26.680	25.140	39.070	1.00	0.00
ATOM	7515	OW	SOL	1811	13.010	18.180	40.930	1.00	0.00
ATOM	7516	HW1	SOL	1811	13.110	19.040	41.340	1.00	0.00
ATOM	7517	HW2	SOL	1811	12.060	18.040	40.910	1.00	0.00
ATOM	7518	OW	SOL	1812	10.680	54.520	29.780	1.00	0.00
ATOM	7519	HW1	SOL	1812	11.570	54.840	29.630	1.00	0.00
ATOM	7520	HW2	SOL	1812	10.320	55.100	30.460	1.00	0.00
ATOM	7521	OW	SOL	1813	1.230	37.830	29.100	1.00	0.00
ATOM	7522	HW1	SOL	1813	1.060	36.940	28.780	1.00	0.00
ATOM	7523	HW2	SOL	1813	0.700	38.390	28.530	1.00	0.00

ATOM	7524	OW	SOL	1814	12.860	33.310	5.210	1.00	0.00
ATOM	7525	HW1	SOL	1814	13.770	33.340	4.930	1.00	0.00
ATOM	7526	HW2	SOL	1814	12.900	33.110	6.140	1.00	0.00
ATOM	7527	OW	SOL	1815	26.640	43.120	36.750	1.00	0.00
ATOM	7528	HW1	SOL	1815	27.410	43.670	36.580	1.00	0.00
ATOM	7529	HW2	SOL	1815	26.230	43.000	35.900	1.00	0.00
ATOM	7530	OW	SOL	1816	54.480	16.950	2.610	1.00	0.00
ATOM	7531	HW1	SOL	1816	53.830	16.640	3.240	1.00	0.00
ATOM	7532	HW2	SOL	1816	54.480	16.290	1.910	1.00	0.00
ATOM	7533	OW	SOL	1817	28.620	2.900	9.300	1.00	0.00
ATOM	7534	HW1	SOL	1817	27.700	2.760	9.540	1.00	0.00
ATOM	7535	HW2	SOL	1817	28.570	3.380	8.470	1.00	0.00
ATOM	7536	OW	SOL	1818	27.550	18.130	51.150	1.00	0.00
ATOM	7537	HW1	SOL	1818	26.960	17.870	50.450	1.00	0.00
ATOM	7538	HW2	SOL	1818	28.420	17.900	50.830	1.00	0.00
ATOM	7539	OW	SOL	1819	26.170	8.320	36.160	1.00	0.00
ATOM	7540	HW1	SOL	1819	26.360	7.540	36.680	1.00	0.00
ATOM	7541	HW2	SOL	1819	25.300	8.150	35.790	1.00	0.00
ATOM	7542	OW	SOL	1820	19.220	5.620	12.990	1.00	0.00
ATOM	7543	HW1	SOL	1820	19.910	5.530	12.340	1.00	0.00
ATOM	7544	HW2	SOL	1820	18.970	6.550	12.940	1.00	0.00
ATOM	7545	OW	SOL	1821	4.740	33.240	23.520	1.00	0.00
ATOM	7546	HW1	SOL	1821	5.390	32.560	23.720	1.00	0.00
ATOM	7547	HW2	SOL	1821	4.710	33.260	22.560	1.00	0.00
ATOM	7548	OW	SOL	1822	44.720	41.780	7.400	1.00	0.00
ATOM	7549	HW1	SOL	1822	44.700	42.070	8.310	1.00	0.00
ATOM	7550	HW2	SOL	1822	44.450	42.540	6.900	1.00	0.00
ATOM	7551	OW	SOL	1823	13.240	25.560	34.230	1.00	0.00
ATOM	7552	HW1	SOL	1823	12.750	26.280	33.840	1.00	0.00
ATOM	7553	HW2	SOL	1823	14.150	25.710	33.980	1.00	0.00
ATOM	7554	OW	SOL	1824	17.250	14.880	11.280	1.00	0.00
ATOM	7555	HW1	SOL	1824	16.530	14.310	11.560	1.00	0.00
ATOM	7556	HW2	SOL	1824	17.920	14.290	10.960	1.00	0.00
ATOM	7557	OW	SOL	1825	30.820	41.300	0.800	1.00	0.00
ATOM	7558	HW1	SOL	1825	31.500	40.700	0.490	1.00	0.00
ATOM	7559	HW2	SOL	1825	30.380	41.600	0.000	1.00	0.00
ATOM	7560	OW	SOL	1826	54.490	12.400	51.440	1.00	0.00
ATOM	7561	HW1	SOL	1826	53.890	12.100	50.760	1.00	0.00
ATOM	7562	HW2	SOL	1826	54.960	13.140	51.040	1.00	0.00
ATOM	7563	OW	SOL	1827	36.660	5.950	19.910	1.00	0.00
ATOM	7564	HW1	SOL	1827	37.240	5.500	20.530	1.00	0.00
ATOM	7565	HW2	SOL	1827	36.830	5.520	19.070	1.00	0.00
ATOM	7566	OW	SOL	1828	36.600	48.320	51.530	1.00	0.00
ATOM	7567	HW1	SOL	1828	36.380	48.740	52.370	1.00	0.00

ATOM	7568	HW2	SOL	1828	37.240	48.910	51.130	1.00	0.00
ATOM	7569	OW	SOL	1829	16.430	10.430	50.350	1.00	0.00
ATOM	7570	HW1	SOL	1829	15.530	10.200	50.580	1.00	0.00
ATOM	7571	HW2	SOL	1829	16.940	9.640	50.560	1.00	0.00
ATOM	7572	OW	SOL	1830	41.840	3.930	9.390	1.00	0.00
ATOM	7573	HW1	SOL	1830	41.190	3.850	8.690	1.00	0.00
ATOM	7574	HW2	SOL	1830	41.910	4.870	9.550	1.00	0.00
ATOM	7575	OW	SOL	1831	48.630	3.630	47.750	1.00	0.00
ATOM	7576	HW1	SOL	1831	48.130	4.420	47.540	1.00	0.00
ATOM	7577	HW2	SOL	1831	47.970	2.940	47.820	1.00	0.00
ATOM	7578	OW	SOL	1832	12.480	12.080	43.570	1.00	0.00
ATOM	7579	HW1	SOL	1832	11.930	11.970	44.340	1.00	0.00
ATOM	7580	HW2	SOL	1832	12.630	11.190	43.250	1.00	0.00
ATOM	7581	OW	SOL	1833	12.930	12.360	34.290	1.00	0.00
ATOM	7582	HW1	SOL	1833	13.180	12.900	33.540	1.00	0.00
ATOM	7583	HW2	SOL	1833	13.600	11.670	34.320	1.00	0.00
ATOM	7584	OW	SOL	1834	53.450	11.230	13.070	1.00	0.00
ATOM	7585	HW1	SOL	1834	54.190	11.720	12.720	1.00	0.00
ATOM	7586	HW2	SOL	1834	52.690	11.800	12.930	1.00	0.00
ATOM	7587	OW	SOL	1835	52.790	46.930	18.840	1.00	0.00
ATOM	7588	HW1	SOL	1835	53.710	47.190	18.930	1.00	0.00
ATOM	7589	HW2	SOL	1835	52.730	46.120	19.340	1.00	0.00
ATOM	7590	OW	SOL	1836	3.200	12.870	3.620	1.00	0.00
ATOM	7591	HW1	SOL	1836	2.920	13.100	4.510	1.00	0.00
ATOM	7592	HW2	SOL	1836	3.560	13.680	3.260	1.00	0.00
ATOM	7593	OW	SOL	1837	45.450	17.850	38.480	1.00	0.00
ATOM	7594	HW1	SOL	1837	46.170	17.270	38.730	1.00	0.00
ATOM	7595	HW2	SOL	1837	45.810	18.730	38.590	1.00	0.00
ATOM	7596	OW	SOL	1838	19.630	15.740	13.860	1.00	0.00
ATOM	7597	HW1	SOL	1838	19.940	16.090	13.020	1.00	0.00
ATOM	7598	HW2	SOL	1838	19.960	14.840	13.880	1.00	0.00
ATOM	7599	OW	SOL	1839	54.640	2.790	44.520	1.00	0.00
ATOM	7600	HW1	SOL	1839	54.350	2.610	43.630	1.00	0.00
ATOM	7601	HW2	SOL	1839	55.370	2.180	44.660	1.00	0.00
ATOM	7602	OW	SOL	1840	3.490	4.240	12.450	1.00	0.00
ATOM	7603	HW1	SOL	1840	3.590	3.470	11.890	1.00	0.00
ATOM	7604	HW2	SOL	1840	2.810	3.990	13.080	1.00	0.00
ATOM	7605	OW	SOL	1841	16.100	19.890	0.860	1.00	0.00
ATOM	7606	HW1	SOL	1841	15.840	19.050	1.250	1.00	0.00
ATOM	7607	HW2	SOL	1841	15.270	20.310	0.630	1.00	0.00
ATOM	7608	OW	SOL	1842	49.540	9.850	48.270	1.00	0.00
ATOM	7609	HW1	SOL	1842	49.510	10.150	47.360	1.00	0.00
ATOM	7610	HW2	SOL	1842	48.720	9.360	48.380	1.00	0.00
ATOM	7611	OW	SOL	1843	42.330	55.680	7.300	1.00	0.00

ATOM	7612	HW1	SOL	1843	41.560	55.640	6.730	1.00	0.00
ATOM	7613	HW2	SOL	1843	42.870	56.370	6.910	1.00	0.00
ATOM	7614	OW	SOL	1844	3.240	5.870	51.040	1.00	0.00
ATOM	7615	HW1	SOL	1844	3.360	6.510	51.740	1.00	0.00
ATOM	7616	HW2	SOL	1844	4.130	5.730	50.700	1.00	0.00
ATOM	7617	OW	SOL	1845	51.950	10.080	18.760	1.00	0.00
ATOM	7618	HW1	SOL	1845	51.340	9.350	18.740	1.00	0.00
ATOM	7619	HW2	SOL	1845	51.400	10.850	18.930	1.00	0.00
ATOM	7620	OW	SOL	1846	3.120	41.900	47.330	1.00	0.00
ATOM	7621	HW1	SOL	1846	2.210	42.140	47.150	1.00	0.00
ATOM	7622	HW2	SOL	1846	3.540	41.910	46.470	1.00	0.00
ATOM	7623	OW	SOL	1847	30.520	48.400	43.480	1.00	0.00
ATOM	7624	HW1	SOL	1847	30.990	47.570	43.560	1.00	0.00
ATOM	7625	HW2	SOL	1847	31.210	49.070	43.520	1.00	0.00
ATOM	7626	OW	SOL	1848	31.900	22.890	13.270	1.00	0.00
ATOM	7627	HW1	SOL	1848	32.740	23.060	13.690	1.00	0.00
ATOM	7628	HW2	SOL	1848	32.000	23.220	12.380	1.00	0.00
ATOM	7629	OW	SOL	1849	4.610	48.950	29.970	1.00	0.00
ATOM	7630	HW1	SOL	1849	4.790	48.050	30.260	1.00	0.00
ATOM	7631	HW2	SOL	1849	5.130	49.050	29.180	1.00	0.00
ATOM	7632	OW	SOL	1850	52.830	24.260	44.760	1.00	0.00
ATOM	7633	HW1	SOL	1850	52.110	24.880	44.650	1.00	0.00
ATOM	7634	HW2	SOL	1850	52.800	24.010	45.680	1.00	0.00
ATOM	7635	OW	SOL	1851	12.930	51.770	34.610	1.00	0.00
ATOM	7636	HW1	SOL	1851	13.550	51.340	34.020	1.00	0.00
ATOM	7637	HW2	SOL	1851	13.170	51.460	35.480	1.00	0.00
ATOM	7638	OW	SOL	1852	12.740	4.420	52.250	1.00	0.00
ATOM	7639	HW1	SOL	1852	12.560	3.890	53.030	1.00	0.00
ATOM	7640	HW2	SOL	1852	12.310	5.260	52.430	1.00	0.00
ATOM	7641	OW	SOL	1853	28.860	27.890	16.300	1.00	0.00
ATOM	7642	HW1	SOL	1853	28.240	28.570	16.550	1.00	0.00
ATOM	7643	HW2	SOL	1853	28.460	27.070	16.580	1.00	0.00
ATOM	7644	OW	SOL	1854	6.240	23.220	31.070	1.00	0.00
ATOM	7645	HW1	SOL	1854	5.430	23.710	30.930	1.00	0.00
ATOM	7646	HW2	SOL	1854	6.540	23.480	31.940	1.00	0.00
ATOM	7647	OW	SOL	1855	8.190	6.920	0.910	1.00	0.00
ATOM	7648	HW1	SOL	1855	7.860	7.780	1.180	1.00	0.00
ATOM	7649	HW2	SOL	1855	8.320	6.440	1.720	1.00	0.00
ATOM	7650	OW	SOL	1856	49.780	11.050	16.190	1.00	0.00
ATOM	7651	HW1	SOL	1856	50.680	11.360	16.220	1.00	0.00
ATOM	7652	HW2	SOL	1856	49.270	11.810	15.910	1.00	0.00
ATOM	7653	OW	SOL	1857	40.420	48.350	6.740	1.00	0.00
ATOM	7654	HW1	SOL	1857	39.940	49.170	6.840	1.00	0.00
ATOM	7655	HW2	SOL	1857	39.760	47.720	6.460	1.00	0.00

ATOM	7656	OW	SOL	1858	43.410	11.900	39.620	1.00	0.00
ATOM	7657	HW1	SOL	1858	43.030	11.460	40.380	1.00	0.00
ATOM	7658	HW2	SOL	1858	42.660	12.230	39.130	1.00	0.00
ATOM	7659	OW	SOL	1859	13.360	28.880	4.690	1.00	0.00
ATOM	7660	HW1	SOL	1859	12.920	29.660	4.360	1.00	0.00
ATOM	7661	HW2	SOL	1859	12.820	28.150	4.370	1.00	0.00
ATOM	7662	OW	SOL	1860	38.210	53.240	19.770	1.00	0.00
ATOM	7663	HW1	SOL	1860	38.000	53.410	18.850	1.00	0.00
ATOM	7664	HW2	SOL	1860	39.050	53.670	19.910	1.00	0.00
ATOM	7665	OW	SOL	1861	37.400	37.990	53.480	1.00	0.00
ATOM	7666	HW1	SOL	1861	38.150	38.580	53.380	1.00	0.00
ATOM	7667	HW2	SOL	1861	37.010	38.240	54.320	1.00	0.00
ATOM	7668	OW	SOL	1862	38.430	39.210	49.080	1.00	0.00
ATOM	7669	HW1	SOL	1862	37.740	38.970	49.710	1.00	0.00
ATOM	7670	HW2	SOL	1862	38.020	39.880	48.530	1.00	0.00
ATOM	7671	OW	SOL	1863	13.530	55.490	5.350	1.00	0.00
ATOM	7672	HW1	SOL	1863	14.290	54.910	5.460	1.00	0.00
ATOM	7673	HW2	SOL	1863	13.500	56.000	6.150	1.00	0.00
ATOM	7674	OW	SOL	1864	9.570	50.730	33.570	1.00	0.00
ATOM	7675	HW1	SOL	1864	9.670	51.210	34.400	1.00	0.00
ATOM	7676	HW2	SOL	1864	8.660	50.440	33.580	1.00	0.00
ATOM	7677	OW	SOL	1865	10.500	48.670	29.480	1.00	0.00
ATOM	7678	HW1	SOL	1865	11.150	49.230	29.920	1.00	0.00
ATOM	7679	HW2	SOL	1865	10.530	48.950	28.560	1.00	0.00
ATOM	7680	OW	SOL	1866	30.440	29.850	26.540	1.00	0.00
ATOM	7681	HW1	SOL	1866	30.830	30.440	25.900	1.00	0.00
ATOM	7682	HW2	SOL	1866	31.170	29.270	26.790	1.00	0.00
ATOM	7683	OW	SOL	1867	41.840	34.070	39.630	1.00	0.00
ATOM	7684	HW1	SOL	1867	42.280	34.050	40.480	1.00	0.00
ATOM	7685	HW2	SOL	1867	41.720	33.140	39.400	1.00	0.00
ATOM	7686	OW	SOL	1868	42.490	18.780	35.180	1.00	0.00
ATOM	7687	HW1	SOL	1868	42.120	18.260	35.890	1.00	0.00
ATOM	7688	HW2	SOL	1868	42.830	19.570	35.610	1.00	0.00
ATOM	7689	OW	SOL	1869	0.280	46.750	48.020	1.00	0.00
ATOM	7690	HW1	SOL	1869	0.450	46.240	48.810	1.00	0.00
ATOM	7691	HW2	SOL	1869	-0.410	47.370	48.280	1.00	0.00
ATOM	7692	OW	SOL	1870	38.570	6.480	33.130	1.00	0.00
ATOM	7693	HW1	SOL	1870	38.200	5.740	32.660	1.00	0.00
ATOM	7694	HW2	SOL	1870	39.490	6.260	33.250	1.00	0.00
ATOM	7695	OW	SOL	1871	15.190	4.800	8.320	1.00	0.00
ATOM	7696	HW1	SOL	1871	15.930	5.340	8.590	1.00	0.00
ATOM	7697	HW2	SOL	1871	14.970	5.110	7.440	1.00	0.00
ATOM	7698	OW	SOL	1872	4.040	6.170	20.920	1.00	0.00
ATOM	7699	HW1	SOL	1872	4.870	5.720	20.760	1.00	0.00

ATOM	7700	HW2	SOL	1872	4.120	6.990	20.430	1.00	0.00
ATOM	7701	OW	SOL	1873	52.080	42.250	45.650	1.00	0.00
ATOM	7702	HW1	SOL	1873	52.510	41.600	46.190	1.00	0.00
ATOM	7703	HW2	SOL	1873	52.180	43.070	46.130	1.00	0.00
ATOM	7704	OW	SOL	1874	42.810	51.950	4.200	1.00	0.00
ATOM	7705	HW1	SOL	1874	42.890	51.020	4.440	1.00	0.00
ATOM	7706	HW2	SOL	1874	42.840	51.950	3.250	1.00	0.00
ATOM	7707	OW	SOL	1875	55.430	29.450	38.910	1.00	0.00
ATOM	7708	HW1	SOL	1875	56.120	30.050	38.640	1.00	0.00
ATOM	7709	HW2	SOL	1875	55.190	29.740	39.790	1.00	0.00
ATOM	7710	OW	SOL	1876	3.140	44.570	21.260	1.00	0.00
ATOM	7711	HW1	SOL	1876	2.960	45.510	21.340	1.00	0.00
ATOM	7712	HW2	SOL	1876	4.060	44.520	20.990	1.00	0.00
ATOM	7713	OW	SOL	1877	33.780	22.090	20.000	1.00	0.00
ATOM	7714	HW1	SOL	1877	34.590	22.250	19.520	1.00	0.00
ATOM	7715	HW2	SOL	1877	33.090	22.360	19.390	1.00	0.00
ATOM	7716	OW	SOL	1878	45.080	9.570	23.810	1.00	0.00
ATOM	7717	HW1	SOL	1878	45.790	9.010	23.500	1.00	0.00
ATOM	7718	HW2	SOL	1878	44.390	9.460	23.150	1.00	0.00
ATOM	7719	OW	SOL	1879	0.090	3.870	11.840	1.00	0.00
ATOM	7720	HW1	SOL	1879	-0.330	4.730	11.890	1.00	0.00
ATOM	7721	HW2	SOL	1879	0.470	3.840	10.960	1.00	0.00
ATOM	7722	OW	SOL	1880	36.080	28.180	49.080	1.00	0.00
ATOM	7723	HW1	SOL	1880	35.640	28.150	48.230	1.00	0.00
ATOM	7724	HW2	SOL	1880	36.700	28.900	49.000	1.00	0.00
ATOM	7725	OW	SOL	1881	50.880	54.610	12.940	1.00	0.00
ATOM	7726	HW1	SOL	1881	51.810	54.380	12.840	1.00	0.00
ATOM	7727	HW2	SOL	1881	50.450	54.210	12.180	1.00	0.00
ATOM	7728	OW	SOL	1882	46.750	44.960	22.360	1.00	0.00
ATOM	7729	HW1	SOL	1882	46.910	45.790	21.910	1.00	0.00
ATOM	7730	HW2	SOL	1882	47.570	44.470	22.250	1.00	0.00
ATOM	7731	OW	SOL	1883	25.040	34.140	42.180	1.00	0.00
ATOM	7732	HW1	SOL	1883	25.120	34.820	41.510	1.00	0.00
ATOM	7733	HW2	SOL	1883	24.330	33.580	41.880	1.00	0.00
ATOM	7734	OW	SOL	1884	29.020	35.460	4.950	1.00	0.00
ATOM	7735	HW1	SOL	1884	28.410	34.730	4.850	1.00	0.00
ATOM	7736	HW2	SOL	1884	29.890	35.070	4.800	1.00	0.00
ATOM	7737	OW	SOL	1885	29.760	38.220	45.460	1.00	0.00
ATOM	7738	HW1	SOL	1885	29.280	37.440	45.190	1.00	0.00
ATOM	7739	HW2	SOL	1885	29.190	38.650	46.090	1.00	0.00
ATOM	7740	OW	SOL	1886	9.540	15.810	5.810	1.00	0.00
ATOM	7741	HW1	SOL	1886	8.980	15.080	6.070	1.00	0.00
ATOM	7742	HW2	SOL	1886	9.890	16.160	6.640	1.00	0.00
ATOM	7743	OW	SOL	1887	21.810	18.080	24.920	1.00	0.00

ATOM	7744	HW1	SOL	1887	20.970	17.620	24.900	1.00	0.00
ATOM	7745	HW2	SOL	1887	22.360	17.590	24.310	1.00	0.00
ATOM	7746	OW	SOL	1888	10.810	24.370	54.190	1.00	0.00
ATOM	7747	HW1	SOL	1888	10.050	23.970	53.780	1.00	0.00
ATOM	7748	HW2	SOL	1888	10.450	25.090	54.710	1.00	0.00
ATOM	7749	OW	SOL	1889	26.220	10.350	8.940	1.00	0.00
ATOM	7750	HW1	SOL	1889	27.150	10.130	8.960	1.00	0.00
ATOM	7751	HW2	SOL	1889	26.200	11.270	8.680	1.00	0.00
ATOM	7752	OW	SOL	1890	38.630	8.250	30.690	1.00	0.00
ATOM	7753	HW1	SOL	1890	39.180	9.000	30.900	1.00	0.00
ATOM	7754	HW2	SOL	1890	38.670	7.680	31.460	1.00	0.00
ATOM	7755	OW	SOL	1891	11.130	23.860	51.160	1.00	0.00
ATOM	7756	HW1	SOL	1891	10.250	23.730	50.810	1.00	0.00
ATOM	7757	HW2	SOL	1891	11.010	23.920	52.110	1.00	0.00
ATOM	7758	OW	SOL	1892	40.650	13.450	40.760	1.00	0.00
ATOM	7759	HW1	SOL	1892	41.000	13.840	41.560	1.00	0.00
ATOM	7760	HW2	SOL	1892	40.760	14.130	40.100	1.00	0.00
ATOM	7761	OW	SOL	1893	35.310	12.360	50.970	1.00	0.00
ATOM	7762	HW1	SOL	1893	35.170	13.260	50.700	1.00	0.00
ATOM	7763	HW2	SOL	1893	34.560	11.880	50.620	1.00	0.00
ATOM	7764	OW	SOL	1894	39.310	6.290	26.450	1.00	0.00
ATOM	7765	HW1	SOL	1894	38.980	6.860	27.150	1.00	0.00
ATOM	7766	HW2	SOL	1894	38.590	6.250	25.820	1.00	0.00
ATOM	7767	OW	SOL	1895	23.420	8.430	23.460	1.00	0.00
ATOM	7768	HW1	SOL	1895	22.600	8.940	23.440	1.00	0.00
ATOM	7769	HW2	SOL	1895	23.430	7.960	22.630	1.00	0.00
ATOM	7770	OW	SOL	1896	3.640	38.760	28.360	1.00	0.00
ATOM	7771	HW1	SOL	1896	2.800	38.320	28.500	1.00	0.00
ATOM	7772	HW2	SOL	1896	4.050	38.260	27.650	1.00	0.00
ATOM	7773	OW	SOL	1897	17.070	10.150	12.830	1.00	0.00
ATOM	7774	HW1	SOL	1897	16.120	10.050	12.900	1.00	0.00
ATOM	7775	HW2	SOL	1897	17.360	10.310	13.730	1.00	0.00
ATOM	7776	OW	SOL	1898	52.400	45.140	28.220	1.00	0.00
ATOM	7777	HW1	SOL	1898	51.530	45.440	28.470	1.00	0.00
ATOM	7778	HW2	SOL	1898	52.240	44.430	27.600	1.00	0.00
ATOM	7779	OW	SOL	1899	2.040	50.020	20.800	1.00	0.00
ATOM	7780	HW1	SOL	1899	2.760	49.830	20.200	1.00	0.00
ATOM	7781	HW2	SOL	1899	2.430	50.560	21.480	1.00	0.00
ATOM	7782	OW	SOL	1900	54.730	14.620	36.060	1.00	0.00
ATOM	7783	HW1	SOL	1900	54.160	15.340	36.330	1.00	0.00
ATOM	7784	HW2	SOL	1900	54.780	14.690	35.110	1.00	0.00
ATOM	7785	OW	SOL	1901	18.740	24.120	31.640	1.00	0.00
ATOM	7786	HW1	SOL	1901	18.230	24.860	31.950	1.00	0.00
ATOM	7787	HW2	SOL	1901	19.490	24.510	31.210	1.00	0.00

ATOM	7788	OW	SOL	1902	2.490	43.080	30.390	1.00	0.00
ATOM	7789	HW1	SOL	1902	3.230	43.670	30.260	1.00	0.00
ATOM	7790	HW2	SOL	1902	2.770	42.470	31.070	1.00	0.00
ATOM	7791	OW	SOL	1903	8.290	41.330	49.840	1.00	0.00
ATOM	7792	HW1	SOL	1903	9.070	41.250	49.280	1.00	0.00
ATOM	7793	HW2	SOL	1903	8.540	41.980	50.500	1.00	0.00
ATOM	7794	OW	SOL	1904	15.760	19.720	26.210	1.00	0.00
ATOM	7795	HW1	SOL	1904	15.720	19.090	26.930	1.00	0.00
ATOM	7796	HW2	SOL	1904	14.850	19.920	26.010	1.00	0.00
ATOM	7797	OW	SOL	1905	43.030	20.230	55.440	1.00	0.00
ATOM	7798	HW1	SOL	1905	43.380	20.880	54.830	1.00	0.00
ATOM	7799	HW2	SOL	1905	42.730	20.740	56.190	1.00	0.00
ATOM	7800	OW	SOL	1906	12.020	38.220	3.680	1.00	0.00
ATOM	7801	HW1	SOL	1906	11.160	38.340	3.270	1.00	0.00
ATOM	7802	HW2	SOL	1906	11.920	37.440	4.230	1.00	0.00
ATOM	7803	OW	SOL	1907	34.190	36.840	45.850	1.00	0.00
ATOM	7804	HW1	SOL	1907	35.030	37.230	46.100	1.00	0.00
ATOM	7805	HW2	SOL	1907	33.540	37.400	46.290	1.00	0.00
ATOM	7806	OW	SOL	1908	6.180	41.360	12.200	1.00	0.00
ATOM	7807	HW1	SOL	1908	6.930	41.220	12.770	1.00	0.00
ATOM	7808	HW2	SOL	1908	6.510	41.960	11.530	1.00	0.00
ATOM	7809	OW	SOL	1909	27.610	50.390	49.940	1.00	0.00
ATOM	7810	HW1	SOL	1909	26.860	50.920	49.680	1.00	0.00
ATOM	7811	HW2	SOL	1909	27.990	50.090	49.110	1.00	0.00
ATOM	7812	OW	SOL	1910	53.040	7.740	16.500	1.00	0.00
ATOM	7813	HW1	SOL	1910	53.620	7.100	16.090	1.00	0.00
ATOM	7814	HW2	SOL	1910	53.460	7.940	17.340	1.00	0.00
ATOM	7815	OW	SOL	1911	26.700	48.820	2.990	1.00	0.00
ATOM	7816	HW1	SOL	1911	26.980	48.010	3.420	1.00	0.00
ATOM	7817	HW2	SOL	1911	25.760	48.700	2.850	1.00	0.00
ATOM	7818	OW	SOL	1912	9.020	52.900	20.600	1.00	0.00
ATOM	7819	HW1	SOL	1912	8.420	53.100	19.890	1.00	0.00
ATOM	7820	HW2	SOL	1912	9.320	53.750	20.920	1.00	0.00
ATOM	7821	OW	SOL	1913	41.820	53.860	53.830	1.00	0.00
ATOM	7822	HW1	SOL	1913	41.300	53.800	54.640	1.00	0.00
ATOM	7823	HW2	SOL	1913	42.450	54.560	54.010	1.00	0.00
ATOM	7824	OW	SOL	1914	46.250	15.560	28.540	1.00	0.00
ATOM	7825	HW1	SOL	1914	46.400	16.040	29.350	1.00	0.00
ATOM	7826	HW2	SOL	1914	45.420	15.920	28.210	1.00	0.00
ATOM	7827	OW	SOL	1915	20.000	28.440	26.120	1.00	0.00
ATOM	7828	HW1	SOL	1915	20.490	29.230	26.350	1.00	0.00
ATOM	7829	HW2	SOL	1915	19.700	28.090	26.960	1.00	0.00
ATOM	7830	OW	SOL	1916	32.300	54.990	34.310	1.00	0.00
ATOM	7831	HW1	SOL	1916	31.950	55.320	35.140	1.00	0.00

ATOM	7832	HW2	SOL	1916	31.550	55.000	33.710	1.00	0.00
ATOM	7833	OW	SOL	1917	26.200	27.210	24.190	1.00	0.00
ATOM	7834	HW1	SOL	1917	26.940	27.820	24.210	1.00	0.00
ATOM	7835	HW2	SOL	1917	25.680	27.430	24.960	1.00	0.00
ATOM	7836	OW	SOL	1918	6.830	0.580	5.420	1.00	0.00
ATOM	7837	HW1	SOL	1918	6.070	-0.010	5.410	1.00	0.00
ATOM	7838	HW2	SOL	1918	7.560	0.020	5.690	1.00	0.00
ATOM	7839	OW	SOL	1919	43.780	46.140	2.780	1.00	0.00
ATOM	7840	HW1	SOL	1919	43.430	46.990	2.500	1.00	0.00
ATOM	7841	HW2	SOL	1919	44.610	46.050	2.320	1.00	0.00
ATOM	7842	OW	SOL	1920	17.680	12.260	32.110	1.00	0.00
ATOM	7843	HW1	SOL	1920	18.120	11.440	32.300	1.00	0.00
ATOM	7844	HW2	SOL	1920	17.850	12.420	31.180	1.00	0.00
ATOM	7845	OW	SOL	1921	22.980	7.250	49.880	1.00	0.00
ATOM	7846	HW1	SOL	1921	23.280	7.090	50.780	1.00	0.00
ATOM	7847	HW2	SOL	1921	22.540	8.100	49.910	1.00	0.00
ATOM	7848	OW	SOL	1922	52.240	9.320	44.390	1.00	0.00
ATOM	7849	HW1	SOL	1922	53.020	9.620	44.870	1.00	0.00
ATOM	7850	HW2	SOL	1922	52.320	9.710	43.530	1.00	0.00
ATOM	7851	OW	SOL	1923	48.960	15.900	5.320	1.00	0.00
ATOM	7852	HW1	SOL	1923	49.420	15.810	4.480	1.00	0.00
ATOM	7853	HW2	SOL	1923	49.650	15.800	5.970	1.00	0.00
ATOM	7854	OW	SOL	1924	45.580	25.360	21.500	1.00	0.00
ATOM	7855	HW1	SOL	1924	45.910	25.910	20.790	1.00	0.00
ATOM	7856	HW2	SOL	1924	44.850	25.850	21.870	1.00	0.00
ATOM	7857	OW	SOL	1925	54.190	36.930	24.490	1.00	0.00
ATOM	7858	HW1	SOL	1925	54.830	36.230	24.550	1.00	0.00
ATOM	7859	HW2	SOL	1925	54.020	37.020	23.550	1.00	0.00
ATOM	7860	OW	SOL	1926	40.830	29.200	4.190	1.00	0.00
ATOM	7861	HW1	SOL	1926	41.120	28.860	5.040	1.00	0.00
ATOM	7862	HW2	SOL	1926	40.440	30.040	4.390	1.00	0.00
ATOM	7863	OW	SOL	1927	14.310	21.430	37.820	1.00	0.00
ATOM	7864	HW1	SOL	1927	13.460	21.030	37.670	1.00	0.00
ATOM	7865	HW2	SOL	1927	14.750	21.400	36.970	1.00	0.00
ATOM	7866	OW	SOL	1928	12.290	26.650	3.400	1.00	0.00
ATOM	7867	HW1	SOL	1928	12.580	26.290	2.560	1.00	0.00
ATOM	7868	HW2	SOL	1928	11.970	25.900	3.890	1.00	0.00
ATOM	7869	OW	SOL	1929	1.300	47.240	8.290	1.00	0.00
ATOM	7870	HW1	SOL	1929	2.240	47.430	8.360	1.00	0.00
ATOM	7871	HW2	SOL	1929	1.070	47.520	7.400	1.00	0.00
ATOM	7872	OW	SOL	1930	1.370	22.430	6.030	1.00	0.00
ATOM	7873	HW1	SOL	1930	1.580	22.170	5.130	1.00	0.00
ATOM	7874	HW2	SOL	1930	1.680	21.710	6.570	1.00	0.00
ATOM	7875	OW	SOL	1931	27.230	8.800	32.920	1.00	0.00

ATOM	7876	HW1	SOL	1931	26.690	8.150	33.360	1.00	0.00
ATOM	7877	HW2	SOL	1931	26.710	9.610	32.970	1.00	0.00
ATOM	7878	OW	SOL	1932	47.750	15.880	8.090	1.00	0.00
ATOM	7879	HW1	SOL	1932	48.030	15.630	8.970	1.00	0.00
ATOM	7880	HW2	SOL	1932	48.520	15.710	7.540	1.00	0.00
ATOM	7881	OW	SOL	1933	9.550	9.720	43.910	1.00	0.00
ATOM	7882	HW1	SOL	1933	9.530	9.070	44.600	1.00	0.00
ATOM	7883	HW2	SOL	1933	10.260	9.440	43.330	1.00	0.00
ATOM	7884	OW	SOL	1934	5.100	19.300	43.090	1.00	0.00
ATOM	7885	HW1	SOL	1934	4.220	19.400	43.460	1.00	0.00
ATOM	7886	HW2	SOL	1934	4.990	18.640	42.390	1.00	0.00
ATOM	7887	OW	SOL	1935	55.460	3.480	32.510	1.00	0.00
ATOM	7888	HW1	SOL	1935	55.980	3.080	31.810	1.00	0.00
ATOM	7889	HW2	SOL	1935	54.600	3.060	32.440	1.00	0.00
ATOM	7890	OW	SOL	1936	20.800	11.500	21.730	1.00	0.00
ATOM	7891	HW1	SOL	1936	21.450	12.010	21.240	1.00	0.00
ATOM	7892	HW2	SOL	1936	21.110	10.590	21.670	1.00	0.00
ATOM	7893	OW	SOL	1937	48.420	32.760	2.580	1.00	0.00
ATOM	7894	HW1	SOL	1937	48.380	33.560	3.100	1.00	0.00
ATOM	7895	HW2	SOL	1937	48.600	32.060	3.210	1.00	0.00
ATOM	7896	OW	SOL	1938	39.420	24.820	39.030	1.00	0.00
ATOM	7897	HW1	SOL	1938	39.230	24.340	39.830	1.00	0.00
ATOM	7898	HW2	SOL	1938	40.360	24.720	38.910	1.00	0.00
ATOM	7899	OW	SOL	1939	26.660	2.110	4.080	1.00	0.00
ATOM	7900	HW1	SOL	1939	27.470	1.870	4.530	1.00	0.00
ATOM	7901	HW2	SOL	1939	25.970	1.910	4.710	1.00	0.00
ATOM	7902	OW	SOL	1940	19.440	26.600	9.340	1.00	0.00
ATOM	7903	HW1	SOL	1940	19.130	27.420	8.970	1.00	0.00
ATOM	7904	HW2	SOL	1940	19.630	26.040	8.590	1.00	0.00
ATOM	7905	OW	SOL	1941	16.560	2.800	2.030	1.00	0.00
ATOM	7906	HW1	SOL	1941	16.230	2.580	1.160	1.00	0.00
ATOM	7907	HW2	SOL	1941	17.490	2.990	1.890	1.00	0.00
ATOM	7908	OW	SOL	1942	9.800	40.230	8.460	1.00	0.00
ATOM	7909	HW1	SOL	1942	10.040	39.980	9.350	1.00	0.00
ATOM	7910	HW2	SOL	1942	10.460	40.860	8.190	1.00	0.00
ATOM	7911	OW	SOL	1943	35.170	42.020	9.540	1.00	0.00
ATOM	7912	HW1	SOL	1943	35.250	41.350	8.860	1.00	0.00
ATOM	7913	HW2	SOL	1943	36.070	42.300	9.720	1.00	0.00
ATOM	7914	OW	SOL	1944	55.660	24.710	2.400	1.00	0.00
ATOM	7915	HW1	SOL	1944	55.430	25.340	1.710	1.00	0.00
ATOM	7916	HW2	SOL	1944	56.050	23.980	1.920	1.00	0.00
ATOM	7917	OW	SOL	1945	20.150	10.820	30.650	1.00	0.00
ATOM	7918	HW1	SOL	1945	20.870	10.210	30.510	1.00	0.00
ATOM	7919	HW2	SOL	1945	19.890	10.690	31.560	1.00	0.00

ATOM	7920	OW	SOL	1946	23.150	53.520	5.960	1.00	0.00
ATOM	7921	HW1	SOL	1946	24.060	53.350	5.710	1.00	0.00
ATOM	7922	HW2	SOL	1946	22.640	52.980	5.360	1.00	0.00
ATOM	7923	OW	SOL	1947	2.730	38.640	54.080	1.00	0.00
ATOM	7924	HW1	SOL	1947	1.820	38.730	53.790	1.00	0.00
ATOM	7925	HW2	SOL	1947	3.250	38.990	53.350	1.00	0.00
ATOM	7926	OW	SOL	1948	20.770	3.830	11.130	1.00	0.00
ATOM	7927	HW1	SOL	1948	21.460	3.190	10.980	1.00	0.00
ATOM	7928	HW2	SOL	1948	20.630	4.240	10.280	1.00	0.00
ATOM	7929	OW	SOL	1949	16.170	32.160	48.750	1.00	0.00
ATOM	7930	HW1	SOL	1949	16.330	32.030	49.690	1.00	0.00
ATOM	7931	HW2	SOL	1949	16.230	31.280	48.380	1.00	0.00
ATOM	7932	OW	SOL	1950	2.840	7.830	42.680	1.00	0.00
ATOM	7933	HW1	SOL	1950	3.500	7.130	42.660	1.00	0.00
ATOM	7934	HW2	SOL	1950	2.940	8.280	41.840	1.00	0.00
ATOM	7935	OW	SOL	1951	47.900	39.640	19.680	1.00	0.00
ATOM	7936	HW1	SOL	1951	47.040	39.350	19.380	1.00	0.00
ATOM	7937	HW2	SOL	1951	47.750	40.520	20.030	1.00	0.00
ATOM	7938	OW	SOL	1952	0.450	27.330	13.880	1.00	0.00
ATOM	7939	HW1	SOL	1952	-0.110	27.610	14.600	1.00	0.00
ATOM	7940	HW2	SOL	1952	-0.140	27.260	13.130	1.00	0.00
ATOM	7941	OW	SOL	1953	14.360	11.260	9.750	1.00	0.00
ATOM	7942	HW1	SOL	1953	15.230	10.910	9.920	1.00	0.00
ATOM	7943	HW2	SOL	1953	14.480	12.210	9.710	1.00	0.00
ATOM	7944	OW	SOL	1954	21.590	21.220	11.560	1.00	0.00
ATOM	7945	HW1	SOL	1954	21.480	22.150	11.380	1.00	0.00
ATOM	7946	HW2	SOL	1954	20.710	20.850	11.470	1.00	0.00
ATOM	7947	OW	SOL	1955	34.540	35.900	0.520	1.00	0.00
ATOM	7948	HW1	SOL	1955	33.950	35.520	-0.130	1.00	0.00
ATOM	7949	HW2	SOL	1955	33.980	36.060	1.280	1.00	0.00
ATOM	7950	OW	SOL	1956	8.280	24.920	34.890	1.00	0.00
ATOM	7951	HW1	SOL	1956	9.100	24.600	35.270	1.00	0.00
ATOM	7952	HW2	SOL	1956	8.510	25.780	34.540	1.00	0.00
ATOM	7953	OW	SOL	1957	31.100	40.930	45.320	1.00	0.00
ATOM	7954	HW1	SOL	1957	31.300	41.180	44.420	1.00	0.00
ATOM	7955	HW2	SOL	1957	30.610	40.110	45.240	1.00	0.00
ATOM	7956	OW	SOL	1958	31.240	1.650	14.070	1.00	0.00
ATOM	7957	HW1	SOL	1958	30.540	2.050	13.570	1.00	0.00
ATOM	7958	HW2	SOL	1958	30.860	0.840	14.420	1.00	0.00
ATOM	7959	OW	SOL	1959	2.530	45.870	50.090	1.00	0.00
ATOM	7960	HW1	SOL	1959	3.150	46.270	49.470	1.00	0.00
ATOM	7961	HW2	SOL	1959	2.990	45.100	50.420	1.00	0.00
ATOM	7962	OW	SOL	1960	27.110	15.800	2.340	1.00	0.00
ATOM	7963	HW1	SOL	1960	27.130	15.210	1.590	1.00	0.00

ATOM	7964	HW2	SOL	1960	27.640	16.550	2.080	1.00	0.00
ATOM	7965	OW	SOL	1961	1.730	40.070	38.960	1.00	0.00
ATOM	7966	HW1	SOL	1961	2.680	40.030	38.810	1.00	0.00
ATOM	7967	HW2	SOL	1961	1.350	39.520	38.280	1.00	0.00
ATOM	7968	OW	SOL	1962	29.230	15.980	38.110	1.00	0.00
ATOM	7969	HW1	SOL	1962	29.270	15.760	37.180	1.00	0.00
ATOM	7970	HW2	SOL	1962	29.640	16.840	38.170	1.00	0.00
ATOM	7971	OW	SOL	1963	55.300	14.680	9.080	1.00	0.00
ATOM	7972	HW1	SOL	1963	55.680	15.190	9.790	1.00	0.00
ATOM	7973	HW2	SOL	1963	55.840	13.900	9.020	1.00	0.00
ATOM	7974	OW	SOL	1964	17.940	21.770	50.270	1.00	0.00
ATOM	7975	HW1	SOL	1964	17.190	21.620	49.700	1.00	0.00
ATOM	7976	HW2	SOL	1964	17.550	22.130	51.080	1.00	0.00
ATOM	7977	OW	SOL	1965	28.090	24.310	52.910	1.00	0.00
ATOM	7978	HW1	SOL	1965	28.940	24.220	53.340	1.00	0.00
ATOM	7979	HW2	SOL	1965	27.470	24.460	53.630	1.00	0.00
ATOM	7980	OW	SOL	1966	12.040	31.920	10.620	1.00	0.00
ATOM	7981	HW1	SOL	1966	11.460	32.260	11.290	1.00	0.00
ATOM	7982	HW2	SOL	1966	11.460	31.630	9.920	1.00	0.00
ATOM	7983	OW	SOL	1967	26.580	8.890	5.050	1.00	0.00
ATOM	7984	HW1	SOL	1967	27.210	9.160	5.720	1.00	0.00
ATOM	7985	HW2	SOL	1967	25.790	9.390	5.260	1.00	0.00
ATOM	7986	OW	SOL	1968	4.760	25.650	24.580	1.00	0.00
ATOM	7987	HW1	SOL	1968	4.470	25.020	23.920	1.00	0.00
ATOM	7988	HW2	SOL	1968	5.050	26.410	24.070	1.00	0.00
ATOM	7989	OW	SOL	1969	33.120	18.340	24.640	1.00	0.00
ATOM	7990	HW1	SOL	1969	32.400	17.950	25.140	1.00	0.00
ATOM	7991	HW2	SOL	1969	33.120	19.260	24.890	1.00	0.00
ATOM	7992	OW	SOL	1970	20.600	28.040	6.590	1.00	0.00
ATOM	7993	HW1	SOL	1970	20.890	28.540	5.820	1.00	0.00
ATOM	7994	HW2	SOL	1970	19.740	28.410	6.800	1.00	0.00
ATOM	7995	OW	SOL	1971	5.390	8.120	28.430	1.00	0.00
ATOM	7996	HW1	SOL	1971	5.450	8.910	27.900	1.00	0.00
ATOM	7997	HW2	SOL	1971	5.830	8.350	29.250	1.00	0.00
ATOM	7998	OW	SOL	1972	37.230	50.170	47.110	1.00	0.00
ATOM	7999	HW1	SOL	1972	38.020	50.490	47.540	1.00	0.00
ATOM	8000	HW2	SOL	1972	36.760	49.690	47.800	1.00	0.00
ATOM	8001	OW	SOL	1973	18.470	29.220	21.940	1.00	0.00
ATOM	8002	HW1	SOL	1973	19.160	28.610	21.660	1.00	0.00
ATOM	8003	HW2	SOL	1973	17.860	29.230	21.200	1.00	0.00
ATOM	8004	OW	SOL	1974	40.200	33.800	12.100	1.00	0.00
ATOM	8005	HW1	SOL	1974	40.200	33.770	13.060	1.00	0.00
ATOM	8006	HW2	SOL	1974	39.460	34.370	11.880	1.00	0.00
ATOM	8007	OW	SOL	1975	14.920	45.620	2.160	1.00	0.00

ATOM	8008	HW1	SOL	1975	15.540	45.580	2.890	1.00	0.00
ATOM	8009	HW2	SOL	1975	14.630	44.710	2.050	1.00	0.00
ATOM	8010	OW	SOL	1976	30.860	2.590	55.010	1.00	0.00
ATOM	8011	HW1	SOL	1976	31.320	2.130	54.310	1.00	0.00
ATOM	8012	HW2	SOL	1976	29.950	2.330	54.900	1.00	0.00
ATOM	8013	OW	SOL	1977	22.400	5.200	41.950	1.00	0.00
ATOM	8014	HW1	SOL	1977	22.270	6.040	42.400	1.00	0.00
ATOM	8015	HW2	SOL	1977	21.630	5.100	41.390	1.00	0.00
ATOM	8016	OW	SOL	1978	16.750	42.990	55.620	1.00	0.00
ATOM	8017	HW1	SOL	1978	15.870	43.310	55.830	1.00	0.00
ATOM	8018	HW2	SOL	1978	16.930	42.330	56.280	1.00	0.00
ATOM	8019	OW	SOL	1979	32.700	12.340	35.430	1.00	0.00
ATOM	8020	HW1	SOL	1979	33.530	12.370	35.910	1.00	0.00
ATOM	8021	HW2	SOL	1979	32.490	11.410	35.350	1.00	0.00
ATOM	8022	OW	SOL	1980	26.720	15.370	6.560	1.00	0.00
ATOM	8023	HW1	SOL	1980	27.200	16.200	6.550	1.00	0.00
ATOM	8024	HW2	SOL	1980	26.350	15.310	7.440	1.00	0.00
ATOM	8025	OW	SOL	1981	36.300	48.280	48.780	1.00	0.00
ATOM	8026	HW1	SOL	1981	35.810	48.420	49.590	1.00	0.00
ATOM	8027	HW2	SOL	1981	36.830	47.500	48.950	1.00	0.00
ATOM	8028	OW	SOL	1982	16.370	22.580	8.580	1.00	0.00
ATOM	8029	HW1	SOL	1982	17.310	22.580	8.790	1.00	0.00
ATOM	8030	HW2	SOL	1982	16.010	21.840	9.060	1.00	0.00
ATOM	8031	OW	SOL	1983	24.390	16.740	3.450	1.00	0.00
ATOM	8032	HW1	SOL	1983	25.310	16.700	3.200	1.00	0.00
ATOM	8033	HW2	SOL	1983	24.050	17.520	3.020	1.00	0.00
ATOM	8034	OW	SOL	1984	6.990	35.370	46.570	1.00	0.00
ATOM	8035	HW1	SOL	1984	6.120	34.990	46.440	1.00	0.00
ATOM	8036	HW2	SOL	1984	7.520	35.010	45.870	1.00	0.00
ATOM	8037	OW	SOL	1985	22.210	24.060	25.390	1.00	0.00
ATOM	8038	HW1	SOL	1985	22.510	23.720	24.550	1.00	0.00
ATOM	8039	HW2	SOL	1985	23.010	24.380	25.820	1.00	0.00
ATOM	8040	OW	SOL	1986	44.430	9.240	26.940	1.00	0.00
ATOM	8041	HW1	SOL	1986	44.120	8.500	26.400	1.00	0.00
ATOM	8042	HW2	SOL	1986	45.300	9.440	26.590	1.00	0.00
ATOM	8043	OW	SOL	1987	31.560	12.820	41.140	1.00	0.00
ATOM	8044	HW1	SOL	1987	31.360	12.660	40.210	1.00	0.00
ATOM	8045	HW2	SOL	1987	31.060	12.150	41.610	1.00	0.00
ATOM	8046	OW	SOL	1988	7.790	51.730	15.890	1.00	0.00
ATOM	8047	HW1	SOL	1988	8.600	51.960	15.440	1.00	0.00
ATOM	8048	HW2	SOL	1988	7.720	52.380	16.600	1.00	0.00
ATOM	8049	OW	SOL	1989	23.800	54.240	36.190	1.00	0.00
ATOM	8050	HW1	SOL	1989	24.110	55.050	36.600	1.00	0.00
ATOM	8051	HW2	SOL	1989	22.860	54.350	36.110	1.00	0.00

ATOM	8052	OW	SOL	1990	16.250	12.920	35.910	1.00	0.00
ATOM	8053	HW1	SOL	1990	15.400	13.360	35.860	1.00	0.00
ATOM	8054	HW2	SOL	1990	16.480	12.960	36.840	1.00	0.00
ATOM	8055	OW	SOL	1991	41.790	3.620	47.370	1.00	0.00
ATOM	8056	HW1	SOL	1991	42.190	2.800	47.650	1.00	0.00
ATOM	8057	HW2	SOL	1991	42.520	4.150	47.050	1.00	0.00
ATOM	8058	OW	SOL	1992	28.370	31.030	45.900	1.00	0.00
ATOM	8059	HW1	SOL	1992	28.420	30.290	45.290	1.00	0.00
ATOM	8060	HW2	SOL	1992	27.790	31.650	45.450	1.00	0.00
ATOM	8061	OW	SOL	1993	49.970	29.110	50.990	1.00	0.00
ATOM	8062	HW1	SOL	1993	49.480	29.480	50.250	1.00	0.00
ATOM	8063	HW2	SOL	1993	50.620	28.540	50.580	1.00	0.00
ATOM	8064	OW	SOL	1994	53.930	46.330	6.860	1.00	0.00
ATOM	8065	HW1	SOL	1994	54.770	46.760	6.990	1.00	0.00
ATOM	8066	HW2	SOL	1994	53.590	46.190	7.750	1.00	0.00
ATOM	8067	OW	SOL	1995	40.770	2.450	50.130	1.00	0.00
ATOM	8068	HW1	SOL	1995	40.570	1.590	49.770	1.00	0.00
ATOM	8069	HW2	SOL	1995	41.000	2.280	51.040	1.00	0.00
ATOM	8070	OW	SOL	1996	42.160	54.220	46.070	1.00	0.00
ATOM	8071	HW1	SOL	1996	41.810	53.430	46.490	1.00	0.00
ATOM	8072	HW2	SOL	1996	43.070	54.270	46.350	1.00	0.00
ATOM	8073	OW	SOL	1997	29.960	24.100	50.690	1.00	0.00
ATOM	8074	HW1	SOL	1997	29.310	24.060	51.390	1.00	0.00
ATOM	8075	HW2	SOL	1997	29.950	23.220	50.300	1.00	0.00
ATOM	8076	OW	SOL	1998	34.630	50.700	14.240	1.00	0.00
ATOM	8077	HW1	SOL	1998	34.880	51.300	14.940	1.00	0.00
ATOM	8078	HW2	SOL	1998	34.550	51.250	13.460	1.00	0.00
ATOM	8079	OW	SOL	1999	53.490	0.080	43.860	1.00	0.00
ATOM	8080	HW1	SOL	1999	52.730	-0.420	43.580	1.00	0.00
ATOM	8081	HW2	SOL	1999	53.550	0.810	43.240	1.00	0.00
ATOM	8082	OW	SOL	2000	45.190	11.960	20.790	1.00	0.00
ATOM	8083	HW1	SOL	2000	45.410	12.630	20.140	1.00	0.00
ATOM	8084	HW2	SOL	2000	45.540	11.150	20.440	1.00	0.00
ATOM	8085	OW	SOL	2001	41.040	32.360	6.720	1.00	0.00
ATOM	8086	HW1	SOL	2001	40.310	31.810	6.980	1.00	0.00
ATOM	8087	HW2	SOL	2001	40.700	32.870	5.980	1.00	0.00
ATOM	8088	OW	SOL	2002	18.790	51.970	52.550	1.00	0.00
ATOM	8089	HW1	SOL	2002	19.650	52.190	52.190	1.00	0.00
ATOM	8090	HW2	SOL	2002	18.860	52.180	53.480	1.00	0.00
ATOM	8091	OW	SOL	2003	5.960	51.140	7.480	1.00	0.00
ATOM	8092	HW1	SOL	2003	5.510	50.940	8.300	1.00	0.00
ATOM	8093	HW2	SOL	2003	5.560	50.550	6.840	1.00	0.00
ATOM	8094	OW	SOL	2004	46.310	6.740	0.140	1.00	0.00
ATOM	8095	HW1	SOL	2004	45.560	7.220	0.500	1.00	0.00

ATOM	8096	HW2	SOL	2004	46.690	6.280	0.890	1.00	0.00
ATOM	8097	OW	SOL	2005	25.400	35.430	7.910	1.00	0.00
ATOM	8098	HW1	SOL	2005	25.780	36.010	7.250	1.00	0.00
ATOM	8099	HW2	SOL	2005	24.800	34.860	7.430	1.00	0.00
ATOM	8100	OW	SOL	2006	6.460	38.280	45.930	1.00	0.00
ATOM	8101	HW1	SOL	2006	6.920	38.010	46.720	1.00	0.00
ATOM	8102	HW2	SOL	2006	5.530	38.280	46.160	1.00	0.00
ATOM	8103	OW	SOL	2007	8.260	53.950	9.930	1.00	0.00
ATOM	8104	HW1	SOL	2007	8.630	53.140	10.280	1.00	0.00
ATOM	8105	HW2	SOL	2007	8.080	53.740	9.010	1.00	0.00
ATOM	8106	OW	SOL	2008	22.400	9.170	4.660	1.00	0.00
ATOM	8107	HW1	SOL	2008	23.050	9.820	4.400	1.00	0.00
ATOM	8108	HW2	SOL	2008	22.500	8.470	4.010	1.00	0.00
ATOM	8109	OW	SOL	2009	49.440	29.150	1.580	1.00	0.00
ATOM	8110	HW1	SOL	2009	49.560	30.100	1.700	1.00	0.00
ATOM	8111	HW2	SOL	2009	48.540	28.980	1.840	1.00	0.00
ATOM	8112	OW	SOL	2010	33.390	2.570	12.640	1.00	0.00
ATOM	8113	HW1	SOL	2010	32.770	2.190	13.260	1.00	0.00
ATOM	8114	HW2	SOL	2010	34.250	2.250	12.930	1.00	0.00
ATOM	8115	OW	SOL	2011	3.070	9.230	26.280	1.00	0.00
ATOM	8116	HW1	SOL	2011	2.950	10.120	25.970	1.00	0.00
ATOM	8117	HW2	SOL	2011	2.250	8.780	26.070	1.00	0.00
ATOM	8118	OW	SOL	2012	14.710	52.570	1.240	1.00	0.00
ATOM	8119	HW1	SOL	2012	14.850	53.410	0.820	1.00	0.00
ATOM	8120	HW2	SOL	2012	13.920	52.220	0.820	1.00	0.00
ATOM	8121	OW	SOL	2013	52.820	1.510	19.390	1.00	0.00
ATOM	8122	HW1	SOL	2013	52.600	0.710	18.910	1.00	0.00
ATOM	8123	HW2	SOL	2013	53.340	2.020	18.780	1.00	0.00
ATOM	8124	OW	SOL	2014	6.090	23.540	7.240	1.00	0.00
ATOM	8125	HW1	SOL	2014	5.230	23.860	7.500	1.00	0.00
ATOM	8126	HW2	SOL	2014	5.970	22.600	7.120	1.00	0.00
ATOM	8127	OW	SOL	2015	8.610	0.570	42.080	1.00	0.00
ATOM	8128	HW1	SOL	2015	7.830	1.090	42.240	1.00	0.00
ATOM	8129	HW2	SOL	2015	8.310	-0.160	41.540	1.00	0.00
ATOM	8130	OW	SOL	2016	25.920	15.830	44.840	1.00	0.00
ATOM	8131	HW1	SOL	2016	25.530	16.280	44.090	1.00	0.00
ATOM	8132	HW2	SOL	2016	26.380	15.080	44.450	1.00	0.00
ATOM	8133	OW	SOL	2017	18.960	5.970	26.730	1.00	0.00
ATOM	8134	HW1	SOL	2017	19.840	6.070	26.380	1.00	0.00
ATOM	8135	HW2	SOL	2017	18.380	6.200	26.000	1.00	0.00
ATOM	8136	OW	SOL	2018	17.150	35.110	48.920	1.00	0.00
ATOM	8137	HW1	SOL	2018	16.270	35.350	49.210	1.00	0.00
ATOM	8138	HW2	SOL	2018	17.110	34.160	48.790	1.00	0.00
ATOM	8139	OW	SOL	2019	55.090	46.200	28.700	1.00	0.00

ATOM	8140	HW1	SOL	2019	55.350	45.550	29.350	1.00	0.00
ATOM	8141	HW2	SOL	2019	54.280	45.850	28.320	1.00	0.00
ATOM	8142	OW	SOL	2020	34.470	52.970	16.120	1.00	0.00
ATOM	8143	HW1	SOL	2020	34.590	53.290	17.010	1.00	0.00
ATOM	8144	HW2	SOL	2020	34.540	53.750	15.570	1.00	0.00
ATOM	8145	OW	SOL	2021	0.710	20.360	53.600	1.00	0.00
ATOM	8146	HW1	SOL	2021	0.530	19.430	53.730	1.00	0.00
ATOM	8147	HW2	SOL	2021	0.650	20.490	52.650	1.00	0.00
ATOM	8148	OW	SOL	2022	37.900	14.970	35.940	1.00	0.00
ATOM	8149	HW1	SOL	2022	37.550	14.650	36.780	1.00	0.00
ATOM	8150	HW2	SOL	2022	37.250	15.610	35.650	1.00	0.00
ATOM	8151	OW	SOL	2023	35.170	45.440	49.680	1.00	0.00
ATOM	8152	HW1	SOL	2023	34.220	45.440	49.750	1.00	0.00
ATOM	8153	HW2	SOL	2023	35.480	45.250	50.570	1.00	0.00
ATOM	8154	OW	SOL	2024	11.550	36.550	40.540	1.00	0.00
ATOM	8155	HW1	SOL	2024	11.690	36.680	39.600	1.00	0.00
ATOM	8156	HW2	SOL	2024	10.620	36.310	40.610	1.00	0.00
ATOM	8157	OW	SOL	2025	50.050	7.250	10.470	1.00	0.00
ATOM	8158	HW1	SOL	2025	50.170	8.110	10.060	1.00	0.00
ATOM	8159	HW2	SOL	2025	49.620	7.440	11.300	1.00	0.00
ATOM	8160	OW	SOL	2026	45.260	42.720	51.880	1.00	0.00
ATOM	8161	HW1	SOL	2026	45.810	43.250	52.460	1.00	0.00
ATOM	8162	HW2	SOL	2026	45.730	42.720	51.040	1.00	0.00
ATOM	8163	OW	SOL	2027	54.610	42.990	40.620	1.00	0.00
ATOM	8164	HW1	SOL	2027	54.210	43.850	40.490	1.00	0.00
ATOM	8165	HW2	SOL	2027	54.430	42.790	41.540	1.00	0.00
ATOM	8166	OW	SOL	2028	12.840	38.190	47.290	1.00	0.00
ATOM	8167	HW1	SOL	2028	12.360	38.450	46.510	1.00	0.00
ATOM	8168	HW2	SOL	2028	12.420	37.380	47.580	1.00	0.00
ATOM	8169	OW	SOL	2029	27.930	35.970	45.000	1.00	0.00
ATOM	8170	HW1	SOL	2029	27.120	36.300	44.610	1.00	0.00
ATOM	8171	HW2	SOL	2029	27.630	35.490	45.770	1.00	0.00
ATOM	8172	OW	SOL	2030	7.060	34.990	50.770	1.00	0.00
ATOM	8173	HW1	SOL	2030	6.240	35.300	51.160	1.00	0.00
ATOM	8174	HW2	SOL	2030	7.730	35.210	51.410	1.00	0.00
ATOM	8175	OW	SOL	2031	52.830	33.340	0.640	1.00	0.00
ATOM	8176	HW1	SOL	2031	52.760	32.450	0.980	1.00	0.00
ATOM	8177	HW2	SOL	2031	52.060	33.450	0.090	1.00	0.00
ATOM	8178	OW	SOL	2032	10.240	51.090	44.480	1.00	0.00
ATOM	8179	HW1	SOL	2032	10.960	51.550	44.050	1.00	0.00
ATOM	8180	HW2	SOL	2032	10.130	51.550	45.310	1.00	0.00
ATOM	8181	OW	SOL	2033	31.300	22.420	33.720	1.00	0.00
ATOM	8182	HW1	SOL	2033	30.440	22.590	33.330	1.00	0.00
ATOM	8183	HW2	SOL	2033	31.630	21.650	33.270	1.00	0.00

ATOM	8184	OW	SOL	2034	18.040	20.090	44.990	1.00	0.00
ATOM	8185	HW1	SOL	2034	18.420	19.370	45.490	1.00	0.00
ATOM	8186	HW2	SOL	2034	17.950	19.740	44.100	1.00	0.00
ATOM	8187	OW	SOL	2035	7.460	20.010	50.210	1.00	0.00
ATOM	8188	HW1	SOL	2035	7.010	20.380	50.970	1.00	0.00
ATOM	8189	HW2	SOL	2035	6.960	20.340	49.460	1.00	0.00
ATOM	8190	OW	SOL	2036	40.160	51.990	4.860	1.00	0.00
ATOM	8191	HW1	SOL	2036	41.080	51.920	4.630	1.00	0.00
ATOM	8192	HW2	SOL	2036	40.080	51.500	5.680	1.00	0.00
ATOM	8193	OW	SOL	2037	19.210	38.860	51.000	1.00	0.00
ATOM	8194	HW1	SOL	2037	18.770	39.680	51.220	1.00	0.00
ATOM	8195	HW2	SOL	2037	18.710	38.510	50.260	1.00	0.00
ATOM	8196	OW	SOL	2038	52.700	10.810	15.550	1.00	0.00
ATOM	8197	HW1	SOL	2038	52.910	10.930	14.630	1.00	0.00
ATOM	8198	HW2	SOL	2038	52.630	9.870	15.660	1.00	0.00
ATOM	8199	OW	SOL	2039	9.730	50.540	19.280	1.00	0.00
ATOM	8200	HW1	SOL	2039	9.680	51.300	19.870	1.00	0.00
ATOM	8201	HW2	SOL	2039	10.520	50.700	18.760	1.00	0.00
ATOM	8202	OW	SOL	2040	18.820	1.550	25.180	1.00	0.00
ATOM	8203	HW1	SOL	2040	18.600	2.370	25.630	1.00	0.00
ATOM	8204	HW2	SOL	2040	19.490	1.800	24.540	1.00	0.00
ATOM	8205	OW	SOL	2041	13.360	20.350	42.490	1.00	0.00
ATOM	8206	HW1	SOL	2041	13.830	20.790	41.780	1.00	0.00
ATOM	8207	HW2	SOL	2041	13.580	20.850	43.270	1.00	0.00
ATOM	8208	OW	SOL	2042	14.240	53.250	8.210	1.00	0.00
ATOM	8209	HW1	SOL	2042	14.540	52.560	7.620	1.00	0.00
ATOM	8210	HW2	SOL	2042	13.280	53.230	8.140	1.00	0.00
ATOM	8211	OW	SOL	2043	52.560	23.070	32.430	1.00	0.00
ATOM	8212	HW1	SOL	2043	52.970	23.140	31.570	1.00	0.00
ATOM	8213	HW2	SOL	2043	51.710	23.490	32.320	1.00	0.00
ATOM	8214	OW	SOL	2044	43.870	53.490	50.490	1.00	0.00
ATOM	8215	HW1	SOL	2044	43.510	52.710	50.080	1.00	0.00
ATOM	8216	HW2	SOL	2044	43.750	53.350	51.430	1.00	0.00
ATOM	8217	OW	SOL	2045	18.430	17.840	48.110	1.00	0.00
ATOM	8218	HW1	SOL	2045	17.510	17.870	47.860	1.00	0.00
ATOM	8219	HW2	SOL	2045	18.620	18.700	48.470	1.00	0.00
ATOM	8220	OW	SOL	2046	2.180	52.510	39.700	1.00	0.00
ATOM	8221	HW1	SOL	2046	1.920	53.320	40.130	1.00	0.00
ATOM	8222	HW2	SOL	2046	1.850	51.820	40.280	1.00	0.00
ATOM	8223	OW	SOL	2047	51.740	3.500	40.660	1.00	0.00
ATOM	8224	HW1	SOL	2047	50.980	3.500	40.090	1.00	0.00
ATOM	8225	HW2	SOL	2047	51.870	4.420	40.890	1.00	0.00
ATOM	8226	OW	SOL	2048	0.150	35.640	48.720	1.00	0.00
ATOM	8227	HW1	SOL	2048	-0.440	35.160	48.140	1.00	0.00

ATOM	8228	HW2	SOL	2048	0.030	36.560	48.490	1.00	0.00
ATOM	8229	OW	SOL	2049	47.910	7.180	12.380	1.00	0.00
ATOM	8230	HW1	SOL	2049	47.550	7.390	13.240	1.00	0.00
ATOM	8231	HW2	SOL	2049	48.510	6.460	12.530	1.00	0.00
ATOM	8232	OW	SOL	2050	14.650	21.710	53.280	1.00	0.00
ATOM	8233	HW1	SOL	2050	14.520	22.630	53.520	1.00	0.00
ATOM	8234	HW2	SOL	2050	15.560	21.670	52.980	1.00	0.00
ATOM	8235	OW	SOL	2051	39.350	1.170	1.790	1.00	0.00
ATOM	8236	HW1	SOL	2051	40.130	0.610	1.790	1.00	0.00
ATOM	8237	HW2	SOL	2051	39.260	1.460	2.690	1.00	0.00
ATOM	8238	OW	SOL	2052	52.800	33.530	3.650	1.00	0.00
ATOM	8239	HW1	SOL	2052	53.710	33.450	3.390	1.00	0.00
ATOM	8240	HW2	SOL	2052	52.560	32.650	3.960	1.00	0.00
ATOM	8241	OW	SOL	2053	40.320	54.940	39.330	1.00	0.00
ATOM	8242	HW1	SOL	2053	39.610	54.340	39.540	1.00	0.00
ATOM	8243	HW2	SOL	2053	40.170	55.700	39.890	1.00	0.00
ATOM	8244	OW	SOL	2054	47.960	11.530	50.770	1.00	0.00
ATOM	8245	HW1	SOL	2054	47.640	12.140	50.110	1.00	0.00
ATOM	8246	HW2	SOL	2054	48.370	10.830	50.270	1.00	0.00
ATOM	8247	OW	SOL	2055	20.080	42.880	10.680	1.00	0.00
ATOM	8248	HW1	SOL	2055	19.920	42.070	11.160	1.00	0.00
ATOM	8249	HW2	SOL	2055	19.510	42.820	9.910	1.00	0.00
ATOM	8250	OW	SOL	2056	33.320	8.730	5.000	1.00	0.00
ATOM	8251	HW1	SOL	2056	33.130	9.530	4.520	1.00	0.00
ATOM	8252	HW2	SOL	2056	33.740	8.160	4.360	1.00	0.00
ATOM	8253	OW	SOL	2057	40.740	15.650	17.230	1.00	0.00
ATOM	8254	HW1	SOL	2057	39.850	15.980	17.100	1.00	0.00
ATOM	8255	HW2	SOL	2057	40.760	14.820	16.750	1.00	0.00
ATOM	8256	OW	SOL	2058	32.800	7.050	13.740	1.00	0.00
ATOM	8257	HW1	SOL	2058	33.470	6.950	13.060	1.00	0.00
ATOM	8258	HW2	SOL	2058	32.060	7.460	13.290	1.00	0.00
ATOM	8259	OW	SOL	2059	6.080	20.030	35.200	1.00	0.00
ATOM	8260	HW1	SOL	2059	6.980	19.690	35.250	1.00	0.00
ATOM	8261	HW2	SOL	2059	5.840	19.930	34.280	1.00	0.00
ATOM	8262	OW	SOL	2060	49.290	24.580	23.160	1.00	0.00
ATOM	8263	HW1	SOL	2060	48.790	25.330	23.490	1.00	0.00
ATOM	8264	HW2	SOL	2060	50.200	24.820	23.320	1.00	0.00
ATOM	8265	OW	SOL	2061	21.530	44.920	2.850	1.00	0.00
ATOM	8266	HW1	SOL	2061	21.340	44.120	3.340	1.00	0.00
ATOM	8267	HW2	SOL	2061	22.060	44.630	2.110	1.00	0.00
ATOM	8268	OW	SOL	2062	3.370	27.650	0.110	1.00	0.00
ATOM	8269	HW1	SOL	2062	2.850	28.090	0.780	1.00	0.00
ATOM	8270	HW2	SOL	2062	4.020	27.140	0.610	1.00	0.00
ATOM	8271	OW	SOL	2063	15.800	17.230	13.940	1.00	0.00

ATOM	8272	HW1	SOL	2063	15.890	16.460	13.380	1.00	0.00
ATOM	8273	HW2	SOL	2063	15.300	16.930	14.700	1.00	0.00
ATOM	8274	OW	SOL	2064	4.630	17.760	5.820	1.00	0.00
ATOM	8275	HW1	SOL	2064	3.700	17.520	5.740	1.00	0.00
ATOM	8276	HW2	SOL	2064	5.070	17.210	5.170	1.00	0.00
ATOM	8277	OW	SOL	2065	25.690	17.280	42.500	1.00	0.00
ATOM	8278	HW1	SOL	2065	25.050	17.240	41.790	1.00	0.00
ATOM	8279	HW2	SOL	2065	26.520	17.500	42.060	1.00	0.00
ATOM	8280	OW	SOL	2066	1.680	48.830	51.810	1.00	0.00
ATOM	8281	HW1	SOL	2066	1.760	49.640	51.310	1.00	0.00
ATOM	8282	HW2	SOL	2066	1.410	48.180	51.170	1.00	0.00
ATOM	8283	OW	SOL	2067	9.150	53.240	24.910	1.00	0.00
ATOM	8284	HW1	SOL	2067	8.490	52.770	25.420	1.00	0.00
ATOM	8285	HW2	SOL	2067	9.760	52.560	24.630	1.00	0.00
ATOM	8286	OW	SOL	2068	47.640	51.400	41.790	1.00	0.00
ATOM	8287	HW1	SOL	2068	47.730	50.730	41.100	1.00	0.00
ATOM	8288	HW2	SOL	2068	47.470	52.210	41.310	1.00	0.00
ATOM	8289	OW	SOL	2069	52.110	38.500	31.630	1.00	0.00
ATOM	8290	HW1	SOL	2069	51.520	38.500	30.880	1.00	0.00
ATOM	8291	HW2	SOL	2069	51.700	37.900	32.250	1.00	0.00
ATOM	8292	OW	SOL	2070	28.470	8.210	30.630	1.00	0.00
ATOM	8293	HW1	SOL	2070	28.020	8.470	31.440	1.00	0.00
ATOM	8294	HW2	SOL	2070	28.770	9.040	30.250	1.00	0.00
ATOM	8295	OW	SOL	2071	30.930	1.550	2.390	1.00	0.00
ATOM	8296	HW1	SOL	2071	31.280	0.670	2.530	1.00	0.00
ATOM	8297	HW2	SOL	2071	31.460	1.910	1.670	1.00	0.00
ATOM	8298	OW	SOL	2072	27.720	39.370	46.670	1.00	0.00
ATOM	8299	HW1	SOL	2072	27.620	40.320	46.550	1.00	0.00
ATOM	8300	HW2	SOL	2072	26.890	39.090	47.030	1.00	0.00
ATOM	8301	OW	SOL	2073	5.910	4.030	20.220	1.00	0.00
ATOM	8302	HW1	SOL	2073	5.250	3.430	20.570	1.00	0.00
ATOM	8303	HW2	SOL	2073	6.010	3.770	19.300	1.00	0.00
ATOM	8304	OW	SOL	2074	53.520	49.800	10.350	1.00	0.00
ATOM	8305	HW1	SOL	2074	53.120	49.360	9.600	1.00	0.00
ATOM	8306	HW2	SOL	2074	54.450	49.560	10.300	1.00	0.00
ATOM	8307	OW	SOL	2075	10.470	53.400	42.680	1.00	0.00
ATOM	8308	HW1	SOL	2075	11.010	54.190	42.580	1.00	0.00
ATOM	8309	HW2	SOL	2075	10.830	52.780	42.050	1.00	0.00
ATOM	8310	OW	SOL	2076	27.250	28.920	13.920	1.00	0.00
ATOM	8311	HW1	SOL	2076	27.320	27.970	13.970	1.00	0.00
ATOM	8312	HW2	SOL	2076	26.350	29.090	13.640	1.00	0.00
ATOM	8313	OW	SOL	2077	39.420	54.570	7.960	1.00	0.00
ATOM	8314	HW1	SOL	2077	40.360	54.560	7.770	1.00	0.00
ATOM	8315	HW2	SOL	2077	39.020	54.950	7.180	1.00	0.00

ATOM	8316	OW	SOL	2078	49.480	49.080	7.920	1.00	0.00
ATOM	8317	HW1	SOL	2078	49.350	48.230	8.340	1.00	0.00
ATOM	8318	HW2	SOL	2078	49.880	48.870	7.070	1.00	0.00
ATOM	8319	OW	SOL	2079	17.210	13.560	24.740	1.00	0.00
ATOM	8320	HW1	SOL	2079	16.850	12.710	24.970	1.00	0.00
ATOM	8321	HW2	SOL	2079	17.730	13.820	25.500	1.00	0.00
ATOM	8322	OW	SOL	2080	16.370	33.450	6.490	1.00	0.00
ATOM	8323	HW1	SOL	2080	15.810	33.640	7.240	1.00	0.00
ATOM	8324	HW2	SOL	2080	16.770	34.300	6.270	1.00	0.00
ATOM	8325	OW	SOL	2081	8.790	46.020	6.330	1.00	0.00
ATOM	8326	HW1	SOL	2081	8.930	45.650	7.200	1.00	0.00
ATOM	8327	HW2	SOL	2081	9.520	46.630	6.210	1.00	0.00
ATOM	8328	OW	SOL	2082	35.480	18.610	37.790	1.00	0.00
ATOM	8329	HW1	SOL	2082	36.110	18.810	37.100	1.00	0.00
ATOM	8330	HW2	SOL	2082	34.640	18.560	37.340	1.00	0.00
ATOM	8331	OW	SOL	2083	38.220	33.700	45.150	1.00	0.00
ATOM	8332	HW1	SOL	2083	38.640	34.480	45.500	1.00	0.00
ATOM	8333	HW2	SOL	2083	37.300	33.960	45.010	1.00	0.00
ATOM	8334	OW	SOL	2084	30.300	18.400	50.480	1.00	0.00
ATOM	8335	HW1	SOL	2084	30.670	18.320	51.360	1.00	0.00
ATOM	8336	HW2	SOL	2084	30.730	17.720	49.970	1.00	0.00
ATOM	8337	OW	SOL	2085	21.340	21.630	6.630	1.00	0.00
ATOM	8338	HW1	SOL	2085	22.220	21.960	6.480	1.00	0.00
ATOM	8339	HW2	SOL	2085	21.270	20.860	6.070	1.00	0.00
ATOM	8340	OW	SOL	2086	7.910	36.980	48.320	1.00	0.00
ATOM	8341	HW1	SOL	2086	7.840	36.300	47.640	1.00	0.00
ATOM	8342	HW2	SOL	2086	7.780	36.520	49.140	1.00	0.00
ATOM	8343	OW	SOL	2087	40.310	50.280	16.420	1.00	0.00
ATOM	8344	HW1	SOL	2087	40.390	50.200	17.370	1.00	0.00
ATOM	8345	HW2	SOL	2087	41.210	50.240	16.100	1.00	0.00
ATOM	8346	OW	SOL	2088	21.420	0.500	41.310	1.00	0.00
ATOM	8347	HW1	SOL	2088	22.180	0.560	41.900	1.00	0.00
ATOM	8348	HW2	SOL	2088	21.650	-0.200	40.700	1.00	0.00
ATOM	8349	OW	SOL	2089	32.050	37.780	54.520	1.00	0.00
ATOM	8350	HW1	SOL	2089	31.310	37.200	54.730	1.00	0.00
ATOM	8351	HW2	SOL	2089	32.130	38.350	55.280	1.00	0.00
ATOM	8352	OW	SOL	2090	11.040	49.620	5.740	1.00	0.00
ATOM	8353	HW1	SOL	2090	11.940	49.620	5.430	1.00	0.00
ATOM	8354	HW2	SOL	2090	10.910	48.740	6.100	1.00	0.00
ATOM	8355	OW	SOL	2091	5.060	32.840	36.530	1.00	0.00
ATOM	8356	HW1	SOL	2091	4.740	32.640	37.410	1.00	0.00
ATOM	8357	HW2	SOL	2091	4.560	33.610	36.270	1.00	0.00
ATOM	8358	OW	SOL	2092	48.560	34.470	14.340	1.00	0.00
ATOM	8359	HW1	SOL	2092	48.380	34.340	13.410	1.00	0.00

ATOM	8360	HW2	SOL	2092	47.850	35.040	14.650	1.00	0.00
ATOM	8361	OW	SOL	2093	10.240	22.380	17.850	1.00	0.00
ATOM	8362	HW1	SOL	2093	10.990	22.300	18.440	1.00	0.00
ATOM	8363	HW2	SOL	2093	10.550	22.020	17.020	1.00	0.00
ATOM	8364	OW	SOL	2094	8.270	16.070	15.460	1.00	0.00
ATOM	8365	HW1	SOL	2094	8.330	16.500	16.310	1.00	0.00
ATOM	8366	HW2	SOL	2094	8.850	16.580	14.890	1.00	0.00
ATOM	8367	OW	SOL	2095	47.370	35.950	11.750	1.00	0.00
ATOM	8368	HW1	SOL	2095	47.660	35.330	11.070	1.00	0.00
ATOM	8369	HW2	SOL	2095	48.160	36.450	11.960	1.00	0.00
ATOM	8370	OW	SOL	2096	10.380	51.920	22.670	1.00	0.00
ATOM	8371	HW1	SOL	2096	9.800	52.100	21.940	1.00	0.00
ATOM	8372	HW2	SOL	2096	10.830	52.760	22.830	1.00	0.00
ATOM	8373	OW	SOL	2097	45.160	32.260	32.500	1.00	0.00
ATOM	8374	HW1	SOL	2097	46.050	32.400	32.180	1.00	0.00
ATOM	8375	HW2	SOL	2097	45.270	31.820	33.340	1.00	0.00
ATOM	8376	OW	SOL	2098	8.400	9.410	18.780	1.00	0.00
ATOM	8377	HW1	SOL	2098	8.240	9.160	19.690	1.00	0.00
ATOM	8378	HW2	SOL	2098	9.150	8.880	18.510	1.00	0.00
ATOM	8379	OW	SOL	2099	17.180	8.310	40.380	1.00	0.00
ATOM	8380	HW1	SOL	2099	18.030	8.760	40.370	1.00	0.00
ATOM	8381	HW2	SOL	2099	16.870	8.410	41.280	1.00	0.00
ATOM	8382	OW	SOL	2100	10.010	11.650	21.930	1.00	0.00
ATOM	8383	HW1	SOL	2100	9.620	12.040	21.150	1.00	0.00
ATOM	8384	HW2	SOL	2100	10.450	10.860	21.610	1.00	0.00
ATOM	8385	OW	SOL	2101	16.630	13.830	20.420	1.00	0.00
ATOM	8386	HW1	SOL	2101	16.620	14.710	20.040	1.00	0.00
ATOM	8387	HW2	SOL	2101	16.240	13.950	21.290	1.00	0.00
ATOM	8388	OW	SOL	2102	11.190	15.260	49.730	1.00	0.00
ATOM	8389	HW1	SOL	2102	10.930	16.130	49.410	1.00	0.00
ATOM	8390	HW2	SOL	2102	12.090	15.390	50.040	1.00	0.00
ATOM	8391	OW	SOL	2103	27.360	53.620	9.750	1.00	0.00
ATOM	8392	HW1	SOL	2103	27.150	52.700	9.590	1.00	0.00
ATOM	8393	HW2	SOL	2103	27.350	54.020	8.880	1.00	0.00
ATOM	8394	OW	SOL	2104	27.290	42.210	46.620	1.00	0.00
ATOM	8395	HW1	SOL	2104	28.210	42.410	46.430	1.00	0.00
ATOM	8396	HW2	SOL	2104	26.810	42.470	45.830	1.00	0.00
ATOM	8397	OW	SOL	2105	52.060	1.810	12.020	1.00	0.00
ATOM	8398	HW1	SOL	2105	51.300	1.850	12.610	1.00	0.00
ATOM	8399	HW2	SOL	2105	51.710	2.020	11.160	1.00	0.00
ATOM	8400	OW	SOL	2106	18.520	33.300	27.920	1.00	0.00
ATOM	8401	HW1	SOL	2106	17.570	33.180	27.920	1.00	0.00
ATOM	8402	HW2	SOL	2106	18.680	33.880	27.170	1.00	0.00
ATOM	8403	OW	SOL	2107	51.350	47.020	30.600	1.00	0.00

ATOM	8404	HW1	SOL	2107	52.170	46.570	30.400	1.00	0.00
ATOM	8405	HW2	SOL	2107	51.630	47.870	30.960	1.00	0.00
ATOM	8406	OW	SOL	2108	47.960	37.890	53.620	1.00	0.00
ATOM	8407	HW1	SOL	2108	48.000	37.470	52.760	1.00	0.00
ATOM	8408	HW2	SOL	2108	47.660	38.780	53.440	1.00	0.00
ATOM	8409	OW	SOL	2109	44.500	2.710	8.980	1.00	0.00
ATOM	8410	HW1	SOL	2109	44.690	3.420	8.360	1.00	0.00
ATOM	8411	HW2	SOL	2109	43.600	2.880	9.260	1.00	0.00
ATOM	8412	OW	SOL	2110	20.550	39.490	6.700	1.00	0.00
ATOM	8413	HW1	SOL	2110	21.320	39.840	6.270	1.00	0.00
ATOM	8414	HW2	SOL	2110	19.880	39.440	6.020	1.00	0.00
ATOM	8415	OW	SOL	2111	23.670	31.340	7.970	1.00	0.00
ATOM	8416	HW1	SOL	2111	23.800	30.450	8.300	1.00	0.00
ATOM	8417	HW2	SOL	2111	22.740	31.510	8.100	1.00	0.00
ATOM	8418	OW	SOL	2112	39.040	38.720	38.860	1.00	0.00
ATOM	8419	HW1	SOL	2112	38.360	38.150	38.490	1.00	0.00
ATOM	8420	HW2	SOL	2112	39.820	38.170	38.900	1.00	0.00
ATOM	8421	OW	SOL	2113	40.720	11.440	5.410	1.00	0.00
ATOM	8422	HW1	SOL	2113	41.190	12.140	4.960	1.00	0.00
ATOM	8423	HW2	SOL	2113	41.090	10.630	5.040	1.00	0.00
ATOM	8424	OW	SOL	2114	5.400	40.450	29.800	1.00	0.00
ATOM	8425	HW1	SOL	2114	5.700	41.070	29.140	1.00	0.00
ATOM	8426	HW2	SOL	2114	4.820	39.860	29.330	1.00	0.00
ATOM	8427	OW	SOL	2115	5.250	32.520	15.860	1.00	0.00
ATOM	8428	HW1	SOL	2115	4.600	32.790	15.210	1.00	0.00
ATOM	8429	HW2	SOL	2115	4.780	31.920	16.430	1.00	0.00
ATOM	8430	OW	SOL	2116	2.970	29.290	49.740	1.00	0.00
ATOM	8431	HW1	SOL	2116	2.770	30.150	49.360	1.00	0.00
ATOM	8432	HW2	SOL	2116	3.920	29.260	49.800	1.00	0.00
ATOM	8433	OW	SOL	2117	48.420	31.800	44.510	1.00	0.00
ATOM	8434	HW1	SOL	2117	48.670	32.530	43.940	1.00	0.00
ATOM	8435	HW2	SOL	2117	48.400	31.040	43.930	1.00	0.00
ATOM	8436	OW	SOL	2118	28.190	17.490	7.270	1.00	0.00
ATOM	8437	HW1	SOL	2118	28.600	18.300	7.560	1.00	0.00
ATOM	8438	HW2	SOL	2118	27.310	17.750	6.980	1.00	0.00
ATOM	8439	OW	SOL	2119	44.940	40.170	25.240	1.00	0.00
ATOM	8440	HW1	SOL	2119	44.560	39.450	25.750	1.00	0.00
ATOM	8441	HW2	SOL	2119	44.200	40.520	24.740	1.00	0.00
ATOM	8442	OW	SOL	2120	13.560	27.220	52.800	1.00	0.00
ATOM	8443	HW1	SOL	2120	12.670	27.380	53.100	1.00	0.00
ATOM	8444	HW2	SOL	2120	14.110	27.630	53.470	1.00	0.00
ATOM	8445	OW	SOL	2121	13.900	10.300	3.810	1.00	0.00
ATOM	8446	HW1	SOL	2121	13.960	11.190	3.460	1.00	0.00
ATOM	8447	HW2	SOL	2121	14.770	10.120	4.160	1.00	0.00

ATOM	8448	OW	SOL	2122	1.710	12.360	27.610	1.00	0.00
ATOM	8449	HW1	SOL	2122	0.770	12.270	27.500	1.00	0.00
ATOM	8450	HW2	SOL	2122	1.810	12.930	28.370	1.00	0.00
ATOM	8451	OW	SOL	2123	2.610	1.260	37.410	1.00	0.00
ATOM	8452	HW1	SOL	2123	2.500	1.800	38.200	1.00	0.00
ATOM	8453	HW2	SOL	2123	1.820	1.440	36.890	1.00	0.00
ATOM	8454	OW	SOL	2124	22.750	1.960	11.630	1.00	0.00
ATOM	8455	HW1	SOL	2124	22.530	1.300	10.970	1.00	0.00
ATOM	8456	HW2	SOL	2124	22.840	1.460	12.440	1.00	0.00
ATOM	8457	OW	SOL	2125	17.660	10.560	0.180	1.00	0.00
ATOM	8458	HW1	SOL	2125	17.320	10.210	-0.650	1.00	0.00
ATOM	8459	HW2	SOL	2125	17.050	10.220	0.840	1.00	0.00
ATOM	8460	OW	SOL	2126	35.990	53.180	10.040	1.00	0.00
ATOM	8461	HW1	SOL	2126	35.600	52.840	9.230	1.00	0.00
ATOM	8462	HW2	SOL	2126	36.900	52.880	10.010	1.00	0.00
ATOM	8463	OW	SOL	2127	20.030	27.280	47.310	1.00	0.00
ATOM	8464	HW1	SOL	2127	20.540	26.670	47.840	1.00	0.00
ATOM	8465	HW2	SOL	2127	19.140	26.930	47.340	1.00	0.00
ATOM	8466	OW	SOL	2128	39.800	26.740	51.220	1.00	0.00
ATOM	8467	HW1	SOL	2128	38.980	27.180	50.980	1.00	0.00
ATOM	8468	HW2	SOL	2128	40.470	27.210	50.720	1.00	0.00
ATOM	8469	OW	SOL	2129	8.870	32.480	47.570	1.00	0.00
ATOM	8470	HW1	SOL	2129	9.620	31.950	47.840	1.00	0.00
ATOM	8471	HW2	SOL	2129	8.970	33.310	48.030	1.00	0.00
ATOM	8472	OW	SOL	2130	11.340	47.220	6.950	1.00	0.00
ATOM	8473	HW1	SOL	2130	11.930	46.620	6.490	1.00	0.00
ATOM	8474	HW2	SOL	2130	11.710	47.280	7.830	1.00	0.00
ATOM	8475	OW	SOL	2131	23.320	25.770	3.740	1.00	0.00
ATOM	8476	HW1	SOL	2131	22.520	26.170	3.400	1.00	0.00
ATOM	8477	HW2	SOL	2131	23.090	24.850	3.870	1.00	0.00
ATOM	8478	OW	SOL	2132	4.960	22.990	47.020	1.00	0.00
ATOM	8479	HW1	SOL	2132	5.170	23.050	46.090	1.00	0.00
ATOM	8480	HW2	SOL	2132	4.000	23.050	47.050	1.00	0.00
ATOM	8481	OW	SOL	2133	48.670	10.810	0.120	1.00	0.00
ATOM	8482	HW1	SOL	2133	47.970	11.150	-0.440	1.00	0.00
ATOM	8483	HW2	SOL	2133	49.400	11.410	-0.030	1.00	0.00
ATOM	8484	OW	SOL	2134	27.140	28.360	48.410	1.00	0.00
ATOM	8485	HW1	SOL	2134	27.500	28.470	47.530	1.00	0.00
ATOM	8486	HW2	SOL	2134	26.300	27.910	48.270	1.00	0.00
ATOM	8487	OW	SOL	2135	12.910	47.740	53.900	1.00	0.00
ATOM	8488	HW1	SOL	2135	12.870	46.960	54.460	1.00	0.00
ATOM	8489	HW2	SOL	2135	12.200	47.630	53.280	1.00	0.00
ATOM	8490	OW	SOL	2136	7.980	48.070	17.440	1.00	0.00
ATOM	8491	HW1	SOL	2136	7.150	48.500	17.620	1.00	0.00

ATOM	8492	HW2	SOL	2136	8.610	48.500	18.010	1.00	0.00
ATOM	8493	OW	SOL	2137	4.840	47.980	16.840	1.00	0.00
ATOM	8494	HW1	SOL	2137	4.020	48.320	16.470	1.00	0.00
ATOM	8495	HW2	SOL	2137	5.330	47.660	16.080	1.00	0.00
ATOM	8496	OW	SOL	2138	26.750	8.030	13.710	1.00	0.00
ATOM	8497	HW1	SOL	2138	27.690	7.860	13.640	1.00	0.00
ATOM	8498	HW2	SOL	2138	26.480	7.590	14.510	1.00	0.00
ATOM	8499	OW	SOL	2139	49.120	16.030	17.540	1.00	0.00
ATOM	8500	HW1	SOL	2139	48.310	16.010	17.030	1.00	0.00
ATOM	8501	HW2	SOL	2139	49.700	16.600	17.040	1.00	0.00
ATOM	8502	OW	SOL	2140	52.010	34.360	27.320	1.00	0.00
ATOM	8503	HW1	SOL	2140	51.770	34.030	28.180	1.00	0.00
ATOM	8504	HW2	SOL	2140	51.190	34.340	26.820	1.00	0.00
ATOM	8505	OW	SOL	2141	8.620	13.560	53.550	1.00	0.00
ATOM	8506	HW1	SOL	2141	9.000	13.700	54.420	1.00	0.00
ATOM	8507	HW2	SOL	2141	7.890	14.170	53.510	1.00	0.00
ATOM	8508	OW	SOL	2142	31.060	21.050	53.900	1.00	0.00
ATOM	8509	HW1	SOL	2142	31.230	21.630	53.160	1.00	0.00
ATOM	8510	HW2	SOL	2142	30.110	21.010	53.960	1.00	0.00
ATOM	8511	OW	SOL	2143	52.660	14.750	50.630	1.00	0.00
ATOM	8512	HW1	SOL	2143	52.870	14.010	51.190	1.00	0.00
ATOM	8513	HW2	SOL	2143	51.760	14.970	50.850	1.00	0.00
ATOM	8514	OW	SOL	2144	48.400	34.520	7.710	1.00	0.00
ATOM	8515	HW1	SOL	2144	47.900	34.160	8.450	1.00	0.00
ATOM	8516	HW2	SOL	2144	47.870	34.310	6.940	1.00	0.00
ATOM	8517	OW	SOL	2145	0.940	47.830	41.240	1.00	0.00
ATOM	8518	HW1	SOL	2145	1.060	47.010	40.760	1.00	0.00
ATOM	8519	HW2	SOL	2145	0.520	47.570	42.060	1.00	0.00
ATOM	8520	OW	SOL	2146	37.470	19.810	31.340	1.00	0.00
ATOM	8521	HW1	SOL	2146	36.760	20.360	31.670	1.00	0.00
ATOM	8522	HW2	SOL	2146	37.120	19.420	30.540	1.00	0.00
ATOM	8523	OW	SOL	2147	30.190	8.620	33.710	1.00	0.00
ATOM	8524	HW1	SOL	2147	29.980	7.750	34.040	1.00	0.00
ATOM	8525	HW2	SOL	2147	29.370	9.110	33.780	1.00	0.00
ATOM	8526	OW	SOL	2148	5.420	6.220	33.000	1.00	0.00
ATOM	8527	HW1	SOL	2148	5.010	5.560	33.560	1.00	0.00
ATOM	8528	HW2	SOL	2148	5.320	5.870	32.110	1.00	0.00
ATOM	8529	OW	SOL	2149	53.380	27.680	40.630	1.00	0.00
ATOM	8530	HW1	SOL	2149	53.620	26.760	40.550	1.00	0.00
ATOM	8531	HW2	SOL	2149	52.870	27.870	39.850	1.00	0.00
ATOM	8532	OW	SOL	2150	36.260	43.250	6.350	1.00	0.00
ATOM	8533	HW1	SOL	2150	36.230	43.340	5.400	1.00	0.00
ATOM	8534	HW2	SOL	2150	37.060	43.700	6.610	1.00	0.00
ATOM	8535	OW	SOL	2151	40.180	17.030	2.440	1.00	0.00

ATOM	8536	HW1	SOL	2151	39.350	17.480	2.310	1.00	0.00
ATOM	8537	HW2	SOL	2151	40.850	17.680	2.250	1.00	0.00
ATOM	8538	OW	SOL	2152	11.930	28.660	0.590	1.00	0.00
ATOM	8539	HW1	SOL	2152	11.240	28.100	0.950	1.00	0.00
ATOM	8540	HW2	SOL	2152	12.730	28.370	1.030	1.00	0.00
ATOM	8541	OW	SOL	2153	11.660	51.330	41.110	1.00	0.00
ATOM	8542	HW1	SOL	2153	11.380	50.410	41.150	1.00	0.00
ATOM	8543	HW2	SOL	2153	12.220	51.440	41.870	1.00	0.00
ATOM	8544	OW	SOL	2154	48.720	0.610	24.400	1.00	0.00
ATOM	8545	HW1	SOL	2154	47.840	0.400	24.700	1.00	0.00
ATOM	8546	HW2	SOL	2154	49.000	1.350	24.940	1.00	0.00
ATOM	8547	OW	SOL	2155	11.280	39.390	45.390	1.00	0.00
ATOM	8548	HW1	SOL	2155	11.550	38.910	44.610	1.00	0.00
ATOM	8549	HW2	SOL	2155	10.380	39.660	45.210	1.00	0.00
ATOM	8550	OW	SOL	2156	11.290	23.100	33.720	1.00	0.00
ATOM	8551	HW1	SOL	2156	10.650	22.420	33.540	1.00	0.00
ATOM	8552	HW2	SOL	2156	10.990	23.500	34.540	1.00	0.00
ATOM	8553	OW	SOL	2157	2.240	37.260	5.490	1.00	0.00
ATOM	8554	HW1	SOL	2157	1.690	38.030	5.640	1.00	0.00
ATOM	8555	HW2	SOL	2157	2.390	36.910	6.370	1.00	0.00
ATOM	8556	OW	SOL	2158	34.740	10.200	27.230	1.00	0.00
ATOM	8557	HW1	SOL	2158	34.450	9.490	27.810	1.00	0.00
ATOM	8558	HW2	SOL	2158	35.450	10.630	27.720	1.00	0.00
ATOM	8559	OW	SOL	2159	55.030	43.760	36.420	1.00	0.00
ATOM	8560	HW1	SOL	2159	54.290	43.770	37.030	1.00	0.00
ATOM	8561	HW2	SOL	2159	55.760	43.400	36.920	1.00	0.00
ATOM	8562	OW	SOL	2160	34.760	28.460	16.850	1.00	0.00
ATOM	8563	HW1	SOL	2160	35.540	27.950	17.030	1.00	0.00
ATOM	8564	HW2	SOL	2160	34.360	28.600	17.710	1.00	0.00
ATOM	8565	OW	SOL	2161	8.020	7.840	8.020	1.00	0.00
ATOM	8566	HW1	SOL	2161	8.720	7.210	8.180	1.00	0.00
ATOM	8567	HW2	SOL	2161	8.430	8.550	7.530	1.00	0.00
ATOM	8568	OW	SOL	2162	9.920	5.820	54.770	1.00	0.00
ATOM	8569	HW1	SOL	2162	9.070	6.000	55.170	1.00	0.00
ATOM	8570	HW2	SOL	2162	9.730	5.150	54.110	1.00	0.00
ATOM	8571	OW	SOL	2163	3.040	30.230	25.540	1.00	0.00
ATOM	8572	HW1	SOL	2163	3.140	30.130	24.600	1.00	0.00
ATOM	8573	HW2	SOL	2163	2.160	30.580	25.660	1.00	0.00
ATOM	8574	OW	SOL	2164	40.240	8.900	9.030	1.00	0.00
ATOM	8575	HW1	SOL	2164	39.430	9.400	8.930	1.00	0.00
ATOM	8576	HW2	SOL	2164	40.880	9.530	9.350	1.00	0.00
ATOM	8577	OW	SOL	2165	30.210	1.200	20.030	1.00	0.00
ATOM	8578	HW1	SOL	2165	29.460	1.210	20.620	1.00	0.00
ATOM	8579	HW2	SOL	2165	30.010	0.520	19.390	1.00	0.00

ATOM	8580	OW	SOL	2166	7.560	40.140	41.960	1.00	0.00
ATOM	8581	HW1	SOL	2166	6.700	40.310	41.590	1.00	0.00
ATOM	8582	HW2	SOL	2166	8.100	40.870	41.660	1.00	0.00
ATOM	8583	OW	SOL	2167	22.030	54.770	0.500	1.00	0.00
ATOM	8584	HW1	SOL	2167	22.360	54.550	-0.370	1.00	0.00
ATOM	8585	HW2	SOL	2167	22.820	54.940	1.020	1.00	0.00
ATOM	8586	OW	SOL	2168	12.680	50.980	29.910	1.00	0.00
ATOM	8587	HW1	SOL	2168	12.540	51.070	28.970	1.00	0.00
ATOM	8588	HW2	SOL	2168	11.830	51.200	30.300	1.00	0.00
ATOM	8589	OW	SOL	2169	24.750	54.900	2.970	1.00	0.00
ATOM	8590	HW1	SOL	2169	24.720	54.000	3.300	1.00	0.00
ATOM	8591	HW2	SOL	2169	25.680	55.090	2.890	1.00	0.00
ATOM	8592	OW	SOL	2170	10.060	0.220	7.900	1.00	0.00
ATOM	8593	HW1	SOL	2170	10.070	0.560	8.800	1.00	0.00
ATOM	8594	HW2	SOL	2170	10.410	-0.670	7.980	1.00	0.00
ATOM	8595	OW	SOL	2171	51.880	5.190	10.620	1.00	0.00
ATOM	8596	HW1	SOL	2171	51.060	5.630	10.410	1.00	0.00
ATOM	8597	HW2	SOL	2171	52.140	5.550	11.460	1.00	0.00
ATOM	8598	OW	SOL	2172	54.870	46.810	37.540	1.00	0.00
ATOM	8599	HW1	SOL	2172	54.780	46.010	38.060	1.00	0.00
ATOM	8600	HW2	SOL	2172	54.600	47.510	38.140	1.00	0.00
ATOM	8601	OW	SOL	2173	33.050	55.090	38.850	1.00	0.00
ATOM	8602	HW1	SOL	2173	33.250	54.360	38.270	1.00	0.00
ATOM	8603	HW2	SOL	2173	33.410	54.830	39.690	1.00	0.00
ATOM	8604	OW	SOL	2174	39.160	50.560	7.390	1.00	0.00
ATOM	8605	HW1	SOL	2174	39.270	50.940	8.260	1.00	0.00
ATOM	8606	HW2	SOL	2174	38.250	50.260	7.370	1.00	0.00
ATOM	8607	OW	SOL	2175	18.880	0.250	51.100	1.00	0.00
ATOM	8608	HW1	SOL	2175	18.070	0.690	50.850	1.00	0.00
ATOM	8609	HW2	SOL	2175	19.360	0.150	50.280	1.00	0.00
ATOM	8610	OW	SOL	2176	18.110	4.020	21.530	1.00	0.00
ATOM	8611	HW1	SOL	2176	17.430	4.380	20.980	1.00	0.00
ATOM	8612	HW2	SOL	2176	18.030	3.070	21.420	1.00	0.00
ATOM	8613	OW	SOL	2177	52.690	18.910	51.890	1.00	0.00
ATOM	8614	HW1	SOL	2177	52.880	19.800	52.180	1.00	0.00
ATOM	8615	HW2	SOL	2177	52.810	18.370	52.680	1.00	0.00
ATOM	8616	OW	SOL	2178	28.600	4.370	6.910	1.00	0.00
ATOM	8617	HW1	SOL	2178	28.370	5.000	6.240	1.00	0.00
ATOM	8618	HW2	SOL	2178	29.540	4.490	7.050	1.00	0.00
ATOM	8619	OW	SOL	2179	51.790	30.230	10.760	1.00	0.00
ATOM	8620	HW1	SOL	2179	50.970	30.230	11.260	1.00	0.00
ATOM	8621	HW2	SOL	2179	51.620	29.640	10.030	1.00	0.00
ATOM	8622	OW	SOL	2180	52.780	48.790	7.980	1.00	0.00
ATOM	8623	HW1	SOL	2180	52.850	49.620	7.500	1.00	0.00

ATOM	8624	HW2	SOL	2180	52.880	48.110	7.320	1.00	0.00
ATOM	8625	OW	SOL	2181	12.660	18.890	47.680	1.00	0.00
ATOM	8626	HW1	SOL	2181	13.540	18.700	47.370	1.00	0.00
ATOM	8627	HW2	SOL	2181	12.080	18.540	47.000	1.00	0.00
ATOM	8628	OW	SOL	2182	37.060	1.010	8.810	1.00	0.00
ATOM	8629	HW1	SOL	2182	37.810	0.700	9.330	1.00	0.00
ATOM	8630	HW2	SOL	2182	36.990	0.380	8.090	1.00	0.00
ATOM	8631	OW	SOL	2183	34.530	28.960	22.100	1.00	0.00
ATOM	8632	HW1	SOL	2183	34.310	28.110	21.710	1.00	0.00
ATOM	8633	HW2	SOL	2183	35.390	28.830	22.480	1.00	0.00
ATOM	8634	OW	SOL	2184	14.200	9.570	19.230	1.00	0.00
ATOM	8635	HW1	SOL	2184	14.710	8.860	18.860	1.00	0.00
ATOM	8636	HW2	SOL	2184	13.490	9.720	18.600	1.00	0.00
ATOM	8637	OW	SOL	2185	46.040	39.410	46.000	1.00	0.00
ATOM	8638	HW1	SOL	2185	46.870	39.800	46.280	1.00	0.00
ATOM	8639	HW2	SOL	2185	45.380	40.070	46.230	1.00	0.00
ATOM	8640	OW	SOL	2186	48.480	23.970	12.230	1.00	0.00
ATOM	8641	HW1	SOL	2186	48.100	24.840	12.370	1.00	0.00
ATOM	8642	HW2	SOL	2186	49.060	23.830	12.970	1.00	0.00
ATOM	8643	OW	SOL	2187	1.140	48.410	31.090	1.00	0.00
ATOM	8644	HW1	SOL	2187	1.180	47.570	30.630	1.00	0.00
ATOM	8645	HW2	SOL	2187	2.040	48.730	31.090	1.00	0.00
ATOM	8646	OW	SOL	2188	41.020	33.600	36.350	1.00	0.00
ATOM	8647	HW1	SOL	2188	40.660	33.680	35.460	1.00	0.00
ATOM	8648	HW2	SOL	2188	40.260	33.690	36.920	1.00	0.00
ATOM	8649	OW	SOL	2189	43.990	55.300	16.420	1.00	0.00
ATOM	8650	HW1	SOL	2189	44.560	55.370	15.660	1.00	0.00
ATOM	8651	HW2	SOL	2189	43.240	55.860	16.210	1.00	0.00
ATOM	8652	OW	SOL	2190	12.360	36.780	37.970	1.00	0.00
ATOM	8653	HW1	SOL	2190	11.540	37.210	37.740	1.00	0.00
ATOM	8654	HW2	SOL	2190	13.010	37.490	37.970	1.00	0.00
ATOM	8655	OW	SOL	2191	49.510	46.340	8.660	1.00	0.00
ATOM	8656	HW1	SOL	2191	49.300	45.780	9.410	1.00	0.00
ATOM	8657	HW2	SOL	2191	48.950	46.040	7.960	1.00	0.00
ATOM	8658	OW	SOL	2192	20.120	15.240	18.910	1.00	0.00
ATOM	8659	HW1	SOL	2192	19.650	16.000	18.560	1.00	0.00
ATOM	8660	HW2	SOL	2192	19.720	14.490	18.470	1.00	0.00
ATOM	8661	OW	SOL	2193	16.970	11.140	10.200	1.00	0.00
ATOM	8662	HW1	SOL	2193	17.370	11.990	10.390	1.00	0.00
ATOM	8663	HW2	SOL	2193	17.060	10.650	11.020	1.00	0.00
ATOM	8664	OW	SOL	2194	27.260	10.500	41.310	1.00	0.00
ATOM	8665	HW1	SOL	2194	27.620	10.410	40.430	1.00	0.00
ATOM	8666	HW2	SOL	2194	26.460	11.020	41.200	1.00	0.00
ATOM	8667	OW	SOL	2195	14.950	7.510	28.860	1.00	0.00

ATOM	8668	HW1	SOL	2195	14.360	6.870	29.250	1.00	0.00
ATOM	8669	HW2	SOL	2195	15.110	7.180	27.970	1.00	0.00
ATOM	8670	OW	SOL	2196	38.780	13.650	13.530	1.00	0.00
ATOM	8671	HW1	SOL	2196	37.990	13.620	14.070	1.00	0.00
ATOM	8672	HW2	SOL	2196	39.450	13.990	14.120	1.00	0.00
ATOM	8673	OW	SOL	2197	36.640	22.710	55.150	1.00	0.00
ATOM	8674	HW1	SOL	2197	36.300	23.520	54.780	1.00	0.00
ATOM	8675	HW2	SOL	2197	37.440	22.970	55.610	1.00	0.00
ATOM	8676	OW	SOL	2198	31.370	8.710	12.200	1.00	0.00
ATOM	8677	HW1	SOL	2198	30.550	9.060	11.850	1.00	0.00
ATOM	8678	HW2	SOL	2198	32.040	9.000	11.580	1.00	0.00
ATOM	8679	OW	SOL	2199	16.760	41.990	7.450	1.00	0.00
ATOM	8680	HW1	SOL	2199	15.840	42.030	7.210	1.00	0.00
ATOM	8681	HW2	SOL	2199	16.830	41.230	8.020	1.00	0.00
ATOM	8682	OW	SOL	2200	26.150	18.770	53.580	1.00	0.00
ATOM	8683	HW1	SOL	2200	26.330	18.410	52.710	1.00	0.00
ATOM	8684	HW2	SOL	2200	25.200	18.860	53.610	1.00	0.00
ATOM	8685	OW	SOL	2201	31.790	5.960	3.720	1.00	0.00
ATOM	8686	HW1	SOL	2201	31.200	5.220	3.890	1.00	0.00
ATOM	8687	HW2	SOL	2201	32.550	5.800	4.280	1.00	0.00
ATOM	8688	OW	SOL	2202	49.120	18.070	54.760	1.00	0.00
ATOM	8689	HW1	SOL	2202	49.010	19.000	54.950	1.00	0.00
ATOM	8690	HW2	SOL	2202	48.370	17.640	55.160	1.00	0.00
ATOM	8691	OW	SOL	2203	12.140	6.740	46.060	1.00	0.00
ATOM	8692	HW1	SOL	2203	11.770	5.880	46.230	1.00	0.00
ATOM	8693	HW2	SOL	2203	11.510	7.360	46.430	1.00	0.00
ATOM	8694	OW	SOL	2204	51.690	11.360	7.280	1.00	0.00
ATOM	8695	HW1	SOL	2204	52.000	11.430	6.380	1.00	0.00
ATOM	8696	HW2	SOL	2204	52.220	12.000	7.760	1.00	0.00
ATOM	8697	OW	SOL	2205	48.900	40.560	41.440	1.00	0.00
ATOM	8698	HW1	SOL	2205	49.780	40.190	41.370	1.00	0.00
ATOM	8699	HW2	SOL	2205	48.700	40.870	40.560	1.00	0.00
ATOM	8700	OW	SOL	2206	42.120	20.060	5.790	1.00	0.00
ATOM	8701	HW1	SOL	2206	42.450	19.730	4.950	1.00	0.00
ATOM	8702	HW2	SOL	2206	41.950	20.990	5.630	1.00	0.00
ATOM	8703	OW	SOL	2207	1.140	38.570	36.730	1.00	0.00
ATOM	8704	HW1	SOL	2207	0.850	37.700	37.000	1.00	0.00
ATOM	8705	HW2	SOL	2207	2.090	38.510	36.730	1.00	0.00
ATOM	8706	OW	SOL	2208	3.930	37.040	2.280	1.00	0.00
ATOM	8707	HW1	SOL	2208	4.450	37.570	2.890	1.00	0.00
ATOM	8708	HW2	SOL	2208	4.380	36.200	2.240	1.00	0.00
ATOM	8709	OW	SOL	2209	0.370	6.410	32.380	1.00	0.00
ATOM	8710	HW1	SOL	2209	1.240	6.630	32.690	1.00	0.00
ATOM	8711	HW2	SOL	2209	0.190	5.550	32.760	1.00	0.00

ATOM	8712	OW	SOL	2210	54.450	6.720	3.110	1.00	0.00
ATOM	8713	HW1	SOL	2210	54.580	5.770	2.990	1.00	0.00
ATOM	8714	HW2	SOL	2210	55.180	7.120	2.630	1.00	0.00
ATOM	8715	OW	SOL	2211	36.310	22.980	39.460	1.00	0.00
ATOM	8716	HW1	SOL	2211	36.610	23.230	38.590	1.00	0.00
ATOM	8717	HW2	SOL	2211	36.360	22.030	39.470	1.00	0.00
ATOM	8718	OW	SOL	2212	34.080	47.470	34.570	1.00	0.00
ATOM	8719	HW1	SOL	2212	34.760	48.140	34.570	1.00	0.00
ATOM	8720	HW2	SOL	2212	33.730	47.480	35.460	1.00	0.00
ATOM	8721	OW	SOL	2213	7.240	16.590	18.130	1.00	0.00
ATOM	8722	HW1	SOL	2213	7.560	16.040	18.850	1.00	0.00
ATOM	8723	HW2	SOL	2213	6.290	16.470	18.130	1.00	0.00
ATOM	8724	OW	SOL	2214	27.690	23.170	9.830	1.00	0.00
ATOM	8725	HW1	SOL	2214	28.240	23.280	10.610	1.00	0.00
ATOM	8726	HW2	SOL	2214	26.920	22.700	10.150	1.00	0.00
ATOM	8727	OW	SOL	2215	39.710	53.840	2.910	1.00	0.00
ATOM	8728	HW1	SOL	2215	40.480	54.400	3.000	1.00	0.00
ATOM	8729	HW2	SOL	2215	39.810	53.190	3.610	1.00	0.00
ATOM	8730	OW	SOL	2216	40.540	22.600	36.700	1.00	0.00
ATOM	8731	HW1	SOL	2216	41.340	22.130	36.460	1.00	0.00
ATOM	8732	HW2	SOL	2216	40.350	22.320	37.600	1.00	0.00
ATOM	8733	OW	SOL	2217	45.210	0.430	18.840	1.00	0.00
ATOM	8734	HW1	SOL	2217	45.810	-0.310	19.010	1.00	0.00
ATOM	8735	HW2	SOL	2217	44.700	0.150	18.080	1.00	0.00
ATOM	8736	OW	SOL	2218	15.260	26.140	26.900	1.00	0.00
ATOM	8737	HW1	SOL	2218	14.330	26.050	26.680	1.00	0.00
ATOM	8738	HW2	SOL	2218	15.710	25.630	26.220	1.00	0.00
ATOM	8739	OW	SOL	2219	37.210	21.220	4.080	1.00	0.00
ATOM	8740	HW1	SOL	2219	36.570	20.990	3.410	1.00	0.00
ATOM	8741	HW2	SOL	2219	37.250	20.460	4.650	1.00	0.00
ATOM	8742	OW	SOL	2220	7.670	30.370	35.680	1.00	0.00
ATOM	8743	HW1	SOL	2220	7.040	30.570	36.380	1.00	0.00
ATOM	8744	HW2	SOL	2220	8.510	30.310	36.120	1.00	0.00
ATOM	8745	OW	SOL	2221	22.530	0.330	6.930	1.00	0.00
ATOM	8746	HW1	SOL	2221	21.880	-0.060	7.530	1.00	0.00
ATOM	8747	HW2	SOL	2221	22.790	-0.400	6.360	1.00	0.00
ATOM	8748	OW	SOL	2222	17.100	0.940	5.410	1.00	0.00
ATOM	8749	HW1	SOL	2222	17.030	1.590	6.120	1.00	0.00
ATOM	8750	HW2	SOL	2222	18.030	0.980	5.150	1.00	0.00
ATOM	8751	OW	SOL	2223	13.520	27.440	48.440	1.00	0.00
ATOM	8752	HW1	SOL	2223	13.950	28.120	48.950	1.00	0.00
ATOM	8753	HW2	SOL	2223	12.940	27.000	49.060	1.00	0.00
ATOM	8754	OW	SOL	2224	13.640	9.740	26.380	1.00	0.00
ATOM	8755	HW1	SOL	2224	13.900	9.880	27.290	1.00	0.00

ATOM	8756	HW2	SOL	2224	12.940	10.370	26.230	1.00	0.00
ATOM	8757	OW	SOL	2225	22.220	27.120	12.120	1.00	0.00
ATOM	8758	HW1	SOL	2225	22.500	28.000	11.850	1.00	0.00
ATOM	8759	HW2	SOL	2225	22.340	27.120	13.070	1.00	0.00
ATOM	8760	OW	SOL	2226	31.770	10.270	32.320	1.00	0.00
ATOM	8761	HW1	SOL	2226	32.400	10.310	33.040	1.00	0.00
ATOM	8762	HW2	SOL	2226	31.150	9.590	32.560	1.00	0.00
ATOM	8763	OW	SOL	2227	19.500	36.500	48.270	1.00	0.00
ATOM	8764	HW1	SOL	2227	18.750	35.940	48.440	1.00	0.00
ATOM	8765	HW2	SOL	2227	19.310	36.900	47.420	1.00	0.00
ATOM	8766	OW	SOL	2228	26.610	19.740	9.230	1.00	0.00
ATOM	8767	HW1	SOL	2228	26.000	19.710	9.970	1.00	0.00
ATOM	8768	HW2	SOL	2228	27.480	19.720	9.630	1.00	0.00
ATOM	8769	OW	SOL	2229	41.940	25.700	19.920	1.00	0.00
ATOM	8770	HW1	SOL	2229	42.020	25.080	20.640	1.00	0.00
ATOM	8771	HW2	SOL	2229	41.450	26.440	20.290	1.00	0.00
ATOM	8772	OW	SOL	2230	20.450	2.920	48.780	1.00	0.00
ATOM	8773	HW1	SOL	2230	19.620	3.380	48.860	1.00	0.00
ATOM	8774	HW2	SOL	2230	21.110	3.600	48.890	1.00	0.00
ATOM	8775	OW	SOL	2231	20.410	22.910	1.470	1.00	0.00
ATOM	8776	HW1	SOL	2231	20.080	23.670	1.950	1.00	0.00
ATOM	8777	HW2	SOL	2231	21.020	22.490	2.070	1.00	0.00
ATOM	8778	OW	SOL	2232	53.450	21.010	7.990	1.00	0.00
ATOM	8779	HW1	SOL	2232	53.060	21.030	8.870	1.00	0.00
ATOM	8780	HW2	SOL	2232	54.240	21.540	8.070	1.00	0.00
ATOM	8781	OW	SOL	2233	6.730	19.840	55.130	1.00	0.00
ATOM	8782	HW1	SOL	2233	6.550	20.160	56.010	1.00	0.00
ATOM	8783	HW2	SOL	2233	5.870	19.800	54.710	1.00	0.00
ATOM	8784	OW	SOL	2234	37.950	16.420	18.480	1.00	0.00
ATOM	8785	HW1	SOL	2234	37.040	16.210	18.260	1.00	0.00
ATOM	8786	HW2	SOL	2234	38.150	15.830	19.210	1.00	0.00
ATOM	8787	OW	SOL	2235	40.110	39.500	42.730	1.00	0.00
ATOM	8788	HW1	SOL	2235	40.830	39.960	43.160	1.00	0.00
ATOM	8789	HW2	SOL	2235	40.290	38.570	42.890	1.00	0.00
ATOM	8790	OW	SOL	2236	4.120	17.710	21.780	1.00	0.00
ATOM	8791	HW1	SOL	2236	3.500	17.340	21.150	1.00	0.00
ATOM	8792	HW2	SOL	2236	3.570	18.110	22.460	1.00	0.00
ATOM	8793	OW	SOL	2237	52.500	19.060	26.310	1.00	0.00
ATOM	8794	HW1	SOL	2237	52.580	18.340	25.690	1.00	0.00
ATOM	8795	HW2	SOL	2237	53.270	18.990	26.870	1.00	0.00
ATOM	8796	OW	SOL	2238	48.750	47.530	25.490	1.00	0.00
ATOM	8797	HW1	SOL	2238	49.130	48.200	24.920	1.00	0.00
ATOM	8798	HW2	SOL	2238	49.150	47.690	26.340	1.00	0.00
ATOM	8799	OW	SOL	2239	12.800	0.450	33.760	1.00	0.00

ATOM	8800	HW1	SOL	2239	12.190	1.190	33.790	1.00	0.00
ATOM	8801	HW2	SOL	2239	13.120	0.370	34.650	1.00	0.00
ATOM	8802	OW	SOL	2240	36.570	29.380	7.520	1.00	0.00
ATOM	8803	HW1	SOL	2240	36.700	29.150	8.440	1.00	0.00
ATOM	8804	HW2	SOL	2240	35.980	28.700	7.190	1.00	0.00
ATOM	8805	OW	SOL	2241	15.380	7.530	17.760	1.00	0.00
ATOM	8806	HW1	SOL	2241	15.660	8.190	17.130	1.00	0.00
ATOM	8807	HW2	SOL	2241	16.140	6.960	17.870	1.00	0.00
ATOM	8808	OW	SOL	2242	7.340	14.100	4.430	1.00	0.00
ATOM	8809	HW1	SOL	2242	7.730	13.390	3.910	1.00	0.00
ATOM	8810	HW2	SOL	2242	6.780	13.650	5.060	1.00	0.00
ATOM	8811	OW	SOL	2243	48.100	49.370	52.470	1.00	0.00
ATOM	8812	HW1	SOL	2243	48.150	48.410	52.420	1.00	0.00
ATOM	8813	HW2	SOL	2243	48.920	49.670	52.080	1.00	0.00
ATOM	8814	OW	SOL	2244	18.170	17.060	4.150	1.00	0.00
ATOM	8815	HW1	SOL	2244	18.170	16.720	3.260	1.00	0.00
ATOM	8816	HW2	SOL	2244	17.320	16.800	4.520	1.00	0.00
ATOM	8817	OW	SOL	2245	26.850	13.470	30.180	1.00	0.00
ATOM	8818	HW1	SOL	2245	27.770	13.650	29.980	1.00	0.00
ATOM	8819	HW2	SOL	2245	26.670	14.010	30.950	1.00	0.00
ATOM	8820	OW	SOL	2246	35.760	44.050	39.230	1.00	0.00
ATOM	8821	HW1	SOL	2246	36.220	43.580	38.540	1.00	0.00
ATOM	8822	HW2	SOL	2246	36.000	43.590	40.030	1.00	0.00
ATOM	8823	OW	SOL	2247	50.490	22.250	23.510	1.00	0.00
ATOM	8824	HW1	SOL	2247	50.190	23.160	23.560	1.00	0.00
ATOM	8825	HW2	SOL	2247	50.860	22.160	22.630	1.00	0.00
ATOM	8826	OW	SOL	2248	52.050	28.280	13.090	1.00	0.00
ATOM	8827	HW1	SOL	2248	52.360	28.060	12.210	1.00	0.00
ATOM	8828	HW2	SOL	2248	52.110	29.240	13.130	1.00	0.00
ATOM	8829	OW	SOL	2249	36.250	31.360	13.350	1.00	0.00
ATOM	8830	HW1	SOL	2249	36.520	31.850	12.580	1.00	0.00
ATOM	8831	HW2	SOL	2249	35.990	30.500	13.020	1.00	0.00
ATOM	8832	OW	SOL	2250	3.830	11.300	22.510	1.00	0.00
ATOM	8833	HW1	SOL	2250	3.880	12.140	22.040	1.00	0.00
ATOM	8834	HW2	SOL	2250	3.050	11.380	23.050	1.00	0.00
ATOM	8835	OW	SOL	2251	1.390	45.720	10.550	1.00	0.00
ATOM	8836	HW1	SOL	2251	0.990	46.530	10.830	1.00	0.00
ATOM	8837	HW2	SOL	2251	1.290	45.720	9.590	1.00	0.00
ATOM	8838	OW	SOL	2252	8.840	52.270	50.180	1.00	0.00
ATOM	8839	HW1	SOL	2252	8.500	51.990	51.030	1.00	0.00
ATOM	8840	HW2	SOL	2252	8.650	53.210	50.150	1.00	0.00
ATOM	8841	OW	SOL	2253	14.330	28.130	2.060	1.00	0.00
ATOM	8842	HW1	SOL	2253	14.150	27.930	2.980	1.00	0.00
ATOM	8843	HW2	SOL	2253	14.700	29.010	2.070	1.00	0.00

ATOM	8844	OW	SOL	2254	53.640	33.230	13.880	1.00	0.00
ATOM	8845	HW1	SOL	2254	54.490	32.820	13.990	1.00	0.00
ATOM	8846	HW2	SOL	2254	53.830	34.160	13.770	1.00	0.00
ATOM	8847	OW	SOL	2255	46.850	19.800	35.270	1.00	0.00
ATOM	8848	HW1	SOL	2255	46.220	20.510	35.250	1.00	0.00
ATOM	8849	HW2	SOL	2255	47.700	20.230	35.230	1.00	0.00
ATOM	8850	OW	SOL	2256	18.160	50.160	50.830	1.00	0.00
ATOM	8851	HW1	SOL	2256	19.010	50.010	50.420	1.00	0.00
ATOM	8852	HW2	SOL	2256	18.320	50.890	51.450	1.00	0.00
ATOM	8853	OW	SOL	2257	10.370	4.170	10.090	1.00	0.00
ATOM	8854	HW1	SOL	2257	9.470	4.070	9.770	1.00	0.00
ATOM	8855	HW2	SOL	2257	10.730	3.280	10.080	1.00	0.00
ATOM	8856	OW	SOL	2258	11.700	35.070	17.920	1.00	0.00
ATOM	8857	HW1	SOL	2258	11.480	35.940	18.270	1.00	0.00
ATOM	8858	HW2	SOL	2258	10.880	34.580	17.960	1.00	0.00
ATOM	8859	OW	SOL	2259	12.160	2.920	26.460	1.00	0.00
ATOM	8860	HW1	SOL	2259	11.910	3.720	26.010	1.00	0.00
ATOM	8861	HW2	SOL	2259	11.790	3.010	27.340	1.00	0.00
ATOM	8862	OW	SOL	2260	54.910	45.880	3.250	1.00	0.00
ATOM	8863	HW1	SOL	2260	55.180	46.060	4.150	1.00	0.00
ATOM	8864	HW2	SOL	2260	54.340	46.620	3.030	1.00	0.00
ATOM	8865	OW	SOL	2261	50.790	37.490	6.400	1.00	0.00
ATOM	8866	HW1	SOL	2261	51.330	37.540	7.190	1.00	0.00
ATOM	8867	HW2	SOL	2261	51.330	37.010	5.770	1.00	0.00
ATOM	8868	OW	SOL	2262	49.420	6.670	46.780	1.00	0.00
ATOM	8869	HW1	SOL	2262	48.610	7.130	46.570	1.00	0.00
ATOM	8870	HW2	SOL	2262	49.570	6.090	46.030	1.00	0.00
ATOM	8871	OW	SOL	2263	5.490	40.510	20.020	1.00	0.00
ATOM	8872	HW1	SOL	2263	4.620	40.120	20.030	1.00	0.00
ATOM	8873	HW2	SOL	2263	5.380	41.350	19.580	1.00	0.00
ATOM	8874	OW	SOL	2264	38.050	10.840	26.670	1.00	0.00
ATOM	8875	HW1	SOL	2264	37.610	11.340	27.360	1.00	0.00
ATOM	8876	HW2	SOL	2264	37.360	10.300	26.280	1.00	0.00
ATOM	8877	OW	SOL	2265	34.890	26.710	20.120	1.00	0.00
ATOM	8878	HW1	SOL	2265	35.000	26.900	19.180	1.00	0.00
ATOM	8879	HW2	SOL	2265	35.590	26.090	20.320	1.00	0.00
ATOM	8880	OW	SOL	2266	49.210	33.740	11.840	1.00	0.00
ATOM	8881	HW1	SOL	2266	48.910	32.830	11.830	1.00	0.00
ATOM	8882	HW2	SOL	2266	49.980	33.730	12.400	1.00	0.00
ATOM	8883	OW	SOL	2267	5.510	41.070	40.290	1.00	0.00
ATOM	8884	HW1	SOL	2267	6.140	41.220	39.590	1.00	0.00
ATOM	8885	HW2	SOL	2267	5.150	40.200	40.100	1.00	0.00
ATOM	8886	OW	SOL	2268	32.910	20.770	38.680	1.00	0.00
ATOM	8887	HW1	SOL	2268	31.960	20.880	38.600	1.00	0.00

ATOM	8888	HW2	SOL	2268	33.020	19.910	39.090	1.00	0.00
ATOM	8889	OW	SOL	2269	44.710	43.090	32.890	1.00	0.00
ATOM	8890	HW1	SOL	2269	44.740	43.230	31.950	1.00	0.00
ATOM	8891	HW2	SOL	2269	45.020	42.190	33.010	1.00	0.00
ATOM	8892	OW	SOL	2270	53.450	50.260	14.580	1.00	0.00
ATOM	8893	HW1	SOL	2270	53.070	49.450	14.910	1.00	0.00
ATOM	8894	HW2	SOL	2270	53.830	50.010	13.730	1.00	0.00
ATOM	8895	OW	SOL	2271	7.280	26.500	50.980	1.00	0.00
ATOM	8896	HW1	SOL	2271	7.080	25.570	50.900	1.00	0.00
ATOM	8897	HW2	SOL	2271	8.080	26.620	50.450	1.00	0.00
ATOM	8898	OW	SOL	2272	5.000	18.090	31.280	1.00	0.00
ATOM	8899	HW1	SOL	2272	4.580	18.430	30.490	1.00	0.00
ATOM	8900	HW2	SOL	2272	4.920	18.790	31.920	1.00	0.00
ATOM	8901	OW	SOL	2273	30.430	22.040	39.460	1.00	0.00
ATOM	8902	HW1	SOL	2273	30.070	22.330	40.290	1.00	0.00
ATOM	8903	HW2	SOL	2273	30.000	22.600	38.800	1.00	0.00
ATOM	8904	OW	SOL	2274	3.040	41.220	49.990	1.00	0.00
ATOM	8905	HW1	SOL	2274	2.410	40.500	50.030	1.00	0.00
ATOM	8906	HW2	SOL	2274	3.000	41.520	49.080	1.00	0.00
ATOM	8907	OW	SOL	2275	35.140	18.030	1.050	1.00	0.00
ATOM	8908	HW1	SOL	2275	34.320	17.730	0.670	1.00	0.00
ATOM	8909	HW2	SOL	2275	34.910	18.810	1.550	1.00	0.00
ATOM	8910	OW	SOL	2276	13.070	22.760	13.050	1.00	0.00
ATOM	8911	HW1	SOL	2276	13.900	22.430	12.720	1.00	0.00
ATOM	8912	HW2	SOL	2276	12.990	22.390	13.930	1.00	0.00
ATOM	8913	OW	SOL	2277	21.410	6.430	34.130	1.00	0.00
ATOM	8914	HW1	SOL	2277	21.460	7.170	33.520	1.00	0.00
ATOM	8915	HW2	SOL	2277	20.490	6.180	34.130	1.00	0.00
ATOM	8916	OW	SOL	2278	3.360	39.460	1.110	1.00	0.00
ATOM	8917	HW1	SOL	2278	3.640	38.590	1.400	1.00	0.00
ATOM	8918	HW2	SOL	2278	3.660	39.520	0.210	1.00	0.00
ATOM	8919	OW	SOL	2279	28.990	29.320	38.550	1.00	0.00
ATOM	8920	HW1	SOL	2279	29.740	29.760	38.140	1.00	0.00
ATOM	8921	HW2	SOL	2279	28.730	28.660	37.910	1.00	0.00
ATOM	8922	OW	SOL	2280	52.460	16.340	4.470	1.00	0.00
ATOM	8923	HW1	SOL	2280	52.340	17.180	4.900	1.00	0.00
ATOM	8924	HW2	SOL	2280	51.610	16.140	4.090	1.00	0.00
ATOM	8925	OW	SOL	2281	24.290	2.060	46.240	1.00	0.00
ATOM	8926	HW1	SOL	2281	24.210	2.960	45.920	1.00	0.00
ATOM	8927	HW2	SOL	2281	24.970	2.100	46.910	1.00	0.00
ATOM	8928	OW	SOL	2282	3.700	38.100	36.110	1.00	0.00
ATOM	8929	HW1	SOL	2282	4.560	38.460	35.870	1.00	0.00
ATOM	8930	HW2	SOL	2282	3.150	38.260	35.350	1.00	0.00
ATOM	8931	OW	SOL	2283	45.040	39.030	54.980	1.00	0.00

ATOM	8932	HW1	SOL	2283	45.340	39.510	55.750	1.00	0.00
ATOM	8933	HW2	SOL	2283	44.130	38.790	55.190	1.00	0.00
ATOM	8934	OW	SOL	2284	14.730	41.030	53.880	1.00	0.00
ATOM	8935	HW1	SOL	2284	15.160	41.740	53.400	1.00	0.00
ATOM	8936	HW2	SOL	2284	15.300	40.850	54.620	1.00	0.00
ATOM	8937	OW	SOL	2285	3.410	21.370	37.590	1.00	0.00
ATOM	8938	HW1	SOL	2285	3.210	20.780	38.310	1.00	0.00
ATOM	8939	HW2	SOL	2285	3.050	22.220	37.860	1.00	0.00
ATOM	8940	OW	SOL	2286	11.620	32.330	24.960	1.00	0.00
ATOM	8941	HW1	SOL	2286	12.290	31.850	24.470	1.00	0.00
ATOM	8942	HW2	SOL	2286	11.740	33.240	24.700	1.00	0.00
ATOM	8943	OW	SOL	2287	4.710	39.670	23.310	1.00	0.00
ATOM	8944	HW1	SOL	2287	3.970	40.180	23.620	1.00	0.00
ATOM	8945	HW2	SOL	2287	4.340	39.060	22.670	1.00	0.00
ATOM	8946	OW	SOL	2288	15.710	47.120	48.340	1.00	0.00
ATOM	8947	HW1	SOL	2288	15.840	46.290	47.880	1.00	0.00
ATOM	8948	HW2	SOL	2288	14.910	47.000	48.850	1.00	0.00
ATOM	8949	OW	SOL	2289	26.070	6.180	40.610	1.00	0.00
ATOM	8950	HW1	SOL	2289	26.980	6.470	40.730	1.00	0.00
ATOM	8951	HW2	SOL	2289	25.630	6.930	40.220	1.00	0.00
ATOM	8952	OW	SOL	2290	34.760	36.570	10.600	1.00	0.00
ATOM	8953	HW1	SOL	2290	33.890	36.600	10.210	1.00	0.00
ATOM	8954	HW2	SOL	2290	34.760	35.770	11.120	1.00	0.00
ATOM	8955	OW	SOL	2291	36.950	22.380	10.700	1.00	0.00
ATOM	8956	HW1	SOL	2291	37.300	23.080	10.140	1.00	0.00
ATOM	8957	HW2	SOL	2291	36.000	22.450	10.610	1.00	0.00
ATOM	8958	OW	SOL	2292	44.680	49.180	46.220	1.00	0.00
ATOM	8959	HW1	SOL	2292	45.580	49.150	45.910	1.00	0.00
ATOM	8960	HW2	SOL	2292	44.580	50.060	46.580	1.00	0.00
ATOM	8961	OW	SOL	2293	44.030	36.730	40.280	1.00	0.00
ATOM	8962	HW1	SOL	2293	44.900	37.130	40.260	1.00	0.00
ATOM	8963	HW2	SOL	2293	43.740	36.850	41.180	1.00	0.00
ATOM	8964	OW	SOL	2294	5.180	13.230	41.980	1.00	0.00
ATOM	8965	HW1	SOL	2294	4.700	12.760	42.660	1.00	0.00
ATOM	8966	HW2	SOL	2294	5.830	12.590	41.660	1.00	0.00
ATOM	8967	OW	SOL	2295	35.220	13.170	20.750	1.00	0.00
ATOM	8968	HW1	SOL	2295	34.700	12.390	20.960	1.00	0.00
ATOM	8969	HW2	SOL	2295	34.660	13.670	20.160	1.00	0.00
ATOM	8970	OW	SOL	2296	45.820	41.300	5.090	1.00	0.00
ATOM	8971	HW1	SOL	2296	45.430	41.350	5.970	1.00	0.00
ATOM	8972	HW2	SOL	2296	45.730	40.390	4.840	1.00	0.00
ATOM	8973	OW	SOL	2297	54.480	25.220	42.640	1.00	0.00
ATOM	8974	HW1	SOL	2297	53.950	24.760	43.280	1.00	0.00
ATOM	8975	HW2	SOL	2297	54.080	25.020	41.800	1.00	0.00

ATOM	8976	OW	SOL	2298	39.990	30.910	32.410	1.00	0.00
ATOM	8977	HW1	SOL	2298	39.680	31.720	32.000	1.00	0.00
ATOM	8978	HW2	SOL	2298	39.210	30.540	32.830	1.00	0.00
ATOM	8979	OW	SOL	2299	15.350	21.640	19.870	1.00	0.00
ATOM	8980	HW1	SOL	2299	15.560	22.490	19.490	1.00	0.00
ATOM	8981	HW2	SOL	2299	15.820	21.620	20.700	1.00	0.00
ATOM	8982	OW	SOL	2300	41.550	15.260	39.000	1.00	0.00
ATOM	8983	HW1	SOL	2300	42.030	15.020	38.200	1.00	0.00
ATOM	8984	HW2	SOL	2300	42.120	15.870	39.450	1.00	0.00
ATOM	8985	OW	SOL	2301	49.870	32.470	17.990	1.00	0.00
ATOM	8986	HW1	SOL	2301	50.130	32.910	17.170	1.00	0.00
ATOM	8987	HW2	SOL	2301	50.510	31.770	18.100	1.00	0.00
ATOM	8988	OW	SOL	2302	1.490	41.440	7.860	1.00	0.00
ATOM	8989	HW1	SOL	2302	0.560	41.240	7.760	1.00	0.00
ATOM	8990	HW2	SOL	2302	1.940	40.690	7.470	1.00	0.00
ATOM	8991	OW	SOL	2303	49.830	9.280	24.900	1.00	0.00
ATOM	8992	HW1	SOL	2303	49.690	10.130	25.320	1.00	0.00
ATOM	8993	HW2	SOL	2303	50.720	9.040	25.130	1.00	0.00
ATOM	8994	OW	SOL	2304	43.670	48.820	2.000	1.00	0.00
ATOM	8995	HW1	SOL	2304	44.210	49.590	2.120	1.00	0.00
ATOM	8996	HW2	SOL	2304	43.400	48.850	1.080	1.00	0.00
ATOM	8997	OW	SOL	2305	3.740	14.060	9.030	1.00	0.00
ATOM	8998	HW1	SOL	2305	4.510	13.500	8.930	1.00	0.00
ATOM	8999	HW2	SOL	2305	4.070	14.940	8.840	1.00	0.00
ATOM	9000	OW	SOL	2306	54.020	30.070	28.390	1.00	0.00
ATOM	9001	HW1	SOL	2306	53.560	29.240	28.560	1.00	0.00
ATOM	9002	HW2	SOL	2306	54.780	30.040	28.970	1.00	0.00
ATOM	9003	OW	SOL	2307	2.790	53.450	7.330	1.00	0.00
ATOM	9004	HW1	SOL	2307	3.550	53.160	6.820	1.00	0.00
ATOM	9005	HW2	SOL	2307	2.680	52.760	7.990	1.00	0.00
ATOM	9006	OW	SOL	2308	41.550	26.120	9.440	1.00	0.00
ATOM	9007	HW1	SOL	2308	41.170	27.000	9.420	1.00	0.00
ATOM	9008	HW2	SOL	2308	42.210	26.170	10.130	1.00	0.00
ATOM	9009	OW	SOL	2309	2.050	31.440	4.690	1.00	0.00
ATOM	9010	HW1	SOL	2309	2.180	32.280	4.250	1.00	0.00
ATOM	9011	HW2	SOL	2309	1.190	31.510	5.100	1.00	0.00
ATOM	9012	OW	SOL	2310	27.420	26.190	19.620	1.00	0.00
ATOM	9013	HW1	SOL	2310	27.810	26.220	18.750	1.00	0.00
ATOM	9014	HW2	SOL	2310	28.120	25.840	20.180	1.00	0.00
ATOM	9015	OW	SOL	2311	9.610	5.030	46.210	1.00	0.00
ATOM	9016	HW1	SOL	2311	9.460	4.670	47.080	1.00	0.00
ATOM	9017	HW2	SOL	2311	8.760	5.380	45.940	1.00	0.00
ATOM	9018	OW	SOL	2312	28.010	48.040	24.090	1.00	0.00
ATOM	9019	HW1	SOL	2312	28.330	48.610	23.390	1.00	0.00

ATOM	9020	HW2	SOL	2312	27.830	48.630	24.820	1.00	0.00
ATOM	9021	OW	SOL	2313	23.900	33.010	47.390	1.00	0.00
ATOM	9022	HW1	SOL	2313	23.370	32.390	46.890	1.00	0.00
ATOM	9023	HW2	SOL	2313	23.640	33.870	47.050	1.00	0.00
ATOM	9024	OW	SOL	2314	28.490	1.190	5.860	1.00	0.00
ATOM	9025	HW1	SOL	2314	28.970	1.390	6.660	1.00	0.00
ATOM	9026	HW2	SOL	2314	28.160	0.300	6.000	1.00	0.00
ATOM	9027	OW	SOL	2315	5.900	20.110	13.120	1.00	0.00
ATOM	9028	HW1	SOL	2315	5.670	20.680	13.850	1.00	0.00
ATOM	9029	HW2	SOL	2315	5.060	19.790	12.790	1.00	0.00
ATOM	9030	OW	SOL	2316	1.640	40.030	44.050	1.00	0.00
ATOM	9031	HW1	SOL	2316	1.280	40.820	44.460	1.00	0.00
ATOM	9032	HW2	SOL	2316	1.510	40.170	43.110	1.00	0.00
ATOM	9033	OW	SOL	2317	42.270	14.920	6.120	1.00	0.00
ATOM	9034	HW1	SOL	2317	42.680	14.840	5.260	1.00	0.00
ATOM	9035	HW2	SOL	2317	42.560	15.770	6.460	1.00	0.00
ATOM	9036	OW	SOL	2318	22.840	53.050	42.010	1.00	0.00
ATOM	9037	HW1	SOL	2318	23.020	52.690	41.130	1.00	0.00
ATOM	9038	HW2	SOL	2318	23.390	52.530	42.590	1.00	0.00
ATOM	9039	OW	SOL	2319	21.640	30.680	39.180	1.00	0.00
ATOM	9040	HW1	SOL	2319	20.880	30.990	39.670	1.00	0.00
ATOM	9041	HW2	SOL	2319	21.800	29.790	39.520	1.00	0.00
ATOM	9042	OW	SOL	2320	35.840	27.450	3.880	1.00	0.00
ATOM	9043	HW1	SOL	2320	36.060	27.060	3.040	1.00	0.00
ATOM	9044	HW2	SOL	2320	35.620	26.710	4.440	1.00	0.00
ATOM	9045	OW	SOL	2321	52.750	36.120	5.250	1.00	0.00
ATOM	9046	HW1	SOL	2321	53.520	35.960	5.800	1.00	0.00
ATOM	9047	HW2	SOL	2321	52.890	35.570	4.480	1.00	0.00
ATOM	9048	OW	SOL	2322	45.530	31.140	28.620	1.00	0.00
ATOM	9049	HW1	SOL	2322	44.620	31.280	28.370	1.00	0.00
ATOM	9050	HW2	SOL	2322	45.980	31.960	28.380	1.00	0.00
ATOM	9051	OW	SOL	2323	10.080	26.670	41.680	1.00	0.00
ATOM	9052	HW1	SOL	2323	9.370	26.370	41.120	1.00	0.00
ATOM	9053	HW2	SOL	2323	10.170	25.980	42.340	1.00	0.00
ATOM	9054	OW	SOL	2324	3.990	22.950	17.450	1.00	0.00
ATOM	9055	HW1	SOL	2324	3.680	22.040	17.380	1.00	0.00
ATOM	9056	HW2	SOL	2324	3.410	23.440	16.860	1.00	0.00
ATOM	9057	OW	SOL	2325	41.540	45.070	51.450	1.00	0.00
ATOM	9058	HW1	SOL	2325	42.290	45.540	51.800	1.00	0.00
ATOM	9059	HW2	SOL	2325	41.920	44.420	50.860	1.00	0.00
ATOM	9060	OW	SOL	2326	29.570	22.010	13.910	1.00	0.00
ATOM	9061	HW1	SOL	2326	29.500	22.450	14.760	1.00	0.00
ATOM	9062	HW2	SOL	2326	30.460	22.200	13.610	1.00	0.00
ATOM	9063	OW	SOL	2327	20.940	8.940	22.210	1.00	0.00

ATOM	9064	HW1	SOL	2327	21.190	8.590	21.350	1.00	0.00
ATOM	9065	HW2	SOL	2327	20.600	8.190	22.690	1.00	0.00
ATOM	9066	OW	SOL	2328	18.860	42.710	14.390	1.00	0.00
ATOM	9067	HW1	SOL	2328	18.110	42.390	14.890	1.00	0.00
ATOM	9068	HW2	SOL	2328	19.410	43.140	15.040	1.00	0.00
ATOM	9069	OW	SOL	2329	43.380	11.890	17.750	1.00	0.00
ATOM	9070	HW1	SOL	2329	42.600	12.430	17.850	1.00	0.00
ATOM	9071	HW2	SOL	2329	43.230	11.400	16.940	1.00	0.00
ATOM	9072	OW	SOL	2330	10.690	3.940	35.800	1.00	0.00
ATOM	9073	HW1	SOL	2330	10.940	3.240	35.190	1.00	0.00
ATOM	9074	HW2	SOL	2330	11.520	4.290	36.120	1.00	0.00
ATOM	9075	OW	SOL	2331	4.580	0.860	44.880	1.00	0.00
ATOM	9076	HW1	SOL	2331	3.790	0.340	45.030	1.00	0.00
ATOM	9077	HW2	SOL	2331	5.160	0.280	44.390	1.00	0.00
ATOM	9078	OW	SOL	2332	27.640	20.590	24.820	1.00	0.00
ATOM	9079	HW1	SOL	2332	26.850	20.730	25.350	1.00	0.00
ATOM	9080	HW2	SOL	2332	28.060	21.450	24.790	1.00	0.00
ATOM	9081	OW	SOL	2333	31.210	7.740	30.130	1.00	0.00
ATOM	9082	HW1	SOL	2333	31.630	8.390	30.690	1.00	0.00
ATOM	9083	HW2	SOL	2333	30.300	7.710	30.440	1.00	0.00
ATOM	9084	OW	SOL	2334	49.810	26.320	31.290	1.00	0.00
ATOM	9085	HW1	SOL	2334	48.990	26.650	30.930	1.00	0.00
[truncated: 606,868 more chars]
